# Studies on the Proteome of Human Hair - Identification of Histones and Deamidated Keratins

**DOI:** 10.1038/s41598-018-20041-9

**Published:** 2018-01-25

**Authors:** Sunil S. Adav, Roopa S. Subbaiaih, Swat Kim Kerk, Amelia Yilin Lee, Hui Ying Lai, Kee Woei Ng, Siu Kwan Sze, Artur Schmidtchen

**Affiliations:** 1 School of Biological Sciences, Nanyang Technological University, 60 Nanyang Drive, Singapore, 637551 Singapore; 2 Lee Kong Chian School of Medicine, Nanyang Technological University, 59 Nanyang Drive, Singapore, 636921 Singapore; 3 School of Materials Science and Engineering, Nanyang Technological University, Singapore, Singapore; 4 Nanyang Environment and Water Research Institute, (Environmental Chemistry and Materials Centre), Interdisciplinary Graduate School, Nanyang Technological University, Singapore, Singapore; 5 Division of Dermatology, Department of Clinical Sciences, Lund University, Lund, Sweden; 6 Wound Healing Center, Bispebjerg Hospital, Department of Biomedical Sciences, University of Copenhagen, Copenhagen, Denmark; 7 Skin Research Institute of Singapore, Singapore, Singapore

## Abstract

Human hair is laminar-fibrous tissue and an evolutionarily old keratinization product of follicle trichocytes. Studies on the hair proteome can give new insights into hair function and lead to the development of novel biomarkers for hair in health and disease. Human hair proteins were extracted by detergent and detergent-free techniques. We adopted a shotgun proteomics approach, which demonstrated a large extractability and variety of hair proteins after detergent extraction. We found an enrichment of keratin, keratin-associated proteins (KAPs), and intermediate filament proteins, which were part of protein networks associated with response to stress, innate immunity, epidermis development, and the hair cycle. Our analysis also revealed a significant deamidation of keratin type I and II, and KAPs. The hair shafts were found to contain several types of histones, which are well known to exert antimicrobial activity. Analysis of the hair proteome, particularly its composition, protein abundances, deamidated hair proteins, and modification sites, may offer a novel approach to explore potential biomarkers of hair health quality, hair diseases, and aging.

## Introduction

Hair is an important and evolutionarily conserved structure. It originates from hair follicles deep within the dermis and is mainly composed of hair keratins and KAPs, which form a complex network that contributes to the rigidity and mechanical properties. Hair keratins comprise type I and type II keratins, which differ from epithelial keratin in their sulfur content^1,2^. Fifty-four keratin genes have been localized, which comprise 28 type I and 26 type II keratins^3^. Hair keratin accounts for 11 of the 28 type I keratins and 6 of the 26 type II keratins^3,4^. Hair keratin belongs to a multi-gene family and is grouped into acidic (K31–K38) and neutral basic proteins (K81–K86)^5,6^. Although hairs are mainly composed of keratins, they are very challenging to analyze due to the extensive cross-linking, which prevents solubilization.

Much interest has recently been drawn to hair follicle interactions with growth factors, cytokines, neuropeptides, neurotransmitters, hormones, and their roles as a source of stem cells. However, the hair shafts have not received much attention, despite playing roles in temperature regulation, overall defense and protection from the environment, and aesthetics. Hypothetically, hair shafts could reflect some aspects of the metabolic and physiological changes occurring at the follicular level. Furthermore, since the shafts are exposed to the environment, physicochemical factors could alter their composition. It is also possible that the human microbiome, which is also present in hair, could interact with and thus affect human hair proteins and peptides.

Hair has a high protein content with about 300 proteins identified so far^7^. It is also more chemically stable, abundant, and environmentally persistent than DNA^8^. There is, therefore, a clear interest in studying hair proteins such as keratins and KAPs from quantitative, qualitative, and functional perspectives. Hair keratins are very sturdy and extremely difficult to solubilize, and it remains technically challenging to identify and quantify these proteins accurately. Overcoming these technical challenges is therefore vital for understanding the abundances of keratin and other proteins or peptides, their structures, and their biological roles. Such information could also help to establish possible biomarkers for hair quality and hair diseases. We, therefore, sought to develop techniques for hair shaft protein extraction, identification, and quantitative profiling of different proteins.

Non-detergent methods using urea-buffers resulted in relatively low yields of protein (20–27%), although adding 2-mercaptoethanol enhanced the protein yield to 50–67%^9^. Similar protein extraction efficacy of human hair samples was achieved by microwave-assisted extraction^10^. However, these methods focused on the protein extraction yield and not the protein identification from a qualitative and quantitative perspective. Furthermore, many long-lived proteins like hair proteins are subjected to degenerative protein modifications (DPMs), which may alter protein structure and functions that regulate physiological pathways with pathological implications, among other effects^11–13^.

There is limited knowledge on the hair proteome and the potential roles of DPMs of hair proteins, including keratins. Thus, the aim of this work is to define the hair proteome after extractions with both detergent and detergent-free buffers using highly sensitive proteomics technologies based on mass spectrometry. In particular, we focused on the morphological changes, protein abundance profiles, identification of DPMs (including deamidation), and characterization of proteins and peptides. Some of these proteins and peptides could provide possible antimicrobial functions, while others serve as interesting biomarkers for hair in health and disease.

## Results

### Identification and analysis of hair proteins and evaluation of extraction techniques

We extracted hair proteins using urea buffer or methods based on sodium dodecyl sulfate (SDS) detergent defined as SDSI and SDSII. In total, 490 proteins were identified (combined search including urea, SDSI, and SDSII, supplementary Table S1). LC-MS/MS analysis of the proteins extracted using urea, SDSI, and SDSII generated 97,833, 100,048, and 98718 spectra, which correspond to 12,000, 23,000 and 20,000 peptides, respectively.

**Figure 1:**
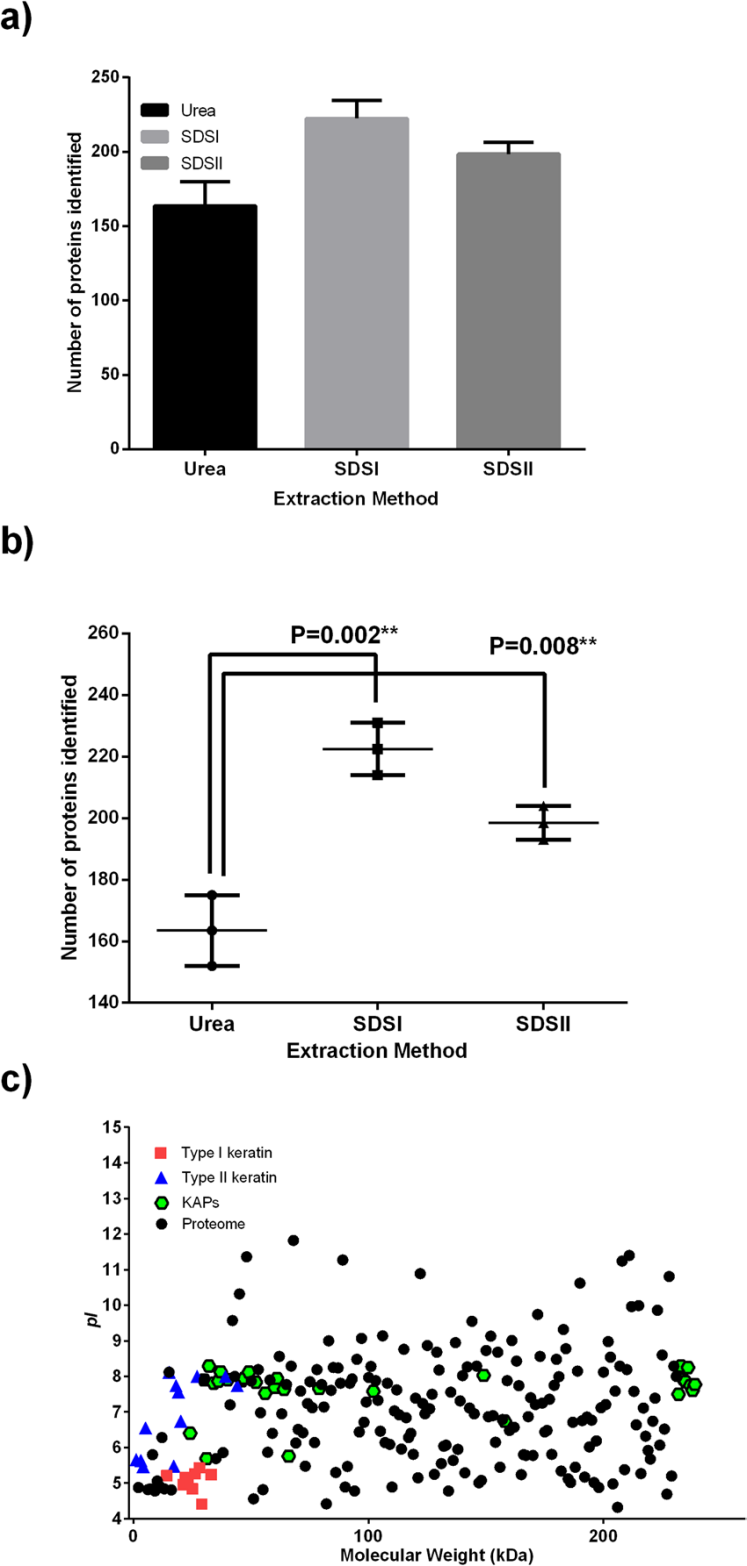
Human hair proteins identified by LC-MS/MS and their physiochemical properties. (**a**) Number of proteins identified in urea, SDSI and SDSII hair extracts by LC-MS/MS. (**b**) Statistical analysis (ANOVA) of proteins identified by LC-MS/MS. (**c**) The molecular weights and *pI* values of LC-MS/MS identified proteins are presented.

**Figure 2:**
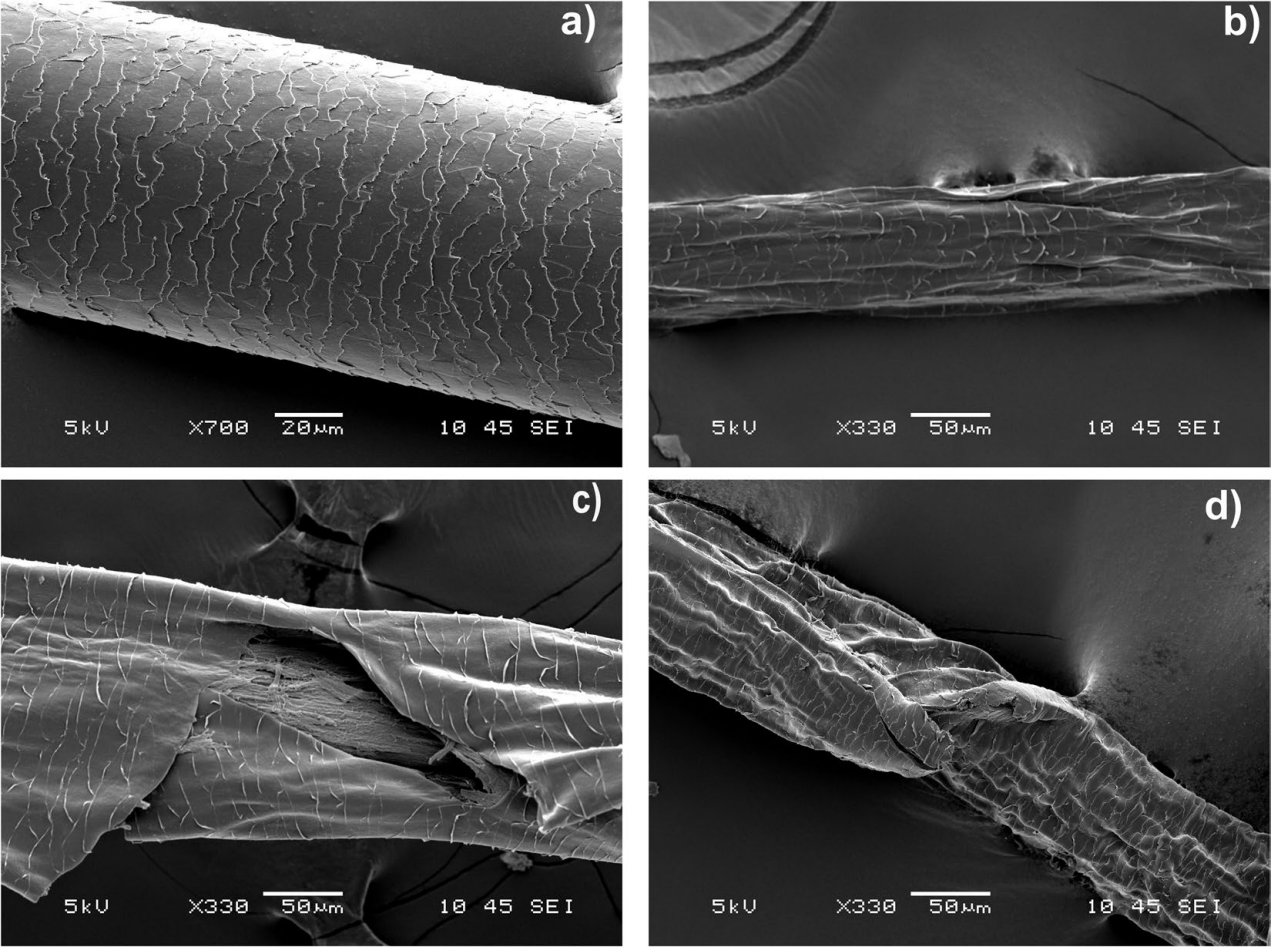
Scanning electron microscope (SEM) images of hair shaft showing surface morphology before and after protein extraction techniques. (**a**) Surface morphology of unextracted hair (Control). (**b**) Hair after one time of SDS extraction (SDSI). (**c**) Hair after two SDS extractions (SDSII). (**d**) Hair after urea extraction.

Proteomic analysis of human hair samples identified 163.5 ± 16.2, 222.5 ± 12.0 and 198.5 ± 7.7 proteins (urea, SDSI, and SDSII extraction, respectively; Fig. 1a). Quantitatively, the SDSI and SDSII extraction techniques yielded significantly higher numbers of proteins (p = 0.002^**^, p = 0.008^**^, ANOVA; Fig. 1b) compared to the urea extraction method. This suggests that SDS facilitates hair proteome solubilization. Figure 1c indicates the physicochemical characteristics of the identified proteins including the molecular weight (MW) and isoelectric point (pI). Physicochemical analysis of type I keratins revealed *pI* values in the range 4.5–5.5 with molecular weights of 40–60 kDa (Fig. 1c). The *pI* values of type II keratins were 5–8 with MW of 50–70 kDa (Fig. 1c). The effects of the protein extraction techniques on the overall morphology of human hair shaft samples were observed by scanning electron microscope (SEM). Compared to normal hair without protein extraction (Fig. 2a), extracted hair shafts using SDSI, SDSII, and urea extraction showed significant shrinking and depletion of mass (Fig. 2b–d). Based on the observed morphological changes i.e. decrease in thickness, the degree of shrinking, mass depletion, overall damage of the hair shaft surface, number of proteins identified and their abundance, the SDS extraction techniques were more efficient than the urea-based extraction method (Table 1).

**Table 1:** Summary of parameters related to different hair extraction techniques.

Parameters	Mean Thickness^#^			Shrunken volume^#^			Mass depletion^#^			Overall surface damage^##^			Total Score	Proteins identified by LC-MS/MS	Protein abundances in emPAI (Table 2 and S1)
Extraction methods	E1	E2	E3	E1	E2	E3	E1	E2	E3	E1	E2	E3			
Normal hair	0	0	0	0	0	0	0	0	0	0	0	0	0	NA	NA
Urea	2	1	1	2	2	2	1	2	2	1	1	2	19	163 ± 16	+
SDS I	1	2	2	2	3	2	2	2	2	2	2	2	24	222 ± 12	+++
SDS II	2	1	2	2	2	3	2	2	3	3	3	2	27	198 ± 8	++

Based on the SEM images, grading of the overall surface damage was scored by three independent evaluators from 0 to 3. A total of 30 hair samples after protein extraction were evaluated and given an overall score. Changes (thickness, shrinking, mass depletion) and hair surface damage were assessed by three evaluators (E1, E2 and E3).

^#^Degree of thickness decrease, shrunken volume and mass depletion- 0: no change; 1:10–25%; 2: 26–50%; 3: over 50%.

^##^Hair surface damage- 0: no; 1: minor, 3: major.

Proteins with FDR ≤1%, protein score greater than 30 and identified with more than two peptides were considered. We selected proteins identified in two experimental sets. Thus, 90, 123, and 99 proteins were identified in two experimental replicates of urea, SDSI, and SDSII extractions, respectively (Supplementary Fig. S1a–c). Of these identified and quantified proteins, 64 proteins were common to all extraction protocols, whereas 16, 35, and 11 proteins were unique to the urea, SDSI, and SDSII extractions, respectively (Supplementary Fig. S1d). GO-enrichment analysis (with p ≥ E-0.03) revealed 47.45% enrichment of total keratin filaments, 35.18% intermediate filament, and 29.46% intermediate filament cytoskeleton proteins (Supplementary Fig. S2).

### Comparative abundances of hair shaft proteins by detergent and detergent-free extraction methods

**Figure 3:**
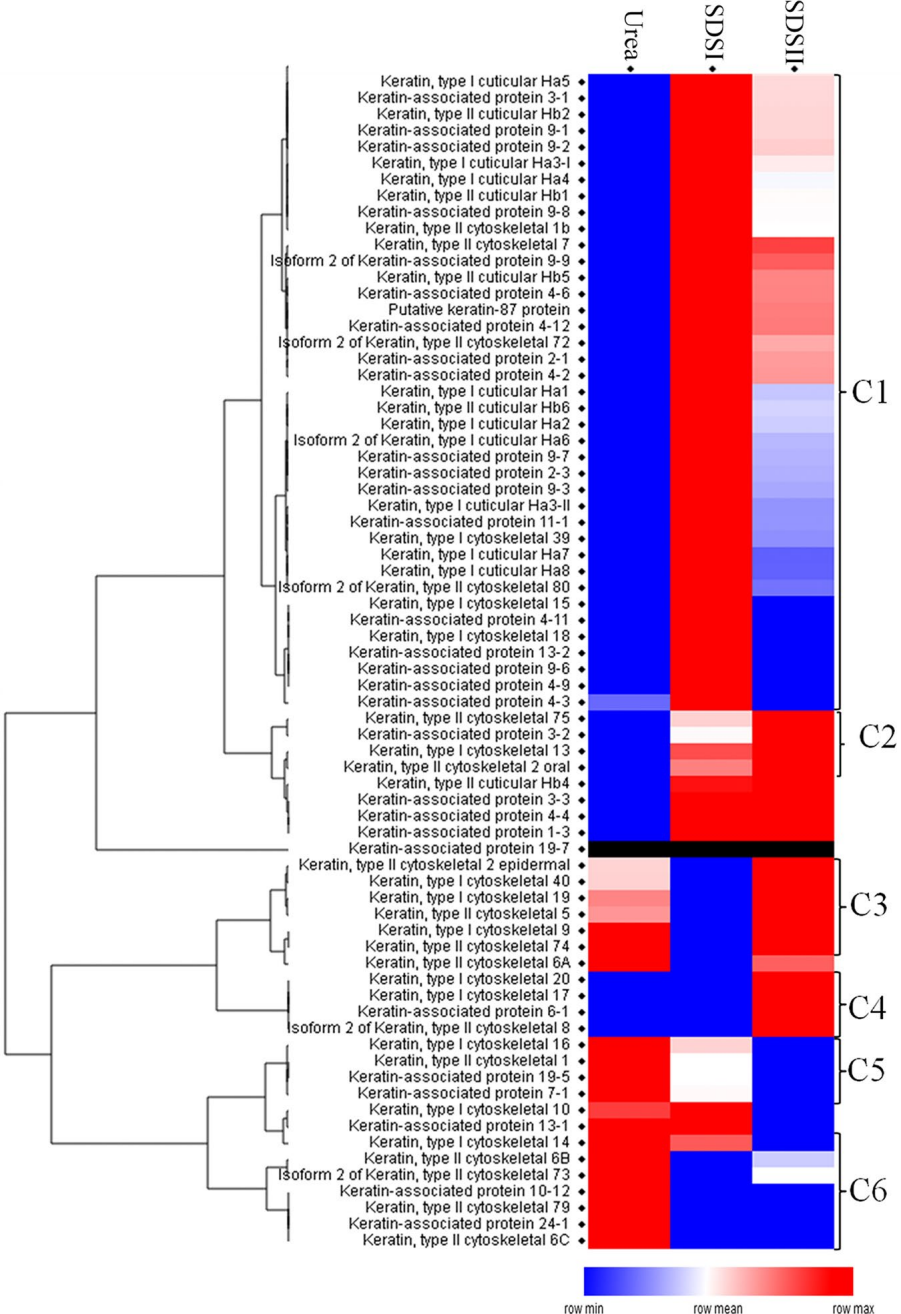
Hierarchical clustering of keratin and keratin-associated proteins identified in the “extractome” of hair using different extraction methods. Highly abundant protein values are displayed in red, low abundance is indicated by blue, and intermediate values are in different shades of red and blue.

Proteomic data showed that keratins and KAPs are the most abundant proteins in human hair. Hierarchical clustering of keratins and KAPs alone revealed six major clusters (C1-C6) (Fig. 3). Interestingly, proteins clustered under cluster C1 were significantly enriched in the first SDS extraction (SDSI), including keratin, type I cuticular K31, K32, K33a, K33b, K34, K35, K37, and K38; keratin; type II cuticular K81, K82, K83, K84, and K85; and several KAPs. Although cuticle keratins are difficult to extract, proteomic identification and quantitative evaluation indicated that there were significantly higher abundances in the SDS-extracted material of cuticular keratin, type II K81, K82, K83, K84, K85, and K86; and type I K31, K33a, K33b, K34, K35, K32, K36, K37, and K38 (Supplementary Fig. S3a,b).

Keratin intermediate filaments remain surrounded by KAPs that form a rigid, inert, and resistant hair shaft. These KAP proteins (Table 2) were significantly abundant in the SDSI extraction compared to urea method. Thus, detergent (2% SDS) not only solubilizes cuticular proteins but also cortex proteins. Proteins in clusters C2-C4 were enriched in the SDSII extraction when compared to the urea and SDSI extracts, indicating that these proteins need prolonged detergent treatment for their solubilization. Proteins in clusters C5-C6 were abundant in the urea-extracted material.

**Table 2:** Abundances of keratins and keratin associated proteins in the hair proteome extracted by using urea, SDSI and SDSII.

Accession	Gene Symbol	Protein designation	Name	Protein Mass	emPAI ± SD			Peptides ± std		
					Urea	SDSI	SDSII	Urea	SDSI	SDSII
O43790	KRT86	K86	Keratin, type II cuticular Hb6	55120	971 ± 139	474053 ± 107988	196787 ± 11084	551 ± 17	1369 ± 23	1126.5 ± 11
Q15323	KRT31	K31	Keratin, type I cuticular Ha1	48633	380 ± 37	89947 ± 28500	35218 ± 9038	378.5 ± 3	1114 ± 40	939 ± 45
P78385	KRT83	K83	Keratin, type II cuticular Hb3	55928	570 ± 32	161681 ± 69193	99410 ± 64419	464 ± 10	1160.5 ± 24	974 ± 15
A0A087X106	KRT81	K81	Keratin, type II cuticular Hb1	54850	519 ± 90	126473 ± 28976	64424 ± 38926	478.5 ± 16	1200.5 ± 28	989.5 ± 10
P78386	KRT85	K85	Keratin, type II cuticular Hb5	57306	651 ± 0	76687 ± 22848	56820 ± 32193	462.5 ± 5	1129.5 ± 9.5	985 ± 6
O76009	KRT33A	K33a	Keratin, type I cuticular Ha3-I	47166	194 ± 13	16335 ± 551	8889 ± 300.39	313 ± 1	893 ± 34	770 ± 30
Q14525	KRT33B	K33b	Keratin, type I cuticular Ha3-II	47325	143 ± 24	34465 ± 7988	10098 ± 1687	308.5 ± 0.5	880 ± 28	734.5 ± 31
A6NCN2	KRT87P		Putative keratin-87 protein	29555	1287 ± 137	98039 ± 15572	74352 ± 39258	302 ± 5	680.5 ± 30	583 ± 25
O76011	KRT34	K34	Keratin, type I cuticular Ha4	50818	24.42 ± 0.8	1033 ± 32	514.855 ± 171	189.5 ± 0.5	571 ± 6	475.5 ± 14
Q92764	KRT35	K35	Keratin, type I cuticular Ha5	51640	6.26 ± 0.45	56.63 ± 1.78	35.21 ± 0	131 ± 3	362 ± 1	303.5 ± 3.5
Q14532	KRT32	K32	Keratin, type I cuticular Ha2	51793	1.6 ± 0.08	8.26 ± 1.13	4.28 ± 0	65.5 ± 1.5	183.5 ± 2.5	140.5 ± 6.5
O76013-2	KRT36	K36	Isoform 2 of Keratin, type I cuticular Ha6	48424	0.64 ± 0.05	2.63 ± 0.35	1.36 ± 0	41.0 ± 0	107 ± 1	96 ± 4
Q9NSB2	KRT84	K84	Keratin, type II cuticular Hb4	65942	0.44 ± 0.03	0.75 ± 0.12	0.77 ± 0.21	31 ± 3	83.5 ± 5.5	68.5 ± 2.5
Q9NSB4	KRT82	K82	Keratin, type II cuticular Hb2	57985	0.89 ± 0.05	3.97 ± 0.27	2.68 ± 0.30	39.5 ± 1.5	126 ± 10	87.5 ± 5.5
Q9BYR8	KRTAP3-1		Keratin-associated protein 3-1	11558	1.19 ± 0	3.79 ± 0.00	2.69 ± 0	21 ± 1	34.5 ± 2.5	30.5 ± 1.5
O76014	KRT37	K37	Keratin, type I cuticular Ha7	51084	0.29 ± 0.08	1.72 ± 0	0.56 ± 0.09	28.5 ± 1.5	88.5 ± 5.5	69.5 ± 7.5
O76015	KRT38	K38	Keratin, type I cuticular Ha8	52044	0.24 ± 0.04	1.59 ± 0.08	0.49 ± 0.04	24 ± 1	69 ± 3	50.5 ± 0.5
Q9BYR6	KRTAP3-3		Keratin-associated protein 3-3	11441	0.69 ± 0	1.2 ± 0	1.2 ± 0	10 ± 1	18.5 ± 1.5	14 ± 0
Q8IUC1	KRTAP11-1		Keratin-associated protein 11-1	18385	2.24 ± 0	3.60 ± 0.76	2.64 ± 0.9	22 ± 3	28.5 ± 5.5	21.5 ± 1.5
F5H1T9	KRTAP2-1		Keratin-associated protein 2-1	14926	ND	1.54 ± 0.26	1.06 ± 0.21	ND	14.5 ± 5.5	11.5 ± 1.5
P0C7H8	KRTAP2-3		Keratin-associated protein 2-3	15465	0.35 ± 0.13	1.7 ± 0	0.82 ± 0	5 ± 0	9.5 ± 6.5	6 ± 3
Q52LG2	KRTAP13-2		Keratin-associated protein 13-2	19912	1.37 ± 0.18	2.79 ± 0.29	1.37 ± 0.18	20 ± 1	26.5 ± 1.5	16 ± 1
Q9BYQ5	KRTAP4-6		Keratin-associated protein 4-6	26141	0.43 ± 0	0.82 ± 0	0.72 ± 0.1	8.5 ± 1.5	16 ± 1	12.5 ± 0.5
Q9BYR7	KRTAP3-2		Keratin-associated protein 3-2	11483	0.69 ± 0	0.94 ± 0.25	1.2 ± 0	10 ± 0	12 ± 1	12 ± 2
Q9BQ66	KRTAP4-12		Keratin-associated protein 4-12	25609	0.28 ± 0	0.73 ± 0.10	0.63 ± 0	4 ± 2	15.5 ± 0.5	11.5 ± 0.5
Q9BYR3	KRTAP4-4		Keratin-associated protein 4-4	21487	0.78 ± 0.00	1.38 ± 0	1.38 ± 0	9 ± 1	12.5 ± 0.5	10 ± 1
Q9BYQ3	KRTAP9-3		Keratin-associated protein 9-3	19805	1.38 ± 0.18	2.82 ± 0.3	1.86 ± 0.66	8.5 ± 1.5	13.5 ± 1.5	12.5 ± 0.5
Q3LI72	KRTAP19-5		Keratin-associated protein 19-5	7847	3.39 ± 0	2.71 ± 0.68	2.03 ± 0	9 ± 1	6 ± 1	6 ± 1
Q9BYP9-2	KRTAP9-9		Isoform 2 of Keratin-associated protein 9-9	21512	0.34 ± 0	1.22 ± 0.16	1.06 ± 0	2 ± 0	9.5 ± 3.5	9 ± 0
A0A087WU60	KRTAP9-2		Keratin-associated protein 9-2	20290	0.71 ± 0.13	2.4 ± 0	1.71 ± 0.20	6.5 ± 0.5	13 ± 3	11.5 ± 1.5
Q8IUC0	KRTAP13-1		Keratin-associated protein 13-1	19505	0.61 ± 0	0.61 ± 0	0.37 ± 0.00	7.5 ± 0.5	6 ± 0	5.5 ± 0.5
A8MXZ3	KRTAP9-1		Keratin-associated protein 9-1	31386	ND	0.5 ± 0	0.28 ± 0.06	ND	4 ± 2	5.5 ± 0.5
A8MTY7	KRTAP9-7		Keratin-associated protein 9-7	20915	0.25 ± 0.09	1.11 ± 0	0.56 ± 0	2.5 ± 0.5	7.5 ± 2.5	6 ± 0
Q9BYQ6	KRTAP4-11		Keratin-associated protein 4-11	24844	0.29 ± 0	0.46 ± 0	0.29 ± 0	5 ± 0	8.5 ± 1.5	6 ± 1
Q9BYR5	KRTAP4-2		Keratin-associated protein 4-2	17130	0.31 ± 0.11	0.89 ± 0.17	0.72 ± 0.00	2.0 ± 0.5	5.5 ± 1.5	4.5 ± 0.5
Q8IUC3	KRTAP7-1		Keratin-associated protein 7-1	9624	0.85 ± 0	0.60 ± 0.24	0.36 ± 0	5 ± 0	2 ± 0	ND
Q9BYR4	KRTAP4-3		Keratin-associated protein 4-3	24536	0.57 ± 0.1	1.3 ± 0.15	0.38 ± 0.09	8 ± 2	12.5 ± 1.5	5 ± 3
Q8IUG1	KRTAP1-3		Keratin-associated protein 1-3	20850	0.16 ± 0	0.25 ± 0.09	0.25 ± 0.09	11 ± 1	11.5 ± 1.5	6.5 ± 1.5
A8MVA2	KRTAP9-6		Keratin-associated protein 9-6	19638	ND	0.27 ± 0.1	0.17 ± 0	ND	3 ± 1	2.0 ± 0.5
A0A087WTB3	KRTAP9-8		Keratin-associated protein 9-8	20295	ND	1.15 ± 0	0.58 ± 0	ND	5.5 ± 3.5	3.5 ± 2.5
A0A087X0S9	KRTAP4-9		Keratin-associated protein 4-9	23399	ND	0.51 ± 0.2	ND	ND	7 ± 2	ND
P60413	KRTAP10-12		Keratin-associated protein 10-12	28680	0.12 ± 0	ND	ND	2 ± 1	ND	ND

ND: Not detected.

### Antimicrobial proteins in hair

**Figure 4:**
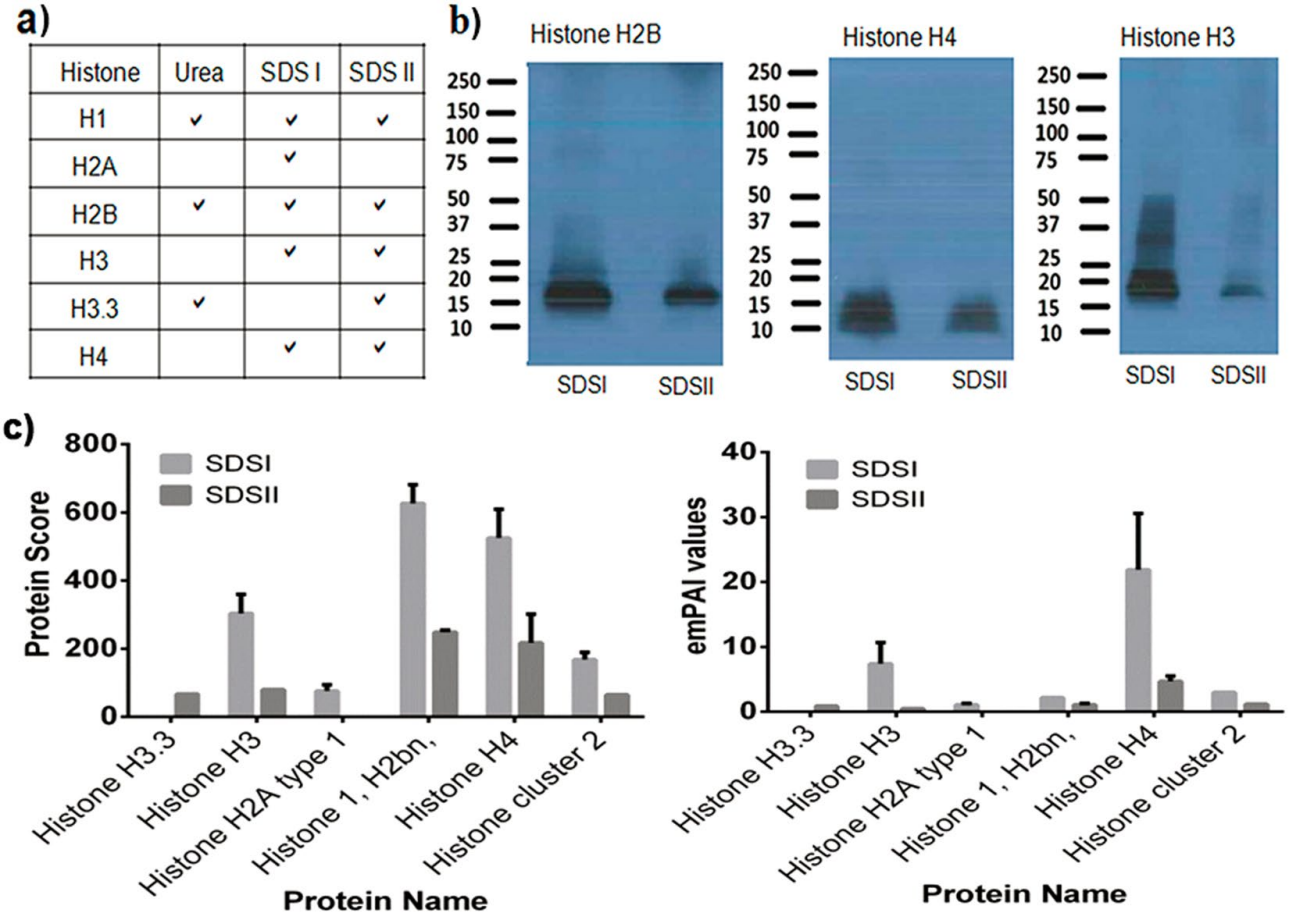
Mass-spectrometry-based proteomic identification and western blot detection of histones, their abundances determined by protein score and emPAI values. (**a**) Table showing different histones identified by mass spectrometry. (**b**) Western blot analysis illustrating presence of different histones (full lengths WBs of histones are provided in supplementary information Figs S4–S6). (**c**) Abundances of different histones.

We also identified lysozyme (Supplementary Table S1), which is widely known for its ability to enzymatically degrade bacterial cell walls and non-enzymatic lysis of bacterial membranes. The protein S100A8, which has antimicrobial activity, was also identified and quantified. The significantly higher abundances of hair shaft protein and better coverage by SDS extraction prompted us to focus on the SDS extractome. In addition to lysozyme and protein S100A8, various histones were quantified in the hair proteome (Fig. 4a).

Given their important roles in antimicrobial function, we next validated the proteomics data by western blot for selected components (Fig. 4b). Both the proteomics data (Fig. 4c) and western blot analysis identified histones H1, H2B, H3, and H4 in the extracts of the hair shafts.

### Hair protein modifications

**Figure 5:**
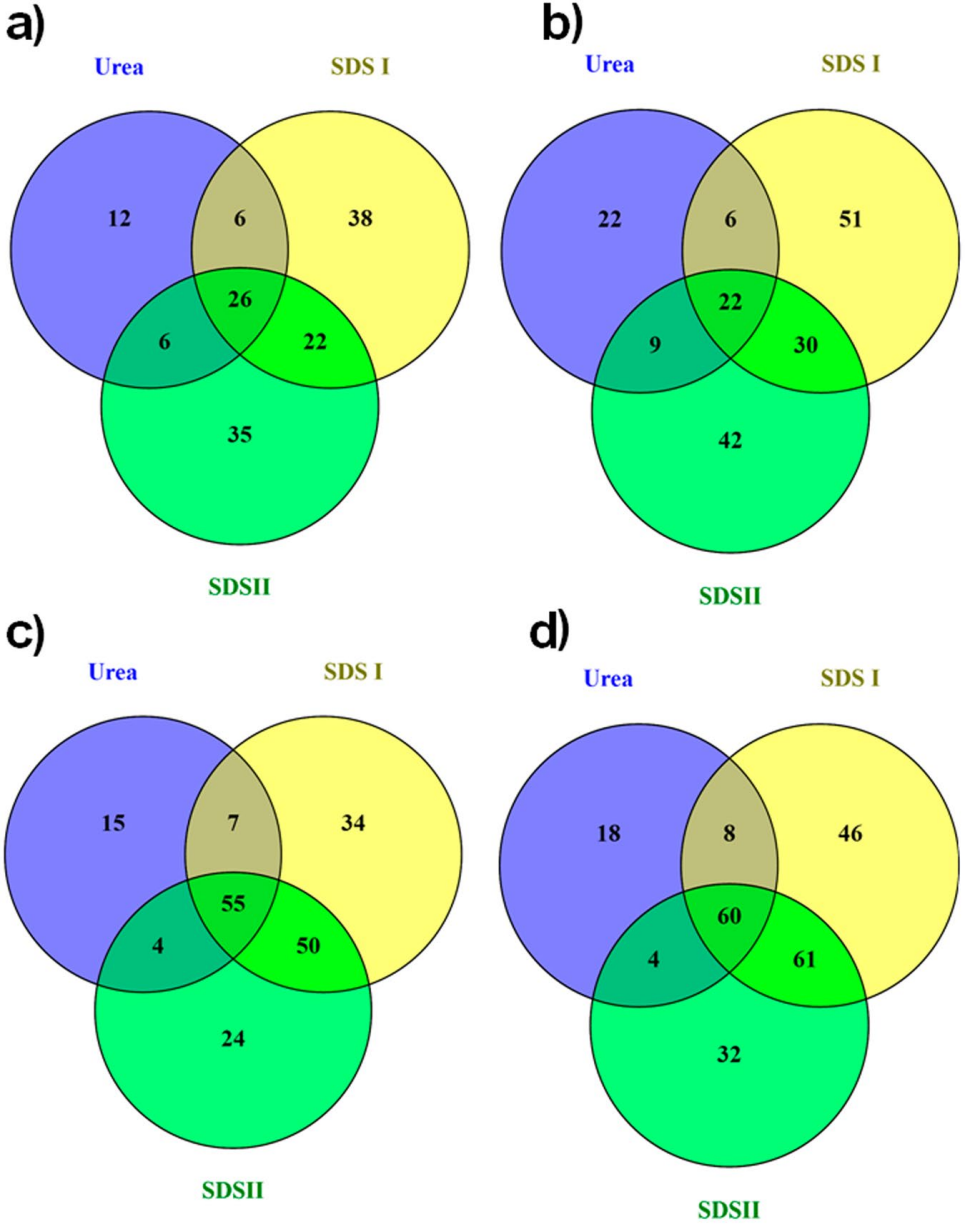
Venn diagram showing overlap of Unique N- and Q-deamidation peptides and modification sites identified in hair proteome extracted by different techniques. Overlap analysis of (**a**) Unique Q-deamidated peptides, (**b**) Unique Q-modified sites, (**c**) Unique N-deamidated peptides, and (**d**) Unique N-deamidated sites detected using the different extraction techniques (Urea, SDSI and SDSII).

We also observed extensive deamidation of hair proteins extracted by the urea, SDSI, and SDSII protocols. The data revealed 26 common Q-modified peptides in all three extractions, whereas 38 and 35 peptides were unique to the SDSI and SDSII extracted proteomes (Fig. 5a). The Q-modification site analysis showed 22 common modification sites, while 51 and 42 sites were identified in the SDSI and SDSII extractome (Fig. 5b). Similarly, 55 N-modified peptides were common for all extraction techniques used, while 34 and 24 peptides were only detected in the SDSI and SDSII extractions, respectively (Fig. 5c). The N-modification site analysis is shown in Fig. 5d. The spectrums showing the peptide sequence and sites of modifications are provided as supplementary information (deamidation spectrum mascot view).

## Discussion

The conventional methods employing urea (in the range 2–8 M) and a reducing agent for extraction of human hair proteome yielded 8–27% of the original hair proteins, but the protein yield was increased to 65% with the Shindai method^9^, which uses 5 M urea with 2.6 M thiourea in the extraction buffer. Thus, the use of thiourea in the extractions may further enhance protein release and increase the coverage of the total hair proteome. Zhang *et al*.^14^ achieved an extraction yield of 57% when using sodium hydroxide (0.4 mol/l). However, these methods focused on only the protein yield, and no efforts were made to identify the protein composition of hair as in the present study.

Notably, proteins extracted by the Shindai method and other conventional techniques indicated microfibril keratins of 40–60 kDa, other matrix proteins of 10–20 kDa, and minor high–molecular-weight components (110–115 and 125–135 kDa)^9^. This study identified proteins using a label-free quantitative method and found that hair proteins are distributed in the range of 3–408 kDa with abundance spanning several orders of magnitude, including the identification of keratin microfibrils, various KAPs, and matrix proteins. The identification of 222 ± 12.0 and 198.5 ± 7.7 proteins by the SDS protocols was significantly higher than the 76 proteins identified by Laatsch *et al*.^15^, who used a shotgun proteomic approach after treatment with 2% SDS and 0.1 M sodium phosphate (pH 7.8). In a pairwise comparison of 76 proteins, Laatsch *et al*.^15^ noted substantial differences in keratin abundances among samples from Caucasian, African-American, Kenyan, and Korean subjects. There were also distinguishable keratin profiles in the hair shaft from the axillary, beard, pubic, and scalp regions of Caucasian subjects. Thus, the protocols reported here should improve future in-depth characterizations of hair and provide more potential biomarkers. The catalog of human hair keratins groups eleven individual members of type I hair keratin into three subfamilies, i.e. group A (K31, K33a, K33b, and K34), group B (K37, and K38), group C (K32, K35, and K36)^5^, and K39 and K40. Furthermore, six members of type II hair keratin fall into two groups, i.e. group A (K81, K83, and K86) and group C (K82, K84, and K85)^6^. It is of note that K37 has mainly been described in vellus hair whereas K84 is specific to the dorsal tongue filiform papilla. However, our study, using a shotgun proteomics approach, identified K37 and K84 (see Table S2, Peptide summary and MS/MS spectrums provided as a supplementary). In their characterization of the hair shaft proteome in human subjects by mass spectrometry-based shotgun proteomics, Parker and colleagues^16^ identified K84 with an abundance of 5.59 and K37 with 2.43. As tabulated in Table 2, this study quantified K84 with emPAI values between 0.44–0.77 and identified with peptides 31 ± 3, 83.5 ± 5.5 and 68.5 ± 2.5 in urea, SDSI and SDII methods, respectively. The corresponding values of K37 were 0.29–1.72 and peptides 28.5 ± 1.5, 88.5 ± 5.5 and 69.5 ± 7.5, respectively (Peptide summary and MS/MS spectrums of K84 and K37 provided as supplementary). It is also notable that Lee *et al*.^7^ identified K37 with 11 and K84 with 89 MS/MS spectra during proteomic analysis of human shaft. Similarly, shotgun proteomic analysis of hair samples from different ethnic groups revealed considerable variation in the K37 profile^15^. Using antisera, Langbein *et al*.^5^ detected K37 in central cortex cells but not in the large anagen follicles of terminal scalp hairs, however, a divergent expression was noted in vellus hair. K84 was not detected in hair but was detected in dorsal tongue filiform papilla^6^. Taken together, the detection of K37 and K84 proteins in hair shaft by proteomics techniques may be due to a high sensitivity of mass spectrometry techniques, which can detect trace amounts of specific keratins that usually may not be detected by classical protein detection methods, including western blotting and immunohistochemistry. As sequence homologies may also influence the results in shotgun proteomics, the identification and quantitation of low abundance molecules, such as K37 and K84, therefore need careful validation of the obtained mass spectrometry data.

The buckling to the surface of the fibres after extraction as seen in the SEM images, are consistent with the removal of cortical material from the hair fibres. SEM, however, although useful for overall assessment of hair fiber damage does not provide exact ultrastructural information on cuticle extraction, as may be obtained by transmission electron microscopy (TEM). Nevertheless, SEM provides high-resolution images with detailed surface information, as previously demonstrated in several studies on hair shafts^17–20^. Furthermore, in the evaluation of hair damage induced by ultraviolet irradiation in Asian, Caucasian and African populations, Ji *et al*.^21^ found structural evidence for hair damage using both SEM and transmission TEM. Also, Richena and Rezende^22^ utilised TEM imaging and found mechanical damage including removal of cuticle cells from the hair, which corresponded with observations using field emission scanning electron microscopy (FESEM). In summary, SEM is a valuable tool to study overall hair shaft abnormalities, whereas TEM, which covers a smaller area is better suited for detailed ultrastructural evaluations. TEM analyses, however, were beyond the scope of the present study aimed at protein identification and optimized extraction methods.

Human skin contains about 10^2^ to 10^6^ microorganisms/cm^2^, and they grow in small colonies on the surface, as well as in the pores of hair follicles^23^. Human skin is rarely infected, partly due to the presence of constitutively and inducible antimicrobial peptides (AMPs) or proteins^24^. Like dermcidin, AMPs have been isolated from sweat glands and demonstrated that sweat plays a role in the regulation of human skin flora via AMPs^25^. Analogously, hair follicles and hairs are also continually exposed to microorganisms, and from an evolutionary perspective, it is reasonable to assume that hair would have some inherent antimicrobial defenses. However, there are only a few reports on AMPs in human hair follicles, as most researchers have focused on the epidermis and dermis. Similarly, there are no published studies on specific antimicrobials in hair shafts to the best of our knowledge. When analyzing proteomics data, we found lysozyme C and lysozyme g-like protein 2, which are both known to exert antibacterial activities^26^. Lysozymes are present in virtually all body fluids of human tissues. Their concentrations are particularly high in tears, gastric juice, and milk^27,28^. In addition to antibacterial activity, they are also active against viruses such as HIV-1, and they have fungistatic effects^29,30^. It is therefore notable that several proteins belonging to the S100 family were identified in this study, including S100A3, S100A6, S100A8, and S100A14. S100A8 and S100A9 exert antimicrobial activity towards bacteria and fungi via chelation of Zn^2+^, and they play a role in the regulation of inflammatory processes^31,32^. Protein S100A7 is also present in hair and is called psoriasin. It was first isolated as an antimicrobial protein from keratinocytes of psoriatic epidermis^33^, and it is highly produced in areas with high bacterial colonization, such as hair follicles and the nose^26^.

Histones play very important roles in gene transcription regulation and are functionally classified into two groups: linker histones (histone H1), which seal loops of DNA and make nucleosome structures compact, and core histones (histone H2A, H2B, H3, and H4), which form an octameric complex to produce the nucleosome^34–36^. Broad-spectrum antimicrobial activity has been reported for histones and histone-derived peptides from shrimps^37^, fish skin^38,39^, frogs^40^, chickens^41^, and mammals^42–44^. It is therefore notable that we identified several histones in the hair samples, such as histones H1, H2A, H2B, H3, and H4. Taken together, these results indicate that human hair contains a multitude of well-known antimicrobials. Of interest is that extracts of hair indeed show antimicrobial activity. Current data demonstrate that urea-extracted material, partially purified by separation using reverse phase high-performance liquid chromatography is antibacterial against *Escherichia coli* in a radial diffusion assay (manuscript in preparation). It remains to be shown whether the hair shafts *per se* display antimicrobial activities.

Protein deamidation has been proposed to represent a “molecular clock” that has been linked to tissue aging, regulating biomolecule longevity and timing key host processes, and altering the host response to therapeutics over prolonged periods^11,45–48^. Deamidation adds a negative charge at sites of modification and thereby alters or manipulates the protein structure^11,46,49^. This study identified 15 of 17 hair keratins (K31-K38, K81–87), and interestingly, deamidation was observed in keratin type I belonging to the families K33A, K33B, K34, and K35; type II belonging to families K82-K86; and several KAPs. The impact of the deamidation of hair proteins on their function, structure, and complex networking has not been documented and requires more research to integrate hair proteomic data with hair protein structural analysis. Information on the extent of deamidation and the specific sites of modification, as reported here, could be crucial for further studies on hair proteins.

Notably, age estimation of wool samples (within the past ~400 years) has been achieved via deamidation studies for museum specimens^50^. A linear correlation was found in the rate of deamidation with age^46^. Deamidation has also been reported in sheep hair samples, and hence, the extent of deamidation of human hair keratin could hypothetically be used as a biomarker of human aging and for dating human and animal specimens. Further research is also required to establish the correlation between the deamidation of different keratins, hair-related diseases, and hair quality. The rate of protein deamidation is affected by environmental factors including pH, temperature, and humidity, as well as the primary, secondary, tertiary, and the quaternary structures of the proteins^11,49,51^. Thus, deamidation could potentially be used to identify underlying conditions in the skin or hair follicles, including inflammatory diseases such as dermatitis of various causes, scarring alopecia disorders, or alopecia areata. Furthermore, the use of different detergents and shampoos for hair washing may influence hair health and the hair protein deamidation profile. In other words, deamidation profiles of keratins may potentially serve as important biomarkers of hair health and hair quality, as well as correlate to various hair diseases.

In conclusion, this study used detergent and detergent-free methods to define the hair proteome, which led to the identification of keratins, multiple KAPs, and other proteins. Several proteins belonging to the family of host defense factors were identified, such as S100 proteins and histones. Deamidation was identified among keratins and KAPs. Information on deamidated keratins, KAPs, and the hair proteome, in general, could be used in future studies to define novel biomarkers for clinical and personalized evaluation of hair, as well as providing clues to novel functions of hair.

## Materials and Methods

### Hair sampling, protein extraction, and scanning electron microscopy

Human scalp hair samples, derived from the distal parts (usually representing 10–30% of total hair length), were obtained from five healthy individuals (age: 24.2 ± 0.44) without prior chemical treatments such as hair dyes, bleach, or perms. Of note, we used distal part of the hairs and thus, avoided vellus hairs which are soft, fine and short. Written informed consent for collection of hair samples and its analysis was obtained from the donors. The study was approved by the Institutional Review Board of Nanyang Technological University under approval number IRB-2016-11-042 and conducted following principles as stated in the Declaration of Helsinki. An equal amount of hair samples from each individual (N = 5) were pooled and hair proteins were extracted in the detergent-free buffer by incubating 50 mg of hair with 8 M urea buffer containing 25 mM Tris-HCl, 25% ethanol, and 200 mM dithiothreitol (DTT) at pH 9.5 and 55 °C for 72 h. The supernatant was collected by centrifugation at 17,000 × g for 15 min and used for further analysis. For extraction with detergents, 80 mg of hair were suspended in 50 mM Tris at pH 7.2 containing 2% SDS and 20 mM DTT, followed by bath sonication and further incubation overnight at 65 °C. The supernatant was collected (termed as SDSI), and the pellet fraction was further extracted (termed as SDSII).

The proteins extracted by these techniques were precipitated using ice-cold acetone and dissolved in 50 mM Tris buffer at pH 6.8. Protein content was estimated using a BCA assay. The hair shaft morphology before and after protein extraction was observed using a scanning electron microscope (SEM, Jeol JSM- 5310, Tokyo, Japan). Hair shaft samples before and after protein extraction were prepared for SEM by fixing in 4% paraformaldehyde for 2 h, followed by dehydration in 30, 50, 70, and 100% ethanol.

### Protein digestion and mass spectrometric analysis

The keratin proteins extracted by different methods were digested, and peptide extraction was performed as described previously^12^. In brief, equal amounts of hair proteins (40 μg) were separated on 12% SDS-PAGE. The gel was stained with Coomassie brilliant blue. Each sample lane was sliced separately, cut into small pieces of approximately 1 mm^2^, and subjected to complete destaining. After destaining the gel pieces were reduced with DTT (10 mM), alkylated using iodoacetamide (55 mM), dehydrated with 100% acetonitrile and then subjected to overnight digestion at 37 °C with sequencing-grade modified trypsin (Promega, Madison, WI). The peptides were extracted, vacuum dried, and reconstituted in 0.1% formic acid for LC-MS/MS analysis. The mass spectrometric analysis involved technical replicates of each sample extracted by the different extraction methods.

Peptides were separated and analyzed on a Dionex Ultimate 3000 RSLC NanoLC system coupled to a Q-Exactive apparatus (Thermo Fisher, MA). Approximately 1.5 µg of material was injected into an Acclaim peptide trap column via the autosampler of a Dionex RSLC NanoLC system. Mobile phase A (0.1% FA in 5% ACN) and mobile phase B (0.1% FA in ACN) were used to establish a 60-min gradient with a flow rate of 300 nl/min. Injected peptides were analyzed on a Dionex EASY-spray column (PepMap® C18, 3um, 100 A) using an EASY nanospray source at an electrospray potential of 1.8 kV.

A full MS scan (350–1600 m/z range) was acquired at a resolution of 70,000 at m/z with a maximum ion accumulation time of 100 ms. The dynamic exclusion was set to 30 s. The resolution for the MS/MS spectra was set to 35,000 at m/z. The AGC setting was 1E6 for the full MS scan and 2E5 for the MS2 scan. The 10 most intense ions above a 1000-count threshold were selected for HCD fragmentation with a maximum ion accumulation time of 120 ms. An isolation width of 2 Da was used for the MS2 scan. Single and unassigned charged ions were excluded from MS/MS. For HCD, the normalized collision energy was set to 28. The underfill ratio was defined as 0.1%

### Mass spectrometry data analysis

Raw data files were converted into the mascot generic file format using Proteome Discoverer version 1.4 (Thermo Electron, Bremen, Germany) with the MS2 spectrum processor for de-isotoping the MS/MS spectra. The concatenated target-decoy UniProt human database (sequence 90411, total residues 358, 86,945, downloaded on April 1, 2015) was used for the data search. The database search was performed using an in-house Mascot server (version 2.4.1, Matrix Science, Boston, MA) with MS tolerance of 10 ppm and MS/MS tolerance of 0.02 Da. Two missed trypsin cleavage sites per peptide were tolerated. Carbamidomethylation (C) was set as a fixed modification, while oxidation (M) and deamidation (N and Q) were variable modifications. Label-free protein quantification was performed using emPAI^52^ values reported by the Mascot search engine, which is based on the following equations:

$$PAI={N}_{obsd}/{N}_{obsb1}$$

$$emPAI={10}^{PAI}-1$$

where N_obsd_ and N_obsbl_ are the number of observed and observable tryptic peptides per protein, respectively. The hierarchical clustering of protein expression was analyzed using GenePattern (http://www.broadinstitute.org/cancer/software/genepattern/).

### Western blot analysis

Hair proteins (50 μg) were separated on 12% polyacrylamide gels, transferred onto 0.45-μm PVDF membranes (BioRad, Hercules, CA), blocked and probed overnight at 4 °C with the primary antibodies. Antibodies against histone H1.0 (Abcam), histone H2A (Cell Signaling Technology), histone H2B (Abcam), histone H3 (Cell Signaling Technology), and histone H4 (Cell Signaling Technology), and secondary antibodies against rabbit immunoglobulins (Dako) were used in this study.

### Data availability

“The mass spectrometry proteomics data have been deposited to the ProteomeXchange Consortium via the PRIDE^53^ partner repository with the dataset identifier “PXD007224”.

## Electronic supplementary material

Table S1 and S2

Supplementary figures
