# Supplementary material for: Studies on the Proteome of Human Hair - Identification of Histones and Deamidated Keratins
Source: Sci Rep. 2018 Jan 25;8:1599. doi: 10.1038/s41598-018-20041-9 (PMC5785504; doi:10.1038/s41598-018-20041-9)
Supplement: Supplementary file 2 — Supplementary figures [file 41598_2018_20041_MOESM2_ESM.pdf]

Studies on the proteome of human hair - identification of histones and deamidated keratins

Sunil S Adav[1]*, Roopa S Subbaiaih[2], Swat Kim Kerk[2], Amelia Yilin Lee[2], Hui Ying Lai[3,4], Kee Woei Ng[3], Siu Kwan Sze[1], Artur Schmidtchen[2,5]

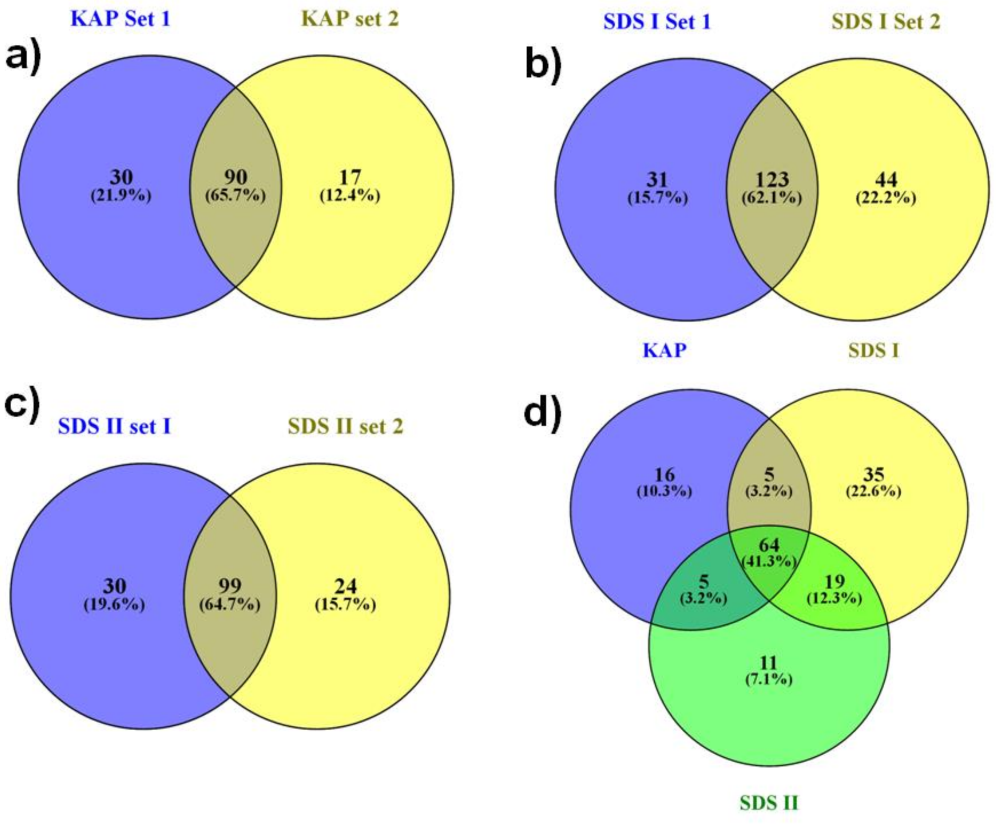

Figure S1. Venn diagram showing the overlap of proteins identified with protein scores more than 30 and more than 2 peptides in two replicates (set 1 and set 2) by different extraction methods. (a) Hair proteome extraction by urea. (b) Hair proteome extraction with SDSI. (c) Hair proteome extracted by SDSII and (d) overlap of proteins identified in all extraction methods (proteins identified in both replicates were considered).

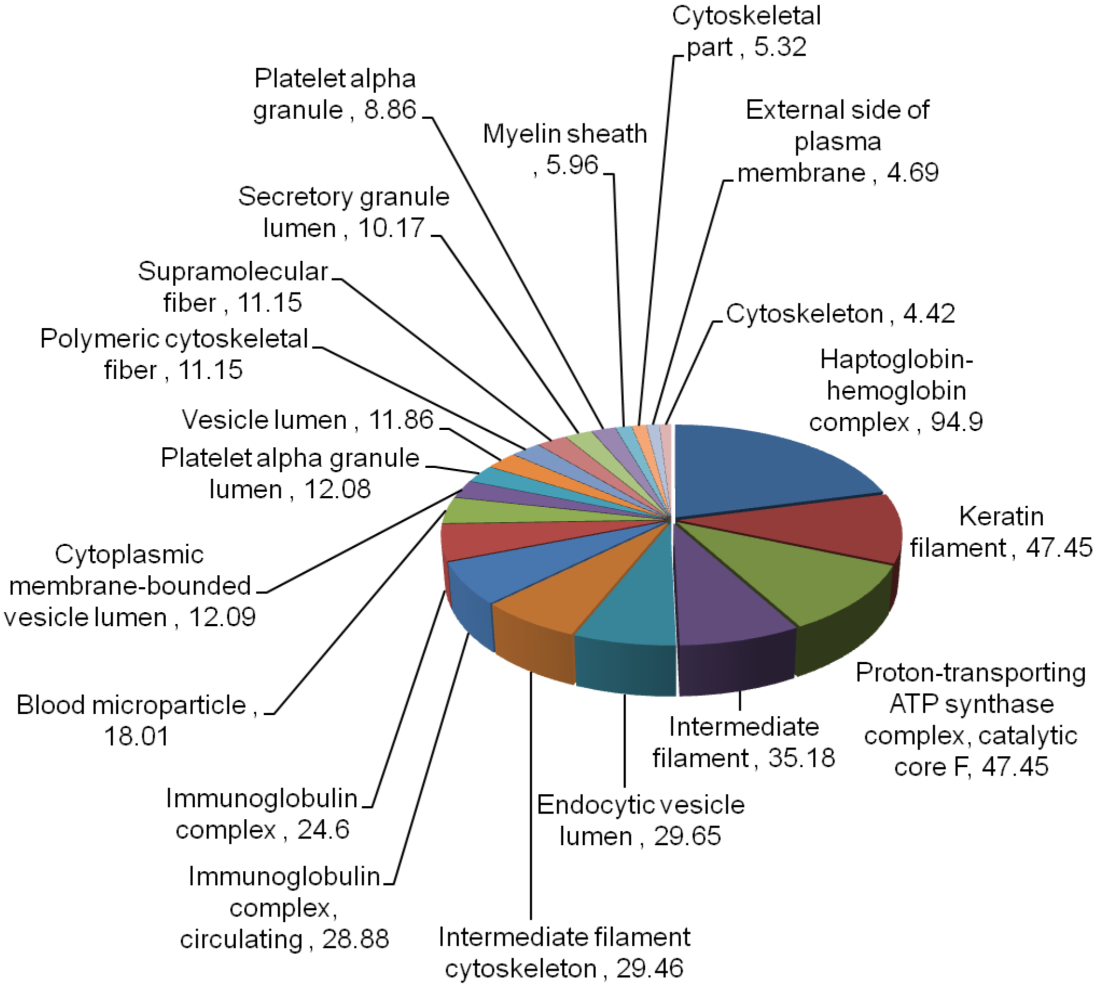

Figure S2. Cellular pathway enrichment analysis. The number in parentheses indicates the percentage of proteins. Cellular pathway enrichment was performed by PANTHER using a GO database released on 2016-06-22.

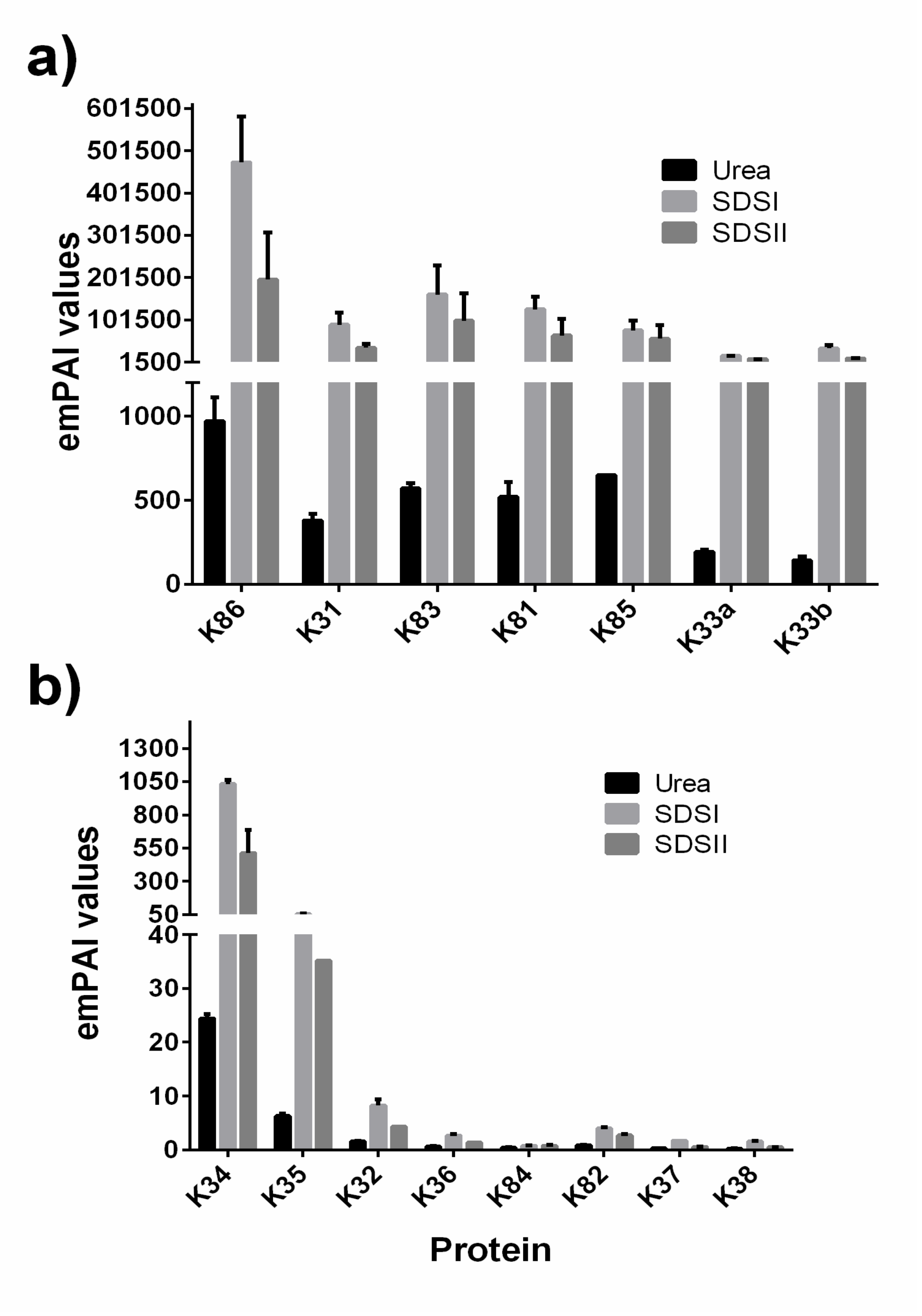

Figure S3. Abundances of cuticular keratin proteins in human hair shaft. Hair shaft proteins were extracted using Urea, SDSI and SDSII methods. (a) Cuticular keratin proteins. (b) Cuticular keratin proteins. Designated proteins (type I: K31-K38, type II: K81-K86) are according to new nomenclature.

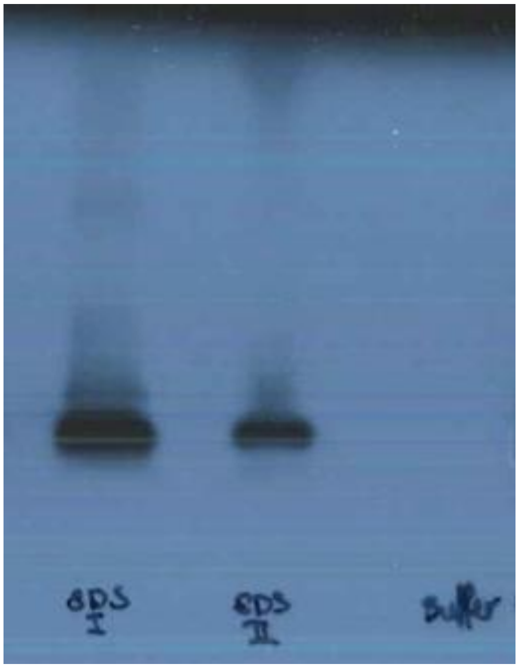

Figure S4. Full length western blot of Histone H2B. Extraction buffer was included as a control. The control run (extraction buffer) has been omitted in main figure for clarity as it does not add to the story.

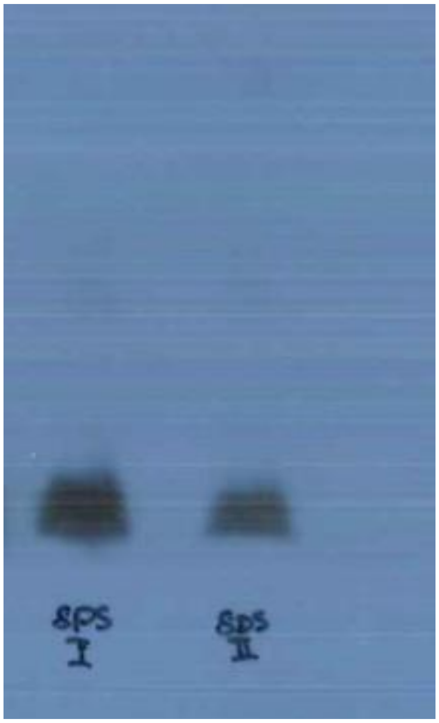

Figure S5. Full length western blot of Histone H4. Extraction buffer was included as a control. The empty late or control run (extraction buffer) has been omitted in main figure.

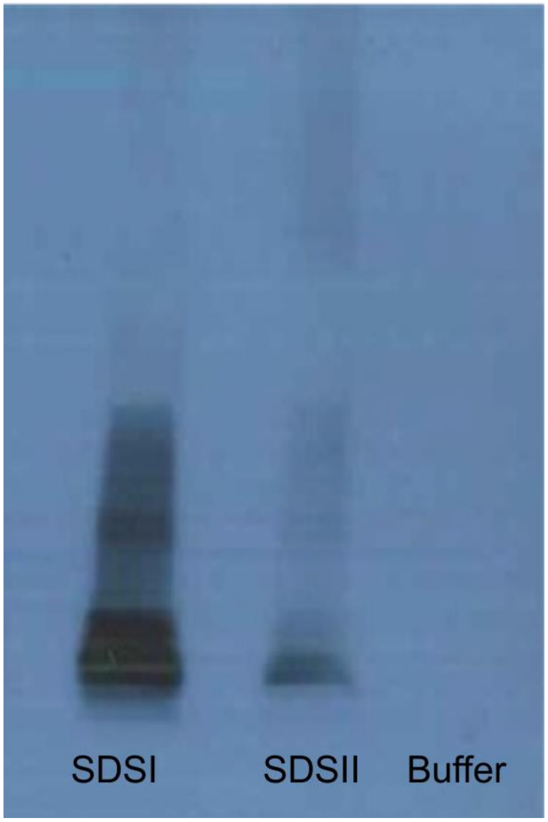

Figure S6. Full length western blot of Histone H3. Extraction buffer was included as a control. The empty late or control run (extraction buffer) has been omitted in main figure.

Mascot Search Results

Peptide View

MS/MS Fragmentation of **VSSVPSNSNVVVGTTNACAPSAR**
Found in **sp|O43790|KRT86_HUMAN**, Keratin, type II cuticular Hb6 OS=Homo sapiens GN=KRT86 PE=1 SV=1

Match to Query 33555: 2274.089322 from(759.037050,3+) intensity(3084413.0000) scans(10073) rtinseconds(1885) index(7751)
Title: 160219_Sunil_SDSI_A_Spectrum059859_scans__10073_RTINSECONDS=1885
Data file C:\\Sunil\\TKAP\\T\\T160219_Sunil_SDSI_A.mgf

Click mouse within plot area to zoom in by factor of two about that point
Or, Plot from 100 to 2000 Da Full range
Label all possible matches ○ Label matches used for scoring ◉

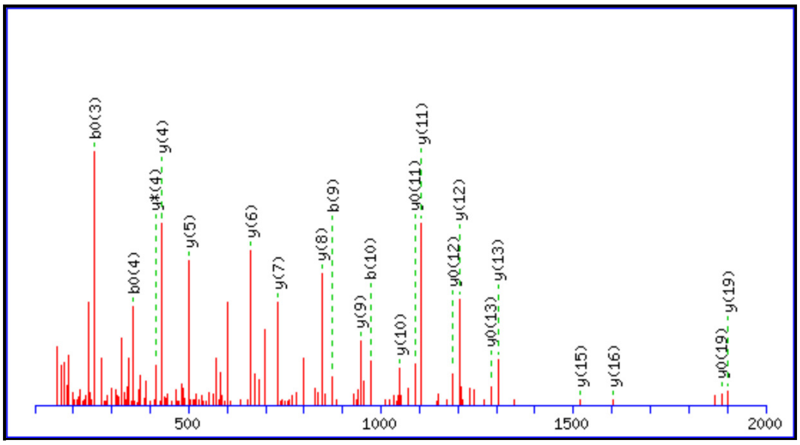

Monoisotopic mass of neutral peptide Mr(calc): 2274.0910
Fixed modifications: Carbamidomethyl (C) (apply to specified residues or termini only)
Variable modifications:
N9 : Deamidated (NQ)
Ions Score: 109 **Expect:** 3.1e-009
Matches : 22/246 fragment ions using 28 most intense peaks (help)

#	b	b++	b*	b*++	b0	b0++	Seq.	y	y++	y*	y*++	y0	y0++	#
1	100.0757	50.5415					V							23
2	187.1077	94.0575			169.0972	85.0522	S	2176.0299	1088.5186	2159.0033	1080.0053	2158.0193	1079.5133	22
3	274.1397	137.5735			256.1292	128.5682	S	2088.9979	1045.0026	2071.9713	1036.4893	2070.9873	1035.9973	21
4	373.2082	187.1077			355.1976	178.1024	V	2001.9658	1001.4866	1984.9393	992.9733	1983.9553	992.4813	20
5	470.2609	235.6341			452.2504	226.6288	P	***1902.8974***	951.9523	1885.8709	943.4391	1884.8869	942.9471	19
6	557.2930	279.1501			539.2824	270.1448	S	1805.8447	903.4260	1788.8181	894.9127	1787.8341	894.4207	18
7	671.3359	336.1716	654.3093	327.6583	653.3253	327.1663	N	1718.8126	859.9099	1701.7861	851.3967	1700.8021	850.9047	17
8	758.3679	379.6876	741.3414	371.1743	740.3573	370.6823	S	***1604.7697***	802.8885	1587.7431	794.3752	1586.7591	793.8832	16
9	**873.3948**	437.2011	856.3683	428.6878	855.3843	428.1958	N	***1517.7377***	759.3725	1500.7111	750.8592	1499.7271	750.3672	15
10	**972.4633**	486.7353	955.4367	478.2220	954.4527	477.7300	V	1402.7107	701.8590	1385.6842	693.3457	1384.7002	692.8537	14
11	1071.5317	536.2695	1054.5051	527.7562	1053.5211	527.2642	V	***1303.6423***	652.3248	1286.6158	643.8115	1285.6317	643.3195	13
12	1170.6001	585.8037	1153.5735	577.2904	1152.5895	576.7984	V	***1204.5739***	602.7906	1187.5473	594.2773	1186.5633	593.7853	12
13	1227.6216	614.3144	1210.5950	605.8011	1209.6110	605.3091	G	***1105.5055***	553.2564	1088.4789	544.7431	1087.4949	544.2511	11
14	1328.6692	664.8383	1311.6427	656.3250	1310.6587	655.8330	T	***1048.4840***	524.7456	1031.4575	516.2324	1030.4735	515.7404	10
15	1429.7169	715.3621	1412.6904	706.8488	1411.7063	706.3568	T	***947.4363***	474.2218	930.4098	465.7085	929.4258	465.2165	9
16	1543.7598	772.3836	1526.7333	763.8703	1525.7493	763.3783	N	***846.3887***	423.6980	829.3621	415.1847	828.3781	414.6927	8
17	1614.7970	807.9021	1597.7704	799.3888	1596.7864	798.8968	A	***732.3457***	366.6765	715.3192	358.1632	714.3352	357.6712	7
18	1774.8276	887.9174	1757.8011	879.4042	1756.8170	878.9122	C	***661.3086***	331.1579	644.2821	322.6447	643.2981	322.1527	6
19	1845.8647	923.4360	1828.8382	914.9227	1827.8542	914.4307	A	***501.2780***	251.1426	484.2514	242.6293	483.2674	242.1373	5
20	1942.9175	971.9624	1925.8909	963.4491	1924.9069	962.9571	P	***430.2409***	215.6241	413.2143	207.1108	412.2303	206.6188	4
21	2029.9495	1015.4784	2012.9230	1006.9651	2011.9389	1006.4731	S	333.1881	167.0977	316.1615	158.5844	315.1775	158.0924	3
22	2100.9866	1050.9969	2083.9601	1042.4837	2082.9761	1041.9917	A	246.1561	123.5817	229.1295	115.0684			2
23							R	175.1190	88.0631	158.0924	79.5498			1

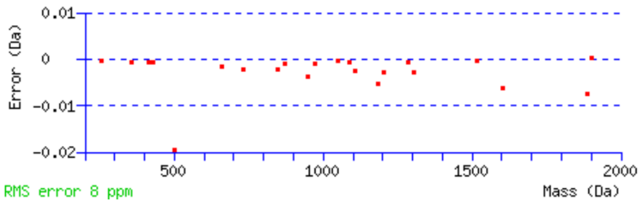

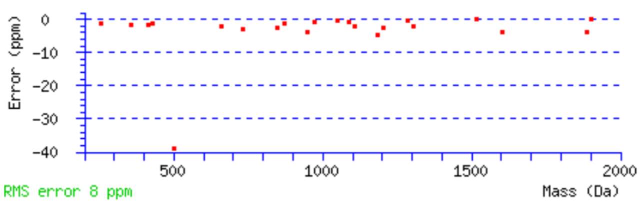

NCBI **BLAST** search of VSSVPSNSNVVVGTTNACAPSAR
(Parameters: blastp, nr protein database, expect=20000, no filter, PAM30)
Other BLAST web gateways

All matches to this query

Score	Mr(calc):	Delta	Sequence
109.2	2274.0910	-0.0017	VSSVPSNSNVVVGTTNACAPSAR
80.0	2274.0910	-0.0017	VSSVPSNSNVVVGTTNACAPSAR
57.3	2273.1070	0.9823	VSSVPSNSNVVVGTTNACAPSAR
22.7	2274.0910	-0.0017	VSSVPSNSNVVVGTTNACAPSAR

Mascot: http://www.matrixscience.com/

Mascot Search Results

Peptide View

MS/MS Fragmentation of **SGGVCGPSPPCITTVSVNESLLTPLNLEIDPNAQCVKQEEKEQIK**
Found in **sp|O43790|KRT86_HUMAN**, Keratin, type II cuticular Hb6 OS=Homo sapiens GN=KRT86 PE=1 SV=1

Match to Query 45141: 4939.424016 from(1235.863280,4+) intensity(3828749.0000) scans(15871) rtinseconds(2880) index(12536)
Title: 160219_Sunil_SDSI_A_Spectrum064672_scans__15871_RTINSECONDS=2880
Data file C:\\Sunil\\TKAP\\T\\T160219_Sunil_SDSI_A.mgf

Click mouse within plot area to zoom in by factor of two about that point
Or, Plot from 200 to 4400 Da Full range
Label all possible matches ○ Label matches used for scoring ◉

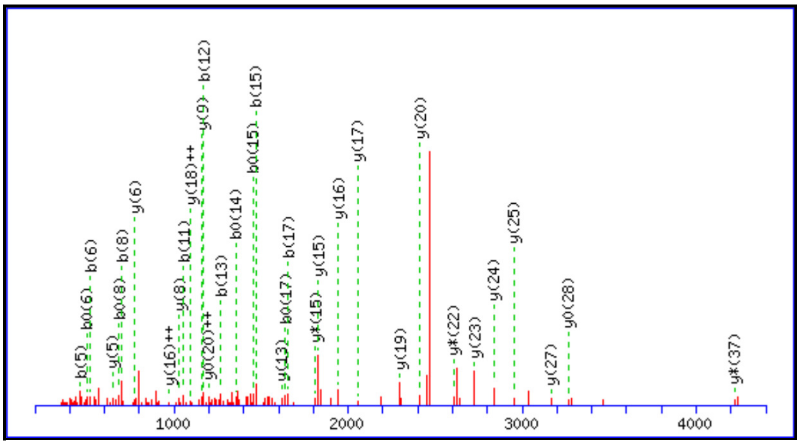

Monoisotopic mass of neutral peptide Mr(calc): 4937.3984
Fixed modifications: Carbamidomethyl (C) (apply to specified residues or termini only)
Variable modifications:
N18 : Deamidated (NQ)
N26 : Deamidated (NQ)
Ions Score: 51 **Expect:** 0.0015
Matches : 34/488 fragment ions using 85 most intense peaks (help)

#	b	b^{++}	b*	b^{*++}	b^0	b^{0++}	Seq.	y	y^{++}	y*	y^{*++}	y^0	y^{0++}	#
1	88.0393	44.5233			70.0287	35.5180	S							45
2	145.0608	73.0340			127.0502	64.0287	G	4851.3737	2426.1905	4834.3471	2417.6772	4833.3631	2417.1852	44
3	202.0822	101.5448			184.0717	92.5395	G	4794.3522	2397.6797	4777.3257	2389.1665	4776.3417	2388.6745	43
4	301.1506	151.0790			283.1401	142.0737	V	4737.3308	2369.1690	4720.3042	2360.6557	4719.3202	2360.1637	42
5	***461.1813***	231.0943			443.1707	222.0890	C	4638.2623	2319.6348	4621.2358	2311.1215	4620.2518	2310.6295	41
6	***518.2028***	259.6050			**500.1922**	250.5997	G	4478.2317	2239.6195	4461.2051	2231.1062	4460.2211	2230.6142	40
7	615.2555	308.1314			597.2450	299.1261	P	4421.2102	2211.1088	4404.1837	2202.5955	4403.1997	2202.1035	39
8	***702.2876***	351.6474			**684.2770**	342.6421	S	4324.1575	2162.5824	4307.1309	2154.0691	4306.1469	2153.5771	38
9	799.3403	400.1738			781.3298	391.1685	P	4237.1254	2119.0664	**4220.0989**	2110.5531	4219.1149	2110.0611	37
10	896.3931	448.7002			878.3825	439.6949	P	4140.0727	2070.5400	4123.0461	2062.0267	4122.0621	2061.5347	36
11	***1056.4237***	528.7155			1038.4132	519.7102	C	4043.0199	2022.0136	4025.9934	2013.5003	4025.0093	2013.0083	35
12	***1169.5078***	585.2575			1151.4972	576.2523	I	3882.9893	1941.9983	3865.9627	1933.4850	3864.9787	1932.9930	34
13	***1270.5555***	635.7814			1252.5449	626.7761	T	3769.9052	1885.4562	3752.8786	1876.9430	3751.8946	1876.4510	33
14	1371.6032	686.3052			**1353.5926**	677.2999	T	3668.8575	1834.9324	3651.8310	1826.4191	3650.8470	1825.9271	32
15	***1470.6716***	735.8394			**1452.6610**	726.8341	V	3567.8098	1784.4086	3550.7833	1775.8953	3549.7993	1775.4033	31
16	1557.7036	779.3554			1539.6930	770.3502	S	3468.7414	1734.8743	3451.7149	1726.3611	3450.7309	1725.8691	30
17	***1656.7720***	828.8896			**1638.7614**	819.8844	V	3381.7094	1691.3583	3364.6828	1682.8451	3363.6988	1682.3531	29
18	1771.7989	886.4031	1754.7724	877.8898	1753.7884	877.3978	N	3282.6410	1641.8241	3265.6144	1633.3109	**3264.6304**	1632.8188	28
19	1900.8415	950.9244	1883.8150	942.4111	1882.8310	941.9191	E	***3167.6140***	1584.3107	3150.5875	1575.7974	3149.6035	1575.3054	27
20	1987.8736	994.4404	1970.8470	985.9271	1969.8630	985.4351	S	3038.5714	1519.7894	3021.5449	1511.2761	3020.5609	1510.7841	26
21	2100.9576	1050.9825	2083.9311	1042.4692	2082.9471	1041.9772	L	***2951.5394***	1476.2733	2934.5129	1467.7601	2933.5289	1467.2681	25
22	2214.0417	1107.5245	2197.0151	1099.0112	2196.0311	1098.5192	L	***2838.4554***	1419.7313	2821.4288	1411.2180	2820.4448	1410.7260	24
23	2315.0894	1158.0483	2298.0628	1149.5351	2297.0788	1149.0430	T	***2725.3713***	1363.1893	2708.3447	1354.6760	2707.3607	1354.1840	23
24	2412.1421	1206.5747	2395.1156	1198.0614	2394.1316	1197.5694	P	2624.3236	1312.6654	**2607.2971**	1304.1522	2606.3130	1303.6602	22
25	2525.2262	1263.1167	2508.1997	1254.6035	2507.2156	1254.1115	L	2527.2708	1264.1391	2510.2443	1255.6258	2509.2603	1255.1338	21
26	2640.2531	1320.6302	2623.2266	1312.1169	2622.2426	1311.6249	N	***2414.1868***	1207.5970	2397.1602	1199.0838	2396.1762	**1198.5917**	20

27	2753.3372	1377.1722	2736.3107	1368.6590	2735.3266	1368.1670	L	2299.1598	1150.0836	2282.1333	1141.5703	2281.1493	1141.0783	19
28	2882.3798	1441.6935	2865.3533	1433.1803	2864.3692	1432.6883	E	2186.0758	1093.5415	2169.0492	1085.0283	2168.0652	1084.5362	18
29	2995.4639	1498.2356	2978.4373	1489.7223	2977.4533	1489.2303	I	2057.0332	1029.0202	2040.0066	1020.5070	2039.0226	1020.0149	17
30	3110.4908	1555.7490	3093.4643	1547.2358	3092.4802	1546.7438	D	1943.9491	972.4782	1926.9226	963.9649	1925.9386	963.4729	16
31	3207.5436	1604.2754	3190.5170	1595.7622	3189.5330	1595.2701	P	1828.9222	914.9647	1811.8956	906.4515	1810.9116	905.9594	15
32	3321.5865	1661.2969	3304.5600	1652.7836	3303.5759	1652.2916	N	1731.8694	866.4383	1714.8429	857.9251	1713.8588	857.4331	14
33	3392.6236	1696.8154	3375.5971	1688.3022	3374.6131	1687.8102	A	1617.8265	809.4169	1600.7999	800.9036	1599.8159	800.4116	13
34	3520.6822	1760.8447	3503.6556	1752.3315	3502.6716	1751.8395	Q	1546.7894	773.8983	1529.7628	765.3850	1528.7788	764.8930	12
35	3680.7128	1840.8601	3663.6863	1832.3468	3662.7023	1831.8548	C	1418.7308	709.8690	1401.7042	701.3558	1400.7202	700.8638	11
36	3779.7813	1890.3943	3762.7547	1881.8810	3761.7707	1881.3890	V	1258.7001	629.8537	1241.6736	621.3404	1240.6896	620.8484	10
37	3907.8762	1954.4417	3890.8497	1945.9285	3889.8657	1945.4365	K	1159.6317	580.3195	1142.6052	571.8062	1141.6212	571.3142	9
38	4035.9348	2018.4710	4018.9083	2009.9578	4017.9242	2009.4658	Q	1031.5368	516.2720	1014.5102	507.7587	1013.5262	507.2667	8
39	4164.9774	2082.9923	4147.9508	2074.4791	4146.9668	2073.9871	E	903.4782	452.2427	886.4516	443.7295	885.4676	443.2374	7
40	4294.0200	2147.5136	4276.9934	2139.0004	4276.0094	2138.5083	E	774.4356	387.7214	757.4090	379.2082	756.4250	378.7162	6
41	4422.1149	2211.5611	4405.0884	2203.0478	4404.1044	2202.5558	K	645.3930	323.2001	628.3665	314.6869	627.3824	314.1949	5
42	4551.1575	2276.0824	4534.1310	2267.5691	4533.1470	2267.0771	E	517.2980	259.1527	500.2715	250.6394	499.2875	250.1474	4
43	4679.2161	2340.1117	4662.1896	2331.5984	4661.2056	2331.1064	Q	388.2554	194.6314	371.2289	186.1181			3
44	4792.3002	2396.6537	4775.2736	2388.1405	4774.2896	2387.6484	I	260.1969	130.6021	243.1703	122.0888			2
45							K	147.1128	74.0600	130.0863	65.5468			1

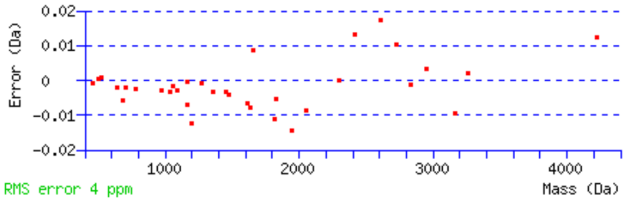

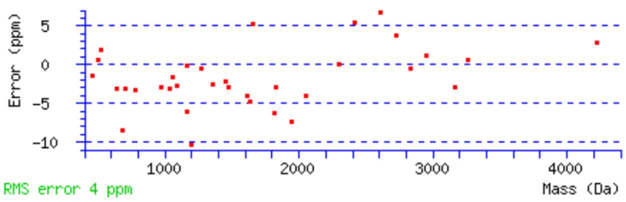

NCBI **BLAST** search of SGGVCGPSPPCITTVSVNESLLTPLNLEIDPNAQCVKQEEKEQIK
(Parameters: blastp, nr protein database, expect=20000, no filter, PAM30)
Other BLAST web gateways

All matches to this query

Score	Mr(calc):	Delta	Sequence
50.6	4937.3984	2.0256	SGGVCGPSPPCITTVSVNESLLTPLNLEIDPNAQCVKQEEKEQIK
42.0	4937.3984	2.0256	SGGVCGPSPPCITTVSVNESLLTPLNLEIDPNAQCVKQEEKEQIK
36.3	4937.3984	2.0256	SGGVCGPSPPCITTVSVNESLLTPLNLEIDPNAQCVKQEEKEQIK
34.0	4937.3984	2.0256	SGGVCGPSPPCITTVSVNESLLTPLNLEIDPNAQCVKQEEKEQIK
32.1	4937.3984	2.0256	SGGVCGPSPPCITTVSVNESLLTPLNLEIDPNAQCVKQEEKEQIK
31.1	4938.3824	1.0416	SGGVCGPSPPCITTVSVNESLLTPLNLEIDPNAQCVKQEEKEQIK
28.7	4937.3984	2.0256	SGGVCGPSPPCITTVSVNESLLTPLNLEIDPNAQCVKQEEKEQIK
27.2	4937.3984	2.0256	SGGVCGPSPPCITTVSVNESLLTPLNLEIDPNAQCVKQEEKEQIK
22.7	4937.3984	2.0256	SGGVCGPSPPCITTVSVNESLLTPLNLEIDPNAQCVKQEEKEQIK
16.8	4937.3984	2.0256	SGGVCGPSPPCITTVSVNESLLTPLNLEIDPNAQCVKQEEKEQIK

Mascot: http://www.matrixscience.com/

Mascot Search Results

Peptide View

MS/MS Fragmentation of **VSSVPSNSNVVVGTTNACAPSAR**
Found in **sp|O43790|KRT86_HUMAN**, Keratin, type II cuticular Hb6 OS=Homo sapiens GN=KRT86 PE=1 SV=1

Match to Query 33560: 2274.090222 from(759.037350,3+) intensity(5573453.5000) scans(8658) rtinseconds(1618) index(21867)
Title: 160219_Sunil_SDSI_A_Spectrum075171_scans__8658_RTINSECONDS=1618
Data file C:\\Sunil\\TKAP\\T\\T160219_Sunil_SDSI_A.mgf

Click mouse within plot area to zoom in by factor of two about that point
Or, Plot from 0 to 2400 Da Full range
Label all possible matches Label matches used for scoring

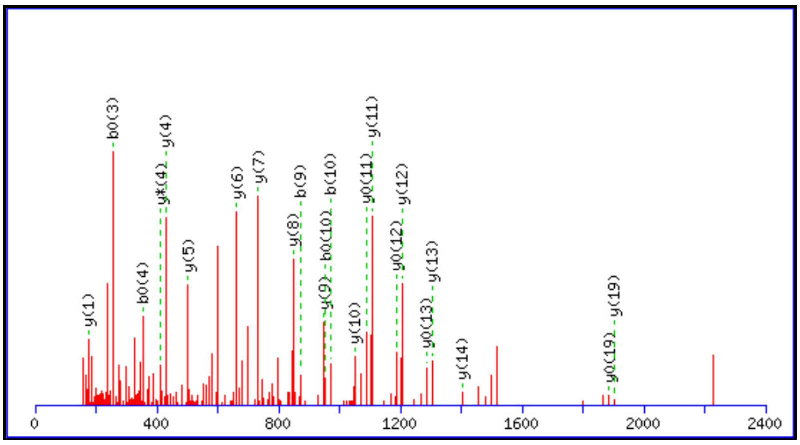

Monoisotopic mass of neutral peptide Mr(calc): 2274.0910
Fixed modifications: Carbamidomethyl (C) (apply to specified residues or termini only)
Variable modifications:
N16 : Deamidated (NQ)
Ions Score: 74 **Expect:** 9.6e-006
Matches : 23/246 fragment ions using 46 most intense peaks (help)

#	b	b^{++}	b^{*}	b^{*++}	b^{0}	b^{0++}	Seq.	y	y^{++}	y^{*}	y^{*++}	y^{0}	y^{0++}	#
1	100.0757	50.5415					V							23
2	187.1077	94.0575			169.0972	85.0522	S	2176.0299	1088.5186	2159.0033	1080.0053	2158.0193	1079.5133	22
3	274.1397	137.5735			256.1292	128.5682	S	2088.9979	1045.0026	2071.9713	1036.4893	2070.9873	1035.9973	21
4	373.2082	187.1077			355.1976	178.1024	V	2001.9658	1001.4866	1984.9393	992.9733	1983.9553	992.4813	20
5	470.2609	235.6341			452.2504	226.6288	P	***1902.8974***	951.9523	1885.8709	943.4391	1884.8869	942.9471	19
6	557.2930	279.1501			539.2824	270.1448	S	1805.8447	903.4260	1788.8181	894.9127	1787.8341	894.4207	18
7	671.3359	336.1716	654.3093	327.6583	653.3253	327.1663	N	1718.8126	859.9100	1701.7861	851.3967	1700.8021	850.9047	17
8	758.3679	379.6876	741.3414	371.1743	740.3573	370.6823	S	1604.7697	802.8885	1587.7431	794.3752	1586.7591	793.8832	16
9	**872.4108**	436.7091	855.3843	428.1958	854.4003	427.7038	N	1517.7377	759.3725	1500.7111	750.8592	1499.7271	750.3672	15
10	**971.4792**	486.2433	954.4527	477.7300	953.4687	477.2380	V	***1403.6947***	702.3510	1386.6682	693.8377	1385.6842	693.3457	14
11	1070.5477	535.7775	1053.5211	527.2642	1052.5371	526.7722	V	***1304.6263***	652.8168	1287.5998	644.3035	1286.6158	643.8115	13
12	1169.6161	585.3117	1152.5895	576.7984	1151.6055	576.3064	V	***1205.5579***	603.2826	1188.5314	594.7693	1187.5473	594.2773	12
13	1226.6375	613.8224	1209.6110	605.3091	1208.6270	604.8171	G	***1106.4895***	553.7484	1089.4630	545.2351	1088.4789	544.7431	11
14	1327.6852	664.3462	1310.6587	655.8330	1309.6747	655.3410	T	***1049.4680***	525.2377	1032.4415	516.7244	1031.4575	516.2324	10
15	1428.7329	714.8701	1411.7063	706.3568	1410.7223	705.8648	T	***948.4204***	474.7138	931.3938	466.2005	930.4098	465.7085	9
16	1543.7598	772.3836	1526.7333	763.8703	1525.7493	763.3783	N	***847.3727***	424.1900	830.3461	415.6767	829.3621	415.1847	8
17	1614.7970	807.9021	1597.7704	799.3888	1596.7864	798.8968	A	***732.3457***	366.6765	715.3192	358.1632	714.3352	357.6712	7
18	1774.8276	887.9174	1757.8011	879.4042	1756.8170	878.9122	C	***661.3086***	331.1579	644.2821	322.6447	643.2981	322.1527	6
19	1845.8647	923.4360	1828.8382	914.9227	1827.8542	914.4307	A	***501.2780***	251.1426	484.2514	242.6293	483.2674	242.1373	5
20	1942.9175	971.9624	1925.8909	963.4491	1924.9069	962.9571	P	***430.2409***	215.6241	413.2143	207.1108	412.2303	206.6188	4
21	2029.9495	1015.4784	2012.9230	1006.9651	2011.9389	1006.4731	S	333.1881	167.0977	316.1615	158.5844	315.1775	158.0924	3
22	2100.9866	1050.9970	2083.9601	1042.4837	2082.9761	1041.9917	A	246.1561	123.5817	229.1295	115.0684			2
23							R	***175.1190***	88.0631	158.0924	79.5498			1

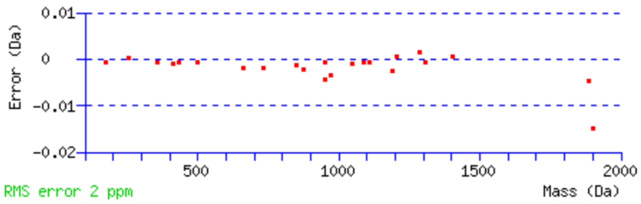

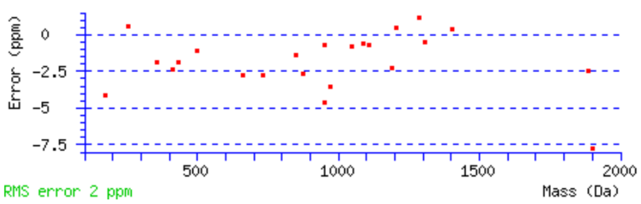

NCBI **BLAST** search of VSSVPSNSNVVVGTTNACAPSAR
(Parameters: blastp, nr protein database, expect=20000, no filter, PAM30)
Other BLAST web gateways

All matches to this query

Score	Mr(calc):	Delta	Sequence
74.2	2274.0910	-0.0008	VSSVPSNSNVVVGTTNACAPSAR
50.0	2274.0910	-0.0008	VSSVPSNSNVVVGTTNACAPSAR
50.0	2274.0910	-0.0008	VSSVPSNSNVVVGTTNACAPSAR
48.3	2273.1070	0.9832	VSSVPSNSNVVVGTTNACAPSAR

Mascot: http://www.matrixscience.com/

Mascot Search Results

Peptide View

MS/MS Fragmentation of **VSSVPSNSNVVVGTTNACAPSAR**
Found in **sp|O43790|KRT86_HUMAN**, Keratin, type II cuticular Hb6 OS=Homo sapiens GN=KRT86 PE=1 SV=1

Match to Query 33572: 2274.092868 from(1138.053710,2+) intensity(7947326.5000) scans(9464) rtinseconds(1781) index(7202)
Title: 160219_Sunil_SDSI_A_Spectrum059310_scans__9464_RTINSECONDS=1781
Data file C:\\Sunil\\TKAP\\T\\T160219_Sunil_SDSI_A.mgf

Click mouse within plot area to zoom in by factor of two about that point
Or, Plot from 100 to 2100 Da Full range
Label all possible matches ○ Label matches used for scoring ◉

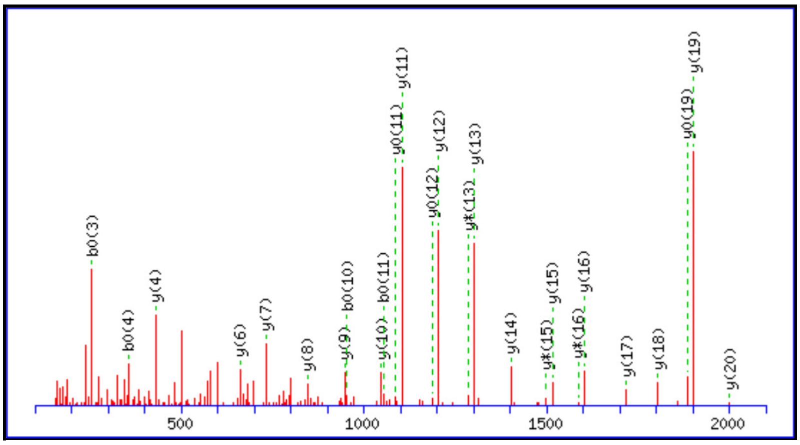

Monoisotopic mass of neutral peptide Mr(calc): 2274.0910
Fixed modifications: Carbamidomethyl (C) (apply to specified residues or termini only)
Variable modifications:
N7 : Deamidated (NQ)
Ions Score: 124 **Expect:** 1e-010
Matches : 26/246 fragment ions using 36 most intense peaks (help)

#	b	b^{++}	b*	b^{*++}	b^{0}	b^{0++}	Seq.	y	y^{++}	y*	y^{*++}	y^{0}	y^{0++}	#
1	100.0757	50.5415					V							23
2	187.1077	94.0575			169.0972	85.0522	S	2176.0299	1088.5186	2159.0033	1080.0053	2158.0193	1079.5133	22
3	274.1397	137.5735			256.1292	128.5682	S	2088.9979	1045.0026	2071.9713	1036.4893	2070.9873	1035.9973	21
4	373.2082	187.1077			355.1976	178.1024	V	***2001.9658***	1001.4866	1984.9393	992.9733	1983.9553	992.4813	20
5	470.2609	235.6341			452.2504	226.6288	P	***1902.8974***	951.9523	1885.8709	943.4391	1884.8869	942.9471	19
6	557.2930	279.1501			539.2824	270.1448	S	***1805.8447***	903.4260	1788.8181	894.9127	1787.8341	894.4207	18
7	672.3199	336.6636	655.2933	328.1503	654.3093	327.6583	N	***1718.8126***	859.9099	1701.7861	851.3967	1700.8021	850.9047	17
8	759.3519	380.1796	742.3254	371.6663	741.3414	371.1743	S	***1603.7857***	802.3965	1586.7591	793.8832	1585.7751	793.3912	16
9	873.3948	437.2011	856.3683	428.6878	855.3843	428.1958	N	***1516.7537***	758.8805	1499.7271	750.3672	1498.7431	749.8752	15
10	972.4633	486.7353	955.4367	478.2220	954.4527	477.7300	V	***1402.7107***	701.8590	1385.6842	693.3457	1384.7002	692.8537	14
11	1071.5317	536.2695	1054.5051	527.7562	1053.5211	527.2642	V	***1303.6423***	652.3248	1286.6158	643.8115	1285.6317	643.3195	13
12	1170.6001	585.8037	1153.5735	577.2904	1152.5895	576.7984	V	***1204.5739***	602.7906	1187.5473	594.2773	1186.5633	593.7853	12
13	1227.6216	614.3144	1210.5950	605.8011	1209.6110	605.3091	G	***1105.5055***	553.2564	1088.4789	544.7431	1087.4949	544.2511	11
14	1328.6692	664.8383	1311.6427	656.3250	1310.6587	655.8330	T	***1048.4840***	524.7456	1031.4575	516.2324	1030.4735	515.7404	10
15	1429.7169	715.3621	1412.6904	706.8488	1411.7063	706.3568	T	***947.4363***	474.2218	930.4098	465.7085	929.4258	465.2165	9
16	1543.7598	772.3836	1526.7333	763.8703	1525.7493	763.3783	N	***846.3887***	423.6980	829.3621	415.1847	828.3781	414.6927	8
17	1614.7970	807.9021	1597.7704	799.3888	1596.7864	798.8968	A	***732.3457***	366.6765	715.3192	358.1632	714.3352	357.6712	7
18	1774.8276	887.9174	1757.8011	879.4042	1756.8170	878.9122	C	***661.3086***	331.1579	644.2821	322.6447	643.2981	322.1527	6
19	1845.8647	923.4360	1828.8382	914.9227	1827.8542	914.4307	A	501.2780	251.1426	484.2514	242.6293	483.2674	242.1373	5
20	1942.9175	971.9624	1925.8909	963.4491	1924.9069	962.9571	P	***430.2409***	215.6241	413.2143	207.1108	412.2303	206.6188	4
21	2029.9495	1015.4784	2012.9230	1006.9651	2011.9389	1006.4731	S	333.1881	167.0977	316.1615	158.5844	315.1775	158.0924	3
22	2100.9866	1050.9969	2083.9601	1042.4837	2082.9761	1041.9917	A	246.1561	123.5817	229.1295	115.0684			2
23							R	175.1190	88.0631	158.0924	79.5498			1

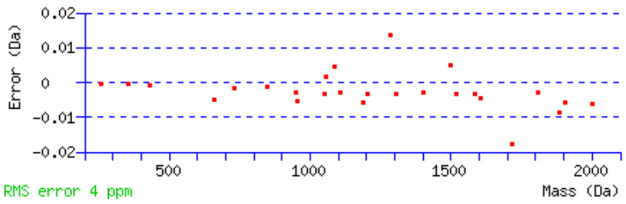

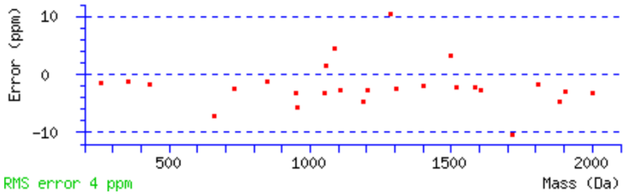

NCBI **BLAST** search of VSSVPSNSNVVVGTTNACAPSAR
(Parameters: blastp, nr protein database, expect=20000, no filter, PAM30)
Other BLAST web gateways

All matches to this query

Score	Mr(calc):	Delta	Sequence
124.0	2274.0910	0.0018	VSSVPSNSNVVVGTTNACAPSAR
95.3	2274.0910	0.0018	VSSVPSNSNVVVGTTNACAPSAR
90.7	2273.1070	0.9858	VSSVPSNSNVVVGTTNACAPSAR
18.2	2274.0910	0.0018	VSSVPSNSNVVVGTTNACAPSAR

Mascot: http://www.matrixscience.com/

Mascot Search Results

Peptide View

MS/MS Fragmentation of **VSSVPSNSNVVVGTTNACAPSAR**
Found in **sp|O43790|KRT86_HUMAN**, Keratin, type II cuticular Hb6 OS=Homo sapiens GN=KRT86 PE=1 SV=1

Match to Query 33572: 2274.092868 from(1138.053710,2+) intensity(7947326.5000) scans(9464) rtinseconds(1781) index(7202)
Title: 160219_Sunil_SDSI_A_Spectrum059310_scans__9464_RTINSECONDS=1781
Data file C:\\Sunil\\TKAP\\T\\T160219_Sunil_SDSI_A.mgf

Click mouse within plot area to zoom in by factor of two about that point
Or, Plot from 100 to 2100 Da Full range
Label all possible matches ○ Label matches used for scoring ◉

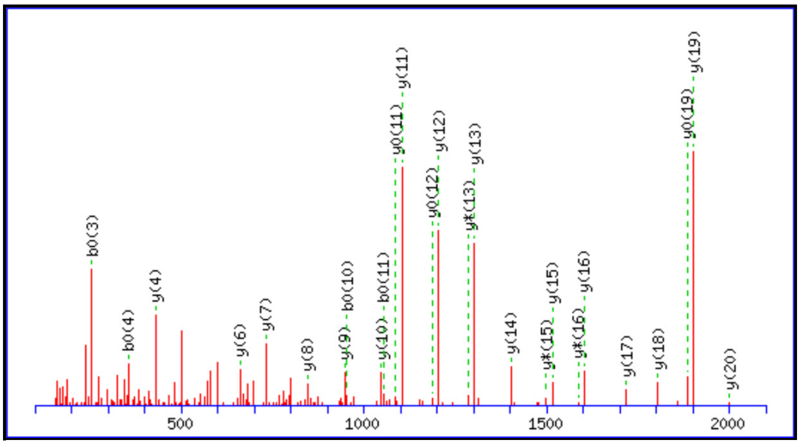

Monoisotopic mass of neutral peptide Mr(calc): 2274.0910
Fixed modifications: Carbamidomethyl (C) (apply to specified residues or termini only)
Variable modifications:
N7 : Deamidated (NQ)
Ions Score: 124 **Expect:** 1e-010
Matches : 26/246 fragment ions using 36 most intense peaks (help)

#	b	b++	b*	b*++	b0	b0++	Seq.	y	y++	y*	y*++	y0	y0++	#
1	100.0757	50.5415					V							23
2	187.1077	94.0575			169.0972	85.0522	S	2176.0299	1088.5186	2159.0033	1080.0053	2158.0193	1079.5133	22
3	274.1397	137.5735			256.1292	128.5682	S	2088.9979	1045.0026	2071.9713	1036.4893	2070.9873	1035.9973	21
4	373.2082	187.1077			355.1976	178.1024	V	2001.9658	1001.4866	1984.9393	992.9733	1983.9553	992.4813	20
5	470.2609	235.6341			452.2504	226.6288	P	1902.8974	951.9523	1885.8709	943.4391	1884.8869	942.9471	19
6	557.2930	279.1501			539.2824	270.1448	S	1805.8447	903.4260	1788.8181	894.9127	1787.8341	894.4207	18
7	672.3199	336.6636	655.2933	328.1503	654.3093	327.6583	N	1718.8126	859.9099	1701.7861	851.3967	1700.8021	850.9047	17
8	759.3519	380.1796	742.3254	371.6663	741.3414	371.1743	S	1603.7857	802.3965	1586.7591	793.8832	1585.7751	793.3912	16
9	873.3948	437.2011	856.3683	428.6878	855.3843	428.1958	N	1516.7537	758.8805	1499.7271	750.3672	1498.7431	749.8752	15
10	972.4633	486.7353	955.4367	478.2220	954.4527	477.7300	V	1402.7107	701.8590	1385.6842	693.3457	1384.7002	692.8537	14
11	1071.5317	536.2695	1054.5051	527.7562	1053.5211	527.2642	V	1303.6423	652.3248	1286.6158	643.8115	1285.6317	643.3195	13
12	1170.6001	585.8037	1153.5735	577.2904	1152.5895	576.7984	V	1204.5739	602.7906	1187.5473	594.2773	1186.5633	593.7853	12
13	1227.6216	614.3144	1210.5950	605.8011	1209.6110	605.3091	G	1105.5055	553.2564	1088.4789	544.7431	1087.4949	544.2511	11
14	1328.6692	664.8383	1311.6427	656.3250	1310.6587	655.8330	T	1048.4840	524.7456	1031.4575	516.2324	1030.4735	515.7404	10
15	1429.7169	715.3621	1412.6904	706.8488	1411.7063	706.3568	T	947.4363	474.2218	930.4098	465.7085	929.4258	465.2165	9
16	1543.7598	772.3836	1526.7333	763.8703	1525.7493	763.3783	N	846.3887	423.6980	829.3621	415.1847	828.3781	414.6927	8
17	1614.7970	807.9021	1597.7704	799.3888	1596.7864	798.8968	A	732.3457	366.6765	715.3192	358.1632	714.3352	357.6712	7
18	1774.8276	887.9174	1757.8011	879.4042	1756.8170	878.9122	C	661.3086	331.1579	644.2821	322.6447	643.2981	322.1527	6
19	1845.8647	923.4360	1828.8382	914.9227	1827.8542	914.4307	A	501.2780	251.1426	484.2514	242.6293	483.2674	242.1373	5
20	1942.9175	971.9624	1925.8909	963.4491	1924.9069	962.9571	P	430.2409	215.6241	413.2143	207.1108	412.2303	206.6188	4
21	2029.9495	1015.4784	2012.9230	1006.9651	2011.9389	1006.4731	S	333.1881	167.0977	316.1615	158.5844	315.1775	158.0924	3
22	2100.9866	1050.9969	2083.9601	1042.4837	2082.9761	1041.9917	A	246.1561	123.5817	229.1295	115.0684			2
23							R	175.1190	88.0631	158.0924	79.5498			1

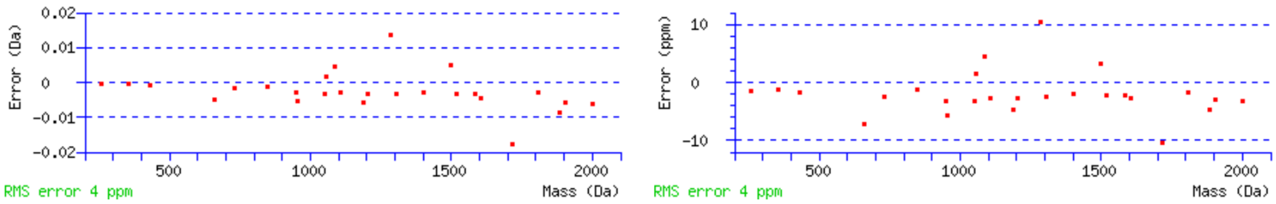

NCBI **BLAST** search of VSSVPSNSNVVVGTTNACAPSAR
(Parameters: blastp, nr protein database, expect=20000, no filter, PAM30)
Other BLAST web gateways

All matches to this query

Score	Mr(calc):	Delta	Sequence
124.0	2274.0910	0.0018	VSSVPSNSNVVVGTTNACAPSAR
95.3	2274.0910	0.0018	VSSVPSNSNVVVGTTNACAPSAR
90.7	2273.1070	0.9858	VSSVPSNSNVVVGTTNACAPSAR
18.2	2274.0910	0.0018	VSSVPSNSNVVVGTTNACAPSAR

Mascot: http://www.matrixscience.com/

Mascot Search Results

Peptide View

MS/MS Fragmentation of **VSSVPSNSNVVVGTTNACAPSAR**
Found in **sp|O43790|KRT86_HUMAN**, Keratin, type II cuticular Hb6 OS=Homo sapiens GN=KRT86 PE=1 SV=1

Match to Query 33577: 2274.094828 from(1138.054690,2+) intensity(1214021.1250) scans(8454) rtinseconds(1604) index(36946)
Title: 160219_Sunil_SDSI_A_Spectrum091531_scans__8454_RTINSECONDS=1604
Data file C:\\Sunil\\TKAP\\T\\T160219_Sunil_SDSI_A.mgf

Click mouse within plot area to zoom in by factor of two about that point
Or, Plot from 100 to 2000 Da Full range
Label all possible matches Label matches used for scoring

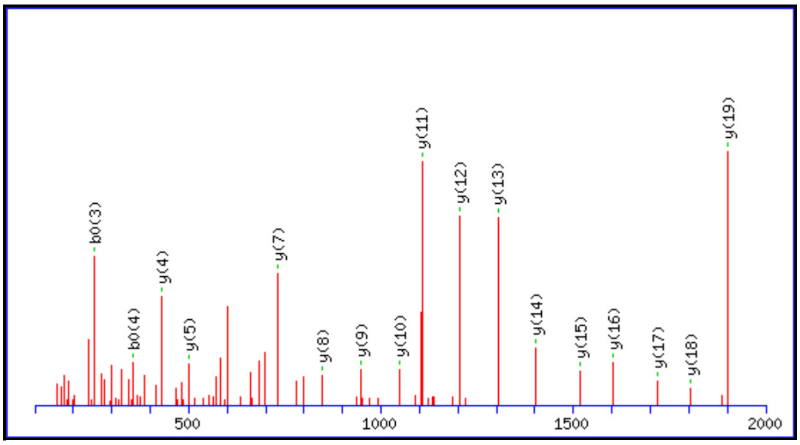

Monoisotopic mass of neutral peptide Mr(calc): 2274.0910
Fixed modifications: Carbamidomethyl (C) (apply to specified residues or termini only)
Variable modifications:
N16 : Deamidated (NQ)
Ions Score: 145 **Expect:** 8.2e-013
Matches : 17/246 fragment ions using 18 most intense peaks (help)

#	b	b^{++}	b*	b^{*++}	b^0	b^{0++}	Seq.	y	y^{++}	y*	y^{*++}	y^0	y^{0++}	#
1	100.0757	50.5415					V							23
2	187.1077	94.0575			169.0972	85.0522	S	2176.0299	1088.5186	2159.0033	1080.0053	2158.0193	1079.5133	22
3	274.1397	137.5735			256.1292	128.5682	S	2088.9979	1045.0026	2071.9713	1036.4893	2070.9873	1035.9973	21
4	373.2082	187.1077			355.1976	178.1024	V	2001.9658	1001.4866	1984.9393	992.9733	1983.9553	992.4813	20
5	470.2609	235.6341			452.2504	226.6288	P	***1902.8974***	951.9523	1885.8709	943.4391	1884.8869	942.9471	19
6	557.2930	279.1501			539.2824	270.1448	S	***1805.8447***	903.4260	1788.8181	894.9127	1787.8341	894.4207	18
7	671.3359	336.1716	654.3093	327.6583	653.3253	327.1663	N	***1718.8126***	859.9100	1701.7861	851.3967	1700.8021	850.9047	17
8	758.3679	379.6876	741.3414	371.1743	740.3573	370.6823	S	***1604.7697***	802.8885	1587.7431	794.3752	1586.7591	793.8832	16
9	872.4108	436.7091	855.3843	428.1958	854.4003	427.7038	N	***1517.7377***	759.3725	1500.7111	750.8592	1499.7271	750.3672	15
10	971.4792	486.2433	954.4527	477.7300	953.4687	477.2380	V	***1403.6947***	702.3510	1386.6682	693.8377	1385.6842	693.3457	14
11	1070.5477	535.7775	1053.5211	527.2642	1052.5371	526.7722	V	***1304.6263***	652.8168	1287.5998	644.3035	1286.6158	643.8115	13
12	1169.6161	585.3117	1152.5895	576.7984	1151.6055	576.3064	V	***1205.5579***	603.2826	1188.5314	594.7693	1187.5473	594.2773	12
13	1226.6375	613.8224	1209.6110	605.3091	1208.6270	604.8171	G	***1106.4895***	553.7484	1089.4630	545.2351	1088.4789	544.7431	11
14	1327.6852	664.3462	1310.6587	655.8330	1309.6747	655.3410	T	***1049.4680***	525.2377	1032.4415	516.7244	1031.4575	516.2324	10
15	1428.7329	714.8701	1411.7063	706.3568	1410.7223	705.8648	T	***948.4204***	474.7138	931.3938	466.2005	930.4098	465.7085	9
16	1543.7598	772.3836	1526.7333	763.8703	1525.7493	763.3783	N	***847.3727***	424.1900	830.3461	415.6767	829.3621	415.1847	8
17	1614.7970	807.9021	1597.7704	799.3888	1596.7864	798.8968	A	***732.3457***	366.6765	715.3192	358.1632	714.3352	357.6712	7
18	1774.8276	887.9174	1757.8011	879.4042	1756.8170	878.9122	C	661.3086	331.1579	644.2821	322.6447	643.2981	322.1527	6
19	1845.8647	923.4360	1828.8382	914.9227	1827.8542	914.4307	A	***501.2780***	251.1426	484.2514	242.6293	483.2674	242.1373	5
20	1942.9175	971.9624	1925.8909	963.4491	1924.9069	962.9571	P	***430.2409***	215.6241	413.2143	207.1108	412.2303	206.6188	4
21	2029.9495	1015.4784	2012.9230	1006.9651	2011.9389	1006.4731	S	333.1881	167.0977	316.1615	158.5844	315.1775	158.0924	3
22	2100.9866	1050.9970	2083.9601	1042.4837	2082.9761	1041.9917	A	246.1561	123.5817	229.1295	115.0684			2
23							R	175.1190	88.0631	158.0924	79.5498			1

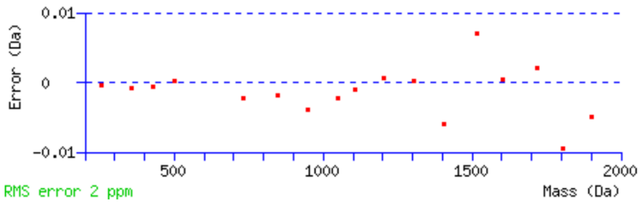

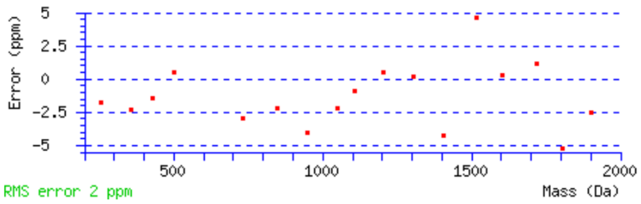

NCBI **BLAST** search of VSSVPSNSNVVVGTTNACAPSAR
(Parameters: blastp, nr protein database, expect=20000, no filter, PAM30)
Other BLAST web gateways

All matches to this query

Score	Mr(calc):	Delta	Sequence
145.0	2274.0910	0.0038	VSSVPSNSNVVVGTTNACAPSAR
106.8	2274.0910	0.0038	VSSVPSNSNVVVGTTNACAPSAR
81.6	2274.0910	0.0038	VSSVPSNSNVVVGTTNACAPSAR
50.1	2273.1070	0.9878	VSSVPSNSNVVVGTTNACAPSAR

Mascot: http://www.matrixscience.com/

Mascot Search Results

Peptide View

MS/MS Fragmentation of **VSSVPSNSNVVVGTTNACAPSAR**
Found in **sp|O43790|KRT86_HUMAN**, Keratin, type II cuticular Hb6 OS=Homo sapiens GN=KRT86 PE=1 SV=1

Match to Query 33597: 2275.077008 from(1138.545780,2+) intensity(2238620.2500) scans(9841) rtinseconds(1845) index(7545)
Title: 160219_Sunil_SDSI_A_Spectrum059653_scans__9841_RTINSECONDS=1845
Data file C:\\Sunil\\TKAP\\T\\T160219_Sunil_SDSI_A.mgf

Click mouse within plot area to zoom in by factor of two about that point
Or, Plot from 100 to 2000 Da Full range
Label all possible matches ○ Label matches used for scoring ◉

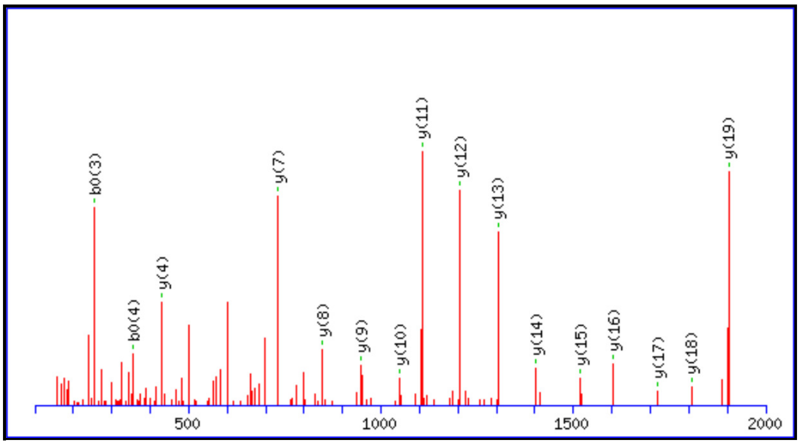

Monoisotopic mass of neutral peptide Mr(calc): 2275.0750
Fixed modifications: Carbamidomethyl (C) (apply to specified residues or termini only)
Variable modifications:
N7 : Deamidated (NQ)
N16 : Deamidated (NQ)
Ions Score: 126 **Expect:** 5.7e-011
Matches : 16/246 fragment ions using 18 most intense peaks (help)

#	b	b^{++}	b*	b^{*++}	b^{0}	b^{0++}	Seq.	y	y^{++}	y*	y^{*++}	y^{0}	y^{0++}	#
1	100.0757	50.5415					V							23
2	187.1077	94.0575			169.0972	85.0522	S	2177.0139	1089.0106	2159.9874	1080.4973	2159.0033	1080.0053	22
3	274.1397	137.5735			256.1292	128.5682	S	2089.9819	1045.4946	2072.9553	1036.9813	2071.9713	1036.4893	21
4	373.2082	187.1077			355.1976	178.1024	V	2002.9498	1001.9786	1985.9233	993.4653	1984.9393	992.9733	20
5	470.2609	235.6341			452.2504	226.6288	P	***1903.8814***	952.4444	1886.8549	943.9311	1885.8709	943.4391	19
6	557.2930	279.1501			539.2824	270.1448	S	***1806.8287***	903.9180	1789.8021	895.4047	1788.8181	894.9127	18
7	672.3199	336.6636	655.2933	328.1503	654.3093	327.6583	N	***1719.7966***	860.4020	1702.7701	851.8887	1701.7861	851.3967	17
8	759.3519	380.1796	742.3254	371.6663	741.3414	371.1743	S	***1604.7697***	802.8885	1587.7431	794.3752	1586.7591	793.8832	16
9	873.3948	437.2011	856.3683	428.6878	855.3843	428.1958	N	***1517.7377***	759.3725	1500.7111	750.8592	1499.7271	750.3672	15
10	972.4633	486.7353	955.4367	478.2220	954.4527	477.7300	V	***1403.6947***	702.3510	1386.6682	693.8377	1385.6842	693.3457	14
11	1071.5317	536.2695	1054.5051	527.7562	1053.5211	527.2642	V	***1304.6263***	652.8168	1287.5998	644.3035	1286.6158	643.8115	13
12	1170.6001	585.8037	1153.5735	577.2904	1152.5895	576.7984	V	***1205.5579***	603.2826	1188.5314	594.7693	1187.5473	594.2773	12
13	1227.6216	614.3144	1210.5950	605.8011	1209.6110	605.3091	G	***1106.4895***	553.7484	1089.4630	545.2351	1088.4789	544.7431	11
14	1328.6692	664.8383	1311.6427	656.3250	1310.6587	655.8330	T	***1049.4680***	525.2377	1032.4415	516.7244	1031.4575	516.2324	10
15	1429.7169	715.3621	1412.6904	706.8488	1411.7063	706.3568	T	***948.4204***	474.7138	931.3938	466.2005	930.4098	465.7085	9
16	1544.7439	772.8756	1527.7173	764.3623	1526.7333	763.8703	N	***847.3727***	424.1900	830.3461	415.6767	829.3621	415.1847	8
17	1615.7810	808.3941	1598.7544	799.8808	1597.7704	799.3888	A	***732.3457***	366.6765	715.3192	358.1632	714.3352	357.6712	7
18	1775.8116	888.4094	1758.7851	879.8962	1757.8011	879.4042	C	661.3086	331.1579	644.2821	322.6447	643.2981	322.1527	6
19	1846.8487	923.9280	1829.8222	915.4147	1828.8382	914.9227	A	501.2780	251.1426	484.2514	242.6293	483.2674	242.1373	5
20	1943.9015	972.4544	1926.8749	963.9411	1925.8909	963.4491	P	***430.2409***	215.6241	413.2143	207.1108	412.2303	206.6188	4
21	2030.9335	1015.9704	2013.9070	1007.4571	2012.9230	1006.9651	S	333.1881	167.0977	316.1615	158.5844	315.1775	158.0924	3
22	2101.9706	1051.4890	2084.9441	1042.9757	2083.9601	1042.4837	A	246.1561	123.5817	229.1295	115.0684			2
23							R	175.1190	88.0631	158.0924	79.5498			1

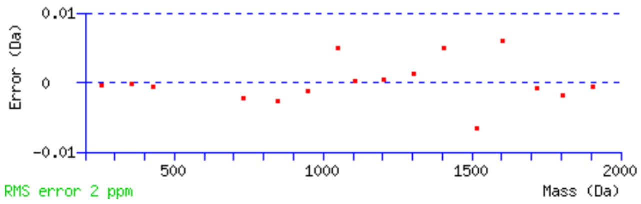

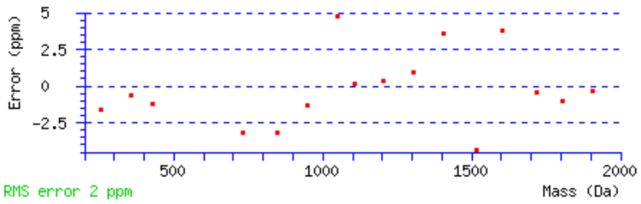

NCBI **BLAST** search of VSSVPSNSNVVVGTTNACAPSAR
(Parameters: blastp, nr protein database, expect=20000, no filter, PAM30)
Other BLAST web gateways

All matches to this query

Score	Mr(calc):	Delta	Sequence
126.1	2275.0750	0.0020	VSSVPSNSNVVVGTTNACAPSAR
92.8	2275.0750	0.0020	VSSVPSNSNVVVGTTNACAPSAR
77.3	2274.0910	0.9860	VSSVPSNSNVVVGTTNACAPSAR
76.8	2275.0750	0.0020	VSSVPSNSNVVVGTTNACAPSAR
58.3	2274.0910	0.9860	VSSVPSNSNVVVGTTNACAPSAR
58.2	2274.0910	0.9860	VSSVPSNSNVVVGTTNACAPSAR

Mascot: http://www.matrixscience.com/

Mascot Search Results

Peptide View

MS/MS Fragmentation of **ATAENEFVALKKDVDCAYLR**
Found in **sp|O43790|KRT86_HUMAN**, Keratin, type II cuticular Hb6 OS=Homo sapiens GN=KRT86 PE=1 SV=1

Match to Query 34214: 2313.129222 from(772.050350,3+) intensity(1684116.5000) scans(14036) rtinseconds(2541) index(26347)
Title: 160219_Sunil_SDSI_A_Spectrum079653_scans__14036_RTINSECONDS=2541
Data file C:\\Sunil\\TKAP\\T\\T160219_Sunil_SDSI_A.mgf

Click mouse within plot area to zoom in by factor of two about that point
Or, Plot from 0 to 2200 Da Full range
Label all possible matches Label matches used for scoring

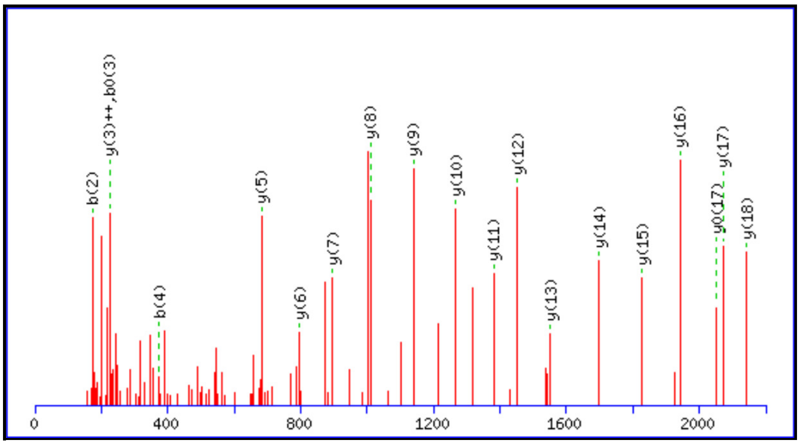

Monoisotopic mass of neutral peptide Mr(calc): 2313.1311
Fixed modifications: Carbamidomethyl (C) (apply to specified residues or termini only)
Variable modifications:
N5 : Deamidated (NQ)
Ions Score: 97 **Expect:** 4.9e-008
Matches : 19/208 fragment ions using 34 most intense peaks (help)

#	b	b++	b*	b*++	b0	b0++	Seq.	y	y++	y*	y*++	y0	y0++	#
1	72.0444	36.5258					A							20
2	**173.0921**	87.0497			155.0815	78.0444	T	2243.1013	1122.0543	2226.0747	1113.5410	2225.0907	1113.0490	19
3	244.1292	122.5682			226.1186	113.5629	A	**2142.0536**	1071.5304	2125.0270	1063.0172	2124.0430	1062.5251	18
4	**373.1718**	187.0895			355.1612	178.0842	E	**2071.0165**	1036.0119	2053.9899	1027.4986	2053.0059	1027.0066	17
5	488.1987	244.6030	471.1722	236.0897	470.1882	235.5977	N	**1941.9739**	971.4906	1924.9473	962.9773	1923.9633	962.4853	16
6	617.2413	309.1243	600.2148	300.6110	599.2307	300.1190	E	**1826.9469**	913.9771	1809.9204	905.4638	1808.9364	904.9718	15
7	764.3097	382.6585	747.2832	374.1452	746.2992	373.6532	F	**1697.9043**	849.4558	1680.8778	840.9425	1679.8938	840.4505	14
8	863.3781	432.1927	846.3516	423.6794	845.3676	423.1874	V	**1550.8359**	775.9216	1533.8094	767.4083	1532.8254	766.9163	13
9	934.4153	467.7113	917.3887	459.1980	916.4047	458.7060	A	**1451.7675**	726.3874	1434.7410	717.8741	1433.7569	717.3821	12
10	1047.4993	524.2533	1030.4728	515.7400	1029.4888	515.2480	L	**1380.7304**	690.8688	1363.7038	682.3556	1362.7198	681.8636	11
11	1175.5943	588.3008	1158.5677	579.7875	1157.5837	579.2955	K	**1267.6463**	634.3268	1250.6198	625.8135	1249.6358	625.3215	10
12	1303.6892	652.3483	1286.6627	643.8350	1285.6787	643.3430	K	**1139.5514**	570.2793	1122.5248	561.7660	1121.5408	561.2740	9
13	1418.7162	709.8617	1401.6896	701.3485	1400.7056	700.8564	D	**1011.4564**	506.2318	994.4299	497.7186	993.4458	497.2266	8
14	1517.7846	759.3959	1500.7581	750.8827	1499.7740	750.3907	V	**896.4295**	448.7184	879.4029	440.2051	878.4189	439.7131	7
15	1632.8115	816.9094	1615.7850	808.3961	1614.8010	807.9041	D	**797.3611**	399.1842	780.3345	390.6709	779.3505	390.1789	6
16	1792.8422	896.9247	1775.8156	888.4115	1774.8316	887.9195	C	**682.3341**	341.6707	665.3076	333.1574			5
17	1863.8793	932.4433	1846.8528	923.9300	1845.8687	923.4380	A	522.3035	261.6554	505.2769	253.1421			4
18	2026.9426	1013.9750	2009.9161	1005.4617	2008.9321	1004.9697	Y	451.2663	226.1368	434.2398	217.6235			3
19	2140.0267	1070.5170	2123.0002	1062.0037	2122.0161	1061.5117	L	288.2030	144.6051	271.1765	136.0919			2
20							R	175.1190	88.0631	158.0924	79.5498			1

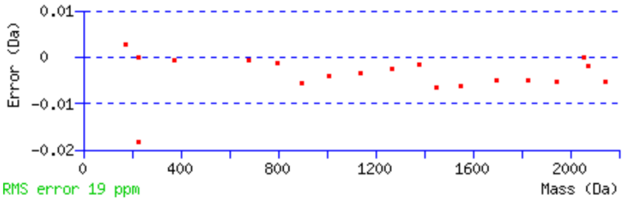

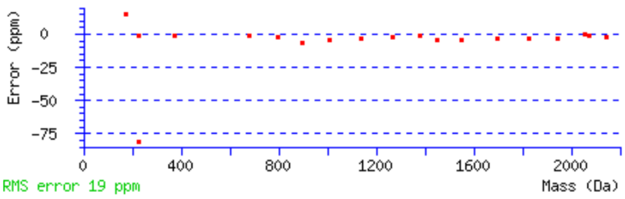

NCBI **BLAST** search of ATAENEFVALKKDVDCAYLR
(Parameters: blastp, nr protein database, expect=20000, no filter, PAM30)
Other BLAST web gateways

All matches to this query

Score	Mr(calc):	Delta	Sequence
97.2	2313.1311	-0.0018	ATAENEFVALKKDVDCAYLR
58.1	2312.1470	0.9822	ATAENEFVALKKDVDCAYLR

Mascot: http://www.matrixscience.com/

Mascot Search Results

Peptide View

MS/MS Fragmentation of **EYQEVMNSKLGLDIEIATYR**
Found in **sp|O43790|KRT86_HUMAN**, Keratin, type II cuticular Hb6 OS=Homo sapiens GN=KRT86 PE=1 SV=1

Match to Query 35276: 2372.158422 from(791.726750,3+) intensity(4437477.5000) scans(15387) rtinseconds(2797) index(12134)
Title: 160219_Sunil_SDSI_A_Spectrum064265_scans__15387_RTINSECONDS=2797
Data file C:\\Sunil\\TKAP\\T\\T160219_Sunil_SDSI_A.mgf

Click mouse within plot area to zoom in by factor of two about that point
Or, Plot from 0 to 2400 Da Full range
Label all possible matches ○ Label matches used for scoring ◉

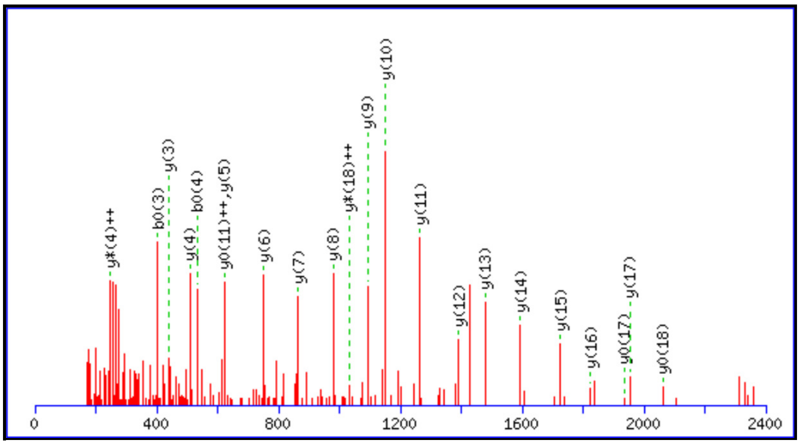

Monoisotopic mass of neutral peptide Mr(calc): 2372.1569
Fixed modifications: Carbamidomethyl (C) (apply to specified residues or termini only)
Variable modifications:
N7 : Deamidated (NQ)
Ions Score: 97 **Expect:** 5.6e-008
Matches : 22/220 fragment ions using 40 most intense peaks (help)

#	b	b++	b*	b*++	b0	b0++	Seq.	y	y++	y*	y*++	y0	y0++	#
1	130.0499	65.5286			112.0393	56.5233	E							20
2	293.1132	147.0602			275.1026	138.0550	Y	2244.1217	1122.5645	2227.0951	1114.0512	2226.1111	1113.5592	19
3	421.1718	211.0895	404.1452	202.5763	403.1612	202.0842	Q	2081.0583	1041.0328	2064.0318	1032.5195	2063.0478	1032.0275	18
4	550.2144	275.6108	533.1878	267.0975	532.2038	266.6055	E	***1952.9998***	977.0035	1935.9732	968.4902	1934.9892	967.9982	17
5	649.2828	325.1450	632.2562	316.6318	631.2722	316.1397	V	***1823.9572***	912.4822	1806.9306	903.9689	1805.9466	903.4769	16
6	780.3233	390.6653	763.2967	382.1520	762.3127	381.6600	M	***1724.8887***	862.9480	1707.8622	854.4347	1706.8782	853.9427	15
7	895.3502	448.1787	878.3237	439.6655	877.3396	439.1735	N	***1593.8483***	797.4278	1576.8217	788.9145	1575.8377	788.4225	14
8	982.3822	491.6948	965.3557	483.1815	964.3717	482.6895	S	***1478.8213***	739.9143	1461.7948	731.4010	1460.8108	730.9090	13
9	1110.4772	555.7422	1093.4507	547.2290	1092.4666	546.7370	K	***1391.7893***	696.3983	1374.7627	687.8850	1373.7787	687.3930	12
10	1223.5613	612.2843	1206.5347	603.7710	1205.5507	603.2790	L	***1263.6943***	632.3508	1246.6678	623.8375	1245.6838	623.3455	11
11	1280.5827	640.7950	1263.5562	632.2817	1262.5722	631.7897	G	***1150.6103***	575.8088	1133.5837	567.2955	1132.5997	566.8035	10
12	1393.6668	697.3370	1376.6402	688.8238	1375.6562	688.3318	L	***1093.5888***	547.2980	1076.5623	538.7848	1075.5782	538.2928	9
13	1508.6937	754.8505	1491.6672	746.3372	1490.6832	745.8452	D	***980.5047***	490.7560	963.4782	482.2427	962.4942	481.7507	8
14	1621.7778	811.3925	1604.7513	802.8793	1603.7672	802.3873	I	***865.4778***	433.2425	848.4512	424.7293	847.4672	424.2373	7
15	1750.8204	875.9138	1733.7938	867.4006	1732.8098	866.9086	E	***752.3937***	376.7005	735.3672	368.1872	734.3832	367.6952	6
16	1863.9045	932.4559	1846.8779	923.9426	1845.8939	923.4506	I	***623.3511***	312.1792	606.3246	303.6659	605.3406	303.1739	5
17	1934.9416	967.9744	1917.9150	959.4611	1916.9310	958.9691	A	***510.2671***	255.6372	493.2405	247.1239	492.2565	246.6319	4
18	2035.9893	1018.4983	2018.9627	1009.9850	2017.9787	1009.4930	T	***439.2300***	220.1186	422.2034	211.6053	421.2194	211.1133	3
19	2199.0526	1100.0299	2182.0260	1091.5167	2181.0420	1091.0246	Y	338.1823	169.5948	321.1557	161.0815			2
20							R	175.1190	88.0631	158.0924	79.5498			1

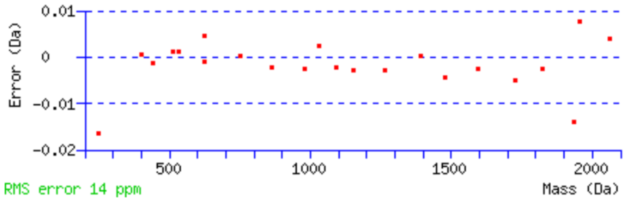

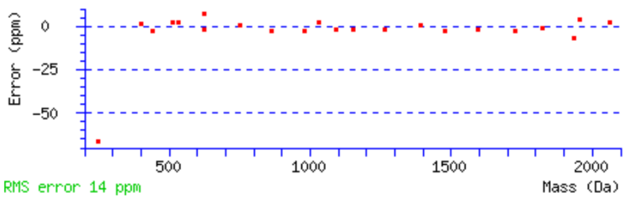

NCBI **BLAST** search of EYQEVMNSKLGLDIEIATYR
(Parameters: blastp, nr protein database, expect=20000, no filter, PAM30)
Other BLAST web gateways

All matches to this query

Score	Mr(calc):	Delta	Sequence
96.7	2372.1569	0.0015	EYQEVMNSKLGLDIEIATYR
48.3	2372.1569	0.0015	EYQEVMNSKLGLDIEIATYR
46.9	2371.1729	0.9855	EYQEVMNSKLGLDIEIATYR

Mascot: http://www.matrixscience.com/

Mascot Search Results

Peptide View

MS/MS Fragmentation of **EAECVEADSGRLASELNHVQEVLEGYK**
Found in **sp|O43790|KRT86_HUMAN**, Keratin, type II cuticular Hb6 OS=Homo sapiens GN=KRT86 PE=1 SV=1

Match to Query 40142: 3032.395902 from(1011.805910,3+) intensity(2680766.5000) scans(14529) rtinseconds(2651) index(11417)
Title: 160219_Sunil_SDSI_A_Spectrum063544_scans__14529_RTINSECONDS=2651
Data file C:\\Sunil\\TKAP\\T\\T160219_Sunil_SDSI_A.mgf

Click mouse within plot area to zoom in by factor of two about that point
Or, Plot from 200 to 3200 Da Full range
Label all possible matches Label matches used for scoring

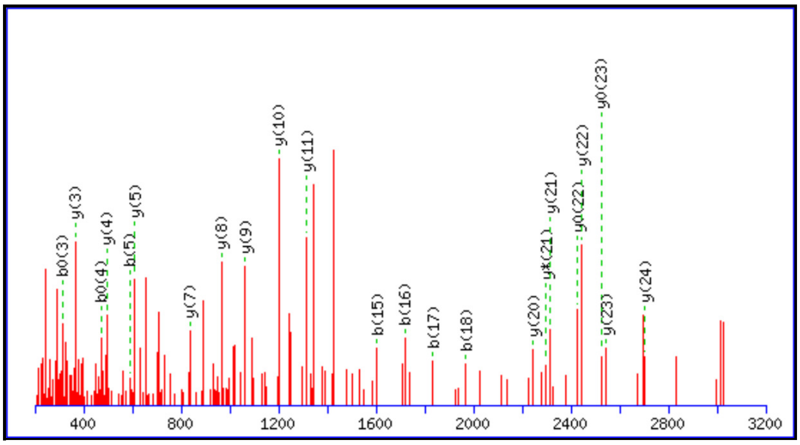

Monoisotopic mass of neutral peptide Mr(calc): 3032.4033
Fixed modifications: Carbamidomethyl (C) (apply to specified residues or termini only)
Variable modifications:
N17 : Deamidated (NQ)
Ions Score: 63 **Expect:** 0.0001
Matches : 23/286 fragment ions using 51 most intense peaks (help)

#	b	b^{++}	b*	b*$^{++}$	b^{0}	b^{0++}	Seq.	y	y^{++}	y*	y*$^{++}$	y^{0}	y^{0++}	#
1	130.0499	65.5286			112.0393	56.5233	E							27
2	201.0870	101.0471			183.0764	92.0418	A	2904.3680	1452.6876	2887.3414	1444.1744	2886.3574	1443.6824	26
3	330.1296	165.5684			312.1190	156.5631	E	2833.3309	1417.1691	2816.3043	1408.6558	2815.3203	1408.1638	25
4	490.1602	245.5838			472.1497	236.5785	C	***2704.2883***	1352.6478	2687.2617	1344.1345	2686.2777	1343.6425	24
5	**589.2286**	295.1180			571.2181	286.1127	V	***2544.2576***	1272.6325	2527.2311	1264.1192	2526.2471	1263.6272	23
6	718.2712	359.6393			700.2607	350.6340	E	***2445.1892***	1223.0982	2428.1627	1214.5850	2427.1787	1214.0930	22
7	789.3083	395.1578			771.2978	386.1525	A	***2316.1466***	1158.5770	2299.1201	1150.0637	2298.1361	1149.5717	21
8	904.3353	452.6713			886.3247	443.6660	D	***2245.1095***	1123.0584	2228.0830	1114.5451	2227.0990	1114.0531	20
9	991.3673	496.1873			973.3568	487.1820	S	2130.0826	1065.5449	2113.0560	1057.0317	2112.0720	1056.5396	19
10	1048.3888	524.6980			1030.3782	515.6927	G	2043.0505	1022.0289	2026.0240	1013.5156	2025.0400	1013.0236	18
11	1204.4899	602.7486	1187.4633	594.2353	1186.4793	593.7433	R	1986.0291	993.5182	1969.0025	985.0049	1968.0185	984.5129	17
12	1317.5740	659.2906	1300.5474	650.7773	1299.5634	650.2853	L	1829.9280	915.4676	1812.9014	906.9543	1811.9174	906.4623	16
13	1388.6111	694.8092	1371.5845	686.2959	1370.6005	685.8039	A	1716.8439	858.9256	1699.8174	850.4123	1698.8333	849.9203	15
14	1475.6431	738.3252	1458.6165	729.8119	1457.6325	729.3199	S	1645.8068	823.4070	1628.7802	814.8938	1627.7962	814.4018	14
15	**1604.6857**	802.8465	1587.6591	794.3332	1586.6751	793.8412	E	1558.7748	779.8910	1541.7482	771.3777	1540.7642	770.8857	13
16	**1717.7698**	859.3885	1700.7432	850.8752	1699.7592	850.3832	L	1429.7322	715.3697	1412.7056	706.8564	1411.7216	706.3644	12
17	**1832.7967**	916.9020	1815.7701	908.3887	1814.7861	907.8967	N	***1316.6481***	658.8277	1299.6216	650.3144	1298.6375	649.8224	11
18	**1969.8556**	985.4314	1952.8291	976.9182	1951.8450	976.4262	H	***1201.6212***	601.3142	1184.5946	592.8009	1183.6106	592.3089	10
19	2068.9240	1034.9656	2051.8975	1026.4524	2050.9135	1025.9604	V	***1064.5623***	532.7848	1047.5357	524.2715	1046.5517	523.7795	9
20	2196.9826	1098.9949	2179.9561	1090.4817	2178.9720	1089.9897	Q	***965.4938***	483.2506	948.4673	474.7373	947.4833	474.2453	8
21	2326.0252	1163.5162	2308.9986	1155.0030	2308.0146	1154.5110	E	***837.4353***	419.2213	820.4087	410.7080	819.4247	410.2160	7
22	2425.0936	1213.0504	2408.0671	1204.5372	2407.0830	1204.0452	V	708.3927	354.7000	691.3661	346.1867	690.3821	345.6947	6
23	2538.1777	1269.5925	2521.1511	1261.0792	2520.1671	1260.5872	L	***609.3243***	305.1658	592.2977	296.6525	591.3137	296.1605	5
24	2667.2203	1334.1138	2650.1937	1325.6005	2649.2097	1325.1085	E	***496.2402***	248.6237	479.2136	240.1105	478.2296	239.6185	4
25	2724.2417	1362.6245	2707.2152	1354.1112	2706.2312	1353.6192	G	***367.1976***	184.1024	350.1710	175.5892			3
26	2887.3051	1444.1562	2870.2785	1435.6429	2869.2945	1435.1509	Y	310.1761	155.5917	293.1496	147.0784			2
27							K	147.1128	74.0600	130.0863	65.5468			1

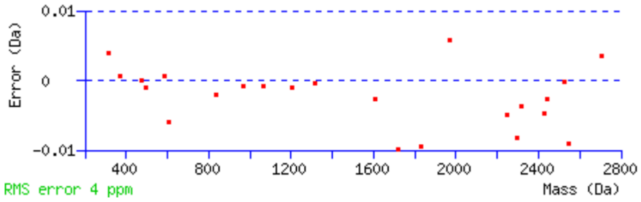

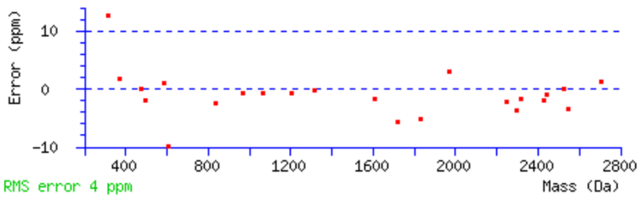

NCBI **BLAST** search of EAECVEADSGRLASELNHVQEVLEGYK
(Parameters: blastp, nr protein database, expect=20000, no filter, PAM30)
Other BLAST web gateways

All matches to this query

Score	Mr(calc):	Delta	Sequence
63.3	3032.4033	-0.0074	EAECVEADSGRLASELNHVQEVLEGYK
31.6	3032.4033	-0.0074	EAECVEADSGRLASELNHVQEVLEGYK
12.7	3031.4192	0.9767	EAECVEADSGRLASELNHVQEVLEGYK

Mascot: http://www.matrixscience.com/

Mascot Search Results

Peptide View

MS/MS Fragmentation of **EAECVEADSGRLASELNHVQEVLEGYK**
Found in **sp|O43790|KRT86_HUMAN**, Keratin, type II cuticular Hb6 OS=Homo sapiens GN=KRT86 PE=1 SV=1

Match to Query 40144: 3032.404976 from(759.108520,4+) intensity(2666729.5000) scans(14507) rtinseconds(2647) index(11397)
Title: 160219_Sunil_SDSI_A_Spectrum063524_scans__14507_RTINSECONDS=2647
Data file C:\\Sunil\\TKAP\\T\\T160219_Sunil_SDSI_A.mgf

Click mouse within plot area to zoom in by factor of two about that point
Or, Plot from 200 to 3000 Da Full range
Label all possible matches Label matches used for scoring

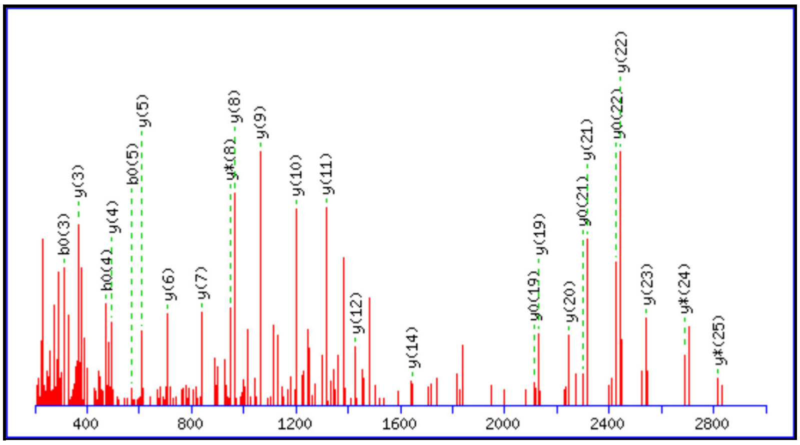

Monoisotopic mass of neutral peptide Mr(calc): 3032.4033
Fixed modifications: Carbamidomethyl (C) (apply to specified residues or termini only)
Variable modifications:
N17 : Deamidated (NQ)
Ions Score: 85 **Expect:** 7.6e-007
Matches : 25/286 fragment ions using 51 most intense peaks (help)

#	b	b^{++}	b*	b^{*++}	b^0	b^{0++}	Seq.	y	y^{++}	y*	y^{*++}	y^0	y^{0++}	#
1	130.0499	65.5286			112.0393	56.5233	E							27
2	201.0870	101.0471			183.0764	92.0418	A	2904.3680	1452.6876	2887.3414	1444.1744	2886.3574	1443.6824	26
3	330.1296	165.5684			312.1190	156.5631	E	2833.3309	1417.1691	2816.3043	1408.6558	2815.3203	1408.1638	25
4	490.1602	245.5838			472.1497	236.5785	C	2704.2883	1352.6478	2687.2617	1344.1345	2686.2777	1343.6425	24
5	589.2286	295.1180			571.2181	286.1127	V	***2544.2576***	1272.6325	2527.2311	1264.1192	2526.2471	1263.6272	23
6	718.2712	359.6393			700.2607	350.6340	E	***2445.1892***	1223.0982	2428.1627	1214.5850	2427.1787	1214.0930	22
7	789.3083	395.1578			771.2978	386.1525	A	***2316.1466***	1158.5770	2299.1201	1150.0637	2298.1361	1149.5717	21
8	904.3353	452.6713			886.3247	443.6660	D	***2245.1095***	1123.0584	2228.0830	1114.5451	2227.0990	1114.0531	20
9	991.3673	496.1873			973.3568	487.1820	S	***2130.0826***	1065.5449	2113.0560	1057.0317	2112.0720	1056.5396	19
10	1048.3888	524.6980			1030.3782	515.6927	G	2043.0505	1022.0289	2026.0240	1013.5156	2025.0400	1013.0236	18
11	1204.4899	602.7486	1187.4633	594.2353	1186.4793	593.7433	R	1986.0291	993.5182	1969.0025	985.0049	1968.0185	984.5129	17
12	1317.5740	659.2906	1300.5474	650.7773	1299.5634	650.2853	L	1829.9280	915.4676	1812.9014	906.9543	1811.9174	906.4623	16
13	1388.6111	694.8092	1371.5845	686.2959	1370.6005	685.8039	A	1716.8439	858.9256	1699.8174	850.4123	1698.8333	849.9203	15
14	1475.6431	738.3252	1458.6165	729.8119	1457.6325	729.3199	S	***1645.8068***	823.4070	1628.7802	814.8938	1627.7962	814.4018	14
15	1604.6857	802.8465	1587.6591	794.3332	1586.6751	793.8412	E	1558.7748	779.8910	1541.7482	771.3777	1540.7642	770.8857	13
16	1717.7698	859.3885	1700.7432	850.8752	1699.7592	850.3832	L	***1429.7322***	715.3697	1412.7056	706.8564	1411.7216	706.3644	12
17	1832.7967	916.9020	1815.7701	908.3887	1814.7861	907.8967	N	***1316.6481***	658.8277	1299.6216	650.3144	1298.6375	649.8224	11
18	1969.8556	985.4314	1952.8291	976.9182	1951.8450	976.4262	H	***1201.6212***	601.3142	1184.5946	592.8009	1183.6106	592.3089	10
19	2068.9240	1034.9656	2051.8975	1026.4524	2050.9135	1025.9604	V	***1064.5623***	532.7848	1047.5357	524.2715	1046.5517	523.7795	9
20	2196.9826	1098.9949	2179.9561	1090.4817	2178.9720	1089.9897	Q	***965.4938***	483.2506	948.4673	474.7373	947.4833	474.2453	8
21	2326.0252	1163.5162	2308.9986	1155.0030	2308.0146	1154.5110	E	***837.4353***	419.2213	820.4087	410.7080	819.4247	410.2160	7
22	2425.0936	1213.0504	2408.0671	1204.5372	2407.0830	1204.0452	V	***708.3927***	354.7000	691.3661	346.1867	690.3821	345.6947	6
23	2538.1777	1269.5925	2521.1511	1261.0792	2520.1671	1260.5872	L	***609.3243***	305.1658	592.2977	296.6525	591.3137	296.1605	5
24	2667.2203	1334.1138	2650.1937	1325.6005	2649.2097	1325.1085	E	***496.2402***	248.6237	479.2136	240.1105	478.2296	239.6185	4
25	2724.2417	1362.6245	2707.2152	1354.1112	2706.2312	1353.6192	G	***367.1976***	184.1024	350.1710	175.5892			3
26	2887.3051	1444.1562	2870.2785	1435.6429	2869.2945	1435.1509	Y	310.1761	155.5917	293.1496	147.0784			2
27							K	147.1128	74.0600	130.0863	65.5468			1

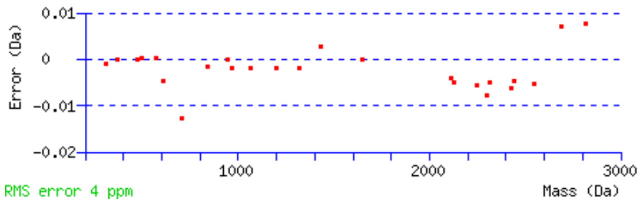

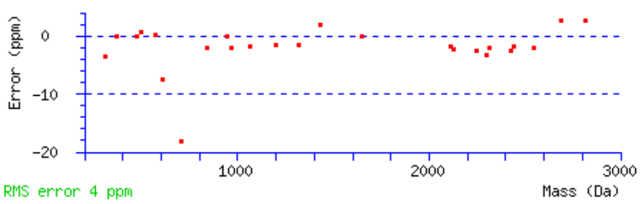

NCBI **BLAST** search of EAECVEADSGRLASELNHVQEVLEGYK
(Parameters: blastp, nr protein database, expect=20000, no filter, PAM30)
Other BLAST web gateways

All matches to this query

Score	Mr(calc):	Delta	Sequence
85.0	3032.4033	0.0017	EAECVEADSGRLASELNHVQEVLEGYK
52.2	3032.4033	0.0017	EAECVEADSGRLASELNHVQEVLEGYK
15.7	3031.4192	0.9857	EAECVEADSGRLASELNHVQEVLEGYK

Mascot: http://www.matrixscience.com/

Mascot Search Results

Peptide View

MS/MS Fragmentation of **LQFYQNRECCQSNLEPLFEGYIETLRR**
Found in **sp|O43790|KRT86_HUMAN**, Keratin, type II cuticular Hb6 OS=Homo sapiens GN=KRT86 PE=1 SV=1

Match to Query 42420: 3463.623976 from(866.913270,4+) intensity(10459158.0000) scans(15946) rtinseconds(2893) index(12600)
Title: 160219_Sunil_SDSI_A_Spectrum064736_scans__15946_RTINSECONDS=2893
Data file C:\\Sunil\\TKAP\\T\\T160219_Sunil_SDSI_A.mgf

Click mouse within plot area to zoom in by factor of two about that point
Or, Plot from 200 to 3400 Da Full range
Label all possible matches Label matches used for scoring

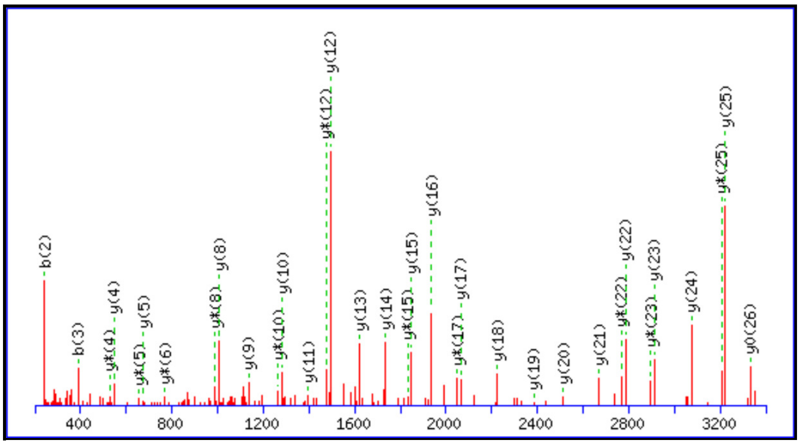

Monoisotopic mass of neutral peptide Mr(calc): 3463.6289
Fixed modifications: Carbamidomethyl (C) (apply to specified residues or termini only)
Variable modifications:
N6 : Deamidated (NQ)
Ions Score: 139 **Expect:** 2.6e-012
Matches : 34/290 fragment ions using 57 most intense peaks (help)

#	b	b++	b*	b*++	b0	b0++	Seq.	y	y++	y*	y*++	y0	y0++	#
1	114.0913	57.5493					L							27
2	**242.1499**	121.5786	225.1234	113.0653			Q	3351.5521	1676.2797	3334.5256	1667.7664	3333.5416	1667.2744	26
3	**389.2183**	195.1128	372.1918	186.5995			F	***3223.4935***	1612.2504	3206.4670	1603.7371	3205.4830	1603.2451	25
4	552.2817	276.6445	535.2551	268.1312			Y	***3076.4251***	1538.7162	3059.3986	1530.2029	3058.4146	1529.7109	24
5	680.3402	340.6738	663.3137	332.1605			Q	***2913.3618***	1457.1845	2896.3353	1448.6713	2895.3512	1448.1793	23
6	795.3672	398.1872	778.3406	389.6740			N	***2785.3032***	1393.1553	2768.2767	1384.6420	2767.2927	1384.1500	22
7	951.4683	476.2378	934.4417	467.7245			R	***2670.2763***	1335.6418	2653.2497	1327.1285	2652.2657	1326.6365	21
8	1080.5109	540.7591	1063.4843	532.2458	1062.5003	531.7538	E	***2514.1752***	1257.5912	2497.1486	1249.0779	2496.1646	1248.5859	20
9	1240.5415	620.7744	1223.5150	612.2611	1222.5310	611.7691	C	***2385.1326***	1193.0699	2368.1060	1184.5567	2367.1220	1184.0646	19
10	1400.5722	700.7897	1383.5456	692.2765	1382.5616	691.7844	C	***2225.1019***	1113.0546	2208.0754	1104.5413	2207.0914	1104.0493	18
11	1528.6308	764.8190	1511.6042	756.3057	1510.6202	755.8137	Q	***2065.0713***	1033.0393	2048.0447	1024.5260	2047.0607	1024.0340	17
12	1615.6628	808.3350	1598.6362	799.8218	1597.6522	799.3298	S	***1937.0127***	969.0100	1919.9862	960.4967	1919.0021	960.0047	16
13	1729.7057	865.3565	1712.6792	856.8432	1711.6952	856.3512	N	***1849.9807***	925.4940	1832.9541	916.9807	1831.9701	916.4887	15
14	1842.7898	921.8985	1825.7632	913.3853	1824.7792	912.8932	L	***1735.9377***	868.4725	1718.9112	859.9592	1717.9272	859.4672	14
15	1971.8324	986.4198	1954.8058	977.9066	1953.8218	977.4145	E	***1622.8537***	811.9305	1605.8271	803.4172	1604.8431	802.9252	13
16	2068.8851	1034.9462	2051.8586	1026.4329	2050.8746	1025.9409	P	***1493.8111***	747.4092	1476.7845	738.8959	1475.8005	738.4039	12
17	2181.9692	1091.4882	2164.9427	1082.9750	2163.9586	1082.4830	L	***1396.7583***	698.8828	1379.7318	690.3695	1378.7478	689.8775	11
18	2329.0376	1165.0224	2312.0111	1156.5092	2311.0271	1156.0172	F	***1283.6743***	642.3408	1266.6477	633.8275	1265.6637	633.3355	10
19	2458.0802	1229.5437	2441.0537	1221.0305	2440.0696	1220.5385	E	***1136.6058***	568.8066	1119.5793	560.2933	1118.5953	559.8013	9
20	2515.1017	1258.0545	2498.0751	1249.5412	2497.0911	1249.0492	G	***1007.5633***	504.2853	990.5367	495.7720	989.5527	495.2800	8
21	2678.1650	1339.5861	2661.1385	1331.0729	2660.1544	1330.5809	Y	950.5418	475.7745	933.5152	467.2613	932.5312	466.7693	7
22	2791.2491	1396.1282	2774.2225	1387.6149	2773.2385	1387.1229	I	787.4785	394.2429	770.4519	385.7296	769.4679	385.2376	6
23	2920.2917	1460.6495	2903.2651	1452.1362	2902.2811	1451.6442	E	***674.3944***	337.7008	657.3678	329.1876	656.3838	328.6956	5
24	3021.3393	1511.1733	3004.3128	1502.6600	3003.3288	1502.1680	T	***545.3518***	273.1795	528.3253	264.6663	527.3412	264.1743	4
25	3134.4234	1567.7153	3117.3969	1559.2021	3116.4128	1558.7101	L	444.3041	222.6557	427.2776	214.1424			3
26	3290.5245	1645.7659	3273.4980	1637.2526	3272.5139	1636.7606	R	331.2201	166.1137	314.1935	157.6004			2
27							R	175.1190	88.0631	158.0924	79.5498			1

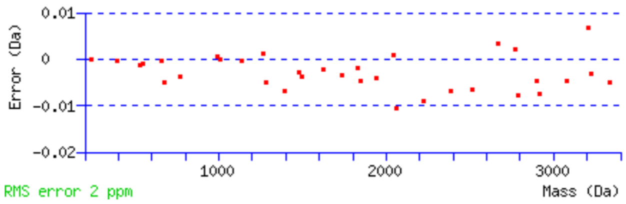

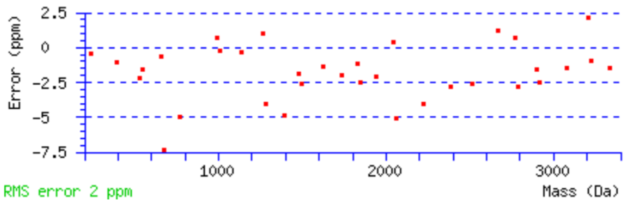

NCBI **BLAST** search of LQFYQNRECCQSNLEPLFEGYIETLRR
(Parameters: blastp, nr protein database, expect=20000, no filter, PAM30)
Other BLAST web gateways

All matches to this query

Score	Mr(calc):	Delta	Sequence
139.4	3463.6289	-0.0049	LQFYQNRECCQSNLEPLFEGYIETLRR
123.5	3463.6289	-0.0049	LQFYQNRECCQSNLEPLFEGYIETLRR
86.5	3463.6289	-0.0049	LQFYQNRECCQSNLEPLFEGYIETLRR
82.4	3462.6449	0.9791	LQFYQNRECCQSNLEPLFEGYIETLRR
77.8	3463.6289	-0.0049	LQFYQNRECCQSNLEPLFEGYIETLRR
73.7	3463.6289	-0.0049	LQFYQNRECCQSNLEPLFEGYIETLRR

Mascot: http://www.matrixscience.com/

Mascot Search Results

Peptide View

MS/MS Fragmentation of **LTAEVENAKCQNSKLEAAVAQSEQQGEAALSDAR**
Found in **sp|O43790|KRT86_HUMAN**, Keratin, type II cuticular Hb6 OS=Homo sapiens GN=KRT86 PE=1 SV=1

Match to Query 43041: 3616.727736 from(905.189210,4+) intensity(1315442.6250) scans(13965) rtinseconds(2557) index(41659)
Title: 160219_Sunil_SDSI_A_Spectrum096244_scans__13965_RTINSECONDS=2557
Data file C:\\Sunil\\TKAP\\T\\T160219_Sunil_SDSI_A.mgf

Click mouse within plot area to zoom in by factor of two about that point
Or, Plot from 200 to 2800 Da Full range
Label all possible matches ○ Label matches used for scoring ◉

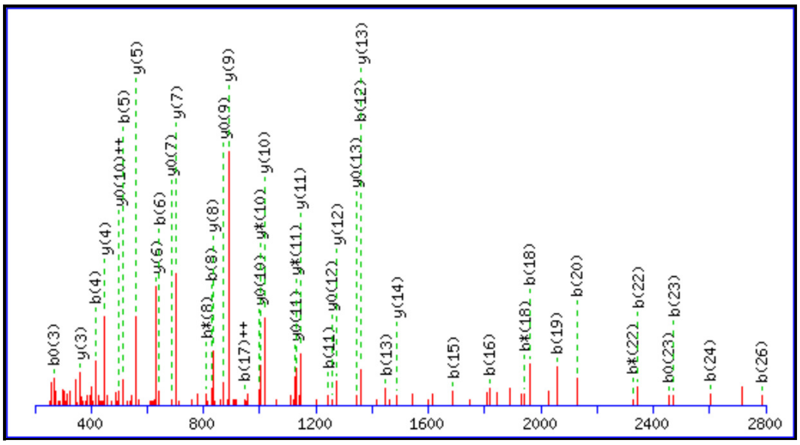

Monoisotopic mass of neutral peptide Mr(calc): 3616.7274
Fixed modifications: Carbamidomethyl (C) (apply to specified residues or termini only)
Variable modifications:
N12 : Deamidated (NQ)
Ions Score: 130 **Expect:** 2.1e-011
Matches : 43/378 fragment ions using 59 most intense peaks (help)

#	b	b^{++}	b*	b^{*++}	b^{0}	b^{0++}	Seq.	y	y^{++}	y*	y^{*++}	y^{0}	y^{0++}	#
1	114.0913	57.5493					L							34
2	215.1390	108.0731			197.1285	99.0679	T	3504.6507	1752.8290	3487.6242	1744.3157	3486.6401	1743.8237	33
3	286.1761	143.5917			268.1656	134.5864	A	3403.6030	1702.3052	3386.5765	1693.7919	3385.5925	1693.2999	32
4	**415.2187**	208.1130			397.2082	199.1077	E	3332.5659	1666.7866	3315.5394	1658.2733	3314.5554	1657.7813	31
5	**514.2871**	257.6472			496.2766	248.6419	V	3203.5233	1602.2653	3186.4968	1593.7520	3185.5128	1593.2600	30
6	**643.3297**	322.1685			625.3192	313.1632	E	3104.4549	1552.7311	3087.4284	1544.2178	3086.4443	1543.7258	29
7	757.3727	379.1900	740.3461	370.6767	739.3621	370.1847	N	2975.4123	1488.2098	2958.3858	1479.6965	2957.4018	1479.2045	28
8	**828.4098**	414.7085	811.3832	406.1953	810.3992	405.7032	A	2861.3694	1431.1883	2844.3428	1422.6751	2843.3588	1422.1831	27
9	956.5047	478.7560	939.4782	470.2427	938.4942	469.7507	K	2790.3323	1395.6698	2773.3057	1387.1565	2772.3217	1386.6645	26
10	1116.5354	558.7713	1099.5088	550.2581	1098.5248	549.7660	C	2662.2373	1331.6223	2645.2108	1323.1090	2644.2268	1322.6170	25
11	**1244.5940**	622.8006	1227.5674	614.2873	1226.5834	613.7953	Q	2502.2067	1251.6070	2485.1801	1243.0937	2484.1961	1242.6017	24
12	**1359.6209**	680.3141	1342.5944	671.8008	1341.6103	671.3088	N	2374.1481	1187.5777	2357.1215	1179.0644	2356.1375	1178.5724	23
13	**1446.6529**	723.8301	1429.6264	715.3168	1428.6424	714.8248	S	2259.1211	1130.0642	2242.0946	1121.5509	2241.1106	1121.0589	22
14	1574.7479	787.8776	1557.7213	779.3643	1556.7373	778.8723	K	2172.0891	1086.5482	2155.0626	1078.0349	2154.0786	1077.5429	21
15	**1687.8320**	844.4196	1670.8054	835.9063	1669.8214	835.4143	L	2043.9942	1022.5007	2026.9676	1013.9874	2025.9836	1013.4954	20
16	**1816.8746**	908.9409	1799.8480	900.4276	1798.8640	899.9356	E	1930.9101	965.9587	1913.8835	957.4454	1912.8995	956.9534	19
17	1887.9117	**944.4595**	1870.8851	935.9462	1869.9011	935.4542	A	1801.8675	901.4374	1784.8409	892.9241	1783.8569	892.4321	18
18	**1958.9488**	979.9780	1941.9222	971.4648	1940.9382	970.9727	A	1730.8304	865.9188	1713.8038	857.4056	1712.8198	856.9135	17
19	**2058.0172**	1029.5122	2040.9906	1020.9990	2040.0066	1020.5070	V	1659.7933	830.4003	1642.7667	821.8870	1641.7827	821.3950	16
20	**2129.0543**	1065.0308	2112.0278	1056.5175	2111.0437	1056.0255	A	1560.7249	780.8661	1543.6983	772.3528	1542.7143	771.8608	15
21	2257.1129	1129.0601	2240.0863	1120.5468	2239.1023	1120.0548	Q	**1489.6877**	745.3475	1472.6612	736.8342	1471.6772	736.3422	14
22	**2344.1449**	1172.5761	2327.1184	1164.0628	2326.1344	1163.5708	S	**1361.6292**	681.3182	1344.6026	672.8049	1343.6186	672.3129	13
23	**2473.1875**	1237.0974	2456.1610	1228.5841	2455.1769	1228.0921	E	**1274.5971**	637.8022	1257.5706	629.2889	1256.5866	628.7969	12
24	**2601.2461**	1301.1267	2584.2195	1292.6134	2583.2355	1292.1214	Q	**1145.5545**	573.2809	1128.5280	564.7676	1127.5440	564.2756	11
25	2729.3047	1365.1560	2712.2781	1356.6427	2711.2941	1356.1507	Q	**1017.4960**	509.2516	1000.4694	500.7383	999.4854	500.2463	10
26	**2786.3261**	1393.6667	2769.2996	1385.1534	2768.3156	1384.6614	G	**889.4374**	445.2223	872.4108	436.7091	871.4268	436.2170	9
27	2915.3687	1458.1880	2898.3422	1449.6747	2897.3582	1449.1827	E	**832.4159**	416.7116	815.3894	408.1983	814.4054	407.7063	8

**28**	2986.4058	1493.7066	2969.3793	1485.1933	2968.3953	1484.7013	**A**	***703.3733***	352.1903	686.3468	343.6770	685.3628	343.1850	**7**
**29**	3057.4430	1529.2251	3040.4164	1520.7118	3039.4324	1520.2198	**A**	***632.3362***	316.6717	615.3097	308.1585	614.3257	307.6665	**6**
**30**	3170.5270	1585.7671	3153.5005	1577.2539	3152.5165	1576.7619	**L**	***561.2991***	281.1532	544.2726	272.6399	543.2885	272.1479	**5**
**31**	3257.5590	1629.2832	3240.5325	1620.7699	3239.5485	1620.2779	**S**	***448.2150***	224.6112	431.1885	216.0979	430.2045	215.6059	**4**
**32**	3372.5860	1686.7966	3355.5594	1678.2834	3354.5754	1677.7913	**D**	***361.1830***	181.0951	344.1565	172.5819	343.1724	172.0899	**3**
**33**	3443.6231	1722.3152	3426.5966	1713.8019	3425.6125	1713.3099	**A**	246.1561	123.5817	229.1295	115.0684			**2**
**34**							**R**	175.1190	88.0631	158.0924	79.5498			**1**

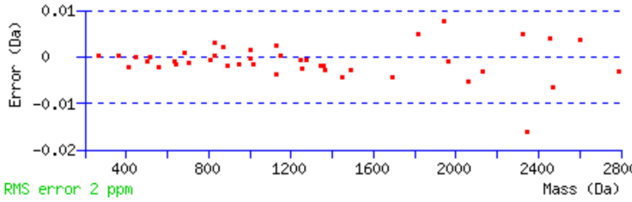

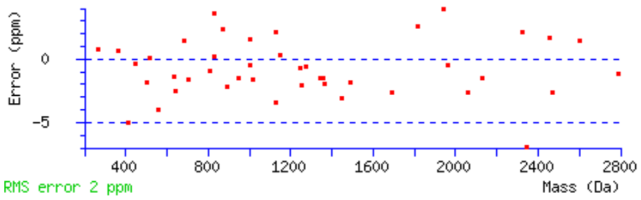

NCBI **BLAST** search of LTAEVENAKCQNSKLEAAVAQSEQQGEAALSDAR
(Parameters: blastp, nr protein database, expect=20000, no filter, PAM30)
Other BLAST web gateways

All matches to this query

Score	Mr(calc):	Delta	Sequence
130.5	3616.7274	0.0003	LTAEVENAKCQNSKLEAAVAQSEQQGEAALSDAR
120.1	3616.7274	0.0003	LTAEVENAKCQNSKLEAAVAQSEQQGEAALSDAR
105.9	3616.7274	0.0003	LTAEVENAKCQNSKLEAAVAQSEQQGEAALSDAR
60.0	3616.7274	0.0003	LTAEVENAKCQNSKLEAAVAQSEQQGEAALSDAR
52.0	3615.7434	0.9843	LTAEVENAKCQNSKLEAAVAQSEQQGEAALSDAR
29.3	3616.7274	0.0003	LTAEVENAKCQNSKLEAAVAQSEQQGEAALSDAR
19.6	3616.7274	0.0003	LTAEVENAKCQNSKLEAAVAQSEQQGEAALSDAR

Mascot: http://www.matrixscience.com/

Mascot Search Results

Peptide View

MS/MS Fragmentation of **LTAEVENAKCQNSKLEAAVAQSEQQGEAALSDAR**
Found in **sp|O43790|KRT86_HUMAN**, Keratin, type II cuticular Hb6 OS=Homo sapiens GN=KRT86 PE=1 SV=1

Match to Query 43046: 3616.735242 from(1206.585690,3+) intensity(1846121.2500) scans(14282) rtinseconds(2609) index(11196)
Title: 160219_Sunil_SDSI_A_Spectrum063322_scans__14282_RTINSECONDS=2609
Data file C:\\Sunil\\TKAP\\T\\T160219_Sunil_SDSI_A.mgf

Click mouse within plot area to zoom in by factor of two about that point
Or, Plot from 200 to 3800 Da Full range
Label all possible matches Label matches used for scoring

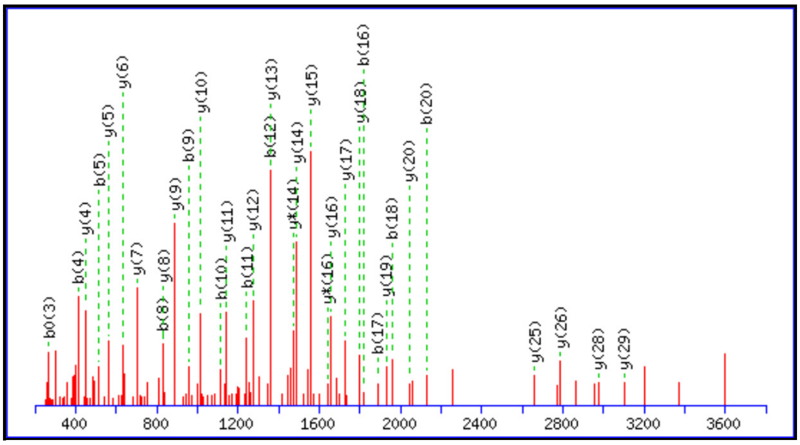

Monoisotopic mass of neutral peptide Mr(calc): 3616.7274
Fixed modifications: Carbamidomethyl (C) (apply to specified residues or termini only)
Variable modifications:
N12 : Deamidated (NQ)
Ions Score: 166 **Expect:** 6.5e-015
Matches : 35/378 fragment ions using 51 most intense peaks (help)

#	b	b++	b*	b*++	b0	b0++	Seq.	y	y++	y*	y*++	y0	y0++	#
1	114.0913	57.5493					L							34
2	215.1390	108.0731			197.1285	99.0679	T	3504.6507	1752.8290	3487.6242	1744.3157	3486.6401	1743.8237	33
3	286.1761	143.5917			268.1656	134.5864	A	3403.6030	1702.3052	3386.5765	1693.7919	3385.5925	1693.2999	32
4	**415.2187**	208.1130			397.2082	199.1077	E	3332.5659	1666.7866	3315.5394	1658.2733	3314.5554	1657.7813	31
5	**514.2871**	257.6472			496.2766	248.6419	V	3203.5233	1602.2653	3186.4968	1593.7520	3185.5128	1593.2600	30
6	643.3297	322.1685			625.3192	313.1632	E	***3104.4549***	1552.7311	3087.4284	1544.2178	3086.4443	1543.7258	29
7	757.3727	379.1900	740.3461	370.6767	739.3621	370.1847	N	***2975.4123***	1488.2098	2958.3858	1479.6965	2957.4018	1479.2045	28
8	**828.4098**	414.7085	811.3832	406.1953	810.3992	405.7032	A	2861.3694	1431.1883	2844.3428	1422.6751	2843.3588	1422.1831	27
9	**956.5047**	478.7560	939.4782	470.2427	938.4942	469.7507	K	***2790.3323***	1395.6698	2773.3057	1387.1565	2772.3217	1386.6645	26
10	**1116.5354**	558.7713	1099.5088	550.2581	1098.5248	549.7660	C	***2662.2373***	1331.6223	2645.2108	1323.1090	2644.2268	1322.6170	25
11	**1244.5940**	622.8006	1227.5674	614.2873	1226.5834	613.7953	Q	2502.2067	1251.6070	2485.1801	1243.0937	2484.1961	1242.6017	24
12	**1359.6209**	680.3141	1342.5944	671.8008	1341.6103	671.3088	N	2374.1481	1187.5777	2357.1215	1179.0644	2356.1375	1178.5724	23
13	1446.6529	723.8301	1429.6264	715.3168	1428.6424	714.8248	S	2259.1211	1130.0642	2242.0946	1121.5509	2241.1106	1121.0589	22
14	1574.7479	787.8776	1557.7213	779.3643	1556.7373	778.8723	K	2172.0891	1086.5482	2155.0626	1078.0349	2154.0786	1077.5429	21
15	1687.8320	844.4196	1670.8054	835.9063	1669.8214	835.4143	L	***2043.9942***	1022.5007	2026.9676	1013.9874	2025.9836	1013.4954	20
16	**1816.8746**	908.9409	1799.8480	900.4276	1798.8640	899.9356	E	***1930.9101***	965.9587	1913.8835	957.4454	1912.8995	956.9534	19
17	**1887.9117**	944.4595	1870.8851	935.9462	1869.9011	935.4542	A	***1801.8675***	901.4374	1784.8409	892.9241	1783.8569	892.4321	18
18	**1958.9488**	979.9780	1941.9222	971.4648	1940.9382	970.9727	A	***1730.8304***	865.9188	1713.8038	857.4056	1712.8198	856.9135	17
19	2058.0172	1029.5122	2040.9906	1020.9990	2040.0066	1020.5070	V	***1659.7933***	830.4003	1642.7667	821.8870	1641.7827	821.3950	16
20	**2129.0543**	1065.0308	2112.0278	1056.5175	2111.0437	1056.0255	A	***1560.7249***	780.8661	1543.6983	772.3528	1542.7143	771.8608	15
21	2257.1129	1129.0601	2240.0863	1120.5468	2239.1023	1120.0548	Q	***1489.6877***	745.3475	1472.6612	736.8342	1471.6772	736.3422	14
22	2344.1449	1172.5761	2327.1184	1164.0628	2326.1344	1163.5708	S	***1361.6292***	681.3182	1344.6026	672.8049	1343.6186	672.3129	13
23	2473.1875	1237.0974	2456.1610	1228.5841	2455.1769	1228.0921	E	***1274.5971***	637.8022	1257.5706	629.2889	1256.5866	628.7969	12
24	2601.2461	1301.1267	2584.2195	1292.6134	2583.2355	1292.1214	Q	***1145.5545***	573.2809	1128.5280	564.7676	1127.5440	564.2756	11
25	2729.3047	1365.1560	2712.2781	1356.6427	2711.2941	1356.1507	Q	***1017.4960***	509.2516	1000.4694	500.7383	999.4854	500.2463	10
26	2786.3261	1393.6667	2769.2996	1385.1534	2768.3156	1384.6614	G	***889.4374***	445.2223	872.4108	436.7091	871.4268	436.2170	9
27	2915.3687	1458.1880	2898.3422	1449.6747	2897.3582	1449.1827	E	***832.4159***	416.7116	815.3894	408.1983	814.4054	407.7063	8

**28**	2986.4058	1493.7066	2969.3793	1485.1933	2968.3953	1484.7013	**A**	***703.3733***	352.1903	686.3468	343.6770	685.3628	343.1850	**7**
**29**	3057.4430	1529.2251	3040.4164	1520.7118	3039.4324	1520.2198	**A**	***632.3362***	316.6717	615.3097	308.1585	614.3257	307.6665	**6**
**30**	3170.5270	1585.7671	3153.5005	1577.2539	3152.5165	1576.7619	**L**	***561.2991***	281.1532	544.2726	272.6399	543.2885	272.1479	**5**
**31**	3257.5590	1629.2832	3240.5325	1620.7699	3239.5485	1620.2779	**S**	***448.2150***	224.6112	431.1885	216.0979	430.2045	215.6059	**4**
**32**	3372.5860	1686.7966	3355.5594	1678.2834	3354.5754	1677.7913	**D**	361.1830	181.0951	344.1565	172.5819	343.1724	172.0899	**3**
**33**	3443.6231	1722.3152	3426.5966	1713.8019	3425.6125	1713.3099	**A**	246.1561	123.5817	229.1295	115.0684			**2**
**34**							**R**	175.1190	88.0631	158.0924	79.5498			**1**

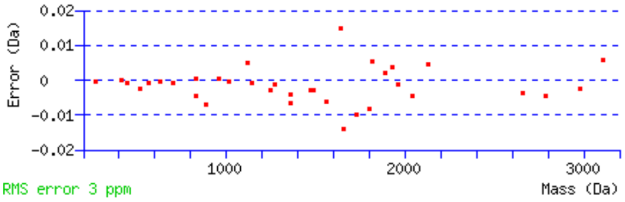

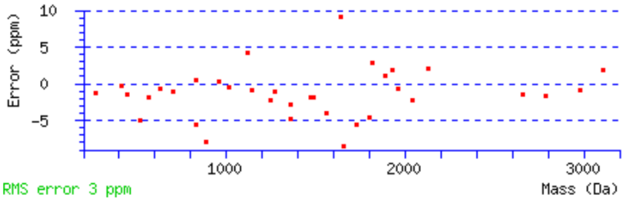

NCBI **BLAST** search of LTAEVENAKCQNSKLEAAVAQSEQQGEAALSDAR
(Parameters: blastp, nr protein database, expect=20000, no filter, PAM30)
Other BLAST web gateways

All matches to this query

Score	Mr(calc):	Delta	Sequence
165.6	3616.7274	0.0078	LTAEVENAKCQNSKLEAAVAQSEQQGEAALSDAR
162.5	3616.7274	0.0078	LTAEVENAKCQNSKLEAAVAQSEQQGEAALSDAR
126.2	3616.7274	0.0078	LTAEVENAKCQNSKLEAAVAQSEQQGEAALSDAR
98.8	3615.7434	0.9918	LTAEVENAKCQNSKLEAAVAQSEQQGEAALSDAR
77.3	3616.7274	0.0078	LTAEVENAKCQNSKLEAAVAQSEQQGEAALSDAR
49.4	3616.7274	0.0078	LTAEVENAKCQNSKLEAAVAQSEQQGEAALSDAR
42.3	3616.7274	0.0078	LTAEVENAKCQNSKLEAAVAQSEQQGEAALSDAR

Mascot: http://www.matrixscience.com/

Mascot Search Results

Peptide View

MS/MS Fragmentation of **TVNALEIELQAQHNLR**
Found in **sp|Q15323|K1H1_HUMAN**, Keratin, type I cuticular Ha1 OS=Homo sapiens GN=KRT31 PE=2 SV=3

Match to Query 25670: 1848.968488 from(925.491520,2+) intensity(5108248.5000) scans(13281) rtinseconds(2412) index(25810)
Title: 160219_Sunil_SDSI_A_Spectrum079116_scans__13281_RTINSECONDS=2412
Data file C:\\Sunil\\TKAP\\T\\T160219_Sunil_SDSI_A.mgf

Click mouse within plot area to zoom in by factor of two about that point
Or, Plot from 100 to 1900 Da Full range
Label all possible matches Label matches used for scoring

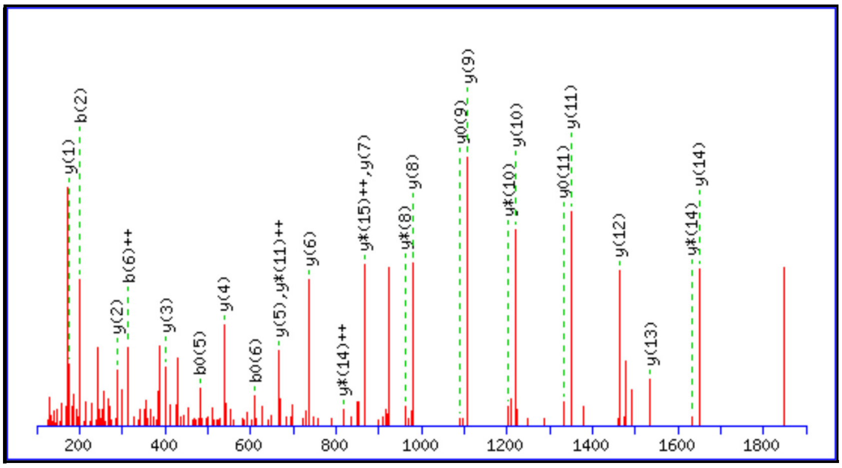

Monoisotopic mass of neutral peptide Mr(calc): 1848.9693
Fixed modifications: Carbamidomethyl (C) (apply to specified residues or termini only)
Variable modifications:
N3 : Deamidated (NQ)
Ions Score: 102 **Expect:** 1.4e-008
Matches : 26/160 fragment ions using 45 most intense peaks (help)

#	b	b++	b*	b*++	b0	b0++	Seq.	y	y++	y*	y*++	y0	y0++	#
1	102.0550	51.5311			84.0444	42.5258	T							16
2	**201.1234**	101.0653			183.1128	92.0600	V	1748.9290	874.9681	1731.9024	866.4549	1730.9184	865.9628	15
3	316.1503	158.5788	299.1238	150.0655	298.1397	149.5735	N	***1649.8606***	825.4339	1632.8340	816.9206	1631.8500	816.4286	14
4	387.1874	194.0974	370.1609	185.5841	369.1769	185.0921	A	***1534.8336***	767.9204	1517.8071	759.4072	1516.8231	758.9152	13
5	500.2715	250.6394	483.2449	242.1261	482.2609	241.6341	L	***1463.7965***	732.4019	1446.7700	723.8886	1445.7859	723.3966	12
6	629.3141	**315.1607**	612.2875	306.6474	611.3035	306.1554	E	***1350.7124***	675.8599	1333.6859	667.3466	1332.7019	666.8546	11
7	742.3981	371.7027	725.3716	363.1894	724.3876	362.6974	I	***1221.6698***	611.3386	1204.6433	602.8253	1203.6593	602.3333	10
8	871.4407	436.2240	854.4142	427.7107	853.4302	427.2187	E	***1108.5858***	554.7965	1091.5592	546.2833	1090.5752	545.7912	9
9	984.5248	492.7660	967.4983	484.2528	966.5142	483.7608	L	***979.5432***	490.2752	962.5166	481.7620			8
10	1112.5834	556.7953	1095.5568	548.2821	1094.5728	547.7900	Q	***866.4591***	433.7332	849.4326	425.2199			7
11	1183.6205	592.3139	1166.5939	583.8006	1165.6099	583.3086	A	***738.4005***	369.7039	721.3740	361.1906			6
12	1311.6791	656.3432	1294.6525	647.8299	1293.6685	647.3379	Q	***667.3634***	334.1854	650.3369	325.6721			5
13	1448.7380	724.8726	1431.7114	716.3594	1430.7274	715.8673	H	***539.3049***	270.1561	522.2783	261.6428			4
14	1562.7809	781.8941	1545.7544	773.3808	1544.7703	772.8888	N	***402.2459***	201.6266	385.2194	193.1133			3
15	1675.8650	838.4361	1658.8384	829.9229	1657.8544	829.4308	L	***288.2030***	144.6051	271.1765	136.0919			2
16							R	***175.1190***	88.0631	158.0924	79.5498			1

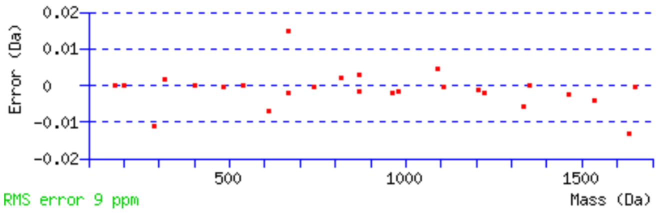

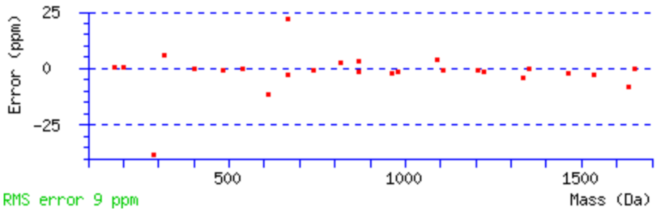

NCBI **BLAST** search of TVNALEIELQAQHNLR

(Parameters: blastp, nr protein database, expect=20000, no filter, PAM30)
Other BLAST web gateways

All matches to this query

Score	Mr(calc):	Delta	Sequence
102.1	1848.9693	-0.0008	TVNALEIELQAQHNLR
21.5	1848.9693	-0.0008	TVNALEIELQAQHNLR
10.4	1848.9693	-0.0008	TVNALEIELQAQHNLR
3.2	1846.9532	2.0152	IQSSGGPLQITMKMVPK
2.5	1848.9693	-0.0008	TVNALEIELQAQHNLR

Mascot: http://www.matrixscience.com/

Mascot Search Results

Peptide View

MS/MS Fragmentation of **TVNALEIELQAQHNLRDSLENTLTESEAR**
Found in **sp|Q15323|K1H1_HUMAN**, Keratin, type I cuticular Ha1 OS=Homo sapiens GN=KRT31 PE=2 SV=3

Match to Query 41478: 3294.622692 from(1099.214840,3+) intensity(2932553.2500) scans(15179) rtinseconds(2761) index(11976)
Title: 160219_Sunil_SDSI_A_Spectrum064105_scans__15179_RTINSECONDS=2761
Data file C:\\Sunil\\TKAP\\T\\T160219_Sunil_SDSI_A.mgf

Click mouse within plot area to zoom in by factor of two about that point
Or, Plot from 200 to 3400 Da Full range
Label all possible matches Label matches used for scoring

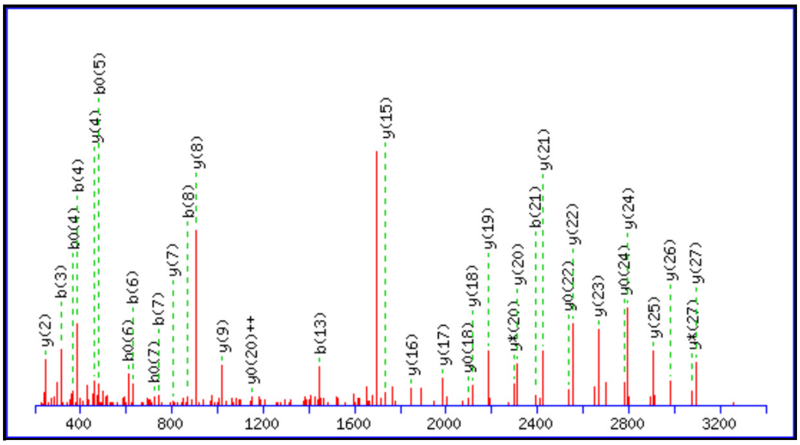

Monoisotopic mass of neutral peptide Mr(calc): 3294.6327
Fixed modifications: Carbamidomethyl (C) (apply to specified residues or termini only)
Variable modifications:
N21 : Deamidated (NQ)
Ions Score: 124 **Expect:** 9.6e-011
Matches : 35/328 fragment ions using 56 most intense peaks (help)

#	b	b++	b*	b*++	b0	b0++	Seq.	y	y++	y*	y*++	y0	y0++	#
1	102.0550	51.5311			84.0444	42.5258	T							29
2	201.1234	101.0653			183.1128	92.0600	V	3194.5924	1597.7998	3177.5658	1589.2866	3176.5818	1588.7946	28
3	**315.1663**	158.0868	298.1397	149.5735	297.1557	149.0815	N	***3095.5240***	1548.2656	3078.4974	1539.7524	3077.5134	1539.2603	27
4	**386.2034**	193.6053	369.1769	185.0921	368.1928	184.6001	A	***2981.4810***	1491.2442	2964.4545	1482.7309	2963.4705	1482.2389	26
5	499.2875	250.1474	482.2609	241.6341	481.2769	241.1421	L	***2910.4439***	1455.7256	2893.4174	1447.2123	2892.4334	1446.7203	25
6	**628.3301**	314.6687	611.3035	306.1554	610.3195	305.6634	E	***2797.3599***	1399.1836	2780.3333	1390.6703	2779.3493	1390.1783	24
7	**741.4141**	371.2107	724.3876	362.6974	723.4036	362.2054	I	***2668.3173***	1334.6623	2651.2907	1326.1490	2650.3067	1325.6570	23
8	**870.4567**	435.7320	853.4302	427.2187	852.4462	426.7267	E	***2555.2332***	1278.1202	2538.2067	1269.6070	2537.2226	1269.1150	22
9	983.5408	492.2740	966.5142	483.7608	965.5302	483.2687	L	***2426.1906***	1213.5989	2409.1641	1205.0857	2408.1801	1204.5937	21
10	1111.5994	556.3033	1094.5728	547.7900	1093.5888	547.2980	Q	***2313.1066***	1157.0569	2296.0800	1148.5436	2295.0960	1148.0516	20
11	1182.6365	591.8219	1165.6099	583.3086	1164.6259	582.8166	A	***2185.0480***	1093.0276	2168.0214	1084.5144	2167.0374	1084.0223	19
12	1310.6951	655.8512	1293.6685	647.3379	1292.6845	646.8459	Q	***2114.0109***	1057.5091	2096.9843	1048.9958	2096.0003	1048.5038	18
13	**1447.7540**	724.3806	1430.7274	715.8673	1429.7434	715.3753	H	***1985.9523***	993.4798	1968.9257	984.9665	1967.9417	984.4745	17
14	1561.7969	781.4021	1544.7703	772.8888	1543.7863	772.3968	N	***1848.8934***	924.9503	1831.8668	916.4371	1830.8828	915.9450	16
15	1674.8810	837.9441	1657.8544	829.4308	1656.8704	828.9388	L	***1734.8504***	867.9289	1717.8239	859.4156	1716.8399	858.9236	15
16	1830.9821	915.9947	1813.9555	907.4814	1812.9715	906.9894	R	1621.7664	811.3868	1604.7398	802.8736	1603.7558	802.3815	14
17	1946.0090	973.5081	1928.9825	964.9949	1927.9984	964.5029	D	1465.6653	733.3363	1448.6387	724.8230	1447.6547	724.3310	13
18	2033.0410	1017.0242	2016.0145	1008.5109	2015.0305	1008.0189	S	1350.6383	675.8228	1333.6118	667.3095	1332.6278	666.8175	12
19	2146.1251	1073.5662	2129.0986	1065.0529	2128.1145	1064.5609	L	1263.6063	632.3068	1246.5798	623.7935	1245.5957	623.3015	11
20	2275.1677	1138.0875	2258.1412	1129.5742	2257.1571	1129.0822	E	1150.5222	575.7648	1133.4957	567.2515	1132.5117	566.7595	10
21	**2390.1946**	1195.6010	2373.1681	1187.0877	2372.1841	1186.5957	N	***1021.4796***	511.2435	1004.4531	502.7302	1003.4691	502.2382	9
22	2491.2423	1246.1248	2474.2158	1237.6115	2473.2318	1237.1195	T	***906.4527***	453.7300	889.4262	445.2167	888.4421	444.7247	8
23	2604.3264	1302.6668	2587.2998	1294.1536	2586.3158	1293.6615	L	***805.4050***	403.2061	788.3785	394.6929	787.3945	394.2009	7
24	2705.3741	1353.1907	2688.3475	1344.6774	2687.3635	1344.1854	T	692.3210	346.6641	675.2944	338.1508	674.3104	337.6588	6
25	2834.4167	1417.7120	2817.3901	1409.1987	2816.4061	1408.7067	E	591.2733	296.1403	574.2467	287.6270	573.2627	287.1350	5
26	2921.4487	1461.2280	2904.4221	1452.7147	2903.4381	1452.2227	S	***462.2307***	231.6190	445.2041	223.1057	444.2201	222.6137	4
27	3050.4913	1525.7493	3033.4647	1517.2360	3032.4807	1516.7440	E	375.1987	188.1030	358.1721	179.5897	357.1881	179.0977	3

**28**	3121.5284	1561.2678	3104.5018	1552.7546	3103.5178	1552.2626	**A**	***246.1561***	123.5817	229.1295	115.0684			**2**
**29**							**R**	175.1190	88.0631	158.0924	79.5498			**1**

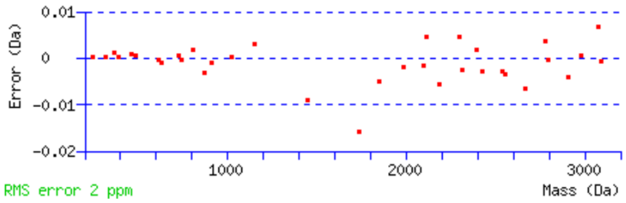

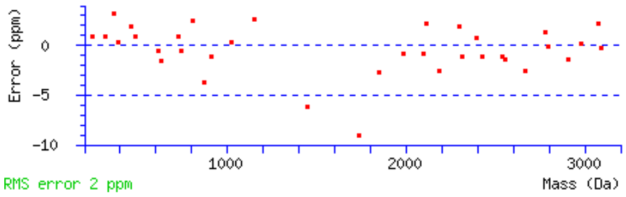

NCBI **BLAST** search of TVNALEIELQAQHNLRDSLENTLTESEAR
(Parameters: blastp, nr protein database, expect=20000, no filter, PAM30)
Other BLAST web gateways

All matches to this query

Score	Mr(calc):	Delta	Sequence
124.1	3294.6327	-0.0100	TVNALEIELQAQHNLRDSLENTLTESEAR
100.6	3294.6327	-0.0100	TVNALEIELQAQHNLRDSLENTLTESEAR
75.6	3294.6327	-0.0100	TVNALEIELQAQHNLRDSLENTLTESEAR
55.1	3294.6327	-0.0100	TVNALEIELQAQHNLRDSLENTLTESEAR
12.9	3293.6487	0.9740	TVNALEIELQAQHNLRDSLENTLTESEAR

Mascot: http://www.matrixscience.com/

Mascot Search Results

Peptide View

MS/MS Fragmentation of **TVNALEIELQAQHNLRDSLENTLTESEAR**
Found in **sp|Q15323|K1H1_HUMAN**, Keratin, type I cuticular Ha1 OS=Homo sapiens GN=KRT31 PE=2 SV=3

Match to Query 41484: 3294.637722 from(1099.219850,3+) intensity(2232826.7500) scans(15399) rtinseconds(2774) index(27456)
Title: 160219_Sunil_SDSI_A_Spectrum080771_scans__15399_RTINSECONDS=2774
Data file C:\\Sunil\\TKAP\\T\\T160219_Sunil_SDSI_A.mgf

Click mouse within plot area to zoom in by factor of two about that point
Or, Plot from 200 to 3400 Da Full range
Label all possible matches Label matches used for scoring

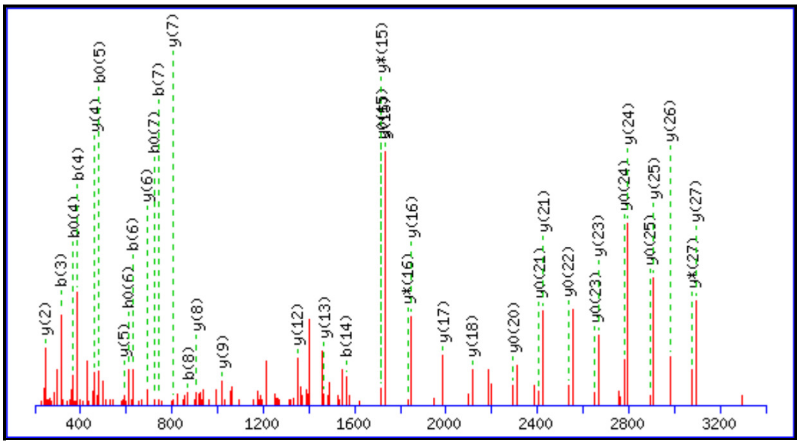

Monoisotopic mass of neutral peptide Mr(calc): 3294.6327
Fixed modifications: Carbamidomethyl (C) (apply to specified residues or termini only)
Variable modifications:
N14 : Deamidated (NQ)
Ions Score: 116 **Expect:** 6e-010
Matches : 39/328 fragment ions using 73 most intense peaks (help)

#	b	b++	b*	b*++	b0	b0++	Seq.	y	y++	y*	y*++	y0	y0++	#
1	102.0550	51.5311			84.0444	42.5258	T							29
2	201.1234	101.0653			183.1128	92.0600	V	3194.5924	1597.7998	3177.5658	1589.2866	3176.5818	1588.7946	28
3	**315.1663**	158.0868	298.1397	149.5735	297.1557	149.0815	N	***3095.5240***	1548.2656	3078.4974	1539.7524	3077.5134	1539.2603	27
4	**386.2034**	193.6053	369.1769	185.0921	368.1928	184.6001	A	***2981.4810***	1491.2442	2964.4545	1482.7309	2963.4705	1482.2389	26
5	499.2875	250.1474	482.2609	241.6341	481.2769	241.1421	L	***2910.4439***	1455.7256	2893.4174	1447.2123	2892.4334	1446.7203	25
6	**628.3301**	314.6687	611.3035	306.1554	610.3195	305.6634	E	***2797.3599***	1399.1836	2780.3333	1390.6703	2779.3493	1390.1783	24
7	**741.4141**	371.2107	724.3876	362.6974	723.4036	362.2054	I	***2668.3173***	1334.6623	2651.2907	1326.1490	2650.3067	1325.6570	23
8	**870.4567**	435.7320	853.4302	427.2187	852.4462	426.7267	E	2555.2332	1278.1202	2538.2067	1269.6070	2537.2226	1269.1150	22
9	983.5408	492.2740	966.5142	483.7608	965.5302	483.2687	L	***2426.1906***	1213.5989	2409.1641	1205.0857	2408.1801	1204.5937	21
10	1111.5994	556.3033	1094.5728	547.7900	1093.5888	547.2980	Q	2313.1066	1157.0569	2296.0800	1148.5436	2295.0960	1148.0516	20
11	1182.6365	591.8219	1165.6099	583.3086	1164.6259	582.8166	A	2185.0480	1093.0276	2168.0214	1084.5144	2167.0374	1084.0223	19
12	1310.6951	655.8512	1293.6685	647.3379	1292.6845	646.8459	Q	***2114.0109***	1057.5091	2096.9843	1048.9958	2096.0003	1048.5038	18
13	1447.7540	724.3806	1430.7274	715.8673	1429.7434	715.3753	H	***1985.9523***	993.4798	1968.9257	984.9665	1967.9417	984.4745	17
14	**1562.7809**	781.8941	1545.7544	773.3808	1544.7703	772.8888	N	***1848.8934***	924.9503	1831.8668	916.4371	1830.8828	915.9450	16
15	1675.8650	838.4361	1658.8384	829.9229	1657.8544	829.4308	L	***1733.8664***	867.4369	1716.8399	858.9236	1715.8559	858.4316	15
16	1831.9661	916.4867	1814.9395	907.9734	1813.9555	907.4814	R	1620.7824	810.8948	1603.7558	802.3815	1602.7718	801.8895	14
17	1946.9930	974.0002	1929.9665	965.4869	1928.9825	964.9949	D	***1464.6813***	732.8443	1447.6547	724.3310	1446.6707	723.8390	13
18	2034.0251	1017.5162	2016.9985	1009.0029	2016.0145	1008.5109	S	***1349.6543***	675.3308	1332.6278	666.8175	1331.6437	666.3255	12
19	2147.1091	1074.0582	2130.0826	1065.5449	2129.0986	1065.0529	L	1262.6223	631.8148	1245.5957	623.3015	1244.6117	622.8095	11
20	2276.1517	1138.5795	2259.1252	1130.0662	2258.1412	1129.5742	E	1149.5382	575.2727	1132.5117	566.7595	1131.5277	566.2675	10
21	2390.1946	1195.6010	2373.1681	1187.0877	2372.1841	1186.5957	N	***1020.4956***	510.7515	1003.4691	502.2382	1002.4851	501.7462	9
22	2491.2423	1246.1248	2474.2158	1237.6115	2473.2318	1237.1195	T	***906.4527***	453.7300	889.4262	445.2167	888.4421	444.7247	8
23	2604.3264	1302.6668	2587.2998	1294.1536	2586.3158	1293.6615	L	***805.4050***	403.2061	788.3785	394.6929	787.3945	394.2009	7
24	2705.3741	1353.1907	2688.3475	1344.6774	2687.3635	1344.1854	T	***692.3210***	346.6641	675.2944	338.1508	674.3104	337.6588	6
25	2834.4167	1417.7120	2817.3901	1409.1987	2816.4061	1408.7067	E	***591.2733***	296.1403	574.2467	287.6270	573.2627	287.1350	5
26	2921.4487	1461.2280	2904.4221	1452.7147	2903.4381	1452.2227	S	***462.2307***	231.6190	445.2041	223.1057	444.2201	222.6137	4
27	3050.4913	1525.7493	3033.4647	1517.2360	3032.4807	1516.7440	E	375.1987	188.1030	358.1721	179.5897	357.1881	179.0977	3

**28**	3121.5284	1561.2678	3104.5018	1552.7546	3103.5178	1552.2626	**A**	***246.1561***	123.5817	229.1295	115.0684			**2**
**29**							**R**	175.1190	88.0631	158.0924	79.5498			**1**

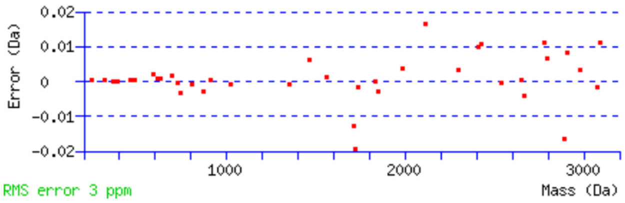

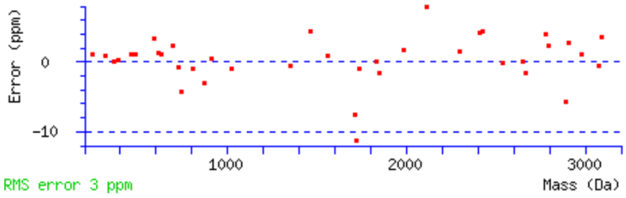

NCBI **BLAST** search of TVNALEIELQAQHNLRDSLENTLTESEAR
(Parameters: blastp, nr protein database, expect=20000, no filter, PAM30)
Other BLAST web gateways

All matches to this query

Score	Mr(calc):	Delta	Sequence
116.1	3294.6327	0.0050	TVNALEIELQAQHNLRDSLENTLTESEAR
95.8	3294.6327	0.0050	TVNALEIELQAQHNLRDSLENTLTESEAR
94.1	3294.6327	0.0050	TVNALEIELQAQHNLRDSLENTLTESEAR
87.0	3293.6487	0.9890	TVNALEIELQAQHNLRDSLENTLTESEAR
77.2	3294.6327	0.0050	TVNALEIELQAQHNLRDSLENTLTESEAR
67.9	3294.6327	0.0050	TVNALEIELQAQHNLRDSLENTLTESEAR
0.6	3294.6283	0.0095	VTNALHSILGEGNVPDPKHHYGVSGFHEQR

Mascot: http://www.matrixscience.com/

Mascot Search Results

Peptide View

MS/MS Fragmentation of **RTVNALEIELQAQHNLRDSLENTLTESEAR**
Found in **sp|Q15323|K1H1_HUMAN**, Keratin, type I cuticular Ha1 OS=Homo sapiens GN=KRT31 PE=2 SV=3

Match to Query 42331: 3450.738216 from(863.691830,4+) intensity(2476408.5000) scans(14112) rtinseconds(2554) index(26416)
Title: 160219_Sunil_SDSI_A_Spectrum079722_scans__14112_RTINSECONDS=2554
Data file C:\\Sunil\\TKAP\\T\\T160219_Sunil_SDSI_A.mgf

Click mouse within plot area to zoom in by factor of two about that point
Or, Plot from 200 to 3600 Da Full range
Label all possible matches Label matches used for scoring

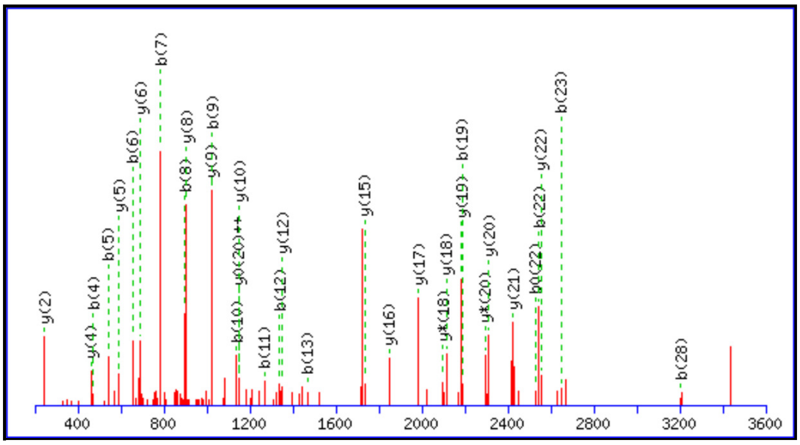

Monoisotopic mass of neutral peptide Mr(calc): 3450.7338
Fixed modifications: Carbamidomethyl (C) (apply to specified residues or termini only)
Variable modifications:
N22 : Deamidated (NQ)
Ions Score: 141 **Expect:** 1.8e-012
Matches : 34/342 fragment ions using 48 most intense peaks (help)

#	b	b++	b*	b*++	b0	b0++	Seq.	y	y++	y*	y*++	y0	y0++	#
1	157.1084	79.0578	140.0818	70.5446			R							30
2	258.1561	129.5817	241.1295	121.0684	240.1455	120.5764	T	3295.6401	1648.3237	3278.6135	1639.8104	3277.6295	1639.3184	29
3	357.2245	179.1159	340.1979	170.6026	339.2139	170.1106	V	3194.5924	1597.7998	3177.5658	1589.2866	3176.5818	1588.7946	28
4	471.2674	236.1373	454.2409	227.6241	453.2568	227.1321	N	3095.5240	1548.2656	3078.4974	1539.7524	3077.5134	1539.2603	27
5	542.3045	271.6559	525.2780	263.1426	524.2940	262.6506	A	2981.4810	1491.2442	2964.4545	1482.7309	2963.4705	1482.2389	26
6	655.3886	328.1979	638.3620	319.6847	637.3780	319.1926	L	2910.4439	1455.7256	2893.4174	1447.2123	2892.4334	1446.7203	25
7	784.4312	392.7192	767.4046	384.2060	766.4206	383.7139	E	2797.3599	1399.1836	2780.3333	1390.6703	2779.3493	1390.1783	24
8	897.5152	449.2613	880.4887	440.7480	879.5047	440.2560	I	2668.3173	1334.6623	2651.2907	1326.1490	2650.3067	1325.6570	23
9	1026.5578	513.7826	1009.5313	505.2693	1008.5473	504.7773	E	2555.2332	1278.1202	2538.2067	1269.6070	2537.2226	1269.1150	22
10	1139.6419	570.3246	1122.6154	561.8113	1121.6313	561.3193	L	2426.1906	1213.5989	2409.1641	1205.0857	2408.1801	1204.5937	21
11	1267.7005	634.3539	1250.6739	625.8406	1249.6899	625.3486	Q	2313.1066	1157.0569	2296.0800	1148.5436	2295.0960	1148.0516	20
12	1338.7376	669.8724	1321.7110	661.3592	1320.7270	660.8672	A	2185.0480	1093.0276	2168.0214	1084.5144	2167.0374	1084.0223	19
13	1466.7962	733.9017	1449.7696	725.3884	1448.7856	724.8964	Q	2114.0109	1057.5091	2096.9843	1048.9958	2096.0003	1048.5038	18
14	1603.8551	802.4312	1586.8285	793.9179	1585.8445	793.4259	H	1985.9523	993.4798	1968.9257	984.9665	1967.9417	984.4745	17
15	1717.8980	859.4526	1700.8715	850.9394	1699.8874	850.4474	N	1848.8934	924.9503	1831.8668	916.4371	1830.8828	915.9450	16
16	1830.9821	915.9947	1813.9555	907.4814	1812.9715	906.9894	L	1734.8504	867.9289	1717.8239	859.4156	1716.8399	858.9236	15
17	1987.0832	994.0452	1970.0566	985.5320	1969.0726	985.0399	R	1621.7664	811.3868	1604.7398	802.8736	1603.7558	802.3815	14
18	2102.1101	1051.5587	2085.0836	1043.0454	2084.0996	1042.5534	D	1465.6653	733.3363	1448.6387	724.8230	1447.6547	724.3310	13
19	2189.1422	1095.0747	2172.1156	1086.5614	2171.1316	1086.0694	S	1350.6383	675.8228	1333.6118	667.3095	1332.6278	666.8175	12
20	2302.2262	1151.6167	2285.1997	1143.1035	2284.2157	1142.6115	L	1263.6063	632.3068	1246.5798	623.7935	1245.5957	623.3015	11
21	2431.2688	1216.1380	2414.2423	1207.6248	2413.2582	1207.1328	E	1150.5222	575.7648	1133.4957	567.2515	1132.5117	566.7595	10
22	2546.2958	1273.6515	2529.2692	1265.1382	2528.2852	1264.6462	N	1021.4796	511.2435	1004.4531	502.7302	1003.4691	502.2382	9
23	2647.3434	1324.1754	2630.3169	1315.6621	2629.3329	1315.1701	T	906.4527	453.7300	889.4262	445.2167	888.4421	444.7247	8
24	2760.4275	1380.7174	2743.4009	1372.2041	2742.4169	1371.7121	L	805.4050	403.2061	788.3785	394.6929	787.3945	394.2009	7
25	2861.4752	1431.2412	2844.4486	1422.7280	2843.4646	1422.2359	T	692.3210	346.6641	675.2944	338.1508	674.3104	337.6588	6
26	2990.5178	1495.7625	2973.4912	1487.2492	2972.5072	1486.7572	E	591.2733	296.1403	574.2467	287.6270	573.2627	287.1350	5
27	3077.5498	1539.2785	3060.5232	1530.7653	3059.5392	1530.2733	S	462.2307	231.6190	445.2041	223.1057	444.2201	222.6137	4

**28**	*3206.5924*	1603.7998	3189.5658	1595.2866	3188.5818	1594.7946	**E**	375.1987	188.1030	358.1721	179.5897	357.1881	179.0977	**3**
**29**	3277.6295	1639.3184	3260.6030	1630.8051	3259.6189	1630.3131	**A**	*246.1561*	123.5817	229.1295	115.0684			**2**
**30**							**R**	175.1190	88.0631	158.0924	79.5498			**1**

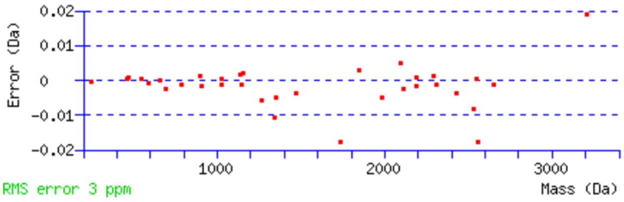

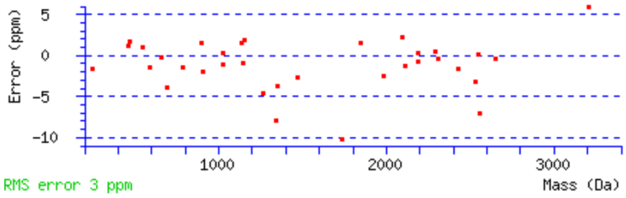

NCBI **BLAST** search of RTVNALEIELQAQHNLRDSLENTLTESEAR
(Parameters: blastp, nr protein database, expect=20000, no filter, PAM30)
Other BLAST web gateways

All matches to this query

Score	Mr(calc):	Delta	Sequence
140.6	3450.7338	0.0044	RTVNALEIELQAQHNLRDSLENTLTESEAR
90.2	3450.7338	0.0044	RTVNALEIELQAQHNLRDSLENTLTESEAR
68.3	3450.7338	0.0044	RTVNALEIELQAQHNLRDSLENTLTESEAR
51.7	3449.7498	0.9884	RTVNALEIELQAQHNLRDSLENTLTESEAR
43.6	3450.7338	0.0044	RTVNALEIELQAQHNLRDSLENTLTESEAR
2.3	3450.7338	0.0044	RTVNALEIELQAQHNLRDSLENTLTESEAR

Mascot: http://www.matrixscience.com/

Mascot Search Results

Peptide View

MS/MS Fragmentation of **TKEEINELNR**
Found in **sp|P78385|KRT83_HUMAN**, Keratin, type II cuticular Hb3 OS=Homo sapiens GN=KRT83 PE=1 SV=2

Match to Query 9756: 1245.621522 from(416.214450,3+) intensity(12807713.0000) scans(4024) rtinseconds(845) index(2585)
Title: 160219_Sunil_SDSI_A_Spectrum054693_scans__4024_RTINSECONDS=845
Data file C:\\Sunil\\TKAP\\T\\T160219_Sunil_SDSI_A.mgf

Click mouse within plot area to zoom in by factor of two about that point
Or, Plot from 0 to 1300 Da Full range
Label all possible matches Label matches used for scoring

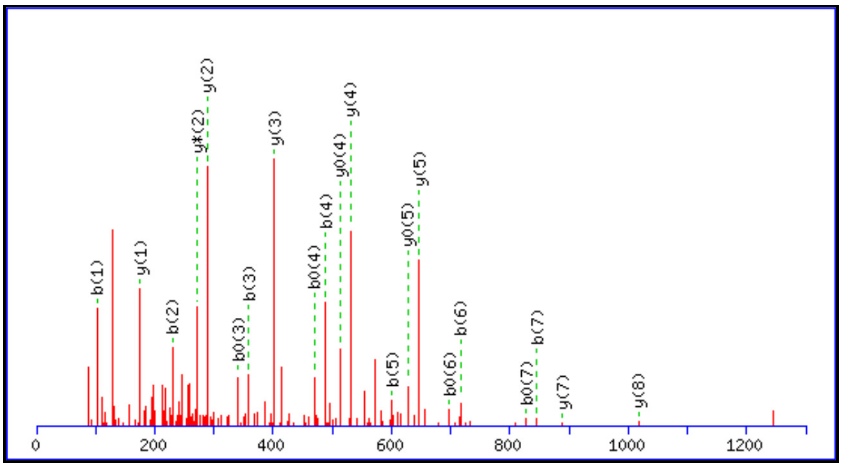

Monoisotopic mass of neutral peptide Mr(calc): 1245.6200
Fixed modifications: Carbamidomethyl (C) (apply to specified residues or termini only)
Variable modifications:
N6 : Deamidated (NQ)
Ions Score: 69 **Expect:** 3.2e-005
Matches : 21/100 fragment ions using 26 most intense peaks (help)

#	b	b++	b*	b*++	b0	b0++	Seq.	y	y++	y*	y*++	y0	y0++	#
1	*102.0550*	51.5311			84.0444	42.5258	T							10
2	*230.1499*	115.5786	213.1234	107.0653	212.1394	106.5733	K	1145.5797	573.2935	1128.5531	564.7802	1127.5691	564.2882	9
3	*359.1925*	180.0999	342.1660	171.5866	341.1819	171.0946	E	*1017.4847*	509.2460	1000.4582	500.7327	999.4742	500.2407	8
4	*488.2351*	244.6212	471.2086	236.1079	470.2245	235.6159	E	*888.4421*	444.7247	871.4156	436.2114	870.4316	435.7194	7
5	*601.3192*	301.1632	584.2926	292.6499	583.3086	292.1579	I	759.3995	380.2034	742.3730	371.6901	741.3890	371.1981	6
6	*716.3461*	358.6767	699.3196	350.1634	698.3355	349.6714	N	*646.3155*	323.6614	629.2889	315.1481	628.3049	314.6561	5
7	*845.3887*	423.1980	828.3622	414.6847	827.3781	414.1927	E	*531.2885*	266.1479	514.2620	257.6346	513.2780	257.1426	4
8	958.4728	479.7400	941.4462	471.2267	940.4622	470.7347	L	*402.2459*	201.6266	385.2194	193.1133			3
9	1072.5157	536.7615	1055.4891	528.2482	1054.5051	527.7562	N	*289.1619*	145.0846	272.1353	136.5713			2
10							R	*175.1190*	88.0631	158.0924	79.5498			1

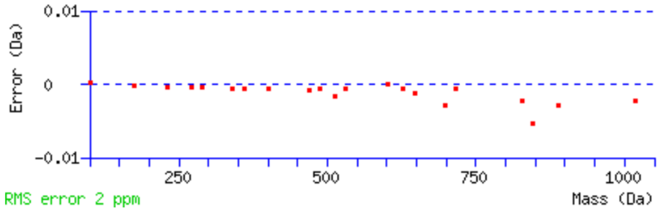

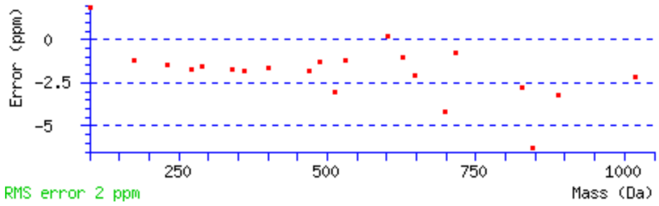

NCBI **BLAST** search of TKEEINELNR
(Parameters: blastp, nr protein database, expect=20000, no filter, PAM30)
Other BLAST web gateways

All matches to this query

Score	Mr(calc):	Delta	Sequence
68.6	1245.6200	0.0015	TKEEINELNR

23.5	1245.6200	0.0015	TKEEINELNR
10.7	1245.6313	-0.0097	KSLEEEQRAR
10.6	1243.6156	2.0059	TNQELQEINR
9.7	1243.6269	1.9947	AELREDERAR
8.7	1245.6136	0.0080	QMVPSSASGQVR
5.6	1245.6288	-0.0073	FCITVSHLNR
5.6	1245.6109	0.0107	RNGSRNCLNR
5.3	1245.6201	0.0015	ETDKQILQNR
4.6	1245.6102	0.0114	LWQSAQQARR

Mascot: http://www.matrixscience.com/

Mascot Search Results

Peptide View

MS/MS Fragmentation of **LTAEVENAKCQNSK**
Found in **sp|P78385|KRT83_HUMAN**, Keratin, type II cuticular Hb3 OS=Homo sapiens GN=KRT83 PE=1 SV=2

Match to Query 20350: 1591.751352 from(531.591060,3+) intensity(809942.8750) scans(2226) rtinseconds(505) index(16250)
Title: 160219_Sunil_SDSI_A_Spectrum069550_scans__2226_RTINSECONDS=505
Data file C:\\Sunil\\TKAP\\T\\T160219_Sunil_SDSI_A.mgf

Click mouse within plot area to zoom in by factor of two about that point
Or, Plot from 100 to 1500 Da Full range
Label all possible matches ○ Label matches used for scoring ◉

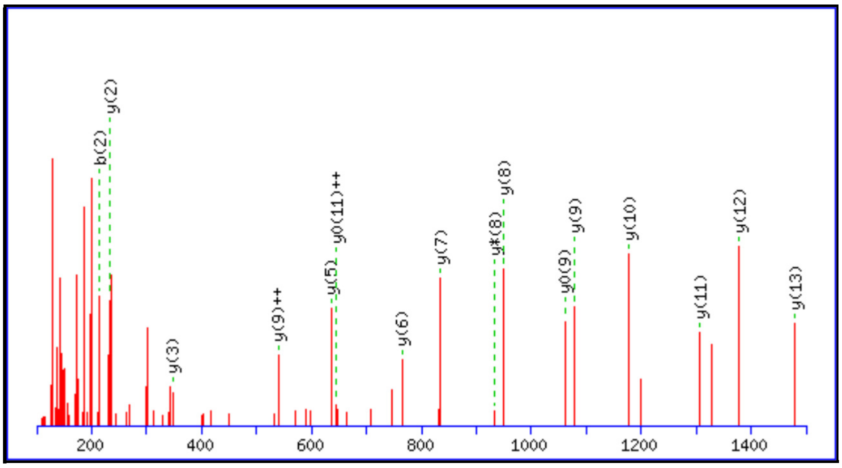

Monoisotopic mass of neutral peptide Mr(calc): 1591.7511
Fixed modifications: Carbamidomethyl (C) (apply to specified residues or termini only)
Variable modifications:
N7 : Deamidated (NQ)
Ions Score: 76 **Expect:** 4.8e-006
Matches : 16/140 fragment ions using 31 most intense peaks (help)

#	b	b++	b*	b*++	b0	b0++	Seq.	y	y++	y*	y*++	y0	y0++	#
1	114.0913	57.5493					L							14
2	**215.1390**	108.0731			197.1285	99.0679	T	***1479.6744***	740.3408	1462.6479	731.8276	1461.6638	731.3356	13
3	286.1761	143.5917			268.1656	134.5864	A	***1378.6267***	689.8170	1361.6002	681.3037	1360.6162	680.8117	12
4	415.2187	208.1130			397.2082	199.1077	E	***1307.5896***	654.2984	1290.5631	645.7852	1289.5790	645.2932	11
5	514.2871	257.6472			496.2766	248.6419	V	***1178.5470***	589.7771	1161.5205	581.2639	1160.5364	580.7719	10
6	643.3297	322.1685			625.3192	313.1632	E	***1079.4786***	540.2429	1062.4520	531.7297	1061.4680	531.2377	9
7	758.3567	379.6820	741.3301	371.1687	740.3461	370.6767	N	***950.4360***	475.7216	933.4095	467.2084	932.4254	466.7164	8
8	829.3938	415.2005	812.3672	406.6873	811.3832	406.1953	A	***835.4091***	418.2082	818.3825	409.6949	817.3985	409.2029	7
9	957.4888	479.2480	940.4622	470.7347	939.4782	470.2427	K	***764.3719***	382.6896	747.3454	374.1763	746.3614	373.6843	6
10	1117.5194	559.2633	1100.4929	550.7501	1099.5088	550.2581	C	***636.2770***	318.6421	619.2504	310.1289	618.2664	309.6368	5
11	1245.5780	623.2926	1228.5514	614.7794	1227.5674	614.2873	Q	476.2463	238.6268	459.2198	230.1135	458.2358	229.6215	4
12	1359.6209	680.3141	1342.5944	671.8008	1341.6103	671.3088	N	***348.1878***	174.5975	331.1612	166.0842	330.1772	165.5922	3
13	1446.6529	723.8301	1429.6264	715.3168	1428.6424	714.8248	S	***234.1448***	117.5761	217.1183	109.0628	216.1343	108.5708	2
14							K	147.1128	74.0600	130.0863	65.5468			1

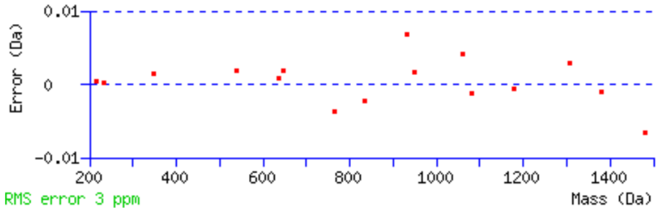

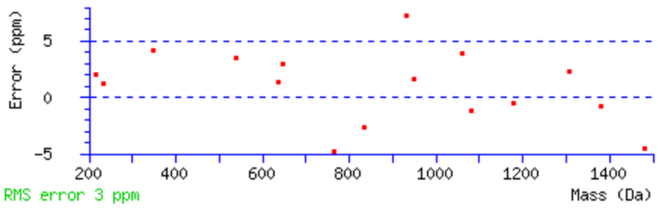

NCBI **BLAST** search of LTAEVENAKCQNSK
(Parameters: blastp, nr protein database, expect=20000, no filter, PAM30)
Other BLAST web gateways

All matches to this query

Score	Mr(calc):	Delta	Sequence
76.0	1591.7511	0.0002	LTAEVENAKCQNSK
38.2	1591.7511	0.0002	LTAEVENAKCQNSK
30.8	1591.7511	0.0002	LTAEVENAKCQNSK

Mascot: http://www.matrixscience.com/

Mascot Search Results

Peptide View

MS/MS Fragmentation of **EYQEVMNSKLGLDIEIATYR**
Found in **sp|P78385|KRT83_HUMAN**, Keratin, type II cuticular Hb3 OS=Homo sapiens GN=KRT83 PE=1 SV=2

Match to Query 35276: 2372.158422 from(791.726750,3+) intensity(4437477.5000) scans(15387) rtinseconds(2797) index(12134)
Title: 160219_Sunil_SDSI_A_Spectrum064265_scans__15387_RTINSECONDS=2797
Data file C:\\Sunil\\TKAP\\T\\T160219_Sunil_SDSI_A.mgf

Click mouse within plot area to zoom in by factor of two about that point
Or, Plot from 0 to 2400 Da Full range
Label all possible matches Label matches used for scoring

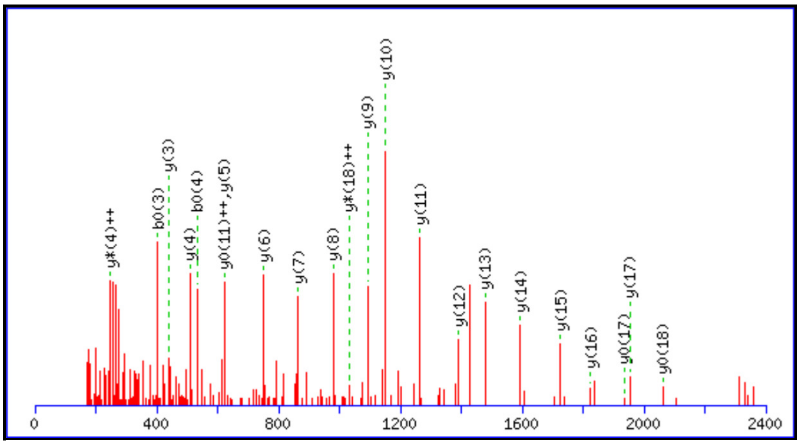

Monoisotopic mass of neutral peptide Mr(calc): 2372.1569
Fixed modifications: Carbamidomethyl (C) (apply to specified residues or termini only)
Variable modifications:
N7 : Deamidated (NQ)
Ions Score: 97 **Expect:** 5.6e-008
Matches : 22/220 fragment ions using 40 most intense peaks (help)

#	b	b^{++}	b*	b^{*++}	b^{0}	b^{0++}	Seq.	y	y^{++}	y*	y^{*++}	y^{0}	y^{0++}	#
1	130.0499	65.5286			112.0393	56.5233	E							20
2	293.1132	147.0602			275.1026	138.0550	Y	2244.1217	1122.5645	2227.0951	1114.0512	2226.1111	1113.5592	19
3	421.1718	211.0895	404.1452	202.5763	403.1612	202.0842	Q	2081.0583	1041.0328	2064.0318	1032.5195	2063.0478	1032.0275	18
4	550.2144	275.6108	533.1878	267.0975	532.2038	266.6055	E	***1952.9998***	977.0035	1935.9732	968.4902	1934.9892	967.9982	17
5	649.2828	325.1450	632.2562	316.6318	631.2722	316.1397	V	***1823.9572***	912.4822	1806.9306	903.9689	1805.9466	903.4769	16
6	780.3233	390.6653	763.2967	382.1520	762.3127	381.6600	M	***1724.8887***	862.9480	1707.8622	854.4347	1706.8782	853.9427	15
7	895.3502	448.1787	878.3237	439.6655	877.3396	439.1735	N	***1593.8483***	797.4278	1576.8217	788.9145	1575.8377	788.4225	14
8	982.3822	491.6948	965.3557	483.1815	964.3717	482.6895	S	***1478.8213***	739.9143	1461.7948	731.4010	1460.8108	730.9090	13
9	1110.4772	555.7422	1093.4507	547.2290	1092.4666	546.7370	K	***1391.7893***	696.3983	1374.7627	687.8850	1373.7787	687.3930	12
10	1223.5613	612.2843	1206.5347	603.7710	1205.5507	603.2790	L	***1263.6943***	632.3508	1246.6678	623.8375	1245.6838	623.3455	11
11	1280.5827	640.7950	1263.5562	632.2817	1262.5722	631.7897	G	***1150.6103***	575.8088	1133.5837	567.2955	1132.5997	566.8035	10
12	1393.6668	697.3370	1376.6402	688.8238	1375.6562	688.3318	L	***1093.5888***	547.2980	1076.5623	538.7848	1075.5782	538.2928	9
13	1508.6937	754.8505	1491.6672	746.3372	1490.6832	745.8452	D	***980.5047***	490.7560	963.4782	482.2427	962.4942	481.7507	8
14	1621.7778	811.3925	1604.7513	802.8793	1603.7672	802.3873	I	***865.4778***	433.2425	848.4512	424.7293	847.4672	424.2373	7
15	1750.8204	875.9138	1733.7938	867.4006	1732.8098	866.9086	E	***752.3937***	376.7005	735.3672	368.1872	734.3832	367.6952	6
16	1863.9045	932.4559	1846.8779	923.9426	1845.8939	923.4506	I	***623.3511***	312.1792	606.3246	303.6659	605.3406	303.1739	5
17	1934.9416	967.9744	1917.9150	959.4611	1916.9310	958.9691	A	***510.2671***	255.6372	493.2405	247.1239	492.2565	246.6319	4
18	2035.9893	1018.4983	2018.9627	1009.9850	2017.9787	1009.4930	T	***439.2300***	220.1186	422.2034	211.6053	421.2194	211.1133	3
19	2199.0526	1100.0299	2182.0260	1091.5167	2181.0420	1091.0246	Y	338.1823	169.5948	321.1557	161.0815			2
20							R	175.1190	88.0631	158.0924	79.5498			1

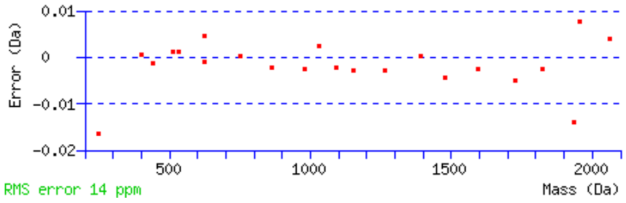

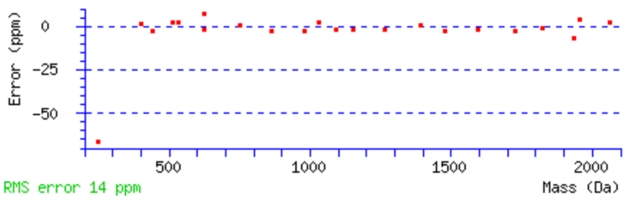

NCBI **BLAST** search of EYQEVMNSKLGLDIEIATYR
(Parameters: blastp, nr protein database, expect=20000, no filter, PAM30)
Other BLAST web gateways

All matches to this query

Score	Mr(calc):	Delta	Sequence
96.7	2372.1569	0.0015	EYQEVMNSKLGLDIEIATYR
48.3	2372.1569	0.0015	EYQEVMNSKLGLDIEIATYR
46.9	2371.1729	0.9855	EYQEVMNSKLGLDIEIATYR

Mascot: http://www.matrixscience.com/

Mascot Search Results

Peptide View

MS/MS Fragmentation of **CQNSKLEAAVAQSEQQGEAALSDAR**
Found in **sp|P78385|KRT83_HUMAN**, Keratin, type II cuticular Hb3 OS=Homo sapiens GN=KRT83 PE=1 SV=2

Match to Query 38304: 2661.219312 from(888.080380,3+) intensity(1496396.0000) scans(14003) rtinseconds(2536) index(26321)
Title: 160219_Sunil_SDSI_A_Spectrum079627_scans__14003_RTINSECONDS=2536
Data file C:\\Sunil\\TKAP\\T\\T160219_Sunil_SDSI_A.mgf

Click mouse within plot area to zoom in by factor of two about that point
Or, Plot from 0 to 2800 Da Full range
Label all possible matches Label matches used for scoring

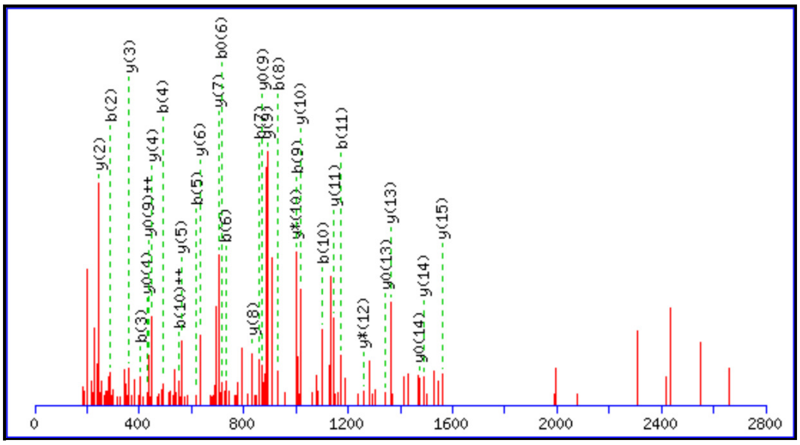

Monoisotopic mass of neutral peptide Mr(calc): 2661.2300
Fixed modifications: Carbamidomethyl (C) (apply to specified residues or termini only)
Variable modifications:
N3 : Deamidated (NQ)
Ions Score: 90 **Expect:** 1.9e-007
Matches : 32/276 fragment ions using 65 most intense peaks (help)

#	b	b^{++}	b*	b*$^{++}$	b^{0}	b^{0++}	Seq.	y	y^{++}	y*	y*$^{++}$	y^{0}	y^{0++}	#
1	161.0379	81.0226					C							25
2	289.0965	145.0519	272.0700	136.5386			Q	2502.2067	1251.6070	2485.1801	1243.0937	2484.1961	1242.6017	24
3	404.1234	202.5654	387.0969	194.0521			N	2374.1481	1187.5777	2357.1215	1179.0644	2356.1375	1178.5724	23
4	491.1555	246.0814	474.1289	237.5681	473.1449	237.0761	S	2259.1211	1130.0642	2242.0946	1121.5509	2241.1106	1121.0589	22
5	619.2504	310.1289	602.2239	301.6156	601.2399	301.1236	K	2172.0891	1086.5482	2155.0626	1078.0349	2154.0786	1077.5429	21
6	732.3345	366.6709	715.3080	358.1576	714.3239	357.6656	L	2043.9942	1022.5007	2026.9676	1013.9874	2025.9836	1013.4954	20
7	861.3771	431.1922	844.3505	422.6789	843.3665	422.1869	E	1930.9101	965.9587	1913.8835	957.4454	1912.8995	956.9534	19
8	932.4142	466.7107	915.3877	458.1975	914.4036	457.7055	A	1801.8675	901.4374	1784.8409	892.9241	1783.8569	892.4321	18
9	1003.4513	502.2293	986.4248	493.7160	985.4408	493.2240	A	1730.8304	865.9188	1713.8038	857.4056	1712.8198	856.9135	17
10	1102.5197	551.7635	1085.4932	543.2502	1084.5092	542.7582	V	1659.7933	830.4003	1642.7667	821.8870	1641.7827	821.3950	16
11	1173.5569	587.2821	1156.5303	578.7688	1155.5463	578.2768	A	1560.7249	780.8661	1543.6983	772.3528	1542.7143	771.8608	15
12	1301.6154	651.3114	1284.5889	642.7981	1283.6049	642.3061	Q	1489.6877	745.3475	1472.6612	736.8342	1471.6772	736.3422	14
13	1388.6475	694.8274	1371.6209	686.3141	1370.6369	685.8221	S	1361.6292	681.3182	1344.6026	672.8049	1343.6186	672.3129	13
14	1517.6900	759.3487	1500.6635	750.8354	1499.6795	750.3434	E	1274.5971	637.8022	1257.5706	629.2889	1256.5866	628.7969	12
15	1645.7486	823.3780	1628.7221	814.8647	1627.7381	814.3727	Q	1145.5545	573.2809	1128.5280	564.7676	1127.5440	564.2756	11
16	1773.8072	887.4072	1756.7807	878.8940	1755.7966	878.4020	Q	1017.4960	509.2516	1000.4694	500.7383	999.4854	500.2463	10
17	1830.8287	915.9180	1813.8021	907.4047	1812.8181	906.9127	G	889.4374	445.2223	872.4108	436.7091	871.4268	436.2170	9
18	1959.8713	980.4393	1942.8447	971.9260	1941.8607	971.4340	E	832.4159	416.7116	815.3894	408.1983	814.4054	407.7063	8
19	2030.9084	1015.9578	2013.8818	1007.4446	2012.8978	1006.9525	A	703.3733	352.1903	686.3468	343.6770	685.3628	343.1850	7
20	2101.9455	1051.4764	2084.9189	1042.9631	2083.9349	1042.4711	A	632.3362	316.6717	615.3097	308.1585	614.3257	307.6665	6
21	2215.0296	1108.0184	2198.0030	1099.5051	2197.0190	1099.0131	L	561.2991	281.1532	544.2726	272.6399	543.2885	272.1479	5
22	2302.0616	1151.5344	2285.0350	1143.0212	2284.0510	1142.5291	S	448.2150	224.6112	431.1885	216.0979	430.2045	215.6059	4
23	2417.0885	1209.0479	2400.0620	1200.5346	2399.0780	1200.0426	D	361.1830	181.0951	344.1565	172.5819	343.1724	172.0899	3
24	2488.1256	1244.5665	2471.0991	1236.0532	2470.1151	1235.5612	A	246.1561	123.5817	229.1295	115.0684			2
25							R	175.1190	88.0631	158.0924	79.5498			1

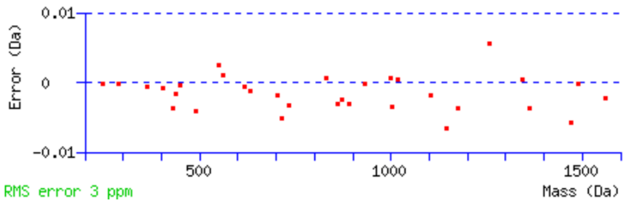

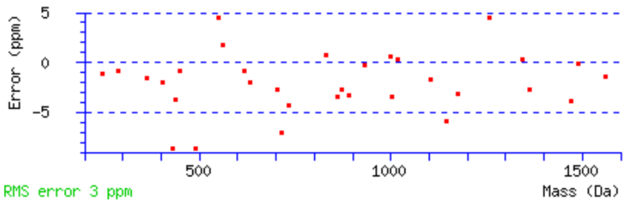

NCBI **BLAST** search of CQNSKLEAAVAQSEQQGEAALSDAR
(Parameters: blastp, nr protein database, expect=20000, no filter, PAM30)
Other BLAST web gateways

All matches to this query

Score	Mr(calc):	Delta	Sequence
90.0	2661.2300	-0.0107	CQNSKLEAAVAQSEQQGEAALSDAR
80.9	2661.2300	-0.0107	CQNSKLEAAVAQSEQQGEAALSDAR
42.7	2661.2300	-0.0107	CQNSKLEAAVAQSEQQGEAALSDAR
24.9	2661.2300	-0.0107	CQNSKLEAAVAQSEQQGEAALSDAR
23.9	2661.2300	-0.0107	CQNSKLEAAVAQSEQQGEAALSDAR

Mascot: http://www.matrixscience.com/

Mascot Search Results

Peptide View

MS/MS Fragmentation of **TVNALEIELQAQHNLR**
Found in **sp|O76009|KT33A_HUMAN**, Keratin, type I cuticular Ha3-I OS=Homo sapiens GN=KRT33A PE=2 SV=2

Match to Query 25670: 1848.968488 from(925.491520,2+) intensity(5108248.5000) scans(13281) rtinseconds(2412) index(25810)
Title: 160219_Sunil_SDSI_A_Spectrum079116_scans__13281_RTINSECONDS=2412
Data file C:\\Sunil\\TKAP\\T\\T160219_Sunil_SDSI_A.mgf

Click mouse within plot area to zoom in by factor of two about that point
Or, Plot from 100 to 1900 Da Full range
Label all possible matches ○ Label matches used for scoring ◉

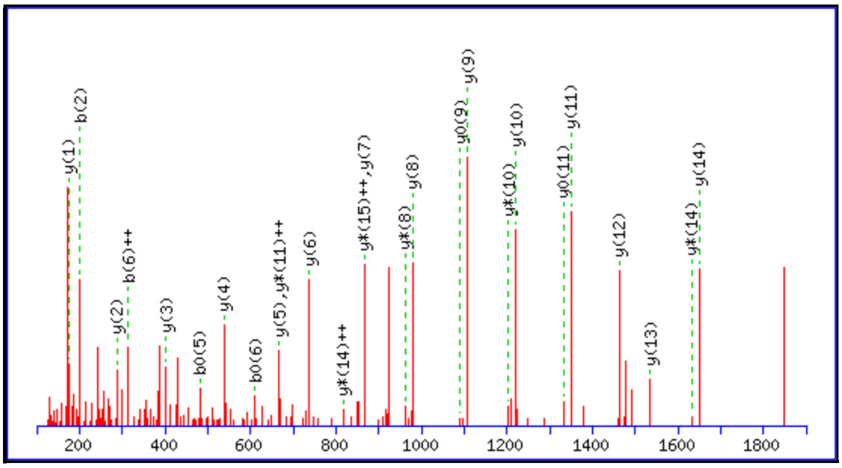

Monoisotopic mass of neutral peptide Mr(calc): 1848.9693
Fixed modifications: Carbamidomethyl (C) (apply to specified residues or termini only)
Variable modifications:
N3 : Deamidated (NQ)
Ions Score: 102 **Expect:** 1.4e-008
Matches : 26/160 fragment ions using 45 most intense peaks (help)

#	b	b++	b*	b*++	b0	b0++	Seq.	y	y++	y*	y*++	y0	y0++	#
1	102.0550	51.5311			84.0444	42.5258	T							16
2	**201.1234**	101.0653			183.1128	92.0600	V	1748.9290	874.9681	1731.9024	866.4549	1730.9184	865.9628	15
3	316.1503	158.5788	299.1238	150.0655	298.1397	149.5735	N	***1649.8606***	825.4339	1632.8340	816.9206	1631.8500	816.4286	14
4	387.1874	194.0974	370.1609	185.5841	369.1769	185.0921	A	***1534.8336***	767.9204	1517.8071	759.4072	1516.8231	758.9152	13
5	500.2715	250.6394	483.2449	242.1261	482.2609	241.6341	L	***1463.7965***	732.4019	1446.7700	723.8886	1445.7859	723.3966	12
6	629.3141	**315.1607**	612.2875	306.6474	611.3035	306.1554	E	***1350.7124***	675.8599	1333.6859	667.3466	1332.7019	666.8546	11
7	742.3981	371.7027	725.3716	363.1894	724.3876	362.6974	I	***1221.6698***	611.3386	1204.6433	602.8253	1203.6593	602.3333	10
8	871.4407	436.2240	854.4142	427.7107	853.4302	427.2187	E	***1108.5858***	554.7965	1091.5592	546.2833	1090.5752	545.7912	9
9	984.5248	492.7660	967.4983	484.2528	966.5142	483.7608	L	***979.5432***	490.2752	962.5166	481.7620			8
10	1112.5834	556.7953	1095.5568	548.2821	1094.5728	547.7900	Q	***866.4591***	433.7332	849.4326	425.2199			7
11	1183.6205	592.3139	1166.5939	583.8006	1165.6099	583.3086	A	***738.4005***	369.7039	721.3740	361.1906			6
12	1311.6791	656.3432	1294.6525	647.8299	1293.6685	647.3379	Q	***667.3634***	334.1854	650.3369	325.6721			5
13	1448.7380	724.8726	1431.7114	716.3594	1430.7274	715.8673	H	***539.3049***	270.1561	522.2783	261.6428			4
14	1562.7809	781.8941	1545.7544	773.3808	1544.7703	772.8888	N	***402.2459***	201.6266	385.2194	193.1133			3
15	1675.8650	838.4361	1658.8384	829.9229	1657.8544	829.4308	L	***288.2030***	144.6051	271.1765	136.0919			2
16							R	***175.1190***	88.0631	158.0924	79.5498			1

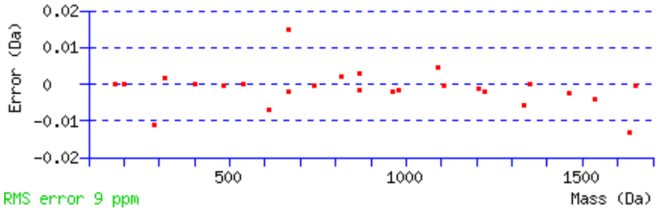

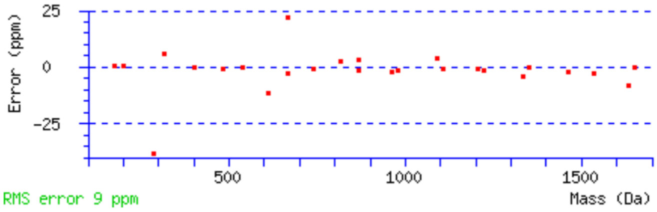

NCBI **BLAST** search of TVNALEIELQAQHNLR

(Parameters: blastp, nr protein database, expect=20000, no filter, PAM30)
Other BLAST web gateways

All matches to this query

Score	Mr(calc):	Delta	Sequence
102.1	1848.9693	-0.0008	TVNALEIELQAQHNLR
21.5	1848.9693	-0.0008	TVNALEIELQAQHNLR
10.4	1848.9693	-0.0008	TVNALEIELQAQHNLR
3.2	1846.9532	2.0152	IQSSGGPLQITMKMVPK
2.5	1848.9693	-0.0008	TVNALEIELQAQHNLR

Mascot: http://www.matrixscience.com/

Mascot Search Results

Peptide View

MS/MS Fragmentation of **SLLESEDCKLPSNPCATTNACDKSTGPCISNPCGLR**
Found in **sp|O76009|KT33A_HUMAN**, Keratin, type I cuticular Ha3-I OS=Homo sapiens GN=KRT33A PE=2 SV=2

Match to Query 43764: 4011.740176 from(1003.942320,4+) intensity(3286178.2500) scans(11710) rtinseconds(2141) index(24563)
Title: 160219_Sunil_SDSI_A_Spectrum077868_scans__11710_RTINSECONDS=2141
Data file C:\\Sunil\\TKAP\\T\\T160219_Sunil_SDSI_A.mgf

Click mouse within plot area to zoom in by factor of two about that point
Or, Plot from 200 to 3800 Da Full range
Label all possible matches Label matches used for scoring

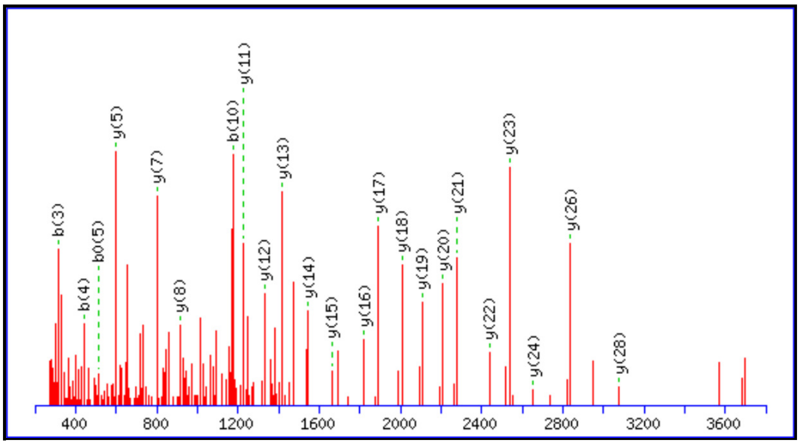

Monoisotopic mass of neutral peptide Mr(calc): 4010.7424
Fixed modifications: Carbamidomethyl (C) (apply to specified residues or termini only)
Variable modifications:
N13 : Deamidated (NQ)
N19 : Deamidated (NQ)
Ions Score: 156 **Expect:** 1.8e-014
Matches : 23/392 fragment ions using 30 most intense peaks (help)

#	b	b++	b*	b*++	b0	b0++	Seq.	y	y++	y*	y*++	y0	y0++	#
1	88.0393	44.5233			70.0287	35.5180	S							36
2	201.1234	101.0653			183.1128	92.0600	L	3924.7177	1962.8625	3907.6911	1954.3492	3906.7071	1953.8572	35
3	**314.2074**	157.6074			296.1969	148.6021	L	3811.6336	1906.3205	3794.6071	1897.8072	3793.6231	1897.3152	34
4	**443.2500**	222.1287			425.2395	213.1234	E	3698.5496	1849.7784	3681.5230	1841.2651	3680.5390	1840.7731	33
5	530.2821	265.6447			512.2715	256.6394	S	3569.5070	1785.2571	3552.4804	1776.7438	3551.4964	1776.2518	32
6	659.3246	330.1660			641.3141	321.1607	E	3482.4749	1741.7411	3465.4484	1733.2278	3464.4644	1732.7358	31
7	774.3516	387.6794			756.3410	378.6742	D	3353.4323	1677.2198	3336.4058	1668.7065	3335.4218	1668.2145	30
8	934.3822	467.6948			916.3717	458.6895	C	3238.4054	1619.7063	3221.3789	1611.1931	3220.3948	1610.7011	29
9	1062.4772	531.7422	1045.4507	523.2290	1044.4666	522.7370	K	***3078.3748***	1539.6910	3061.3482	1531.1777	3060.3642	1530.6857	28
10	**1175.5613**	588.2843	1158.5347	579.7710	1157.5507	579.2790	L	2950.2798	1475.6435	2933.2532	1467.1303	2932.2692	1466.6383	27
11	1272.6140	636.8107	1255.5875	628.2974	1254.6035	627.8054	P	***2837.1957***	1419.1015	2820.1692	1410.5882	2819.1852	1410.0962	26
12	1359.6461	680.3267	1342.6195	671.8134	1341.6355	671.3214	S	2740.1430	1370.5751	2723.1164	1362.0618	2722.1324	1361.5698	25
13	1474.6730	737.8401	1457.6465	729.3269	1456.6624	728.8349	N	***2653.1109***	1327.0591	2636.0844	1318.5458	2635.1004	1318.0538	24
14	1571.7258	786.3665	1554.6992	777.8532	1553.7152	777.3612	P	***2538.0840***	1269.5456	2521.0574	1261.0324	2520.0734	1260.5404	23
15	1731.7564	866.3818	1714.7299	857.8686	1713.7458	857.3766	C	***2441.0312***	1221.0193	2424.0047	1212.5060	2423.0207	1212.0140	22
16	1802.7935	901.9004	1785.7670	893.3871	1784.7830	892.8951	A	***2281.0006***	1141.0039	2263.9740	1132.4907	2262.9900	1131.9986	21
17	1903.8412	952.4242	1886.8147	943.9110	1885.8306	943.4190	T	***2209.9635***	1105.4854	2192.9369	1096.9721	2191.9529	1096.4801	20
18	2004.8889	1002.9481	1987.8623	994.4348	1986.8783	993.9428	T	***2108.9158***	1054.9615	2091.8892	1046.4483	2090.9052	1045.9562	19
19	2119.9158	1060.4616	2102.8893	1051.9483	2101.9053	1051.4563	N	***2007.8681***	1004.4377	1990.8416	995.9244	1989.8575	995.4324	18
20	2190.9529	1095.9801	2173.9264	1087.4668	2172.9424	1086.9748	A	***1892.8412***	946.9242	1875.8146	938.4109	1874.8306	937.9189	17
21	2350.9836	1175.9954	2333.9570	1167.4822	2332.9730	1166.9902	C	***1821.8041***	911.4057	1804.7775	902.8924	1803.7935	902.4004	16
22	2466.0105	1233.5089	2448.9840	1224.9956	2448.0000	1224.5036	D	***1661.7734***	831.3903	1644.7469	822.8771	1643.7628	822.3851	15
23	2594.1055	1297.5564	2577.0789	1289.0431	2576.0949	1288.5511	K	***1546.7465***	773.8769	1529.7199	765.3636	1528.7359	764.8716	14
24	2681.1375	1341.0724	2664.1110	1332.5591	2663.1270	1332.0671	S	***1418.6515***	709.8294	1401.6249	701.3161	1400.6409	700.8241	13
25	2782.1852	1391.5962	2765.1587	1383.0830	2764.1746	1382.5910	T	***1331.6195***	666.3134	1314.5929	657.8001	1313.6089	657.3081	12
26	2839.2067	1420.1070	2822.1801	1411.5937	2821.1961	1411.1017	G	***1230.5718***	615.7895	1213.5452	607.2763	1212.5612	606.7842	11

**27**	2936.2594	1468.6334	2919.2329	1460.1201	2918.2489	1459.6281	**P**	1173.5503	587.2788	1156.5238	578.7655	1155.5398	578.2735	**10**
**28**	3096.2901	1548.6487	3079.2635	1540.1354	3078.2795	1539.6434	**C**	1076.4976	538.7524	1059.4710	530.2391	1058.4870	529.7471	**9**
**29**	3209.3741	1605.1907	3192.3476	1596.6774	3191.3636	1596.1854	**I**	***916.4669***	458.7371	899.4404	450.2238	898.4563	449.7318	**8**
**30**	3296.4062	1648.7067	3279.3796	1640.1934	3278.3956	1639.7014	**S**	***803.3828***	402.1951	786.3563	393.6818	785.3723	393.1898	**7**
**31**	3410.4491	1705.7282	3393.4226	1697.2149	3392.4385	1696.7229	**N**	716.3508	358.6790	699.3243	350.1658			**6**
**32**	3507.5019	1754.2546	3490.4753	1745.7413	3489.4913	1745.2493	**P**	***602.3079***	301.6576	585.2813	293.1443			**5**
**33**	3667.5325	1834.2699	3650.5060	1825.7566	3649.5219	1825.2646	**C**	505.2551	253.1312	488.2286	244.6179			**4**
**34**	3724.5540	1862.7806	3707.5274	1854.2674	3706.5434	1853.7753	**G**	345.2245	173.1159	328.1979	164.6026			**3**
**35**	3837.6380	1919.3227	3820.6115	1910.8094	3819.6275	1910.3174	**L**	288.2030	144.6051	271.1765	136.0919			**2**
**36**							**R**	175.1190	88.0631	158.0924	79.5498			**1**

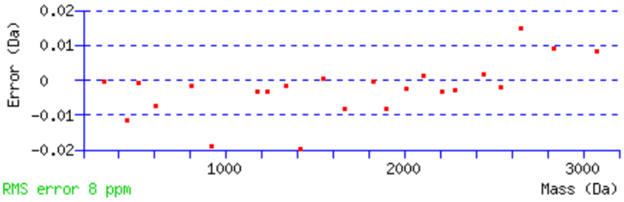

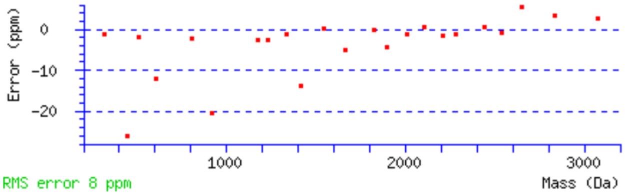

NCBI **BLAST** search of SLLESEDCKLPSNPCATTNACDKSTGPCISNPCGLR
(Parameters: blastp, nr protein database, expect=20000, no filter, PAM30)
Other BLAST web gateways

All matches to this query

Score	Mr(calc):	Delta	Sequence
155.7	4010.7424	0.9978	SLLESEDCKLPSNPCATTNACDKSTGPCISNPCGLR
139.8	4009.7584	1.9818	SLLESEDCKLPSNPCATTNACDKSTGPCISNPCGLR
62.3	4009.7584	1.9818	SLLESEDCKLPSNPCATTNACDKSTGPCISNPCGLR
39.8	4010.7424	0.9978	SLLESEDCKLPSNPCATTNACDKSTGPCISNPCGLR
30.9	4009.7584	1.9818	SLLESEDCKLPSNPCATTNACDKSTGPCISNPCGLR
0.5	4010.7424	0.9978	SLLESEDCKLPSNPCATTNACDKSTGPCISNPCGLR

Mascot: http://www.matrixscience.com/

Mascot Search Results

Peptide View

MS/MS Fragmentation of **ARLECEINTYR**
Found in **sp|Q15323|K1H1_HUMAN**, Keratin, type I cuticular Ha1 OS=Homo sapiens GN=KRT31 PE=2 SV=3

Match to Query 15235: 1424.670748 from(713.342650,2+) intensity(1864242.1250) scans(6312) rtinseconds(1242) index(4457)
Title: 160219_Sunil_SDSI_A_Spectrum056565_scans__6312_RTINSECONDS=1242
Data file C:\\Sunil\\TKAP\\T\\T160219_Sunil_SDSI_A.mgf

Click mouse within plot area to zoom in by factor of two about that point
Or, Plot from 100 to 1500 Da Full range
Label all possible matches Label matches used for scoring

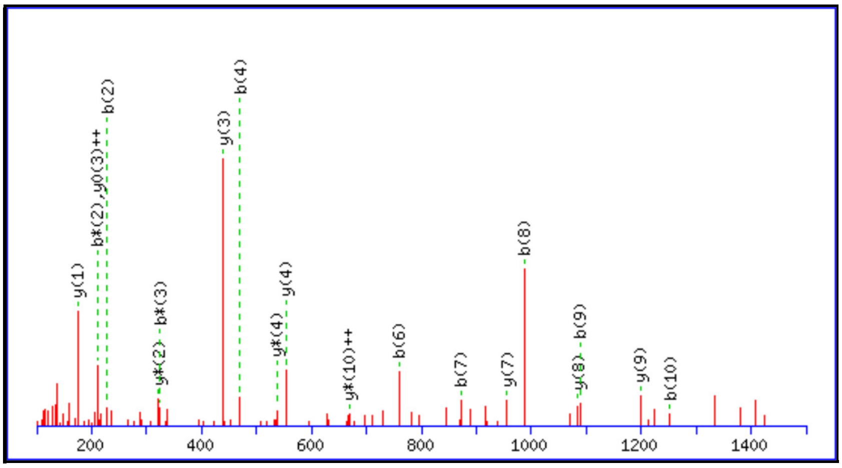

Monoisotopic mass of neutral peptide Mr(calc): 1424.6718
Fixed modifications: Carbamidomethyl (C) (apply to specified residues or termini only)
Variable modifications:
N8 : Deamidated (NQ)
Ions Score: 52 **Expect:** 0.0011
Matches : 19/108 fragment ions using 27 most intense peaks (help)

#	b	b^{++}	b*	b^{*++}	b^{0}	b^{0++}	Seq.	y	y^{++}	y*	y^{*++}	y^{0}	y^{0++}	#
1	72.0444	36.5258					A							11
2	***228.1455***	114.5764	211.1190	106.0631			R	1354.6420	677.8246	1337.6154	669.3114	1336.6314	668.8193	10
3	341.2296	171.1184	324.2030	162.6051			L	***1198.5409***	599.7741	1181.5143	591.2608	1180.5303	590.7688	9
4	***470.2722***	235.6397	453.2456	227.1264	452.2616	226.6344	E	***1085.4568***	543.2320	1068.4303	534.7188	1067.4462	534.2268	8
5	630.3028	315.6550	613.2763	307.1418	612.2922	306.6498	C	***956.4142***	478.7107	939.3877	470.1975	938.4036	469.7055	7
6	***759.3454***	380.1763	742.3189	371.6631	741.3348	371.1711	E	796.3836	398.6954	779.3570	390.1821	778.3730	389.6901	6
7	***872.4295***	436.7184	855.4029	428.2051	854.4189	427.7131	I	667.3410	334.1741	650.3144	325.6608	649.3304	325.1688	5
8	***987.4564***	494.2318	970.4299	485.7186	969.4458	485.2266	N	***554.2569***	277.6321	537.2304	269.1188	536.2463	268.6628	4
9	***1088.5041***	544.7557	1071.4775	536.2424	1070.4935	535.7504	T	***439.2300***	220.1186	422.2034	211.6053	421.2194	211.1133	3
10	***1251.5674***	626.2873	1234.5409	617.7741	1233.5568	617.2821	Y	338.1823	169.5948	321.1557	161.0815			2
11							R	***175.1190***	88.0631	158.0924	79.5498			1

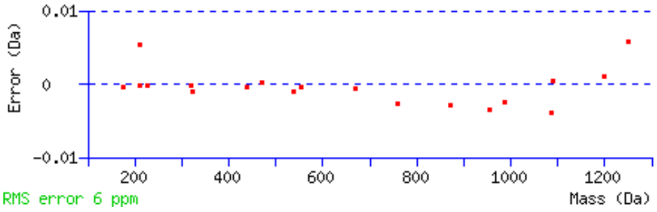

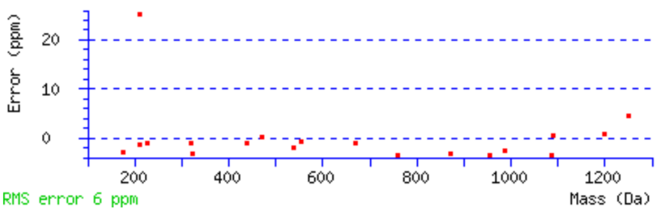

NCBI **BLAST** search of ARLECEINTYR
(Parameters: blastp, nr protein database, expect=20000, no filter, PAM30)
Other BLAST web gateways

All matches to this query

Score	Mr(calc):	Delta	Sequence

52.1	1424.6718	-0.0010	ARLECEINTYR
5.9	1424.6606	0.0102	TGQPMINLYTDR
5.9	1424.6606	0.0102	TGQPMINLYTDR
3.6	1424.6718	-0.0010	EMNIQQLTNYR
0.6	1423.6806	0.9902	LWITMNEPYTR

Mascot: http://www.matrixscience.com/

Mascot Search Results

Peptide View

MS/MS Fragmentation of **ATAENEFVALK**
Found in **sp|O43790|KRT86_HUMAN**, Keratin, type II cuticular Hb6 OS=Homo sapiens GN=KRT86 PE=1 SV=1

Match to Query 8089: 1192.597268 from(597.305910,2+) intensity(1444807.5000) scans(11561) rtinseconds(2141) index(8971)
Title: 160219_Sunil_SDSI_A_Spectrum061084_scans__11561_RTINSECONDS=2141
Data file C:\\Sunil\\TKAP\\T\\T160219_Sunil_SDSI_A.mgf

Click mouse within plot area to zoom in by factor of two about that point
Or, Plot from 50 to 1050 Da Full range
Label all possible matches ○ Label matches used for scoring ⦿

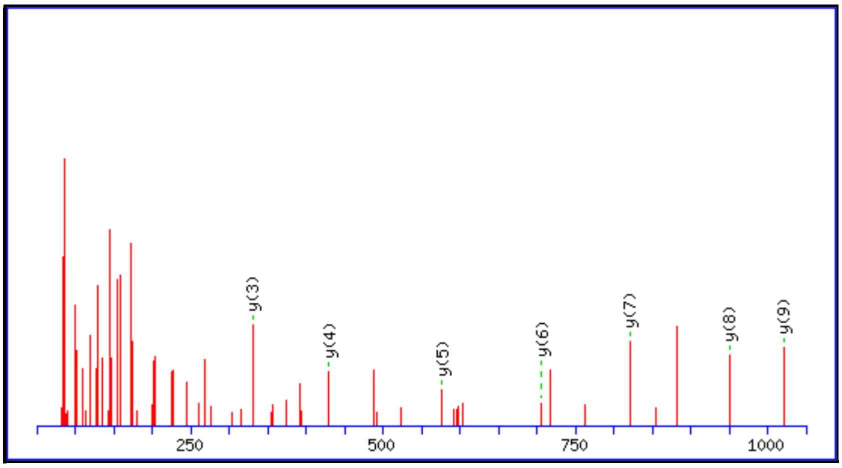

Monoisotopic mass of neutral peptide Mr(calc): 1192.5975
Fixed modifications: Carbamidomethyl (C) (apply to specified residues or termini only)
Variable modifications:
N5 : Deamidated (NQ)
Ions Score: 36 **Expect:** 0.054
Matches : 7/100 fragment ions using 18 most intense peaks (help)

#	b	b^{++}	b*	b^{*++}	b^0	b^{0++}	Seq.	y	y^{++}	y*	y^{*++}	y^0	y^{0++}	#
1	72.0444	36.5258					A							11
2	173.0921	87.0497			155.0815	78.0444	T	1122.5677	561.7875	1105.5412	553.2742	1104.5572	552.7822	10
3	244.1292	122.5682			226.1186	113.5629	A	***1021.5201***	511.2637	1004.4935	502.7504	1003.5095	502.2584	9
4	373.1718	187.0895			355.1612	178.0842	E	***950.4829***	475.7451	933.4564	467.2318	932.4724	466.7398	8
5	488.1987	244.6030	471.1722	236.0897	470.1882	235.5977	N	***821.4403***	411.2238	804.4138	402.7105	803.4298	402.2185	7
6	617.2413	309.1243	600.2148	300.6110	599.2307	300.1190	E	***706.4134***	353.7103	689.3869	345.1971	688.4028	344.7051	6
7	764.3097	382.6585	747.2832	374.1452	746.2992	373.6532	F	***577.3708***	289.1890	560.3443	280.6758			5
8	863.3781	432.1927	846.3516	423.6794	845.3676	423.1874	V	***430.3024***	215.6548	413.2758	207.1416			4
9	934.4153	467.7113	917.3887	459.1980	916.4047	458.7060	A	***331.2340***	166.1206	314.2074	157.6074			3
10	1047.4993	524.2533	1030.4728	515.7400	1029.4888	515.2480	L	260.1969	130.6021	243.1703	122.0888			2
11							K	147.1128	74.0600	130.0863	65.5468			1

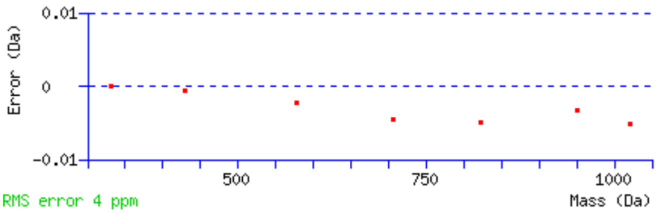

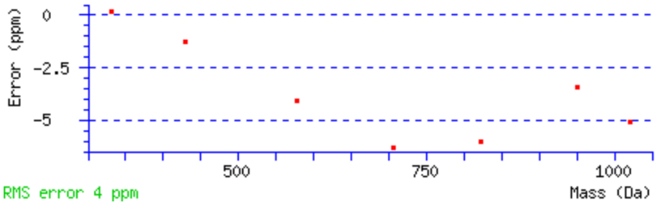

NCBI **BLAST** search of ATAENEFVALK
(Parameters: blastp, nr protein database, expect=20000, no filter, PAM30)
Other BLAST web gateways

All matches to this query

Score	Mr(calc):	Delta	Sequence

35.9	1192.5975	-0.0003	ATAENEFVALK
35.9	1192.5975	-0.0003	TAAENEFVALK

Mascot: http://www.matrixscience.com/

Mascot Search Results

Peptide View

MS/MS Fragmentation of **ATAENEFVALKK**
Found in **sp|O43790|KRT86_HUMAN**, Keratin, type II cuticular Hb6 OS=Homo sapiens GN=KRT86 PE=1 SV=1

Match to Query 12121: 1320.691272 from(441.237700,3+) intensity(28649608.0000) scans(8479) rtinseconds(1613) index(6337)
Title: 160219_Sunil_SDSI_A_Spectrum058445_scans__8479_RTINSECONDS=1613
Data file C:\\Sunil\\TKAP\\T\\T160219_Sunil_SDSI_A.mgf

Click mouse within plot area to zoom in by factor of two about that point
Or, Plot from 100 to 1200 Da Full range
Label all possible matches ○ Label matches used for scoring ◉

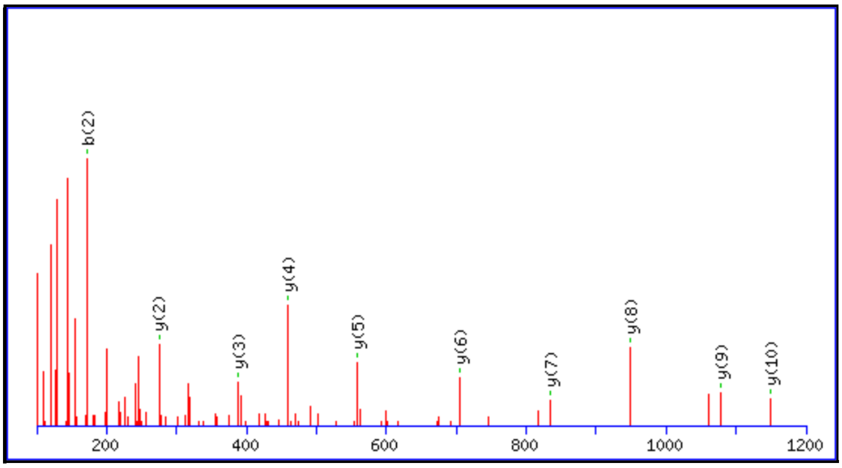

Monoisotopic mass of neutral peptide Mr(calc): 1320.6925
Fixed modifications: Carbamidomethyl (C) (apply to specified residues or termini only)
Variable modifications:
N5 : Deamidated (NQ)
Ions Score: 94 **Expect:** 9.1e-008
Matches : 10/110 fragment ions using 11 most intense peaks (help)

#	b	b++	b*	b*++	b0	b0++	Seq.	y	y++	y*	y*++	y0	y0++	#
1	72.0444	36.5258					A							12
2	**173.0921**	87.0497			155.0815	78.0444	T	1250.6627	625.8350	1233.6361	617.3217	1232.6521	616.8297	11
3	244.1292	122.5682			226.1186	113.5629	A	***1149.6150***	575.3111	1132.5885	566.7979	1131.6045	566.3059	10
4	373.1718	187.0895			355.1612	178.0842	E	***1078.5779***	539.7926	1061.5514	531.2793	1060.5673	530.7873	9
5	488.1987	244.6030	471.1722	236.0897	470.1882	235.5977	N	***949.5353***	475.2713	932.5088	466.7580	931.5247	466.2660	8
6	617.2413	309.1243	600.2148	300.6110	599.2307	300.1190	E	***834.5084***	417.7578	817.4818	409.2445	816.4978	408.7525	7
7	764.3097	382.6585	747.2832	374.1452	746.2992	373.6532	F	***705.4658***	353.2365	688.4392	344.7232			6
8	863.3781	432.1927	846.3516	423.6794	845.3676	423.1874	V	***558.3974***	279.7023	541.3708	271.1890			5
9	934.4153	467.7113	917.3887	459.1980	916.4047	458.7060	A	***459.3289***	230.1681	442.3024	221.6548			4
10	1047.4993	524.2533	1030.4728	515.7400	1029.4888	515.2480	L	***388.2918***	194.6496	371.2653	186.1363			3
11	1175.5943	588.3008	1158.5677	579.7875	1157.5837	579.2955	K	***275.2078***	138.1075	258.1812	129.5942			2
12							K	147.1128	74.0600	130.0863	65.5468			1

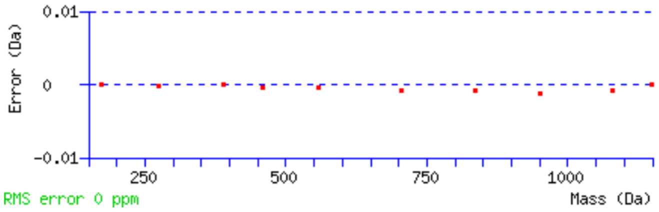

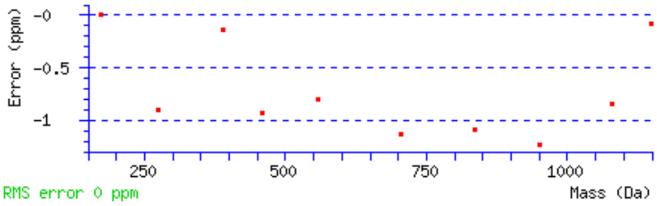

NCBI **BLAST** search of ATAENEFVALKK
(Parameters: blastp, nr protein database, expect=20000, no filter, PAM30)
Other BLAST web gateways

All matches to this query

Score	Mr(calc):	Delta	Sequence
93.8	1320.6925	-0.0012	ATAENEFVALKK
93.8	1320.6925	-0.0012	TAAENEFVALKK

Mascot: http://www.matrixscience.com/

Mascot Search Results

Peptide View

MS/MS Fragmentation of **ATAENEFVALKK**
Found in **sp|P78385|KRT83_HUMAN**, Keratin, type II cuticular Hb3 OS=Homo sapiens GN=KRT83 PE=1 SV=2

Match to Query 12121: 1320.691272 from(441.237700,3+) intensity(28649608.0000) scans(8479) rtinseconds(1613) index(6337)
Title: 160219_Sunil_SDSI_A_Spectrum058445_scans__8479_RTINSECONDS=1613
Data file C:\\Sunil\\TKAP\\T\\T160219_Sunil_SDSI_A.mgf

Click mouse within plot area to zoom in by factor of two about that point
Or, Plot from 100 to 1200 Da Full range
Label all possible matches ○ Label matches used for scoring ◉

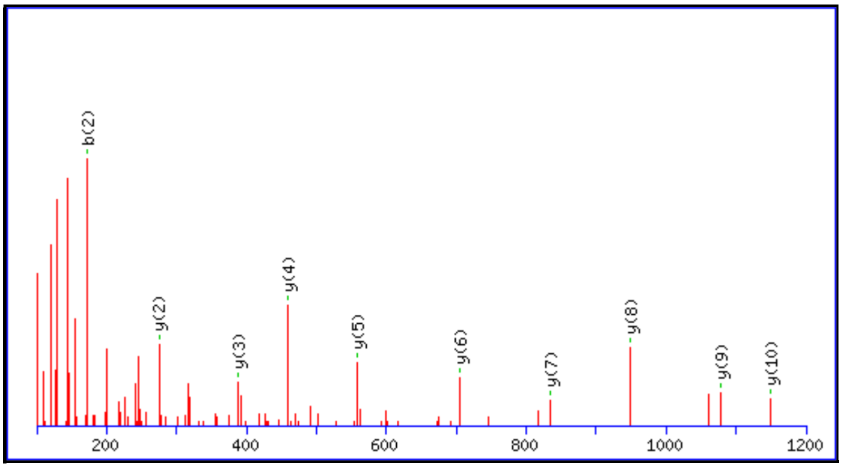

Monoisotopic mass of neutral peptide Mr(calc): 1320.6925
Fixed modifications: Carbamidomethyl (C) (apply to specified residues or termini only)
Variable modifications:
N5 : Deamidated (NQ)
Ions Score: 94 **Expect:** 9.1e-008
Matches : 10/110 fragment ions using 11 most intense peaks (help)

#	b	b^{++}	b^{*}	b^{*++}	b^{0}	b^{0++}	Seq.	y	y^{++}	y^{*}	y^{*++}	y^{0}	y^{0++}	#
1	72.0444	36.5258					A							12
2	**173.0921**	87.0497			155.0815	78.0444	T	1250.6627	625.8350	1233.6361	617.3217	1232.6521	616.8297	11
3	244.1292	122.5682			226.1186	113.5629	A	**1149.6150**	575.3111	1132.5885	566.7979	1131.6045	566.3059	10
4	373.1718	187.0895			355.1612	178.0842	E	**1078.5779**	539.7926	1061.5514	531.2793	1060.5673	530.7873	9
5	488.1987	244.6030	471.1722	236.0897	470.1882	235.5977	N	**949.5353**	475.2713	932.5088	466.7580	931.5247	466.2660	8
6	617.2413	309.1243	600.2148	300.6110	599.2307	300.1190	E	**834.5084**	417.7578	817.4818	409.2445	816.4978	408.7525	7
7	764.3097	382.6585	747.2832	374.1452	746.2992	373.6532	F	**705.4658**	353.2365	688.4392	344.7232			6
8	863.3781	432.1927	846.3516	423.6794	845.3676	423.1874	V	**558.3974**	279.7023	541.3708	271.1890			5
9	934.4153	467.7113	917.3887	459.1980	916.4047	458.7060	A	**459.3289**	230.1681	442.3024	221.6548			4
10	1047.4993	524.2533	1030.4728	515.7400	1029.4888	515.2480	L	**388.2918**	194.6496	371.2653	186.1363			3
11	1175.5943	588.3008	1158.5677	579.7875	1157.5837	579.2955	K	**275.2078**	138.1075	258.1812	129.5942			2
12							K	147.1128	74.0600	130.0863	65.5468			1

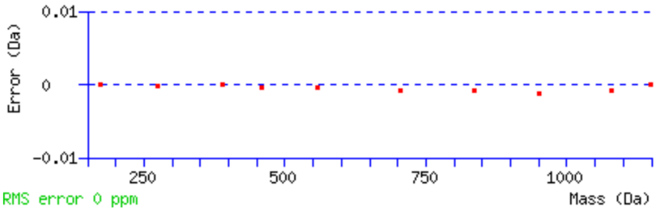

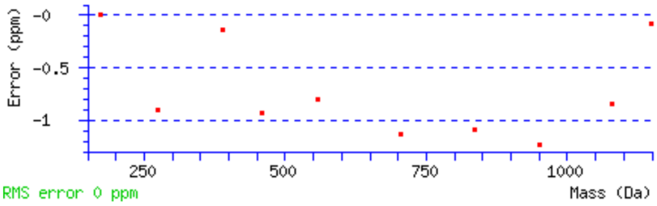

NCBI **BLAST** search of ATAENEFVALKK
(Parameters: blastp, nr protein database, expect=20000, no filter, PAM30)
Other BLAST web gateways

All matches to this query

Score	Mr(calc):	Delta	Sequence
93.8	1320.6925	-0.0012	ATAENEFVALKK
93.8	1320.6925	-0.0012	TAAENEFVALKK

Mascot: http://www.matrixscience.com/

Mascot Search Results

Peptide View

MS/MS Fragmentation of **ATAENEFVALKKDVDCAYLR**
Found in **sp|O43790|KRT86_HUMAN**, Keratin, type II cuticular Hb6 OS=Homo sapiens GN=KRT86 PE=1 SV=1

Match to Query 34219: 2313.136896 from(579.291500,4+) intensity(2008287.1250) scans(14443) rtinseconds(2637) index(11339)
Title: 160219_Sunil_SDSI_A_Spectrum063466_scans__14443_RTINSECONDS=2637
Data file C:\\Sunil\\TKAP\\T\\T160219_Sunil_SDSI_A.mgf

Click mouse within plot area to zoom in by factor of two about that point
Or, Plot from 0 to 2200 Da Full range
Label all possible matches ○ Label matches used for scoring ◉

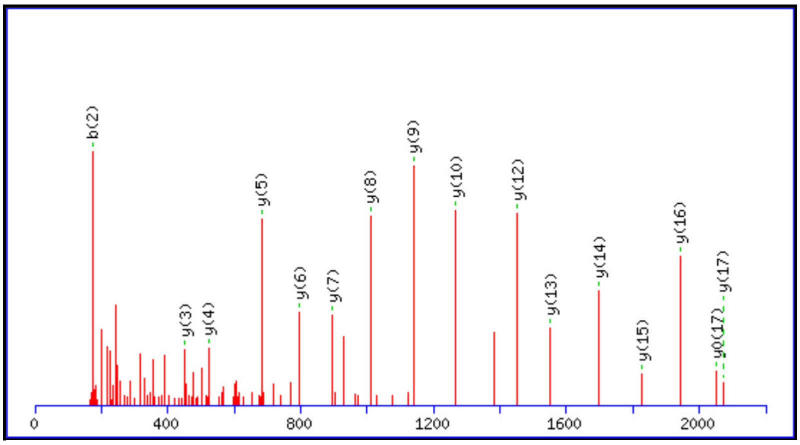

Monoisotopic mass of neutral peptide Mr(calc): 2313.1311
Fixed modifications: Carbamidomethyl (C) (apply to specified residues or termini only)
Variable modifications:
N5 : Deamidated (NQ)
Ions Score: 142 **Expect:** 1.8e-012
Matches : 16/208 fragment ions using 18 most intense peaks (help)

#	b	b^{++}	b^*	b^{*++}	b^0	b^{0++}	Seq.	y	y^{++}	y^*	y^{*++}	y^0	y^{0++}	#
1	72.0444	36.5258					A							20
2	**173.0921**	87.0497			155.0815	78.0444	T	2243.1013	1122.0543	2226.0747	1113.5410	2225.0907	1113.0490	19
3	244.1292	122.5682			226.1186	113.5629	A	2142.0536	1071.5304	2125.0270	1063.0172	2124.0430	1062.5251	18
4	373.1718	187.0895			355.1612	178.0842	E	***2071.0165***	1036.0119	2053.9899	1027.4986	2053.0059	1027.0066	17
5	488.1987	244.6030	471.1722	236.0897	470.1882	235.5977	N	***1941.9739***	971.4906	1924.9473	962.9773	1923.9633	962.4853	16
6	617.2413	309.1243	600.2148	300.6110	599.2307	300.1190	E	***1826.9469***	913.9771	1809.9204	905.4638	1808.9364	904.9718	15
7	764.3097	382.6585	747.2832	374.1452	746.2992	373.6532	F	***1697.9043***	849.4558	1680.8778	840.9425	1679.8938	840.4505	14
8	863.3781	432.1927	846.3516	423.6794	845.3676	423.1874	V	***1550.8359***	775.9216	1533.8094	767.4083	1532.8254	766.9163	13
9	934.4153	467.7113	917.3887	459.1980	916.4047	458.7060	A	***1451.7675***	726.3874	1434.7410	717.8741	1433.7569	717.3821	12
10	1047.4993	524.2533	1030.4728	515.7400	1029.4888	515.2480	L	1380.7304	690.8688	1363.7038	682.3556	1362.7198	681.8636	11
11	1175.5943	588.3008	1158.5677	579.7875	1157.5837	579.2955	K	***1267.6463***	634.3268	1250.6198	625.8135	1249.6358	625.3215	10
12	1303.6892	652.3483	1286.6627	643.8350	1285.6787	643.3430	K	***1139.5514***	570.2793	1122.5248	561.7660	1121.5408	561.2740	9
13	1418.7162	709.8617	1401.6896	701.3485	1400.7056	700.8564	D	***1011.4564***	506.2318	994.4299	497.7186	993.4458	497.2266	8
14	1517.7846	759.3959	1500.7581	750.8827	1499.7740	750.3907	V	***896.4295***	448.7184	879.4029	440.2051	878.4189	439.7131	7
15	1632.8115	816.9094	1615.7850	808.3961	1614.8010	807.9041	D	***797.3611***	399.1842	780.3345	390.6709	779.3505	390.1789	6
16	1792.8422	896.9247	1775.8156	888.4115	1774.8316	887.9195	C	***682.3341***	341.6707	665.3076	333.1574			5
17	1863.8793	932.4433	1846.8528	923.9300	1845.8687	923.4380	A	***522.3035***	261.6554	505.2769	253.1421			4
18	2026.9426	1013.9750	2009.9161	1005.4617	2008.9321	1004.9697	Y	***451.2663***	226.1368	434.2398	217.6235			3
19	2140.0267	1070.5170	2123.0002	1062.0037	2122.0161	1061.5117	L	288.2030	144.6051	271.1765	136.0919			2
20							R	175.1190	88.0631	158.0924	79.5498			1

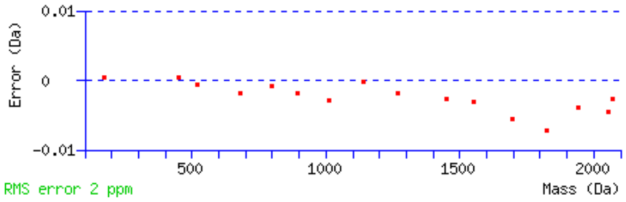

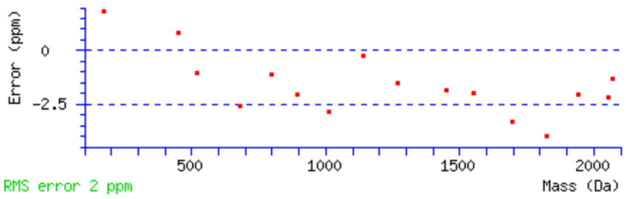

NCBI **BLAST** search of ATAENEFVALKKDVDCAYLR
(Parameters: blastp, nr protein database, expect=20000, no filter, PAM30)
Other BLAST web gateways

All matches to this query

Score	Mr(calc):	Delta	Sequence
141.6	2313.1311	0.0058	ATAENEFVALKKDVDCAYLR
104.9	2312.1470	0.9898	ATAENEFVALKKDVDCAYLR

Mascot: http://www.matrixscience.com/

Mascot Search Results

Peptide View

MS/MS Fragmentation of **ATAENEFVALKKDVDCAYLR**
Found in **sp|P78385|KRT83_HUMAN**, Keratin, type II cuticular Hb3 OS=Homo sapiens GN=KRT83 PE=1 SV=2

Match to Query 34219: 2313.136896 from(579.291500,4+) intensity(2008287.1250) scans(14443) rtinseconds(2637) index(11339)
Title: 160219_Sunil_SDSI_A_Spectrum063466_scans__14443_RTINSECONDS=2637
Data file C:\\Sunil\\TKAP\\T\\T160219_Sunil_SDSI_A.mgf

Click mouse within plot area to zoom in by factor of two about that point
Or, Plot from 0 to 2200 Da Full range
Label all possible matches ○ Label matches used for scoring ◉

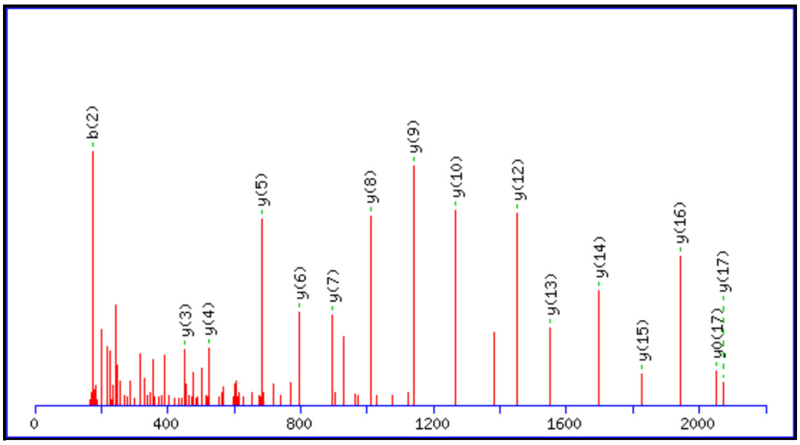

Monoisotopic mass of neutral peptide Mr(calc): 2313.1311
Fixed modifications: Carbamidomethyl (C) (apply to specified residues or termini only)
Variable modifications:
N5 : Deamidated (NQ)
Ions Score: 142 **Expect:** 1.8e-012
Matches : 16/208 fragment ions using 18 most intense peaks (help)

#	b	b++	b*	b*++	b0	b0++	Seq.	y	y++	y*	y*++	y0	y0++	#
1	72.0444	36.5258					A							20
2	**173.0921**	87.0497			155.0815	78.0444	T	2243.1013	1122.0543	2226.0747	1113.5410	2225.0907	1113.0490	19
3	244.1292	122.5682			226.1186	113.5629	A	2142.0536	1071.5304	2125.0270	1063.0172	2124.0430	1062.5251	18
4	373.1718	187.0895			355.1612	178.0842	E	***2071.0165***	1036.0119	2053.9899	1027.4986	2053.0059	1027.0066	17
5	488.1987	244.6030	471.1722	236.0897	470.1882	235.5977	N	***1941.9739***	971.4906	1924.9473	962.9773	1923.9633	962.4853	16
6	617.2413	309.1243	600.2148	300.6110	599.2307	300.1190	E	***1826.9469***	913.9771	1809.9204	905.4638	1808.9364	904.9718	15
7	764.3097	382.6585	747.2832	374.1452	746.2992	373.6532	F	***1697.9043***	849.4558	1680.8778	840.9425	1679.8938	840.4505	14
8	863.3781	432.1927	846.3516	423.6794	845.3676	423.1874	V	***1550.8359***	775.9216	1533.8094	767.4083	1532.8254	766.9163	13
9	934.4153	467.7113	917.3887	459.1980	916.4047	458.7060	A	***1451.7675***	726.3874	1434.7410	717.8741	1433.7569	717.3821	12
10	1047.4993	524.2533	1030.4728	515.7400	1029.4888	515.2480	L	1380.7304	690.8688	1363.7038	682.3556	1362.7198	681.8636	11
11	1175.5943	588.3008	1158.5677	579.7875	1157.5837	579.2955	K	***1267.6463***	634.3268	1250.6198	625.8135	1249.6358	625.3215	10
12	1303.6892	652.3483	1286.6627	643.8350	1285.6787	643.3430	K	***1139.5514***	570.2793	1122.5248	561.7660	1121.5408	561.2740	9
13	1418.7162	709.8617	1401.6896	701.3485	1400.7056	700.8564	D	***1011.4564***	506.2318	994.4299	497.7186	993.4458	497.2266	8
14	1517.7846	759.3959	1500.7581	750.8827	1499.7740	750.3907	V	***896.4295***	448.7184	879.4029	440.2051	878.4189	439.7131	7
15	1632.8115	816.9094	1615.7850	808.3961	1614.8010	807.9041	D	***797.3611***	399.1842	780.3345	390.6709	779.3505	390.1789	6
16	1792.8422	896.9247	1775.8156	888.4115	1774.8316	887.9195	C	***682.3341***	341.6707	665.3076	333.1574			5
17	1863.8793	932.4433	1846.8528	923.9300	1845.8687	923.4380	A	***522.3035***	261.6554	505.2769	253.1421			4
18	2026.9426	1013.9750	2009.9161	1005.4617	2008.9321	1004.9697	Y	***451.2663***	226.1368	434.2398	217.6235			3
19	2140.0267	1070.5170	2123.0002	1062.0037	2122.0161	1061.5117	L	288.2030	144.6051	271.1765	136.0919			2
20							R	175.1190	88.0631	158.0924	79.5498			1

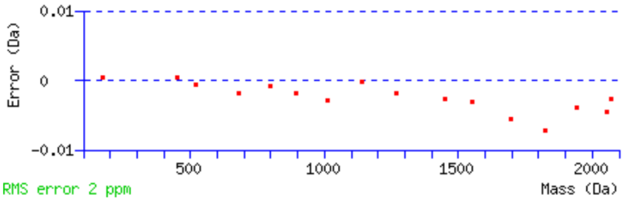

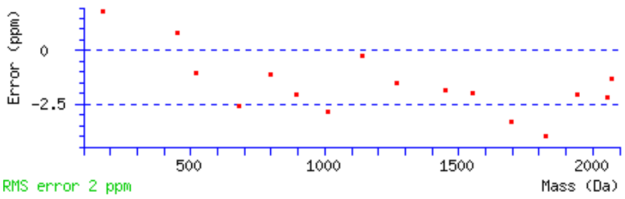

NCBI **BLAST** search of ATAENEFVALKKDVDCAYLR
(Parameters: blastp, nr protein database, expect=20000, no filter, PAM30)
Other BLAST web gateways

All matches to this query

Score	Mr(calc):	Delta	Sequence
141.6	2313.1311	0.0058	ATAENEFVALKKDVDCAYLR
104.9	2312.1470	0.9898	ATAENEFVALKKDVDCAYLR

Mascot: http://www.matrixscience.com/

Mascot Search Results

Peptide View

MS/MS Fragmentation of **ATAENEFVALKKDVDCAYLR**
Found in **tr|A0A087X106|A0A087X106_HUMAN**, Keratin, type II cuticular Hb1 OS=Homo sapiens GN=KRT81 PE=1 SV=1

Match to Query 34219: 2313.136896 from(579.291500,4+) intensity(2008287.1250) scans(14443) rtinseconds(2637) index(11339)
Title: 160219_Sunil_SDSI_A_Spectrum063466_scans__14443_RTINSECONDS=2637
Data file C:\\Sunil\\TKAP\\T\\T160219_Sunil_SDSI_A.mgf

Click mouse within plot area to zoom in by factor of two about that point
Or, Plot from 0 to 2200 Da Full range
Label all possible matches Label matches used for scoring

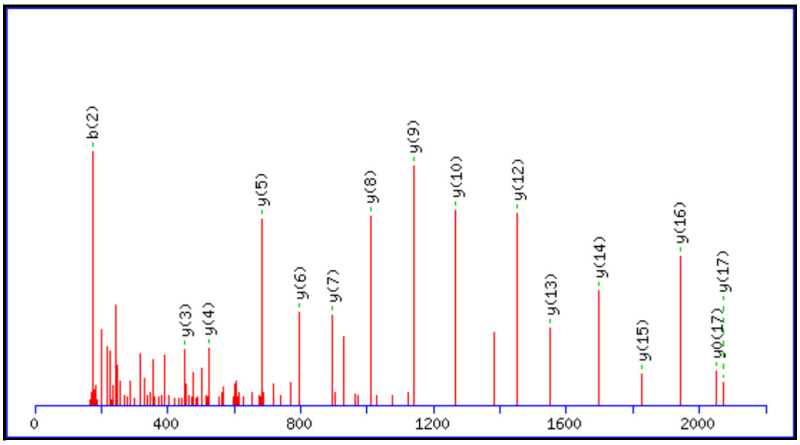

Monoisotopic mass of neutral peptide Mr(calc): 2313.1311
Fixed modifications: Carbamidomethyl (C) (apply to specified residues or termini only)
Variable modifications:
N5 : Deamidated (NQ)
Ions Score: 142 **Expect:** 1.8e-012
Matches : 16/208 fragment ions using 18 most intense peaks (help)

#	b	b++	b*	b*++	b0	b0++	Seq.	y	y++	y*	y*++	y0	y0++	#
1	72.0444	36.5258					A							20
2	**173.0921**	87.0497			155.0815	78.0444	T	2243.1013	1122.0543	2226.0747	1113.5410	2225.0907	1113.0490	19
3	244.1292	122.5682			226.1186	113.5629	A	2142.0536	1071.5304	2125.0270	1063.0172	2124.0430	1062.5251	18
4	373.1718	187.0895			355.1612	178.0842	E	***2071.0165***	1036.0119	2053.9899	1027.4986	2053.0059	1027.0066	17
5	488.1987	244.6030	471.1722	236.0897	470.1882	235.5977	N	***1941.9739***	971.4906	1924.9473	962.9773	1923.9633	962.4853	16
6	617.2413	309.1243	600.2148	300.6110	599.2307	300.1190	E	***1826.9469***	913.9771	1809.9204	905.4638	1808.9364	904.9718	15
7	764.3097	382.6585	747.2832	374.1452	746.2992	373.6532	F	***1697.9043***	849.4558	1680.8778	840.9425	1679.8938	840.4505	14
8	863.3781	432.1927	846.3516	423.6794	845.3676	423.1874	V	***1550.8359***	775.9216	1533.8094	767.4083	1532.8254	766.9163	13
9	934.4153	467.7113	917.3887	459.1980	916.4047	458.7060	A	***1451.7675***	726.3874	1434.7410	717.8741	1433.7569	717.3821	12
10	1047.4993	524.2533	1030.4728	515.7400	1029.4888	515.2480	L	1380.7304	690.8688	1363.7038	682.3556	1362.7198	681.8636	11
11	1175.5943	588.3008	1158.5677	579.7875	1157.5837	579.2955	K	***1267.6463***	634.3268	1250.6198	625.8135	1249.6358	625.3215	10
12	1303.6892	652.3483	1286.6627	643.8350	1285.6787	643.3430	K	***1139.5514***	570.2793	1122.5248	561.7660	1121.5408	561.2740	9
13	1418.7162	709.8617	1401.6896	701.3485	1400.7056	700.8564	D	***1011.4564***	506.2318	994.4299	497.7186	993.4458	497.2266	8
14	1517.7846	759.3959	1500.7581	750.8827	1499.7740	750.3907	V	***896.4295***	448.7184	879.4029	440.2051	878.4189	439.7131	7
15	1632.8115	816.9094	1615.7850	808.3961	1614.8010	807.9041	D	***797.3611***	399.1842	780.3345	390.6709	779.3505	390.1789	6
16	1792.8422	896.9247	1775.8156	888.4115	1774.8316	887.9195	C	***682.3341***	341.6707	665.3076	333.1574			5
17	1863.8793	932.4433	1846.8528	923.9300	1845.8687	923.4380	A	***522.3035***	261.6554	505.2769	253.1421			4
18	2026.9426	1013.9750	2009.9161	1005.4617	2008.9321	1004.9697	Y	***451.2663***	226.1368	434.2398	217.6235			3
19	2140.0267	1070.5170	2123.0002	1062.0037	2122.0161	1061.5117	L	288.2030	144.6051	271.1765	136.0919			2
20							R	175.1190	88.0631	158.0924	79.5498			1

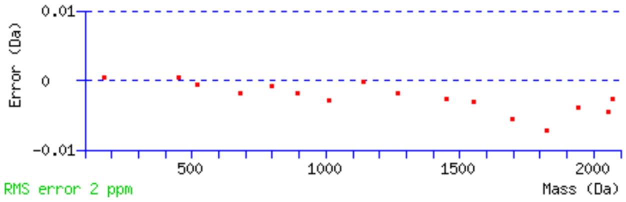

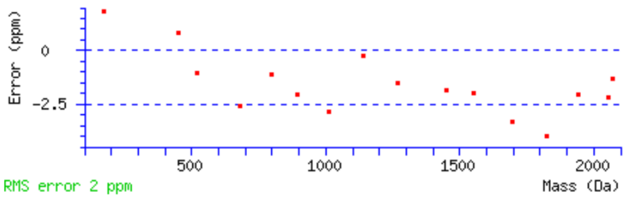

NCBI **BLAST** search of ATAENEFVALKKDVDCAYLR
(Parameters: blastp, nr protein database, expect=20000, no filter, PAM30)
Other BLAST web gateways

All matches to this query

Score	Mr(calc):	Delta	Sequence
141.6	2313.1311	0.0058	ATAENEFVALKKDVDCAYLR
104.9	2312.1470	0.9898	ATAENEFVALKKDVDCAYLR

Mascot: http://www.matrixscience.com/

Mascot Search Results

Peptide View

MS/MS Fragmentation of **CGPCNSFVR**
Found in **sp|Q15323|K1H1_HUMAN**, Keratin, type I cuticular Ha1 OS=Homo sapiens GN=KRT31 PE=2 SV=3

Match to Query 5082: 1096.442348 from(549.228450,2+) intensity(709862.6250) scans(5432) rtinseconds(1082) index(34301)
Title: 160219_Sunil_SDSI_A_Spectrum088886_scans__5432_RTINSECONDS=1082
Data file C:\\Sunil\\TKAP\\T\\T160219_Sunil_SDSI_A.mgf

Click mouse within plot area to zoom in by factor of two about that point
Or, Plot from 50 to 1100 Da Full range
Label all possible matches Label matches used for scoring

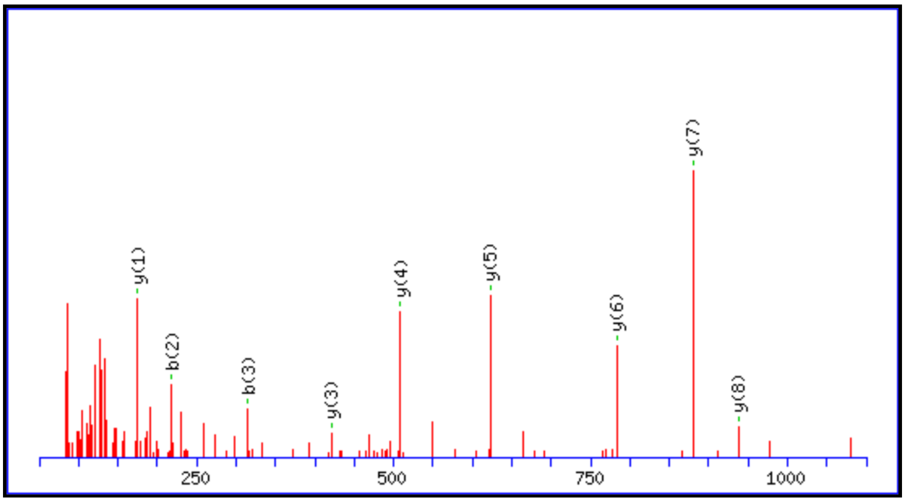

Monoisotopic mass of neutral peptide Mr(calc): 1096.4430
Fixed modifications: Carbamidomethyl (C) (apply to specified residues or termini only)
Variable modifications:
N5 : Deamidated (NQ)
Ions Score: 77 **Expect:** 6.6e-007
Matches : 9/72 fragment ions using 10 most intense peaks (help)

#	b	b++	b*	b*++	b0	b0++	Seq.	y	y++	y*	y*++	y0	y0++	#
1	161.0379	81.0226					C							9
2	**218.0594**	109.5333					G	***937.4196***	469.2135	920.3931	460.7002	919.4091	460.2082	8
3	**315.1122**	158.0597					P	***880.3982***	440.7027	863.3716	432.1894	862.3876	431.6974	7
4	475.1428	238.0750					C	***783.3454***	392.1763	766.3189	383.6631	765.3348	383.1711	6
5	590.1697	295.5885	573.1432	287.0752			N	***623.3148***	312.1610	606.2882	303.6477	605.3042	303.1557	5
6	677.2018	339.1045	660.1752	330.5913	659.1912	330.0992	S	***508.2878***	254.6475	491.2613	246.1343	490.2772	245.6423	4
7	824.2702	412.6387	807.2436	404.1255	806.2596	403.6334	F	***421.2558***	211.1315	404.2292	202.6183			3
8	923.3386	462.1729	906.3121	453.6597	905.3280	453.1677	V	274.1874	137.5973	257.1608	129.0840			2
9							R	***175.1190***	88.0631	158.0924	79.5498			1

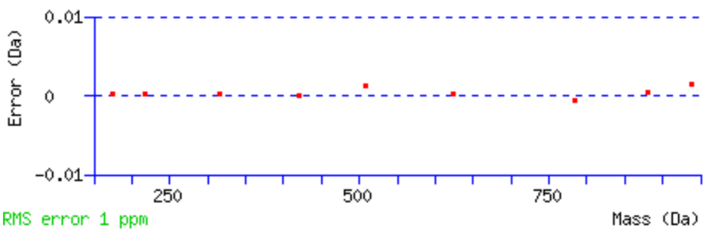

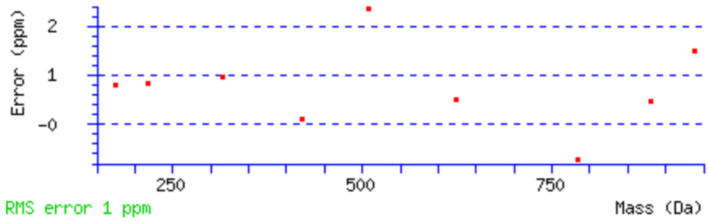

NCBI **BLAST** search of CGPCNSFVR
(Parameters: blastp, nr protein database, expect=20000, no filter, PAM30)
Other BLAST web gateways

All matches to this query

Score	Mr(calc):	Delta	Sequence
77.5	1096.4430	-0.0007	CGPCNSFVR

Mascot: http://www.matrixscience.com/

Mascot Search Results

Peptide View

MS/MS Fragmentation of **CGPCNSFVR**
Found in **sp|Q15323|K1H1_HUMAN**, Keratin, type I cuticular Ha1 OS=Homo sapiens GN=KRT31 PE=2 SV=3

Match to Query 5082: 1096.442348 from(549.228450,2+) intensity(709862.6250) scans(5432) rtinseconds(1082) index(34301)
Title: 160219_Sunil_SDSI_A_Spectrum088886_scans__5432_RTINSECONDS=1082
Data file C:\\Sunil\\TKAP\\T\\T160219_Sunil_SDSI_A.mgf

Click mouse within plot area to zoom in by factor of two about that point
Or, Plot from 50 to 1100 Da Full range
Label all possible matches Label matches used for scoring

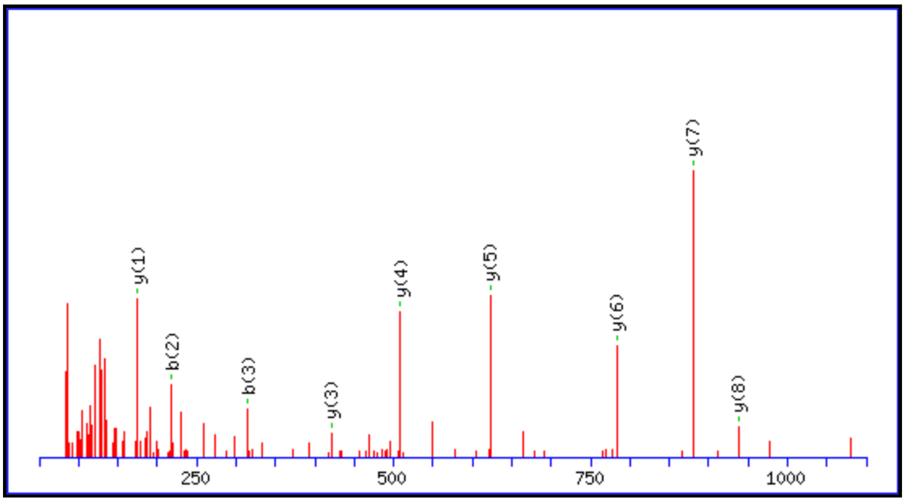

Monoisotopic mass of neutral peptide Mr(calc): 1096.4430
Fixed modifications: Carbamidomethyl (C) (apply to specified residues or termini only)
Variable modifications:
N5 : Deamidated (NQ)
Ions Score: 77 **Expect:** 6.6e-007
Matches : 9/72 fragment ions using 10 most intense peaks (help)

#	b	b++	b*	b*++	b0	b0++	Seq.	y	y++	y*	y*++	y0	y0++	#
1	161.0379	81.0226					C							9
2	**218.0594**	109.5333					G	***937.4196***	469.2135	920.3931	460.7002	919.4091	460.2082	8
3	**315.1122**	158.0597					P	***880.3982***	440.7027	863.3716	432.1894	862.3876	431.6974	7
4	475.1428	238.0750					C	***783.3454***	392.1763	766.3189	383.6631	765.3348	383.1711	6
5	590.1697	295.5885	573.1432	287.0752			N	***623.3148***	312.1610	606.2882	303.6477	605.3042	303.1557	5
6	677.2018	339.1045	660.1752	330.5913	659.1912	330.0992	S	***508.2878***	254.6475	491.2613	246.1343	490.2772	245.6423	4
7	824.2702	412.6387	807.2436	404.1255	806.2596	403.6334	F	***421.2558***	211.1315	404.2292	202.6183			3
8	923.3386	462.1729	906.3121	453.6597	905.3280	453.1677	V	274.1874	137.5973	257.1608	129.0840			2
9							R	***175.1190***	88.0631	158.0924	79.5498			1

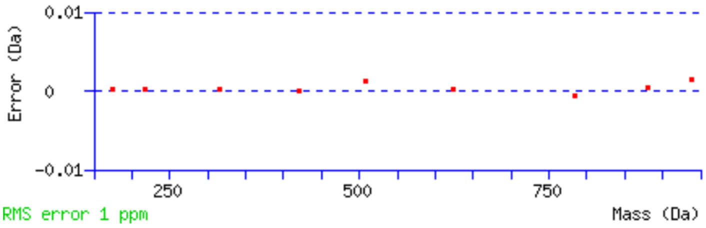

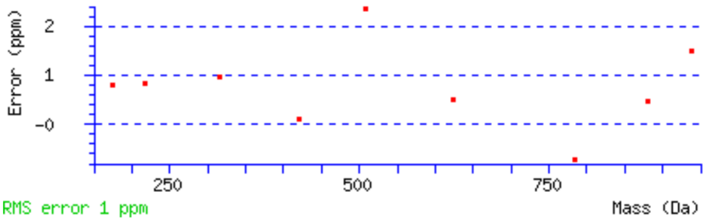

NCBI **BLAST** search of CGPCNSFVR
(Parameters: blastp, nr protein database, expect=20000, no filter, PAM30)
Other BLAST web gateways

All matches to this query

Score	Mr(calc):	Delta	Sequence
77.5	1096.4430	-0.0007	CGPCNSFVR

Mascot: http://www.matrixscience.com/

Mascot Search Results

Peptide View

MS/MS Fragmentation of **CQNSKLEAAVAQSEQQGEAALSDAR**
Found in **sp|O43790|KRT86_HUMAN**, Keratin, type II cuticular Hb6 OS=Homo sapiens GN=KRT86 PE=1 SV=1

Match to Query 38304: 2661.219312 from(888.080380,3+) intensity(1496396.0000) scans(14003) rtinseconds(2536) index(26321)
Title: 160219_Sunil_SDSI_A_Spectrum079627_scans__14003_RTINSECONDS=2536
Data file C:\\Sunil\\TKAP\\T\\T160219_Sunil_SDSI_A.mgf

Click mouse within plot area to zoom in by factor of two about that point
Or, Plot from 0 to 2800 Da Full range
Label all possible matches Label matches used for scoring

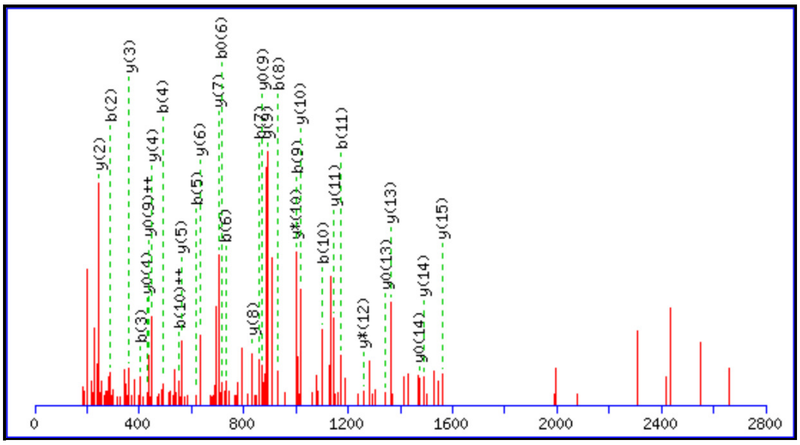

Monoisotopic mass of neutral peptide Mr(calc): 2661.2300
Fixed modifications: Carbamidomethyl (C) (apply to specified residues or termini only)
Variable modifications:
N3 : Deamidated (NQ)
Ions Score: 90 **Expect:** 1.9e-007
Matches : 32/276 fragment ions using 65 most intense peaks (help)

#	b	b^{++}	b*	b^{*++}	b^0	b^{0++}	Seq.	y	y^{++}	y*	y^{*++}	y^0	y^{0++}	#
1	161.0379	81.0226					C							25
2	**289.0965**	145.0519	272.0700	136.5386			Q	2502.2067	1251.6070	2485.1801	1243.0937	2484.1961	1242.6017	24
3	**404.1234**	202.5654	387.0969	194.0521			N	2374.1481	1187.5777	2357.1215	1179.0644	2356.1375	1178.5724	23
4	**491.1555**	246.0814	474.1289	237.5681	473.1449	237.0761	S	2259.1211	1130.0642	2242.0946	1121.5509	2241.1106	1121.0589	22
5	**619.2504**	310.1289	602.2239	301.6156	601.2399	301.1236	K	2172.0891	1086.5482	2155.0626	1078.0349	2154.0786	1077.5429	21
6	**732.3345**	366.6709	715.3080	358.1576	714.3239	357.6656	L	2043.9942	1022.5007	2026.9676	1013.9874	2025.9836	1013.4954	20
7	**861.3771**	431.1922	844.3505	422.6789	843.3665	422.1869	E	1930.9101	965.9587	1913.8835	957.4454	1912.8995	956.9534	19
8	**932.4142**	466.7107	915.3877	458.1975	914.4036	457.7055	A	1801.8675	901.4374	1784.8409	892.9241	1783.8569	892.4321	18
9	**1003.4513**	502.2293	986.4248	493.7160	985.4408	493.2240	A	1730.8304	865.9188	1713.8038	857.4056	1712.8198	856.9135	17
10	**1102.5197**	**551.7635**	1085.4932	543.2502	1084.5092	542.7582	V	1659.7933	830.4003	1642.7667	821.8870	1641.7827	821.3950	16
11	**1173.5569**	587.2821	1156.5303	578.7688	1155.5463	578.2768	A	**1560.7249**	780.8661	1543.6983	772.3528	1542.7143	771.8608	15
12	1301.6154	651.3114	1284.5889	642.7981	1283.6049	642.3061	Q	**1489.6877**	745.3475	1472.6612	736.8342	1471.6772	736.3422	14
13	1388.6475	694.8274	1371.6209	686.3141	1370.6369	685.8221	S	**1361.6292**	681.3182	1344.6026	672.8049	1343.6186	672.3129	13
14	1517.6900	759.3487	1500.6635	750.8354	1499.6795	750.3434	E	1274.5971	637.8022	1257.5706	629.2889	1256.5866	628.7969	12
15	1645.7486	823.3780	1628.7221	814.8647	1627.7381	814.3727	Q	**1145.5545**	573.2809	1128.5280	564.7676	1127.5440	564.2756	11
16	1773.8072	887.4072	1756.7807	878.8940	1755.7966	878.4020	Q	**1017.4960**	509.2516	1000.4694	500.7383	999.4854	500.2463	10
17	1830.8287	915.9180	1813.8021	907.4047	1812.8181	906.9127	G	**889.4374**	445.2223	872.4108	436.7091	871.4268	436.2170	9
18	1959.8713	980.4393	1942.8447	971.9260	1941.8607	971.4340	E	**832.4159**	416.7116	815.3894	408.1983	814.4054	407.7063	8
19	2030.9084	1015.9578	2013.8818	1007.4446	2012.8978	1006.9525	A	**703.3733**	352.1903	686.3468	343.6770	685.3628	343.1850	7
20	2101.9455	1051.4764	2084.9189	1042.9631	2083.9349	1042.4711	A	**632.3362**	316.6717	615.3097	308.1585	614.3257	307.6665	6
21	2215.0296	1108.0184	2198.0030	1099.5051	2197.0190	1099.0131	L	**561.2991**	281.1532	544.2726	272.6399	543.2885	272.1479	5
22	2302.0616	1151.5344	2285.0350	1143.0212	2284.0510	1142.5291	S	**448.2150**	224.6112	431.1885	216.0979	430.2045	215.6059	4
23	2417.0885	1209.0479	2400.0620	1200.5346	2399.0780	1200.0426	D	**361.1830**	181.0951	344.1565	172.5819	343.1724	172.0899	3
24	2488.1256	1244.5665	2471.0991	1236.0532	2470.1151	1235.5612	A	**246.1561**	123.5817	229.1295	115.0684			2
25							R	175.1190	88.0631	158.0924	79.5498			1

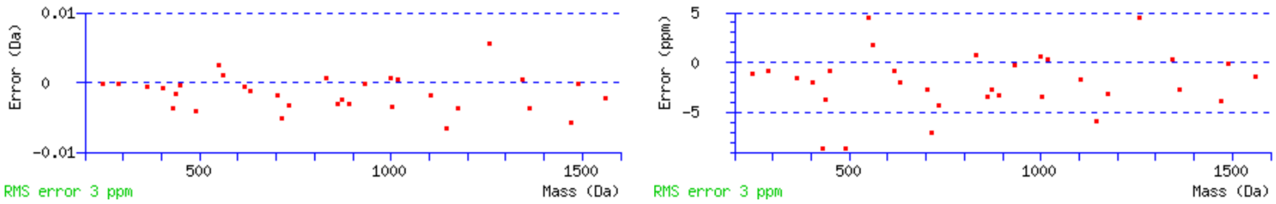

NCBI **BLAST** search of CQNSKLEAAVAQSEQQGEAALSDAR
(Parameters: blastp, nr protein database, expect=20000, no filter, PAM30)
Other BLAST web gateways

All matches to this query

Score	Mr(calc):	Delta	Sequence
90.0	2661.2300	-0.0107	CQNSKLEAAVAQSEQQGEAALSDAR
80.9	2661.2300	-0.0107	CQNSKLEAAVAQSEQQGEAALSDAR
42.7	2661.2300	-0.0107	CQNSKLEAAVAQSEQQGEAALSDAR
24.9	2661.2300	-0.0107	CQNSKLEAAVAQSEQQGEAALSDAR
23.9	2661.2300	-0.0107	CQNSKLEAAVAQSEQQGEAALSDAR

Mascot: http://www.matrixscience.com/

Mascot Search Results

Peptide View

MS/MS Fragmentation of **DNAELENLIR**
Found in **sp|Q15323|K1H1_HUMAN**, Keratin, type I cuticular Ha1 OS=Homo sapiens GN=KRT31 PE=2 SV=3

Match to Query 7898: 1186.583108 from(594.298830,2+) intensity(2134942.7500) scans(14593) rtinseconds(2662) index(11473)
Title: 160219_Sunil_SDSI_A_Spectrum063601_scans__14593_RTINSECONDS=2662
Data file C:\\Sunil\\TKAP\\T\\T160219_Sunil_SDSI_A.mgf

Click mouse within plot area to zoom in by factor of two about that point
Or, Plot from 50 to 1000 Da Full range
Label all possible matches ○ Label matches used for scoring ◉

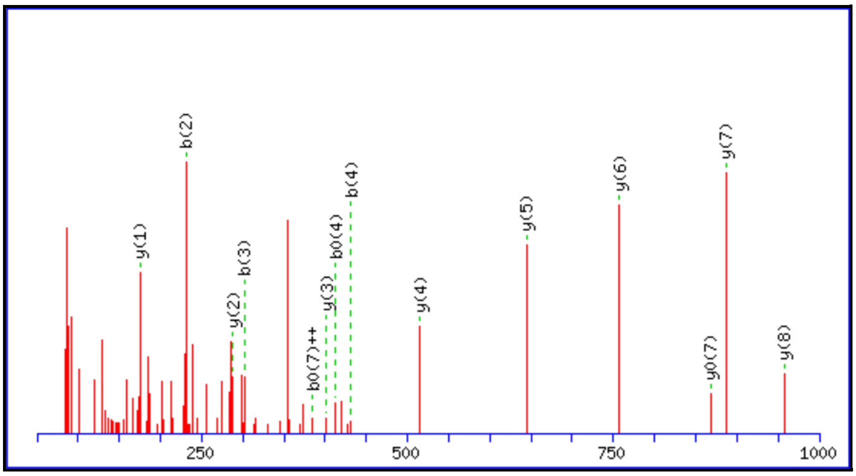

Monoisotopic mass of neutral peptide Mr(calc): 1186.5829
Fixed modifications: Carbamidomethyl (C) (apply to specified residues or termini only)
Variable modifications:
N2 : Deamidated (NQ)
Ions Score: 55 **Expect:** 0.00046
Matches : 14/98 fragment ions using 26 most intense peaks (help)

#	b	b^{++}	b^{*}	b^{*++}	b^{0}	b^{0++}	Seq.	y	y^{++}	y^{*}	y^{*++}	y^{0}	y^{0++}	#
1	116.0342	58.5207			98.0237	49.5155	D							10
2	**231.0612**	116.0342	214.0346	107.5209	213.0506	107.0289	N	1072.5633	536.7853	1055.5368	528.2720	1054.5527	527.7800	9
3	**302.0983**	151.5528	285.0717	143.0395	284.0877	142.5475	A	***957.5364***	479.2718	940.5098	470.7585	939.5258	470.2665	8
4	**431.1409**	216.0741	414.1143	207.5608	413.1303	207.0688	E	***886.4993***	443.7533	869.4727	435.2400	868.4887	434.7480	7
5	544.2249	272.6161	527.1984	264.1028	526.2144	263.6108	L	***757.4567***	379.2320	740.4301	370.7187	739.4461	370.2267	6
6	673.2675	337.1374	656.2410	328.6241	655.2570	328.1321	E	***644.3726***	322.6899	627.3461	314.1767	626.3620	313.6847	5
7	787.3105	394.1589	770.2839	385.6456	769.2999	385.1536	N	***515.3300***	258.1686	498.3035	249.6554			4
8	900.3945	450.7009	883.3680	442.1876	882.3840	441.6956	L	***401.2871***	201.1472	384.2605	192.6339			3
9	1013.4786	507.2429	996.4520	498.7297	995.4680	498.2376	I	***288.2030***	144.6051	271.1765	136.0919			2
10							R	***175.1190***	88.0631	158.0924	79.5498			1

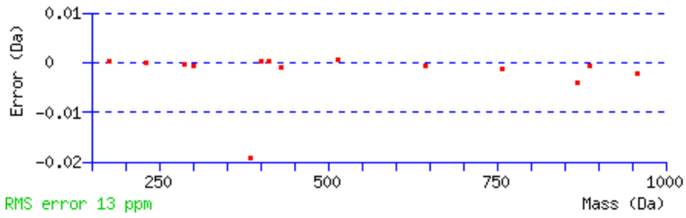

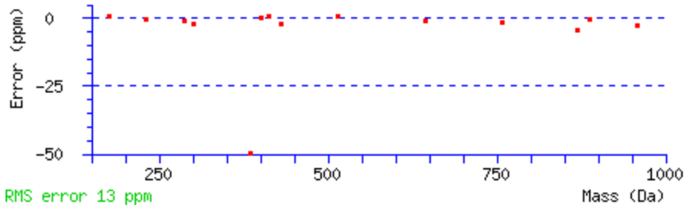

NCBI **BLAST** search of DNAELENLIR
(Parameters: blastp, nr protein database, expect=20000, no filter, PAM30)
Other BLAST web gateways

All matches to this query

Score	Mr(calc):	Delta	Sequence
55.2	1186.5829	0.0002	DNAELENLIR
3.9	1185.5850	0.9981	NNAKEGPGRSR
3.9	1185.5850	0.9981	NNAKEGPGRSR

Mascot: http://www.matrixscience.com/

Mascot Search Results

Peptide View

MS/MS Fragmentation of **DSLENTLTESEAR**
Found in **sp|Q15323|K1H1_HUMAN**, Keratin, type I cuticular Ha1 OS=Homo sapiens GN=KRT31 PE=2 SV=3

Match to Query 16507: 1464.660368 from(733.337460,2+) intensity(755864.5000) scans(8757) rtinseconds(1661) index(6577)
Title: 160219_Sunil_SDSI_A_Spectrum058685_scans__8757_RTINSECONDS=1661
Data file C:\\Sunil\\TKAP\\T\\T160219_Sunil_SDSI_A.mgf

Click mouse within plot area to zoom in by factor of two about that point
Or, Plot from 100 to 1200 Da Full range
Label all possible matches Label matches used for scoring

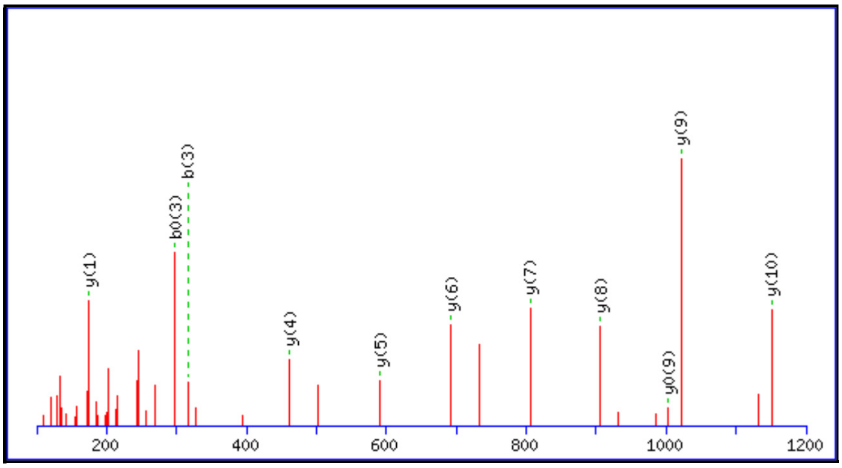

Monoisotopic mass of neutral peptide Mr(calc): 1464.6580
Fixed modifications: Carbamidomethyl (C) (apply to specified residues or termini only)
Variable modifications:
N5 : Deamidated (NQ)
Ions Score: 85 **Expect:** 3.7e-007
Matches : 11/132 fragment ions using 11 most intense peaks (help)

#	b	b^{++}	b^{*}	b^{*++}	b^{0}	b^{0++}	Seq.	y	y^{++}	y^{*}	y^{*++}	y^{0}	y^{0++}	#
1	116.0342	58.5207			98.0237	49.5155	D							13
2	203.0662	102.0368			185.0557	93.0315	S	1350.6383	675.8228	1333.6118	667.3095	1332.6278	666.8175	12
3	**316.1503**	158.5788			298.1397	149.5735	L	1263.6063	632.3068	1246.5798	623.7935	1245.5957	623.3015	11
4	445.1929	223.1001			427.1823	214.0948	E	***1150.5222***	575.7648	1133.4957	567.2515	1132.5117	566.7595	10
5	560.2198	280.6136	543.1933	272.1003	542.2093	271.6083	N	***1021.4796***	511.2435	1004.4531	502.7302	1003.4691	502.2382	9
6	661.2675	331.1374	644.2410	322.6241	643.2570	322.1321	T	***906.4527***	453.7300	889.4262	445.2167	888.4421	444.7247	8
7	774.3516	387.6794	757.3250	379.1662	756.3410	378.6742	L	***805.4050***	403.2061	788.3785	394.6929	787.3945	394.2009	7
8	875.3993	438.2033	858.3727	429.6900	857.3887	429.1980	T	***692.3210***	346.6641	675.2944	338.1508	674.3104	337.6588	6
9	1004.4419	502.7246	987.4153	494.2113	986.4313	493.7193	E	***591.2733***	296.1403	574.2467	287.6270	573.2627	287.1350	5
10	1091.4739	546.2406	1074.4473	537.7273	1073.4633	537.2353	S	***462.2307***	231.6190	445.2041	223.1057	444.2201	222.6137	4
11	1220.5165	610.7619	1203.4899	602.2486	1202.5059	601.7566	E	375.1987	188.1030	358.1721	179.5897	357.1881	179.0977	3
12	1291.5536	646.2804	1274.5270	637.7672	1273.5430	637.2752	A	246.1561	123.5817	229.1295	115.0684			2
13							R	***175.1190***	88.0631	158.0924	79.5498			1

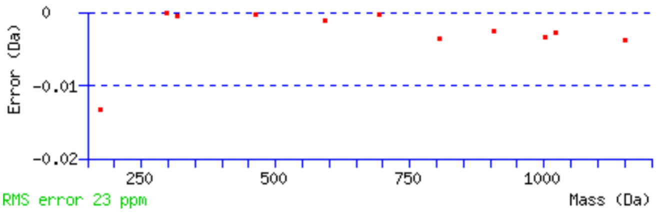

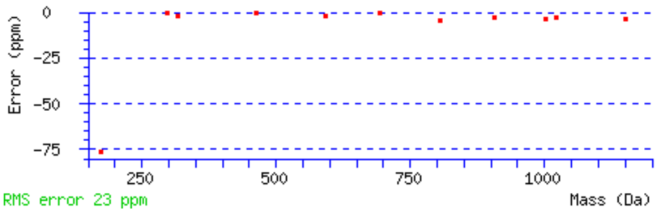

NCBI **BLAST** search of DSLENTLTESEAR
(Parameters: blastp, nr protein database, expect=20000, no filter, PAM30)
Other BLAST web gateways

All matches to this query

Score	Mr(calc):	Delta	Sequence
85.3	1464.6580	0.0024	DSLENTLTESEAR
12.2	1464.6580	0.0024	EDILTNSTEASER
11.9	1464.6701	-0.0097	MIAETPMNQASTR
6.1	1464.6667	-0.0064	MSGSTQPVAQTWR
4.7	1464.6515	0.0089	XSLETGVGDQCRK
4.7	1464.6515	0.0089	XSLETGVGDQCRK
3.5	1464.6732	-0.0128	DESLNIFQNLNR
3.5	1464.6732	-0.0128	DESLNIFQNLNR
3.5	1464.6732	-0.0128	DESLNIFQNLNR
1.2	1464.6514	0.0089	VESSQEANAEVMR

Mascot: http://www.matrixscience.com/

Mascot Search Results

Peptide View

MS/MS Fragmentation of **DSLENTLTESEAR**
Found in **sp|O76009|KT33A_HUMAN**, Keratin, type I cuticular Ha3-I OS=Homo sapiens GN=KRT33A PE=2 SV=2

Match to Query 16507: 1464.660368 from(733.337460,2+) intensity(755864.5000) scans(8757) rtinseconds(1661) index(6577)
Title: 160219_Sunil_SDSI_A_Spectrum058685_scans__8757_RTINSECONDS=1661
Data file C:\\Sunil\\TKAP\\T\\T160219_Sunil_SDSI_A.mgf

Click mouse within plot area to zoom in by factor of two about that point
Or, Plot from 100 to 1200 Da Full range
Label all possible matches Label matches used for scoring

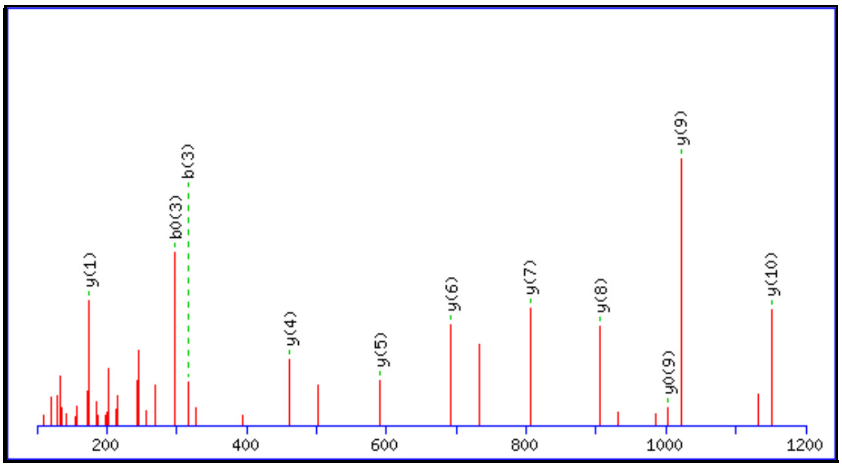

Monoisotopic mass of neutral peptide Mr(calc): 1464.6580
Fixed modifications: Carbamidomethyl (C) (apply to specified residues or termini only)
Variable modifications:
N5 : Deamidated (NQ)
Ions Score: 85 **Expect:** 3.7e-007
Matches : 11/132 fragment ions using 11 most intense peaks (help)

#	b	b++	b*	b*++	b0	b0++	Seq.	y	y++	y*	y*++	y0	y0++	#
1	116.0342	58.5207			98.0237	49.5155	D							13
2	203.0662	102.0368			185.0557	93.0315	S	1350.6383	675.8228	1333.6118	667.3095	1332.6278	666.8175	12
3	**316.1503**	158.5788			298.1397	149.5735	L	1263.6063	632.3068	1246.5798	623.7935	1245.5957	623.3015	11
4	445.1929	223.1001			427.1823	214.0948	E	***1150.5222***	575.7648	1133.4957	567.2515	1132.5117	566.7595	10
5	560.2198	280.6136	543.1933	272.1003	542.2093	271.6083	N	***1021.4796***	511.2435	1004.4531	502.7302	1003.4691	502.2382	9
6	661.2675	331.1374	644.2410	322.6241	643.2570	322.1321	T	***906.4527***	453.7300	889.4262	445.2167	888.4421	444.7247	8
7	774.3516	387.6794	757.3250	379.1662	756.3410	378.6742	L	***805.4050***	403.2061	788.3785	394.6929	787.3945	394.2009	7
8	875.3993	438.2033	858.3727	429.6900	857.3887	429.1980	T	***692.3210***	346.6641	675.2944	338.1508	674.3104	337.6588	6
9	1004.4419	502.7246	987.4153	494.2113	986.4313	493.7193	E	***591.2733***	296.1403	574.2467	287.6270	573.2627	287.1350	5
10	1091.4739	546.2406	1074.4473	537.7273	1073.4633	537.2353	S	***462.2307***	231.6190	445.2041	223.1057	444.2201	222.6137	4
11	1220.5165	610.7619	1203.4899	602.2486	1202.5059	601.7566	E	375.1987	188.1030	358.1721	179.5897	357.1881	179.0977	3
12	1291.5536	646.2804	1274.5270	637.7672	1273.5430	637.2752	A	246.1561	123.5817	229.1295	115.0684			2
13							R	***175.1190***	88.0631	158.0924	79.5498			1

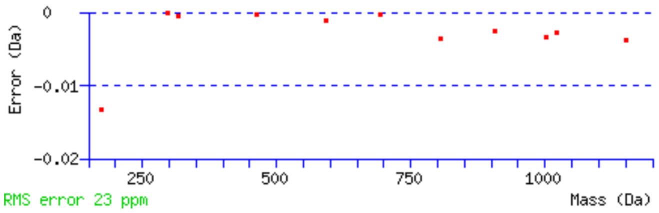

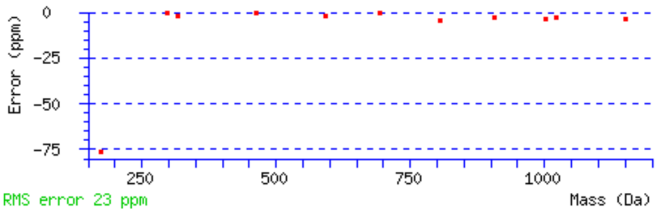

NCBI **BLAST** search of DSLENTLTESEAR
(Parameters: blastp, nr protein database, expect=20000, no filter, PAM30)
Other BLAST web gateways

All matches to this query

Score	Mr(calc):	Delta	Sequence
85.3	1464.6580	0.0024	DSLENTLTESEAR
12.2	1464.6580	0.0024	EDILTNSTEASER
11.9	1464.6701	-0.0097	MIAETPMNQASTR
6.1	1464.6667	-0.0064	MSGSTQPVAQTWR
4.7	1464.6515	0.0089	XSLETGVGDQCRK
4.7	1464.6515	0.0089	XSLETGVGDQCRK
3.5	1464.6732	-0.0128	DESLNIFQNLNR
3.5	1464.6732	-0.0128	DESLNIFQNLNR
3.5	1464.6732	-0.0128	DESLNIFQNLNR
1.2	1464.6514	0.0089	VESSQEANAEVMR

Mascot: http://www.matrixscience.com/

Mascot Search Results

Peptide View

MS/MS Fragmentation of **EAECVEADSGRLASELNHVQEVLEGYK**
Found in **sp|O43790|KRT86_HUMAN**, Keratin, type II cuticular Hb6 OS=Homo sapiens GN=KRT86 PE=1 SV=1

Match to Query 40142: 3032.395902 from(1011.805910,3+) intensity(2680766.5000) scans(14529) rtinseconds(2651) index(11417)
Title: 160219_Sunil_SDSI_A_Spectrum063544_scans__14529_RTINSECONDS=2651
Data file C:\\Sunil\\TKAP\\T\\T160219_Sunil_SDSI_A.mgf

Click mouse within plot area to zoom in by factor of two about that point
Or, Plot from 200 to 3200 Da Full range
Label all possible matches Label matches used for scoring

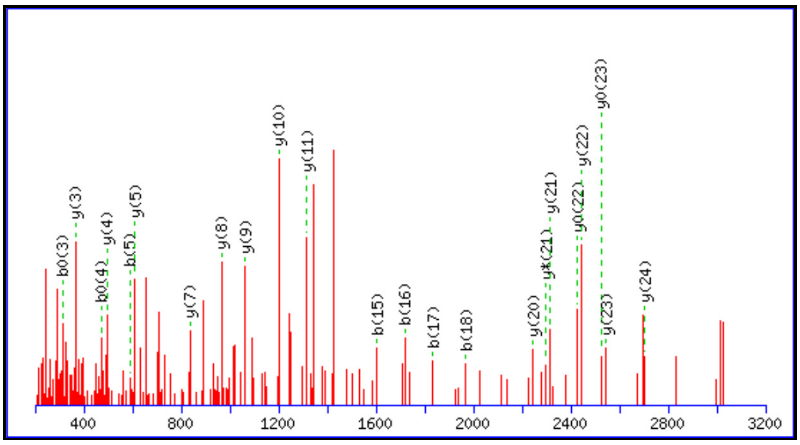

Monoisotopic mass of neutral peptide Mr(calc): 3032.4033
Fixed modifications: Carbamidomethyl (C) (apply to specified residues or termini only)
Variable modifications:
N17 : Deamidated (NQ)
Ions Score: 63 **Expect:** 0.0001
Matches : 23/286 fragment ions using 51 most intense peaks (help)

#	b	b++	b*	b*++	b0	b0++	Seq.	y	y++	y*	y*++	y0	y0++	#
1	130.0499	65.5286			112.0393	56.5233	E							27
2	201.0870	101.0471			183.0764	92.0418	A	2904.3680	1452.6876	2887.3414	1444.1744	2886.3574	1443.6824	26
3	330.1296	165.5684			312.1190	156.5631	E	2833.3309	1417.1691	2816.3043	1408.6558	2815.3203	1408.1638	25
4	490.1602	245.5838			472.1497	236.5785	C	**2704.2883**	1352.6478	2687.2617	1344.1345	2686.2777	1343.6425	24
5	**589.2286**	295.1180			571.2181	286.1127	V	**2544.2576**	1272.6325	2527.2311	1264.1192	2526.2471	1263.6272	23
6	718.2712	359.6393			700.2607	350.6340	E	**2445.1892**	1223.0982	2428.1627	1214.5850	2427.1787	1214.0930	22
7	789.3083	395.1578			771.2978	386.1525	A	**2316.1466**	1158.5770	2299.1201	1150.0637	2298.1361	1149.5717	21
8	904.3353	452.6713			886.3247	443.6660	D	**2245.1095**	1123.0584	2228.0830	1114.5451	2227.0990	1114.0531	20
9	991.3673	496.1873			973.3568	487.1820	S	2130.0826	1065.5449	2113.0560	1057.0317	2112.0720	1056.5396	19
10	1048.3888	524.6980			1030.3782	515.6927	G	2043.0505	1022.0289	2026.0240	1013.5156	2025.0400	1013.0236	18
11	1204.4899	602.7486	1187.4633	594.2353	1186.4793	593.7433	R	1986.0291	993.5182	1969.0025	985.0049	1968.0185	984.5129	17
12	1317.5740	659.2906	1300.5474	650.7773	1299.5634	650.2853	L	1829.9280	915.4676	1812.9014	906.9543	1811.9174	906.4623	16
13	1388.6111	694.8092	1371.5845	686.2959	1370.6005	685.8039	A	1716.8439	858.9256	1699.8174	850.4123	1698.8333	849.9203	15
14	1475.6431	738.3252	1458.6165	729.8119	1457.6325	729.3199	S	1645.8068	823.4070	1628.7802	814.8938	1627.7962	814.4018	14
15	**1604.6857**	802.8465	1587.6591	794.3332	1586.6751	793.8412	E	1558.7748	779.8910	1541.7482	771.3777	1540.7642	770.8857	13
16	**1717.7698**	859.3885	1700.7432	850.8752	1699.7592	850.3832	L	1429.7322	715.3697	1412.7056	706.8564	1411.7216	706.3644	12
17	**1832.7967**	916.9020	1815.7701	908.3887	1814.7861	907.8967	N	**1316.6481**	658.8277	1299.6216	650.3144	1298.6375	649.8224	11
18	**1969.8556**	985.4314	1952.8291	976.9182	1951.8450	976.4262	H	**1201.6212**	601.3142	1184.5946	592.8009	1183.6106	592.3089	10
19	2068.9240	1034.9656	2051.8975	1026.4524	2050.9135	1025.9604	V	**1064.5623**	532.7848	1047.5357	524.2715	1046.5517	523.7795	9
20	2196.9826	1098.9949	2179.9561	1090.4817	2178.9720	1089.9897	Q	**965.4938**	483.2506	948.4673	474.7373	947.4833	474.2453	8
21	2326.0252	1163.5162	2308.9986	1155.0030	2308.0146	1154.5110	E	**837.4353**	419.2213	820.4087	410.7080	819.4247	410.2160	7
22	2425.0936	1213.0504	2408.0671	1204.5372	2407.0830	1204.0452	V	708.3927	354.7000	691.3661	346.1867	690.3821	345.6947	6
23	2538.1777	1269.5925	2521.1511	1261.0792	2520.1671	1260.5872	L	**609.3243**	305.1658	592.2977	296.6525	591.3137	296.1605	5
24	2667.2203	1334.1138	2650.1937	1325.6005	2649.2097	1325.1085	E	**496.2402**	248.6237	479.2136	240.1105	478.2296	239.6185	4
25	2724.2417	1362.6245	2707.2152	1354.1112	2706.2312	1353.6192	G	**367.1976**	184.1024	350.1710	175.5892			3
26	2887.3051	1444.1562	2870.2785	1435.6429	2869.2945	1435.1509	Y	310.1761	155.5917	293.1496	147.0784			2
27							K	147.1128	74.0600	130.0863	65.5468			1

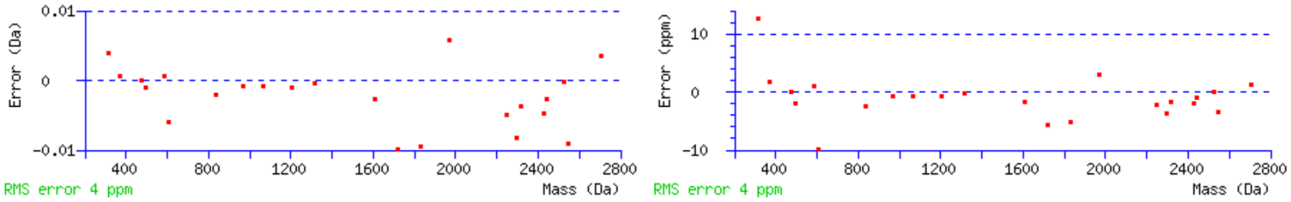

NCBI **BLAST** search of EAECVEADSGRLASELNHVQEVLEGYK
(Parameters: blastp, nr protein database, expect=20000, no filter, PAM30)
Other BLAST web gateways

All matches to this query

Score	Mr(calc):	Delta	Sequence
63.3	3032.4033	-0.0074	EAECVEADSGRLASELNHVQEVLEGYK
31.6	3032.4033	-0.0074	EAECVEADSGRLASELNHVQEVLEGYK
12.7	3031.4192	0.9767	EAECVEADSGRLASELNHVQEVLEGYK

Mascot: http://www.matrixscience.com/

Mascot Search Results

Peptide View

MS/MS Fragmentation of **EAECVEADSGRLASELNHVQEVLEGYK**
Found in **sp|P78385|KRT83_HUMAN**, Keratin, type II cuticular Hb3 OS=Homo sapiens GN=KRT83 PE=1 SV=2

Match to Query 40144: 3032.404976 from(759.108520,4+) intensity(2666729.5000) scans(14507) rtinseconds(2647) index(11397)
Title: 160219_Sunil_SDSI_A_Spectrum063524_scans__14507_RTINSECONDS=2647
Data file C:\\Sunil\\TKAP\\T\\T160219_Sunil_SDSI_A.mgf

Click mouse within plot area to zoom in by factor of two about that point
Or, Plot from 200 to 3000 Da Full range
Label all possible matches Label matches used for scoring

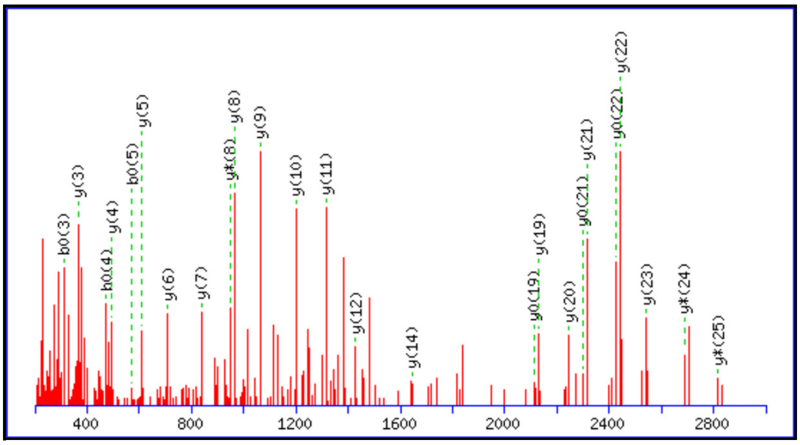

Monoisotopic mass of neutral peptide Mr(calc): 3032.4033
Fixed modifications: Carbamidomethyl (C) (apply to specified residues or termini only)
Variable modifications:
N17 : Deamidated (NQ)
Ions Score: 85 **Expect:** 7.6e-007
Matches : 25/286 fragment ions using 51 most intense peaks (help)

#	b	b^{++}	b*	b^{*++}	b^{0}	b^{0++}	Seq.	y	y^{++}	y*	y^{*++}	y^{0}	y^{0++}	#
1	130.0499	65.5286			112.0393	56.5233	E							27
2	201.0870	101.0471			183.0764	92.0418	A	2904.3680	1452.6876	2887.3414	1444.1744	2886.3574	1443.6824	26
3	330.1296	165.5684			312.1190	156.5631	E	2833.3309	1417.1691	2816.3043	1408.6558	2815.3203	1408.1638	25
4	490.1602	245.5838			472.1497	236.5785	C	2704.2883	1352.6478	2687.2617	1344.1345	2686.2777	1343.6425	24
5	589.2286	295.1180			571.2181	286.1127	V	2544.2576	1272.6325	2527.2311	1264.1192	2526.2471	1263.6272	23
6	718.2712	359.6393			700.2607	350.6340	E	2445.1892	1223.0982	2428.1627	1214.5850	2427.1787	1214.0930	22
7	789.3083	395.1578			771.2978	386.1525	A	2316.1466	1158.5770	2299.1201	1150.0637	2298.1361	1149.5717	21
8	904.3353	452.6713			886.3247	443.6660	D	2245.1095	1123.0584	2228.0830	1114.5451	2227.0990	1114.0531	20
9	991.3673	496.1873			973.3568	487.1820	S	2130.0826	1065.5449	2113.0560	1057.0317	2112.0720	1056.5396	19
10	1048.3888	524.6980			1030.3782	515.6927	G	2043.0505	1022.0289	2026.0240	1013.5156	2025.0400	1013.0236	18
11	1204.4899	602.7486	1187.4633	594.2353	1186.4793	593.7433	R	1986.0291	993.5182	1969.0025	985.0049	1968.0185	984.5129	17
12	1317.5740	659.2906	1300.5474	650.7773	1299.5634	650.2853	L	1829.9280	915.4676	1812.9014	906.9543	1811.9174	906.4623	16
13	1388.6111	694.8092	1371.5845	686.2959	1370.6005	685.8039	A	1716.8439	858.9256	1699.8174	850.4123	1698.8333	849.9203	15
14	1475.6431	738.3252	1458.6165	729.8119	1457.6325	729.3199	S	1645.8068	823.4070	1628.7802	814.8938	1627.7962	814.4018	14
15	1604.6857	802.8465	1587.6591	794.3332	1586.6751	793.8412	E	1558.7748	779.8910	1541.7482	771.3777	1540.7642	770.8857	13
16	1717.7698	859.3885	1700.7432	850.8752	1699.7592	850.3832	L	1429.7322	715.3697	1412.7056	706.8564	1411.7216	706.3644	12
17	1832.7967	916.9020	1815.7701	908.3887	1814.7861	907.8967	N	1316.6481	658.8277	1299.6216	650.3144	1298.6375	649.8224	11
18	1969.8556	985.4314	1952.8291	976.9182	1951.8450	976.4262	H	1201.6212	601.3142	1184.5946	592.8009	1183.6106	592.3089	10
19	2068.9240	1034.9656	2051.8975	1026.4524	2050.9135	1025.9604	V	1064.5623	532.7848	1047.5357	524.2715	1046.5517	523.7795	9
20	2196.9826	1098.9949	2179.9561	1090.4817	2178.9720	1089.9897	Q	965.4938	483.2506	948.4673	474.7373	947.4833	474.2453	8
21	2326.0252	1163.5162	2308.9986	1155.0030	2308.0146	1154.5110	E	837.4353	419.2213	820.4087	410.7080	819.4247	410.2160	7
22	2425.0936	1213.0504	2408.0671	1204.5372	2407.0830	1204.0452	V	708.3927	354.7000	691.3661	346.1867	690.3821	345.6947	6
23	2538.1777	1269.5925	2521.1511	1261.0792	2520.1671	1260.5872	L	609.3243	305.1658	592.2977	296.6525	591.3137	296.1605	5
24	2667.2203	1334.1138	2650.1937	1325.6005	2649.2097	1325.1085	E	496.2402	248.6237	479.2136	240.1105	478.2296	239.6185	4
25	2724.2417	1362.6245	2707.2152	1354.1112	2706.2312	1353.6192	G	367.1976	184.1024	350.1710	175.5892			3
26	2887.3051	1444.1562	2870.2785	1435.6429	2869.2945	1435.1509	Y	310.1761	155.5917	293.1496	147.0784			2
27							K	147.1128	74.0600	130.0863	65.5468			1

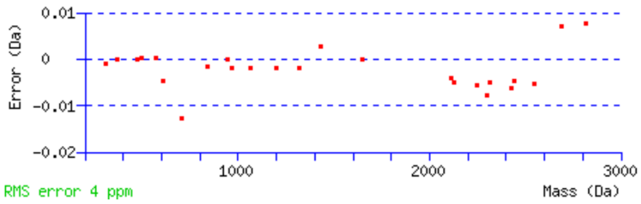

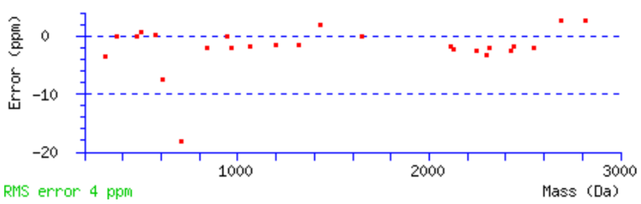

NCBI **BLAST** search of EAECVEADSGRLASELNHVQEVLEGYK
(Parameters: blastp, nr protein database, expect=20000, no filter, PAM30)
Other BLAST web gateways

All matches to this query

Score	Mr(calc):	Delta	Sequence
85.0	3032.4033	0.0017	EAECVEADSGRLASELNHVQEVLEGYK
52.2	3032.4033	0.0017	EAECVEADSGRLASELNHVQEVLEGYK
15.7	3031.4192	0.9857	EAECVEADSGRLASELNHVQEVLEGYK

Mascot: http://www.matrixscience.com/

Mascot Search Results

Peptide View

MS/MS Fragmentation of **EAECVEADSGRLASELNHVQEVLEGYKK**
Found in **sp|P78385|KRT83_HUMAN**, Keratin, type II cuticular Hb3 OS=Homo sapiens GN=KRT83 PE=1 SV=2

Match to Query 40813: 3160.501020 from(633.107480,5+) intensity(477941.4063) scans(12966) rtinseconds(2384) index(40799)
Title: 160219_Sunil_SDSI_A_Spectrum095384_scans__12966_RTINSECONDS=2384
Data file C:\\Sunil\\TKAP\\T\\T160219_Sunil_SDSI_A.mgf

Click mouse within plot area to zoom in by factor of two about that point
Or, Plot from 200 to 3000 Da Full range
Label all possible matches Label matches used for scoring

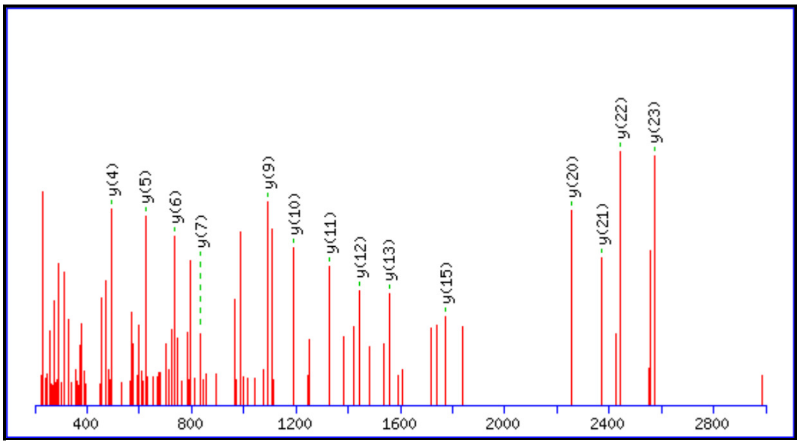

Monoisotopic mass of neutral peptide Mr(calc): 3160.4982
Fixed modifications: Carbamidomethyl (C) (apply to specified residues or termini only)
Variable modifications:
N17 : Deamidated (NQ)
Ions Score: 100 **Expect:** 2.4e-008
Matches : 14/296 fragment ions using 22 most intense peaks (help)

#	b	b++	b*	b*++	b0	b0++	Seq.	y	y++	y*	y*++	y0	y0++	#
1	130.0499	65.5286			112.0393	56.5233	E							28
2	201.0870	101.0471			183.0764	92.0418	A	3032.4630	1516.7351	3015.4364	1508.2218	3014.4524	1507.7298	27
3	330.1296	165.5684			312.1190	156.5631	E	2961.4258	1481.2166	2944.3993	1472.7033	2943.4153	1472.2113	26
4	490.1602	245.5838			472.1497	236.5785	C	2832.3832	1416.6953	2815.3567	1408.1820	2814.3727	1407.6900	25
5	589.2286	295.1180			571.2181	286.1127	V	2672.3526	1336.6799	2655.3261	1328.1667	2654.3420	1327.6747	24
6	718.2712	359.6393			700.2607	350.6340	E	***2573.2842***	1287.1457	2556.2576	1278.6325	2555.2736	1278.1404	23
7	789.3083	395.1578			771.2978	386.1525	A	***2444.2416***	1222.6244	2427.2150	1214.1112	2426.2310	1213.6192	22
8	904.3353	452.6713			886.3247	443.6660	D	***2373.2045***	1187.1059	2356.1779	1178.5926	2355.1939	1178.1006	21
9	991.3673	496.1873			973.3568	487.1820	S	***2258.1775***	1129.5924	2241.1510	1121.0791	2240.1670	1120.5871	20
10	1048.3888	524.6980			1030.3782	515.6927	G	2171.1455	1086.0764	2154.1190	1077.5631	2153.1349	1077.0711	19
11	1204.4899	602.7486	1187.4633	594.2353	1186.4793	593.7433	R	2114.1240	1057.5657	2097.0975	1049.0524	2096.1135	1048.5604	18
12	1317.5740	659.2906	1300.5474	650.7773	1299.5634	650.2853	L	1958.0229	979.5151	1940.9964	971.0018	1940.0124	970.5098	17
13	1388.6111	694.8092	1371.5845	686.2959	1370.6005	685.8039	A	1844.9389	922.9731	1827.9123	914.4598	1826.9283	913.9678	16
14	1475.6431	738.3252	1458.6165	729.8119	1457.6325	729.3199	S	***1773.9018***	887.4545	1756.8752	878.9412	1755.8912	878.4492	15
15	1604.6857	802.8465	1587.6591	794.3332	1586.6751	793.8412	E	1686.8697	843.9385	1669.8432	835.4252	1668.8592	834.9332	14
16	1717.7698	859.3885	1700.7432	850.8752	1699.7592	850.3832	L	***1557.8271***	779.4172	1540.8006	770.9039	1539.8166	770.4119	13
17	1832.7967	916.9020	1815.7701	908.3887	1814.7861	907.8967	N	***1444.7431***	722.8752	1427.7165	714.3619	1426.7325	713.8699	12
18	1969.8556	985.4314	1952.8291	976.9182	1951.8450	976.4262	H	***1329.7161***	665.3617	1312.6896	656.8484	1311.7056	656.3564	11
19	2068.9240	1034.9656	2051.8975	1026.4524	2050.9135	1025.9604	V	***1192.6572***	596.8322	1175.6307	588.3190	1174.6467	587.8270	10
20	2196.9826	1098.9949	2179.9561	1090.4817	2178.9720	1089.9897	Q	***1093.5888***	547.2980	1076.5623	538.7848	1075.5782	538.2928	9
21	2326.0252	1163.5162	2308.9986	1155.0030	2308.0146	1154.5110	E	965.5302	483.2688	948.5037	474.7555	947.5197	474.2635	8
22	2425.0936	1213.0504	2408.0671	1204.5372	2407.0830	1204.0452	V	***836.4876***	418.7475	819.4611	410.2342	818.4771	409.7422	7
23	2538.1777	1269.5925	2521.1511	1261.0792	2520.1671	1260.5872	L	***737.4192***	369.2132	720.3927	360.7000	719.4087	360.2080	6
24	2667.2203	1334.1138	2650.1937	1325.6005	2649.2097	1325.1085	E	***624.3352***	312.6712	607.3086	304.1579	606.3246	303.6659	5
25	2724.2417	1362.6245	2707.2152	1354.1112	2706.2312	1353.6192	G	***495.2926***	248.1499	478.2660	239.6366			4
26	2887.3051	1444.1562	2870.2785	1435.6429	2869.2945	1435.1509	Y	438.2711	219.6392	421.2445	211.1259			3
27	3015.4000	1508.2036	2998.3735	1499.6904	2997.3895	1499.1984	K	275.2078	138.1075	258.1812	129.5942			2

**28**							**K**	147.1128	74.0600	130.0863	65.5468			**1**

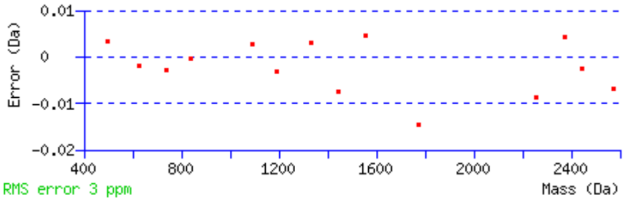

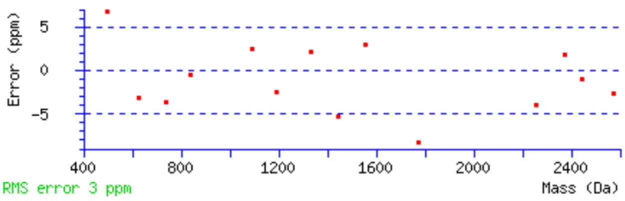

NCBI **BLAST** search of EAECVEADSGRLASELNHVQEVLEGYKK
(Parameters: blastp, nr protein database, expect=20000, no filter, PAM30)
Other BLAST web gateways

All matches to this query

Score	Mr(calc):	Delta	Sequence
100.2	3160.4982	0.0028	EAECVEADSGRLASELNHVQEVLEGYKK
59.1	3160.4982	0.0028	EAECVEADSGRLASELNHVQEVLEGYKK
18.6	3159.5142	0.9868	EAECVEADSGRLASELNHVQEVLEGYKK

Mascot: http://www.matrixscience.com/

Mascot Search Results

Peptide View

MS/MS Fragmentation of **EAECVEADSGRLASELNHVQEVLEGYKK**
Found in **sp|O43790|KRT86_HUMAN**, Keratin, type II cuticular Hb6 OS=Homo sapiens GN=KRT86 PE=1 SV=1

Match to Query 40808: 3160.488792 from(1054.503540,3+) intensity(682206.7500) scans(13582) rtinseconds(2488) index(10651)
Title: 160219_Sunil_SDSI_A_Spectrum062774_scans__13582_RTINSECONDS=2488
Data file C:\\Sunil\\TKAP\\T\\T160219_Sunil_SDSI_A.mgf

Click mouse within plot area to zoom in by factor of two about that point
Or, Plot from 200 to 3200 Da Full range
Label all possible matches Label matches used for scoring

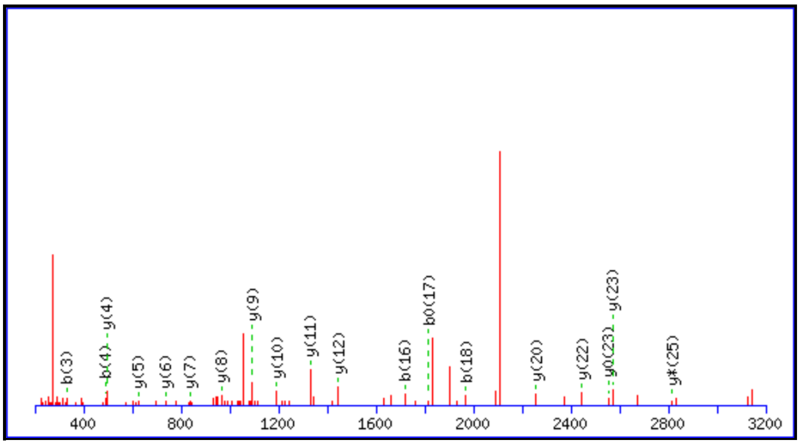

Monoisotopic mass of neutral peptide Mr(calc): 3160.4982
Fixed modifications: Carbamidomethyl (C) (apply to specified residues or termini only)
Variable modifications:
N17 : Deamidated (NQ)
Ions Score: 52 **Expect:** 0.0014
Matches : 19/296 fragment ions using 45 most intense peaks (help)

#	b	b++	b*	b*++	b0	b0++	Seq.	y	y++	y*	y*++	y0	y0++	#
1	130.0499	65.5286			112.0393	56.5233	E							28
2	201.0870	101.0471			183.0764	92.0418	A	3032.4630	1516.7351	3015.4364	1508.2218	3014.4524	1507.7298	27
3	**330.1296**	165.5684			312.1190	156.5631	E	2961.4258	1481.2166	2944.3993	1472.7033	2943.4153	1472.2113	26
4	**490.1602**	245.5838			472.1497	236.5785	C	2832.3832	1416.6953	2815.3567	1408.1820	2814.3727	1407.6900	25
5	589.2286	295.1180			571.2181	286.1127	V	2672.3526	1336.6799	2655.3261	1328.1667	2654.3420	1327.6747	24
6	718.2712	359.6393			700.2607	350.6340	E	***2573.2842***	1287.1457	2556.2576	1278.6325	2555.2736	1278.1404	23
7	789.3083	395.1578			771.2978	386.1525	A	***2444.2416***	1222.6244	2427.2150	1214.1112	2426.2310	1213.6192	22
8	904.3353	452.6713			886.3247	443.6660	D	2373.2045	1187.1059	2356.1779	1178.5926	2355.1939	1178.1006	21
9	991.3673	496.1873			973.3568	487.1820	S	***2258.1775***	1129.5924	2241.1510	1121.0791	2240.1670	1120.5871	20
10	1048.3888	524.6980			1030.3782	515.6927	G	2171.1455	1086.0764	2154.1190	1077.5631	2153.1349	1077.0711	19
11	1204.4899	602.7486	1187.4633	594.2353	1186.4793	593.7433	R	2114.1240	1057.5657	2097.0975	1049.0524	2096.1135	1048.5604	18
12	1317.5740	659.2906	1300.5474	650.7773	1299.5634	650.2853	L	1958.0229	979.5151	1940.9964	971.0018	1940.0124	970.5098	17
13	1388.6111	694.8092	1371.5845	686.2959	1370.6005	685.8039	A	1844.9389	922.9731	1827.9123	914.4598	1826.9283	913.9678	16
14	1475.6431	738.3252	1458.6165	729.8119	1457.6325	729.3199	S	1773.9018	887.4545	1756.8752	878.9412	1755.8912	878.4492	15
15	1604.6857	802.8465	1587.6591	794.3332	1586.6751	793.8412	E	1686.8697	843.9385	1669.8432	835.4252	1668.8592	834.9332	14
16	**1717.7698**	859.3885	1700.7432	850.8752	1699.7592	850.3832	L	1557.8271	779.4172	1540.8006	770.9039	1539.8166	770.4119	13
17	1832.7967	916.9020	1815.7701	908.3887	1814.7861	907.8967	N	***1444.7431***	722.8752	1427.7165	714.3619	1426.7325	713.8699	12
18	**1969.8556**	985.4314	1952.8291	976.9182	1951.8450	976.4262	H	***1329.7161***	665.3617	1312.6896	656.8484	1311.7056	656.3564	11
19	2068.9240	1034.9656	2051.8975	1026.4524	2050.9135	1025.9604	V	***1192.6572***	596.8322	1175.6307	588.3190	1174.6467	587.8270	10
20	2196.9826	1098.9949	2179.9561	1090.4817	2178.9720	1089.9897	Q	***1093.5888***	547.2980	1076.5623	538.7848	1075.5782	538.2928	9
21	2326.0252	1163.5162	2308.9986	1155.0030	2308.0146	1154.5110	E	***965.5302***	483.2688	948.5037	474.7555	947.5197	474.2635	8
22	2425.0936	1213.0504	2408.0671	1204.5372	2407.0830	1204.0452	V	***836.4876***	418.7475	819.4611	410.2342	818.4771	409.7422	7
23	2538.1777	1269.5925	2521.1511	1261.0792	2520.1671	1260.5872	L	***737.4192***	369.2132	720.3927	360.7000	719.4087	360.2080	6
24	2667.2203	1334.1138	2650.1937	1325.6005	2649.2097	1325.1085	E	***624.3352***	312.6712	607.3086	304.1579	606.3246	303.6659	5
25	2724.2417	1362.6245	2707.2152	1354.1112	2706.2312	1353.6192	G	***495.2926***	248.1499	478.2660	239.6366			4
26	2887.3051	1444.1562	2870.2785	1435.6429	2869.2945	1435.1509	Y	438.2711	219.6392	421.2445	211.1259			3
27	3015.4000	1508.2036	2998.3735	1499.6904	2997.3895	1499.1984	K	275.2078	138.1075	258.1812	129.5942			2

**28**							**K**	147.1128	74.0600	130.0863	65.5468			**1**

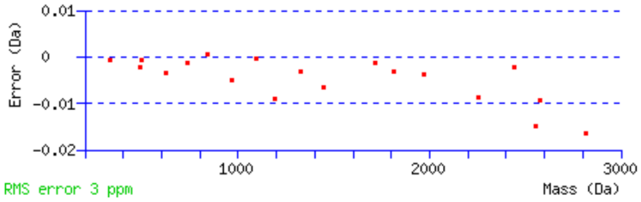

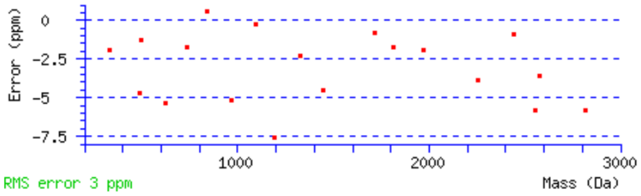

NCBI **BLAST** search of EAECVEADSGRLASELNHVQEVLEGYKK
(Parameters: blastp, nr protein database, expect=20000, no filter, PAM30)
Other BLAST web gateways

All matches to this query

Score	Mr(calc):	Delta	Sequence
52.3	3160.4982	-0.0094	EAECVEADSGRLASELNHVQEVLEGYKK
27.1	3160.4982	-0.0094	EAECVEADSGRLASELNHVQEVLEGYKK
17.4	3159.5142	0.9746	EAECVEADSGRLASELNHVQEVLEGYKK

Mascot: http://www.matrixscience.com/

Mascot Search Results

Peptide View

MS/MS Fragmentation of **EAECVEADSGRLASELNHVQEVLEGYKK**
Found in **sp|O43790|KRT86_HUMAN**, Keratin, type II cuticular Hb6 OS=Homo sapiens GN=KRT86 PE=1 SV=1

Match to Query 40813: 3160.501020 from(633.107480,5+) intensity(477941.4063) scans(12966) rtinseconds(2384) index(40799)
Title: 160219_Sunil_SDSI_A_Spectrum095384_scans__12966_RTINSECONDS=2384
Data file C:\\Sunil\\TKAP\\T\\T160219_Sunil_SDSI_A.mgf

Click mouse within plot area to zoom in by factor of two about that point
Or, Plot from 200 to 3000 Da Full range
Label all possible matches ○ Label matches used for scoring ◉

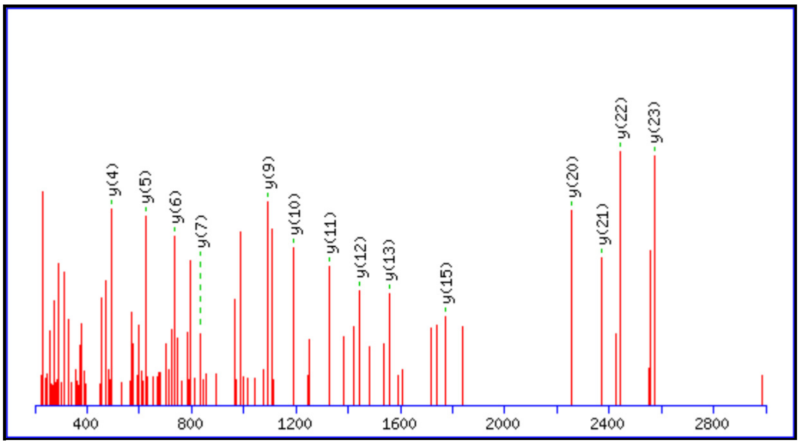

Monoisotopic mass of neutral peptide Mr(calc): 3160.4982
Fixed modifications: Carbamidomethyl (C) (apply to specified residues or termini only)
Variable modifications:
N17 : Deamidated (NQ)
Ions Score: 100 **Expect:** 2.4e-008
Matches : 14/296 fragment ions using 22 most intense peaks (help)

#	b	b^{++}	b*	b^{*++}	b^0	b^{0++}	Seq.	y	y^{++}	y*	y^{*++}	y^0	y^{0++}	#
1	130.0499	65.5286			112.0393	56.5233	E							28
2	201.0870	101.0471			183.0764	92.0418	A	3032.4630	1516.7351	3015.4364	1508.2218	3014.4524	1507.7298	27
3	330.1296	165.5684			312.1190	156.5631	E	2961.4258	1481.2166	2944.3993	1472.7033	2943.4153	1472.2113	26
4	490.1602	245.5838			472.1497	236.5785	C	2832.3832	1416.6953	2815.3567	1408.1820	2814.3727	1407.6900	25
5	589.2286	295.1180			571.2181	286.1127	V	2672.3526	1336.6799	2655.3261	1328.1667	2654.3420	1327.6747	24
6	718.2712	359.6393			700.2607	350.6340	E	***2573.2842***	1287.1457	2556.2576	1278.6325	2555.2736	1278.1404	23
7	789.3083	395.1578			771.2978	386.1525	A	***2444.2416***	1222.6244	2427.2150	1214.1112	2426.2310	1213.6192	22
8	904.3353	452.6713			886.3247	443.6660	D	***2373.2045***	1187.1059	2356.1779	1178.5926	2355.1939	1178.1006	21
9	991.3673	496.1873			973.3568	487.1820	S	***2258.1775***	1129.5924	2241.1510	1121.0791	2240.1670	1120.5871	20
10	1048.3888	524.6980			1030.3782	515.6927	G	2171.1455	1086.0764	2154.1190	1077.5631	2153.1349	1077.0711	19
11	1204.4899	602.7486	1187.4633	594.2353	1186.4793	593.7433	R	2114.1240	1057.5657	2097.0975	1049.0524	2096.1135	1048.5604	18
12	1317.5740	659.2906	1300.5474	650.7773	1299.5634	650.2853	L	1958.0229	979.5151	1940.9964	971.0018	1940.0124	970.5098	17
13	1388.6111	694.8092	1371.5845	686.2959	1370.6005	685.8039	A	1844.9389	922.9731	1827.9123	914.4598	1826.9283	913.9678	16
14	1475.6431	738.3252	1458.6165	729.8119	1457.6325	729.3199	S	***1773.9018***	887.4545	1756.8752	878.9412	1755.8912	878.4492	15
15	1604.6857	802.8465	1587.6591	794.3332	1586.6751	793.8412	E	1686.8697	843.9385	1669.8432	835.4252	1668.8592	834.9332	14
16	1717.7698	859.3885	1700.7432	850.8752	1699.7592	850.3832	L	***1557.8271***	779.4172	1540.8006	770.9039	1539.8166	770.4119	13
17	1832.7967	916.9020	1815.7701	908.3887	1814.7861	907.8967	N	***1444.7431***	722.8752	1427.7165	714.3619	1426.7325	713.8699	12
18	1969.8556	985.4314	1952.8291	976.9182	1951.8450	976.4262	H	***1329.7161***	665.3617	1312.6896	656.8484	1311.7056	656.3564	11
19	2068.9240	1034.9656	2051.8975	1026.4524	2050.9135	1025.9604	V	***1192.6572***	596.8322	1175.6307	588.3190	1174.6467	587.8270	10
20	2196.9826	1098.9949	2179.9561	1090.4817	2178.9720	1089.9897	Q	***1093.5888***	547.2980	1076.5623	538.7848	1075.5782	538.2928	9
21	2326.0252	1163.5162	2308.9986	1155.0030	2308.0146	1154.5110	E	965.5302	483.2688	948.5037	474.7555	947.5197	474.2635	8
22	2425.0936	1213.0504	2408.0671	1204.5372	2407.0830	1204.0452	V	***836.4876***	418.7475	819.4611	410.2342	818.4771	409.7422	7
23	2538.1777	1269.5925	2521.1511	1261.0792	2520.1671	1260.5872	L	***737.4192***	369.2132	720.3927	360.7000	719.4087	360.2080	6
24	2667.2203	1334.1138	2650.1937	1325.6005	2649.2097	1325.1085	E	***624.3352***	312.6712	607.3086	304.1579	606.3246	303.6659	5
25	2724.2417	1362.6245	2707.2152	1354.1112	2706.2312	1353.6192	G	***495.2926***	248.1499	478.2660	239.6366			4
26	2887.3051	1444.1562	2870.2785	1435.6429	2869.2945	1435.1509	Y	438.2711	219.6392	421.2445	211.1259			3
27	3015.4000	1508.2036	2998.3735	1499.6904	2997.3895	1499.1984	K	275.2078	138.1075	258.1812	129.5942			2

**28**							**K**	147.1128	74.0600	130.0863	65.5468			**1**

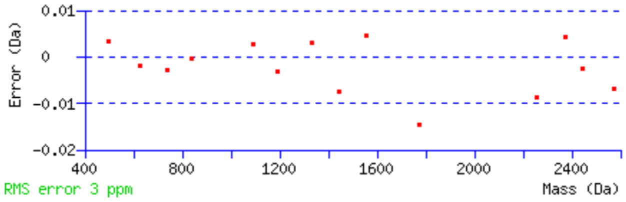

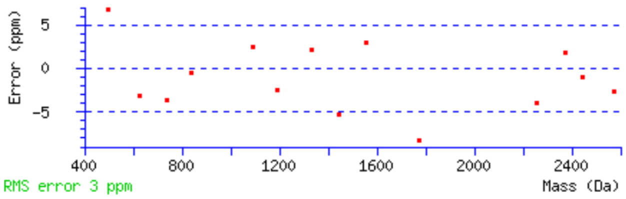

NCBI **BLAST** search of EAECVEADSGRLASELNHVQEVLEGYKK
(Parameters: blastp, nr protein database, expect=20000, no filter, PAM30)
Other BLAST web gateways

All matches to this query

Score	Mr(calc):	Delta	Sequence
100.2	3160.4982	0.0028	EAECVEADSGRLASELNHVQEVLEGYKK
59.1	3160.4982	0.0028	EAECVEADSGRLASELNHVQEVLEGYKK
18.6	3159.5142	0.9868	EAECVEADSGRLASELNHVQEVLEGYKK

Mascot: http://www.matrixscience.com/

Mascot Search Results

Peptide View

MS/MS Fragmentation of **EAECVEADSGRLASELNHVQEVLEGYKK**
Found in **sp|O43790|KRT86_HUMAN**, Keratin, type II cuticular Hb6 OS=Homo sapiens GN=KRT86 PE=1 SV=1

Match to Query 40813: 3160.501020 from(633.107480,5+) intensity(477941.4063) scans(12966) rtinseconds(2384) index(40799)
Title: 160219_Sunil_SDSI_A_Spectrum095384_scans__12966_RTINSECONDS=2384
Data file C:\\Sunil\\TKAP\\T\\T160219_Sunil_SDSI_A.mgf

Click mouse within plot area to zoom in by factor of two about that point
Or, Plot from 200 to 3000 Da Full range
Label all possible matches ○ Label matches used for scoring ◉

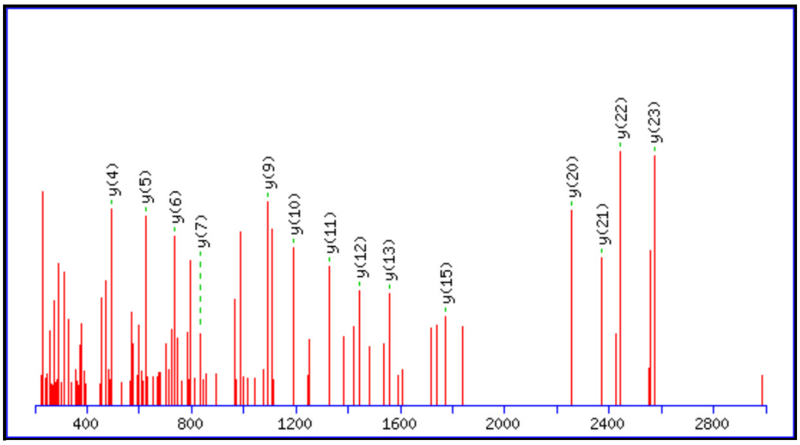

Monoisotopic mass of neutral peptide Mr(calc): 3160.4982
Fixed modifications: Carbamidomethyl (C) (apply to specified residues or termini only)
Variable modifications:
N17 : Deamidated (NQ)
Ions Score: 100 **Expect:** 2.4e-008
Matches : 14/296 fragment ions using 22 most intense peaks (help)

#	b	b^{++}	b^{*}	b^{*++}	b^{0}	b^{0++}	Seq.	y	y^{++}	y^{*}	y^{*++}	y^{0}	y^{0++}	#
1	130.0499	65.5286			112.0393	56.5233	E							28
2	201.0870	101.0471			183.0764	92.0418	A	3032.4630	1516.7351	3015.4364	1508.2218	3014.4524	1507.7298	27
3	330.1296	165.5684			312.1190	156.5631	E	2961.4258	1481.2166	2944.3993	1472.7033	2943.4153	1472.2113	26
4	490.1602	245.5838			472.1497	236.5785	C	2832.3832	1416.6953	2815.3567	1408.1820	2814.3727	1407.6900	25
5	589.2286	295.1180			571.2181	286.1127	V	2672.3526	1336.6799	2655.3261	1328.1667	2654.3420	1327.6747	24
6	718.2712	359.6393			700.2607	350.6340	E	***2573.2842***	1287.1457	2556.2576	1278.6325	2555.2736	1278.1404	23
7	789.3083	395.1578			771.2978	386.1525	A	***2444.2416***	1222.6244	2427.2150	1214.1112	2426.2310	1213.6192	22
8	904.3353	452.6713			886.3247	443.6660	D	***2373.2045***	1187.1059	2356.1779	1178.5926	2355.1939	1178.1006	21
9	991.3673	496.1873			973.3568	487.1820	S	***2258.1775***	1129.5924	2241.1510	1121.0791	2240.1670	1120.5871	20
10	1048.3888	524.6980			1030.3782	515.6927	G	2171.1455	1086.0764	2154.1190	1077.5631	2153.1349	1077.0711	19
11	1204.4899	602.7486	1187.4633	594.2353	1186.4793	593.7433	R	2114.1240	1057.5657	2097.0975	1049.0524	2096.1135	1048.5604	18
12	1317.5740	659.2906	1300.5474	650.7773	1299.5634	650.2853	L	1958.0229	979.5151	1940.9964	971.0018	1940.0124	970.5098	17
13	1388.6111	694.8092	1371.5845	686.2959	1370.6005	685.8039	A	1844.9389	922.9731	1827.9123	914.4598	1826.9283	913.9678	16
14	1475.6431	738.3252	1458.6165	729.8119	1457.6325	729.3199	S	***1773.9018***	887.4545	1756.8752	878.9412	1755.8912	878.4492	15
15	1604.6857	802.8465	1587.6591	794.3332	1586.6751	793.8412	E	1686.8697	843.9385	1669.8432	835.4252	1668.8592	834.9332	14
16	1717.7698	859.3885	1700.7432	850.8752	1699.7592	850.3832	L	***1557.8271***	779.4172	1540.8006	770.9039	1539.8166	770.4119	13
17	1832.7967	916.9020	1815.7701	908.3887	1814.7861	907.8967	N	***1444.7431***	722.8752	1427.7165	714.3619	1426.7325	713.8699	12
18	1969.8556	985.4314	1952.8291	976.9182	1951.8450	976.4262	H	***1329.7161***	665.3617	1312.6896	656.8484	1311.7056	656.3564	11
19	2068.9240	1034.9656	2051.8975	1026.4524	2050.9135	1025.9604	V	***1192.6572***	596.8322	1175.6307	588.3190	1174.6467	587.8270	10
20	2196.9826	1098.9949	2179.9561	1090.4817	2178.9720	1089.9897	Q	***1093.5888***	547.2980	1076.5623	538.7848	1075.5782	538.2928	9
21	2326.0252	1163.5162	2308.9986	1155.0030	2308.0146	1154.5110	E	965.5302	483.2688	948.5037	474.7555	947.5197	474.2635	8
22	2425.0936	1213.0504	2408.0671	1204.5372	2407.0830	1204.0452	V	***836.4876***	418.7475	819.4611	410.2342	818.4771	409.7422	7
23	2538.1777	1269.5925	2521.1511	1261.0792	2520.1671	1260.5872	L	***737.4192***	369.2132	720.3927	360.7000	719.4087	360.2080	6
24	2667.2203	1334.1138	2650.1937	1325.6005	2649.2097	1325.1085	E	***624.3352***	312.6712	607.3086	304.1579	606.3246	303.6659	5
25	2724.2417	1362.6245	2707.2152	1354.1112	2706.2312	1353.6192	G	***495.2926***	248.1499	478.2660	239.6366			4
26	2887.3051	1444.1562	2870.2785	1435.6429	2869.2945	1435.1509	Y	438.2711	219.6392	421.2445	211.1259			3
27	3015.4000	1508.2036	2998.3735	1499.6904	2997.3895	1499.1984	K	275.2078	138.1075	258.1812	129.5942			2

**28**							**K**	147.1128	74.0600	130.0863	65.5468			**1**

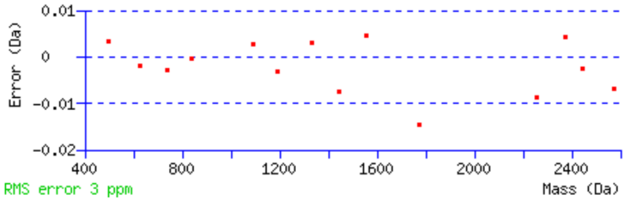

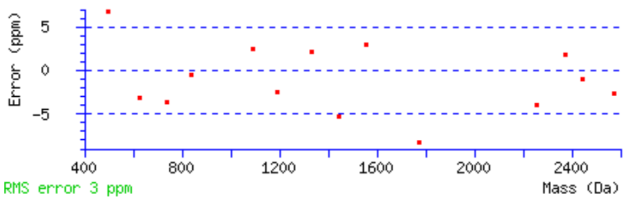

NCBI **BLAST** search of EAECVEADSGRLASELNHVQEVLEGYKK
(Parameters: blastp, nr protein database, expect=20000, no filter, PAM30)
Other BLAST web gateways

All matches to this query

Score	Mr(calc):	Delta	Sequence
100.2	3160.4982	0.0028	EAECVEADSGRLASELNHVQEVLEGYKK
59.1	3160.4982	0.0028	EAECVEADSGRLASELNHVQEVLEGYKK
18.6	3159.5142	0.9868	EAECVEADSGRLASELNHVQEVLEGYKK

Mascot: http://www.matrixscience.com/

Mascot Search Results

Peptide View

MS/MS Fragmentation of **ECCQSNLEPLFAGYIETLRR**
Found in **sp|P78385|KRT83_HUMAN**, Keratin, type II cuticular Hb3 OS=Homo sapiens GN=KRT83 PE=1 SV=2

Match to Query 36308: 2459.130762 from(820.717530,3+) intensity(3796026.0000) scans(16666) rtinseconds(3018) index(13223)
Title: 160219_Sunil_SDSI_A_Spectrum065373_scans__16666_RTINSECONDS=3018
Data file C:\\Sunil\\TKAP\\T\\T160219_Sunil_SDSI_A.mgf

Click mouse within plot area to zoom in by factor of two about that point
Or, Plot from 0 to 2600 Da Full range
Label all possible matches Label matches used for scoring

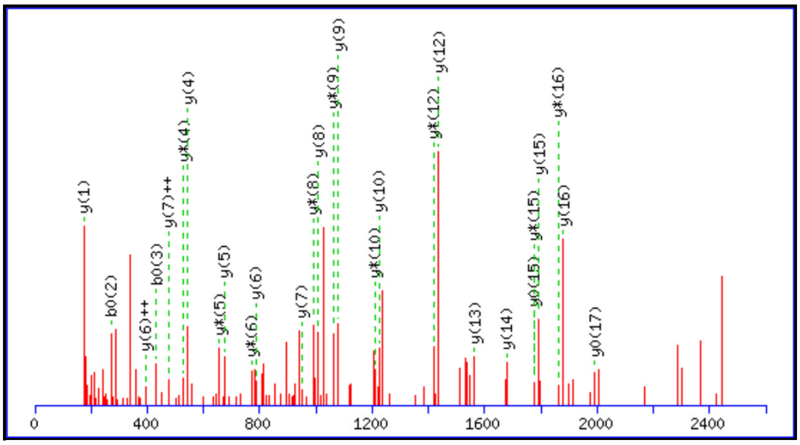

Monoisotopic mass of neutral peptide Mr(calc): 2457.1304
Fixed modifications: Carbamidomethyl (C) (apply to specified residues or termini only)
Variable modifications:
Q4 : Deamidated (NQ)
N6 : Deamidated (NQ)
Ions Score: 52 **Expect:** 0.0014
Matches : 28/216 fragment ions using 72 most intense peaks (help)

#	b	b^{++}	b^{*}	b^{*++}	b^{0}	b^{0++}	Seq.	y	y^{++}	y^{*}	y^{*++}	y^{0}	y^{0++}	#
1	130.0499	65.5286			112.0393	56.5233	E							20
2	290.0805	145.5439			272.0700	136.5386	C	2329.0951	1165.0512	2312.0686	1156.5379	2311.0846	1156.0459	19
3	450.1112	225.5592			432.1006	216.5539	C	2169.0645	1085.0359	2152.0379	1076.5226	2151.0539	1076.0306	18
4	579.1538	290.0805	562.1272	281.5672	561.1432	281.0752	Q	2009.0338	1005.0206	1992.0073	996.5073	1991.0233	996.0153	17
5	666.1858	333.5965	649.1592	325.0833	648.1752	324.5913	S	***1879.9912***	940.4993	1862.9647	931.9860	1861.9807	931.4940	16
6	781.2127	391.1100	764.1862	382.5967	763.2022	382.1047	N	***1792.9592***	896.9832	1775.9327	888.4700	1774.9486	887.9780	15
7	894.2968	447.6520	877.2702	439.1388	876.2862	438.6468	L	***1677.9323***	839.4698	1660.9057	830.9565	1659.9217	830.4645	14
8	1023.3394	512.1733	1006.3128	503.6601	1005.3288	503.1681	E	***1564.8482***	782.9277	1547.8217	774.4145	1546.8376	773.9225	13
9	1120.3922	560.6997	1103.3656	552.1864	1102.3816	551.6944	P	***1435.8056***	718.4064	1418.7791	709.8932	1417.7950	709.4012	12
10	1233.4762	617.2417	1216.4497	608.7285	1215.4657	608.2365	L	1338.7528	669.8801	1321.7263	661.3668	1320.7423	660.8748	11
11	1380.5446	690.7760	1363.5181	682.2627	1362.5341	681.7707	F	***1225.6688***	613.3380	1208.6422	604.8248	1207.6582	604.3327	10
12	1451.5817	726.2945	1434.5552	717.7812	1433.5712	717.2892	A	***1078.6004***	539.8038	1061.5738	531.2905	1060.5898	530.7985	9
13	1508.6032	754.8052	1491.5767	746.2920	1490.5926	745.8000	G	***1007.5633***	504.2853	990.5367	495.7720	989.5527	495.2800	8
14	1671.6665	836.3369	1654.6400	827.8236	1653.6560	827.3316	Y	***950.5418***	475.7745	933.5152	467.2613	932.5312	466.7693	7
15	1784.7506	892.8789	1767.7241	884.3657	1766.7400	883.8737	I	***787.4785***	394.2429	770.4519	385.7296	769.4679	385.2376	6
16	1913.7932	957.4002	1896.7666	948.8870	1895.7826	948.3950	E	***674.3944***	337.7008	657.3678	329.1876	656.3838	328.6956	5
17	2014.8409	1007.9241	1997.8143	999.4108	1996.8303	998.9188	T	***545.3518***	273.1795	528.3253	264.6663	527.3412	264.1743	4
18	2127.9249	1064.4661	2110.8984	1055.9528	2109.9144	1055.4608	L	444.3041	222.6557	427.2776	214.1424			3
19	2284.0260	1142.5167	2266.9995	1134.0034	2266.0155	1133.5114	R	331.2201	166.1137	314.1935	157.6004			2
20							R	***175.1190***	88.0631	158.0924	79.5498			1

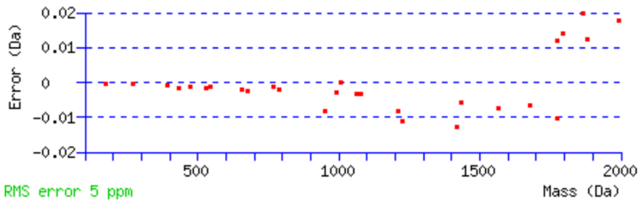

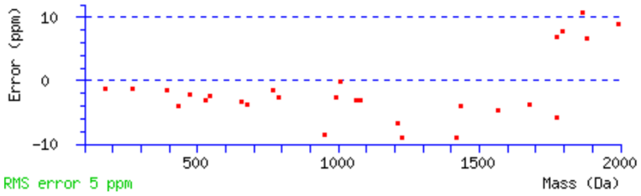

NCBI **BLAST** search of ECCQSNLEPLFAGYIETLRR
(Parameters: blastp, nr protein database, expect=20000, no filter, PAM30)
Other BLAST web gateways

All matches to this query

Score	Mr(calc):	Delta	Sequence
51.5	2457.1304	2.0004	ECCQSNLEPLFAGYIETLRR

Mascot: http://www.matrixscience.com/

Mascot Search Results

Peptide View

MS/MS Fragmentation of **EEINELNR**
Found in **sp|O43790|KRT86_HUMAN**, Keratin, type II cuticular Hb6 OS=Homo sapiens GN=KRT86 PE=1 SV=1

Match to Query 3199: 1016.479348 from(509.246950,2+) intensity(890604.8750) scans(6347) rtinseconds(1248) index(4485)
Title: 160219_Sunil_SDSI_A_Spectrum056593_scans__6347_RTINSECONDS=1248
Data file C:\\Sunil\\TKAP\\T\\T160219_Sunil_SDSI_A.mgf

Click mouse within plot area to zoom in by factor of two about that point
Or, Plot from 50 to 1050 Da Full range
Label all possible matches ○ Label matches used for scoring ◉

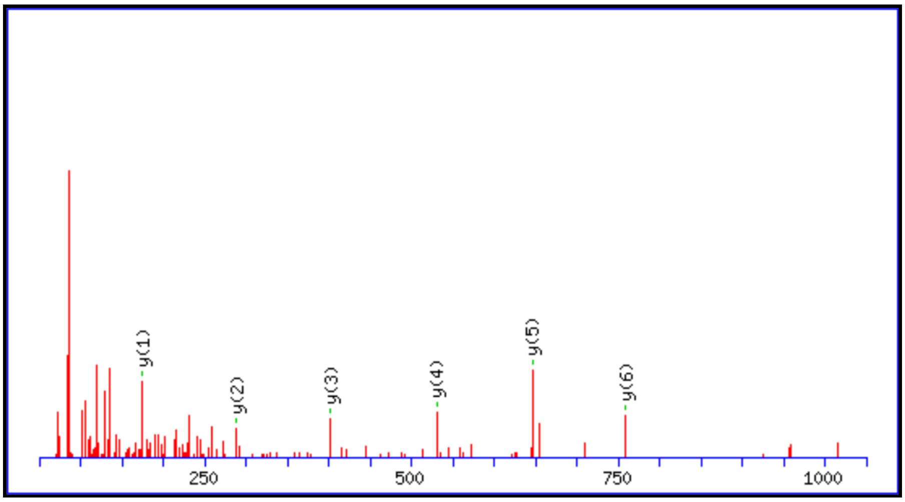

Monoisotopic mass of neutral peptide Mr(calc): 1016.4774
Fixed modifications: Carbamidomethyl (C) (apply to specified residues or termini only)
Variable modifications:
N4 : Deamidated (NQ)
Ions Score: 46 **Expect:** 0.0027
Matches : 6/72 fragment ions using 9 most intense peaks (help)

#	b	b++	b*	b*++	b0	b0++	Seq.	y	y++	y*	y*++	y0	y0++	#
1	130.0499	65.5286			112.0393	56.5233	E							8
2	259.0925	130.0499			241.0819	121.0446	E	888.4421	444.7247	871.4156	436.2114	870.4316	435.7194	7
3	372.1765	186.5919			354.1660	177.5866	I	***759.3995***	380.2034	742.3730	371.6901	741.3890	371.1981	6
4	487.2035	244.1054	470.1769	235.5921	469.1929	235.1001	N	***646.3155***	323.6614	629.2889	315.1481	628.3049	314.6561	5
5	616.2461	308.6267	599.2195	300.1134	598.2355	299.6214	E	***531.2885***	266.1479	514.2620	257.6346	513.2780	257.1426	4
6	729.3301	365.1687	712.3036	356.6554	711.3196	356.1634	L	***402.2459***	201.6266	385.2194	193.1133			3
7	843.3731	422.1902	826.3465	413.6769	825.3625	413.1849	N	***289.1619***	145.0846	272.1353	136.5713			2
8							R	***175.1190***	88.0631	158.0924	79.5498			1

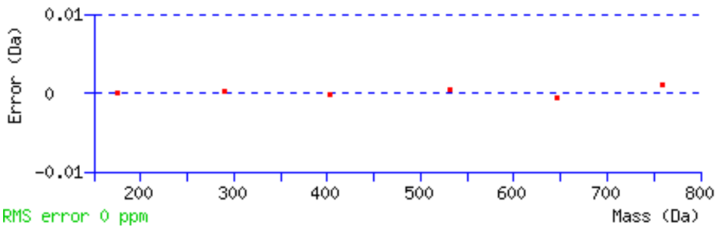

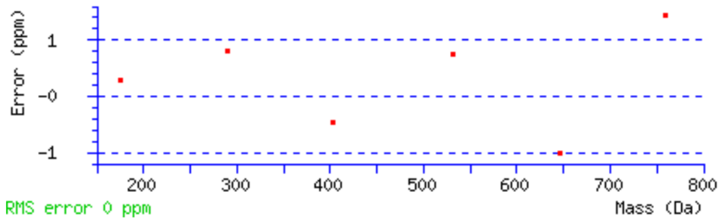

NCBI **BLAST** search of EEINELNR
(Parameters: blastp, nr protein database, expect=20000, no filter, PAM30)
Other BLAST web gateways

All matches to this query

Score	Mr(calc):	Delta	Sequence
45.5	1016.4774	0.0019	EEINELNR
6.1	1016.4774	0.0019	ELQDGIGQR
6.1	1016.4774	0.0019	QLEDNIQR
6.1	1016.4774	0.0019	QLEDNIQR
6.0	1016.4774	0.0019	EEINELNR
6.0	1015.4795	0.9999	QNQEDARR
5.0	1015.4835	0.9958	ANNWEGGIR
5.0	1016.4774	0.0019	EVEQEVQR
5.0	1016.4709	0.0085	MEEPQRAR
5.0	1016.4886	-0.0093	NQELQRAR

Mascot: http://www.matrixscience.com/

Mascot Search Results

Peptide View

MS/MS Fragmentation of **ERSQQQEPLVCASYQSYFK**
Found in **sp|O76009|KT33A_HUMAN**, Keratin, type I cuticular Ha3-I OS=Homo sapiens GN=KRT33A PE=2 SV=2

Match to Query 34877: 2349.074682 from(784.032170,3+) intensity(764581.9375) scans(12384) rtinseconds(2257) index(25139)
Title: 160219_Sunil_SDSI_A_Spectrum078444_scans__12384_RTINSECONDS=2257
Data file C:\\Sunil\\TKAP\\T\\T160219_Sunil_SDSI_A.mgf

Click mouse within plot area to zoom in by factor of two about that point
Or, Plot from 100 to 2100 Da Full range
Label all possible matches ○ Label matches used for scoring ◉

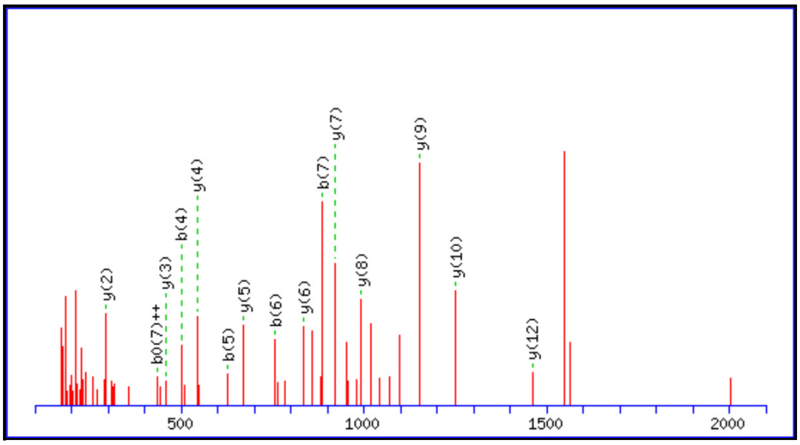

Monoisotopic mass of neutral peptide Mr(calc): 2348.0743
Fixed modifications: Carbamidomethyl (C) (apply to specified residues or termini only)
Variable modifications:
Q6 : Deamidated (NQ)
Ions Score: 64 **Expect:** 7.8e-005
Matches : 15/208 fragment ions using 25 most intense peaks (help)

#	b	b++	b*	b*++	b0	b0++	Seq.	y	y++	y*	y*++	y0	y0++	#
1	130.0499	65.5286			112.0393	56.5233	E							19
2	286.1510	143.5791	269.1244	135.0659	268.1404	134.5738	R	2220.0390	1110.5231	2203.0124	1102.0099	2202.0284	1101.5179	18
3	373.1830	187.0951	356.1565	178.5819	355.1724	178.0899	S	2063.9379	1032.4726	2046.9113	1023.9593	2045.9273	1023.4673	17
4	**501.2416**	251.1244	484.2150	242.6112	483.2310	242.1191	Q	1976.9059	988.9566	1959.8793	980.4433	1958.8953	979.9513	16
5	**629.3002**	315.1537	612.2736	306.6404	611.2896	306.1484	Q	1848.8473	924.9273	1831.8207	916.4140	1830.8367	915.9220	15
6	**758.3428**	379.6750	741.3162	371.1617	740.3322	370.6697	Q	1720.7887	860.8980	1703.7622	852.3847	1702.7781	851.8927	14
7	**887.3854**	444.1963	870.3588	435.6830	869.3748	435.1910	E	1591.7461	796.3767	1574.7196	787.8634	1573.7355	787.3714	13
8	984.4381	492.7227	967.4116	484.2094	966.4276	483.7174	P	***1462.7035***	731.8554	1445.6770	723.3421	1444.6930	722.8501	12
9	1097.5222	549.2647	1080.4956	540.7515	1079.5116	540.2594	L	1365.6508	683.3290	1348.6242	674.8157	1347.6402	674.3237	11
10	1196.5906	598.7989	1179.5640	590.2857	1178.5800	589.7937	V	***1252.5667***	626.7870	1235.5401	618.2737	1234.5561	617.7817	10
11	1356.6212	678.8143	1339.5947	670.3010	1338.6107	669.8090	C	***1153.4983***	577.2528	1136.4717	568.7395	1135.4877	568.2475	9
12	1427.6584	714.3328	1410.6318	705.8195	1409.6478	705.3275	A	***993.4676***	497.2375	976.4411	488.7242	975.4571	488.2322	8
13	1514.6904	757.8488	1497.6638	749.3356	1496.6798	748.8435	S	***922.4305***	461.7189	905.4040	453.2056	904.4199	452.7136	7
14	1677.7537	839.3805	1660.7272	830.8672	1659.7431	830.3752	Y	***835.3985***	418.2029	818.3719	409.6896	817.3879	409.1976	6
15	1805.8123	903.4098	1788.7857	894.8965	1787.8017	894.4045	Q	***672.3352***	336.6712	655.3086	328.1579	654.3246	327.6659	5
16	1892.8443	946.9258	1875.8178	938.4125	1874.8338	937.9205	S	***544.2766***	272.6419	527.2500	264.1287	526.2660	263.6366	4
17	2055.9076	1028.4575	2038.8811	1019.9442	2037.8971	1019.4522	Y	***457.2445***	229.1259	440.2180	220.6126			3
18	2202.9761	1101.9917	2185.9495	1093.4784	2184.9655	1092.9864	F	***294.1812***	147.5942	277.1547	139.0810			2
19							K	147.1128	74.0600	130.0863	65.5468			1

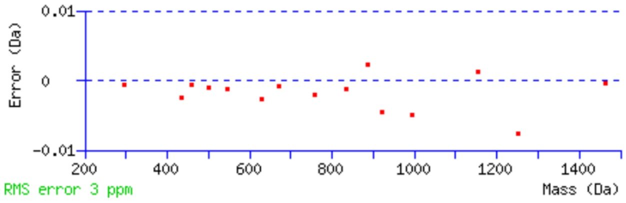

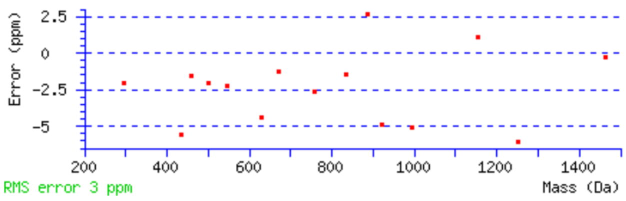

NCBI **BLAST** search of ERSQQQEPLVCASYQSYFK

(Parameters: blastp, nr protein database, expect=20000, no filter, PAM30)
Other BLAST web gateways

All matches to this query

Score	Mr(calc):	Delta	Sequence
63.6	2348.0743	1.0004	ERSQQQEPLVCASYQSYFK
63.0	2347.0903	1.9844	ERSQQQEPLVCASYQSYFK
60.9	2348.0743	1.0004	ERSQQQEPLVCASYQSYFK
59.3	2348.0743	1.0004	ERSQQQEPLVCASYQSYFK
54.8	2349.0583	0.0164	ERSQQQEPLVCASYQSYFK
51.9	2349.0583	0.0164	ERSQQQEPLVCASYQSYFK
51.9	2349.0583	0.0164	ERSQQQEPLVCASYQSYFK
8.0	2349.0583	0.0164	ERSQQQEPLVCASYQSYFK
6.1	2348.0743	1.0004	ERSQQQEPLVCASYQSYFK
3.8	2349.0583	0.0164	ERSQQQEPLVCASYQSYFK

Mascot: http://www.matrixscience.com/

Mascot Search Results

Peptide View

MS/MS Fragmentation of **ETMQFLNDRLASYLEK**
Found in **sp|O76009|KT33A_HUMAN**, Keratin, type I cuticular Ha3-I OS=Homo sapiens GN=KRT33A PE=2 SV=2

Match to Query 27782: 1957.944912 from(653.655580,3+) intensity(1538018.7500) scans(15497) rtinseconds(2791) index(27542)
Title: 160219_Sunil_SDSI_A_Spectrum080857_scans__15497_RTINSECONDS=2791
Data file C:\\Sunil\\TKAP\\T\\T160219_Sunil_SDSI_A.mgf

Click mouse within plot area to zoom in by factor of two about that point
Or, Plot from 100 to 1800 Da Full range
Label all possible matches Label matches used for scoring

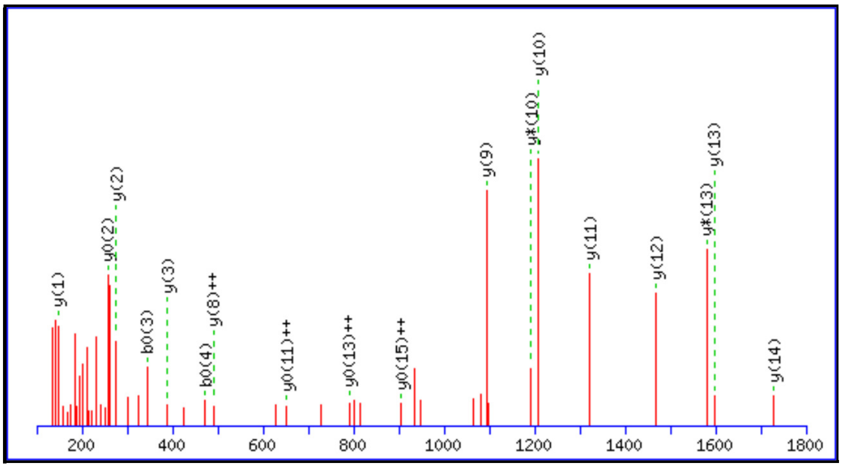

Monoisotopic mass of neutral peptide Mr(calc): 1957.9455
Fixed modifications: Carbamidomethyl (C) (apply to specified residues or termini only)
Variable modifications:
N7 : Deamidated (NQ)
Ions Score: 53 **Expect:** 0.0011
Matches : 18/172 fragment ions using 30 most intense peaks (help)

#	b	b++	b*	b*++	b0	b0++	Seq.	y	y++	y*	y*++	y0	y0++	#
1	130.0499	65.5286			112.0393	56.5233	E							16
2	231.0975	116.0524			213.0870	107.0471	T	1829.9102	915.4587	1812.8837	906.9455	1811.8996	906.4535	15
3	362.1380	181.5727			344.1275	172.5674	M	1728.8625	864.9349	1711.8360	856.4216	1710.8520	855.9296	14
4	490.1966	245.6019	473.1701	237.0887	472.1860	236.5967	Q	1597.8220	799.4147	1580.7955	790.9014	1579.8115	790.4094	13
5	637.2650	319.1362	620.2385	310.6229	619.2545	310.1309	F	1469.7635	735.3854	1452.7369	726.8721	1451.7529	726.3801	12
6	750.3491	375.6782	733.3225	367.1649	732.3385	366.6729	L	1322.6951	661.8512	1305.6685	653.3379	1304.6845	652.8459	11
7	865.3760	433.1917	848.3495	424.6784	847.3655	424.1864	N	1209.6110	605.3091	1192.5844	596.7959	1191.6004	596.3039	10
8	980.4030	490.7051	963.3764	482.1919	962.3924	481.6998	D	1094.5841	547.7957	1077.5575	539.2824	1076.5735	538.7904	9
9	1136.5041	568.7557	1119.4775	560.2424	1118.4935	559.7504	R	979.5571	490.2822	962.5306	481.7689	961.5465	481.2769	8
10	1249.5881	625.2977	1232.5616	616.7844	1231.5776	616.2924	L	823.4560	412.2316	806.4294	403.7184	805.4454	403.2264	7
11	1320.6253	660.8163	1303.5987	652.3030	1302.6147	651.8110	A	710.3719	355.6896	693.3454	347.1763	692.3614	346.6843	6
12	1407.6573	704.3323	1390.6307	695.8190	1389.6467	695.3270	S	639.3348	320.1710	622.3083	311.6578	621.3243	311.1658	5
13	1570.7206	785.8639	1553.6941	777.3507	1552.7101	776.8587	Y	552.3028	276.6550	535.2762	268.1418	534.2922	267.6498	4
14	1683.8047	842.4060	1666.7781	833.8927	1665.7941	833.4007	L	389.2395	195.1234	372.2129	186.6101	371.2289	186.1181	3
15	1812.8473	906.9273	1795.8207	898.4140	1794.8367	897.9220	E	276.1554	138.5813	259.1288	130.0681	258.1448	129.5761	2
16							K	147.1128	74.0600	130.0863	65.5468			1

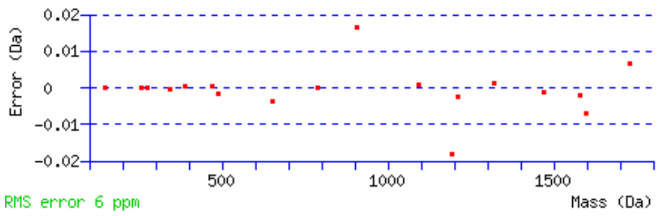

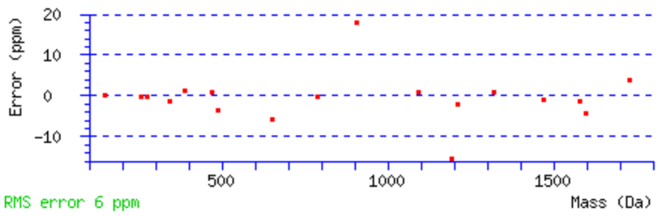

NCBI **BLAST** search of ETMQFLNDRLASYLEK

(Parameters: blastp, nr protein database, expect=20000, no filter, PAM30)
Other BLAST web gateways

All matches to this query

Score	Mr(calc):	Delta	Sequence
53.0	1957.9455	-0.0006	ETMQFLNDRLASYLEK
39.8	1957.9567	-0.0118	ETMQFLNDRLASYLTR
20.1	1957.9455	-0.0006	ETMQFLNDRLASYLEK
12.6	1957.9567	-0.0118	ETMQFLNDRLASYLTR
1.5	1956.9503	0.9947	EKGDGVYQKGMDFILEK

Mascot: http://www.matrixscience.com/

Mascot Search Results

Peptide View

MS/MS Fragmentation of **EVEQWFTTQTEELNKQVVSSSEQLQSYQAEIIELRR**
Found in **sp|Q15323|K1H1_HUMAN**, Keratin, type I cuticular Ha1 OS=Homo sapiens GN=KRT31 PE=2 SV=3

Match to Query 44450: 4326.147070 from(866.236690,5+) intensity(986581.6875) scans(16141) rtinseconds(2935) index(43604)
Title: 160219_Sunil_SDSI_A_Spectrum098190_scans__16141_RTINSECONDS=2935
Data file C:\\Sunil\\TKAP\\T\\T160219_Sunil_SDSI_A.mgf

Click mouse within plot area to zoom in by factor of two about that point
Or, Plot from 200 to 3400 Da Full range
Label all possible matches Label matches used for scoring

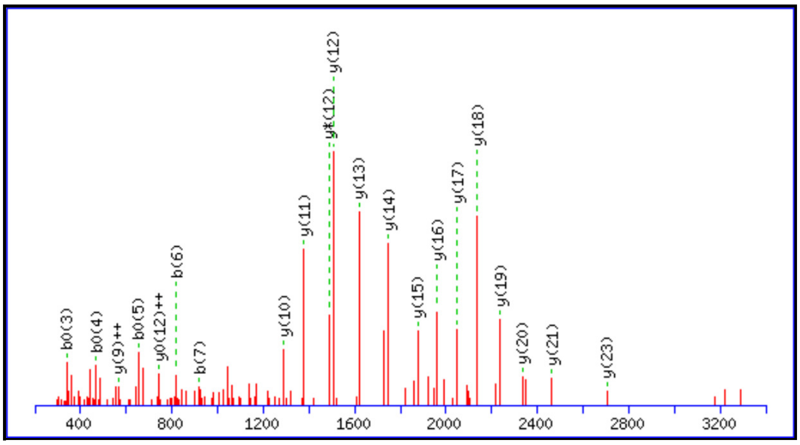

Monoisotopic mass of neutral peptide Mr(calc): 4326.1291
Fixed modifications: Carbamidomethyl (C) (apply to specified residues or termini only)
Variable modifications:
N14 : Deamidated (NQ)
Ions Score: 99 **Expect:** 2.3e-008
Matches : 21/408 fragment ions using 25 most intense peaks (help)

#	b	b++	b*	b*++	b0	b0++	Seq.	y	y++	y*	y*++	y0	y0++	#
1	130.0499	65.5286			112.0393	56.5233	E							36
2	229.1183	115.0628			211.1077	106.0575	V	4198.0939	2099.5506	4181.0673	2091.0373	4180.0833	2090.5453	35
3	358.1609	179.5841			340.1503	170.5788	E	4099.0255	2050.0164	4081.9989	2041.5031	4081.0149	2041.0111	34
4	486.2195	243.6134	469.1929	235.1001	468.2089	234.6081	Q	3969.9829	1985.4951	3952.9563	1976.9818	3951.9723	1976.4898	33
5	672.2988	336.6530	655.2722	328.1397	654.2882	327.6477	W	3841.9243	1921.4658	3824.8977	1912.9525	3823.9137	1912.4605	32
6	**819.3672**	410.1872	802.3406	401.6740	801.3566	401.1819	F	3655.8450	1828.4261	3638.8184	1819.9129	3637.8344	1819.4208	31
7	**920.4149**	460.7111	903.3883	452.1978	902.4043	451.7058	T	3508.7766	1754.8919	3491.7500	1746.3786	3490.7660	1745.8866	30
8	1021.4625	511.2349	1004.4360	502.7216	1003.4520	502.2296	T	3407.7289	1704.3681	3390.7023	1695.8548	3389.7183	1695.3628	29
9	1149.5211	575.2642	1132.4946	566.7509	1131.5106	566.2589	Q	3306.6812	1653.8442	3289.6547	1645.3310	3288.6706	1644.8390	28
10	1250.5688	625.7880	1233.5422	617.2748	1232.5582	616.7828	T	3178.6226	1589.8150	3161.5961	1581.3017	3160.6121	1580.8097	27
11	1379.6114	690.3093	1362.5848	681.7961	1361.6008	681.3040	E	3077.5749	1539.2911	3060.5484	1530.7778	3059.5644	1530.2858	26
12	1508.6540	754.8306	1491.6274	746.3174	1490.6434	745.8253	E	2948.5324	1474.7698	2931.5058	1466.2565	2930.5218	1465.7645	25
13	1621.7380	811.3727	1604.7115	802.8594	1603.7275	802.3674	L	2819.4898	1410.2485	2802.4632	1401.7352	2801.4792	1401.2432	24
14	1736.7650	868.8861	1719.7384	860.3729	1718.7544	859.8809	N	***2706.4057***	1353.7065	2689.3792	1345.1932	2688.3951	1344.7012	23
15	1864.8600	932.9336	1847.8334	924.4203	1846.8494	923.9283	K	2591.3788	1296.1930	2574.3522	1287.6797	2573.3682	1287.1877	22
16	1992.9185	996.9629	1975.8920	988.4496	1974.9080	987.9576	Q	***2463.2838***	1232.1455	2446.2572	1223.6323	2445.2732	1223.1403	21
17	2091.9869	1046.4971	2074.9604	1037.9838	2073.9764	1037.4918	V	***2335.2252***	1168.1162	2318.1987	1159.6030	2317.2147	1159.1110	20
18	2191.0554	1096.0313	2174.0288	1087.5180	2173.0448	1087.0260	V	***2236.1568***	1118.5820	2219.1303	1110.0688	2218.1462	1109.5768	19
19	2278.0874	1139.5473	2261.0608	1131.0341	2260.0768	1130.5420	S	***2137.0884***	1069.0478	2120.0618	1060.5346	2119.0778	1060.0425	18
20	2365.1194	1183.0633	2348.0929	1174.5501	2347.1088	1174.0581	S	***2050.0564***	1025.5318	2033.0298	1017.0185	2032.0458	1016.5265	17
21	2452.1514	1226.5794	2435.1249	1218.0661	2434.1409	1217.5741	S	***1963.0243***	982.0158	1945.9978	973.5025	1945.0138	973.0105	16
22	2581.1940	1291.1007	2564.1675	1282.5874	2563.1835	1282.0954	E	***1875.9923***	938.4998	1858.9658	929.9865	1857.9817	929.4945	15
23	2709.2526	1355.1299	2692.2261	1346.6167	2691.2420	1346.1247	Q	***1746.9497***	873.9785	1729.9232	865.4652	1728.9391	864.9732	14
24	2822.3367	1411.6720	2805.3101	1403.1587	2804.3261	1402.6667	L	***1618.8911***	809.9492	1601.8646	801.4359	1600.8806	800.9439	13
25	2950.3953	1475.7013	2933.3687	1467.1880	2932.3847	1466.6960	Q	***1505.8071***	753.4072	1488.7805	744.8939	1487.7965	744.4019	12
26	3037.4273	1519.2173	3020.4007	1510.7040	3019.4167	1510.2120	S	***1377.7485***	689.3779	1360.7219	680.8646	1359.7379	680.3726	11
27	3200.4906	1600.7489	3183.4641	1592.2357	3182.4800	1591.7437	Y	***1290.7165***	645.8619	1273.6899	637.3486	1272.7059	636.8566	10

**28**	3328.5492	1664.7782	3311.5226	1656.2650	3310.5386	1655.7730	**Q**	1127.6531	564.3302	1110.6266	555.8169	1109.6426	555.3249	**9**
**29**	3399.5863	1700.2968	3382.5598	1691.7835	3381.5757	1691.2915	**A**	999.5946	500.3009	982.5680	491.7876	981.5840	491.2956	**8**
**30**	3528.6289	1764.8181	3511.6023	1756.3048	3510.6183	1755.8128	**E**	928.5574	464.7824	911.5309	456.2691	910.5469	455.7771	**7**
**31**	3641.7130	1821.3601	3624.6864	1812.8468	3623.7024	1812.3548	**I**	799.5148	400.2611	782.4883	391.7478	781.5043	391.2558	**6**
**32**	3754.7970	1877.9022	3737.7705	1869.3889	3736.7865	1868.8969	**I**	686.4308	343.7190	669.4042	335.2058	668.4202	334.7137	**5**
**33**	3883.8396	1942.4234	3866.8131	1933.9102	3865.8291	1933.4182	**E**	573.3467	287.1770	556.3202	278.6637	555.3362	278.1717	**4**
**34**	3996.9237	1998.9655	3979.8971	1990.4522	3978.9131	1989.9602	**L**	444.3041	222.6557	427.2776	214.1424			**3**
**35**	4153.0248	2077.0160	4135.9982	2068.5028	4135.0142	2068.0108	**R**	331.2201	166.1137	314.1935	157.6004			**2**
**36**							**R**	175.1190	88.0631	158.0924	79.5498			**1**

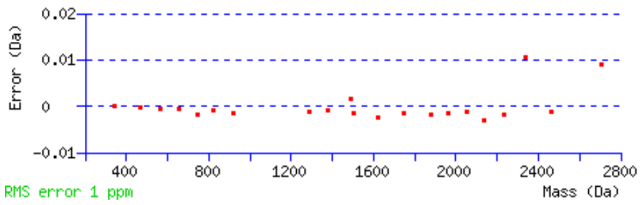

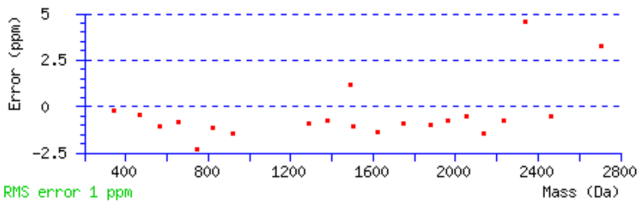

NCBI **BLAST** search of EVEQWFTTQTEELNKQVVSSSEQLQSYQAEIIELRR
(Parameters: blastp, nr protein database, expect=20000, no filter, PAM30)
Other BLAST web gateways

All matches to this query

Score	Mr(calc):	Delta	Sequence
99.4	4326.1291	0.0179	EVEQWFTTQTEELNKQVVSSSEQLQSYQAEIIELRR
99.4	4325.1451	1.0019	EVEQWFTTQTEELNKQVVSSSEQLQSYQAEIIELRR
99.4	4326.1291	0.0179	EVEQWFTTQTEELNKQVVSSSEQLQSYQAEIIELRR
85.4	4326.1291	0.0179	EVEQWFTTQTEELNKQVVSSSEQLQSYQAEIIELRR
81.5	4326.1291	0.0179	EVEQWFTTQTEELNKQVVSSSEQLQSYQAEIIELRR
14.3	4326.1291	0.0179	EVEQWFTTQTEELNKQVVSSSEQLQSYQAEIIELRR

Mascot: http://www.matrixscience.com/

Mascot Search Results

Peptide View

MS/MS Fragmentation of **EYQEVMNSKLGLDIEIATYRR**
Found in **sp|O43790|KRT86_HUMAN**, Keratin, type II cuticular Hb6 OS=Homo sapiens GN=KRT86 PE=1 SV=1

Match to Query 37114: 2528.249052 from(843.756960,3+) intensity(3075383.0000) scans(14614) rtinseconds(2665) index(11492)
Title: 160219_Sunil_SDSI_A_Spectrum063620_scans__14614_RTINSECONDS=2665
Data file C:\\Sunil\\TKAP\\T\\T160219_Sunil_SDSI_A.mgf

Click mouse within plot area to zoom in by factor of two about that point
Or, Plot from 0 to 2600 Da Full range
Label all possible matches Label matches used for scoring

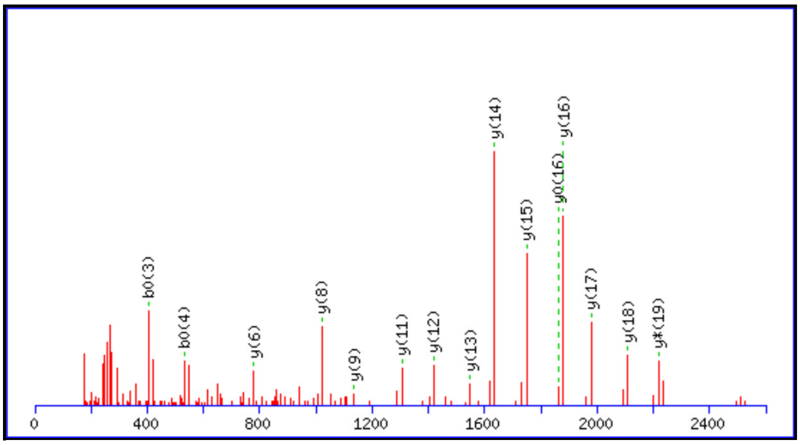

Monoisotopic mass of neutral peptide Mr(calc): 2528.2580
Fixed modifications: Carbamidomethyl (C) (apply to specified residues or termini only)
Variable modifications:
N7 : Deamidated (NQ)
Ions Score: 77 **Expect:** 5.2e-006
Matches : 15/230 fragment ions using 22 most intense peaks (help)

#	b	b^{++}	b*	b^{*++}	b^{0}	b^{0++}	Seq.	y	y^{++}	y*	y^{*++}	y^{0}	y^{0++}	#
1	130.0499	65.5286			112.0393	56.5233	E							21
2	293.1132	147.0602			275.1026	138.0550	Y	2400.2228	1200.6150	2383.1962	1192.1018	2382.2122	1191.6097	20
3	421.1718	211.0895	404.1452	202.5763	403.1612	202.0842	Q	2237.1594	1119.0834	2220.1329	1110.5701	2219.1489	1110.0781	19
4	550.2144	275.6108	533.1878	267.0975	532.2038	266.6055	E	***2109.1009***	1055.0541	2092.0743	1046.5408	2091.0903	1046.0488	18
5	649.2828	325.1450	632.2562	316.6318	631.2722	316.1397	V	***1980.0583***	990.5328	1963.0317	982.0195	1962.0477	981.5275	17
6	780.3233	390.6653	763.2967	382.1520	762.3127	381.6600	M	***1880.9899***	940.9986	1863.9633	932.4853	1862.9793	931.9933	16
7	895.3502	448.1787	878.3237	439.6655	877.3396	439.1735	N	***1749.9494***	875.4783	1732.9228	866.9651	1731.9388	866.4730	15
8	982.3822	491.6948	965.3557	483.1815	964.3717	482.6895	S	***1634.9224***	817.9649	1617.8959	809.4516	1616.9119	808.9596	14
9	1110.4772	555.7422	1093.4507	547.2290	1092.4666	546.7370	K	***1547.8904***	774.4488	1530.8639	765.9356	1529.8798	765.4436	13
10	1223.5613	612.2843	1206.5347	603.7710	1205.5507	603.2790	L	***1419.7954***	710.4014	1402.7689	701.8881	1401.7849	701.3961	12
11	1280.5827	640.7950	1263.5562	632.2817	1262.5722	631.7897	G	***1306.7114***	653.8593	1289.6848	645.3461	1288.7008	644.8540	11
12	1393.6668	697.3370	1376.6402	688.8238	1375.6562	688.3318	L	1249.6899	625.3486	1232.6634	616.8353	1231.6793	616.3433	10
13	1508.6937	754.8505	1491.6672	746.3372	1490.6832	745.8452	D	***1136.6058***	568.8066	1119.5793	560.2933	1118.5953	559.8013	9
14	1621.7778	811.3925	1604.7513	802.8793	1603.7672	802.3873	I	***1021.5789***	511.2931	1004.5524	502.7798	1003.5683	502.2878	8
15	1750.8204	875.9138	1733.7938	867.4006	1732.8098	866.9086	E	908.4948	454.7511	891.4683	446.2378	890.4843	445.7458	7
16	1863.9045	932.4559	1846.8779	923.9426	1845.8939	923.4506	I	***779.4522***	390.2298	762.4257	381.7165	761.4417	381.2245	6
17	1934.9416	967.9744	1917.9150	959.4611	1916.9310	958.9691	A	666.3682	333.6877	649.3416	325.1745	648.3576	324.6824	5
18	2035.9893	1018.4983	2018.9627	1009.9850	2017.9787	1009.4930	T	595.3311	298.1692	578.3045	289.6559	577.3205	289.1639	4
19	2199.0526	1100.0299	2182.0260	1091.5167	2181.0420	1091.0246	Y	494.2834	247.6453	477.2568	239.1321			3
20	2355.1537	1178.0805	2338.1271	1169.5672	2337.1431	1169.0752	R	331.2201	166.1137	314.1935	157.6004			2
21							R	175.1190	88.0631	158.0924	79.5498			1

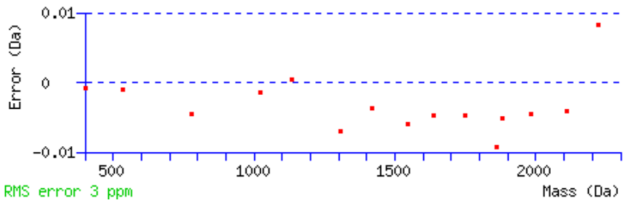

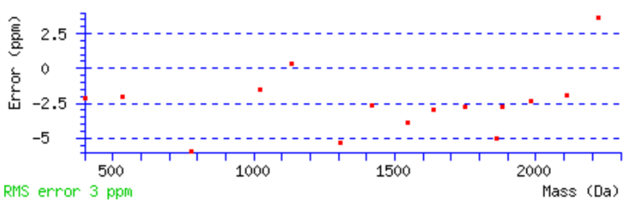

NCBI **BLAST** search of EYQEVMNSKLGLDIEIATYRR
(Parameters: blastp, nr protein database, expect=20000, no filter, PAM30)
Other BLAST web gateways

All matches to this query

Score	Mr(calc):	Delta	Sequence
77.4	2528.2580	-0.0090	EYQEVMNSKLGLDIEIATYRR
33.9	2528.2580	-0.0090	EYQEVMNSKLGLDIEIATYRR

Mascot: http://www.matrixscience.com/

Mascot Search Results

Peptide View

MS/MS Fragmentation of **EYQEVMNSKLGLDIEIATYRR**
Found in **sp|O43790|KRT86_HUMAN**, Keratin, type II cuticular Hb6 OS=Homo sapiens GN=KRT86 PE=1 SV=1

Match to Query 37116: 2528.261656 from(633.072690,4+) intensity(1130195.5000) scans(14198) rtinseconds(2569) index(26490)
Title: 160219_Sunil_SDSI_A_Spectrum079796_scans__14198_RTINSECONDS=2569
Data file C:\\Sunil\\TKAP\\T\\T160219_Sunil_SDSI_A.mgf

Click mouse within plot area to zoom in by factor of two about that point
Or, Plot from 0 to 2400 Da Full range
Label all possible matches Label matches used for scoring

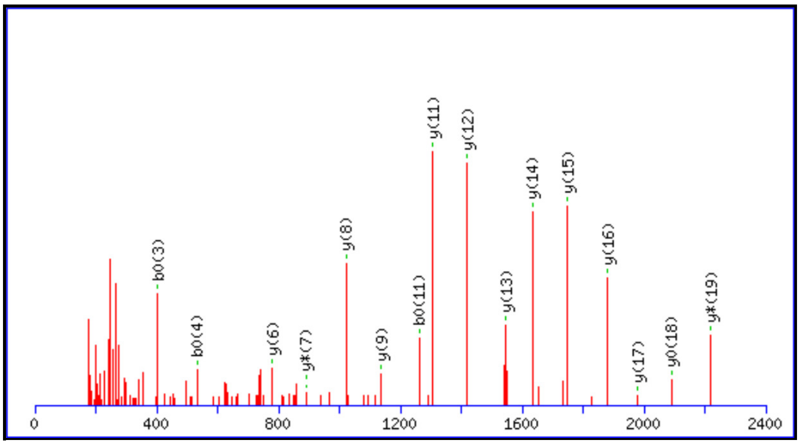

Monoisotopic mass of neutral peptide Mr(calc): 2528.2580
Fixed modifications: Carbamidomethyl (C) (apply to specified residues or termini only)
Variable modifications:
N7 : Deamidated (NQ)
Ions Score: 66 **Expect:** 7e-005
Matches : 16/230 fragment ions using 21 most intense peaks (help)

#	b	b++	b*	b*++	b0	b0++	Seq.	y	y++	y*	y*++	y0	y0++	#
1	130.0499	65.5286			112.0393	56.5233	E							21
2	293.1132	147.0602			275.1026	138.0550	Y	2400.2228	1200.6150	2383.1962	1192.1018	2382.2122	1191.6097	20
3	421.1718	211.0895	404.1452	202.5763	403.1612	202.0842	Q	2237.1594	1119.0834	2220.1329	1110.5701	2219.1489	1110.0781	19
4	550.2144	275.6108	533.1878	267.0975	532.2038	266.6055	E	2109.1009	1055.0541	2092.0743	1046.5408	2091.0903	1046.0488	18
5	649.2828	325.1450	632.2562	316.6318	631.2722	316.1397	V	***1980.0583***	990.5328	1963.0317	982.0195	1962.0477	981.5275	17
6	780.3233	390.6653	763.2967	382.1520	762.3127	381.6600	M	***1880.9899***	940.9986	1863.9633	932.4853	1862.9793	931.9933	16
7	895.3502	448.1787	878.3237	439.6655	877.3396	439.1735	N	***1749.9494***	875.4783	1732.9228	866.9651	1731.9388	866.4730	15
8	982.3822	491.6948	965.3557	483.1815	964.3717	482.6895	S	***1634.9224***	817.9649	1617.8959	809.4516	1616.9119	808.9596	14
9	1110.4772	555.7422	1093.4507	547.2290	1092.4666	546.7370	K	***1547.8904***	774.4488	1530.8639	765.9356	1529.8798	765.4436	13
10	1223.5613	612.2843	1206.5347	603.7710	1205.5507	603.2790	L	***1419.7954***	710.4014	1402.7689	701.8881	1401.7849	701.3961	12
11	1280.5827	640.7950	1263.5562	632.2817	1262.5722	631.7897	G	***1306.7114***	653.8593	1289.6848	645.3461	1288.7008	644.8540	11
12	1393.6668	697.3370	1376.6402	688.8238	1375.6562	688.3318	L	1249.6899	625.3486	1232.6634	616.8353	1231.6793	616.3433	10
13	1508.6937	754.8505	1491.6672	746.3372	1490.6832	745.8452	D	***1136.6058***	568.8066	1119.5793	560.2933	1118.5953	559.8013	9
14	1621.7778	811.3925	1604.7513	802.8793	1603.7672	802.3873	I	***1021.5789***	511.2931	1004.5524	502.7798	1003.5683	502.2878	8
15	1750.8204	875.9138	1733.7938	867.4006	1732.8098	866.9086	E	908.4948	454.7511	891.4683	446.2378	890.4843	445.7458	7
16	1863.9045	932.4559	1846.8779	923.9426	1845.8939	923.4506	I	***779.4522***	390.2298	762.4257	381.7165	761.4417	381.2245	6
17	1934.9416	967.9744	1917.9150	959.4611	1916.9310	958.9691	A	666.3682	333.6877	649.3416	325.1745	648.3576	324.6824	5
18	2035.9893	1018.4983	2018.9627	1009.9850	2017.9787	1009.4930	T	595.3311	298.1692	578.3045	289.6559	577.3205	289.1639	4
19	2199.0526	1100.0299	2182.0260	1091.5167	2181.0420	1091.0246	Y	494.2834	247.6453	477.2568	239.1321			3
20	2355.1537	1178.0805	2338.1271	1169.5672	2337.1431	1169.0752	R	331.2201	166.1137	314.1935	157.6004			2
21							R	175.1190	88.0631	158.0924	79.5498			1

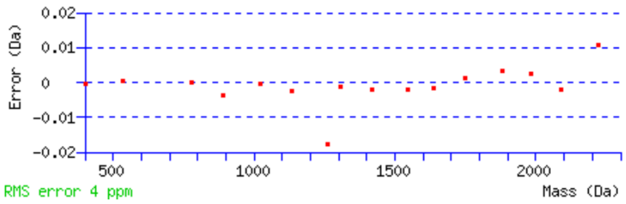

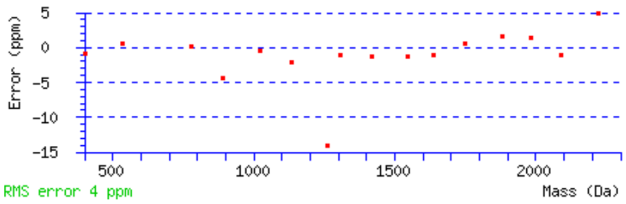

NCBI **BLAST** search of EYQEVMNSKLGLDIEIATYRR
(Parameters: blastp, nr protein database, expect=20000, no filter, PAM30)
Other BLAST web gateways

All matches to this query

Score	Mr(calc):	Delta	Sequence
66.1	2528.2580	0.0036	EYQEVMNSKLGLDIEIATYRR
30.9	2528.2580	0.0036	EYQEVMNSKLGLDIEIATYRR
28.5	2527.2740	0.9876	EYQEVMNSKLGLDIEIATYRR

Mascot: http://www.matrixscience.com/

Mascot Search Results

Peptide View

MS/MS Fragmentation of **EYQEVMNSKLGLDIEIATYRR**
Found in **sp|P78385|KRT83_HUMAN**, Keratin, type II cuticular Hb3 OS=Homo sapiens GN=KRT83 PE=1 SV=2

Match to Query 37116: 2528.261656 from(633.072690,4+) intensity(1130195.5000) scans(14198) rtinseconds(2569) index(26490)
Title: 160219_Sunil_SDSI_A_Spectrum079796_scans__14198_RTINSECONDS=2569
Data file C:\\Sunil\\TKAP\\T\\T160219_Sunil_SDSI_A.mgf

Click mouse within plot area to zoom in by factor of two about that point
Or, Plot from 0 to 2400 Da Full range
Label all possible matches ○ Label matches used for scoring ●

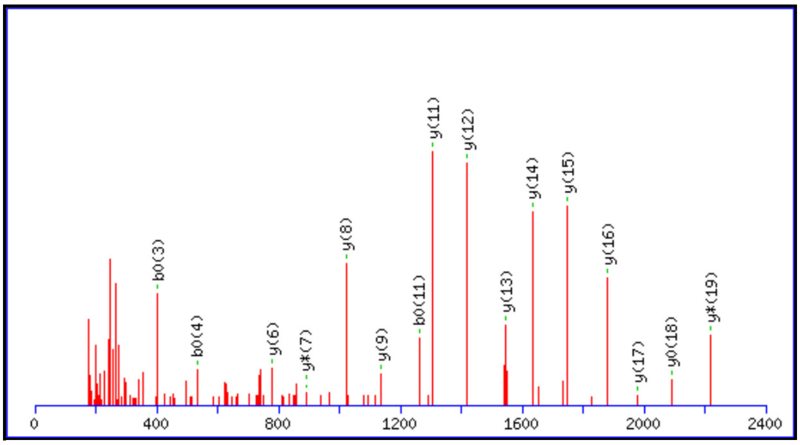

Monoisotopic mass of neutral peptide Mr(calc): 2528.2580
Fixed modifications: Carbamidomethyl (C) (apply to specified residues or termini only)
Variable modifications:
N7 : Deamidated (NQ)
Ions Score: 66 **Expect:** 7e-005
Matches : 16/230 fragment ions using 21 most intense peaks (help)

#	b	b^{++}	b^{*}	b^{*++}	b^{0}	b^{0++}	Seq.	y	y^{++}	y^{*}	y^{*++}	y^{0}	y^{0++}	#
1	130.0499	65.5286			112.0393	56.5233	E							21
2	293.1132	147.0602			275.1026	138.0550	Y	2400.2228	1200.6150	2383.1962	1192.1018	2382.2122	1191.6097	20
3	421.1718	211.0895	404.1452	202.5763	403.1612	202.0842	Q	2237.1594	1119.0834	2220.1329	1110.5701	2219.1489	1110.0781	19
4	550.2144	275.6108	533.1878	267.0975	532.2038	266.6055	E	2109.1009	1055.0541	2092.0743	1046.5408	2091.0903	1046.0488	18
5	649.2828	325.1450	632.2562	316.6318	631.2722	316.1397	V	***1980.0583***	990.5328	1963.0317	982.0195	1962.0477	981.5275	17
6	780.3233	390.6653	763.2967	382.1520	762.3127	381.6600	M	***1880.9899***	940.9986	1863.9633	932.4853	1862.9793	931.9933	16
7	895.3502	448.1787	878.3237	439.6655	877.3396	439.1735	N	***1749.9494***	875.4783	1732.9228	866.9651	1731.9388	866.4730	15
8	982.3822	491.6948	965.3557	483.1815	964.3717	482.6895	S	***1634.9224***	817.9649	1617.8959	809.4516	1616.9119	808.9596	14
9	1110.4772	555.7422	1093.4507	547.2290	1092.4666	546.7370	K	***1547.8904***	774.4488	1530.8639	765.9356	1529.8798	765.4436	13
10	1223.5613	612.2843	1206.5347	603.7710	1205.5507	603.2790	L	***1419.7954***	710.4014	1402.7689	701.8881	1401.7849	701.3961	12
11	1280.5827	640.7950	1263.5562	632.2817	1262.5722	631.7897	G	***1306.7114***	653.8593	1289.6848	645.3461	1288.7008	644.8540	11
12	1393.6668	697.3370	1376.6402	688.8238	1375.6562	688.3318	L	1249.6899	625.3486	1232.6634	616.8353	1231.6793	616.3433	10
13	1508.6937	754.8505	1491.6672	746.3372	1490.6832	745.8452	D	***1136.6058***	568.8066	1119.5793	560.2933	1118.5953	559.8013	9
14	1621.7778	811.3925	1604.7513	802.8793	1603.7672	802.3873	I	***1021.5789***	511.2931	1004.5524	502.7798	1003.5683	502.2878	8
15	1750.8204	875.9138	1733.7938	867.4006	1732.8098	866.9086	E	908.4948	454.7511	891.4683	446.2378	890.4843	445.7458	7
16	1863.9045	932.4559	1846.8779	923.9426	1845.8939	923.4506	I	***779.4522***	390.2298	762.4257	381.7165	761.4417	381.2245	6
17	1934.9416	967.9744	1917.9150	959.4611	1916.9310	958.9691	A	666.3682	333.6877	649.3416	325.1745	648.3576	324.6824	5
18	2035.9893	1018.4983	2018.9627	1009.9850	2017.9787	1009.4930	T	595.3311	298.1692	578.3045	289.6559	577.3205	289.1639	4
19	2199.0526	1100.0299	2182.0260	1091.5167	2181.0420	1091.0246	Y	494.2834	247.6453	477.2568	239.1321			3
20	2355.1537	1178.0805	2338.1271	1169.5672	2337.1431	1169.0752	R	331.2201	166.1137	314.1935	157.6004			2
21							R	175.1190	88.0631	158.0924	79.5498			1

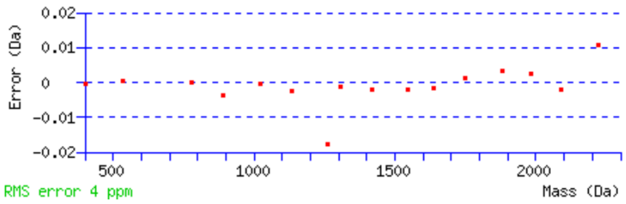

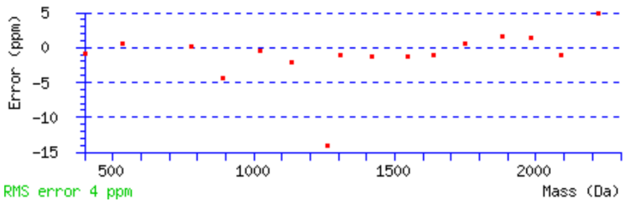

NCBI **BLAST** search of EYQEVMNSKLGLDIEIATYRR
(Parameters: blastp, nr protein database, expect=20000, no filter, PAM30)
Other BLAST web gateways

All matches to this query

Score	Mr(calc):	Delta	Sequence
66.1	2528.2580	0.0036	EYQEVMNSKLGLDIEIATYRR
30.9	2528.2580	0.0036	EYQEVMNSKLGLDIEIATYRR
28.5	2527.2740	0.9876	EYQEVMNSKLGLDIEIATYRR

Mascot: http://www.matrixscience.com/

Mascot Search Results

Peptide View

MS/MS Fragmentation of **FLEQQNKLLETK**
Found in **sp|O43790|KRT86_HUMAN**, Keratin, type II cuticular Hb6 OS=Homo sapiens GN=KRT86 PE=1 SV=1

Match to Query 17449: 1490.798352 from(497.940060,3+) intensity(5874160.0000) scans(9629) rtinseconds(1809) index(7352)
Title: 160219_Sunil_SDSI_A_Spectrum059460_scans__9629_RTINSECONDS=1809
Data file C:\\Sunil\\TKAP\\T\\T160219_Sunil_SDSI_A.mgf

Click mouse within plot area to zoom in by factor of two about that point
Or, Plot from 100 to 1400 Da Full range
Label all possible matches ○ Label matches used for scoring ◉

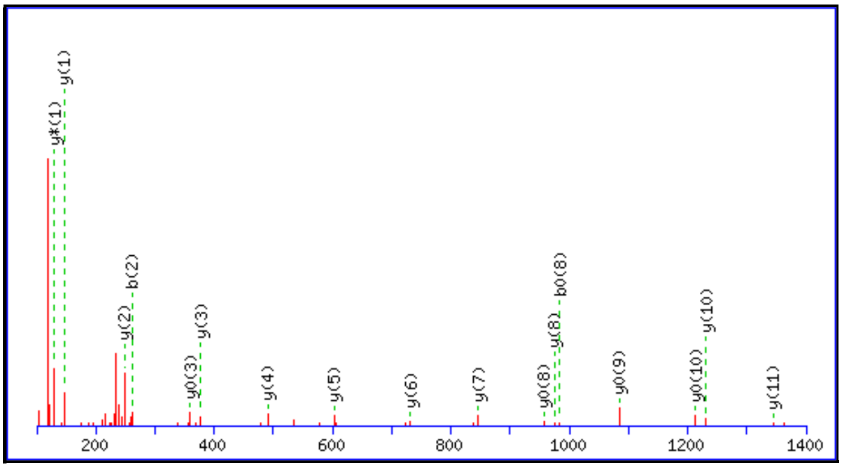

Monoisotopic mass of neutral peptide Mr(calc): 1490.7980
Fixed modifications: Carbamidomethyl (C) (apply to specified residues or termini only)
Variable modifications:
N6 : Deamidated (NQ)
Ions Score: 69 **Expect:** 2.2e-005
Matches : 17/120 fragment ions using 30 most intense peaks (help)

#	b	b++	b*	b*++	b0	b0++	Seq.	y	y++	y*	y*++	y0	y0++	#
1	148.0757	74.5415					F							12
2	**261.1598**	131.0835					L	***1344.7369***	672.8721	1327.7104	664.3588	1326.7264	663.8668	11
3	390.2023	195.6048			372.1918	186.5995	E	***1231.6529***	616.3301	1214.6263	607.8168	1213.6423	607.3248	10
4	518.2609	259.6341	501.2344	251.1208	500.2504	250.6288	Q	1102.6103	551.8088	1085.5837	543.2955	1084.5997	542.8035	9
5	646.3195	323.6634	629.2930	315.1501	628.3089	314.6581	Q	***974.5517***	487.7795	957.5251	479.2662	956.5411	478.7742	8
6	761.3464	381.1769	744.3199	372.6636	743.3359	372.1716	N	***846.4931***	423.7502	829.4666	415.2369	828.4825	414.7449	7
7	889.4414	445.2243	872.4149	436.7111	871.4308	436.2191	K	***731.4662***	366.2367	714.4396	357.7234	713.4556	357.2314	6
8	1002.5255	501.7664	985.4989	493.2531	984.5149	492.7611	L	***603.3712***	302.1892	586.3447	293.6760	585.3606	293.1840	5
9	1115.6095	558.3084	1098.5830	549.7951	1097.5990	549.3031	L	***490.2871***	245.6472	473.2606	237.1339	472.2766	236.6419	4
10	1244.6521	622.8297	1227.6256	614.3164	1226.6416	613.8244	E	***377.2031***	189.1052	360.1765	180.5919	359.1925	180.0999	3
11	1345.6998	673.3535	1328.6733	664.8403	1327.6892	664.3483	T	***248.1605***	124.5839	231.1339	116.0706	230.1499	115.5786	2
12							K	***147.1128***	74.0600	130.0863	65.5468			1

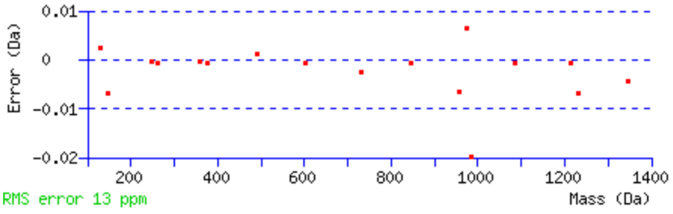

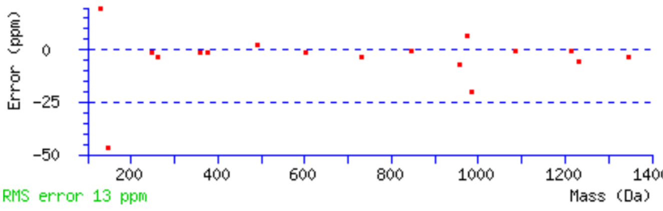

NCBI **BLAST** search of FLEQQNKLLETK
(Parameters: blastp, nr protein database, expect=20000, no filter, PAM30)
Other BLAST web gateways

All matches to this query

Score	Mr(calc):	Delta	Sequence
69.4	1490.7980	0.0003	FLEQQNKLLETK
55.9	1490.7980	0.0003	FLEQQNKLLETK
43.7	1490.7980	0.0003	FLEQQNKLLETK
14.7	1490.7981	0.0003	FQALQSVDILETK
1.6	1488.7909	2.0074	NVHRPPRQRDIT
1.6	1488.7909	2.0074	NVHRPPRQRDIT

Mascot: http://www.matrixscience.com/

Mascot Search Results

Peptide View

MS/MS Fragmentation of **FLEQQNKLLETK**
Found in **sp|O43790|KRT86_HUMAN**, Keratin, type II cuticular Hb6 OS=Homo sapiens GN=KRT86 PE=1 SV=1

Match to Query 17452: 1490.801008 from(746.407780,2+) intensity(6235103.5000) scans(9531) rtinseconds(1792) index(7263)
Title: 160219_Sunil_SDSI_A_Spectrum059371_scans__9531_RTINSECONDS=1792
Data file C:\\Sunil\\TKAP\\T\\T160219_Sunil_SDSI_A.mgf

Click mouse within plot area to zoom in by factor of two about that point
Or, Plot from 100 to 1500 Da Full range
Label all possible matches ○ Label matches used for scoring ◉

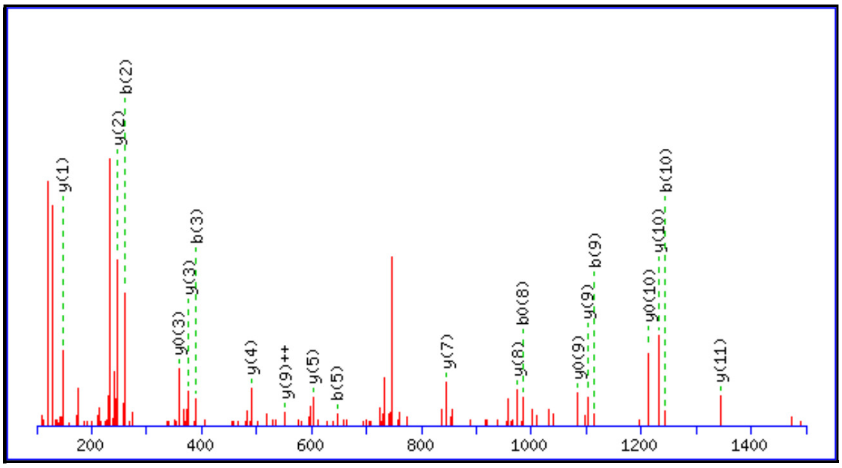

Monoisotopic mass of neutral peptide Mr(calc): 1490.7980
Fixed modifications: Carbamidomethyl (C) (apply to specified residues or termini only)
Variable modifications:
N6 : Deamidated (NQ)
Ions Score: 66 **Expect:** 4.9e-005
Matches : 20/120 fragment ions using 38 most intense peaks (help)

#	b	b^{++}	b^{*}	b^{*++}	b^{0}	b^{0++}	Seq.	y	y^{++}	y^{*}	y^{*++}	y^{0}	y^{0++}	#
1	148.0757	74.5415					F							12
2	**261.1598**	131.0835					L	***1344.7369***	672.8721	1327.7104	664.3588	1326.7264	663.8668	11
3	**390.2023**	195.6048			372.1918	186.5995	E	***1231.6529***	616.3301	1214.6263	607.8168	1213.6423	607.3248	10
4	518.2609	259.6341	501.2344	251.1208	500.2504	250.6288	Q	***1102.6103***	551.8088	1085.5837	543.2955	1084.5997	542.8035	9
5	**646.3195**	323.6634	629.2930	315.1501	628.3089	314.6581	Q	***974.5517***	487.7795	957.5251	479.2662	956.5411	478.7742	8
6	761.3464	381.1769	744.3199	372.6636	743.3359	372.1716	N	***846.4931***	423.7502	829.4666	415.2369	828.4825	414.7449	7
7	889.4414	445.2243	872.4149	436.7111	871.4308	436.2191	K	731.4662	366.2367	714.4396	357.7234	713.4556	357.2314	6
8	1002.5255	501.7664	985.4989	493.2531	984.5149	492.7611	L	***603.3712***	302.1892	586.3447	293.6760	585.3606	293.1840	5
9	**1115.6095**	558.3084	1098.5830	549.7951	1097.5990	549.3031	L	***490.2871***	245.6472	473.2606	237.1339	472.2766	236.6419	4
10	**1244.6521**	622.8297	1227.6256	614.3164	1226.6416	613.8244	E	***377.2031***	189.1052	360.1765	180.5919	359.1925	180.0999	3
11	1345.6998	673.3535	1328.6733	664.8403	1327.6892	664.3483	T	***248.1605***	124.5839	231.1339	116.0706	230.1499	115.5786	2
12							K	***147.1128***	74.0600	130.0863	65.5468			1

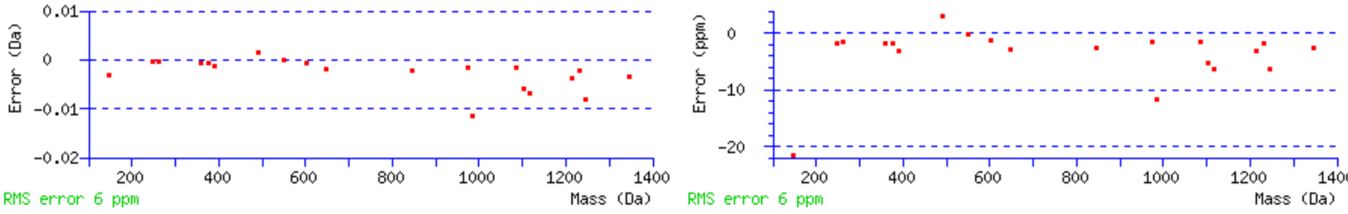

NCBI **BLAST** search of FLEQQNKLLETK
(Parameters: blastp, nr protein database, expect=20000, no filter, PAM30)
Other BLAST web gateways

All matches to this query

Score	Mr(calc):	Delta	Sequence
65.6	1490.7980	0.0030	FLEQQNKLLETK
51.2	1490.7980	0.0030	FLEQQNKLLETK
39.8	1490.7980	0.0030	FLEQQNKLLETK
14.1	1490.7981	0.0030	FQALQSVDILETK

Mascot: http://www.matrixscience.com/

Mascot Search Results

Peptide View

MS/MS Fragmentation of **FLEQQNKLLETK**
Found in **sp|P78385|KRT83_HUMAN**, Keratin, type II cuticular Hb3 OS=Homo sapiens GN=KRT83 PE=1 SV=2

Match to Query 17449: 1490.798352 from(497.940060,3+) intensity(5874160.0000) scans(9629) rtinseconds(1809) index(7352)
Title: 160219_Sunil_SDSI_A_Spectrum059460_scans__9629_RTINSECONDS=1809
Data file C:\\Sunil\\TKAP\\T\\T160219_Sunil_SDSI_A.mgf

Click mouse within plot area to zoom in by factor of two about that point
Or, Plot from 100 to 1400 Da Full range
Label all possible matches Label matches used for scoring

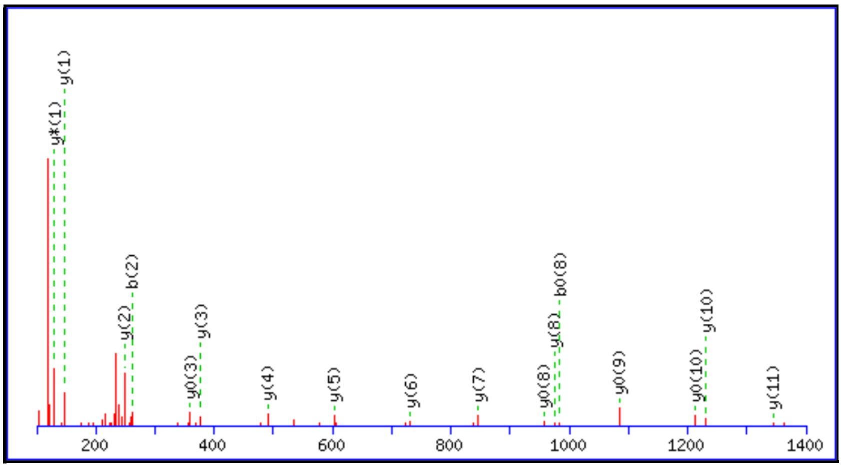

Monoisotopic mass of neutral peptide Mr(calc): 1490.7980
Fixed modifications: Carbamidomethyl (C) (apply to specified residues or termini only)
Variable modifications:
N6 : Deamidated (NQ)
Ions Score: 69 **Expect:** 2.2e-005
Matches : 17/120 fragment ions using 30 most intense peaks (help)

#	b	b^{++}	b*	b^{*++}	b^{0}	b^{0++}	Seq.	y	y^{++}	y*	y^{*++}	y^{0}	y^{0++}	#
1	148.0757	74.5415					F							12
2	**261.1598**	131.0835					L	***1344.7369***	672.8721	1327.7104	664.3588	1326.7264	663.8668	11
3	390.2023	195.6048			372.1918	186.5995	E	***1231.6529***	616.3301	1214.6263	607.8168	1213.6423	607.3248	10
4	518.2609	259.6341	501.2344	251.1208	500.2504	250.6288	Q	1102.6103	551.8088	1085.5837	543.2955	1084.5997	542.8035	9
5	646.3195	323.6634	629.2930	315.1501	628.3089	314.6581	Q	***974.5517***	487.7795	957.5251	479.2662	956.5411	478.7742	8
6	761.3464	381.1769	744.3199	372.6636	743.3359	372.1716	N	***846.4931***	423.7502	829.4666	415.2369	828.4825	414.7449	7
7	889.4414	445.2243	872.4149	436.7111	871.4308	436.2191	K	***731.4662***	366.2367	714.4396	357.7234	713.4556	357.2314	6
8	1002.5255	501.7664	985.4989	493.2531	984.5149	492.7611	L	***603.3712***	302.1892	586.3447	293.6760	585.3606	293.1840	5
9	1115.6095	558.3084	1098.5830	549.7951	1097.5990	549.3031	L	***490.2871***	245.6472	473.2606	237.1339	472.2766	236.6419	4
10	1244.6521	622.8297	1227.6256	614.3164	1226.6416	613.8244	E	***377.2031***	189.1052	360.1765	180.5919	359.1925	180.0999	3
11	1345.6998	673.3535	1328.6733	664.8403	1327.6892	664.3483	T	***248.1605***	124.5839	231.1339	116.0706	230.1499	115.5786	2
12							K	***147.1128***	74.0600	130.0863	65.5468			1

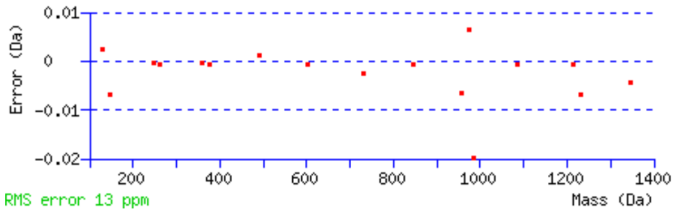

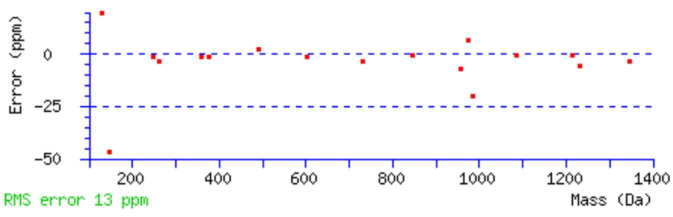

NCBI **BLAST** search of FLEQQNKLLETK
(Parameters: blastp, nr protein database, expect=20000, no filter, PAM30)
Other BLAST web gateways

All matches to this query

Score	Mr(calc):	Delta	Sequence
69.4	1490.7980	0.0003	FLEQQNKLLETK
55.9	1490.7980	0.0003	FLEQQNKLLETK
43.7	1490.7980	0.0003	FLEQQNKLLETK
14.7	1490.7981	0.0003	FQALQSVDILETK
1.6	1488.7909	2.0074	NVHRPPRQRDIT
1.6	1488.7909	2.0074	NVHRPPRQRDIT

Mascot: http://www.matrixscience.com/

Mascot Search Results

Peptide View

MS/MS Fragmentation of **ILCSKSENARLVVQIDNAK**
Found in **sp|O76009|KT33A_HUMAN**, Keratin, type I cuticular Ha3-I OS=Homo sapiens GN=KRT33A PE=2 SV=2

Match to Query 31774: 2158.138512 from(720.386780,3+) intensity(1857554.3750) scans(9342) rtinseconds(1735) index(22489)
Title: 160219_Sunil_SDSI_A_Spectrum075793_scans__9342_RTINSECONDS=1735
Data file C:\\Sunil\\TKAP\\T\\T160219_Sunil_SDSI_A.mgf

Click mouse within plot area to zoom in by factor of two about that point
Or, Plot from 0 to 2200 Da Full range
Label all possible matches ○ Label matches used for scoring ◉

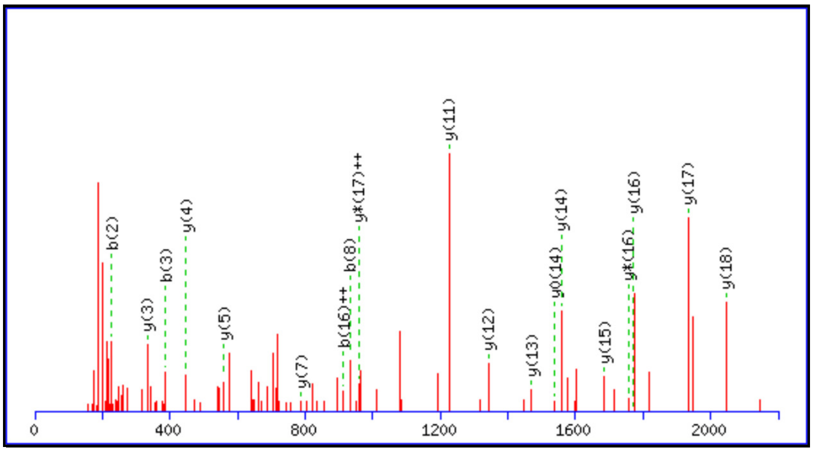

Monoisotopic mass of neutral peptide Mr(calc): 2158.1416
Fixed modifications: Carbamidomethyl (C) (apply to specified residues or termini only)
Variable modifications:
N8 : Deamidated (NQ)
Ions Score: 58 **Expect:** 0.00034
Matches : 19/196 fragment ions using 49 most intense peaks (help)

#	b	b^{++}	b^{*}	b^{*++}	b^{0}	b^{0++}	Seq.	y	y^{++}	y^{*}	y^{*++}	y^{0}	y^{0++}	#
1	114.0913	57.5493					I							19
2	**227.1754**	114.0913					L	***2046.0648***	1023.5360	2029.0383	1015.0228	2028.0542	1014.5308	18
3	**387.2061**	194.1067					C	***1932.9808***	966.9940	1915.9542	958.4807	1914.9702	957.9887	17
4	474.2381	237.6227			456.2275	228.6174	S	***1772.9501***	886.9787	1755.9236	878.4654	1754.9395	877.9734	16
5	602.3330	301.6702	585.3065	293.1569	584.3225	292.6649	K	***1685.9181***	843.4627	1668.8915	834.9494	1667.9075	834.4574	15
6	689.3651	345.1862	672.3385	336.6729	671.3545	336.1809	S	***1557.8231***	779.4152	1540.7966	770.9019	1539.8125	770.4099	14
7	818.4077	409.7075	801.3811	401.1942	800.3971	400.7022	E	***1470.7911***	735.8992	1453.7645	727.3859	1452.7805	726.8939	13
8	**933.4346**	467.2209	916.4081	458.7077	915.4240	458.2157	N	***1341.7485***	671.3779	1324.7219	662.8646	1323.7379	662.3726	12
9	1004.4717	502.7395	987.4452	494.2262	986.4612	493.7342	A	***1226.7215***	613.8644	1209.6950	605.3511	1208.7110	604.8591	11
10	1160.5728	580.7901	1143.5463	572.2768	1142.5623	571.7848	R	1155.6844	578.3459	1138.6579	569.8326	1137.6739	569.3406	10
11	1273.6569	637.3321	1256.6303	628.8188	1255.6463	628.3268	L	999.5833	500.2953	982.5568	491.7820	981.5728	491.2900	9
12	1372.7253	686.8663	1355.6988	678.3530	1354.7147	677.8610	V	886.4993	443.7533	869.4727	435.2400	868.4887	434.7480	8
13	1471.7937	736.4005	1454.7672	727.8872	1453.7832	727.3952	V	***787.4308***	394.2191	770.4043	385.7058	769.4203	385.2138	7
14	1599.8523	800.4298	1582.8258	791.9165	1581.8417	791.4245	Q	688.3624	344.6849	671.3359	336.1716	670.3519	335.6796	6
15	1712.9364	856.9718	1695.9098	848.4585	1694.9258	847.9665	I	***560.3039***	280.6556	543.2773	272.1423	542.2933	271.6503	5
16	1827.9633	**914.4853**	1810.9368	905.9720	1809.9527	905.4800	D	***447.2198***	224.1135	430.1932	215.6003	429.2092	215.1082	4
17	1942.0062	971.5068	1924.9797	962.9935	1923.9957	962.5015	N	***332.1928***	166.6001	315.1663	158.0868			3
18	2013.0434	1007.0253	1996.0168	998.5120	1995.0328	998.0200	A	218.1499	109.5786	201.1234	101.0653			2
19							K	147.1128	74.0600	130.0863	65.5468			1

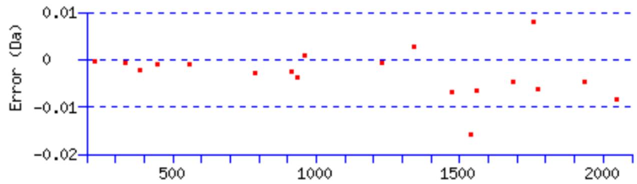

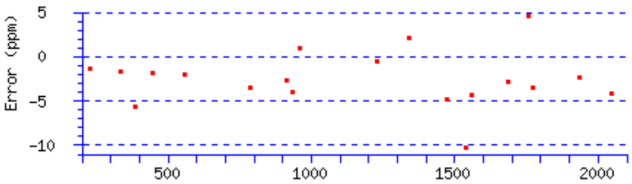

NCBI **BLAST** search of ILCSKSENARLVVQIDNAK
(Parameters: blastp, nr protein database, expect=20000, no filter, PAM30)
Other BLAST web gateways

All matches to this query

Score	Mr(calc):	Delta	Sequence
57.5	2158.1416	-0.0030	ILCSKSENARLVVQIDNAK
38.3	2158.1416	-0.0030	ILCSKSENARLVVQIDNAK
27.6	2158.1416	-0.0030	ILCSKSENARLVVQIDNAK
11.0	2158.1303	0.0082	ILCSKAENARLIVQIDNAK
6.0	2158.1310	0.0075	LTEQLDTYILKNFVAFSR

Mascot: http://www.matrixscience.com/

Mascot Search Results

Peptide View

MS/MS Fragmentation of **ILCTKSENARLVVQIDNAK**
Found in **sp|Q15323|K1H1_HUMAN**, Keratin, type I cuticular Ha1 OS=Homo sapiens GN=KRT31 PE=2 SV=3

Match to Query 32019: 2172.155052 from(725.058960,3+) intensity(2027060.7500) scans(9660) rtinseconds(1789) index(22777)
Title: 160219_Sunil_SDSI_A_Spectrum076081_scans__9660_RTINSECONDS=1789
Data file C:\\Sunil\\TKAP\\T\\T160219_Sunil_SDSI_A.mgf

Click mouse within plot area to zoom in by factor of two about that point
Or, Plot from 0 to 2200 Da Full range
Label all possible matches ○ Label matches used for scoring ◉

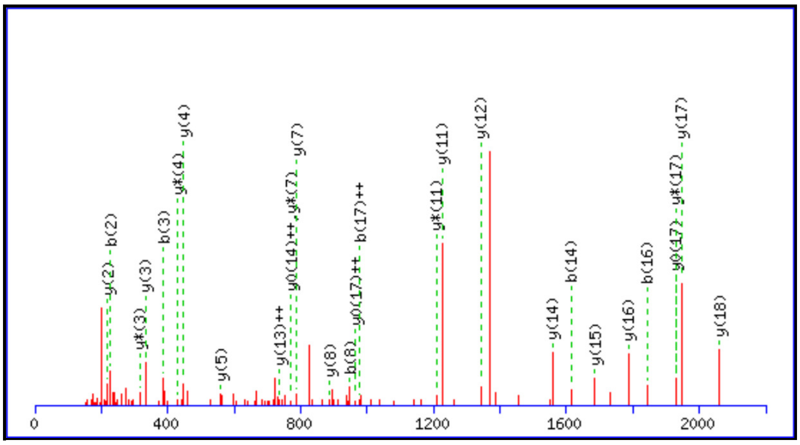

Monoisotopic mass of neutral peptide Mr(calc): 2172.1572
Fixed modifications: Carbamidomethyl (C) (apply to specified residues or termini only)
Variable modifications:
N8 : Deamidated (NQ)
Ions Score: 64 **Expect:** 7.9e-005
Matches : 28/196 fragment ions using 66 most intense peaks (help)

#	b	b^{++}	b*	b^{*++}	b^{0}	b^{0++}	Seq.	y	y^{++}	y*	y^{*++}	y^{0}	y^{0++}	#
1	114.0913	57.5493					I							19
2	227.1754	114.0913					L	2060.0805	1030.5439	2043.0539	1022.0306	2042.0699	1021.5386	18
3	387.2061	194.1067					C	1946.9964	974.0018	1929.9699	965.4886	1928.9858	964.9966	17
4	488.2537	244.6305			470.2432	235.6252	T	1786.9658	893.9865	1769.9392	885.4732	1768.9552	884.9812	16
5	616.3487	308.6780	599.3221	300.1647	598.3381	299.6727	K	1685.9181	843.4627	1668.8915	834.9494	1667.9075	834.4574	15
6	703.3807	352.1940	686.3542	343.6807	685.3702	343.1887	S	1557.8231	779.4152	1540.7966	770.9019	1539.8125	770.4099	14
7	832.4233	416.7153	815.3968	408.2020	814.4128	407.7100	E	1470.7911	735.8992	1453.7645	727.3859	1452.7805	726.8939	13
8	947.4503	474.2288	930.4237	465.7155	929.4397	465.2235	N	1341.7485	671.3779	1324.7219	662.8646	1323.7379	662.3726	12
9	1018.4874	509.7473	1001.4608	501.2341	1000.4768	500.7420	A	1226.7215	613.8644	1209.6950	605.3511	1208.7110	604.8591	11
10	1174.5885	587.7979	1157.5619	579.2846	1156.5779	578.7926	R	1155.6844	578.3459	1138.6579	569.8326	1137.6739	569.3406	10
11	1287.6725	644.3399	1270.6460	635.8266	1269.6620	635.3346	L	999.5833	500.2953	982.5568	491.7820	981.5728	491.2900	9
12	1386.7410	693.8741	1369.7144	685.3608	1368.7304	684.8688	V	886.4993	443.7533	869.4727	435.2400	868.4887	434.7480	8
13	1485.8094	743.4083	1468.7828	734.8951	1467.7988	734.4030	V	787.4308	394.2191	770.4043	385.7058	769.4203	385.2138	7
14	1613.8680	807.4376	1596.8414	798.9243	1595.8574	798.4323	Q	688.3624	344.6849	671.3359	336.1716	670.3519	335.6796	6
15	1726.9520	863.9796	1709.9255	855.4664	1708.9415	854.9744	I	560.3039	280.6556	543.2773	272.1423	542.2933	271.6503	5
16	1841.9790	921.4931	1824.9524	912.9798	1823.9684	912.4878	D	447.2198	224.1135	430.1932	215.6003	429.2092	215.1082	4
17	1956.0219	978.5146	1938.9953	970.0013	1938.0113	969.5093	N	332.1928	166.6001	315.1663	158.0868			3
18	2027.0590	1014.0331	2010.0325	1005.5199	2009.0484	1005.0279	A	218.1499	109.5786	201.1234	101.0653			2
19							K	147.1128	74.0600	130.0863	65.5468			1

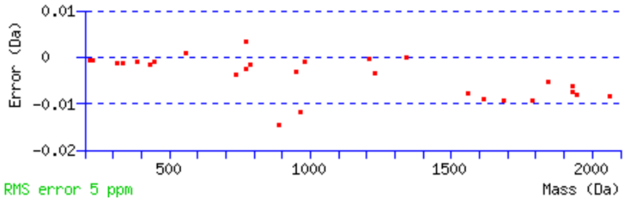

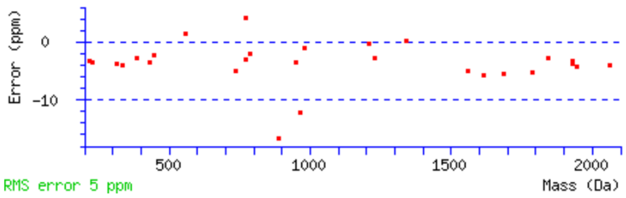

NCBI **BLAST** search of ILCTKSENARLVVQIDNAK

(Parameters: blastp, nr protein database, expect=20000, no filter, PAM30)
Other BLAST web gateways

All matches to this query

Score	Mr(calc):	Delta	Sequence
63.6	2172.1572	-0.0022	ILCTKSENARLVVQIDNAK
40.4	2172.1572	-0.0022	ILCTKSENARLVVQIDNAK
19.9	2172.1572	-0.0022	ILCTKSENARLVVQIDNAK
8.1	2171.1732	0.9818	ILCTKSENARLVVQIDNAK

Mascot: http://www.matrixscience.com/

Mascot Search Results

Peptide View

MS/MS Fragmentation of **ILCTKSENARLVVQIDNAK**
Found in **sp|Q15323|K1H1_HUMAN**, Keratin, type I cuticular Ha1 OS=Homo sapiens GN=KRT31 PE=2 SV=3

Match to Query 32022: 2172.157176 from(544.046570,4+) intensity(4691859.5000) scans(9641) rtinseconds(1786) index(22760)
Title: 160219_Sunil_SDSI_A_Spectrum076064_scans__9641_RTINSECONDS=1786
Data file C:\\Sunil\\TKAP\\T\\T160219_Sunil_SDSI_A.mgf

Click mouse within plot area to zoom in by factor of two about that point
Or, Plot from 0 to 2200 Da Full range
Label all possible matches Label matches used for scoring

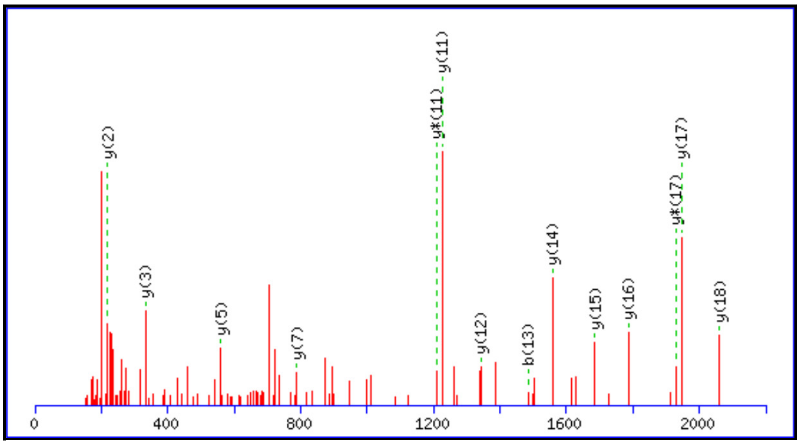

Monoisotopic mass of neutral peptide Mr(calc): 2172.1572
Fixed modifications: Carbamidomethyl (C) (apply to specified residues or termini only)
Variable modifications:
N8 : Deamidated (NQ)
Ions Score: 55 **Expect:** 0.00051
Matches : 14/196 fragment ions using 35 most intense peaks (help)

#	b	b++	b*	b*++	b0	b0++	Seq.	y	y++	y*	y*++	y0	y0++	#
1	114.0913	57.5493					I							19
2	227.1754	114.0913					L	2060.0805	1030.5439	2043.0539	1022.0306	2042.0699	1021.5386	18
3	387.2061	194.1067					C	1946.9964	974.0018	1929.9699	965.4886	1928.9858	964.9966	17
4	488.2537	244.6305			470.2432	235.6252	T	1786.9658	893.9865	1769.9392	885.4732	1768.9552	884.9812	16
5	616.3487	308.6780	599.3221	300.1647	598.3381	299.6727	K	1685.9181	843.4627	1668.8915	834.9494	1667.9075	834.4574	15
6	703.3807	352.1940	686.3542	343.6807	685.3702	343.1887	S	1557.8231	779.4152	1540.7966	770.9019	1539.8125	770.4099	14
7	832.4233	416.7153	815.3968	408.2020	814.4128	407.7100	E	1470.7911	735.8992	1453.7645	727.3859	1452.7805	726.8939	13
8	947.4503	474.2288	930.4237	465.7155	929.4397	465.2235	N	1341.7485	671.3779	1324.7219	662.8646	1323.7379	662.3726	12
9	1018.4874	509.7473	1001.4608	501.2341	1000.4768	500.7420	A	1226.7215	613.8644	1209.6950	605.3511	1208.7110	604.8591	11
10	1174.5885	587.7979	1157.5619	579.2846	1156.5779	578.7926	R	1155.6844	578.3459	1138.6579	569.8326	1137.6739	569.3406	10
11	1287.6725	644.3399	1270.6460	635.8266	1269.6620	635.3346	L	999.5833	500.2953	982.5568	491.7820	981.5728	491.2900	9
12	1386.7410	693.8741	1369.7144	685.3608	1368.7304	684.8688	V	886.4993	443.7533	869.4727	435.2400	868.4887	434.7480	8
13	1485.8094	743.4083	1468.7828	734.8951	1467.7988	734.4030	V	787.4308	394.2191	770.4043	385.7058	769.4203	385.2138	7
14	1613.8680	807.4376	1596.8414	798.9243	1595.8574	798.4323	Q	688.3624	344.6849	671.3359	336.1716	670.3519	335.6796	6
15	1726.9520	863.9796	1709.9255	855.4664	1708.9415	854.9744	I	560.3039	280.6556	543.2773	272.1423	542.2933	271.6503	5
16	1841.9790	921.4931	1824.9524	912.9798	1823.9684	912.4878	D	447.2198	224.1135	430.1932	215.6003	429.2092	215.1082	4
17	1956.0219	978.5146	1938.9953	970.0013	1938.0113	969.5093	N	332.1928	166.6001	315.1663	158.0868			3
18	2027.0590	1014.0331	2010.0325	1005.5199	2009.0484	1005.0279	A	218.1499	109.5786	201.1234	101.0653			2
19							K	147.1128	74.0600	130.0863	65.5468			1

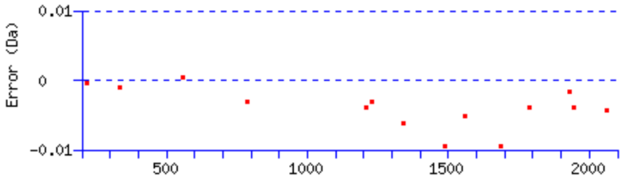

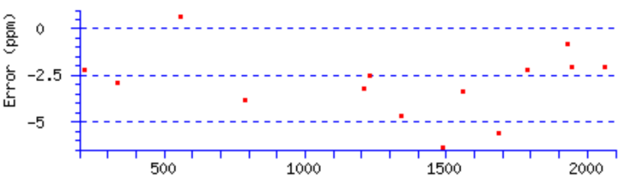

NCBI **BLAST** search of ILCTKSENARLVVQIDNAK

(Parameters: blastp, nr protein database, expect=20000, no filter, PAM30)
Other BLAST web gateways

All matches to this query

Score	Mr(calc):	Delta	Sequence
55.4	2172.1572	-0.0000	ILCTKSENARLVVQIDNAK
31.8	2172.1572	-0.0000	ILCTKSENARLVVQIDNAK
15.6	2172.1572	-0.0000	ILCTKSENARLVVQIDNAK
1.5	2171.1732	0.9840	ILCTKSENARLVVQIDNAK

Mascot: http://www.matrixscience.com/

Mascot Search Results

Peptide View

MS/MS Fragmentation of **ILQSHISDTSVVVKLDNSR**
Found in **sp|P78385|KRT83_HUMAN**, Keratin, type II cuticular Hb3 OS=Homo sapiens GN=KRT83 PE=1 SV=2

Match to Query 30992: 2111.125452 from(704.715760,3+) intensity(299957.9375) scans(10624) rtinseconds(1980) index(8207)
Title: 160219_Sunil_SDSI_A_Spectrum060317_scans__10624_RTINSECONDS=1980
Data file C:\\Sunil\\TKAP\\T\\T160219_Sunil_SDSI_A.mgf

Click mouse within plot area to zoom in by factor of two about that point
Or, Plot from 100 to 1200 Da Full range
Label all possible matches ○ Label matches used for scoring ◉

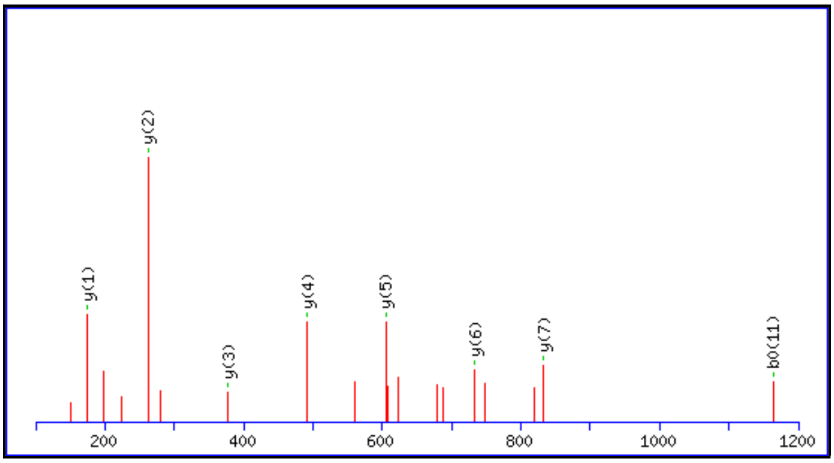

Monoisotopic mass of neutral peptide Mr(calc): 2111.1222
Fixed modifications: Carbamidomethyl (C) (apply to specified residues or termini only)
Variable modifications:
N17 : Deamidated (NQ)
Ions Score: 60 **Expect:** 0.00017
Matches : 8/204 fragment ions using 8 most intense peaks (help)

#	b	b^{++}	b^{*}	b^{*++}	b^{0}	b^{0++}	Seq.	y	y^{++}	y^{*}	y^{*++}	y^{0}	y^{0++}	#
1	114.0913	57.5493					I							19
2	227.1754	114.0913					L	1999.0455	1000.0264	1982.0189	991.5131	1981.0349	991.0211	18
3	355.2340	178.1206	338.2074	169.6074			Q	1885.9614	943.4843	1868.9348	934.9711	1867.9508	934.4791	17
4	442.2660	221.6366	425.2395	213.1234	424.2554	212.6314	S	1757.9028	879.4550	1740.8763	870.9418	1739.8923	870.4498	16
5	579.3249	290.1661	562.2984	281.6528	561.3144	281.1608	H	1670.8708	835.9390	1653.8442	827.4258	1652.8602	826.9338	15
6	692.4090	346.7081	675.3824	338.1949	674.3984	337.7028	I	1533.8119	767.4096	1516.7853	758.8963	1515.8013	758.4043	14
7	779.4410	390.2241	762.4145	381.7109	761.4304	381.2189	S	1420.7278	710.8675	1403.7013	702.3543	1402.7172	701.8623	13
8	894.4680	447.7376	877.4414	439.2243	876.4574	438.7323	D	1333.6958	667.3515	1316.6692	658.8383	1315.6852	658.3462	12
9	995.5156	498.2615	978.4891	489.7482	977.5051	489.2562	T	1218.6688	609.8381	1201.6423	601.3248	1200.6583	600.8328	11
10	1082.5477	541.7775	1065.5211	533.2642	1064.5371	532.7722	S	1117.6212	559.3142	1100.5946	550.8009	1099.6106	550.3089	10
11	1181.6161	591.3117	1164.5895	582.7984	1163.6055	582.3064	V	1030.5891	515.7982	1013.5626	507.2849	1012.5786	506.7929	9
12	1280.6845	640.8459	1263.6579	632.3326	1262.6739	631.8406	V	931.5207	466.2640	914.4942	457.7507	913.5102	457.2587	8
13	1379.7529	690.3801	1362.7264	681.8668	1361.7423	681.3748	V	***832.4523***	416.7298	815.4258	408.2165	814.4417	407.7245	7
14	1507.8479	754.4276	1490.8213	745.9143	1489.8373	745.4223	K	***733.3839***	367.1956	716.3573	358.6823	715.3733	358.1903	6
15	1620.9319	810.9696	1603.9054	802.4563	1602.9214	801.9643	L	***605.2889***	303.1481	588.2624	294.6348	587.2784	294.1428	5
16	1735.9589	868.4831	1718.9323	859.9698	1717.9483	859.4778	D	***492.2049***	246.6061	475.1783	238.0928	474.1943	237.6008	4
17	1850.9858	925.9965	1833.9593	917.4833	1832.9753	916.9913	N	***377.1779***	189.0926	360.1514	180.5793	359.1674	180.0873	3
18	1938.0178	969.5126	1920.9913	960.9993	1920.0073	960.5073	S	***262.1510***	131.5791	245.1244	123.0659	244.1404	122.5738	2
19							R	***175.1190***	88.0631	158.0924	79.5498			1

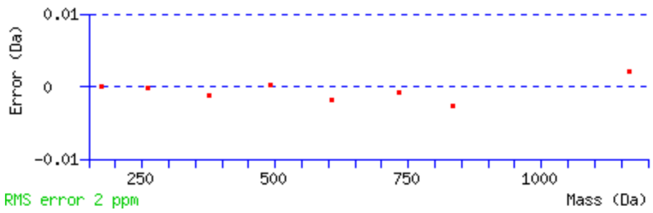

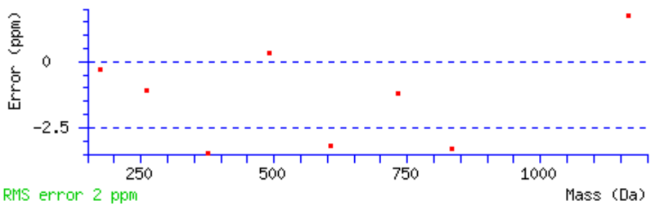

NCBI **BLAST** search of ILQSHISDTSVVVKLDNSR
(Parameters: blastp, nr protein database, expect=20000, no filter, PAM30)
Other BLAST web gateways

All matches to this query

Score	Mr(calc):	Delta	Sequence
59.5	2111.1222	0.0032	ILQSHISDTSVVVKLDNSR
6.5	2110.1355	0.9900	QTENVSRTRKPLARPNSR
6.5	2111.1195	0.0059	QTENVSRTRKPLARPNSR
6.5	2111.1195	0.0059	QTENVSRTRKPLARPNSR
4.7	2111.1044	0.0210	XEKRFADAASLLLSLMTSR
4.7	2111.1375	-0.0120	SSIRIPGPPETLQIFDVSR
4.5	2110.1139	1.0115	MGMRIKLQSTNHPNNLLK
4.5	2110.1139	1.0115	MGMRIKLQSTNHPNNLLK
3.3	2110.1382	0.9872	ILQSHISDTSVVVKLDNSR
3.3	2111.1222	0.0032	ILQSHISDTSVVVKLDNSR

Mascot: http://www.matrixscience.com/

Mascot Search Results

Peptide View

MS/MS Fragmentation of **LASELNHVQEVLEGYK**
Found in **sp|O43790|KRT86_HUMAN**, Keratin, type II cuticular Hb6 OS=Homo sapiens GN=KRT86 PE=1 SV=1

Match to Query 25222: 1828.918308 from(915.466430,2+) intensity(759231.3125) scans(13040) rtinseconds(2395) index(10217)
Title: 160219_Sunil_SDSI_A_Spectrum062338_scans__13040_RTINSECONDS=2395
Data file C:\\Sunil\\TKAP\\T\\T160219_Sunil_SDSI_A.mgf

Click mouse within plot area to zoom in by factor of two about that point
Or, Plot from 100 to 1700 Da Full range
Label all possible matches ○ Label matches used for scoring ◉

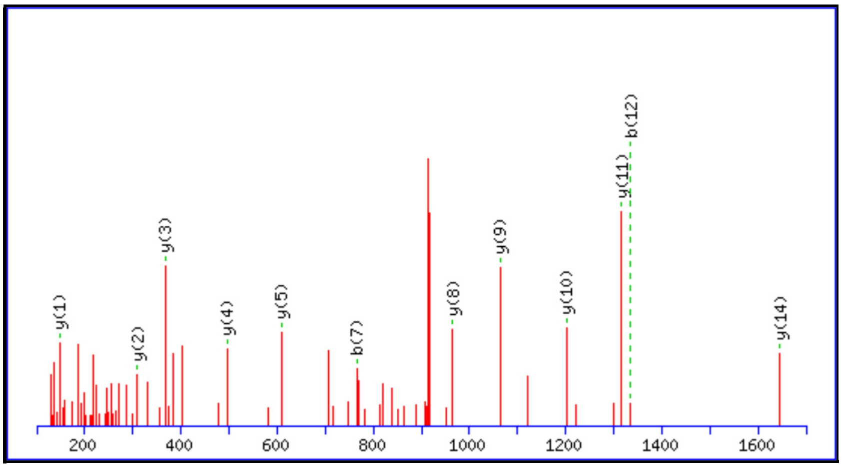

Monoisotopic mass of neutral peptide Mr(calc): 1828.9207
Fixed modifications: Carbamidomethyl (C) (apply to specified residues or termini only)
Variable modifications:
N6 : Deamidated (NQ)
Ions Score: 93 **Expect:** 1.2e-007
Matches : 12/160 fragment ions using 14 most intense peaks (help)

#	b	b^{++}	b*	b^{*++}	b^0	b^{0++}	Seq.	y	y^{++}	y*	y^{*++}	y^0	y^{0++}	#
1	114.0913	57.5493					L							16
2	185.1285	93.0679					A	1716.8439	858.9256	1699.8174	850.4123	1698.8333	849.9203	15
3	272.1605	136.5839			254.1499	127.5786	S	***1645.8068***	823.4070	1628.7802	814.8938	1627.7962	814.4018	14
4	401.2031	201.1052			383.1925	192.0999	E	1558.7748	779.8910	1541.7482	771.3777	1540.7642	770.8857	13
5	514.2871	257.6472			496.2766	248.6419	L	1429.7322	715.3697	1412.7056	706.8564	1411.7216	706.3644	12
6	629.3141	315.1607	612.2875	306.6474	611.3035	306.1554	N	***1316.6481***	658.8277	1299.6216	650.3144	1298.6375	649.8224	11
7	**766.3730**	383.6901	749.3464	375.1769	748.3624	374.6849	H	***1201.6212***	601.3142	1184.5946	592.8009	1183.6106	592.3089	10
8	865.4414	433.2243	848.4149	424.7111	847.4308	424.2191	V	***1064.5623***	532.7848	1047.5357	524.2715	1046.5517	523.7795	9
9	993.5000	497.2536	976.4734	488.7404	975.4894	488.2483	Q	***965.4938***	483.2506	948.4673	474.7373	947.4833	474.2453	8
10	1122.5426	561.7749	1105.5160	553.2617	1104.5320	552.7696	E	837.4353	419.2213	820.4087	410.7080	819.4247	410.2160	7
11	1221.6110	611.3091	1204.5844	602.7959	1203.6004	602.3039	V	708.3927	354.7000	691.3661	346.1867	690.3821	345.6947	6
12	**1334.6951**	667.8512	1317.6685	659.3379	1316.6845	658.8459	L	***609.3243***	305.1658	592.2977	296.6525	591.3137	296.1605	5
13	1463.7376	732.3725	1446.7111	723.8592	1445.7271	723.3672	E	***496.2402***	248.6237	479.2136	240.1105	478.2296	239.6185	4
14	1520.7591	760.8832	1503.7326	752.3699	1502.7485	751.8779	G	***367.1976***	184.1024	350.1710	175.5892			3
15	1683.8224	842.4149	1666.7959	833.9016	1665.8119	833.4096	Y	***310.1761***	155.5917	293.1496	147.0784			2
16							K	***147.1128***	74.0600	130.0863	65.5468			1

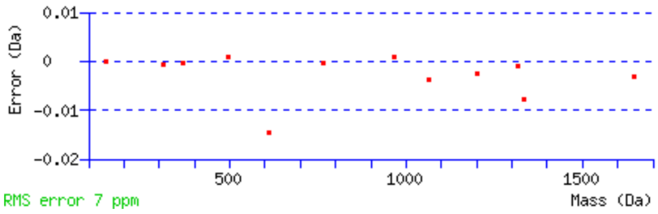

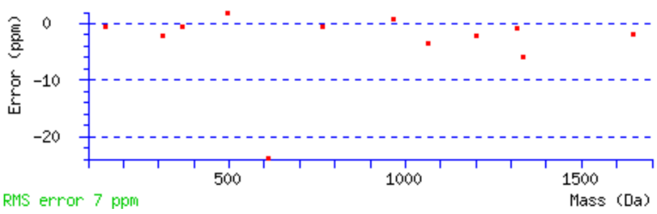

NCBI **BLAST** search of LASELNHVQEVLEGYK

(Parameters: blastp, nr protein database, expect=20000, no filter, PAM30)
Other BLAST web gateways

All matches to this query

Score	Mr(calc):	Delta	Sequence
93.0	1828.9207	-0.0023	LASELNHVQEVLEGYK
48.8	1828.9207	-0.0023	LASELNHVQEVLEGYK
5.9	1826.9170	2.0013	XARGQGGGPQGVGRMAEGK

Mascot: http://www.matrixscience.com/

Mascot Search Results

Peptide View

MS/MS Fragmentation of **LASELNHVQEVLEGYK**
Found in **sp|P78385|KRT83_HUMAN**, Keratin, type II cuticular Hb3 OS=Homo sapiens GN=KRT83 PE=1 SV=2

Match to Query 25222: 1828.918308 from(915.466430,2+) intensity(759231.3125) scans(13040) rtinseconds(2395) index(10217)
Title: 160219_Sunil_SDSI_A_Spectrum062338_scans__13040_RTINSECONDS=2395
Data file C:\\Sunil\\TKAP\\T\\T160219_Sunil_SDSI_A.mgf

Click mouse within plot area to zoom in by factor of two about that point
Or, Plot from 100 to 1700 Da Full range
Label all possible matches ○ Label matches used for scoring ◉

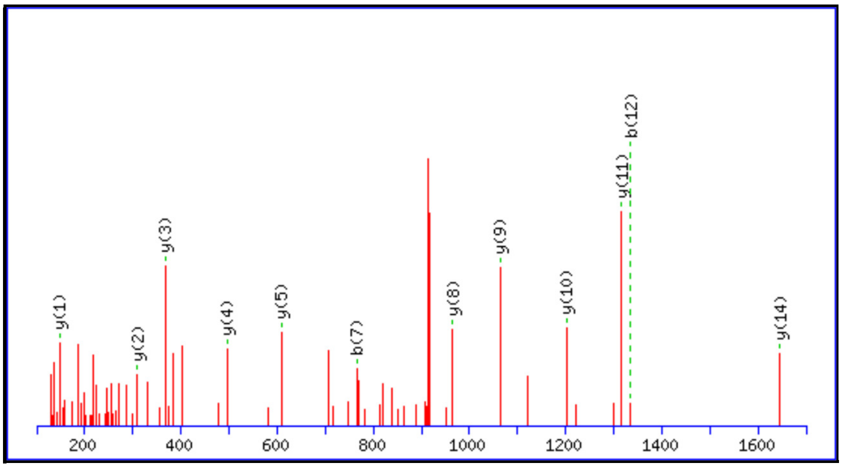

Monoisotopic mass of neutral peptide Mr(calc): 1828.9207
Fixed modifications: Carbamidomethyl (C) (apply to specified residues or termini only)
Variable modifications:
N6 : Deamidated (NQ)
Ions Score: 93 **Expect:** 1.2e-007
Matches : 12/160 fragment ions using 14 most intense peaks (help)

#	b	b^{++}	b*	b^{*++}	b^0	b^{0++}	Seq.	y	y^{++}	y*	y^{*++}	y^0	y^{0++}	#
1	114.0913	57.5493					L							16
2	185.1285	93.0679					A	1716.8439	858.9256	1699.8174	850.4123	1698.8333	849.9203	15
3	272.1605	136.5839			254.1499	127.5786	S	***1645.8068***	823.4070	1628.7802	814.8938	1627.7962	814.4018	14
4	401.2031	201.1052			383.1925	192.0999	E	1558.7748	779.8910	1541.7482	771.3777	1540.7642	770.8857	13
5	514.2871	257.6472			496.2766	248.6419	L	1429.7322	715.3697	1412.7056	706.8564	1411.7216	706.3644	12
6	629.3141	315.1607	612.2875	306.6474	611.3035	306.1554	N	***1316.6481***	658.8277	1299.6216	650.3144	1298.6375	649.8224	11
7	**766.3730**	383.6901	749.3464	375.1769	748.3624	374.6849	H	***1201.6212***	601.3142	1184.5946	592.8009	1183.6106	592.3089	10
8	865.4414	433.2243	848.4149	424.7111	847.4308	424.2191	V	***1064.5623***	532.7848	1047.5357	524.2715	1046.5517	523.7795	9
9	993.5000	497.2536	976.4734	488.7404	975.4894	488.2483	Q	***965.4938***	483.2506	948.4673	474.7373	947.4833	474.2453	8
10	1122.5426	561.7749	1105.5160	553.2617	1104.5320	552.7696	E	837.4353	419.2213	820.4087	410.7080	819.4247	410.2160	7
11	1221.6110	611.3091	1204.5844	602.7959	1203.6004	602.3039	V	708.3927	354.7000	691.3661	346.1867	690.3821	345.6947	6
12	**1334.6951**	667.8512	1317.6685	659.3379	1316.6845	658.8459	L	***609.3243***	305.1658	592.2977	296.6525	591.3137	296.1605	5
13	1463.7376	732.3725	1446.7111	723.8592	1445.7271	723.3672	E	***496.2402***	248.6237	479.2136	240.1105	478.2296	239.6185	4
14	1520.7591	760.8832	1503.7326	752.3699	1502.7485	751.8779	G	***367.1976***	184.1024	350.1710	175.5892			3
15	1683.8224	842.4149	1666.7959	833.9016	1665.8119	833.4096	Y	***310.1761***	155.5917	293.1496	147.0784			2
16							K	***147.1128***	74.0600	130.0863	65.5468			1

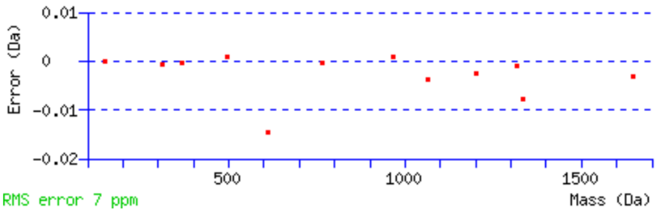

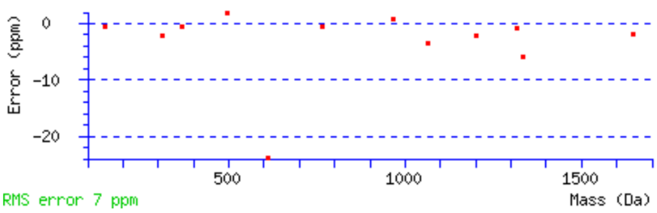

NCBI **BLAST** search of LASELNHVQEVLEGYK

(Parameters: blastp, nr protein database, expect=20000, no filter, PAM30)
Other BLAST web gateways

All matches to this query

Score	Mr(calc):	Delta	Sequence
93.0	1828.9207	-0.0023	LASELNHVQEVLEGYK
48.8	1828.9207	-0.0023	LASELNHVQEVLEGYK
5.9	1826.9170	2.0013	XARGQGGGPQGVGRMAEGK

Mascot: http://www.matrixscience.com/

Mascot Search Results

Peptide View

MS/MS Fragmentation of **LASELNHVQEVLEGYKK**
Found in **sp|O43790|KRT86_HUMAN**, Keratin, type II cuticular Hb6 OS=Homo sapiens GN=KRT86 PE=1 SV=1

Match to Query 27751: 1957.014856 from(490.260990,4+) intensity(1760902.8750) scans(10291) rtinseconds(1922) index(38551)
Title: 160219_Sunil_SDSI_A_Spectrum093136_scans__10291_RTINSECONDS=1922
Data file C:\\Sunil\\TKAP\\T\\T160219_Sunil_SDSI_A.mgf

Click mouse within plot area to zoom in by factor of two about that point
Or, Plot from 100 to 1800 Da Full range
Label all possible matches ○ Label matches used for scoring ●

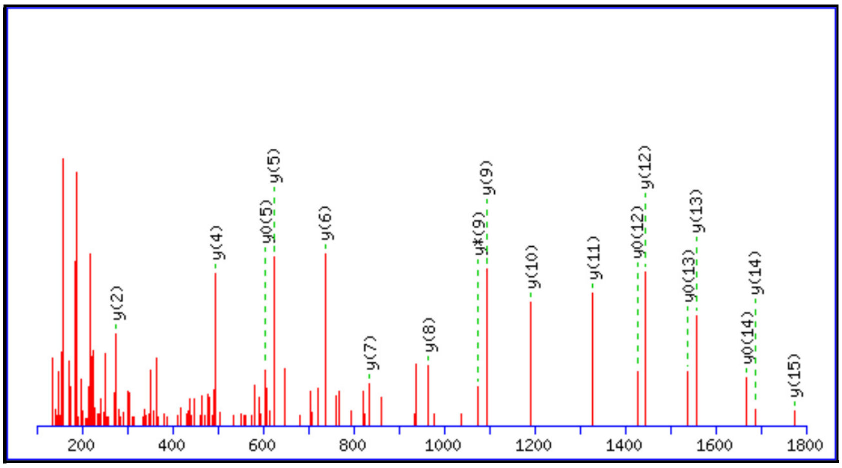

Monoisotopic mass of neutral peptide Mr(calc): 1957.0156
Fixed modifications: Carbamidomethyl (C) (apply to specified residues or termini only)
Variable modifications:
N6 : Deamidated (NQ)
Ions Score: 102 **Expect:** 1.4e-008
Matches : 18/170 fragment ions using 29 most intense peaks (help)

#	b	b++	b*	b*++	b0	b0++	Seq.	y	y++	y*	y*++	y0	y0++	#
1	114.0913	57.5493					L							17
2	185.1285	93.0679					A	1844.9389	922.9731	1827.9123	914.4598	1826.9283	913.9678	16
3	272.1605	136.5839			254.1499	127.5786	S	***1773.9018***	887.4545	1756.8752	878.9412	1755.8912	878.4492	15
4	401.2031	201.1052			383.1925	192.0999	E	***1686.8697***	843.9385	1669.8432	835.4252	1668.8592	834.9332	14
5	514.2871	257.6472			496.2766	248.6419	L	***1557.8271***	779.4172	1540.8006	770.9039	1539.8166	770.4119	13
6	629.3141	315.1607	612.2875	306.6474	611.3035	306.1554	N	***1444.7431***	722.8752	1427.7165	714.3619	1426.7325	713.8699	12
7	766.3730	383.6901	749.3464	375.1769	748.3624	374.6849	H	***1329.7161***	665.3617	1312.6896	656.8484	1311.7056	656.3564	11
8	865.4414	433.2243	848.4149	424.7111	847.4308	424.2191	V	***1192.6572***	596.8322	1175.6307	588.3190	1174.6467	587.8270	10
9	993.5000	497.2536	976.4734	488.7404	975.4894	488.2483	Q	***1093.5888***	547.2980	1076.5623	538.7848	1075.5782	538.2928	9
10	1122.5426	561.7749	1105.5160	553.2617	1104.5320	552.7696	E	***965.5302***	483.2688	948.5037	474.7555	947.5197	474.2635	8
11	1221.6110	611.3091	1204.5844	602.7959	1203.6004	602.3039	V	***836.4876***	418.7475	819.4611	410.2342	818.4771	409.7422	7
12	1334.6951	667.8512	1317.6685	659.3379	1316.6845	658.8459	L	***737.4192***	369.2132	720.3927	360.7000	719.4087	360.2080	6
13	1463.7376	732.3725	1446.7111	723.8592	1445.7271	723.3672	E	***624.3352***	312.6712	607.3086	304.1579	606.3246	303.6659	5
14	1520.7591	760.8832	1503.7326	752.3699	1502.7485	751.8779	G	***495.2926***	248.1499	478.2660	239.6366			4
15	1683.8224	842.4149	1666.7959	833.9016	1665.8119	833.4096	Y	438.2711	219.6392	421.2445	211.1259			3
16	1811.9174	906.4623	1794.8909	897.9491	1793.9068	897.4571	K	***275.2078***	138.1075	258.1812	129.5942			2
17							K	147.1128	74.0600	130.0863	65.5468			1

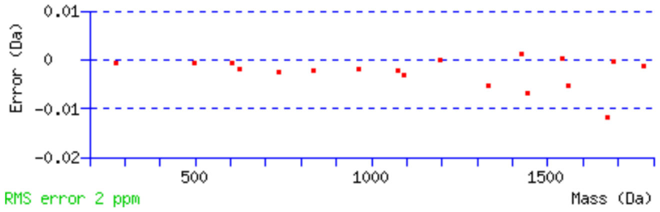

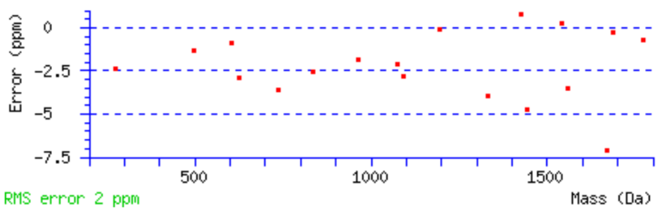

NCBI **BLAST** search of LASELNHVQEVLEGYKK
(Parameters: blastp, nr protein database, expect=20000, no filter, PAM30)
Other BLAST web gateways

All matches to this query

Score	Mr(calc):	Delta	Sequence
102.1	1957.0156	-0.0008	LASELNHVQEVLEGYKK
59.1	1957.0156	-0.0008	LASELNHVQEVLEGYKK

Mascot: http://www.matrixscience.com/

Mascot Search Results

Peptide View

MS/MS Fragmentation of **LASELNHVQEVLEGYKK**
Found in **sp|O43790|KRT86_HUMAN**, Keratin, type II cuticular Hb6 OS=Homo sapiens GN=KRT86 PE=1 SV=1

Match to Query 27767: 1957.019502 from(653.347110,3+) intensity(4794437.5000) scans(13776) rtinseconds(2522) index(10796)
Title: 160219_Sunil_SDSI_A_Spectrum062920_scans__13776_RTINSECONDS=2522
Data file C:\\Sunil\\TKAP\\T\\T160219_Sunil_SDSI_A.mgf

Click mouse within plot area to zoom in by factor of two about that point
Or, Plot from 100 to 1900 Da Full range
Label all possible matches ○ Label matches used for scoring ◉

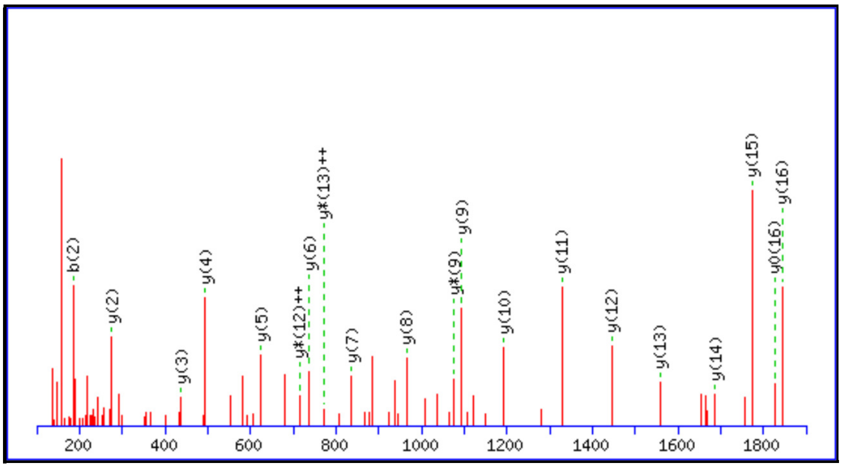

Monoisotopic mass of neutral peptide Mr(calc): 1957.0156
Fixed modifications: Carbamidomethyl (C) (apply to specified residues or termini only)
Variable modifications:
N6 : Deamidated (NQ)
Ions Score: 131 **Expect:** 1.8e-011
Matches : 20/170 fragment ions using 31 most intense peaks (help)

#	b	b++	b*	b*++	b0	b0++	Seq.	y	y++	y*	y*++	y0	y0++	#
1	114.0913	57.5493					L							17
2	**185.1285**	93.0679					A	***1844.9389***	922.9731	1827.9123	914.4598	1826.9283	913.9678	16
3	272.1605	136.5839			254.1499	127.5786	S	***1773.9018***	887.4545	1756.8752	878.9412	1755.8912	878.4492	15
4	401.2031	201.1052			383.1925	192.0999	E	***1686.8697***	843.9385	1669.8432	835.4252	1668.8592	834.9332	14
5	514.2871	257.6472			496.2766	248.6419	L	***1557.8271***	779.4172	1540.8006	770.9039	1539.8166	770.4119	13
6	629.3141	315.1607	612.2875	306.6474	611.3035	306.1554	N	***1444.7431***	722.8752	1427.7165	714.3619	1426.7325	713.8699	12
7	766.3730	383.6901	749.3464	375.1769	748.3624	374.6849	H	***1329.7161***	665.3617	1312.6896	656.8484	1311.7056	656.3564	11
8	865.4414	433.2243	848.4149	424.7111	847.4308	424.2191	V	***1192.6572***	596.8322	1175.6307	588.3190	1174.6467	587.8270	10
9	993.5000	497.2536	976.4734	488.7404	975.4894	488.2483	Q	***1093.5888***	547.2980	1076.5623	538.7848	1075.5782	538.2928	9
10	1122.5426	561.7749	1105.5160	553.2617	1104.5320	552.7696	E	***965.5302***	483.2688	948.5037	474.7555	947.5197	474.2635	8
11	1221.6110	611.3091	1204.5844	602.7959	1203.6004	602.3039	V	***836.4876***	418.7475	819.4611	410.2342	818.4771	409.7422	7
12	1334.6951	667.8512	1317.6685	659.3379	1316.6845	658.8459	L	***737.4192***	369.2132	720.3927	360.7000	719.4087	360.2080	6
13	1463.7376	732.3725	1446.7111	723.8592	1445.7271	723.3672	E	***624.3352***	312.6712	607.3086	304.1579	606.3246	303.6659	5
14	1520.7591	760.8832	1503.7326	752.3699	1502.7485	751.8779	G	***495.2926***	248.1499	478.2660	239.6366			4
15	1683.8224	842.4149	1666.7959	833.9016	1665.8119	833.4096	Y	***438.2711***	219.6392	421.2445	211.1259			3
16	1811.9174	906.4623	1794.8909	897.9491	1793.9068	897.4571	K	***275.2078***	138.1075	258.1812	129.5942			2
17							K	147.1128	74.0600	130.0863	65.5468			1

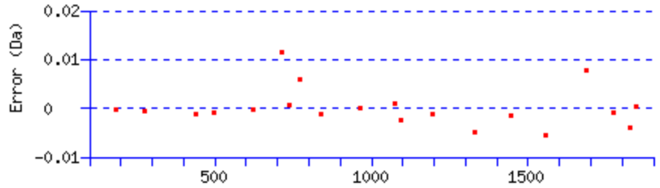

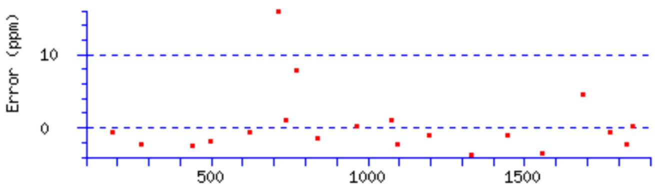

NCBI **BLAST** search of LASELNHVQEVLEGYKK
(Parameters: blastp, nr protein database, expect=20000, no filter, PAM30)
Other BLAST web gateways

All matches to this query

Score	Mr(calc):	Delta	Sequence
131.0	1957.0156	0.0039	LASELNHVQEVLEGYKK
83.3	1957.0156	0.0039	LASELNHVQEVLEGYKK
57.2	1956.0316	0.9879	LASELNHVQEVLEGYKK

Mascot: http://www.matrixscience.com/

Mascot Search Results

Peptide View

MS/MS Fragmentation of **LASELNHVQEVLEGYKK**
Found in **sp|P78385|KRT83_HUMAN**, Keratin, type II cuticular Hb3 OS=Homo sapiens GN=KRT83 PE=1 SV=2

Match to Query 27767: 1957.019502 from(653.347110,3+) intensity(4794437.5000) scans(13776) rtinseconds(2522) index(10796)
Title: 160219_Sunil_SDSI_A_Spectrum062920_scans__13776_RTINSECONDS=2522
Data file C:\\Sunil\\TKAP\\T\\T160219_Sunil_SDSI_A.mgf

Click mouse within plot area to zoom in by factor of two about that point
Or, Plot from 100 to 1900 Da Full range
Label all possible matches ○ Label matches used for scoring ◉

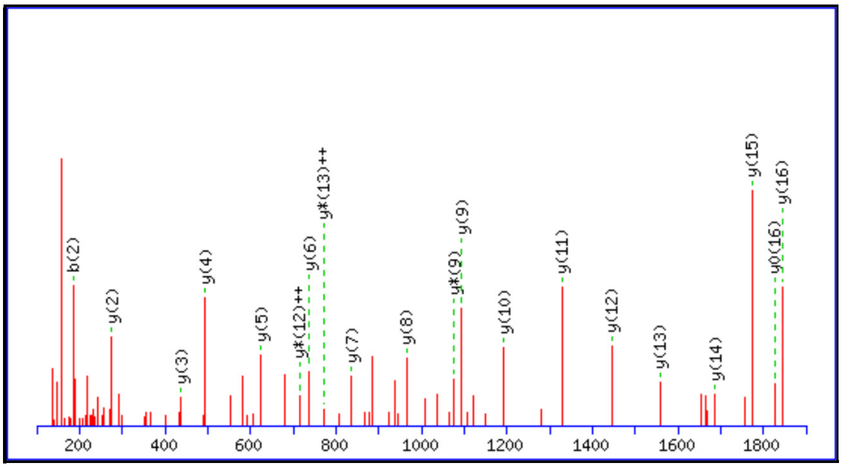

Monoisotopic mass of neutral peptide Mr(calc): 1957.0156
Fixed modifications: Carbamidomethyl (C) (apply to specified residues or termini only)
Variable modifications:
N6 : Deamidated (NQ)
Ions Score: 131 **Expect:** 1.8e-011
Matches : 20/170 fragment ions using 31 most intense peaks (help)

#	b	b++	b*	b*++	b0	b0++	Seq.	y	y++	y*	y*++	y0	y0++	#
1	114.0913	57.5493					L							17
2	**185.1285**	93.0679					A	***1844.9389***	922.9731	1827.9123	914.4598	1826.9283	913.9678	16
3	272.1605	136.5839			254.1499	127.5786	S	***1773.9018***	887.4545	1756.8752	878.9412	1755.8912	878.4492	15
4	401.2031	201.1052			383.1925	192.0999	E	***1686.8697***	843.9385	1669.8432	835.4252	1668.8592	834.9332	14
5	514.2871	257.6472			496.2766	248.6419	L	***1557.8271***	779.4172	1540.8006	770.9039	1539.8166	770.4119	13
6	629.3141	315.1607	612.2875	306.6474	611.3035	306.1554	N	***1444.7431***	722.8752	1427.7165	714.3619	1426.7325	713.8699	12
7	766.3730	383.6901	749.3464	375.1769	748.3624	374.6849	H	***1329.7161***	665.3617	1312.6896	656.8484	1311.7056	656.3564	11
8	865.4414	433.2243	848.4149	424.7111	847.4308	424.2191	V	***1192.6572***	596.8322	1175.6307	588.3190	1174.6467	587.8270	10
9	993.5000	497.2536	976.4734	488.7404	975.4894	488.2483	Q	***1093.5888***	547.2980	1076.5623	538.7848	1075.5782	538.2928	9
10	1122.5426	561.7749	1105.5160	553.2617	1104.5320	552.7696	E	***965.5302***	483.2688	948.5037	474.7555	947.5197	474.2635	8
11	1221.6110	611.3091	1204.5844	602.7959	1203.6004	602.3039	V	***836.4876***	418.7475	819.4611	410.2342	818.4771	409.7422	7
12	1334.6951	667.8512	1317.6685	659.3379	1316.6845	658.8459	L	***737.4192***	369.2132	720.3927	360.7000	719.4087	360.2080	6
13	1463.7376	732.3725	1446.7111	723.8592	1445.7271	723.3672	E	***624.3352***	312.6712	607.3086	304.1579	606.3246	303.6659	5
14	1520.7591	760.8832	1503.7326	752.3699	1502.7485	751.8779	G	***495.2926***	248.1499	478.2660	239.6366			4
15	1683.8224	842.4149	1666.7959	833.9016	1665.8119	833.4096	Y	***438.2711***	219.6392	421.2445	211.1259			3
16	1811.9174	906.4623	1794.8909	897.9491	1793.9068	897.4571	K	***275.2078***	138.1075	258.1812	129.5942			2
17							K	147.1128	74.0600	130.0863	65.5468			1

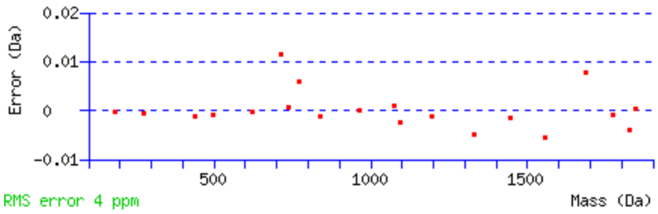

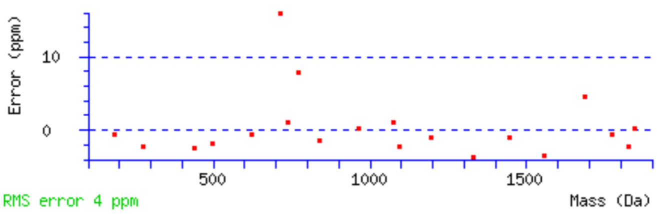

NCBI **BLAST** search of LASELNHVQEVLEGYKK
(Parameters: blastp, nr protein database, expect=20000, no filter, PAM30)
Other BLAST web gateways

All matches to this query

Score	Mr(calc):	Delta	Sequence
131.0	1957.0156	0.0039	LASELNHVQEVLEGYKK
83.3	1957.0156	0.0039	LASELNHVQEVLEGYKK
57.2	1956.0316	0.9879	LASELNHVQEVLEGYKK

Mascot: http://www.matrixscience.com/

Mascot Search Results

Peptide View

MS/MS Fragmentation of **LCEGVEAVNVCVSSSR**
Found in **sp|P78385|KRT83_HUMAN**, Keratin, type II cuticular Hb3 OS=Homo sapiens GN=KRT83 PE=1 SV=2

Match to Query 24068: 1765.804168 from(883.909360,2+) intensity(561425.0625) scans(10334) rtinseconds(1930) index(7967)
Title: 160219_Sunil_SDSI_A_Spectrum060076_scans__10334_RTINSECONDS=1930
Data file C:\\Sunil\\TKAP\\T\\T160219_Sunil_SDSI_A.mgf

Click mouse within plot area to zoom in by factor of two about that point
Or, Plot from 100 to 1800 Da Full range
Label all possible matches ○ Label matches used for scoring ◉

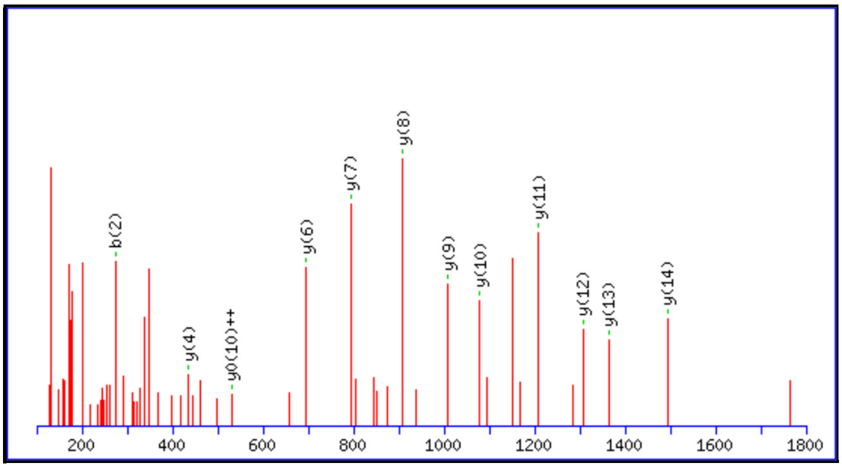

Monoisotopic mass of neutral peptide Mr(calc): 1765.7975
Fixed modifications: Carbamidomethyl (C) (apply to specified residues or termini only)
Variable modifications:
N9 : Deamidated (NQ)
Ions Score: 91 **Expect:** 1.3e-007
Matches : 12/158 fragment ions using 15 most intense peaks (help)

#	b	b^{++}	b*	b^{*++}	b^0	b^{0++}	Seq.	y	y^{++}	y*	y^{*++}	y^0	y^{0++}	#
1	114.0913	57.5493					L							16
2	**274.1220**	137.5646					C	1653.7207	827.3640	1636.6941	818.8507	1635.7101	818.3587	15
3	403.1646	202.0859			385.1540	193.0806	E	***1493.6900***	747.3487	1476.6635	738.8354	1475.6795	738.3434	14
4	460.1860	230.5967			442.1755	221.5914	G	***1364.6475***	682.8274	1347.6209	674.3141	1346.6369	673.8221	13
5	559.2545	280.1309			541.2439	271.1256	V	***1307.6260***	654.3166	1290.5994	645.8034	1289.6154	645.3114	12
6	688.2971	344.6522			670.2865	335.6469	E	***1208.5576***	604.7824	1191.5310	596.2692	1190.5470	595.7771	11
7	759.3342	380.1707			741.3236	371.1654	A	***1079.5150***	540.2611	1062.4884	531.7479	1061.5044	531.2558	10
8	858.4026	429.7049			840.3920	420.6996	V	***1008.4779***	504.7426	991.4513	496.2293	990.4673	495.7373	9
9	973.4295	487.2184	956.4030	478.7051	955.4190	478.2131	N	***909.4095***	455.2084	892.3829	446.6951	891.3989	446.2031	8
10	1072.4979	536.7526	1055.4714	528.2393	1054.4874	527.7473	V	***794.3825***	397.6949	777.3560	389.1816	776.3719	388.6896	7
11	1232.5286	616.7679	1215.5020	608.2547	1214.5180	607.7626	C	***695.3141***	348.1607	678.2876	339.6474	677.3035	339.1554	6
12	1331.5970	666.3021	1314.5705	657.7889	1313.5864	657.2969	V	535.2835	268.1454	518.2569	259.6321	517.2729	259.1401	5
13	1418.6290	709.8182	1401.6025	701.3049	1400.6185	700.8129	S	***436.2150***	218.6112	419.1885	210.0979	418.2045	209.6059	4
14	1505.6611	753.3342	1488.6345	744.8209	1487.6505	744.3289	S	349.1830	175.0951	332.1565	166.5819	331.1724	166.0899	3
15	1592.6931	796.8502	1575.6665	788.3369	1574.6825	787.8449	S	262.1510	131.5791	245.1244	123.0659	244.1404	122.5738	2
16							R	175.1190	88.0631	158.0924	79.5498			1

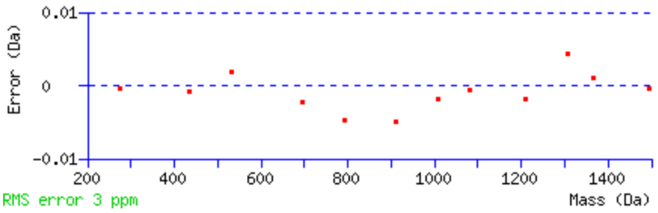

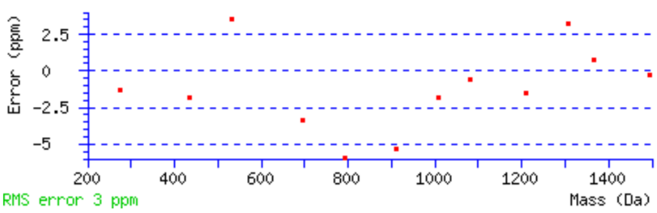

NCBI **BLAST** search of LCEGVEAVNVCVSSSR

(Parameters: blastp, nr protein database, expect=20000, no filter, PAM30)
Other BLAST web gateways

All matches to this query

Score	Mr(calc):	Delta	Sequence
90.7	1765.7975	0.0067	LCEGVEAVNVCVSSSR
0.3	1764.8135	0.9907	LCEGVEAVNVCVSSSR

Mascot: http://www.matrixscience.com/

Mascot Search Results

Peptide View

MS/MS Fragmentation of **LCEGVGSVNVCVSSSR**
Found in **sp|O43790|KRT86_HUMAN**, Keratin, type II cuticular Hb6 OS=Homo sapiens GN=KRT86 PE=1 SV=1

Match to Query 23058: 1709.774868 from(855.894710,2+) intensity(2184987.7500) scans(9773) rtinseconds(1833) index(7483)
Title: 160219_Sunil_SDSI_A_Spectrum059591_scans__9773_RTINSECONDS=1833
Data file C:\\Sunil\\TKAP\\T\\T160219_Sunil_SDSI_A.mgf

Click mouse within plot area to zoom in by factor of two about that point
Or, Plot from 100 to 1500 Da Full range
Label all possible matches ○ Label matches used for scoring ◉

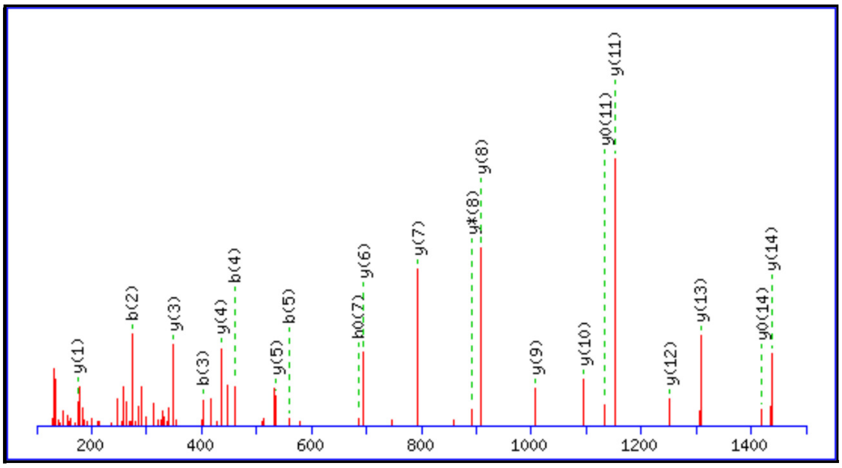

Monoisotopic mass of neutral peptide Mr(calc): 1709.7713
Fixed modifications: Carbamidomethyl (C) (apply to specified residues or termini only)
Variable modifications:
N9 : Deamidated (NQ)
Ions Score: 93 **Expect:** 6.6e-008
Matches : 21/158 fragment ions using 41 most intense peaks (help)

#	b	b++	b*	b*++	b0	b0++	Seq.	y	y++	y*	y*++	y0	y0++	#
1	114.0913	57.5493					L							16
2	**274.1220**	137.5646					C	1597.6945	799.3509	1580.6679	790.8376	1579.6839	790.3456	15
3	**403.1646**	202.0859			385.1540	193.0806	E	***1437.6638***	719.3356	1420.6373	710.8223	1419.6533	710.3303	14
4	**460.1860**	230.5967			442.1755	221.5914	G	***1308.6212***	654.8143	1291.5947	646.3010	1290.6107	645.8090	13
5	**559.2545**	280.1309			541.2439	271.1256	V	***1251.5998***	626.3035	1234.5732	617.7903	1233.5892	617.2982	12
6	616.2759	308.6416			598.2654	299.6363	G	***1152.5314***	576.7693	1135.5048	568.2560	1134.5208	567.7640	11
7	703.3080	352.1576			685.2974	343.1523	S	***1095.5099***	548.2586	1078.4833	539.7453	1077.4993	539.2533	10
8	802.3764	401.6918			784.3658	392.6865	V	***1008.4779***	504.7426	991.4513	496.2293	990.4673	495.7373	9
9	917.4033	459.2053	900.3768	450.6920	899.3927	450.2000	N	***909.4095***	455.2084	892.3829	446.6951	891.3989	446.2031	8
10	1016.4717	508.7395	999.4452	500.2262	998.4612	499.7342	V	***794.3825***	397.6949	777.3560	389.1816	776.3719	388.6896	7
11	1176.5024	588.7548	1159.4758	580.2415	1158.4918	579.7495	C	***695.3141***	348.1607	678.2876	339.6474	677.3035	339.1554	6
12	1275.5708	638.2890	1258.5442	629.7758	1257.5602	629.2837	V	***535.2835***	268.1454	518.2569	259.6321	517.2729	259.1401	5
13	1362.6028	681.8050	1345.5763	673.2918	1344.5922	672.7998	S	***436.2150***	218.6112	419.1885	210.0979	418.2045	209.6059	4
14	1449.6348	725.3211	1432.6083	716.8078	1431.6243	716.3158	S	***349.1830***	175.0951	332.1565	166.5819	331.1724	166.0899	3
15	1536.6669	768.8371	1519.6403	760.3238	1518.6563	759.8318	S	262.1510	131.5791	245.1244	123.0659	244.1404	122.5738	2
16							R	***175.1190***	88.0631	158.0924	79.5498			1

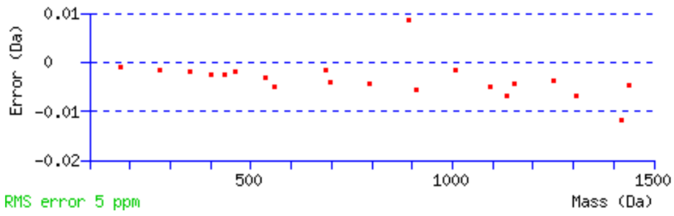

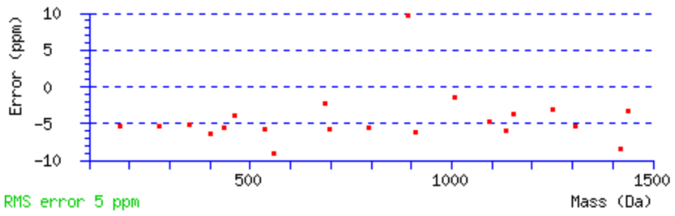

NCBI **BLAST** search of LCEGVGSVNVCVSSSR

(Parameters: blastp, nr protein database, expect=20000, no filter, PAM30)
Other BLAST web gateways

All matches to this query

Score	Mr(calc):	Delta	Sequence
93.5	1709.7713	0.0036	LCEGVGSVNVCVSSSR
75.0	1708.7873	0.9876	LCEGVGSVNVCVSSSR

Mascot: http://www.matrixscience.com/

Mascot Search Results

Peptide View

MS/MS Fragmentation of **LLEGEEQRLCEGVEAVNVCVSSSR**
Found in **sp|P78385|KRT83_HUMAN**, Keratin, type II cuticular Hb3 OS=Homo sapiens GN=KRT83 PE=1 SV=2

Match to Query 38825: 2720.284752 from(907.768860,3+) intensity(1947554.2500) scans(12995) rtinseconds(2388) index(10180)
Title: 160219_Sunil_SDSI_A_Spectrum062300_scans__12995_RTINSECONDS=2388
Data file C:\\Sunil\\TKAP\\T\\T160219_Sunil_SDSI_A.mgf

Click mouse within plot area to zoom in by factor of two about that point
Or, Plot from 0 to 2800 Da Full range
Label all possible matches Label matches used for scoring

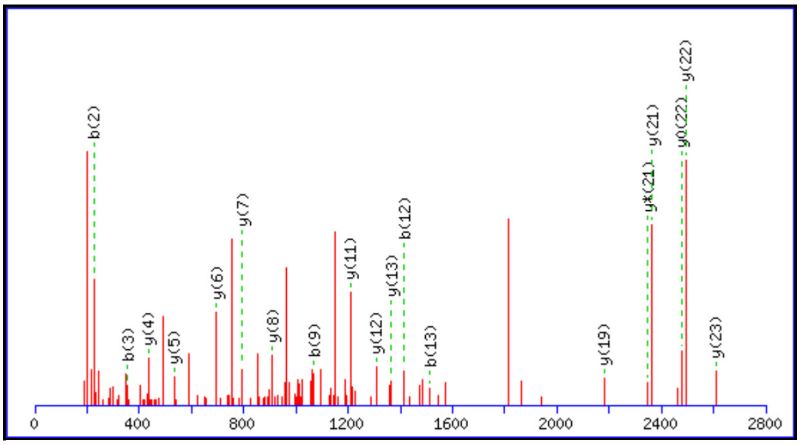

Monoisotopic mass of neutral peptide Mr(calc): 2720.2745
Fixed modifications: Carbamidomethyl (C) (apply to specified residues or termini only)
Variable modifications:
N17 : Deamidated (NQ)
Ions Score: 64 **Expect:** 9e-005
Matches : 19/258 fragment ions using 38 most intense peaks (help)

#	b	b^{++}	b^{*}	b^{*++}	b^{0}	b^{0++}	Seq.	y	y^{++}	y^{*}	y^{*++}	y^{0}	y^{0++}	#
1	114.0913	57.5493					L							24
2	227.1754	114.0913					L	2608.1978	1304.6025	2591.1712	1296.0892	2590.1872	1295.5972	23
3	356.2180	178.6126			338.2074	169.6074	E	2495.1137	1248.0605	2478.0871	1239.5472	2477.1031	1239.0552	22
4	413.2395	207.1234			395.2289	198.1181	G	2366.0711	1183.5392	2349.0446	1175.0259	2348.0605	1174.5339	21
5	542.2821	271.6447			524.2715	262.6394	E	2309.0496	1155.0285	2292.0231	1146.5152	2291.0391	1146.0232	20
6	671.3246	336.1660			653.3141	327.1607	E	2180.0070	1090.5072	2162.9805	1081.9939	2161.9965	1081.5019	19
7	799.3832	400.1953	782.3567	391.6820	781.3727	391.1900	Q	2050.9644	1025.9859	2033.9379	1017.4726	2032.9539	1016.9806	18
8	955.4843	478.2458	938.4578	469.7325	937.4738	469.2405	R	1922.9059	961.9566	1905.8793	953.4433	1904.8953	952.9513	17
9	1068.5684	534.7878	1051.5419	526.2746	1050.5578	525.7826	L	1766.8048	883.9060	1749.7782	875.3927	1748.7942	874.9007	16
10	1228.5990	614.8032	1211.5725	606.2899	1210.5885	605.7979	C	1653.7207	827.3640	1636.6941	818.8507	1635.7101	818.3587	15
11	1357.6416	679.3245	1340.6151	670.8112	1339.6311	670.3192	E	1493.6900	747.3487	1476.6635	738.8354	1475.6795	738.3434	14
12	1414.6631	707.8352	1397.6366	699.3219	1396.6525	698.8299	G	1364.6475	682.8274	1347.6209	674.3141	1346.6369	673.8221	13
13	1513.7315	757.3694	1496.7050	748.8561	1495.7210	748.3641	V	1307.6260	654.3166	1290.5994	645.8034	1289.6154	645.3114	12
14	1642.7741	821.8907	1625.7476	813.3774	1624.7635	812.8854	E	1208.5576	604.7824	1191.5310	596.2692	1190.5470	595.7771	11
15	1713.8112	857.4093	1696.7847	848.8960	1695.8007	848.4040	A	1079.5150	540.2611	1062.4884	531.7479	1061.5044	531.2558	10
16	1812.8796	906.9435	1795.8531	898.4302	1794.8691	897.9382	V	1008.4779	504.7426	991.4513	496.2293	990.4673	495.7373	9
17	1927.9066	964.4569	1910.8800	955.9437	1909.8960	955.4516	N	909.4095	455.2084	892.3829	446.6951	891.3989	446.2031	8
18	2026.9750	1013.9911	2009.9484	1005.4779	2008.9644	1004.9859	V	794.3825	397.6949	777.3560	389.1816	776.3719	388.6896	7
19	2187.0056	1094.0065	2169.9791	1085.4932	2168.9951	1085.0012	C	695.3141	348.1607	678.2875	339.6474	677.3035	339.1554	6
20	2286.0741	1143.5407	2269.0475	1135.0274	2268.0635	1134.5354	V	535.2835	268.1454	518.2569	259.6321	517.2729	259.1401	5
21	2373.1061	1187.0567	2356.0795	1178.5434	2355.0955	1178.0514	S	436.2150	218.6112	419.1885	210.0979	418.2045	209.6059	4
22	2460.1381	1230.5727	2443.1116	1222.0594	2442.1276	1221.5674	S	349.1830	175.0951	332.1565	166.5819	331.1724	166.0899	3
23	2547.1701	1274.0887	2530.1436	1265.5754	2529.1596	1265.0834	S	262.1510	131.5791	245.1244	123.0659	244.1404	122.5738	2
24							R	175.1190	88.0631	158.0924	79.5498			1

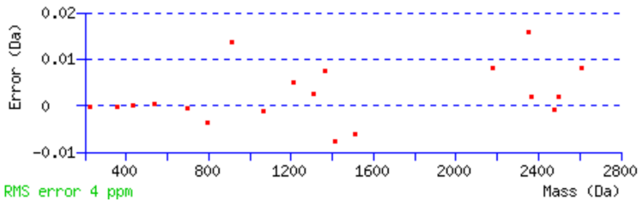

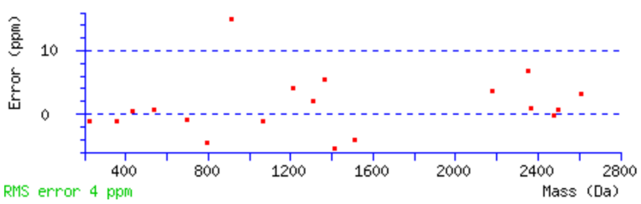

NCBI **BLAST** search of LLEGEEQRLCEGVEAVNVCVSSSR
(Parameters: blastp, nr protein database, expect=20000, no filter, PAM30)
Other BLAST web gateways

All matches to this query

Score	Mr(calc):	Delta	Sequence
64.5	2720.2745	0.0102	LLEGEEQRLCEGVEAVNVCVSSSR
21.2	2720.2745	0.0102	LLEGEEQRLCEGVEAVNVCVSSSR
14.6	2719.2905	0.9942	LLEGEEQRLCEGVEAVNVCVSSSR

Mascot: http://www.matrixscience.com/

Mascot Search Results

Peptide View

MS/MS Fragmentation of **LLETKLQFYQNR**
Found in **sp|O43790|KRT86_HUMAN**, Keratin, type II cuticular Hb6 OS=Homo sapiens GN=KRT86 PE=1 SV=1

Match to Query 19288: 1552.822848 from(777.418700,2+) intensity(597792.2500) scans(11128) rtinseconds(2067) index(8606)
Title: 160219_Sunil_SDSI_A_Spectrum060717_scans__11128_RTINSECONDS=2067
Data file C:\\Sunil\\TKAP\\T\\T160219_Sunil_SDSI_A.mgf

Click mouse within plot area to zoom in by factor of two about that point
Or, Plot from 100 to 1600 Da Full range
Label all possible matches Label matches used for scoring

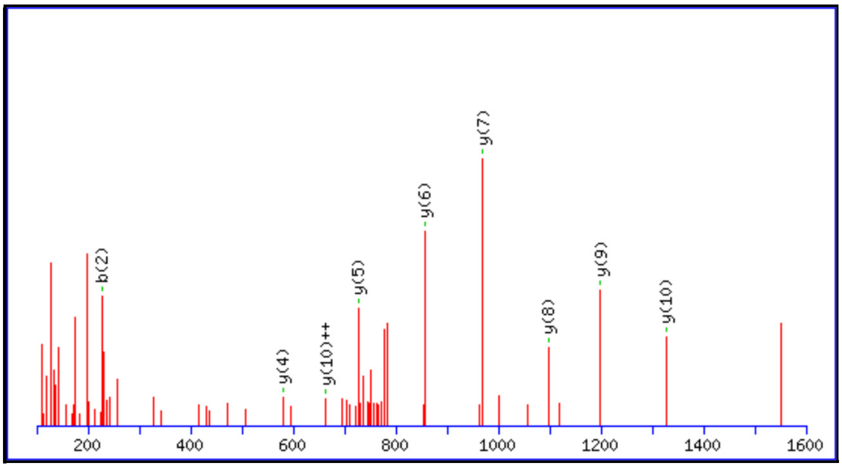

Monoisotopic mass of neutral peptide Mr(calc): 1552.8249
Fixed modifications: Carbamidomethyl (C) (apply to specified residues or termini only)
Variable modifications:
Q7 : Deamidated (NQ)
Ions Score: 52 **Expect:** 0.001
Matches : 9/104 fragment ions using 13 most intense peaks (help)

#	b	b++	b*	b*++	b0	b0++	Seq.	y	y++	y*	y*++	y0	y0++	#
1	114.0913	57.5493					L							12
2	**227.1754**	114.0913					L	1440.7482	720.8777	1423.7216	712.3644	1422.7376	711.8724	11
3	356.2180	178.6126			338.2074	169.6074	E	***1327.6641***	664.3357	1310.6375	655.8224	1309.6535	655.3304	10
4	457.2657	229.1365			439.2551	220.1312	T	***1198.6215***	599.8144	1181.5950	591.3011	1180.6109	590.8091	9
5	585.3606	293.1840	568.3341	284.6707	567.3501	284.1787	K	***1097.5738***	549.2905	1080.5473	540.7773			8
6	698.4447	349.7260	681.4182	341.2127	680.4341	340.7207	L	***969.4789***	485.2431	952.4523	476.7298			7
7	827.4873	414.2473	810.4607	405.7340	809.4767	405.2420	Q	***856.3948***	428.7010	839.3682	420.1878			6
8	974.5557	487.7815	957.5292	479.2682	956.5451	478.7762	F	***727.3522***	364.1797	710.3257	355.6665			5
9	1137.6190	569.3132	1120.5925	560.7999	1119.6085	560.3079	Y	***580.2838***	290.6455	563.2572	282.1323			4
10	1265.6776	633.3424	1248.6511	624.8292	1247.6671	624.3372	Q	417.2205	209.1139	400.1939	200.6006			3
11	1379.7205	690.3639	1362.6940	681.8506	1361.7100	681.3586	N	289.1619	145.0846	272.1353	136.5713			2
12							R	175.1190	88.0631	158.0924	79.5498			1

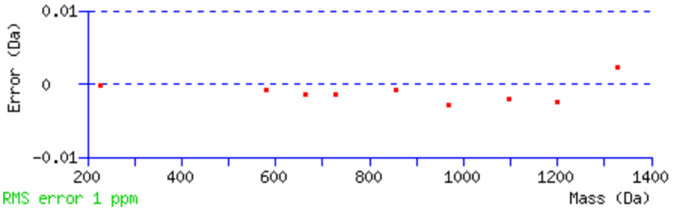

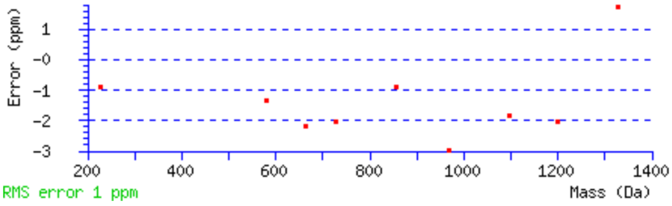

NCBI **BLAST** search of LLETKLQFYQNR
(Parameters: blastp, nr protein database, expect=20000, no filter, PAM30)
Other BLAST web gateways

All matches to this query

Score	Mr(calc):	Delta	Sequence
52.4	1552.8249	-0.0021	LLETKLQFYQNR
26.0	1552.8249	-0.0021	LLETKLQFYQNR
26.0	1552.8249	-0.0021	LLETKLQFYQNR

Mascot: http://www.matrixscience.com/

Mascot Search Results

Peptide View

MS/MS Fragmentation of **LLETKLQFYQNR**
Found in **sp|P78385|KRT83_HUMAN**, Keratin, type II cuticular Hb3 OS=Homo sapiens GN=KRT83 PE=1 SV=2

Match to Query 19290: 1552.825332 from(518.615720,3+) intensity(9145513.0000) scans(11202) rtinseconds(2080) index(8667)
Title: 160219_Sunil_SDSI_A_Spectrum060778_scans__11202_RTINSECONDS=2080
Data file C:\\Sunil\\TKAP\\T\\T160219_Sunil_SDSI_A.mgf

Click mouse within plot area to zoom in by factor of two about that point
Or, Plot from 100 to 1400 Da Full range
Label all possible matches ○ Label matches used for scoring ◉

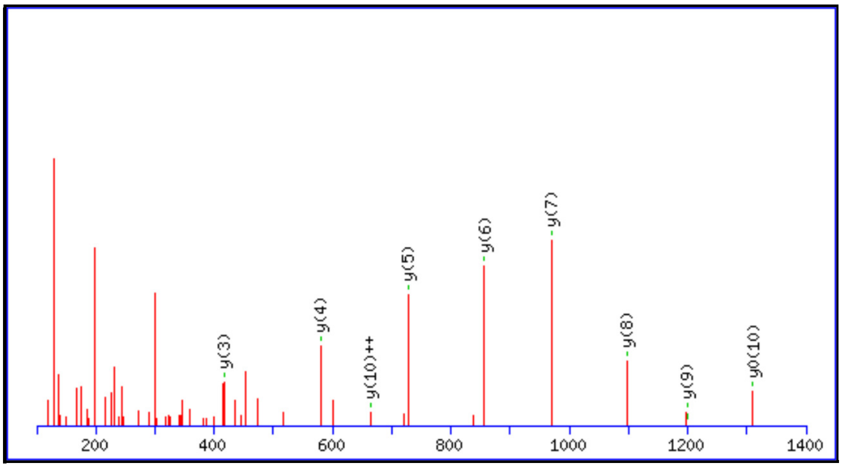

Monoisotopic mass of neutral peptide Mr(calc): 1552.8249
Fixed modifications: Carbamidomethyl (C) (apply to specified residues or termini only)
Variable modifications:
Q7 : Deamidated (NQ)
Ions Score: 55 **Expect:** 0.00049
Matches : 9/104 fragment ions using 12 most intense peaks (help)

#	b	b^{++}	b*	b^{*++}	b^0	b^{0++}	Seq.	y	y^{++}	y*	y^{*++}	y^0	y^{0++}	#
1	114.0913	57.5493					L							12
2	227.1754	114.0913					L	1440.7482	720.8777	1423.7216	712.3644	1422.7376	711.8724	11
3	356.2180	178.6126			338.2074	169.6074	E	1327.6641	664.3357	1310.6375	655.8224	1309.6535	655.3304	10
4	457.2657	229.1365			439.2551	220.1312	T	***1198.6215***	599.8144	1181.5950	591.3011	1180.6109	590.8091	9
5	585.3606	293.1840	568.3341	284.6707	567.3501	284.1787	K	***1097.5738***	549.2905	1080.5473	540.7773			8
6	698.4447	349.7260	681.4182	341.2127	680.4341	340.7207	L	***969.4789***	485.2431	952.4523	476.7298			7
7	827.4873	414.2473	810.4607	405.7340	809.4767	405.2420	Q	***856.3948***	428.7010	839.3682	420.1878			6
8	974.5557	487.7815	957.5292	479.2682	956.5451	478.7762	F	***727.3522***	364.1797	710.3257	355.6665			5
9	1137.6190	569.3132	1120.5925	560.7999	1119.6085	560.3079	Y	***580.2838***	290.6455	563.2572	282.1323			4
10	1265.6776	633.3424	1248.6511	624.8292	1247.6671	624.3372	Q	***417.2205***	209.1139	400.1939	200.6006			3
11	1379.7205	690.3639	1362.6940	681.8506	1361.7100	681.3586	N	289.1619	145.0846	272.1353	136.5713			2
12							R	175.1190	88.0631	158.0924	79.5498			1

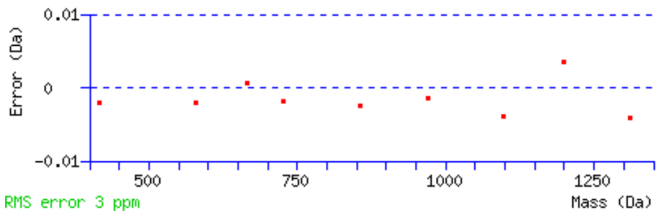

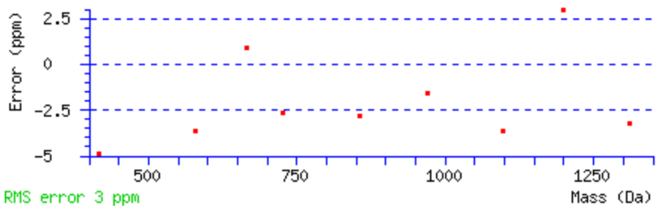

NCBI **BLAST** search of LLETKLQFYQNR
(Parameters: blastp, nr protein database, expect=20000, no filter, PAM30)
Other BLAST web gateways

All matches to this query

Score	Mr(calc):	Delta	Sequence
55.5	1552.8249	0.0004	LLETKLQFYQNR
18.9	1552.8249	0.0004	LLETKLQFYQNR
16.0	1552.8249	0.0004	LLETKLQFYQNR

Mascot: http://www.matrixscience.com/

Mascot Search Results

Peptide View

MS/MS Fragmentation of **LLETKLQFYQNR**
Found in **sp|O43790|KRT86_HUMAN**, Keratin, type II cuticular Hb6 OS=Homo sapiens GN=KRT86 PE=1 SV=1

Match to Query 19290: 1552.825332 from(518.615720,3+) intensity(9145513.0000) scans(11202) rtinseconds(2080) index(8667)
Title: 160219_Sunil_SDSI_A_Spectrum060778_scans__11202_RTINSECONDS=2080
Data file C:\\Sunil\\TKAP\\T\\T160219_Sunil_SDSI_A.mgf

Click mouse within plot area to zoom in by factor of two about that point
Or, Plot from 100 to 1400 Da Full range
Label all possible matches Label matches used for scoring

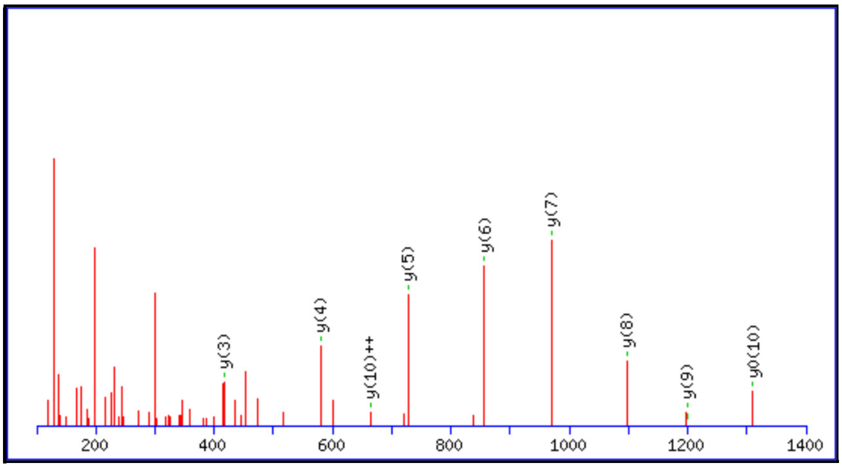

Monoisotopic mass of neutral peptide Mr(calc): 1552.8249
Fixed modifications: Carbamidomethyl (C) (apply to specified residues or termini only)
Variable modifications:
Q7 : Deamidated (NQ)
Ions Score: 55 **Expect:** 0.00049
Matches : 9/104 fragment ions using 12 most intense peaks (help)

#	b	b++	b*	b*++	b0	b0++	Seq.	y	y++	y*	y*++	y0	y0++	#
1	114.0913	57.5493					L							12
2	227.1754	114.0913					L	1440.7482	720.8777	1423.7216	712.3644	1422.7376	711.8724	11
3	356.2180	178.6126			338.2074	169.6074	E	1327.6641	664.3357	1310.6375	655.8224	1309.6535	655.3304	10
4	457.2657	229.1365			439.2551	220.1312	T	***1198.6215***	599.8144	1181.5950	591.3011	1180.6109	590.8091	9
5	585.3606	293.1840	568.3341	284.6707	567.3501	284.1787	K	***1097.5738***	549.2905	1080.5473	540.7773			8
6	698.4447	349.7260	681.4182	341.2127	680.4341	340.7207	L	***969.4789***	485.2431	952.4523	476.7298			7
7	827.4873	414.2473	810.4607	405.7340	809.4767	405.2420	Q	***856.3948***	428.7010	839.3682	420.1878			6
8	974.5557	487.7815	957.5292	479.2682	956.5451	478.7762	F	***727.3522***	364.1797	710.3257	355.6665			5
9	1137.6190	569.3132	1120.5925	560.7999	1119.6085	560.3079	Y	***580.2838***	290.6455	563.2572	282.1323			4
10	1265.6776	633.3424	1248.6511	624.8292	1247.6671	624.3372	Q	***417.2205***	209.1139	400.1939	200.6006			3
11	1379.7205	690.3639	1362.6940	681.8506	1361.7100	681.3586	N	289.1619	145.0846	272.1353	136.5713			2
12							R	175.1190	88.0631	158.0924	79.5498			1

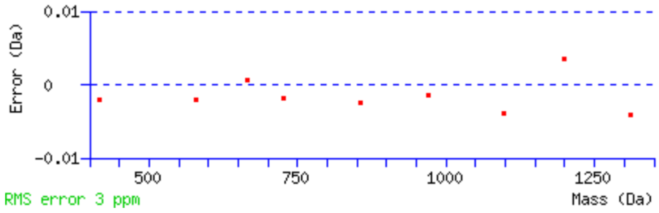

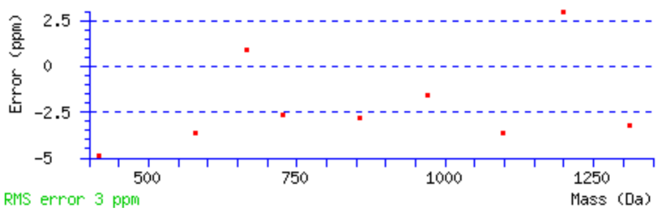

NCBI **BLAST** search of LLETKLQFYQNR
(Parameters: blastp, nr protein database, expect=20000, no filter, PAM30)
Other BLAST web gateways

All matches to this query

Score	Mr(calc):	Delta	Sequence
55.5	1552.8249	0.0004	LLETKLQFYQNR
18.9	1552.8249	0.0004	LLETKLQFYQNR
16.0	1552.8249	0.0004	LLETKLQFYQNR

Mascot: http://www.matrixscience.com/

Mascot Search Results

Peptide View

MS/MS Fragmentation of **LQFYQNRECCQSNLEPLFEGYIETLRR**
Found in **sp|O43790|KRT86_HUMAN**, Keratin, type II cuticular Hb6 OS=Homo sapiens GN=KRT86 PE=1 SV=1

Match to Query 42422: 3463.636176 from(866.916320,4+) intensity(755042.5625) scans(16130) rtinseconds(2924) index(12752)
Title: 160219_Sunil_SDSI_A_Spectrum064895_scans__16130_RTINSECONDS=2924
Data file C:\\Sunil\\TKAP\\T\\T160219_Sunil_SDSI_A.mgf

Click mouse within plot area to zoom in by factor of two about that point
Or, Plot from 200 to 3400 Da Full range
Label all possible matches Label matches used for scoring

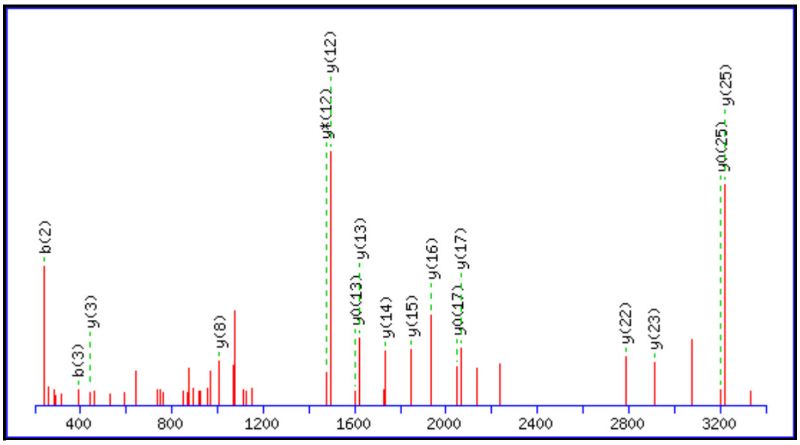

Monoisotopic mass of neutral peptide Mr(calc): 3463.6289
Fixed modifications: Carbamidomethyl (C) (apply to specified residues or termini only)
Variable modifications:
Q11 : Deamidated (NQ)
Ions Score: 52 **Expect:** 0.0013
Matches : 17/290 fragment ions using 35 most intense peaks (help)

#	b	b^{++}	b*	b^{*++}	b^0	b^{0++}	Seq.	y	y^{++}	y*	y^{*++}	y^0	y^{0++}	#
1	114.0913	57.5493					L							27
2	**242.1499**	121.5786	225.1234	113.0653			Q	3351.5521	1676.2797	3334.5256	1667.7664	3333.5416	1667.2744	26
3	**389.2183**	195.1128	372.1918	186.5995			F	***3223.4935***	1612.2504	3206.4670	1603.7371	3205.4830	1603.2451	25
4	552.2817	276.6445	535.2551	268.1312			Y	3076.4251	1538.7162	3059.3986	1530.2029	3058.4146	1529.7109	24
5	680.3402	340.6738	663.3137	332.1605			Q	***2913.3618***	1457.1845	2896.3353	1448.6713	2895.3512	1448.1793	23
6	794.3832	397.6952	777.3566	389.1819			N	***2785.3032***	1393.1553	2768.2767	1384.6420	2767.2927	1384.1500	22
7	950.4843	475.7458	933.4577	467.2325			R	2671.2603	1336.1338	2654.2337	1327.6205	2653.2497	1327.1285	21
8	1079.5269	540.2671	1062.5003	531.7538	1061.5163	531.2618	E	2515.1592	1258.0832	2498.1326	1249.5700	2497.1486	1249.0779	20
9	1239.5575	620.2824	1222.5310	611.7691	1221.5470	611.2771	C	2386.1166	1193.5619	2369.0900	1185.0487	2368.1060	1184.5567	19
10	1399.5882	700.2977	1382.5616	691.7844	1381.5776	691.2924	C	2226.0859	1113.5466	2209.0594	1105.0333	2208.0754	1104.5413	18
11	1528.6308	764.8190	1511.6042	756.3057	1510.6202	755.8137	Q	***2066.0553***	1033.5313	2049.0287	1025.0180	2048.0447	1024.5260	17
12	1615.6628	808.3350	1598.6362	799.8218	1597.6522	799.3298	S	***1937.0127***	969.0100	1919.9862	960.4967	1919.0021	960.0047	16
13	1729.7057	865.3565	1712.6792	856.8432	1711.6952	856.3512	N	***1849.9807***	925.4940	1832.9541	916.9807	1831.9701	916.4887	15
14	1842.7898	921.8985	1825.7632	913.3853	1824.7792	912.8932	L	***1735.9377***	868.4725	1718.9112	859.9592	1717.9272	859.4672	14
15	1971.8324	986.4198	1954.8058	977.9066	1953.8218	977.4145	E	***1622.8537***	811.9305	1605.8271	803.4172	1604.8431	802.9252	13
16	2068.8851	1034.9462	2051.8586	1026.4329	2050.8746	1025.9409	P	***1493.8111***	747.4092	1476.7845	738.8959	1475.8005	738.4039	12
17	2181.9692	1091.4882	2164.9427	1082.9750	2163.9586	1082.4830	L	1396.7583	698.8828	1379.7318	690.3695	1378.7478	689.8775	11
18	2329.0376	1165.0224	2312.0111	1156.5092	2311.0271	1156.0172	F	1283.6743	642.3408	1266.6477	633.8275	1265.6637	633.3355	10
19	2458.0802	1229.5437	2441.0537	1221.0305	2440.0696	1220.5385	E	1136.6058	568.8066	1119.5793	560.2933	1118.5953	559.8013	9
20	2515.1017	1258.0545	2498.0751	1249.5412	2497.0911	1249.0492	G	***1007.5633***	504.2853	990.5367	495.7720	989.5527	495.2800	8
21	2678.1650	1339.5861	2661.1385	1331.0729	2660.1544	1330.5809	Y	950.5418	475.7745	933.5152	467.2613	932.5312	466.7693	7
22	2791.2491	1396.1282	2774.2225	1387.6149	2773.2385	1387.1229	I	787.4785	394.2429	770.4519	385.7296	769.4679	385.2376	6
23	2920.2917	1460.6495	2903.2651	1452.1362	2902.2811	1451.6442	E	674.3944	337.7008	657.3678	329.1876	656.3838	328.6956	5
24	3021.3393	1511.1733	3004.3128	1502.6600	3003.3288	1502.1680	T	545.3518	273.1795	528.3253	264.6663	527.3412	264.1743	4
25	3134.4234	1567.7153	3117.3969	1559.2021	3116.4128	1558.7101	L	***444.3041***	222.6557	427.2776	214.1424			3
26	3290.5245	1645.7659	3273.4980	1637.2526	3272.5139	1636.7606	R	331.2201	166.1137	314.1935	157.6004			2
27							R	175.1190	88.0631	158.0924	79.5498			1

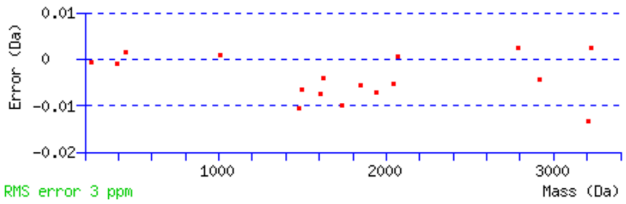

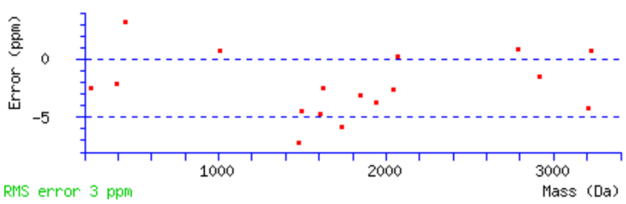

NCBI **BLAST** search of LQFYQNRECCQSNLEPLFEGYIETLRR
(Parameters: blastp, nr protein database, expect=20000, no filter, PAM30)
Other BLAST web gateways

All matches to this query

Score	Mr(calc):	Delta	Sequence
52.4	3463.6289	0.0073	LQFYQNRECCQSNLEPLFEGYIETLRR
42.8	3463.6289	0.0073	LQFYQNRECCQSNLEPLFEGYIETLRR
34.3	3463.6289	0.0073	LQFYQNRECCQSNLEPLFEGYIETLRR
34.0	3463.6289	0.0073	LQFYQNRECCQSNLEPLFEGYIETLRR
25.9	3462.6449	0.9913	LQFYQNRECCQSNLEPLFEGYIETLRR
22.5	3463.6289	0.0073	LQFYQNRECCQSNLEPLFEGYIETLRR

Mascot: http://www.matrixscience.com/

Mascot Search Results

Peptide View

MS/MS Fragmentation of **LTAEVENAK**
Found in **sp|O43790|KRT86_HUMAN**, Keratin, type II cuticular Hb6 OS=Homo sapiens GN=KRT86 PE=1 SV=1

Match to Query 2409: 974.491788 from(488.253170,2+) intensity(3478429.2500) scans(3010) rtinseconds(642) index(16961)
Title: 160219_Sunil_SDSI_A_Spectrum070261_scans__3010_RTINSECONDS=642
Data file C:\\Sunil\\TKAP\\T\\T160219_Sunil_SDSI_A.mgf

Click mouse within plot area to zoom in by factor of two about that point
Or, Plot from 50 to 900 Da Full range
Label all possible matches ○ Label matches used for scoring ◉

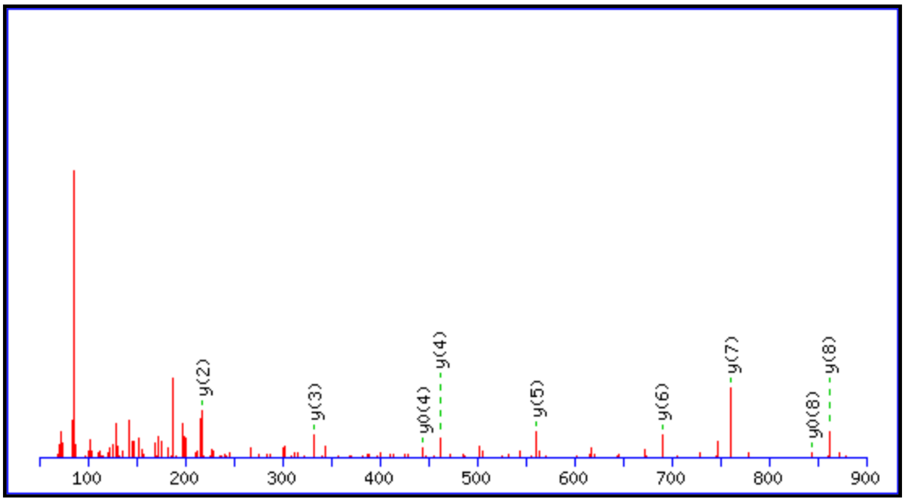

Monoisotopic mass of neutral peptide Mr(calc): 974.4920
Fixed modifications: Carbamidomethyl (C) (apply to specified residues or termini only)
Variable modifications:
N7 : Deamidated (NQ)
Ions Score: 47 **Expect:** 0.0042
Matches : 9/76 fragment ions using 18 most intense peaks (help)

#	b	b^{++}	b^{*}	b^{*++}	b^{0}	b^{0++}	Seq.	y	y^{++}	y^{*}	y^{*++}	y^{0}	y^{0++}	#
1	114.0913	57.5493					L							9
2	215.1390	108.0731			197.1285	99.0679	T	***862.4153***	431.7113	845.3887	423.1980	844.4047	422.7060	8
3	286.1761	143.5917			268.1656	134.5864	A	***761.3676***	381.1874	744.3410	372.6742	743.3570	372.1821	7
4	415.2187	208.1130			397.2082	199.1077	E	***690.3305***	345.6689	673.3039	337.1556	672.3199	336.6636	6
5	514.2871	257.6472			496.2766	248.6419	V	***561.2879***	281.1476	544.2613	272.6343	543.2773	272.1423	5
6	643.3297	322.1685			625.3192	313.1632	E	***462.2195***	231.6134	445.1929	223.1001	444.2089	222.6081	4
7	758.3567	379.6820	741.3301	371.1687	740.3461	370.6767	N	***333.1769***	167.0921	316.1503	158.5788			3
8	829.3938	415.2005	812.3672	406.6873	811.3832	406.1953	A	***218.1499***	109.5786	201.1234	101.0653			2
9							K	147.1128	74.0600	130.0863	65.5468			1

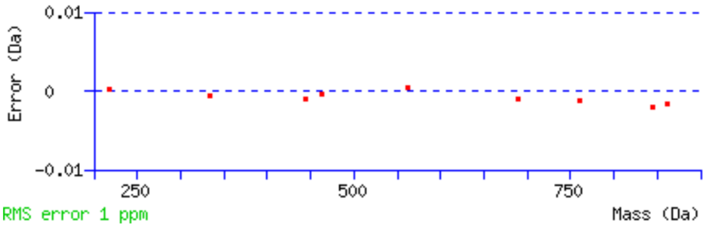

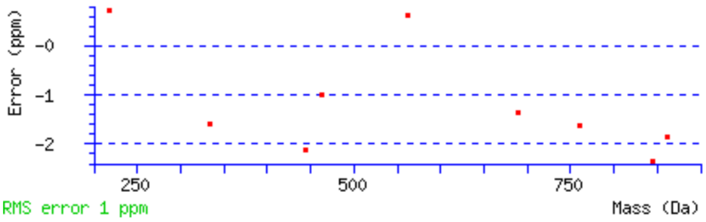

NCBI **BLAST** search of LTAEVENAK
(Parameters: blastp, nr protein database, expect=20000, no filter, PAM30)
Other BLAST web gateways

All matches to this query

Score	Mr(calc):	Delta	Sequence
47.1	974.4920	-0.0002	LTAEVENAK

**Mascot:** http://www.matrixscience.com/

Mascot Search Results

Peptide View

MS/MS Fragmentation of **LTAEVENAK**
Found in **sp|P78385|KRT83_HUMAN**, Keratin, type II cuticular Hb3 OS=Homo sapiens GN=KRT83 PE=1 SV=2

Match to Query 2409: 974.491788 from(488.253170,2+) intensity(3478429.2500) scans(3010) rtinseconds(642) index(16961)
Title: 160219_Sunil_SDSI_A_Spectrum070261_scans__3010_RTINSECONDS=642
Data file C:\\Sunil\\TKAP\\T\\T160219_Sunil_SDSI_A.mgf

Click mouse within plot area to zoom in by factor of two about that point
Or, Plot from 50 to 900 Da Full range
Label all possible matches ○ Label matches used for scoring ●

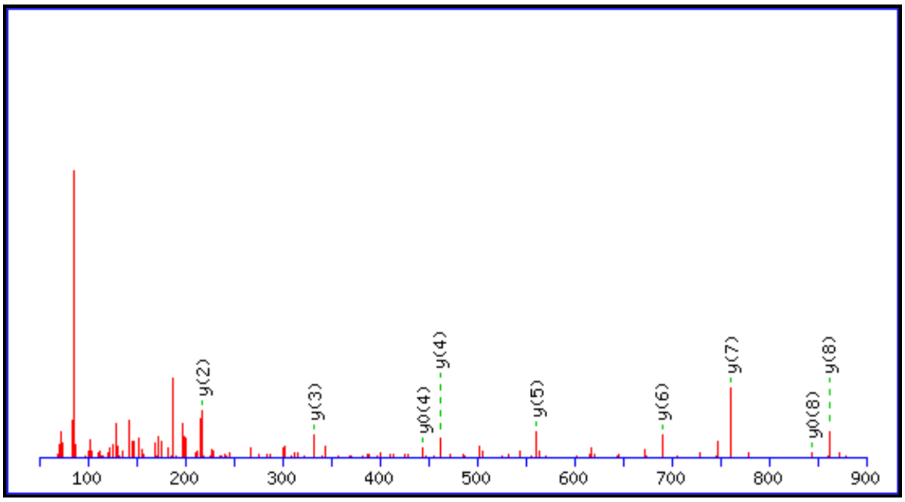

Monoisotopic mass of neutral peptide Mr(calc): 974.4920
Fixed modifications: Carbamidomethyl (C) (apply to specified residues or termini only)
Variable modifications:
N7 : Deamidated (NQ)
Ions Score: 47 **Expect:** 0.0042
Matches : 9/76 fragment ions using 18 most intense peaks (help)

#	b	b^{++}	b^{*}	b^{*++}	b^{0}	b^{0++}	Seq.	y	y^{++}	y^{*}	y^{*++}	y^{0}	y^{0++}	#
1	114.0913	57.5493					L							9
2	215.1390	108.0731			197.1285	99.0679	T	***862.4153***	431.7113	845.3887	423.1980	844.4047	422.7060	8
3	286.1761	143.5917			268.1656	134.5864	A	***761.3676***	381.1874	744.3410	372.6742	743.3570	372.1821	7
4	415.2187	208.1130			397.2082	199.1077	E	***690.3305***	345.6689	673.3039	337.1556	672.3199	336.6636	6
5	514.2871	257.6472			496.2766	248.6419	V	***561.2879***	281.1476	544.2613	272.6343	543.2773	272.1423	5
6	643.3297	322.1685			625.3192	313.1632	E	***462.2195***	231.6134	445.1929	223.1001	444.2089	222.6081	4
7	758.3567	379.6820	741.3301	371.1687	740.3461	370.6767	N	***333.1769***	167.0921	316.1503	158.5788			3
8	829.3938	415.2005	812.3672	406.6873	811.3832	406.1953	A	***218.1499***	109.5786	201.1234	101.0653			2
9							K	147.1128	74.0600	130.0863	65.5468			1

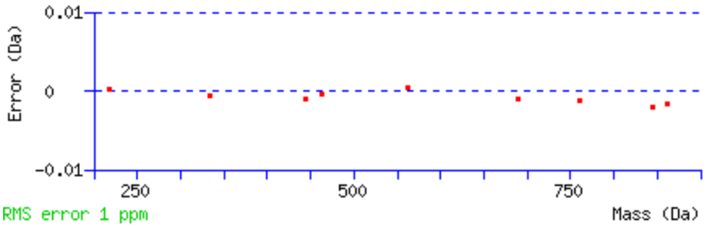

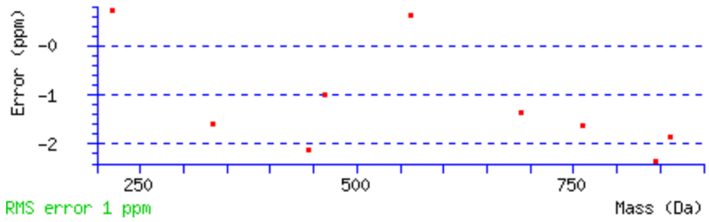

NCBI **BLAST** search of LTAEVENAK
(Parameters: blastp, nr protein database, expect=20000, no filter, PAM30)
Other BLAST web gateways

All matches to this query

Score	Mr(calc):	Delta	Sequence
47.1	974.4920	-0.0002	LTAEVENAK

Mascot: http://www.matrixscience.com/

Mascot Search Results

Peptide View

MS/MS Fragmentation of **LTAEVENAKCQNSK**
Found in **sp|O43790|KRT86_HUMAN**, Keratin, type II cuticular Hb6 OS=Homo sapiens GN=KRT86 PE=1 SV=1

Match to Query 20350: 1591.751352 from(531.591060,3+) intensity(809942.8750) scans(2226) rtinseconds(505) index(16250)
Title: 160219_Sunil_SDSI_A_Spectrum069550_scans__2226_RTINSECONDS=505
Data file C:\\Sunil\\TKAP\\T\\T160219_Sunil_SDSI_A.mgf

Click mouse within plot area to zoom in by factor of two about that point
Or, Plot from 100 to 1500 Da Full range
Label all possible matches ○ Label matches used for scoring ◉

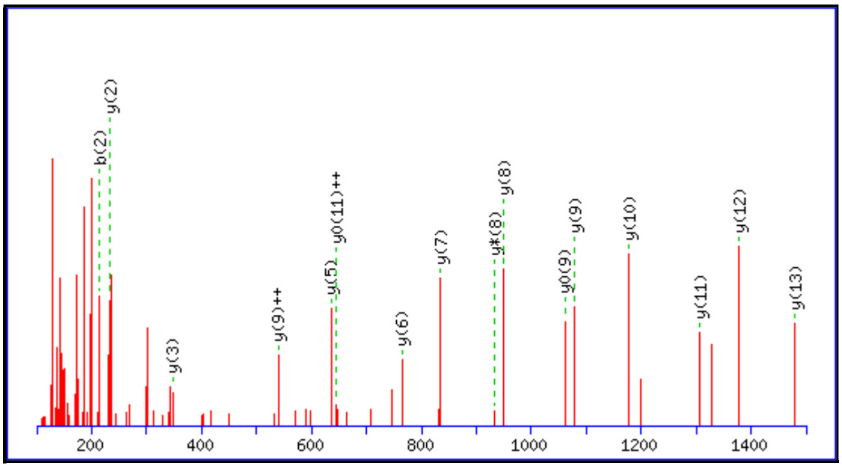

Monoisotopic mass of neutral peptide Mr(calc): 1591.7511
Fixed modifications: Carbamidomethyl (C) (apply to specified residues or termini only)
Variable modifications:
N7 : Deamidated (NQ)
Ions Score: 76 **Expect:** 4.8e-006
Matches : 16/140 fragment ions using 31 most intense peaks (help)

#	b	b^{++}	b*	b^{*++}	b^0	b^{0++}	Seq.	y	y^{++}	y*	y^{*++}	y^0	y^{0++}	#
1	114.0913	57.5493					L							14
2	**215.1390**	108.0731			197.1285	99.0679	T	***1479.6744***	740.3408	1462.6479	731.8276	1461.6638	731.3356	13
3	286.1761	143.5917			268.1656	134.5864	A	***1378.6267***	689.8170	1361.6002	681.3037	1360.6162	680.8117	12
4	415.2187	208.1130			397.2082	199.1077	E	***1307.5896***	654.2984	1290.5631	645.7852	1289.5790	645.2932	11
5	514.2871	257.6472			496.2766	248.6419	V	***1178.5470***	589.7771	1161.5205	581.2639	1160.5364	580.7719	10
6	643.3297	322.1685			625.3192	313.1632	E	***1079.4786***	540.2429	1062.4520	531.7297	1061.4680	531.2377	9
7	758.3567	379.6820	741.3301	371.1687	740.3461	370.6767	N	***950.4360***	475.7216	933.4095	467.2084	932.4254	466.7164	8
8	829.3938	415.2005	812.3672	406.6873	811.3832	406.1953	A	***835.4091***	418.2082	818.3825	409.6949	817.3985	409.2029	7
9	957.4888	479.2480	940.4622	470.7347	939.4782	470.2427	K	***764.3719***	382.6896	747.3454	374.1763	746.3614	373.6843	6
10	1117.5194	559.2633	1100.4929	550.7501	1099.5088	550.2581	C	***636.2770***	318.6421	619.2504	310.1289	618.2664	309.6368	5
11	1245.5780	623.2926	1228.5514	614.7794	1227.5674	614.2873	Q	476.2463	238.6268	459.2198	230.1135	458.2358	229.6215	4
12	1359.6209	680.3141	1342.5944	671.8008	1341.6103	671.3088	N	***348.1878***	174.5975	331.1612	166.0842	330.1772	165.5922	3
13	1446.6529	723.8301	1429.6264	715.3168	1428.6424	714.8248	S	***234.1448***	117.5761	217.1183	109.0628	216.1343	108.5708	2
14							K	147.1128	74.0600	130.0863	65.5468			1

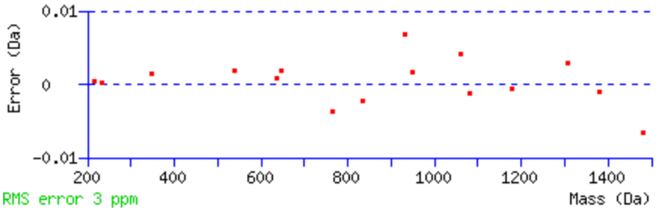

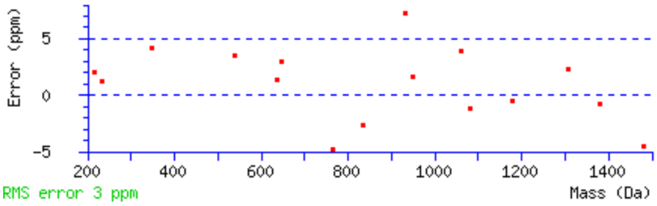

NCBI **BLAST** search of LTAEVENAKCQNSK
(Parameters: blastp, nr protein database, expect=20000, no filter, PAM30)
Other BLAST web gateways

All matches to this query

Score	Mr(calc):	Delta	Sequence
76.0	1591.7511	0.0002	LTAEVENAKCQNSK
38.2	1591.7511	0.0002	LTAEVENAKCQNSK
30.8	1591.7511	0.0002	LTAEVENAKCQNSK

Mascot: http://www.matrixscience.com/

Mascot Search Results

Peptide View

MS/MS Fragmentation of **LTAEVENAKCQNSK**
Found in **sp|O43790|KRT86_HUMAN**, Keratin, type II cuticular Hb6 OS=Homo sapiens GN=KRT86 PE=1 SV=1

Match to Query 20388: 1592.734632 from(531.918820,3+) intensity(1318796.5000) scans(2926) rtinseconds(655) index(1594)
Title: 160219_Sunil_SDSI_A_Spectrum053702_scans__2926_RTINSECONDS=655
Data file C:\\Sunil\\TKAP\\T\\T160219_Sunil_SDSI_A.mgf

Click mouse within plot area to zoom in by factor of two about that point
Or, Plot from 100 to 1500 Da Full range
Label all possible matches ○ Label matches used for scoring ◉

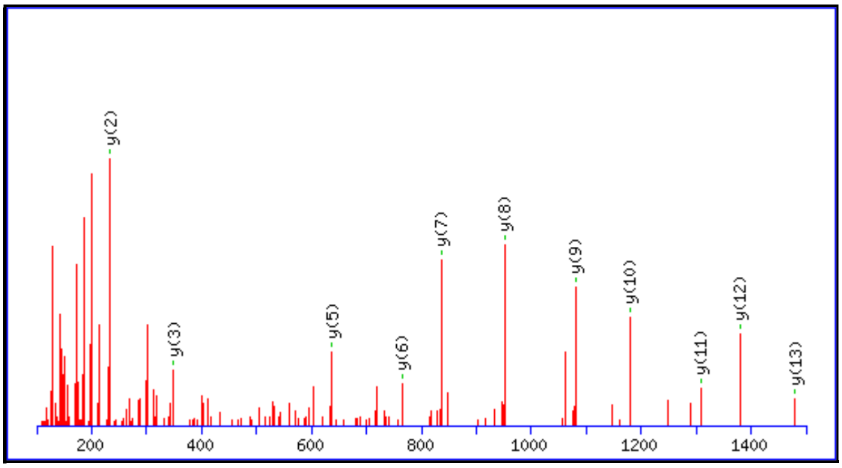

Monoisotopic mass of neutral peptide Mr(calc): 1592.7352
Fixed modifications: Carbamidomethyl (C) (apply to specified residues or termini only)
Variable modifications:
N7 : Deamidated (NQ)
N12 : Deamidated (NQ)
Ions Score: 105 **Expect:** 5.7e-009
Matches : 11/140 fragment ions using 14 most intense peaks (help)

#	b	b++	b*	b*++	b0	b0++	Seq.	y	y++	y*	y*++	y0	y0++	#
1	114.0913	57.5493					L							14
2	215.1390	108.0731			197.1285	99.0679	T	***1480.6584***	740.8328	1463.6319	732.3196	1462.6479	731.8276	13
3	286.1761	143.5917			268.1656	134.5864	A	***1379.6107***	690.3090	1362.5842	681.7957	1361.6002	681.3037	12
4	415.2187	208.1130			397.2082	199.1077	E	***1308.5736***	654.7904	1291.5471	646.2772	1290.5631	645.7852	11
5	514.2871	257.6472			496.2766	248.6419	V	***1179.5310***	590.2692	1162.5045	581.7559	1161.5205	581.2639	10
6	643.3297	322.1685			625.3192	313.1632	E	***1080.4626***	540.7349	1063.4361	532.2217	1062.4521	531.7297	9
7	758.3567	379.6820	741.3301	371.1687	740.3461	370.6767	N	***951.4200***	476.2136	934.3935	467.7004	933.4095	467.2084	8
8	829.3938	415.2005	812.3672	406.6873	811.3832	406.1953	A	***836.3931***	418.7002	819.3665	410.1869	818.3825	409.6949	7
9	957.4888	479.2480	940.4622	470.7347	939.4782	470.2427	K	***765.3560***	383.1816	748.3294	374.6683	747.3454	374.1763	6
10	1117.5194	559.2633	1100.4929	550.7501	1099.5088	550.2581	C	***637.2610***	319.1341	620.2345	310.6209	619.2504	310.1289	5
11	1245.5780	623.2926	1228.5514	614.7794	1227.5674	614.2873	Q	477.2304	239.1188	460.2038	230.6055	459.2198	230.1135	4
12	1360.6049	680.8061	1343.5784	672.2928	1342.5944	671.8008	N	***349.1718***	175.0895	332.1452	166.5763	331.1612	166.0842	3
13	1447.6370	724.3221	1430.6104	715.8088	1429.6264	715.3168	S	***234.1448***	117.5761	217.1183	109.0628	216.1343	108.5708	2
14							K	147.1128	74.0600	130.0863	65.5468			1

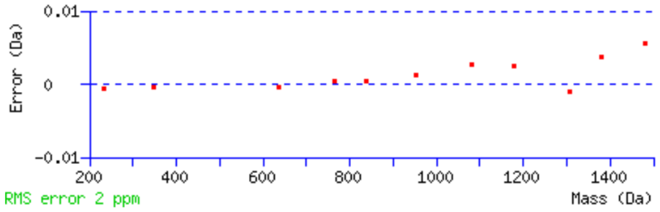

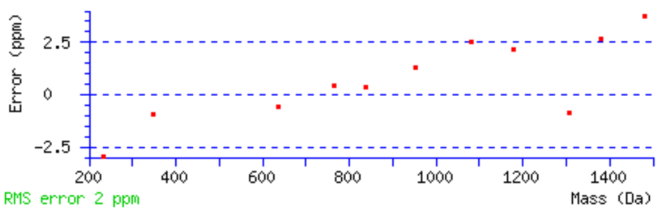

NCBI **BLAST** search of LTAEVENAKCQNSK
(Parameters: blastp, nr protein database, expect=20000, no filter, PAM30)

Other BLAST web gateways

All matches to this query

Score	Mr(calc):	Delta	Sequence
104.7	1592.7352	-0.0005	LTAEVENAKCQNSK
86.4	1592.7352	-0.0005	LTAEVENAKCQNSK
53.8	1592.7352	-0.0005	LTAEVENAKCQNSK

Mascot: http://www.matrixscience.com/

Mascot Search Results

Peptide View

MS/MS Fragmentation of **LTAEVENAKCQNSK**
Found in **sp|O43790|KRT86_HUMAN**, Keratin, type II cuticular Hb6 OS=Homo sapiens GN=KRT86 PE=1 SV=1

Match to Query 20388: 1592.734632 from(531.918820,3+) intensity(1318796.5000) scans(2926) rtinseconds(655) index(1594)
Title: 160219_Sunil_SDSI_A_Spectrum053702_scans__2926_RTINSECONDS=655
Data file C:\\Sunil\\TKAP\\T\\T160219_Sunil_SDSI_A.mgf

Click mouse within plot area to zoom in by factor of two about that point
Or, Plot from 100 to 1500 Da Full range
Label all possible matches ○ Label matches used for scoring ◉

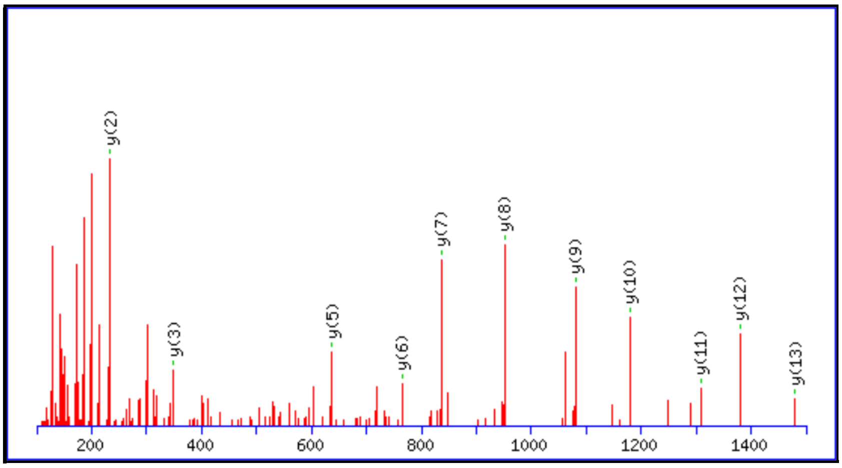

Monoisotopic mass of neutral peptide Mr(calc): 1592.7352
Fixed modifications: Carbamidomethyl (C) (apply to specified residues or termini only)
Variable modifications:
N7 : Deamidated (NQ)
N12 : Deamidated (NQ)
Ions Score: 105 **Expect:** 5.7e-009
Matches : 11/140 fragment ions using 14 most intense peaks (help)

#	b	b^{++}	b*	b^{*++}	b^0	b^{0++}	Seq.	y	y^{++}	y*	y^{*++}	y^0	y^{0++}	#
1	114.0913	57.5493					L							14
2	215.1390	108.0731			197.1285	99.0679	T	***1480.6584***	740.8328	1463.6319	732.3196	1462.6479	731.8276	13
3	286.1761	143.5917			268.1656	134.5864	A	***1379.6107***	690.3090	1362.5842	681.7957	1361.6002	681.3037	12
4	415.2187	208.1130			397.2082	199.1077	E	***1308.5736***	654.7904	1291.5471	646.2772	1290.5631	645.7852	11
5	514.2871	257.6472			496.2766	248.6419	V	***1179.5310***	590.2692	1162.5045	581.7559	1161.5205	581.2639	10
6	643.3297	322.1685			625.3192	313.1632	E	***1080.4626***	540.7349	1063.4361	532.2217	1062.4521	531.7297	9
7	758.3567	379.6820	741.3301	371.1687	740.3461	370.6767	N	***951.4200***	476.2136	934.3935	467.7004	933.4095	467.2084	8
8	829.3938	415.2005	812.3672	406.6873	811.3832	406.1953	A	***836.3931***	418.7002	819.3665	410.1869	818.3825	409.6949	7
9	957.4888	479.2480	940.4622	470.7347	939.4782	470.2427	K	***765.3560***	383.1816	748.3294	374.6683	747.3454	374.1763	6
10	1117.5194	559.2633	1100.4929	550.7501	1099.5088	550.2581	C	***637.2610***	319.1341	620.2345	310.6209	619.2504	310.1289	5
11	1245.5780	623.2926	1228.5514	614.7794	1227.5674	614.2873	Q	477.2304	239.1188	460.2038	230.6055	459.2198	230.1135	4
12	1360.6049	680.8061	1343.5784	672.2928	1342.5944	671.8008	N	***349.1718***	175.0895	332.1452	166.5763	331.1612	166.0842	3
13	1447.6370	724.3221	1430.6104	715.8088	1429.6264	715.3168	S	***234.1448***	117.5761	217.1183	109.0628	216.1343	108.5708	2
14							K	147.1128	74.0600	130.0863	65.5468			1

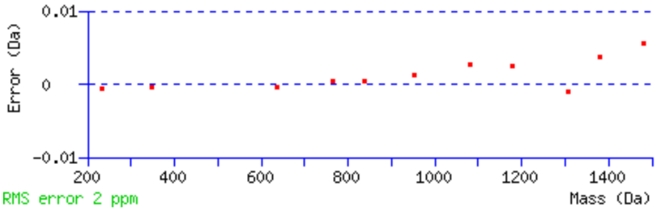

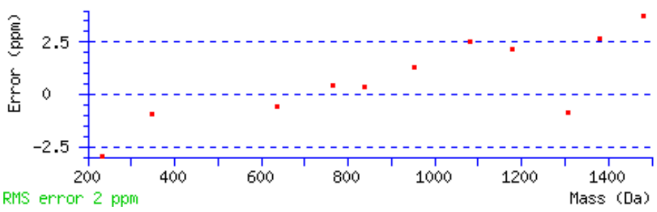

NCBI **BLAST** search of LTAEVENAKCQNSK
(Parameters: blastp, nr protein database, expect=20000, no filter, PAM30)

Other BLAST web gateways

All matches to this query

Score	Mr(calc):	Delta	Sequence
104.7	1592.7352	-0.0005	LTAEVENAKCQNSK
86.4	1592.7352	-0.0005	LTAEVENAKCQNSK
53.8	1592.7352	-0.0005	LTAEVENAKCQNSK

Mascot: http://www.matrixscience.com/

Mascot Search Results

Peptide View

MS/MS Fragmentation of **LTAEVENAKCQNSK**
Found in **sp|P78385|KRT83_HUMAN**, Keratin, type II cuticular Hb3 OS=Homo sapiens GN=KRT83 PE=1 SV=2

Match to Query 20388: 1592.734632 from(531.918820,3+) intensity(1318796.5000) scans(2926) rtinseconds(655) index(1594)
Title: 160219_Sunil_SDSI_A_Spectrum053702_scans__2926_RTINSECONDS=655
Data file C:\\Sunil\\TKAP\\T\\T160219_Sunil_SDSI_A.mgf

Click mouse within plot area to zoom in by factor of two about that point
Or, Plot from 100 to 1500 Da Full range
Label all possible matches ○ Label matches used for scoring ◉

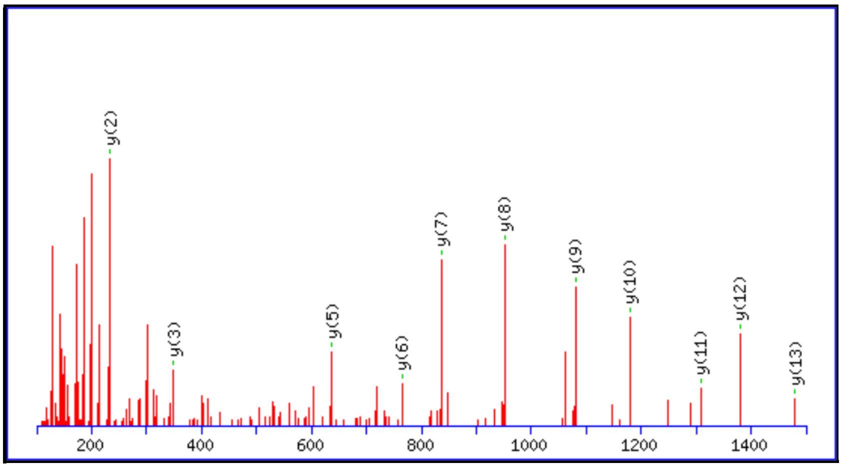

Monoisotopic mass of neutral peptide Mr(calc): 1592.7352
Fixed modifications: Carbamidomethyl (C) (apply to specified residues or termini only)
Variable modifications:
N7 : Deamidated (NQ)
N12 : Deamidated (NQ)
Ions Score: 105 **Expect:** 5.7e-009
Matches : 11/140 fragment ions using 14 most intense peaks (help)

#	b	b^{++}	b*	b^{*++}	b^0	b^{0++}	Seq.	y	y^{++}	y*	y^{*++}	y^0	y^{0++}	#
1	114.0913	57.5493					L							14
2	215.1390	108.0731			197.1285	99.0679	T	***1480.6584***	740.8328	1463.6319	732.3196	1462.6479	731.8276	13
3	286.1761	143.5917			268.1656	134.5864	A	***1379.6107***	690.3090	1362.5842	681.7957	1361.6002	681.3037	12
4	415.2187	208.1130			397.2082	199.1077	E	***1308.5736***	654.7904	1291.5471	646.2772	1290.5631	645.7852	11
5	514.2871	257.6472			496.2766	248.6419	V	***1179.5310***	590.2692	1162.5045	581.7559	1161.5205	581.2639	10
6	643.3297	322.1685			625.3192	313.1632	E	***1080.4626***	540.7349	1063.4361	532.2217	1062.4521	531.7297	9
7	758.3567	379.6820	741.3301	371.1687	740.3461	370.6767	N	***951.4200***	476.2136	934.3935	467.7004	933.4095	467.2084	8
8	829.3938	415.2005	812.3672	406.6873	811.3832	406.1953	A	***836.3931***	418.7002	819.3665	410.1869	818.3825	409.6949	7
9	957.4888	479.2480	940.4622	470.7347	939.4782	470.2427	K	***765.3560***	383.1816	748.3294	374.6683	747.3454	374.1763	6
10	1117.5194	559.2633	1100.4929	550.7501	1099.5088	550.2581	C	***637.2610***	319.1341	620.2345	310.6209	619.2504	310.1289	5
11	1245.5780	623.2926	1228.5514	614.7794	1227.5674	614.2873	Q	477.2304	239.1188	460.2038	230.6055	459.2198	230.1135	4
12	1360.6049	680.8061	1343.5784	672.2928	1342.5944	671.8008	N	***349.1718***	175.0895	332.1452	166.5763	331.1612	166.0842	3
13	1447.6370	724.3221	1430.6104	715.8088	1429.6264	715.3168	S	***234.1448***	117.5761	217.1183	109.0628	216.1343	108.5708	2
14							K	147.1128	74.0600	130.0863	65.5468			1

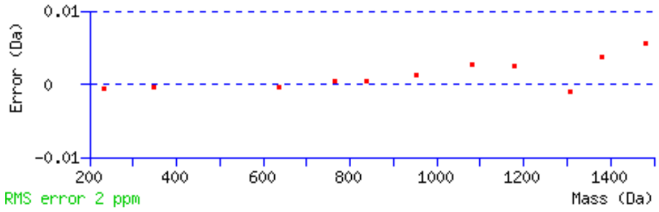

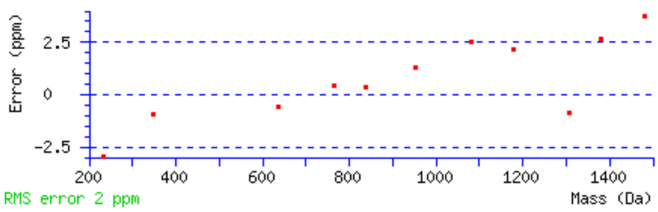

NCBI **BLAST** search of LTAEVENAKCQNSK
(Parameters: blastp, nr protein database, expect=20000, no filter, PAM30)

Other BLAST web gateways

All matches to this query

Score	Mr(calc):	Delta	Sequence
104.7	1592.7352	-0.0005	LTAEVENAKCQNSK
86.4	1592.7352	-0.0005	LTAEVENAKCQNSK
53.8	1592.7352	-0.0005	LTAEVENAKCQNSK

Mascot: http://www.matrixscience.com/

Mascot Search Results

Peptide View

MS/MS Fragmentation of **LTAEVENAKCQNSKLEAAVAQSEQQGEAALSDAR**
Found in **sp|O43790|KRT86_HUMAN**, Keratin, type II cuticular Hb6 OS=Homo sapiens GN=KRT86 PE=1 SV=1

Match to Query 43046: 3616.735242 from(1206.585690,3+) intensity(1846121.2500) scans(14282) rtinseconds(2609) index(11196)
Title: 160219_Sunil_SDSI_A_Spectrum063322_scans__14282_RTINSECONDS=2609
Data file C:\\Sunil\\TKAP\\T\\T160219_Sunil_SDSI_A.mgf

Click mouse within plot area to zoom in by factor of two about that point
Or, Plot from 200 to 3800 Da Full range
Label all possible matches Label matches used for scoring

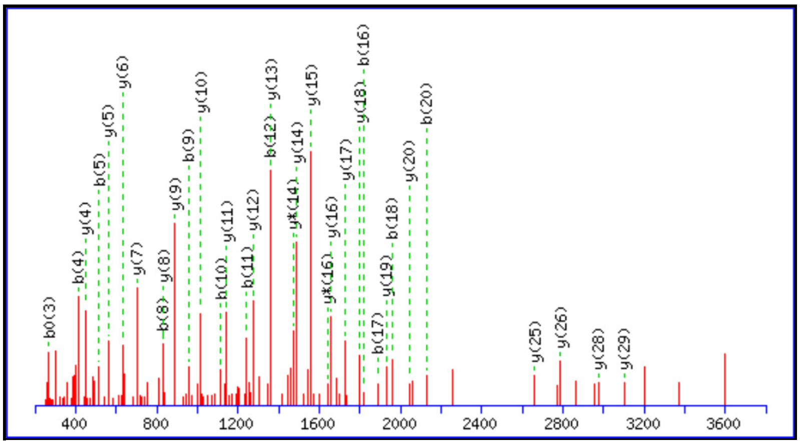

Monoisotopic mass of neutral peptide Mr(calc): 3616.7274
Fixed modifications: Carbamidomethyl (C) (apply to specified residues or termini only)
Variable modifications:
N12 : Deamidated (NQ)
Ions Score: 166 **Expect:** 6.5e-015
Matches : 35/378 fragment ions using 51 most intense peaks (help)

#	b	b++	b*	b*++	b0	b0++	Seq.	y	y++	y*	y*++	y0	y0++	#
1	114.0913	57.5493					L							34
2	215.1390	108.0731			197.1285	99.0679	T	3504.6507	1752.8290	3487.6242	1744.3157	3486.6401	1743.8237	33
3	286.1761	143.5917			268.1656	134.5864	A	3403.6030	1702.3052	3386.5765	1693.7919	3385.5925	1693.2999	32
4	**415.2187**	208.1130			397.2082	199.1077	E	3332.5659	1666.7866	3315.5394	1658.2733	3314.5554	1657.7813	31
5	**514.2871**	257.6472			496.2766	248.6419	V	3203.5233	1602.2653	3186.4968	1593.7520	3185.5128	1593.2600	30
6	643.3297	322.1685			625.3192	313.1632	E	***3104.4549***	1552.7311	3087.4284	1544.2178	3086.4443	1543.7258	29
7	757.3727	379.1900	740.3461	370.6767	739.3621	370.1847	N	***2975.4123***	1488.2098	2958.3858	1479.6965	2957.4018	1479.2045	28
8	**828.4098**	414.7085	811.3832	406.1953	810.3992	405.7032	A	2861.3694	1431.1883	2844.3428	1422.6751	2843.3588	1422.1831	27
9	**956.5047**	478.7560	939.4782	470.2427	938.4942	469.7507	K	***2790.3323***	1395.6698	2773.3057	1387.1565	2772.3217	1386.6645	26
10	**1116.5354**	558.7713	1099.5088	550.2581	1098.5248	549.7660	C	***2662.2373***	1331.6223	2645.2108	1323.1090	2644.2268	1322.6170	25
11	**1244.5940**	622.8006	1227.5674	614.2873	1226.5834	613.7953	Q	2502.2067	1251.6070	2485.1801	1243.0937	2484.1961	1242.6017	24
12	**1359.6209**	680.3141	1342.5944	671.8008	1341.6103	671.3088	N	2374.1481	1187.5777	2357.1215	1179.0644	2356.1375	1178.5724	23
13	1446.6529	723.8301	1429.6264	715.3168	1428.6424	714.8248	S	2259.1211	1130.0642	2242.0946	1121.5509	2241.1106	1121.0589	22
14	1574.7479	787.8776	1557.7213	779.3643	1556.7373	778.8723	K	2172.0891	1086.5482	2155.0626	1078.0349	2154.0786	1077.5429	21
15	1687.8320	844.4196	1670.8054	835.9063	1669.8214	835.4143	L	***2043.9942***	1022.5007	2026.9676	1013.9874	2025.9836	1013.4954	20
16	**1816.8746**	908.9409	1799.8480	900.4276	1798.8640	899.9356	E	***1930.9101***	965.9587	1913.8835	957.4454	1912.8995	956.9534	19
17	**1887.9117**	944.4595	1870.8851	935.9462	1869.9011	935.4542	A	***1801.8675***	901.4374	1784.8409	892.9241	1783.8569	892.4321	18
18	**1958.9488**	979.9780	1941.9222	971.4648	1940.9382	970.9727	A	***1730.8304***	865.9188	1713.8038	857.4056	1712.8198	856.9135	17
19	2058.0172	1029.5122	2040.9906	1020.9990	2040.0066	1020.5070	V	***1659.7933***	830.4003	1642.7667	821.8870	1641.7827	821.3950	16
20	**2129.0543**	1065.0308	2112.0278	1056.5175	2111.0437	1056.0255	A	***1560.7249***	780.8661	1543.6983	772.3528	1542.7143	771.8608	15
21	2257.1129	1129.0601	2240.0863	1120.5468	2239.1023	1120.0548	Q	***1489.6877***	745.3475	1472.6612	736.8342	1471.6772	736.3422	14
22	2344.1449	1172.5761	2327.1184	1164.0628	2326.1344	1163.5708	S	***1361.6292***	681.3182	1344.6026	672.8049	1343.6186	672.3129	13
23	2473.1875	1237.0974	2456.1610	1228.5841	2455.1769	1228.0921	E	***1274.5971***	637.8022	1257.5706	629.2889	1256.5866	628.7969	12
24	2601.2461	1301.1267	2584.2195	1292.6134	2583.2355	1292.1214	Q	***1145.5545***	573.2809	1128.5280	564.7676	1127.5440	564.2756	11
25	2729.3047	1365.1560	2712.2781	1356.6427	2711.2941	1356.1507	Q	***1017.4960***	509.2516	1000.4694	500.7383	999.4854	500.2463	10
26	2786.3261	1393.6667	2769.2996	1385.1534	2768.3156	1384.6614	G	***889.4374***	445.2223	872.4108	436.7091	871.4268	436.2170	9
27	2915.3687	1458.1880	2898.3422	1449.6747	2897.3582	1449.1827	E	***832.4159***	416.7116	815.3894	408.1983	814.4054	407.7063	8

**28**	2986.4058	1493.7066	2969.3793	1485.1933	2968.3953	1484.7013	**A**	***703.3733***	352.1903	686.3468	343.6770	685.3628	343.1850	**7**
**29**	3057.4430	1529.2251	3040.4164	1520.7118	3039.4324	1520.2198	**A**	***632.3362***	316.6717	615.3097	308.1585	614.3257	307.6665	**6**
**30**	3170.5270	1585.7671	3153.5005	1577.2539	3152.5165	1576.7619	**L**	***561.2991***	281.1532	544.2726	272.6399	543.2885	272.1479	**5**
**31**	3257.5590	1629.2832	3240.5325	1620.7699	3239.5485	1620.2779	**S**	***448.2150***	224.6112	431.1885	216.0979	430.2045	215.6059	**4**
**32**	3372.5860	1686.7966	3355.5594	1678.2834	3354.5754	1677.7913	**D**	361.1830	181.0951	344.1565	172.5819	343.1724	172.0899	**3**
**33**	3443.6231	1722.3152	3426.5966	1713.8019	3425.6125	1713.3099	**A**	246.1561	123.5817	229.1295	115.0684			**2**
**34**							**R**	175.1190	88.0631	158.0924	79.5498			**1**

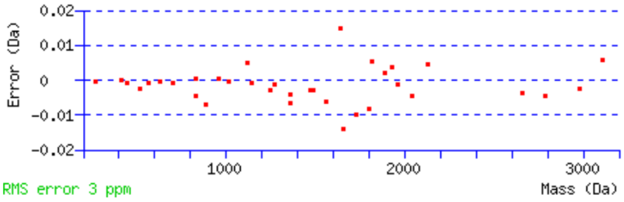

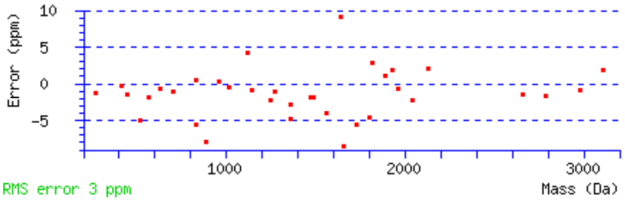

NCBI **BLAST** search of LTAEVENAKCQNSKLEAAVAQSEQQGEAALSDAR
(Parameters: blastp, nr protein database, expect=20000, no filter, PAM30)
Other BLAST web gateways

All matches to this query

Score	Mr(calc):	Delta	Sequence
165.6	3616.7274	0.0078	LTAEVENAKCQNSKLEAAVAQSEQQGEAALSDAR
162.5	3616.7274	0.0078	LTAEVENAKCQNSKLEAAVAQSEQQGEAALSDAR
126.2	3616.7274	0.0078	LTAEVENAKCQNSKLEAAVAQSEQQGEAALSDAR
98.8	3615.7434	0.9918	LTAEVENAKCQNSKLEAAVAQSEQQGEAALSDAR
77.3	3616.7274	0.0078	LTAEVENAKCQNSKLEAAVAQSEQQGEAALSDAR
49.4	3616.7274	0.0078	LTAEVENAKCQNSKLEAAVAQSEQQGEAALSDAR
42.3	3616.7274	0.0078	LTAEVENAKCQNSKLEAAVAQSEQQGEAALSDAR

Mascot: http://www.matrixscience.com/

Mascot Search Results

Peptide View

MS/MS Fragmentation of **LTAEVENAKCQNSKLEAAVAQSEQQGEAALSDAR**
Found in **sp|O43790|KRT86_HUMAN**, Keratin, type II cuticular Hb6 OS=Homo sapiens GN=KRT86 PE=1 SV=1

Match to Query 43044: 3616.730896 from(905.190000,4+) intensity(4660492.0000) scans(14006) rtinseconds(2536) index(26323)
Title: 160219_Sunil_SDSI_A_Spectrum079629_scans__14006_RTINSECONDS=2536
Data file C:\\Sunil\\TKAP\\T\\T160219_Sunil_SDSI_A.mgf

Click mouse within plot area to zoom in by factor of two about that point
Or, Plot from 200 to 2800 Da Full range
Label all possible matches ○ Label matches used for scoring ◉

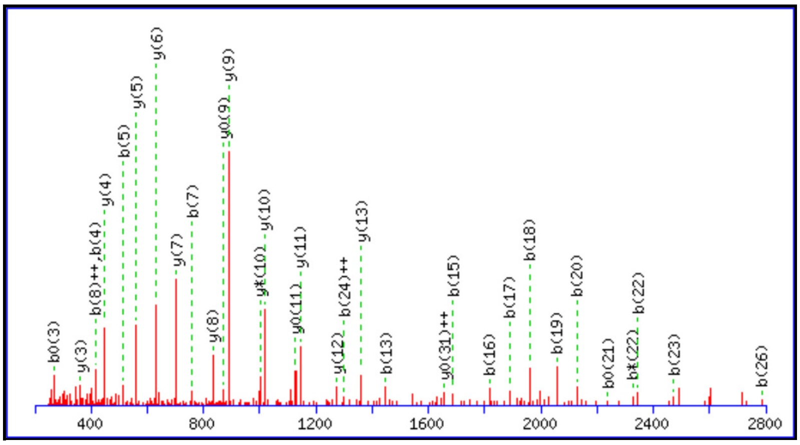

Monoisotopic mass of neutral peptide Mr(calc): 3616.7274
Fixed modifications: Carbamidomethyl (C) (apply to specified residues or termini only)
Variable modifications:
N7 : Deamidated (NQ)
Ions Score: 99 **Expect:** 3.2e-008
Matches : 33/378 fragment ions using 49 most intense peaks (help)

#	b	b++	b*	b*++	b0	b0++	Seq.	y	y++	y*	y*++	y0	y0++	#
1	114.0913	57.5493					L							34
2	215.1390	108.0731			197.1285	99.0679	T	3504.6507	1752.8290	3487.6242	1744.3157	3486.6401	1743.8237	33
3	286.1761	143.5917			268.1656	134.5864	A	3403.6030	1702.3052	3386.5765	1693.7919	3385.5925	1693.2999	32
4	***415.2187***	208.1130			397.2082	199.1077	E	3332.5659	1666.7866	3315.5394	1658.2733	3314.5554	1657.7813	31
5	***514.2871***	257.6472			496.2766	248.6419	V	3203.5233	1602.2653	3186.4968	1593.7520	3185.5128	1593.2600	30
6	643.3297	322.1685			625.3192	313.1632	E	3104.4549	1552.7311	3087.4284	1544.2178	3086.4443	1543.7258	29
7	***758.3567***	379.6820	741.3301	371.1687	740.3461	370.6767	N	2975.4123	1488.2098	2958.3858	1479.6965	2957.4018	1479.2045	28
8	829.3938	**415.2005**	812.3672	406.6873	811.3832	406.1953	A	2860.3854	1430.6963	2843.3588	1422.1831	2842.3748	1421.6910	27
9	957.4888	479.2480	940.4622	470.7347	939.4782	470.2427	K	2789.3483	1395.1778	2772.3217	1386.6645	2771.3377	1386.1725	26
10	1117.5194	559.2633	1100.4929	550.7501	1099.5088	550.2581	C	2661.2533	1331.1303	2644.2268	1322.6170	2643.2427	1322.1250	25
11	1245.5780	623.2926	1228.5514	614.7794	1227.5674	614.2873	Q	2501.2227	1251.1150	2484.1961	1242.6017	2483.2121	1242.1097	24
12	1359.6209	680.3141	1342.5944	671.8008	1341.6103	671.3088	N	2373.1641	1187.0857	2356.1375	1178.5724	2355.1535	1178.0804	23
13	***1446.6529***	723.8301	1429.6264	715.3168	1428.6424	714.8248	S	2259.1211	1130.0642	2242.0946	1121.5509	2241.1106	1121.0589	22
14	1574.7479	787.8776	1557.7213	779.3643	1556.7373	778.8723	K	2172.0891	1086.5482	2155.0626	1078.0349	2154.0786	1077.5429	21
15	***1687.8320***	844.4196	1670.8054	835.9063	1669.8214	835.4143	L	2043.9942	1022.5007	2026.9676	1013.9874	2025.9836	1013.4954	20
16	***1816.8746***	908.9409	1799.8480	900.4276	1798.8640	899.9356	E	1930.9101	965.9587	1913.8835	957.4454	1912.8995	956.9534	19
17	***1887.9117***	944.4595	1870.8851	935.9462	1869.9011	935.4542	A	1801.8675	901.4374	1784.8409	892.9241	1783.8569	892.4321	18
18	***1958.9488***	979.9780	1941.9222	971.4648	1940.9382	970.9727	A	1730.8304	865.9188	1713.8038	857.4056	1712.8198	856.9135	17
19	***2058.0172***	1029.5122	2040.9906	1020.9990	2040.0066	1020.5070	V	1659.7933	830.4003	1642.7667	821.8870	1641.7827	821.3950	16
20	***2129.0543***	1065.0308	2112.0278	1056.5175	2111.0437	1056.0255	A	1560.7249	780.8661	1543.6983	772.3528	1542.7143	771.8608	15
21	2257.1129	1129.0601	2240.0863	1120.5468	2239.1023	1120.0548	Q	1489.6877	745.3475	1472.6612	736.8342	1471.6772	736.3422	14
22	***2344.1449***	1172.5761	2327.1184	1164.0628	2326.1344	1163.5708	S	***1361.6292***	681.3182	1344.6026	672.8049	1343.6186	672.3129	13
23	***2473.1875***	1237.0974	2456.1610	1228.5841	2455.1769	1228.0921	E	***1274.5971***	637.8022	1257.5706	629.2889	1256.5866	628.7969	12
24	2601.2461	**1301.1267**	2584.2195	1292.6134	2583.2355	1292.1214	Q	***1145.5545***	573.2809	1128.5280	564.7676	1127.5440	564.2756	11
25	2729.3047	1365.1560	2712.2781	1356.6427	2711.2941	1356.1507	Q	***1017.4960***	509.2516	1000.4694	500.7383	999.4854	500.2463	10
26	***2786.3261***	1393.6667	2769.2996	1385.1534	2768.3156	1384.6614	G	***889.4374***	445.2223	872.4108	436.7091	871.4268	436.2170	9
27	2915.3687	1458.1880	2898.3422	1449.6747	2897.3582	1449.1827	E	***832.4159***	416.7116	815.3894	408.1983	814.4054	407.7063	8

**28**	2986.4058	1493.7066	2969.3793	1485.1933	2968.3953	1484.7013	**A**	***703.3733***	352.1903	686.3468	343.6770	685.3628	343.1850	**7**
**29**	3057.4430	1529.2251	3040.4164	1520.7118	3039.4324	1520.2198	**A**	***632.3362***	316.6717	615.3097	308.1585	614.3257	307.6665	**6**
**30**	3170.5270	1585.7671	3153.5005	1577.2539	3152.5165	1576.7619	**L**	***561.2991***	281.1532	544.2726	272.6399	543.2885	272.1479	**5**
**31**	3257.5590	1629.2832	3240.5325	1620.7699	3239.5485	1620.2779	**S**	***448.2150***	224.6112	431.1885	216.0979	430.2045	215.6059	**4**
**32**	3372.5860	1686.7966	3355.5594	1678.2834	3354.5754	1677.7913	**D**	***361.1830***	181.0951	344.1565	172.5819	343.1724	172.0899	**3**
**33**	3443.6231	1722.3152	3426.5966	1713.8019	3425.6125	1713.3099	**A**	246.1561	123.5817	229.1295	115.0684			**2**
**34**							**R**	175.1190	88.0631	158.0924	79.5498			**1**

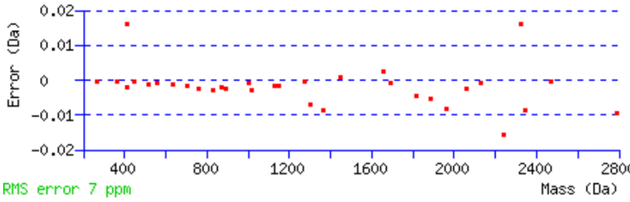

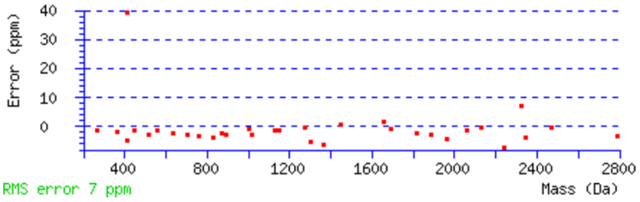

NCBI **BLAST** search of LTAEVENAKCQNSKLEAAVAQSEQQGEAALSDAR
(Parameters: blastp, nr protein database, expect=20000, no filter, PAM30)
Other BLAST web gateways

All matches to this query

Score	Mr(calc):	Delta	Sequence
98.6	3616.7274	0.0035	LTAEVENAKCQNSKLEAAVAQSEQQGEAALSDAR
95.7	3616.7274	0.0035	LTAEVENAKCQNSKLEAAVAQSEQQGEAALSDAR
93.3	3616.7274	0.0035	LTAEVENAKCQNSKLEAAVAQSEQQGEAALSDAR
45.8	3616.7274	0.0035	LTAEVENAKCQNSKLEAAVAQSEQQGEAALSDAR
41.7	3615.7434	0.9875	LTAEVENAKCQNSKLEAAVAQSEQQGEAALSDAR
21.7	3616.7274	0.0035	LTAEVENAKCQNSKLEAAVAQSEQQGEAALSDAR
15.5	3616.7274	0.0035	LTAEVENAKCQNSKLEAAVAQSEQQGEAALSDAR

Mascot: http://www.matrixscience.com/

Mascot Search Results

Peptide View

MS/MS Fragmentation of **LTAEVENAKCQNSKLEAAVAQSEQQGEAALSDAR**
Found in **sp|P78385|KRT83_HUMAN**, Keratin, type II cuticular Hb3 OS=Homo sapiens GN=KRT83 PE=1 SV=2

Match to Query 43046: 3616.735242 from(1206.585690,3+) intensity(1846121.2500) scans(14282) rtinseconds(2609) index(11196)
Title: 160219_Sunil_SDSI_A_Spectrum063322_scans__14282_RTINSECONDS=2609
Data file C:\\Sunil\\TKAP\\T\\T160219_Sunil_SDSI_A.mgf

Click mouse within plot area to zoom in by factor of two about that point
Or, Plot from 200 to 3800 Da Full range
Label all possible matches ○ Label matches used for scoring ◉

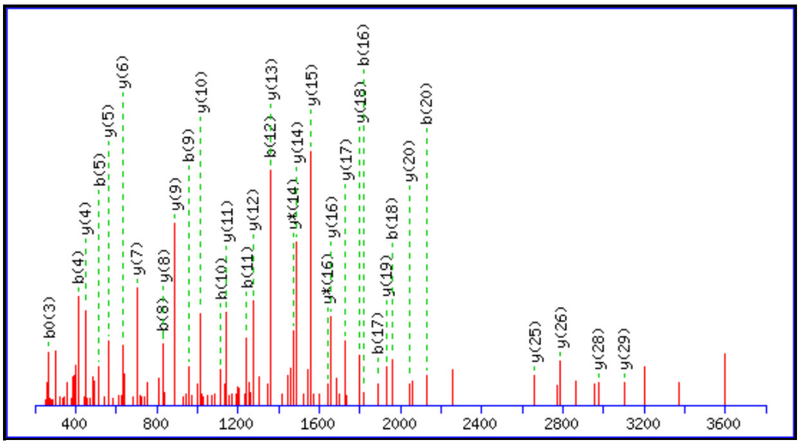

Monoisotopic mass of neutral peptide Mr(calc): 3616.7274
Fixed modifications: Carbamidomethyl (C) (apply to specified residues or termini only)
Variable modifications:
N12 : Deamidated (NQ)
Ions Score: 166 **Expect:** 6.5e-015
Matches : 35/378 fragment ions using 51 most intense peaks (help)

#	b	b++	b*	b*++	b0	b0++	Seq.	y	y++	y*	y*++	y0	y0++	#
1	114.0913	57.5493					L							34
2	215.1390	108.0731			197.1285	99.0679	T	3504.6507	1752.8290	3487.6242	1744.3157	3486.6401	1743.8237	33
3	286.1761	143.5917			268.1656	134.5864	A	3403.6030	1702.3052	3386.5765	1693.7919	3385.5925	1693.2999	32
4	**415.2187**	208.1130			397.2082	199.1077	E	3332.5659	1666.7866	3315.5394	1658.2733	3314.5554	1657.7813	31
5	**514.2871**	257.6472			496.2766	248.6419	V	3203.5233	1602.2653	3186.4968	1593.7520	3185.5128	1593.2600	30
6	643.3297	322.1685			625.3192	313.1632	E	***3104.4549***	1552.7311	3087.4284	1544.2178	3086.4443	1543.7258	29
7	757.3727	379.1900	740.3461	370.6767	739.3621	370.1847	N	***2975.4123***	1488.2098	2958.3858	1479.6965	2957.4018	1479.2045	28
8	**828.4098**	414.7085	811.3832	406.1953	810.3992	405.7032	A	2861.3694	1431.1883	2844.3428	1422.6751	2843.3588	1422.1831	27
9	**956.5047**	478.7560	939.4782	470.2427	938.4942	469.7507	K	***2790.3323***	1395.6698	2773.3057	1387.1565	2772.3217	1386.6645	26
10	**1116.5354**	558.7713	1099.5088	550.2581	1098.5248	549.7660	C	***2662.2373***	1331.6223	2645.2108	1323.1090	2644.2268	1322.6170	25
11	**1244.5940**	622.8006	1227.5674	614.2873	1226.5834	613.7953	Q	2502.2067	1251.6070	2485.1801	1243.0937	2484.1961	1242.6017	24
12	**1359.6209**	680.3141	1342.5944	671.8008	1341.6103	671.3088	N	2374.1481	1187.5777	2357.1215	1179.0644	2356.1375	1178.5724	23
13	1446.6529	723.8301	1429.6264	715.3168	1428.6424	714.8248	S	2259.1211	1130.0642	2242.0946	1121.5509	2241.1106	1121.0589	22
14	1574.7479	787.8776	1557.7213	779.3643	1556.7373	778.8723	K	2172.0891	1086.5482	2155.0626	1078.0349	2154.0786	1077.5429	21
15	1687.8320	844.4196	1670.8054	835.9063	1669.8214	835.4143	L	***2043.9942***	1022.5007	2026.9676	1013.9874	2025.9836	1013.4954	20
16	**1816.8746**	908.9409	1799.8480	900.4276	1798.8640	899.9356	E	***1930.9101***	965.9587	1913.8835	957.4454	1912.8995	956.9534	19
17	**1887.9117**	944.4595	1870.8851	935.9462	1869.9011	935.4542	A	***1801.8675***	901.4374	1784.8409	892.9241	1783.8569	892.4321	18
18	**1958.9488**	979.9780	1941.9222	971.4648	1940.9382	970.9727	A	***1730.8304***	865.9188	1713.8038	857.4056	1712.8198	856.9135	17
19	2058.0172	1029.5122	2040.9906	1020.9990	2040.0066	1020.5070	V	***1659.7933***	830.4003	1642.7667	821.8870	1641.7827	821.3950	16
20	**2129.0543**	1065.0308	2112.0278	1056.5175	2111.0437	1056.0255	A	***1560.7249***	780.8661	1543.6983	772.3528	1542.7143	771.8608	15
21	2257.1129	1129.0601	2240.0863	1120.5468	2239.1023	1120.0548	Q	***1489.6877***	745.3475	1472.6612	736.8342	1471.6772	736.3422	14
22	2344.1449	1172.5761	2327.1184	1164.0628	2326.1344	1163.5708	S	***1361.6292***	681.3182	1344.6026	672.8049	1343.6186	672.3129	13
23	2473.1875	1237.0974	2456.1610	1228.5841	2455.1769	1228.0921	E	***1274.5971***	637.8022	1257.5706	629.2889	1256.5866	628.7969	12
24	2601.2461	1301.1267	2584.2195	1292.6134	2583.2355	1292.1214	Q	***1145.5545***	573.2809	1128.5280	564.7676	1127.5440	564.2756	11
25	2729.3047	1365.1560	2712.2781	1356.6427	2711.2941	1356.1507	Q	***1017.4960***	509.2516	1000.4694	500.7383	999.4854	500.2463	10
26	2786.3261	1393.6667	2769.2996	1385.1534	2768.3156	1384.6614	G	***889.4374***	445.2223	872.4108	436.7091	871.4268	436.2170	9
27	2915.3687	1458.1880	2898.3422	1449.6747	2897.3582	1449.1827	E	***832.4159***	416.7116	815.3894	408.1983	814.4054	407.7063	8

**28**	2986.4058	1493.7066	2969.3793	1485.1933	2968.3953	1484.7013	**A**	***703.3733***	352.1903	686.3468	343.6770	685.3628	343.1850	**7**
**29**	3057.4430	1529.2251	3040.4164	1520.7118	3039.4324	1520.2198	**A**	***632.3362***	316.6717	615.3097	308.1585	614.3257	307.6665	**6**
**30**	3170.5270	1585.7671	3153.5005	1577.2539	3152.5165	1576.7619	**L**	***561.2991***	281.1532	544.2726	272.6399	543.2885	272.1479	**5**
**31**	3257.5590	1629.2832	3240.5325	1620.7699	3239.5485	1620.2779	**S**	***448.2150***	224.6112	431.1885	216.0979	430.2045	215.6059	**4**
**32**	3372.5860	1686.7966	3355.5594	1678.2834	3354.5754	1677.7913	**D**	361.1830	181.0951	344.1565	172.5819	343.1724	172.0899	**3**
**33**	3443.6231	1722.3152	3426.5966	1713.8019	3425.6125	1713.3099	**A**	246.1561	123.5817	229.1295	115.0684			**2**
**34**							**R**	175.1190	88.0631	158.0924	79.5498			**1**

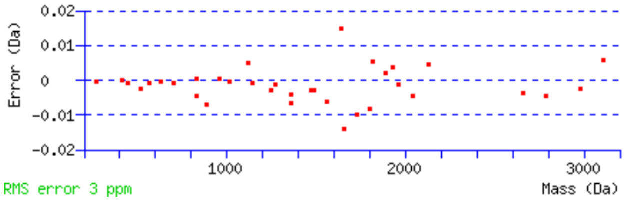

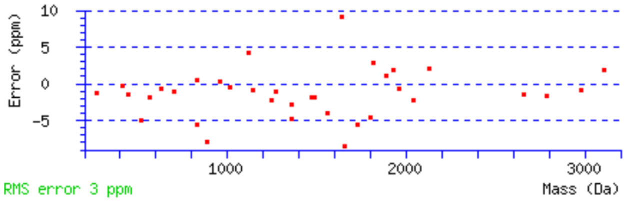

NCBI **BLAST** search of LTAEVENAKCQNSKLEAAVAQSEQQGEAALSDAR
(Parameters: blastp, nr protein database, expect=20000, no filter, PAM30)
Other BLAST web gateways

All matches to this query

Score	Mr(calc):	Delta	Sequence
165.6	3616.7274	0.0078	LTAEVENAKCQNSKLEAAVAQSEQQGEAALSDAR
162.5	3616.7274	0.0078	LTAEVENAKCQNSKLEAAVAQSEQQGEAALSDAR
126.2	3616.7274	0.0078	LTAEVENAKCQNSKLEAAVAQSEQQGEAALSDAR
98.8	3615.7434	0.9918	LTAEVENAKCQNSKLEAAVAQSEQQGEAALSDAR
77.3	3616.7274	0.0078	LTAEVENAKCQNSKLEAAVAQSEQQGEAALSDAR
49.4	3616.7274	0.0078	LTAEVENAKCQNSKLEAAVAQSEQQGEAALSDAR
42.3	3616.7274	0.0078	LTAEVENAKCQNSKLEAAVAQSEQQGEAALSDAR

Mascot: http://www.matrixscience.com/

Mascot Search Results

Peptide View

MS/MS Fragmentation of **LVVQIDNAK**
Found in **sp|Q15323|K1H1_HUMAN**, Keratin, type I cuticular Ha1 OS=Homo sapiens GN=KRT31 PE=2 SV=3

Match to Query 2885: 999.556068 from(500.785310,2+) intensity(15853963.0000) scans(7408) rtinseconds(1403) index(20752)
Title: 160219_Sunil_SDSI_A_Spectrum074056_scans__7408_RTINSECONDS=1403
Data file C:\\Sunil\\TKAP\\T\\T160219_Sunil_SDSI_A.mgf

Click mouse within plot area to zoom in by factor of two about that point
Or, Plot from 50 to 900 Da Full range
Label all possible matches ○ Label matches used for scoring ◉

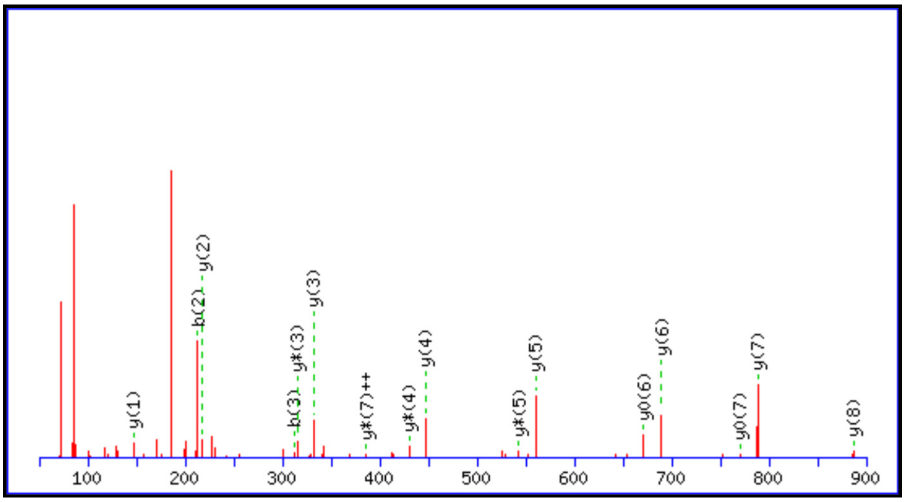

Monoisotopic mass of neutral peptide Mr(calc): 999.5600
Fixed modifications: Carbamidomethyl (C) (apply to specified residues or termini only)
Variable modifications:
Q4 : Deamidated (NQ)
Ions Score: 46 **Expect:** 0.0035
Matches : 16/74 fragment ions using 37 most intense peaks (help)

#	b	b^{++}	b*	b^{*++}	b^{0}	b^{0++}	Seq.	y	y^{++}	y*	y^{*++}	y^{0}	y^{0++}	#
1	114.0913	57.5493					L							9
2	**213.1598**	107.0835					V	**887.4833**	444.2453	870.4567	435.7320	869.4727	435.2400	8
3	**312.2282**	156.6177					V	**788.4149**	394.7111	771.3883	386.1978	770.4043	385.7058	7
4	441.2708	221.1390	424.2442	212.6257			Q	**689.3464**	345.1769	672.3199	336.6636	671.3359	336.1716	6
5	554.3548	277.6811	537.3283	269.1678			I	**560.3039**	280.6556	543.2773	272.1423	542.2933	271.6503	5
6	669.3818	335.1945	652.3552	326.6812	651.3712	326.1892	D	**447.2198**	224.1135	430.1932	215.6003	429.2092	215.1082	4
7	783.4247	392.2160	766.3981	383.7027	765.4141	383.2107	N	**332.1928**	166.6001	315.1663	158.0868			3
8	854.4618	427.7345	837.4353	419.2213	836.4512	418.7293	A	**218.1499**	109.5786	201.1234	101.0653			2
9							K	**147.1128**	74.0600	130.0863	65.5468			1

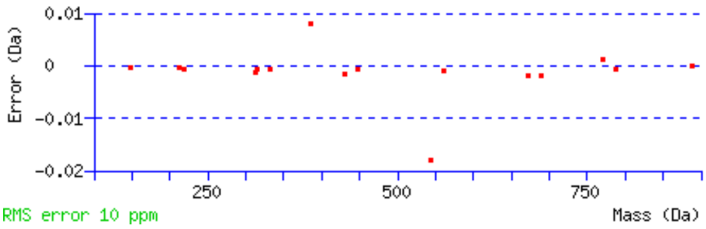

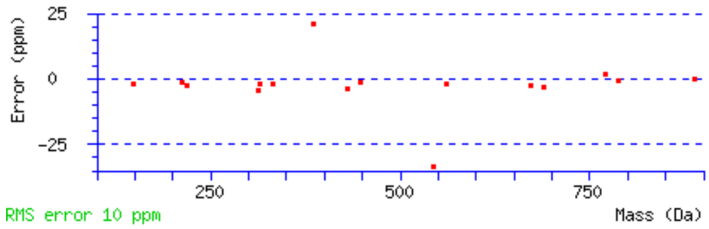

NCBI **BLAST** search of LVVQIDNAK
(Parameters: blastp, nr protein database, expect=20000, no filter, PAM30)
Other BLAST web gateways

All matches to this query

Score	Mr(calc):	Delta	Sequence
46.1	999.5600	-0.0040	LVVEIDNAK
46.1	999.5600	-0.0040	LVVQIDNAK
13.1	999.5600	-0.0040	LVVQIDNAK
6.3	999.5461	0.0100	NVEAVRNAK
3.9	999.5614	-0.0053	RGNLLGGWK

Mascot: http://www.matrixscience.com/

Mascot Search Results

Peptide View

MS/MS Fragmentation of **LVVQIDNAK**
Found in **sp|Q15323|K1H1_HUMAN**, Keratin, type I cuticular Ha1 OS=Homo sapiens GN=KRT31 PE=2 SV=3

Match to Query 2892: 999.558808 from(500.786680,2+) intensity(33847728.0000) scans(7919) rtinseconds(1491) index(21214)
Title: 160219_Sunil_SDSI_A_Spectrum074518_scans__7919_RTINSECONDS=1491
Data file C:\\Sunil\\TKAP\\T\\T160219_Sunil_SDSI_A.mgf

Click mouse within plot area to zoom in by factor of two about that point
Or, Plot from 50 to 900 Da Full range
Label all possible matches Label matches used for scoring

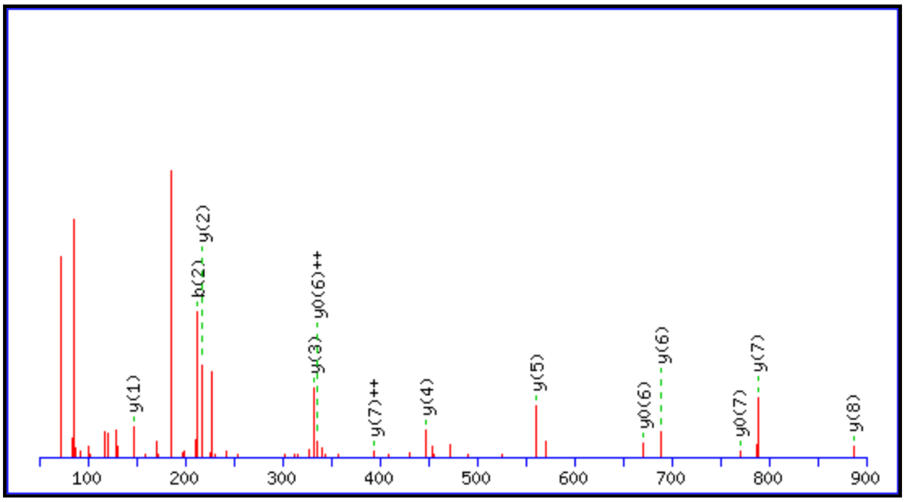

Monoisotopic mass of neutral peptide Mr(calc): 999.5600
Fixed modifications: Carbamidomethyl (C) (apply to specified residues or termini only)
Variable modifications:
N7 : Deamidated (NQ)
Ions Score: 61 **Expect:** 8.2e-005
Matches : 13/74 fragment ions using 22 most intense peaks (help)

#	b	b^{++}	b*	b^{*++}	b^{0}	b^{0++}	Seq.	y	y^{++}	y*	y^{*++}	y^{0}	y^{0++}	#
1	114.0913	57.5493					L							9
2	**213.1598**	107.0835					V	***887.4833***	444.2453	870.4567	435.7320	869.4727	435.2400	8
3	312.2282	156.6177					V	***788.4149***	394.7111	771.3883	386.1978	770.4043	385.7058	7
4	440.2867	220.6470	423.2602	212.1337			Q	***689.3464***	345.1769	672.3199	336.6636	671.3359	336.1716	6
5	553.3708	277.1890	536.3443	268.6758			I	***561.2879***	281.1476	544.2613	272.6343	543.2773	272.1423	5
6	668.3978	334.7025	651.3712	326.1892	650.3872	325.6972	D	***448.2038***	224.6055	431.1773	216.0923	430.1932	215.6003	4
7	783.4247	392.2160	766.3981	383.7027	765.4141	383.2107	N	***333.1769***	167.0921	316.1503	158.5788			3
8	854.4618	427.7345	837.4353	419.2213	836.4512	418.7293	A	***218.1499***	109.5786	201.1234	101.0653			2
9							K	***147.1128***	74.0600	130.0863	65.5468			1

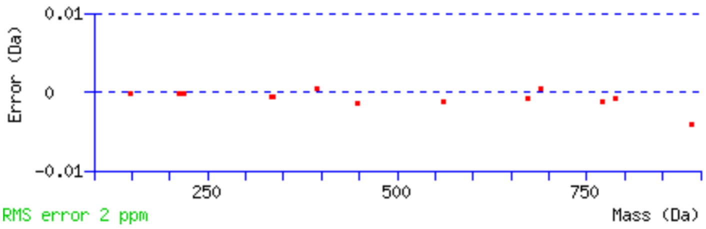

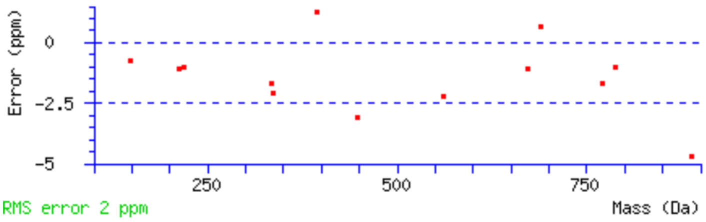

NCBI **BLAST** search of LVVQIDNAK
(Parameters: blastp, nr protein database, expect=20000, no filter, PAM30)
Other BLAST web gateways

All matches to this query

Score	Mr(calc):	Delta	Sequence
61.4	999.5600	-0.0012	LVVQIDNAK
41.7	999.5600	-0.0012	LVVEIDNAK
41.7	999.5600	-0.0012	LVVQIDNAK

Mascot: http://www.matrixscience.com/

Mascot Search Results

Peptide View

MS/MS Fragmentation of **LVVQIDNAK**
Found in **sp|Q15323|K1H1_HUMAN**, Keratin, type I cuticular Ha1 OS=Homo sapiens GN=KRT31 PE=2 SV=3

Match to Query 2892: 999.558808 from(500.786680,2+) intensity(33847728.0000) scans(7919) rtinseconds(1491) index(21214)
Title: 160219_Sunil_SDSI_A_Spectrum074518_scans__7919_RTINSECONDS=1491
Data file C:\\Sunil\\TKAP\\T\\T160219_Sunil_SDSI_A.mgf

Click mouse within plot area to zoom in by factor of two about that point
Or, Plot from 50 to 900 Da Full range
Label all possible matches Label matches used for scoring

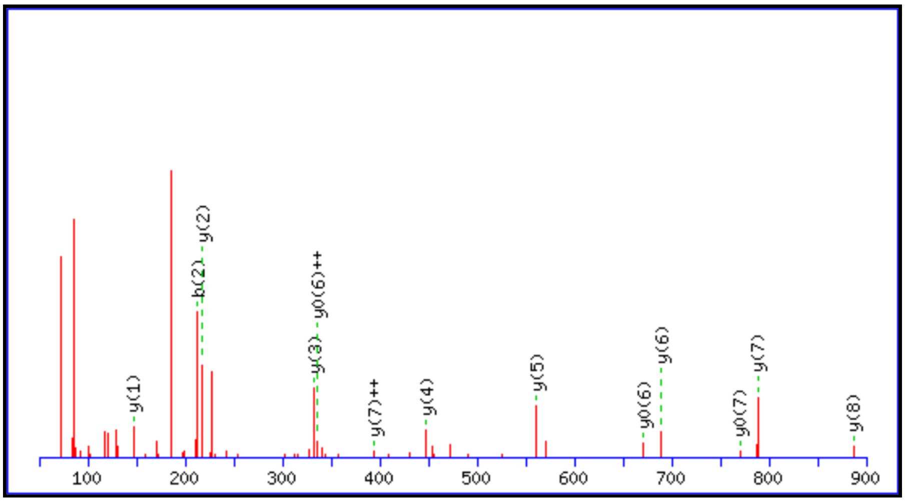

Monoisotopic mass of neutral peptide Mr(calc): 999.5600
Fixed modifications: Carbamidomethyl (C) (apply to specified residues or termini only)
Variable modifications:
N7 : Deamidated (NQ)
Ions Score: 61 **Expect:** 8.2e-005
Matches : 13/74 fragment ions using 22 most intense peaks (help)

#	b	b++	b*	b*++	b0	b0++	Seq.	y	y++	y*	y*++	y0	y0++	#
1	114.0913	57.5493					L							9
2	**213.1598**	107.0835					V	***887.4833***	444.2453	870.4567	435.7320	869.4727	435.2400	8
3	312.2282	156.6177					V	***788.4149***	394.7111	771.3883	386.1978	770.4043	385.7058	7
4	440.2867	220.6470	423.2602	212.1337			Q	***689.3464***	345.1769	672.3199	336.6636	671.3359	336.1716	6
5	553.3708	277.1890	536.3443	268.6758			I	***561.2879***	281.1476	544.2613	272.6343	543.2773	272.1423	5
6	668.3978	334.7025	651.3712	326.1892	650.3872	325.6972	D	***448.2038***	224.6055	431.1773	216.0923	430.1932	215.6003	4
7	783.4247	392.2160	766.3981	383.7027	765.4141	383.2107	N	***333.1769***	167.0921	316.1503	158.5788			3
8	854.4618	427.7345	837.4353	419.2213	836.4512	418.7293	A	***218.1499***	109.5786	201.1234	101.0653			2
9							K	***147.1128***	74.0600	130.0863	65.5468			1

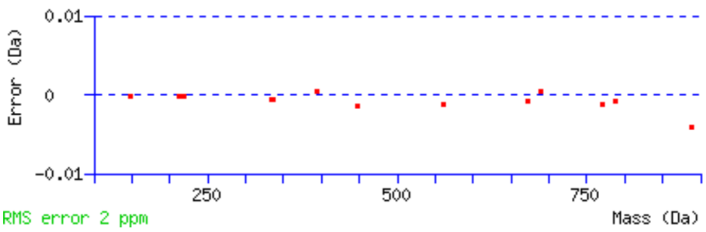

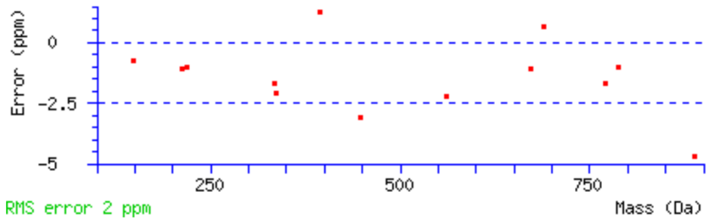

NCBI **BLAST** search of LVVQIDNAK
(Parameters: blastp, nr protein database, expect=20000, no filter, PAM30)
Other BLAST web gateways

All matches to this query

Score	Mr(calc):	Delta	Sequence
61.4	999.5600	-0.0012	LVVQIDNAK
41.7	999.5600	-0.0012	LVVEIDNAK
41.7	999.5600	-0.0012	LVVQIDNAK

Mascot: http://www.matrixscience.com/

Mascot Search Results

Peptide View

MS/MS Fragmentation of **LVVQIDNAKLAADDFR**
Found in **sp|Q15323|K1H1_HUMAN**, Keratin, type I cuticular Ha1 OS=Homo sapiens GN=KRT31 PE=2 SV=3

Match to Query 24475: 1787.941182 from(596.987670,3+) intensity(906301.6875) scans(13208) rtinseconds(2426) index(40996)
Title: 160219_Sunil_SDSI_A_Spectrum095581_scans__13208_RTINSECONDS=2426
Data file C:\\Sunil\\TKAP\\T\\T160219_Sunil_SDSI_A.mgf

Click mouse within plot area to zoom in by factor of two about that point
Or, Plot from 100 to 1600 Da Full range
Label all possible matches Label matches used for scoring

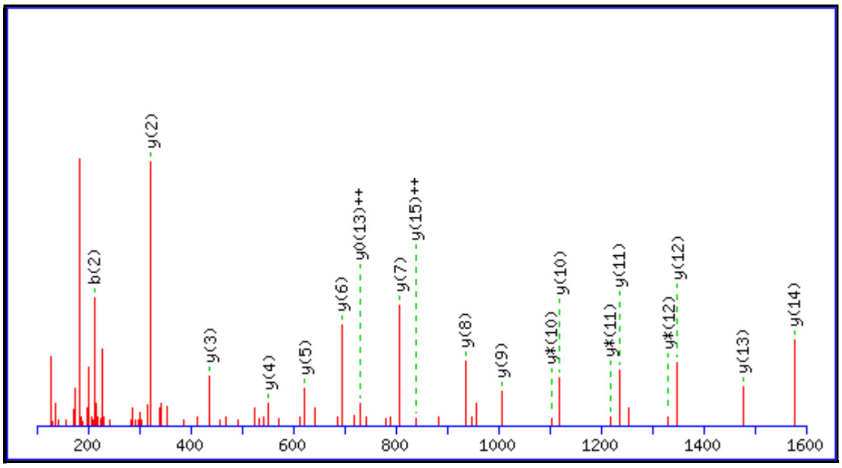

Monoisotopic mass of neutral peptide Mr(calc): 1787.9417
Fixed modifications: Carbamidomethyl (C) (apply to specified residues or termini only)
Variable modifications:
Q4 : Deamidated (NQ)
Ions Score: 115 **Expect:** 8.4e-010
Matches : 19/160 fragment ions using 27 most intense peaks (help)

#	b	b^{++}	b*	b^{*++}	b^0	b^{0++}	Seq.	y	y^{++}	y*	y^{*++}	y^0	y^{0++}	#
1	114.0913	57.5493					L							16
2	**213.1598**	107.0835					V	1675.8650	838.4361	1658.8384	829.9229	1657.8544	829.4308	15
3	312.2282	156.6177					V	***1576.7966***	788.9019	1559.7700	780.3886	1558.7860	779.8966	14
4	441.2708	221.1390	424.2442	212.6257			Q	***1477.7281***	739.3677	1460.7016	730.8544	1459.7176	730.3624	13
5	554.3548	277.6811	537.3283	269.1678			I	***1348.6856***	674.8464	1331.6590	666.3331	1330.6750	665.8411	12
6	669.3818	335.1945	652.3552	326.6812	651.3712	326.1892	D	***1235.6015***	618.3044	1218.5749	609.7911	1217.5909	609.2991	11
7	783.4247	392.2160	766.3981	383.7027	765.4141	383.2107	N	***1120.5745***	560.7909	1103.5480	552.2776	1102.5640	551.7856	10
8	854.4618	427.7345	837.4353	419.2213	836.4512	418.7293	A	***1006.5316***	503.7694	989.5051	495.2562	988.5211	494.7642	9
9	982.5568	491.7820	965.5302	483.2687	964.5462	482.7767	K	***935.4945***	468.2509	918.4680	459.7376	917.4839	459.2456	8
10	1095.6408	548.3241	1078.6143	539.8108	1077.6303	539.3188	L	***807.3995***	404.2034	790.3730	395.6901	789.3890	395.1981	7
11	1166.6780	583.8426	1149.6514	575.3293	1148.6674	574.8373	A	***694.3155***	347.6614	677.2889	339.1481	676.3049	338.6561	6
12	1237.7151	619.3612	1220.6885	610.8479	1219.7045	610.3559	A	***623.2784***	312.1428	606.2518	303.6295	605.2678	303.1375	5
13	1352.7420	676.8746	1335.7155	668.3614	1334.7314	667.8694	D	***552.2413***	276.6243	535.2147	268.1110	534.2307	267.6190	4
14	1467.7690	734.3881	1450.7424	725.8748	1449.7584	725.3828	D	***437.2143***	219.1108	420.1878	210.5975	419.2037	210.1055	3
15	1614.8374	807.9223	1597.8108	799.4090	1596.8268	798.9170	F	***322.1874***	161.5973	305.1608	153.0840			2
16							R	175.1190	88.0631	158.0924	79.5498			1

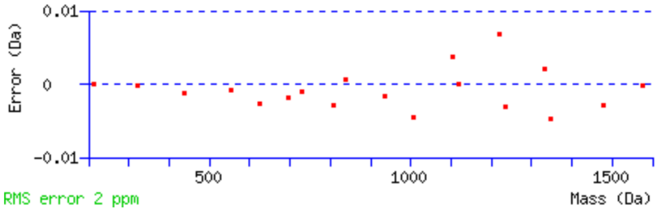

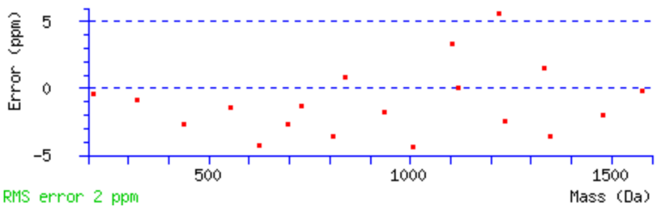

NCBI **BLAST** search of LVVQIDNAKLAADDFR

(Parameters: blastp, nr protein database, expect=20000, no filter, PAM30)
Other BLAST web gateways

All matches to this query

Score	Mr(calc):	Delta	Sequence
114.7	1787.9417	-0.0006	LVVEIDNAKLAADDFR
114.7	1787.9417	-0.0006	LVVQIDNAKLAADDFR
68.9	1787.9417	-0.0006	LVVQIDNAKLAADDFR

Mascot: http://www.matrixscience.com/

Mascot Search Results

Peptide View

MS/MS Fragmentation of **LVVQIDNAKLAADDFR**
Found in **sp|Q15323|K1H1_HUMAN**, Keratin, type I cuticular Ha1 OS=Homo sapiens GN=KRT31 PE=2 SV=3

Match to Query 24560: 1789.922022 from(597.647950,3+) intensity(30069212.0000) scans(13697) rtinseconds(2483) index(26088)
Title: 160219_Sunil_SDSI_A_Spectrum079394_scans__13697_RTINSECONDS=2483
Data file C:\\Sunil\\TKAP\\T\\T160219_Sunil_SDSI_A.mgf

Click mouse within plot area to zoom in by factor of two about that point
Or, Plot from 100 to 1600 Da Full range
Label all possible matches Label matches used for scoring

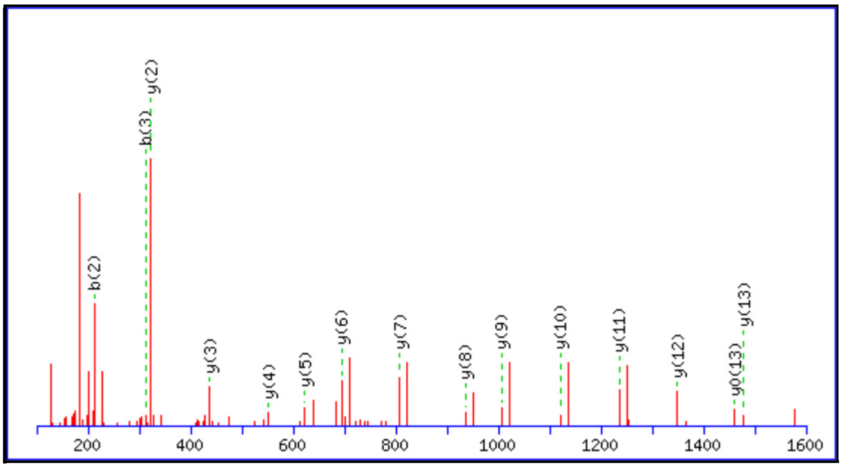

Monoisotopic mass of neutral peptide Mr(calc): 1788.9258
Fixed modifications: Carbamidomethyl (C) (apply to specified residues or termini only)
Variable modifications:
Q4 : Deamidated (NQ)
N7 : Deamidated (NQ)
Ions Score: 63 **Expect:** 0.00014
Matches : 15/160 fragment ions using 42 most intense peaks (help)

#	b	b++	b*	b*++	b0	b0++	Seq.	y	y++	y*	y*++	y0	y0++	#
1	114.0913	57.5493					L							16
2	**213.1598**	107.0835					V	1676.8490	838.9281	1659.8224	830.4149	1658.8384	829.9229	15
3	**312.2282**	156.6177					V	1577.7806	789.3939	1560.7540	780.8807	1559.7700	780.3886	14
4	441.2708	221.1390	424.2442	212.6257			Q	***1478.7122***	739.8597	1461.6856	731.3464	1460.7016	730.8544	13
5	554.3548	277.6811	537.3283	269.1678			I	***1349.6696***	675.3384	1332.6430	666.8251	1331.6590	666.3331	12
6	669.3818	335.1945	652.3552	326.6812	651.3712	326.1892	D	***1236.5855***	618.7964	1219.5590	610.2831	1218.5749	609.7911	11
7	784.4087	392.7080	767.3822	384.1947	766.3981	383.7027	N	***1121.5586***	561.2829	1104.5320	552.7696	1103.5480	552.2776	10
8	855.4458	428.2266	838.4193	419.7133	837.4353	419.2213	A	***1006.5316***	503.7694	989.5051	495.2562	988.5211	494.7642	9
9	983.5408	492.2740	966.5142	483.7608	965.5302	483.2687	K	***935.4945***	468.2509	918.4680	459.7376	917.4839	459.2456	8
10	1096.6249	548.8161	1079.5983	540.3028	1078.6143	539.8108	L	***807.3995***	404.2034	790.3730	395.6901	789.3890	395.1981	7
11	1167.6620	584.3346	1150.6354	575.8213	1149.6514	575.3293	A	***694.3155***	347.6614	677.2889	339.1481	676.3049	338.6561	6
12	1238.6991	619.8532	1221.6725	611.3399	1220.6885	610.8479	A	***623.2784***	312.1428	606.2518	303.6295	605.2678	303.1375	5
13	1353.7260	677.3666	1336.6995	668.8534	1335.7155	668.3614	D	***552.2413***	276.6243	535.2147	268.1110	534.2307	267.6190	4
14	1468.7530	734.8801	1451.7264	726.3668	1450.7424	725.8748	D	***437.2143***	219.1108	420.1878	210.5975	419.2037	210.1055	3
15	1615.8214	808.4143	1598.7948	799.9011	1597.8108	799.4090	F	***322.1874***	161.5973	305.1608	153.0840			2
16							R	175.1190	88.0631	158.0924	79.5498			1

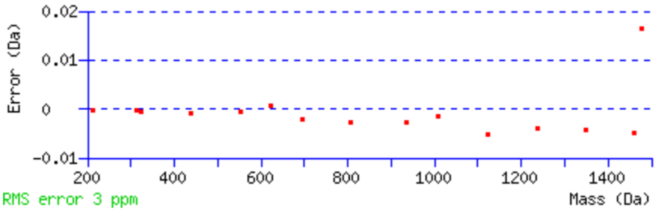

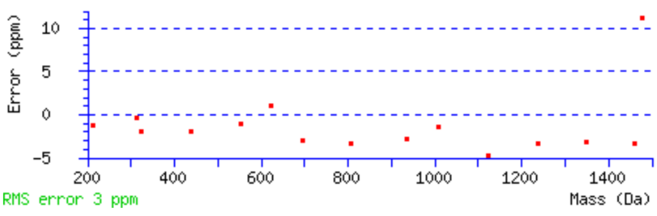

NCBI **BLAST** search of LVVQIDNAKLAADDFR
(Parameters: blastp, nr protein database, expect=20000, no filter, PAM30)
Other BLAST web gateways

All matches to this query

Score	Mr(calc):	Delta	Sequence
62.9	1788.9258	0.9963	LVVEIDNAKLAADDFR
62.9	1788.9258	0.9963	LVVQIDNAKLAADDFR
62.0	1789.9210	0.0010	LVVNIDNAKLASDDFR
30.2	1789.9210	0.0010	LVVNIDNAKLASDDFR

Mascot: http://www.matrixscience.com/

Mascot Search Results

Peptide View

MS/MS Fragmentation of **LVVQIDNAKLAADDFRTK**
Found in **sp|Q15323|K1H1_HUMAN**, Keratin, type I cuticular Ha1 OS=Homo sapiens GN=KRT31 PE=2 SV=3

Match to Query 29122: 2017.086856 from(505.278990,4+) intensity(1167629.0000) scans(12616) rtinseconds(2297) index(25332)
Title: 160219_Sunil_SDSI_A_Spectrum078638_scans__12616_RTINSECONDS=2297
Data file C:\\Sunil\\TKAP\\T\\T160219_Sunil_SDSI_A.mgf

Click mouse within plot area to zoom in by factor of two about that point
Or, Plot from 100 to 1600 Da Full range
Label all possible matches ○ Label matches used for scoring ◉

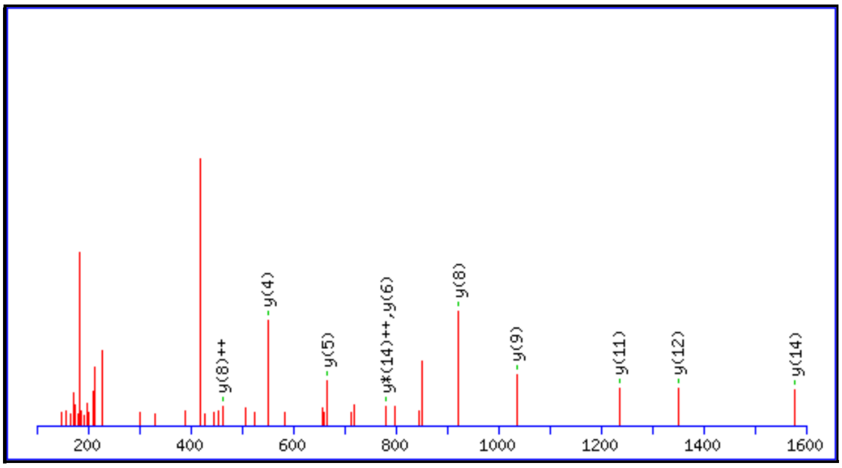

Monoisotopic mass of neutral peptide Mr(calc): 2017.0844
Fixed modifications: Carbamidomethyl (C) (apply to specified residues or termini only)
Variable modifications:
N7 : Deamidated (NQ)
Ions Score: 61 **Expect:** 0.00012
Matches : 10/186 fragment ions using 12 most intense peaks (help)

#	b	b++	b*	b*++	b0	b0++	Seq.	y	y++	y*	y*++	y0	y0++	#
1	114.0913	57.5493					L							18
2	213.1598	107.0835					V	1905.0076	953.0074	1887.9811	944.4942	1886.9971	944.0022	17
3	312.2282	156.6177					V	1805.9392	903.4732	1788.9127	894.9600	1787.9286	894.4680	16
4	440.2867	220.6470	423.2602	212.1337			Q	1706.8708	853.9390	1689.8442	845.4258	1688.8602	844.9338	15
5	553.3708	277.1890	536.3443	268.6758			I	***1578.8122***	789.9097	1561.7857	781.3965	1560.8016	780.9045	14
6	668.3978	334.7025	651.3712	326.1892	650.3872	325.6972	D	1465.7281	733.3677	1448.7016	724.8544	1447.7176	724.3624	13
7	783.4247	392.2160	766.3981	383.7027	765.4141	383.2107	N	***1350.7012***	675.8542	1333.6747	667.3410	1332.6906	666.8490	12
8	854.4618	427.7345	837.4353	419.2213	836.4512	418.7293	A	***1235.6743***	618.3408	1218.6477	609.8275	1217.6637	609.3355	11
9	982.5568	491.7820	965.5302	483.2687	964.5462	482.7767	K	1164.6371	582.8222	1147.6106	574.3089	1146.6266	573.8169	10
10	1095.6408	548.3241	1078.6143	539.8108	1077.6303	539.3188	L	***1036.5422***	518.7747	1019.5156	510.2615	1018.5316	509.7694	9
11	1166.6780	583.8426	1149.6514	575.3293	1148.6674	574.8373	A	***923.4581***	462.2327	906.4316	453.7194	905.4476	453.2274	8
12	1237.7151	619.3612	1220.6885	610.8479	1219.7045	610.3559	A	852.4210	426.7141	835.3945	418.2009	834.4104	417.7089	7
13	1352.7420	676.8746	1335.7155	668.3614	1334.7314	667.8694	D	***781.3839***	391.1956	764.3573	382.6823	763.3733	382.1903	6
14	1467.7690	734.3881	1450.7424	725.8748	1449.7584	725.3828	D	***666.3570***	333.6821	649.3304	325.1688	648.3464	324.6768	5
15	1614.8374	807.9223	1597.8108	799.4090	1596.8268	798.9170	F	***551.3300***	276.1686	534.3035	267.6554	533.3194	267.1634	4
16	1770.9385	885.9729	1753.9119	877.4596	1752.9279	876.9676	R	404.2616	202.6344	387.2350	194.1212	386.2510	193.6292	3
17	1871.9862	936.4967	1854.9596	927.9834	1853.9756	927.4914	T	248.1605	124.5839	231.1339	116.0706	230.1499	115.5786	2
18							K	147.1128	74.0600	130.0863	65.5468			1

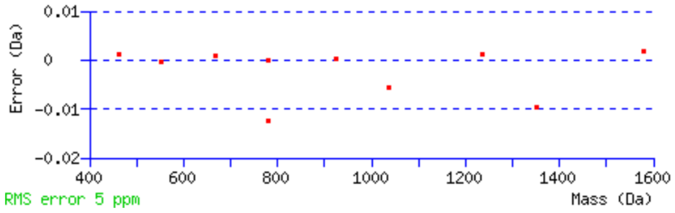

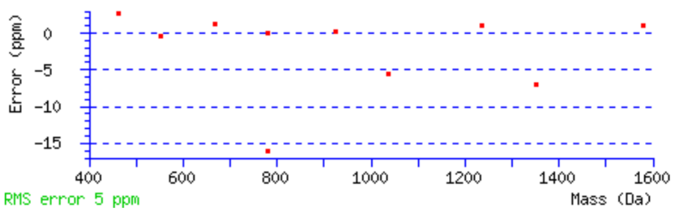

NCBI **BLAST** search of LVVQIDNAKLAADDFRTK
(Parameters: blastp, nr protein database, expect=20000, no filter, PAM30)
Other BLAST web gateways

All matches to this query

Score	Mr(calc):	Delta	Sequence
60.6	2017.0844	0.0025	LVVQIDNAKLAADDFRTK
32.9	2017.0844	0.0025	LVVEIDNAKLAADDFRTK
32.9	2016.1004	0.9865	LVVQIDNAKLAADDFRTK
32.9	2017.0844	0.0025	LVVQIDNAKLAADDFRTK

Mascot: http://www.matrixscience.com/

Mascot Search Results

Peptide View

MS/MS Fragmentation of **PYNFCLPSLSCR**
Found in **sp|Q15323|K1H1_HUMAN**, Keratin, type I cuticular Ha1 OS=Homo sapiens GN=KRT31 PE=2 SV=3

Match to Query 18144: 1513.668428 from(757.841490,2+) intensity(13303786.0000) scans(14786) rtinseconds(2669) index(26968)
Title: 160219_Sunil_SDSI_A_Spectrum080276_scans__14786_RTINSECONDS=2669
Data file C:\\Sunil\\TKAP\\T\\T160219_Sunil_SDSI_A.mgf

Click mouse within plot area to zoom in by factor of two about that point
Or, Plot from 100 to 1500 Da Full range
Label all possible matches Label matches used for scoring

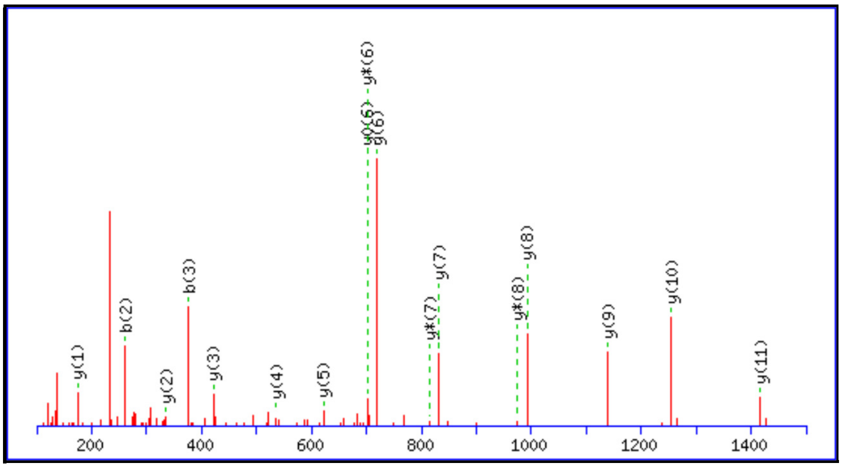

Monoisotopic mass of neutral peptide Mr(calc): 1513.6694
Fixed modifications: Carbamidomethyl (C) (apply to specified residues or termini only)
Variable modifications:
N3 : Deamidated (NQ)
Ions Score: 81 **Expect:** 6.9e-007
Matches : 17/110 fragment ions using 33 most intense peaks (help)

#	b	b^{++}	b^{*}	b^{*++}	b^{0}	b^{0++}	Seq.	y	y^{++}	y^{*}	y^{*++}	y^{0}	y^{0++}	#
1	98.0600	49.5337					P							12
2	**261.1234**	131.0653					Y	***1417.6239***	709.3156	1400.5973	700.8023	1399.6133	700.3103	11
3	**376.1503**	188.5788	359.1238	180.0655			N	***1254.5606***	627.7839	1237.5340	619.2706	1236.5500	618.7786	10
4	523.2187	262.1130	506.1922	253.5997			F	***1139.5336***	570.2704	1122.5071	561.7572	1121.5230	561.2652	9
5	683.2494	342.1283	666.2228	333.6151			C	***992.4652***	496.7362	975.4386	488.2230	974.4546	487.7310	8
6	796.3334	398.6704	779.3069	390.1571			L	***832.4345***	416.7209	815.4080	408.2076	814.4240	407.7156	7
7	893.3862	447.1967	876.3597	438.6835			P	***719.3505***	360.1789	702.3239	351.6656	701.3399	351.1736	6
8	980.4182	490.7128	963.3917	482.1995	962.4077	481.7075	S	***622.2977***	311.6525	605.2712	303.1392	604.2872	302.6472	5
9	1093.5023	547.2548	1076.4757	538.7415	1075.4917	538.2495	L	***535.2657***	268.1365	518.2391	259.6232	517.2551	259.1312	4
10	1180.5343	590.7708	1163.5078	582.2575	1162.5238	581.7655	S	***422.1816***	211.5945	405.1551	203.0812	404.1711	202.5892	3
11	1340.5650	670.7861	1323.5384	662.2728	1322.5544	661.7808	C	***335.1496***	168.0784	318.1231	159.5652			2
12							R	***175.1190***	88.0631	158.0924	79.5498			1

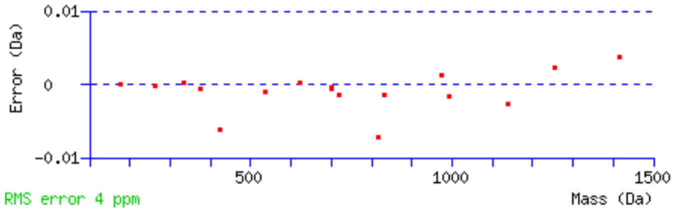

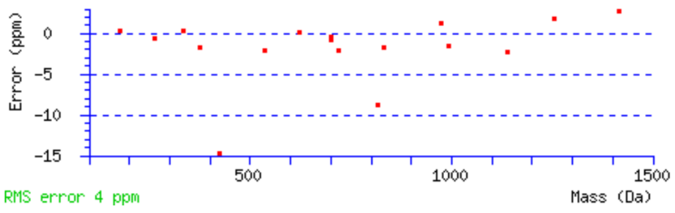

NCBI **BLAST** search of PYNFCLPSLSCR
(Parameters: blastp, nr protein database, expect=20000, no filter, PAM30)
Other BLAST web gateways

All matches to this query

Score	Mr(calc):	Delta	Sequence
81.4	1513.6694	-0.0009	PYNFCLPSLSCR
0.3	1513.6792	-0.0108	MDLASYVSNPICK

Mascot: http://www.matrixscience.com/

Mascot Search Results

Peptide View

MS/MS Fragmentation of **PYNFCLPSLSCR**
Found in **sp|Q15323|K1H1_HUMAN**, Keratin, type I cuticular Ha1 OS=Homo sapiens GN=KRT31 PE=2 SV=3

Match to Query 18144: 1513.668428 from(757.841490,2+) intensity(13303786.0000) scans(14786) rtinseconds(2669) index(26968)
Title: 160219_Sunil_SDSI_A_Spectrum080276_scans__14786_RTINSECONDS=2669
Data file C:\\Sunil\\TKAP\\T\\T160219_Sunil_SDSI_A.mgf

Click mouse within plot area to zoom in by factor of two about that point
Or, Plot from 100 to 1500 Da Full range
Label all possible matches ○ Label matches used for scoring ◉

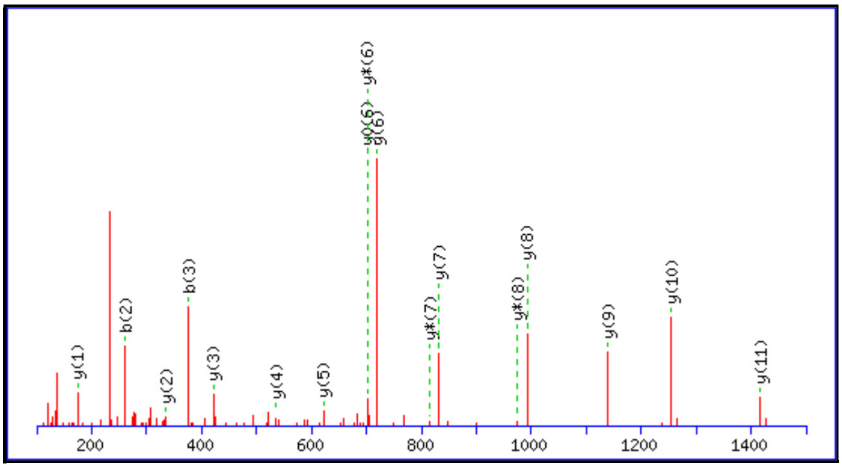

Monoisotopic mass of neutral peptide Mr(calc): 1513.6694
Fixed modifications: Carbamidomethyl (C) (apply to specified residues or termini only)
Variable modifications:
N3 : Deamidated (NQ)
Ions Score: 81 **Expect:** 6.9e-007
Matches : 17/110 fragment ions using 33 most intense peaks (help)

#	b	b++	b*	b*++	b0	b0++	Seq.	y	y++	y*	y*++	y0	y0++	#
1	98.0600	49.5337					P							12
2	**261.1234**	131.0653					Y	***1417.6239***	709.3156	1400.5973	700.8023	1399.6133	700.3103	11
3	**376.1503**	188.5788	359.1238	180.0655			N	***1254.5606***	627.7839	1237.5340	619.2706	1236.5500	618.7786	10
4	523.2187	262.1130	506.1922	253.5997			F	***1139.5336***	570.2704	1122.5071	561.7572	1121.5230	561.2652	9
5	683.2494	342.1283	666.2228	333.6151			C	***992.4652***	496.7362	975.4386	488.2230	974.4546	487.7310	8
6	796.3334	398.6704	779.3069	390.1571			L	***832.4345***	416.7209	815.4080	408.2076	814.4240	407.7156	7
7	893.3862	447.1967	876.3597	438.6835			P	***719.3505***	360.1789	702.3239	351.6656	701.3399	351.1736	6
8	980.4182	490.7128	963.3917	482.1995	962.4077	481.7075	S	***622.2977***	311.6525	605.2712	303.1392	604.2872	302.6472	5
9	1093.5023	547.2548	1076.4757	538.7415	1075.4917	538.2495	L	***535.2657***	268.1365	518.2391	259.6232	517.2551	259.1312	4
10	1180.5343	590.7708	1163.5078	582.2575	1162.5238	581.7655	S	***422.1816***	211.5945	405.1551	203.0812	404.1711	202.5892	3
11	1340.5650	670.7861	1323.5384	662.2728	1322.5544	661.7808	C	***335.1496***	168.0784	318.1231	159.5652			2
12							R	***175.1190***	88.0631	158.0924	79.5498			1

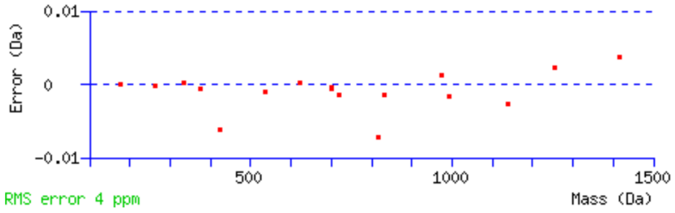

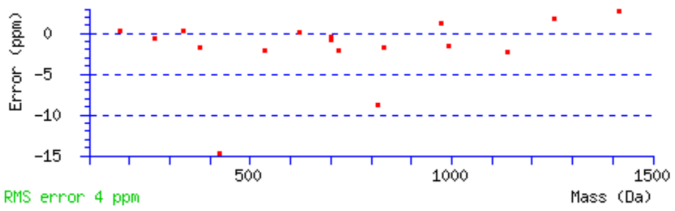

NCBI **BLAST** search of PYNFCLPSLSCR
(Parameters: blastp, nr protein database, expect=20000, no filter, PAM30)
Other BLAST web gateways

All matches to this query

Score	Mr(calc):	Delta	Sequence
81.4	1513.6694	-0.0009	PYNFCLPSLSCR
0.3	1513.6792	-0.0108	MDLASYVSNPICK

Mascot: http://www.matrixscience.com/

Mascot Search Results

Peptide View

MS/MS Fragmentation of **QLERDNAELENLIR**
Found in **sp|Q15323|K1H1_HUMAN**, Keratin, type I cuticular Ha1 OS=Homo sapiens GN=KRT31 PE=2 SV=3

Match to Query 23142: 1712.867772 from(571.963200,3+) intensity(1506930.0000) scans(10411) rtinseconds(1919) index(23418)
Title: 160219_Sunil_SDSI_A_Spectrum076722_scans__10411_RTINSECONDS=1919
Data file C:\\Sunil\\TKAP\\T\\T160219_Sunil_SDSI_A.mgf

Click mouse within plot area to zoom in by factor of two about that point
Or, Plot from 100 to 1700 Da Full range
Label all possible matches ○ Label matches used for scoring ◉

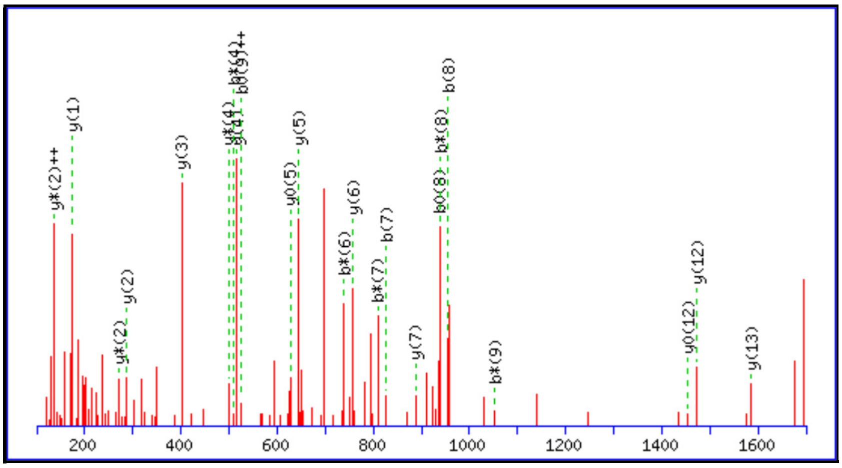

Monoisotopic mass of neutral peptide Mr(calc): 1712.8693
Fixed modifications: Carbamidomethyl (C) (apply to specified residues or termini only)
Variable modifications:
N11 : Deamidated (NQ)
Ions Score: 49 **Expect:** 0.0025
Matches : 23/144 fragment ions using 47 most intense peaks (help)

#	b	b++	b*	b*++	b0	b0++	Seq.	y	y++	y*	y*++	y0	y0++	#
1	129.0659	65.0366	112.0393	56.5233			Q							14
2	242.1499	121.5786	225.1234	113.0653			L	**1585.8180**	793.4127	1568.7915	784.8994	1567.8075	784.4074	13
3	371.1925	186.0999	354.1660	177.5866	353.1819	177.0946	E	**1472.7340**	736.8706	1455.7074	728.3573	1454.7234	727.8653	12
4	527.2936	264.1504	510.2671	255.6372	509.2831	255.1452	R	1343.6914	672.3493	1326.6648	663.8360	1325.6808	663.3440	11
5	642.3206	321.6639	625.2940	313.1506	624.3100	312.6586	D	1187.5903	594.2988	1170.5637	585.7855	1169.5797	585.2935	10
6	756.3635	378.6854	739.3369	370.1721	738.3529	369.6801	N	1072.5633	536.7853	1055.5368	528.2720	1054.5527	527.7800	9
7	**827.4006**	414.2039	810.3741	405.6907	809.3900	405.1987	A	958.5204	479.7638	941.4938	471.2506	940.5098	470.7585	8
8	**956.4432**	478.7252	939.4167	470.2120	938.4326	469.7200	E	**887.4833**	444.2453	870.4567	435.7320	869.4727	435.2400	7
9	1069.5273	535.2673	1052.5007	526.7540	1051.5167	526.2620	L	**758.4407**	379.7240	741.4141	371.2107	740.4301	370.7187	6
10	1198.5699	599.7886	1181.5433	591.2753	1180.5593	590.7833	E	**645.3566**	323.1819	628.3301	314.6687	627.3461	314.1767	5
11	1313.5968	657.3020	1296.5703	648.7888	1295.5862	648.2968	N	**516.3140**	258.6606	499.2875	250.1474			4
12	1426.6809	713.8441	1409.6543	705.3308	1408.6703	704.8388	L	**401.2871**	201.1472	384.2605	192.6339			3
13	1539.7649	770.3861	1522.7384	761.8728	1521.7544	761.3808	I	**288.2030**	144.6051	271.1765	136.0919			2
14							R	**175.1190**	88.0631	158.0924	79.5498			1

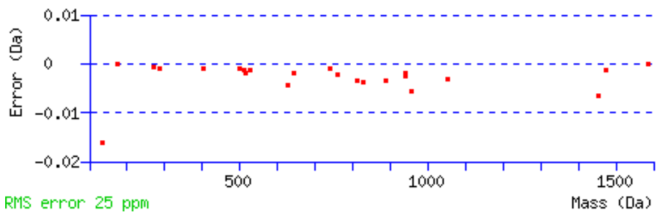

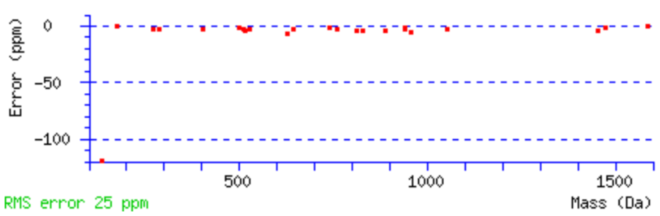

NCBI **BLAST** search of QLERDNAELENLIR
(Parameters: blastp, nr protein database, expect=20000, no filter, PAM30)
Other BLAST web gateways

All matches to this query

Score	Mr(calc):	Delta	Sequence
49.3	1712.8693	-0.0015	QLERDNAELENLIR
9.8	1712.8693	-0.0015	QLERDNAELENLIR
9.6	1712.8845	-0.0167	ASKFAAQNLHQNLIR
9.6	1712.8845	-0.0167	ASKFAAQNLHQNLIR
6.0	1711.8576	1.0102	RAVCRYYNNDLLR
1.8	1712.8555	0.0122	EVNKFQMAYSNLLR
0.9	1712.8845	-0.0167	ASKFAAQNLHQNLIR

Mascot: http://www.matrixscience.com/

Mascot Search Results

Peptide View

MS/MS Fragmentation of **QLERDNAELENLIR**
Found in **sp|Q15323|K1H1_HUMAN**, Keratin, type I cuticular Ha1 OS=Homo sapiens GN=KRT31 PE=2 SV=3

Match to Query 23149: 1712.876352 from(571.966060,3+) intensity(2210090.7500) scans(12705) rtinseconds(2338) index(9964)
Title: 160219_Sunil_SDSI_A_Spectrum062083_scans__12705_RTINSECONDS=2338
Data file C:\\Sunil\\TKAP\\T\\T160219_Sunil_SDSI_A.mgf

Click mouse within plot area to zoom in by factor of two about that point
Or, Plot from 100 to 1700 Da Full range
Label all possible matches ○ Label matches used for scoring ◉

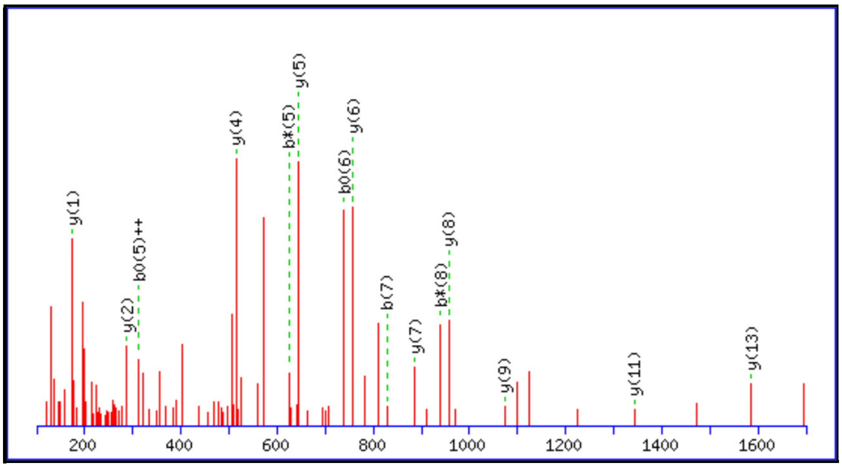

Monoisotopic mass of neutral peptide Mr(calc): 1712.8693
Fixed modifications: Carbamidomethyl (C) (apply to specified residues or termini only)
Variable modifications:
N6 : Deamidated (NQ)
Ions Score: 66 **Expect:** 5.5e-005
Matches : 15/144 fragment ions using 28 most intense peaks (help)

#	b	b++	b*	b*++	b0	b0++	Seq.	y	y++	y*	y*++	y0	y0++	#
1	129.0659	65.0366	112.0393	56.5233			Q							14
2	242.1499	121.5786	225.1234	113.0653			L	***1585.8180***	793.4127	1568.7915	784.8994	1567.8075	784.4074	13
3	371.1925	186.0999	354.1660	177.5866	353.1819	177.0946	E	1472.7340	736.8706	1455.7074	728.3573	1454.7234	727.8653	12
4	527.2936	264.1504	510.2671	255.6372	509.2831	255.1452	R	***1343.6914***	672.3493	1326.6648	663.8360	1325.6808	663.3440	11
5	642.3206	321.6639	625.2940	313.1506	624.3100	312.6586	D	1187.5903	594.2988	1170.5637	585.7855	1169.5797	585.2935	10
6	757.3475	379.1774	740.3210	370.6641	739.3369	370.1721	N	***1072.5633***	536.7853	1055.5368	528.2720	1054.5527	527.7800	9
7	**828.3846**	414.6959	811.3581	406.1827	810.3741	405.6907	A	***957.5364***	479.2718	940.5098	470.7585	939.5258	470.2665	8
8	957.4272	479.2172	940.4007	470.7040	939.4167	470.2120	E	***886.4993***	443.7533	869.4727	435.2400	868.4887	434.7480	7
9	1070.5113	535.7593	1053.4847	527.2460	1052.5007	526.7540	L	***757.4567***	379.2320	740.4301	370.7187	739.4461	370.2267	6
10	1199.5539	600.2806	1182.5273	591.7673	1181.5433	591.2753	E	***644.3726***	322.6899	627.3461	314.1767	626.3620	313.6847	5
11	1313.5968	657.3020	1296.5703	648.7888	1295.5862	648.2968	N	***515.3300***	258.1686	498.3035	249.6554			4
12	1426.6809	713.8441	1409.6543	705.3308	1408.6703	704.8388	L	401.2871	201.1472	384.2605	192.6339			3
13	1539.7649	770.3861	1522.7384	761.8728	1521.7544	761.3808	I	***288.2030***	144.6051	271.1765	136.0919			2
14							R	***175.1190***	88.0631	158.0924	79.5498			1

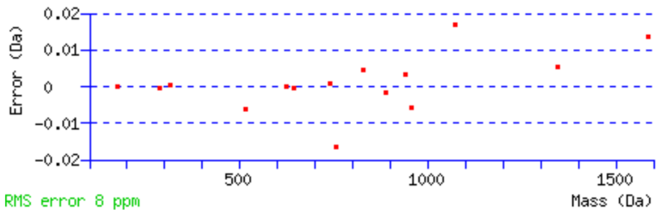

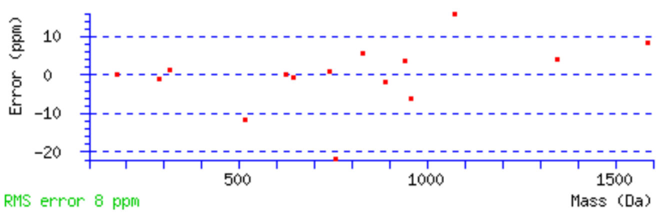

NCBI **BLAST** search of QLERDNAELENLIR
(Parameters: blastp, nr protein database, expect=20000, no filter, PAM30)
Other BLAST web gateways

All matches to this query

Score	Mr(calc):	Delta	Sequence
65.7	1712.8693	0.0071	QLERDNAELENLIR
51.8	1711.8853	0.9911	QLERDNAELENLIR
50.6	1712.8693	0.0071	QLERDNAELENLIR
8.4	1712.8693	0.0071	QLERDNAELENLIR
6.2	1712.8805	-0.0041	ELQRLQEENGRLAR
4.0	1712.8845	-0.0082	ASKFAAQNLHQNLIR
4.0	1712.8805	-0.0041	LRQEEEERENLLR

Mascot: http://www.matrixscience.com/

Mascot Search Results

Peptide View

MS/MS Fragmentation of **QLERDNAELENLIRER**
Found in **sp|Q15323|K1H1_HUMAN**, Keratin, type I cuticular Ha1 OS=Homo sapiens GN=KRT31 PE=2 SV=3

Match to Query 28715: 1998.013256 from(500.510590,4+) intensity(2497427.0000) scans(9070) rtinseconds(1688) index(22242)
Title: 160219_Sunil_SDSI_A_Spectrum075546_scans__9070_RTINSECONDS=1688
Data file C:\\Sunil\\TKAP\\T\\T160219_Sunil_SDSI_A.mgf

Click mouse within plot area to zoom in by factor of two about that point
Or, Plot from 100 to 2000 Da Full range
Label all possible matches ○ Label matches used for scoring ◉

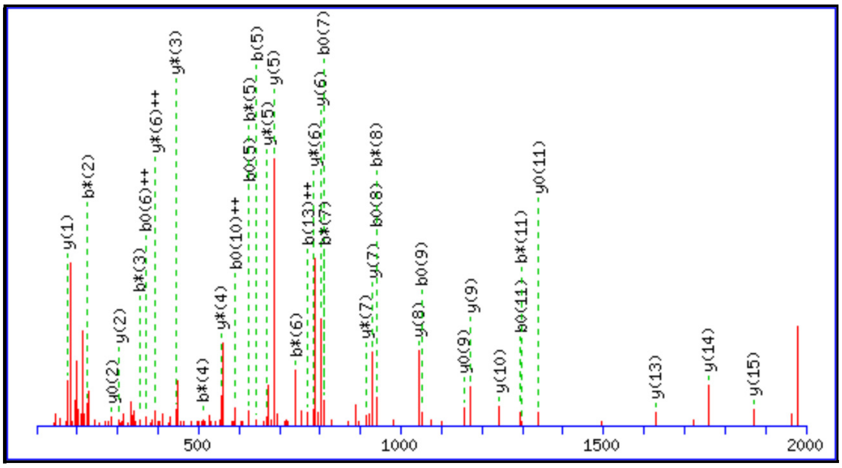

Monoisotopic mass of neutral peptide Mr(calc): 1998.0130
Fixed modifications: Carbamidomethyl (C) (apply to specified residues or termini only)
Variable modifications:
N11 : Deamidated (NQ)
Ions Score: 46 **Expect:** 0.0058
Matches : 37/174 fragment ions using 90 most intense peaks (help)

#	b	b^{++}	b*	b^{*++}	b^0	b^{0++}	Seq.	y	y^{++}	y*	y^{*++}	y^0	y^{0++}	#
1	129.0659	65.0366	112.0393	56.5233			Q							16
2	242.1499	121.5786	225.1234	113.0653			L	***1870.9617***	935.9845	1853.9352	927.4712	1852.9512	926.9792	15
3	371.1925	186.0999	354.1660	177.5866	353.1819	177.0946	E	***1757.8777***	879.4425	1740.8511	870.9292	1739.8671	870.4372	14
4	527.2936	264.1504	510.2671	255.6372	509.2831	255.1452	R	***1628.8351***	814.9212	1611.8085	806.4079	1610.8245	805.9159	13
5	**642.3206**	321.6639	625.2940	313.1506	624.3100	312.6586	D	1472.7340	736.8706	1455.7074	728.3573	1454.7234	727.8653	12
6	756.3635	378.6854	739.3369	370.1721	738.3529	369.6801	N	1357.7070	679.3571	1340.6805	670.8439	1339.6965	670.3519	11
7	827.4006	414.2039	810.3741	405.6907	809.3900	405.1987	A	***1243.6641***	622.3357	1226.6375	613.8224	1225.6535	613.3304	10
8	956.4432	478.7252	939.4167	470.2120	938.4326	469.7200	E	***1172.6270***	586.8171	1155.6004	578.3039	1154.6164	577.8118	9
9	1069.5273	535.2673	1052.5007	526.7540	1051.5167	526.2620	L	***1043.5844***	522.2958	1026.5578	513.7826	1025.5738	513.2905	8
10	1198.5699	599.7886	1181.5433	591.2753	1180.5593	590.7833	E	***930.5003***	465.7538	913.4738	457.2405	912.4898	456.7485	7
11	1313.5968	657.3020	1296.5703	648.7888	1295.5862	648.2968	N	***801.4577***	401.2325	784.4312	392.7192	783.4472	392.2272	6
12	1426.6809	713.8441	1409.6543	705.3308	1408.6703	704.8388	L	***686.4308***	343.7190	669.4042	335.2058	668.4202	334.7137	5
13	1539.7649	**770.3861**	1522.7384	761.8728	1521.7544	761.3808	I	573.3467	287.1770	556.3202	278.6637	555.3362	278.1717	4
14	1695.8660	848.4367	1678.8395	839.9234	1677.8555	839.4314	R	460.2627	230.6350	443.2361	222.1217	442.2521	221.6297	3
15	1824.9086	912.9580	1807.8821	904.4447	1806.8981	903.9527	E	***304.1615***	152.5844	287.1350	144.0711	286.1510	143.5791	2
16							R	***175.1190***	88.0631	158.0924	79.5498			1

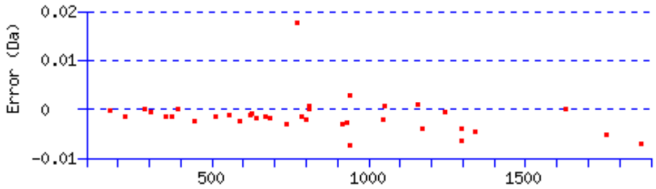

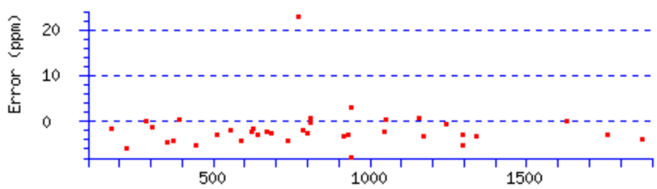

NCBI **BLAST** search of QLERDNAELENLIRER

(Parameters: blastp, nr protein database, expect=20000, no filter, PAM30)
Other BLAST web gateways

All matches to this query

Score	Mr(calc):	Delta	Sequence
46.0	1998.0130	0.0003	QLERDNAELENLIRER
7.3	1998.0130	0.0003	QLERDNAELENLIRER
0.8	1996.9901	1.0232	VCLNQNPQLAWLRNNR
0.8	1996.9901	1.0232	VCLNQNPQLAWLRNNR

Mascot: http://www.matrixscience.com/

Mascot Search Results

Peptide View

MS/MS Fragmentation of **QLERDNAELENLIRER**
Found in **sp|Q15323|K1H1_HUMAN**, Keratin, type I cuticular Ha1 OS=Homo sapiens GN=KRT31 PE=2 SV=3

Match to Query 28716: 1998.014412 from(667.012080,3+) intensity(13097689.0000) scans(11123) rtinseconds(2041) index(24044)
Title: 160219_Sunil_SDSI_A_Spectrum077349_scans__11123_RTINSECONDS=2041
Data file C:\\Sunil\\TKAP\\T\\T160219_Sunil_SDSI_A.mgf

Click mouse within plot area to zoom in by factor of two about that point
Or, Plot from 100 to 2000 Da Full range
Label all possible matches ○ Label matches used for scoring ◉

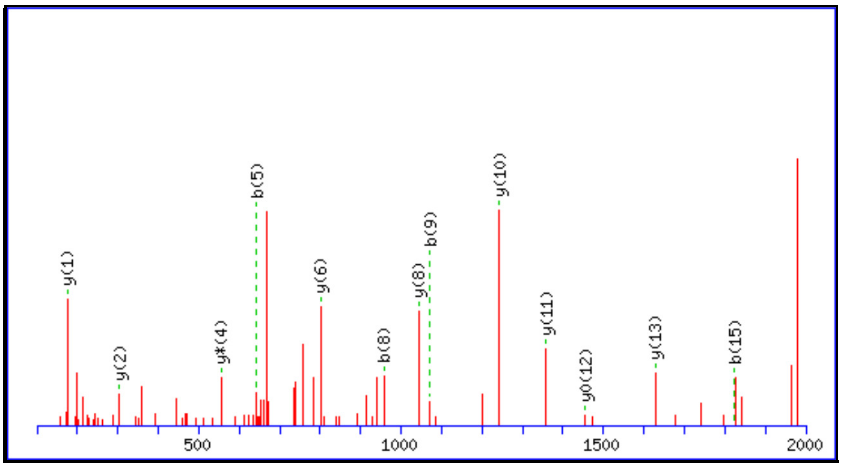

Monoisotopic mass of neutral peptide Mr(calc): 1998.0130
Fixed modifications: Carbamidomethyl (C) (apply to specified residues or termini only)
Variable modifications:
N6 : Deamidated (NQ)
Ions Score: 50 **Expect:** 0.0022
Matches : 13/174 fragment ions using 18 most intense peaks (help)

#	b	b++	b*	b*++	b0	b0++	Seq.	y	y++	y*	y*++	y0	y0++	#
1	129.0659	65.0366	112.0393	56.5233			Q							16
2	242.1499	121.5786	225.1234	113.0653			L	1870.9617	935.9845	1853.9352	927.4712	1852.9512	926.9792	15
3	371.1925	186.0999	354.1660	177.5866	353.1819	177.0946	E	1757.8777	879.4425	1740.8511	870.9292	1739.8671	870.4372	14
4	527.2936	264.1504	510.2671	255.6372	509.2831	255.1452	R	***1628.8351***	814.9212	1611.8085	806.4079	1610.8245	805.9159	13
5	**642.3206**	321.6639	625.2940	313.1506	624.3100	312.6586	D	1472.7340	736.8706	1455.7074	728.3573	1454.7234	727.8653	12
6	757.3475	379.1774	740.3210	370.6641	739.3369	370.1721	N	***1357.7070***	679.3571	1340.6805	670.8439	1339.6965	670.3519	11
7	828.3846	414.6959	811.3581	406.1827	810.3741	405.6907	A	***1242.6801***	621.8437	1225.6535	613.3304	1224.6695	612.8384	10
8	**957.4272**	479.2172	940.4007	470.7040	939.4167	470.2120	E	1171.6430	586.3251	1154.6164	577.8118	1153.6324	577.3198	9
9	**1070.5113**	535.7593	1053.4847	527.2460	1052.5007	526.7540	L	***1042.6004***	521.8038	1025.5738	513.2905	1024.5898	512.7985	8
10	1199.5539	600.2806	1182.5273	591.7673	1181.5433	591.2753	E	929.5163	465.2618	912.4898	456.7485	911.5057	456.2565	7
11	1313.5968	657.3020	1296.5703	648.7888	1295.5862	648.2968	N	***800.4737***	400.7405	783.4472	392.2272	782.4631	391.7352	6
12	1426.6809	713.8441	1409.6543	705.3308	1408.6703	704.8388	L	686.4308	343.7190	669.4042	335.2058	668.4202	334.7137	5
13	1539.7649	770.3861	1522.7384	761.8728	1521.7544	761.3808	I	573.3467	287.1770	556.3202	278.6637	555.3362	278.1717	4
14	1695.8660	848.4367	1678.8395	839.9234	1677.8555	839.4314	R	460.2627	230.6350	443.2361	222.1217	442.2521	221.6297	3
15	**1824.9086**	912.9580	1807.8821	904.4447	1806.8981	903.9527	E	***304.1615***	152.5844	287.1350	144.0711	286.1510	143.5791	2
16							R	***175.1190***	88.0631	158.0924	79.5498			1

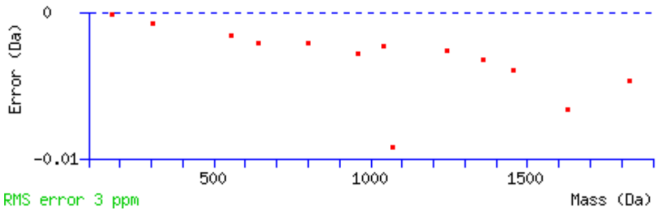

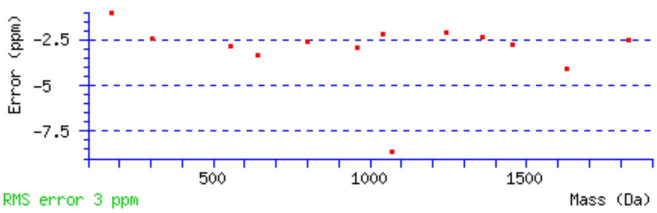

NCBI **BLAST** search of QLERDNAELENLIRER

(Parameters: blastp, nr protein database, expect=20000, no filter, PAM30)
Other BLAST web gateways

All matches to this query

Score	Mr(calc):	Delta	Sequence
50.3	1998.0130	0.0015	QLERDNAELENLIRER
35.0	1998.0130	0.0015	QLERDNAELENLIRER
30.4	1997.0289	0.9855	QLERDNAELENLIRER
15.8	1998.0130	0.0015	QLERDNAELENLIRER
5.2	1998.0017	0.0127	LQQEEEDRQLALQLQR
4.0	1998.0017	0.0127	LQQEEEDRQLALQLQR
4.0	1998.0017	0.0127	LQQEEEDRQLALQLQR
2.4	1998.0283	-0.0138	RPGGPQYGIAREDVVLNR
1.8	1998.0017	0.0127	LQQEEEDRQLALQLQR
0.3	1998.0017	0.0127	LQQEEEDRQLALQLQR

Mascot: http://www.matrixscience.com/

Mascot Search Results

Peptide View

MS/MS Fragmentation of **QLVESDINGLR**
Found in **sp|Q15323|K1H1_HUMAN**, Keratin, type I cuticular Ha1 OS=Homo sapiens GN=KRT31 PE=2 SV=3

Match to Query 9642: 1243.638648 from(622.826600,2+) intensity(1030321.5625) scans(10059) rtinseconds(1858) index(23113)
Title: 160219_Sunil_SDSI_A_Spectrum076417_scans__10059_RTINSECONDS=1858
Data file C:\\Sunil\\TKAP\\T\\T160219_Sunil_SDSI_A.mgf

Click mouse within plot area to zoom in by factor of two about that point
Or, Plot from 0 to 1300 Da Full range
Label all possible matches ○ Label matches used for scoring ◉

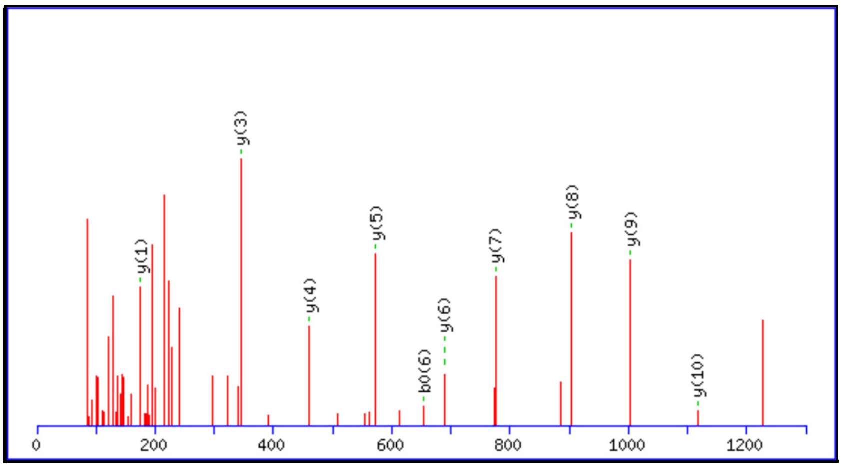

Monoisotopic mass of neutral peptide Mr(calc): 1243.6408
Fixed modifications: Carbamidomethyl (C) (apply to specified residues or termini only)
Variable modifications:
N8 : Deamidated (NQ)
Ions Score: 65 **Expect:** 6.2e-005
Matches : 10/104 fragment ions using 20 most intense peaks (help)

#	b	b^{++}	b^{*}	b^{*++}	b^{0}	b^{0++}	Seq.	y	y^{++}	y^{*}	y^{*++}	y^{0}	y^{0++}	#
1	129.0659	65.0366	112.0393	56.5233			Q							11
2	242.1499	121.5786	225.1234	113.0653			L	***1116.5895***	558.7984	1099.5630	550.2851	1098.5790	549.7931	10
3	341.2183	171.1128	324.1918	162.5995			V	***1003.5055***	502.2564	986.4789	493.7431	985.4949	493.2511	9
4	470.2609	235.6341	453.2344	227.1208	452.2504	226.6288	E	***904.4371***	452.7222	887.4105	444.2089	886.4265	443.7169	8
5	557.2930	279.1501	540.2664	270.6368	539.2824	270.1448	S	***775.3945***	388.2009	758.3679	379.6876	757.3839	379.1956	7
6	672.3199	336.6636	655.2933	328.1503	654.3093	327.6583	D	***688.3624***	344.6849	671.3359	336.1716	670.3519	335.6796	6
7	785.4040	393.2056	768.3774	384.6923	767.3934	384.2003	I	***573.3355***	287.1714	556.3089	278.6581			5
8	900.4309	450.7191	883.4044	442.2058	882.4203	441.7138	N	***460.2514***	230.6293	443.2249	222.1161			4
9	957.4524	479.2298	940.4258	470.7165	939.4418	470.2245	G	***345.2245***	173.1159	328.1979	164.6026			3
10	1070.5364	535.7719	1053.5099	527.2586	1052.5259	526.7666	L	288.2030	144.6051	271.1765	136.0919			2
11							R	***175.1190***	88.0631	158.0924	79.5498			1

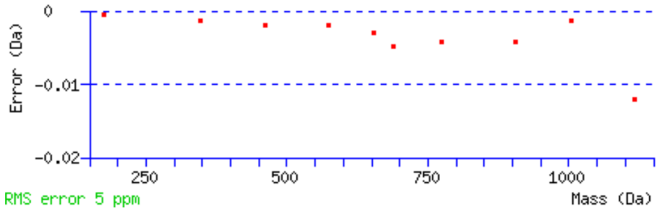

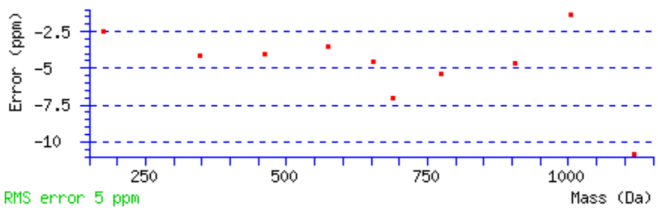

NCBI **BLAST** search of QLVESDINGLR
(Parameters: blastp, nr protein database, expect=20000, no filter, PAM30)
Other BLAST web gateways

All matches to this query

Score	Mr(calc):	Delta	Sequence

65.0	1243.6408	-0.0022	QLVESDINGLR
19.8	1243.6408	-0.0022	QLVESDINGLR
7.3	1243.6421	-0.0035	RALQEWRER
6.9	1243.6381	0.0005	QQRERSNAVR
6.4	1243.6482	-0.0096	QLVNMLLPGDK
6.1	1243.6269	0.0118	QLEQERRER
5.8	1243.6408	-0.0022	ELVQTEIQER

Mascot: http://www.matrixscience.com/

Mascot Search Results

Peptide View

MS/MS Fragmentation of **QLVESDINGLRR**
Found in **sp|Q15323|K1H1_HUMAN**, Keratin, type I cuticular Ha1 OS=Homo sapiens GN=KRT31 PE=2 SV=3

Match to Query 14337: 1399.740688 from(700.877620,2+) intensity(7092063.0000) scans(7094) rtinseconds(1349) index(20471)
Title: 160219_Sunil_SDSI_A_Spectrum073775_scans__7094_RTINSECONDS=1349
Data file C:\\Sunil\\TKAP\\T\\T160219_Sunil_SDSI_A.mgf

Click mouse within plot area to zoom in by factor of two about that point
Or, Plot from 100 to 1400 Da Full range
Label all possible matches ○ Label matches used for scoring ◉

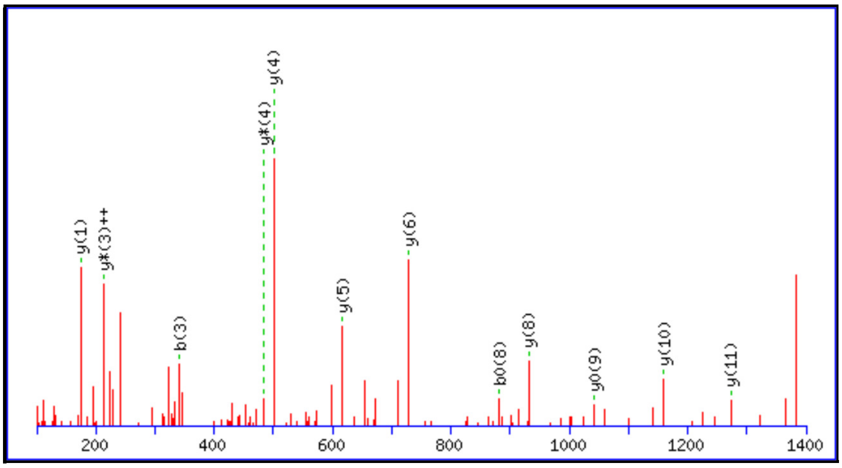

Monoisotopic mass of neutral peptide Mr(calc): 1399.7419
Fixed modifications: Carbamidomethyl (C) (apply to specified residues or termini only)
Variable modifications:
N8 : Deamidated (NQ)
Ions Score: 73 **Expect:** 9e-006
Matches : 12/114 fragment ions using 13 most intense peaks (help)

#	b	b++	b*	b*++	b0	b0++	Seq.	y	y++	y*	y*++	y0	y0++	#
1	129.0659	65.0366	112.0393	56.5233			Q							12
2	242.1499	121.5786	225.1234	113.0653			L	***1272.6906***	636.8490	1255.6641	628.3357	1254.6801	627.8437	11
3	**341.2183**	171.1128	324.1918	162.5995			V	***1159.6066***	580.3069	1142.5800	571.7937	1141.5960	571.3016	10
4	470.2609	235.6341	453.2344	227.1208	452.2504	226.6288	E	1060.5382	530.7727	1043.5116	522.2594	1042.5276	521.7674	9
5	557.2930	279.1501	540.2664	270.6368	539.2824	270.1448	S	***931.4956***	466.2514	914.4690	457.7381	913.4850	457.2461	8
6	672.3199	336.6636	655.2933	328.1503	654.3093	327.6583	D	844.4635	422.7354	827.4370	414.2221	826.4530	413.7301	7
7	785.4040	393.2056	768.3774	384.6923	767.3934	384.2003	I	***729.4366***	365.2219	712.4100	356.7087			6
8	900.4309	450.7191	883.4044	442.2058	882.4203	441.7138	N	***616.3525***	308.6799	599.3260	300.1666			5
9	957.4524	479.2298	940.4258	470.7165	939.4418	470.2245	G	***501.3256***	251.1664	484.2990	242.6532			4
10	1070.5364	535.7719	1053.5099	527.2586	1052.5259	526.7666	L	444.3041	222.6557	427.2776	214.1424			3
11	1226.6375	613.8224	1209.6110	605.3091	1208.6270	604.8171	R	331.2201	166.1137	314.1935	157.6004			2
12							R	***175.1190***	88.0631	158.0924	79.5498			1

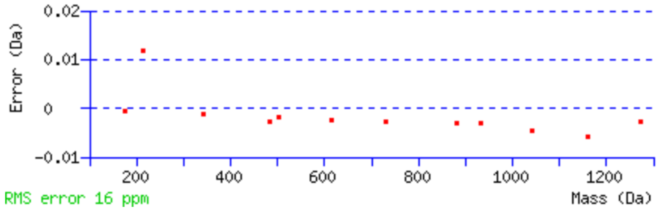

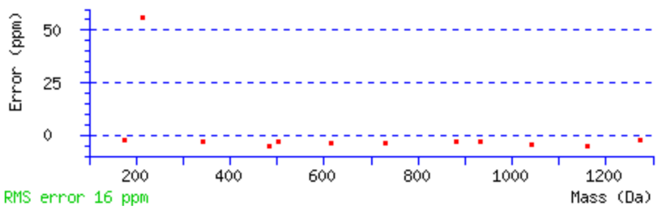

NCBI **BLAST** search of QLVESDINGLRR
(Parameters: blastp, nr protein database, expect=20000, no filter, PAM30)
Other BLAST web gateways

All matches to this query

Score	Mr(calc):	Delta	Sequence
72.8	1399.7419	-0.0012	QLVESDINGLRR
10.6	1399.7307	0.0100	LENLDSDVVQLR
5.7	1399.7419	-0.0012	QITTVAQVAAQNR
4.8	1399.7419	-0.0012	KEQERLAQLER
4.3	1399.7307	0.0100	XLLQAGAELSVER
3.6	1399.7307	0.0100	ALGLEESLVQEGR
2.8	1399.7532	-0.0125	XRLAEVSGGGTGLR
2.2	1399.7419	-0.0012	QLVESDINGLRR
2.1	1399.7460	-0.0053	QVLQGLDYLHSK
2.1	1399.7531	-0.0125	QRNSSVAAAQLVR

Mascot: http://www.matrixscience.com/

Mascot Search Results

Peptide View

MS/MS Fragmentation of **QLVESDINGLRR**
Found in **sp|Q15323|K1H1_HUMAN**, Keratin, type I cuticular Ha1 OS=Homo sapiens GN=KRT31 PE=2 SV=3

Match to Query 14349: 1399.742112 from(467.587980,3+) intensity(218274896.0000) scans(8184) rtinseconds(1536) index(21448)
Title: 160219_Sunil_SDSI_A_Spectrum074752_scans__8184_RTINSECONDS=1536
Data file C:\\Sunil\\TKAP\\T\\T160219_Sunil_SDSI_A.mgf

Click mouse within plot area to zoom in by factor of two about that point
Or, Plot from 100 to 1400 Da Full range
Label all possible matches Label matches used for scoring

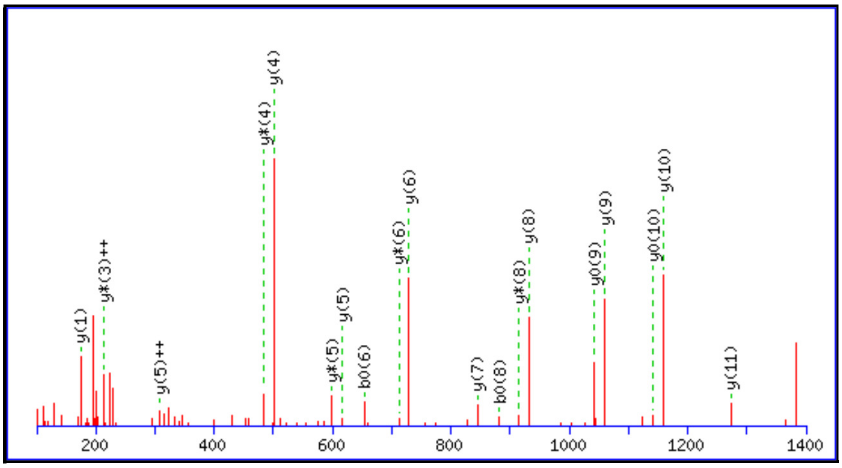

Monoisotopic mass of neutral peptide Mr(calc): 1399.7419
Fixed modifications: Carbamidomethyl (C) (apply to specified residues or termini only)
Variable modifications:
N8 : Deamidated (NQ)
Ions Score: 76 **Expect:** 4.6e-006
Matches : 19/114 fragment ions using 25 most intense peaks (help)

#	b	b++	b*	b*++	b0	b0++	Seq.	y	y++	y*	y*++	y0	y0++	#
1	129.0659	65.0366	112.0393	56.5233			Q							12
2	242.1499	121.5786	225.1234	113.0653			L	1272.6906	636.8490	1255.6641	628.3357	1254.6801	627.8437	11
3	341.2183	171.1128	324.1918	162.5995			V	1159.6066	580.3069	1142.5800	571.7937	1141.5960	571.3016	10
4	470.2609	235.6341	453.2344	227.1208	452.2504	226.6288	E	1060.5382	530.7727	1043.5116	522.2594	1042.5276	521.7674	9
5	557.2930	279.1501	540.2664	270.6368	539.2824	270.1448	S	931.4956	466.2514	914.4690	457.7381	913.4850	457.2461	8
6	672.3199	336.6636	655.2933	328.1503	654.3093	327.6583	D	844.4635	422.7354	827.4370	414.2221	826.4530	413.7301	7
7	785.4040	393.2056	768.3774	384.6923	767.3934	384.2003	I	729.4366	365.2219	712.4100	356.7087			6
8	900.4309	450.7191	883.4044	442.2058	882.4203	441.7138	N	616.3525	308.6799	599.3260	300.1666			5
9	957.4524	479.2298	940.4258	470.7165	939.4418	470.2245	G	501.3256	251.1664	484.2990	242.6532			4
10	1070.5364	535.7719	1053.5099	527.2586	1052.5259	526.7666	L	444.3041	222.6557	427.2776	214.1424			3
11	1226.6375	613.8224	1209.6110	605.3091	1208.6270	604.8171	R	331.2201	166.1137	314.1935	157.6004			2
12							R	175.1190	88.0631	158.0924	79.5498			1

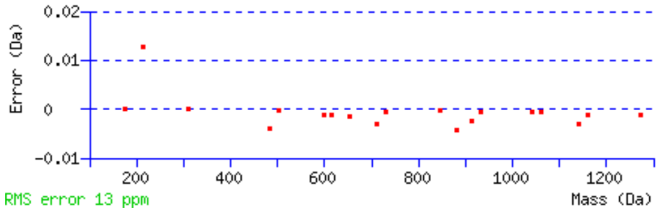

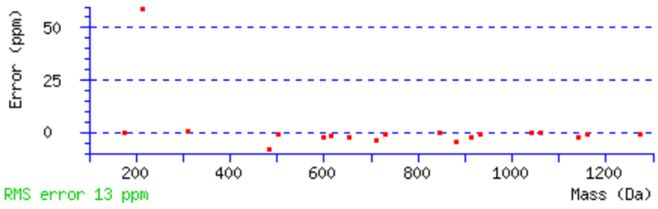

NCBI **BLAST** search of QLVESDINGLRR
(Parameters: blastp, nr protein database, expect=20000, no filter, PAM30)
Other BLAST web gateways

All matches to this query

Score	Mr(calc):	Delta	Sequence
76.0	1399.7419	0.0002	QLVESDINGLRR
7.0	1397.7336	2.0085	YIMKQLNSTRK
2.1	1399.7558	-0.0137	XLVDLAANSLLNK
1.9	1399.7460	-0.0038	QILDGVNYLHTK
0.5	1399.7460	-0.0038	QVLQGLDYLHSK
0.0	1399.7307	0.0114	LENLDSDVVQLR

Mascot: http://www.matrixscience.com/

Mascot Search Results

Peptide View

MS/MS Fragmentation of **QLVESDINGLRR**
Found in **sp|O76009|KT33A_HUMAN**, Keratin, type I cuticular Ha3-I OS=Homo sapiens GN=KRT33A PE=2 SV=2

Match to Query 14337: 1399.740688 from(700.877620,2+) intensity(7092063.0000) scans(7094) rtinseconds(1349) index(20471)
Title: 160219_Sunil_SDSI_A_Spectrum073775_scans__7094_RTINSECONDS=1349
Data file C:\\Sunil\\TKAP\\T\\T160219_Sunil_SDSI_A.mgf

Click mouse within plot area to zoom in by factor of two about that point
Or, Plot from 100 to 1400 Da Full range
Label all possible matches ○ Label matches used for scoring ◉

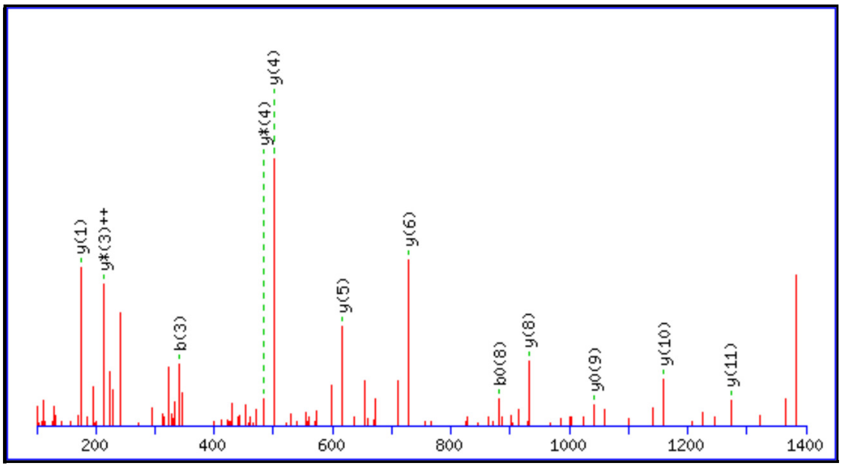

Monoisotopic mass of neutral peptide Mr(calc): 1399.7419
Fixed modifications: Carbamidomethyl (C) (apply to specified residues or termini only)
Variable modifications:
N8 : Deamidated (NQ)
Ions Score: 73 **Expect:** 9e-006
Matches : 12/114 fragment ions using 13 most intense peaks (help)

#	b	b^{++}	b*	b^{*++}	b^0	b^{0++}	Seq.	y	y^{++}	y*	y^{*++}	y^0	y^{0++}	#
1	129.0659	65.0366	112.0393	56.5233			Q							12
2	242.1499	121.5786	225.1234	113.0653			L	***1272.6906***	636.8490	1255.6641	628.3357	1254.6801	627.8437	11
3	**341.2183**	171.1128	324.1918	162.5995			V	***1159.6066***	580.3069	1142.5800	571.7937	1141.5960	571.3016	10
4	470.2609	235.6341	453.2344	227.1208	452.2504	226.6288	E	1060.5382	530.7727	1043.5116	522.2594	1042.5276	521.7674	9
5	557.2930	279.1501	540.2664	270.6368	539.2824	270.1448	S	***931.4956***	466.2514	914.4690	457.7381	913.4850	457.2461	8
6	672.3199	336.6636	655.2933	328.1503	654.3093	327.6583	D	844.4635	422.7354	827.4370	414.2221	826.4530	413.7301	7
7	785.4040	393.2056	768.3774	384.6923	767.3934	384.2003	I	***729.4366***	365.2219	712.4100	356.7087			6
8	900.4309	450.7191	883.4044	442.2058	882.4203	441.7138	N	***616.3525***	308.6799	599.3260	300.1666			5
9	957.4524	479.2298	940.4258	470.7165	939.4418	470.2245	G	***501.3256***	251.1664	484.2990	242.6532			4
10	1070.5364	535.7719	1053.5099	527.2586	1052.5259	526.7666	L	444.3041	222.6557	427.2776	214.1424			3
11	1226.6375	613.8224	1209.6110	605.3091	1208.6270	604.8171	R	331.2201	166.1137	314.1935	157.6004			2
12							R	***175.1190***	88.0631	158.0924	79.5498			1

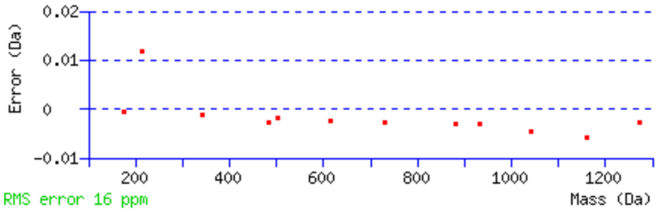

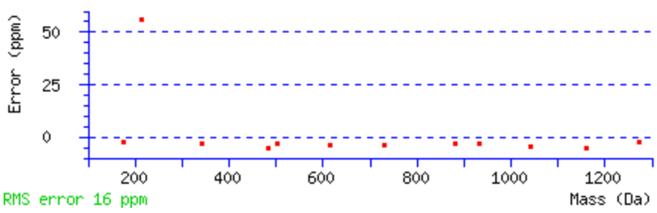

NCBI **BLAST** search of QLVESDINGLRR
(Parameters: blastp, nr protein database, expect=20000, no filter, PAM30)
Other BLAST web gateways

All matches to this query

Score	Mr(calc):	Delta	Sequence
72.8	1399.7419	-0.0012	QLVESDINGLRR
10.6	1399.7307	0.0100	LENLDSDVVQLR
5.7	1399.7419	-0.0012	QITTVAQVAAQNR
4.8	1399.7419	-0.0012	KEQERLAQLER
4.3	1399.7307	0.0100	XLLQAGAELSVER
3.6	1399.7307	0.0100	ALGLEESLVQEGR
2.8	1399.7532	-0.0125	XRLAEVSGGGTGLR
2.2	1399.7419	-0.0012	QLVESDINGLRR
2.1	1399.7460	-0.0053	QVLQGLDYLHSK
2.1	1399.7531	-0.0125	QRNSSVAAAQLVR

Mascot: http://www.matrixscience.com/

Mascot Search Results

Peptide View

MS/MS Fragmentation of **QNQEYQVLLDVR**
Found in **sp|O76009|KT33A_HUMAN**, Keratin, type I cuticular Ha3-I OS=Homo sapiens GN=KRT33A PE=2 SV=2

Match to Query 17898: 1504.750948 from(753.382750,2+) intensity(3760134.7500) scans(14856) rtinseconds(2706) index(11709)
Title: 160219_Sunil_SDSI_A_Spectrum063837_scans__14856_RTINSECONDS=2706
Data file C:\\Sunil\\TKAP\\T\\T160219_Sunil_SDSI_A.mgf

Click mouse within plot area to zoom in by factor of two about that point
Or, Plot from 100 to 1500 Da Full range
Label all possible matches ○ Label matches used for scoring ◉

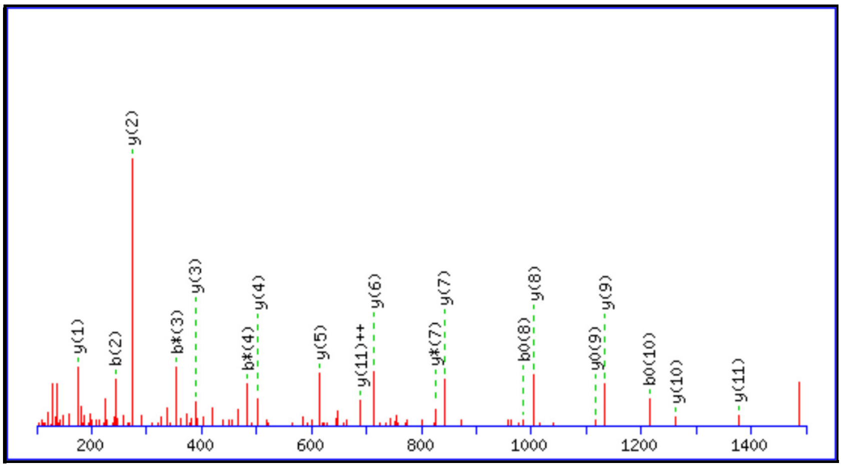

Monoisotopic mass of neutral peptide Mr(calc): 1504.7522
Fixed modifications: Carbamidomethyl (C) (apply to specified residues or termini only)
Variable modifications:
N2 : Deamidated (NQ)
Ions Score: 103 **Expect:** 1.3e-008
Matches : 19/122 fragment ions using 26 most intense peaks (help)

#	b	b^{++}	b*	b^{*++}	b^0	b^{0++}	Seq.	y	y^{++}	y*	y^{*++}	y^0	y^{0++}	#
1	129.0659	65.0366	112.0393	56.5233			Q							12
2	**244.0928**	122.5500	227.0662	114.0368			N	***1377.7009***	689.3541	1360.6743	680.8408	1359.6903	680.3488	11
3	372.1514	186.5793	355.1248	178.0661			Q	***1262.6739***	631.8406	1245.6474	623.3273	1244.6634	622.8353	10
4	501.1940	251.1006	484.1674	242.5873	483.1834	242.0953	E	***1134.6154***	567.8113	1117.5888	559.2980	1116.6048	558.8060	9
5	664.2573	332.6323	647.2307	324.1190	646.2467	323.6270	Y	***1005.5728***	503.2900	988.5462	494.7767	987.5622	494.2847	8
6	792.3159	396.6616	775.2893	388.1483	774.3053	387.6563	Q	***842.5094***	421.7584	825.4829	413.2451	824.4989	412.7531	7
7	891.3843	446.1958	874.3577	437.6825	873.3737	437.1905	V	***714.4509***	357.7291	697.4243	349.2158	696.4403	348.7238	6
8	1004.4684	502.7378	987.4418	494.2245	986.4578	493.7325	L	***615.3824***	308.1949	598.3559	299.6816	597.3719	299.1896	5
9	1117.5524	559.2798	1100.5259	550.7666	1099.5419	550.2746	L	***502.2984***	251.6528	485.2718	243.1396	484.2878	242.6475	4
10	1232.5794	616.7933	1215.5528	608.2800	1214.5688	607.7880	D	***389.2143***	195.1108	372.1878	186.5975	371.2037	186.1055	3
11	1331.6478	666.3275	1314.6212	657.8143	1313.6372	657.3222	V	***274.1874***	137.5973	257.1608	129.0840			2
12							R	***175.1190***	88.0631	158.0924	79.5498			1

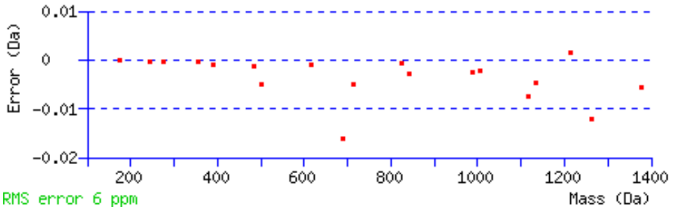

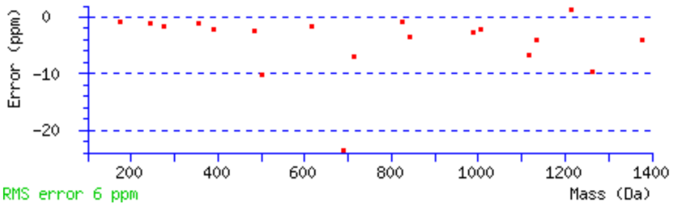

NCBI **BLAST** search of QNQEYQVLLDVR
(Parameters: blastp, nr protein database, expect=20000, no filter, PAM30)
Other BLAST web gateways

All matches to this query

Score	Mr(calc):	Delta	Sequence
102.7	1504.7522	-0.0012	QNQEYQVLLDVR
83.3	1504.7522	-0.0012	QNQEYQVLLDVR
72.5	1504.7522	-0.0012	QNQEYQVLLDVR
32.1	1504.7522	-0.0012	QNQEYQVLLDVR
2.8	1504.7456	0.0053	QMRELLSGWDVR
2.8	1504.7416	0.0094	QEAGKMRSSQEVR
2.5	1504.7569	-0.0059	AGHCPNPGISLGAVR
2.5	1504.7555	-0.0046	CDLLSKSQALNVR
0.4	1504.7481	0.0028	DQLSSRSELNTVR

Mascot: http://www.matrixscience.com/

Mascot Search Results

Peptide View

MS/MS Fragmentation of **QNQEYQVLLDVR**
Found in **sp|Q15323|K1H1_HUMAN**, Keratin, type I cuticular Ha1 OS=Homo sapiens GN=KRT31 PE=2 SV=3

Match to Query 17898: 1504.750948 from(753.382750,2+) intensity(3760134.7500) scans(14856) rtinseconds(2706) index(11709)
Title: 160219_Sunil_SDSI_A_Spectrum063837_scans__14856_RTINSECONDS=2706
Data file C:\\Sunil\\TKAP\\T\\T160219_Sunil_SDSI_A.mgf

Click mouse within plot area to zoom in by factor of two about that point
Or, Plot from 100 to 1500 Da Full range
Label all possible matches ○ Label matches used for scoring ●

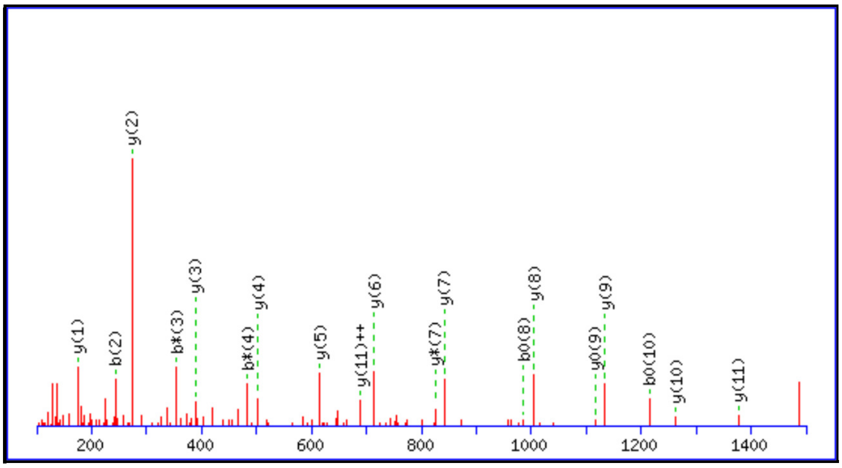

Monoisotopic mass of neutral peptide Mr(calc): 1504.7522
Fixed modifications: Carbamidomethyl (C) (apply to specified residues or termini only)
Variable modifications:
N2 : Deamidated (NQ)
Ions Score: 103 **Expect:** 1.3e-008
Matches : 19/122 fragment ions using 26 most intense peaks (help)

#	b	b++	b*	b*++	b0	b0++	Seq.	y	y++	y*	y*++	y0	y0++	#
1	129.0659	65.0366	112.0393	56.5233			Q							12
2	**244.0928**	122.5500	227.0662	114.0368			N	***1377.7009***	689.3541	1360.6743	680.8408	1359.6903	680.3488	11
3	372.1514	186.5793	355.1248	178.0661			Q	***1262.6739***	631.8406	1245.6474	623.3273	1244.6634	622.8353	10
4	501.1940	251.1006	484.1674	242.5873	483.1834	242.0953	E	***1134.6154***	567.8113	1117.5888	559.2980	1116.6048	558.8060	9
5	664.2573	332.6323	647.2307	324.1190	646.2467	323.6270	Y	***1005.5728***	503.2900	988.5462	494.7767	987.5622	494.2847	8
6	792.3159	396.6616	775.2893	388.1483	774.3053	387.6563	Q	***842.5094***	421.7584	825.4829	413.2451	824.4989	412.7531	7
7	891.3843	446.1958	874.3577	437.6825	873.3737	437.1905	V	***714.4509***	357.7291	697.4243	349.2158	696.4403	348.7238	6
8	1004.4684	502.7378	987.4418	494.2245	986.4578	493.7325	L	***615.3824***	308.1949	598.3559	299.6816	597.3719	299.1896	5
9	1117.5524	559.2798	1100.5259	550.7666	1099.5419	550.2746	L	***502.2984***	251.6528	485.2718	243.1396	484.2878	242.6475	4
10	1232.5794	616.7933	1215.5528	608.2800	1214.5688	607.7880	D	***389.2143***	195.1108	372.1878	186.5975	371.2037	186.1055	3
11	1331.6478	666.3275	1314.6212	657.8143	1313.6372	657.3222	V	***274.1874***	137.5973	257.1608	129.0840			2
12							R	***175.1190***	88.0631	158.0924	79.5498			1

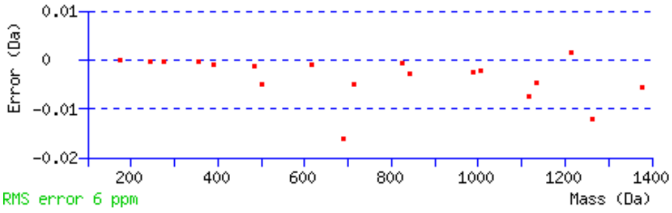

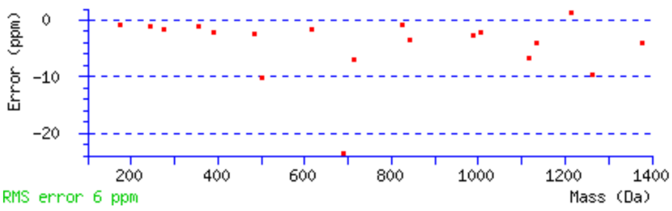

NCBI **BLAST** search of QNQEYQVLLDVR
(Parameters: blastp, nr protein database, expect=20000, no filter, PAM30)
Other BLAST web gateways

All matches to this query

Score	Mr(calc):	Delta	Sequence
102.7	1504.7522	-0.0012	QNQEYQVLLDVR
83.3	1504.7522	-0.0012	QNQEYQVLLDVR
72.5	1504.7522	-0.0012	QNQEYQVLLDVR
32.1	1504.7522	-0.0012	QNQEYQVLLDVR
2.8	1504.7456	0.0053	QMRELLSGWDVR
2.8	1504.7416	0.0094	QEAGKMRSSQEVR
2.5	1504.7569	-0.0059	AGHCPNPGISLGAVR
2.5	1504.7555	-0.0046	CDLLSKSQALNVR
0.4	1504.7481	0.0028	DQLSSRSELNTVR

Mascot: http://www.matrixscience.com/

Mascot Search Results

Peptide View

MS/MS Fragmentation of **QNQEYQVLLDVR**
Found in **sp|Q15323|K1H1_HUMAN**, Keratin, type I cuticular Ha1 OS=Homo sapiens GN=KRT31 PE=2 SV=3

Match to Query 17898: 1504.750948 from(753.382750,2+) intensity(3760134.7500) scans(14856) rtinseconds(2706) index(11709)
Title: 160219_Sunil_SDSI_A_Spectrum063837_scans__14856_RTINSECONDS=2706
Data file C:\\Sunil\\TKAP\\T\\T160219_Sunil_SDSI_A.mgf

Click mouse within plot area to zoom in by factor of two about that point
Or, Plot from 100 to 1500 Da Full range
Label all possible matches Label matches used for scoring

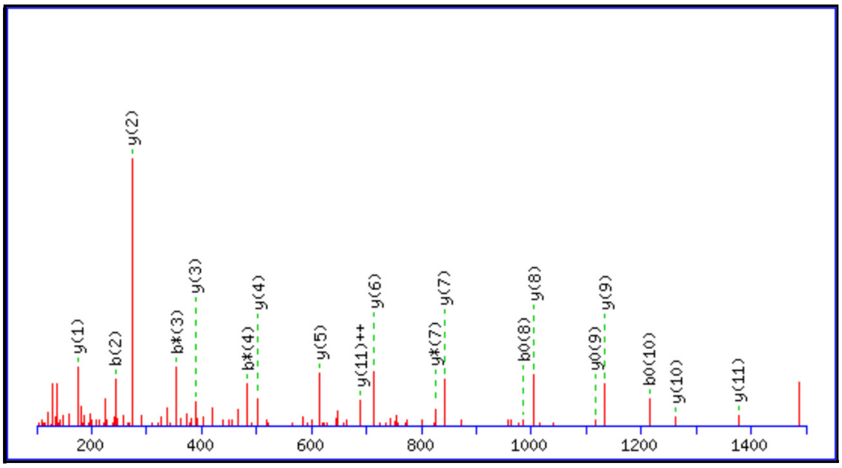

Monoisotopic mass of neutral peptide Mr(calc): 1504.7522
Fixed modifications: Carbamidomethyl (C) (apply to specified residues or termini only)
Variable modifications:
N2 : Deamidated (NQ)
Ions Score: 103 **Expect:** 1.3e-008
Matches : 19/122 fragment ions using 26 most intense peaks (help)

#	b	b++	b*	b*++	b0	b0++	Seq.	y	y++	y*	y*++	y0	y0++	#
1	129.0659	65.0366	112.0393	56.5233			Q							12
2	**244.0928**	122.5500	227.0662	114.0368			N	***1377.7009***	689.3541	1360.6743	680.8408	1359.6903	680.3488	11
3	372.1514	186.5793	355.1248	178.0661			Q	***1262.6739***	631.8406	1245.6474	623.3273	1244.6634	622.8353	10
4	501.1940	251.1006	484.1674	242.5873	483.1834	242.0953	E	***1134.6154***	567.8113	1117.5888	559.2980	1116.6048	558.8060	9
5	664.2573	332.6323	647.2307	324.1190	646.2467	323.6270	Y	***1005.5728***	503.2900	988.5462	494.7767	987.5622	494.2847	8
6	792.3159	396.6616	775.2893	388.1483	774.3053	387.6563	Q	***842.5094***	421.7584	825.4829	413.2451	824.4989	412.7531	7
7	891.3843	446.1958	874.3577	437.6825	873.3737	437.1905	V	***714.4509***	357.7291	697.4243	349.2158	696.4403	348.7238	6
8	1004.4684	502.7378	987.4418	494.2245	986.4578	493.7325	L	***615.3824***	308.1949	598.3559	299.6816	597.3719	299.1896	5
9	1117.5524	559.2798	1100.5259	550.7666	1099.5419	550.2746	L	***502.2984***	251.6528	485.2718	243.1396	484.2878	242.6475	4
10	1232.5794	616.7933	1215.5528	608.2800	1214.5688	607.7880	D	***389.2143***	195.1108	372.1878	186.5975	371.2037	186.1055	3
11	1331.6478	666.3275	1314.6212	657.8143	1313.6372	657.3222	V	***274.1874***	137.5973	257.1608	129.0840			2
12							R	***175.1190***	88.0631	158.0924	79.5498			1

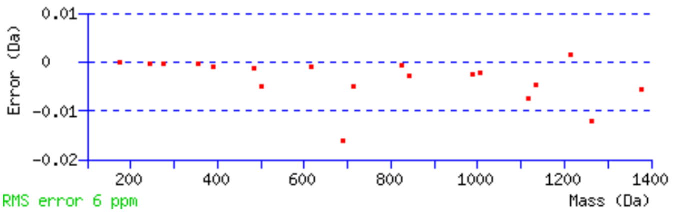

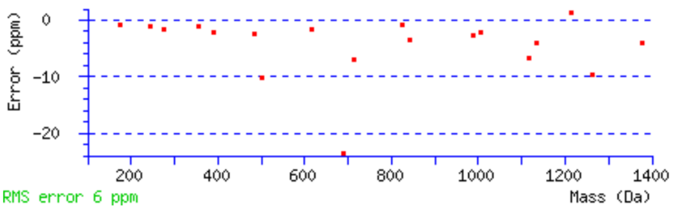

NCBI **BLAST** search of QNQEYQVLLDVR
(Parameters: blastp, nr protein database, expect=20000, no filter, PAM30)
Other BLAST web gateways

All matches to this query

Score	Mr(calc):	Delta	Sequence
102.7	1504.7522	-0.0012	QNQEYQVLLDVR
83.3	1504.7522	-0.0012	QNQEYQVLLDVR
72.5	1504.7522	-0.0012	QNQEYQVLLDVR
32.1	1504.7522	-0.0012	QNQEYQVLLDVR
2.8	1504.7456	0.0053	QMRELLSGWDVR
2.8	1504.7416	0.0094	QEAGKMRSSQEVR
2.5	1504.7569	-0.0059	AGHCPNPGISLGAVR
2.5	1504.7555	-0.0046	CDLLSKSQALNVR
0.4	1504.7481	0.0028	DQLSSRSELNTVR

Mascot: http://www.matrixscience.com/

Mascot Search Results

Peptide View

MS/MS Fragmentation of **QNQEYQVLLDVR**
Found in **sp|Q15323|K1H1_HUMAN**, Keratin, type I cuticular Ha1 OS=Homo sapiens GN=KRT31 PE=2 SV=3

Match to Query 17898: 1504.750948 from(753.382750,2+) intensity(3760134.7500) scans(14856) rtinseconds(2706) index(11709)
Title: 160219_Sunil_SDSI_A_Spectrum063837_scans__14856_RTINSECONDS=2706
Data file C:\\Sunil\\TKAP\\T\\T160219_Sunil_SDSI_A.mgf

Click mouse within plot area to zoom in by factor of two about that point
Or, Plot from 100 to 1500 Da Full range
Label all possible matches Label matches used for scoring

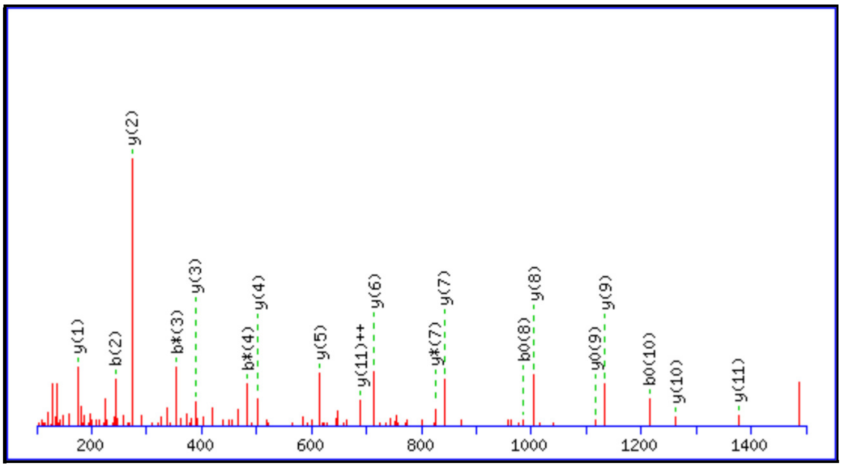

Monoisotopic mass of neutral peptide Mr(calc): 1504.7522
Fixed modifications: Carbamidomethyl (C) (apply to specified residues or termini only)
Variable modifications:
N2 : Deamidated (NQ)
Ions Score: 103 **Expect:** 1.3e-008
Matches : 19/122 fragment ions using 26 most intense peaks (help)

#	b	b++	b*	b*++	b0	b0++	Seq.	y	y++	y*	y*++	y0	y0++	#
1	129.0659	65.0366	112.0393	56.5233			Q							12
2	**244.0928**	122.5500	227.0662	114.0368			N	***1377.7009***	689.3541	1360.6743	680.8408	1359.6903	680.3488	11
3	372.1514	186.5793	355.1248	178.0661			Q	***1262.6739***	631.8406	1245.6474	623.3273	1244.6634	622.8353	10
4	501.1940	251.1006	484.1674	242.5873	483.1834	242.0953	E	***1134.6154***	567.8113	1117.5888	559.2980	1116.6048	558.8060	9
5	664.2573	332.6323	647.2307	324.1190	646.2467	323.6270	Y	***1005.5728***	503.2900	988.5462	494.7767	987.5622	494.2847	8
6	792.3159	396.6616	775.2893	388.1483	774.3053	387.6563	Q	***842.5094***	421.7584	825.4829	413.2451	824.4989	412.7531	7
7	891.3843	446.1958	874.3577	437.6825	873.3737	437.1905	V	***714.4509***	357.7291	697.4243	349.2158	696.4403	348.7238	6
8	1004.4684	502.7378	987.4418	494.2245	986.4578	493.7325	L	***615.3824***	308.1949	598.3559	299.6816	597.3719	299.1896	5
9	1117.5524	559.2798	1100.5259	550.7666	1099.5419	550.2746	L	***502.2984***	251.6528	485.2718	243.1396	484.2878	242.6475	4
10	1232.5794	616.7933	1215.5528	608.2800	1214.5688	607.7880	D	***389.2143***	195.1108	372.1878	186.5975	371.2037	186.1055	3
11	1331.6478	666.3275	1314.6212	657.8143	1313.6372	657.3222	V	***274.1874***	137.5973	257.1608	129.0840			2
12							R	***175.1190***	88.0631	158.0924	79.5498			1

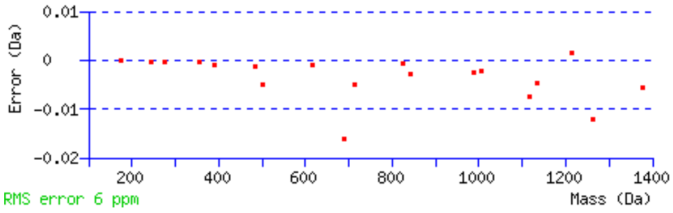

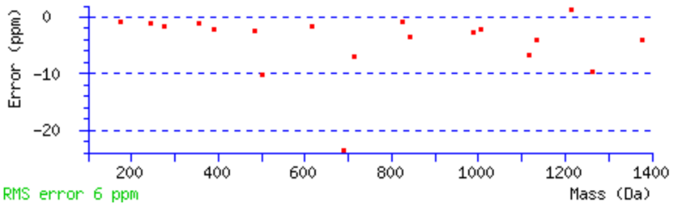

NCBI **BLAST** search of QNQEYQVLLDVR
(Parameters: blastp, nr protein database, expect=20000, no filter, PAM30)
Other BLAST web gateways

All matches to this query

Score	Mr(calc):	Delta	Sequence
102.7	1504.7522	-0.0012	QNQEYQVLLDVR
83.3	1504.7522	-0.0012	QNQEYQVLLDVR
72.5	1504.7522	-0.0012	QNQEYQVLLDVR
32.1	1504.7522	-0.0012	QNQEYQVLLDVR
2.8	1504.7456	0.0053	QMRELLSGWDVR
2.8	1504.7416	0.0094	QEAGKMRSSQEVR
2.5	1504.7569	-0.0059	AGHCPNPGISLGAVR
2.5	1504.7555	-0.0046	CDLLSKSQALNVR
0.4	1504.7481	0.0028	DQLSSRSELNTVR

Mascot: http://www.matrixscience.com/

Mascot Search Results

Peptide View

MS/MS Fragmentation of **QNQEYQVLLDVR**
Found in **sp|Q15323|K1H1_HUMAN**, Keratin, type I cuticular Ha1 OS=Homo sapiens GN=KRT31 PE=2 SV=3

Match to Query 17898: 1504.750948 from(753.382750,2+) intensity(3760134.7500) scans(14856) rtinseconds(2706) index(11709)
Title: 160219_Sunil_SDSI_A_Spectrum063837_scans__14856_RTINSECONDS=2706
Data file C:\\Sunil\\TKAP\\T\\T160219_Sunil_SDSI_A.mgf

Click mouse within plot area to zoom in by factor of two about that point
Or, Plot from 100 to 1500 Da Full range
Label all possible matches ○ Label matches used for scoring ●

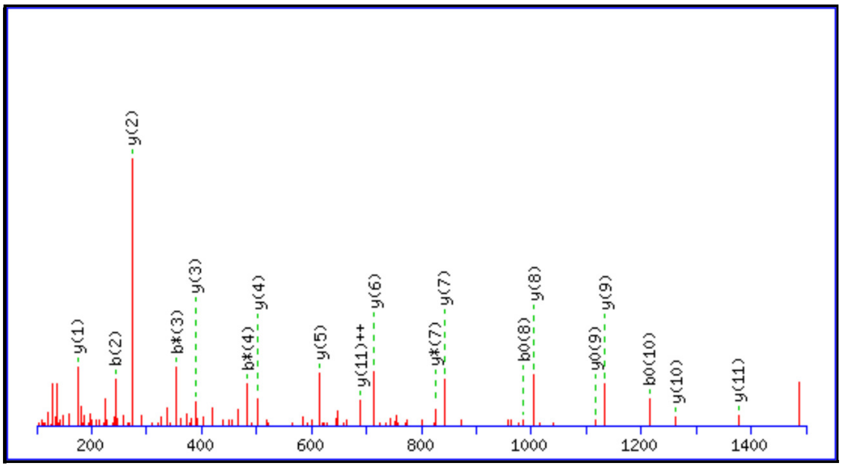

Monoisotopic mass of neutral peptide Mr(calc): 1504.7522
Fixed modifications: Carbamidomethyl (C) (apply to specified residues or termini only)
Variable modifications:
N2 : Deamidated (NQ)
Ions Score: 103 **Expect:** 1.3e-008
Matches : 19/122 fragment ions using 26 most intense peaks (help)

#	b	b++	b*	b*++	b0	b0++	Seq.	y	y++	y*	y*++	y0	y0++	#
1	129.0659	65.0366	112.0393	56.5233			Q							12
2	244.0928	122.5500	227.0662	114.0368			N	1377.7009	689.3541	1360.6743	680.8408	1359.6903	680.3488	11
3	372.1514	186.5793	355.1248	178.0661			Q	1262.6739	631.8406	1245.6474	623.3273	1244.6634	622.8353	10
4	501.1940	251.1006	484.1674	242.5873	483.1834	242.0953	E	1134.6154	567.8113	1117.5888	559.2980	1116.6048	558.8060	9
5	664.2573	332.6323	647.2307	324.1190	646.2467	323.6270	Y	1005.5728	503.2900	988.5462	494.7767	987.5622	494.2847	8
6	792.3159	396.6616	775.2893	388.1483	774.3053	387.6563	Q	842.5094	421.7584	825.4829	413.2451	824.4989	412.7531	7
7	891.3843	446.1958	874.3577	437.6825	873.3737	437.1905	V	714.4509	357.7291	697.4243	349.2158	696.4403	348.7238	6
8	1004.4684	502.7378	987.4418	494.2245	986.4578	493.7325	L	615.3824	308.1949	598.3559	299.6816	597.3719	299.1896	5
9	1117.5524	559.2798	1100.5259	550.7666	1099.5419	550.2746	L	502.2984	251.6528	485.2718	243.1396	484.2878	242.6475	4
10	1232.5794	616.7933	1215.5528	608.2800	1214.5688	607.7880	D	389.2143	195.1108	372.1878	186.5975	371.2037	186.1055	3
11	1331.6478	666.3275	1314.6212	657.8143	1313.6372	657.3222	V	274.1874	137.5973	257.1608	129.0840			2
12							R	175.1190	88.0631	158.0924	79.5498			1

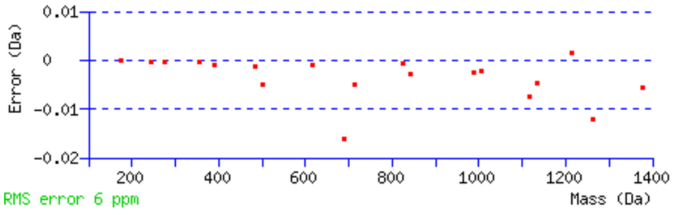

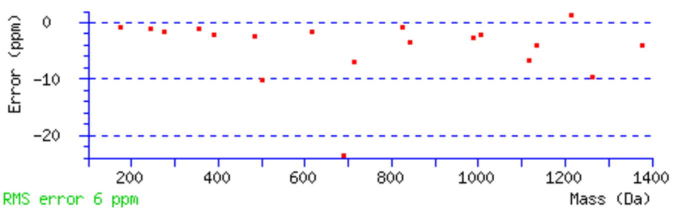

NCBI **BLAST** search of QNQEYQVLLDVR
(Parameters: blastp, nr protein database, expect=20000, no filter, PAM30)
Other BLAST web gateways

All matches to this query

Score	Mr(calc):	Delta	Sequence
102.7	1504.7522	-0.0012	QNQEYQVLLDVR
83.3	1504.7522	-0.0012	QNQEYQVLLDVR
72.5	1504.7522	-0.0012	QNQEYQVLLDVR
32.1	1504.7522	-0.0012	QNQEYQVLLDVR
2.8	1504.7456	0.0053	QMRELLSGWDVR
2.8	1504.7416	0.0094	QEAGKMRSSQEVR
2.5	1504.7569	-0.0059	AGHCPNPGISLGAVR
2.5	1504.7555	-0.0046	CDLLSKSQALNVR
0.4	1504.7481	0.0028	DQLSSRSELNTVR

Mascot: http://www.matrixscience.com/

Mascot Search Results

Peptide View

MS/MS Fragmentation of **SDLERQNQEYQVLLDVR**
Found in **sp|Q15323|K1H1_HUMAN**, Keratin, type I cuticular Ha1 OS=Homo sapiens GN=KRT31 PE=2 SV=3

Match to Query 30904: 2105.041212 from(702.687680,3+) intensity(24180566.0000) scans(13642) rtinseconds(2474) index(26047)
Title: 160219_Sunil_SDSI_A_Spectrum079353_scans__13642_RTINSECONDS=2474
Data file C:\\Sunil\\TKAP\\T\\T160219_Sunil_SDSI_A.mgf

Click mouse within plot area to zoom in by factor of two about that point
Or, Plot from 100 to 2000 Da Full range
Label all possible matches ○ Label matches used for scoring ●

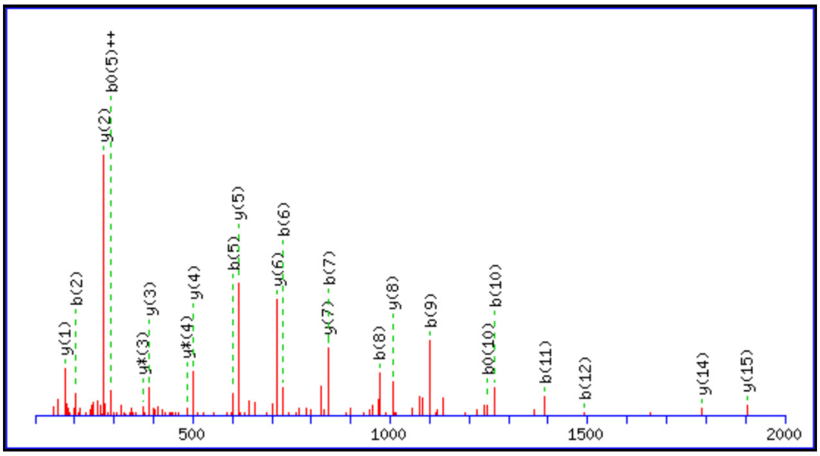

Monoisotopic mass of neutral peptide Mr(calc): 2105.0389
Fixed modifications: Carbamidomethyl (C) (apply to specified residues or termini only)
Variable modifications:
N7 : Deamidated (NQ)
Ions Score: 96 **Expect:** 6.8e-008
Matches : 23/180 fragment ions using 30 most intense peaks (help)

#	b	b++	b*	b*++	b0	b0++	Seq.	y	y++	y*	y*++	y0	y0++	#
1	88.0393	44.5233			70.0287	35.5180	S							17
2	***203.0662***	102.0368			185.0557	93.0315	D	2019.0142	1010.0107	2001.9876	1001.4974	2001.0036	1001.0054	16
3	316.1503	158.5788			298.1397	149.5735	L	***1903.9872***	952.4972	1886.9607	943.9840	1885.9767	943.4920	15
4	445.1929	223.1001			427.1823	214.0948	E	***1790.9032***	895.9552	1773.8766	887.4419	1772.8926	886.9499	14
5	***601.2940***	301.1506	584.2675	292.6374	583.2835	292.1454	R	1661.8606	831.4339	1644.8340	822.9206	1643.8500	822.4286	13
6	***729.3526***	365.1799	712.3260	356.6667	711.3420	356.1747	Q	1505.7595	753.3834	1488.7329	744.8701	1487.7489	744.3781	12
7	***844.3795***	422.6934	827.3530	414.1801	826.3690	413.6881	N	1377.7009	689.3541	1360.6743	680.8408	1359.6903	680.3488	11
8	***972.4381***	486.7227	955.4116	478.2094	954.4275	477.7174	Q	1262.6739	631.8406	1245.6474	623.3273	1244.6634	622.8353	10
9	***1101.4807***	551.2440	1084.4542	542.7307	1083.4701	542.2387	E	1134.6154	567.8113	1117.5888	559.2980	1116.6048	558.8060	9
10	***1264.5440***	632.7757	1247.5175	624.2624	1246.5335	623.7704	Y	***1005.5728***	503.2900	988.5462	494.7767	987.5622	494.2847	8
11	***1392.6026***	696.8049	1375.5761	688.2917	1374.5920	687.7997	Q	***842.5094***	421.7584	825.4829	413.2451	824.4989	412.7531	7
12	***1491.6710***	746.3392	1474.6445	737.8259	1473.6605	737.3339	V	***714.4509***	357.7291	697.4243	349.2158	696.4403	348.7238	6
13	1604.7551	802.8812	1587.7285	794.3679	1586.7445	793.8759	L	***615.3824***	308.1949	598.3559	299.6816	597.3719	299.1896	5
14	1717.8392	859.4232	1700.8126	850.9099	1699.8286	850.4179	L	***502.2984***	251.6528	485.2718	243.1396	484.2878	242.6475	4
15	1832.8661	916.9367	1815.8396	908.4234	1814.8555	907.9314	D	***389.2143***	195.1108	372.1878	186.5975	371.2037	186.1055	3
16	1931.9345	966.4709	1914.9080	957.9576	1913.9239	957.4656	V	***274.1874***	137.5973	257.1608	129.0840			2
17							R	***175.1190***	88.0631	158.0924	79.5498			1

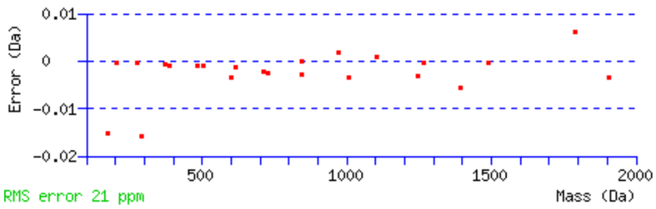

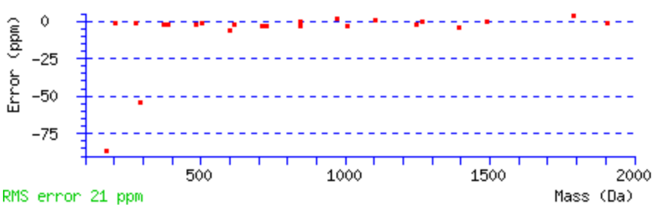

NCBI **BLAST** search of SDLERQNQEYQVLLDVR

(Parameters: blastp, nr protein database, expect=20000, no filter, PAM30)
Other BLAST web gateways

All matches to this query

Score	Mr(calc):	Delta	Sequence
96.1	2105.0389	0.0023	SDLERQNQEYQVLLDVR
84.6	2105.0389	0.0023	SDLERQNQEYQVLLDVR
84.6	2105.0389	0.0023	SDLERQNQEYQVLLDVR
66.6	2104.0549	0.9863	SDLERQNQEYQVLLDVR
61.8	2105.0389	0.0023	SDLERQNQEYQVLLDVR
4.4	2105.0370	0.0042	NWLEEWWLNVAYLDVR
0.5	2104.0380	1.0032	RMNTLQAIWMMDPKDVR
0.4	2104.0445	0.9967	FGTKCAACQLGIPPTQVVR
0.2	2103.0214	2.0198	MGSIMHPSQHARRPEDVR
0.2	2105.0220	0.0192	RMNTLQAIWMMDPKDVR

Mascot: http://www.matrixscience.com/

Mascot Search Results

Peptide View

MS/MS Fragmentation of **SDLERQNQEYQVLLDVR**
Found in **sp|O76009|KT33A_HUMAN**, Keratin, type I cuticular Ha3-I OS=Homo sapiens GN=KRT33A PE=2 SV=2

Match to Query 30904: 2105.041212 from(702.687680,3+) intensity(24180566.0000) scans(13642) rtinseconds(2474) index(26047)
Title: 160219_Sunil_SDSI_A_Spectrum079353_scans__13642_RTINSECONDS=2474
Data file C:\\Sunil\\TKAP\\T\\T160219_Sunil_SDSI_A.mgf

Click mouse within plot area to zoom in by factor of two about that point
Or, Plot from 100 to 2000 Da Full range
Label all possible matches ○ Label matches used for scoring ◉

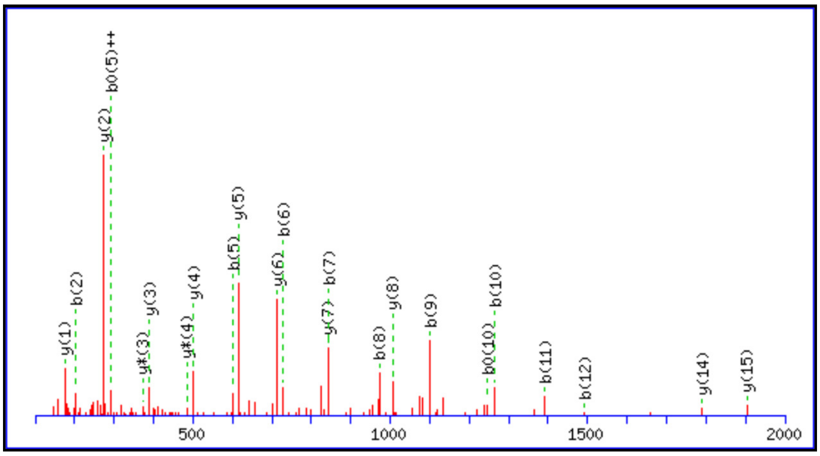

Monoisotopic mass of neutral peptide Mr(calc): 2105.0389
Fixed modifications: Carbamidomethyl (C) (apply to specified residues or termini only)
Variable modifications:
N7 : Deamidated (NQ)
Ions Score: 96 **Expect:** 6.8e-008
Matches : 23/180 fragment ions using 30 most intense peaks (help)

#	b	b^{++}	b*	b^{*++}	b^0	b^{0++}	Seq.	y	y^{++}	y*	y^{*++}	y^0	y^{0++}	#
1	88.0393	44.5233			70.0287	35.5180	S							17
2	***203.0662***	102.0368			185.0557	93.0315	D	2019.0142	1010.0107	2001.9876	1001.4974	2001.0036	1001.0054	16
3	316.1503	158.5788			298.1397	149.5735	L	***1903.9872***	952.4972	1886.9607	943.9840	1885.9767	943.4920	15
4	445.1929	223.1001			427.1823	214.0948	E	***1790.9032***	895.9552	1773.8766	887.4419	1772.8926	886.9499	14
5	***601.2940***	301.1506	584.2675	292.6374	583.2835	292.1454	R	1661.8606	831.4339	1644.8340	822.9206	1643.8500	822.4286	13
6	***729.3526***	365.1799	712.3260	356.6667	711.3420	356.1747	Q	1505.7595	753.3834	1488.7329	744.8701	1487.7489	744.3781	12
7	***844.3795***	422.6934	827.3530	414.1801	826.3690	413.6881	N	1377.7009	689.3541	1360.6743	680.8408	1359.6903	680.3488	11
8	***972.4381***	486.7227	955.4116	478.2094	954.4275	477.7174	Q	1262.6739	631.8406	1245.6474	623.3273	1244.6634	622.8353	10
9	***1101.4807***	551.2440	1084.4542	542.7307	1083.4701	542.2387	E	1134.6154	567.8113	1117.5888	559.2980	1116.6048	558.8060	9
10	***1264.5440***	632.7757	1247.5175	624.2624	1246.5335	623.7704	Y	***1005.5728***	503.2900	988.5462	494.7767	987.5622	494.2847	8
11	***1392.6026***	696.8049	1375.5761	688.2917	1374.5920	687.7997	Q	***842.5094***	421.7584	825.4829	413.2451	824.4989	412.7531	7
12	***1491.6710***	746.3392	1474.6445	737.8259	1473.6605	737.3339	V	***714.4509***	357.7291	697.4243	349.2158	696.4403	348.7238	6
13	1604.7551	802.8812	1587.7285	794.3679	1586.7445	793.8759	L	***615.3824***	308.1949	598.3559	299.6816	597.3719	299.1896	5
14	1717.8392	859.4232	1700.8126	850.9099	1699.8286	850.4179	L	***502.2984***	251.6528	485.2718	243.1396	484.2878	242.6475	4
15	1832.8661	916.9367	1815.8396	908.4234	1814.8555	907.9314	D	***389.2143***	195.1108	372.1878	186.5975	371.2037	186.1055	3
16	1931.9345	966.4709	1914.9080	957.9576	1913.9239	957.4656	V	***274.1874***	137.5973	257.1608	129.0840			2
17							R	***175.1190***	88.0631	158.0924	79.5498			1

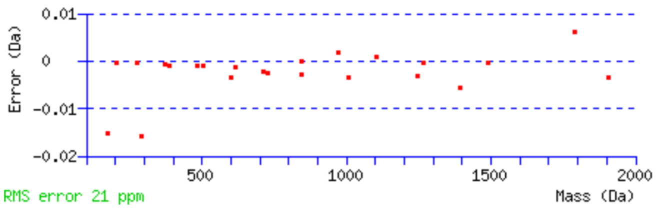

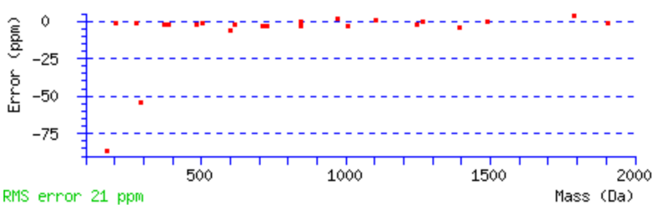

NCBI **BLAST** search of SDLERQNQEYQVLLDVR

(Parameters: blastp, nr protein database, expect=20000, no filter, PAM30)
Other BLAST web gateways

All matches to this query

Score	Mr(calc):	Delta	Sequence
96.1	2105.0389	0.0023	SDLERQNQEYQVLLDVR
84.6	2105.0389	0.0023	SDLERQNQEYQVLLDVR
84.6	2105.0389	0.0023	SDLERQNQEYQVLLDVR
66.6	2104.0549	0.9863	SDLERQNQEYQVLLDVR
61.8	2105.0389	0.0023	SDLERQNQEYQVLLDVR
4.4	2105.0370	0.0042	NWLEEWWLNVAYLDVR
0.5	2104.0380	1.0032	RMNTLQAIWMMDPKDVR
0.4	2104.0445	0.9967	FGTKCAACQLGIPPTQVVR
0.2	2103.0214	2.0198	MGSIMHPSQHARRPEDVR
0.2	2105.0220	0.0192	RMNTLQAIWMMDPKDVR

Mascot: http://www.matrixscience.com/

Mascot Search Results

Peptide View

MS/MS Fragmentation of **SENARLVVQIDNAK**
Found in **sp|Q15323|K1H1_HUMAN**, Keratin, type I cuticular Ha1 OS=Homo sapiens GN=KRT31 PE=2 SV=3

Match to Query 19384: 1556.815932 from(519.945920,3+) intensity(19003694.0000) scans(9965) rtinseconds(1867) index(7655)
Title: 160219_Sunil_SDSI_A_Spectrum059763_scans__9965_RTINSECONDS=1867
Data file C:\\Sunil\\TKAP\\T\\T160219_Sunil_SDSI_A.mgf

Click mouse within plot area to zoom in by factor of two about that point
Or, Plot from 100 to 1500 Da Full range
Label all possible matches ○ Label matches used for scoring ◉

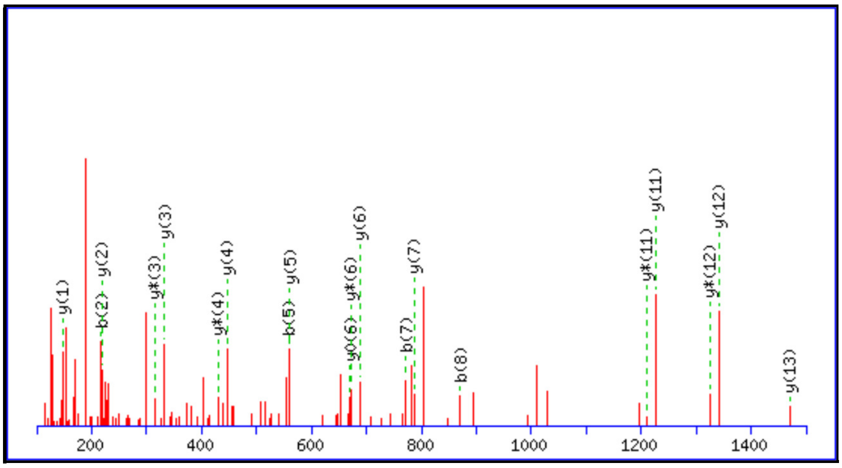

Monoisotopic mass of neutral peptide Mr(calc): 1556.8158
Fixed modifications: Carbamidomethyl (C) (apply to specified residues or termini only)
Variable modifications:
N3 : Deamidated (NQ)
Ions Score: 59 **Expect:** 0.00024
Matches : 20/146 fragment ions using 40 most intense peaks (help)

#	b	b++	b*	b*++	b0	b0++	Seq.	y	y++	y*	y*++	y0	y0++	#
1	88.0393	44.5233			70.0287	35.5180	S							14
2	**217.0819**	109.0446			199.0713	100.0393	E	***1470.7911***	735.8992	1453.7645	727.3859	1452.7805	726.8939	13
3	332.1088	166.5581	315.0823	158.0448	314.0983	157.5528	N	***1341.7485***	671.3779	1324.7219	662.8646	1323.7379	662.3726	12
4	403.1460	202.0766	386.1194	193.5633	385.1354	193.0713	A	***1226.7215***	613.8644	1209.6950	605.3511	1208.7110	604.8591	11
5	**559.2471**	280.1272	542.2205	271.6139	541.2365	271.1219	R	1155.6844	578.3459	1138.6579	569.8326	1137.6739	569.3406	10
6	672.3311	336.6692	655.3046	328.1559	654.3206	327.6639	L	999.5833	500.2953	982.5568	491.7820	981.5728	491.2900	9
7	**771.3995**	386.2034	754.3730	377.6901	753.3890	377.1981	V	886.4993	443.7533	869.4727	435.2400	868.4887	434.7480	8
8	**870.4680**	435.7376	853.4414	427.2243	852.4574	426.7323	V	***787.4308***	394.2191	770.4043	385.7058	769.4203	385.2138	7
9	998.5265	499.7669	981.5000	491.2536	980.5160	490.7616	Q	***688.3624***	344.6849	671.3359	336.1716	670.3519	335.6796	6
10	1111.6106	556.3089	1094.5840	547.7957	1093.6000	547.3037	I	***560.3039***	280.6556	543.2773	272.1423	542.2933	271.6503	5
11	1226.6375	613.8224	1209.6110	605.3091	1208.6270	604.8171	D	***447.2198***	224.1135	430.1932	215.6003	429.2092	215.1082	4
12	1340.6805	670.8439	1323.6539	662.3306	1322.6699	661.8386	N	***332.1928***	166.6001	315.1663	158.0868			3
13	1411.7176	706.3624	1394.6910	697.8492	1393.7070	697.3571	A	***218.1499***	109.5786	201.1234	101.0653			2
14							K	***147.1128***	74.0600	130.0863	65.5468			1

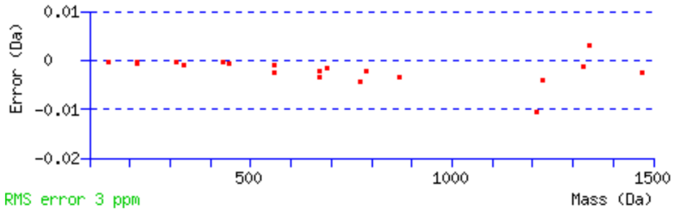

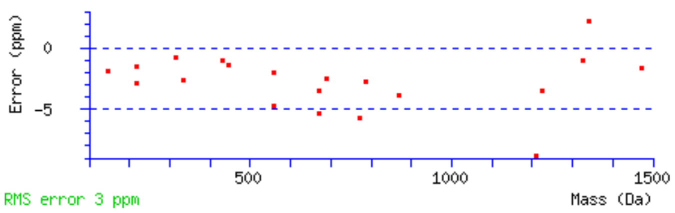

NCBI **BLAST** search of SENARLVVQIDNAK
(Parameters: blastp, nr protein database, expect=20000, no filter, PAM30)
Other BLAST web gateways

All matches to this query

Score	Mr(calc):	Delta	Sequence
59.2	1556.8158	0.0001	SENARLVVQIDNAK
24.1	1556.8158	0.0001	SENARLVVQIDNAK
5.9	1555.8205	0.9954	AENARLIVQIDNAK
2.7	1556.8158	0.0001	SENARLVVQIDNAK

Mascot: http://www.matrixscience.com/

Mascot Search Results

Peptide View

MS/MS Fragmentation of **SGGVCGPSPPCITTVSVNESLLTPLNLEIDPNAQCVKQEEKEQIK**
Found in **sp|O43790|KRT86_HUMAN**, Keratin, type II cuticular Hb6 OS=Homo sapiens GN=KRT86 PE=1 SV=1

Match to Query 45137: 4938.432336 from(1235.615360,4+) intensity(4445033.5000) scans(15232) rtinseconds(2745) index(27317)
Title: 160219_Sunil_SDSI_A_Spectrum080632_scans__15232_RTINSECONDS=2745
Data file C:\\Sunil\\TKAP\\T\\T160219_Sunil_SDSI_A.mgf

Click mouse within plot area to zoom in by factor of two about that point
Or, Plot from 200 to 4400 Da Full range
Label all possible matches ○ Label matches used for scoring ◉

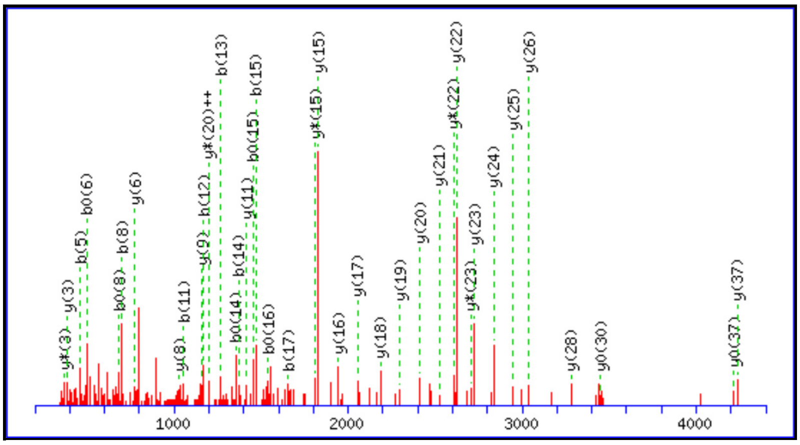

Monoisotopic mass of neutral peptide Mr(calc): 4936.4144
Fixed modifications: Carbamidomethyl (C) (apply to specified residues or termini only)
Variable modifications:
N18 : Deamidated (NQ)
Ions Score: 69 **Expect:** 2e-005
Matches : 39/488 fragment ions using 98 most intense peaks (help)

#	b	b++	b*	b*++	b0	b0++	Seq.	y	y++	y*	y*++	y0	y0++	#
1	88.0393	44.5233			70.0287	35.5180	S							45
2	145.0608	73.0340			127.0502	64.0287	G	4850.3897	2425.6985	4833.3631	2417.1852	4832.3791	2416.6932	44
3	202.0822	101.5448			184.0717	92.5395	G	4793.3682	2397.1877	4776.3417	2388.6745	4775.3576	2388.1825	43
4	301.1506	151.0790			283.1401	142.0737	V	4736.3467	2368.6770	4719.3202	2360.1637	4718.3362	2359.6717	42
5	**461.1813**	231.0943			443.1707	222.0890	C	4637.2783	2319.1428	4620.2518	2310.6295	4619.2678	2310.1375	41
6	518.2028	259.6050			500.1922	250.5997	G	4477.2477	2239.1275	4460.2211	2230.6142	4459.2371	2230.1222	40
7	615.2555	308.1314			597.2450	299.1261	P	4420.2262	2210.6167	4403.1997	2202.1035	4402.2156	2201.6115	39
8	**702.2876**	351.6474			684.2770	342.6421	S	4323.1734	2162.0904	4306.1469	2153.5771	4305.1629	2153.0851	38
9	799.3403	400.1738			781.3298	391.1685	P	***4236.1414***	2118.5743	4219.1149	2110.0611	4218.1309	2109.5691	37
10	896.3931	448.7002			878.3825	439.6949	P	4139.0887	2070.0480	4122.0621	2061.5347	4121.0781	2061.0427	36
11	**1056.4237**	528.7155			1038.4132	519.7102	C	4042.0359	2021.5216	4025.0093	2013.0083	4024.0253	2012.5163	35
12	**1169.5078**	585.2575			1151.4972	576.2523	I	3882.0052	1941.5063	3864.9787	1932.9930	3863.9947	1932.5010	34
13	**1270.5555**	635.7814			1252.5449	626.7761	T	3768.9212	1884.9642	3751.8946	1876.4510	3750.9106	1875.9589	33
14	**1371.6032**	686.3052			1353.5926	677.2999	T	3667.8735	1834.4404	3650.8470	1825.9271	3649.8629	1825.4351	32
15	**1470.6716**	735.8394			1452.6610	726.8341	V	3566.8258	1783.9165	3549.7993	1775.4033	3548.8153	1774.9113	31
16	1557.7036	779.3554			1539.6930	770.3502	S	3467.7574	1734.3823	3450.7309	1725.8691	3449.7468	1725.3771	30
17	**1656.7720**	828.8896			1638.7614	819.8844	V	3380.7254	1690.8663	3363.6988	1682.3531	3362.7148	1681.8610	29
18	1771.7989	886.4031	1754.7724	877.8898	1753.7884	877.3978	N	***3281.6570***	1641.3321	3264.6304	1632.8188	3263.6464	1632.3268	28
19	1900.8415	950.9244	1883.8150	942.4111	1882.8310	941.9191	E	3166.6300	1583.8186	3149.6035	1575.3054	3148.6195	1574.8134	27
20	1987.8736	994.4404	1970.8470	985.9271	1969.8630	985.4351	S	***3037.5874***	1519.2974	3020.5609	1510.7841	3019.5769	1510.2921	26
21	2100.9576	1050.9825	2083.9311	1042.4692	2082.9471	1041.9772	L	***2950.5554***	1475.7813	2933.5289	1467.2681	2932.5448	1466.7761	25
22	2214.0417	1107.5245	2197.0151	1099.0112	2196.0311	1098.5192	L	***2837.4713***	1419.2393	2820.4448	1410.7260	2819.4608	1410.2340	24
23	2315.0894	1158.0483	2298.0628	1149.5351	2297.0788	1149.0430	T	***2724.3873***	1362.6973	2707.3607	1354.1840	2706.3767	1353.6920	23
24	2412.1421	1206.5747	2395.1156	1198.0614	2394.1316	1197.5694	P	***2623.3396***	1312.1734	2606.3130	1303.6602	2605.3290	1303.1682	22
25	2525.2262	1263.1167	2508.1997	1254.6035	2507.2156	1254.1115	L	***2526.2868***	1263.6471	2509.2603	1255.1338	2508.2763	1254.6418	21
26	2639.2691	1320.1382	2622.2426	1311.6249	2621.2586	1311.1329	N	***2413.2028***	1207.1050	2396.1762	1198.5917	2395.1922	1198.0997	20
27	2752.3532	1376.6802	2735.3266	1368.1670	2734.3426	1367.6750	L	***2299.1598***	1150.0836	2282.1333	1141.5703	2281.1493	1141.0783	19

28	2881.3958	1441.2015	2864.3692	1432.6883	2863.3852	1432.1963	E	2186.0758	1093.5415	2169.0492	1085.0283	2168.0652	1084.5362	18
29	2994.4799	1497.7436	2977.4533	1489.2303	2976.4693	1488.7383	I	2057.0332	1029.0202	2040.0066	1020.5070	2039.0226	1020.0149	17
30	3109.5068	1555.2570	3092.4802	1546.7438	3091.4962	1546.2518	D	1943.9491	972.4782	1926.9226	963.9649	1925.9386	963.4729	16
31	3206.5596	1603.7834	3189.5330	1595.2701	3188.5490	1594.7781	P	1828.9222	914.9647	1811.8956	906.4515	1810.9116	905.9594	15
32	3320.6025	1660.8049	3303.5759	1652.2916	3302.5919	1651.7996	N	1731.8694	866.4383	1714.8429	857.9251	1713.8588	857.4331	14
33	3391.6396	1696.3234	3374.6131	1687.8102	3373.6290	1687.3182	A	1617.8265	809.4169	1600.7999	800.9036	1599.8159	800.4116	13
34	3519.6982	1760.3527	3502.6716	1751.8395	3501.6876	1751.3474	Q	1546.7894	773.8983	1529.7628	765.3850	1528.7788	764.8930	12
35	3679.7288	1840.3681	3662.7023	1831.8548	3661.7183	1831.3628	C	1418.7308	709.8690	1401.7042	701.3558	1400.7202	700.8638	11
36	3778.7972	1889.9023	3761.7707	1881.3890	3760.7867	1880.8970	V	1258.7001	629.8537	1241.6736	621.3404	1240.6896	620.8484	10
37	3906.8922	1953.9497	3889.8657	1945.4365	3888.8816	1944.9445	K	1159.6317	580.3195	1142.6052	571.8062	1141.6212	571.3142	9
38	4034.9508	2017.9790	4017.9242	2009.4658	4016.9402	2008.9737	Q	1031.5368	516.2720	1014.5102	507.7587	1013.5262	507.2667	8
39	4163.9934	2082.5003	4146.9668	2073.9871	4145.9828	2073.4950	E	903.4782	452.2427	886.4516	443.7295	885.4676	443.2374	7
40	4293.0360	2147.0216	4276.0094	2138.5083	4275.0254	2138.0163	E	774.4356	387.7214	757.4090	379.2082	756.4250	378.7162	6
41	4421.1309	2211.0691	4404.1044	2202.5558	4403.1204	2202.0638	K	645.3930	323.2001	628.3665	314.6869	627.3824	314.1949	5
42	4550.1735	2275.5904	4533.1470	2267.0771	4532.1630	2266.5851	E	517.2980	259.1527	500.2715	250.6394	499.2875	250.1474	4
43	4678.2321	2339.6197	4661.2056	2331.1064	4660.2215	2330.6144	Q	388.2554	194.6314	371.2289	186.1181			3
44	4791.3162	2396.1617	4774.2896	2387.6484	4773.3056	2387.1564	I	260.1969	130.6021	243.1703	122.0888			2
45							K	147.1128	74.0600	130.0863	65.5468			1

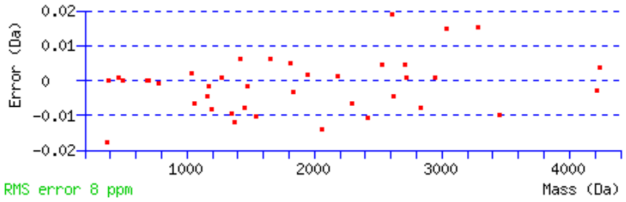

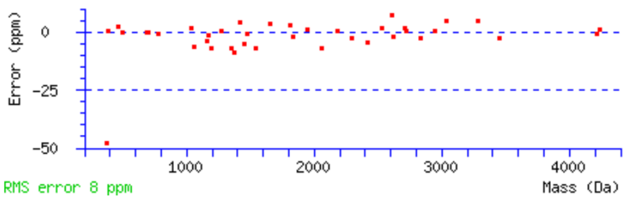

NCBI **BLAST** search of SGGVCGPSPPCITTVSVNESLLTPLNLEIDPNAQCVKQEEKEQIK
(Parameters: blastp, nr protein database, expect=20000, no filter, PAM30)
Other BLAST web gateways

All matches to this query

Score	Mr(calc):	Delta	Sequence
69.1	4936.4144	2.0179	SGGVCGPSPPCITTVSVNESLLTPLNLEIDPNAQCVKQEEKEQIK
47.9	4936.4144	2.0179	SGGVCGPSPPCITTVSVNESLLTPLNLEIDPNAQCVKQEEKEQIK
36.8	4937.3984	1.0339	SGGVCGPSPPCITTVSVNESLLTPLNLEIDPNAQCVKQEEKEQIK
23.5	4936.4144	2.0179	SGGVCGPSPPCITTVSVNESLLTPLNLEIDPNAQCVKQEEKEQIK
21.7	4936.4144	2.0179	SGGVCGPSPPCITTVSVNESLLTPLNLEIDPNAQCVKQEEKEQIK
14.0	4937.3984	1.0339	SGGVCGPSPPCITTVSVNESLLTPLNLEIDPNAQCVKQEEKEQIK
12.8	4937.3984	1.0339	SGGVCGPSPPCITTVSVNESLLTPLNLEIDPNAQCVKQEEKEQIK
12.3	4936.4144	2.0179	SGGVCGPSPPCITTVSVNESLLTPLNLEIDPNAQCVKQEEKEQIK
11.6	4936.4144	2.0179	SGGVCGPSPPCITTVSVNESLLTPLNLEIDPNAQCVKQEEKEQIK
8.0	4937.3984	1.0339	SGGVCGPSPPCITTVSVNESLLTPLNLEIDPNAQCVKQEEKEQIK

Mascot: http://www.matrixscience.com/

Mascot Search Results

Peptide View

MS/MS Fragmentation of **SGGVCGPSPPCITTVSVNESLLTPLNLEIDPNAQCVKQEEKEQIK**
Found in **sp|P78385|KRT83_HUMAN**, Keratin, type II cuticular Hb3 OS=Homo sapiens GN=KRT83 PE=1 SV=2

Match to Query 45137: 4938.432336 from(1235.615360,4+) intensity(4445033.5000) scans(15232) rtinseconds(2745) index(27317)
Title: 160219_Sunil_SDSI_A_Spectrum080632_scans__15232_RTINSECONDS=2745
Data file C:\\Sunil\\TKAP\\T\\T160219_Sunil_SDSI_A.mgf

Click mouse within plot area to zoom in by factor of two about that point
Or, Plot from 200 to 4400 Da Full range
Label all possible matches ○ Label matches used for scoring ●

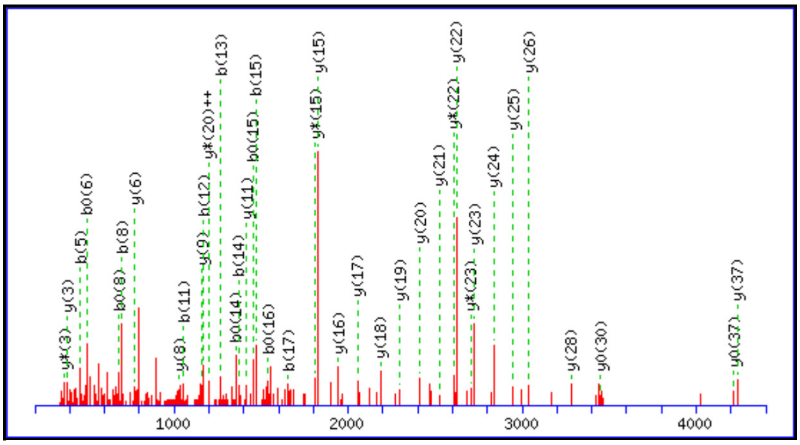

Monoisotopic mass of neutral peptide Mr(calc): 4936.4144
Fixed modifications: Carbamidomethyl (C) (apply to specified residues or termini only)
Variable modifications:
N18 : Deamidated (NQ)
Ions Score: 69 **Expect:** 2e-005
Matches : 39/488 fragment ions using 98 most intense peaks (help)

#	b	b^{++}	b*	b^{*++}	b^0	b^{0++}	Seq.	y	y^{++}	y*	y^{*++}	y^0	y^{0++}	#
1	88.0393	44.5233			70.0287	35.5180	S							45
2	145.0608	73.0340			127.0502	64.0287	G	4850.3897	2425.6985	4833.3631	2417.1852	4832.3791	2416.6932	44
3	202.0822	101.5448			184.0717	92.5395	G	4793.3682	2397.1877	4776.3417	2388.6745	4775.3576	2388.1825	43
4	301.1506	151.0790			283.1401	142.0737	V	4736.3467	2368.6770	4719.3202	2360.1637	4718.3362	2359.6717	42
5	**461.1813**	231.0943			443.1707	222.0890	C	4637.2783	2319.1428	4620.2518	2310.6295	4619.2678	2310.1375	41
6	518.2028	259.6050			500.1922	250.5997	G	4477.2477	2239.1275	4460.2211	2230.6142	4459.2371	2230.1222	40
7	615.2555	308.1314			597.2450	299.1261	P	4420.2262	2210.6167	4403.1997	2202.1035	4402.2156	2201.6115	39
8	**702.2876**	351.6474			684.2770	342.6421	S	4323.1734	2162.0904	4306.1469	2153.5771	4305.1629	2153.0851	38
9	799.3403	400.1738			781.3298	391.1685	P	***4236.1414***	2118.5743	4219.1149	2110.0611	4218.1309	2109.5691	37
10	896.3931	448.7002			878.3825	439.6949	P	4139.0887	2070.0480	4122.0621	2061.5347	4121.0781	2061.0427	36
11	**1056.4237**	528.7155			1038.4132	519.7102	C	4042.0359	2021.5216	4025.0093	2013.0083	4024.0253	2012.5163	35
12	**1169.5078**	585.2575			1151.4972	576.2523	I	3882.0052	1941.5063	3864.9787	1932.9930	3863.9947	1932.5010	34
13	**1270.5555**	635.7814			1252.5449	626.7761	T	3768.9212	1884.9642	3751.8946	1876.4510	3750.9106	1875.9589	33
14	**1371.6032**	686.3052			1353.5926	677.2999	T	3667.8735	1834.4404	3650.8470	1825.9271	3649.8629	1825.4351	32
15	**1470.6716**	735.8394			1452.6610	726.8341	V	3566.8258	1783.9165	3549.7993	1775.4033	3548.8153	1774.9113	31
16	1557.7036	779.3554			1539.6930	770.3502	S	3467.7574	1734.3823	3450.7309	1725.8691	3449.7468	1725.3771	30
17	**1656.7720**	828.8896			1638.7614	819.8844	V	3380.7254	1690.8663	3363.6988	1682.3531	3362.7148	1681.8610	29
18	1771.7989	886.4031	1754.7724	877.8898	1753.7884	877.3978	N	***3281.6570***	1641.3321	3264.6304	1632.8188	3263.6464	1632.3268	28
19	1900.8415	950.9244	1883.8150	942.4111	1882.8310	941.9191	E	3166.6300	1583.8186	3149.6035	1575.3054	3148.6195	1574.8134	27
20	1987.8736	994.4404	1970.8470	985.9271	1969.8630	985.4351	S	***3037.5874***	1519.2974	3020.5609	1510.7841	3019.5769	1510.2921	26
21	2100.9576	1050.9825	2083.9311	1042.4692	2082.9471	1041.9772	L	***2950.5554***	1475.7813	2933.5289	1467.2681	2932.5448	1466.7761	25
22	2214.0417	1107.5245	2197.0151	1099.0112	2196.0311	1098.5192	L	***2837.4713***	1419.2393	2820.4448	1410.7260	2819.4608	1410.2340	24
23	2315.0894	1158.0483	2298.0628	1149.5351	2297.0788	1149.0430	T	***2724.3873***	1362.6973	2707.3607	1354.1840	2706.3767	1353.6920	23
24	2412.1421	1206.5747	2395.1156	1198.0614	2394.1316	1197.5694	P	***2623.3396***	1312.1734	2606.3130	1303.6602	2605.3290	1303.1682	22
25	2525.2262	1263.1167	2508.1997	1254.6035	2507.2156	1254.1115	L	***2526.2868***	1263.6471	2509.2603	1255.1338	2508.2763	1254.6418	21
26	2639.2691	1320.1382	2622.2426	1311.6249	2621.2586	1311.1329	N	***2413.2028***	1207.1050	2396.1762	1198.5917	2395.1922	1198.0997	20
27	2752.3532	1376.6802	2735.3266	1368.1670	2734.3426	1367.6750	L	***2299.1598***	1150.0836	2282.1333	1141.5703	2281.1493	1141.0783	19

**28**	2881.3958	1441.2015	2864.3692	1432.6883	2863.3852	1432.1963	**E**	***2186.0758***	1093.5415	2169.0492	1085.0283	2168.0652	1084.5362	**18**
**29**	2994.4799	1497.7436	2977.4533	1489.2303	2976.4693	1488.7383	**I**	***2057.0332***	1029.0202	2040.0066	1020.5070	2039.0226	1020.0149	**17**
**30**	3109.5068	1555.2570	3092.4802	1546.7438	3091.4962	1546.2518	**D**	***1943.9491***	972.4782	1926.9226	963.9649	1925.9386	963.4729	**16**
**31**	3206.5596	1603.7834	3189.5330	1595.2701	3188.5490	1594.7781	**P**	***1828.9222***	914.9647	1811.8956	906.4515	1810.9116	905.9594	**15**
**32**	3320.6025	1660.8049	3303.5759	1652.2916	3302.5919	1651.7996	**N**	1731.8694	866.4383	1714.8429	857.9251	1713.8588	857.4331	**14**
**33**	3391.6396	1696.3234	3374.6131	1687.8102	3373.6290	1687.3182	**A**	1617.8265	809.4169	1600.7999	800.9036	1599.8159	800.4116	**13**
**34**	3519.6982	1760.3527	3502.6716	1751.8395	3501.6876	1751.3474	**Q**	1546.7894	773.8983	1529.7628	765.3850	1528.7788	764.8930	**12**
**35**	3679.7288	1840.3681	3662.7023	1831.8548	3661.7183	1831.3628	**C**	***1418.7308***	709.8690	1401.7042	701.3558	1400.7202	700.8638	**11**
**36**	3778.7972	1889.9023	3761.7707	1881.3890	3760.7867	1880.8970	**V**	1258.7001	629.8537	1241.6736	621.3404	1240.6896	620.8484	**10**
**37**	3906.8922	1953.9497	3889.8657	1945.4365	3888.8816	1944.9445	**K**	***1159.6317***	580.3195	1142.6052	571.8062	1141.6212	571.3142	**9**
**38**	4034.9508	2017.9790	4017.9242	2009.4658	4016.9402	2008.9737	**Q**	***1031.5368***	516.2720	1014.5102	507.7587	1013.5262	507.2667	**8**
**39**	4163.9934	2082.5003	4146.9668	2073.9871	4145.9828	2073.4950	**E**	903.4782	452.2427	886.4516	443.7295	885.4676	443.2374	**7**
**40**	4293.0360	2147.0216	4276.0094	2138.5083	4275.0254	2138.0163	**E**	***774.4356***	387.7214	757.4090	379.2082	756.4250	378.7162	**6**
**41**	4421.1309	2211.0691	4404.1044	2202.5558	4403.1204	2202.0638	**K**	645.3930	323.2001	628.3665	314.6869	627.3824	314.1949	**5**
**42**	4550.1735	2275.5904	4533.1470	2267.0771	4532.1630	2266.5851	**E**	517.2980	259.1527	500.2715	250.6394	499.2875	250.1474	**4**
**43**	4678.2321	2339.6197	4661.2056	2331.1064	4660.2215	2330.6144	**Q**	***388.2554***	194.6314	371.2289	186.1181			**3**
**44**	4791.3162	2396.1617	4774.2896	2387.6484	4773.3056	2387.1564	**I**	260.1969	130.6021	243.1703	122.0888			**2**
**45**							**K**	147.1128	74.0600	130.0863	65.5468			**1**

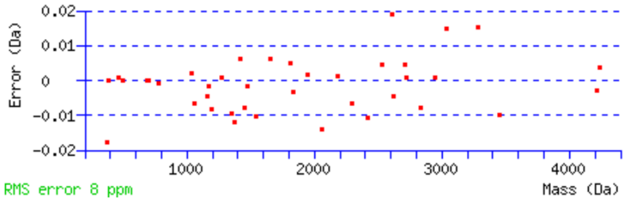

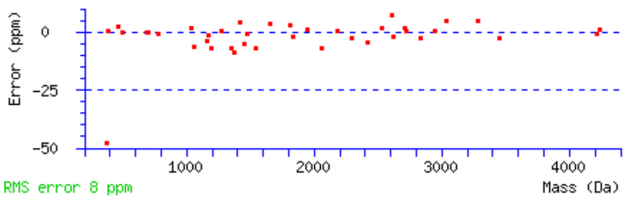

NCBI **BLAST** search of SGGVCGPSPPCITTVSVNESLLTPLNLEIDPNAQCVKQEEKEQIK
(Parameters: blastp, nr protein database, expect=20000, no filter, PAM30)
Other BLAST web gateways

All matches to this query

Score	Mr(calc):	Delta	Sequence
69.1	4936.4144	2.0179	SGGVCGPSPPCITTVSVNESLLTPLNLEIDPNAQCVKQEEKEQIK
47.9	4936.4144	2.0179	SGGVCGPSPPCITTVSVNESLLTPLNLEIDPNAQCVKQEEKEQIK
36.8	4937.3984	1.0339	SGGVCGPSPPCITTVSVNESLLTPLNLEIDPNAQCVKQEEKEQIK
23.5	4936.4144	2.0179	SGGVCGPSPPCITTVSVNESLLTPLNLEIDPNAQCVKQEEKEQIK
21.7	4936.4144	2.0179	SGGVCGPSPPCITTVSVNESLLTPLNLEIDPNAQCVKQEEKEQIK
14.0	4937.3984	1.0339	SGGVCGPSPPCITTVSVNESLLTPLNLEIDPNAQCVKQEEKEQIK
12.8	4937.3984	1.0339	SGGVCGPSPPCITTVSVNESLLTPLNLEIDPNAQCVKQEEKEQIK
12.3	4936.4144	2.0179	SGGVCGPSPPCITTVSVNESLLTPLNLEIDPNAQCVKQEEKEQIK
11.6	4936.4144	2.0179	SGGVCGPSPPCITTVSVNESLLTPLNLEIDPNAQCVKQEEKEQIK
8.0	4937.3984	1.0339	SGGVCGPSPPCITTVSVNESLLTPLNLEIDPNAQCVKQEEKEQIK

Mascot: http://www.matrixscience.com/

Mascot Search Results

Peptide View

MS/MS Fragmentation of **SGGVCGPSPPCITTVSVNESLLTPLNLEIDPNAQCVKQEEKEQIK**
Found in **sp|O43790|KRT86_HUMAN**, Keratin, type II cuticular Hb6 OS=Homo sapiens GN=KRT86 PE=1 SV=1

Match to Query 45138: 4938.434776 from(1235.615970,4+) intensity(4329913.0000) scans(15500) rtinseconds(2816) index(12231)
Title: 160219_Sunil_SDSI_A_Spectrum064363_scans__15500_RTINSECONDS=2816
Data file C:\\Sunil\\TKAP\\T\\T160219_Sunil_SDSI_A.mgf

Click mouse within plot area to zoom in by factor of two about that point
Or, Plot from 200 to 4400 Da Full range
Label all possible matches Label matches used for scoring

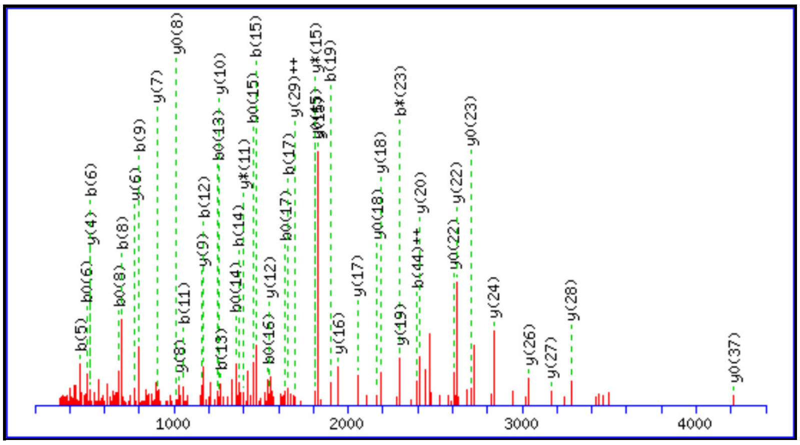

Monoisotopic mass of neutral peptide Mr(calc): 4936.4144
Fixed modifications: Carbamidomethyl (C) (apply to specified residues or termini only)
Variable modifications:
N26 : Deamidated (NQ)
Ions Score: 74 **Expect:** 6e-006
Matches : 47/488 fragment ions using 114 most intense peaks (help)

#	b	b++	b*	b*++	b0	b0++	Seq.	y	y++	y*	y*++	y0	y0++	#
1	88.0393	44.5233			70.0287	35.5180	S							45
2	145.0608	73.0340			127.0502	64.0287	G	4850.3897	2425.6985	4833.3631	2417.1852	4832.3791	2416.6932	44
3	202.0822	101.5448			184.0717	92.5395	G	4793.3682	2397.1877	4776.3417	2388.6745	4775.3576	2388.1825	43
4	301.1506	151.0790			283.1401	142.0737	V	4736.3467	2368.6770	4719.3202	2360.1637	4718.3362	2359.6717	42
5	***461.1813***	231.0943			443.1707	222.0890	C	4637.2783	2319.1428	4620.2518	2310.6295	4619.2678	2310.1375	41
6	***518.2028***	259.6050			500.1922	250.5997	G	4477.2477	2239.1275	4460.2211	2230.6142	4459.2371	2230.1222	40
7	615.2555	308.1314			597.2450	299.1261	P	4420.2262	2210.6167	4403.1997	2202.1035	4402.2156	2201.6115	39
8	***702.2876***	351.6474			684.2770	342.6421	S	4323.1734	2162.0904	4306.1469	2153.5771	4305.1629	2153.0851	38
9	***799.3403***	400.1738			781.3298	391.1685	P	4236.1414	2118.5743	4219.1149	2110.0611	4218.1309	2109.5691	37
10	896.3931	448.7002			878.3825	439.6949	P	4139.0887	2070.0480	4122.0621	2061.5347	4121.0781	2061.0427	36
11	***1056.4237***	528.7155			1038.4132	519.7102	C	4042.0359	2021.5216	4025.0093	2013.0083	4024.0253	2012.5163	35
12	***1169.5078***	585.2575			1151.4972	576.2523	I	3882.0052	1941.5063	3864.9787	1932.9930	3863.9947	1932.5010	34
13	***1270.5555***	635.7814			1252.5449	626.7761	T	3768.9212	1884.9642	3751.8946	1876.4510	3750.9106	1875.9589	33
14	***1371.6032***	686.3052			1353.5926	677.2999	T	3667.8735	1834.4404	3650.8470	1825.9271	3649.8629	1825.4351	32
15	***1470.6716***	735.8394			1452.6610	726.8341	V	3566.8258	1783.9165	3549.7993	1775.4033	3548.8153	1774.9113	31
16	1557.7036	779.3554			1539.6930	770.3502	S	3467.7574	1734.3823	3450.7309	1725.8691	3449.7468	1725.3771	30
17	***1656.7720***	828.8896			1638.7614	819.8844	V	3380.7254	1690.8663	3363.6988	1682.3531	3362.7148	1681.8610	29
18	1770.8149	885.9111	1753.7884	877.3978	1752.8044	876.9058	N	***3281.6570***	1641.3321	3264.6304	1632.8188	3263.6464	1632.3268	28
19	***1899.8575***	950.4324	1882.8310	941.9191	1881.8470	941.4271	E	***3167.6140***	1584.3107	3150.5875	1575.7974	3149.6035	1575.3054	27
20	1986.8896	993.9484	1969.8630	985.4351	1968.8790	984.9431	S	***3038.5714***	1519.7894	3021.5449	1511.2761	3020.5609	1510.7841	26
21	2099.9736	1050.4904	2082.9471	1041.9772	2081.9631	1041.4852	L	2951.5394	1476.2733	2934.5129	1467.7601	2933.5289	1467.2681	25
22	2213.0577	1107.0325	2196.0311	1098.5192	2195.0471	1098.0272	L	***2838.4554***	1419.7313	2821.4288	1411.2180	2820.4448	1410.7260	24
23	2314.1054	1157.5563	2297.0788	1149.0430	2296.0948	1148.5510	T	2725.3713	1363.1893	2708.3447	1354.6760	2707.3607	1354.1840	23
24	2411.1581	1206.0827	2394.1316	1197.5694	2393.1476	1197.0774	P	***2624.3236***	1312.6654	2607.2971	1304.1522	2606.3130	1303.6602	22
25	2524.2422	1262.6247	2507.2156	1254.1115	2506.2316	1253.6195	L	2527.2708	1264.1391	2510.2443	1255.6258	2509.2603	1255.1338	21
26	2639.2691	1320.1382	2622.2426	1311.6249	2621.2586	1311.1329	N	***2414.1868***	1207.5970	2397.1602	1199.0838	2396.1762	1198.5917	20
27	2752.3532	1376.6802	2735.3266	1368.1670	2734.3426	1367.6750	L	***2299.1598***	1150.0836	2282.1333	1141.5703	2281.1493	1141.0783	19

**28**	2881.3958	1441.2015	2864.3692	1432.6883	2863.3852	1432.1963	**E**	***2186.0758***	1093.5415	2169.0492	1085.0283	2168.0652	1084.5362	**18**
**29**	2994.4799	1497.7436	2977.4533	1489.2303	2976.4693	1488.7383	**I**	***2057.0332***	1029.0202	2040.0066	1020.5070	2039.0226	1020.0149	**17**
**30**	3109.5068	1555.2570	3092.4802	1546.7438	3091.4962	1546.2518	**D**	***1943.9491***	972.4782	1926.9226	963.9649	1925.9386	963.4729	**16**
**31**	3206.5596	1603.7834	3189.5330	1595.2701	3188.5490	1594.7781	**P**	***1828.9222***	914.9647	1811.8956	906.4515	1810.9116	905.9594	**15**
**32**	3320.6025	1660.8049	3303.5759	1652.2916	3302.5919	1651.7996	**N**	1731.8694	866.4383	1714.8429	857.9251	1713.8588	857.4331	**14**
**33**	3391.6396	1696.3234	3374.6131	1687.8102	3373.6290	1687.3182	**A**	1617.8265	809.4169	1600.7999	800.9036	1599.8159	800.4116	**13**
**34**	3519.6982	1760.3527	3502.6716	1751.8395	3501.6876	1751.3474	**Q**	***1546.7894***	773.8983	1529.7628	765.3850	1528.7788	764.8930	**12**
**35**	3679.7288	1840.3681	3662.7023	1831.8548	3661.7183	1831.3628	**C**	1418.7308	709.8690	1401.7042	701.3558	1400.7202	700.8638	**11**
**36**	3778.7972	1889.9023	3761.7707	1881.3890	3760.7867	1880.8970	**V**	***1258.7001***	629.8537	1241.6736	621.3404	1240.6896	620.8484	**10**
**37**	3906.8922	1953.9497	3889.8657	1945.4365	3888.8816	1944.9445	**K**	***1159.6317***	580.3195	1142.6052	571.8062	1141.6212	571.3142	**9**
**38**	4034.9508	2017.9790	4017.9242	2009.4658	4016.9402	2008.9737	**Q**	***1031.5368***	516.2720	1014.5102	507.7587	1013.5262	507.2667	**8**
**39**	4163.9934	2082.5003	4146.9668	2073.9871	4145.9828	2073.4950	**E**	***903.4782***	452.2427	886.4516	443.7295	885.4676	443.2374	**7**
**40**	4293.0360	2147.0216	4276.0094	2138.5083	4275.0254	2138.0163	**E**	***774.4356***	387.7214	757.4090	379.2082	756.4250	378.7162	**6**
**41**	4421.1309	2211.0691	4404.1044	2202.5558	4403.1204	2202.0638	**K**	645.3930	323.2001	628.3665	314.6869	627.3824	314.1949	**5**
**42**	4550.1735	2275.5904	4533.1470	2267.0771	4532.1630	2266.5851	**E**	***517.2980***	259.1527	500.2715	250.6394	499.2875	250.1474	**4**
**43**	4678.2321	2339.6197	4661.2056	2331.1064	4660.2215	2330.6144	**Q**	388.2554	194.6314	371.2289	186.1181			**3**
**44**	4791.3162	**2396.1617**	4774.2896	2387.6484	4773.3056	2387.1564	**I**	260.1969	130.6021	243.1703	122.0888			**2**
**45**							**K**	147.1128	74.0600	130.0863	65.5468			**1**

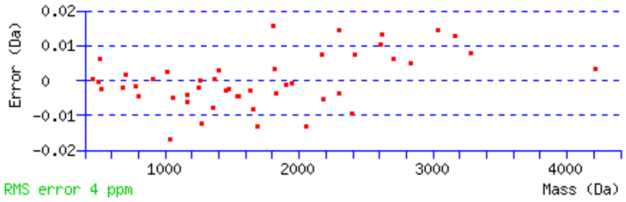

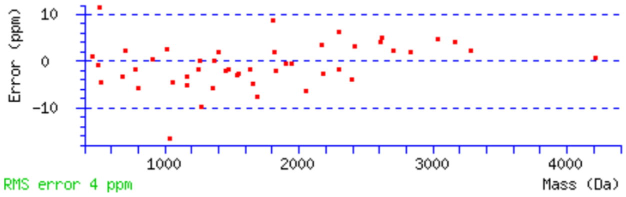

NCBI **BLAST** search of SGGVCGPSPPCITTVSVNESLLTPLNLEIDPNAQCVKQEEKEQIK
(Parameters: blastp, nr protein database, expect=20000, no filter, PAM30)
Other BLAST web gateways

All matches to this query

Score	Mr(calc):	Delta	Sequence
74.3	4936.4144	2.0204	SGGVCGPSPPCITTVSVNESLLTPLNLEIDPNAQCVKQEEKEQIK
68.6	4936.4144	2.0204	SGGVCGPSPPCITTVSVNESLLTPLNLEIDPNAQCVKQEEKEQIK
65.9	4937.3984	1.0364	SGGVCGPSPPCITTVSVNESLLTPLNLEIDPNAQCVKQEEKEQIK
41.0	4936.4144	2.0204	SGGVCGPSPPCITTVSVNESLLTPLNLEIDPNAQCVKQEEKEQIK
36.0	4936.4144	2.0204	SGGVCGPSPPCITTVSVNESLLTPLNLEIDPNAQCVKQEEKEQIK
33.0	4937.3984	1.0364	SGGVCGPSPPCITTVSVNESLLTPLNLEIDPNAQCVKQEEKEQIK
28.4	4937.3984	1.0364	SGGVCGPSPPCITTVSVNESLLTPLNLEIDPNAQCVKQEEKEQIK
22.3	4936.4144	2.0204	SGGVCGPSPPCITTVSVNESLLTPLNLEIDPNAQCVKQEEKEQIK
19.9	4936.4144	2.0204	SGGVCGPSPPCITTVSVNESLLTPLNLEIDPNAQCVKQEEKEQIK
16.1	4937.3984	1.0364	SGGVCGPSPPCITTVSVNESLLTPLNLEIDPNAQCVKQEEKEQIK

Mascot: http://www.matrixscience.com/

Mascot Search Results

Peptide View

MS/MS Fragmentation of **SLLESEDCKLPSNPCATTNACDK**
Found in **sp|O76009|KT33A_HUMAN**, Keratin, type I cuticular Ha3-I OS=Homo sapiens GN=KRT33A PE=2 SV=2

Match to Query 37847: 2610.126852 from(871.049560,3+) intensity(894716.2500) scans(9916) rtinseconds(1834) index(22995)
Title: 160219_Sunil_SDSI_A_Spectrum076299_scans__9916_RTINSECONDS=1834
Data file C:\\Sunil\\TKAP\\T\\T160219_Sunil_SDSI_A.mgf

Click mouse within plot area to zoom in by factor of two about that point
Or, Plot from 0 to 2400 Da Full range
Label all possible matches ○ Label matches used for scoring ●

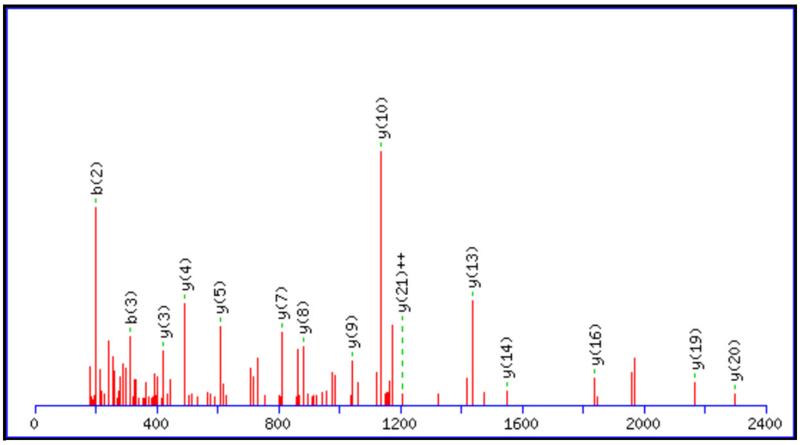

Monoisotopic mass of neutral peptide Mr(calc): 2610.1247
Fixed modifications: Carbamidomethyl (C) (apply to specified residues or termini only)
Variable modifications:
N19 : Deamidated (NQ)
Ions Score: 104 **Expect:** 3.2e-009
Matches : 15/246 fragment ions using 18 most intense peaks (help)

#	b	b^{++}	b*	b^{*++}	b^{0}	b^{0++}	Seq.	y	y^{++}	y*	y^{*++}	y^{0}	y^{0++}	#
1	88.0393	44.5233			70.0287	35.5180	S							23
2	**201.1234**	101.0653			183.1128	92.0600	L	2524.1000	1262.5536	2507.0735	1254.0404	2506.0895	1253.5484	22
3	**314.2074**	157.6074			296.1969	148.6021	L	2411.0160	1206.0116	2393.9894	1197.4983	2393.0054	1197.0063	21
4	443.2500	222.1287			425.2395	213.1234	E	***2297.9319***	1149.4696	2280.9053	1140.9563	2279.9213	1140.4643	20
5	530.2821	265.6447			512.2715	256.6394	S	***2168.8893***	1084.9483	2151.8627	1076.4350	2150.8787	1075.9430	19
6	659.3246	330.1660			641.3141	321.1607	E	2081.8573	1041.4323	2064.8307	1032.9190	2063.8467	1032.4270	18
7	774.3516	387.6794			756.3410	378.6742	D	1952.8147	976.9110	1935.7881	968.3977	1934.8041	967.9057	17
8	934.3822	467.6948			916.3717	458.6895	C	***1837.7877***	919.3975	1820.7612	910.8842	1819.7772	910.3922	16
9	1062.4772	531.7422	1045.4507	523.2290	1044.4666	522.7370	K	1677.7571	839.3822	1660.7305	830.8689	1659.7465	830.3769	15
10	1175.5613	588.2843	1158.5347	579.7710	1157.5507	579.2790	L	***1549.6621***	775.3347	1532.6356	766.8214	1531.6516	766.3294	14
11	1272.6140	636.8107	1255.5875	628.2974	1254.6035	627.8054	P	***1436.5781***	718.7927	1419.5515	710.2794	1418.5675	709.7874	13
12	1359.6461	680.3267	1342.6195	671.8134	1341.6355	671.3214	S	1339.5253	670.2663	1322.4987	661.7530	1321.5147	661.2610	12
13	1473.6890	737.3481	1456.6624	728.8349	1455.6784	728.3428	N	1252.4933	626.7503	1235.4667	618.2370	1234.4827	617.7450	11
14	1570.7417	785.8745	1553.7152	777.3612	1552.7312	776.8692	P	***1138.4503***	569.7288	1121.4238	561.2155	1120.4398	560.7235	10
15	1730.7724	865.8898	1713.7458	857.3766	1712.7618	856.8846	C	***1041.3976***	521.2024	1024.3710	512.6892	1023.3870	512.1971	9
16	1801.8095	901.4084	1784.7830	892.8951	1783.7989	892.4031	A	***881.3669***	441.1871	864.3404	432.6738	863.3564	432.1818	8
17	1902.8572	951.9322	1885.8306	943.4190	1884.8466	942.9270	T	***810.3298***	405.6685	793.3033	397.1553	792.3192	396.6633	7
18	2003.9049	1002.4561	1986.8783	993.9428	1985.8943	993.4508	T	709.2821	355.1447	692.2556	346.6314	691.2716	346.1394	6
19	2118.9318	1059.9695	2101.9053	1051.4563	2100.9212	1050.9643	N	***608.2345***	304.6209	591.2079	296.1076	590.2239	295.6156	5
20	2189.9689	1095.4881	2172.9424	1086.9748	2171.9584	1086.4828	A	***493.2075***	247.1074	476.1810	238.5941	475.1969	238.1021	4
21	2349.9996	1175.5034	2332.9730	1166.9902	2331.9890	1166.4981	C	***422.1704***	211.5888	405.1438	203.0756	404.1598	202.5836	3
22	2465.0265	1233.0169	2448.0000	1224.5036	2447.0160	1224.0116	D	262.1397	131.5735	245.1132	123.0602	244.1292	122.5682	2
23							K	147.1128	74.0600	130.0863	65.5468			1

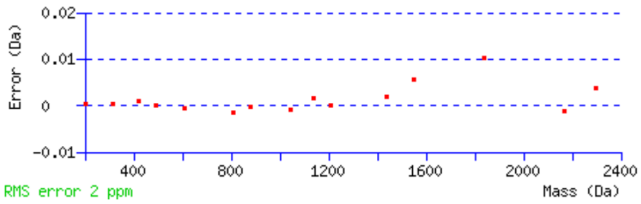

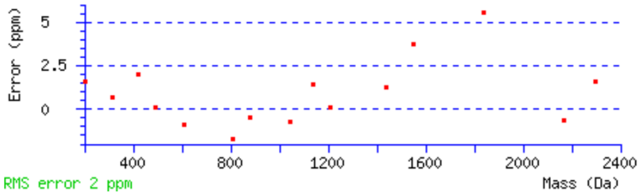

NCBI **BLAST** search of SLLESEDCKLPSNPCATTNACDK
(Parameters: blastp, nr protein database, expect=20000, no filter, PAM30)
Other BLAST web gateways

All matches to this query

Score	Mr(calc):	Delta	Sequence
104.1	2610.1247	0.0021	SLLESEDCKLPSNPCATTNACDK
37.1	2610.1247	0.0021	SLLESEDCKLPSNPCATTNACDK
17.6	2609.1407	0.9861	SLLESEDCKLPSNPCATTNACDK

Mascot: http://www.matrixscience.com/

Mascot Search Results

Peptide View

MS/MS Fragmentation of **SLLESEDCKLPSNPCATTNACDKSTGPCISNPCGLR**
Found in **sp|O76009|KT33A_HUMAN**, Keratin, type I cuticular Ha3-I OS=Homo sapiens GN=KRT33A PE=2 SV=2

Match to Query 43764: 4011.740176 from(1003.942320,4+) intensity(3286178.2500) scans(11710) rtinseconds(2141) index(24563)
Title: 160219_Sunil_SDSI_A_Spectrum077868_scans__11710_RTINSECONDS=2141
Data file C:\\Sunil\\TKAP\\T\\T160219_Sunil_SDSI_A.mgf

Click mouse within plot area to zoom in by factor of two about that point
Or, Plot from 200 to 3800 Da Full range
Label all possible matches ○ Label matches used for scoring ◉

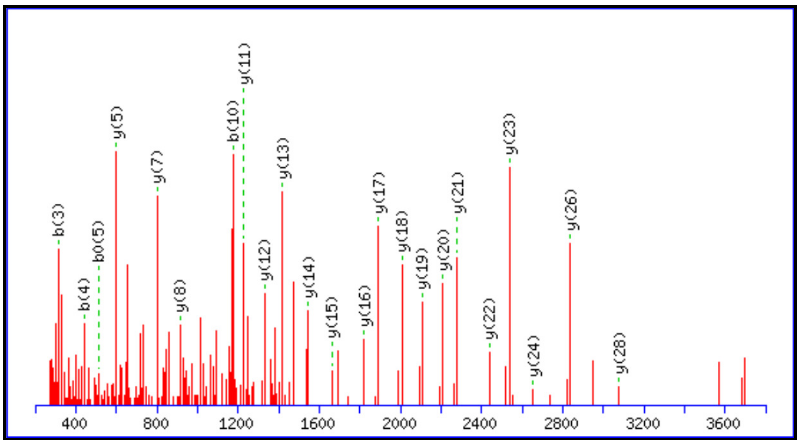

Monoisotopic mass of neutral peptide Mr(calc): 4010.7424
Fixed modifications: Carbamidomethyl (C) (apply to specified residues or termini only)
Variable modifications:
N13 : Deamidated (NQ)
N19 : Deamidated (NQ)
Ions Score: 156 **Expect:** 1.8e-014
Matches : 23/392 fragment ions using 30 most intense peaks (help)

#	b	b++	b*	b*++	b0	b0++	Seq.	y	y++	y*	y*++	y0	y0++	#
1	88.0393	44.5233			70.0287	35.5180	S							36
2	201.1234	101.0653			183.1128	92.0600	L	3924.7177	1962.8625	3907.6911	1954.3492	3906.7071	1953.8572	35
3	**314.2074**	157.6074			296.1969	148.6021	L	3811.6336	1906.3205	3794.6071	1897.8072	3793.6231	1897.3152	34
4	**443.2500**	222.1287			425.2395	213.1234	E	3698.5496	1849.7784	3681.5230	1841.2651	3680.5390	1840.7731	33
5	530.2821	265.6447			512.2715	256.6394	S	3569.5070	1785.2571	3552.4804	1776.7438	3551.4964	1776.2518	32
6	659.3246	330.1660			641.3141	321.1607	E	3482.4749	1741.7411	3465.4484	1733.2278	3464.4644	1732.7358	31
7	774.3516	387.6794			756.3410	378.6742	D	3353.4323	1677.2198	3336.4058	1668.7065	3335.4218	1668.2145	30
8	934.3822	467.6948			916.3717	458.6895	C	3238.4054	1619.7063	3221.3789	1611.1931	3220.3948	1610.7011	29
9	1062.4772	531.7422	1045.4507	523.2290	1044.4666	522.7370	K	***3078.3748***	1539.6910	3061.3482	1531.1777	3060.3642	1530.6857	28
10	**1175.5613**	588.2843	1158.5347	579.7710	1157.5507	579.2790	L	2950.2798	1475.6435	2933.2532	1467.1303	2932.2692	1466.6383	27
11	1272.6140	636.8107	1255.5875	628.2974	1254.6035	627.8054	P	***2837.1957***	1419.1015	2820.1692	1410.5882	2819.1852	1410.0962	26
12	1359.6461	680.3267	1342.6195	671.8134	1341.6355	671.3214	S	2740.1430	1370.5751	2723.1164	1362.0618	2722.1324	1361.5698	25
13	1474.6730	737.8401	1457.6465	729.3269	1456.6624	728.8349	N	***2653.1109***	1327.0591	2636.0844	1318.5458	2635.1004	1318.0538	24
14	1571.7258	786.3665	1554.6992	777.8532	1553.7152	777.3612	P	***2538.0840***	1269.5456	2521.0574	1261.0324	2520.0734	1260.5404	23
15	1731.7564	866.3818	1714.7299	857.8686	1713.7458	857.3766	C	***2441.0312***	1221.0193	2424.0047	1212.5060	2423.0207	1212.0140	22
16	1802.7935	901.9004	1785.7670	893.3871	1784.7830	892.8951	A	***2281.0006***	1141.0039	2263.9740	1132.4907	2262.9900	1131.9986	21
17	1903.8412	952.4242	1886.8147	943.9110	1885.8306	943.4190	T	***2209.9635***	1105.4854	2192.9369	1096.9721	2191.9529	1096.4801	20
18	2004.8889	1002.9481	1987.8623	994.4348	1986.8783	993.9428	T	***2108.9158***	1054.9615	2091.8892	1046.4483	2090.9052	1045.9562	19
19	2119.9158	1060.4616	2102.8893	1051.9483	2101.9053	1051.4563	N	***2007.8681***	1004.4377	1990.8416	995.9244	1989.8575	995.4324	18
20	2190.9529	1095.9801	2173.9264	1087.4668	2172.9424	1086.9748	A	***1892.8412***	946.9242	1875.8146	938.4109	1874.8306	937.9189	17
21	2350.9836	1175.9954	2333.9570	1167.4822	2332.9730	1166.9902	C	***1821.8041***	911.4057	1804.7775	902.8924	1803.7935	902.4004	16
22	2466.0105	1233.5089	2448.9840	1224.9956	2448.0000	1224.5036	D	***1661.7734***	831.3903	1644.7469	822.8771	1643.7628	822.3851	15
23	2594.1055	1297.5564	2577.0789	1289.0431	2576.0949	1288.5511	K	***1546.7465***	773.8769	1529.7199	765.3636	1528.7359	764.8716	14
24	2681.1375	1341.0724	2664.1110	1332.5591	2663.1270	1332.0671	S	***1418.6515***	709.8294	1401.6249	701.3161	1400.6409	700.8241	13
25	2782.1852	1391.5962	2765.1587	1383.0830	2764.1746	1382.5910	T	***1331.6195***	666.3134	1314.5929	657.8001	1313.6089	657.3081	12
26	2839.2067	1420.1070	2822.1801	1411.5937	2821.1961	1411.1017	G	***1230.5718***	615.7895	1213.5452	607.2763	1212.5612	606.7842	11

27	2936.2594	1468.6334	2919.2329	1460.1201	2918.2489	1459.6281	P	1173.5503	587.2788	1156.5238	578.7655	1155.5398	578.2735	10
28	3096.2901	1548.6487	3079.2635	1540.1354	3078.2795	1539.6434	C	1076.4976	538.7524	1059.4710	530.2391	1058.4870	529.7471	9
29	3209.3741	1605.1907	3192.3476	1596.6774	3191.3636	1596.1854	I	***916.4669***	458.7371	899.4404	450.2238	898.4563	449.7318	8
30	3296.4062	1648.7067	3279.3796	1640.1934	3278.3956	1639.7014	S	***803.3828***	402.1951	786.3563	393.6818	785.3723	393.1898	7
31	3410.4491	1705.7282	3393.4226	1697.2149	3392.4385	1696.7229	N	716.3508	358.6790	699.3243	350.1658			6
32	3507.5019	1754.2546	3490.4753	1745.7413	3489.4913	1745.2493	P	***602.3079***	301.6576	585.2813	293.1443			5
33	3667.5325	1834.2699	3650.5060	1825.7566	3649.5219	1825.2646	C	505.2551	253.1312	488.2286	244.6179			4
34	3724.5540	1862.7806	3707.5274	1854.2674	3706.5434	1853.7753	G	345.2245	173.1159	328.1979	164.6026			3
35	3837.6380	1919.3227	3820.6115	1910.8094	3819.6275	1910.3174	L	288.2030	144.6051	271.1765	136.0919			2
36							R	175.1190	88.0631	158.0924	79.5498			1

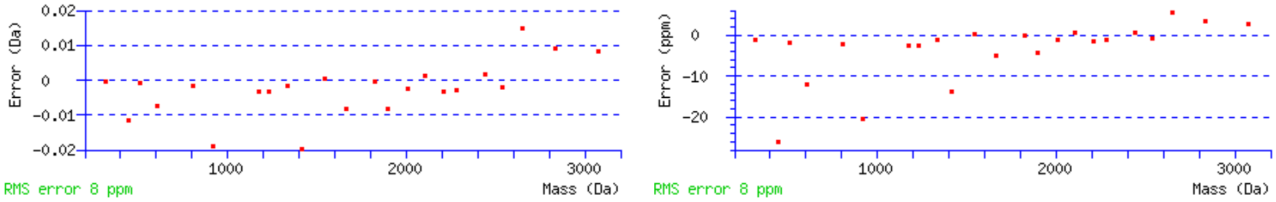

NCBI **BLAST** search of SLLESEDCKLPSNPCATTNACDKSTGPCISNPCGLR
(Parameters: blastp, nr protein database, expect=20000, no filter, PAM30)
Other BLAST web gateways

All matches to this query

Score	Mr(calc):	Delta	Sequence
155.7	4010.7424	0.9978	SLLESEDCKLPSNPCATTNACDKSTGPCISNPCGLR
139.8	4009.7584	1.9818	SLLESEDCKLPSNPCATTNACDKSTGPCISNPCGLR
62.3	4009.7584	1.9818	SLLESEDCKLPSNPCATTNACDKSTGPCISNPCGLR
39.8	4010.7424	0.9978	SLLESEDCKLPSNPCATTNACDKSTGPCISNPCGLR
30.9	4009.7584	1.9818	SLLESEDCKLPSNPCATTNACDKSTGPCISNPCGLR
0.5	4010.7424	0.9978	SLLESEDCKLPSNPCATTNACDKSTGPCISNPCGLR

Mascot: http://www.matrixscience.com/

Mascot Search Results

Peptide View

MS/MS Fragmentation of **SLNSRFAAFIDKVR**
Found in **sp|O43790|KRT86_HUMAN**, Keratin, type II cuticular Hb6 OS=Homo sapiens GN=KRT86 PE=1 SV=1

Match to Query 21149: 1623.873552 from(542.298460,3+) intensity(1703711.1250) scans(14216) rtinseconds(2600) index(41888)
Title: 160219_Sunil_SDSI_A_Spectrum096473_scans__14216_RTINSECONDS=2600
Data file C:\\Sunil\\TKAP\\T\\T160219_Sunil_SDSI_A.mgf

Click mouse within plot area to zoom in by factor of two about that point
Or, Plot from 100 to 1600 Da Full range
Label all possible matches ○ Label matches used for scoring ◉

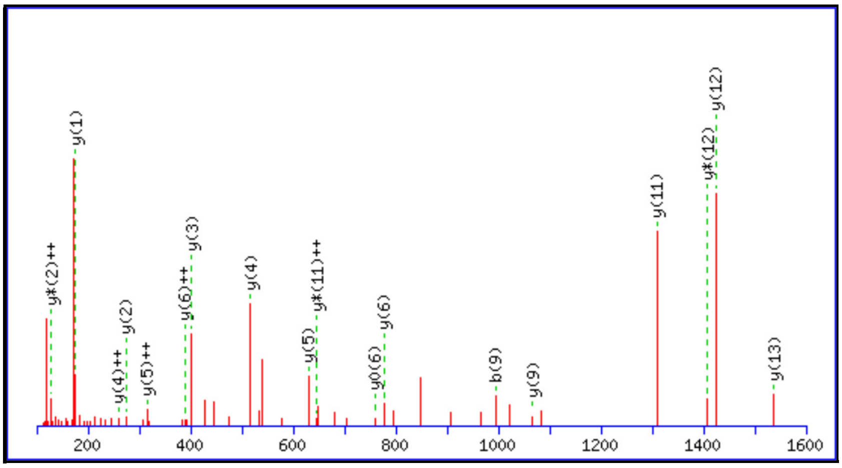

Monoisotopic mass of neutral peptide Mr(calc): 1623.8733
Fixed modifications: Carbamidomethyl (C) (apply to specified residues or termini only)
Variable modifications:
N3 : Deamidated (NQ)
Ions Score: 61 **Expect:** 0.00013
Matches : 18/146 fragment ions using 37 most intense peaks (help)

#	b	b^{++}	b*	b^{*++}	b^0	b^{0++}	Seq.	y	y^{++}	y*	y^{*++}	y^0	y^{0++}	#
1	88.0393	44.5233			70.0287	35.5180	S							14
2	201.1234	101.0653			183.1128	92.0600	L	***1537.8485***	769.4279	1520.8220	760.9146	1519.8380	760.4226	13
3	316.1503	158.5788	299.1238	150.0655	298.1397	149.5735	N	***1424.7645***	712.8859	**1407.7379**	704.3726	1406.7539	703.8806	12
4	403.1823	202.0948	386.1558	193.5815	385.1718	193.0895	S	***1309.7375***	655.3724	1292.7110	**646.8591**	1291.7270	646.3671	11
5	559.2835	280.1454	542.2569	271.6321	541.2729	271.1401	R	1222.7055	611.8564	1205.6790	603.3431	1204.6949	602.8511	10
6	706.3519	353.6796	689.3253	345.1663	688.3413	344.6743	F	***1066.6044***	533.8058	1049.5778	525.2926	1048.5938	524.8006	9
7	777.3890	389.1981	760.3624	380.6849	759.3784	380.1928	A	919.5360	460.2716	902.5094	451.7584	901.5254	451.2663	8
8	848.4261	424.7167	831.3995	416.2034	830.4155	415.7114	A	848.4989	424.7531	831.4723	416.2398	830.4883	415.7478	7
9	**995.4945**	498.2509	978.4680	489.7376	977.4839	489.2456	F	***777.4618***	**389.2345**	760.4352	380.7212	**759.4512**	380.2292	6
10	1108.5786	554.7929	1091.5520	546.2796	1090.5680	545.7876	I	***630.3933***	**315.7003**	613.3668	307.1870	612.3828	306.6950	5
11	1223.6055	612.3064	1206.5790	603.7931	1205.5949	603.3011	D	***517.3093***	**259.1583**	500.2827	250.6450	499.2987	250.1530	4
12	1351.7005	676.3539	1334.6739	667.8406	1333.6899	667.3486	K	***402.2823***	201.6448	385.2558	193.1315			3
13	1450.7689	725.8881	1433.7423	717.3748	1432.7583	716.8828	V	***274.1874***	137.5973	257.1608	**129.0840**			2
14							R	***175.1190***	88.0631	158.0924	79.5498			1

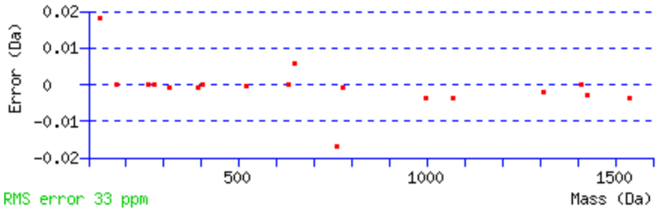

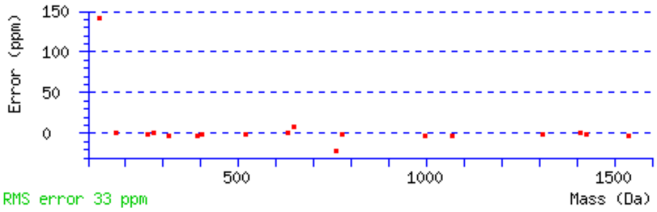

NCBI **BLAST** search of SLNSRFAAFIDKVR
(Parameters: blastp, nr protein database, expect=20000, no filter, PAM30)
Other BLAST web gateways

All matches to this query

Score	Mr(calc):	Delta	Sequence
61.2	1623.8733	0.0003	SLNSRFAAFIDKVR
16.9	1623.8620	0.0115	ALNNKFASFIDKVR
15.4	1622.8780	0.9955	ALNNKFASFIDKVR
15.4	1622.8780	0.9955	ALNNKFASFIDKVR
1.1	1623.8688	0.0048	TVLKSTSKLMTQMR
0.6	1623.8807	-0.0071	QMFLLQYALDVKR

Mascot: http://www.matrixscience.com/

Mascot Search Results

Peptide View

MS/MS Fragmentation of **SLNSRFAAFIDKVR**
Found in **sp|P78385|KRT83_HUMAN**, Keratin, type II cuticular Hb3 OS=Homo sapiens GN=KRT83 PE=1 SV=2

Match to Query 21149: 1623.873552 from(542.298460,3+) intensity(1703711.1250) scans(14216) rtinseconds(2600) index(41888)
Title: 160219_Sunil_SDSI_A_Spectrum096473_scans__14216_RTINSECONDS=2600
Data file C:\\Sunil\\TKAP\\T\\T160219_Sunil_SDSI_A.mgf

Click mouse within plot area to zoom in by factor of two about that point
Or, Plot from 100 to 1600 Da Full range
Label all possible matches ○ Label matches used for scoring ◉

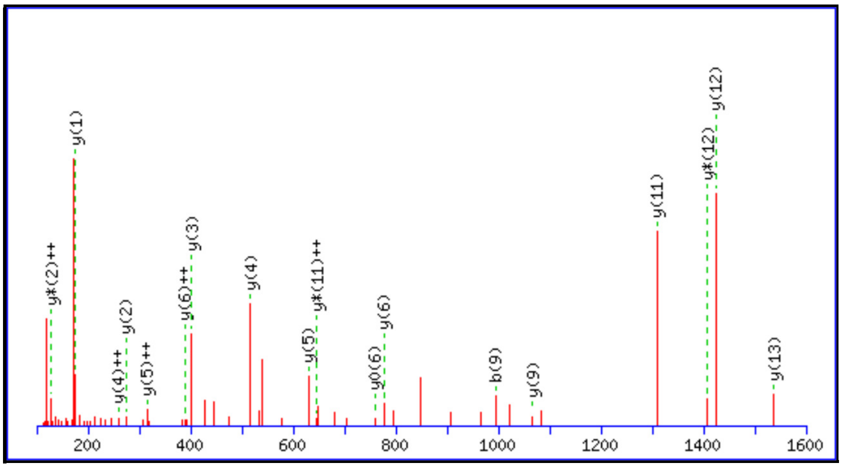

Monoisotopic mass of neutral peptide Mr(calc): 1623.8733
Fixed modifications: Carbamidomethyl (C) (apply to specified residues or termini only)
Variable modifications:
N3 : Deamidated (NQ)
Ions Score: 61 **Expect:** 0.00013
Matches : 18/146 fragment ions using 37 most intense peaks (help)

#	b	b^{++}	b*	b^{*++}	b^{0}	b^{0++}	Seq.	y	y^{++}	y*	y^{*++}	y^{0}	y^{0++}	#
1	88.0393	44.5233			70.0287	35.5180	S							14
2	201.1234	101.0653			183.1128	92.0600	L	***1537.8485***	769.4279	1520.8220	760.9146	1519.8380	760.4226	13
3	316.1503	158.5788	299.1238	150.0655	298.1397	149.5735	N	***1424.7645***	712.8859	1407.7379	704.3726	1406.7539	703.8806	12
4	403.1823	202.0948	386.1558	193.5815	385.1718	193.0895	S	***1309.7375***	655.3724	1292.7110	646.8591	1291.7270	646.3671	11
5	559.2835	280.1454	542.2569	271.6321	541.2729	271.1401	R	1222.7055	611.8564	1205.6790	603.3431	1204.6949	602.8511	10
6	706.3519	353.6796	689.3253	345.1663	688.3413	344.6743	F	***1066.6044***	533.8058	1049.5778	525.2926	1048.5938	524.8006	9
7	777.3890	389.1981	760.3624	380.6849	759.3784	380.1928	A	919.5360	460.2716	902.5094	451.7584	901.5254	451.2663	8
8	848.4261	424.7167	831.3995	416.2034	830.4155	415.7114	A	848.4989	424.7531	831.4723	416.2398	830.4883	415.7478	7
9	**995.4945**	498.2509	978.4680	489.7376	977.4839	489.2456	F	***777.4618***	389.2345	760.4352	380.7212	759.4512	380.2292	6
10	1108.5786	554.7929	1091.5520	546.2796	1090.5680	545.7876	I	***630.3933***	315.7003	613.3668	307.1870	612.3828	306.6950	5
11	1223.6055	612.3064	1206.5790	603.7931	1205.5949	603.3011	D	***517.3093***	259.1583	500.2827	250.6450	499.2987	250.1530	4
12	1351.7005	676.3539	1334.6739	667.8406	1333.6899	667.3486	K	***402.2823***	201.6448	385.2558	193.1315			3
13	1450.7689	725.8881	1433.7423	717.3748	1432.7583	716.8828	V	***274.1874***	137.5973	257.1608	129.0840			2
14							R	***175.1190***	88.0631	158.0924	79.5498			1

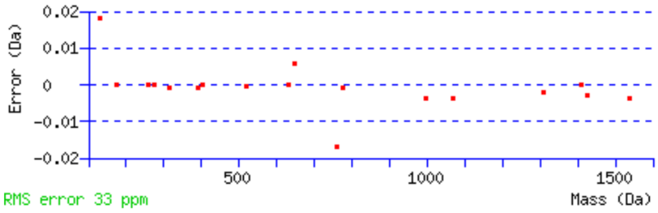

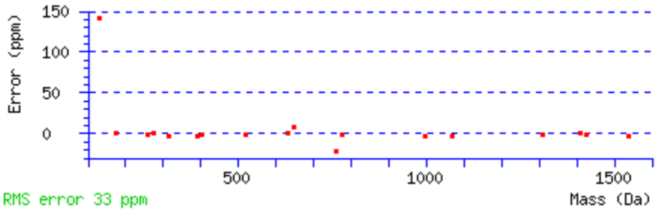

NCBI **BLAST** search of SLNSRFAAFIDKVR
(Parameters: blastp, nr protein database, expect=20000, no filter, PAM30)
Other BLAST web gateways

All matches to this query

Score	Mr(calc):	Delta	Sequence
61.2	1623.8733	0.0003	SLNSRFAAFIDKVR
16.9	1623.8620	0.0115	ALNNKFASFIDKVR
15.4	1622.8780	0.9955	ALNNKFASFIDKVR
15.4	1622.8780	0.9955	ALNNKFASFIDKVR
1.1	1623.8688	0.0048	TVLKSTSKLMTQMR
0.6	1623.8807	-0.0071	QMFLLQYALDVKR

Mascot: http://www.matrixscience.com/

Mascot Search Results

Peptide View

MS/MS Fragmentation of **SQYEALVETNRR**
Found in **sp|Q15323|K1H1_HUMAN**, Keratin, type I cuticular Ha1 OS=Homo sapiens GN=KRT31 PE=2 SV=3

Match to Query 16602: 1465.713848 from(733.864200,2+) intensity(17400520.0000) scans(6175) rtinseconds(1191) index(19680)
Title: 160219_Sunil_SDSI_A_Spectrum072984_scans__6175_RTINSECONDS=1191
Data file C:\\Sunil\\TKAP\\T\\T160219_Sunil_SDSI_A.mgf

Click mouse within plot area to zoom in by factor of two about that point
Or, Plot from 100 to 1500 Da Full range
Label all possible matches Label matches used for scoring

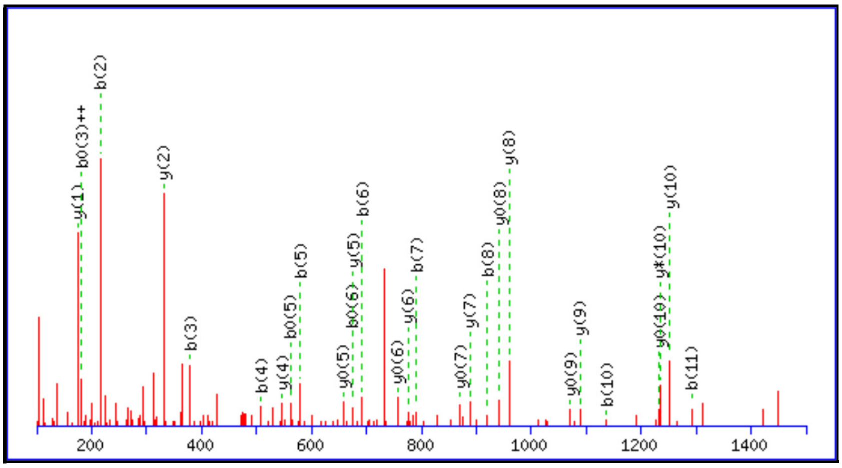

Monoisotopic mass of neutral peptide Mr(calc): 1465.7161
Fixed modifications: Carbamidomethyl (C) (apply to specified residues or termini only)
Variable modifications:
N10 : Deamidated (NQ)
Ions Score: 57 **Expect:** 0.00041
Matches : 28/124 fragment ions using 48 most intense peaks (help)

#	b	b++	b*	b*++	b0	b0++	Seq.	y	y++	y*	y*++	y0	y0++	#
1	88.0393	44.5233			70.0287	35.5180	S							12
2	216.0979	108.5526	199.0713	100.0393	198.0873	99.5473	Q	1379.6914	690.3493	1362.6648	681.8360	1361.6808	681.3440	11
3	379.1612	190.0842	362.1347	181.5710	361.1506	181.0790	Y	1251.6328	626.3200	1234.6062	617.8068	1233.6222	617.3148	10
4	508.2038	254.6055	491.1773	246.0923	490.1932	245.6003	E	1088.5695	544.7884	1071.5429	536.2751	1070.5589	535.7831	9
5	579.2409	290.1241	562.2144	281.6108	561.2304	281.1188	A	959.5269	480.2671	942.5003	471.7538	941.5163	471.2618	8
6	692.3250	346.6661	675.2984	338.1529	674.3144	337.6608	L	888.4898	444.7485	871.4632	436.2352	870.4792	435.7432	7
7	791.3934	396.2003	774.3668	387.6871	773.3828	387.1951	V	775.4057	388.2065	758.3791	379.6932	757.3951	379.2012	6
8	920.4360	460.7216	903.4094	452.2084	902.4254	451.7164	E	676.3373	338.6723	659.3107	330.1590	658.3267	329.6670	5
9	1021.4837	511.2455	1004.4571	502.7322	1003.4731	502.2402	T	547.2947	274.1510	530.2681	265.6377	529.2841	265.1457	4
10	1136.5106	568.7589	1119.4841	560.2457	1118.5000	559.7537	N	446.2470	223.6271	429.2205	215.1139			3
11	1292.6117	646.8095	1275.5852	638.2962	1274.6012	637.8042	R	331.2201	166.1137	314.1935	157.6004			2
12							R	175.1190	88.0631	158.0924	79.5498			1

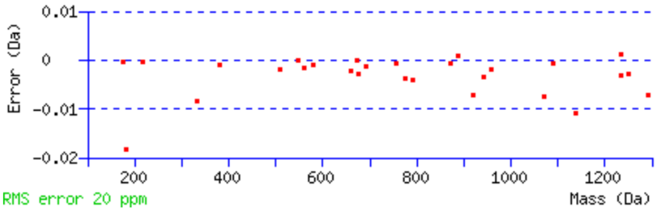

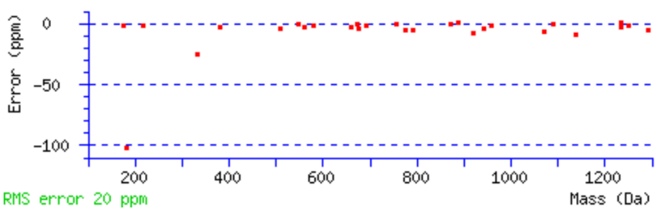

NCBI **BLAST** search of SQYEALVETNRR
(Parameters: blastp, nr protein database, expect=20000, no filter, PAM30)
Other BLAST web gateways

All matches to this query

Score	Mr(calc):	Delta	Sequence
57.2	1465.7161	-0.0022	SQYEALVETNRR
2.6	1464.7143	0.9995	CSSDKAIQFPRR
1.4	1465.7170	-0.0031	RFCLVPSMEGVR
1.3	1464.7249	0.9890	DLYDAVWIQVSR
1.2	1465.7235	-0.0097	VDFIKDAVAMADR

Mascot: http://www.matrixscience.com/

Mascot Search Results

Peptide View

MS/MS Fragmentation of **TKEEINELNR**
Found in **sp|O43790|KRT86_HUMAN**, Keratin, type II cuticular Hb6 OS=Homo sapiens GN=KRT86 PE=1 SV=1

Match to Query 9756: 1245.621522 from(416.214450,3+) intensity(12807713.0000) scans(4024) rtinseconds(845) index(2585)
Title: 160219_Sunil_SDSI_A_Spectrum054693_scans__4024_RTINSECONDS=845
Data file C:\\Sunil\\TKAP\\T\\T160219_Sunil_SDSI_A.mgf

Click mouse within plot area to zoom in by factor of two about that point
Or, Plot from 0 to 1300 Da Full range
Label all possible matches ○ Label matches used for scoring ◉

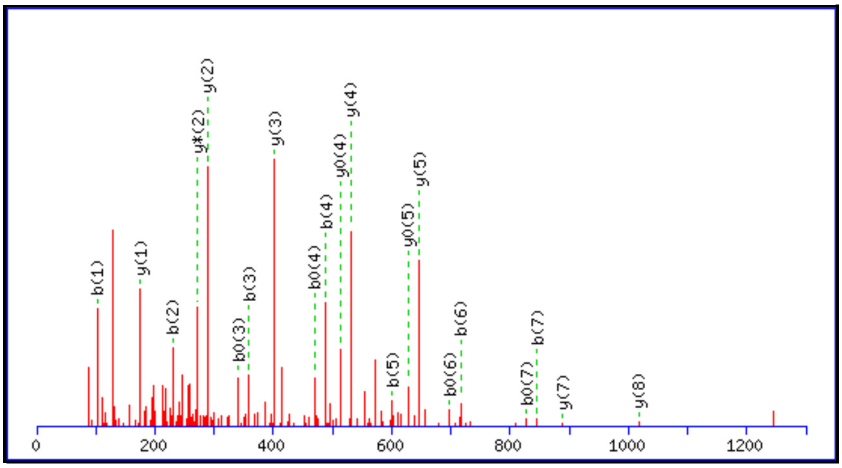

Monoisotopic mass of neutral peptide Mr(calc): 1245.6200
Fixed modifications: Carbamidomethyl (C) (apply to specified residues or termini only)
Variable modifications:
N6 : Deamidated (NQ)
Ions Score: 69 **Expect:** 3.2e-005
Matches : 21/100 fragment ions using 26 most intense peaks (help)

#	b	b++	b*	b*++	b0	b0++	Seq.	y	y++	y*	y*++	y0	y0++	#
1	***102.0550***	51.5311			84.0444	42.5258	T							10
2	***230.1499***	115.5786	213.1234	107.0653	212.1394	106.5733	K	1145.5797	573.2935	1128.5531	564.7802	1127.5691	564.2882	9
3	***359.1925***	180.0999	342.1660	171.5866	341.1819	171.0946	E	***1017.4847***	509.2460	1000.4582	500.7327	999.4742	500.2407	8
4	***488.2351***	244.6212	471.2086	236.1079	470.2245	235.6159	E	***888.4421***	444.7247	871.4156	436.2114	870.4316	435.7194	7
5	***601.3192***	301.1632	584.2926	292.6499	583.3086	292.1579	I	759.3995	380.2034	742.3730	371.6901	741.3890	371.1981	6
6	***716.3461***	358.6767	699.3196	350.1634	698.3355	349.6714	N	***646.3155***	323.6614	629.2889	315.1481	628.3049	314.6561	5
7	***845.3887***	423.1980	828.3622	414.6847	827.3781	414.1927	E	***531.2885***	266.1479	514.2620	257.6346	513.2780	257.1426	4
8	958.4728	479.7400	941.4462	471.2267	940.4622	470.7347	L	***402.2459***	201.6266	385.2194	193.1133			3
9	1072.5157	536.7615	1055.4891	528.2482	1054.5051	527.7562	N	***289.1619***	145.0846	272.1353	136.5713			2
10							R	***175.1190***	88.0631	158.0924	79.5498			1

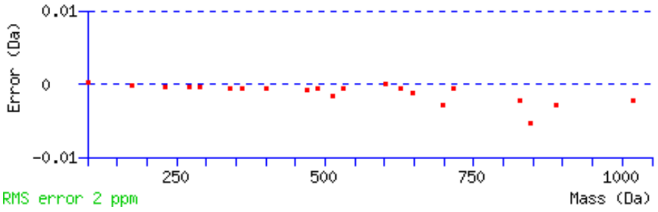

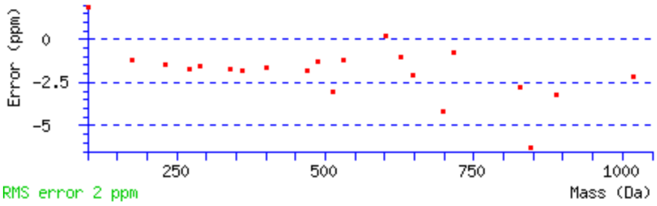

NCBI **BLAST** search of TKEEINELNR
(Parameters: blastp, nr protein database, expect=20000, no filter, PAM30)
Other BLAST web gateways

All matches to this query

Score	Mr(calc):	Delta	Sequence
68.6	1245.6200	0.0015	TKEEINELNR

23.5	1245.6200	0.0015	TKEEINELNR
10.7	1245.6313	-0.0097	KSLEEEQRAR
10.6	1243.6156	2.0059	TNQELQEINR
9.7	1243.6269	1.9947	AELREDERAR
8.7	1245.6136	0.0080	QMVPSSASGQVR
5.6	1245.6288	-0.0073	FCITVSHLNR
5.6	1245.6109	0.0107	RNGSRNCLNR
5.3	1245.6201	0.0015	ETDKQILQNR
4.6	1245.6102	0.0114	LWQSAQQARR

Mascot: http://www.matrixscience.com/

Mascot Search Results

Peptide View

MS/MS Fragmentation of **TKEEINELNR**
Found in **sp|O43790|KRT86_HUMAN**, Keratin, type II cuticular Hb6 OS=Homo sapiens GN=KRT86 PE=1 SV=1

Match to Query 9756: 1245.621522 from(416.214450,3+) intensity(12807713.0000) scans(4024) rtinseconds(845) index(2585)
Title: 160219_Sunil_SDSI_A_Spectrum054693_scans__4024_RTINSECONDS=845
Data file C:\\Sunil\\TKAP\\T\\T160219_Sunil_SDSI_A.mgf

Click mouse within plot area to zoom in by factor of two about that point
Or, Plot from 0 to 1300 Da Full range
Label all possible matches Label matches used for scoring

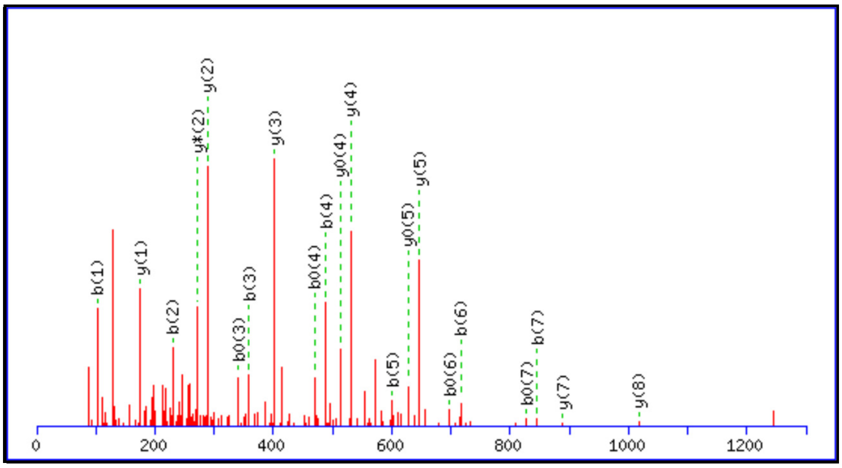

Monoisotopic mass of neutral peptide Mr(calc): 1245.6200
Fixed modifications: Carbamidomethyl (C) (apply to specified residues or termini only)
Variable modifications:
N6 : Deamidated (NQ)
Ions Score: 69 **Expect:** 3.2e-005
Matches : 21/100 fragment ions using 26 most intense peaks (help)

#	b	b++	b*	b*++	b0	b0++	Seq.	y	y++	y*	y*++	y0	y0++	#
1	***102.0550***	51.5311			84.0444	42.5258	T							10
2	***230.1499***	115.5786	213.1234	107.0653	212.1394	106.5733	K	1145.5797	573.2935	1128.5531	564.7802	1127.5691	564.2882	9
3	***359.1925***	180.0999	342.1660	171.5866	341.1819	171.0946	E	***1017.4847***	509.2460	1000.4582	500.7327	999.4742	500.2407	8
4	***488.2351***	244.6212	471.2086	236.1079	470.2245	235.6159	E	***888.4421***	444.7247	871.4156	436.2114	870.4316	435.7194	7
5	***601.3192***	301.1632	584.2926	292.6499	583.3086	292.1579	I	759.3995	380.2034	742.3730	371.6901	741.3890	371.1981	6
6	***716.3461***	358.6767	699.3196	350.1634	698.3355	349.6714	N	***646.3155***	323.6614	629.2889	315.1481	628.3049	314.6561	5
7	***845.3887***	423.1980	828.3622	414.6847	827.3781	414.1927	E	***531.2885***	266.1479	514.2620	257.6346	513.2780	257.1426	4
8	958.4728	479.7400	941.4462	471.2267	940.4622	470.7347	L	***402.2459***	201.6266	385.2194	193.1133			3
9	1072.5157	536.7615	1055.4891	528.2482	1054.5051	527.7562	N	***289.1619***	145.0846	272.1353	136.5713			2
10							R	***175.1190***	88.0631	158.0924	79.5498			1

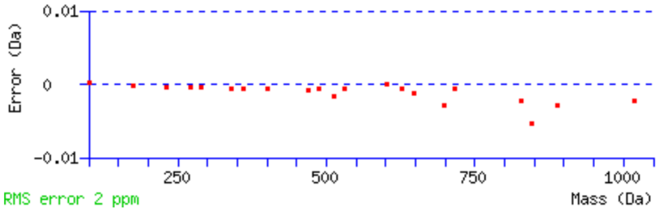

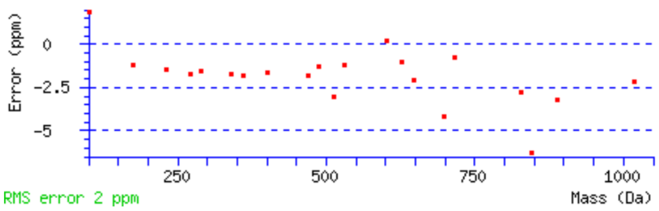

NCBI **BLAST** search of TKEEINELNR
(Parameters: blastp, nr protein database, expect=20000, no filter, PAM30)
Other BLAST web gateways

All matches to this query

Score	Mr(calc):	Delta	Sequence
68.6	1245.6200	0.0015	TKEEINELNR

23.5	1245.6200	0.0015	TKEEINELNR
10.7	1245.6313	-0.0097	KSLEEEQRAR
10.6	1243.6156	2.0059	TNQELQEINR
9.7	1243.6269	1.9947	AELREDERAR
8.7	1245.6136	0.0080	QMVPSSASGQVR
5.6	1245.6288	-0.0073	FCITVSHLNR
5.6	1245.6109	0.0107	RNGSRNCLNR
5.3	1245.6201	0.0015	ETDKQILQNR
4.6	1245.6102	0.0114	LWQSAQQARR

Mascot: http://www.matrixscience.com/

Mascot Search Results

Peptide View

MS/MS Fragmentation of **TKYQTELSLR**
Found in **sp|Q15323|K1H1_HUMAN**, Keratin, type I cuticular Ha1 OS=Homo sapiens GN=KRT31 PE=2 SV=3

Match to Query 9520: 1238.648788 from(620.331670,2+) intensity(380505088.0000) scans(6176) rtinseconds(1191) index(19681)
Title: 160219_Sunil_SDSI_A_Spectrum072985_scans__6176_RTINSECONDS=1191
Data file C:\\Sunil\\TKAP\\T\\T160219_Sunil_SDSI_A.mgf

Click mouse within plot area to zoom in by factor of two about that point
Or, Plot from 0 to 1300 Da Full range
Label all possible matches ○ Label matches used for scoring ◉

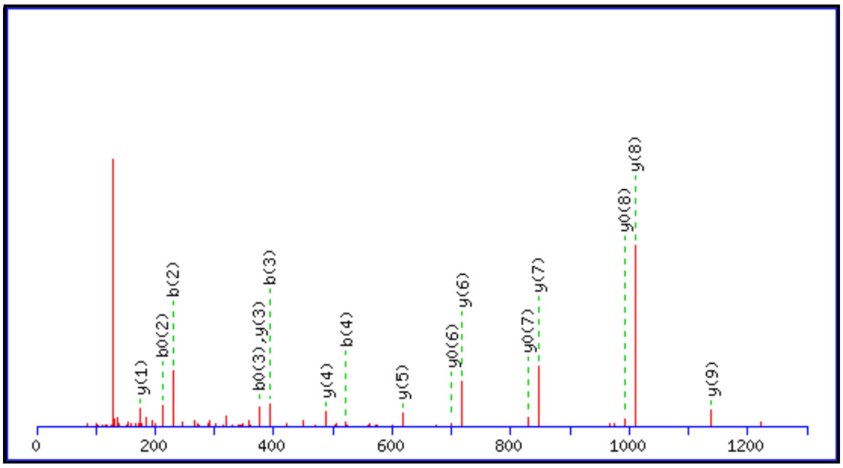

Monoisotopic mass of neutral peptide Mr(calc): 1238.6506
Fixed modifications: Carbamidomethyl (C) (apply to specified residues or termini only)
Variable modifications:
Q4 : Deamidated (NQ)
Ions Score: 66 **Expect:** 3.5e-005
Matches : 16/102 fragment ions using 22 most intense peaks (help)

#	b	b++	b*	b*++	b0	b0++	Seq.	y	y++	y*	y*++	y0	y0++	#
1	102.0550	51.5311			84.0444	42.5258	T							10
2	**230.1499**	115.5786	213.1234	107.0653	212.1394	106.5733	K	***1138.6103***	569.8088	1121.5837	561.2955	1120.5997	560.8035	9
3	**393.2132**	197.1103	376.1867	188.5970	375.2027	188.1050	Y	***1010.5153***	505.7613	993.4888	497.2480	992.5047	496.7560	8
4	**522.2558**	261.6316	505.2293	253.1183	504.2453	252.6263	Q	***847.4520***	424.2296	830.4254	415.7164	829.4414	415.2243	7
5	623.3035	312.1554	606.2770	303.6421	605.2930	303.1501	T	***718.4094***	359.7083	701.3828	351.1951	700.3988	350.7030	6
6	752.3461	376.6767	735.3196	368.1634	734.3355	367.6714	E	***617.3617***	309.1845	600.3352	300.6712	599.3511	300.1792	5
7	865.4302	433.2187	848.4036	424.7055	847.4196	424.2134	L	***488.3191***	244.6632	471.2926	236.1499	470.3085	235.6579	4
8	952.4622	476.7347	935.4357	468.2215	934.4516	467.7295	S	***375.2350***	188.1212	358.2085	179.6079	357.2245	179.1159	3
9	1065.5463	533.2768	1048.5197	524.7635	1047.5357	524.2715	L	288.2030	144.6051	271.1765	136.0919			2
10							R	***175.1190***	88.0631	158.0924	79.5498			1

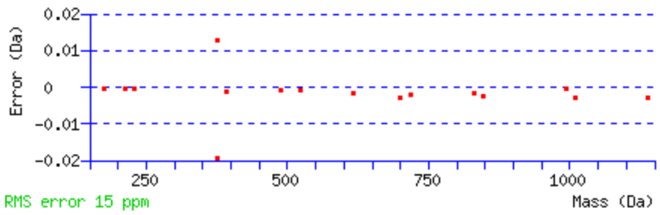

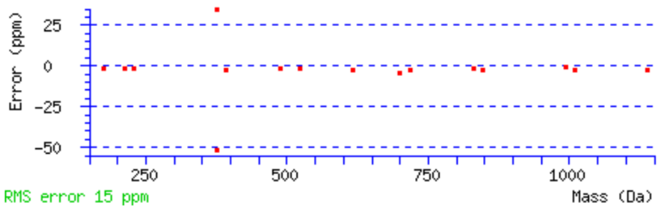

NCBI **BLAST** search of TKYQTELSLR
(Parameters: blastp, nr protein database, expect=20000, no filter, PAM30)
Other BLAST web gateways

All matches to this query

Score	Mr(calc):	Delta	Sequence
66.4	1238.6506	-0.0018	TKYETELSLR

66.4	1238.6506	-0.0018	TKYQTELSLR
6.4	1238.6367	0.0121	KTEVQAHSPSR
3.9	1237.6342	1.0145	DLYQKWKQK
3.3	1238.6394	0.0094	KTTIYEIQDK
2.9	1238.6507	-0.0019	TKTKYTGNPTK
1.7	1238.6367	0.0121	AHERELETVR
1.5	1238.6560	-0.0072	LQHWLWSIR
1.0	1238.6506	-0.0018	KLHNQEQVIK
0.7	1237.6554	0.9934	KTKITFEEDK

Mascot: http://www.matrixscience.com/

Mascot Search Results

Peptide View

MS/MS Fragmentation of **TVNALEIELQAQHNLR**
Found in **sp|O76009|KT33A_HUMAN**, Keratin, type I cuticular Ha3-I OS=Homo sapiens GN=KRT33A PE=2 SV=2

Match to Query 25670: 1848.968488 from(925.491520,2+) intensity(5108248.5000) scans(13281) rtinseconds(2412) index(25810)
Title: 160219_Sunil_SDSI_A_Spectrum079116_scans__13281_RTINSECONDS=2412
Data file C:\\Sunil\\TKAP\\T\\T160219_Sunil_SDSI_A.mgf

Click mouse within plot area to zoom in by factor of two about that point
Or, Plot from 100 to 1900 Da Full range
Label all possible matches ○ Label matches used for scoring ●

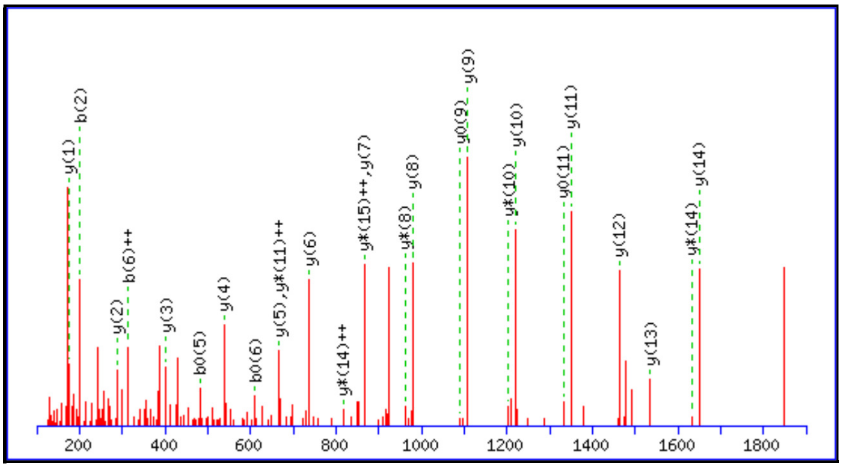

Monoisotopic mass of neutral peptide Mr(calc): 1848.9693
Fixed modifications: Carbamidomethyl (C) (apply to specified residues or termini only)
Variable modifications:
N3 : Deamidated (NQ)
Ions Score: 102 **Expect:** 1.4e-008
Matches : 26/160 fragment ions using 45 most intense peaks (help)

#	b	b++	b*	b*++	b0	b0++	Seq.	y	y++	y*	y*++	y0	y0++	#
1	102.0550	51.5311			84.0444	42.5258	T							16
2	**201.1234**	101.0653			183.1128	92.0600	V	1748.9290	874.9681	1731.9024	866.4549	1730.9184	865.9628	15
3	316.1503	158.5788	299.1238	150.0655	298.1397	149.5735	N	***1649.8606***	825.4339	1632.8340	816.9206	1631.8500	816.4286	14
4	387.1874	194.0974	370.1609	185.5841	369.1769	185.0921	A	***1534.8336***	767.9204	1517.8071	759.4072	1516.8231	758.9152	13
5	500.2715	250.6394	483.2449	242.1261	482.2609	241.6341	L	***1463.7965***	732.4019	1446.7700	723.8886	1445.7859	723.3966	12
6	629.3141	**315.1607**	612.2875	306.6474	611.3035	306.1554	E	***1350.7124***	675.8599	1333.6859	667.3466	1332.7019	666.8546	11
7	742.3981	371.7027	725.3716	363.1894	724.3876	362.6974	I	***1221.6698***	611.3386	1204.6433	602.8253	1203.6593	602.3333	10
8	871.4407	436.2240	854.4142	427.7107	853.4302	427.2187	E	***1108.5858***	554.7965	1091.5592	546.2833	1090.5752	545.7912	9
9	984.5248	492.7660	967.4983	484.2528	966.5142	483.7608	L	***979.5432***	490.2752	962.5166	481.7620			8
10	1112.5834	556.7953	1095.5568	548.2821	1094.5728	547.7900	Q	***866.4591***	433.7332	849.4326	425.2199			7
11	1183.6205	592.3139	1166.5939	583.8006	1165.6099	583.3086	A	***738.4005***	369.7039	721.3740	361.1906			6
12	1311.6791	656.3432	1294.6525	647.8299	1293.6685	647.3379	Q	***667.3634***	334.1854	650.3369	325.6721			5
13	1448.7380	724.8726	1431.7114	716.3594	1430.7274	715.8673	H	***539.3049***	270.1561	522.2783	261.6428			4
14	1562.7809	781.8941	1545.7544	773.3808	1544.7703	772.8888	N	***402.2459***	201.6266	385.2194	193.1133			3
15	1675.8650	838.4361	1658.8384	829.9229	1657.8544	829.4308	L	***288.2030***	144.6051	271.1765	136.0919			2
16							R	***175.1190***	88.0631	158.0924	79.5498			1

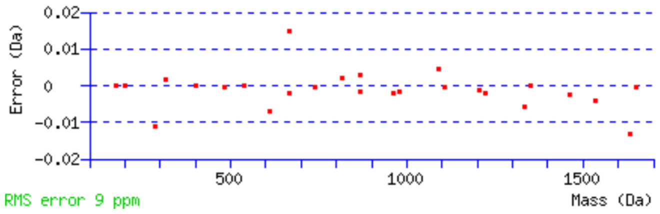

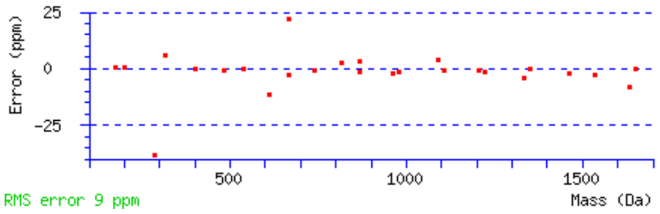

NCBI **BLAST** search of TVNALEIELQAQHNLR

(Parameters: blastp, nr protein database, expect=20000, no filter, PAM30)
Other BLAST web gateways

All matches to this query

Score	Mr(calc):	Delta	Sequence
102.1	1848.9693	-0.0008	TVNALEIELQAQHNLR
21.5	1848.9693	-0.0008	TVNALEIELQAQHNLR
10.4	1848.9693	-0.0008	TVNALEIELQAQHNLR
3.2	1846.9532	2.0152	IQSSGGPLQITMKMVPK
2.5	1848.9693	-0.0008	TVNALEIELQAQHNLR

Mascot: http://www.matrixscience.com/

Mascot Search Results

Peptide View

MS/MS Fragmentation of **TVNALEIELQAQHNLRDSLENTLTESEAR**
Found in **sp|Q15323|K1H1_HUMAN**, Keratin, type I cuticular Ha1 OS=Homo sapiens GN=KRT31 PE=2 SV=3

Match to Query 41480: 3294.629096 from(824.664550,4+) intensity(51466172.0000) scans(14853) rtinseconds(2680) index(27016)
Title: 160219_Sunil_SDSI_A_Spectrum080326_scans__14853_RTINSECONDS=2680
Data file C:\\Sunil\\TKAP\\T\\T160219_Sunil_SDSI_A.mgf

Click mouse within plot area to zoom in by factor of two about that point
Or, Plot from 200 to 3200 Da Full range
Label all possible matches ○ Label matches used for scoring ◉

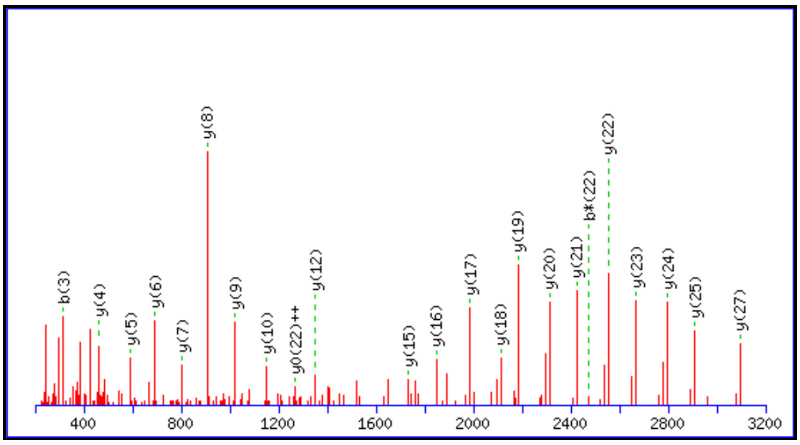

Monoisotopic mass of neutral peptide Mr(calc): 3294.6327
Fixed modifications: Carbamidomethyl (C) (apply to specified residues or termini only)
Variable modifications:
N21 : Deamidated (NQ)
Ions Score: 185 **Expect:** 7.4e-017
Matches : 23/328 fragment ions using 29 most intense peaks (help)

#	b	b++	b*	b*++	b0	b0++	Seq.	y	y++	y*	y*++	y0	y0++	#
1	102.0550	51.5311			84.0444	42.5258	T							29
2	201.1234	101.0653			183.1128	92.0600	V	3194.5924	1597.7998	3177.5658	1589.2866	3176.5818	1588.7946	28
3	**315.1663**	158.0868	298.1397	149.5735	297.1557	149.0815	N	***3095.5240***	1548.2656	3078.4974	1539.7524	3077.5134	1539.2603	27
4	386.2034	193.6053	369.1769	185.0921	368.1928	184.6001	A	2981.4810	1491.2442	2964.4545	1482.7309	2963.4705	1482.2389	26
5	499.2875	250.1474	482.2609	241.6341	481.2769	241.1421	L	***2910.4439***	1455.7256	2893.4174	1447.2123	2892.4334	1446.7203	25
6	628.3301	314.6687	611.3035	306.1554	610.3195	305.6634	E	***2797.3599***	1399.1836	2780.3333	1390.6703	2779.3493	1390.1783	24
7	741.4141	371.2107	724.3876	362.6974	723.4036	362.2054	I	***2668.3173***	1334.6623	2651.2907	1326.1490	2650.3067	1325.6570	23
8	870.4567	435.7320	853.4302	427.2187	852.4462	426.7267	E	***2555.2332***	1278.1202	2538.2067	1269.6070	2537.2226	1269.1150	22
9	983.5408	492.2740	966.5142	483.7608	965.5302	483.2687	L	***2426.1906***	1213.5989	2409.1641	1205.0857	2408.1801	1204.5937	21
10	1111.5994	556.3033	1094.5728	547.7900	1093.5888	547.2980	Q	***2313.1066***	1157.0569	2296.0800	1148.5436	2295.0960	1148.0516	20
11	1182.6365	591.8219	1165.6099	583.3086	1164.6259	582.8166	A	***2185.0480***	1093.0276	2168.0214	1084.5144	2167.0374	1084.0223	19
12	1310.6951	655.8512	1293.6685	647.3379	1292.6845	646.8459	Q	***2114.0109***	1057.5091	2096.9843	1048.9958	2096.0003	1048.5038	18
13	1447.7540	724.3806	1430.7274	715.8673	1429.7434	715.3753	H	***1985.9523***	993.4798	1968.9257	984.9665	1967.9417	984.4745	17
14	1561.7969	781.4021	1544.7703	772.8888	1543.7863	772.3968	N	***1848.8934***	924.9503	1831.8668	916.4371	1830.8828	915.9450	16
15	1674.8810	837.9441	1657.8544	829.4308	1656.8704	828.9388	L	***1734.8504***	867.9289	1717.8239	859.4156	1716.8399	858.9236	15
16	1830.9821	915.9947	1813.9555	907.4814	1812.9715	906.9894	R	1621.7664	811.3868	1604.7398	802.8736	1603.7558	802.3815	14
17	1946.0090	973.5081	1928.9825	964.9949	1927.9984	964.5029	D	1465.6653	733.3363	1448.6387	724.8230	1447.6547	724.3310	13
18	2033.0410	1017.0242	2016.0145	1008.5109	2015.0305	1008.0189	S	***1350.6383***	675.8228	1333.6118	667.3095	1332.6278	666.8175	12
19	2146.1251	1073.5662	2129.0986	1065.0529	2128.1145	1064.5609	L	1263.6063	632.3068	1246.5798	623.7935	1245.5957	623.3015	11
20	2275.1677	1138.0875	2258.1412	1129.5742	2257.1571	1129.0822	E	***1150.5222***	575.7648	1133.4957	567.2515	1132.5117	566.7595	10
21	2390.1946	1195.6010	2373.1681	1187.0877	2372.1841	1186.5957	N	***1021.4796***	511.2435	1004.4531	502.7302	1003.4691	502.2382	9
22	2491.2423	1246.1248	2474.2158	1237.6115	2473.2318	1237.1195	T	***906.4527***	453.7300	889.4262	445.2167	888.4421	444.7247	8
23	2604.3264	1302.6668	2587.2998	1294.1536	2586.3158	1293.6615	L	***805.4050***	403.2061	788.3785	394.6929	787.3945	394.2009	7
24	2705.3741	1353.1907	2688.3475	1344.6774	2687.3635	1344.1854	T	***692.3210***	346.6641	675.2944	338.1508	674.3104	337.6588	6
25	2834.4167	1417.7120	2817.3901	1409.1987	2816.4061	1408.7067	E	***591.2733***	296.1403	574.2467	287.6270	573.2627	287.1350	5
26	2921.4487	1461.2280	2904.4221	1452.7147	2903.4381	1452.2227	S	***462.2307***	231.6190	445.2041	223.1057	444.2201	222.6137	4
27	3050.4913	1525.7493	3033.4647	1517.2360	3032.4807	1516.7440	E	375.1987	188.1030	358.1721	179.5897	357.1881	179.0977	3

**28**	3121.5284	1561.2678	3104.5018	1552.7546	3103.5178	1552.2626	**A**	246.1561	123.5817	229.1295	115.0684			**2**
**29**							**R**	175.1190	88.0631	158.0924	79.5498			**1**

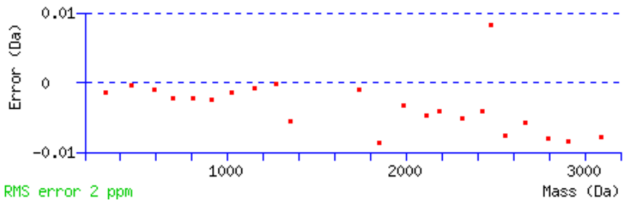

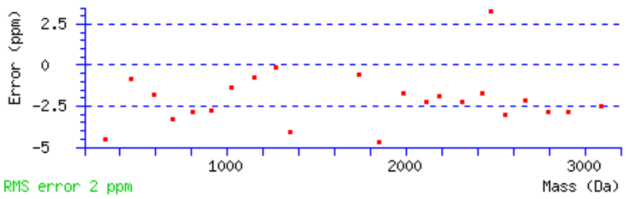

NCBI **BLAST** search of TVNALEIELQAQHNLRDSLENTLTESEAR
(Parameters: blastp, nr protein database, expect=20000, no filter, PAM30)
Other BLAST web gateways

All matches to this query

Score	Mr(calc):	Delta	Sequence
185.3	3294.6327	-0.0036	TVNALEIELQAQHNLRDSLENTLTESEAR
119.4	3294.6327	-0.0036	TVNALEIELQAQHNLRDSLENTLTESEAR
91.1	3294.6327	-0.0036	TVNALEIELQAQHNLRDSLENTLTESEAR
65.7	3294.6327	-0.0036	TVNALEIELQAQHNLRDSLENTLTESEAR
6.9	3294.6327	-0.0036	TVNALEIELQAQHNLRDSLENTLTESEAR
2.9	3293.6487	0.9804	TVNALEIELQAQHNLRDSLENTLTESEAR

Mascot: http://www.matrixscience.com/

Mascot Search Results

Peptide View

MS/MS Fragmentation of **TVNALEIELQAQHNLRDSLENTLTESEAR**
Found in **sp|O76009|KT33A_HUMAN**, Keratin, type I cuticular Ha3-I OS=Homo sapiens GN=KRT33A PE=2 SV=2

Match to Query 41513: 3297.637896 from(825.416750,4+) intensity(2217478.2500) scans(14199) rtinseconds(2569) index(26491)
Title: 160219_Sunil_SDSI_A_Spectrum079797_scans__14199_RTINSECONDS=2569
Data file C:\\Sunil\\TKAP\\T\\T160219_Sunil_SDSI_A.mgf

Click mouse within plot area to zoom in by factor of two about that point
Or, Plot from 200 to 3200 Da Full range
Label all possible matches ○ Label matches used for scoring ◉

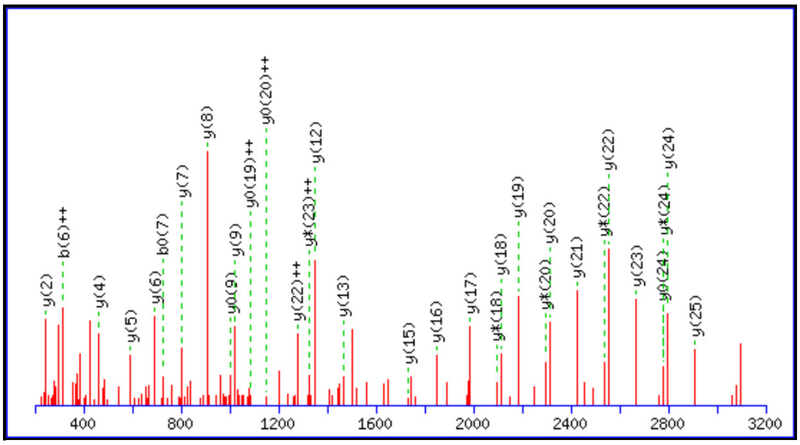

Monoisotopic mass of neutral peptide Mr(calc): 3295.6167
Fixed modifications: Carbamidomethyl (C) (apply to specified residues or termini only)
Variable modifications:
N3 : Deamidated (NQ)
N21 : Deamidated (NQ)
Ions Score: 112 **Expect:** 1.8e-009
Matches : 32/328 fragment ions using 72 most intense peaks (help)

#	b	b^{++}	b^{*}	b^{*++}	b^{0}	b^{0++}	Seq.	y	y^{++}	y^{*}	y^{*++}	y^{0}	y^{0++}	#
1	102.0550	51.5311			84.0444	42.5258	T							29
2	201.1234	101.0653			183.1128	92.0600	V	3195.5764	1598.2918	3178.5499	1589.7786	3177.5658	1589.2866	28
3	316.1503	158.5788	299.1238	150.0655	298.1397	149.5735	N	3096.5080	1548.7576	3079.4814	1540.2444	3078.4974	1539.7524	27
4	387.1874	194.0974	370.1609	185.5841	369.1769	185.0921	A	2981.4810	1491.2442	2964.4545	1482.7309	2963.4705	1482.2389	26
5	500.2715	250.6394	483.2449	242.1261	482.2609	241.6341	L	***2910.4439***	1455.7256	2893.4174	1447.2123	2892.4334	1446.7203	25
6	629.3141	**315.1607**	612.2875	306.6474	611.3035	306.1554	E	***2797.3599***	1399.1836	2780.3333	1390.6703	2779.3493	1390.1783	24
7	742.3981	371.7027	725.3716	363.1894	724.3876	362.6974	I	***2668.3173***	1334.6623	2651.2907	1326.1490	2650.3067	1325.6570	23
8	871.4407	436.2240	854.4142	427.7107	853.4302	427.2187	E	***2555.2332***	1278.1202	2538.2067	1269.6070	2537.2226	1269.1150	22
9	984.5248	492.7660	967.4983	484.2528	966.5142	483.7608	L	***2426.1906***	1213.5989	2409.1641	1205.0857	2408.1801	1204.5937	21
10	1112.5834	556.7953	1095.5568	548.2821	1094.5728	547.7900	Q	***2313.1066***	1157.0569	2296.0800	1148.5436	2295.0960	1148.0516	20
11	1183.6205	592.3139	1166.5939	583.8006	1165.6099	583.3086	A	***2185.0480***	1093.0276	2168.0214	1084.5144	2167.0374	1084.0223	19
12	1311.6791	656.3432	1294.6525	647.8299	1293.6685	647.3379	Q	***2114.0109***	1057.5091	2096.9843	1048.9958	2096.0003	1048.5038	18
13	1448.7380	724.8726	1431.7114	716.3594	1430.7274	715.8673	H	***1985.9523***	993.4798	1968.9257	984.9665	1967.9417	984.4745	17
14	1562.7809	781.8941	1545.7544	773.3808	1544.7703	772.8888	N	***1848.8934***	924.9503	1831.8668	916.4371	1830.8828	915.9450	16
15	1675.8650	838.4361	1658.8384	829.9229	1657.8544	829.4308	L	***1734.8504***	867.9289	1717.8239	859.4156	1716.8399	858.9236	15
16	1831.9661	916.4867	1814.9395	907.9734	1813.9555	907.4814	R	1621.7664	811.3868	1604.7398	802.8736	1603.7558	802.3815	14
17	1946.9930	974.0002	1929.9665	965.4869	1928.9825	964.9949	D	***1465.6653***	733.3363	1448.6387	724.8230	1447.6547	724.3310	13
18	2034.0251	1017.5162	2016.9985	1009.0029	2016.0145	1008.5109	S	***1350.6383***	675.8228	1333.6118	667.3095	1332.6278	666.8175	12
19	2147.1091	1074.0582	2130.0826	1065.5449	2129.0986	1065.0529	L	1263.6063	632.3068	1246.5798	623.7935	1245.5957	623.3015	11
20	2276.1517	1138.5795	2259.1252	1130.0662	2258.1412	1129.5742	E	1150.5222	575.7648	1133.4957	567.2515	1132.5117	566.7595	10
21	2391.1787	1196.0930	2374.1521	1187.5797	2373.1681	1187.0877	N	***1021.4796***	511.2435	1004.4531	502.7302	1003.4691	502.2382	9
22	2492.2263	1246.6168	2475.1998	1238.1035	2474.2158	1237.6115	T	***906.4527***	453.7300	889.4262	445.2167	888.4421	444.7247	8
23	2605.3104	1303.1588	2588.2839	1294.6456	2587.2998	1294.1536	L	***805.4050***	403.2061	788.3785	394.6929	787.3945	394.2009	7
24	2706.3581	1353.6827	2689.3315	1345.1694	2688.3475	1344.6774	T	***692.3210***	346.6641	675.2944	338.1508	674.3104	337.6588	6
25	2835.4007	1418.2040	2818.3741	1409.6907	2817.3901	1409.1987	E	***591.2733***	296.1403	574.2467	287.6270	573.2627	287.1350	5
26	2922.4327	1461.7200	2905.4062	1453.2067	2904.4221	1452.7147	S	***462.2307***	231.6190	445.2041	223.1057	444.2201	222.6137	4

**27**	3051.4753	1526.2413	3034.4487	1517.7280	3033.4647	1517.2360	**E**	375.1987	188.1030	358.1721	179.5897	357.1881	179.0977	**3**
**28**	3122.5124	1561.7598	3105.4859	1553.2466	3104.5018	1552.7546	**A**	***246.1561***	123.5817	229.1295	115.0684			**2**
**29**							**R**	175.1190	88.0631	158.0924	79.5498			**1**

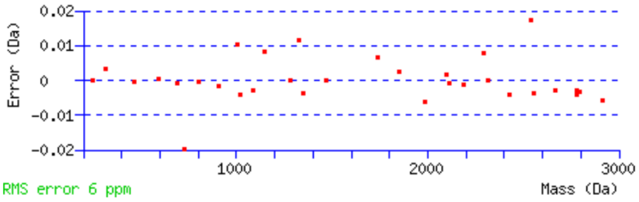

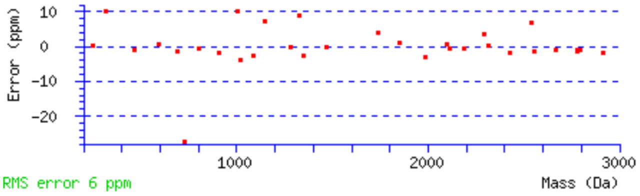

NCBI **BLAST** search of TVNALEIELQAQHNLRDSLENTLTESEAR
(Parameters: blastp, nr protein database, expect=20000, no filter, PAM30)
Other BLAST web gateways

All matches to this query

Score	Mr(calc):	Delta	Sequence
111.7	3295.6167	2.0212	TVNALEIELQAQHNLRDSLENTLTESEAR
79.1	3295.6167	2.0212	TVNALEIELQAQHNLRDSLENTLTESEAR
66.5	3295.6167	2.0212	TVNALEIELQAQHNLRDSLENTLTESEAR
58.1	3295.6167	2.0212	TVNALEIELQAQHNLRDSLENTLTESEAR
47.1	3295.6167	2.0212	TVNALEIELQAQHNLRDSLENTLTESEAR
39.8	3295.6167	2.0212	TVNALEIELQAQHNLRDSLENTLTESEAR
38.4	3295.6167	2.0212	TVNALEIELQAQHNLRDSLENTLTESEAR
30.7	3295.6167	2.0212	TVNALEIELQAQHNLRDSLENTLTESEAR
24.6	3295.6167	2.0212	TVNALEIELQAQHNLRDSLENTLTESEAR
24.5	3295.6167	2.0212	TVNALEIELQAQHNLRDSLENTLTESEAR

Mascot: http://www.matrixscience.com/

Mascot Search Results

Peptide View

MS/MS Fragmentation of **TVNALEIELQAQHNLRDSLENTLTESEAR**
Found in **sp|Q15323|K1H1_HUMAN**, Keratin, type I cuticular Ha1 OS=Homo sapiens GN=KRT31 PE=2 SV=3

Match to Query 41513: 3297.637896 from(825.416750,4+) intensity(2217478.2500) scans(14199) rtinseconds(2569) index(26491)
Title: 160219_Sunil_SDSI_A_Spectrum079797_scans__14199_RTINSECONDS=2569
Data file C:\\Sunil\\TKAP\\T\\T160219_Sunil_SDSI_A.mgf

Click mouse within plot area to zoom in by factor of two about that point
Or, Plot from 200 to 3200 Da Full range
Label all possible matches Label matches used for scoring

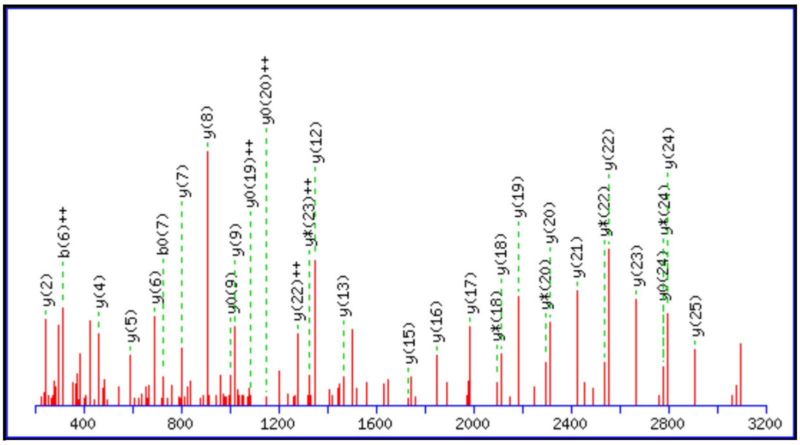

Monoisotopic mass of neutral peptide Mr(calc): 3295.6167
Fixed modifications: Carbamidomethyl (C) (apply to specified residues or termini only)
Variable modifications:
N3 : Deamidated (NQ)
N21 : Deamidated (NQ)
Ions Score: 112 **Expect:** 1.8e-009
Matches : 32/328 fragment ions using 72 most intense peaks (help)

#	b	b++	b*	b*++	b0	b0++	Seq.	y	y++	y*	y*++	y0	y0++	#
1	102.0550	51.5311			84.0444	42.5258	T							29
2	201.1234	101.0653			183.1128	92.0600	V	3195.5764	1598.2918	3178.5499	1589.7786	3177.5658	1589.2866	28
3	316.1503	158.5788	299.1238	150.0655	298.1397	149.5735	N	3096.5080	1548.7576	3079.4814	1540.2444	3078.4974	1539.7524	27
4	387.1874	194.0974	370.1609	185.5841	369.1769	185.0921	A	2981.4810	1491.2442	2964.4545	1482.7309	2963.4705	1482.2389	26
5	500.2715	250.6394	483.2449	242.1261	482.2609	241.6341	L	2910.4439	1455.7256	2893.4174	1447.2123	2892.4334	1446.7203	25
6	629.3141	315.1607	612.2875	306.6474	611.3035	306.1554	E	2797.3599	1399.1836	2780.3333	1390.6703	2779.3493	1390.1783	24
7	742.3981	371.7027	725.3716	363.1894	724.3876	362.6974	I	2668.3173	1334.6623	2651.2907	1326.1490	2650.3067	1325.6570	23
8	871.4407	436.2240	854.4142	427.7107	853.4302	427.2187	E	2555.2332	1278.1202	2538.2067	1269.6070	2537.2226	1269.1150	22
9	984.5248	492.7660	967.4983	484.2528	966.5142	483.7608	L	2426.1906	1213.5989	2409.1641	1205.0857	2408.1801	1204.5937	21
10	1112.5834	556.7953	1095.5568	548.2821	1094.5728	547.7900	Q	2313.1066	1157.0569	2296.0800	1148.5436	2295.0960	1148.0516	20
11	1183.6205	592.3139	1166.5939	583.8006	1165.6099	583.3086	A	2185.0480	1093.0276	2168.0214	1084.5144	2167.0374	1084.0223	19
12	1311.6791	656.3432	1294.6525	647.8299	1293.6685	647.3379	Q	2114.0109	1057.5091	2096.9843	1048.9958	2096.0003	1048.5038	18
13	1448.7380	724.8726	1431.7114	716.3594	1430.7274	715.8673	H	1985.9523	993.4798	1968.9257	984.9665	1967.9417	984.4745	17
14	1562.7809	781.8941	1545.7544	773.3808	1544.7703	772.8888	N	1848.8934	924.9503	1831.8668	916.4371	1830.8828	915.9450	16
15	1675.8650	838.4361	1658.8384	829.9229	1657.8544	829.4308	L	1734.8504	867.9289	1717.8239	859.4156	1716.8399	858.9236	15
16	1831.9661	916.4867	1814.9395	907.9734	1813.9555	907.4814	R	1621.7664	811.3868	1604.7398	802.8736	1603.7558	802.3815	14
17	1946.9930	974.0002	1929.9665	965.4869	1928.9825	964.9949	D	1465.6653	733.3363	1448.6387	724.8230	1447.6547	724.3310	13
18	2034.0251	1017.5162	2016.9985	1009.0029	2016.0145	1008.5109	S	1350.6383	675.8228	1333.6118	667.3095	1332.6278	666.8175	12
19	2147.1091	1074.0582	2130.0826	1065.5449	2129.0986	1065.0529	L	1263.6063	632.3068	1246.5798	623.7935	1245.5957	623.3015	11
20	2276.1517	1138.5795	2259.1252	1130.0662	2258.1412	1129.5742	E	1150.5222	575.7648	1133.4957	567.2515	1132.5117	566.7595	10
21	2391.1787	1196.0930	2374.1521	1187.5797	2373.1681	1187.0877	N	1021.4796	511.2435	1004.4531	502.7302	1003.4691	502.2382	9
22	2492.2263	1246.6168	2475.1998	1238.1035	2474.2158	1237.6115	T	906.4527	453.7300	889.4262	445.2167	888.4421	444.7247	8
23	2605.3104	1303.1588	2588.2839	1294.6456	2587.2998	1294.1536	L	805.4050	403.2061	788.3785	394.6929	787.3945	394.2009	7
24	2706.3581	1353.6827	2689.3315	1345.1694	2688.3475	1344.6774	T	692.3210	346.6641	675.2944	338.1508	674.3104	337.6588	6
25	2835.4007	1418.2040	2818.3741	1409.6907	2817.3901	1409.1987	E	591.2733	296.1403	574.2467	287.6270	573.2627	287.1350	5
26	2922.4327	1461.7200	2905.4062	1453.2067	2904.4221	1452.7147	S	462.2307	231.6190	445.2041	223.1057	444.2201	222.6137	4

**27**	3051.4753	1526.2413	3034.4487	1517.7280	3033.4647	1517.2360	**E**	375.1987	188.1030	358.1721	179.5897	357.1881	179.0977	**3**
**28**	3122.5124	1561.7598	3105.4859	1553.2466	3104.5018	1552.7546	**A**	***246.1561***	123.5817	229.1295	115.0684			**2**
**29**							**R**	175.1190	88.0631	158.0924	79.5498			**1**

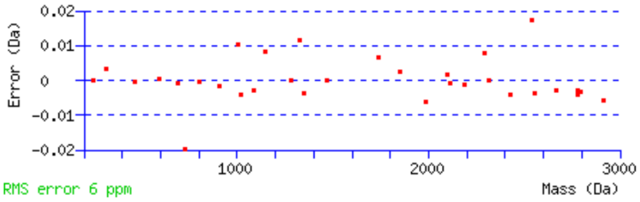

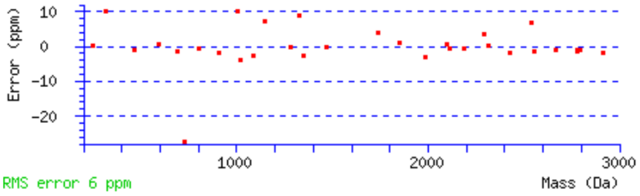

NCBI **BLAST** search of TVNALEIELQAQHNLRDSLENTLTESEAR
(Parameters: blastp, nr protein database, expect=20000, no filter, PAM30)
Other BLAST web gateways

All matches to this query

Score	Mr(calc):	Delta	Sequence
111.7	3295.6167	2.0212	TVNALEIELQAQHNLRDSLENTLTESEAR
79.1	3295.6167	2.0212	TVNALEIELQAQHNLRDSLENTLTESEAR
66.5	3295.6167	2.0212	TVNALEIELQAQHNLRDSLENTLTESEAR
58.1	3295.6167	2.0212	TVNALEIELQAQHNLRDSLENTLTESEAR
47.1	3295.6167	2.0212	TVNALEIELQAQHNLRDSLENTLTESEAR
39.8	3295.6167	2.0212	TVNALEIELQAQHNLRDSLENTLTESEAR
38.4	3295.6167	2.0212	TVNALEIELQAQHNLRDSLENTLTESEAR
30.7	3295.6167	2.0212	TVNALEIELQAQHNLRDSLENTLTESEAR
24.6	3295.6167	2.0212	TVNALEIELQAQHNLRDSLENTLTESEAR
24.5	3295.6167	2.0212	TVNALEIELQAQHNLRDSLENTLTESEAR

Mascot: http://www.matrixscience.com/

Mascot Search Results

Peptide View

MS/MS Fragmentation of **VLNETRSQYEALVETNRR**
Found in **sp|Q15323|K1H1_HUMAN**, Keratin, type I cuticular Ha1 OS=Homo sapiens GN=KRT31 PE=2 SV=3

Match to Query 32147: 2178.099402 from(727.040410,3+) intensity(944077.1250) scans(8719) rtinseconds(1650) index(37187)
Title: 160219_Sunil_SDSI_A_Spectrum091772_scans__8719_RTINSECONDS=1650
Data file C:\\Sunil\\TKAP\\T\\T160219_Sunil_SDSI_A.mgf

Click mouse within plot area to zoom in by factor of two about that point
Or, Plot from 0 to 2200 Da Full range
Label all possible matches ○ Label matches used for scoring ◉

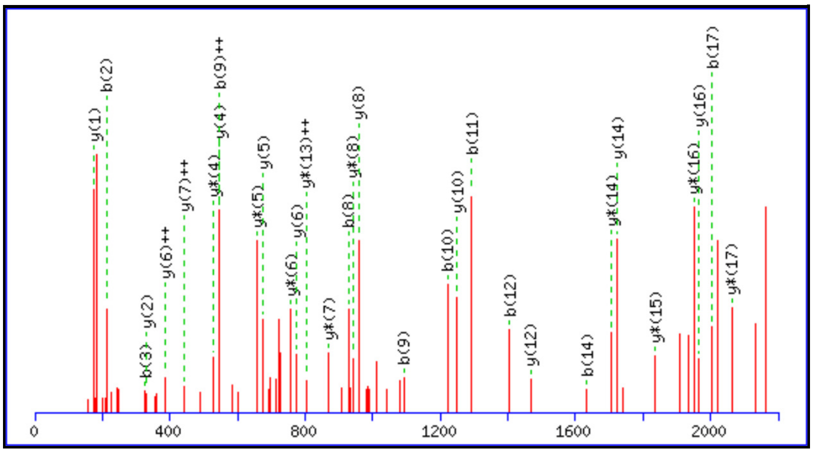

Monoisotopic mass of neutral peptide Mr(calc): 2178.1029
Fixed modifications: Carbamidomethyl (C) (apply to specified residues or termini only)
Variable modifications:
Q8 : Deamidated (NQ)
Ions Score: 78 **Expect:** 4.1e-006
Matches : 32/188 fragment ions using 46 most intense peaks (help)

#	b	b++	b*	b*++	b0	b0++	Seq.	y	y++	y*	y*++	y0	y0++	#
1	100.0757	50.5415					V							18
2	***213.1598***	107.0835					L	2080.0418	1040.5245	2063.0152	1032.0112	2062.0312	1031.5192	17
3	***327.2027***	164.1050	310.1761	155.5917			N	***1966.9577***	983.9825	1949.9312	975.4692	1948.9471	974.9772	16
4	456.2453	228.6263	439.2187	220.1130	438.2347	219.6210	E	1852.9148	926.9610	1835.8882	918.4478	1834.9042	917.9557	15
5	557.2930	279.1501	540.2664	270.6368	539.2824	270.1448	T	***1723.8722***	862.4397	1706.8456	853.9265	1705.8616	853.4344	14
6	713.3941	357.2007	696.3675	348.6874	695.3835	348.1954	R	1622.8245	811.9159	1605.7980	803.4026	1604.8139	802.9106	13
7	800.4261	400.7167	783.3995	392.2034	782.4155	391.7114	S	***1466.7234***	733.8653	1449.6968	725.3521	1448.7128	724.8601	12
8	***929.4687***	465.2380	912.4421	456.7247	911.4581	456.2327	Q	1379.6914	690.3493	1362.6648	681.8360	1361.6808	681.3440	11
9	***1092.5320***	**546.7696**	1075.5055	538.2564	1074.5215	537.7644	Y	***1250.6488***	625.8280	1233.6222	617.3148	1232.6382	616.8227	10
10	***1221.5746***	611.2909	1204.5481	602.7777	1203.5640	602.2857	E	1087.5854	544.2964	1070.5589	535.7831	1069.5749	535.2911	9
11	***1292.6117***	646.8095	1275.5852	638.2962	1274.6012	637.8042	A	***958.5429***	479.7751	941.5163	471.2618	940.5323	470.7698	8
12	***1405.6958***	703.3515	1388.6692	694.8383	1387.6852	694.3462	L	887.5057	444.2565	870.4792	435.7432	869.4952	435.2512	7
13	1504.7642	752.8857	1487.7377	744.3725	1486.7536	743.8805	V	***774.4217***	387.7145	757.3951	379.2012	756.4111	378.7092	6
14	***1633.8068***	817.4070	1616.7802	808.8938	1615.7962	808.4018	E	***675.3533***	338.1803	658.3267	329.6670	657.3427	329.1750	5
15	1734.8545	867.9309	1717.8279	859.4176	1716.8439	858.9256	T	***546.3107***	273.6590	529.2841	265.1457	528.3001	264.6537	4
16	1848.8974	924.9523	1831.8709	916.4391	1830.8868	915.9471	N	445.2630	223.1351	428.2364	214.6219			3
17	***2004.9985***	1003.0029	1987.9720	994.4896	1986.9879	993.9976	R	***331.2201***	166.1137	314.1935	157.6004			2
18							R	***175.1190***	88.0631	158.0924	79.5498			1

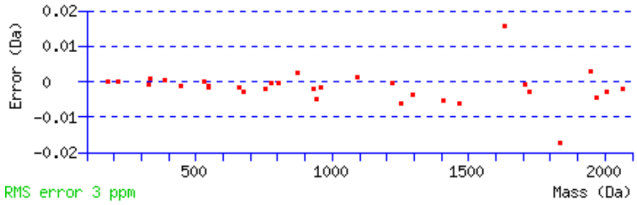

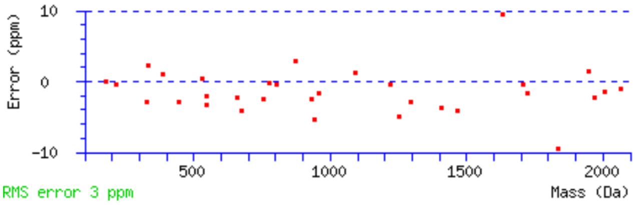

NCBI **BLAST** search of VLNETRSQYEALVETNRR
(Parameters: blastp, nr protein database, expect=20000, no filter, PAM30)
Other BLAST web gateways

All matches to this query

Score	Mr(calc):	Delta	Sequence
78.1	2178.1029	-0.0035	VLNETRSQYEALVETNRR
47.1	2178.1029	-0.0035	VLNETRSQYEALVETNRR
11.2	2178.1029	-0.0035	VLNETRSQYEALVETNRR
0.3	2178.1168	-0.0174	VLDQQHILQVQSLQLQER
0.3	2178.1168	-0.0174	VLDQQHILQVQSLQLQER
0.3	2178.1168	-0.0174	VLDQQHILQVQSLQLQER

Mascot: http://www.matrixscience.com/

Mascot Search Results

Peptide View

MS/MS Fragmentation of **VLNETRSQYEALVETNRR**
Found in **sp|Q15323|K1H1_HUMAN**, Keratin, type I cuticular Ha1 OS=Homo sapiens GN=KRT31 PE=2 SV=3

Match to Query 32162: 2178.103696 from(545.533200,4+) intensity(3993332.0000) scans(9603) rtinseconds(1780) index(22725)
Title: 160219_Sunil_SDSI_A_Spectrum076029_scans__9603_RTINSECONDS=1780
Data file C:\\Sunil\\TKAP\\T\\T160219_Sunil_SDSI_A.mgf

Click mouse within plot area to zoom in by factor of two about that point
Or, Plot from 0 to 2200 Da Full range
Label all possible matches Label matches used for scoring

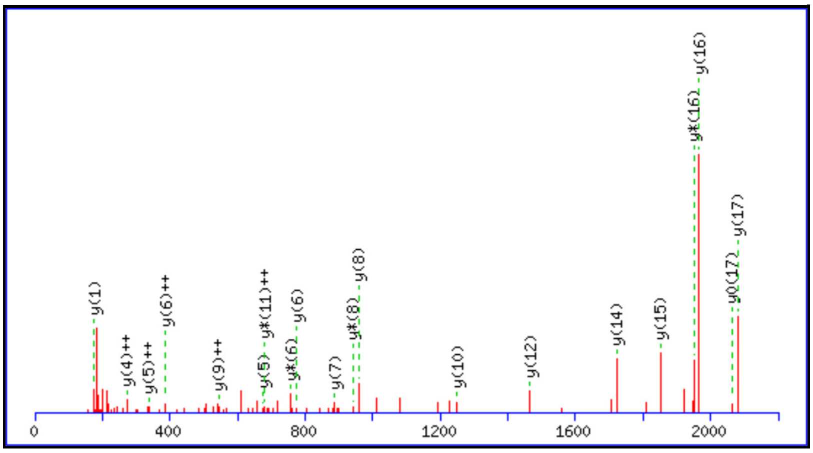

Monoisotopic mass of neutral peptide Mr(calc): 2178.1029
Fixed modifications: Carbamidomethyl (C) (apply to specified residues or termini only)
Variable modifications:
N3 : Deamidated (NQ)
Ions Score: 60 **Expect:** 0.00024
Matches : 20/188 fragment ions using 42 most intense peaks (help)

#	b	b^{++}	b*	b^{*++}	b^0	b^{0++}	Seq.	y	y^{++}	y*	y^{*++}	y^0	y^{0++}	#
1	100.0757	50.5415					V							18
2	213.1598	107.0835					L	***2080.0418***	1040.5245	2063.0152	1032.0112	2062.0312	1031.5192	17
3	328.1867	164.5970	311.1601	156.0837			N	***1966.9577***	983.9825	1949.9312	975.4692	1948.9471	974.9772	16
4	457.2293	229.1183	440.2027	220.6050	439.2187	220.1130	E	***1851.9308***	926.4690	1834.9042	917.9557	1833.9202	917.4637	15
5	558.2770	279.6421	541.2504	271.1288	540.2664	270.6368	T	***1722.8882***	861.9477	1705.8616	853.4344	1704.8776	852.9424	14
6	714.3781	357.6927	697.3515	349.1794	696.3675	348.6874	R	1621.8405	811.4239	1604.8139	802.9106	1603.8299	802.4186	13
7	801.4101	401.2087	784.3836	392.6954	783.3995	392.2034	S	***1465.7394***	733.3733	1448.7128	724.8601	1447.7288	724.3680	12
8	929.4687	465.2380	912.4421	456.7247	911.4581	456.2327	Q	1378.7074	689.8573	1361.6808	681.3440	1360.6968	680.8520	11
9	1092.5320	546.7696	1075.5055	538.2564	1074.5215	537.7644	Y	***1250.6488***	625.8280	1233.6222	617.3148	1232.6382	616.8227	10
10	1221.5746	611.2909	1204.5481	602.7777	1203.5640	602.2857	E	1087.5854	544.2964	1070.5589	535.7831	1069.5749	535.2911	9
11	1292.6117	646.8095	1275.5852	638.2962	1274.6012	637.8042	A	***958.5429***	479.7751	941.5163	471.2618	940.5323	470.7698	8
12	1405.6958	703.3515	1388.6692	694.8383	1387.6852	694.3462	L	***887.5057***	444.2565	870.4792	435.7432	869.4952	435.2512	7
13	1504.7642	752.8857	1487.7377	744.3725	1486.7536	743.8805	V	***774.4217***	387.7145	757.3951	379.2012	756.4111	378.7092	6
14	1633.8068	817.4070	1616.7802	808.8938	1615.7962	808.4018	E	***675.3533***	338.1803	658.3267	329.6670	657.3427	329.1750	5
15	1734.8545	867.9309	1717.8279	859.4176	1716.8439	858.9256	T	546.3107	273.6590	529.2841	265.1457	528.3001	264.6537	4
16	1848.8974	924.9523	1831.8709	916.4391	1830.8868	915.9471	N	445.2630	223.1351	428.2364	214.6219			3
17	2004.9985	1003.0029	1987.9720	994.4896	1986.9879	993.9976	R	331.2201	166.1137	314.1935	157.6004			2
18							R	***175.1190***	88.0631	158.0924	79.5498			1

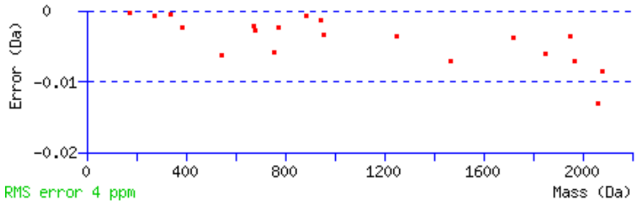

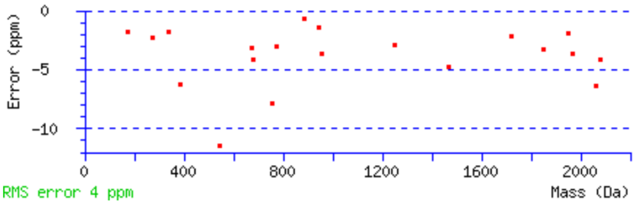

NCBI **BLAST** search of VLNETRSQYEALVETNRR

(Parameters: blastp, nr protein database, expect=20000, no filter, PAM30)
Other BLAST web gateways

All matches to this query

Score	Mr(calc):	Delta	Sequence
60.3	2178.1029	0.0008	VLNETRSQYEALVETNRR
34.6	2177.1189	0.9848	VLNETRSQYEALVETNRR
31.7	2178.1029	0.0008	VLNETRSQYEALVETNRR
3.5	2178.0999	0.0038	VLDKLTLCMCPERPFTAK
3.1	2178.1141	-0.0104	VLQDASNSIDHRIPNSQRK
1.3	2178.1029	0.0008	VLNETRSQYEALVETNRR

Mascot: http://www.matrixscience.com/

Mascot Search Results

Peptide View

MS/MS Fragmentation of **VLNETRSQYEALVETNRR**
Found in **sp|Q15323|K1H1_HUMAN**, Keratin, type I cuticular Ha1 OS=Homo sapiens GN=KRT31 PE=2 SV=3

Match to Query 32196: 2179.090722 from(727.370850,3+) intensity(1840300.0000) scans(10052) rtinseconds(1857) index(23107)
Title: 160219_Sunil_SDSI_A_Spectrum076411_scans__10052_RTINSECONDS=1857
Data file C:\\Sunil\\TKAP\\T\\T160219_Sunil_SDSI_A.mgf

Click mouse within plot area to zoom in by factor of two about that point
Or, Plot from 0 to 2200 Da Full range
Label all possible matches ○ Label matches used for scoring ●

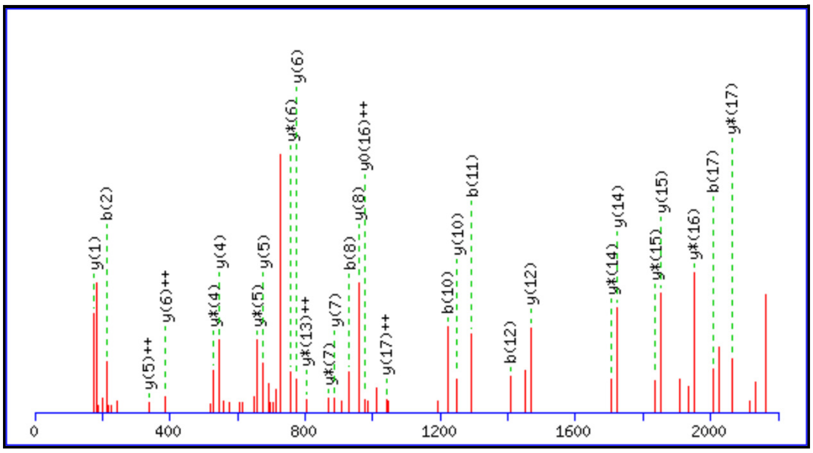

Monoisotopic mass of neutral peptide Mr(calc): 2179.0869
Fixed modifications: Carbamidomethyl (C) (apply to specified residues or termini only)
Variable modifications:
N3 : Deamidated (NQ)
Q8 : Deamidated (NQ)
Ions Score: 67 **Expect:** 5.5e-005
Matches : 29/188 fragment ions using 44 most intense peaks (help)

#	b	b++	b*	b*++	b0	b0++	Seq.	y	y++	y*	y*++	y0	y0++	#
1	100.0757	50.5415					V							18
2	**213.1598**	107.0835					L	2081.0258	**1041.0165**	**2063.9992**	1032.5033	2063.0152	1032.0112	17
3	328.1867	164.5970	311.1601	156.0837			N	1967.9417	984.4745	**1950.9152**	975.9612	1949.9312	**975.4692**	16
4	457.2293	229.1183	440.2027	220.6050	439.2187	220.1130	E	***1852.9148***	926.9610	**1835.8882**	918.4478	1834.9042	917.9557	15
5	558.2770	279.6421	541.2504	271.1288	540.2664	270.6368	T	***1723.8722***	862.4397	**1706.8456**	853.9265	1705.8616	853.4344	14
6	714.3781	357.6927	697.3515	349.1794	696.3675	348.6874	R	1622.8245	811.9159	1605.7980	**803.4026**	1604.8139	802.9106	13
7	801.4101	401.2087	784.3836	392.6954	783.3995	392.2034	S	***1466.7234***	733.8653	1449.6968	725.3521	1448.7128	724.8601	12
8	**930.4527**	465.7300	913.4262	457.2167	912.4421	456.7247	Q	1379.6914	690.3493	1362.6648	681.8360	1361.6808	681.3440	11
9	1093.5160	547.2617	1076.4895	538.7484	1075.5055	538.2564	Y	***1250.6488***	625.8280	1233.6222	617.3148	1232.6382	616.8227	10
10	**1222.5586**	611.7830	1205.5321	603.2697	1204.5481	602.7777	E	1087.5854	544.2964	1070.5589	535.7831	1069.5749	535.2911	9
11	**1293.5957**	647.3015	1276.5692	638.7882	1275.5852	638.2962	A	***958.5429***	479.7751	941.5163	471.2618	940.5323	470.7698	8
12	**1406.6798**	703.8435	1389.6533	695.3303	1388.6692	694.8383	L	***887.5057***	444.2565	**870.4792**	435.7432	869.4952	435.2512	7
13	1505.7482	753.3777	1488.7217	744.8645	1487.7377	744.3725	V	***774.4217***	**387.7145**	**757.3951**	379.2012	756.4111	378.7092	6
14	1634.7908	817.8990	1617.7643	809.3858	1616.7802	808.8938	E	***675.3533***	**338.1803**	**658.3267**	329.6670	657.3427	329.1750	5
15	1735.8385	868.4229	1718.8119	859.9096	1717.8279	859.4176	T	***546.3107***	273.6590	**529.2841**	265.1457	528.3001	264.6537	4
16	1849.8814	925.4443	1832.8549	916.9311	1831.8708	916.4391	N	445.2630	223.1351	428.2364	214.6219			3
17	**2005.9825**	1003.4949	1988.9560	994.9816	1987.9720	994.4896	R	331.2201	166.1137	314.1935	157.6004			2
18							R	***175.1190***	88.0631	158.0924	79.5498			1

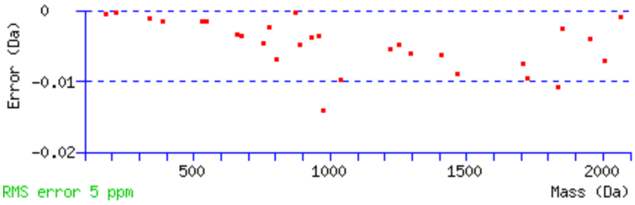

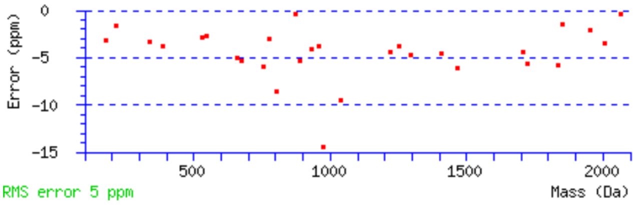

NCBI **BLAST** search of VLNETRSQYEALVETNRR
(Parameters: blastp, nr protein database, expect=20000, no filter, PAM30)
Other BLAST web gateways

All matches to this query

Score	Mr(calc):	Delta	Sequence
67.0	2179.0869	0.0039	VLNETRSQYEALVETNRR
53.8	2178.1029	0.9879	VLNETRSQYEALVETNRR
21.0	2178.1029	0.9879	VLNETRSQYEALVETNRR
14.8	2179.0869	0.0039	VLNETRSQYEALVETNRR
6.1	2179.0869	0.0039	VLNETRSQYEALVETNRR
5.5	2178.1029	0.9879	VLNETRSQYEALVETNRR
0.9	2179.0757	0.0151	TPDHEIQGSKEALIQDLER

Mascot: http://www.matrixscience.com/

Mascot Search Results

Peptide View

MS/MS Fragmentation of **VLQSHISDTSVVVKLDNSR**
Found in **sp|O43790|KRT86_HUMAN**, Keratin, type II cuticular Hb6 OS=Homo sapiens GN=KRT86 PE=1 SV=1

Match to Query 30657: 2097.106332 from(700.042720,3+) intensity(1151934.0000) scans(9804) rtinseconds(1839) index(7511)
Title: 160219_Sunil_SDSI_A_Spectrum059619_scans__9804_RTINSECONDS=1839
Data file C:\\Sunil\\TKAP\\T\\T160219_Sunil_SDSI_A.mgf

Click mouse within plot area to zoom in by factor of two about that point
Or, Plot from 100 to 1900 Da Full range
Label all possible matches ○ Label matches used for scoring ◉

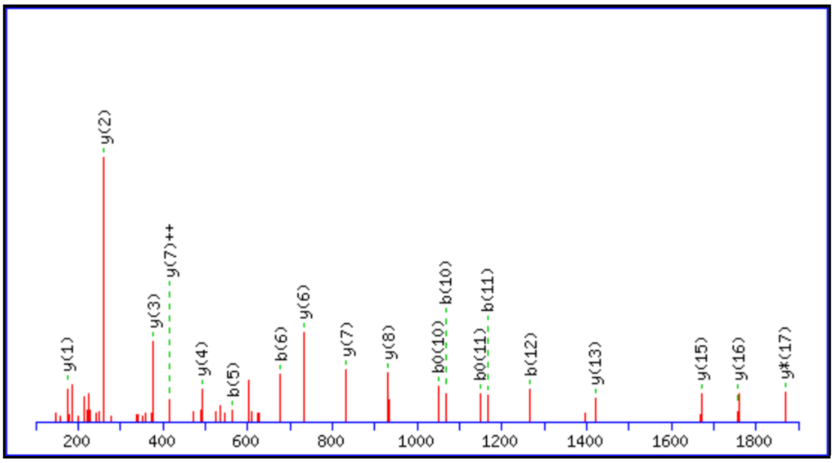

Monoisotopic mass of neutral peptide Mr(calc): 2097.1066
Fixed modifications: Carbamidomethyl (C) (apply to specified residues or termini only)
Variable modifications:
N17 : Deamidated (NQ)
Ions Score: 73 **Expect:** 1.1e-005
Matches : 19/204 fragment ions using 28 most intense peaks (help)

#	b	b^{++}	b^{*}	b^{*++}	b^{0}	b^{0++}	Seq.	y	y^{++}	y^{*}	y^{*++}	y^{0}	y^{0++}	#
1	100.0757	50.5415					V							19
2	213.1598	107.0835					L	1999.0455	1000.0264	1982.0189	991.5131	1981.0349	991.0211	18
3	341.2183	171.1128	324.1918	162.5995			Q	1885.9614	943.4843	1868.9348	934.9711	1867.9508	934.4791	17
4	428.2504	214.6288	411.2238	206.1155	410.2398	205.6235	S	1757.9028	879.4550	1740.8763	870.9418	1739.8923	870.4498	16
5	565.3093	283.1583	548.2827	274.6450	547.2987	274.1530	H	1670.8708	835.9390	1653.8442	827.4258	1652.8602	826.9338	15
6	678.3933	339.7003	661.3668	331.1870	660.3828	330.6950	I	1533.8119	767.4096	1516.7853	758.8963	1515.8013	758.4043	14
7	765.4254	383.2163	748.3988	374.7030	747.4148	374.2110	S	1420.7278	710.8675	1403.7013	702.3543	1402.7172	701.8623	13
8	880.4523	440.7298	863.4258	432.2165	862.4417	431.7245	D	1333.6958	667.3515	1316.6692	658.8383	1315.6852	658.3462	12
9	981.5000	491.2536	964.4734	482.7404	963.4894	482.2483	T	1218.6688	609.8381	1201.6423	601.3248	1200.6583	600.8328	11
10	1068.5320	534.7696	1051.5055	526.2564	1050.5214	525.7644	S	1117.6212	559.3142	1100.5946	550.8009	1099.6106	550.3089	10
11	1167.6004	584.3039	1150.5739	575.7906	1149.5899	575.2986	V	1030.5891	515.7982	1013.5626	507.2849	1012.5786	506.7929	9
12	1266.6688	633.8381	1249.6423	625.3248	1248.6583	624.8328	V	931.5207	466.2640	914.4942	457.7507	913.5102	457.2587	8
13	1365.7373	683.3723	1348.7107	674.8590	1347.7267	674.3670	V	832.4523	416.7298	815.4258	408.2165	814.4417	407.7245	7
14	1493.8322	747.4197	1476.8057	738.9065	1475.8217	738.4145	K	733.3839	367.1956	716.3573	358.6823	715.3733	358.1903	6
15	1606.9163	803.9618	1589.8897	795.4485	1588.9057	794.9565	L	605.2889	303.1481	588.2624	294.6348	587.2784	294.1428	5
16	1721.9432	861.4753	1704.9167	852.9620	1703.9327	852.4700	D	492.2049	246.6061	475.1783	238.0928	474.1943	237.6008	4
17	1836.9702	918.9887	1819.9436	910.4754	1818.9596	909.9834	N	377.1779	189.0926	360.1514	180.5793	359.1674	180.0873	3
18	1924.0022	962.5047	1906.9756	953.9915	1905.9916	953.4995	S	262.1510	131.5791	245.1244	123.0659	244.1404	122.5738	2
19							R	175.1190	88.0631	158.0924	79.5498			1

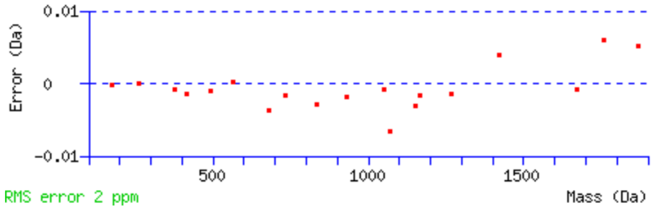

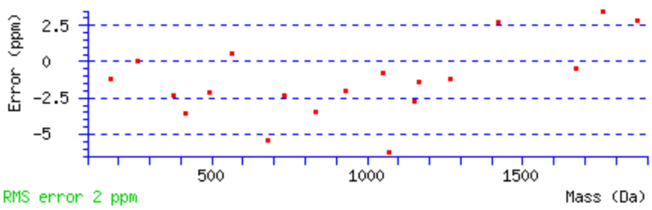

NCBI **BLAST** search of VLQSHISDTSVVVKLDNSR
(Parameters: blastp, nr protein database, expect=20000, no filter, PAM30)
Other BLAST web gateways

All matches to this query

Score	Mr(calc):	Delta	Sequence
72.7	2097.1066	-0.0003	VLQSHISDTSVVVKLDNSR
39.1	2096.1226	0.9837	VLQSHISDTSVVVKLDNSR
15.4	2097.1066	-0.0003	VLQSHISDTSVVVKLDNSR
1.9	2095.0983	2.0081	ALHLLAVLEKMISQGNNNK
1.9	2096.0823	1.0241	ALHLLAVLEKMISQGNNNK
1.9	2095.1023	2.0040	KMKVPYLSSLLQPFSDDK

Mascot: http://www.matrixscience.com/

Mascot Search Results

Peptide View

MS/MS Fragmentation of **VLQSHISDTSVVVKLDNSRDLNMDCIIAEIK**
Found in **sp|O43790|KRT86_HUMAN**, Keratin, type II cuticular Hb6 OS=Homo sapiens GN=KRT86 PE=1 SV=1

Match to Query 42689: 3512.779016 from(879.202030,4+) intensity(1045356.6875) scans(15463) rtinseconds(2810) index(12198)
Title: 160219_Sunil_SDSI_A_Spectrum064329_scans__15463_RTINSECONDS=2810
Data file C:\\Sunil\\TKAP\\T\\T160219_Sunil_SDSI_A.mgf

Click mouse within plot area to zoom in by factor of two about that point
Or, Plot from 200 to 3600 Da Full range
Label all possible matches Label matches used for scoring

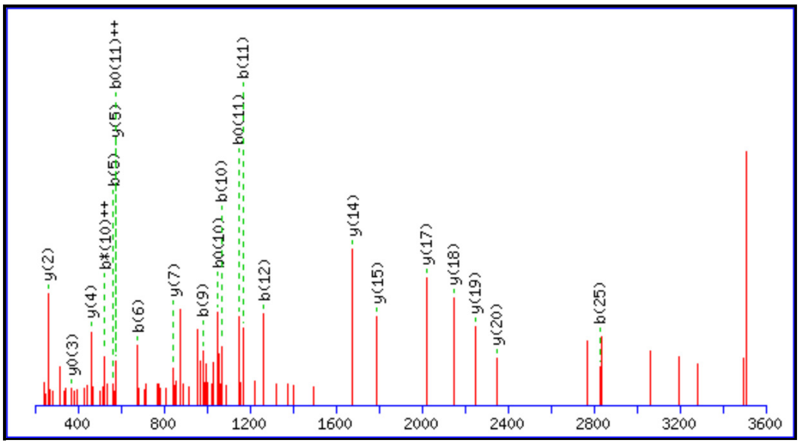

Monoisotopic mass of neutral peptide Mr(calc): 3512.7854
Fixed modifications: Carbamidomethyl (C) (apply to specified residues or termini only)
Variable modifications:
N17 : Deamidated (NQ)
Ions Score: 45 **Expect:** 0.007
Matches : 22/346 fragment ions using 49 most intense peaks (help)

#	b	b++	b*	b*++	b0	b0++	Seq.	y	y++	y*	y*++	y0	y0++	#
1	100.0757	50.5415					V							31
2	213.1598	107.0835					L	3414.7243	1707.8658	3397.6978	1699.3525	3396.7138	1698.8605	30
3	341.2183	171.1128	324.1918	162.5995			Q	3301.6403	1651.3238	3284.6137	1642.8105	3283.6297	1642.3185	29
4	428.2504	214.6288	411.2238	206.1155	410.2398	205.6235	S	3173.5817	1587.2945	3156.5551	1578.7812	3155.5711	1578.2892	28
5	***565.3093***	283.1583	548.2827	274.6450	547.2987	274.1530	H	3086.5497	1543.7785	3069.5231	1535.2652	3068.5391	1534.7732	27
6	***678.3933***	339.7003	661.3668	331.1870	660.3828	330.6950	I	2949.4908	1475.2490	2932.4642	1466.7357	2931.4802	1466.2437	26
7	765.4254	383.2163	748.3988	374.7030	747.4148	374.2110	S	2836.4067	1418.7070	2819.3801	1410.1937	2818.3961	1409.7017	25
8	880.4523	440.7298	863.4258	432.2165	862.4417	431.7245	D	2749.3747	1375.1910	2732.3481	1366.6777	2731.3641	1366.1857	24
9	***981.5000***	491.2536	964.4734	482.7404	963.4894	482.2483	T	2634.3477	1317.6775	2617.3212	1309.1642	2616.3372	1308.6722	23
10	***1068.5320***	534.7696	1051.5055	526.2564	1050.5214	525.7644	S	2533.3000	1267.1537	2516.2735	1258.6404	2515.2895	1258.1484	22
11	***1167.6004***	584.3039	1150.5739	575.7906	1149.5899	575.2986	V	2446.2680	1223.6376	2429.2415	1215.1244	2428.2574	1214.6324	21
12	***1266.6688***	633.8381	1249.6423	625.3248	1248.6583	624.8328	V	***2347.1996***	1174.1034	2330.1730	1165.5902	2329.1890	1165.0982	20
13	1365.7373	683.3723	1348.7107	674.8590	1347.7267	674.3670	V	***2248.1312***	1124.5692	2231.1046	1116.0560	2230.1206	1115.5639	19
14	1493.8322	747.4197	1476.8057	738.9065	1475.8217	738.4145	K	***2149.0628***	1075.0350	2132.0362	1066.5217	2131.0522	1066.0297	18
15	1606.9163	803.9618	1589.8897	795.4485	1588.9057	794.9565	L	***2020.9678***	1010.9875	2003.9413	1002.4743	2002.9572	1001.9823	17
16	1721.9432	861.4753	1704.9167	852.9620	1703.9327	852.4700	D	1907.8837	954.4455	1890.8572	945.9322	1889.8732	945.4402	16
17	1836.9702	918.9887	1819.9436	910.4754	1818.9596	909.9834	N	***1792.8568***	896.9320	1775.8302	888.4188	1774.8462	887.9268	15
18	1924.0022	962.5047	1906.9756	953.9915	1905.9916	953.4995	S	***1677.8299***	839.4186	1660.8033	830.9053	1659.8193	830.4133	14
19	2080.1033	1040.5553	2063.0768	1032.0420	2062.0927	1031.5500	R	1590.7978	795.9026	1573.7713	787.3893	1572.7873	786.8973	13
20	2195.1303	1098.0688	2178.1037	1089.5555	2177.1197	1089.0635	D	1434.6967	717.8520	1417.6702	709.3387	1416.6861	708.8467	12
21	2308.2143	1154.6108	2291.1878	1146.0975	2290.2038	1145.6055	L	1319.6698	660.3385	1302.6432	651.8252	1301.6592	651.3332	11
22	2422.2572	1211.6323	2405.2307	1203.1190	2404.2467	1202.6270	N	1206.5857	603.7965	1189.5592	595.2832	1188.5751	594.7912	10
23	2553.2977	1277.1525	2536.2712	1268.6392	2535.2872	1268.1472	M	1092.5428	546.7750	1075.5162	538.2618	1074.5322	537.7697	9
24	2668.3247	1334.6660	2651.2981	1326.1527	2650.3141	1325.6607	D	961.5023	481.2548	944.4757	472.7415	943.4917	472.2495	8
25	***2828.3553***	1414.6813	2811.3288	1406.1680	2810.3448	1405.6760	C	***846.4754***	423.7413	829.4488	415.2280	828.4648	414.7360	7
26	2941.4394	1471.2233	2924.4128	1462.7101	2923.4288	1462.2180	I	686.4447	343.7260	669.4182	335.2127	668.4341	334.7207	6
27	3054.5234	1527.7654	3037.4969	1519.2521	3036.5129	1518.7601	I	***573.3606***	287.1840	556.3341	278.6707	555.3501	278.1787	5

**28**	3125.5606	1563.2839	3108.5340	1554.7706	3107.5500	1554.2786	**A**	***460.2766***	230.6419	443.2500	222.1287	442.2660	221.6366	**4**
**29**	3254.6032	1627.8052	3237.5766	1619.2919	3236.5926	1618.7999	**E**	389.2395	195.1234	372.2129	186.6101	371.2289	186.1181	**3**
**30**	3367.6872	1684.3472	3350.6607	1675.8340	3349.6767	1675.3420	**I**	***260.1969***	130.6021	243.1703	122.0888			**2**
**31**							**K**	147.1128	74.0600	130.0863	65.5468			**1**

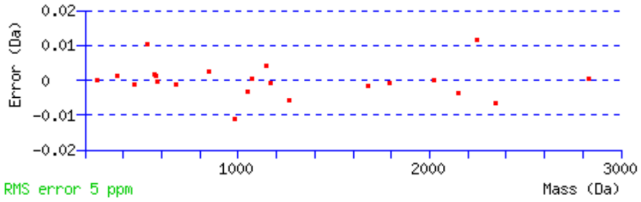

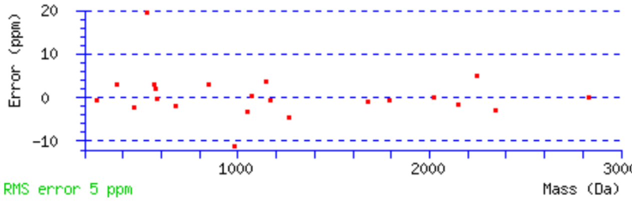

NCBI **BLAST** search of VLQSHISDTSVVVKLDNSRDLNMDCIIAEIK
(Parameters: blastp, nr protein database, expect=20000, no filter, PAM30)
Other BLAST web gateways

All matches to this query

Score	Mr(calc):	Delta	Sequence
44.8	3512.7854	-0.0064	VLQSHISDTSVVVKLDNSRDLNMDCIIAEIK
37.8	3512.7854	-0.0064	VLQSHISDTSVVVKLDNSRDLNMDCIIAEIK
15.1	3511.8014	0.9776	VLQSHISDTSVVVKLDNSRDLNMDCIIAEIK

Mascot: http://www.matrixscience.com/

Mascot Search Results

Peptide View

MS/MS Fragmentation of **VRQLERDNAELENLIR**
Found in **sp|Q15323|K1H1_HUMAN**, Keratin, type I cuticular Ha1 OS=Homo sapiens GN=KRT31 PE=2 SV=3

Match to Query 28043: 1968.048492 from(657.023440,3+) intensity(1886122.6250) scans(10651) rtinseconds(1960) index(23624)
Title: 160219_Sunil_SDSI_A_Spectrum076928_scans__10651_RTINSECONDS=1960
Data file C:\\Sunil\\TKAP\\T\\T160219_Sunil_SDSI_A.mgf

Click mouse within plot area to zoom in by factor of two about that point
Or, Plot from 100 to 2000 Da Full range
Label all possible matches ○ Label matches used for scoring ◉

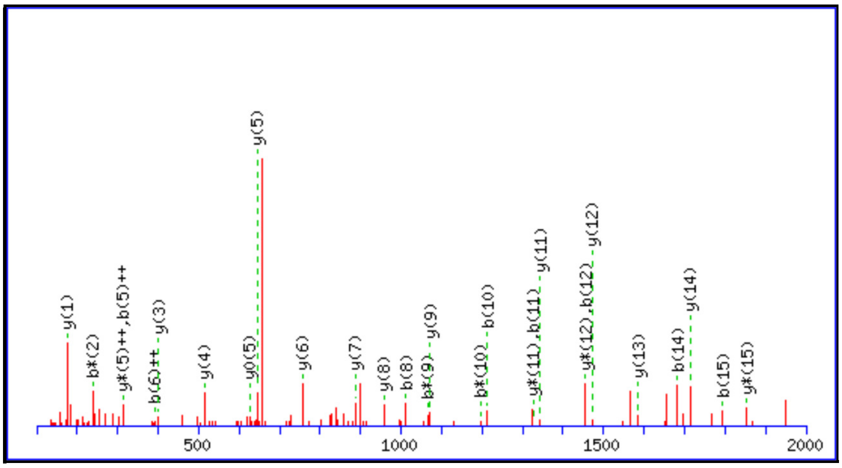

Monoisotopic mass of neutral peptide Mr(calc): 1968.0388
Fixed modifications: Carbamidomethyl (C) (apply to specified residues or termini only)
Variable modifications:
Q3 : Deamidated (NQ)
Ions Score: 98 **Expect:** 2.6e-008
Matches : 28/162 fragment ions using 35 most intense peaks (help)

#	b	b++	b*	b*++	b^0	b^{0++}	Seq.	y	y++	y*	y*++	y^0	y^{0++}	#
1	100.0757	50.5415					V							16
2	256.1768	128.5920	239.1503	120.0788			R	1869.9777	935.4925	1852.9512	926.9792	1851.9671	926.4872	15
3	385.2194	193.1133	368.1928	184.6001			Q	***1713.8766***	857.4419	1696.8501	848.9287	1695.8660	848.4367	14
4	498.3035	249.6554	481.2769	241.1421			L	***1584.8340***	792.9206	1567.8075	784.4074	1566.8234	783.9154	13
5	627.3461	**314.1767**	610.3195	305.6634	609.3355	305.1714	E	***1471.7499***	736.3786	1454.7234	727.8653	1453.7394	727.3733	12
6	783.4472	**392.2272**	766.4206	383.7139	765.4366	383.2219	R	***1342.7074***	671.8573	1325.6808	663.3440	1324.6968	662.8520	11
7	898.4741	449.7407	881.4476	441.2274	880.4635	440.7354	D	1186.6062	593.8068	1169.5797	585.2935	1168.5957	584.8015	10
8	**1012.5170**	506.7622	995.4905	498.2489	994.5065	497.7569	N	***1071.5793***	536.2933	1054.5527	527.7800	1053.5687	527.2880	9
9	1083.5541	542.2807	1066.5276	533.7674	1065.5436	533.2754	A	***957.5364***	479.2718	940.5098	470.7585	939.5258	470.2665	8
10	**1212.5967**	606.8020	1195.5702	598.2887	1194.5862	597.7967	E	***886.4993***	443.7533	869.4727	435.2400	868.4887	434.7480	7
11	**1325.6808**	663.3440	1308.6543	654.8308	1307.6702	654.3388	L	***757.4567***	379.2320	740.4301	370.7187	739.4461	370.2267	6
12	**1454.7234**	727.8653	1437.6968	719.3521	1436.7128	718.8601	E	***644.3726***	322.6899	627.3461	314.1767	626.3620	313.6847	5
13	1568.7663	784.8868	1551.7398	776.3735	1550.7558	775.8815	N	***515.3300***	258.1686	498.3035	249.6554			4
14	**1681.8504**	841.4288	1664.8238	832.9156	1663.8398	832.4235	L	***401.2871***	201.1472	384.2605	192.6339			3
15	**1794.9345**	897.9709	1777.9079	889.4576	1776.9239	888.9656	I	288.2030	144.6051	271.1765	136.0919			2
16							R	***175.1190***	88.0631	158.0924	79.5498			1

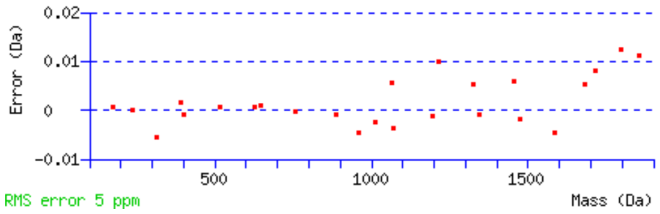

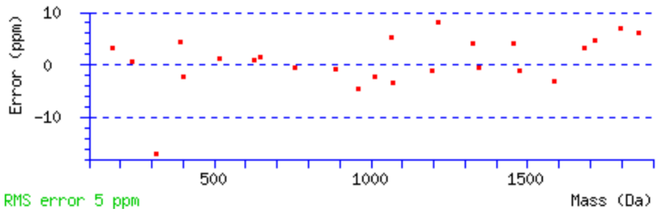

NCBI **BLAST** search of VRQLERDNAELENLIR

(Parameters: blastp, nr protein database, expect=20000, no filter, PAM30)
Other BLAST web gateways

All matches to this query

Score	Mr(calc):	Delta	Sequence
98.0	1968.0388	0.0097	VRQLERDNAELENLIR
82.5	1967.0548	0.9937	VRQLERDNAELENLIR
58.5	1968.0388	0.0097	VRQLERDNAELENLIR
10.1	1968.0388	0.0097	VRQLERDNAELENLIR
8.1	1968.0363	0.0122	RLEPFMVQPNPEARLR
5.6	1968.0540	-0.0056	REIEIQAHLQHPNILR

Mascot: http://www.matrixscience.com/

Mascot Search Results

Peptide View

MS/MS Fragmentation of **VRQLERDNAELENLIR**
Found in **sp|O76009|KT33A_HUMAN**, Keratin, type I cuticular Ha3-I OS=Homo sapiens GN=KRT33A PE=2 SV=2

Match to Query 28043: 1968.048492 from(657.023440,3+) intensity(1886122.6250) scans(10651) rtinseconds(1960) index(23624)
Title: 160219_Sunil_SDSI_A_Spectrum076928_scans__10651_RTINSECONDS=1960
Data file C:\\Sunil\\TKAP\\T\\T160219_Sunil_SDSI_A.mgf

Click mouse within plot area to zoom in by factor of two about that point
Or, Plot from 100 to 2000 Da Full range
Label all possible matches ○ Label matches used for scoring ◉

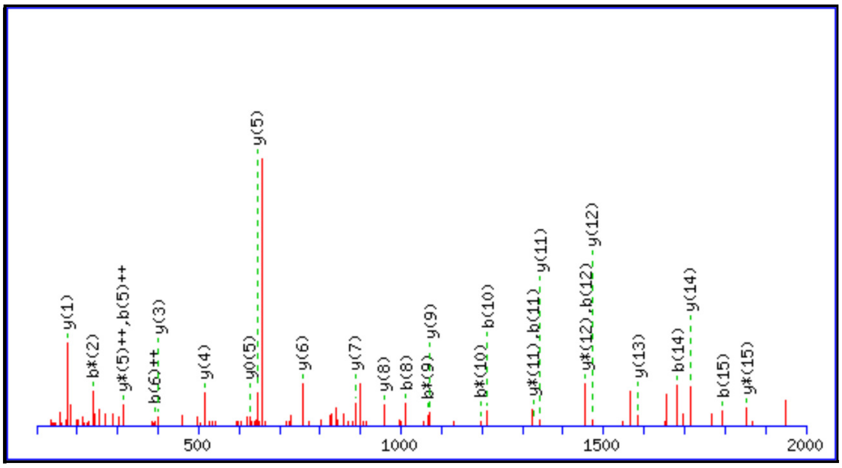

Monoisotopic mass of neutral peptide Mr(calc): 1968.0388
Fixed modifications: Carbamidomethyl (C) (apply to specified residues or termini only)
Variable modifications:
Q3 : Deamidated (NQ)
Ions Score: 98 **Expect:** 2.6e-008
Matches : 28/162 fragment ions using 35 most intense peaks (help)

#	b	b^{++}	b*	b^{*++}	b^{0}	b^{0++}	Seq.	y	y^{++}	y*	y^{*++}	y^{0}	y^{0++}	#
1	100.0757	50.5415					V							16
2	256.1768	128.5920	239.1503	120.0788			R	1869.9777	935.4925	1852.9512	926.9792	1851.9671	926.4872	15
3	385.2194	193.1133	368.1928	184.6001			Q	***1713.8766***	857.4419	1696.8501	848.9287	1695.8660	848.4367	14
4	498.3035	249.6554	481.2769	241.1421			L	***1584.8340***	792.9206	1567.8075	784.4074	1566.8234	783.9154	13
5	627.3461	**314.1767**	610.3195	305.6634	609.3355	305.1714	E	***1471.7499***	736.3786	1454.7234	727.8653	1453.7394	727.3733	12
6	783.4472	**392.2272**	766.4206	383.7139	765.4366	383.2219	R	***1342.7074***	671.8573	1325.6808	663.3440	1324.6968	662.8520	11
7	898.4741	449.7407	881.4476	441.2274	880.4635	440.7354	D	1186.6062	593.8068	1169.5797	585.2935	1168.5957	584.8015	10
8	**1012.5170**	506.7622	995.4905	498.2489	994.5065	497.7569	N	***1071.5793***	536.2933	1054.5527	527.7800	1053.5687	527.2880	9
9	1083.5541	542.2807	1066.5276	533.7674	1065.5436	533.2754	A	***957.5364***	479.2718	940.5098	470.7585	939.5258	470.2665	8
10	**1212.5967**	606.8020	1195.5702	598.2887	1194.5862	597.7967	E	***886.4993***	443.7533	869.4727	435.2400	868.4887	434.7480	7
11	**1325.6808**	663.3440	1308.6543	654.8308	1307.6702	654.3388	L	***757.4567***	379.2320	740.4301	370.7187	739.4461	370.2267	6
12	**1454.7234**	727.8653	1437.6968	719.3521	1436.7128	718.8601	E	***644.3726***	322.6899	627.3461	314.1767	626.3620	313.6847	5
13	1568.7663	784.8868	1551.7398	776.3735	1550.7558	775.8815	N	***515.3300***	258.1686	498.3035	249.6554			4
14	**1681.8504**	841.4288	1664.8238	832.9156	1663.8398	832.4235	L	***401.2871***	201.1472	384.2605	192.6339			3
15	**1794.9345**	897.9709	1777.9079	889.4576	1776.9239	888.9656	I	288.2030	144.6051	271.1765	136.0919			2
16							R	***175.1190***	88.0631	158.0924	79.5498			1

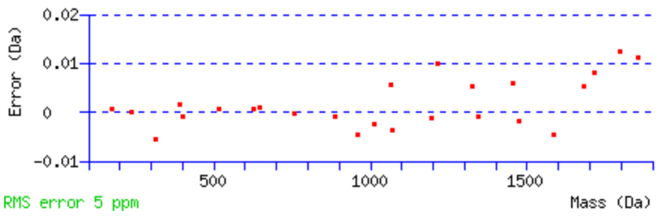

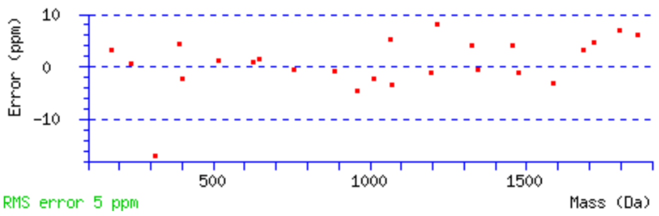

NCBI **BLAST** search of VRQLERDNAELENLIR

(Parameters: blastp, nr protein database, expect=20000, no filter, PAM30)
Other BLAST web gateways

All matches to this query

Score	Mr(calc):	Delta	Sequence
98.0	1968.0388	0.0097	VRQLERDNAELENLIR
82.5	1967.0548	0.9937	VRQLERDNAELENLIR
58.5	1968.0388	0.0097	VRQLERDNAELENLIR
10.1	1968.0388	0.0097	VRQLERDNAELENLIR
8.1	1968.0363	0.0122	RLEPFMVQPNPEARLR
5.6	1968.0540	-0.0056	REIEIQAHLQHPNILR

Mascot: http://www.matrixscience.com/

Mascot Search Results

Peptide View

MS/MS Fragmentation of **VSSVPSNSNVVVGTTNACAPSAR**
Found in **sp|O43790|KRT86_HUMAN**, Keratin, type II cuticular Hb6 OS=Homo sapiens GN=KRT86 PE=1 SV=1

Match to Query 33555: 2274.089322 from(759.037050,3+) intensity(3084413.0000) scans(10073) rtinseconds(1885) index(7751)
Title: 160219_Sunil_SDSI_A_Spectrum059859_scans__10073_RTINSECONDS=1885
Data file C:\\Sunil\\TKAP\\T\\T160219_Sunil_SDSI_A.mgf

Click mouse within plot area to zoom in by factor of two about that point
Or, Plot from 100 to 2000 Da Full range
Label all possible matches ○ Label matches used for scoring ●

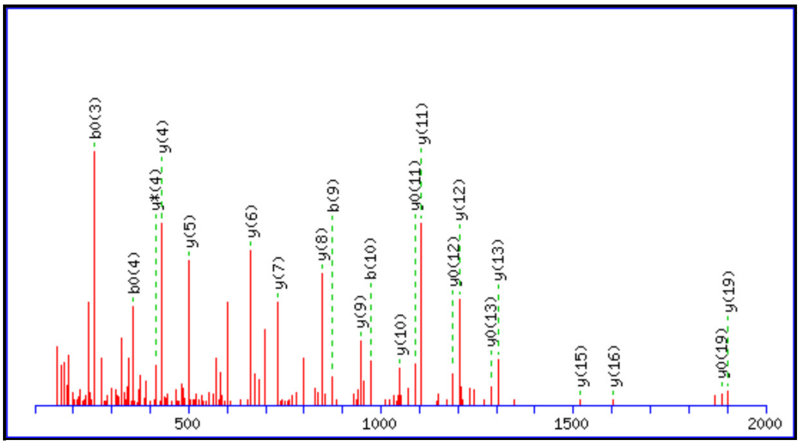

Monoisotopic mass of neutral peptide Mr(calc): 2274.0910
Fixed modifications: Carbamidomethyl (C) (apply to specified residues or termini only)
Variable modifications:
N9 : Deamidated (NQ)
Ions Score: 109 **Expect:** 3.1e-009
Matches : 22/246 fragment ions using 28 most intense peaks (help)

#	b	b^{++}	b*	b^{*++}	b^{0}	b^{0++}	Seq.	y	y^{++}	y*	y^{*++}	y^{0}	y^{0++}	#
1	100.0757	50.5415					V							23
2	187.1077	94.0575			169.0972	85.0522	S	2176.0299	1088.5186	2159.0033	1080.0053	2158.0193	1079.5133	22
3	274.1397	137.5735			256.1292	128.5682	S	2088.9979	1045.0026	2071.9713	1036.4893	2070.9873	1035.9973	21
4	373.2082	187.1077			355.1976	178.1024	V	2001.9658	1001.4866	1984.9393	992.9733	1983.9553	992.4813	20
5	470.2609	235.6341			452.2504	226.6288	P	***1902.8974***	951.9523	1885.8709	943.4391	1884.8869	942.9471	19
6	557.2930	279.1501			539.2824	270.1448	S	1805.8447	903.4260	1788.8181	894.9127	1787.8341	894.4207	18
7	671.3359	336.1716	654.3093	327.6583	653.3253	327.1663	N	1718.8126	859.9099	1701.7861	851.3967	1700.8021	850.9047	17
8	758.3679	379.6876	741.3414	371.1743	740.3573	370.6823	S	***1604.7697***	802.8885	1587.7431	794.3752	1586.7591	793.8832	16
9	**873.3948**	437.2011	856.3683	428.6878	855.3843	428.1958	N	***1517.7377***	759.3725	1500.7111	750.8592	1499.7271	750.3672	15
10	**972.4633**	486.7353	955.4367	478.2220	954.4527	477.7300	V	1402.7107	701.8590	1385.6842	693.3457	1384.7002	692.8537	14
11	1071.5317	536.2695	1054.5051	527.7562	1053.5211	527.2642	V	***1303.6423***	652.3248	1286.6158	643.8115	1285.6317	643.3195	13
12	1170.6001	585.8037	1153.5735	577.2904	1152.5895	576.7984	V	***1204.5739***	602.7906	1187.5473	594.2773	1186.5633	593.7853	12
13	1227.6216	614.3144	1210.5950	605.8011	1209.6110	605.3091	G	***1105.5055***	553.2564	1088.4789	544.7431	1087.4949	544.2511	11
14	1328.6692	664.8383	1311.6427	656.3250	1310.6587	655.8330	T	***1048.4840***	524.7456	1031.4575	516.2324	1030.4735	515.7404	10
15	1429.7169	715.3621	1412.6904	706.8488	1411.7063	706.3568	T	***947.4363***	474.2218	930.4098	465.7085	929.4258	465.2165	9
16	1543.7598	772.3836	1526.7333	763.8703	1525.7493	763.3783	N	***846.3887***	423.6980	829.3621	415.1847	828.3781	414.6927	8
17	1614.7970	807.9021	1597.7704	799.3888	1596.7864	798.8968	A	***732.3457***	366.6765	715.3192	358.1632	714.3352	357.6712	7
18	1774.8276	887.9174	1757.8011	879.4042	1756.8170	878.9122	C	***661.3086***	331.1579	644.2821	322.6447	643.2981	322.1527	6
19	1845.8647	923.4360	1828.8382	914.9227	1827.8542	914.4307	A	***501.2780***	251.1426	484.2514	242.6293	483.2674	242.1373	5
20	1942.9175	971.9624	1925.8909	963.4491	1924.9069	962.9571	P	***430.2409***	215.6241	413.2143	207.1108	412.2303	206.6188	4
21	2029.9495	1015.4784	2012.9230	1006.9651	2011.9389	1006.4731	S	333.1881	167.0977	316.1615	158.5844	315.1775	158.0924	3
22	2100.9866	1050.9969	2083.9601	1042.4837	2082.9761	1041.9917	A	246.1561	123.5817	229.1295	115.0684			2
23							R	175.1190	88.0631	158.0924	79.5498			1

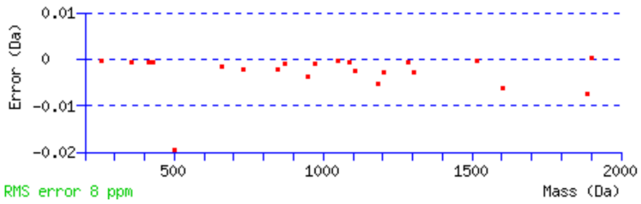

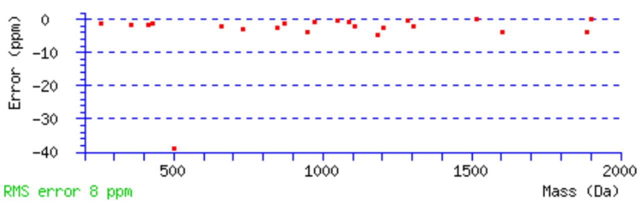

NCBI **BLAST** search of VSSVPSNSNVVVGTTNACAPSAR
(Parameters: blastp, nr protein database, expect=20000, no filter, PAM30)
Other BLAST web gateways

All matches to this query

Score	Mr(calc):	Delta	Sequence
109.2	2274.0910	-0.0017	VSSVPSNSNVVVGTTNACAPSAR
80.0	2274.0910	-0.0017	VSSVPSNSNVVVGTTNACAPSAR
57.3	2273.1070	0.9823	VSSVPSNSNVVVGTTNACAPSAR
22.7	2274.0910	-0.0017	VSSVPSNSNVVVGTTNACAPSAR

Mascot: http://www.matrixscience.com/

Mascot Search Results

Peptide View

MS/MS Fragmentation of **VSSVPSNSNVVVGTTNACAPSAR**
Found in **sp|O43790|KRT86_HUMAN**, Keratin, type II cuticular Hb6 OS=Homo sapiens GN=KRT86 PE=1 SV=1

Match to Query 33555: 2274.089322 from(759.037050,3+) intensity(3084413.0000) scans(10073) rtinseconds(1885) index(7751)
Title: 160219_Sunil_SDSI_A_Spectrum059859_scans__10073_RTINSECONDS=1885
Data file C:\\Sunil\\TKAP\\T\\T160219_Sunil_SDSI_A.mgf

Click mouse within plot area to zoom in by factor of two about that point
Or, Plot from 100 to 2000 Da Full range
Label all possible matches ○ Label matches used for scoring ◉

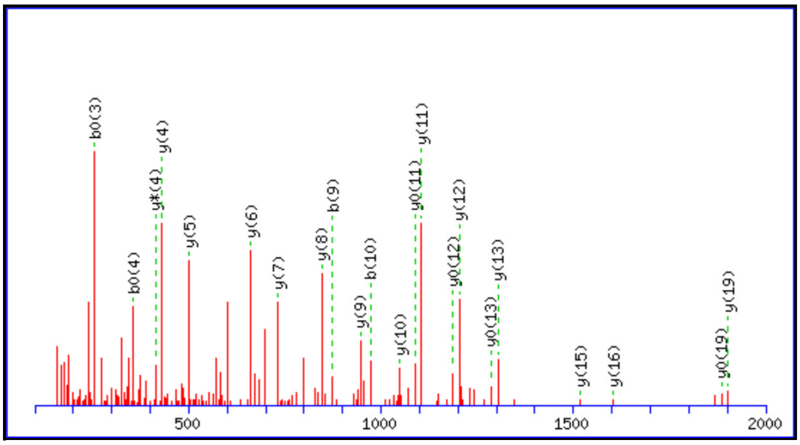

Monoisotopic mass of neutral peptide Mr(calc): 2274.0910
Fixed modifications: Carbamidomethyl (C) (apply to specified residues or termini only)
Variable modifications:
N9 : Deamidated (NQ)
Ions Score: 109 **Expect:** 3.1e-009
Matches : 22/246 fragment ions using 28 most intense peaks (help)

#	b	b^{++}	b^{*}	b^{*++}	b^{0}	b^{0++}	Seq.	y	y^{++}	y^{*}	y^{*++}	y^{0}	y^{0++}	#
1	100.0757	50.5415					V							23
2	187.1077	94.0575			169.0972	85.0522	S	2176.0299	1088.5186	2159.0033	1080.0053	2158.0193	1079.5133	22
3	274.1397	137.5735			256.1292	128.5682	S	2088.9979	1045.0026	2071.9713	1036.4893	2070.9873	1035.9973	21
4	373.2082	187.1077			355.1976	178.1024	V	2001.9658	1001.4866	1984.9393	992.9733	1983.9553	992.4813	20
5	470.2609	235.6341			452.2504	226.6288	P	***1902.8974***	951.9523	1885.8709	943.4391	1884.8869	942.9471	19
6	557.2930	279.1501			539.2824	270.1448	S	1805.8447	903.4260	1788.8181	894.9127	1787.8341	894.4207	18
7	671.3359	336.1716	654.3093	327.6583	653.3253	327.1663	N	1718.8126	859.9099	1701.7861	851.3967	1700.8021	850.9047	17
8	758.3679	379.6876	741.3414	371.1743	740.3573	370.6823	S	***1604.7697***	802.8885	1587.7431	794.3752	1586.7591	793.8832	16
9	**873.3948**	437.2011	856.3683	428.6878	855.3843	428.1958	N	***1517.7377***	759.3725	1500.7111	750.8592	1499.7271	750.3672	15
10	**972.4633**	486.7353	955.4367	478.2220	954.4527	477.7300	V	1402.7107	701.8590	1385.6842	693.3457	1384.7002	692.8537	14
11	1071.5317	536.2695	1054.5051	527.7562	1053.5211	527.2642	V	***1303.6423***	652.3248	1286.6158	643.8115	1285.6317	643.3195	13
12	1170.6001	585.8037	1153.5735	577.2904	1152.5895	576.7984	V	***1204.5739***	602.7906	1187.5473	594.2773	1186.5633	593.7853	12
13	1227.6216	614.3144	1210.5950	605.8011	1209.6110	605.3091	G	***1105.5055***	553.2564	1088.4789	544.7431	1087.4949	544.2511	11
14	1328.6692	664.8383	1311.6427	656.3250	1310.6587	655.8330	T	***1048.4840***	524.7456	1031.4575	516.2324	1030.4735	515.7404	10
15	1429.7169	715.3621	1412.6904	706.8488	1411.7063	706.3568	T	***947.4363***	474.2218	930.4098	465.7085	929.4258	465.2165	9
16	1543.7598	772.3836	1526.7333	763.8703	1525.7493	763.3783	N	***846.3887***	423.6980	829.3621	415.1847	828.3781	414.6927	8
17	1614.7970	807.9021	1597.7704	799.3888	1596.7864	798.8968	A	***732.3457***	366.6765	715.3192	358.1632	714.3352	357.6712	7
18	1774.8276	887.9174	1757.8011	879.4042	1756.8170	878.9122	C	***661.3086***	331.1579	644.2821	322.6447	643.2981	322.1527	6
19	1845.8647	923.4360	1828.8382	914.9227	1827.8542	914.4307	A	***501.2780***	251.1426	484.2514	242.6293	483.2674	242.1373	5
20	1942.9175	971.9624	1925.8909	963.4491	1924.9069	962.9571	P	***430.2409***	215.6241	413.2143	207.1108	412.2303	206.6188	4
21	2029.9495	1015.4784	2012.9230	1006.9651	2011.9389	1006.4731	S	333.1881	167.0977	316.1615	158.5844	315.1775	158.0924	3
22	2100.9866	1050.9969	2083.9601	1042.4837	2082.9761	1041.9917	A	246.1561	123.5817	229.1295	115.0684			2
23							R	175.1190	88.0631	158.0924	79.5498			1

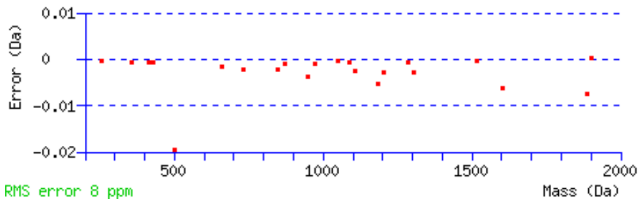

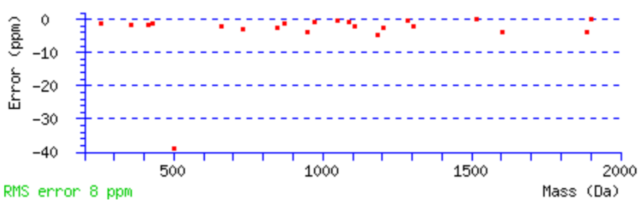

NCBI **BLAST** search of VSSVPSNSNVVVGTTNACAPSAR
(Parameters: blastp, nr protein database, expect=20000, no filter, PAM30)
Other BLAST web gateways

All matches to this query

Score	Mr(calc):	Delta	Sequence
109.2	2274.0910	-0.0017	VSSVPSNSNVVVGTTNACAPSAR
80.0	2274.0910	-0.0017	VSSVPSNSNVVVGTTNACAPSAR
57.3	2273.1070	0.9823	VSSVPSNSNVVVGTTNACAPSAR
22.7	2274.0910	-0.0017	VSSVPSNSNVVVGTTNACAPSAR

Mascot: http://www.matrixscience.com/

Mascot Search Results

Peptide View

MS/MS Fragmentation of **YQTELSLR**
Found in **sp|Q15323|K1H1_HUMAN**, Keratin, type I cuticular Ha1 OS=Homo sapiens GN=KRT31 PE=2 SV=3

Match to Query 3042: 1009.506448 from(505.760500,2+) intensity(12557124.0000) scans(8290) rtinseconds(1554) index(21541)
Title: 160219_Sunil_SDSI_A_Spectrum074845_scans__8290_RTINSECONDS=1554
Data file C:\\Sunil\\TKAP\\T\\T160219_Sunil_SDSI_A.mgf

Click mouse within plot area to zoom in by factor of two about that point
Or, Plot from 50 to 850 Da Full range
Label all possible matches Label matches used for scoring

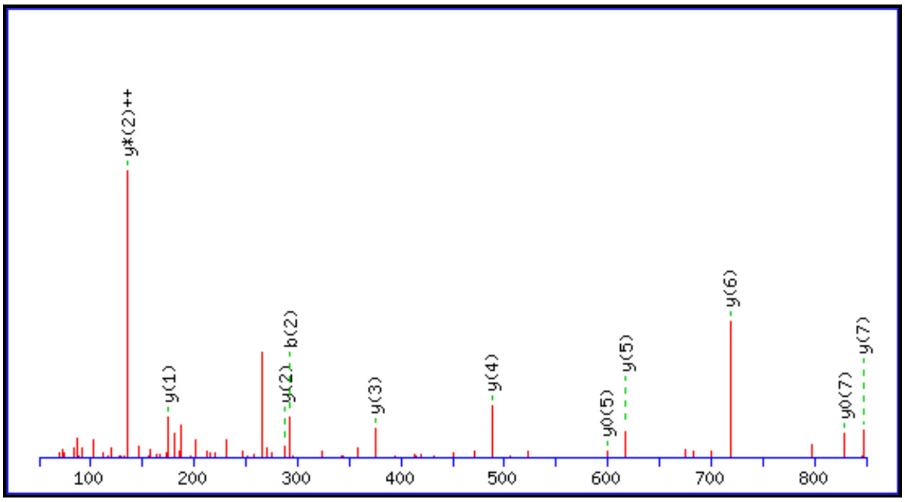

Monoisotopic mass of neutral peptide Mr(calc): 1009.5080
Fixed modifications: Carbamidomethyl (C) (apply to specified residues or termini only)
Variable modifications:
Q2 : Deamidated (NQ)
Ions Score: 59 **Expect:** 0.00017
Matches : 11/74 fragment ions using 16 most intense peaks (help)

#	b	b^{++}	b*	b^{*++}	b^0	b^{0++}	Seq.	y	y^{++}	y*	y^{*++}	y^0	y^{0++}	#
1	164.0706	82.5389					Y							8
2	**293.1132**	147.0602	276.0867	138.5470			Q	***847.4520***	424.2296	830.4254	415.7164	829.4414	415.2243	7
3	394.1609	197.5841	377.1343	189.0708	376.1503	188.5788	T	***718.4094***	359.7083	701.3828	351.1951	700.3988	350.7030	6
4	523.2035	262.1054	506.1769	253.5921	505.1929	253.1001	E	***617.3617***	309.1845	600.3352	300.6712	599.3511	300.1792	5
5	636.2875	318.6474	619.2610	310.1341	618.2770	309.6421	L	***488.3191***	244.6632	471.2926	236.1499	470.3085	235.6579	4
6	723.3196	362.1634	706.2930	353.6501	705.3090	353.1581	S	***375.2350***	188.1212	358.2085	179.6079	357.2245	179.1159	3
7	836.4036	418.7055	819.3771	410.1922	818.3931	409.7002	L	***288.2030***	144.6051	271.1765	136.0919			2
8							R	***175.1190***	88.0631	158.0924	79.5498			1

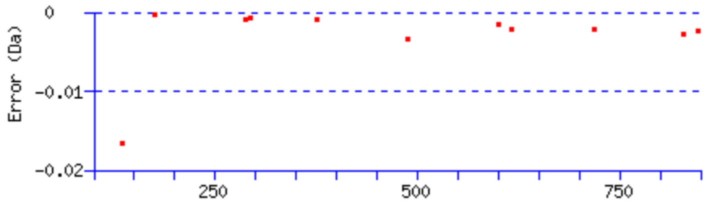

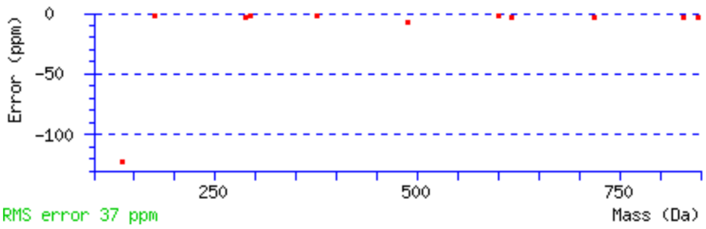

NCBI **BLAST** search of YQTELSLR
(Parameters: blastp, nr protein database, expect=20000, no filter, PAM30)
Other BLAST web gateways

All matches to this query

Score	Mr(calc):	Delta	Sequence
59.5	1009.5080	-0.0015	YETELSLR
59.5	1009.5080	-0.0015	YQTELSLR
15.8	1009.5080	-0.0015	YLEETSIR
14.0	1009.5015	0.0050	QFSCLSLR
13.5	1008.5062	1.0002	MKPNFSLR
13.5	1008.5062	1.0002	MNLEFSIR
13.2	1007.5036	2.0029	SVNEGAYIR
7.7	1009.5015	0.0050	YGNEMKLR
7.7	1009.5080	-0.0015	YLEESTIR
7.5	1007.4924	2.0141	EFDSQILR

Mascot: http://www.matrixscience.com/

Mascot Search Results

Peptide View

MS/MS Fragmentation of **YQTELSLR**
Found in **sp|Q15323|K1H1_HUMAN**, Keratin, type I cuticular Ha1 OS=Homo sapiens GN=KRT31 PE=2 SV=3

Match to Query 3042: 1009.506448 from(505.760500,2+) intensity(12557124.0000) scans(8290) rtinseconds(1554) index(21541)
Title: 160219_Sunil_SDSI_A_Spectrum074845_scans__8290_RTINSECONDS=1554
Data file C:\\Sunil\\TKAP\\T\\T160219_Sunil_SDSI_A.mgf

Click mouse within plot area to zoom in by factor of two about that point
Or, Plot from 50 to 850 Da Full range
Label all possible matches Label matches used for scoring

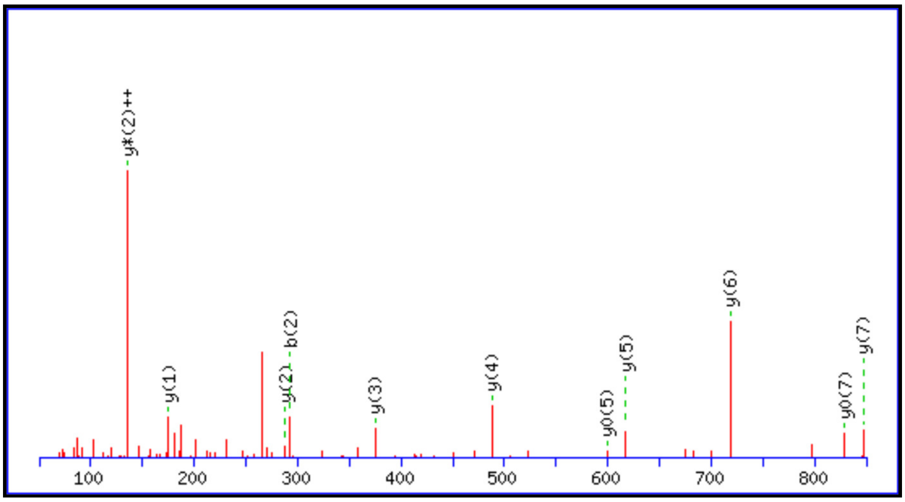

Monoisotopic mass of neutral peptide Mr(calc): 1009.5080
Fixed modifications: Carbamidomethyl (C) (apply to specified residues or termini only)
Variable modifications:
Q2 : Deamidated (NQ)
Ions Score: 59 **Expect:** 0.00017
Matches : 11/74 fragment ions using 16 most intense peaks (help)

#	b	b^{++}	b*	b^{*++}	b^0	b^{0++}	Seq.	y	y^{++}	y*	y^{*++}	y^0	y^{0++}	#
1	164.0706	82.5389					Y							8
2	**293.1132**	147.0602	276.0867	138.5470			Q	***847.4520***	424.2296	830.4254	415.7164	829.4414	415.2243	7
3	394.1609	197.5841	377.1343	189.0708	376.1503	188.5788	T	***718.4094***	359.7083	701.3828	351.1951	700.3988	350.7030	6
4	523.2035	262.1054	506.1769	253.5921	505.1929	253.1001	E	***617.3617***	309.1845	600.3352	300.6712	599.3511	300.1792	5
5	636.2875	318.6474	619.2610	310.1341	618.2770	309.6421	L	***488.3191***	244.6632	471.2926	236.1499	470.3085	235.6579	4
6	723.3196	362.1634	706.2930	353.6501	705.3090	353.1581	S	***375.2350***	188.1212	358.2085	179.6079	357.2245	179.1159	3
7	836.4036	418.7055	819.3771	410.1922	818.3931	409.7002	L	***288.2030***	144.6051	271.1765	136.0919			2
8							R	***175.1190***	88.0631	158.0924	79.5498			1

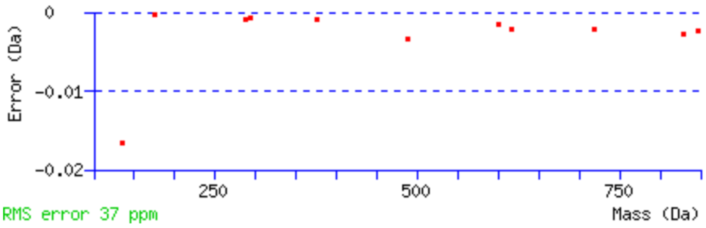

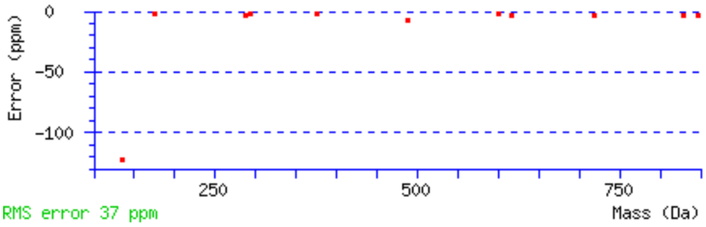

NCBI **BLAST** search of YQTELSLR
(Parameters: blastp, nr protein database, expect=20000, no filter, PAM30)
Other BLAST web gateways

All matches to this query

Score	Mr(calc):	Delta	Sequence
59.5	1009.5080	-0.0015	YETELSLR
59.5	1009.5080	-0.0015	YQTELSLR
15.8	1009.5080	-0.0015	YLEETSIR
14.0	1009.5015	0.0050	QFSCLSLR
13.5	1008.5062	1.0002	MKPNFSLR
13.5	1008.5062	1.0002	MNLEFSIR
13.2	1007.5036	2.0029	SVNEGAYIR
7.7	1009.5015	0.0050	YGNEMKLR
7.7	1009.5080	-0.0015	YLEESTIR
7.5	1007.4924	2.0141	EFDSQILR

Mascot: http://www.matrixscience.com/

Mascot Search Results

Peptide View

MS/MS Fragmentation of **YSSQLSQVQSLITNVESQLAEIR**
Found in **sp|Q15323|K1H1_HUMAN**, Keratin, type I cuticular Ha1 OS=Homo sapiens GN=KRT31 PE=2 SV=3

Match to Query 37732: 2593.324068 from(1297.669310,2+) intensity(624507.1875) scans(16449) rtinseconds(2956) index(28360)
Title: 160219_Sunil_SDSI_A_Spectrum081689_scans__16449_RTINSECONDS=2956
Data file C:\\Sunil\\TKAP\\T\\T160219_Sunil_SDSI_A.mgf

Click mouse within plot area to zoom in by factor of two about that point
Or, Plot from 200 to 2100 Da Full range
Label all possible matches Label matches used for scoring

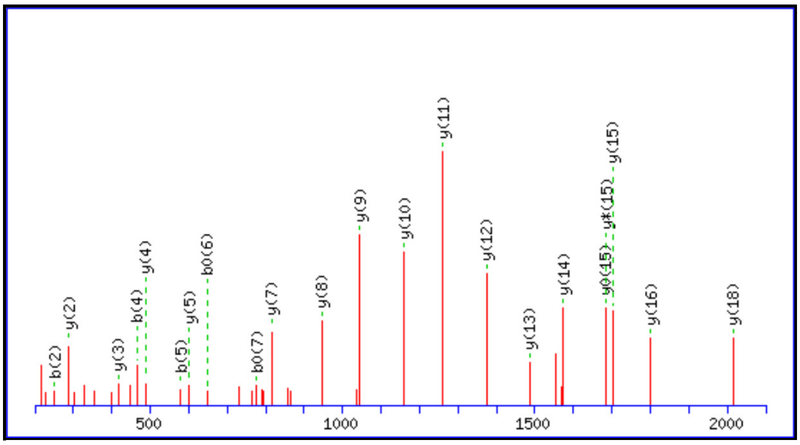

Monoisotopic mass of neutral peptide Mr(calc): 2593.3235
Fixed modifications: Carbamidomethyl (C) (apply to specified residues or termini only)
Variable modifications:
N14 : Deamidated (NQ)
Ions Score: 119 **Expect:** 3e-010
Matches : 22/252 fragment ions using 33 most intense peaks (help)

#	b	b++	b*	b*++	b0	b0++	Seq.	y	y++	y*	y*++	y0	y0++	#
1	164.0706	82.5389					Y							23
2	**251.1026**	126.0550			233.0921	117.0497	S	2431.2675	1216.1374	2414.2409	1207.6241	2413.2569	1207.1321	22
3	338.1347	169.5710			320.1241	160.5657	S	2344.2354	1172.6214	2327.2089	1164.1081	2326.2249	1163.6161	21
4	**466.1932**	233.6003	449.1667	225.0870	448.1827	224.5950	Q	2257.2034	1129.1053	2240.1769	1120.5921	2239.1929	1120.1001	20
5	**579.2773**	290.1423	562.2508	281.6290	561.2667	281.1370	L	2129.1448	1065.0761	2112.1183	1056.5628	2111.1343	1056.0708	19
6	666.3093	333.6583	649.2828	325.1450	648.2988	324.6530	S	***2016.0608***	1008.5340	1999.0342	1000.0208	1998.0502	999.5287	18
7	794.3679	397.6876	777.3414	389.1743	776.3573	388.6823	Q	1929.0287	965.0180	1912.0022	956.5047	1911.0182	956.0127	17
8	893.4363	447.2218	876.4098	438.7085	875.4258	438.2165	V	***1800.9702***	900.9887	1783.9436	892.4754	1782.9596	891.9834	16
9	1021.4949	511.2511	1004.4684	502.7378	1003.4843	502.2458	Q	***1701.9018***	851.4545	1684.8752	842.9412	1683.8912	842.4492	15
10	1108.5269	554.7671	1091.5004	546.2538	1090.5164	545.7618	S	***1573.8432***	787.4252	1556.8166	778.9120	1555.8326	778.4199	14
11	1221.6110	611.3091	1204.5844	602.7959	1203.6004	602.3039	L	***1486.8112***	743.9092	1469.7846	735.3959	1468.8006	734.9039	13
12	1334.6951	667.8512	1317.6685	659.3379	1316.6845	658.8459	I	***1373.7271***	687.3672	1356.7005	678.8539	1355.7165	678.3619	12
13	1435.7427	718.3750	1418.7162	709.8617	1417.7322	709.3697	T	***1260.6430***	630.8251	1243.6165	622.3119	1242.6325	621.8199	11
14	1550.7697	775.8885	1533.7431	767.3752	1532.7591	766.8832	N	***1159.5953***	580.3013	1142.5688	571.7880	1141.5848	571.2960	10
15	1649.8381	825.4227	1632.8115	816.9094	1631.8275	816.4174	V	***1044.5684***	522.7878	1027.5419	514.2746	1026.5578	513.7826	9
16	1778.8807	889.9440	1761.8541	881.4307	1760.8701	880.9387	E	***945.5000***	473.2536	928.4734	464.7404	927.4894	464.2483	8
17	1865.9127	933.4600	1848.8862	924.9467	1847.9021	924.4547	S	***816.4574***	408.7323	799.4308	400.2191	798.4468	399.7271	7
18	1993.9713	997.4893	1976.9447	988.9760	1975.9607	988.4840	Q	729.4254	365.2163	712.3988	356.7030	711.4148	356.2110	6
19	2107.0554	1054.0313	2090.0288	1045.5180	2089.0448	1045.0260	L	***601.3668***	301.1870	584.3402	292.6738	583.3562	292.1817	5
20	2178.0925	1089.5499	2161.0659	1081.0366	2160.0819	1080.5446	A	***488.2827***	244.6450	471.2562	236.1317	470.2722	235.6397	4
21	2307.1351	1154.0712	2290.1085	1145.5579	2289.1245	1145.0659	E	***417.2456***	209.1264	400.2191	200.6132	399.2350	200.1212	3
22	2420.2191	1210.6132	2403.1926	1202.0999	2402.2086	1201.6079	I	***288.2030***	144.6051	271.1765	136.0919			2
23							R	175.1190	88.0631	158.0924	79.5498			1

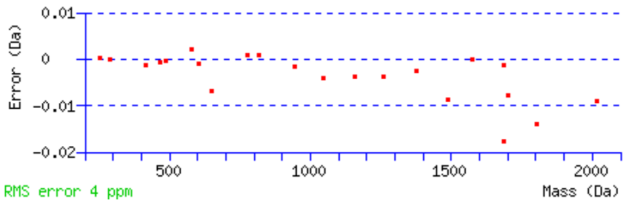

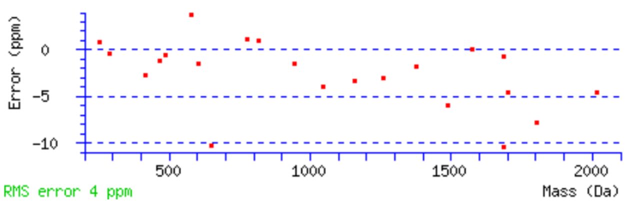

NCBI **BLAST** search of YSSQLSQVQSLITNVESQLAEIR
(Parameters: blastp, nr protein database, expect=20000, no filter, PAM30)
Other BLAST web gateways

All matches to this query

Score	Mr(calc):	Delta	Sequence
118.9	2593.3235	0.0006	YSSQLSQVQSLITNVESQLAEIR
76.8	2593.3235	0.0006	YSSQLSQVQSLITNVESQLAEIR
52.3	2593.3235	0.0006	YSSQLSQVQSLITNVESQLAEIR
28.2	2593.3235	0.0006	YSSQLSQVQSLITNVESQLAEIR
22.2	2592.3395	0.9846	YSSQLSQVQSLITNVESQLAEIR
15.8	2593.3235	0.0006	YSSQLSQVQSLITNVESQLAEIR
1.6	2591.3014	2.0227	VMNFTVDSPLVLQSDRNVTVNAR

Mascot: http://www.matrixscience.com/

Mascot Search Results

Peptide View

MS/MS Fragmentation of **YSSQLSQVQSLITNVESQLAEIR**
Found in **sp|Q15323|K1H1_HUMAN**, Keratin, type I cuticular Ha1 OS=Homo sapiens GN=KRT31 PE=2 SV=3

Match to Query 37731: 2593.322532 from(865.448120,3+) intensity(1233425.7500) scans(16443) rtinseconds(2955) index(28355)
Title: 160219_Sunil_SDSI_A_Spectrum081684_scans__16443_RTINSECONDS=2955
Data file C:\\Sunil\\TKAP\\T\\T160219_Sunil_SDSI_A.mgf

Click mouse within plot area to zoom in by factor of two about that point
Or, Plot from 0 to 2600 Da Full range
Label all possible matches Label matches used for scoring

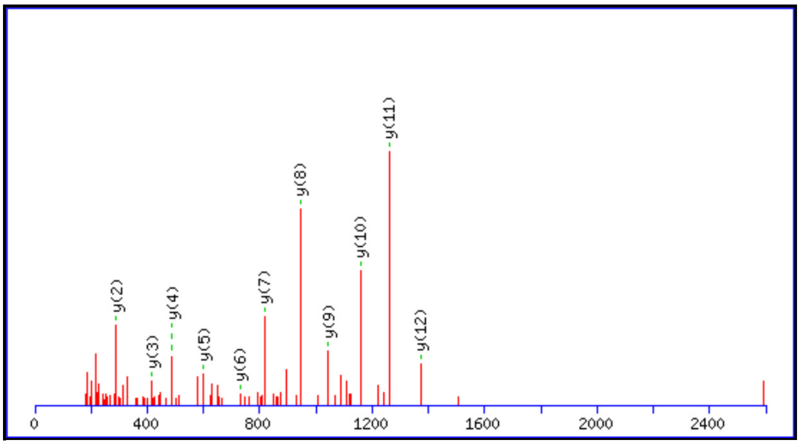

Monoisotopic mass of neutral peptide Mr(calc): 2593.3235
Fixed modifications: Carbamidomethyl (C) (apply to specified residues or termini only)
Variable modifications:
N14 : Deamidated (NQ)
Ions Score: 92 **Expect:** 1.4e-007
Matches : 11/252 fragment ions using 14 most intense peaks (help)

#	b	b^{++}	b^{*}	b^{*++}	b^{0}	b^{0++}	Seq.	y	y^{++}	y^{*}	y^{*++}	y^{0}	y^{0++}	#
1	164.0706	82.5389					Y							23
2	251.1026	126.0550			233.0921	117.0497	S	2431.2675	1216.1374	2414.2409	1207.6241	2413.2569	1207.1321	22
3	338.1347	169.5710			320.1241	160.5657	S	2344.2354	1172.6214	2327.2089	1164.1081	2326.2249	1163.6161	21
4	466.1932	233.6003	449.1667	225.0870	448.1827	224.5950	Q	2257.2034	1129.1053	2240.1769	1120.5921	2239.1929	1120.1001	20
5	579.2773	290.1423	562.2508	281.6290	561.2667	281.1370	L	2129.1448	1065.0761	2112.1183	1056.5628	2111.1343	1056.0708	19
6	666.3093	333.6583	649.2828	325.1450	648.2988	324.6530	S	2016.0608	1008.5340	1999.0342	1000.0208	1998.0502	999.5287	18
7	794.3679	397.6876	777.3414	389.1743	776.3573	388.6823	Q	1929.0287	965.0180	1912.0022	956.5047	1911.0182	956.0127	17
8	893.4363	447.2218	876.4098	438.7085	875.4258	438.2165	V	1800.9702	900.9887	1783.9436	892.4754	1782.9596	891.9834	16
9	1021.4949	511.2511	1004.4684	502.7378	1003.4843	502.2458	Q	1701.9018	851.4545	1684.8752	842.9412	1683.8912	842.4492	15
10	1108.5269	554.7671	1091.5004	546.2538	1090.5164	545.7618	S	1573.8432	787.4252	1556.8166	778.9120	1555.8326	778.4199	14
11	1221.6110	611.3091	1204.5844	602.7959	1203.6004	602.3039	L	1486.8112	743.9092	1469.7846	735.3959	1468.8006	734.9039	13
12	1334.6951	667.8512	1317.6685	659.3379	1316.6845	658.8459	I	*1373.7271*	687.3672	1356.7005	678.8539	1355.7165	678.3619	12
13	1435.7427	718.3750	1418.7162	709.8617	1417.7322	709.3697	T	*1260.6430*	630.8251	1243.6165	622.3119	1242.6325	621.8199	11
14	1550.7697	775.8885	1533.7431	767.3752	1532.7591	766.8832	N	*1159.5953*	580.3013	1142.5688	571.7880	1141.5848	571.2960	10
15	1649.8381	825.4227	1632.8115	816.9094	1631.8275	816.4174	V	*1044.5684*	522.7878	1027.5419	514.2746	1026.5578	513.7826	9
16	1778.8807	889.9440	1761.8541	881.4307	1760.8701	880.9387	E	*945.5000*	473.2536	928.4734	464.7404	927.4894	464.2483	8
17	1865.9127	933.4600	1848.8862	924.9467	1847.9021	924.4547	S	*816.4574*	408.7323	799.4308	400.2191	798.4468	399.7271	7
18	1993.9713	997.4893	1976.9447	988.9760	1975.9607	988.4840	Q	*729.4254*	365.2163	712.3988	356.7030	711.4148	356.2110	6
19	2107.0554	1054.0313	2090.0288	1045.5180	2089.0448	1045.0260	L	*601.3668*	301.1870	584.3402	292.6738	583.3562	292.1817	5
20	2178.0925	1089.5499	2161.0659	1081.0366	2160.0819	1080.5446	A	*488.2827*	244.6450	471.2562	236.1317	470.2722	235.6397	4
21	2307.1351	1154.0712	2290.1085	1145.5579	2289.1245	1145.0659	E	*417.2456*	209.1264	400.2191	200.6132	399.2350	200.1212	3
22	2420.2191	1210.6132	2403.1926	1202.0999	2402.2086	1201.6079	I	*288.2030*	144.6051	271.1765	136.0919			2
23							R	175.1190	88.0631	158.0924	79.5498			1

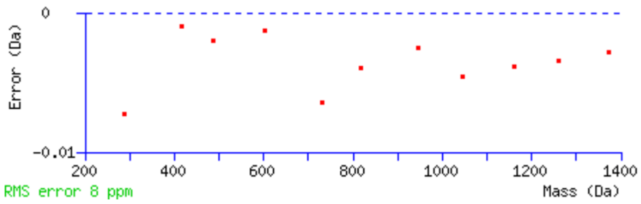

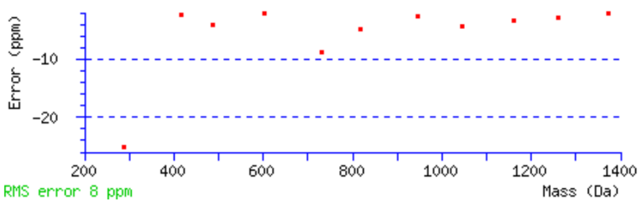

NCBI **BLAST** search of YSSQLSQVQSLITNVESQLAEIR
(Parameters: blastp, nr protein database, expect=20000, no filter, PAM30)
Other BLAST web gateways

All matches to this query

Score	Mr(calc):	Delta	Sequence
92.2	2593.3235	-0.0010	YSSQLSQVQSLITNVESQLAEIR
46.8	2592.3395	0.9831	YSSQLSQVQSLITNVESQLAEIR
46.4	2593.3235	-0.0010	YSSQLSQVQSLITNVESQLAEIR
46.4	2593.3235	-0.0010	YSSQLSQVQSLITNVESQLAEIR
46.4	2593.3235	-0.0010	YSSQLSQVQSLITNVESQLAEIR
37.9	2593.3235	-0.0010	YSSQLSQVQSLITNVESQLAEIR
1.2	2593.3234	-0.0009	ESELKEIEQELHLAQAEIQSLR
1.0	2591.3014	2.0212	VMNFTVDSPLVLQSDRNVTVNAR
0.9	2593.3057	0.0168	VQLSESPASLPSCPPVETALINQR
0.9	2593.3057	0.0168	VQLSESPASLPSCPPVETALINQR

Mascot: http://www.matrixscience.com/

MS/MS spectrums and fragmentation profile of K37 in Urea, SDSI and SDSII extracted hair shaft

Mascot Search Results

Peptide View

MS/MS Fragmentation of **LLDDATLAKADLEAQQESLKEEQLSLK**
Found in **ch17u_O76014|KRT37_HUMAN** in **uni_human**, Keratin, type I cuticular Ha7 OS=Homo sapiens GN=KRT37 PE=3 SV=3

Match to Query 39984: 2998.567576 from(750.649170,4+) intensity(7421416.0000) rtinseconds(2689) scans(14757) index(11622)
Title: 160219_Sunil_SDSI_A_Spectrum063750_scans__14757_RTINSECONDS=2689
Data file L:\\QE_2016\\160219_Sunil_KAP_LKC\\TMgf\\T\\T160219_Sunil_SDSI_A.mgf

Click mouse within plot area to zoom in by factor of two about that point
Or, Plot from 200 to 3000 Da Full range
Label all possible matches ○ Label matches used for scoring ◉
Show Y-axis ☐

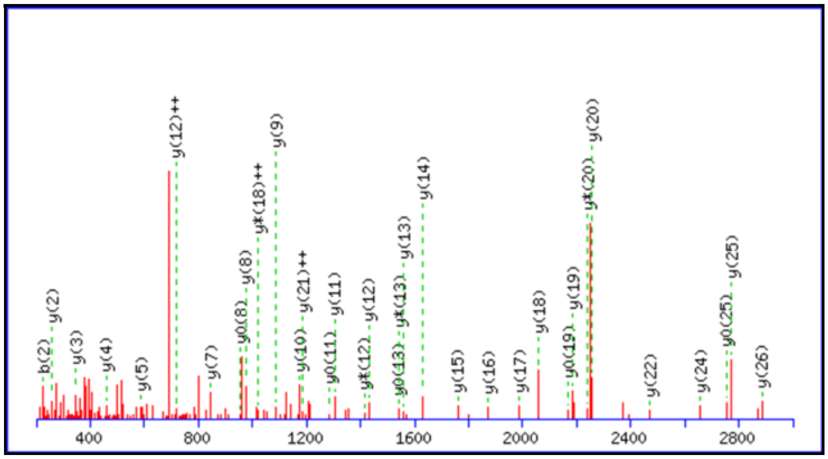

Monoisotopic mass of neutral peptide Mr(calc): 2998.5710
Fixed modifications: Carbamidomethyl (C) (apply to specified residues or termini only)
Ions Score: 131 **Expect:** 8.5e-012
Matches : 34/288 fragment ions using 88 most intense peaks (help)

#	b	b^{++}	b^*	b^{*++}	b^0	b^{0++}	Seq.	y	y^{++}	y^*	y^{*++}	y^0	y^{0++}	#
1	114.0913	57.5493					L							27
2	227.1754	114.0913					L	2886.4942	1443.7508	2869.4677	1435.2375	2868.4837	1434.7455	26
3	342.2023	171.6048			324.1918	162.5995	D	2773.4102	1387.2087	2756.3836	1378.6955	2755.3996	1378.2034	25
4	457.2293	229.1183			439.2187	220.1130	D	2658.3832	1329.6953	2641.3567	1321.1820	2640.3727	1320.6900	24
5	528.2664	264.6368			510.2558	255.6316	A	2543.3563	1272.1818	2526.3297	1263.6685	2525.3457	1263.1765	23
6	629.3141	315.1607			611.3035	306.1554	T	2472.3192	1236.6632	2455.2926	1228.1500	2454.3086	1227.6579	22
7	742.3981	371.7027			724.3876	362.6974	L	2371.2715	1186.1394	2354.2449	1177.6261	2353.2609	1177.1341	21
8	813.4353	407.2213			795.4247	398.2160	A	2258.1874	1129.5974	2241.1609	1121.0841	2240.1769	1120.5921	20
9	941.5302	471.2688	924.5037	462.7555	923.5197	462.2635	K	2187.1503	1094.0788	2170.1238	1085.5655	2169.1398	1085.0735	19
10	1012.5673	506.7873	995.5408	498.2740	994.5568	497.7820	A	2059.0554	1030.0313	2042.0288	1021.5180	2041.0448	1021.0260	18
11	1127.5943	564.3008	1110.5677	555.7875	1109.5837	555.2955	D	1988.0182	994.5128	1970.9917	985.9995	1970.0077	985.5075	17
12	1240.6783	620.8428	1223.6518	612.3295	1222.6678	611.8375	L	1872.9913	936.9993	1855.9648	928.4860	1854.9807	927.9940	16
13	1369.7209	685.3641	1352.6944	676.8508	1351.7104	676.3588	E	1759.9072	880.4573	1742.8807	871.9440	1741.8967	871.4520	15
14	1440.7581	720.8827	1423.7315	712.3694	1422.7475	711.8774	A	1630.8646	815.9360	1613.8381	807.4227	1612.8541	806.9307	14
15	1568.8166	784.9120	1551.7901	776.3987	1550.8061	775.9067	Q	1559.8275	780.4174	1542.8010	771.9041	1541.8170	771.4121	13
16	1696.8752	848.9412	1679.8487	840.4280	1678.8646	839.9360	Q	1431.7690	716.3881	1414.7424	707.8748	1413.7584	707.3828	12
17	1825.9178	913.4625	1808.8913	904.9493	1807.9072	904.4573	E	1303.7104	652.3588	1286.6838	643.8455	1285.6998	643.3535	11
18	1912.9498	956.9786	1895.9233	948.4653	1894.9393	947.9733	S	1174.6678	587.8375	1157.6412	579.3243	1156.6572	578.8322	10
19	2026.0339	1013.5206	2009.0073	1005.0073	2008.0233	1004.5153	L	1087.6358	544.3215	1070.6092	535.8082	1069.6252	535.3162	9
20	2154.1289	1077.5681	2137.1023	1069.0548	2136.1183	1068.5628	K	974.5517	487.7795	957.5251	479.2662	956.5411	478.7742	8
21	2283.1714	1142.0894	2266.1449	1133.5761	2265.1609	1133.0841	E	846.4567	423.7320	829.4302	415.2187	828.4462	414.7267	7

22	2412.2140	1206.6107	2395.1875	1198.0974	2394.2035	1197.6054	E	717.4141	359.2107	700.3876	350.6974	699.4036	350.2054	6
23	2540.2726	1270.6399	2523.2461	1262.1267	2522.2621	1261.6347	Q	***588.3715***	294.6894	571.3450	286.1761	570.3610	285.6841	5
24	2653.3567	1327.1820	2636.3301	1318.6687	2635.3461	1318.1767	L	***460.3130***	230.6601	443.2864	222.1468	442.3024	221.6548	4
25	2740.3887	1370.6980	2723.3622	1362.1847	2722.3781	1361.6927	S	***347.2289***	174.1181	330.2023	165.6048	329.2183	165.1128	3
26	2853.4728	1427.2400	2836.4462	1418.7268	2835.4622	1418.2347	L	***260.1969***	130.6021	243.1703	122.0888			2
27							K	147.1128	74.0600	130.0863	65.5468			1

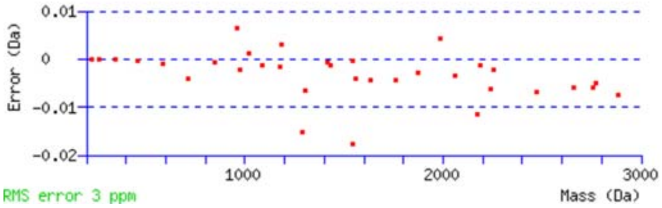

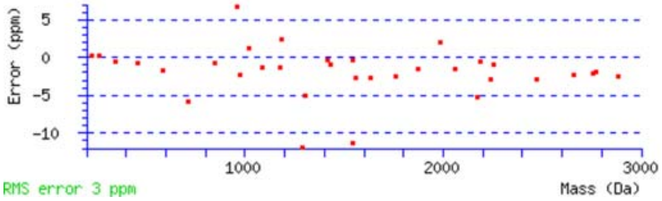

NCBI **BLAST** search of LLDDATLAKADLEAQQESLKEEQLSLK
(Parameters: blastp, nr protein database, expect=20000, no filter, PAM30)
Other BLAST web gateways

All matches to this query

Score	Mr(calc)	Delta	Sequence
131.0	2998.5710	-0.0034	LLDDATLAKADLEAQQESLKEEQLSLK

Mascot: http://www.matrixscience.com/

Mascot Search Results

Peptide View

MS/MS Fragmentation of **ADLEAQQESLKEEQLSLKSNHEQEVK**
Found in **ch17u_O76014|KRT37_HUMAN** in **uni_human**, Keratin, type I cuticular Ha7 OS=Homo sapiens GN=KRT37 PE=3 SV=3

Match to Query 40023: 3009.493856 from(753.380740,4+) intensity(565858.8750) rtinseconds(1571) scans(8239) index(6127)
Title: 160219_Sunil_SDSI_A_Spectrum058235_scans__8239_RTINSECONDS=1571
Data file L:\\QE_2016\\160219_Sunil_KAP_LKC\\TMgf\\T\\T160219_Sunil_SDSI_A.mgf

Click mouse within plot area to zoom in by factor of two about that point
Or, Plot from 200 to 3000 Da Full range
Label all possible matches ○ Label matches used for scoring ◉
Show Y-axis ☐

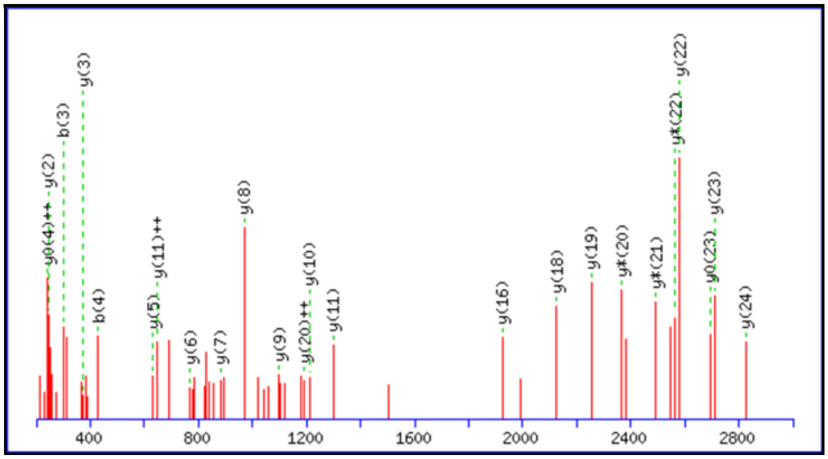

Monoisotopic mass of neutral peptide Mr(calc): 3009.4890
Fixed modifications: Carbamidomethyl (C) (apply to specified residues or termini only)
Ions Score: 97 **Expect:** 4.2e-008
Matches : 24/284 fragment ions using 43 most intense peaks (help)

#	b	b^{++}	b^{*}	b^{*++}	b^{0}	b^{0++}	Seq.	y	y^{++}	y^{*}	y^{*++}	y^{0}	y^{0++}	#
1	72.0444	36.5258					A							26
2	187.0713	94.0393			169.0608	85.0340	D	2939.4592	1470.2333	2922.4327	1461.7200	2921.4487	1461.2280	25
3	**300.1554**	150.5813			282.1448	141.5761	L	**2824.4323**	1412.7198	2807.4058	1404.2065	2806.4217	1403.7145	24
4	**429.1980**	215.1026			411.1874	206.0974	E	**2711.3482**	1356.1778	2694.3217	1347.6645	2693.3377	1347.1725	23
5	500.2351	250.6212			482.2245	241.6159	A	**2582.3056**	1291.6565	2565.2791	1283.1432	2564.2951	1282.6512	22
6	628.2937	314.6505	611.2671	306.1372	610.2831	305.6452	Q	2511.2685	1256.1379	2494.2420	1247.6246	2493.2580	1247.1326	21
7	756.3523	378.6798	739.3257	370.1665	738.3417	369.6745	Q	2383.2100	1192.1086	2366.1834	1183.5953	2365.1994	1183.1033	20
8	885.3949	443.2011	868.3683	434.6878	867.3843	434.1958	E	**2255.1514**	1128.0793	2238.1248	1119.5661	2237.1408	1119.0740	19
9	972.4269	486.7171	955.4003	478.2038	954.4163	477.7118	S	**2126.1088**	1063.5580	2109.0822	1055.0448	2108.0982	1054.5527	18
10	1085.5109	543.2591	1068.4844	534.7458	1067.5004	534.2538	L	2039.0768	1020.0420	2022.0502	1011.5287	2021.0662	1011.0367	17
11	1213.6059	607.3066	1196.5794	598.7933	1195.5953	598.3013	K	**1925.9927**	963.5000	1908.9661	954.9867	1907.9821	954.4947	16
12	1342.6485	671.8279	1325.6220	663.3146	1324.6379	662.8226	E	1797.8977	899.4525	1780.8712	890.9392	1779.8872	890.4472	15
13	1471.6911	736.3492	1454.6645	727.8359	1453.6805	727.3439	E	1668.8551	834.9312	1651.8286	826.4179	1650.8446	825.9259	14
14	1599.7497	800.3785	1582.7231	791.8652	1581.7391	791.3732	Q	1539.8125	770.4099	1522.7860	761.8966	1521.8020	761.4046	13
15	1712.8337	856.9205	1695.8072	848.4072	1694.8232	847.9152	L	1411.7540	706.3806	1394.7274	697.8673	1393.7434	697.3753	12
16	1799.8658	900.4365	1782.8392	891.9232	1781.8552	891.4312	S	**1298.6699**	649.8386	1281.6434	641.3253	1280.6593	640.8333	11
17	1912.9498	956.9786	1895.9233	948.4653	1894.9393	947.9733	L	**1211.6379**	606.3226	1194.6113	597.8093	1193.6273	597.3173	10
18	2041.0448	1021.0260	2024.0182	1012.5128	2023.0342	1012.0208	K	**1098.5538**	549.7805	1081.5273	541.2673	1080.5432	540.7753	9
19	2128.0768	1064.5420	2111.0503	1056.0288	2110.0663	1055.5368	S	**970.4588**	485.7331	953.4323	477.2198	952.4483	476.7278	8
20	2242.1197	1121.5635	2225.0932	1113.0502	2224.1092	1112.5582	N	**883.4268**	442.2170	866.4003	433.7038	865.4163	433.2118	7
21	2379.1787	1190.0930	2362.1521	1181.5797	2361.1681	1181.0877	H	**769.3839**	385.1956	752.3573	376.6823	751.3733	376.1903	6

22	2508.2213	1254.6143	2491.1947	1246.1010	2490.2107	1245.6090	E	***632.3250***	316.6661	615.2984	308.1529	614.3144	307.6608	5
23	2636.2798	1318.6436	2619.2533	1310.1303	2618.2693	1309.6383	Q	503.2824	252.1448	486.2558	243.6316	485.2718	243.1396	4
24	2765.3224	1383.1648	2748.2959	1374.6516	2747.3119	1374.1596	E	***375.2238***	188.1155	358.1973	179.6023	357.2132	179.1103	3
25	2864.3908	1432.6991	2847.3643	1424.1858	2846.3803	1423.6938	V	***246.1812***	123.5942	229.1547	115.0810			2
26							K	147.1128	74.0600	130.0863	65.5468			1

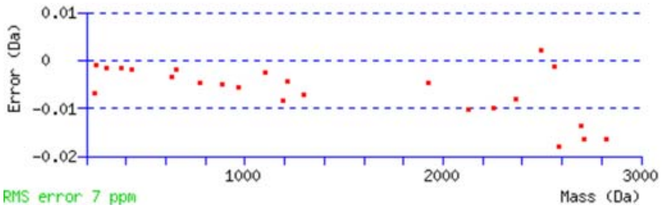

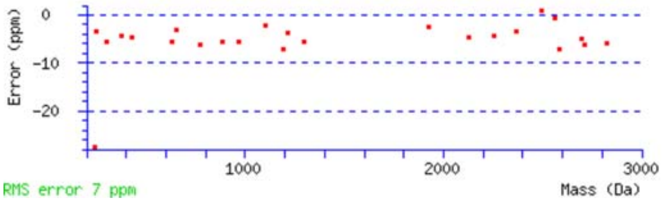

NCBI **BLAST** search of ADLEAQQESLKEEQLSLKSNHEQEVK
(Parameters: blastp, nr protein database, expect=20000, no filter, PAM30)
Other BLAST web gateways

All matches to this query

Score	Mr(calc)	Delta	Sequence
96.6	3009.4890	0.0048	ADLEAQQESLKEEQLSLKSNHEQEVK
3.5	3009.5229	-0.0291	ELRVEQESLLTAFRCGLQFLGNIASR

Mascot: http://www.matrixscience.com/

Mascot Search Results

Peptide View

MS/MS Fragmentation of **ADLERQNQEYQVLLDVK**
Found in **ch17u_O76014|KRT37_HUMAN** in **uni_human**, Keratin, type I cuticular Ha7 OS=Homo sapiens GN=KRT37 PE=3 SV=3

Match to Query 30004: 2060.051832 from(687.691220,3+) intensity(1042116.9375) rtinseconds(2424) scans(13207) index(10353)
Title: 160219_Sunil_SDSI_A_Spectrum062474_scans__13207_RTINSECONDS=2424
Data file L:\\QE_2016\\160219_Sunil_KAP_LKC\\TMgf\\T\\T160219_Sunil_SDSI_A.mgf

Click mouse within plot area to zoom in by factor of two about that point
Or, Plot from 100 to 1600 Da Full range
Label all possible matches ○ Label matches used for scoring ◉
Show Y-axis ☐

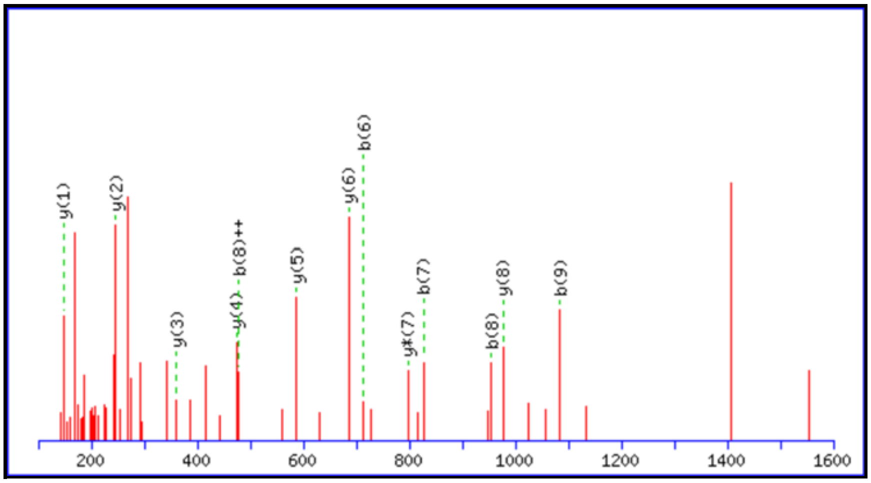

Monoisotopic mass of neutral peptide Mr(calc): 2060.0538
Fixed modifications: Carbamidomethyl (C) (apply to specified residues or termini only)
Ions Score: 44 **Expect:** 0.0065
Matches : 13/178 fragment ions using 21 most intense peaks (help)

#	b	b++	b*	b*++	b0	b0++	Seq.	y	y++	y*	y*++	y0	y0++	#
1	72.0444	36.5258					A							17
2	187.0713	94.0393			169.0608	85.0340	D	1990.0240	995.5156	1972.9974	987.0024	1972.0134	986.5104	16
3	300.1554	150.5813			282.1448	141.5761	L	1874.9971	938.0022	1857.9705	929.4889	1856.9865	928.9969	15
4	429.1980	215.1026			411.1874	206.0974	E	1761.9130	881.4601	1744.8864	872.9469	1743.9024	872.4549	14
5	585.2991	293.1532	568.2726	284.6399	567.2885	284.1479	R	1632.8704	816.9388	1615.8438	808.4256	1614.8598	807.9336	13
6	***713.3577***	357.1825	696.3311	348.6692	695.3471	348.1772	Q	1476.7693	738.8883	1459.7427	730.3750	1458.7587	729.8830	12
7	***827.4006***	414.2039	810.3741	405.6907	809.3900	405.1987	N	1348.7107	674.8590	1331.6842	666.3457	1330.7001	665.8537	11
8	***955.4592***	***478.2332***	938.4326	469.7200	937.4486	469.2279	Q	1234.6678	617.8375	1217.6412	609.3243	1216.6572	608.8322	10
9	***1084.5018***	542.7545	1067.4752	534.2413	1066.4912	533.7492	E	1106.6092	553.8082	1089.5827	545.2950	1088.5986	544.8030	9
10	1247.5651	624.2862	1230.5386	615.7729	1229.5545	615.2809	Y	***977.5666***	489.2869	960.5401	480.7737	959.5560	480.2817	8
11	1375.6237	688.3155	1358.5971	679.8022	1357.6131	679.3102	Q	814.5033	407.7553	797.4767	399.2420	796.4927	398.7500	7
12	1474.6921	737.8497	1457.6655	729.3364	1456.6815	728.8444	V	***686.4447***	343.7260	669.4182	335.2127	668.4341	334.7207	6
13	1587.7762	794.3917	1570.7496	785.8784	1569.7656	785.3864	L	***587.3763***	294.1918	570.3497	285.6785	569.3657	285.1865	5
14	1700.8602	850.9338	1683.8337	842.4205	1682.8497	841.9285	L	***474.2922***	237.6498	457.2657	229.1365	456.2817	228.6445	4
15	1815.8872	908.4472	1798.8606	899.9339	1797.8766	899.4419	D	***361.2082***	181.1077	344.1816	172.5944	343.1976	172.1024	3
16	1914.9556	957.9814	1897.9290	949.4682	1896.9450	948.9761	V	***246.1812***	123.5942	229.1547	115.0810			2
17							K	***147.1128***	74.0600	130.0863	65.5468			1

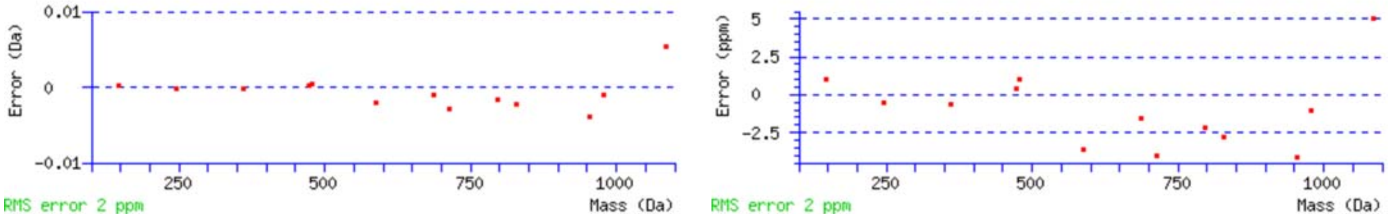

NCBI **BLAST** search of ADLERQNQEYQVLLDVK
(Parameters: blastp, nr protein database, expect=20000, no filter, PAM30)
Other BLAST web gateways

All matches to this query

Score	Mr(calc)	Delta	Sequence
44.3	2060.0538	-0.0020	ADLERQNQEYQVLLDVK
0.7	2059.0619	0.9899	EEVTPEQLKLMRTQDVK
0.3	2060.0441	0.0077	AFQPFFVELTMPYSVIR

Mascot: http://www.matrixscience.com/

Mascot Search Results

Peptide View

MS/MS Fragmentation of **VRQLEQENAELETTLLER**
Found in **ch17u_O76014|KRT37_HUMAN** in **uni_human**, Keratin, type I cuticular Ha7 OS=Homo sapiens GN=KRT37 PE=3 SV=3

Match to Query 31993: 2170.120752 from(724.380860,3+) intensity(307696.0000) rtinseconds(2382) scans(12966) index(10158)
Title: 160219_Sunil_SDSI_A_Spectrum062278_scans__12966_RTINSECONDS=2382
Data file L:\\QE_2016\\160219_Sunil_KAP_LKC\\TMgf\\T\\T160219_Sunil_SDSI_A.mgf

Click mouse within plot area to zoom in by factor of two about that point
Or, Plot from 100 to 1500 Da Full range
Label all possible matches ○ Label matches used for scoring ◉
Show Y-axis ☐

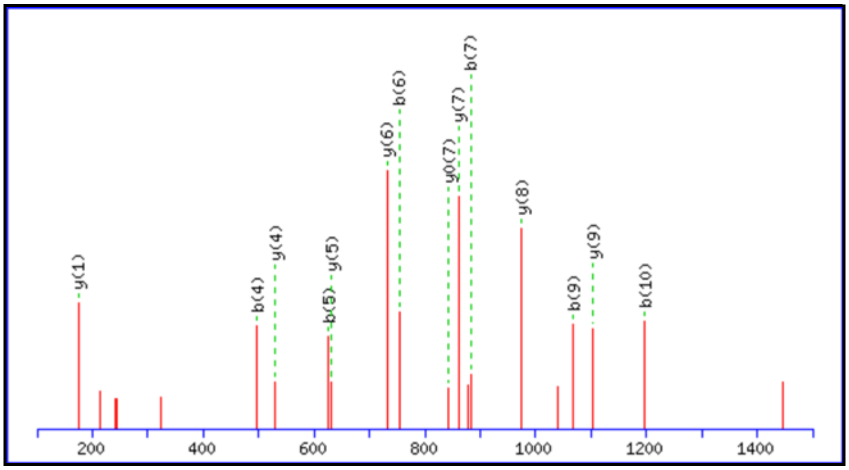

Monoisotopic mass of neutral peptide Mr(calc): 2170.1229
Fixed modifications: Carbamidomethyl (C) (apply to specified residues or termini only)
Ions Score: 67 **Expect:** 2.8e-005
Matches : 14/192 fragment ions using 18 most intense peaks (help)

#	b	b^{++}	b*	b^{*++}	b^0	b^{0++}	Seq.	y	y^{++}	y*	y^{*++}	y^0	y^{0++}	#
1	100.0757	50.5415					V							18
2	256.1768	128.5920	239.1503	120.0788			R	2072.0618	1036.5346	2055.0353	1028.0213	2054.0513	1027.5293	17
3	384.2354	192.6213	367.2088	184.1081			Q	1915.9607	958.4840	1898.9342	949.9707	1897.9502	949.4787	16
4	497.3194	249.1634	480.2929	240.6501			L	1787.9022	894.4547	1770.8756	885.9414	1769.8916	885.4494	15
5	626.3620	313.6847	609.3355	305.1714	608.3515	304.6794	E	1674.8181	837.9127	1657.7915	829.3994	1656.8075	828.9074	14
6	754.4206	377.7139	737.3941	369.2007	736.4100	368.7087	Q	1545.7755	773.3914	1528.7489	764.8781	1527.7649	764.3861	13
7	883.4632	442.2352	866.4367	433.7220	865.4526	433.2300	E	1417.7169	709.3621	1400.6904	700.8488	1399.7063	700.3568	12
8	997.5061	499.2567	980.4796	490.7434	979.4956	490.2514	N	1288.6743	644.8408	1271.6478	636.3275	1270.6638	635.8355	11
9	1068.5432	534.7753	1051.5167	526.2620	1050.5327	525.7700	A	1174.6314	587.8193	1157.6048	579.3061	1156.6208	578.8141	10
10	1197.5858	599.2966	1180.5593	590.7833	1179.5753	590.2913	E	1103.5943	552.3008	1086.5677	543.7875	1085.5837	543.2955	9
11	1310.6699	655.8386	1293.6434	647.3253	1292.6593	646.8333	L	974.5517	487.7795	957.5251	479.2662	956.5411	478.7742	8
12	1439.7125	720.3599	1422.6859	711.8466	1421.7019	711.3546	E	861.4676	431.2374	844.4411	422.7242	843.4571	422.2322	7
13	1540.7602	770.8837	1523.7336	762.3705	1522.7496	761.8784	T	732.4250	366.7162	715.3985	358.2029	714.4145	357.7109	6
14	1641.8079	821.4076	1624.7813	812.8943	1623.7973	812.4023	T	631.3774	316.1923	614.3508	307.6790	613.3668	307.1870	5
15	1754.8919	877.9496	1737.8654	869.4363	1736.8814	868.9443	L	530.3297	265.6685	513.3031	257.1552	512.3191	256.6632	4
16	1867.9760	934.4916	1850.9494	925.9784	1849.9654	925.4863	L	417.2456	209.1264	400.2191	200.6132	399.2350	200.1212	3
17	1997.0186	999.0129	1979.9920	990.4997	1979.0080	990.0076	E	304.1615	152.5844	287.1350	144.0711	286.1510	143.5791	2
18							R	175.1190	88.0631	158.0924	79.5498			1

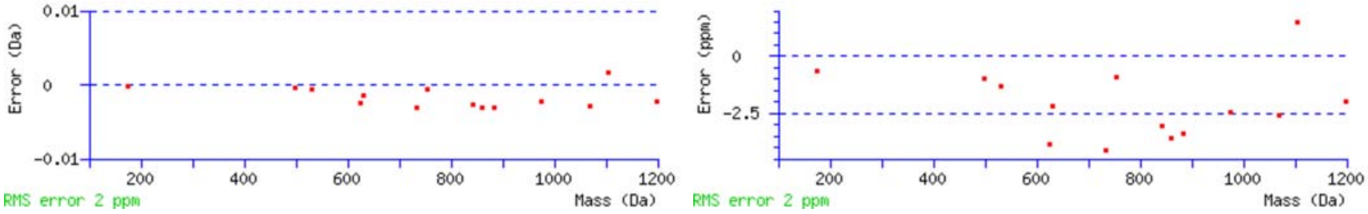

NCBI **BLAST** search of VRQLEQENAELETTLLER
(Parameters: blastp, nr protein database, expect=20000, no filter, PAM30)
Other BLAST web gateways

All matches to this query

Score	Mr(calc)	Delta	Sequence
67.3	2170.1229	-0.0022	VRQLEQENAELETTLLER
0.6	2170.1018	0.0189	EVGGGGVGGGLLENANPLIYQR
0.6	2170.1018	0.0189	QVGGGGVGGGLLENANPLIYQR
0.3	2168.1122	2.0085	QGLLFSTLLLAGLAQFCCR
0.3	2169.0962	1.0245	QGLLFSTLLLAGLAQFCCR

Mascot: http://www.matrixscience.com/

Mascot Search Results

Peptide View

MS/MS Fragmentation of **VRQLEQENAELETTLLER**
Found in **ch17u_O76014|KRT37_HUMAN** in **uni_human**, Keratin, type I cuticular Ha7 OS=Homo sapiens GN=KRT37 PE=3 SV=3

Match to Query 31993: 2170.120752 from(724.380860,3+) intensity(307696.0000) rtinseconds(2382) scans(12966) index(10158)
Title: 160219_Sunil_SDSI_A_Spectrum062278_scans__12966_RTINSECONDS=2382
Data file L:\\QE_2016\\160219_Sunil_KAP_LKC\\TMgf\\T\\T160219_Sunil_SDSI_A.mgf

Click mouse within plot area to zoom in by factor of two about that point
Or, Plot from 100 to 1500 Da Full range
Label all possible matches ○ Label matches used for scoring ◉
Show Y-axis ☐

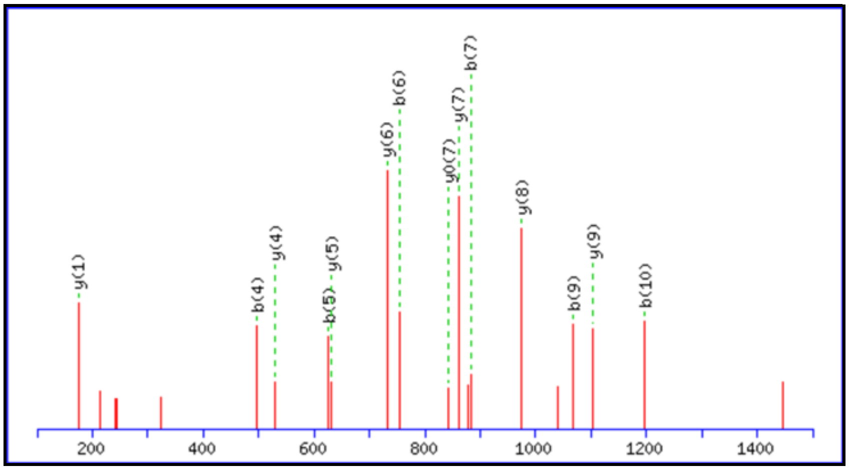

Monoisotopic mass of neutral peptide Mr(calc): 2170.1229
Fixed modifications: Carbamidomethyl (C) (apply to specified residues or termini only)
Ions Score: 67 **Expect:** 2.8e-005
Matches : 14/192 fragment ions using 18 most intense peaks (help)

#	b	b^{++}	b*	b^{*++}	b^0	b^{0++}	Seq.	y	y^{++}	y*	y^{*++}	y^0	y^{0++}	#
1	100.0757	50.5415					V							18
2	256.1768	128.5920	239.1503	120.0788			R	2072.0618	1036.5346	2055.0353	1028.0213	2054.0513	1027.5293	17
3	384.2354	192.6213	367.2088	184.1081			Q	1915.9607	958.4840	1898.9342	949.9707	1897.9502	949.4787	16
4	***497.3194***	249.1634	480.2929	240.6501			L	1787.9022	894.4547	1770.8756	885.9414	1769.8916	885.4494	15
5	***626.3620***	313.6847	609.3355	305.1714	608.3515	304.6794	E	1674.8181	837.9127	1657.7915	829.3994	1656.8075	828.9074	14
6	***754.4206***	377.7139	737.3941	369.2007	736.4100	368.7087	Q	1545.7755	773.3914	1528.7489	764.8781	1527.7649	764.3861	13
7	***883.4632***	442.2352	866.4367	433.7220	865.4526	433.2300	E	1417.7169	709.3621	1400.6904	700.8488	1399.7063	700.3568	12
8	997.5061	499.2567	980.4796	490.7434	979.4956	490.2514	N	1288.6743	644.8408	1271.6478	636.3275	1270.6638	635.8355	11
9	***1068.5432***	534.7753	1051.5167	526.2620	1050.5327	525.7700	A	1174.6314	587.8193	1157.6048	579.3061	1156.6208	578.8141	10
10	***1197.5858***	599.2966	1180.5593	590.7833	1179.5753	590.2913	E	***1103.5943***	552.3008	1086.5677	543.7875	1085.5837	543.2955	9
11	1310.6699	655.8386	1293.6434	647.3253	1292.6593	646.8333	L	***974.5517***	487.7795	957.5251	479.2662	956.5411	478.7742	8
12	1439.7125	720.3599	1422.6859	711.8466	1421.7019	711.3546	E	***861.4676***	431.2374	844.4411	422.7242	843.4571	422.2322	7
13	1540.7602	770.8837	1523.7336	762.3705	1522.7496	761.8784	T	***732.4250***	366.7162	715.3985	358.2029	714.4145	357.7109	6
14	1641.8079	821.4076	1624.7813	812.8943	1623.7973	812.4023	T	***631.3774***	316.1923	614.3508	307.6790	613.3668	307.1870	5
15	1754.8919	877.9496	1737.8654	869.4363	1736.8814	868.9443	L	***530.3297***	265.6685	513.3031	257.1552	512.3191	256.6632	4
16	1867.9760	934.4916	1850.9494	925.9784	1849.9654	925.4863	L	417.2456	209.1264	400.2191	200.6132	399.2350	200.1212	3
17	1997.0186	999.0129	1979.9920	990.4997	1979.0080	990.0076	E	304.1615	152.5844	287.1350	144.0711	286.1510	143.5791	2
18							R	***175.1190***	88.0631	158.0924	79.5498			1

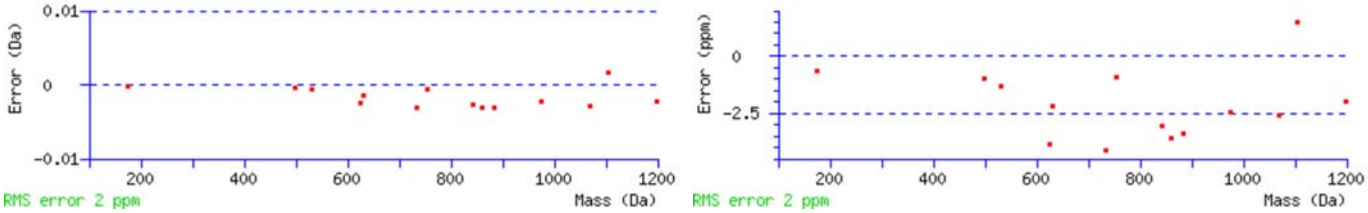

NCBI **BLAST** search of VRQLEQENAELETTLLER
(Parameters: blastp, nr protein database, expect=20000, no filter, PAM30)
Other BLAST web gateways

All matches to this query

Score	Mr(calc)	Delta	Sequence
67.3	2170.1229	-0.0022	VRQLEQENAELETTLLER
0.6	2170.1018	0.0189	EVGGGGVGGGLLENANPLIYQR
0.6	2170.1018	0.0189	QVGGGGVGGGLLENANPLIYQR
0.3	2168.1122	2.0085	QGLLFSTLLLAGLAQFCCR
0.3	2169.0962	1.0245	QGLLFSTLLLAGLAQFCCR

Mascot: http://www.matrixscience.com/

Mascot Search Results

Peptide View

MS/MS Fragmentation of **SLHQLVEADKCGTQK**
Found in **ch17u_O76014|KRT37_HUMAN** in **uni_human**, Keratin, type I cuticular Ha7 OS=Homo sapiens GN=KRT37 PE=3 SV=3

Match to Query 23141: 1712.852202 from(571.958010,3+) intensity(1212948.0000) rtinseconds(730) scans(3361) index(1983)
Title: 160219_Sunil_SDSI_A_Spectrum054091_scans__3361_RTINSECONDS=730
Data file L:\\QE_2016\\160219_Sunil_KAP_LKC\\TMgf\\T\\T160219_Sunil_SDSI_A.mgf

Click mouse within plot area to zoom in by factor of two about that point
Or, Plot from 100 to 1600 Da Full range
Label all possible matches ○ Label matches used for scoring ◉
Show Y-axis □

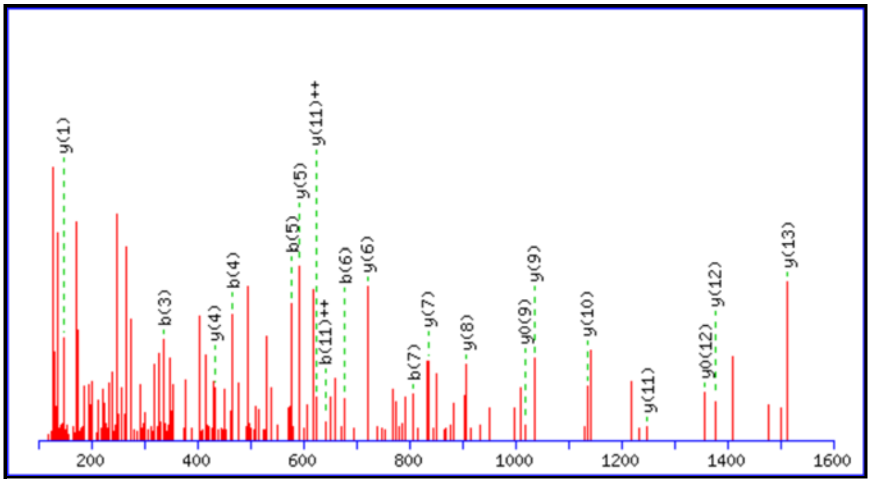

Monoisotopic mass of neutral peptide Mr(calc): 1712.8516
Fixed modifications: Carbamidomethyl (C) (apply to specified residues or termini only)
Ions Score: 48 **Expect:** 0.0021
Matches : 20/158 fragment ions using 60 most intense peaks (help)

#	b	b^{++}	b*	b^{*++}	b^0	b^{0++}	Seq.	y	y^{++}	y*	y^{*++}	y^0	y^{0++}	#
1	88.0393	44.5233			70.0287	35.5180	S							15
2	201.1234	101.0653			183.1128	92.0600	L	1626.8268	813.9170	1609.8003	805.4038	1608.8163	804.9118	14
3	**338.1823**	169.5948			320.1717	160.5895	H	***1513.7428***	757.3750	1496.7162	748.8617	1495.7322	748.3697	13
4	**466.2409**	233.6241	449.2143	225.1108	448.2303	224.6188	Q	***1376.6838***	688.8456	1359.6573	680.3323	1358.6733	679.8403	12
5	**579.3249**	290.1661	562.2984	281.6528	561.3144	281.1608	L	***1248.6253***	624.8163	1231.5987	616.3030	1230.6147	615.8110	11
6	**678.3933**	339.7003	661.3668	331.1870	660.3828	330.6950	V	***1135.5412***	568.2742	1118.5147	559.7610	1117.5306	559.2690	10
7	**807.4359**	404.2216	790.4094	395.7083	789.4254	395.2163	E	***1036.4728***	518.7400	1019.4462	510.2268	1018.4622	509.7347	9
8	878.4730	439.7402	861.4465	431.2269	860.4625	430.7349	A	***907.4302***	454.2187	890.4036	445.7055	889.4196	445.2135	8
9	993.5000	497.2536	976.4734	488.7404	975.4894	488.2483	D	***836.3931***	418.7002	819.3665	410.1869	818.3825	409.6949	7
10	1121.5949	561.3011	1104.5684	552.7878	1103.5844	552.2958	K	***721.3661***	361.1867	704.3396	352.6734	703.3556	352.1814	6
11	1281.6256	**641.3164**	1264.5990	632.8032	1263.6150	632.3112	C	***593.2712***	297.1392	576.2446	288.6260	575.2606	288.1339	5
12	1338.6471	669.8272	1321.6205	661.3139	1320.6365	660.8219	G	***433.2405***	217.1239	416.2140	208.6106	415.2300	208.1186	4
13	1439.6947	720.3510	1422.6682	711.8377	1421.6842	711.3457	T	376.2191	188.6132	359.1925	180.0999	358.2085	179.6079	3
14	1567.7533	784.3803	1550.7268	775.8670	1549.7428	775.3750	Q	275.1714	138.0893	258.1448	129.5761			2
15							K	***147.1128***	74.0600	130.0863	65.5468			1

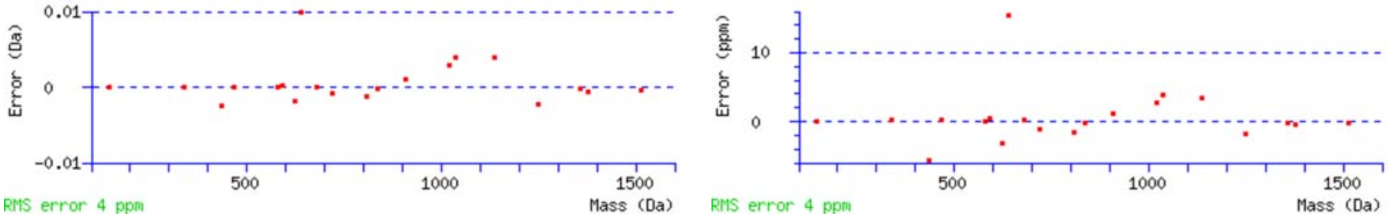

NCBI **BLAST** search of SLHQLVEADKCGTQK
(Parameters: blastp, nr protein database, expect=20000, no filter, PAM30)
Other BLAST web gateways

All matches to this query

Score	Mr(calc)	Delta	Sequence
48.3	1712.8516	0.0006	SLHQLVEADKCGTQK

Mascot: http://www.matrixscience.com/

Mascot Search Results

Peptide View

MS/MS Fragmentation of **LIVQIDNAKLAADDFR**
Found in **ch17u_O76014|KRT37_HUMAN** in **uni_human**, Keratin, type I cuticular Ha7 OS=Homo sapiens GN=KRT37 PE=3 SV=3

Match to Query 24767: 1800.976092 from(601.332640,3+) intensity(527309.5000) rtinseconds(2546) scans(13914) index(10898)
Title: 160219_Sunil_SDSI_A_Spectrum063023_scans__13914_RTINSECONDS=2546
Data file L:\\QE_2016\\160219_Sunil_KAP_LKC\\TMgf\\T\\T160219_Sunil_SDSI_A.mgf

Click mouse within plot area to zoom in by factor of two about that point
Or, Plot from 100 to 1400 Da Full range
Label all possible matches ○ Label matches used for scoring ◉
Show Y-axis ☐

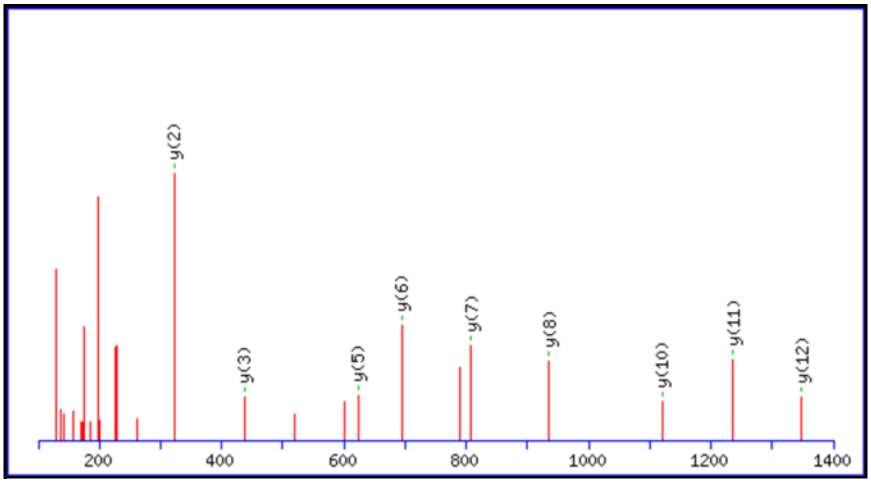

Monoisotopic mass of neutral peptide Mr(calc): 1800.9734
Fixed modifications: Carbamidomethyl (C) (apply to specified residues or termini only)
Ions Score: 86 **Expect:** 3e-007
Matches : 9/160 fragment ions using 10 most intense peaks (help)

#	b	b^{++}	b*	b^{*++}	b^0	b^{0++}	Seq.	y	y^{++}	y*	y^{*++}	y^0	y^{0++}	#
1	114.0913	57.5493					L							16
2	227.1754	114.0913					I	1688.8966	844.9519	1671.8701	836.4387	1670.8860	835.9467	15
3	326.2438	163.6255					V	1575.8125	788.4099	1558.7860	779.8966	1557.8020	779.4046	14
4	454.3024	227.6548	437.2758	219.1416			Q	1476.7441	738.8757	1459.7176	730.3624	1458.7336	729.8704	13
5	567.3865	284.1969	550.3599	275.6836			I	***1348.6856***	674.8464	1331.6590	666.3331	1330.6750	665.8411	12
6	682.4134	341.7103	665.3869	333.1971	664.4028	332.7051	D	***1235.6015***	618.3044	1218.5749	609.7911	1217.5909	609.2991	11
7	796.4563	398.7318	779.4298	390.2185	778.4458	389.7265	N	***1120.5745***	560.7909	1103.5480	552.2776	1102.5640	551.7856	10
8	867.4934	434.2504	850.4669	425.7371	849.4829	425.2451	A	1006.5316	503.7694	989.5051	495.2562	988.5211	494.7642	9
9	995.5884	498.2978	978.5619	489.7846	977.5778	489.2926	K	***935.4945***	468.2509	918.4680	459.7376	917.4839	459.2456	8
10	1108.6725	554.8399	1091.6459	546.3266	1090.6619	545.8346	L	***807.3995***	404.2034	790.3730	395.6901	789.3890	395.1981	7
11	1179.7096	590.3584	1162.6830	581.8452	1161.6990	581.3531	A	***694.3155***	347.6614	677.2889	339.1481	676.3049	338.6561	6
12	1250.7467	625.8770	1233.7201	617.3637	1232.7361	616.8717	A	***623.2784***	312.1428	606.2518	303.6295	605.2678	303.1375	5
13	1365.7736	683.3905	1348.7471	674.8772	1347.7631	674.3852	D	552.2413	276.6243	535.2147	268.1110	534.2307	267.6190	4
14	1480.8006	740.9039	1463.7740	732.3907	1462.7900	731.8986	D	***437.2143***	219.1108	420.1878	210.5975	419.2037	210.1055	3
15	1627.8690	814.4381	1610.8425	805.9249	1609.8584	805.4329	F	***322.1874***	161.5973	305.1608	153.0840			2
16							R	175.1190	88.0631	158.0924	79.5498			1

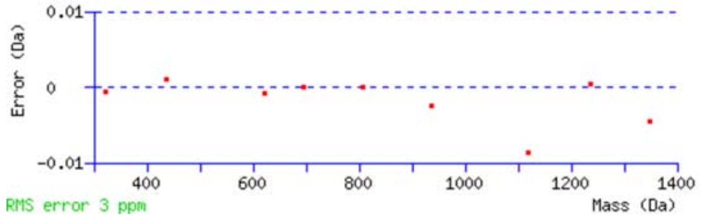

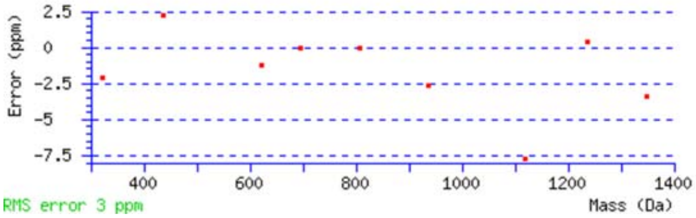

NCBI **BLAST** search of LIVQIDNAKLAADDFR
(Parameters: blastp, nr protein database, expect=20000, no filter, PAM30)
Other BLAST web gateways

All matches to this query

Score	Mr(calc)	Delta	Sequence
85.7	1800.9734	0.0027	LIVQIDNAKLAADDFR
85.7	1800.9734	0.0027	LVLQIDNAKLAADDFR
0.9	1800.9635	0.0126	STVNKWTLKGIHNFR
0.4	1799.9716	1.0045	AGQKPWLLVIRSMER

Mascot: http://www.matrixscience.com/

Mascot Search Results

Peptide View

MS/MS Fragmentation of **LIVQIDNAKLAADDFR**
Found in **ch17u_O76014|KRT37_HUMAN** in **uni_human**, Keratin, type I cuticular Ha7 OS=Homo sapiens GN=KRT37 PE=3 SV=3

Match to Query 24767: 1800.976092 from(601.332640,3+) intensity(527309.5000) rtinseconds(2546) scans(13914) index(10898)
Title: 160219_Sunil_SDSI_A_Spectrum063023_scans__13914_RTINSECONDS=2546
Data file L:\\QE_2016\\160219_Sunil_KAP_LKC\\TMgf\\T\\T160219_Sunil_SDSI_A.mgf

Click mouse within plot area to zoom in by factor of two about that point
Or, Plot from 100 to 1400 Da Full range
Label all possible matches ○ Label matches used for scoring ◉
Show Y-axis ☐

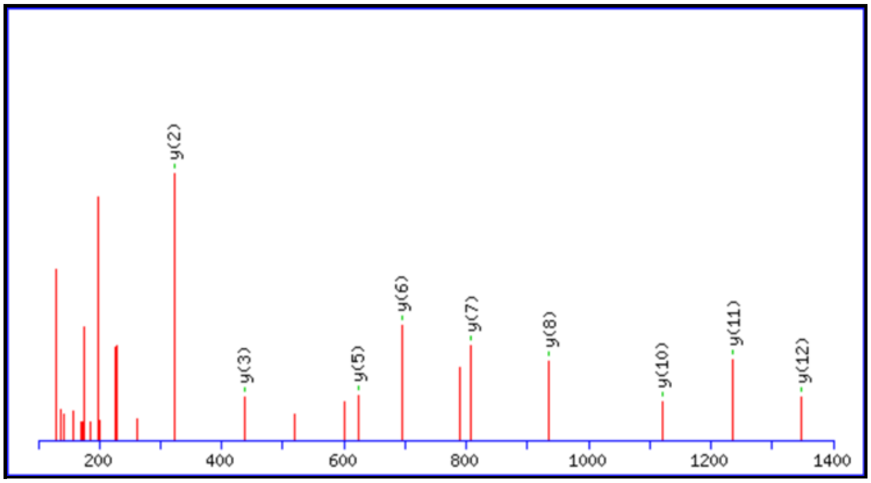

Monoisotopic mass of neutral peptide Mr(calc): 1800.9734
Fixed modifications: Carbamidomethyl (C) (apply to specified residues or termini only)
Ions Score: 86 **Expect:** 3e-007
Matches : 9/160 fragment ions using 10 most intense peaks (help)

#	b	b^{++}	b*	b^{*++}	b^0	b^{0++}	Seq.	y	y^{++}	y*	y^{*++}	y^0	y^{0++}	#
1	114.0913	57.5493					L							16
2	227.1754	114.0913					I	1688.8966	844.9519	1671.8701	836.4387	1670.8860	835.9467	15
3	326.2438	163.6255					V	1575.8125	788.4099	1558.7860	779.8966	1557.8020	779.4046	14
4	454.3024	227.6548	437.2758	219.1416			Q	1476.7441	738.8757	1459.7176	730.3624	1458.7336	729.8704	13
5	567.3865	284.1969	550.3599	275.6836			I	***1348.6856***	674.8464	1331.6590	666.3331	1330.6750	665.8411	12
6	682.4134	341.7103	665.3869	333.1971	664.4028	332.7051	D	***1235.6015***	618.3044	1218.5749	609.7911	1217.5909	609.2991	11
7	796.4563	398.7318	779.4298	390.2185	778.4458	389.7265	N	***1120.5745***	560.7909	1103.5480	552.2776	1102.5640	551.7856	10
8	867.4934	434.2504	850.4669	425.7371	849.4829	425.2451	A	1006.5316	503.7694	989.5051	495.2562	988.5211	494.7642	9
9	995.5884	498.2978	978.5619	489.7846	977.5778	489.2926	K	***935.4945***	468.2509	918.4680	459.7376	917.4839	459.2456	8
10	1108.6725	554.8399	1091.6459	546.3266	1090.6619	545.8346	L	***807.3995***	404.2034	790.3730	395.6901	789.3890	395.1981	7
11	1179.7096	590.3584	1162.6830	581.8452	1161.6990	581.3531	A	***694.3155***	347.6614	677.2889	339.1481	676.3049	338.6561	6
12	1250.7467	625.8770	1233.7201	617.3637	1232.7361	616.8717	A	***623.2784***	312.1428	606.2518	303.6295	605.2678	303.1375	5
13	1365.7736	683.3905	1348.7471	674.8772	1347.7631	674.3852	D	552.2413	276.6243	535.2147	268.1110	534.2307	267.6190	4
14	1480.8006	740.9039	1463.7740	732.3907	1462.7900	731.8986	D	***437.2143***	219.1108	420.1878	210.5975	419.2037	210.1055	3
15	1627.8690	814.4381	1610.8425	805.9249	1609.8584	805.4329	F	***322.1874***	161.5973	305.1608	153.0840			2
16							R	175.1190	88.0631	158.0924	79.5498			1

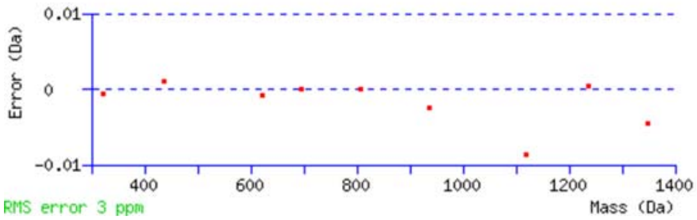

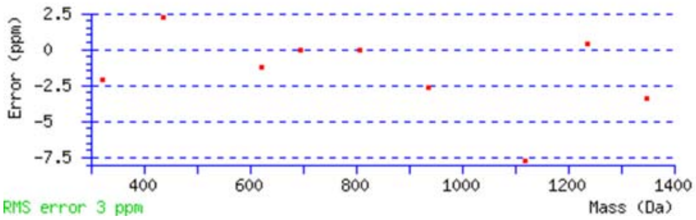

NCBI **BLAST** search of LIVQIDNAKLAADDFR
(Parameters: blastp, nr protein database, expect=20000, no filter, PAM30)
Other BLAST web gateways

All matches to this query

Score	Mr(calc)	Delta	Sequence
85.7	1800.9734	0.0027	LIVQIDNAKLAADDFR
85.7	1800.9734	0.0027	LVLQIDNAKLAADDFR
0.9	1800.9635	0.0126	STVNKWTLKGIHNFR
0.4	1799.9716	1.0045	AGQKPWLLVIRSMER

Mascot: http://www.matrixscience.com/

Mascot Search Results

Peptide View

MS/MS Fragmentation of **LIVQIDNAKLAADDFR**
Found in **ch17u_O76014|KRT37_HUMAN** in **uni_human**, Keratin, type I cuticular Ha7 OS=Homo sapiens GN=KRT37 PE=3 SV=3

Match to Query 24789: 1801.975302 from(601.665710,3+) intensity(1111974.1250) rtinseconds(2432) scans(13398) index(25884)
Title: 160219_Sunil_SDSI_A_Spectrum079190_scans__13398_RTINSECONDS=2432
Data file L:\\QE_2016\\160219_Sunil_KAP_LKC\\TMgf\\T\\T160219_Sunil_SDSI_A.mgf

Click mouse within plot area to zoom in by factor of two about that point
Or, Plot from 100 to 1300 Da Full range
Label all possible matches ○ Label matches used for scoring ◉
Show Y-axis ☐

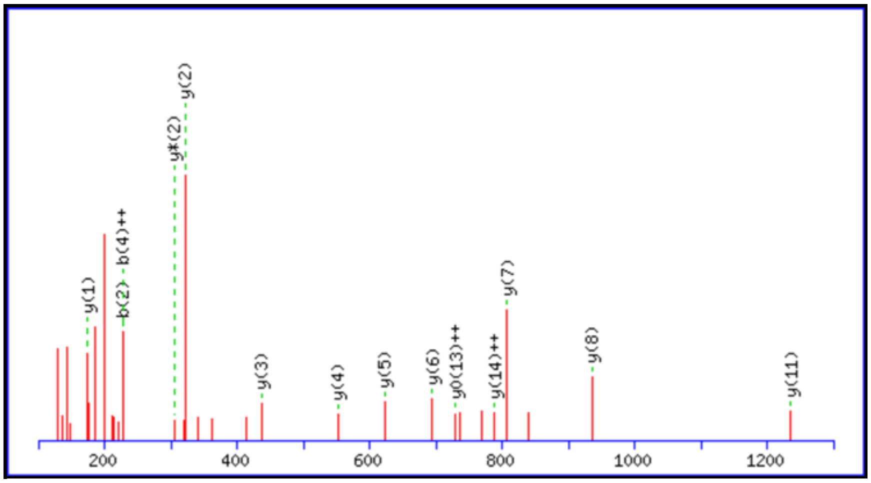

Monoisotopic mass of neutral peptide Mr(calc): 1801.9574
Fixed modifications: Carbamidomethyl (C) (apply to specified residues or termini only)
Variable modifications:
Q4 : Deamidated (NQ)
Ions Score: 56 **Expect:** 0.00026
Matches : 14/160 fragment ions using 26 most intense peaks (help)

#	b	b++	b*	b*++	b0	b0++	Seq.	y	y++	y*	y*++	y0	y0++	#
1	114.0913	57.5493					L							16
2	227.1754	114.0913					I	1689.8806	845.4440	1672.8541	836.9307	1671.8701	836.4387	15
3	326.2438	163.6255					V	1576.7966	788.9019	1559.7700	780.3886	1558.7860	779.8966	14
4	455.2864	228.1468	438.2599	219.6336			Q	1477.7281	739.3677	1460.7016	730.8544	1459.7176	730.3624	13
5	568.3705	284.6889	551.3439	276.1756			I	1348.6856	674.8464	1331.6590	666.3331	1330.6750	665.8411	12
6	683.3974	342.2023	666.3709	333.6891	665.3869	333.1971	D	1235.6015	618.3044	1218.5749	609.7911	1217.5909	609.2991	11
7	797.4403	399.2238	780.4138	390.7105	779.4298	390.2185	N	1120.5745	560.7909	1103.5480	552.2776	1102.5640	551.7856	10
8	868.4775	434.7424	851.4509	426.2291	850.4669	425.7371	A	1006.5316	503.7694	989.5051	495.2562	988.5211	494.7642	9
9	996.5724	498.7898	979.5459	490.2766	978.5619	489.7846	K	935.4945	468.2509	918.4680	459.7376	917.4839	459.2456	8
10	1109.6565	555.3319	1092.6299	546.8186	1091.6459	546.3266	L	807.3995	404.2034	790.3730	395.6901	789.3890	395.1981	7
11	1180.6936	590.8504	1163.6671	582.3372	1162.6830	581.8452	A	694.3155	347.6614	677.2889	339.1481	676.3049	338.6561	6
12	1251.7307	626.3690	1234.7042	617.8557	1233.7201	617.3637	A	623.2784	312.1428	606.2518	303.6295	605.2678	303.1375	5
13	1366.7577	683.8825	1349.7311	675.3692	1348.7471	674.8772	D	552.2413	276.6243	535.2147	268.1110	534.2307	267.6190	4
14	1481.7846	741.3959	1464.7581	732.8827	1463.7740	732.3907	D	437.2143	219.1108	420.1878	210.5975	419.2037	210.1055	3
15	1628.8530	814.9301	1611.8265	806.4169	1610.8425	805.9249	F	322.1874	161.5973	305.1608	153.0840			2
16							R	175.1190	88.0631	158.0924	79.5498			1

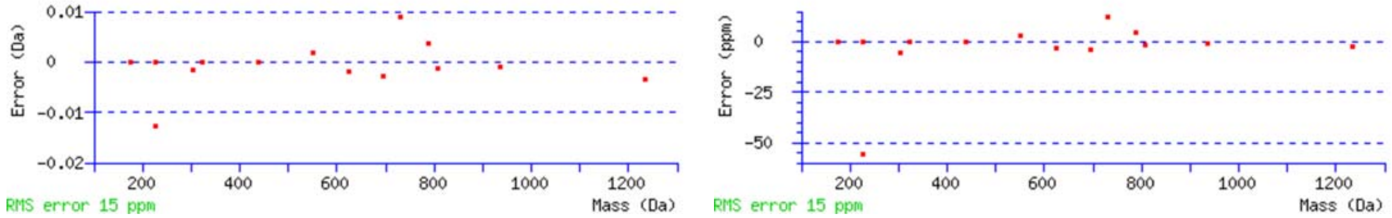

NCBI **BLAST** search of LIVQIDNAKLAADDFR
(Parameters: blastp, nr protein database, expect=20000, no filter, PAM30)
Other BLAST web gateways

All matches to this query

Score	Mr(calc)	Delta	Sequence	Site Analysis
56.2	1801.9574	0.0179	LIVQIDNAKLAADDFR	Deamidated Q4 96.50%
53.9	1800.9734	1.0019	LIVQIDNAKLAADDFR	
53.9	1800.9734	1.0019	LVLQIDNAKLAADDFR	
53.9	1801.9574	0.0179	LVLQIDNAKLAADDFR	
41.8	1801.9574	0.0179	LIVQIDNAKLAADDFR	Deamidated N7 3.50%
40.7	1801.9574	0.0179	LVLQIDNAKLAADDFR	

Mascot: http://www.matrixscience.com/

Mascot Search Results

Peptide View

MS/MS Fragmentation of **TIEELQQKILCSK**
Found in **ch17u_O76014|KRT37_HUMAN** in **uni_human**, Keratin, type I cuticular Ha7 OS=Homo sapiens GN=KRT37 PE=3 SV=3

Match to Query 20249: 1588.848972 from(530.623600,3+) intensity(2640728.7500) rtinseconds(1992) scans(10839) index(23789)
Title: 160219_Sunil_SDSI_A_Spectrum077094_scans__10839_RTINSECONDS=1992
Data file L:\\QE_2016\\160219_Sunil_KAP_LKC\\TMgf\\T\\T160219_Sunil_SDSI_A.mgf

Click mouse within plot area to zoom in by factor of two about that point
Or, Plot from 100 to 1600 Da Full range
Label all possible matches ○ Label matches used for scoring ◉
Show Y-axis ☐

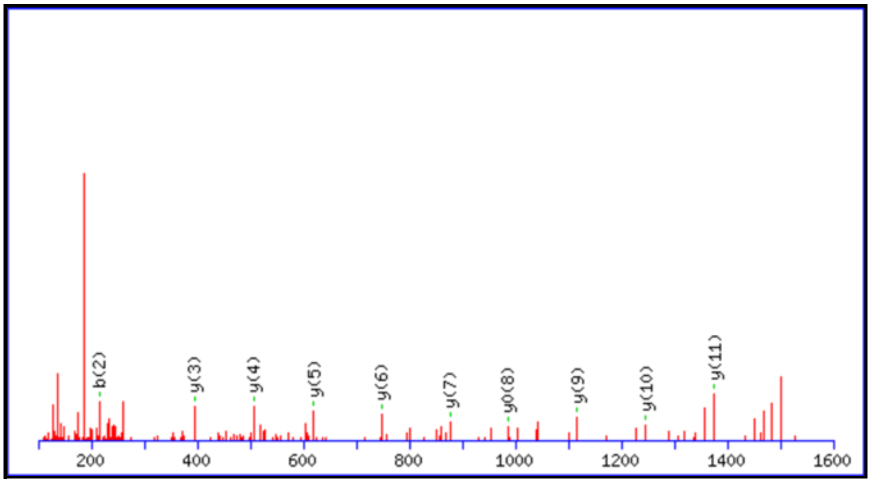

Monoisotopic mass of neutral peptide Mr(calc): 1588.8494
Fixed modifications: Carbamidomethyl (C) (apply to specified residues or termini only)
Ions Score: 57 **Expect:** 0.0003
Matches : 10/132 fragment ions using 15 most intense peaks (help)

#	b	b^{++}	b*	b^{*++}	b^0	b^{0++}	Seq.	y	y^{++}	y*	y^{*++}	y^0	y^{0++}	#
1	102.0550	51.5311			84.0444	42.5258	T							13
2	**215.1390**	108.0731			197.1285	99.0679	I	1488.8090	744.9082	1471.7825	736.3949	1470.7985	735.9029	12
3	344.1816	172.5944			326.1710	163.5892	E	***1375.7250***	688.3661	1358.6984	679.8529	1357.7144	679.3608	11
4	473.2242	237.1157			455.2136	228.1105	E	***1246.6824***	623.8448	1229.6558	615.3316	1228.6718	614.8395	10
5	586.3083	293.6578			568.2977	284.6525	L	***1117.6398***	559.3235	1100.6132	550.8103	1099.6292	550.3183	9
6	714.3668	357.6871	697.3403	349.1738	696.3563	348.6818	Q	1004.5557	502.7815	987.5292	494.2682	986.5452	493.7762	8
7	842.4254	421.7164	825.3989	413.2031	824.4149	412.7111	Q	***876.4972***	438.7522	859.4706	430.2389	858.4866	429.7469	7
8	970.5204	485.7638	953.4938	477.2506	952.5098	476.7585	K	***748.4386***	374.7229	731.4120	366.2096	730.4280	365.7176	6
9	1083.6045	542.3059	1066.5779	533.7926	1065.5939	533.3006	I	***620.3436***	310.6754	603.3171	302.1622	602.3330	301.6702	5
10	1196.6885	598.8479	1179.6620	590.3346	1178.6780	589.8426	L	***507.2595***	254.1334	490.2330	245.6201	489.2490	245.1281	4
11	1356.7192	678.8632	1339.6926	670.3499	1338.7086	669.8579	C	***394.1755***	197.5914	377.1489	189.0781	376.1649	188.5861	3
12	1443.7512	722.3792	1426.7246	713.8660	1425.7406	713.3740	S	234.1448	117.5761	217.1183	109.0628	216.1343	108.5708	2
13							K	147.1128	74.0600	130.0863	65.5468			1

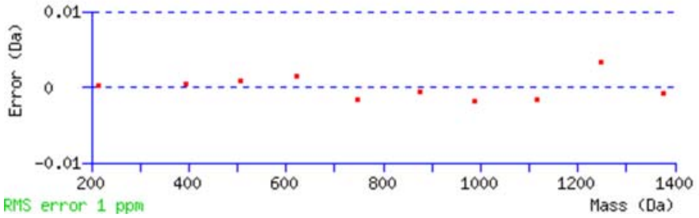

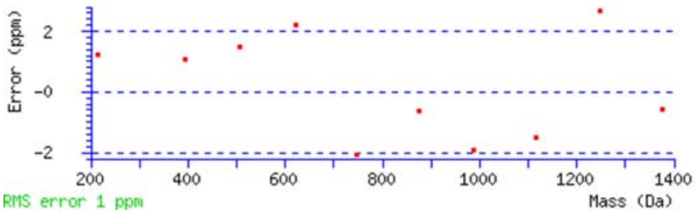

NCBI **BLAST** search of TIEELQQKILCSK
(Parameters: blastp, nr protein database, expect=20000, no filter, PAM30)
Other BLAST web gateways

All matches to this query

Score	Mr(calc)	Delta	Sequence
57.0	1588.8494	-0.0004	TIEELQQKILCSK

Mascot: http://www.matrixscience.com/

Mascot Search Results

Peptide View

MS/MS Fragmentation of **LENEIATYR**
Found in **ch17u_O76014|KRT37_HUMAN** in **uni_human**, Keratin, type I cuticular Ha7 OS=Homo sapiens GN=KRT37 PE=3 SV=3

Match to Query 5387: 1107.556488 from(554.785520,2+) intensity(1763561.8750) rtinseconds(1443) scans(7485) index(5450)
Title: 160219_Sunil_SDSI_A_Spectrum057558_scans__7485_RTINSECONDS=1443
Data file L:\\QE_2016\\160219_Sunil_KAP_LKC\\TMgf\\T\\T160219_Sunil_SDSI_A.mgf

Click mouse within plot area to zoom in by factor of two about that point
Or, Plot from 0 to 1200 Da Full range
Label all possible matches ○ Label matches used for scoring ◉
Show Y-axis ☐

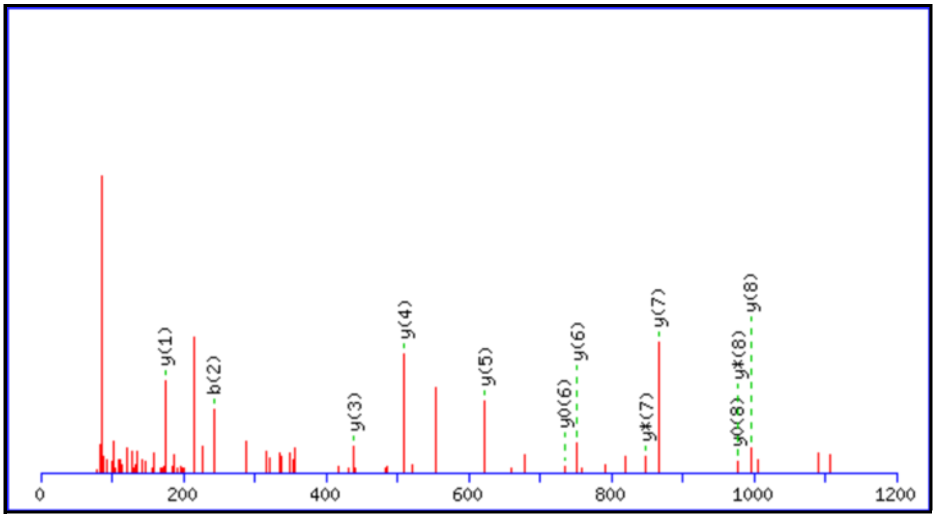

Monoisotopic mass of neutral peptide Mr(calc): 1107.5560
Fixed modifications: Carbamidomethyl (C) (apply to specified residues or termini only)
Ions Score: 49 **Expect:** 0.0016
Matches : 12/86 fragment ions using 22 most intense peaks (help)

#	b	b^{++}	b*	b^{*++}	b^0	b^{0++}	Seq.	y	y^{++}	y*	y^{*++}	y^0	y^{0++}	#
1	114.0913	57.5493					L							9
2	**243.1339**	122.0706			225.1234	113.0653	E	***995.4793***	498.2433	978.4527	489.7300	977.4687	489.2380	8
3	357.1769	179.0921	340.1503	170.5788	339.1663	170.0868	N	***866.4367***	433.7220	849.4101	425.2087	848.4261	424.7167	7
4	486.2195	243.6134	469.1929	235.1001	468.2089	234.6081	E	***752.3937***	376.7005	735.3672	368.1872	734.3832	367.6952	6
5	599.3035	300.1554	582.2770	291.6421	581.2930	291.1501	I	***623.3511***	312.1792	606.3246	303.6659	605.3406	303.1739	5
6	670.3406	335.6740	653.3141	327.1607	652.3301	326.6687	A	***510.2671***	255.6372	493.2405	247.1239	492.2565	246.6319	4
7	771.3883	386.1978	754.3618	377.6845	753.3777	377.1925	T	***439.2300***	220.1186	422.2034	211.6053	421.2194	211.1133	3
8	934.4516	467.7295	917.4251	459.2162	916.4411	458.7242	Y	338.1823	169.5948	321.1557	161.0815			2
9							R	***175.1190***	88.0631	158.0924	79.5498			1

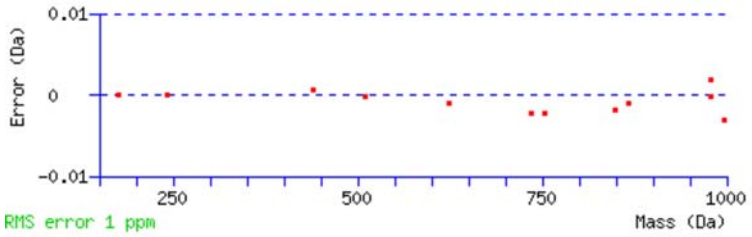

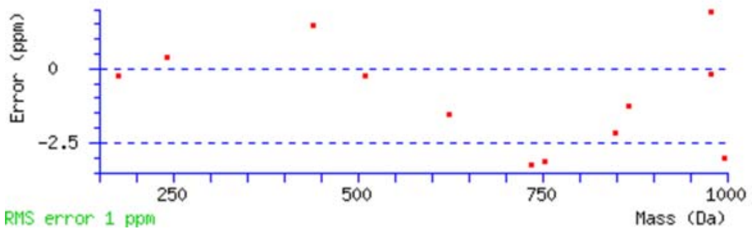

NCBI **BLAST** search of LENEIATYR
(Parameters: blastp, nr protein database, expect=20000, no filter, PAM30)
Other BLAST web gateways

All matches to this query

Score	Mr(calc)	Delta	Sequence
49.4	1107.5560	0.0005	LENEIATYR
11.1	1107.5560	0.0005	ELGGYVSNLR
5.1	1107.5560	0.0005	LYSSSGPELR
4.8	1107.5594	-0.0029	IEEMLSKSR
4.8	1107.5594	-0.0029	LQSMLKSER
3.9	1107.5672	-0.0108	IQESHPELR
3.6	1106.5641	0.9923	LTTEVMQIR
3.1	1107.5560	0.0005	LTFNITAGNR
0.6	1107.5495	0.0070	LCTIPHPDR
0.2	1107.5560	0.0005	LGNEVGYSIR

Mascot: http://www.matrixscience.com/

Mascot Search Results

Peptide View

MS/MS Fragmentation of **TIEELQQKILCSK**
Found in **ch17u_O76014|KRT37_HUMAN** in **uni_human**, Keratin, type I cuticular Ha7 OS=Homo sapiens GN=KRT37 PE=3 SV=3

Match to Query 20248: 1588.847268 from(795.430910,2+) intensity(1868119.0000) rtinseconds(1997) scans(10866) index(23813)
Title: 160219_Sunil_SDSI_A_Spectrum077118_scans__10866_RTINSECONDS=1997
Data file L:\\QE_2016\\160219_Sunil_KAP_LKC\\TMgf\\T\\T160219_Sunil_SDSI_A.mgf

Click mouse within plot area to zoom in by factor of two about that point
Or, Plot from 100 to 1600 Da Full range
Label all possible matches ○ Label matches used for scoring ◉
Show Y-axis □

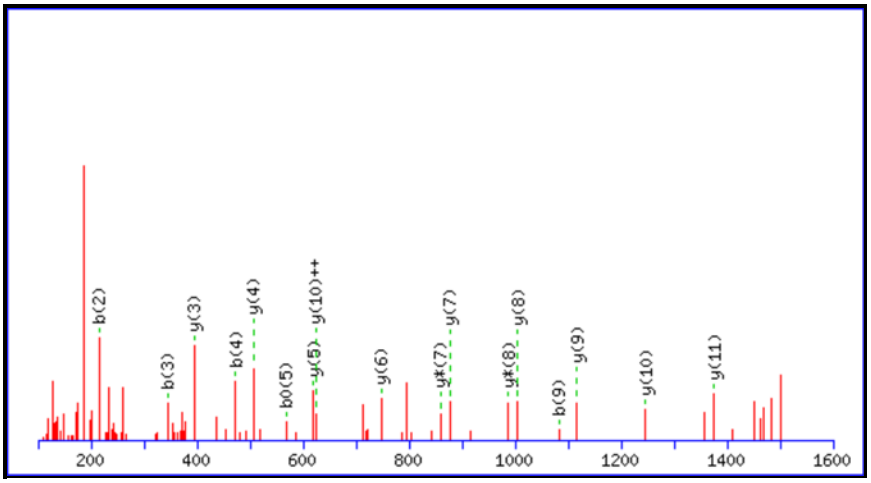

Monoisotopic mass of neutral peptide Mr(calc): 1588.8494
Fixed modifications: Carbamidomethyl (C) (apply to specified residues or termini only)
Ions Score: 70 **Expect:** 1.7e-005
Matches : 17/132 fragment ions using 26 most intense peaks (help)

#	b	b^{++}	b^{*}	b^{*++}	b^{0}	b^{0++}	Seq.	y	y^{++}	y^{*}	y^{*++}	y^{0}	y^{0++}	#
1	102.0550	51.5311			84.0444	42.5258	T							13
2	**215.1390**	108.0731			197.1285	99.0679	I	1488.8090	744.9082	1471.7825	736.3949	1470.7985	735.9029	12
3	**344.1816**	172.5944			326.1710	163.5892	E	***1375.7250***	688.3661	1358.6984	679.8529	1357.7144	679.3608	11
4	**473.2242**	237.1157			455.2136	228.1105	E	***1246.6824***	623.8448	1229.6558	615.3316	1228.6718	614.8395	10
5	586.3083	293.6578			568.2977	284.6525	L	***1117.6398***	559.3235	1100.6132	550.8103	1099.6292	550.3183	9
6	714.3668	357.6871	697.3403	349.1738	696.3563	348.6818	Q	***1004.5557***	502.7815	987.5292	494.2682	986.5452	493.7762	8
7	842.4254	421.7164	825.3989	413.2031	824.4149	412.7111	Q	***876.4972***	438.7522	859.4706	430.2389	858.4866	429.7469	7
8	970.5204	485.7638	953.4938	477.2506	952.5098	476.7585	K	***748.4386***	374.7229	731.4120	366.2096	730.4280	365.7176	6
9	**1083.6045**	542.3059	1066.5779	533.7926	1065.5939	533.3006	I	***620.3436***	310.6754	603.3171	302.1622	602.3330	301.6702	5
10	1196.6885	598.8479	1179.6620	590.3346	1178.6780	589.8426	L	***507.2595***	254.1334	490.2330	245.6201	489.2490	245.1281	4
11	1356.7192	678.8632	1339.6926	670.3499	1338.7086	669.8579	C	***394.1755***	197.5914	377.1489	189.0781	376.1649	188.5861	3
12	1443.7512	722.3792	1426.7246	713.8660	1425.7406	713.3740	S	234.1448	117.5761	217.1183	109.0628	216.1343	108.5708	2
13							K	147.1128	74.0600	130.0863	65.5468			1

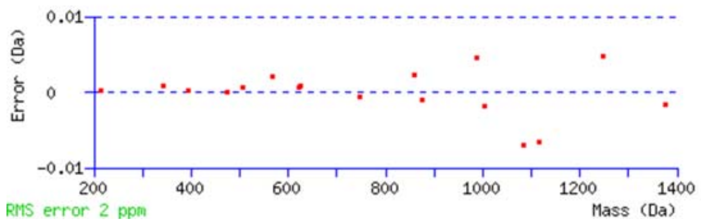

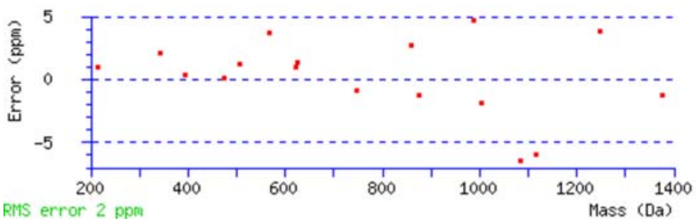

NCBI **BLAST** search of TIEELQQKILCSK
(Parameters: blastp, nr protein database, expect=20000, no filter, PAM30)
Other BLAST web gateways

All matches to this query

Score	Mr(calc)	Delta	Sequence
69.8	1588.8494	-0.0021	TIEELQQKILCSK

Mascot: http://www.matrixscience.com/

Mascot Search Results

Peptide View

MS/MS Fragmentation of **TIEELQQKILCSK**
Found in **ch17u_O76014|KRT37_HUMAN** in **uni_human**, Keratin, type I cuticular Ha7 OS=Homo sapiens GN=KRT37 PE=3 SV=3

Match to Query 20248: 1588.847268 from(795.430910,2+) intensity(1868119.0000) rtinseconds(1997) scans(10866) index(23813)
Title: 160219_Sunil_SDSI_A_Spectrum077118_scans__10866_RTINSECONDS=1997
Data file L:\\QE_2016\\160219_Sunil_KAP_LKC\\TMgf\\T\\T160219_Sunil_SDSI_A.mgf

Click mouse within plot area to zoom in by factor of two about that point
Or, Plot from 100 to 1600 Da Full range
Label all possible matches ○ Label matches used for scoring ◉
Show Y-axis ☐

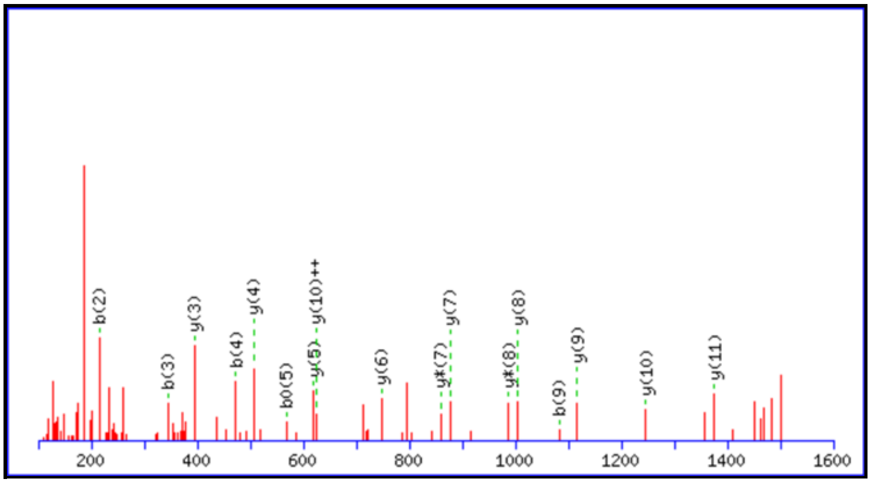

Monoisotopic mass of neutral peptide Mr(calc): 1588.8494
Fixed modifications: Carbamidomethyl (C) (apply to specified residues or termini only)
Ions Score: 70 **Expect:** 1.7e-005
Matches : 17/132 fragment ions using 26 most intense peaks (help)

#	b	b^{++}	b*	b^{*++}	b^0	b^{0++}	Seq.	y	y^{++}	y*	y^{*++}	y^0	y^{0++}	#
1	102.0550	51.5311			84.0444	42.5258	T							13
2	**215.1390**	108.0731			197.1285	99.0679	I	1488.8090	744.9082	1471.7825	736.3949	1470.7985	735.9029	12
3	**344.1816**	172.5944			326.1710	163.5892	E	***1375.7250***	688.3661	1358.6984	679.8529	1357.7144	679.3608	11
4	**473.2242**	237.1157			455.2136	228.1105	E	***1246.6824***	623.8448	1229.6558	615.3316	1228.6718	614.8395	10
5	586.3083	293.6578			568.2977	284.6525	L	***1117.6398***	559.3235	1100.6132	550.8103	1099.6292	550.3183	9
6	714.3668	357.6871	697.3403	349.1738	696.3563	348.6818	Q	***1004.5557***	502.7815	987.5292	494.2682	986.5452	493.7762	8
7	842.4254	421.7164	825.3989	413.2031	824.4149	412.7111	Q	***876.4972***	438.7522	859.4706	430.2389	858.4866	429.7469	7
8	970.5204	485.7638	953.4938	477.2506	952.5098	476.7585	K	***748.4386***	374.7229	731.4120	366.2096	730.4280	365.7176	6
9	**1083.6045**	542.3059	1066.5779	533.7926	1065.5939	533.3006	I	***620.3436***	310.6754	603.3171	302.1622	602.3330	301.6702	5
10	1196.6885	598.8479	1179.6620	590.3346	1178.6780	589.8426	L	***507.2595***	254.1334	490.2330	245.6201	489.2490	245.1281	4
11	1356.7192	678.8632	1339.6926	670.3499	1338.7086	669.8579	C	***394.1755***	197.5914	377.1489	189.0781	376.1649	188.5861	3
12	1443.7512	722.3792	1426.7246	713.8660	1425.7406	713.3740	S	234.1448	117.5761	217.1183	109.0628	216.1343	108.5708	2
13							K	147.1128	74.0600	130.0863	65.5468			1

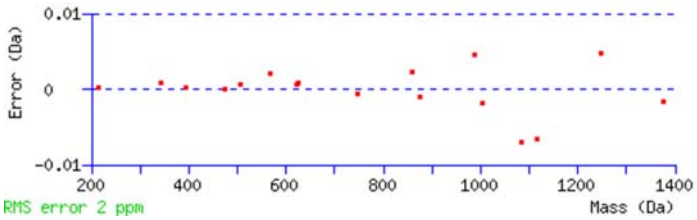

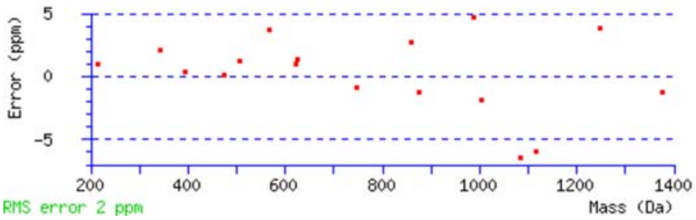

NCBI **BLAST** search of TIEELQQKILCSK
(Parameters: blastp, nr protein database, expect=20000, no filter, PAM30)
Other BLAST web gateways

All matches to this query

Score	Mr(calc)	Delta	Sequence
69.8	1588.8494	-0.0021	TIEELQQKILCSK

Mascot: http://www.matrixscience.com/

Mascot Search Results

Peptide View

MS/MS Fragmentation of **TIEELQQKILCSK**
Found in **ch17u_O76014|KRT37_HUMAN** in **uni_human**, Keratin, type I cuticular Ha7 OS=Homo sapiens GN=KRT37 PE=3 SV=3

Match to Query 20249: 1588.848972 from(530.623600,3+) intensity(2640728.7500) rtinseconds(1992) scans(10839) index(23789)
Title: 160219_Sunil_SDSI_A_Spectrum077094_scans__10839_RTINSECONDS=1992
Data file L:\\QE_2016\\160219_Sunil_KAP_LKC\\TMgf\\T\\T160219_Sunil_SDSI_A.mgf

Click mouse within plot area to zoom in by factor of two about that point
Or, Plot from 100 to 1600 Da Full range
Label all possible matches ○ Label matches used for scoring ◉
Show Y-axis ☐

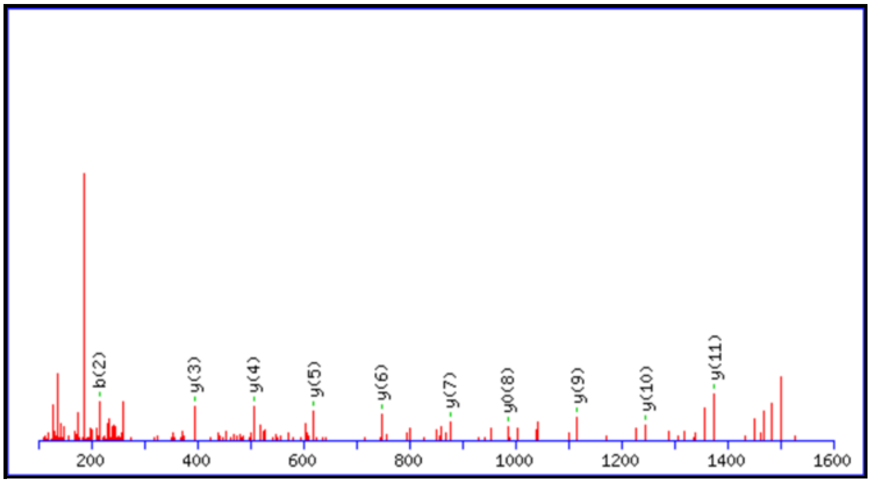

Monoisotopic mass of neutral peptide Mr(calc): 1588.8494
Fixed modifications: Carbamidomethyl (C) (apply to specified residues or termini only)
Ions Score: 57 **Expect:** 0.0003
Matches : 10/132 fragment ions using 15 most intense peaks (help)

#	b	b^{++}	b*	b^{*++}	b^{0}	b^{0++}	Seq.	y	y^{++}	y*	y^{*++}	y^{0}	y^{0++}	#
1	102.0550	51.5311			84.0444	42.5258	T							13
2	**215.1390**	108.0731			197.1285	99.0679	I	1488.8090	744.9082	1471.7825	736.3949	1470.7985	735.9029	12
3	344.1816	172.5944			326.1710	163.5892	E	***1375.7250***	688.3661	1358.6984	679.8529	1357.7144	679.3608	11
4	473.2242	237.1157			455.2136	228.1105	E	***1246.6824***	623.8448	1229.6558	615.3316	1228.6718	614.8395	10
5	586.3083	293.6578			568.2977	284.6525	L	***1117.6398***	559.3235	1100.6132	550.8103	1099.6292	550.3183	9
6	714.3668	357.6871	697.3403	349.1738	696.3563	348.6818	Q	1004.5557	502.7815	987.5292	494.2682	986.5452	493.7762	8
7	842.4254	421.7164	825.3989	413.2031	824.4149	412.7111	Q	***876.4972***	438.7522	859.4706	430.2389	858.4866	429.7469	7
8	970.5204	485.7638	953.4938	477.2506	952.5098	476.7585	K	***748.4386***	374.7229	731.4120	366.2096	730.4280	365.7176	6
9	1083.6045	542.3059	1066.5779	533.7926	1065.5939	533.3006	I	***620.3436***	310.6754	603.3171	302.1622	602.3330	301.6702	5
10	1196.6885	598.8479	1179.6620	590.3346	1178.6780	589.8426	L	***507.2595***	254.1334	490.2330	245.6201	489.2490	245.1281	4
11	1356.7192	678.8632	1339.6926	670.3499	1338.7086	669.8579	C	***394.1755***	197.5914	377.1489	189.0781	376.1649	188.5861	3
12	1443.7512	722.3792	1426.7246	713.8660	1425.7406	713.3740	S	234.1448	117.5761	217.1183	109.0628	216.1343	108.5708	2
13							K	147.1128	74.0600	130.0863	65.5468			1

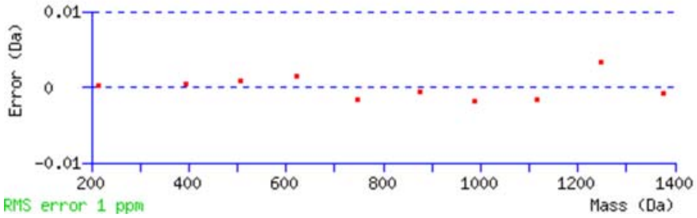

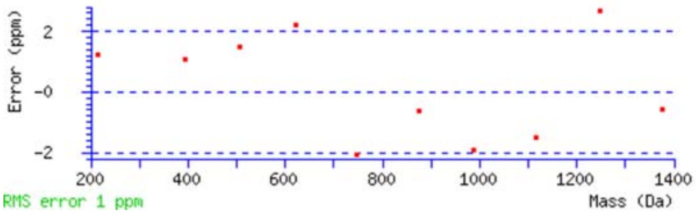

NCBI **BLAST** search of TIEELQQKILCSK
(Parameters: blastp, nr protein database, expect=20000, no filter, PAM30)
Other BLAST web gateways

All matches to this query

Score	Mr(calc)	Delta	Sequence
57.0	1588.8494	-0.0004	TIEELQQKILCSK

Mascot: http://www.matrixscience.com/

Mascot Search Results

Peptide View

MS/MS Fragmentation of **LAADDFR**
Found in **ch17u_O76014|KRT37_HUMAN** in **uni_human**, Keratin, type I cuticular Ha7 OS=Homo sapiens GN=KRT37 PE=3 SV=3

Match to Query 504: 806.391088 from(404.202820,2+) intensity(781061.6875) rtinseconds(1481) scans(7709) index(5647)
Title: 160219_Sunil_SDSI_A_Spectrum057755_scans__7709_RTINSECONDS=1481
Data file L:\\QE_2016\\160219_Sunil_KAP_LKC\\TMgf\\T\\T160219_Sunil_SDSI_A.mgf

Click mouse within plot area to zoom in by factor of two about that point
Or, Plot from 50 to 700 Da Full range
Label all possible matches ○ Label matches used for scoring ◉
Show Y-axis ☐

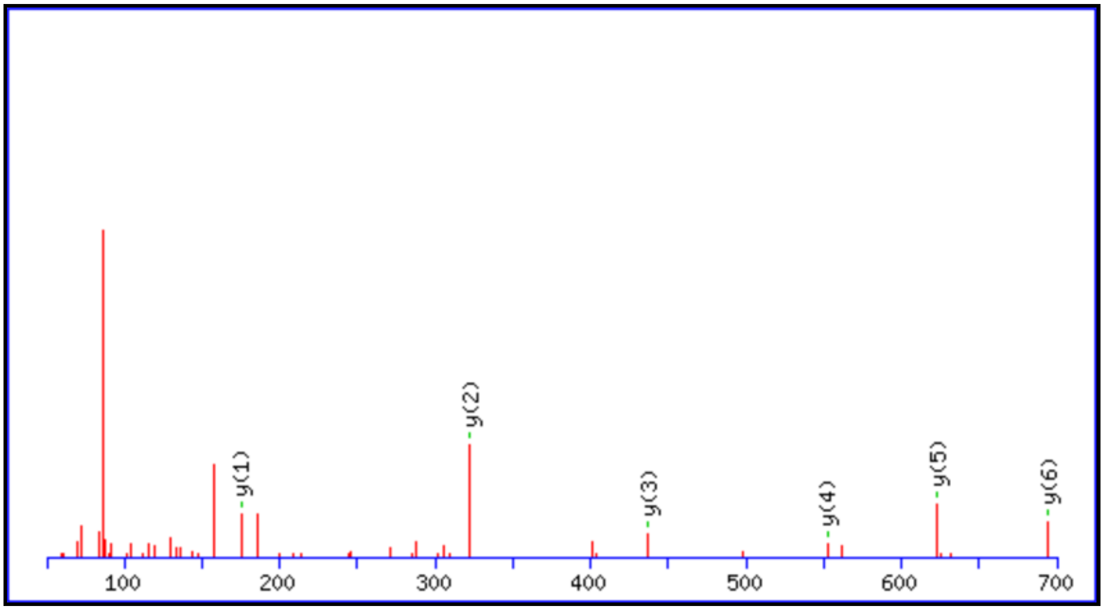

Monoisotopic mass of neutral peptide Mr(calc): 806.3923
Fixed modifications: Carbamidomethyl (C) (apply to specified residues or termini only)
Ions Score: 59 **Expect:** 0.00013
Matches : 6/50 fragment ions using 7 most intense peaks (help)

#	b	b^{++}	b^{0}	b^{0++}	Seq.	y	y^{++}	y^{*}	y^{*++}	y^{0}	y^{0++}	#
1	114.0913	57.5493			L							7
2	185.1285	93.0679			A	***694.3155***	347.6614	677.2889	339.1481	676.3049	338.6561	6
3	256.1656	128.5864			A	***623.2784***	312.1428	606.2518	303.6295	605.2678	303.1375	5
4	371.1925	186.0999	353.1819	177.0946	D	***552.2413***	276.6243	535.2147	268.1110	534.2307	267.6190	4
5	486.2195	243.6134	468.2089	234.6081	D	***437.2143***	219.1108	420.1878	210.5975	419.2037	210.1055	3
6	633.2879	317.1476	615.2773	308.1423	F	***322.1874***	161.5973	305.1608	153.0840			2

7					R	*175.1190*	88.0631	158.0924	79.5498			1

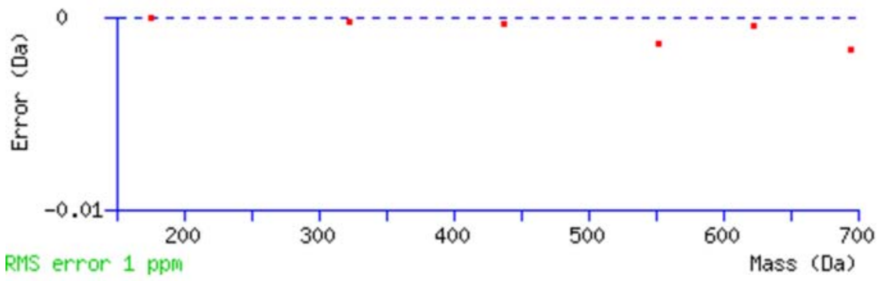

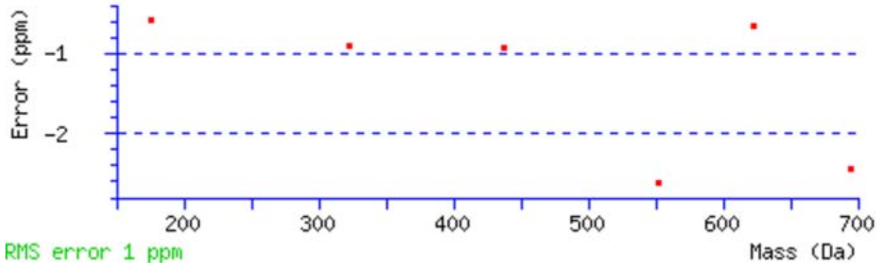

NCBI **BLAST** search of LAADDFR
(Parameters: blastp, nr protein database, expect=20000, no filter, PAM30)
Other BLAST web gateways

All matches to this query

Score	Mr(calc)	Delta	Sequence
59.5	806.3923	-0.0012	LAADDFR
24.3	804.3878	2.0033	RPDNFR
11.7	806.3923	-0.0012	LDQQFR
11.7	806.3923	-0.0012	LDQQFR
11.4	806.3922	-0.0012	GELANFR
11.4	804.3878	2.0033	NRPDFR
11.4	806.3923	-0.0012	QLEDFR
10.1	806.3923	-0.0012	IAGFDER
8.9	806.3844	0.0067	LAAQMQK
6.6	806.3956	-0.0045	MGNSLLR

Mascot: http://www.matrixscience.com/

Mascot Search Results

Peptide View

MS/MS Fragmentation of **QNQEYQVLLDVK**
Found in **ch17u_O76014|KRT37_HUMAN** in **uni_human**, Keratin, type I cuticular Ha7 OS=Homo sapiens GN=KRT37 PE=3 SV=3

Match to Query 16888: 1475.760968 from(738.887760,2+) intensity(1328210.1250) rtinseconds(2460) scans(13418) index(10524)
Title: 160219_Sunil_SDSI_A_Spectrum062647_scans__13418_RTINSECONDS=2460
Data file L:\\QE_2016\\160219_Sunil_KAP_LKC\\TMgf\\T\\T160219_Sunil_SDSI_A.mgf

Click mouse within plot area to zoom in by factor of two about that point
Or, Plot from 100 to 1500 Da Full range
Label all possible matches ○ Label matches used for scoring ◉
Show Y-axis ☐

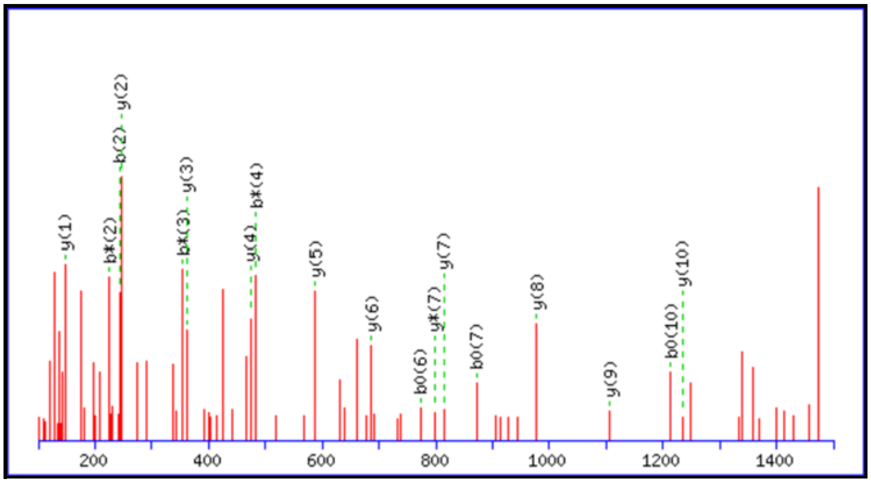

Monoisotopic mass of neutral peptide Mr(calc): 1475.7620
Fixed modifications: Carbamidomethyl (C) (apply to specified residues or termini only)
Ions Score: 63 **Expect:** 8.4e-005
Matches : 18/122 fragment ions using 36 most intense peaks (help)

#	b	b^{++}	b^{*}	b^{*++}	b^{0}	b^{0++}	Seq.	y	y^{++}	y^{*}	y^{*++}	y^{0}	y^{0++}	#
1	129.0659	65.0366	112.0393	56.5233			Q							12
2	243.1088	122.0580	226.0822	113.5448			N	1348.7107	674.8590	1331.6842	666.3457	1330.7001	665.8537	11
3	371.1674	186.0873	354.1408	177.5740			Q	1234.6678	617.8375	1217.6412	609.3243	1216.6572	608.8322	10
4	500.2100	250.6086	483.1834	242.0953	482.1994	241.6033	E	1106.6092	553.8082	1089.5827	545.2950	1088.5986	544.8030	9
5	663.2733	332.1403	646.2467	323.6270	645.2627	323.1350	Y	977.5666	489.2869	960.5401	480.7737	959.5560	480.2817	8
6	791.3319	396.1696	774.3053	387.6563	773.3213	387.1643	Q	814.5033	407.7553	797.4767	399.2420	796.4927	398.7500	7
7	890.4003	445.7038	873.3737	437.1905	872.3897	436.6985	V	686.4447	343.7260	669.4182	335.2127	668.4341	334.7207	6
8	1003.4843	502.2458	986.4578	493.7325	985.4738	493.2405	L	587.3763	294.1918	570.3497	285.6785	569.3657	285.1865	5
9	1116.5684	558.7878	1099.5419	550.2746	1098.5578	549.7826	L	474.2922	237.6498	457.2657	229.1365	456.2817	228.6445	4
10	1231.5953	616.3013	1214.5688	607.7880	1213.5848	607.2960	D	361.2082	181.1077	344.1816	172.5944	343.1976	172.1024	3
11	1330.6638	665.8355	1313.6372	657.3222	1312.6532	656.8302	V	246.1812	123.5942	229.1547	115.0810			2
12							K	147.1128	74.0600	130.0863	65.5468			1

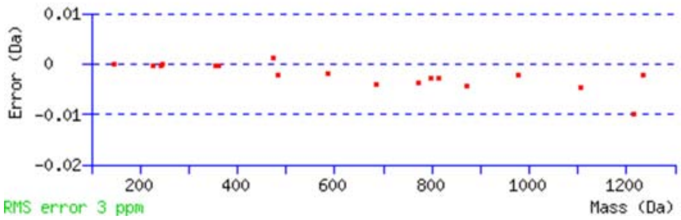

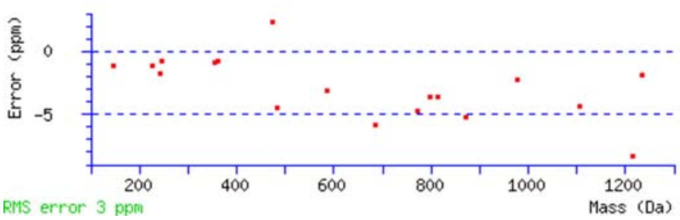

NCBI **BLAST** search of QNQEYQVLLDVK
(Parameters: blastp, nr protein database, expect=20000, no filter, PAM30)
Other BLAST web gateways

All matches to this query

Score	Mr(calc)	Delta	Sequence
63.4	1475.7620	-0.0010	QNQEYQVLLDVK

Mascot: http://www.matrixscience.com/

Mascot Search Results

Peptide View

MS/MS Fragmentation of **ADLEAQQESLKEEQLSLK**
Found in **ch17u_O76014|KRT37_HUMAN** in **uni_human**, Keratin, type I cuticular Ha7 OS=Homo sapiens GN=KRT37 PE=3 SV=3

Match to Query 29986: 2058.046662 from(687.022830,3+) intensity(1533306.0000) rtinseconds(2001) scans(10746) index(8305)
Title: 160219_Sunil_SDSI_A_Spectrum060416_scans__10746_RTINSECONDS=2001
Data file L:\\QE_2016\\160219_Sunil_KAP_LKC\\TMgf\\T\\T160219_Sunil_SDSI_A.mgf

Click mouse within plot area to zoom in by factor of two about that point
Or, Plot from 0 to 2200 Da Full range
Label all possible matches ○ Label matches used for scoring ◉
Show Y-axis □

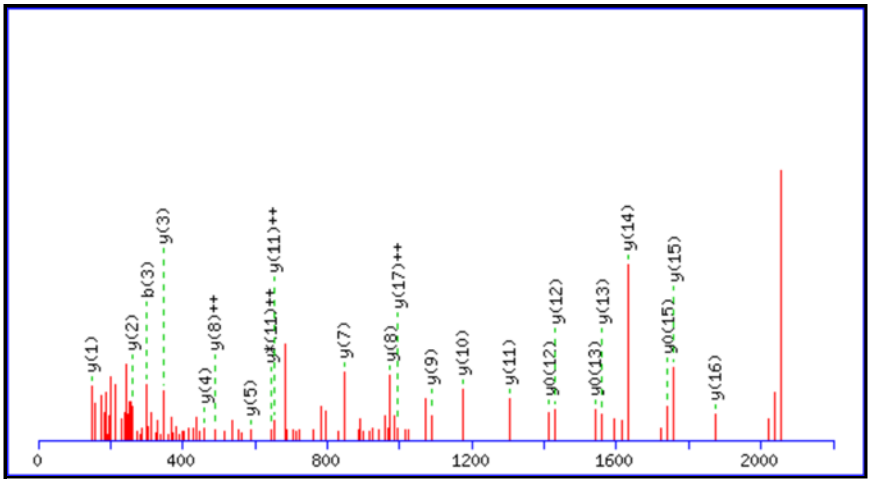

Monoisotopic mass of neutral peptide Mr(calc): 2058.0480
Fixed modifications: Carbamidomethyl (C) (apply to specified residues or termini only)
Ions Score: 91 **Expect:** 1.4e-007
Matches : 23/188 fragment ions using 56 most intense peaks (help)

#	b	b^{++}	b*	b^{*++}	b^0	b^{0++}	Seq.	y	y^{++}	y*	y^{*++}	y^0	y^{0++}	#
1	72.0444	36.5258					A							18
2	187.0713	94.0393			169.0608	85.0340	D	1988.0182	994.5128	1970.9917	985.9995	1970.0077	985.5075	17
3	**300.1554**	150.5813			282.1448	141.5761	L	***1872.9913***	936.9993	1855.9648	928.4860	1854.9807	927.9940	16
4	429.1980	215.1026			411.1874	206.0974	E	***1759.9072***	880.4573	1742.8807	871.9440	1741.8967	871.4520	15
5	500.2351	250.6212			482.2245	241.6159	A	***1630.8646***	815.9360	1613.8381	807.4227	1612.8541	806.9307	14
6	628.2937	314.6505	611.2671	306.1372	610.2831	305.6452	Q	***1559.8275***	780.4174	1542.8010	771.9041	1541.8170	771.4121	13
7	756.3523	378.6798	739.3257	370.1665	738.3417	369.6745	Q	***1431.7689***	716.3881	1414.7424	707.8748	1413.7584	707.3828	12
8	885.3949	443.2011	868.3683	434.6878	867.3843	434.1958	E	***1303.7104***	652.3588	1286.6838	643.8455	1285.6998	643.3535	11
9	972.4269	486.7171	955.4003	478.2038	954.4163	477.7118	S	***1174.6678***	587.8375	1157.6412	579.3243	1156.6572	578.8322	10
10	1085.5109	543.2591	1068.4844	534.7458	1067.5004	534.2538	L	***1087.6358***	544.3215	1070.6092	535.8082	1069.6252	535.3162	9
11	1213.6059	607.3066	1196.5794	598.7933	1195.5953	598.3013	K	***974.5517***	487.7795	957.5251	479.2662	956.5411	478.7742	8
12	1342.6485	671.8279	1325.6220	663.3146	1324.6379	662.8226	E	***846.4567***	423.7320	829.4302	415.2187	828.4462	414.7267	7
13	1471.6911	736.3492	1454.6645	727.8359	1453.6805	727.3439	E	717.4141	359.2107	700.3876	350.6974	699.4036	350.2054	6
14	1599.7497	800.3785	1582.7231	791.8652	1581.7391	791.3732	Q	***588.3715***	294.6894	571.3450	286.1761	570.3610	285.6841	5
15	1712.8337	856.9205	1695.8072	848.4072	1694.8232	847.9152	L	***460.3130***	230.6601	443.2864	222.1468	442.3024	221.6548	4
16	1799.8658	900.4365	1782.8392	891.9232	1781.8552	891.4312	S	***347.2289***	174.1181	330.2023	165.6048	329.2183	165.1128	3
17	1912.9498	956.9786	1895.9233	948.4653	1894.9393	947.9733	L	***260.1969***	130.6021	243.1703	122.0888			2
18							K	***147.1128***	74.0600	130.0863	65.5468			1

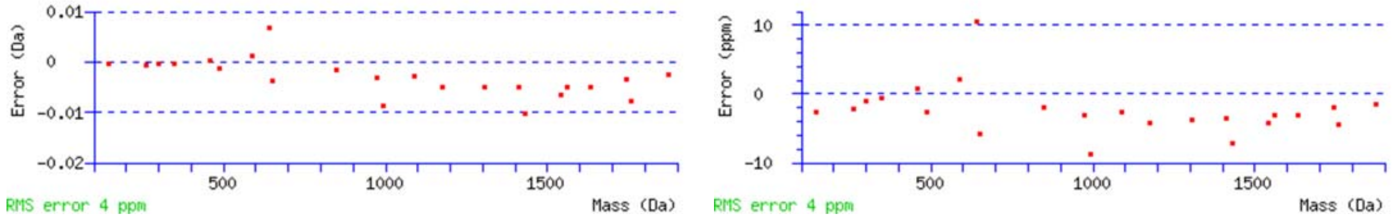

NCBI **BLAST** search of ADLEAQQESLKEEQLSLK
(Parameters: blastp, nr protein database, expect=20000, no filter, PAM30)
Other BLAST web gateways

All matches to this query

Score	Mr(calc)	Delta	Sequence
90.7	2058.0480	-0.0014	ADLEAQQESLKEEQLSLK

Mascot: http://www.matrixscience.com/

Mascot Search Results

Peptide View

MS/MS Fragmentation of **LLDDATLAKADLEAQQESLKEEQLSLK**
Found in **ch17u_O76014|KRT37_HUMAN** in **uni_human**, Keratin, type I cuticular Ha7 OS=Homo sapiens GN=KRT37 PE=3 SV=3

Match to Query 39984: 2998.567576 from(750.649170,4+) intensity(7421416.0000) rtinseconds(2689) scans(14757) index(11622)
Title: 160219_Sunil_SDSI_A_Spectrum063750_scans__14757_RTINSECONDS=2689
Data file L:\\QE_2016\\160219_Sunil_KAP_LKC\\TMgf\\T\\T160219_Sunil_SDSI_A.mgf

Click mouse within plot area to zoom in by factor of two about that point
Or, Plot from 200 to 3000 Da Full range
Label all possible matches ○ Label matches used for scoring ◉
Show Y-axis ☐

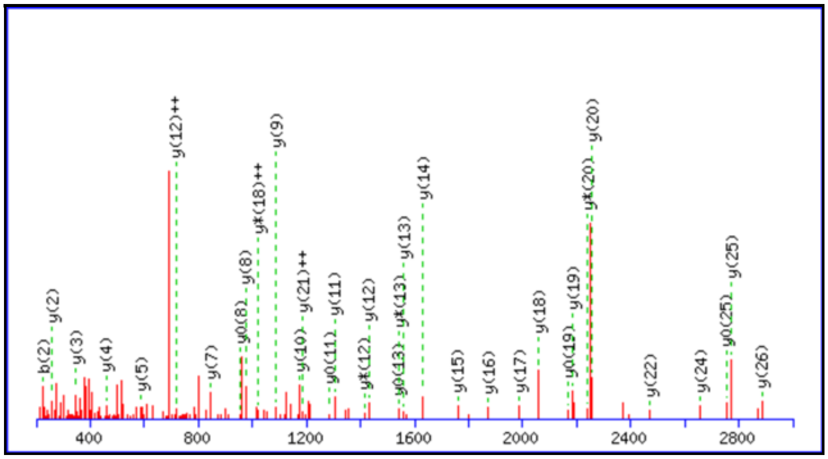

Monoisotopic mass of neutral peptide Mr(calc): 2998.5710
Fixed modifications: Carbamidomethyl (C) (apply to specified residues or termini only)
Ions Score: 131 **Expect:** 8.5e-012
Matches : 34/288 fragment ions using 88 most intense peaks (help)

#	b	b^{++}	b*	b^{*++}	b^0	b^{0++}	Seq.	y	y^{++}	y*	y^{*++}	y^0	y^{0++}	#
1	114.0913	57.5493					L							27
2	227.1754	114.0913					L	2886.4942	1443.7508	2869.4677	1435.2375	2868.4837	1434.7455	26
3	342.2023	171.6048			324.1918	162.5995	D	2773.4102	1387.2087	2756.3836	1378.6955	2755.3996	1378.2034	25
4	457.2293	229.1183			439.2187	220.1130	D	2658.3832	1329.6953	2641.3567	1321.1820	2640.3727	1320.6900	24
5	528.2664	264.6368			510.2558	255.6316	A	2543.3563	1272.1818	2526.3297	1263.6685	2525.3457	1263.1765	23
6	629.3141	315.1607			611.3035	306.1554	T	2472.3192	1236.6632	2455.2926	1228.1500	2454.3086	1227.6579	22
7	742.3981	371.7027			724.3876	362.6974	L	2371.2715	1186.1394	2354.2449	1177.6261	2353.2609	1177.1341	21
8	813.4353	407.2213			795.4247	398.2160	A	2258.1874	1129.5974	2241.1609	1121.0841	2240.1769	1120.5921	20
9	941.5302	471.2688	924.5037	462.7555	923.5197	462.2635	K	2187.1503	1094.0788	2170.1238	1085.5655	2169.1398	1085.0735	19
10	1012.5673	506.7873	995.5408	498.2740	994.5568	497.7820	A	2059.0554	1030.0313	2042.0288	1021.5180	2041.0448	1021.0260	18
11	1127.5943	564.3008	1110.5677	555.7875	1109.5837	555.2955	D	1988.0182	994.5128	1970.9917	985.9995	1970.0077	985.5075	17
12	1240.6783	620.8428	1223.6518	612.3295	1222.6678	611.8375	L	1872.9913	936.9993	1855.9648	928.4860	1854.9807	927.9940	16
13	1369.7209	685.3641	1352.6944	676.8508	1351.7104	676.3588	E	1759.9072	880.4573	1742.8807	871.9440	1741.8967	871.4520	15
14	1440.7581	720.8827	1423.7315	712.3694	1422.7475	711.8774	A	1630.8646	815.9360	1613.8381	807.4227	1612.8541	806.9307	14
15	1568.8166	784.9120	1551.7901	776.3987	1550.8061	775.9067	Q	1559.8275	780.4174	1542.8010	771.9041	1541.8170	771.4121	13
16	1696.8752	848.9412	1679.8487	840.4280	1678.8646	839.9360	Q	1431.7690	716.3881	1414.7424	707.8748	1413.7584	707.3828	12
17	1825.9178	913.4625	1808.8913	904.9493	1807.9072	904.4573	E	1303.7104	652.3588	1286.6838	643.8455	1285.6998	643.3535	11
18	1912.9498	956.9786	1895.9233	948.4653	1894.9393	947.9733	S	1174.6678	587.8375	1157.6412	579.3243	1156.6572	578.8322	10
19	2026.0339	1013.5206	2009.0073	1005.0073	2008.0233	1004.5153	L	1087.6358	544.3215	1070.6092	535.8082	1069.6252	535.3162	9
20	2154.1289	1077.5681	2137.1023	1069.0548	2136.1183	1068.5628	K	974.5517	487.7795	957.5251	479.2662	956.5411	478.7742	8
21	2283.1714	1142.0894	2266.1449	1133.5761	2265.1609	1133.0841	E	846.4567	423.7320	829.4302	415.2187	828.4462	414.7267	7

**22**	2412.2140	1206.6107	2395.1875	1198.0974	2394.2035	1197.6054	**E**	717.4141	359.2107	700.3876	350.6974	699.4036	350.2054	**6**
**23**	2540.2726	1270.6399	2523.2461	1262.1267	2522.2621	1261.6347	**Q**	***588.3715***	294.6894	571.3450	286.1761	570.3610	285.6841	**5**
**24**	2653.3567	1327.1820	2636.3301	1318.6687	2635.3461	1318.1767	**L**	***460.3130***	230.6601	443.2864	222.1468	442.3024	221.6548	**4**
**25**	2740.3887	1370.6980	2723.3622	1362.1847	2722.3781	1361.6927	**S**	***347.2289***	174.1181	330.2023	165.6048	329.2183	165.1128	**3**
**26**	2853.4728	1427.2400	2836.4462	1418.7268	2835.4622	1418.2347	**L**	***260.1969***	130.6021	243.1703	122.0888			**2**
**27**							**K**	147.1128	74.0600	130.0863	65.5468			**1**

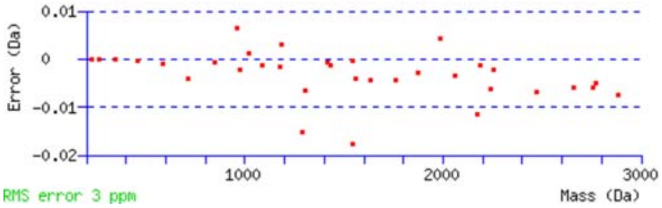

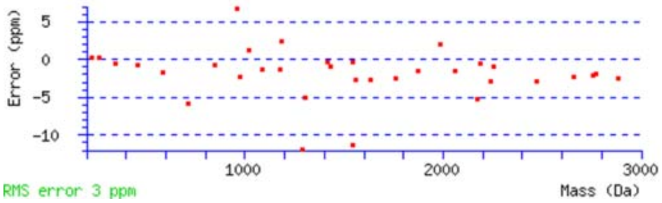

NCBI **BLAST** search of LLDDATLAKADLEAQQESLKEEQLSLK
(Parameters: blastp, nr protein database, expect=20000, no filter, PAM30)
Other BLAST web gateways

All matches to this query

Score	Mr(calc)	Delta	Sequence
131.0	2998.5710	-0.0034	LLDDATLAKADLEAQQESLKEEQLSLK

Mascot: http://www.matrixscience.com/

Mascot Search Results

Peptide View

MS/MS Fragmentation of **ADLEAQQESLKEEQLSLKSNHEQEVK**
Found in **ch17u_O76014|KRT37_HUMAN** in **uni_human**, Keratin, type I cuticular Ha7 OS=Homo sapiens GN=KRT37 PE=3 SV=3

Match to Query 40023: 3009.493856 from(753.380740,4+) intensity(565858.8750) rtinseconds(1571) scans(8239) index(6127)
Title: 160219_Sunil_SDSI_A_Spectrum058235_scans__8239_RTINSECONDS=1571
Data file L:\\QE_2016\\160219_Sunil_KAP_LKC\\TMgf\\T\\T160219_Sunil_SDSI_A.mgf

Click mouse within plot area to zoom in by factor of two about that point
Or, Plot from 200 to 3000 Da Full range
Label all possible matches ○ Label matches used for scoring ◉
Show Y-axis ☐

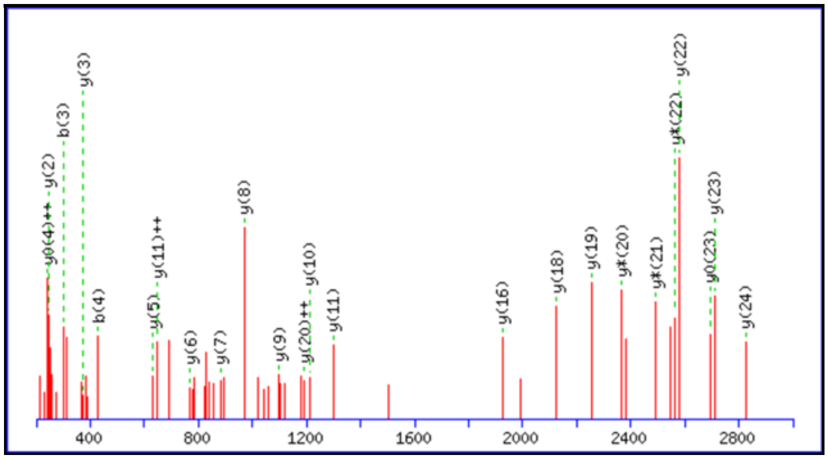

Monoisotopic mass of neutral peptide Mr(calc): 3009.4890
Fixed modifications: Carbamidomethyl (C) (apply to specified residues or termini only)
Ions Score: 97 **Expect:** 4.2e-008
Matches : 24/284 fragment ions using 43 most intense peaks (help)

#	b	b^{++}	b^{*}	b^{*++}	b^{0}	b^{0++}	Seq.	y	y^{++}	y^{*}	y^{*++}	y^{0}	y^{0++}	#
1	72.0444	36.5258					A							26
2	187.0713	94.0393			169.0608	85.0340	D	2939.4592	1470.2333	2922.4327	1461.7200	2921.4487	1461.2280	25
3	**300.1554**	150.5813			282.1448	141.5761	L	***2824.4323***	1412.7198	2807.4058	1404.2065	2806.4217	1403.7145	24
4	**429.1980**	215.1026			411.1874	206.0974	E	***2711.3482***	1356.1778	2694.3217	1347.6645	2693.3377	1347.1725	23
5	500.2351	250.6212			482.2245	241.6159	A	***2582.3056***	1291.6565	2565.2791	1283.1432	2564.2951	1282.6512	22
6	628.2937	314.6505	611.2671	306.1372	610.2831	305.6452	Q	2511.2685	1256.1379	2494.2420	1247.6246	2493.2580	1247.1326	21
7	756.3523	378.6798	739.3257	370.1665	738.3417	369.6745	Q	2383.2100	1192.1086	2366.1834	1183.5953	2365.1994	1183.1033	20
8	885.3949	443.2011	868.3683	434.6878	867.3843	434.1958	E	***2255.1514***	1128.0793	2238.1248	1119.5661	2237.1408	1119.0740	19
9	972.4269	486.7171	955.4003	478.2038	954.4163	477.7118	S	***2126.1088***	1063.5580	2109.0822	1055.0448	2108.0982	1054.5527	18
10	1085.5109	543.2591	1068.4844	534.7458	1067.5004	534.2538	L	2039.0768	1020.0420	2022.0502	1011.5287	2021.0662	1011.0367	17
11	1213.6059	607.3066	1196.5794	598.7933	1195.5953	598.3013	K	***1925.9927***	963.5000	1908.9661	954.9867	1907.9821	954.4947	16
12	1342.6485	671.8279	1325.6220	663.3146	1324.6379	662.8226	E	1797.8977	899.4525	1780.8712	890.9392	1779.8872	890.4472	15
13	1471.6911	736.3492	1454.6645	727.8359	1453.6805	727.3439	E	1668.8551	834.9312	1651.8286	826.4179	1650.8446	825.9259	14
14	1599.7497	800.3785	1582.7231	791.8652	1581.7391	791.3732	Q	1539.8125	770.4099	1522.7860	761.8966	1521.8020	761.4046	13
15	1712.8337	856.9205	1695.8072	848.4072	1694.8232	847.9152	L	1411.7540	706.3806	1394.7274	697.8673	1393.7434	697.3753	12
16	1799.8658	900.4365	1782.8392	891.9232	1781.8552	891.4312	S	***1298.6699***	649.8386	1281.6434	641.3253	1280.6593	640.8333	11
17	1912.9498	956.9786	1895.9233	948.4653	1894.9393	947.9733	L	***1211.6379***	606.3226	1194.6113	597.8093	1193.6273	597.3173	10
18	2041.0448	1021.0260	2024.0182	1012.5128	2023.0342	1012.0208	K	***1098.5538***	549.7805	1081.5273	541.2673	1080.5432	540.7753	9
19	2128.0768	1064.5420	2111.0503	1056.0288	2110.0663	1055.5368	S	***970.4588***	485.7331	953.4323	477.2198	952.4483	476.7278	8
20	2242.1197	1121.5635	2225.0932	1113.0502	2224.1092	1112.5582	N	***883.4268***	442.2170	866.4003	433.7038	865.4163	433.2118	7
21	2379.1787	1190.0930	2362.1521	1181.5797	2361.1681	1181.0877	H	***769.3839***	385.1956	752.3573	376.6823	751.3733	376.1903	6

22	2508.2213	1254.6143	2491.1947	1246.1010	2490.2107	1245.6090	E	632.3250	316.6661	615.2984	308.1529	614.3144	307.6608	5
23	2636.2798	1318.6436	2619.2533	1310.1303	2618.2693	1309.6383	Q	503.2824	252.1448	486.2558	243.6316	485.2718	243.1396	4
24	2765.3224	1383.1648	2748.2959	1374.6516	2747.3119	1374.1596	E	375.2238	188.1155	358.1973	179.6023	357.2132	179.1103	3
25	2864.3908	1432.6991	2847.3643	1424.1858	2846.3803	1423.6938	V	246.1812	123.5942	229.1547	115.0810			2
26							K	147.1128	74.0600	130.0863	65.5468			1

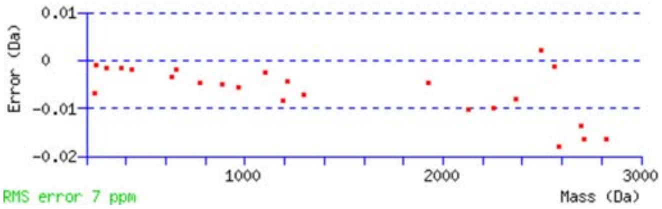

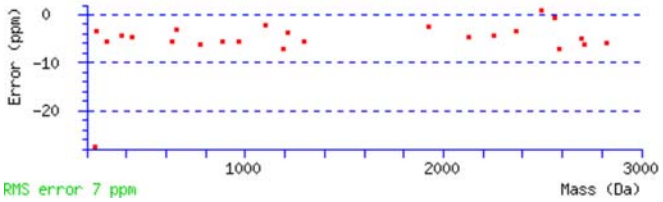

NCBI **BLAST** search of ADLEAQQESLKEEQLSLKSNHEQEVK
(Parameters: blastp, nr protein database, expect=20000, no filter, PAM30)
Other BLAST web gateways

All matches to this query

Score	Mr(calc)	Delta	Sequence
96.6	3009.4890	0.0048	ADLEAQQESLKEEQLSLKSNHEQEVK
3.5	3009.5229	-0.0291	ELRVEQESLLTAFRCGLQFLGNIASR

Mascot: http://www.matrixscience.com/

Mascot Search Results

Peptide View

MS/MS Fragmentation of **ADLERQNQEYQVLLDVK**
Found in **ch17u_O76014|KRT37_HUMAN** in **uni_human**, Keratin, type I cuticular Ha7 OS=Homo sapiens GN=KRT37 PE=3 SV=3

Match to Query 30004: 2060.051832 from(687.691220,3+) intensity(1042116.9375) rtinseconds(2424) scans(13207) index(10353)
Title: 160219_Sunil_SDSI_A_Spectrum062474_scans__13207_RTINSECONDS=2424
Data file L:\\QE_2016\\160219_Sunil_KAP_LKC\\TMgf\\T\\T160219_Sunil_SDSI_A.mgf

Click mouse within plot area to zoom in by factor of two about that point
Or, Plot from 100 to 1600 Da Full range
Label all possible matches ○ Label matches used for scoring ◉
Show Y-axis ☐

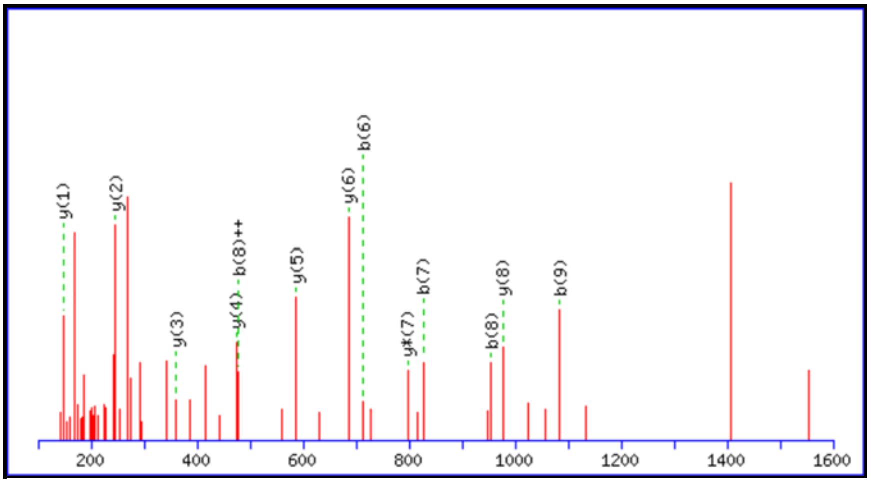

Monoisotopic mass of neutral peptide Mr(calc): 2060.0538
Fixed modifications: Carbamidomethyl (C) (apply to specified residues or termini only)
Ions Score: 44 **Expect:** 0.0065
Matches : 13/178 fragment ions using 21 most intense peaks (help)

#	b	b++	b*	b*++	b0	b0++	Seq.	y	y++	y*	y*++	y0	y0++	#
1	72.0444	36.5258					A							17
2	187.0713	94.0393			169.0608	85.0340	D	1990.0240	995.5156	1972.9974	987.0024	1972.0134	986.5104	16
3	300.1554	150.5813			282.1448	141.5761	L	1874.9971	938.0022	1857.9705	929.4889	1856.9865	928.9969	15
4	429.1980	215.1026			411.1874	206.0974	E	1761.9130	881.4601	1744.8864	872.9469	1743.9024	872.4549	14
5	585.2991	293.1532	568.2726	284.6399	567.2885	284.1479	R	1632.8704	816.9388	1615.8438	808.4256	1614.8598	807.9336	13
6	***713.3577***	357.1825	696.3311	348.6692	695.3471	348.1772	Q	1476.7693	738.8883	1459.7427	730.3750	1458.7587	729.8830	12
7	***827.4006***	414.2039	810.3741	405.6907	809.3900	405.1987	N	1348.7107	674.8590	1331.6842	666.3457	1330.7001	665.8537	11
8	***955.4592***	**478.2332**	938.4326	469.7200	937.4486	469.2279	Q	1234.6678	617.8375	1217.6412	609.3243	1216.6572	608.8322	10
9	***1084.5018***	542.7545	1067.4752	534.2413	1066.4912	533.7492	E	1106.6092	553.8082	1089.5827	545.2950	1088.5986	544.8030	9
10	1247.5651	624.2862	1230.5386	615.7729	1229.5545	615.2809	Y	***977.5666***	489.2869	960.5401	480.7737	959.5560	480.2817	8
11	1375.6237	688.3155	1358.5971	679.8022	1357.6131	679.3102	Q	814.5033	407.7553	797.4767	399.2420	796.4927	398.7500	7
12	1474.6921	737.8497	1457.6655	729.3364	1456.6815	728.8444	V	***686.4447***	343.7260	669.4182	335.2127	668.4341	334.7207	6
13	1587.7762	794.3917	1570.7496	785.8784	1569.7656	785.3864	L	***587.3763***	294.1918	570.3497	285.6785	569.3657	285.1865	5
14	1700.8602	850.9338	1683.8337	842.4205	1682.8497	841.9285	L	***474.2922***	237.6498	457.2657	229.1365	456.2817	228.6445	4
15	1815.8872	908.4472	1798.8606	899.9339	1797.8766	899.4419	D	***361.2082***	181.1077	344.1816	172.5944	343.1976	172.1024	3
16	1914.9556	957.9814	1897.9290	949.4682	1896.9450	948.9761	V	***246.1812***	123.5942	229.1547	115.0810			2
17							K	***147.1128***	74.0600	130.0863	65.5468			1

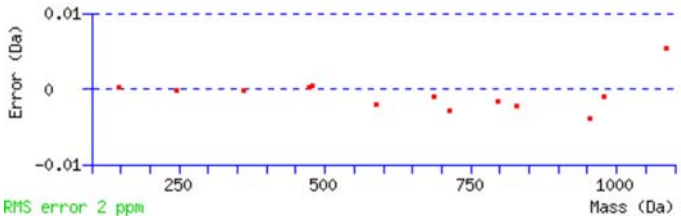

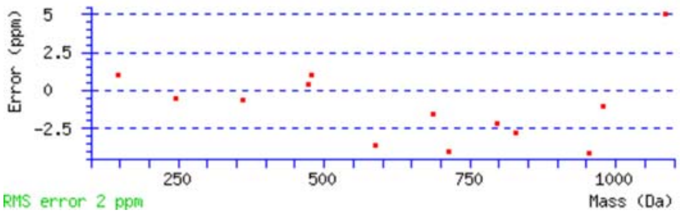

NCBI **BLAST** search of ADLERQNQEYQVLLDVK
(Parameters: blastp, nr protein database, expect=20000, no filter, PAM30)
Other BLAST web gateways

All matches to this query

Score	Mr(calc)	Delta	Sequence
44.3	2060.0538	-0.0020	ADLERQNQEYQVLLDVK
0.7	2059.0619	0.9899	EEVTPEQLKLMRTQDVK
0.3	2060.0441	0.0077	AFQPFFVELTMPYSVIR

Mascot: http://www.matrixscience.com/

Mascot Search Results

Peptide View

MS/MS Fragmentation of **VRQLEQENAELETTLLER**
Found in **ch17u_O76014|KRT37_HUMAN** in **uni_human**, Keratin, type I cuticular Ha7 OS=Homo sapiens GN=KRT37 PE=3 SV=3

Match to Query 31993: 2170.120752 from(724.380860,3+) intensity(307696.0000) rtinseconds(2382) scans(12966) index(10158)
Title: 160219_Sunil_SDSI_A_Spectrum062278_scans__12966_RTINSECONDS=2382
Data file L:\\QE_2016\\160219_Sunil_KAP_LKC\\TMgf\\T\\T160219_Sunil_SDSI_A.mgf

Click mouse within plot area to zoom in by factor of two about that point
Or, Plot from 100 to 1500 Da Full range
Label all possible matches ○ Label matches used for scoring ◉
Show Y-axis ☐

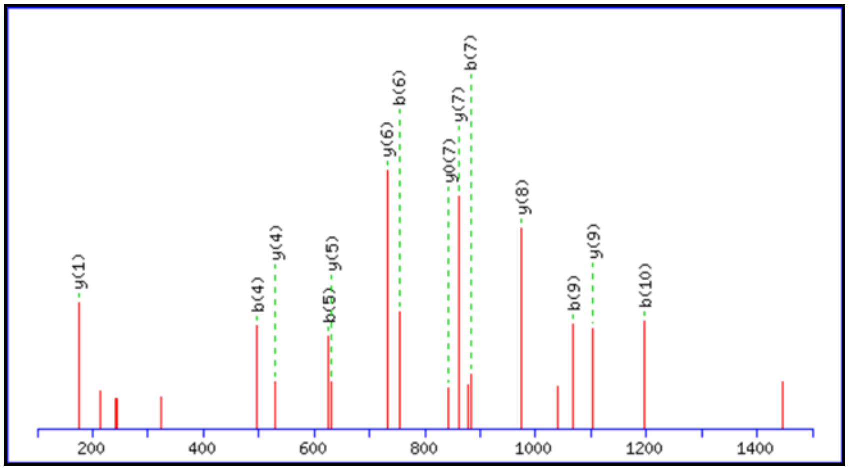

Monoisotopic mass of neutral peptide Mr(calc): 2170.1229
Fixed modifications: Carbamidomethyl (C) (apply to specified residues or termini only)
Ions Score: 67 **Expect:** 2.8e-005
Matches : 14/192 fragment ions using 18 most intense peaks (help)

#	b	b^{++}	b*	b^{*++}	b^0	b^{0++}	Seq.	y	y^{++}	y*	y^{*++}	y^0	y^{0++}	#
1	100.0757	50.5415					V							18
2	256.1768	128.5920	239.1503	120.0788			R	2072.0618	1036.5346	2055.0353	1028.0213	2054.0513	1027.5293	17
3	384.2354	192.6213	367.2088	184.1081			Q	1915.9607	958.4840	1898.9342	949.9707	1897.9502	949.4787	16
4	***497.3194***	249.1634	480.2929	240.6501			L	1787.9022	894.4547	1770.8756	885.9414	1769.8916	885.4494	15
5	***626.3620***	313.6847	609.3355	305.1714	608.3515	304.6794	E	1674.8181	837.9127	1657.7915	829.3994	1656.8075	828.9074	14
6	***754.4206***	377.7139	737.3941	369.2007	736.4100	368.7087	Q	1545.7755	773.3914	1528.7489	764.8781	1527.7649	764.3861	13
7	***883.4632***	442.2352	866.4367	433.7220	865.4526	433.2300	E	1417.7169	709.3621	1400.6904	700.8488	1399.7063	700.3568	12
8	997.5061	499.2567	980.4796	490.7434	979.4956	490.2514	N	1288.6743	644.8408	1271.6478	636.3275	1270.6638	635.8355	11
9	***1068.5432***	534.7753	1051.5167	526.2620	1050.5327	525.7700	A	1174.6314	587.8193	1157.6048	579.3061	1156.6208	578.8141	10
10	***1197.5858***	599.2966	1180.5593	590.7833	1179.5753	590.2913	E	***1103.5943***	552.3008	1086.5677	543.7875	1085.5837	543.2955	9
11	1310.6699	655.8386	1293.6434	647.3253	1292.6593	646.8333	L	***974.5517***	487.7795	957.5251	479.2662	956.5411	478.7742	8
12	1439.7125	720.3599	1422.6859	711.8466	1421.7019	711.3546	E	***861.4676***	431.2374	844.4411	422.7242	843.4571	422.2322	7
13	1540.7602	770.8837	1523.7336	762.3705	1522.7496	761.8784	T	***732.4250***	366.7162	715.3985	358.2029	714.4145	357.7109	6
14	1641.8079	821.4076	1624.7813	812.8943	1623.7973	812.4023	T	***631.3774***	316.1923	614.3508	307.6790	613.3668	307.1870	5
15	1754.8919	877.9496	1737.8654	869.4363	1736.8814	868.9443	L	***530.3297***	265.6685	513.3031	257.1552	512.3191	256.6632	4
16	1867.9760	934.4916	1850.9494	925.9784	1849.9654	925.4863	L	417.2456	209.1264	400.2191	200.6132	399.2350	200.1212	3
17	1997.0186	999.0129	1979.9920	990.4997	1979.0080	990.0076	E	304.1615	152.5844	287.1350	144.0711	286.1510	143.5791	2
18							R	***175.1190***	88.0631	158.0924	79.5498			1

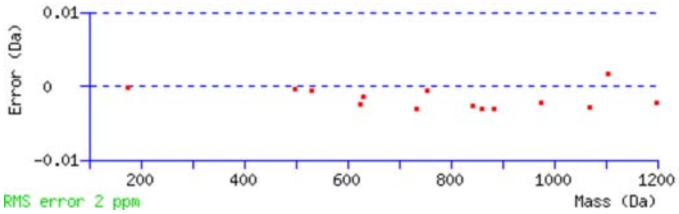

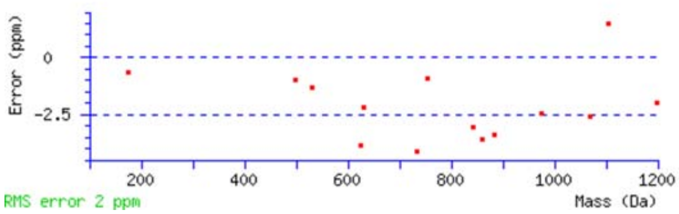

NCBI **BLAST** search of VRQLEQENAELETTLLER
(Parameters: blastp, nr protein database, expect=20000, no filter, PAM30)
Other BLAST web gateways

All matches to this query

Score	Mr(calc)	Delta	Sequence
67.3	2170.1229	-0.0022	VRQLEQENAELETTLLER
0.6	2170.1018	0.0189	EVGGGGVGGGLLENANPLIYQR
0.6	2170.1018	0.0189	QVGGGGVGGGLLENANPLIYQR
0.3	2168.1122	2.0085	QGLLFSTLLLAGLAQFCCR
0.3	2169.0962	1.0245	QGLLFSTLLLAGLAQFCCR

Mascot: http://www.matrixscience.com/

Mascot Search Results

Peptide View

MS/MS Fragmentation of **VRQLEQENAELETTLLER**
Found in **ch17u_O76014|KRT37_HUMAN** in **uni_human**, Keratin, type I cuticular Ha7 OS=Homo sapiens GN=KRT37 PE=3 SV=3

Match to Query 31993: 2170.120752 from(724.380860,3+) intensity(307696.0000) rtinseconds(2382) scans(12966) index(10158)
Title: 160219_Sunil_SDSI_A_Spectrum062278_scans__12966_RTINSECONDS=2382
Data file L:\\QE_2016\\160219_Sunil_KAP_LKC\\TMgf\\T\\T160219_Sunil_SDSI_A.mgf

Click mouse within plot area to zoom in by factor of two about that point
Or, Plot from 100 to 1500 Da Full range
Label all possible matches ○ Label matches used for scoring ◉
Show Y-axis ☐

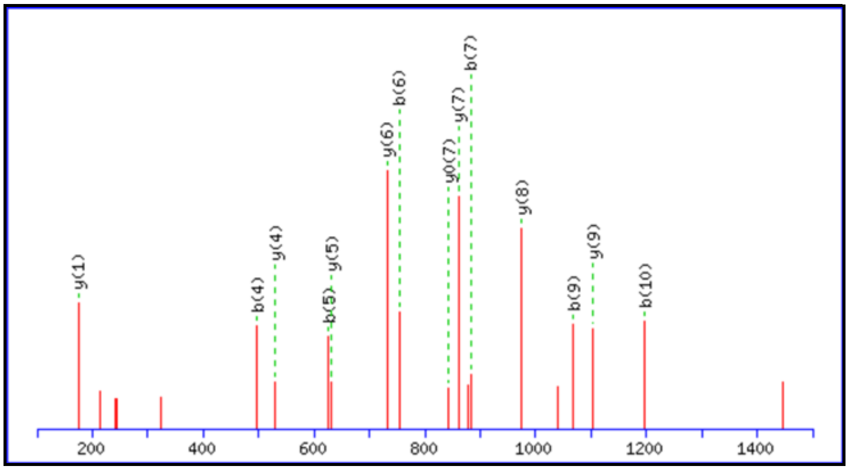

Monoisotopic mass of neutral peptide Mr(calc): 2170.1229
Fixed modifications: Carbamidomethyl (C) (apply to specified residues or termini only)
Ions Score: 67 **Expect:** 2.8e-005
Matches : 14/192 fragment ions using 18 most intense peaks (help)

#	b	b^{++}	b*	b^{*++}	b^0	b^{0++}	Seq.	y	y^{++}	y*	y^{*++}	y^0	y^{0++}	#
1	100.0757	50.5415					V							18
2	256.1768	128.5920	239.1503	120.0788			R	2072.0618	1036.5346	2055.0353	1028.0213	2054.0513	1027.5293	17
3	384.2354	192.6213	367.2088	184.1081			Q	1915.9607	958.4840	1898.9342	949.9707	1897.9502	949.4787	16
4	***497.3194***	249.1634	480.2929	240.6501			L	1787.9022	894.4547	1770.8756	885.9414	1769.8916	885.4494	15
5	***626.3620***	313.6847	609.3355	305.1714	608.3515	304.6794	E	1674.8181	837.9127	1657.7915	829.3994	1656.8075	828.9074	14
6	***754.4206***	377.7139	737.3941	369.2007	736.4100	368.7087	Q	1545.7755	773.3914	1528.7489	764.8781	1527.7649	764.3861	13
7	***883.4632***	442.2352	866.4367	433.7220	865.4526	433.2300	E	1417.7169	709.3621	1400.6904	700.8488	1399.7063	700.3568	12
8	997.5061	499.2567	980.4796	490.7434	979.4956	490.2514	N	1288.6743	644.8408	1271.6478	636.3275	1270.6638	635.8355	11
9	***1068.5432***	534.7753	1051.5167	526.2620	1050.5327	525.7700	A	1174.6314	587.8193	1157.6048	579.3061	1156.6208	578.8141	10
10	***1197.5858***	599.2966	1180.5593	590.7833	1179.5753	590.2913	E	***1103.5943***	552.3008	1086.5677	543.7875	1085.5837	543.2955	9
11	1310.6699	655.8386	1293.6434	647.3253	1292.6593	646.8333	L	***974.5517***	487.7795	957.5251	479.2662	956.5411	478.7742	8
12	1439.7125	720.3599	1422.6859	711.8466	1421.7019	711.3546	E	***861.4676***	431.2374	844.4411	422.7242	843.4571	422.2322	7
13	1540.7602	770.8837	1523.7336	762.3705	1522.7496	761.8784	T	***732.4250***	366.7162	715.3985	358.2029	714.4145	357.7109	6
14	1641.8079	821.4076	1624.7813	812.8943	1623.7973	812.4023	T	***631.3774***	316.1923	614.3508	307.6790	613.3668	307.1870	5
15	1754.8919	877.9496	1737.8654	869.4363	1736.8814	868.9443	L	***530.3297***	265.6685	513.3031	257.1552	512.3191	256.6632	4
16	1867.9760	934.4916	1850.9494	925.9784	1849.9654	925.4863	L	417.2456	209.1264	400.2191	200.6132	399.2350	200.1212	3
17	1997.0186	999.0129	1979.9920	990.4997	1979.0080	990.0076	E	304.1615	152.5844	287.1350	144.0711	286.1510	143.5791	2
18							R	***175.1190***	88.0631	158.0924	79.5498			1

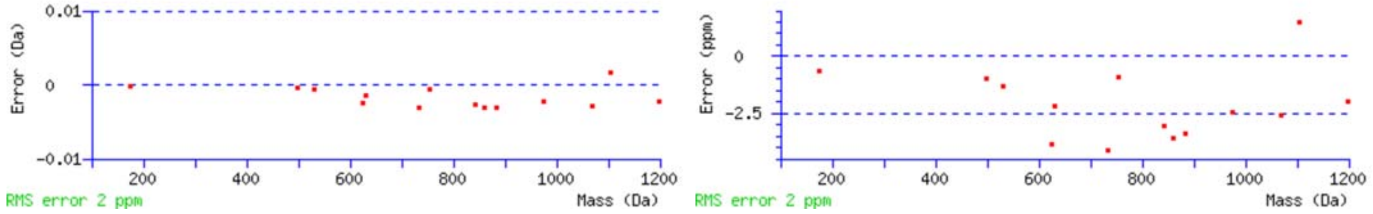

NCBI **BLAST** search of VRQLEQENAELETTLLER
(Parameters: blastp, nr protein database, expect=20000, no filter, PAM30)
Other BLAST web gateways

All matches to this query

Score	Mr(calc)	Delta	Sequence
67.3	2170.1229	-0.0022	VRQLEQENAELETTLLER
0.6	2170.1018	0.0189	EVGGGGVGGGLLENANPLIYQR
0.6	2170.1018	0.0189	QVGGGGVGGGLLENANPLIYQR
0.3	2168.1122	2.0085	QGLLFSTLLLAGLAQFCCR
0.3	2169.0962	1.0245	QGLLFSTLLLAGLAQFCCR

Mascot: http://www.matrixscience.com/

Mascot Search Results

Peptide View

MS/MS Fragmentation of **SLHQLVEADKCGTQK**
Found in **ch17u_O76014|KRT37_HUMAN** in **uni_human**, Keratin, type I cuticular Ha7 OS=Homo sapiens GN=KRT37 PE=3 SV=3

Match to Query 23141: 1712.852202 from(571.958010,3+) intensity(1212948.0000) rtinseconds(730) scans(3361) index(1983)
Title: 160219_Sunil_SDSI_A_Spectrum054091_scans__3361_RTINSECONDS=730
Data file L:\\QE_2016\\160219_Sunil_KAP_LKC\\TMgf\\T\\T160219_Sunil_SDSI_A.mgf

Click mouse within plot area to zoom in by factor of two about that point
Or, Plot from 100 to 1600 Da Full range
Label all possible matches ○ Label matches used for scoring ◉
Show Y-axis ☐

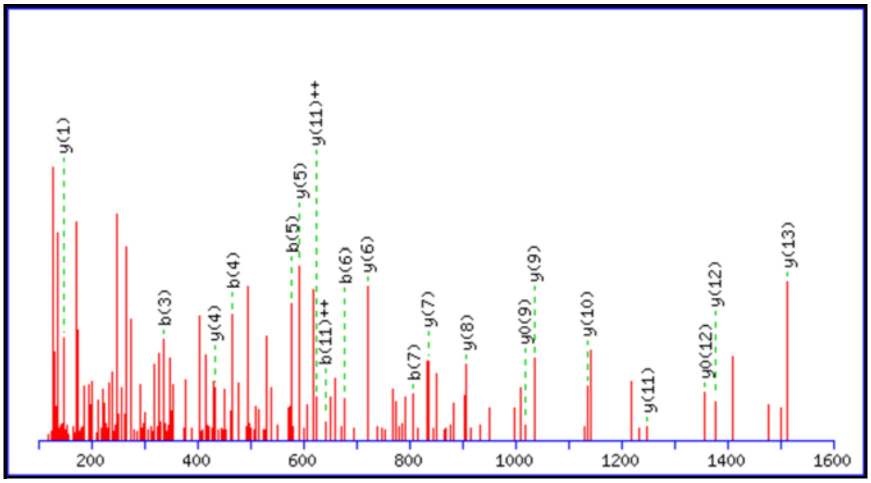

Monoisotopic mass of neutral peptide Mr(calc): 1712.8516
Fixed modifications: Carbamidomethyl (C) (apply to specified residues or termini only)
Ions Score: 48 **Expect:** 0.0021
Matches : 20/158 fragment ions using 60 most intense peaks (help)

#	b	b^{++}	b*	b^{*++}	b^0	b^{0++}	Seq.	y	y^{++}	y*	y^{*++}	y^0	y^{0++}	#
1	88.0393	44.5233			70.0287	35.5180	S							15
2	201.1234	101.0653			183.1128	92.0600	L	1626.8268	813.9170	1609.8003	805.4038	1608.8163	804.9118	14
3	**338.1823**	169.5948			320.1717	160.5895	H	***1513.7428***	757.3750	1496.7162	748.8617	1495.7322	748.3697	13
4	**466.2409**	233.6241	449.2143	225.1108	448.2303	224.6188	Q	***1376.6838***	688.8456	1359.6573	680.3323	1358.6733	679.8403	12
5	**579.3249**	290.1661	562.2984	281.6528	561.3144	281.1608	L	***1248.6253***	624.8163	1231.5987	616.3030	1230.6147	615.8110	11
6	**678.3933**	339.7003	661.3668	331.1870	660.3828	330.6950	V	***1135.5412***	568.2742	1118.5147	559.7610	1117.5306	559.2690	10
7	**807.4359**	404.2216	790.4094	395.7083	789.4254	395.2163	E	***1036.4728***	518.7400	1019.4462	510.2268	1018.4622	509.7347	9
8	878.4730	439.7402	861.4465	431.2269	860.4625	430.7349	A	***907.4302***	454.2187	890.4036	445.7055	889.4196	445.2135	8
9	993.5000	497.2536	976.4734	488.7404	975.4894	488.2483	D	***836.3931***	418.7002	819.3665	410.1869	818.3825	409.6949	7
10	1121.5949	561.3011	1104.5684	552.7878	1103.5844	552.2958	K	***721.3661***	361.1867	704.3396	352.6734	703.3556	352.1814	6
11	1281.6256	**641.3164**	1264.5990	632.8032	1263.6150	632.3112	C	***593.2712***	297.1392	576.2446	288.6260	575.2606	288.1339	5
12	1338.6471	669.8272	1321.6205	661.3139	1320.6365	660.8219	G	***433.2405***	217.1239	416.2140	208.6106	415.2300	208.1186	4
13	1439.6947	720.3510	1422.6682	711.8377	1421.6842	711.3457	T	376.2191	188.6132	359.1925	180.0999	358.2085	179.6079	3
14	1567.7533	784.3803	1550.7268	775.8670	1549.7428	775.3750	Q	275.1714	138.0893	258.1448	129.5761			2
15							K	***147.1128***	74.0600	130.0863	65.5468			1

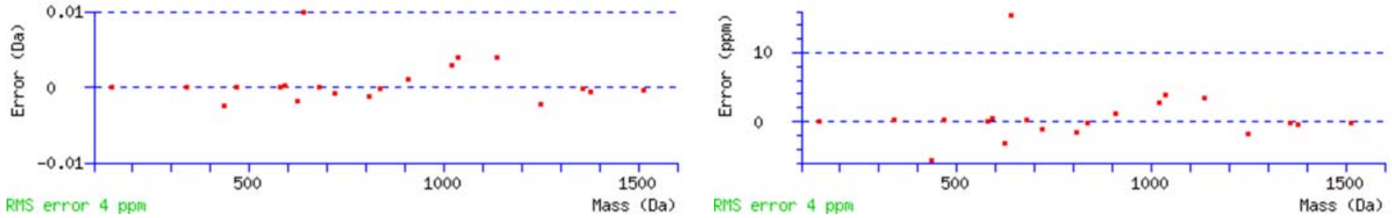

NCBI **BLAST** search of SLHQLVEADKCGTQK
(Parameters: blastp, nr protein database, expect=20000, no filter, PAM30)
Other BLAST web gateways

All matches to this query

Score	Mr(calc)	Delta	Sequence
48.3	1712.8516	0.0006	SLHQLVEADKCGTQK

Mascot: http://www.matrixscience.com/

Mascot Search Results

Peptide View

MS/MS Fragmentation of **LIVQIDNAKLAADDFR**
Found in **ch17u_O76014|KRT37_HUMAN** in **uni_human**, Keratin, type I cuticular Ha7 OS=Homo sapiens GN=KRT37 PE=3 SV=3

Match to Query 24767: 1800.976092 from(601.332640,3+) intensity(527309.5000) rtinseconds(2546) scans(13914) index(10898)
Title: 160219_Sunil_SDSI_A_Spectrum063023_scans__13914_RTINSECONDS=2546
Data file L:\\QE_2016\\160219_Sunil_KAP_LKC\\TMgf\\T\\T160219_Sunil_SDSI_A.mgf

Click mouse within plot area to zoom in by factor of two about that point
Or, Plot from 100 to 1400 Da Full range
Label all possible matches ○ Label matches used for scoring ◉
Show Y-axis □

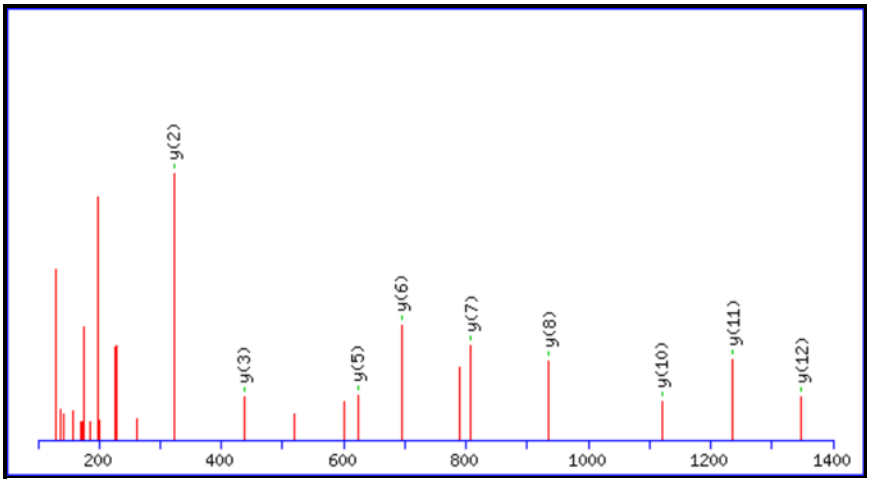

Monoisotopic mass of neutral peptide Mr(calc): 1800.9734
Fixed modifications: Carbamidomethyl (C) (apply to specified residues or termini only)
Ions Score: 86 **Expect:** 3e-007
Matches : 9/160 fragment ions using 10 most intense peaks (help)

#	b	b^{++}	b*	b^{*++}	b^{0}	b^{0++}	Seq.	y	y^{++}	y*	y^{*++}	y^{0}	y^{0++}	#
1	114.0913	57.5493					L							16
2	227.1754	114.0913					I	1688.8966	844.9519	1671.8701	836.4387	1670.8860	835.9467	15
3	326.2438	163.6255					V	1575.8125	788.4099	1558.7860	779.8966	1557.8020	779.4046	14
4	454.3024	227.6548	437.2758	219.1416			Q	1476.7441	738.8757	1459.7176	730.3624	1458.7336	729.8704	13
5	567.3865	284.1969	550.3599	275.6836			I	***1348.6856***	674.8464	1331.6590	666.3331	1330.6750	665.8411	12
6	682.4134	341.7103	665.3869	333.1971	664.4028	332.7051	D	***1235.6015***	618.3044	1218.5749	609.7911	1217.5909	609.2991	11
7	796.4563	398.7318	779.4298	390.2185	778.4458	389.7265	N	***1120.5745***	560.7909	1103.5480	552.2776	1102.5640	551.7856	10
8	867.4934	434.2504	850.4669	425.7371	849.4829	425.2451	A	1006.5316	503.7694	989.5051	495.2562	988.5211	494.7642	9
9	995.5884	498.2978	978.5619	489.7846	977.5778	489.2926	K	***935.4945***	468.2509	918.4680	459.7376	917.4839	459.2456	8
10	1108.6725	554.8399	1091.6459	546.3266	1090.6619	545.8346	L	***807.3995***	404.2034	790.3730	395.6901	789.3890	395.1981	7
11	1179.7096	590.3584	1162.6830	581.8452	1161.6990	581.3531	A	***694.3155***	347.6614	677.2889	339.1481	676.3049	338.6561	6
12	1250.7467	625.8770	1233.7201	617.3637	1232.7361	616.8717	A	***623.2784***	312.1428	606.2518	303.6295	605.2678	303.1375	5
13	1365.7736	683.3905	1348.7471	674.8772	1347.7631	674.3852	D	552.2413	276.6243	535.2147	268.1110	534.2307	267.6190	4
14	1480.8006	740.9039	1463.7740	732.3907	1462.7900	731.8986	D	***437.2143***	219.1108	420.1878	210.5975	419.2037	210.1055	3
15	1627.8690	814.4381	1610.8425	805.9249	1609.8584	805.4329	F	***322.1874***	161.5973	305.1608	153.0840			2
16							R	175.1190	88.0631	158.0924	79.5498			1

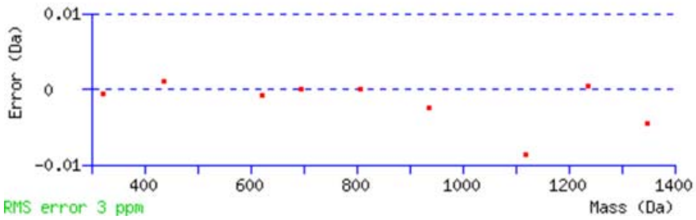

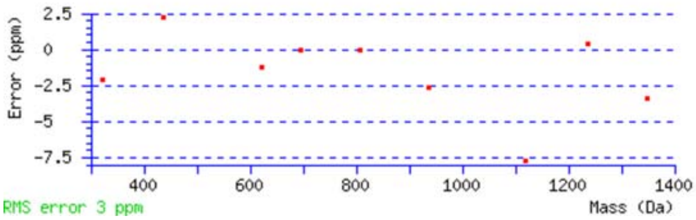

NCBI **BLAST** search of LIVQIDNAKLAADDFR
(Parameters: blastp, nr protein database, expect=20000, no filter, PAM30)
Other BLAST web gateways

All matches to this query

Score	Mr(calc)	Delta	Sequence
85.7	1800.9734	0.0027	LIVQIDNAKLAADDFR
85.7	1800.9734	0.0027	LVLQIDNAKLAADDFR
0.9	1800.9635	0.0126	STVNKWTLKGIHNFR
0.4	1799.9716	1.0045	AGQKPWLLVIRSMER

Mascot: http://www.matrixscience.com/

Mascot Search Results

Peptide View

MS/MS Fragmentation of **LIVQIDNAKLAADDFR**
Found in **ch17u_O76014|KRT37_HUMAN** in **uni_human**, Keratin, type I cuticular Ha7 OS=Homo sapiens GN=KRT37 PE=3 SV=3

Match to Query 24767: 1800.976092 from(601.332640,3+) intensity(527309.5000) rtinseconds(2546) scans(13914) index(10898)
Title: 160219_Sunil_SDSI_A_Spectrum063023_scans__13914_RTINSECONDS=2546
Data file L:\\QE_2016\\160219_Sunil_KAP_LKC\\TMgf\\T\\T160219_Sunil_SDSI_A.mgf

Click mouse within plot area to zoom in by factor of two about that point
Or, Plot from 100 to 1400 Da Full range
Label all possible matches ○ Label matches used for scoring ◉
Show Y-axis □

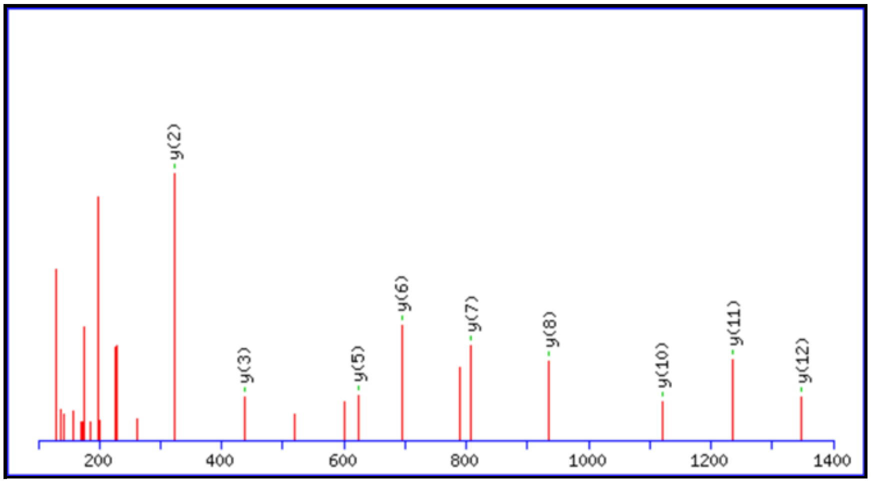

Monoisotopic mass of neutral peptide Mr(calc): 1800.9734
Fixed modifications: Carbamidomethyl (C) (apply to specified residues or termini only)
Ions Score: 86 **Expect:** 3e-007
Matches : 9/160 fragment ions using 10 most intense peaks (help)

#	b	b^{++}	b*	b^{*++}	b^{0}	b^{0++}	Seq.	y	y^{++}	y*	y^{*++}	y^{0}	y^{0++}	#
1	114.0913	57.5493					L							16
2	227.1754	114.0913					I	1688.8966	844.9519	1671.8701	836.4387	1670.8860	835.9467	15
3	326.2438	163.6255					V	1575.8125	788.4099	1558.7860	779.8966	1557.8020	779.4046	14
4	454.3024	227.6548	437.2758	219.1416			Q	1476.7441	738.8757	1459.7176	730.3624	1458.7336	729.8704	13
5	567.3865	284.1969	550.3599	275.6836			I	***1348.6856***	674.8464	1331.6590	666.3331	1330.6750	665.8411	12
6	682.4134	341.7103	665.3869	333.1971	664.4028	332.7051	D	***1235.6015***	618.3044	1218.5749	609.7911	1217.5909	609.2991	11
7	796.4563	398.7318	779.4298	390.2185	778.4458	389.7265	N	***1120.5745***	560.7909	1103.5480	552.2776	1102.5640	551.7856	10
8	867.4934	434.2504	850.4669	425.7371	849.4829	425.2451	A	1006.5316	503.7694	989.5051	495.2562	988.5211	494.7642	9
9	995.5884	498.2978	978.5619	489.7846	977.5778	489.2926	K	***935.4945***	468.2509	918.4680	459.7376	917.4839	459.2456	8
10	1108.6725	554.8399	1091.6459	546.3266	1090.6619	545.8346	L	***807.3995***	404.2034	790.3730	395.6901	789.3890	395.1981	7
11	1179.7096	590.3584	1162.6830	581.8452	1161.6990	581.3531	A	***694.3155***	347.6614	677.2889	339.1481	676.3049	338.6561	6
12	1250.7467	625.8770	1233.7201	617.3637	1232.7361	616.8717	A	***623.2784***	312.1428	606.2518	303.6295	605.2678	303.1375	5
13	1365.7736	683.3905	1348.7471	674.8772	1347.7631	674.3852	D	552.2413	276.6243	535.2147	268.1110	534.2307	267.6190	4
14	1480.8006	740.9039	1463.7740	732.3907	1462.7900	731.8986	D	***437.2143***	219.1108	420.1878	210.5975	419.2037	210.1055	3
15	1627.8690	814.4381	1610.8425	805.9249	1609.8584	805.4329	F	***322.1874***	161.5973	305.1608	153.0840			2
16							R	175.1190	88.0631	158.0924	79.5498			1

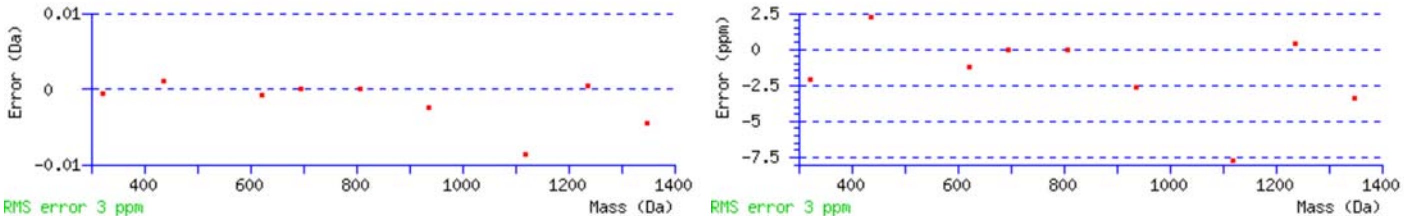

NCBI **BLAST** search of LIVQIDNAKLAADDFR
(Parameters: blastp, nr protein database, expect=20000, no filter, PAM30)
Other BLAST web gateways

All matches to this query

Score	Mr(calc)	Delta	Sequence
85.7	1800.9734	0.0027	LIVQIDNAKLAADDFR
85.7	1800.9734	0.0027	LVLQIDNAKLAADDFR
0.9	1800.9635	0.0126	STVNKWTLKGIHNFR
0.4	1799.9716	1.0045	AGQKPWLLVIRSMER

Mascot: http://www.matrixscience.com/

Mascot Search Results

Peptide View

MS/MS Fragmentation of **LIVQIDNAKLAADDFR**
Found in **ch17u_O76014|KRT37_HUMAN** in **uni_human**, Keratin, type I cuticular Ha7 OS=Homo sapiens GN=KRT37 PE=3 SV=3

Match to Query 24789: 1801.975302 from(601.665710,3+) intensity(1111974.1250) rtinseconds(2432) scans(13398) index(25884)
Title: 160219_Sunil_SDSI_A_Spectrum079190_scans__13398_RTINSECONDS=2432
Data file L:\\QE_2016\\160219_Sunil_KAP_LKC\\TMgf\\T\\T160219_Sunil_SDSI_A.mgf

Click mouse within plot area to zoom in by factor of two about that point
Or, Plot from 100 to 1300 Da Full range
Label all possible matches ○ Label matches used for scoring ◉
Show Y-axis ☐

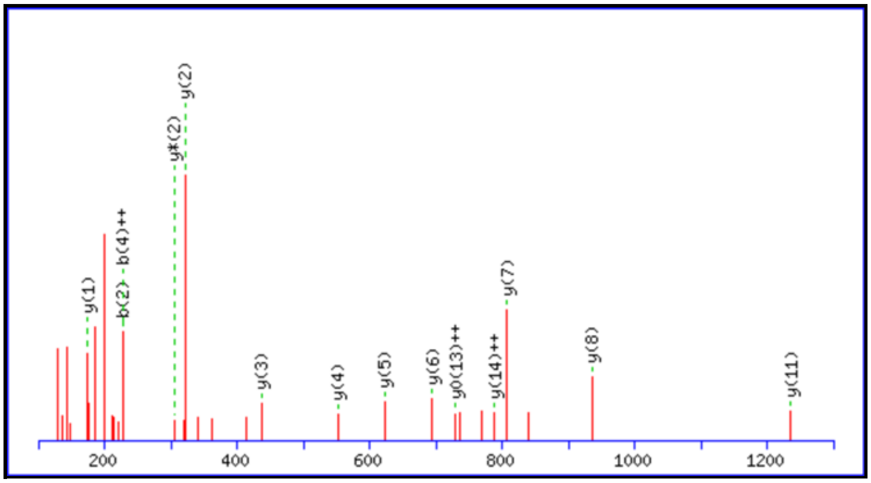

Monoisotopic mass of neutral peptide Mr(calc): 1801.9574
Fixed modifications: Carbamidomethyl (C) (apply to specified residues or termini only)
Variable modifications:
Q4 : Deamidated (NQ)
Ions Score: 56 **Expect:** 0.00026
Matches : 14/160 fragment ions using 26 most intense peaks (help)

#	b	b++	b*	b*++	b0	b0++	Seq.	y	y++	y*	y*++	y0	y0++	#
1	114.0913	57.5493					L							16
2	**227.1754**	114.0913					I	1689.8806	845.4440	1672.8541	836.9307	1671.8701	836.4387	15
3	326.2438	163.6255					V	1576.7966	788.9019	1559.7700	780.3886	1558.7860	779.8966	14
4	455.2864	**228.1468**	438.2599	219.6336			Q	1477.7281	739.3677	1460.7016	730.8544	1459.7176	730.3624	13
5	568.3705	284.6889	551.3439	276.1756			I	1348.6856	674.8464	1331.6590	666.3331	1330.6750	665.8411	12
6	683.3974	342.2023	666.3709	333.6891	665.3869	333.1971	D	***1235.6015***	618.3044	1218.5749	609.7911	1217.5909	609.2991	11
7	797.4403	399.2238	780.4138	390.7105	779.4298	390.2185	N	1120.5745	560.7909	1103.5480	552.2776	1102.5640	551.7856	10
8	868.4775	434.7424	851.4509	426.2291	850.4669	425.7371	A	1006.5316	503.7694	989.5051	495.2562	988.5211	494.7642	9
9	996.5724	498.7898	979.5459	490.2766	978.5619	489.7846	K	***935.4945***	468.2509	918.4680	459.7376	917.4839	459.2456	8
10	1109.6565	555.3319	1092.6299	546.8186	1091.6459	546.3266	L	***807.3995***	404.2034	790.3730	395.6901	789.3890	395.1981	7
11	1180.6936	590.8504	1163.6671	582.3372	1162.6830	581.8452	A	***694.3155***	347.6614	677.2889	339.1481	676.3049	338.6561	6
12	1251.7307	626.3690	1234.7042	617.8557	1233.7201	617.3637	A	***623.2784***	312.1428	606.2518	303.6295	605.2678	303.1375	5
13	1366.7577	683.8825	1349.7311	675.3692	1348.7471	674.8772	D	***552.2413***	276.6243	535.2147	268.1110	534.2307	267.6190	4
14	1481.7846	741.3959	1464.7581	732.8827	1463.7740	732.3907	D	***437.2143***	219.1108	420.1878	210.5975	419.2037	210.1055	3
15	1628.8530	814.9301	1611.8265	806.4169	1610.8425	805.9249	F	***322.1874***	161.5973	305.1608	153.0840			2
16							R	***175.1190***	88.0631	158.0924	79.5498			1

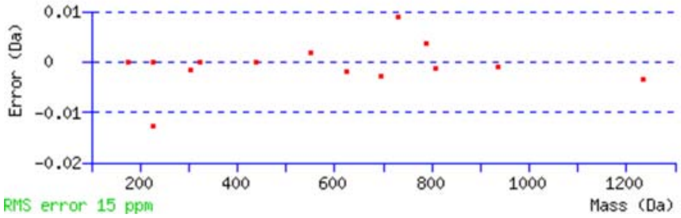

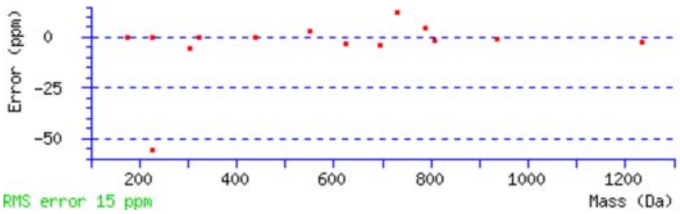

NCBI **BLAST** search of LIVQIDNAKLAADDFR
(Parameters: blastp, nr protein database, expect=20000, no filter, PAM30)
Other BLAST web gateways

All matches to this query

Score	Mr(calc)	Delta	Sequence	Site Analysis
56.2	1801.9574	0.0179	LIVQIDNAKLAADDFR	Deamidated Q4 96.50%
53.9	1800.9734	1.0019	LIVQIDNAKLAADDFR	
53.9	1800.9734	1.0019	LVLQIDNAKLAADDFR	
53.9	1801.9574	0.0179	LVLQIDNAKLAADDFR	
41.8	1801.9574	0.0179	LIVQIDNAKLAADDFR	Deamidated N7 3.50%
40.7	1801.9574	0.0179	LVLQIDNAKLAADDFR	

Mascot: http://www.matrixscience.com/

Mascot Search Results

Peptide View

MS/MS Fragmentation of **TIEELQQKILCSK**
Found in **ch17u_O76014|KRT37_HUMAN** in **uni_human**, Keratin, type I cuticular Ha7 OS=Homo sapiens GN=KRT37 PE=3 SV=3

Match to Query 20249: 1588.848972 from(530.623600,3+) intensity(2640728.7500) rtinseconds(1992) scans(10839) index(23789)
Title: 160219_Sunil_SDSI_A_Spectrum077094_scans__10839_RTINSECONDS=1992
Data file L:\\QE_2016\\160219_Sunil_KAP_LKC\\TMgf\\T\\T160219_Sunil_SDSI_A.mgf

Click mouse within plot area to zoom in by factor of two about that point
Or, Plot from 100 to 1600 Da Full range
Label all possible matches ○ Label matches used for scoring ◉
Show Y-axis ☐

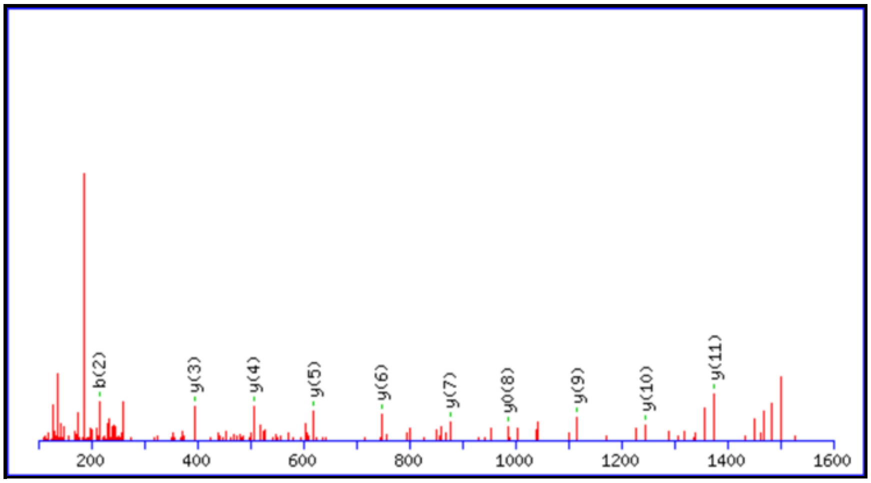

Monoisotopic mass of neutral peptide Mr(calc): 1588.8494
Fixed modifications: Carbamidomethyl (C) (apply to specified residues or termini only)
Ions Score: 57 **Expect:** 0.0003
Matches : 10/132 fragment ions using 15 most intense peaks (help)

#	b	b^{++}	b*	b^{*++}	b^0	b^{0++}	Seq.	y	y^{++}	y*	y^{*++}	y^0	y^{0++}	#
1	102.0550	51.5311			84.0444	42.5258	T							13
2	**215.1390**	108.0731			197.1285	99.0679	I	1488.8090	744.9082	1471.7825	736.3949	1470.7985	735.9029	12
3	344.1816	172.5944			326.1710	163.5892	E	***1375.7250***	688.3661	1358.6984	679.8529	1357.7144	679.3608	11
4	473.2242	237.1157			455.2136	228.1105	E	***1246.6824***	623.8448	1229.6558	615.3316	1228.6718	614.8395	10
5	586.3083	293.6578			568.2977	284.6525	L	***1117.6398***	559.3235	1100.6132	550.8103	1099.6292	550.3183	9
6	714.3668	357.6871	697.3403	349.1738	696.3563	348.6818	Q	1004.5557	502.7815	987.5292	494.2682	986.5452	493.7762	8
7	842.4254	421.7164	825.3989	413.2031	824.4149	412.7111	Q	***876.4972***	438.7522	859.4706	430.2389	858.4866	429.7469	7
8	970.5204	485.7638	953.4938	477.2506	952.5098	476.7585	K	***748.4386***	374.7229	731.4120	366.2096	730.4280	365.7176	6
9	1083.6045	542.3059	1066.5779	533.7926	1065.5939	533.3006	I	***620.3436***	310.6754	603.3171	302.1622	602.3330	301.6702	5
10	1196.6885	598.8479	1179.6620	590.3346	1178.6780	589.8426	L	***507.2595***	254.1334	490.2330	245.6201	489.2490	245.1281	4
11	1356.7192	678.8632	1339.6926	670.3499	1338.7086	669.8579	C	***394.1755***	197.5914	377.1489	189.0781	376.1649	188.5861	3
12	1443.7512	722.3792	1426.7246	713.8660	1425.7406	713.3740	S	234.1448	117.5761	217.1183	109.0628	216.1343	108.5708	2
13							K	147.1128	74.0600	130.0863	65.5468			1

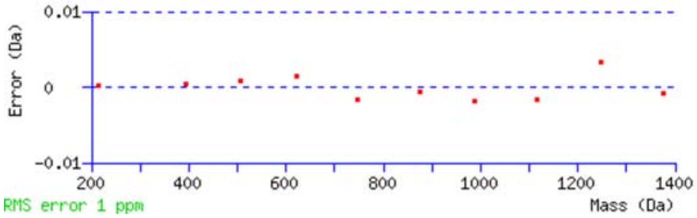

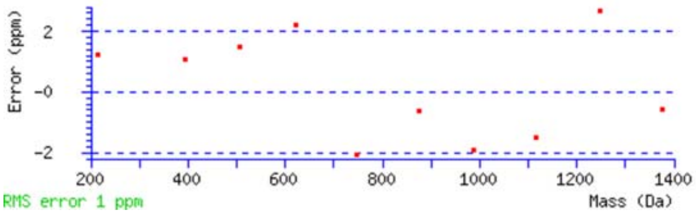

NCBI **BLAST** search of TIEELQQKILCSK
(Parameters: blastp, nr protein database, expect=20000, no filter, PAM30)
Other BLAST web gateways

All matches to this query

Score	Mr(calc)	Delta	Sequence
57.0	1588.8494	-0.0004	TIEELQQKILCSK

Mascot: http://www.matrixscience.com/

Mascot Search Results

Peptide View

MS/MS Fragmentation of **LENEIATYR**
Found in **ch17u_O76014|KRT37_HUMAN** in **uni_human**, Keratin, type I cuticular Ha7 OS=Homo sapiens GN=KRT37 PE=3 SV=3

Match to Query 5387: 1107.556488 from(554.785520,2+) intensity(1763561.8750) rtinseconds(1443) scans(7485) index(5450)
Title: 160219_Sunil_SDSI_A_Spectrum057558_scans__7485_RTINSECONDS=1443
Data file L:\\QE_2016\\160219_Sunil_KAP_LKC\\TMgf\\T\\T160219_Sunil_SDSI_A.mgf

Click mouse within plot area to zoom in by factor of two about that point
Or, Plot from 0 to 1200 Da Full range
Label all possible matches ○ Label matches used for scoring ◉
Show Y-axis ☐

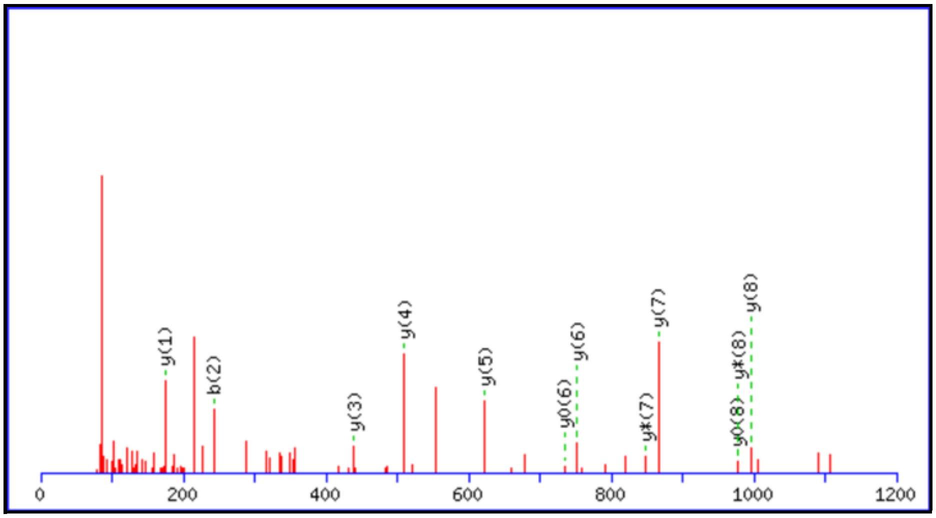

Monoisotopic mass of neutral peptide Mr(calc): 1107.5560
Fixed modifications: Carbamidomethyl (C) (apply to specified residues or termini only)
Ions Score: 49 **Expect:** 0.0016
Matches : 12/86 fragment ions using 22 most intense peaks (help)

#	b	b^{++}	b*	b^{*++}	b^0	b^{0++}	Seq.	y	y^{++}	y*	y^{*++}	y^0	y^{0++}	#
1	114.0913	57.5493					L							9
2	**243.1339**	122.0706			225.1234	113.0653	E	***995.4793***	498.2433	978.4527	489.7300	977.4687	489.2380	8
3	357.1769	179.0921	340.1503	170.5788	339.1663	170.0868	N	***866.4367***	433.7220	849.4101	425.2087	848.4261	424.7167	7
4	486.2195	243.6134	469.1929	235.1001	468.2089	234.6081	E	***752.3937***	376.7005	735.3672	368.1872	734.3832	367.6952	6
5	599.3035	300.1554	582.2770	291.6421	581.2930	291.1501	I	***623.3511***	312.1792	606.3246	303.6659	605.3406	303.1739	5
6	670.3406	335.6740	653.3141	327.1607	652.3301	326.6687	A	***510.2671***	255.6372	493.2405	247.1239	492.2565	246.6319	4
7	771.3883	386.1978	754.3618	377.6845	753.3777	377.1925	T	***439.2300***	220.1186	422.2034	211.6053	421.2194	211.1133	3
8	934.4516	467.7295	917.4251	459.2162	916.4411	458.7242	Y	338.1823	169.5948	321.1557	161.0815			2
9							R	***175.1190***	88.0631	158.0924	79.5498			1

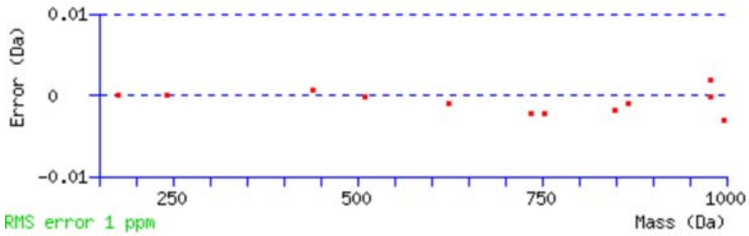

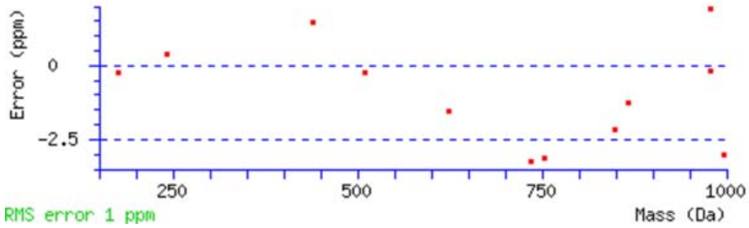

NCBI **BLAST** search of LENEIATYR
(Parameters: blastp, nr protein database, expect=20000, no filter, PAM30)
Other BLAST web gateways

All matches to this query

Score	Mr(calc)	Delta	Sequence
49.4	1107.5560	0.0005	LENEIATYR
11.1	1107.5560	0.0005	ELGGYVSNLR
5.1	1107.5560	0.0005	LYSSSGPELR
4.8	1107.5594	-0.0029	IEEMLSKSR
4.8	1107.5594	-0.0029	LQSMLKSER
3.9	1107.5672	-0.0108	IQESHPELR
3.6	1106.5641	0.9923	LTTEVMQIR
3.1	1107.5560	0.0005	LTFNITAGNR
0.6	1107.5495	0.0070	LCTIPHPDR
0.2	1107.5560	0.0005	LGNEVGYSIR

Mascot: http://www.matrixscience.com/

Mascot Search Results

Peptide View

MS/MS Fragmentation of **TIEELQQKILCSK**
Found in **ch17u_O76014|KRT37_HUMAN** in **uni_human**, Keratin, type I cuticular Ha7 OS=Homo sapiens GN=KRT37 PE=3 SV=3

Match to Query 20248: 1588.847268 from(795.430910,2+) intensity(1868119.0000) rtinseconds(1997) scans(10866) index(23813)
Title: 160219_Sunil_SDSI_A_Spectrum077118_scans__10866_RTINSECONDS=1997
Data file L:\\QE_2016\\160219_Sunil_KAP_LKC\\TMgf\\T\\T160219_Sunil_SDSI_A.mgf

Click mouse within plot area to zoom in by factor of two about that point
Or, Plot from 100 to 1600 Da Full range
Label all possible matches ○ Label matches used for scoring ◉
Show Y-axis ☐

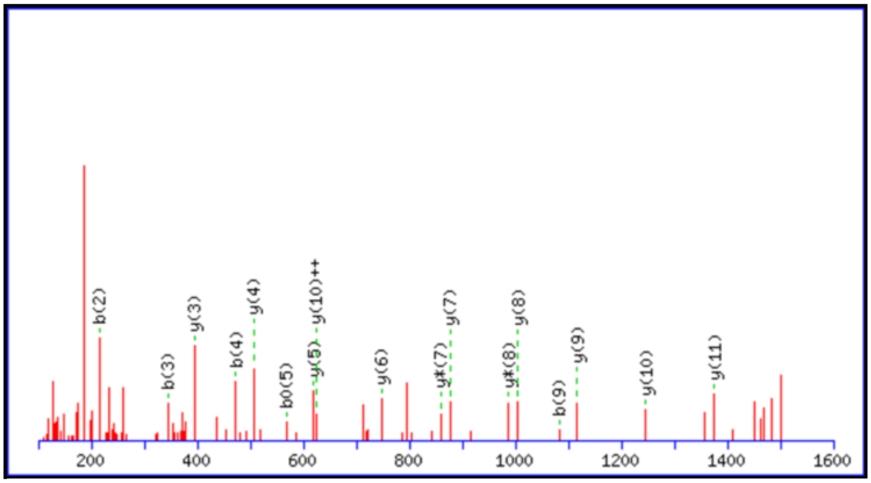

Monoisotopic mass of neutral peptide Mr(calc): 1588.8494
Fixed modifications: Carbamidomethyl (C) (apply to specified residues or termini only)
Ions Score: 70 **Expect:** 1.7e-005
Matches : 17/132 fragment ions using 26 most intense peaks (help)

#	b	b^{++}	b*	b^{*++}	b^0	b^{0++}	Seq.	y	y^{++}	y*	y^{*++}	y^0	y^{0++}	#
1	102.0550	51.5311			84.0444	42.5258	T							13
2	**215.1390**	108.0731			197.1285	99.0679	I	1488.8090	744.9082	1471.7825	736.3949	1470.7985	735.9029	12
3	**344.1816**	172.5944			326.1710	163.5892	E	***1375.7250***	688.3661	1358.6984	679.8529	1357.7144	679.3608	11
4	**473.2242**	237.1157			455.2136	228.1105	E	***1246.6824***	623.8448	1229.6558	615.3316	1228.6718	614.8395	10
5	586.3083	293.6578			568.2977	284.6525	L	***1117.6398***	559.3235	1100.6132	550.8103	1099.6292	550.3183	9
6	714.3668	357.6871	697.3403	349.1738	696.3563	348.6818	Q	***1004.5557***	502.7815	987.5292	494.2682	986.5452	493.7762	8
7	842.4254	421.7164	825.3989	413.2031	824.4149	412.7111	Q	***876.4972***	438.7522	859.4706	430.2389	858.4866	429.7469	7
8	970.5204	485.7638	953.4938	477.2506	952.5098	476.7585	K	***748.4386***	374.7229	731.4120	366.2096	730.4280	365.7176	6
9	**1083.6045**	542.3059	1066.5779	533.7926	1065.5939	533.3006	I	***620.3436***	310.6754	603.3171	302.1622	602.3330	301.6702	5
10	1196.6885	598.8479	1179.6620	590.3346	1178.6780	589.8426	L	***507.2595***	254.1334	490.2330	245.6201	489.2490	245.1281	4
11	1356.7192	678.8632	1339.6926	670.3499	1338.7086	669.8579	C	***394.1755***	197.5914	377.1489	189.0781	376.1649	188.5861	3
12	1443.7512	722.3792	1426.7246	713.8660	1425.7406	713.3740	S	234.1448	117.5761	217.1183	109.0628	216.1343	108.5708	2
13							K	147.1128	74.0600	130.0863	65.5468			1

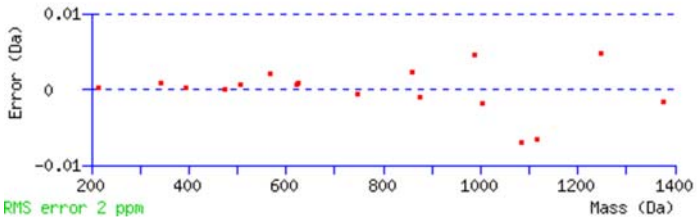

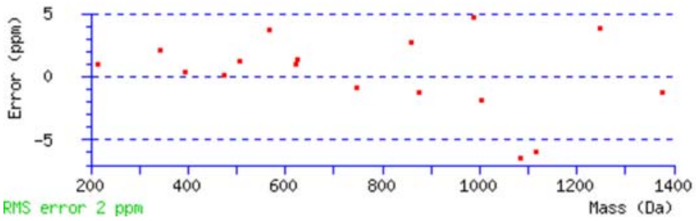

NCBI **BLAST** search of TIEELQQKILCSK
(Parameters: blastp, nr protein database, expect=20000, no filter, PAM30)
Other BLAST web gateways

All matches to this query

Score	Mr(calc)	Delta	Sequence
69.8	1588.8494	-0.0021	TIEELQQKILCSK

Mascot: http://www.matrixscience.com/

Mascot Search Results

Peptide View

MS/MS Fragmentation of **TIEELQQKILCSK**
Found in **ch17u_O76014|KRT37_HUMAN** in **uni_human**, Keratin, type I cuticular Ha7 OS=Homo sapiens GN=KRT37 PE=3 SV=3

Match to Query 20248: 1588.847268 from(795.430910,2+) intensity(1868119.0000) rtinseconds(1997) scans(10866) index(23813)
Title: 160219_Sunil_SDSI_A_Spectrum077118_scans__10866_RTINSECONDS=1997
Data file L:\\QE_2016\\160219_Sunil_KAP_LKC\\TMgf\\T\\T160219_Sunil_SDSI_A.mgf

Click mouse within plot area to zoom in by factor of two about that point
Or, Plot from 100 to 1600 Da Full range
Label all possible matches ○ Label matches used for scoring ◉
Show Y-axis ☐

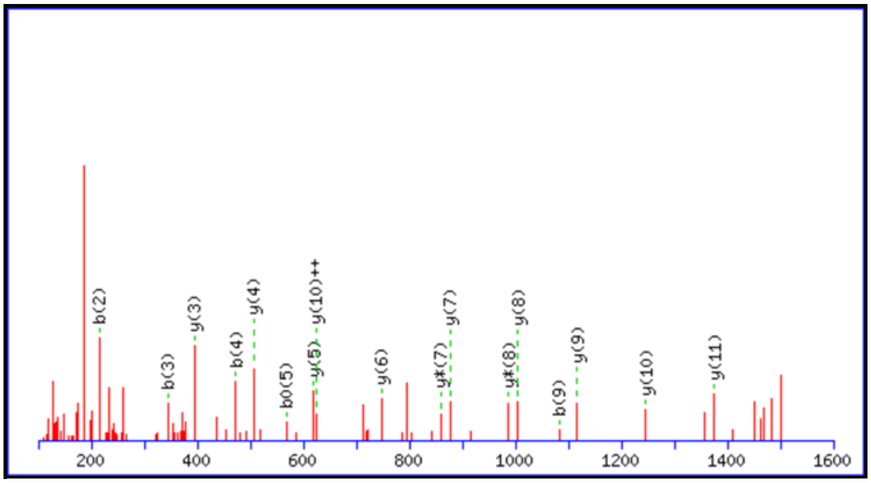

Monoisotopic mass of neutral peptide Mr(calc): 1588.8494
Fixed modifications: Carbamidomethyl (C) (apply to specified residues or termini only)
Ions Score: 70 **Expect:** 1.7e-005
Matches : 17/132 fragment ions using 26 most intense peaks (help)

#	b	b^{++}	b*	b^{*++}	b^{0}	b^{0++}	Seq.	y	y^{++}	y*	y^{*++}	y^{0}	y^{0++}	#
1	102.0550	51.5311			84.0444	42.5258	T							13
2	**215.1390**	108.0731			197.1285	99.0679	I	1488.8090	744.9082	1471.7825	736.3949	1470.7985	735.9029	12
3	**344.1816**	172.5944			326.1710	163.5892	E	***1375.7250***	688.3661	1358.6984	679.8529	1357.7144	679.3608	11
4	**473.2242**	237.1157			455.2136	228.1105	E	***1246.6824***	623.8448	1229.6558	615.3316	1228.6718	614.8395	10
5	586.3083	293.6578			568.2977	284.6525	L	***1117.6398***	559.3235	1100.6132	550.8103	1099.6292	550.3183	9
6	714.3668	357.6871	697.3403	349.1738	696.3563	348.6818	Q	***1004.5557***	502.7815	987.5292	494.2682	986.5452	493.7762	8
7	842.4254	421.7164	825.3989	413.2031	824.4149	412.7111	Q	***876.4972***	438.7522	859.4706	430.2389	858.4866	429.7469	7
8	970.5204	485.7638	953.4938	477.2506	952.5098	476.7585	K	***748.4386***	374.7229	731.4120	366.2096	730.4280	365.7176	6
9	**1083.6045**	542.3059	1066.5779	533.7926	1065.5939	533.3006	I	***620.3436***	310.6754	603.3171	302.1622	602.3330	301.6702	5
10	1196.6885	598.8479	1179.6620	590.3346	1178.6780	589.8426	L	***507.2595***	254.1334	490.2330	245.6201	489.2490	245.1281	4
11	1356.7192	678.8632	1339.6926	670.3499	1338.7086	669.8579	C	***394.1755***	197.5914	377.1489	189.0781	376.1649	188.5861	3
12	1443.7512	722.3792	1426.7246	713.8660	1425.7406	713.3740	S	234.1448	117.5761	217.1183	109.0628	216.1343	108.5708	2
13							K	147.1128	74.0600	130.0863	65.5468			1

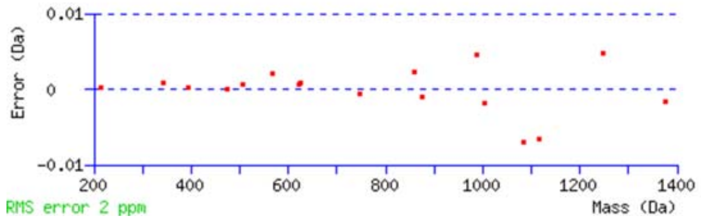

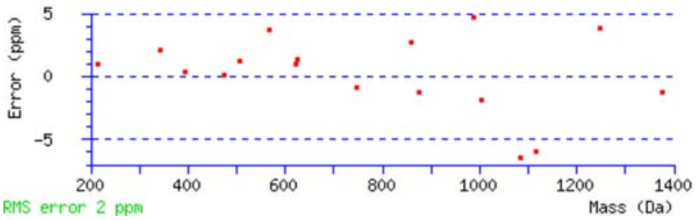

NCBI **BLAST** search of TIEELQQKILCSK
(Parameters: blastp, nr protein database, expect=20000, no filter, PAM30)
Other BLAST web gateways

All matches to this query

Score	Mr(calc)	Delta	Sequence
69.8	1588.8494	-0.0021	TIEELQQKILCSK

Mascot: http://www.matrixscience.com/

Mascot Search Results

Peptide View

MS/MS Fragmentation of **TIEELQQKILCSK**
Found in **ch17u_O76014|KRT37_HUMAN** in **uni_human**, Keratin, type I cuticular Ha7 OS=Homo sapiens GN=KRT37 PE=3 SV=3

Match to Query 20249: 1588.848972 from(530.623600,3+) intensity(2640728.7500) rtinseconds(1992) scans(10839) index(23789)
Title: 160219_Sunil_SDSI_A_Spectrum077094_scans__10839_RTINSECONDS=1992
Data file L:\\QE_2016\\160219_Sunil_KAP_LKC\\TMgf\\T\\T160219_Sunil_SDSI_A.mgf

Click mouse within plot area to zoom in by factor of two about that point
Or, Plot from 100 to 1600 Da Full range
Label all possible matches ○ Label matches used for scoring ◉
Show Y-axis ☐

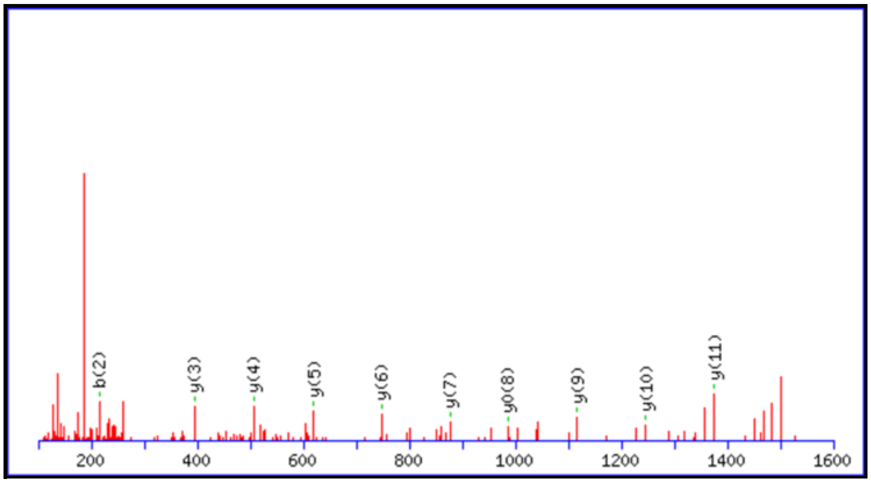

Monoisotopic mass of neutral peptide Mr(calc): 1588.8494
Fixed modifications: Carbamidomethyl (C) (apply to specified residues or termini only)
Ions Score: 57 **Expect:** 0.0003
Matches : 10/132 fragment ions using 15 most intense peaks (help)

#	b	b^{++}	b*	b^{*++}	b^0	b^{0++}	Seq.	y	y^{++}	y*	y^{*++}	y^0	y^{0++}	#
1	102.0550	51.5311			84.0444	42.5258	T							13
2	**215.1390**	108.0731			197.1285	99.0679	I	1488.8090	744.9082	1471.7825	736.3949	1470.7985	735.9029	12
3	344.1816	172.5944			326.1710	163.5892	E	***1375.7250***	688.3661	1358.6984	679.8529	1357.7144	679.3608	11
4	473.2242	237.1157			455.2136	228.1105	E	***1246.6824***	623.8448	1229.6558	615.3316	1228.6718	614.8395	10
5	586.3083	293.6578			568.2977	284.6525	L	***1117.6398***	559.3235	1100.6132	550.8103	1099.6292	550.3183	9
6	714.3668	357.6871	697.3403	349.1738	696.3563	348.6818	Q	1004.5557	502.7815	987.5292	494.2682	986.5452	493.7762	8
7	842.4254	421.7164	825.3989	413.2031	824.4149	412.7111	Q	***876.4972***	438.7522	859.4706	430.2389	858.4866	429.7469	7
8	970.5204	485.7638	953.4938	477.2506	952.5098	476.7585	K	***748.4386***	374.7229	731.4120	366.2096	730.4280	365.7176	6
9	1083.6045	542.3059	1066.5779	533.7926	1065.5939	533.3006	I	***620.3436***	310.6754	603.3171	302.1622	602.3330	301.6702	5
10	1196.6885	598.8479	1179.6620	590.3346	1178.6780	589.8426	L	***507.2595***	254.1334	490.2330	245.6201	489.2490	245.1281	4
11	1356.7192	678.8632	1339.6926	670.3499	1338.7086	669.8579	C	***394.1755***	197.5914	377.1489	189.0781	376.1649	188.5861	3
12	1443.7512	722.3792	1426.7246	713.8660	1425.7406	713.3740	S	234.1448	117.5761	217.1183	109.0628	216.1343	108.5708	2
13							K	147.1128	74.0600	130.0863	65.5468			1

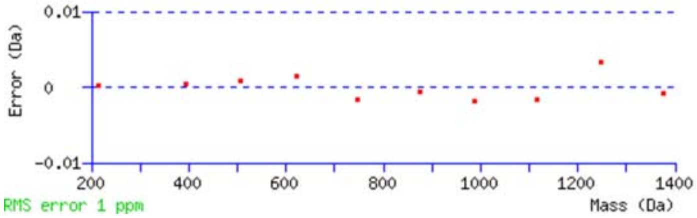

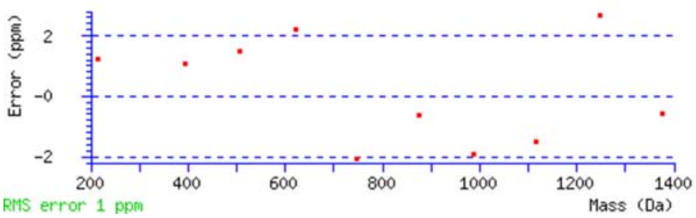

NCBI **BLAST** search of TIEELQQKILCSK
(Parameters: blastp, nr protein database, expect=20000, no filter, PAM30)
Other BLAST web gateways

All matches to this query

Score	Mr(calc)	Delta	Sequence
57.0	1588.8494	-0.0004	TIEELQQKILCSK

Mascot: http://www.matrixscience.com/

Mascot Search Results

Peptide View

MS/MS Fragmentation of **LAADDFR**
Found in **ch17u_O76014|KRT37_HUMAN** in **uni_human**, Keratin, type I cuticular Ha7 OS=Homo sapiens GN=KRT37 PE=3 SV=3

Match to Query 500: 806.390348 from(404.202450,2+) intensity(1269729.6250) rtinseconds(1072) scans(5316) index(3693)
Title: 160219_Sunil_SDSI_A_Spectrum055801_scans__5316_RTINSECONDS=1072
Data file L:\\QE_2016\\160219_Sunil_KAP_LKC\\TMgf\\T\\T160219_Sunil_SDSI_A.mgf

Click mouse within plot area to zoom in by factor of two about that point
Or, Plot from 50 to 750 Da Full range
Label all possible matches ○ Label matches used for scoring ◉
Show Y-axis ☐

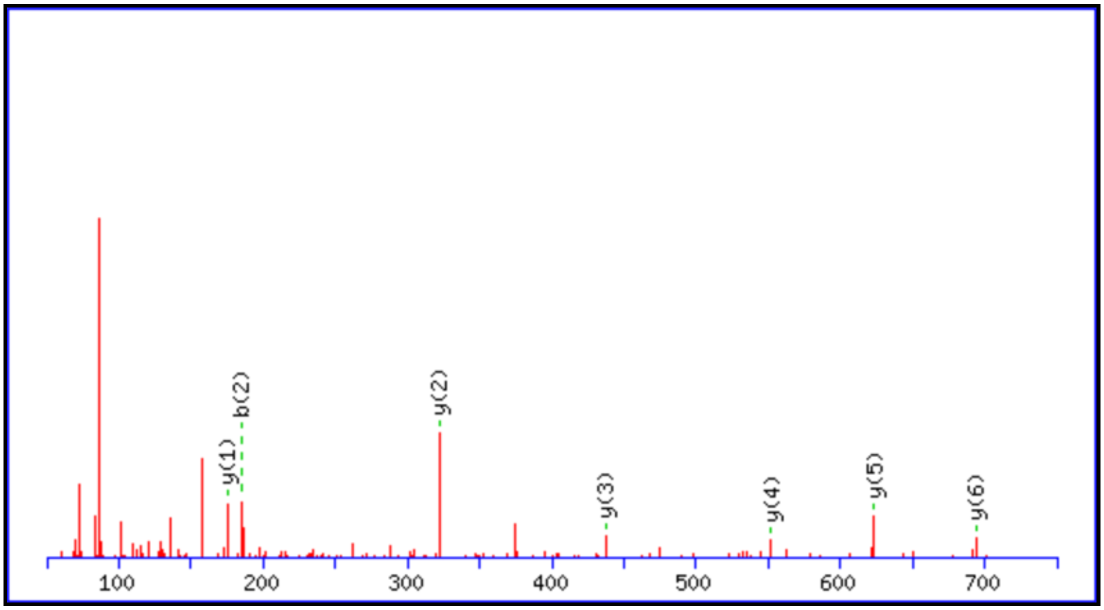

Monoisotopic mass of neutral peptide Mr(calc): 806.3923
Fixed modifications: Carbamidomethyl (C) (apply to specified residues or termini only)
Ions Score: 41 **Expect:** 0.0097
Matches : 7/50 fragment ions using 14 most intense peaks (help)

#	b	b^{++}	b^0	b^{0++}	Seq.	y	y^{++}	y*	y^{*++}	y^0	y^{0++}	#
1	114.0913	57.5493			L							7
2	**185.1285**	93.0679			A	***694.3155***	347.6614	677.2889	339.1481	676.3049	338.6561	6
3	256.1656	128.5864			A	***623.2784***	312.1428	606.2518	303.6295	605.2678	303.1375	5
4	371.1925	186.0999	353.1819	177.0946	D	***552.2413***	276.6243	535.2147	268.1110	534.2307	267.6190	4
5	486.2195	243.6134	468.2089	234.6081	D	***437.2143***	219.1108	420.1878	210.5975	419.2037	210.1055	3
6	633.2879	317.1476	615.2773	308.1423	F	***322.1874***	161.5973	305.1608	153.0840			2

7					R	*175.1190*	88.0631	158.0924	79.5498			1

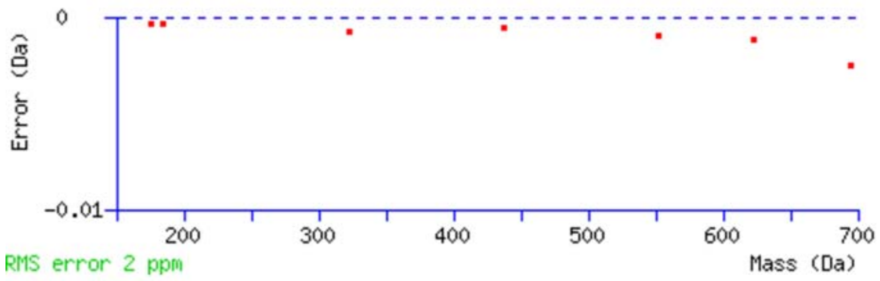

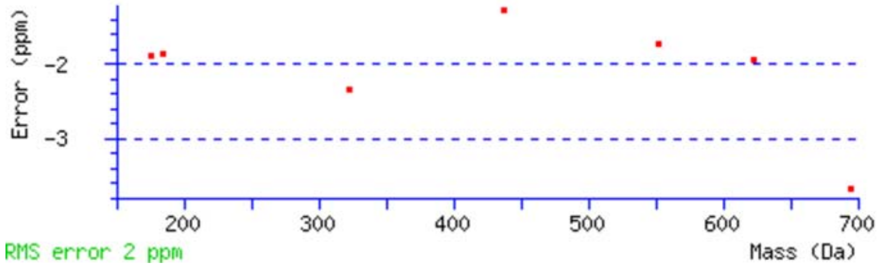

NCBI **BLAST** search of LAADDFR
(Parameters: blastp, nr protein database, expect=20000, no filter, PAM30)
Other BLAST web gateways

All matches to this query

Score	Mr(calc)	Delta	Sequence
40.9	806.3923	-0.0019	LAADDFR
11.9	804.3878	2.0025	RPDNFR
11.5	806.3844	0.0060	LAAQMQK
5.4	806.3956	-0.0053	LGDMSLR
4.9	805.3930	0.9974	DGASTSLR
4.1	806.3923	-0.0019	IAGFDER
3.4	806.3922	-0.0019	GELANFR
3.4	804.3878	2.0025	NRPDFR
3.4	806.3923	-0.0019	QLEDFR
3.3	806.3923	-0.0019	LDQQFR

Mascot: http://www.matrixscience.com/

Mascot Search Results

Peptide View

MS/MS Fragmentation of **LAADDFR**
Found in **ch17u_O76014|KRT37_HUMAN** in **uni_human**, Keratin, type I cuticular Ha7 OS=Homo sapiens GN=KRT37 PE=3 SV=3

Match to Query 501: 806.390848 from(404.202700,2+) intensity(1398080.3750) rtinseconds(1473) scans(7818) index(21125)
Title: 160219_Sunil_SDSI_A_Spectrum074429_scans__7818_RTINSECONDS=1473
Data file L:\\QE_2016\\160219_Sunil_KAP_LKC\\TMgf\\T\\T160219_Sunil_SDSI_A.mgf

Click mouse within plot area to zoom in by factor of two about that point
Or, Plot from 50 to 700 Da Full range
Label all possible matches ○ Label matches used for scoring ◉
Show Y-axis ☐

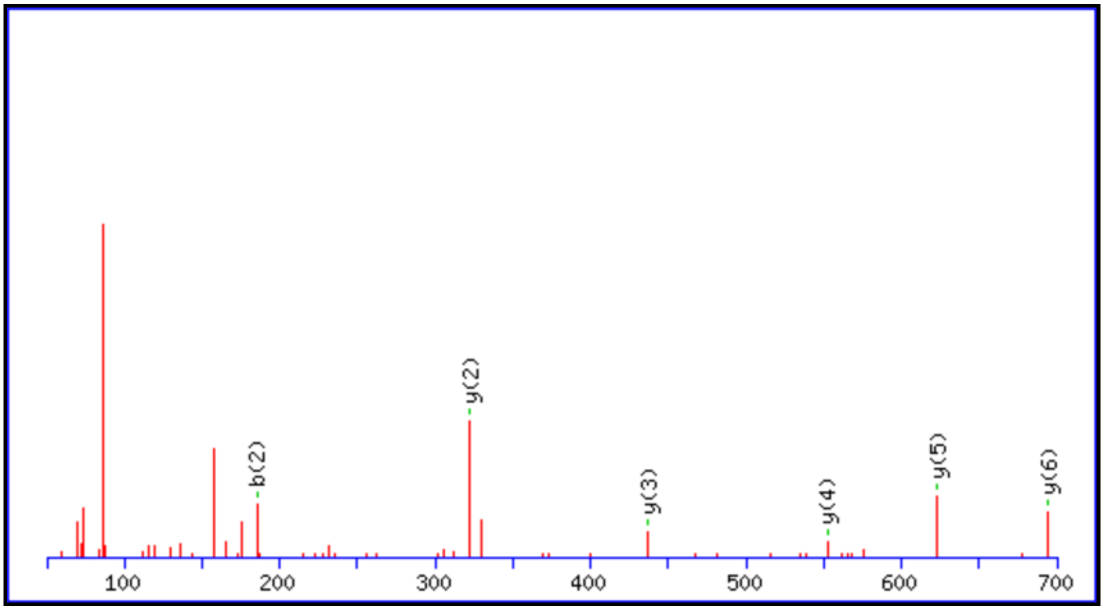

Monoisotopic mass of neutral peptide Mr(calc): 806.3923
Fixed modifications: Carbamidomethyl (C) (apply to specified residues or termini only)
Ions Score: 46 **Expect:** 0.0029
Matches : 6/50 fragment ions using 7 most intense peaks (help)

#	b	b^{++}	b^0	b^{0++}	Seq.	y	y^{++}	y*	y^{*++}	y^0	y^{0++}	#
1	114.0913	57.5493			L							7
2	**185.1285**	93.0679			A	***694.3155***	347.6614	677.2889	339.1481	676.3049	338.6561	6
3	256.1656	128.5864			A	***623.2784***	312.1428	606.2518	303.6295	605.2678	303.1375	5
4	371.1925	186.0999	353.1819	177.0946	D	***552.2413***	276.6243	535.2147	268.1110	534.2307	267.6190	4
5	486.2195	243.6134	468.2089	234.6081	D	***437.2143***	219.1108	420.1878	210.5975	419.2037	210.1055	3
6	633.2879	317.1476	615.2773	308.1423	F	***322.1874***	161.5973	305.1608	153.0840			2

7					R	175.1190	88.0631	158.0924	79.5498			1

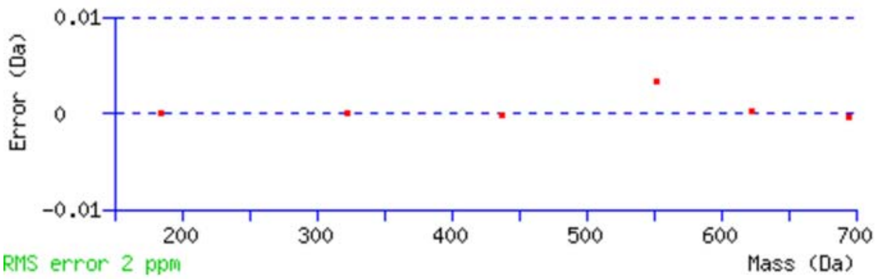

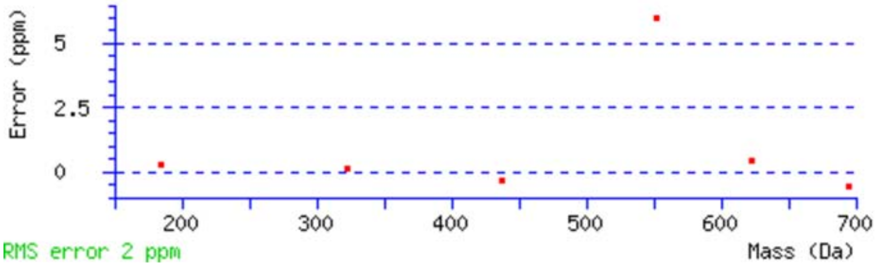

NCBI **BLAST** search of LAADDFR
(Parameters: blastp, nr protein database, expect=20000, no filter, PAM30)
Other BLAST web gateways

All matches to this query

Score	Mr(calc)	Delta	Sequence
46.1	806.3923	-0.0014	LAADDFR
13.5	806.3844	0.0065	LAAQMQK
12.5	804.3878	2.0030	RPDNFR
5.5	806.3923	-0.0014	IAGFDER
4.5	806.3923	-0.0014	LDQQFR
4.5	806.3923	-0.0014	LDQQFR
4.0	806.3922	-0.0014	GELANFR
4.0	804.3878	2.0030	NRPDFR
4.0	806.3923	-0.0014	QLEDFR
2.9	806.3891	0.0017	IACCRK

Mascot: http://www.matrixscience.com/

Mascot Search Results

Peptide View

MS/MS Fragmentation of **LAADDFR**
Found in **ch17u_O76014|KRT37_HUMAN** in **uni_human**, Keratin, type I cuticular Ha7 OS=Homo sapiens GN=KRT37 PE=3 SV=3

Match to Query 502: 806.390968 from(404.202760,2+) intensity(403740.6250) rtinseconds(1037) scans(5172) index(34085)
Title: 160219_Sunil_SDSI_A_Spectrum088670_scans__5172_RTINSECONDS=1037
Data file L:\\QE_2016\\160219_Sunil_KAP_LKC\\TMgf\\T\\T160219_Sunil_SDSI_A.mgf

Click mouse within plot area to zoom in by factor of two about that point
Or, Plot from 50 to 750 Da Full range
Label all possible matches ○ Label matches used for scoring ◉
Show Y-axis ☐

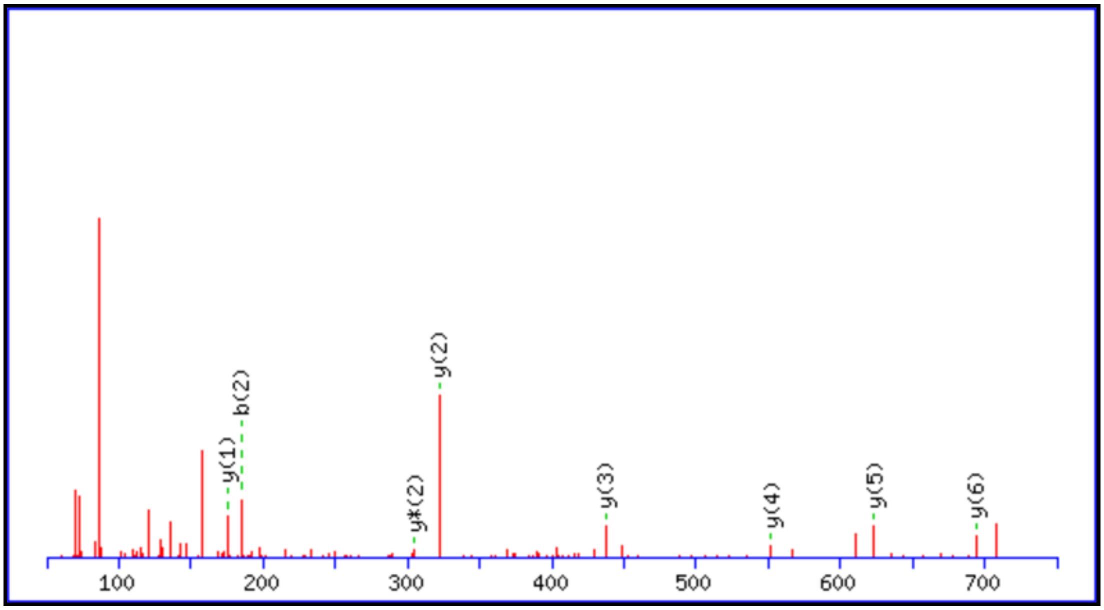

Monoisotopic mass of neutral peptide Mr(calc): 806.3923
Fixed modifications: Carbamidomethyl (C) (apply to specified residues or termini only)
Ions Score: 44 **Expect:** 0.0044
Matches : 8/50 fragment ions using 14 most intense peaks (help)

#	b	b^{++}	b^{0}	b^{0++}	Seq.	y	y^{++}	y*	y^{*++}	y^{0}	y^{0++}	#
1	114.0913	57.5493			L							7
2	**185.1285**	93.0679			A	***694.3155***	347.6614	677.2889	339.1481	676.3049	338.6561	6
3	256.1656	128.5864			A	***623.2784***	312.1428	606.2518	303.6295	605.2678	303.1375	5
4	371.1925	186.0999	353.1819	177.0946	D	***552.2413***	276.6243	535.2147	268.1110	534.2307	267.6190	4
5	486.2195	243.6134	468.2089	234.6081	D	***437.2143***	219.1108	420.1878	210.5975	419.2037	210.1055	3
6	633.2879	317.1476	615.2773	308.1423	F	***322.1874***	161.5973	305.1608	153.0840			2

7					R	*175.1190*	88.0631	158.0924	79.5498			1

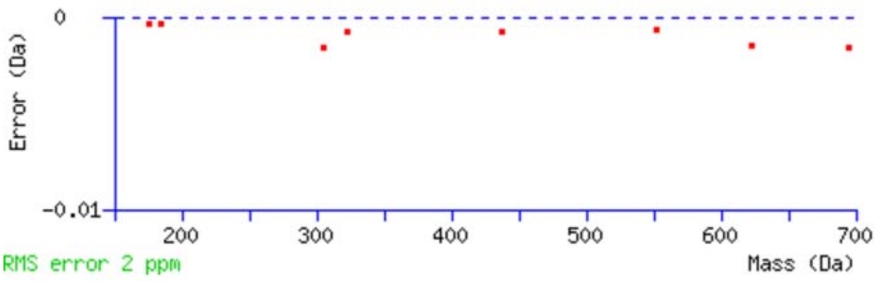

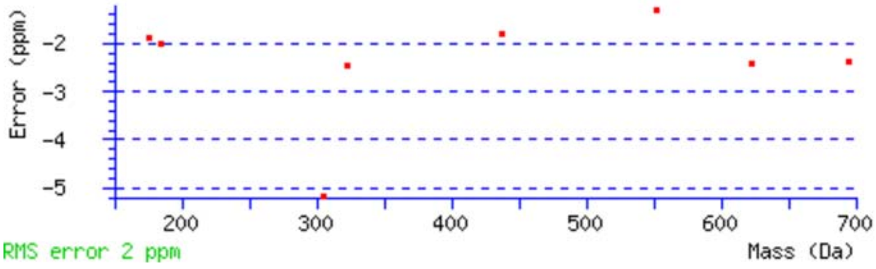

NCBI **BLAST** search of LAADDFR
(Parameters: blastp, nr protein database, expect=20000, no filter, PAM30)
Other BLAST web gateways

All matches to this query

Score	Mr(calc)	Delta	Sequence
44.3	806.3923	-0.0013	LAADDFR
14.8	804.3878	2.0031	RPDNFR
5.8	804.3919	1.9991	PWQAFR
5.8	806.3922	-0.0013	GELANFR
5.8	804.3878	2.0031	NRPDFR
5.8	806.3923	-0.0013	QLEDFR
5.6	806.3923	-0.0013	LDQQFR
5.6	806.3923	-0.0013	LDQQFR
3.7	806.3923	-0.0013	IAGFDER
2.7	806.3844	0.0066	LAAQMQK

Mascot: http://www.matrixscience.com/

Mascot Search Results

Peptide View

MS/MS Fragmentation of **LAADDFR**
Found in **ch17u_O76014|KRT37_HUMAN** in **uni_human**, Keratin, type I cuticular Ha7 OS=Homo sapiens GN=KRT37 PE=3 SV=3

Match to Query 503: 806.391028 from(404.202790,2+) intensity(1894143.7500) rtinseconds(1186) scans(5983) index(4208)
Title: 160219_Sunil_SDSI_A_Spectrum056316_scans__5983_RTINSECONDS=1186
Data file L:\\QE_2016\\160219_Sunil_KAP_LKC\\TMgf\\T\\T160219_Sunil_SDSI_A.mgf

Click mouse within plot area to zoom in by factor of two about that point
Or, Plot from 50 to 750 Da Full range
Label all possible matches ○ Label matches used for scoring ◉
Show Y-axis ☐

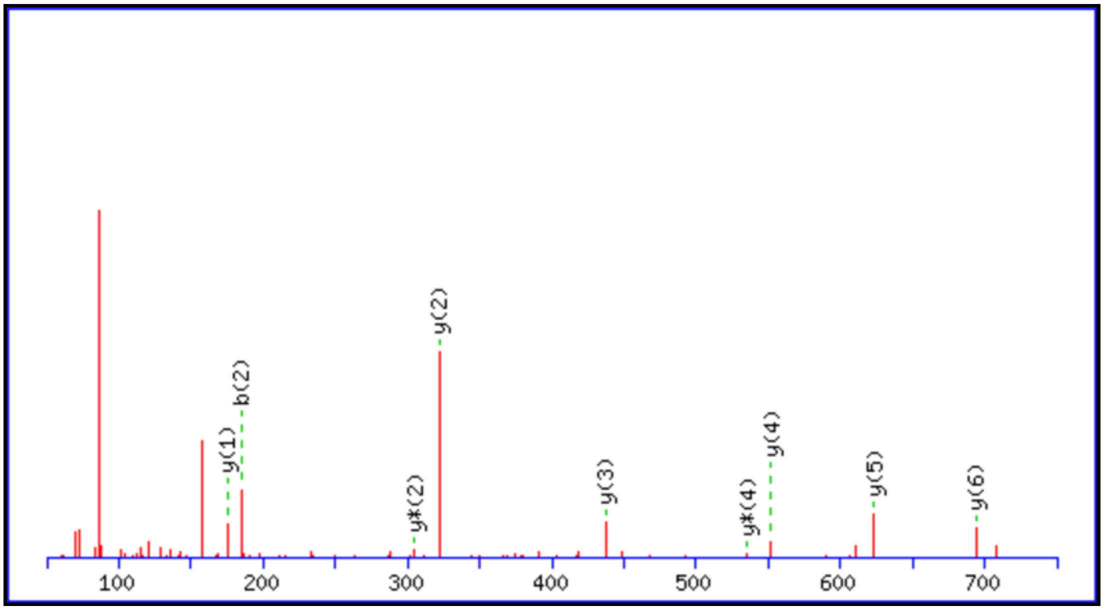

Monoisotopic mass of neutral peptide Mr(calc): 806.3923
Fixed modifications: Carbamidomethyl (C) (apply to specified residues or termini only)
Ions Score: 49 **Expect:** 0.0016
Matches : 9/50 fragment ions using 14 most intense peaks (help)

#	b	b^{++}	b^{0}	b^{0++}	Seq.	y	y^{++}	y*	y^{*++}	y^{0}	y^{0++}	#
1	114.0913	57.5493			L							7
2	**185.1285**	93.0679			A	***694.3155***	347.6614	677.2889	339.1481	676.3049	338.6561	6
3	256.1656	128.5864			A	***623.2784***	312.1428	606.2518	303.6295	605.2678	303.1375	5
4	371.1925	186.0999	353.1819	177.0946	D	***552.2413***	276.6243	535.2147	268.1110	534.2307	267.6190	4
5	486.2195	243.6134	468.2089	234.6081	D	***437.2143***	219.1108	420.1878	210.5975	419.2037	210.1055	3
6	633.2879	317.1476	615.2773	308.1423	F	***322.1874***	161.5973	305.1608	153.0840			2

7					R	*175.1190*	88.0631	158.0924	79.5498			1

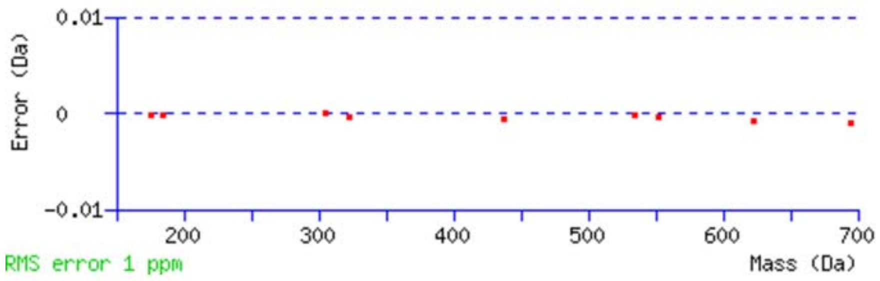

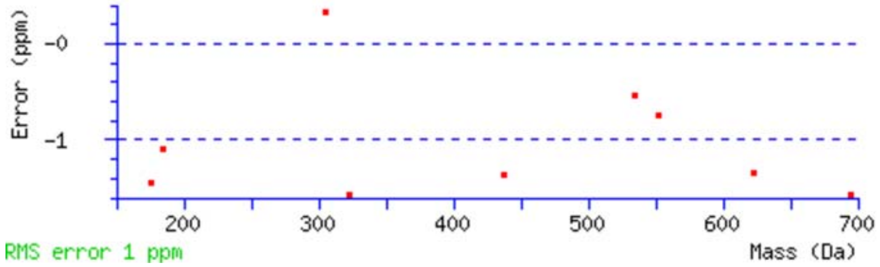

NCBI **BLAST** search of LAADDFR
(Parameters: blastp, nr protein database, expect=20000, no filter, PAM30)
Other BLAST web gateways

All matches to this query

Score	Mr(calc)	Delta	Sequence
48.7	806.3923	-0.0012	LAADDFR
18.3	804.3878	2.0032	RPDNFR
12.9	806.3844	0.0067	LAAQMQK
9.4	806.3923	-0.0012	IAGFDER
7.1	806.3922	-0.0012	GELANFR
7.1	804.3878	2.0032	NRPDFR
7.1	806.3923	-0.0012	QLEDFR
7.0	806.3923	-0.0012	LDQQFR
7.0	806.3923	-0.0012	LDQQFR
6.8	804.3919	1.9992	PWQAFR

Mascot: http://www.matrixscience.com/

Mascot Search Results

Peptide View

MS/MS Fragmentation of **LAADDFR**
Found in **ch17u_O76014|KRT37_HUMAN** in **uni_human**, Keratin, type I cuticular Ha7 OS=Homo sapiens GN=KRT37 PE=3 SV=3

Match to Query 507: 806.391568 from(404.203060,2+) intensity(1122270.1250) rtinseconds(926) scans(4529) index(33523)
Title: 160219_Sunil_SDSI_A_Spectrum088108_scans__4529_RTINSECONDS=926
Data file L:\\QE_2016\\160219_Sunil_KAP_LKC\\TMgf\\T\\T160219_Sunil_SDSI_A.mgf

Click mouse within plot area to zoom in by factor of two about that point
Or, Plot from 50 to 750 Da Full range
Label all possible matches ○ Label matches used for scoring ◉
Show Y-axis ☐

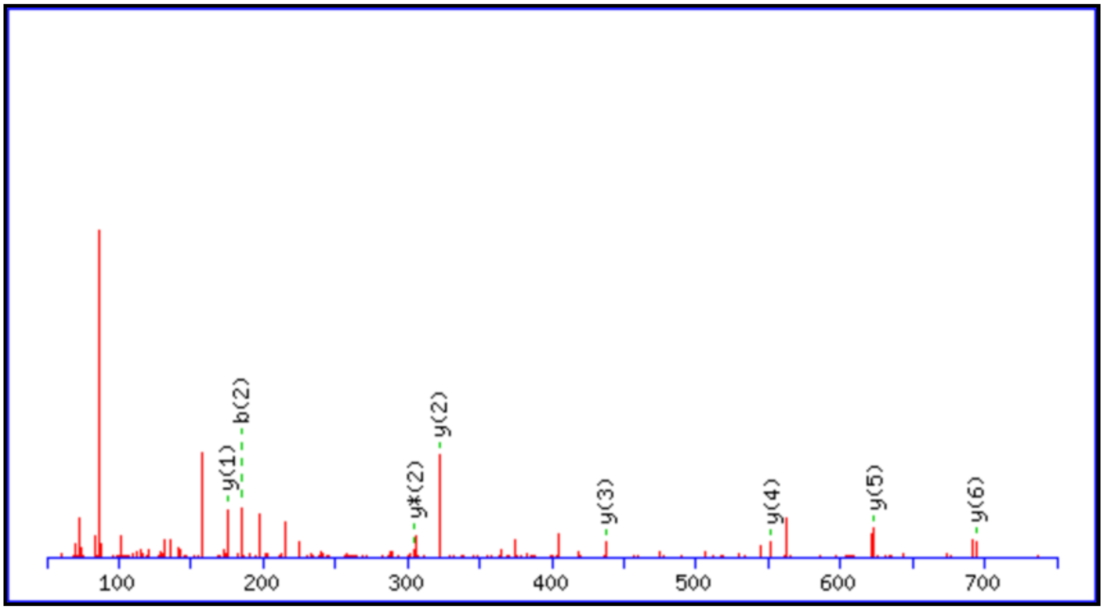

Monoisotopic mass of neutral peptide Mr(calc): 806.3923
Fixed modifications: Carbamidomethyl (C) (apply to specified residues or termini only)
Ions Score: 32 **Expect:** 0.074
Matches : 8/50 fragment ions using 21 most intense peaks (help)

#	b	b^{++}	b^0	b^{0++}	Seq.	y	y^{++}	y^*	y^{*++}	y^0	y^{0++}	#
1	114.0913	57.5493			L							7
2	**185.1285**	93.0679			A	***694.3155***	347.6614	677.2889	339.1481	676.3049	338.6561	6
3	256.1656	128.5864			A	***623.2784***	312.1428	606.2518	303.6295	605.2678	303.1375	5
4	371.1925	186.0999	353.1819	177.0946	D	***552.2413***	276.6243	535.2147	268.1110	534.2307	267.6190	4
5	486.2195	243.6134	468.2089	234.6081	D	***437.2143***	219.1108	420.1878	210.5975	419.2037	210.1055	3
6	633.2879	317.1476	615.2773	308.1423	F	***322.1874***	161.5973	305.1608	153.0840			2

7					R	*175.1190*	88.0631	158.0924	79.5498			1

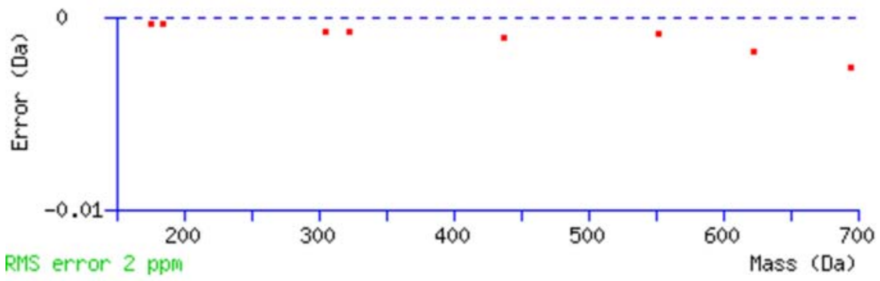

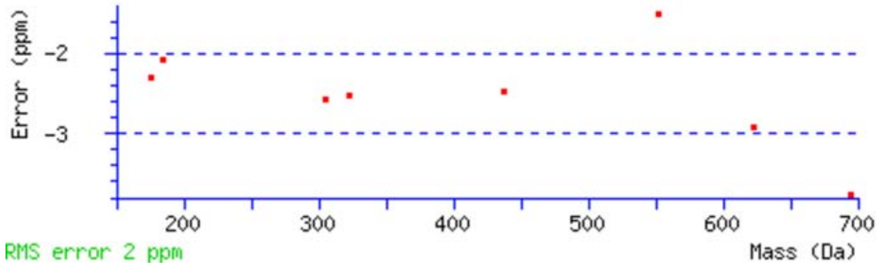

NCBI **BLAST** search of LAADDFR
(Parameters: blastp, nr protein database, expect=20000, no filter, PAM30)
Other BLAST web gateways

All matches to this query

Score	Mr(calc)	Delta	Sequence
31.8	806.3923	-0.0007	LAADDFR
7.6	804.3878	2.0037	RPDNFR
3.7	806.3923	-0.0007	IAGFDER
2.9	806.3923	-0.0007	LDQQFR
2.9	806.3923	-0.0007	LDQQFR
2.8	805.3930	0.9986	DGASTSLR
2.6	806.3844	0.0072	LAAQMQK
1.0	804.3912	2.0004	LASQACR
0.1	804.3800	2.0116	LEAEGMR

Mascot: http://www.matrixscience.com/

Mascot Search Results

Peptide View

MS/MS Fragmentation of **LAADDFR**
Found in **ch17u_O76014|KRT37_HUMAN** in **uni_human**, Keratin, type I cuticular Ha7 OS=Homo sapiens GN=KRT37 PE=3 SV=3

Match to Query 511: 806.391708 from(404.203130,2+) intensity(533370.1875) rtinseconds(1545) scans(8086) index(5990)
Title: 160219_Sunil_SDSI_A_Spectrum058098_scans__8086_RTINSECONDS=1545
Data file L:\\QE_2016\\160219_Sunil_KAP_LKC\\TMgf\\T\\T160219_Sunil_SDSI_A.mgf

Click mouse within plot area to zoom in by factor of two about that point
Or, Plot from 50 to 700 Da Full range
Label all possible matches ○ Label matches used for scoring ◉
Show Y-axis ☐

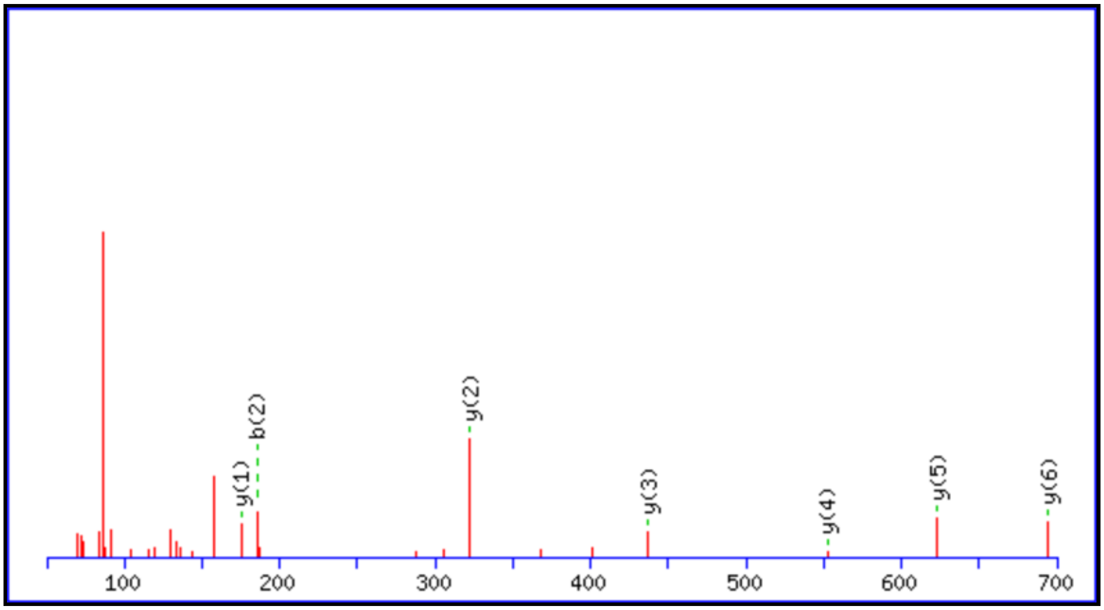

Monoisotopic mass of neutral peptide Mr(calc): 806.3923
Fixed modifications: Carbamidomethyl (C) (apply to specified residues or termini only)
Ions Score: 49 **Expect:** 0.0013
Matches : 7/50 fragment ions using 11 most intense peaks (help)

#	b	b^{++}	b^0	b^{0++}	Seq.	y	y^{++}	y*	y^{*++}	y^0	y^{0++}	#
1	114.0913	57.5493			L							7
2	**185.1285**	93.0679			A	***694.3155***	347.6614	677.2889	339.1481	676.3049	338.6561	6
3	256.1656	128.5864			A	***623.2784***	312.1428	606.2518	303.6295	605.2678	303.1375	5
4	371.1925	186.0999	353.1819	177.0946	D	***552.2413***	276.6243	535.2147	268.1110	534.2307	267.6190	4
5	486.2195	243.6134	468.2089	234.6081	D	***437.2143***	219.1108	420.1878	210.5975	419.2037	210.1055	3
6	633.2879	317.1476	615.2773	308.1423	F	***322.1874***	161.5973	305.1608	153.0840			2

7					R	175.1190	88.0631	158.0924	79.5498			1

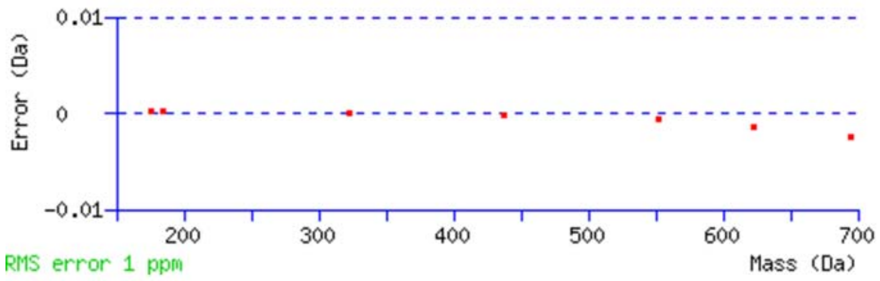

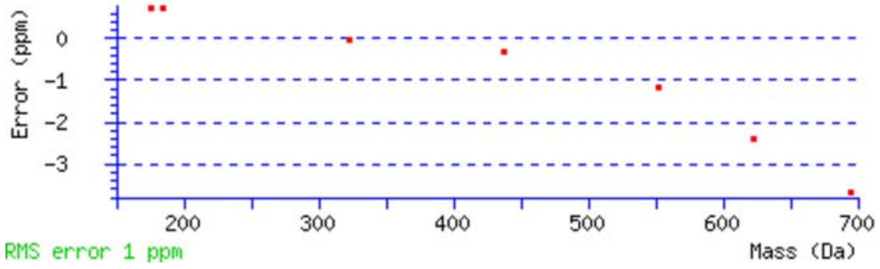

NCBI **BLAST** search of LAADDFR
(Parameters: blastp, nr protein database, expect=20000, no filter, PAM30)
Other BLAST web gateways

All matches to this query

Score	Mr(calc)	Delta	Sequence
49.4	806.3923	-0.0006	LAADDFR
16.5	804.3878	2.0039	RPDNFR
12.9	806.3844	0.0073	LAAQMQK
7.9	806.3923	-0.0006	IAGFDER
7.0	806.3923	-0.0006	LDQQFR
7.0	806.3923	-0.0006	LDQQFR
6.7	806.3922	-0.0005	GELANFR
6.7	804.3878	2.0039	NRPDFR
6.7	806.3923	-0.0006	QLEDFR
4.4	806.3956	-0.0039	MGNSLLR

Mascot: http://www.matrixscience.com/

Mascot Search Results

Peptide View

MS/MS Fragmentation of **LAADDFR**
Found in **ch17u_O76014|KRT37_HUMAN** in **uni_human**, Keratin, type I cuticular Ha7 OS=Homo sapiens GN=KRT37 PE=3 SV=3

Match to Query 512: 806.391708 from(404.203130,2+) intensity(859646.3750) rtinseconds(1513) scans(7896) index(5817)
Title: 160219_Sunil_SDSI_A_Spectrum057925_scans__7896_RTINSECONDS=1513
Data file L:\\QE_2016\\160219_Sunil_KAP_LKC\\TMgf\\T\\T160219_Sunil_SDSI_A.mgf

Click mouse within plot area to zoom in by factor of two about that point
Or, Plot from 50 to 700 Da Full range
Label all possible matches ○ Label matches used for scoring ◉
Show Y-axis ☐

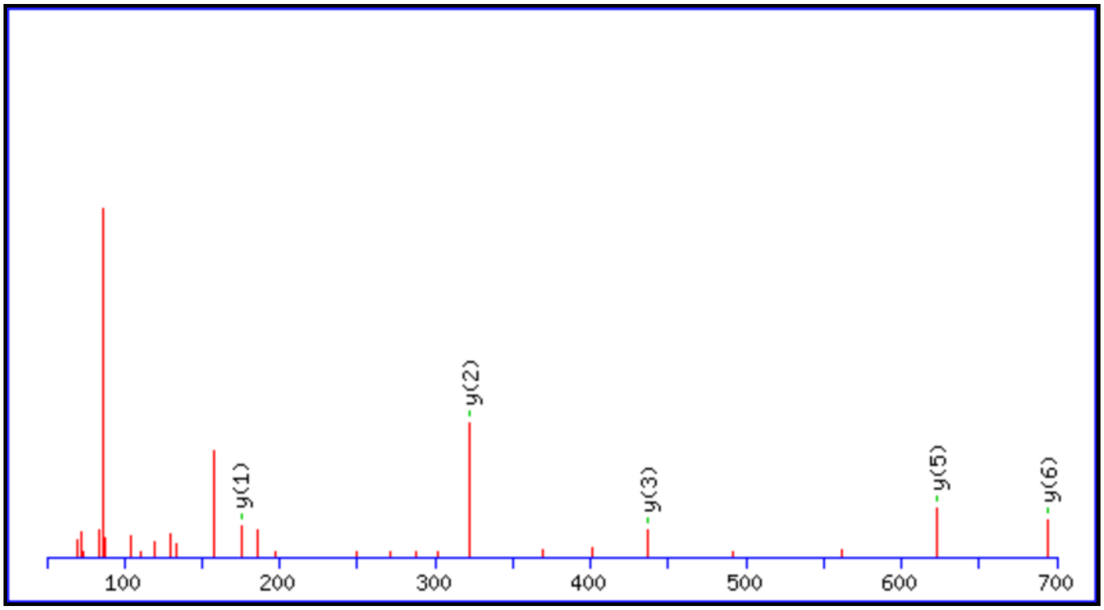

Monoisotopic mass of neutral peptide Mr(calc): 806.3923
Fixed modifications: Carbamidomethyl (C) (apply to specified residues or termini only)
Ions Score: 41 **Expect:** 0.0084
Matches : 5/50 fragment ions using 7 most intense peaks (help)

#	b	b^{++}	b^{0}	b^{0++}	Seq.	y	y^{++}	y*	y^{*++}	y^{0}	y^{0++}	#
1	114.0913	57.5493			L							7
2	185.1285	93.0679			A	***694.3155***	347.6614	677.2889	339.1481	676.3049	338.6561	6
3	256.1656	128.5864			A	***623.2784***	312.1428	606.2518	303.6295	605.2678	303.1375	5
4	371.1925	186.0999	353.1819	177.0946	D	552.2413	276.6243	535.2147	268.1110	534.2307	267.6190	4
5	486.2195	243.6134	468.2089	234.6081	D	***437.2143***	219.1108	420.1878	210.5975	419.2037	210.1055	3
6	633.2879	317.1476	615.2773	308.1423	F	***322.1874***	161.5973	305.1608	153.0840			2

7					R	*175.1190*	88.0631	158.0924	79.5498			1

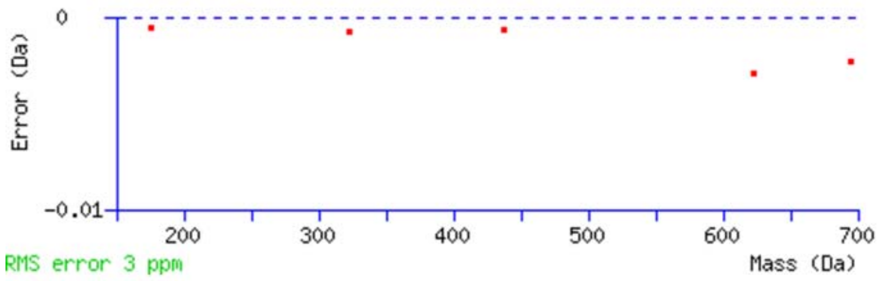

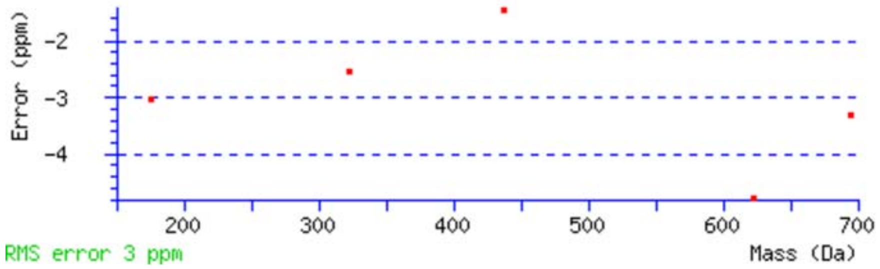

NCBI **BLAST** search of LAADDFR
(Parameters: blastp, nr protein database, expect=20000, no filter, PAM30)
Other BLAST web gateways

All matches to this query

Score	Mr(calc)	Delta	Sequence
41.3	806.3923	-0.0006	LAADDFR
12.0	806.3923	-0.0006	LDQQFR
12.0	806.3923	-0.0006	LDQQFR
11.8	806.3922	-0.0005	GELANFR
11.8	804.3878	2.0039	NRPDFR
11.8	806.3923	-0.0006	QLEDFR
11.8	804.3878	2.0039	RPDNFR
9.6	806.3923	-0.0006	IAGFDER
5.0	806.3956	-0.0039	MGNSLLR

Mascot: http://www.matrixscience.com/

Mascot Search Results

Peptide View

MS/MS Fragmentation of **LAADDFR**
Found in **ch17u_O76014|KRT37_HUMAN** in **uni_human**, Keratin, type I cuticular Ha7 OS=Homo sapiens GN=KRT37 PE=3 SV=3

Match to Query 514: 806.391768 from(404.203160,2+) intensity(701090.4375) rtinseconds(1574) scans(8404) index(21642)
Title: 160219_Sunil_SDSI_A_Spectrum074946_scans__8404_RTINSECONDS=1574
Data file L:\\QE_2016\\160219_Sunil_KAP_LKC\\TMgf\\T\\T160219_Sunil_SDSI_A.mgf

Click mouse within plot area to zoom in by factor of two about that point
Or, Plot from 50 to 700 Da Full range
Label all possible matches ○ Label matches used for scoring ◉
Show Y-axis ☐

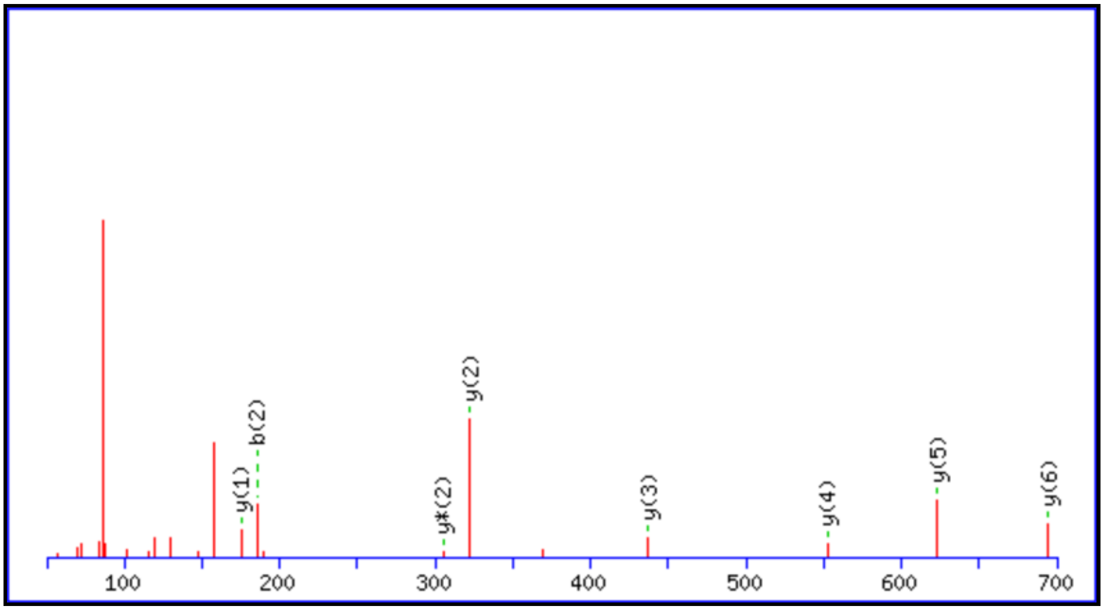

Monoisotopic mass of neutral peptide Mr(calc): 806.3923
Fixed modifications: Carbamidomethyl (C) (apply to specified residues or termini only)
Ions Score: 48 **Expect:** 0.0018
Matches : 8/50 fragment ions using 13 most intense peaks (help)

#	b	b^{++}	b^{0}	b^{0++}	Seq.	y	y^{++}	y*	y^{*++}	y^{0}	y^{0++}	#
1	114.0913	57.5493			L							7
2	**185.1285**	93.0679			A	***694.3155***	347.6614	677.2889	339.1481	676.3049	338.6561	6
3	256.1656	128.5864			A	***623.2784***	312.1428	606.2518	303.6295	605.2678	303.1375	5
4	371.1925	186.0999	353.1819	177.0946	D	***552.2413***	276.6243	535.2147	268.1110	534.2307	267.6190	4
5	486.2195	243.6134	468.2089	234.6081	D	***437.2143***	219.1108	420.1878	210.5975	419.2037	210.1055	3
6	633.2879	317.1476	615.2773	308.1423	F	***322.1874***	161.5973	305.1608	153.0840			2

**7**					**R**	***175.1190***	88.0631	158.0924	79.5498			**1**

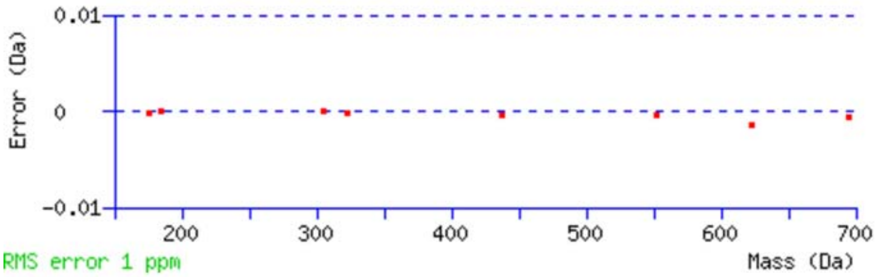

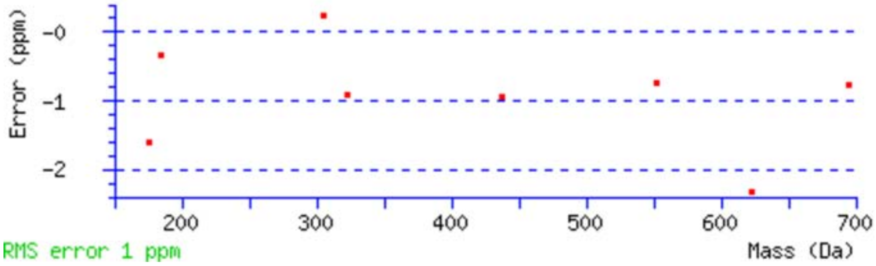

NCBI **BLAST** search of LAADDFR
(Parameters: blastp, nr protein database, expect=20000, no filter, PAM30)
Other BLAST web gateways

All matches to this query

Score	Mr(calc)	Delta	Sequence
47.9	806.3923	-0.0005	LAADDFR
16.6	804.3878	2.0039	RPDNFR
9.4	806.3844	0.0074	LAAQMQK
7.3	806.3923	-0.0005	LDQQFR
7.3	806.3923	-0.0005	LDQQFR
7.0	806.3922	-0.0005	GELANFR
7.0	804.3878	2.0039	NRPDFR
7.0	806.3923	-0.0005	QLEDFR
6.5	806.3923	-0.0005	IAGFDER

Mascot: http://www.matrixscience.com/

Mascot Search Results

Peptide View

MS/MS Fragmentation of **LAADDFR**
Found in **ch17u_O76014|KRT37_HUMAN** in **uni_human**, Keratin, type I cuticular Ha7 OS=Homo sapiens GN=KRT37 PE=3 SV=3

Match to Query 515: 806.391768 from(404.203160,2+) intensity(1510723.0000) rtinseconds(1449) scans(7524) index(5481)
Title: 160219_Sunil_SDSI_A_Spectrum057589_scans__7524_RTINSECONDS=1449
Data file L:\\QE_2016\\160219_Sunil_KAP_LKC\\TMgf\\T\\T160219_Sunil_SDSI_A.mgf

Click mouse within plot area to zoom in by factor of two about that point
Or, Plot from 50 to 700 Da Full range
Label all possible matches ○ Label matches used for scoring ◉
Show Y-axis ☐

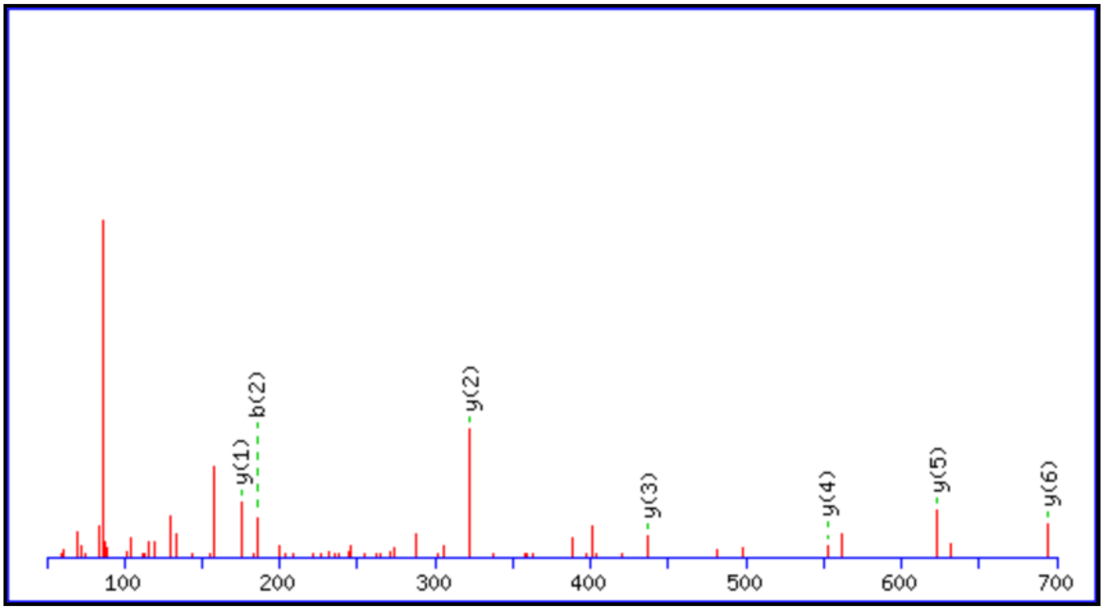

Monoisotopic mass of neutral peptide Mr(calc): 806.3923
Fixed modifications: Carbamidomethyl (C) (apply to specified residues or termini only)
Ions Score: 43 **Expect:** 0.0051
Matches : 7/50 fragment ions using 13 most intense peaks (help)

#	b	b^{++}	b^{0}	b^{0++}	Seq.	y	y^{++}	y^{*}	y^{*++}	y^{0}	y^{0++}	#
1	114.0913	57.5493			L							7
2	**185.1285**	93.0679			A	***694.3155***	347.6614	677.2889	339.1481	676.3049	338.6561	6
3	256.1656	128.5864			A	***623.2784***	312.1428	606.2518	303.6295	605.2678	303.1375	5
4	371.1925	186.0999	353.1819	177.0946	D	***552.2413***	276.6243	535.2147	268.1110	534.2307	267.6190	4
5	486.2195	243.6134	468.2089	234.6081	D	***437.2143***	219.1108	420.1878	210.5975	419.2037	210.1055	3
6	633.2879	317.1476	615.2773	308.1423	F	***322.1874***	161.5973	305.1608	153.0840			2

7					R	*175.1190*	88.0631	158.0924	79.5498			1

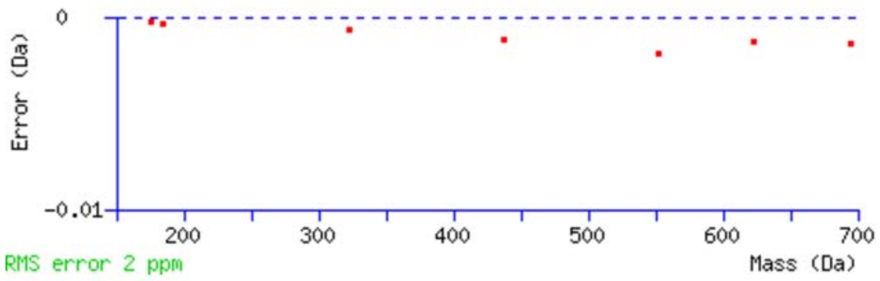

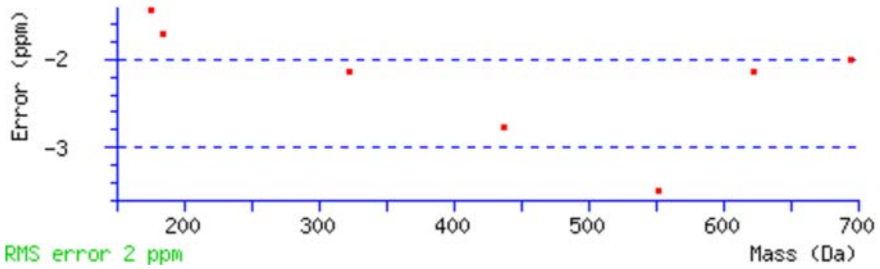

NCBI **BLAST** search of LAADDFR
(Parameters: blastp, nr protein database, expect=20000, no filter, PAM30)
Other BLAST web gateways

All matches to this query

Score	Mr(calc)	Delta	Sequence
43.5	806.3923	-0.0005	LAADDFR
13.4	804.3878	2.0039	RPDNFR
11.4	806.3923	-0.0005	LDQQFR
11.4	806.3923	-0.0005	LDQQFR
9.3	806.3923	-0.0005	IAGFDER
7.6	806.3844	0.0074	LAAQMQK
6.8	806.3956	-0.0038	MGNSLLR
4.5	806.3922	-0.0005	GELANFR
4.5	804.3878	2.0039	NRPDFR
4.5	806.3923	-0.0005	QLEDFR

Mascot: http://www.matrixscience.com/

Mascot Search Results

Peptide View

MS/MS Fragmentation of **LAADDFR**
Found in **ch17u_O76014|KRT37_HUMAN** in **uni_human**, Keratin, type I cuticular Ha7 OS=Homo sapiens GN=KRT37 PE=3 SV=3

Match to Query 521: 806.391948 from(404.203250,2+) intensity(876516.5625) rtinseconds(1506) scans(8012) index(21297)
Title: 160219_Sunil_SDSI_A_Spectrum074601_scans__8012_RTINSECONDS=1506
Data file L:\\QE_2016\\160219_Sunil_KAP_LKC\\TMgf\\T\\T160219_Sunil_SDSI_A.mgf

Click mouse within plot area to zoom in by factor of two about that point
Or, Plot from 50 to 700 Da Full range
Label all possible matches ○ Label matches used for scoring ◉
Show Y-axis ☐

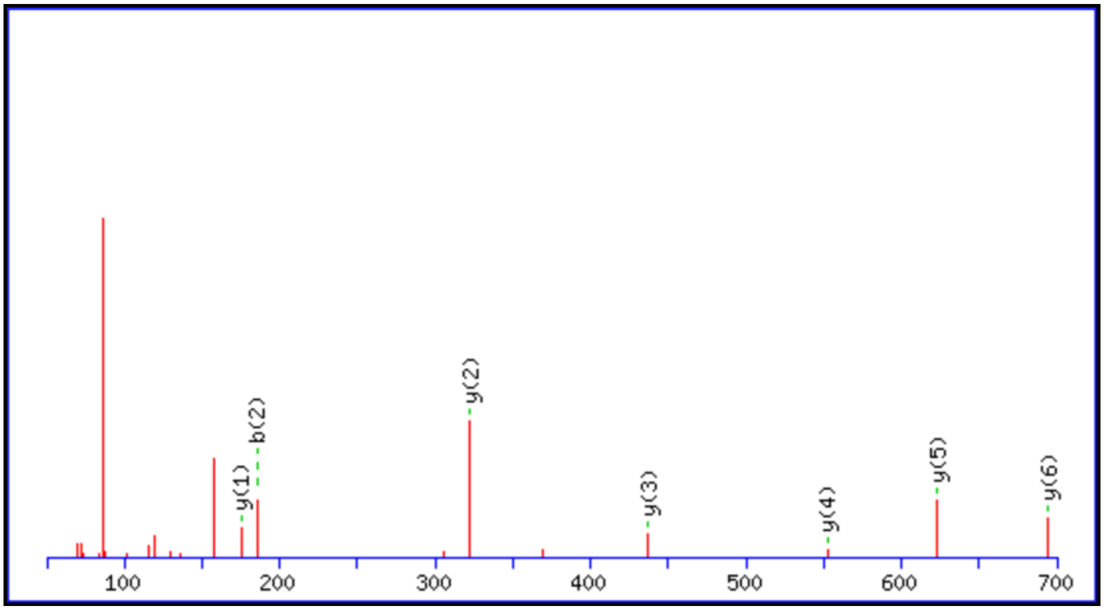

Monoisotopic mass of neutral peptide Mr(calc): 806.3923
Fixed modifications: Carbamidomethyl (C) (apply to specified residues or termini only)
Ions Score: 53 **Expect:** 0.00054
Matches : 7/50 fragment ions using 10 most intense peaks (help)

#	b	b^{++}	b^0	b^{0++}	Seq.	y	y^{++}	y^*	y^{*++}	y^0	y^{0++}	#
1	114.0913	57.5493			L							7
2	**185.1285**	93.0679			A	***694.3155***	347.6614	677.2889	339.1481	676.3049	338.6561	6
3	256.1656	128.5864			A	***623.2784***	312.1428	606.2518	303.6295	605.2678	303.1375	5
4	371.1925	186.0999	353.1819	177.0946	D	***552.2413***	276.6243	535.2147	268.1110	534.2307	267.6190	4
5	486.2195	243.6134	468.2089	234.6081	D	***437.2143***	219.1108	420.1878	210.5975	419.2037	210.1055	3
6	633.2879	317.1476	615.2773	308.1423	F	***322.1874***	161.5973	305.1608	153.0840			2

7					R	175.1190	88.0631	158.0924	79.5498			1

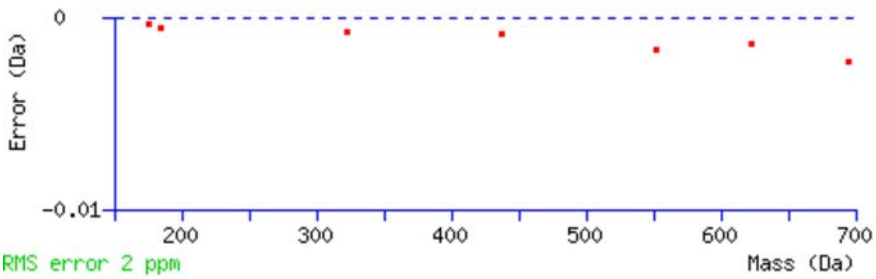

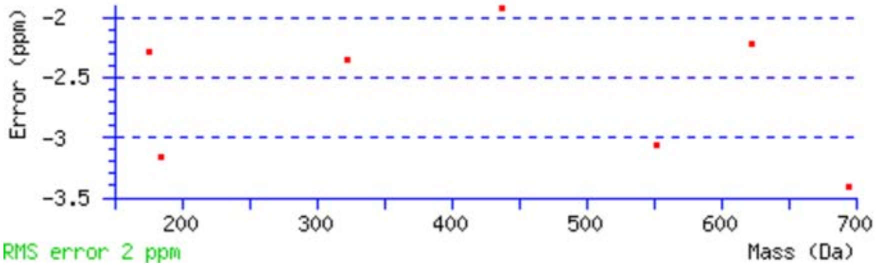

NCBI **BLAST** search of LAADDFR
(Parameters: blastp, nr protein database, expect=20000, no filter, PAM30)
Other BLAST web gateways

All matches to this query

Score	Mr(calc)	Delta	Sequence
53.2	806.3923	-0.0003	LAADDFR
18.6	804.3878	2.0041	RPDNFR
14.0	806.3844	0.0076	LAAQMQK
10.0	806.3923	-0.0003	IAGFDER
8.5	806.3923	-0.0003	LDQQFR
8.5	806.3923	-0.0003	LDQQFR
8.2	806.3922	-0.0003	GELANFR
8.2	804.3878	2.0041	NRPDFR
8.2	806.3923	-0.0003	QLEDFR
3.6	806.3891	0.0028	IACCRK

Mascot: http://www.matrixscience.com/

Mascot Search Results

Peptide View

MS/MS Fragmentation of **LAADDFR**
Found in **ch17u_O76014|KRT37_HUMAN** in **uni_human**, Keratin, type I cuticular Ha7 OS=Homo sapiens GN=KRT37 PE=3 SV=3

Match to Query 522: 806.391948 from(404.203250,2+) intensity(1817783.0000) rtinseconds(1409) scans(7444) index(20785)
Title: 160219_Sunil_SDSI_A_Spectrum074089_scans__7444_RTINSECONDS=1409
Data file L:\\QE_2016\\160219_Sunil_KAP_LKC\\TMgf\\T\\T160219_Sunil_SDSI_A.mgf

Click mouse within plot area to zoom in by factor of two about that point
Or, Plot from 50 to 700 Da Full range
Label all possible matches ○ Label matches used for scoring ◉
Show Y-axis ☐

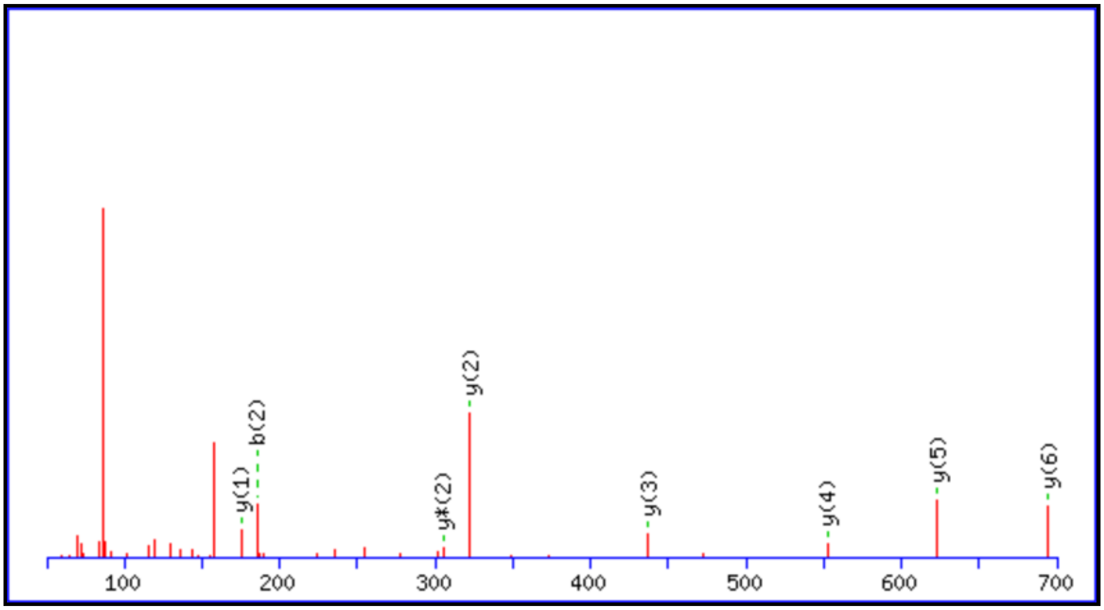

Monoisotopic mass of neutral peptide Mr(calc): 806.3923
Fixed modifications: Carbamidomethyl (C) (apply to specified residues or termini only)
Ions Score: 51 **Expect:** 0.001
Matches : 8/50 fragment ions using 12 most intense peaks (help)

#	b	b^{++}	b^{0}	b^{0++}	Seq.	y	y^{++}	y*	y^{*++}	y^{0}	y^{0++}	#
1	114.0913	57.5493			L							7
2	**185.1285**	93.0679			A	***694.3155***	347.6614	677.2889	339.1481	676.3049	338.6561	6
3	256.1656	128.5864			A	***623.2784***	312.1428	606.2518	303.6295	605.2678	303.1375	5
4	371.1925	186.0999	353.1819	177.0946	D	***552.2413***	276.6243	535.2147	268.1110	534.2307	267.6190	4
5	486.2195	243.6134	468.2089	234.6081	D	***437.2143***	219.1108	420.1878	210.5975	419.2037	210.1055	3
6	633.2879	317.1476	615.2773	308.1423	F	***322.1874***	161.5973	305.1608	153.0840			2

7					R	*175.1190*	88.0631	158.0924	79.5498			1

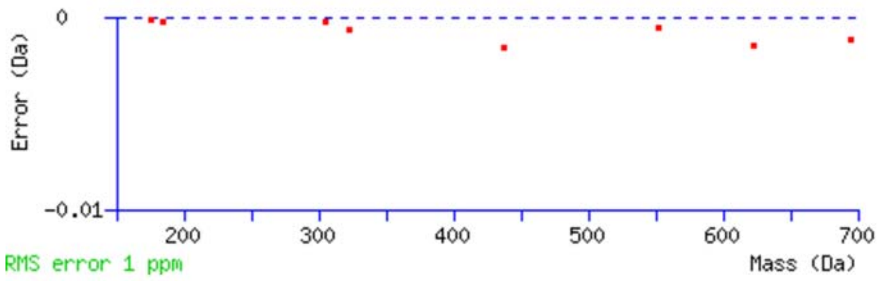

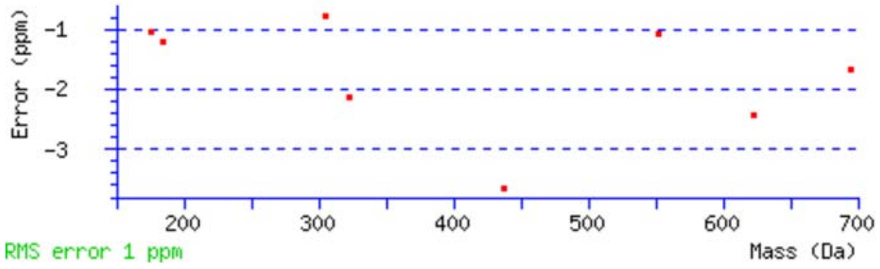

NCBI **BLAST** search of LAADDFR
(Parameters: blastp, nr protein database, expect=20000, no filter, PAM30)
Other BLAST web gateways

All matches to this query

Score	Mr(calc)	Delta	Sequence
50.5	806.3923	-0.0003	LAADDFR
17.9	804.3878	2.0041	RPDNFR
13.9	806.3844	0.0076	LAAQMQK
8.4	806.3923	-0.0003	LDQQFR
8.4	806.3923	-0.0003	LDQQFR
7.8	806.3922	-0.0003	GELANFR
7.8	804.3878	2.0041	NRPDFR
7.8	806.3923	-0.0003	QLEDFR
7.5	806.3923	-0.0003	IAGFDER
3.4	806.3891	0.0028	IACCRK

Mascot: http://www.matrixscience.com/

Mascot Search Results

Peptide View

MS/MS Fragmentation of **LAADDFR**
Found in **ch17u_O76014|KRT37_HUMAN** in **uni_human**, Keratin, type I cuticular Ha7 OS=Homo sapiens GN=KRT37 PE=3 SV=3

Match to Query 524: 806.392008 from(404.203280,2+) intensity(3733101.2500) rtinseconds(1312) scans(6874) index(20276)
Title: 160219_Sunil_SDSI_A_Spectrum073580_scans__6874_RTINSECONDS=1312
Data file L:\\QE_2016\\160219_Sunil_KAP_LKC\\TMgf\\T\\T160219_Sunil_SDSI_A.mgf

Click mouse within plot area to zoom in by factor of two about that point
Or, Plot from 50 to 750 Da Full range
Label all possible matches ○ Label matches used for scoring ◉
Show Y-axis ☐

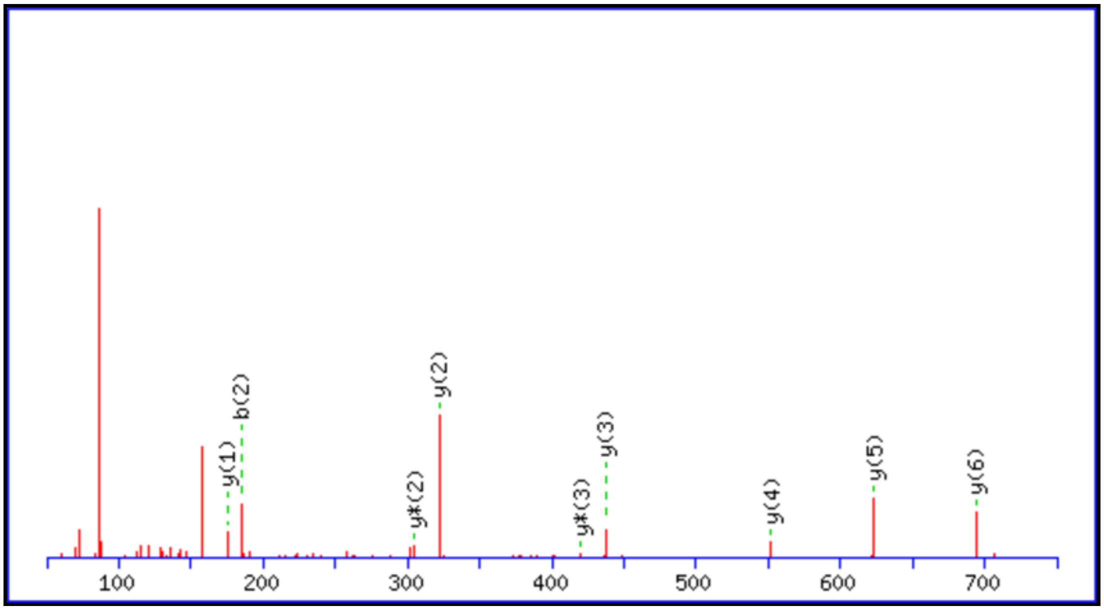

Monoisotopic mass of neutral peptide Mr(calc): 806.3923
Fixed modifications: Carbamidomethyl (C) (apply to specified residues or termini only)
Ions Score: 48 **Expect:** 0.0016
Matches : 9/50 fragment ions using 14 most intense peaks (help)

#	b	b^{++}	b^0	b^{0++}	Seq.	y	y^{++}	y*	y^{*++}	y^0	y^{0++}	#
1	114.0913	57.5493			L							7
2	**185.1285**	93.0679			A	***694.3155***	347.6614	677.2889	339.1481	676.3049	338.6561	6
3	256.1656	128.5864			A	***623.2784***	312.1428	606.2518	303.6295	605.2678	303.1375	5
4	371.1925	186.0999	353.1819	177.0946	D	***552.2413***	276.6243	535.2147	268.1110	534.2307	267.6190	4
5	486.2195	243.6134	468.2089	234.6081	D	***437.2143***	219.1108	420.1878	210.5975	419.2037	210.1055	3
6	633.2879	317.1476	615.2773	308.1423	F	***322.1874***	161.5973	305.1608	153.0840			2

7					R	*175.1190*	88.0631	158.0924	79.5498			1

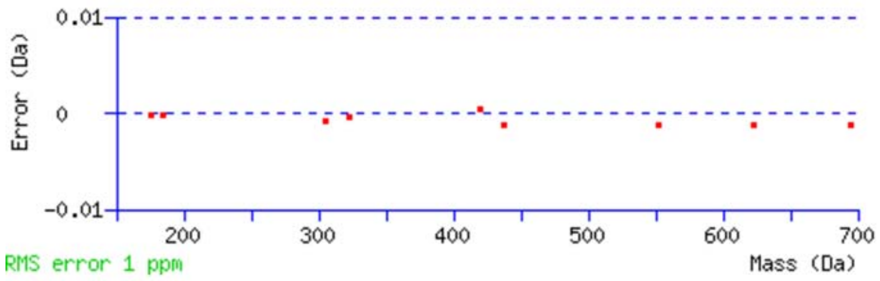

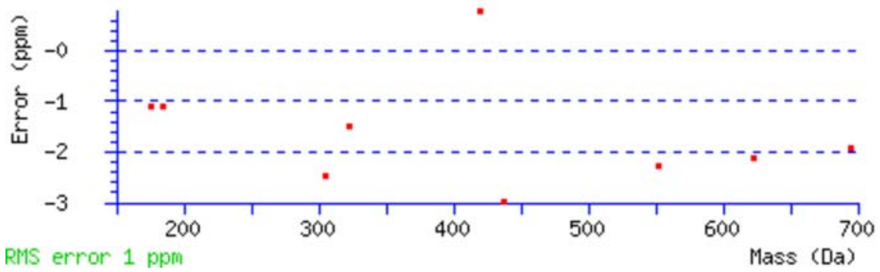

NCBI **BLAST** search of LAADDFR
(Parameters: blastp, nr protein database, expect=20000, no filter, PAM30)
Other BLAST web gateways

All matches to this query

Score	Mr(calc)	Delta	Sequence
48.5	806.3923	-0.0003	LAADDFR
17.5	804.3878	2.0042	RPDNFR
13.8	806.3844	0.0076	LAAQMQK
7.7	806.3922	-0.0002	GELANFR
7.7	804.3878	2.0042	NRPDFR
7.7	806.3923	-0.0003	QLEDFR
6.5	806.3923	-0.0003	LDQQFR
6.5	806.3923	-0.0003	LDQQFR
5.6	806.3923	-0.0003	IAGFDER
3.2	806.3891	0.0029	IACCRK

Mascot: http://www.matrixscience.com/

Mascot Search Results

Peptide View

MS/MS Fragmentation of **LAADDFR**
Found in **ch17u_O76014|KRT37_HUMAN** in **uni_human**, Keratin, type I cuticular Ha7 OS=Homo sapiens GN=KRT37 PE=3 SV=3

Match to Query 526: 806.392068 from(404.203310,2+) intensity(2834633.5000) rtinseconds(1344) scans(7061) index(20443)
Title: 160219_Sunil_SDSI_A_Spectrum073747_scans__7061_RTINSECONDS=1344
Data file L:\\QE_2016\\160219_Sunil_KAP_LKC\\TMgf\\T\\T160219_Sunil_SDSI_A.mgf

Click mouse within plot area to zoom in by factor of two about that point
Or, Plot from 50 to 750 Da Full range
Label all possible matches ○ Label matches used for scoring ◉
Show Y-axis ☐

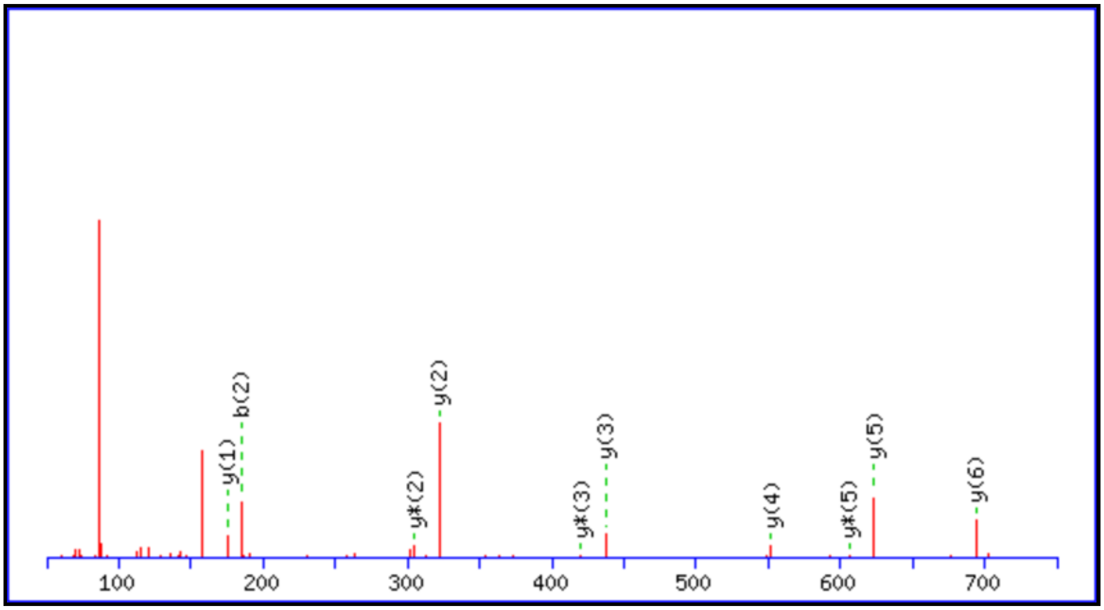

Monoisotopic mass of neutral peptide Mr(calc): 806.3923
Fixed modifications: Carbamidomethyl (C) (apply to specified residues or termini only)
Ions Score: 52 **Expect:** 0.00077
Matches : 10/50 fragment ions using 14 most intense peaks (help)

#	b	b^{++}	b^0	b^{0++}	Seq.	y	y^{++}	y*	y^{*++}	y^0	y^{0++}	#
1	114.0913	57.5493			L							7
2	**185.1285**	93.0679			A	***694.3155***	347.6614	677.2889	339.1481	676.3049	338.6561	6
3	256.1656	128.5864			A	***623.2784***	312.1428	606.2518	303.6295	605.2678	303.1375	5
4	371.1925	186.0999	353.1819	177.0946	D	***552.2413***	276.6243	535.2147	268.1110	534.2307	267.6190	4
5	486.2195	243.6134	468.2089	234.6081	D	***437.2143***	219.1108	420.1878	210.5975	419.2037	210.1055	3
6	633.2879	317.1476	615.2773	308.1423	F	***322.1874***	161.5973	305.1608	153.0840			2

7					R	*175.1190*	88.0631	158.0924	79.5498			1

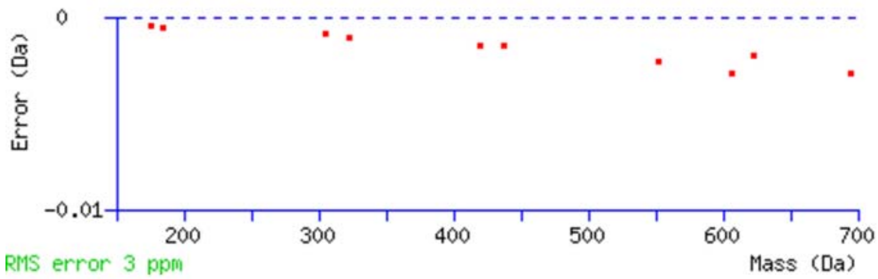

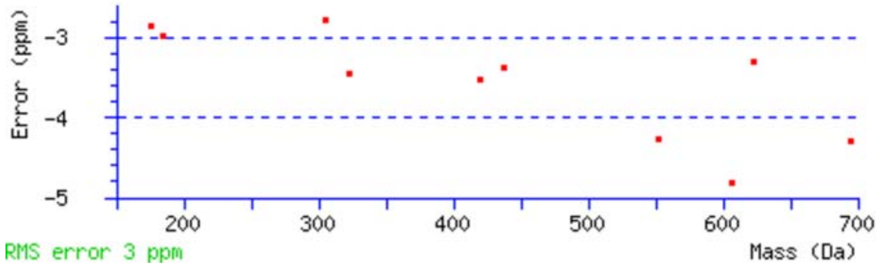

NCBI **BLAST** search of LAADDFR
(Parameters: blastp, nr protein database, expect=20000, no filter, PAM30)
Other BLAST web gateways

All matches to this query

Score	Mr(calc)	Delta	Sequence
51.6	806.3923	-0.0002	LAADDFR
17.6	804.3878	2.0042	RPDNFR
14.1	806.3844	0.0077	LAAQMQK
7.9	806.3922	-0.0002	GELANFR
7.9	804.3878	2.0042	NRPDFR
7.9	806.3923	-0.0002	QLEDFR
7.4	806.3923	-0.0002	IAGFDER
6.7	806.3923	-0.0002	LDQQFR
6.7	806.3923	-0.0002	LDQQFR
5.8	806.3923	-0.0002	QQQVFR

Mascot: http://www.matrixscience.com/

Mascot Search Results

Peptide View

MS/MS Fragmentation of **LAADDFR**
Found in **ch17u_O76014|KRT37_HUMAN** in **uni_human**, Keratin, type I cuticular Ha7 OS=Homo sapiens GN=KRT37 PE=3 SV=3

Match to Query 526: 806.392068 from(404.203310,2+) intensity(2834633.5000) rtinseconds(1344) scans(7061) index(20443)
Title: 160219_Sunil_SDSI_A_Spectrum073747_scans__7061_RTINSECONDS=1344
Data file L:\\QE_2016\\160219_Sunil_KAP_LKC\\TMgf\\T\\T160219_Sunil_SDSI_A.mgf

Click mouse within plot area to zoom in by factor of two about that point
Or, Plot from 50 to 750 Da Full range
Label all possible matches ○ Label matches used for scoring ◉
Show Y-axis ☐

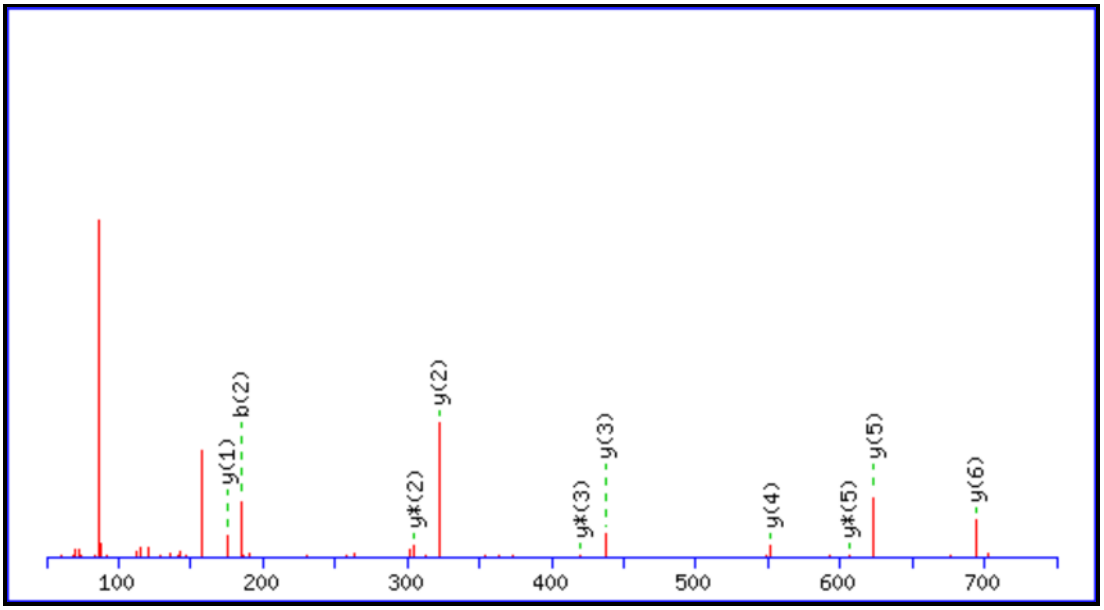

Monoisotopic mass of neutral peptide Mr(calc): 806.3923
Fixed modifications: Carbamidomethyl (C) (apply to specified residues or termini only)
Ions Score: 52 **Expect:** 0.00077
Matches : 10/50 fragment ions using 14 most intense peaks (help)

#	b	b^{++}	b^{0}	b^{0++}	Seq.	y	y^{++}	y*	y^{*++}	y^{0}	y^{0++}	#
1	114.0913	57.5493			L							7
2	**185.1285**	93.0679			A	***694.3155***	347.6614	677.2889	339.1481	676.3049	338.6561	6
3	256.1656	128.5864			A	***623.2784***	312.1428	606.2518	303.6295	605.2678	303.1375	5
4	371.1925	186.0999	353.1819	177.0946	D	***552.2413***	276.6243	535.2147	268.1110	534.2307	267.6190	4
5	486.2195	243.6134	468.2089	234.6081	D	***437.2143***	219.1108	420.1878	210.5975	419.2037	210.1055	3
6	633.2879	317.1476	615.2773	308.1423	F	***322.1874***	161.5973	305.1608	153.0840			2

7					R	*175.1190*	88.0631	158.0924	79.5498			1

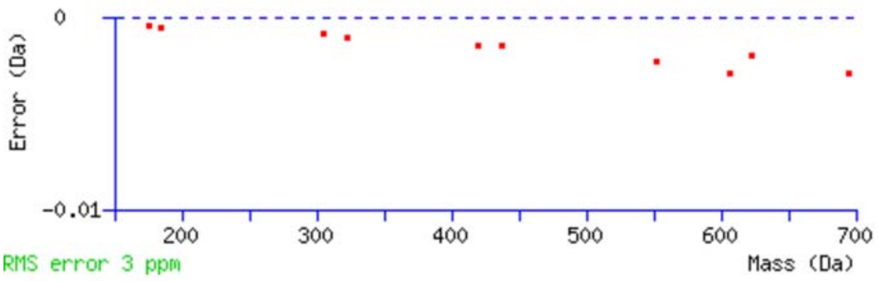

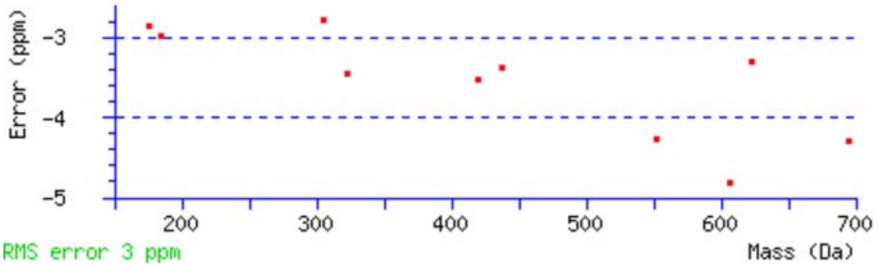

NCBI **BLAST** search of LAADDFR
(Parameters: blastp, nr protein database, expect=20000, no filter, PAM30)
Other BLAST web gateways

All matches to this query

Score	Mr(calc)	Delta	Sequence
51.6	806.3923	-0.0002	LAADDFR
17.6	804.3878	2.0042	RPDNFR
14.1	806.3844	0.0077	LAAQMQK
7.9	806.3922	-0.0002	GELANFR
7.9	804.3878	2.0042	NRPDFR
7.9	806.3923	-0.0002	QLEDFR
7.4	806.3923	-0.0002	IAGFDER
6.7	806.3923	-0.0002	LDQQFR
6.7	806.3923	-0.0002	LDQQFR
5.8	806.3923	-0.0002	QQQVFR

Mascot: http://www.matrixscience.com/

Mascot Search Results

Peptide View

MS/MS Fragmentation of **LAADDFR**
Found in **ch17u_O76014|KRT37_HUMAN** in **uni_human**, Keratin, type I cuticular Ha7 OS=Homo sapiens GN=KRT37 PE=3 SV=3

Match to Query 527: 806.392068 from(404.203310,2+) intensity(3727980.5000) rtinseconds(1384) scans(7144) index(5166)
Title: 160219_Sunil_SDSI_A_Spectrum057274_scans__7144_RTINSECONDS=1384
Data file L:\\QE_2016\\160219_Sunil_KAP_LKC\\TMgf\\T\\T160219_Sunil_SDSI_A.mgf

Click mouse within plot area to zoom in by factor of two about that point
Or, Plot from 50 to 700 Da Full range
Label all possible matches ○ Label matches used for scoring ◉
Show Y-axis ☐

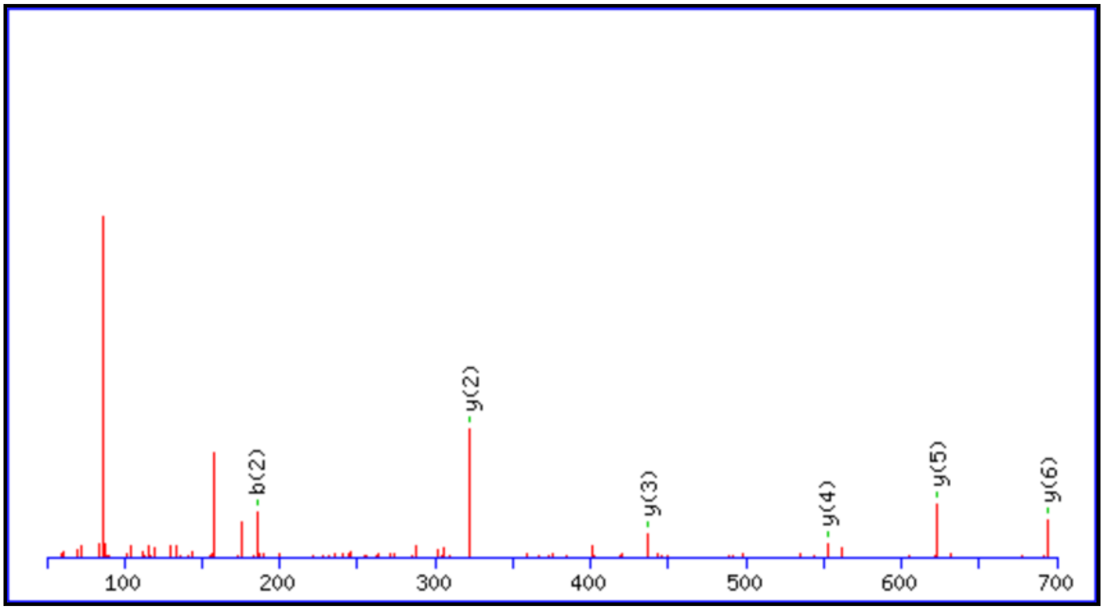

Monoisotopic mass of neutral peptide Mr(calc): 806.3923
Fixed modifications: Carbamidomethyl (C) (apply to specified residues or termini only)
Ions Score: 46 **Expect:** 0.0028
Matches : 6/50 fragment ions using 7 most intense peaks (help)

#	b	b^{++}	b^0	b^{0++}	Seq.	y	y^{++}	y^*	y^{*++}	y^0	y^{0++}	#
1	114.0913	57.5493			L							7
2	**185.1285**	93.0679			A	***694.3155***	347.6614	677.2889	339.1481	676.3049	338.6561	6
3	256.1656	128.5864			A	***623.2784***	312.1428	606.2518	303.6295	605.2678	303.1375	5
4	371.1925	186.0999	353.1819	177.0946	D	***552.2413***	276.6243	535.2147	268.1110	534.2307	267.6190	4
5	486.2195	243.6134	468.2089	234.6081	D	***437.2143***	219.1108	420.1878	210.5975	419.2037	210.1055	3
6	633.2879	317.1476	615.2773	308.1423	F	***322.1874***	161.5973	305.1608	153.0840			2

7					R	175.1190	88.0631	158.0924	79.5498			1

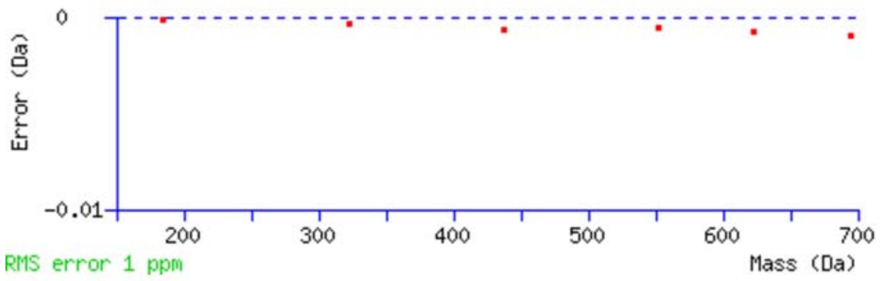

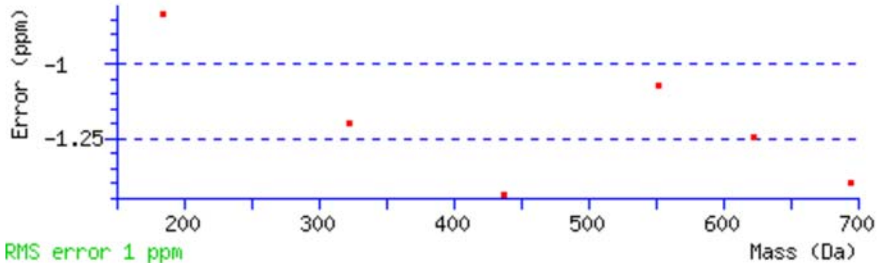

NCBI **BLAST** search of LAADDFR
(Parameters: blastp, nr protein database, expect=20000, no filter, PAM30)
Other BLAST web gateways

All matches to this query

Score	Mr(calc)	Delta	Sequence
46.1	806.3923	-0.0002	LAADDFR
13.2	806.3844	0.0077	LAAQMQK
12.8	804.3878	2.0042	RPDNFR
5.8	806.3956	-0.0035	MGNSLLR
5.4	806.3923	-0.0002	IAGFDER
4.7	806.3923	-0.0002	LDQQFR
4.7	806.3923	-0.0002	LDQQFR
4.4	806.3922	-0.0002	GELANFR
4.4	804.3878	2.0042	NRPDFR
4.4	806.3923	-0.0002	QLEDFR

Mascot: http://www.matrixscience.com/

Mascot Search Results

Peptide View

MS/MS Fragmentation of **LAADDFR**
Found in **ch17u_O76014|KRT37_HUMAN** in **uni_human**, Keratin, type I cuticular Ha7 OS=Homo sapiens GN=KRT37 PE=3 SV=3

Match to Query 528: 806.392068 from(404.203310,2+) intensity(5943353.5000) rtinseconds(1290) scans(6589) index(4690)
Title: 160219_Sunil_SDSI_A_Spectrum056798_scans__6589_RTINSECONDS=1290
Data file L:\\QE_2016\\160219_Sunil_KAP_LKC\\TMgf\\T\\T160219_Sunil_SDSI_A.mgf

Click mouse within plot area to zoom in by factor of two about that point
Or, Plot from 50 to 700 Da Full range
Label all possible matches ○ Label matches used for scoring ◉
Show Y-axis ☐

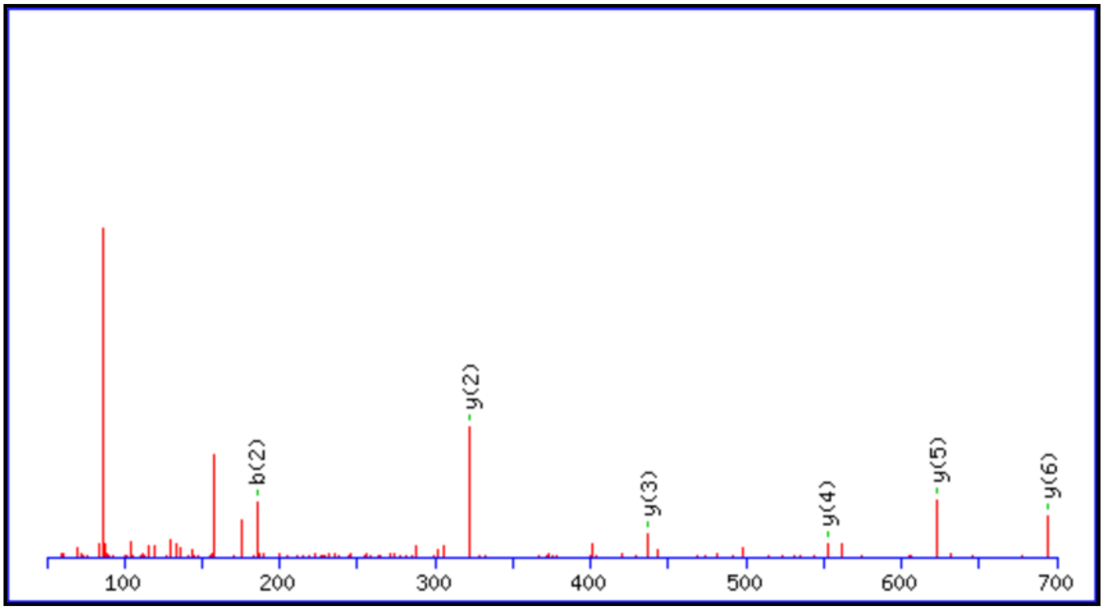

Monoisotopic mass of neutral peptide Mr(calc): 806.3923
Fixed modifications: Carbamidomethyl (C) (apply to specified residues or termini only)
Ions Score: 46 **Expect:** 0.0027
Matches : 6/50 fragment ions using 7 most intense peaks (help)

#	b	b^{++}	b^{0}	b^{0++}	Seq.	y	y^{++}	y*	y^{*++}	y^{0}	y^{0++}	#
1	114.0913	57.5493			L							7
2	**185.1285**	93.0679			A	***694.3155***	347.6614	677.2889	339.1481	676.3049	338.6561	6
3	256.1656	128.5864			A	***623.2784***	312.1428	606.2518	303.6295	605.2678	303.1375	5
4	371.1925	186.0999	353.1819	177.0946	D	***552.2413***	276.6243	535.2147	268.1110	534.2307	267.6190	4
5	486.2195	243.6134	468.2089	234.6081	D	***437.2143***	219.1108	420.1878	210.5975	419.2037	210.1055	3
6	633.2879	317.1476	615.2773	308.1423	F	***322.1874***	161.5973	305.1608	153.0840			2

**7**					**R**	175.1190	88.0631	158.0924	79.5498			**1**

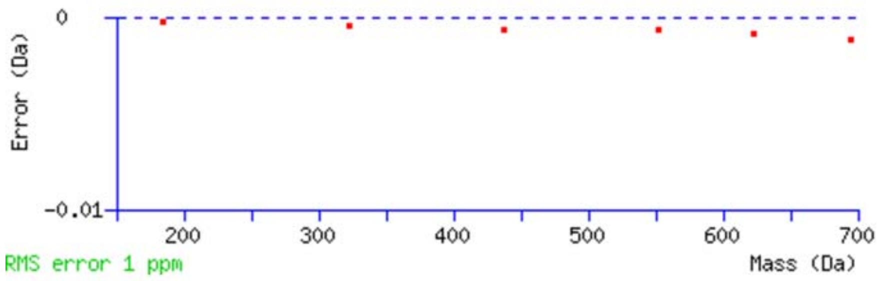

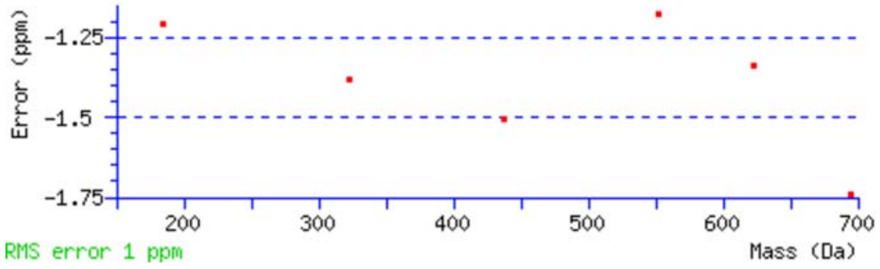

NCBI **BLAST** search of LAADDFR
(Parameters: blastp, nr protein database, expect=20000, no filter, PAM30)
Other BLAST web gateways

All matches to this query

Score	Mr(calc)	Delta	Sequence
46.2	806.3923	-0.0002	LAADDFR
13.6	806.3844	0.0077	LAAQMQK
12.7	804.3878	2.0042	RPDNFR
7.2	806.3923	-0.0002	IAGFDER
6.2	806.3923	-0.0002	LDQQFR
6.2	806.3923	-0.0002	LDQQFR
5.8	806.3956	-0.0035	MGNSLLR
4.3	806.3922	-0.0002	GELANFR
4.3	804.3878	2.0042	NRPDFR
4.3	806.3923	-0.0002	QLEDFR

Mascot: http://www.matrixscience.com/

Mascot Search Results

Peptide View

MS/MS Fragmentation of **LAADDFR**
Found in **ch17u_O76014|KRT37_HUMAN** in **uni_human**, Keratin, type I cuticular Ha7 OS=Homo sapiens GN=KRT37 PE=3 SV=3

Match to Query 530: 806.392128 from(404.203340,2+) intensity(9116559.0000) rtinseconds(1248) scans(6509) index(19969)
Title: 160219_Sunil_SDSI_A_Spectrum073273_scans__6509_RTINSECONDS=1248
Data file L:\\QE_2016\\160219_Sunil_KAP_LKC\\TMgf\\T\\T160219_Sunil_SDSI_A.mgf

Click mouse within plot area to zoom in by factor of two about that point
Or, Plot from 50 to 700 Da Full range
Label all possible matches ○ Label matches used for scoring ◉
Show Y-axis ☐

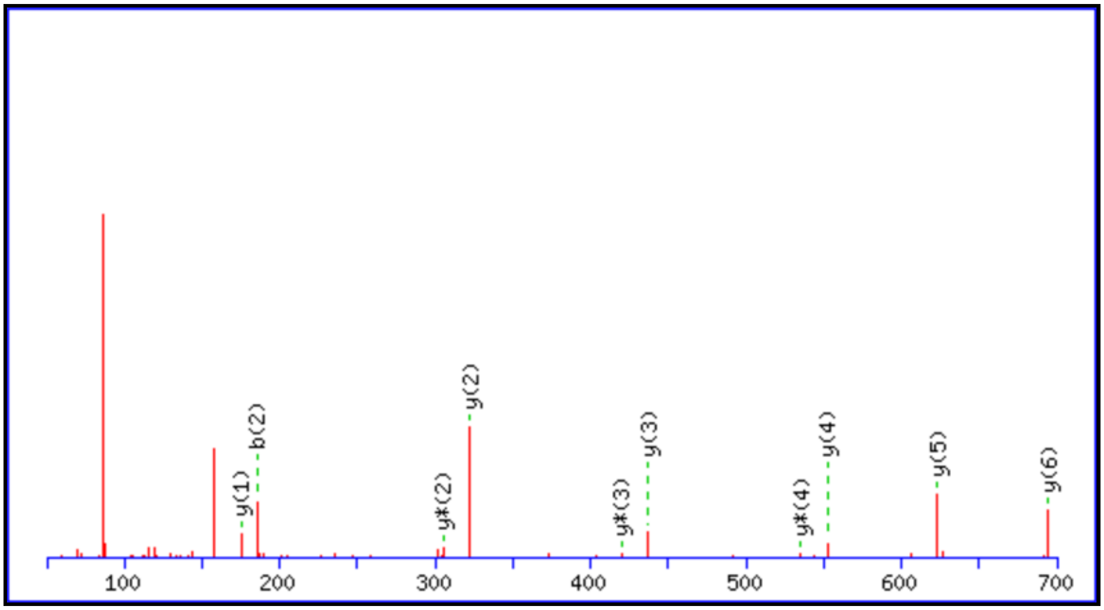

Monoisotopic mass of neutral peptide Mr(calc): 806.3923
Fixed modifications: Carbamidomethyl (C) (apply to specified residues or termini only)
Ions Score: 52 **Expect:** 0.00077
Matches : 10/50 fragment ions using 14 most intense peaks (help)

#	b	b^{++}	b^0	b^{0++}	Seq.	y	y^{++}	y*	y^{*++}	y^0	y^{0++}	#
1	114.0913	57.5493			L							7
2	**185.1285**	93.0679			A	***694.3155***	347.6614	677.2889	339.1481	676.3049	338.6561	6
3	256.1656	128.5864			A	***623.2784***	312.1428	606.2518	303.6295	605.2678	303.1375	5
4	371.1925	186.0999	353.1819	177.0946	D	***552.2413***	276.6243	535.2147	268.1110	534.2307	267.6190	4
5	486.2195	243.6134	468.2089	234.6081	D	***437.2143***	219.1108	420.1878	210.5975	419.2037	210.1055	3
6	633.2879	317.1476	615.2773	308.1423	F	***322.1874***	161.5973	305.1608	153.0840			2

7					R	*175.1190*	88.0631	158.0924	79.5498			1

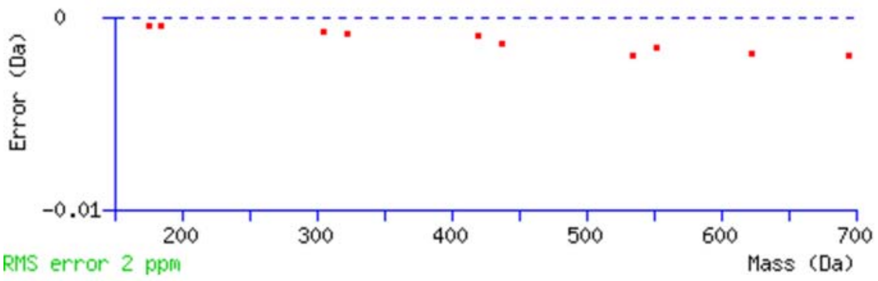

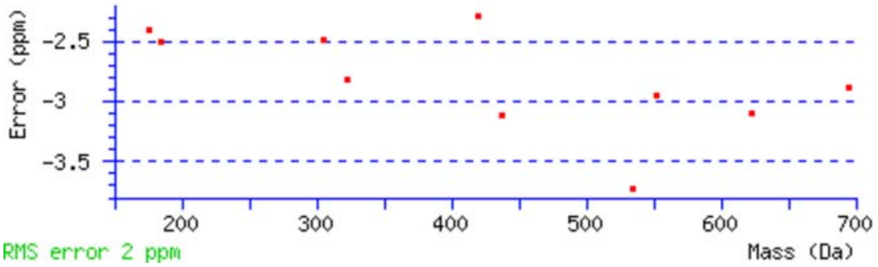

NCBI **BLAST** search of LAADDFR
(Parameters: blastp, nr protein database, expect=20000, no filter, PAM30)
Other BLAST web gateways

All matches to this query

Score	Mr(calc)	Delta	Sequence
51.7	806.3923	-0.0001	LAADDFR
19.4	804.3878	2.0043	RPDNFR
14.4	806.3844	0.0078	LAAQMQK
8.1	806.3923	-0.0001	IAGFDER
7.7	806.3922	-0.0001	GELANFR
7.7	804.3878	2.0043	NRPDFR
7.7	806.3923	-0.0001	QLEDFR
6.7	806.3923	-0.0001	LDQQFR
6.7	806.3923	-0.0001	LDQQFR
3.9	806.3891	0.0030	IACCRK

Mascot: http://www.matrixscience.com/

Mascot Search Results

Peptide View

MS/MS Fragmentation of **LAADDFR**
Found in **ch17u_O76014|KRT37_HUMAN** in **uni_human**, Keratin, type I cuticular Ha7 OS=Homo sapiens GN=KRT37 PE=3 SV=3

Match to Query 532: 806.392128 from(404.203340,2+) intensity(12699940.0000) rtinseconds(1178) scans(5987) index(34767)
Title: 160219_Sunil_SDSI_A_Spectrum089352_scans__5987_RTINSECONDS=1178
Data file L:\\QE_2016\\160219_Sunil_KAP_LKC\\TMgf\\T\\T160219_Sunil_SDSI_A.mgf

Click mouse within plot area to zoom in by factor of two about that point
Or, Plot from 50 to 700 Da Full range
Label all possible matches ○ Label matches used for scoring ◉
Show Y-axis ☐

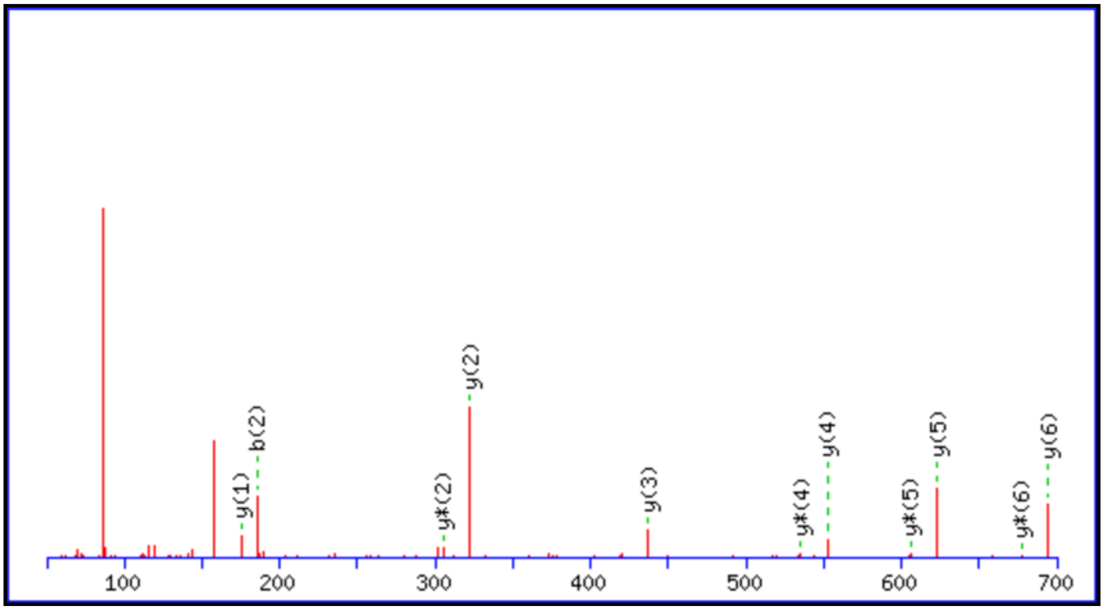

Monoisotopic mass of neutral peptide Mr(calc): 806.3923
Fixed modifications: Carbamidomethyl (C) (apply to specified residues or termini only)
Ions Score: 55 **Expect:** 0.00036
Matches : 11/50 fragment ions using 14 most intense peaks (help)

#	b	b^{++}	b^0	b^{0++}	Seq.	y	y^{++}	y*	y^{*++}	y^0	y^{0++}	#
1	114.0913	57.5493			L							7
2	**185.1285**	93.0679			A	***694.3155***	347.6614	677.2889	339.1481	676.3049	338.6561	6
3	256.1656	128.5864			A	***623.2784***	312.1428	606.2518	303.6295	605.2678	303.1375	5
4	371.1925	186.0999	353.1819	177.0946	D	***552.2413***	276.6243	535.2147	268.1110	534.2307	267.6190	4
5	486.2195	243.6134	468.2089	234.6081	D	***437.2143***	219.1108	420.1878	210.5975	419.2037	210.1055	3
6	633.2879	317.1476	615.2773	308.1423	F	***322.1874***	161.5973	305.1608	153.0840			2

7					R	*175.1190*	88.0631	158.0924	79.5498			1

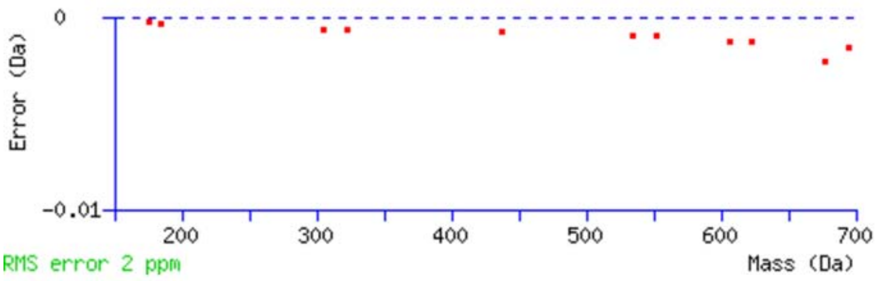

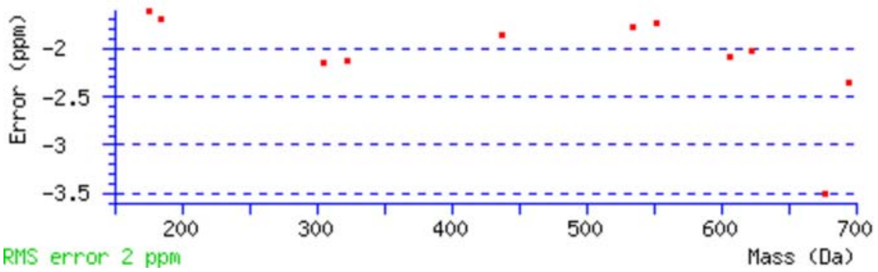

NCBI **BLAST** search of LAADDFR
(Parameters: blastp, nr protein database, expect=20000, no filter, PAM30)
Other BLAST web gateways

All matches to this query

Score	Mr(calc)	Delta	Sequence
54.9	806.3923	-0.0001	LAADDFR
17.6	804.3878	2.0043	RPDNFR
14.5	806.3844	0.0078	LAAQMQK
9.2	806.3923	-0.0001	IAGFDER
8.3	806.3923	-0.0001	LDQQFR
8.3	806.3923	-0.0001	LDQQFR
6.3	806.3922	-0.0001	GELANFR
6.3	804.3878	2.0043	NRPDFR
6.3	806.3923	-0.0001	QLEDFR
4.0	806.3891	0.0030	IACCRK

Mascot: http://www.matrixscience.com/

Mascot Search Results

Peptide View

MS/MS Fragmentation of **LAADDFR**
Found in **ch17u_O76014|KRT37_HUMAN** in **uni_human**, Keratin, type I cuticular Ha7 OS=Homo sapiens GN=KRT37 PE=3 SV=3

Match to Query 533: 806.392128 from(404.203340,2+) intensity(1147708.3750) rtinseconds(1241) scans(6353) index(35081)
Title: 160219_Sunil_SDSI_A_Spectrum089666_scans__6353_RTINSECONDS=1241
Data file L:\\QE_2016\\160219_Sunil_KAP_LKC\\TMgf\\T\\T160219_Sunil_SDSI_A.mgf

Click mouse within plot area to zoom in by factor of two about that point
Or, Plot from 50 to 700 Da Full range
Label all possible matches ○ Label matches used for scoring ◉
Show Y-axis ☐

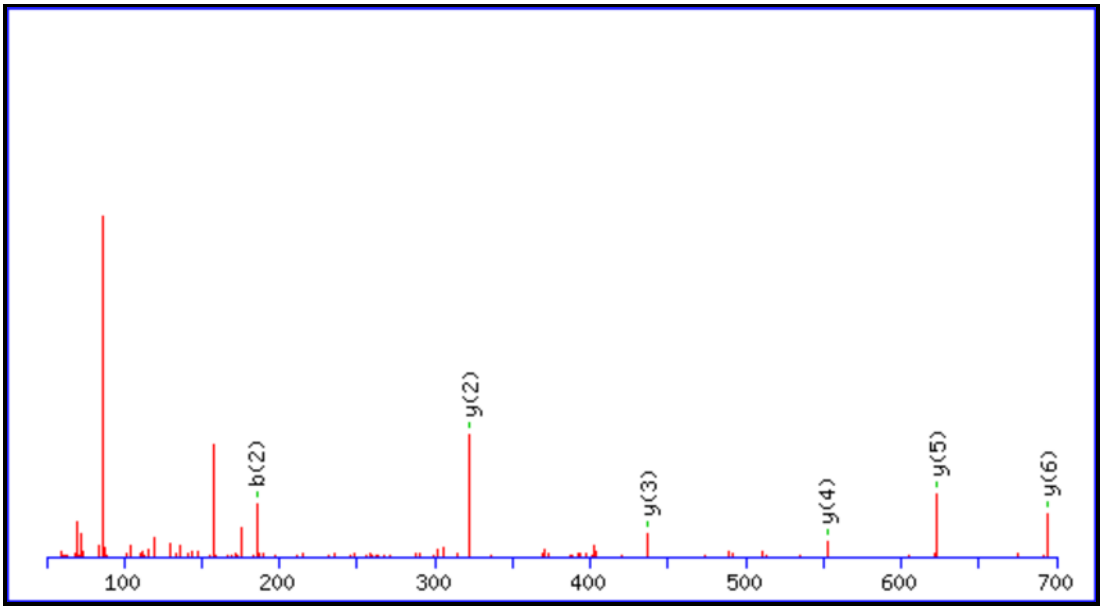

Monoisotopic mass of neutral peptide Mr(calc): 806.3923
Fixed modifications: Carbamidomethyl (C) (apply to specified residues or termini only)
Ions Score: 46 **Expect:** 0.0028
Matches : 6/50 fragment ions using 7 most intense peaks (help)

#	b	b^{++}	b^0	b^{0++}	Seq.	y	y^{++}	y^*	y^{*++}	y^0	y^{0++}	#
1	114.0913	57.5493			L							7
2	**185.1285**	93.0679			A	***694.3155***	347.6614	677.2889	339.1481	676.3049	338.6561	6
3	256.1656	128.5864			A	***623.2784***	312.1428	606.2518	303.6295	605.2678	303.1375	5
4	371.1925	186.0999	353.1819	177.0946	D	***552.2413***	276.6243	535.2147	268.1110	534.2307	267.6190	4
5	486.2195	243.6134	468.2089	234.6081	D	***437.2143***	219.1108	420.1878	210.5975	419.2037	210.1055	3
6	633.2879	317.1476	615.2773	308.1423	F	***322.1874***	161.5973	305.1608	153.0840			2

7					R	175.1190	88.0631	158.0924	79.5498			1

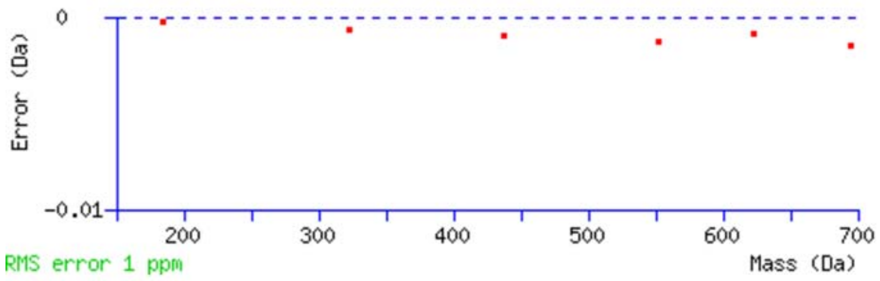

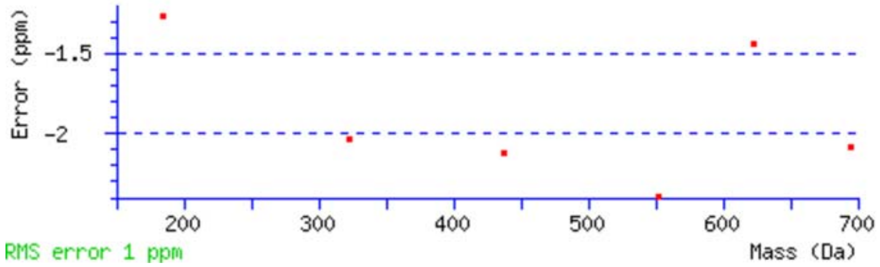

NCBI **BLAST** search of LAADDFR
(Parameters: blastp, nr protein database, expect=20000, no filter, PAM30)
Other BLAST web gateways

All matches to this query

Score	Mr(calc)	Delta	Sequence
46.1	806.3923	-0.0001	LAADDFR
14.7	804.3878	2.0043	RPDNFR
13.6	806.3844	0.0078	LAAQMQK
5.9	806.3923	-0.0001	LDQQFR
5.9	806.3923	-0.0001	LDQQFR
5.5	806.3923	-0.0001	IAGFDER
5.5	806.3922	-0.0001	GELANFR
5.5	804.3878	2.0043	NRPDFR
5.5	806.3923	-0.0001	QLEDFR
3.1	806.3891	0.0030	IACCRK

Mascot: http://www.matrixscience.com/

Mascot Search Results

Peptide View

MS/MS Fragmentation of **LAADDFR**
Found in **ch17u_O76014|KRT37_HUMAN** in **uni_human**, Keratin, type I cuticular Ha7 OS=Homo sapiens GN=KRT37 PE=3 SV=3

Match to Query 534: 806.392128 from(404.203340,2+) intensity(8877412.0000) rtinseconds(1144) scans(5789) index(34593)
Title: 160219_Sunil_SDSI_A_Spectrum089178_scans__5789_RTINSECONDS=1144
Data file L:\\QE_2016\\160219_Sunil_KAP_LKC\\TMgf\\T\\T160219_Sunil_SDSI_A.mgf

Click mouse within plot area to zoom in by factor of two about that point
Or, Plot from 50 to 750 Da Full range
Label all possible matches ○ Label matches used for scoring ◉
Show Y-axis ☐

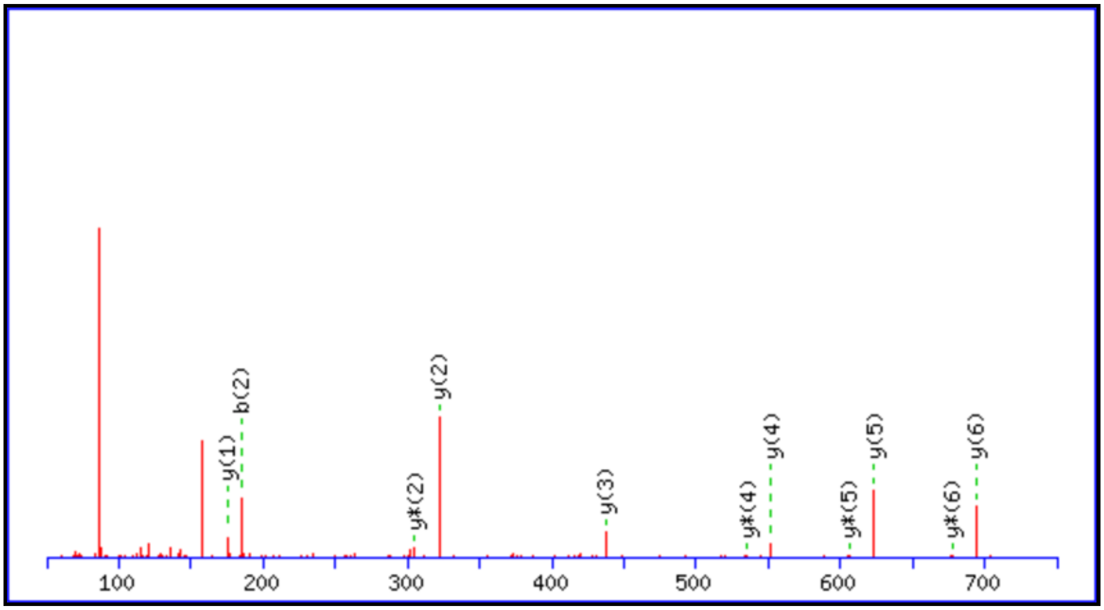

Monoisotopic mass of neutral peptide Mr(calc): 806.3923
Fixed modifications: Carbamidomethyl (C) (apply to specified residues or termini only)
Ions Score: 55 **Expect:** 0.00037
Matches : 11/50 fragment ions using 14 most intense peaks (help)

#	b	b^{++}	b^0	b^{0++}	Seq.	y	y^{++}	y*	y^{*++}	y^0	y^{0++}	#
1	114.0913	57.5493			L							7
2	**185.1285**	93.0679			A	***694.3155***	347.6614	677.2889	339.1481	676.3049	338.6561	6
3	256.1656	128.5864			A	***623.2784***	312.1428	606.2518	303.6295	605.2678	303.1375	5
4	371.1925	186.0999	353.1819	177.0946	D	***552.2413***	276.6243	535.2147	268.1110	534.2307	267.6190	4
5	486.2195	243.6134	468.2089	234.6081	D	***437.2143***	219.1108	420.1878	210.5975	419.2037	210.1055	3
6	633.2879	317.1476	615.2773	308.1423	F	***322.1874***	161.5973	305.1608	153.0840			2

7					R	*175.1190*	88.0631	158.0924	79.5498			1

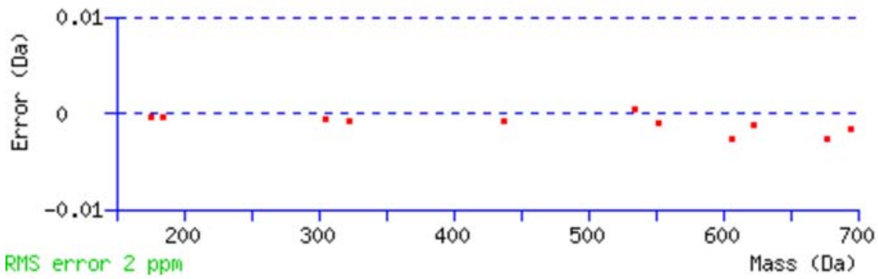

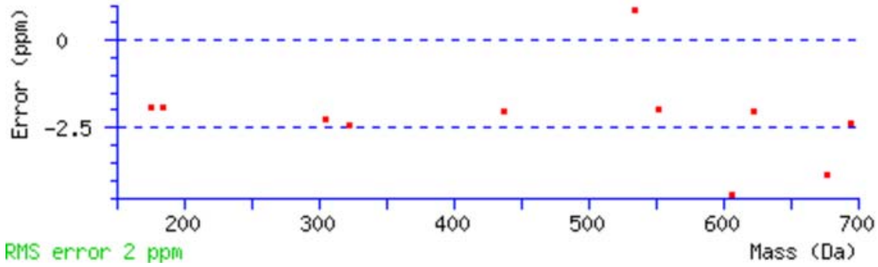

NCBI **BLAST** search of LAADDFR
(Parameters: blastp, nr protein database, expect=20000, no filter, PAM30)
Other BLAST web gateways

All matches to this query

Score	Mr(calc)	Delta	Sequence
54.9	806.3923	-0.0001	LAADDFR
17.4	804.3878	2.0043	RPDNFR
14.5	806.3844	0.0078	LAAQMQK
12.8	806.3923	-0.0001	IAGFDER
8.2	806.3923	-0.0001	LDQQFR
8.2	806.3923	-0.0001	LDQQFR
6.2	806.3922	-0.0001	GELANFR
6.2	804.3878	2.0043	NRPDFR
6.2	806.3923	-0.0001	QLEDFR
4.0	806.3891	0.0030	IACCRK

Mascot: http://www.matrixscience.com/

Mascot Search Results

Peptide View

MS/MS Fragmentation of **LAADDFR**
Found in **ch17u_O76014|KRT37_HUMAN** in **uni_human**, Keratin, type I cuticular Ha7 OS=Homo sapiens GN=KRT37 PE=3 SV=3

Match to Query 537: 806.392188 from(404.203370,2+) intensity(82804752.0000) rtinseconds(1185) scans(6140) index(19653)
Title: 160219_Sunil_SDSI_A_Spectrum072957_scans__6140_RTINSECONDS=1185
Data file L:\\QE_2016\\160219_Sunil_KAP_LKC\\TMgf\\T\\T160219_Sunil_SDSI_A.mgf

Click mouse within plot area to zoom in by factor of two about that point
Or, Plot from 50 to 700 Da Full range
Label all possible matches ○ Label matches used for scoring ◉
Show Y-axis ☐

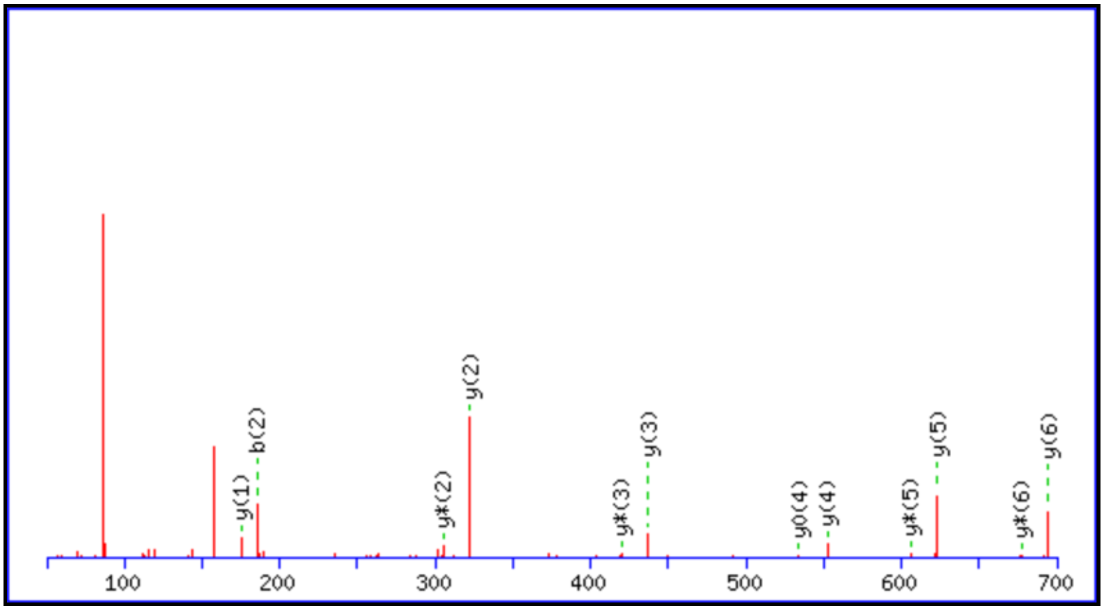

Monoisotopic mass of neutral peptide Mr(calc): 806.3923
Fixed modifications: Carbamidomethyl (C) (apply to specified residues or termini only)
Ions Score: 43 **Expect:** 0.0063
Matches : 12/50 fragment ions using 21 most intense peaks (help)

#	b	b^{++}	b^0	b^{0++}	Seq.	y	y^{++}	y*	y^{*++}	y^0	y^{0++}	#
1	114.0913	57.5493			L							7
2	**185.1285**	93.0679			A	***694.3155***	347.6614	677.2889	339.1481	676.3049	338.6561	6
3	256.1656	128.5864			A	***623.2784***	312.1428	606.2518	303.6295	605.2678	303.1375	5
4	371.1925	186.0999	353.1819	177.0946	D	***552.2413***	276.6243	535.2147	268.1110	534.2307	267.6190	4
5	486.2195	243.6134	468.2089	234.6081	D	***437.2143***	219.1108	420.1878	210.5975	419.2037	210.1055	3
6	633.2879	317.1476	615.2773	308.1423	F	***322.1874***	161.5973	305.1608	153.0840			2

7					R	*175.1190*	88.0631	158.0924	79.5498			1

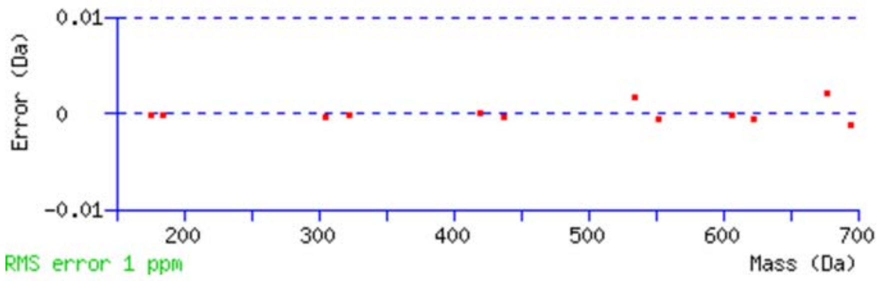

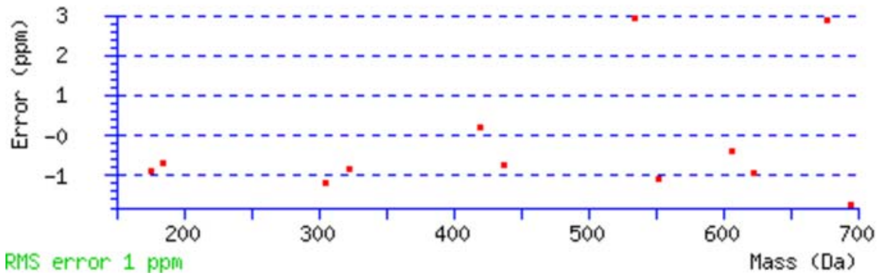

NCBI **BLAST** search of LAADDFR
(Parameters: blastp, nr protein database, expect=20000, no filter, PAM30)
Other BLAST web gateways

All matches to this query

Score	Mr(calc)	Delta	Sequence
42.5	806.3923	-0.0001	LAADDFR
12.6	806.3923	-0.0001	IAGFDER
12.4	804.3878	2.0044	RPDNFR
10.0	806.3844	0.0078	LAAQMQK
5.0	804.3912	2.0010	LASQACR
4.1	806.3923	-0.0001	LDQQFR
4.1	806.3923	-0.0001	LDQQFR
3.6	806.3922	-0.0001	GELANFR
3.6	804.3878	2.0044	NRPDFR
3.6	806.3923	-0.0001	QLEDFR

Mascot: http://www.matrixscience.com/

Mascot Search Results

Peptide View

MS/MS Fragmentation of **LAADDFR**
Found in **ch17u_O76014|KRT37_HUMAN** in **uni_human**, Keratin, type I cuticular Ha7 OS=Homo sapiens GN=KRT37 PE=3 SV=3

Match to Query 537: 806.392188 from(404.203370,2+) intensity(82804752.0000) rtinseconds(1185) scans(6140) index(19653)
Title: 160219_Sunil_SDSI_A_Spectrum072957_scans__6140_RTINSECONDS=1185
Data file L:\\QE_2016\\160219_Sunil_KAP_LKC\\TMgf\\T\\T160219_Sunil_SDSI_A.mgf

Click mouse within plot area to zoom in by factor of two about that point
Or, Plot from 50 to 700 Da Full range
Label all possible matches ○ Label matches used for scoring ◉
Show Y-axis ☐

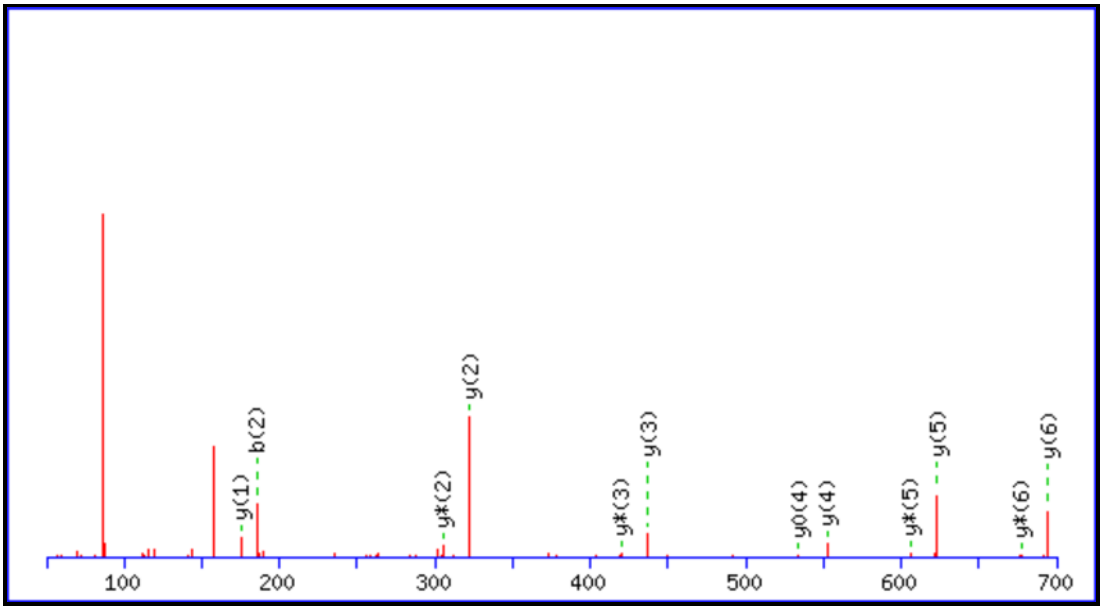

Monoisotopic mass of neutral peptide Mr(calc): 806.3923
Fixed modifications: Carbamidomethyl (C) (apply to specified residues or termini only)
Ions Score: 43 **Expect:** 0.0063
Matches : 12/50 fragment ions using 21 most intense peaks (help)

#	b	b^{++}	b^0	b^{0++}	Seq.	y	y^{++}	y*	y^{*++}	y^0	y^{0++}	#
1	114.0913	57.5493			L							7
2	**185.1285**	93.0679			A	***694.3155***	347.6614	677.2889	339.1481	676.3049	338.6561	6
3	256.1656	128.5864			A	***623.2784***	312.1428	606.2518	303.6295	605.2678	303.1375	5
4	371.1925	186.0999	353.1819	177.0946	D	***552.2413***	276.6243	535.2147	268.1110	534.2307	267.6190	4
5	486.2195	243.6134	468.2089	234.6081	D	***437.2143***	219.1108	420.1878	210.5975	419.2037	210.1055	3
6	633.2879	317.1476	615.2773	308.1423	F	***322.1874***	161.5973	305.1608	153.0840			2

7					R	175.1190	88.0631	158.0924	79.5498			1

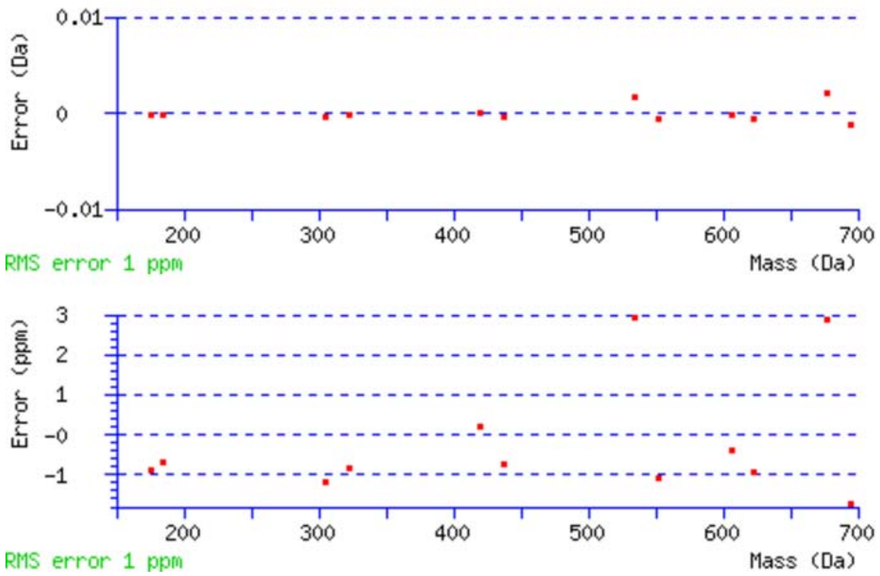

NCBI **BLAST** search of LAADDFR
(Parameters: blastp, nr protein database, expect=20000, no filter, PAM30)
Other BLAST web gateways

All matches to this query

Score	Mr(calc)	Delta	Sequence
42.5	806.3923	-0.0001	LAADDFR
12.6	806.3923	-0.0001	IAGFDER
12.4	804.3878	2.0044	RPDNFR
10.0	806.3844	0.0078	LAAQMQK
5.0	804.3912	2.0010	LASQACR
4.1	806.3923	-0.0001	LDQQFR
4.1	806.3923	-0.0001	LDQQFR
3.6	806.3922	-0.0001	GELANFR
3.6	804.3878	2.0044	NRPDFR
3.6	806.3923	-0.0001	QLEDFR

Mascot: http://www.matrixscience.com/

Mascot Search Results

Peptide View

MS/MS Fragmentation of **LAADDFR**
Found in **ch17u_O76014|KRT37_HUMAN** in **uni_human**, Keratin, type I cuticular Ha7 OS=Homo sapiens GN=KRT37 PE=3 SV=3

Match to Query 539: 806.392188 from(404.203370,2+) intensity(2402318.5000) rtinseconds(1209) scans(6167) index(34922)
Title: 160219_Sunil_SDSI_A_Spectrum089507_scans__6167_RTINSECONDS=1209
Data file L:\\QE_2016\\160219_Sunil_KAP_LKC\\TMgf\\T\\T160219_Sunil_SDSI_A.mgf

Click mouse within plot area to zoom in by factor of two about that point
Or, Plot from 50 to 750 Da Full range
Label all possible matches ○ Label matches used for scoring ◉
Show Y-axis ☐

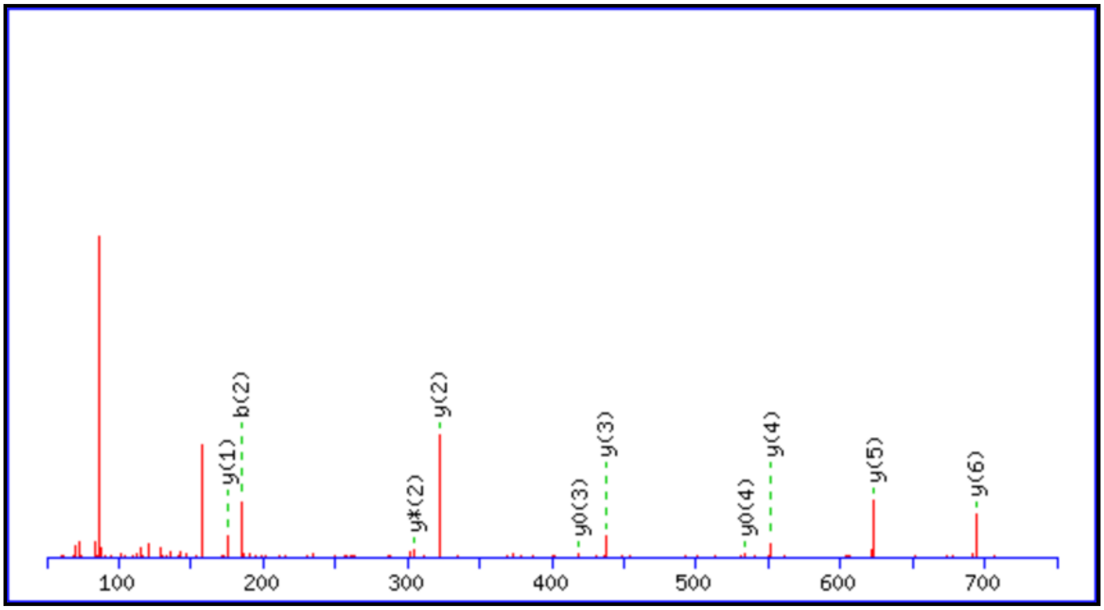

Monoisotopic mass of neutral peptide Mr(calc): 806.3923
Fixed modifications: Carbamidomethyl (C) (apply to specified residues or termini only)
Ions Score: 51 **Expect:** 0.00084
Matches : 10/50 fragment ions using 14 most intense peaks (help)

#	b	b^{++}	b^{0}	b^{0++}	Seq.	y	y^{++}	y*	y^{*++}	y^{0}	y^{0++}	#
1	114.0913	57.5493			L							7
2	**185.1285**	93.0679			A	***694.3155***	347.6614	677.2889	339.1481	676.3049	338.6561	6
3	256.1656	128.5864			A	***623.2784***	312.1428	606.2518	303.6295	605.2678	303.1375	5
4	371.1925	186.0999	353.1819	177.0946	D	***552.2413***	276.6243	535.2147	268.1110	534.2307	267.6190	4
5	486.2195	243.6134	468.2089	234.6081	D	***437.2143***	219.1108	420.1878	210.5975	419.2037	210.1055	3
6	633.2879	317.1476	615.2773	308.1423	F	***322.1874***	161.5973	305.1608	153.0840			2

7					R	*175.1190*	88.0631	158.0924	79.5498			1

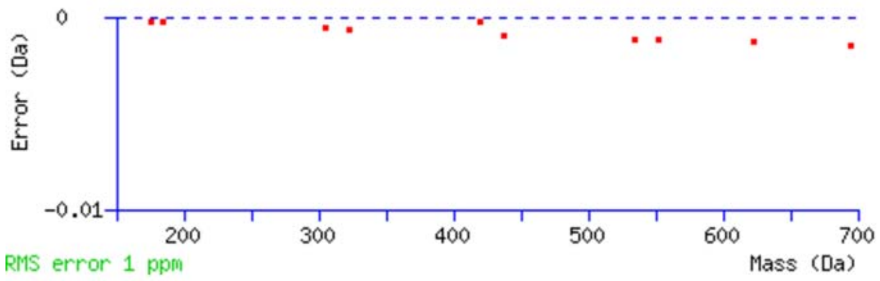

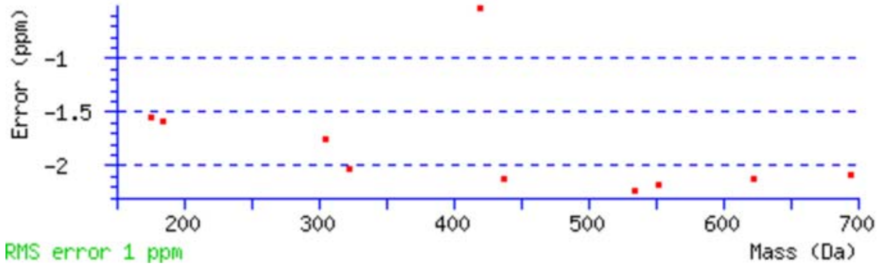

NCBI **BLAST** search of LAADDFR
(Parameters: blastp, nr protein database, expect=20000, no filter, PAM30)
Other BLAST web gateways

All matches to this query

Score	Mr(calc)	Delta	Sequence
51.3	806.3923	-0.0001	LAADDFR
17.0	804.3878	2.0044	RPDNFR
14.7	806.3923	-0.0001	IAGFDER
14.0	806.3844	0.0078	LAAQMQK
11.7	804.3912	2.0010	LASQACR
7.2	804.3878	2.0044	NRPDFR
7.2	806.3923	-0.0001	QLEDFR
6.3	806.3923	-0.0001	LDQQFR
6.3	806.3923	-0.0001	LDQQFR
5.7	806.3922	-0.0001	GELANFR

Mascot: http://www.matrixscience.com/

Mascot Search Results

Peptide View

MS/MS Fragmentation of **LAADDFR**
Found in **ch17u_O76014|KRT37_HUMAN** in **uni_human**, Keratin, type I cuticular Ha7 OS=Homo sapiens GN=KRT37 PE=3 SV=3

Match to Query 540: 806.392248 from(404.203400,2+) intensity(448258.5313) rtinseconds(1583) scans(8303) index(6184)
Title: 160219_Sunil_SDSI_A_Spectrum058292_scans__8303_RTINSECONDS=1583
Data file L:\\QE_2016\\160219_Sunil_KAP_LKC\\TMgf\\T\\T160219_Sunil_SDSI_A.mgf

Click mouse within plot area to zoom in by factor of two about that point
Or, Plot from 50 to 700 Da Full range
Label all possible matches ○ Label matches used for scoring ◉
Show Y-axis ☐

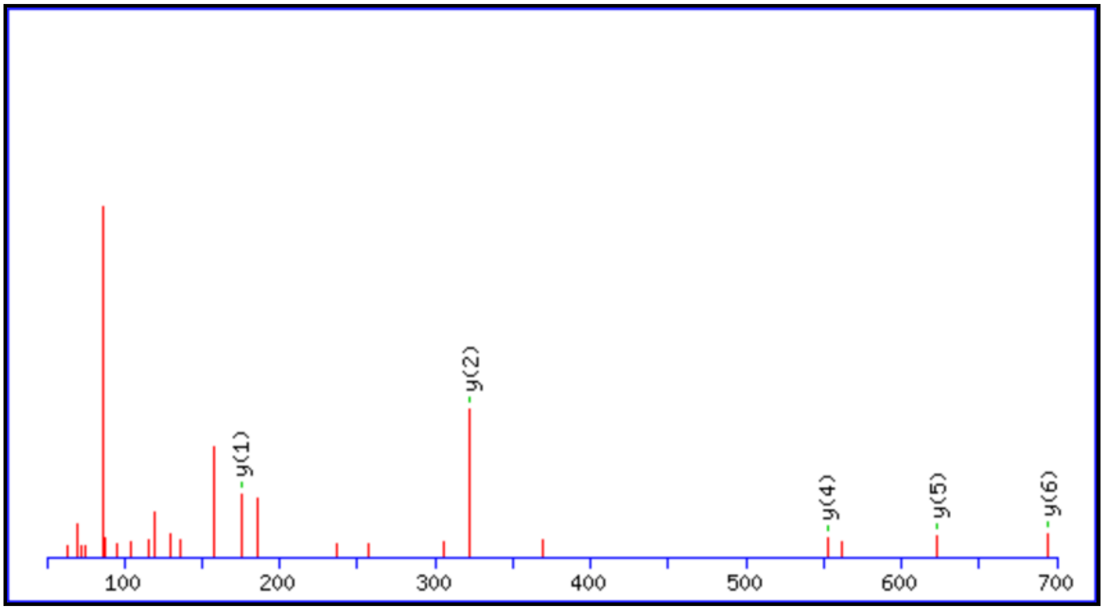

Monoisotopic mass of neutral peptide Mr(calc): 806.3923
Fixed modifications: Carbamidomethyl (C) (apply to specified residues or termini only)
Ions Score: 41 **Expect:** 0.0091
Matches : 5/50 fragment ions using 7 most intense peaks (help)

#	b	b^{++}	b^{0}	b^{0++}	Seq.	y	y^{++}	y^{*}	y^{*++}	y^{0}	y^{0++}	#
1	114.0913	57.5493			L							7
2	185.1285	93.0679			A	***694.3155***	347.6614	677.2889	339.1481	676.3049	338.6561	6
3	256.1656	128.5864			A	***623.2784***	312.1428	606.2518	303.6295	605.2678	303.1375	5
4	371.1925	186.0999	353.1819	177.0946	D	***552.2413***	276.6243	535.2147	268.1110	534.2307	267.6190	4
5	486.2195	243.6134	468.2089	234.6081	D	437.2143	219.1108	420.1878	210.5975	419.2037	210.1055	3
6	633.2879	317.1476	615.2773	308.1423	F	***322.1874***	161.5973	305.1608	153.0840			2

7					R	*175.1190*	88.0631	158.0924	79.5498			1

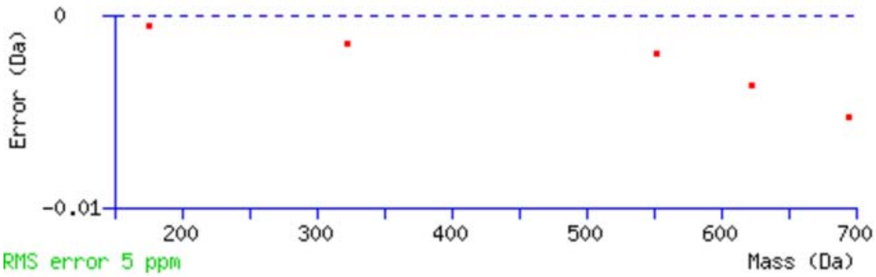

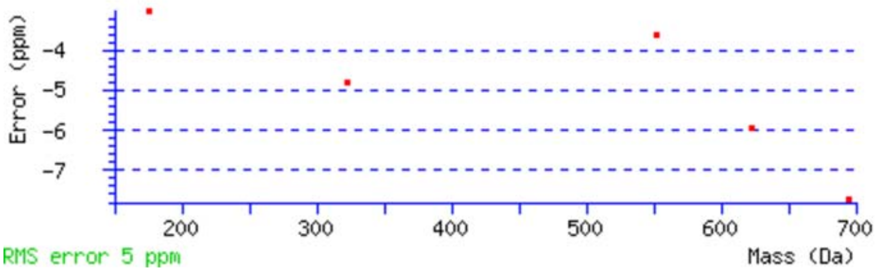

NCBI **BLAST** search of LAADDFR
(Parameters: blastp, nr protein database, expect=20000, no filter, PAM30)
Other BLAST web gateways

All matches to this query

Score	Mr(calc)	Delta	Sequence
40.9	806.3923	-0.0000	LAADDFR
12.3	806.3923	-0.0000	LDQQFR
12.3	806.3923	-0.0000	LDQQFR
12.3	804.3878	2.0044	RPDNFR
8.9	806.3923	-0.0000	IAGFDER
6.6	806.3844	0.0079	LAAQMQK
0.0	805.3871	1.0051	EHAFFR
0.0	806.3922	0.0000	ELENFR
0.0	806.3922	0.0000	ELNEFR
0.0	806.3922	0.0000	GELANFR

Mascot: http://www.matrixscience.com/

Mascot Search Results

Peptide View

MS/MS Fragmentation of **LAADDFR**
Found in **ch17u_O76014|KRT37_HUMAN** in **uni_human**, Keratin, type I cuticular Ha7 OS=Homo sapiens GN=KRT37 PE=3 SV=3

Match to Query 541: 806.392248 from(404.203400,2+) intensity(1292910.3750) rtinseconds(1440) scans(7623) index(20947)
Title: 160219_Sunil_SDSI_A_Spectrum074251_scans__7623_RTINSECONDS=1440
Data file L:\\QE_2016\\160219_Sunil_KAP_LKC\\TMgf\\T\\T160219_Sunil_SDSI_A.mgf

Click mouse within plot area to zoom in by factor of two about that point
Or, Plot from 50 to 700 Da Full range
Label all possible matches ○ Label matches used for scoring ◉
Show Y-axis ☐

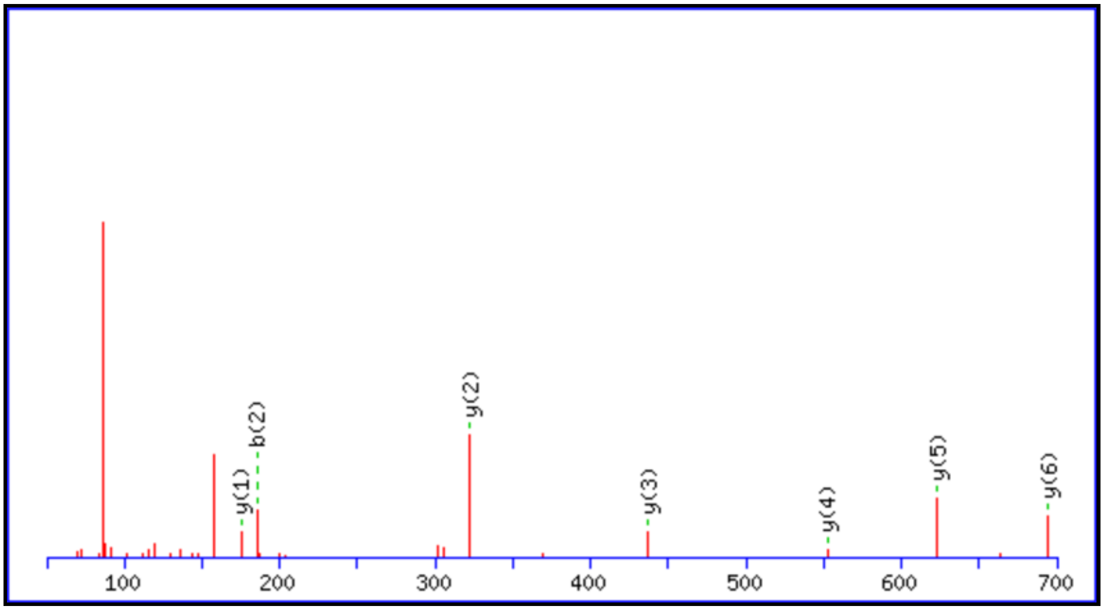

Monoisotopic mass of neutral peptide Mr(calc): 806.3923
Fixed modifications: Carbamidomethyl (C) (apply to specified residues or termini only)
Ions Score: 50 **Expect:** 0.0012
Matches : 7/50 fragment ions using 11 most intense peaks (help)

#	b	b^{++}	b^0	b^{0++}	Seq.	y	y^{++}	y*	y^{*++}	y^0	y^{0++}	#
1	114.0913	57.5493			L							7
2	**185.1285**	93.0679			A	***694.3155***	347.6614	677.2889	339.1481	676.3049	338.6561	6
3	256.1656	128.5864			A	***623.2784***	312.1428	606.2518	303.6295	605.2678	303.1375	5
4	371.1925	186.0999	353.1819	177.0946	D	***552.2413***	276.6243	535.2147	268.1110	534.2307	267.6190	4
5	486.2195	243.6134	468.2089	234.6081	D	***437.2143***	219.1108	420.1878	210.5975	419.2037	210.1055	3
6	633.2879	317.1476	615.2773	308.1423	F	***322.1874***	161.5973	305.1608	153.0840			2

7					R	175.1190	88.0631	158.0924	79.5498			1

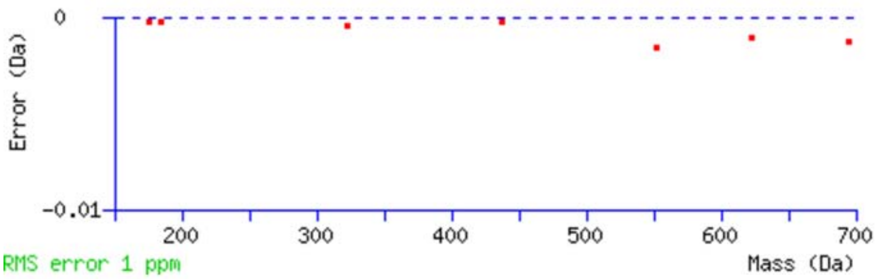

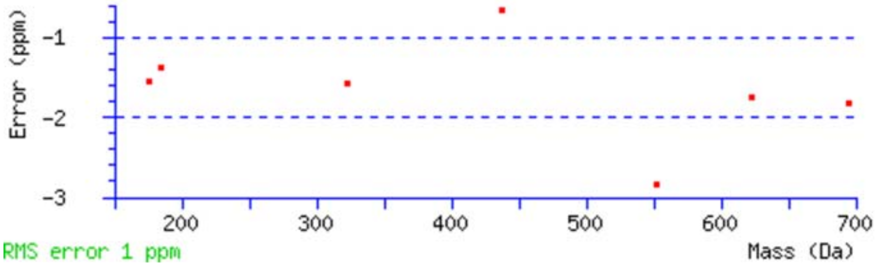

NCBI **BLAST** search of LAADDFR
(Parameters: blastp, nr protein database, expect=20000, no filter, PAM30)
Other BLAST web gateways

All matches to this query

Score	Mr(calc)	Delta	Sequence
49.9	806.3923	-0.0000	LAADDFR
16.6	804.3878	2.0044	RPDNFR
13.9	806.3844	0.0079	LAAQMQK
8.6	806.3923	-0.0000	IAGFDER
7.8	806.3922	0.0000	GELANFR
7.2	806.3923	-0.0000	LDQQFR
7.2	806.3923	-0.0000	LDQQFR
6.8	804.3878	2.0044	NRPDFR
6.8	806.3923	-0.0000	QLEDFR
3.6	806.3891	0.0031	IACCRK

Mascot: http://www.matrixscience.com/

Mascot Search Results

Peptide View

MS/MS Fragmentation of **LAADDFR**
Found in **ch17u_O76014|KRT37_HUMAN** in **uni_human**, Keratin, type I cuticular Ha7 OS=Homo sapiens GN=KRT37 PE=3 SV=3

Match to Query 541: 806.392248 from(404.203400,2+) intensity(1292910.3750) rtinseconds(1440) scans(7623) index(20947)
Title: 160219_Sunil_SDSI_A_Spectrum074251_scans__7623_RTINSECONDS=1440
Data file L:\\QE_2016\\160219_Sunil_KAP_LKC\\TMgf\\T\\T160219_Sunil_SDSI_A.mgf

Click mouse within plot area to zoom in by factor of two about that point
Or, Plot from 50 to 700 Da Full range
Label all possible matches ○ Label matches used for scoring ◉
Show Y-axis ☐

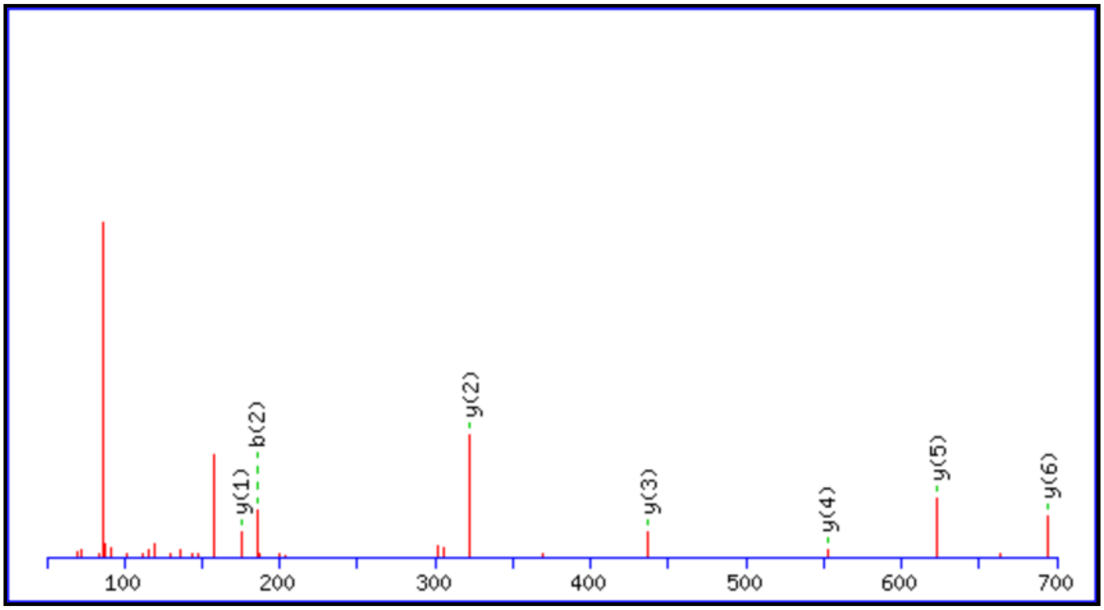

Monoisotopic mass of neutral peptide Mr(calc): 806.3923
Fixed modifications: Carbamidomethyl (C) (apply to specified residues or termini only)
Ions Score: 50 **Expect:** 0.0012
Matches : 7/50 fragment ions using 11 most intense peaks (help)

#	b	b^{++}	b^0	b^{0++}	Seq.	y	y^{++}	y*	y^{*++}	y^0	y^{0++}	#
1	114.0913	57.5493			L							7
2	**185.1285**	93.0679			A	***694.3155***	347.6614	677.2889	339.1481	676.3049	338.6561	6
3	256.1656	128.5864			A	***623.2784***	312.1428	606.2518	303.6295	605.2678	303.1375	5
4	371.1925	186.0999	353.1819	177.0946	D	***552.2413***	276.6243	535.2147	268.1110	534.2307	267.6190	4
5	486.2195	243.6134	468.2089	234.6081	D	***437.2143***	219.1108	420.1878	210.5975	419.2037	210.1055	3
6	633.2879	317.1476	615.2773	308.1423	F	***322.1874***	161.5973	305.1608	153.0840			2

7					R	*175.1190*	88.0631	158.0924	79.5498			1

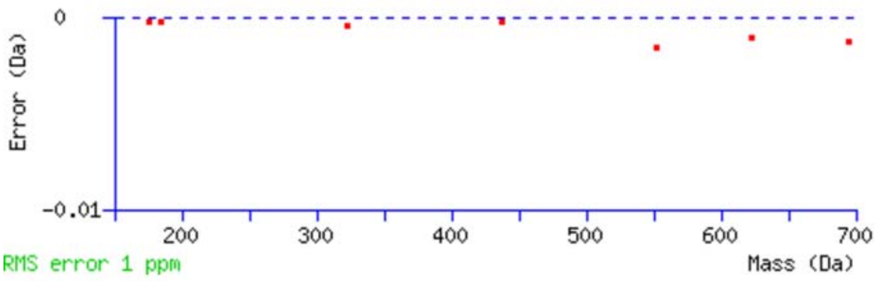

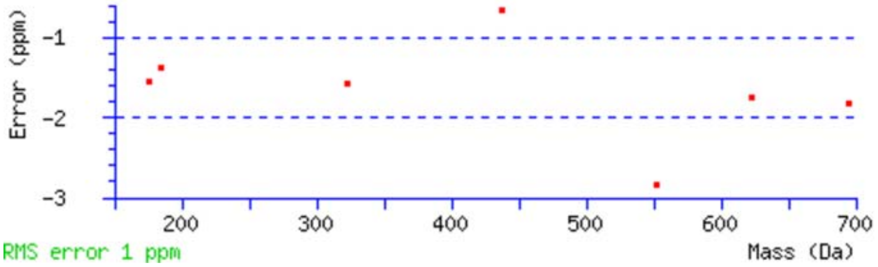

NCBI **BLAST** search of LAADDFR
(Parameters: blastp, nr protein database, expect=20000, no filter, PAM30)
Other BLAST web gateways

All matches to this query

Score	Mr(calc)	Delta	Sequence
49.9	806.3923	-0.0000	LAADDFR
16.6	804.3878	2.0044	RPDNFR
13.9	806.3844	0.0079	LAAQMQK
8.6	806.3923	-0.0000	IAGFDER
7.8	806.3922	0.0000	GELANFR
7.2	806.3923	-0.0000	LDQQFR
7.2	806.3923	-0.0000	LDQQFR
6.8	804.3878	2.0044	NRPDFR
6.8	806.3923	-0.0000	QLEDFR
3.6	806.3891	0.0031	IACCRK

Mascot: http://www.matrixscience.com/

Mascot Search Results

Peptide View

MS/MS Fragmentation of **LAADDFR**
Found in **ch17u_O76014|KRT37_HUMAN** in **uni_human**, Keratin, type I cuticular Ha7 OS=Homo sapiens GN=KRT37 PE=3 SV=3

Match to Query 542: 806.392248 from(404.203400,2+) intensity(164081.1719) rtinseconds(1427) scans(7427) index(36025)
Title: 160219_Sunil_SDSI_A_Spectrum090610_scans__7427_RTINSECONDS=1427
Data file L:\\QE_2016\\160219_Sunil_KAP_LKC\\TMgf\\T\\T160219_Sunil_SDSI_A.mgf

Click mouse within plot area to zoom in by factor of two about that point
Or, Plot from 50 to 700 Da Full range
Label all possible matches ○ Label matches used for scoring ◉
Show Y-axis ☐

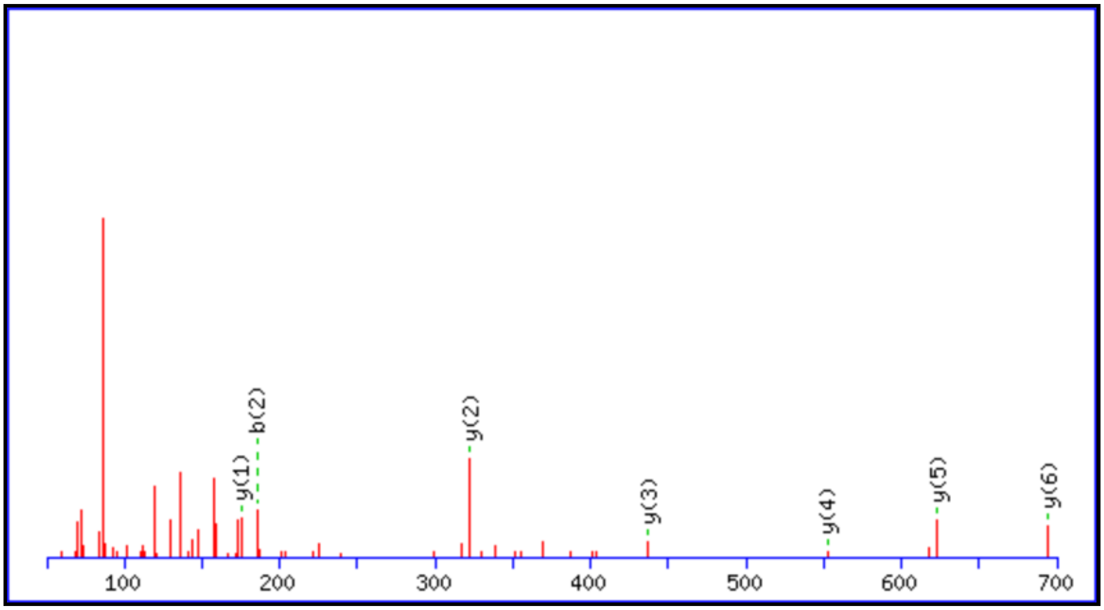

Monoisotopic mass of neutral peptide Mr(calc): 806.3923
Fixed modifications: Carbamidomethyl (C) (apply to specified residues or termini only)
Ions Score: 45 **Expect:** 0.0036
Matches : 7/50 fragment ions using 12 most intense peaks (help)

#	b	b^{++}	b^{0}	b^{0++}	Seq.	y	y^{++}	y^{*}	y^{*++}	y^{0}	y^{0++}	#
1	114.0913	57.5493			L							7
2	**185.1285**	93.0679			A	***694.3155***	347.6614	677.2889	339.1481	676.3049	338.6561	6
3	256.1656	128.5864			A	***623.2784***	312.1428	606.2518	303.6295	605.2678	303.1375	5
4	371.1925	186.0999	353.1819	177.0946	D	***552.2413***	276.6243	535.2147	268.1110	534.2307	267.6190	4
5	486.2195	243.6134	468.2089	234.6081	D	***437.2143***	219.1108	420.1878	210.5975	419.2037	210.1055	3
6	633.2879	317.1476	615.2773	308.1423	F	***322.1874***	161.5973	305.1608	153.0840			2

7					R	*175.1190*	88.0631	158.0924	79.5498			1

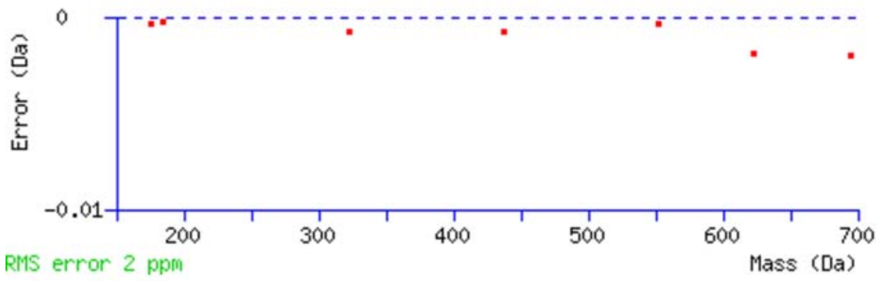

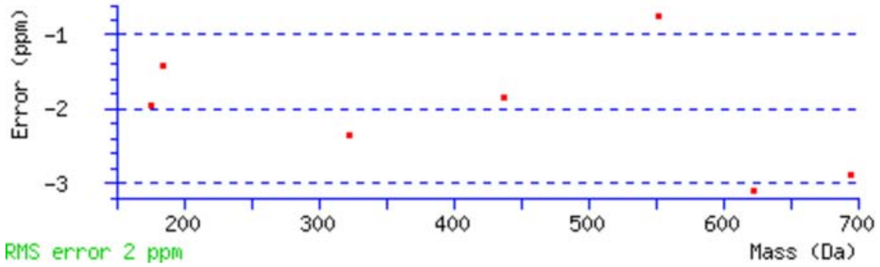

NCBI **BLAST** search of LAADDFR
(Parameters: blastp, nr protein database, expect=20000, no filter, PAM30)
Other BLAST web gateways

All matches to this query

Score	Mr(calc)	Delta	Sequence
44.9	806.3923	-0.0000	LAADDFR
13.2	804.3878	2.0044	RPDNFR
11.4	806.3844	0.0079	LAAQMQK
5.7	806.3923	-0.0000	IAGFDER
4.4	806.3923	-0.0000	LDQQFR
4.4	806.3923	-0.0000	LDQQFR
4.0	806.3922	0.0000	GELANFR
4.0	804.3878	2.0044	NRPDFR
4.0	806.3923	-0.0000	QLEDFR
1.0	806.3891	0.0031	IACCRK

Mascot: http://www.matrixscience.com/

Mascot Search Results

Peptide View

MS/MS Fragmentation of **LAADDFR**
Found in **ch17u_O76014|KRT37_HUMAN** in **uni_human**, Keratin, type I cuticular Ha7 OS=Homo sapiens GN=KRT37 PE=3 SV=3

Match to Query 543: 806.392248 from(404.203400,2+) intensity(845714.8750) rtinseconds(1272) scans(6529) index(35235)
Title: 160219_Sunil_SDSI_A_Spectrum089820_scans__6529_RTINSECONDS=1272
Data file L:\\QE_2016\\160219_Sunil_KAP_LKC\\TMgf\\T\\T160219_Sunil_SDSI_A.mgf

Click mouse within plot area to zoom in by factor of two about that point
Or, Plot from 50 to 750 Da Full range
Label all possible matches ○ Label matches used for scoring ◉
Show Y-axis ☐

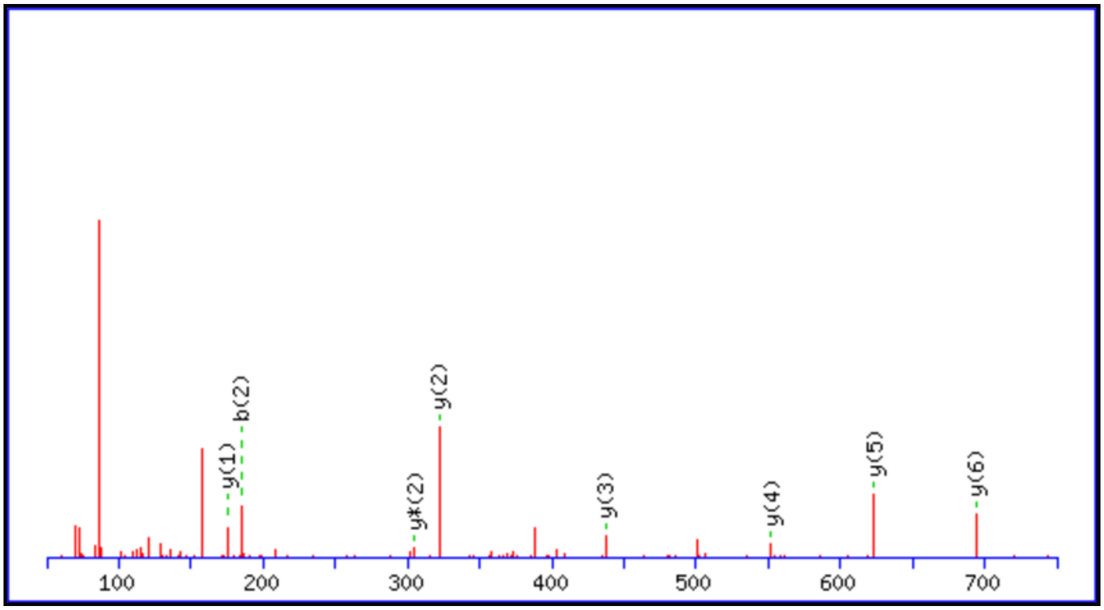

Monoisotopic mass of neutral peptide Mr(calc): 806.3923
Fixed modifications: Carbamidomethyl (C) (apply to specified residues or termini only)
Ions Score: 45 **Expect:** 0.0036
Matches : 8/50 fragment ions using 14 most intense peaks (help)

#	b	b^{++}	b^0	b^{0++}	Seq.	y	y^{++}	y*	y^{*++}	y^0	y^{0++}	#
1	114.0913	57.5493			L							7
2	**185.1285**	93.0679			A	***694.3155***	347.6614	677.2889	339.1481	676.3049	338.6561	6
3	256.1656	128.5864			A	***623.2784***	312.1428	606.2518	303.6295	605.2678	303.1375	5
4	371.1925	186.0999	353.1819	177.0946	D	***552.2413***	276.6243	535.2147	268.1110	534.2307	267.6190	4
5	486.2195	243.6134	468.2089	234.6081	D	***437.2143***	219.1108	420.1878	210.5975	419.2037	210.1055	3
6	633.2879	317.1476	615.2773	308.1423	F	***322.1874***	161.5973	305.1608	153.0840			2

7					R	*175.1190*	88.0631	158.0924	79.5498			1

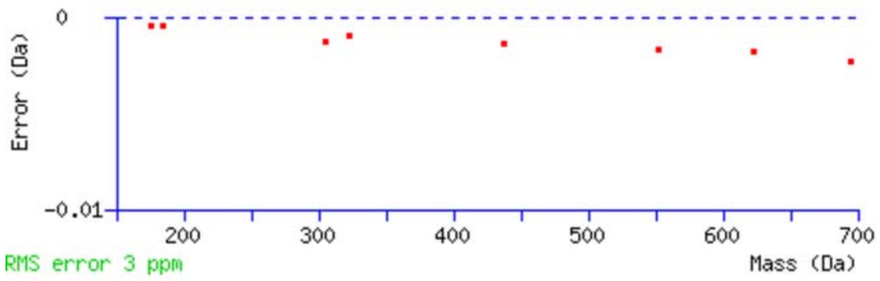

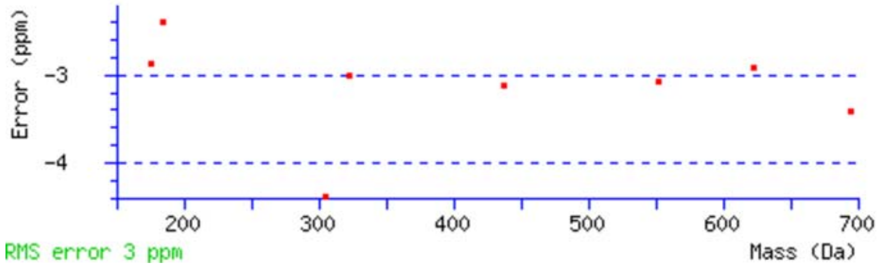

NCBI **BLAST** search of LAADDFR
(Parameters: blastp, nr protein database, expect=20000, no filter, PAM30)
Other BLAST web gateways

All matches to this query

Score	Mr(calc)	Delta	Sequence
45.0	806.3923	-0.0000	LAADDFR
14.6	804.3878	2.0044	RPDNFR
6.0	806.3923	-0.0000	LDQQFR
6.0	806.3923	-0.0000	LDQQFR
5.5	806.3922	0.0000	GELANFR
5.5	804.3878	2.0044	NRPDFR
5.5	806.3923	-0.0000	QLEDFR
5.4	806.3923	-0.0000	IAGFDER
5.0	806.3844	0.0079	LAAQMQK
2.9	806.3891	0.0031	IACCRK

Mascot: http://www.matrixscience.com/

Mascot Search Results

Peptide View

MS/MS Fragmentation of **LAADDFR**
Found in **ch17u_O76014|KRT37_HUMAN** in **uni_human**, Keratin, type I cuticular Ha7 OS=Homo sapiens GN=KRT37 PE=3 SV=3

Match to Query 545: 806.392308 from(404.203430,2+) intensity(6653584.5000) rtinseconds(1280) scans(6695) index(20127)
Title: 160219_Sunil_SDSI_A_Spectrum073431_scans__6695_RTINSECONDS=1280
Data file L:\\QE_2016\\160219_Sunil_KAP_LKC\\TMgf\\T\\T160219_Sunil_SDSI_A.mgf

Click mouse within plot area to zoom in by factor of two about that point
Or, Plot from 50 to 700 Da Full range
Label all possible matches ○ Label matches used for scoring ◉
Show Y-axis ☐

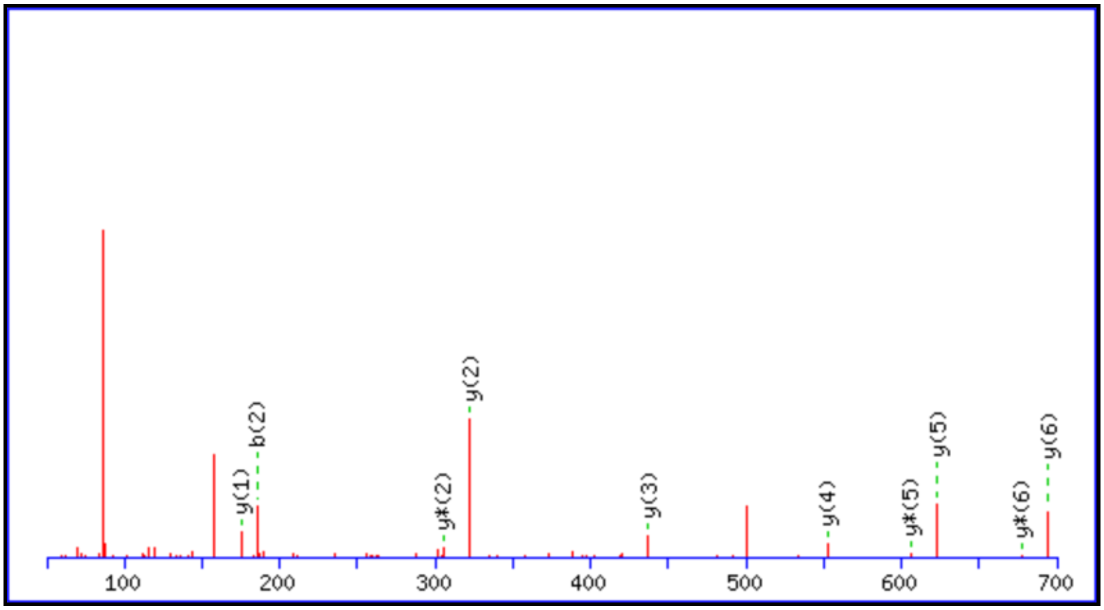

Monoisotopic mass of neutral peptide Mr(calc): 806.3923
Fixed modifications: Carbamidomethyl (C) (apply to specified residues or termini only)
Ions Score: 51 **Expect:** 0.00086
Matches : 10/50 fragment ions using 14 most intense peaks (help)

#	b	b^{++}	b^0	b^{0++}	Seq.	y	y^{++}	y*	y^{*++}	y^0	y^{0++}	#
1	114.0913	57.5493			L							7
2	**185.1285**	93.0679			A	***694.3155***	347.6614	677.2889	339.1481	676.3049	338.6561	6
3	256.1656	128.5864			A	***623.2784***	312.1428	606.2518	303.6295	605.2678	303.1375	5
4	371.1925	186.0999	353.1819	177.0946	D	***552.2413***	276.6243	535.2147	268.1110	534.2307	267.6190	4
5	486.2195	243.6134	468.2089	234.6081	D	***437.2143***	219.1108	420.1878	210.5975	419.2037	210.1055	3
6	633.2879	317.1476	615.2773	308.1423	F	***322.1874***	161.5973	305.1608	153.0840			2

7					R	*175.1190*	88.0631	158.0924	79.5498			1

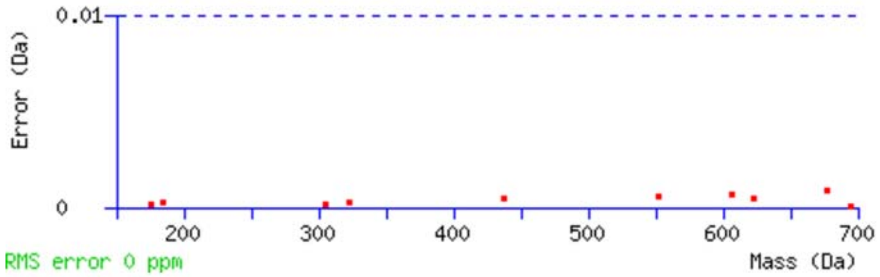

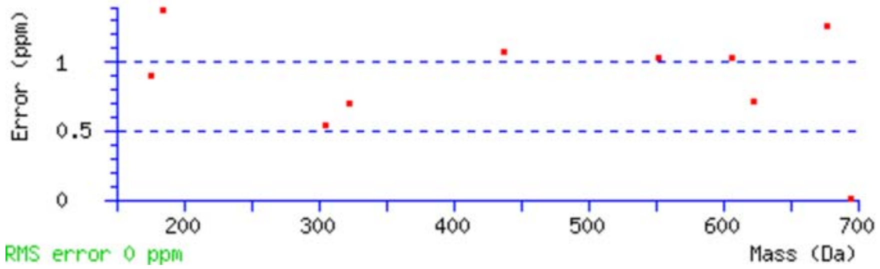

NCBI **BLAST** search of LAADDFR
(Parameters: blastp, nr protein database, expect=20000, no filter, PAM30)
Other BLAST web gateways

All matches to this query

Score	Mr(calc)	Delta	Sequence
51.4	806.3923	0.0001	LAADDFR
15.3	804.3878	2.0045	RPDNFR
8.6	806.3923	0.0001	IAGFDER
8.3	806.3844	0.0079	LAAQMQK
8.1	806.3923	0.0001	LDQQFR
8.1	806.3923	0.0001	LDQQFR
6.1	806.3922	0.0001	GELANFR
6.1	804.3878	2.0045	NRPDFR
6.1	806.3923	0.0001	QLEDFR
3.2	806.3891	0.0032	IACCRK

Mascot: http://www.matrixscience.com/

Mascot Search Results

Peptide View

MS/MS Fragmentation of **LAADDFR**
Found in **ch17u_O76014|KRT37_HUMAN** in **uni_human**, Keratin, type I cuticular Ha7 OS=Homo sapiens GN=KRT37 PE=3 SV=3

Match to Query 546: 806.392368 from(404.203460,2+) intensity(2980319.2500) rtinseconds(1417) scans(7337) index(5327)
Title: 160219_Sunil_SDSI_A_Spectrum057435_scans__7337_RTINSECONDS=1417
Data file L:\\QE_2016\\160219_Sunil_KAP_LKC\\TMgf\\T\\T160219_Sunil_SDSI_A.mgf

Click mouse within plot area to zoom in by factor of two about that point
Or, Plot from 50 to 700 Da Full range
Label all possible matches ○ Label matches used for scoring ◉
Show Y-axis ☐

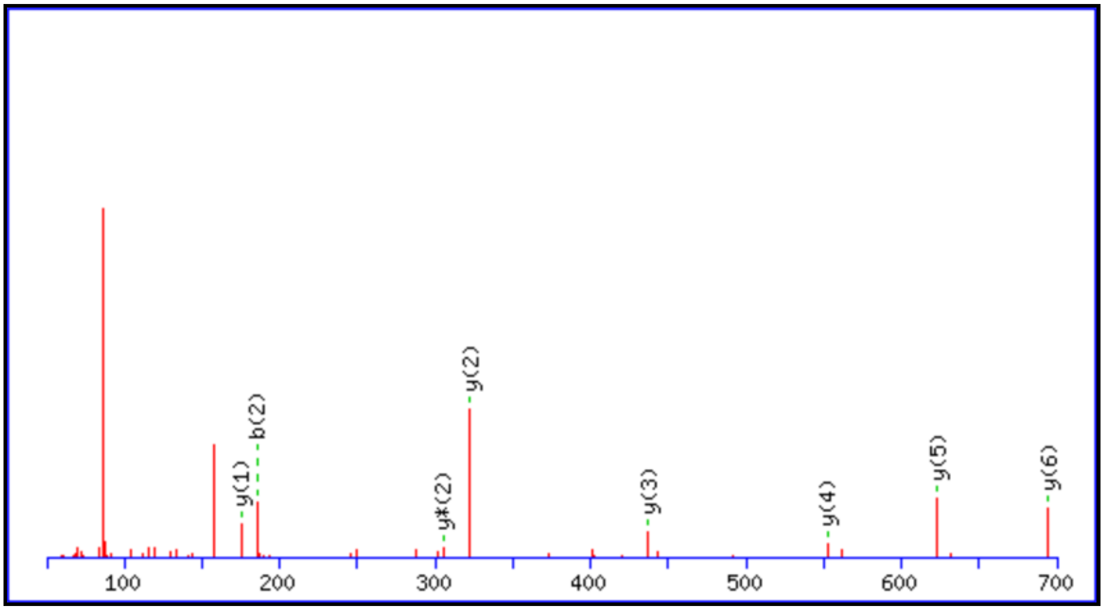

Monoisotopic mass of neutral peptide Mr(calc): 806.3923
Fixed modifications: Carbamidomethyl (C) (apply to specified residues or termini only)
Ions Score: 48 **Expect:** 0.0018
Matches : 8/50 fragment ions using 13 most intense peaks (help)

#	b	b^{++}	b^0	b^{0++}	Seq.	y	y^{++}	y*	y^{*++}	y^0	y^{0++}	#
1	114.0913	57.5493			L							7
2	**185.1285**	93.0679			A	***694.3155***	347.6614	677.2889	339.1481	676.3049	338.6561	6
3	256.1656	128.5864			A	***623.2784***	312.1428	606.2518	303.6295	605.2678	303.1375	5
4	371.1925	186.0999	353.1819	177.0946	D	***552.2413***	276.6243	535.2147	268.1110	534.2307	267.6190	4
5	486.2195	243.6134	468.2089	234.6081	D	***437.2143***	219.1108	420.1878	210.5975	419.2037	210.1055	3
6	633.2879	317.1476	615.2773	308.1423	F	***322.1874***	161.5973	305.1608	153.0840			2

7					R	175.1190	88.0631	158.0924	79.5498			1

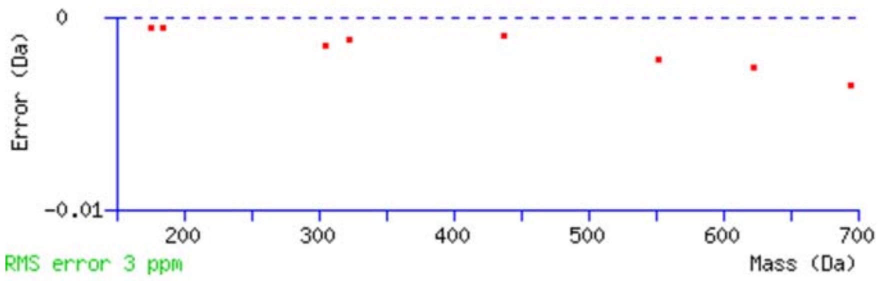

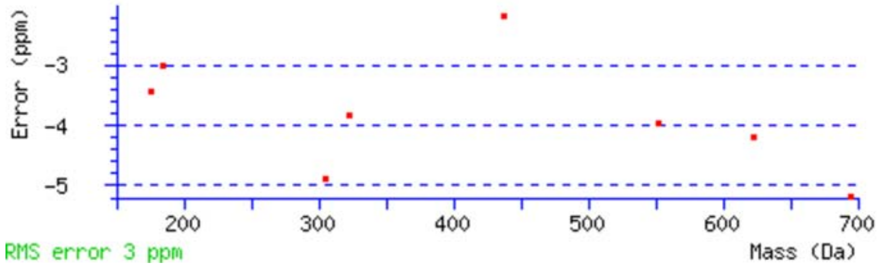

NCBI **BLAST** search of LAADDFR
(Parameters: blastp, nr protein database, expect=20000, no filter, PAM30)
Other BLAST web gateways

All matches to this query

Score	Mr(calc)	Delta	Sequence
48.1	806.3923	0.0001	LAADDFR
16.8	804.3878	2.0045	RPDNFR
14.0	806.3844	0.0080	LAAQMQK
7.7	806.3923	0.0001	LDQQFR
7.7	806.3923	0.0001	LDQQFR
7.3	806.3922	0.0001	GELANFR
7.3	804.3878	2.0045	NRPDFR
7.3	806.3923	0.0001	QLEDFR
6.8	806.3923	0.0001	IAGFDER
3.7	806.3956	-0.0032	MGNSLLR

Mascot: http://www.matrixscience.com/

Mascot Search Results

Peptide View

MS/MS Fragmentation of **LAADDFR**
Found in **ch17u_O76014|KRT37_HUMAN** in **uni_human**, Keratin, type I cuticular Ha7 OS=Homo sapiens GN=KRT37 PE=3 SV=3

Match to Query 547: 806.392428 from(404.203490,2+) intensity(17079980.0000) rtinseconds(1215) scans(6317) index(19803)
Title: 160219_Sunil_SDSI_A_Spectrum073107_scans__6317_RTINSECONDS=1215
Data file L:\\QE_2016\\160219_Sunil_KAP_LKC\\TMgf\\T\\T160219_Sunil_SDSI_A.mgf

Click mouse within plot area to zoom in by factor of two about that point
Or, Plot from 50 to 700 Da Full range
Label all possible matches ○ Label matches used for scoring ◉
Show Y-axis ☐

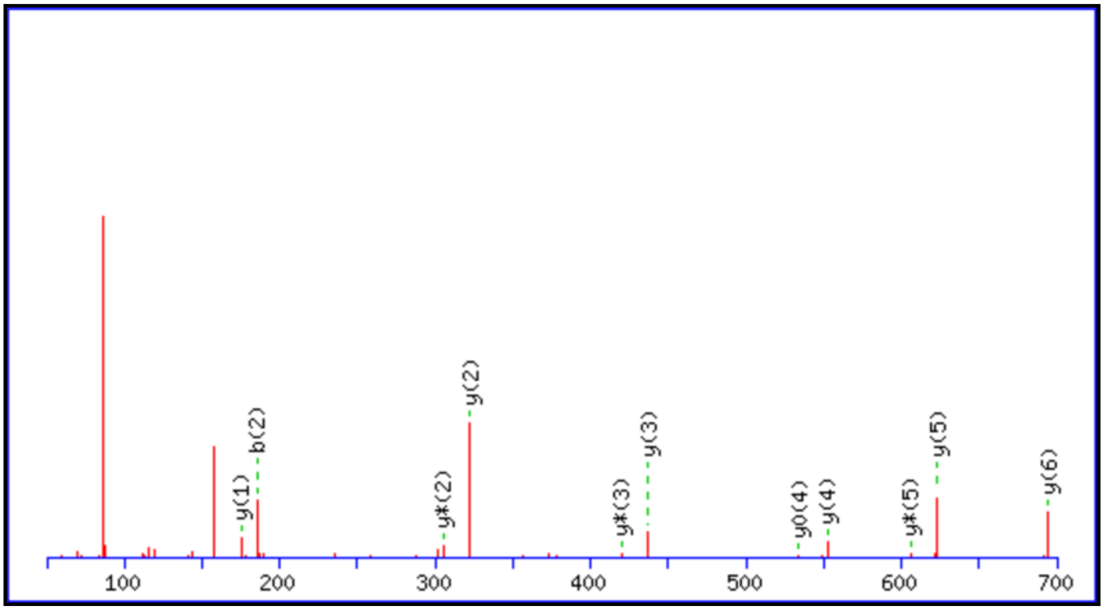

Monoisotopic mass of neutral peptide Mr(calc): 806.3923
Fixed modifications: Carbamidomethyl (C) (apply to specified residues or termini only)
Ions Score: 55 **Expect:** 0.00038
Matches : 11/50 fragment ions using 14 most intense peaks (help)

#	b	b++	b0	b0++	Seq.	y	y++	y*	y*++	y0	y0++	#
1	114.0913	57.5493			L							7
2	**185.1285**	93.0679			A	***694.3155***	347.6614	677.2889	339.1481	676.3049	338.6561	6
3	256.1656	128.5864			A	***623.2784***	312.1428	606.2518	303.6295	605.2678	303.1375	5
4	371.1925	186.0999	353.1819	177.0946	D	***552.2413***	276.6243	535.2147	268.1110	534.2307	267.6190	4
5	486.2195	243.6134	468.2089	234.6081	D	***437.2143***	219.1108	420.1878	210.5975	419.2037	210.1055	3
6	633.2879	317.1476	615.2773	308.1423	F	***322.1874***	161.5973	305.1608	153.0840			2

7					R	*175.1190*	88.0631	158.0924	79.5498			1

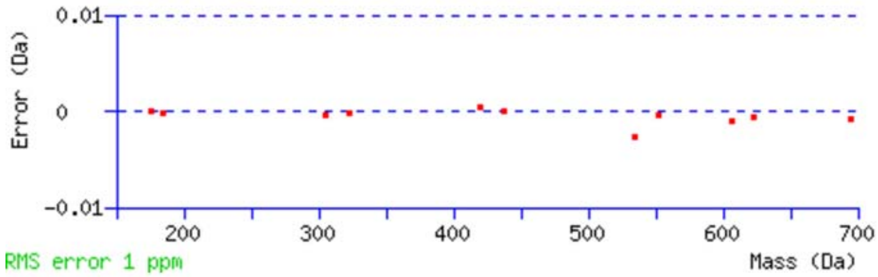

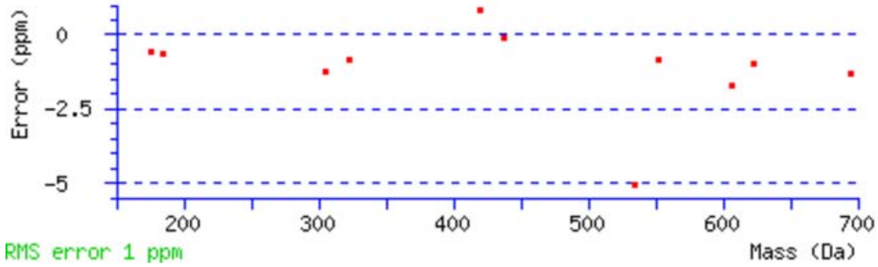

NCBI **BLAST** search of LAADDFR
(Parameters: blastp, nr protein database, expect=20000, no filter, PAM30)
Other BLAST web gateways

All matches to this query

Score	Mr(calc)	Delta	Sequence
54.8	806.3923	0.0002	LAADDFR
19.5	804.3878	2.0046	RPDNFR
14.4	806.3844	0.0081	LAAQMQK
7.8	806.3922	0.0002	GELANFR
7.8	804.3878	2.0046	NRPDFR
7.8	806.3923	0.0002	QLEDFR
7.6	806.3923	0.0002	IAGFDER
6.7	806.3923	0.0002	LDQQFR
6.7	806.3923	0.0002	LDQQFR
5.9	804.3912	2.0012	LASQACR

Mascot: http://www.matrixscience.com/

Mascot Search Results

Peptide View

MS/MS Fragmentation of **LAADDFR**
Found in **ch17u_O76014|KRT37_HUMAN** in **uni_human**, Keratin, type I cuticular Ha7 OS=Homo sapiens GN=KRT37 PE=3 SV=3

Match to Query 548: 806.392488 from(404.203520,2+) intensity(73705648.0000) rtinseconds(1321) scans(6774) index(4845)
Title: 160219_Sunil_SDSI_A_Spectrum056953_scans__6774_RTINSECONDS=1321
Data file L:\\QE_2016\\160219_Sunil_KAP_LKC\\TMgf\\T\\T160219_Sunil_SDSI_A.mgf

Click mouse within plot area to zoom in by factor of two about that point
Or, Plot from 50 to 700 Da Full range
Label all possible matches ○ Label matches used for scoring ◉
Show Y-axis ☐

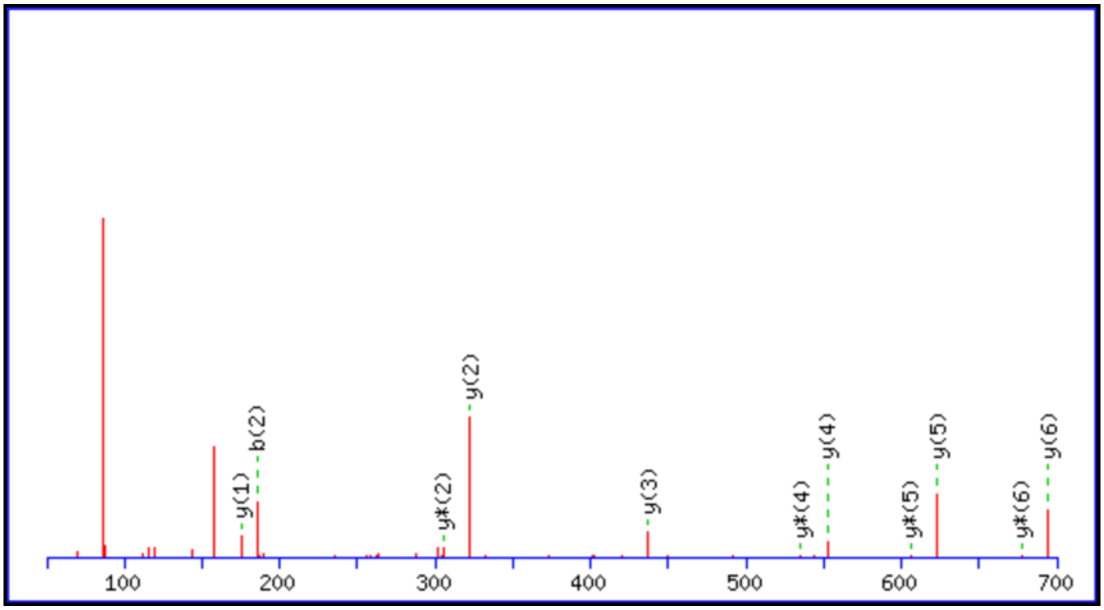

Monoisotopic mass of neutral peptide Mr(calc): 806.3923
Fixed modifications: Carbamidomethyl (C) (apply to specified residues or termini only)
Ions Score: 55 **Expect:** 0.00036
Matches : 11/50 fragment ions using 14 most intense peaks (help)

#	b	b^{++}	b^0	b^{0++}	Seq.	y	y^{++}	y*	y^{*++}	y^0	y^{0++}	#
1	114.0913	57.5493			L							7
2	**185.1285**	93.0679			A	***694.3155***	347.6614	677.2889	339.1481	676.3049	338.6561	6
3	256.1656	128.5864			A	***623.2784***	312.1428	606.2518	303.6295	605.2678	303.1375	5
4	371.1925	186.0999	353.1819	177.0946	D	***552.2413***	276.6243	535.2147	268.1110	534.2307	267.6190	4
5	486.2195	243.6134	468.2089	234.6081	D	***437.2143***	219.1108	420.1878	210.5975	419.2037	210.1055	3
6	633.2879	317.1476	615.2773	308.1423	F	***322.1874***	161.5973	305.1608	153.0840			2

7					R	*175.1190*	88.0631	158.0924	79.5498			1

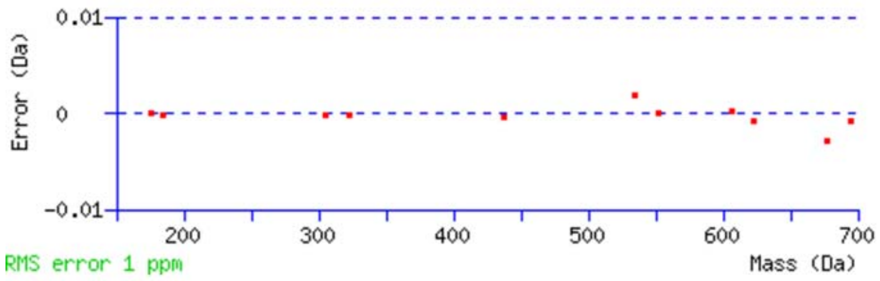

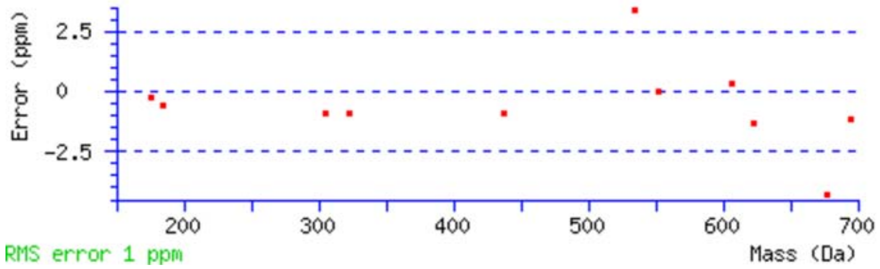

NCBI **BLAST** search of LAADDFR
(Parameters: blastp, nr protein database, expect=20000, no filter, PAM30)
Other BLAST web gateways

All matches to this query

Score	Mr(calc)	Delta	Sequence
54.8	806.3923	0.0002	LAADDFR
17.6	804.3878	2.0047	RPDNFR
9.1	806.3923	0.0002	IAGFDER
8.3	806.3923	0.0002	LDQQFR
8.3	806.3923	0.0002	LDQQFR
6.4	806.3922	0.0002	GELANFR
6.4	804.3878	2.0047	NRPDFR
6.4	806.3923	0.0002	QLEDFR
3.8	806.3891	0.0034	IACCRK
3.8	806.3956	-0.0031	LACTNTK

Mascot: http://www.matrixscience.com/

Mascot Search Results

Peptide View

MS/MS Fragmentation of **LAADDFR**
Found in **ch17u_O76014|KRT37_HUMAN** in **uni_human**, Keratin, type I cuticular Ha7 OS=Homo sapiens GN=KRT37 PE=3 SV=3

Match to Query 549: 806.392488 from(404.203520,2+) intensity(10192460.0000) rtinseconds(917) scans(4582) index(18351)
Title: 160219_Sunil_SDSI_A_Spectrum071655_scans__4582_RTINSECONDS=917
Data file L:\\QE_2016\\160219_Sunil_KAP_LKC\\TMgf\\T\\T160219_Sunil_SDSI_A.mgf

Click mouse within plot area to zoom in by factor of two about that point
Or, Plot from 50 to 750 Da Full range
Label all possible matches ○ Label matches used for scoring ◉
Show Y-axis ☐

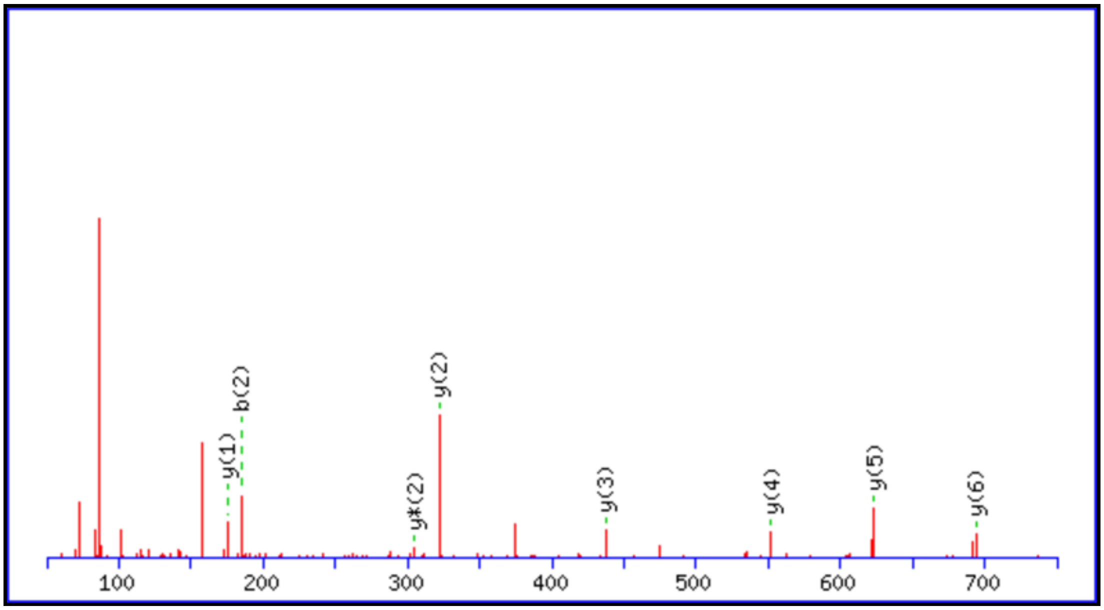

Monoisotopic mass of neutral peptide Mr(calc): 806.3923
Fixed modifications: Carbamidomethyl (C) (apply to specified residues or termini only)
Ions Score: 45 **Expect:** 0.0035
Matches : 8/50 fragment ions using 14 most intense peaks (help)

#	b	b^{++}	b^0	b^{0++}	Seq.	y	y^{++}	y*	y^{*++}	y^0	y^{0++}	#
1	114.0913	57.5493			L							7
2	**185.1285**	93.0679			A	***694.3155***	347.6614	677.2889	339.1481	676.3049	338.6561	6
3	256.1656	128.5864			A	***623.2784***	312.1428	606.2518	303.6295	605.2678	303.1375	5
4	371.1925	186.0999	353.1819	177.0946	D	***552.2413***	276.6243	535.2147	268.1110	534.2307	267.6190	4
5	486.2195	243.6134	468.2089	234.6081	D	***437.2143***	219.1108	420.1878	210.5975	419.2037	210.1055	3
6	633.2879	317.1476	615.2773	308.1423	F	***322.1874***	161.5973	305.1608	153.0840			2

7					R	*175.1190*	88.0631	158.0924	79.5498			1

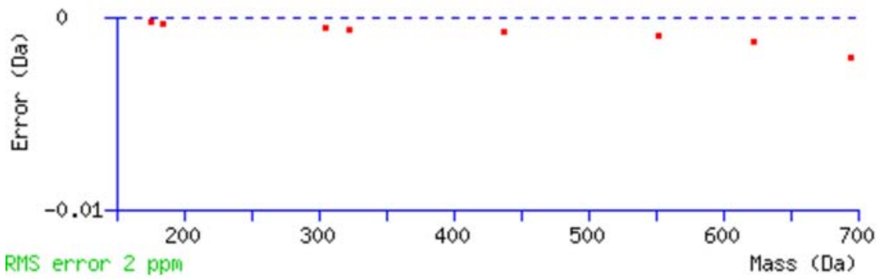

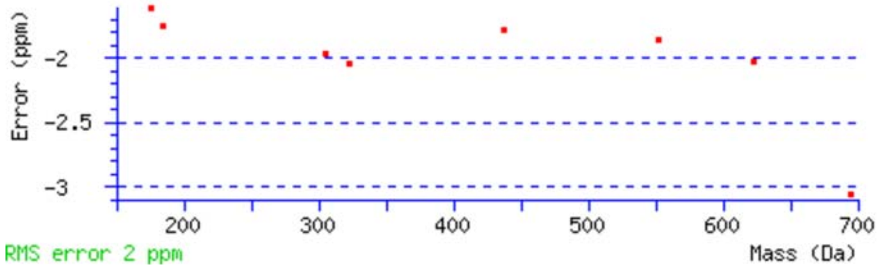

NCBI **BLAST** search of LAADDFR
(Parameters: blastp, nr protein database, expect=20000, no filter, PAM30)
Other BLAST web gateways

All matches to this query

Score	Mr(calc)	Delta	Sequence
44.9	806.3923	0.0002	LAADDFR
15.1	804.3878	2.0047	RPDNFR
5.8	806.3922	0.0002	GELANFR
5.8	804.3878	2.0047	NRPDFR
5.8	806.3923	0.0002	QLEDFR
5.7	806.3923	0.0002	LDQQFR
5.7	806.3923	0.0002	LDQQFR
4.7	806.3923	0.0002	IAGFDER
4.0	804.3912	2.0013	LASQACR
3.4	806.3956	-0.0031	LGDMSLR

Mascot: http://www.matrixscience.com/

Mascot Search Results

Peptide View

MS/MS Fragmentation of **LAADDFR**
Found in **ch17u_O76014|KRT37_HUMAN** in **uni_human**, Keratin, type I cuticular Ha7 OS=Homo sapiens GN=KRT37 PE=3 SV=3

Match to Query 554: 806.392668 from(404.203610,2+) intensity(10161025.0000) rtinseconds(1353) scans(6965) index(5011)
Title: 160219_Sunil_SDSI_A_Spectrum057119_scans__6965_RTINSECONDS=1353
Data file L:\\QE_2016\\160219_Sunil_KAP_LKC\\TMgf\\T\\T160219_Sunil_SDSI_A.mgf

Click mouse within plot area to zoom in by factor of two about that point
Or, Plot from 50 to 700 Da Full range
Label all possible matches ○ Label matches used for scoring ◉
Show Y-axis ☐

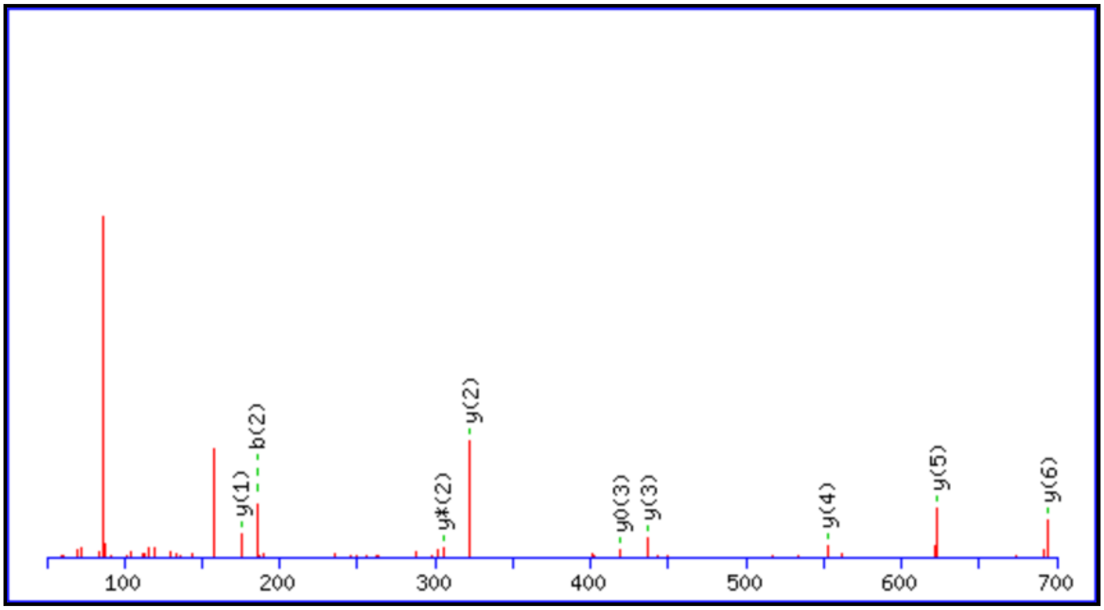

Monoisotopic mass of neutral peptide Mr(calc): 806.3923
Fixed modifications: Carbamidomethyl (C) (apply to specified residues or termini only)
Ions Score: 48 **Expect:** 0.0016
Matches : 9/50 fragment ions using 14 most intense peaks (help)

#	b	b^{++}	b^0	b^{0++}	Seq.	y	y^{++}	y*	y^{*++}	y^0	y^{0++}	#
1	114.0913	57.5493			L							7
2	**185.1285**	93.0679			A	***694.3155***	347.6614	677.2889	339.1481	676.3049	338.6561	6
3	256.1656	128.5864			A	***623.2784***	312.1428	606.2518	303.6295	605.2678	303.1375	5
4	371.1925	186.0999	353.1819	177.0946	D	***552.2413***	276.6243	535.2147	268.1110	534.2307	267.6190	4
5	486.2195	243.6134	468.2089	234.6081	D	***437.2143***	219.1108	420.1878	210.5975	419.2037	210.1055	3
6	633.2879	317.1476	615.2773	308.1423	F	***322.1874***	161.5973	305.1608	153.0840			2

7					R	175.1190	88.0631	158.0924	79.5498			1

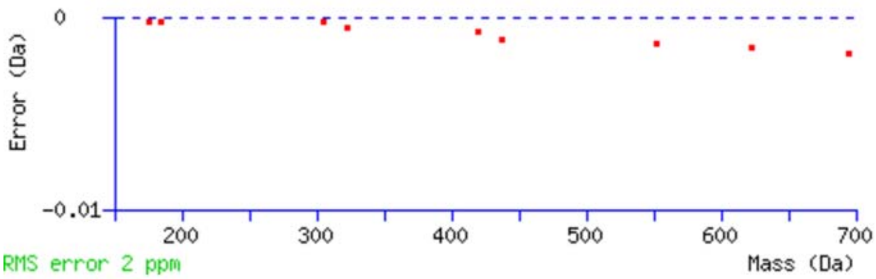

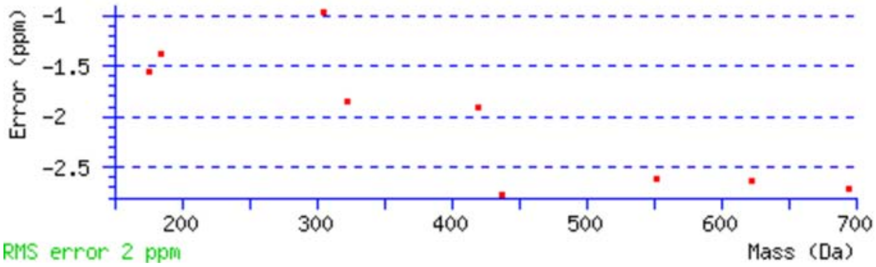

NCBI **BLAST** search of LAADDFR
(Parameters: blastp, nr protein database, expect=20000, no filter, PAM30)
Other BLAST web gateways

All matches to this query

Score	Mr(calc)	Delta	Sequence
48.2	806.3923	0.0004	LAADDFR
14.9	804.3878	2.0048	RPDNFR
14.6	806.3923	0.0004	IAGFDER
9.2	804.3912	2.0015	LASQACR
7.4	804.3878	2.0048	NRPDFR
7.4	806.3923	0.0004	QLEDFR
6.2	806.3923	0.0004	LDQQFR
6.2	806.3923	0.0004	LDQQFR
5.8	806.3922	0.0004	GELANFR
3.2	806.3891	0.0036	IACCRK

Mascot: http://www.matrixscience.com/

Mascot Search Results

Peptide View

MS/MS Fragmentation of **LAADDFR**
Found in **ch17u_O76014|KRT37_HUMAN** in **uni_human**, Keratin, type I cuticular Ha7 OS=Homo sapiens GN=KRT37 PE=3 SV=3

Match to Query 557: 806.392728 from(404.203640,2+) intensity(1488045568.0000) rtinseconds(1154) scans(5958) index(19494)
Title: 160219_Sunil_SDSI_A_Spectrum072798_scans__5958_RTINSECONDS=1154
Data file L:\\QE_2016\\160219_Sunil_KAP_LKC\\TMgf\\T\\T160219_Sunil_SDSI_A.mgf

Click mouse within plot area to zoom in by factor of two about that point
Or, Plot from 50 to 750 Da Full range
Label all possible matches ○ Label matches used for scoring ◉
Show Y-axis ☐

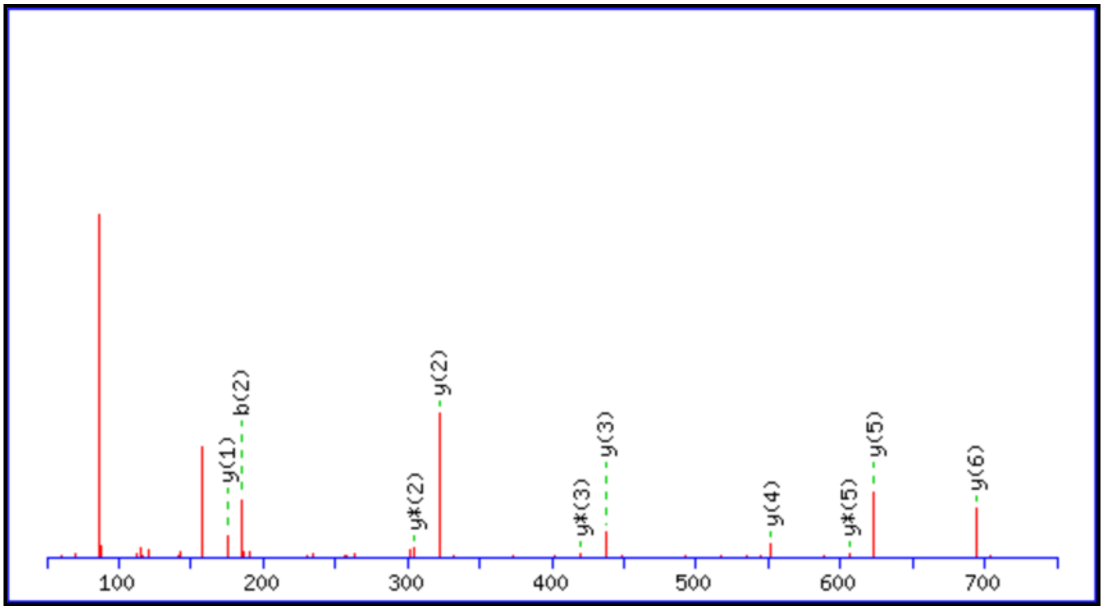

Monoisotopic mass of neutral peptide Mr(calc): 806.3923
Fixed modifications: Carbamidomethyl (C) (apply to specified residues or termini only)
Ions Score: 52 **Expect:** 0.00072
Matches : 10/50 fragment ions using 14 most intense peaks (help)

#	b	b^{++}	b^{0}	b^{0++}	Seq.	y	y^{++}	y^{*}	y^{*++}	y^{0}	y^{0++}	#
1	114.0913	57.5493			L							7
2	**185.1285**	93.0679			A	***694.3155***	347.6614	677.2889	339.1481	676.3049	338.6561	6
3	256.1656	128.5864			A	***623.2784***	312.1428	606.2518	303.6295	605.2678	303.1375	5
4	371.1925	186.0999	353.1819	177.0946	D	***552.2413***	276.6243	535.2147	268.1110	534.2307	267.6190	4
5	486.2195	243.6134	468.2089	234.6081	D	***437.2143***	219.1108	420.1878	210.5975	419.2037	210.1055	3
6	633.2879	317.1476	615.2773	308.1423	F	***322.1874***	161.5973	305.1608	153.0840			2

7					R	175.1190	88.0631	158.0924	79.5498			1

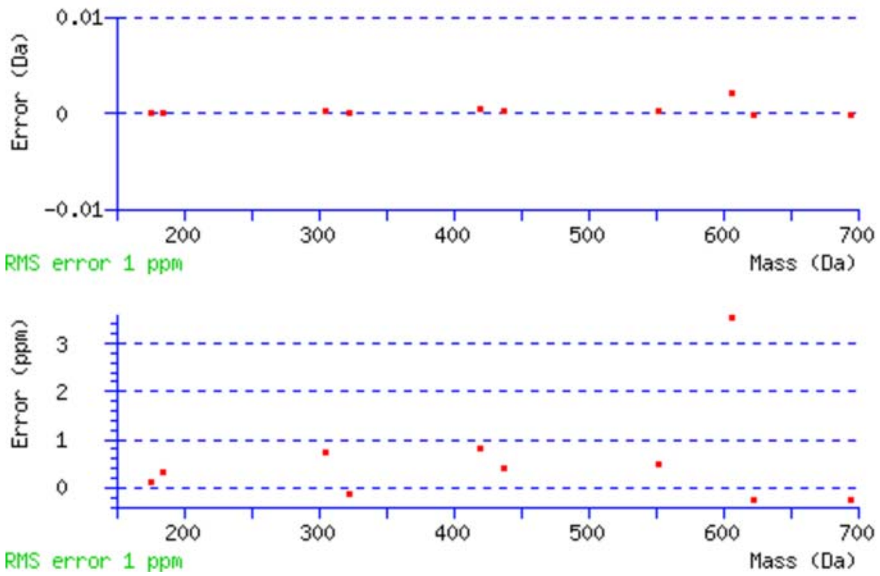

NCBI **BLAST** search of LAADDFR
(Parameters: blastp, nr protein database, expect=20000, no filter, PAM30)
Other BLAST web gateways

All matches to this query

Score	Mr(calc)	Delta	Sequence
51.8	806.3923	0.0005	LAADDFR
17.6	804.3878	2.0049	RPDNFR
7.9	806.3922	0.0005	GELANFR
7.9	804.3878	2.0049	NRPDFR
7.9	806.3923	0.0005	QLEDFR
7.8	806.3923	0.0005	IAGFDER
6.9	806.3923	0.0005	LDQQFR
6.9	806.3923	0.0005	LDQQFR
4.0	806.3891	0.0036	IACCRK
4.0	806.3956	-0.0029	LACTNTK

Mascot: http://www.matrixscience.com/

Mascot Search Results

Peptide View

MS/MS Fragmentation of **LAADDFR**
Found in **ch17u_O76014|KRT37_HUMAN** in **uni_human**, Keratin, type I cuticular Ha7 OS=Homo sapiens GN=KRT37 PE=3 SV=3

Match to Query 560: 806.392928 from(404.203740,2+) intensity(570921.4375) rtinseconds(1611) scans(8620) index(21833)
Title: 160219_Sunil_SDSI_A_Spectrum075137_scans__8620_RTINSECONDS=1611
Data file L:\\QE_2016\\160219_Sunil_KAP_LKC\\TMgf\\T\\T160219_Sunil_SDSI_A.mgf

Click mouse within plot area to zoom in by factor of two about that point
Or, Plot from 50 to 700 Da Full range
Label all possible matches ○ Label matches used for scoring ◉
Show Y-axis ☐

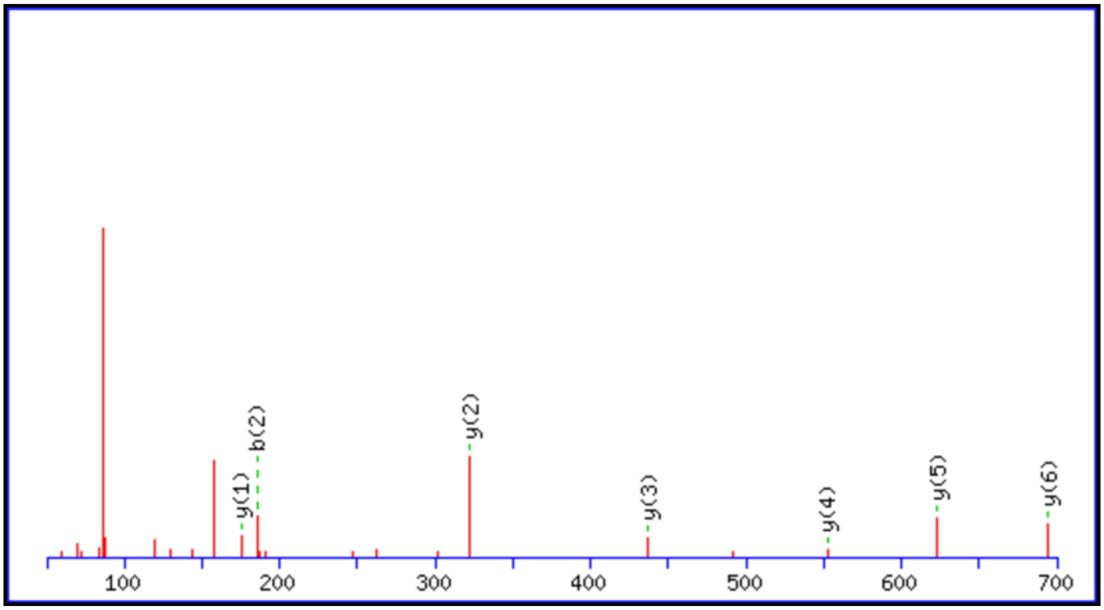

Monoisotopic mass of neutral peptide Mr(calc): 806.3923
Fixed modifications: Carbamidomethyl (C) (apply to specified residues or termini only)
Ions Score: 49 **Expect:** 0.0013
Matches : 7/50 fragment ions using 11 most intense peaks (help)

#	b	b^{++}	b^{0}	b^{0++}	Seq.	y	y^{++}	y*	y^{*++}	y^{0}	y^{0++}	#
1	114.0913	57.5493			L							7
2	**185.1285**	93.0679			A	***694.3155***	347.6614	677.2889	339.1481	676.3049	338.6561	6
3	256.1656	128.5864			A	***623.2784***	312.1428	606.2518	303.6295	605.2678	303.1375	5
4	371.1925	186.0999	353.1819	177.0946	D	***552.2413***	276.6243	535.2147	268.1110	534.2307	267.6190	4
5	486.2195	243.6134	468.2089	234.6081	D	***437.2143***	219.1108	420.1878	210.5975	419.2037	210.1055	3
6	633.2879	317.1476	615.2773	308.1423	F	***322.1874***	161.5973	305.1608	153.0840			2

7					R	*175.1190*	88.0631	158.0924	79.5498			1

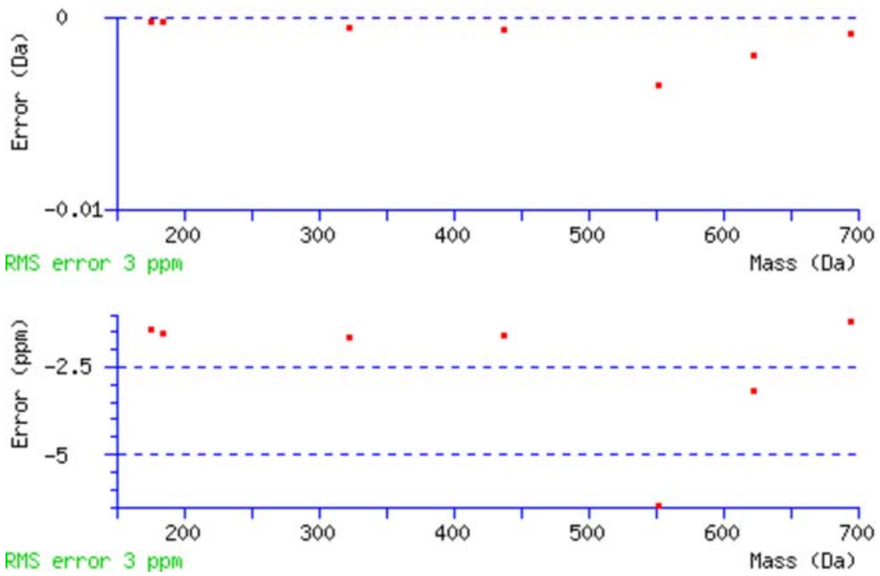

NCBI **BLAST** search of LAADDFR
(Parameters: blastp, nr protein database, expect=20000, no filter, PAM30)
Other BLAST web gateways

All matches to this query

Score	Mr(calc)	Delta	Sequence
49.3	806.3923	0.0007	LAADDFR
16.2	804.3878	2.0051	RPDNFR
7.9	806.3923	0.0007	IAGFDER
6.7	806.3923	0.0007	LDQQFR
6.7	806.3923	0.0007	LDQQFR
6.3	806.3922	0.0007	GELANFR
6.3	804.3878	2.0051	NRPDFR
6.3	806.3923	0.0007	QLEDFR
2.8	806.3891	0.0038	IACCRK
2.8	806.3956	-0.0027	LACTNTK

Mascot: http://www.matrixscience.com/

Mascot Search Results

Peptide View

MS/MS Fragmentation of **LAADDFR**
Found in **ch17u_O76014|KRT37_HUMAN** in **uni_human**, Keratin, type I cuticular Ha7 OS=Homo sapiens GN=KRT37 PE=3 SV=3

Match to Query 561: 806.393408 from(404.203980,2+) intensity(710469.8125) rtinseconds(1542) scans(8219) index(21478)
Title: 160219_Sunil_SDSI_A_Spectrum074782_scans__8219_RTINSECONDS=1542
Data file L:\\QE_2016\\160219_Sunil_KAP_LKC\\TMgf\\T\\T160219_Sunil_SDSI_A.mgf

Click mouse within plot area to zoom in by factor of two about that point
Or, Plot from 50 to 700 Da Full range
Label all possible matches ○ Label matches used for scoring ◉
Show Y-axis ☐

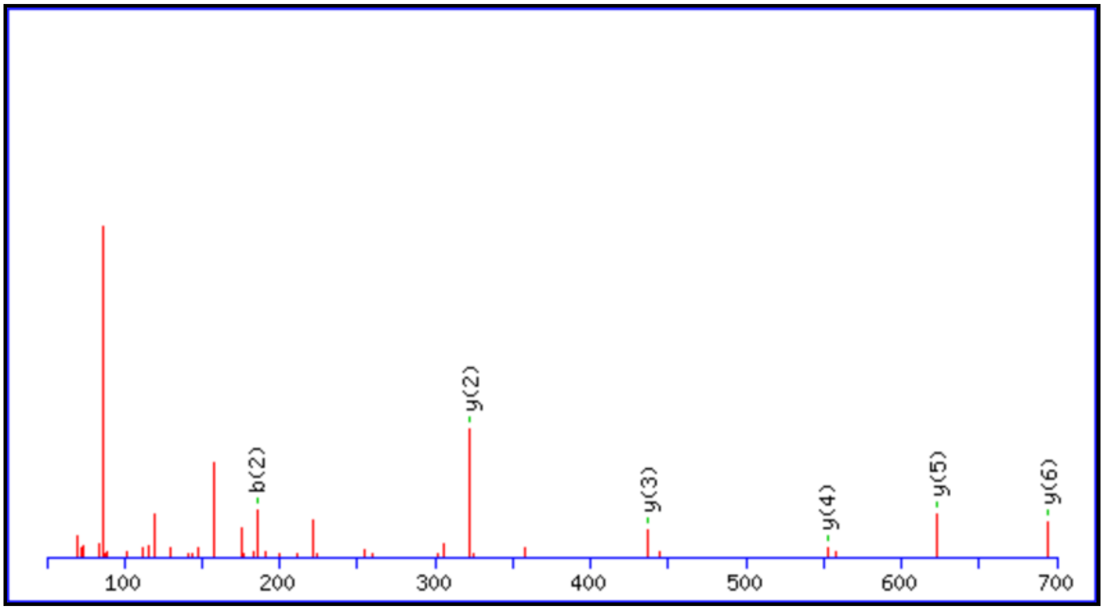

Monoisotopic mass of neutral peptide Mr(calc): 806.3923
Fixed modifications: Carbamidomethyl (C) (apply to specified residues or termini only)
Ions Score: 46 **Expect:** 0.0025
Matches : 6/50 fragment ions using 7 most intense peaks (help)

#	b	b^{++}	b^{0}	b^{0++}	Seq.	y	y^{++}	y*	y^{*++}	y^{0}	y^{0++}	#
1	114.0913	57.5493			L							7
2	**185.1285**	93.0679			A	***694.3155***	347.6614	677.2889	339.1481	676.3049	338.6561	6
3	256.1656	128.5864			A	***623.2784***	312.1428	606.2518	303.6295	605.2678	303.1375	5
4	371.1925	186.0999	353.1819	177.0946	D	***552.2413***	276.6243	535.2147	268.1110	534.2307	267.6190	4
5	486.2195	243.6134	468.2089	234.6081	D	***437.2143***	219.1108	420.1878	210.5975	419.2037	210.1055	3
6	633.2879	317.1476	615.2773	308.1423	F	***322.1874***	161.5973	305.1608	153.0840			2

**7**					**R**	175.1190	88.0631	158.0924	79.5498			**1**

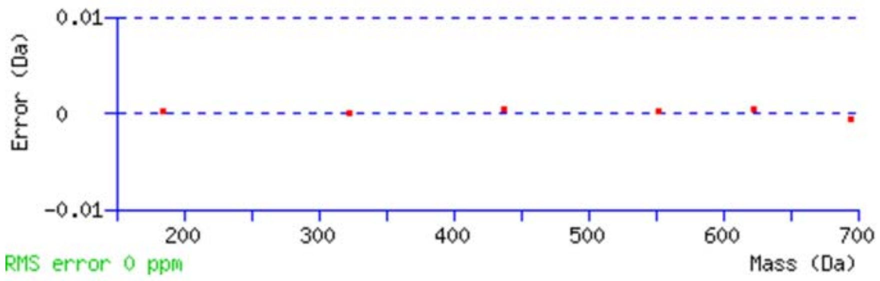

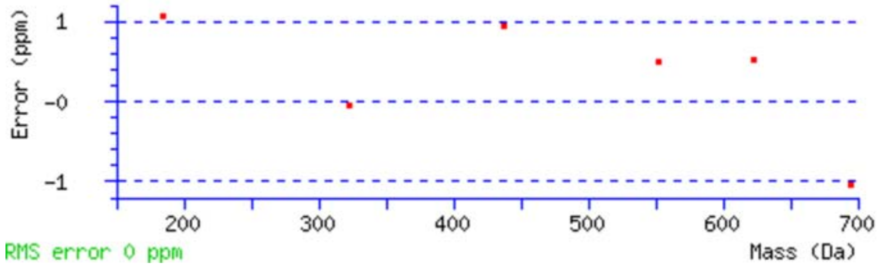

NCBI **BLAST** search of LAADDFR
(Parameters: blastp, nr protein database, expect=20000, no filter, PAM30)
Other BLAST web gateways

All matches to this query

Score	Mr(calc)	Delta	Sequence
46.0	806.3923	0.0012	LAADDFR
14.1	804.3878	2.0056	RPDNFR
5.6	806.3923	0.0012	LDQQFR
5.6	806.3923	0.0012	LDQQFR
5.4	806.3922	0.0012	GELANFR
5.4	804.3878	2.0056	NRPDFR
5.4	806.3923	0.0012	QLEDFR
4.3	806.3923	0.0012	IAGFDER
2.3	806.3891	0.0043	IACCRK
2.3	806.3956	-0.0022	LACTNTK

Mascot: http://www.matrixscience.com/

Mascot Search Results

Peptide View

MS/MS Fragmentation of **LAADDFR**
Found in **ch17u_O76014|KRT37_HUMAN** in **uni_human**, Keratin, type I cuticular Ha7 OS=Homo sapiens GN=KRT37 PE=3 SV=3

Match to Query 561: 806.393408 from(404.203980,2+) intensity(710469.8125) rtinseconds(1542) scans(8219) index(21478)
Title: 160219_Sunil_SDSI_A_Spectrum074782_scans__8219_RTINSECONDS=1542
Data file L:\\QE_2016\\160219_Sunil_KAP_LKC\\TMgf\\T\\T160219_Sunil_SDSI_A.mgf

Click mouse within plot area to zoom in by factor of two about that point
Or, Plot from 50 to 700 Da Full range
Label all possible matches ○ Label matches used for scoring ◉
Show Y-axis ☐

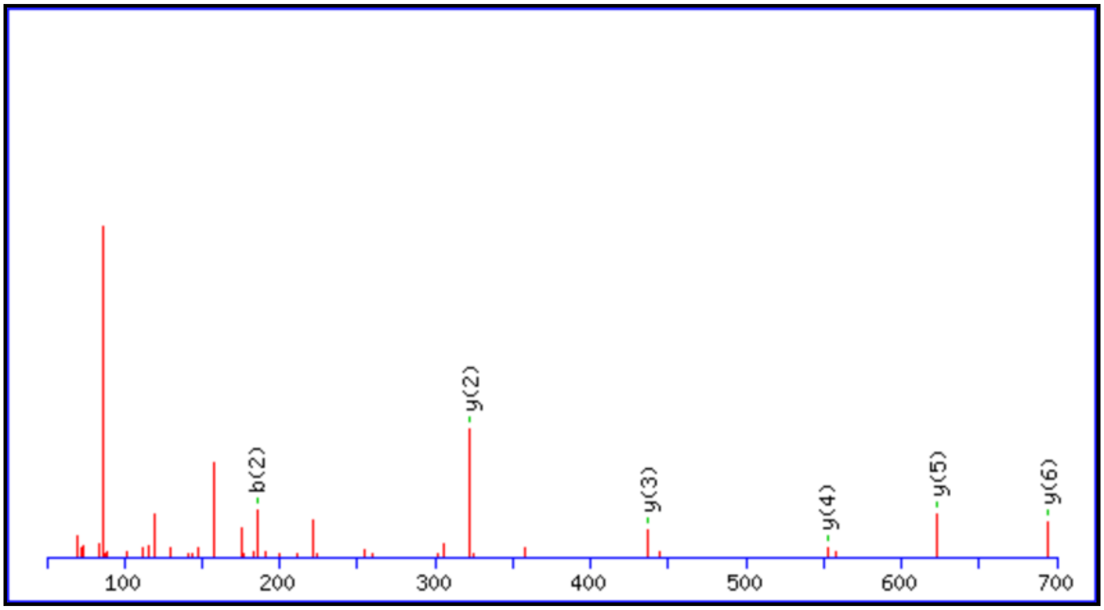

Monoisotopic mass of neutral peptide Mr(calc): 806.3923
Fixed modifications: Carbamidomethyl (C) (apply to specified residues or termini only)
Ions Score: 46 **Expect:** 0.0025
Matches : 6/50 fragment ions using 7 most intense peaks (help)

#	b	b^{++}	b^0	b^{0++}	Seq.	y	y^{++}	y^*	y^{*++}	y^0	y^{0++}	#
1	114.0913	57.5493			L							7
2	**185.1285**	93.0679			A	***694.3155***	347.6614	677.2889	339.1481	676.3049	338.6561	6
3	256.1656	128.5864			A	***623.2784***	312.1428	606.2518	303.6295	605.2678	303.1375	5
4	371.1925	186.0999	353.1819	177.0946	D	***552.2413***	276.6243	535.2147	268.1110	534.2307	267.6190	4
5	486.2195	243.6134	468.2089	234.6081	D	***437.2143***	219.1108	420.1878	210.5975	419.2037	210.1055	3
6	633.2879	317.1476	615.2773	308.1423	F	***322.1874***	161.5973	305.1608	153.0840			2

**7**					**R**	175.1190	88.0631	158.0924	79.5498			**1**

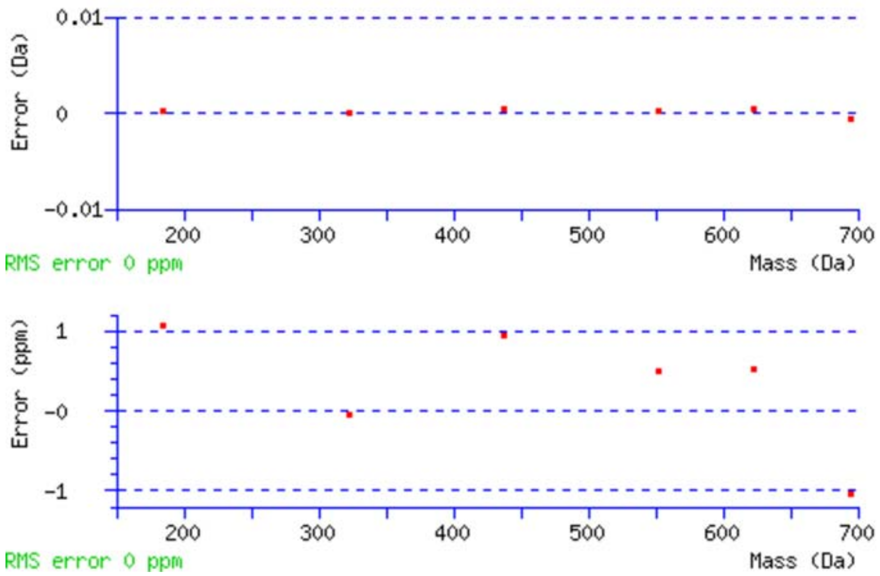

NCBI **BLAST** search of LAADDFR
(Parameters: blastp, nr protein database, expect=20000, no filter, PAM30)
Other BLAST web gateways

All matches to this query

Score	Mr(calc)	Delta	Sequence
46.0	806.3923	0.0012	LAADDFR
14.1	804.3878	2.0056	RPDNFR
5.6	806.3923	0.0012	LDQQFR
5.6	806.3923	0.0012	LDQQFR
5.4	806.3922	0.0012	GELANFR
5.4	804.3878	2.0056	NRPDFR
5.4	806.3923	0.0012	QLEDFR
4.3	806.3923	0.0012	IAGFDER
2.3	806.3891	0.0043	IACCRK
2.3	806.3956	-0.0022	LACTNTK

Mascot: http://www.matrixscience.com/

Mascot Search Results

Peptide View

MS/MS Fragmentation of **LLDDATLAK**
Found in **ch17u_O76014|KRT37_HUMAN** in **uni_human**, Keratin, type I cuticular Ha7 OS=Homo sapiens GN=KRT37 PE=3 SV=3

Match to Query 2139: 958.534088 from(480.274320,2+) intensity(5881803.5000) rtinseconds(1607) scans(8445) index(6310)
Title: 160219_Sunil_SDSI_A_Spectrum058418_scans__8445_RTINSECONDS=1607
Data file L:\\QE_2016\\160219_Sunil_KAP_LKC\\TMgf\\T\\T160219_Sunil_SDSI_A.mgf

Click mouse within plot area to zoom in by factor of two about that point
Or, Plot from 50 to 850 Da Full range
Label all possible matches ○ Label matches used for scoring ◉
Show Y-axis ☐

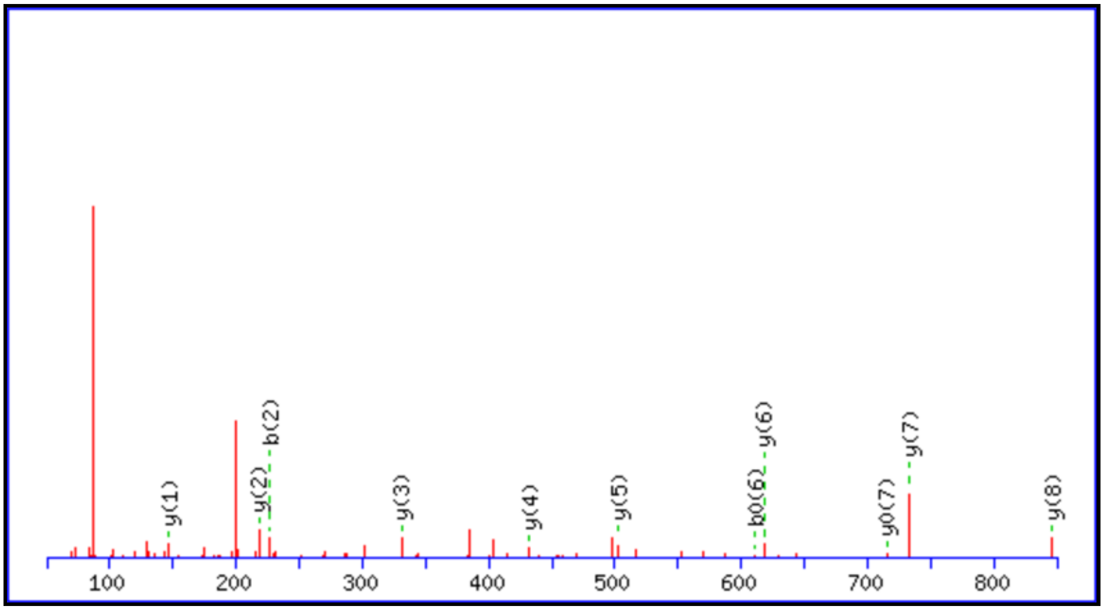

Monoisotopic mass of neutral peptide Mr(calc): 958.5335
Fixed modifications: Carbamidomethyl (C) (apply to specified residues or termini only)
Ions Score: 50 **Expect:** 0.0014
Matches : 11/70 fragment ions using 27 most intense peaks (help)

#	b	b^{++}	b^0	b^{0++}	Seq.	y	y^{++}	y*	y^{*++}	y^0	y^{0++}	#
1	114.0913	57.5493			L							9
2	**227.1754**	114.0913			L	***846.4567***	423.7320	829.4302	415.2187	828.4462	414.7267	8
3	342.2023	171.6048	324.1918	162.5995	D	***733.3727***	367.1900	716.3461	358.6767	715.3621	358.1847	7
4	457.2293	229.1183	439.2187	220.1130	D	***618.3457***	309.6765	601.3192	301.1632	600.3352	300.6712	6
5	528.2664	264.6368	510.2558	255.6316	A	***503.3188***	252.1630	486.2922	243.6498	485.3082	243.1577	5
6	629.3141	315.1607	611.3035	306.1554	T	***432.2817***	216.6445	415.2551	208.1312	414.2711	207.6392	4

7	742.3981	371.7027	724.3876	362.6974	L	331.2340	166.1206	314.2074	157.6074			3
8	813.4353	407.2213	795.4247	398.2160	A	218.1499	109.5786	201.1234	101.0653			2
9					K	147.1128	74.0600	130.0863	65.5468			1

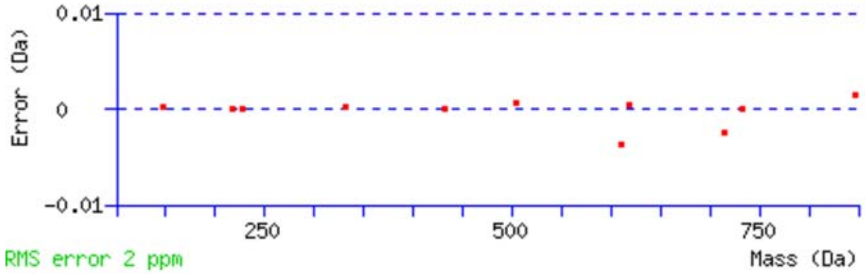

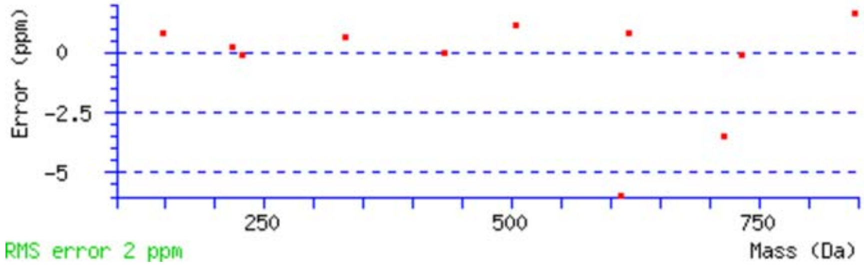

NCBI **BLAST** search of LLDDATLAK
(Parameters: blastp, nr protein database, expect=20000, no filter, PAM30)
Other BLAST web gateways

All matches to this query

Score	Mr(calc)	Delta	Sequence
49.8	958.5335	0.0006	LLDDATLAK

Mascot: http://www.matrixscience.com/

Mascot Search Results

Peptide View

MS/MS Fragmentation of **LAADDFR**
Found in **ch17u_O76014|KRT37_HUMAN** in **uni_human**, Keratin, type I cuticular Ha7 OS=Homo sapiens GN=KRT37 PE=3 SV=3

Match to Query 381: 806.392488 from(404.203520,2+) intensity(1165177.1250) rtinseconds(994) scans(5168) index(32479)
Title: 160219_Sunil_KAP_A1_Spectrum086736_scans__5168_RTINSECONDS=994
Data file L:\\QE_2016\\160219_Sunil_KAP_LKC\\TMgf\\T\\T160219_Sunil_KAP_A1.mgf

Click mouse within plot area to zoom in by factor of two about that point
Or, Plot from 50 to 750 Da Full range
Label all possible matches ○ Label matches used for scoring ◉
Show Y-axis ☐

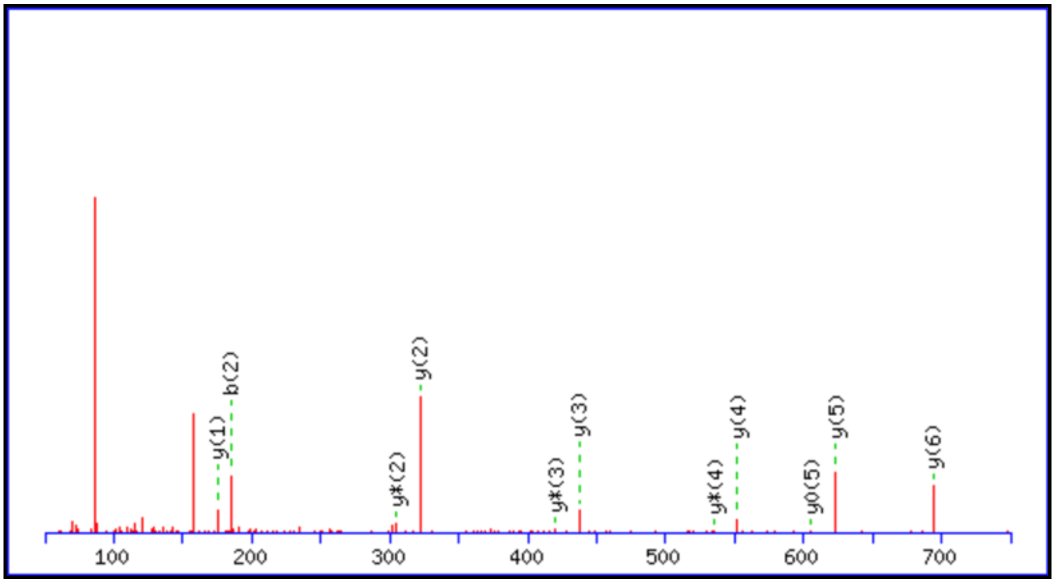

Monoisotopic mass of neutral peptide Mr(calc): 806.3923
Fixed modifications: Carbamidomethyl (C) (apply to specified residues or termini only)
Ions Score: 55 **Expect:** 0.00038
Matches : 11/50 fragment ions using 14 most intense peaks (help)

#	b	b^{++}	b^0	b^{0++}	Seq.	y	y^{++}	y*	y^{*++}	y^0	y^{0++}	#
1	114.0913	57.5493			L							7
2	**185.1285**	93.0679			A	***694.3155***	347.6614	677.2889	339.1481	676.3049	338.6561	6
3	256.1656	128.5864			A	***623.2784***	312.1428	606.2518	303.6295	605.2678	303.1375	5
4	371.1925	186.0999	353.1819	177.0946	D	***552.2413***	276.6243	535.2147	268.1110	534.2307	267.6190	4
5	486.2195	243.6134	468.2089	234.6081	D	***437.2143***	219.1108	420.1878	210.5975	419.2037	210.1055	3
6	633.2879	317.1476	615.2773	308.1423	F	***322.1874***	161.5973	305.1608	153.0840			2
7					R	***175.1190***	88.0631	158.0924	79.5498			1

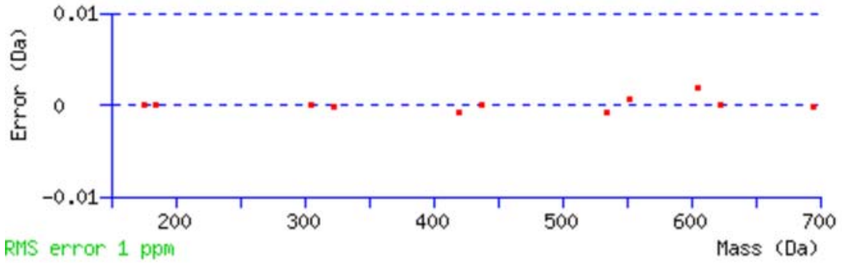

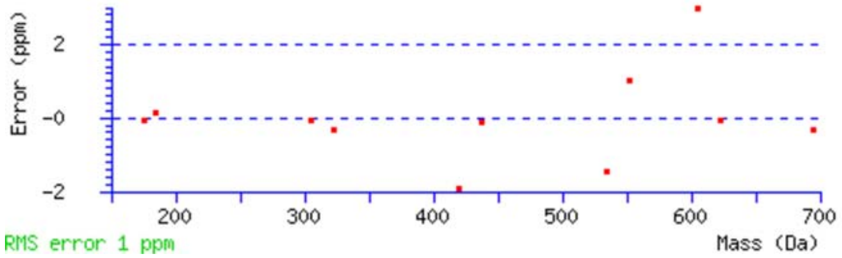

NCBI **BLAST** search of LAADDFR
(Parameters: blastp, nr protein database, expect=20000, no filter, PAM30)
Other BLAST web gateways

All matches to this query

Score	Mr(calc)	Delta	Sequence
54.6	806.3923	0.0002	LAADDFR
19.3	804.3878	2.0047	RPDNFR
7.5	806.3922	0.0002	GELANFR
7.5	804.3878	2.0047	NRPDFR
7.5	806.3923	0.0002	QLEDFR
7.5	806.3923	0.0002	IAGFDER
6.9	806.3923	0.0002	LDQQFR
6.9	806.3923	0.0002	LDQQFR
3.7	806.3891	0.0034	IACCRK
3.7	806.3956	-0.0031	LACTNTK

Mascot: http://www.matrixscience.com/

Mascot Search Results

Peptide View

MS/MS Fragmentation of **LLDDATLAK**
Found in **ch17u_O76014|KRT37_HUMAN** in **uni_human**, Keratin, type I cuticular Ha7 OS=Homo sapiens GN=KRT37 PE=3 SV=3

Match to Query 1814: 958.534588 from(480.274570,2+) intensity(298482.7188) rtinseconds(1324) scans(7127) index(19877)
Title: 160219_Sunil_KAP_A1_Spectrum072326_scans__7127_RTINSECONDS=1324
Data file L:\\QE_2016\\160219_Sunil_KAP_LKC\\TMgf\\T\\T160219_Sunil_KAP_A1.mgf

Click mouse within plot area to zoom in by factor of two about that point
Or, Plot from 50 to 850 Da Full range
Label all possible matches ○ Label matches used for scoring ◉
Show Y-axis ☐

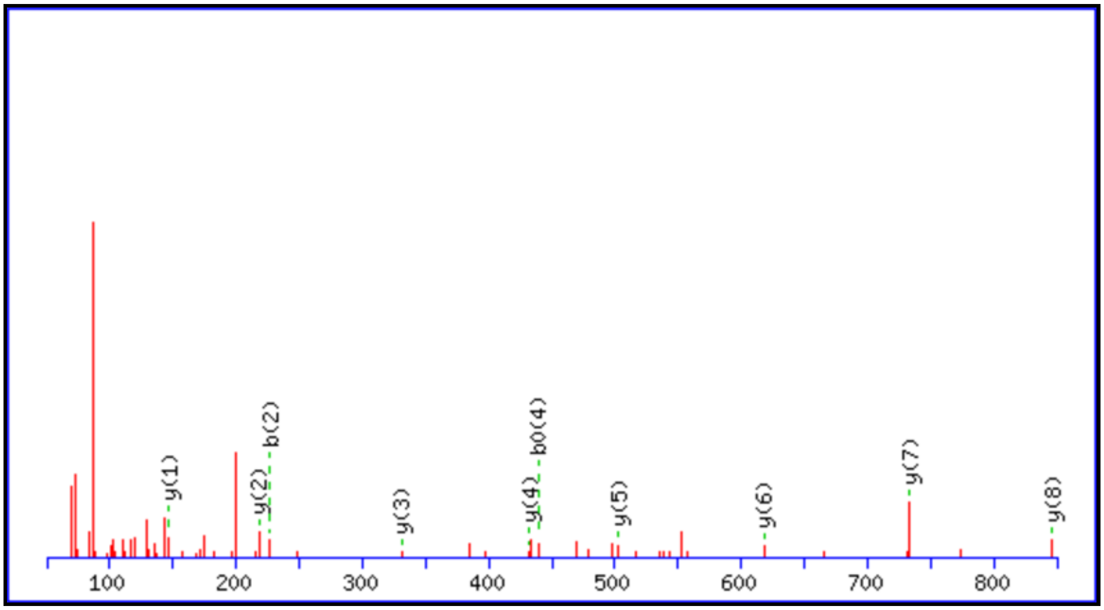

Monoisotopic mass of neutral peptide Mr(calc): 958.5335
Fixed modifications: Carbamidomethyl (C) (apply to specified residues or termini only)
Ions Score: 33 **Expect:** 0.057
Matches : 10/70 fragment ions using 39 most intense peaks (help)

#	b	b^{++}	b^{0}	b^{0++}	Seq.	y	y^{++}	y^{*}	y^{*++}	y^{0}	y^{0++}	#
1	114.0913	57.5493			L							9
2	**227.1754**	114.0913			L	***846.4567***	423.7320	829.4302	415.2187	828.4462	414.7267	8
3	342.2023	171.6048	324.1918	162.5995	D	***733.3727***	367.1900	716.3461	358.6767	715.3621	358.1847	7
4	457.2293	229.1183	439.2187	220.1130	D	***618.3457***	309.6765	601.3192	301.1632	600.3352	300.6712	6
5	528.2664	264.6368	510.2558	255.6316	A	***503.3188***	252.1630	486.2922	243.6498	485.3082	243.1577	5
6	629.3141	315.1607	611.3035	306.1554	T	***432.2817***	216.6445	415.2551	208.1312	414.2711	207.6392	4

7	742.3981	371.7027	724.3876	362.6974	L	331.2340	166.1206	314.2074	157.6074			3
8	813.4353	407.2213	795.4247	398.2160	A	218.1499	109.5786	201.1234	101.0653			2
9					K	147.1128	74.0600	130.0863	65.5468			1

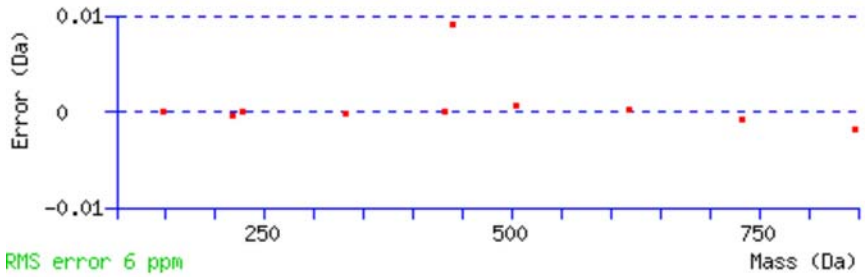

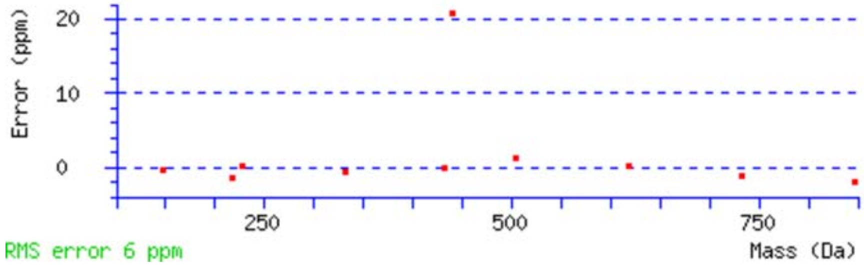

NCBI **BLAST** search of LLDDATLAK
(Parameters: blastp, nr protein database, expect=20000, no filter, PAM30)
Other BLAST web gateways

All matches to this query

Score	Mr(calc)	Delta	Sequence
33.5	958.5335	0.0011	LLDDATLAK

Mascot: http://www.matrixscience.com/

Mascot Search Results

Peptide View

MS/MS Fragmentation of **TIEELQQKILCSK**
Found in **ch17u_O76014|KRT37_HUMAN** in **uni_human**, Keratin, type I cuticular Ha7 OS=Homo sapiens GN=KRT37 PE=3 SV=3

Match to Query 17616: 1588.849692 from(530.623840,3+) intensity(633571.9375) rtinseconds(1895) scans(10405) index(22599)
Title: 160219_Sunil_KAP_A1_Spectrum075060_scans__10405_RTINSECONDS=1895
Data file L:\\QE_2016\\160219_Sunil_KAP_LKC\\TMgf\\T\\T160219_Sunil_KAP_A1.mgf

Click mouse within plot area to zoom in by factor of two about that point
Or, Plot from 100 to 1600 Da Full range
Label all possible matches ○ Label matches used for scoring ◉
Show Y-axis ☐

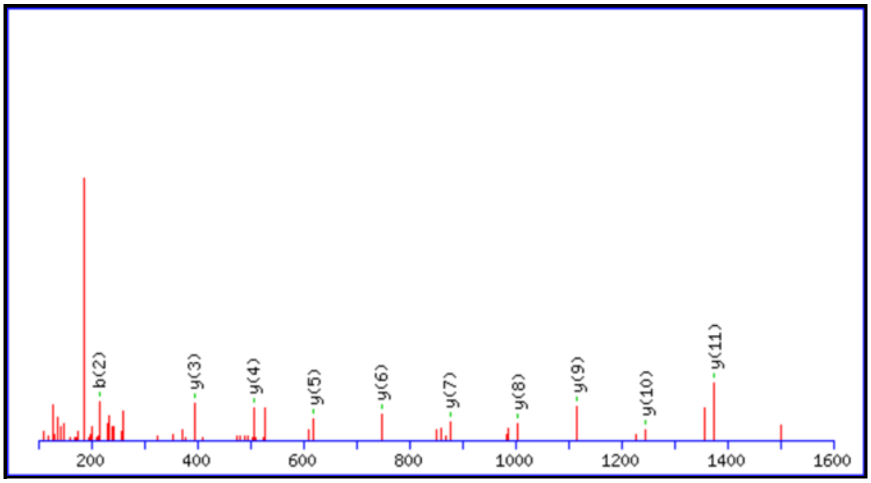

Monoisotopic mass of neutral peptide Mr(calc): 1588.8494
Fixed modifications: Carbamidomethyl (C) (apply to specified residues or termini only)
Ions Score: 78 **Expect:** 2.6e-006
Matches : 10/132 fragment ions using 13 most intense peaks (help)

#	b	b^{++}	b*	b^{*++}	b^0	b^{0++}	Seq.	y	y^{++}	y*	y^{*++}	y^0	y^{0++}	#
1	102.0550	51.5311			84.0444	42.5258	T							13
2	**215.1390**	108.0731			197.1285	99.0679	I	1488.8090	744.9082	1471.7825	736.3949	1470.7985	735.9029	12
3	344.1816	172.5944			326.1710	163.5892	E	***1375.7250***	688.3661	1358.6984	679.8529	1357.7144	679.3608	11
4	473.2242	237.1157			455.2136	228.1105	E	***1246.6824***	623.8448	1229.6558	615.3316	1228.6718	614.8395	10
5	586.3083	293.6578			568.2977	284.6525	L	***1117.6398***	559.3235	1100.6132	550.8103	1099.6292	550.3183	9
6	714.3668	357.6871	697.3403	349.1738	696.3563	348.6818	Q	***1004.5557***	502.7815	987.5292	494.2682	986.5452	493.7762	8
7	842.4254	421.7164	825.3989	413.2031	824.4149	412.7111	Q	***876.4972***	438.7522	859.4706	430.2389	858.4866	429.7469	7
8	970.5204	485.7638	953.4938	477.2506	952.5098	476.7585	K	***748.4386***	374.7229	731.4120	366.2096	730.4280	365.7176	6
9	1083.6045	542.3059	1066.5779	533.7926	1065.5939	533.3006	I	***620.3436***	310.6754	603.3171	302.1622	602.3330	301.6702	5
10	1196.6885	598.8479	1179.6620	590.3346	1178.6780	589.8426	L	***507.2595***	254.1334	490.2330	245.6201	489.2490	245.1281	4
11	1356.7192	678.8632	1339.6926	670.3499	1338.7086	669.8579	C	***394.1755***	197.5914	377.1489	189.0781	376.1649	188.5861	3
12	1443.7512	722.3792	1426.7246	713.8660	1425.7406	713.3740	S	234.1448	117.5761	217.1183	109.0628	216.1343	108.5708	2
13							K	147.1128	74.0600	130.0863	65.5468			1

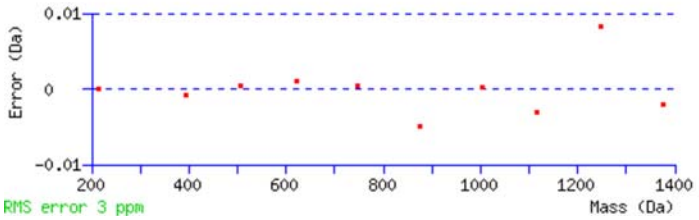

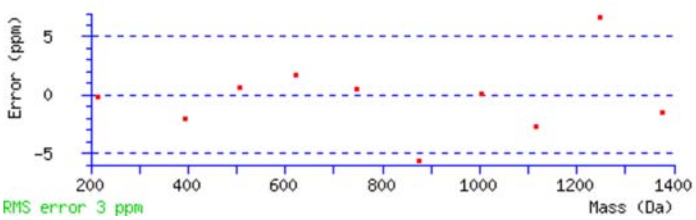

NCBI **BLAST** search of TIEELQQKILCSK
(Parameters: blastp, nr protein database, expect=20000, no filter, PAM30)
Other BLAST web gateways

All matches to this query

Score	Mr(calc)	Delta	Sequence
77.6	1588.8494	0.0003	TIEELQQKILCSK

Mascot: http://www.matrixscience.com/

Mascot Search Results

Peptide View

MS/MS Fragmentation of **LAADDFR**
Found in **ch17u_O76014|KRT37_HUMAN** in **uni_human**, Keratin, type I cuticular Ha7 OS=Homo sapiens GN=KRT37 PE=3 SV=3

Match to Query 412: 806.391948 from(404.203250,2+) intensity(445841.9063) rtinseconds(1349) scans(6881) index(19179)
Title: 160219_Sunil_KAP_B_Spectrum069044_scans__6881_RTINSECONDS=1349
Data file L:\\QE_2016\\160219_Sunil_KAP_LKC\\TMgf\\T\\T160219_Sunil_KAP_B.mgf

Click mouse within plot area to zoom in by factor of two about that point
Or, Plot from 50 to 700 Da Full range
Label all possible matches ○ Label matches used for scoring ◉
Show Y-axis ☐

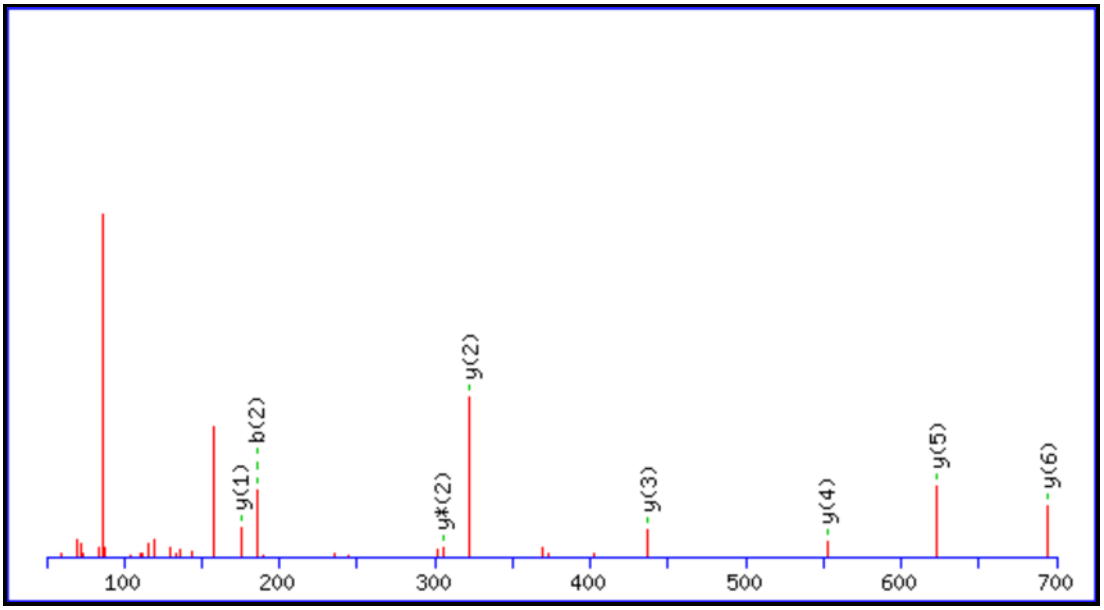

Monoisotopic mass of neutral peptide Mr(calc): 806.3923
Fixed modifications: Carbamidomethyl (C) (apply to specified residues or termini only)
Ions Score: 54 **Expect:** 0.00046
Matches : 8/50 fragment ions using 11 most intense peaks (help)

#	b	b^{++}	b^{0}	b^{0++}	Seq.	y	y^{++}	y*	y^{*++}	y^{0}	y^{0++}	#
1	114.0913	57.5493			L							7
2	**185.1285**	93.0679			A	***694.3155***	347.6614	677.2889	339.1481	676.3049	338.6561	6
3	256.1656	128.5864			A	***623.2784***	312.1428	606.2518	303.6295	605.2678	303.1375	5
4	371.1925	186.0999	353.1819	177.0946	D	***552.2413***	276.6243	535.2147	268.1110	534.2307	267.6190	4
5	486.2195	243.6134	468.2089	234.6081	D	***437.2143***	219.1108	420.1878	210.5975	419.2037	210.1055	3
6	633.2879	317.1476	615.2773	308.1423	F	***322.1874***	161.5973	305.1608	153.0840			2

7					R	175.1190	88.0631	158.0924	79.5498			1

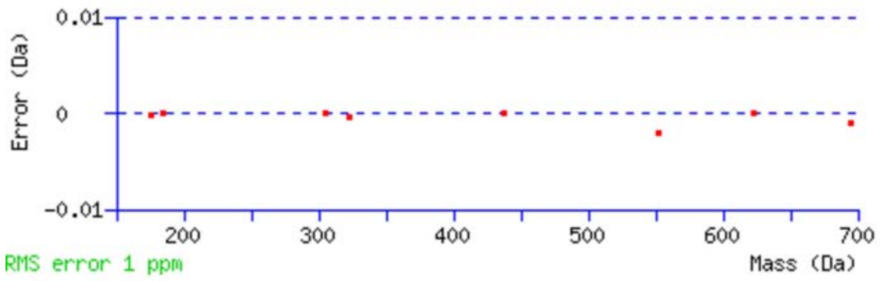

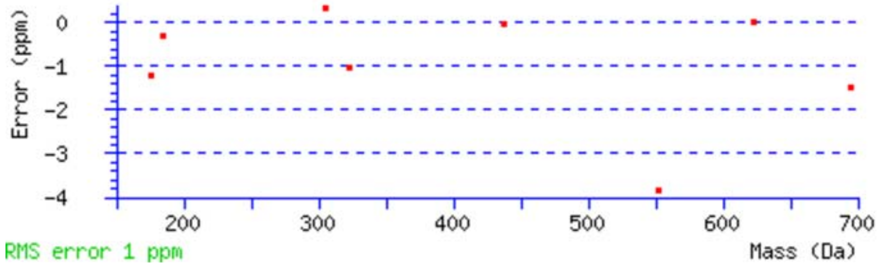

NCBI **BLAST** search of LAADDFR
(Parameters: blastp, nr protein database, expect=20000, no filter, PAM30)
Other BLAST web gateways

All matches to this query

Score	Mr(calc)	Delta	Sequence
53.9	806.3923	-0.0003	LAADDFR
19.8	804.3878	2.0041	RPDNFR
14.4	806.3844	0.0076	LAAQMQK
9.6	806.3923	-0.0003	LDQQFR
9.6	806.3923	-0.0003	LDQQFR
9.1	806.3922	-0.0003	GELANFR
9.1	804.3878	2.0041	NRPDFR
9.1	806.3923	-0.0003	QLEDFR
8.9	806.3923	-0.0003	IAGFDER
3.9	806.3891	0.0028	IACCRK

Mascot: http://www.matrixscience.com/

Mascot Search Results

Peptide View

MS/MS Fragmentation of **LAADDFR**
Found in **ch17u_O76014|KRT37_HUMAN** in **uni_human**, Keratin, type I cuticular Ha7 OS=Homo sapiens GN=KRT37 PE=3 SV=3

Match to Query 381: 806.392488 from(404.203520,2+) intensity(1165177.1250) rtinseconds(994) scans(5168) index(32479)
Title: 160219_Sunil_KAP_A1_Spectrum086736_scans__5168_RTINSECONDS=994
Data file L:\\QE_2016\\160219_Sunil_KAP_LKC\\TMgf\\T\\T160219_Sunil_KAP_A1.mgf

Click mouse within plot area to zoom in by factor of two about that point
Or, Plot from 50 to 750 Da Full range
Label all possible matches ○ Label matches used for scoring ◉
Show Y-axis ☐

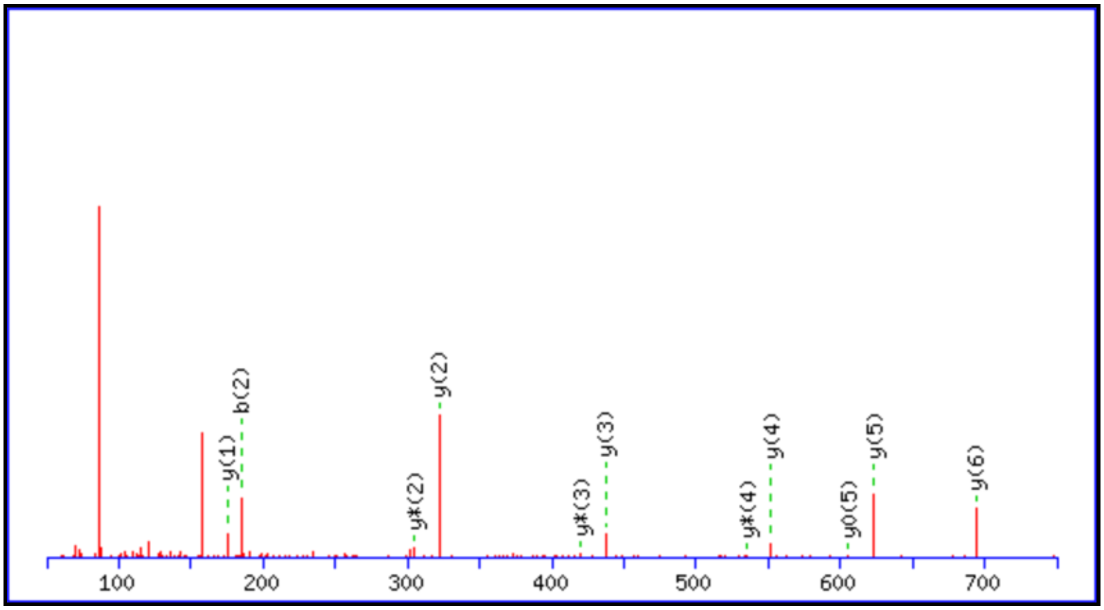

Monoisotopic mass of neutral peptide Mr(calc): 806.3923
Fixed modifications: Carbamidomethyl (C) (apply to specified residues or termini only)
Ions Score: 55 **Expect:** 0.00038
Matches : 11/50 fragment ions using 14 most intense peaks (help)

#	b	b^{++}	b^{0}	b^{0++}	Seq.	y	y^{++}	y*	y^{*++}	y^{0}	y^{0++}	#
1	114.0913	57.5493			L							7
2	**185.1285**	93.0679			A	***694.3155***	347.6614	677.2889	339.1481	676.3049	338.6561	6
3	256.1656	128.5864			A	***623.2784***	312.1428	606.2518	303.6295	605.2678	303.1375	5
4	371.1925	186.0999	353.1819	177.0946	D	***552.2413***	276.6243	535.2147	268.1110	534.2307	267.6190	4
5	486.2195	243.6134	468.2089	234.6081	D	***437.2143***	219.1108	420.1878	210.5975	419.2037	210.1055	3
6	633.2879	317.1476	615.2773	308.1423	F	***322.1874***	161.5973	305.1608	153.0840			2

7					R	175.1190	88.0631	158.0924	79.5498			1

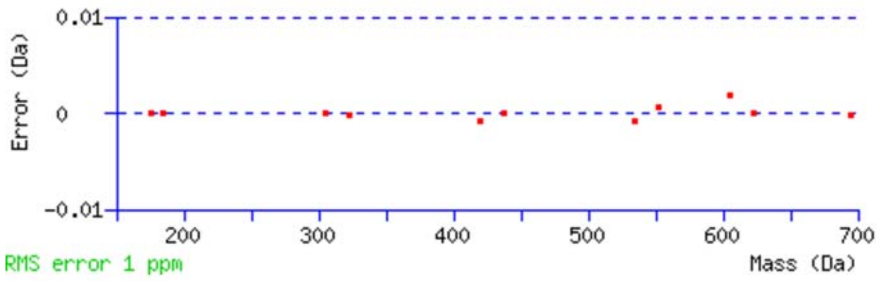

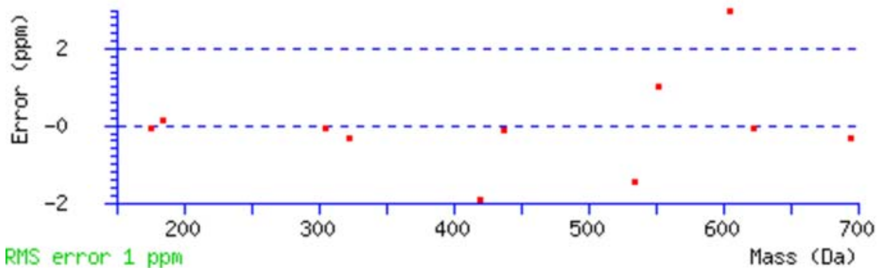

NCBI **BLAST** search of LAADDFR
(Parameters: blastp, nr protein database, expect=20000, no filter, PAM30)
Other BLAST web gateways

All matches to this query

Score	Mr(calc)	Delta	Sequence
54.6	806.3923	0.0002	LAADDFR
19.3	804.3878	2.0047	RPDNFR
7.5	806.3922	0.0002	GELANFR
7.5	804.3878	2.0047	NRPDFR
7.5	806.3923	0.0002	QLEDFR
7.5	806.3923	0.0002	IAGFDER
6.9	806.3923	0.0002	LDQQFR
6.9	806.3923	0.0002	LDQQFR
3.7	806.3891	0.0034	IACCRK
3.7	806.3956	-0.0031	LACTNTK

Mascot: http://www.matrixscience.com/

Mascot Search Results

Peptide View

MS/MS Fragmentation of **TIEELQQKILCSK**
Found in **ch17u_O76014|KRT37_HUMAN** in **uni_human**, Keratin, type I cuticular Ha7 OS=Homo sapiens GN=KRT37 PE=3 SV=3

Match to Query 17616: 1588.849692 from(530.623840,3+) intensity(633571.9375) rtinseconds(1895) scans(10405) index(22599)
Title: 160219_Sunil_KAP_A1_Spectrum075060_scans__10405_RTINSECONDS=1895
Data file L:\\QE_2016\\160219_Sunil_KAP_LKC\\TMgf\\T\\T160219_Sunil_KAP_A1.mgf

Click mouse within plot area to zoom in by factor of two about that point
Or, Plot from 100 to 1600 Da Full range
Label all possible matches ○ Label matches used for scoring ◉
Show Y-axis ☐

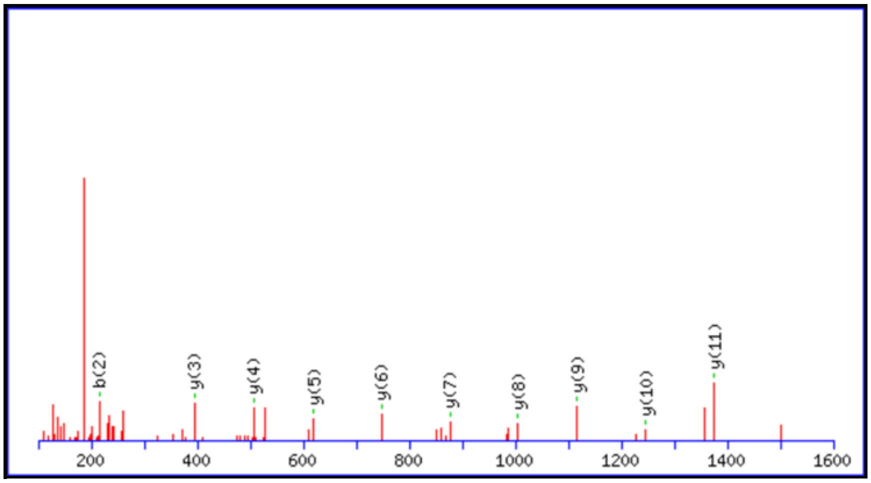

Monoisotopic mass of neutral peptide Mr(calc): 1588.8494
Fixed modifications: Carbamidomethyl (C) (apply to specified residues or termini only)
Ions Score: 78 **Expect:** 2.6e-006
Matches : 10/132 fragment ions using 13 most intense peaks (help)

#	b	b++	b*	b*++	b0	b0++	Seq.	y	y++	y*	y*++	y0	y0++	#
1	102.0550	51.5311			84.0444	42.5258	T							13
2	**215.1390**	108.0731			197.1285	99.0679	I	1488.8090	744.9082	1471.7825	736.3949	1470.7985	735.9029	12
3	344.1816	172.5944			326.1710	163.5892	E	***1375.7250***	688.3661	1358.6984	679.8529	1357.7144	679.3608	11
4	473.2242	237.1157			455.2136	228.1105	E	***1246.6824***	623.8448	1229.6558	615.3316	1228.6718	614.8395	10
5	586.3083	293.6578			568.2977	284.6525	L	***1117.6398***	559.3235	1100.6132	550.8103	1099.6292	550.3183	9
6	714.3668	357.6871	697.3403	349.1738	696.3563	348.6818	Q	***1004.5557***	502.7815	987.5292	494.2682	986.5452	493.7762	8
7	842.4254	421.7164	825.3989	413.2031	824.4149	412.7111	Q	***876.4972***	438.7522	859.4706	430.2389	858.4866	429.7469	7
8	970.5204	485.7638	953.4938	477.2506	952.5098	476.7585	K	***748.4386***	374.7229	731.4120	366.2096	730.4280	365.7176	6
9	1083.6045	542.3059	1066.5779	533.7926	1065.5939	533.3006	I	***620.3436***	310.6754	603.3171	302.1622	602.3330	301.6702	5
10	1196.6885	598.8479	1179.6620	590.3346	1178.6780	589.8426	L	***507.2595***	254.1334	490.2330	245.6201	489.2490	245.1281	4
11	1356.7192	678.8632	1339.6926	670.3499	1338.7086	669.8579	C	***394.1755***	197.5914	377.1489	189.0781	376.1649	188.5861	3
12	1443.7512	722.3792	1426.7246	713.8660	1425.7406	713.3740	S	234.1448	117.5761	217.1183	109.0628	216.1343	108.5708	2
13							K	147.1128	74.0600	130.0863	65.5468			1

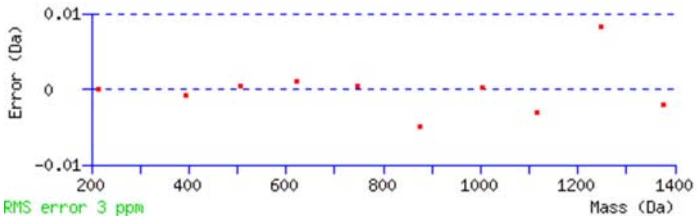

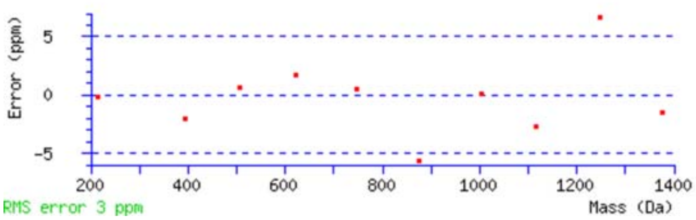

NCBI **BLAST** search of TIEELQQKILCSK
(Parameters: blastp, nr protein database, expect=20000, no filter, PAM30)
Other BLAST web gateways

All matches to this query

Score	Mr(calc)	Delta	Sequence
77.6	1588.8494	0.0003	TIEELQQKILCSK

Mascot: http://www.matrixscience.com/

Mascot Search Results

Peptide View

MS/MS Fragmentation of **LAADDFR**
Found in **ch17u_O76014|KRT37_HUMAN** in **uni_human**, Keratin, type I cuticular Ha7 OS=Homo sapiens GN=KRT37 PE=3 SV=3

Match to Query 367: 806.390468 from(404.202510,2+) intensity(108570.0234) rtinseconds(994) scans(5090) index(3774)
Title: 160219_Sunil_KAP_A1_Spectrum054737_scans__5090_RTINSECONDS=994
Data file L:\\QE_2016\\160219_Sunil_KAP_LKC\\TMgf\\T\\T160219_Sunil_KAP_A1.mgf

Click mouse within plot area to zoom in by factor of two about that point
Or, Plot from 50 to 750 Da Full range
Label all possible matches ○ Label matches used for scoring ◉
Show Y-axis ☐

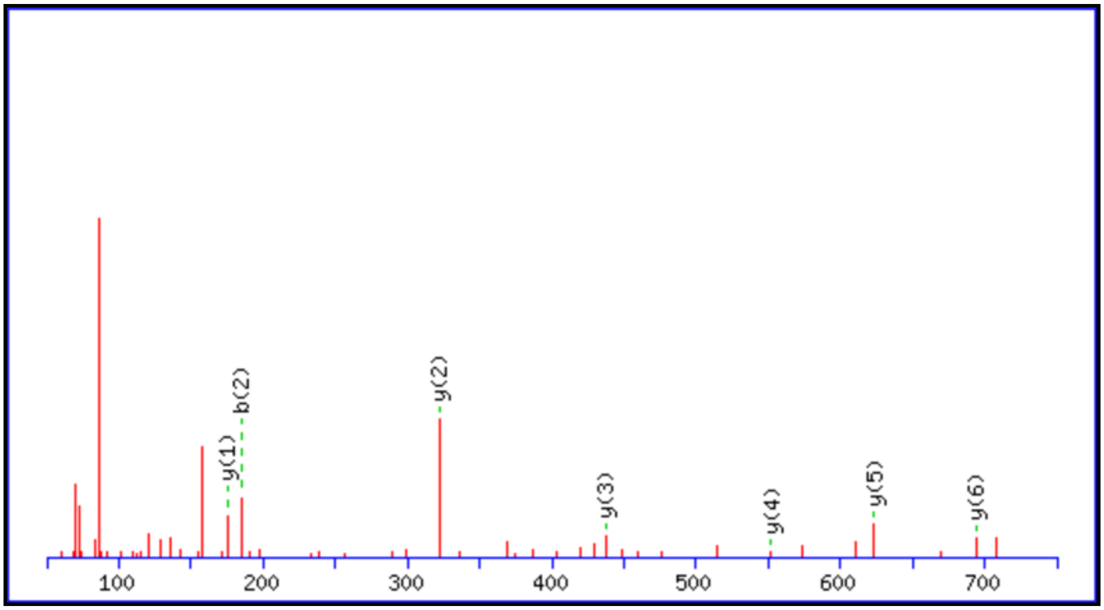

Monoisotopic mass of neutral peptide Mr(calc): 806.3923
Fixed modifications: Carbamidomethyl (C) (apply to specified residues or termini only)
Ions Score: 41 **Expect:** 0.0092
Matches : 7/50 fragment ions using 14 most intense peaks (help)

#	b	b^{++}	b^{0}	b^{0++}	Seq.	y	y^{++}	y*	y^{*++}	y^{0}	y^{0++}	#
1	114.0913	57.5493			L							7
2	**185.1285**	93.0679			A	***694.3155***	347.6614	677.2889	339.1481	676.3049	338.6561	6
3	256.1656	128.5864			A	***623.2784***	312.1428	606.2518	303.6295	605.2678	303.1375	5
4	371.1925	186.0999	353.1819	177.0946	D	***552.2413***	276.6243	535.2147	268.1110	534.2307	267.6190	4
5	486.2195	243.6134	468.2089	234.6081	D	***437.2143***	219.1108	420.1878	210.5975	419.2037	210.1055	3
6	633.2879	317.1476	615.2773	308.1423	F	***322.1874***	161.5973	305.1608	153.0840			2

7					R	*175.1190*	88.0631	158.0924	79.5498			1

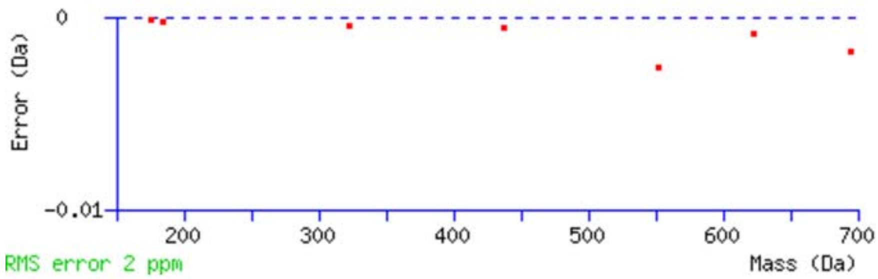

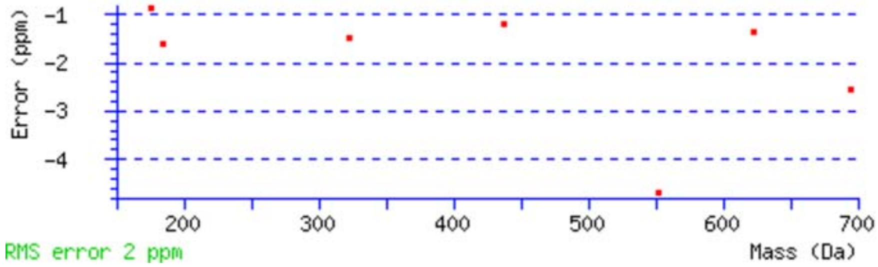

NCBI **BLAST** search of LAADDFR
(Parameters: blastp, nr protein database, expect=20000, no filter, PAM30)
Other BLAST web gateways

All matches to this query

Score	Mr(calc)	Delta	Sequence
41.1	806.3923	-0.0018	LAADDFR
12.1	804.3878	2.0026	RPDNFR
4.1	806.3923	-0.0018	IAGFDER
3.9	806.3922	-0.0018	GELANFR
3.9	804.3878	2.0026	NRPDFR
3.9	806.3923	-0.0018	QLEDFR
3.8	806.3923	-0.0018	LDQQFR
3.8	806.3923	-0.0018	LDQQFR
2.9	806.3844	0.0061	LAAQMQK

Mascot: http://www.matrixscience.com/

Mascot Search Results

Peptide View

MS/MS Fragmentation of **LAADDFR**
Found in **ch17u_O76014|KRT37_HUMAN** in **uni_human**, Keratin, type I cuticular Ha7 OS=Homo sapiens GN=KRT37 PE=3 SV=3

Match to Query 368: 806.390788 from(404.202670,2+) intensity(282131.5000) rtinseconds(863) scans(4335) index(3176)
Title: 160219_Sunil_KAP_A1_Spectrum054138_scans__4335_RTINSECONDS=863
Data file L:\\QE_2016\\160219_Sunil_KAP_LKC\\TMgf\\T\\T160219_Sunil_KAP_A1.mgf

Click mouse within plot area to zoom in by factor of two about that point
Or, Plot from 50 to 700 Da Full range
Label all possible matches ○ Label matches used for scoring ◉
Show Y-axis ☐

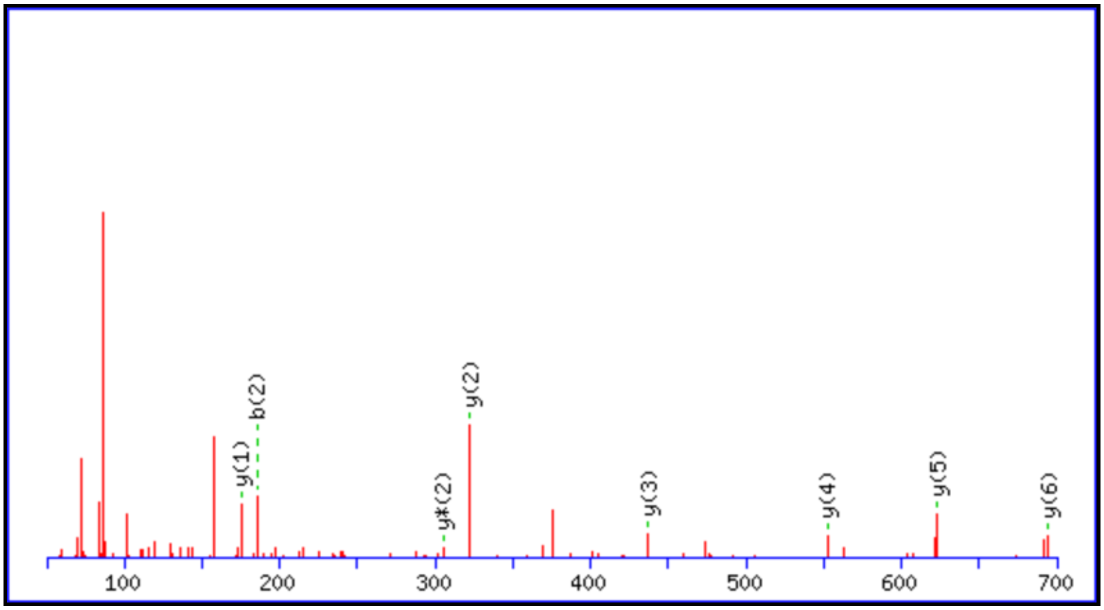

Monoisotopic mass of neutral peptide Mr(calc): 806.3923
Fixed modifications: Carbamidomethyl (C) (apply to specified residues or termini only)
Ions Score: 44 **Expect:** 0.0046
Matches : 8/50 fragment ions using 14 most intense peaks (help)

#	b	b^{++}	b^{0}	b^{0++}	Seq.	y	y^{++}	y*	y^{*++}	y^{0}	y^{0++}	#
1	114.0913	57.5493			L							7
2	**185.1285**	93.0679			A	***694.3155***	347.6614	677.2889	339.1481	676.3049	338.6561	6
3	256.1656	128.5864			A	***623.2784***	312.1428	606.2518	303.6295	605.2678	303.1375	5
4	371.1925	186.0999	353.1819	177.0946	D	***552.2413***	276.6243	535.2147	268.1110	534.2307	267.6190	4
5	486.2195	243.6134	468.2089	234.6081	D	***437.2143***	219.1108	420.1878	210.5975	419.2037	210.1055	3
6	633.2879	317.1476	615.2773	308.1423	F	***322.1874***	161.5973	305.1608	153.0840			2

7					R	175.1190	88.0631	158.0924	79.5498			1

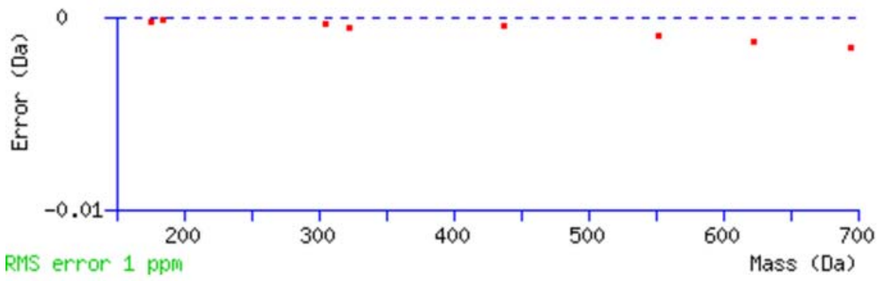

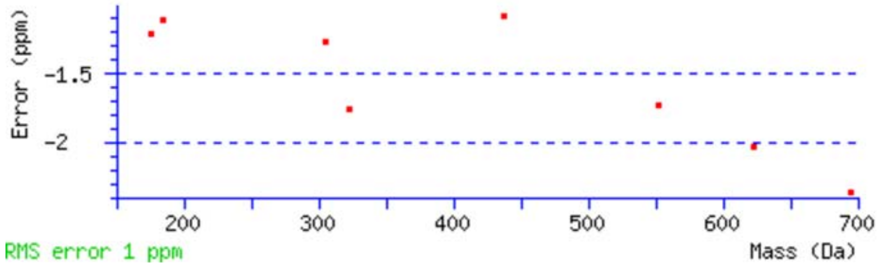

NCBI **BLAST** search of LAADDFR
(Parameters: blastp, nr protein database, expect=20000, no filter, PAM30)
Other BLAST web gateways

All matches to this query

Score	Mr(calc)	Delta	Sequence
44.1	806.3923	-0.0015	LAADDFR
14.4	804.3878	2.0030	RPDNFR
11.8	806.3844	0.0064	LAAQMQK
7.2	805.3930	0.9978	DGASTSLR
5.9	806.3956	-0.0048	LGDMSLR
5.1	806.3922	-0.0015	GELANFR
5.1	804.3878	2.0030	NRPDFR
5.1	806.3923	-0.0015	QLEDFR
5.1	806.3923	-0.0015	LDQQFR
5.1	806.3923	-0.0015	LDQQFR

Mascot: http://www.matrixscience.com/

Mascot Search Results

Peptide View

MS/MS Fragmentation of **LAADDFR**
Found in **ch17u_O76014|KRT37_HUMAN** in **uni_human**, Keratin, type I cuticular Ha7 OS=Homo sapiens GN=KRT37 PE=3 SV=3

Match to Query 370: 806.391208 from(404.202880,2+) intensity(94538.2266) rtinseconds(1274) scans(6700) index(5047)
Title: 160219_Sunil_KAP_A1_Spectrum056012_scans__6700_RTINSECONDS=1274
Data file L:\\QE_2016\\160219_Sunil_KAP_LKC\\TMgf\\T\\T160219_Sunil_KAP_A1.mgf

Click mouse within plot area to zoom in by factor of two about that point
Or, Plot from 50 to 700 Da Full range
Label all possible matches ○ Label matches used for scoring ◉
Show Y-axis ☐

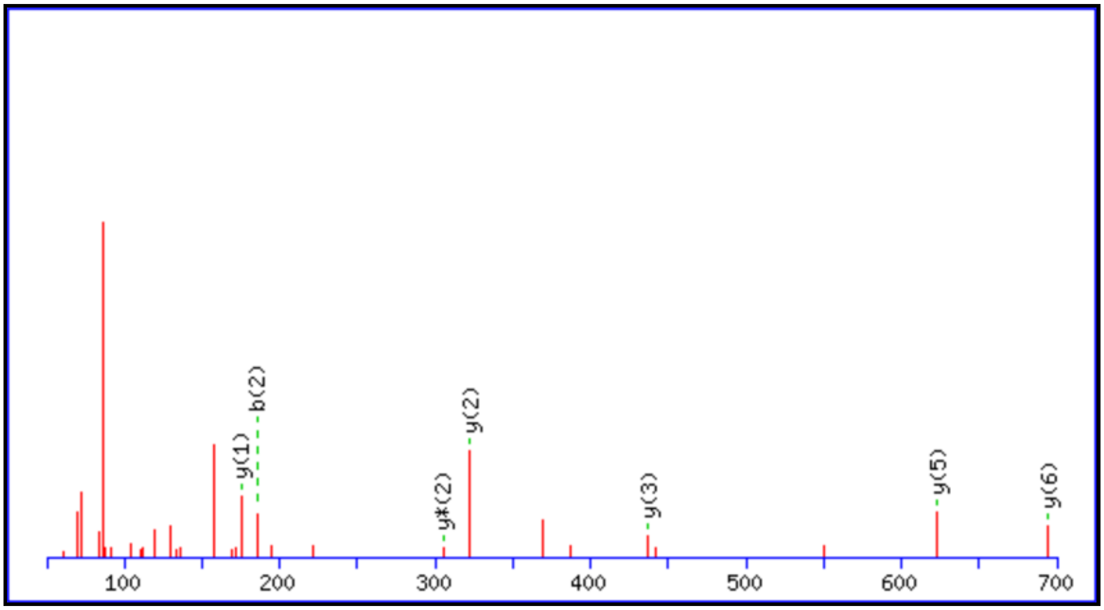

Monoisotopic mass of neutral peptide Mr(calc): 806.3923
Fixed modifications: Carbamidomethyl (C) (apply to specified residues or termini only)
Ions Score: 36 **Expect:** 0.027
Matches : 7/50 fragment ions using 11 most intense peaks (help)

#	b	b^{++}	b^{0}	b^{0++}	Seq.	y	y^{++}	y*	y^{*++}	y^{0}	y^{0++}	#
1	114.0913	57.5493			L							7
2	**185.1285**	93.0679			A	***694.3155***	347.6614	677.2889	339.1481	676.3049	338.6561	6
3	256.1656	128.5864			A	***623.2784***	312.1428	606.2518	303.6295	605.2678	303.1375	5
4	371.1925	186.0999	353.1819	177.0946	D	552.2413	276.6243	535.2147	268.1110	534.2307	267.6190	4
5	486.2195	243.6134	468.2089	234.6081	D	***437.2143***	219.1108	420.1878	210.5975	419.2037	210.1055	3
6	633.2879	317.1476	615.2773	308.1423	F	***322.1874***	161.5973	305.1608	153.0840			2

7					R	*175.1190*	88.0631	158.0924	79.5498			1

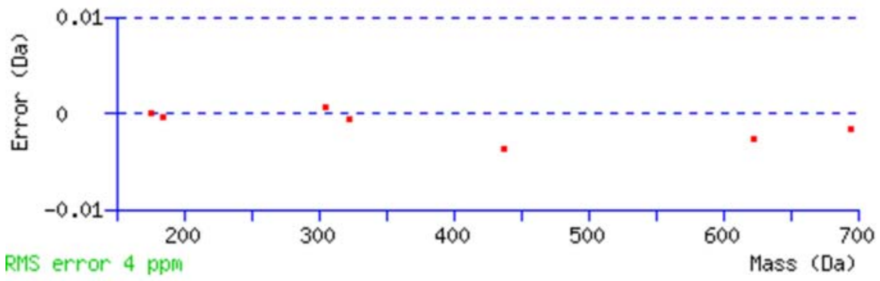

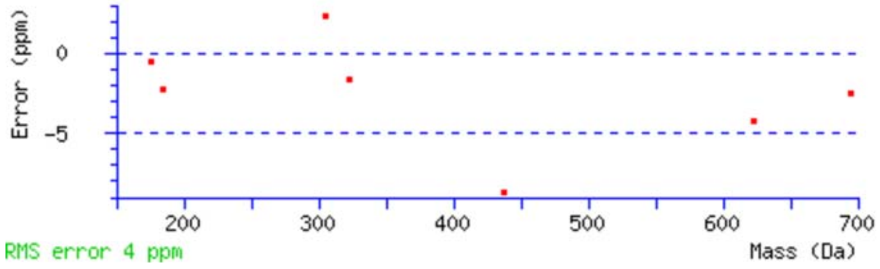

NCBI **BLAST** search of LAADDFR
(Parameters: blastp, nr protein database, expect=20000, no filter, PAM30)
Other BLAST web gateways

All matches to this query

Score	Mr(calc)	Delta	Sequence
36.5	806.3923	-0.0010	LAADDFR
11.4	806.3923	-0.0010	LDQQFR
11.4	806.3923	-0.0010	LDQQFR
10.9	806.3923	-0.0010	QQQVFR
10.9	806.3923	-0.0010	QQQVFR
9.8	806.3923	-0.0010	IAGFDER
8.2	806.3922	-0.0010	GELANFR
8.2	804.3878	2.0034	NRPDFR
8.2	806.3923	-0.0010	QLEDFR
8.2	804.3878	2.0034	RPDNFR

Mascot: http://www.matrixscience.com/

Mascot Search Results

Peptide View

MS/MS Fragmentation of **LAADDFR**
Found in **ch17u_O76014|KRT37_HUMAN** in **uni_human**, Keratin, type I cuticular Ha7 OS=Homo sapiens GN=KRT37 PE=3 SV=3

Match to Query 371: 806.391388 from(404.202970,2+) intensity(61420.0664) rtinseconds(880) scans(4573) index(17772)
Title: 160219_Sunil_KAP_A1_Spectrum070217_scans__4573_RTINSECONDS=880
Data file L:\\QE_2016\\160219_Sunil_KAP_LKC\\TMgf\\T\\T160219_Sunil_KAP_A1.mgf

Click mouse within plot area to zoom in by factor of two about that point
Or, Plot from 50 to 700 Da Full range
Label all possible matches ○ Label matches used for scoring ◉
Show Y-axis ☐

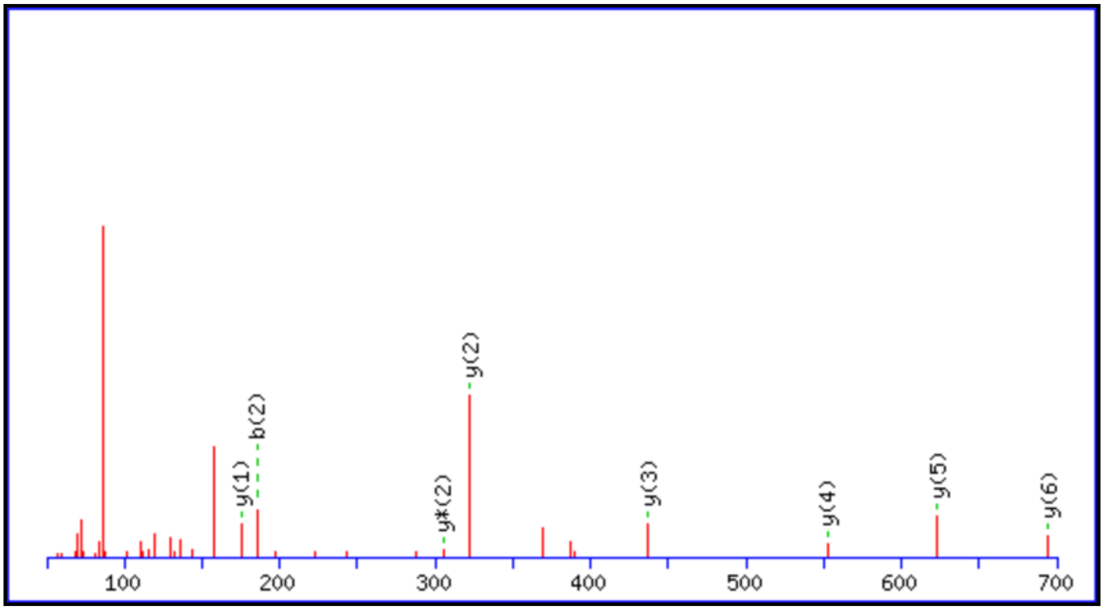

Monoisotopic mass of neutral peptide Mr(calc): 806.3923
Fixed modifications: Carbamidomethyl (C) (apply to specified residues or termini only)
Ions Score: 43 **Expect:** 0.0056
Matches : 8/50 fragment ions using 15 most intense peaks (help)

#	b	b^{++}	b^0	b^{0++}	Seq.	y	y^{++}	y^*	y^{*++}	y^0	y^{0++}	#
1	114.0913	57.5493			L							7
2	**185.1285**	93.0679			A	***694.3155***	347.6614	677.2889	339.1481	676.3049	338.6561	6
3	256.1656	128.5864			A	***623.2784***	312.1428	606.2518	303.6295	605.2678	303.1375	5
4	371.1925	186.0999	353.1819	177.0946	D	***552.2413***	276.6243	535.2147	268.1110	534.2307	267.6190	4
5	486.2195	243.6134	468.2089	234.6081	D	***437.2143***	219.1108	420.1878	210.5975	419.2037	210.1055	3
6	633.2879	317.1476	615.2773	308.1423	F	***322.1874***	161.5973	305.1608	153.0840			2

7					R	*175.1190*	88.0631	158.0924	79.5498			1

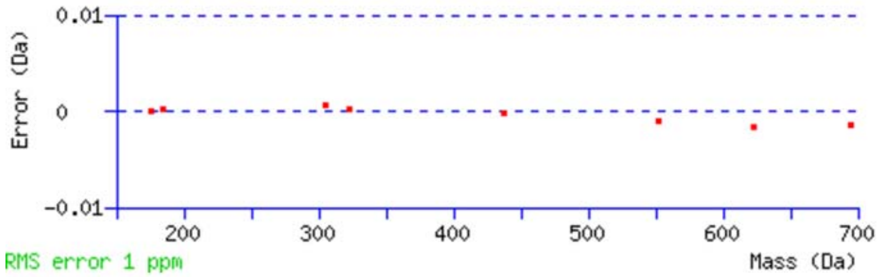

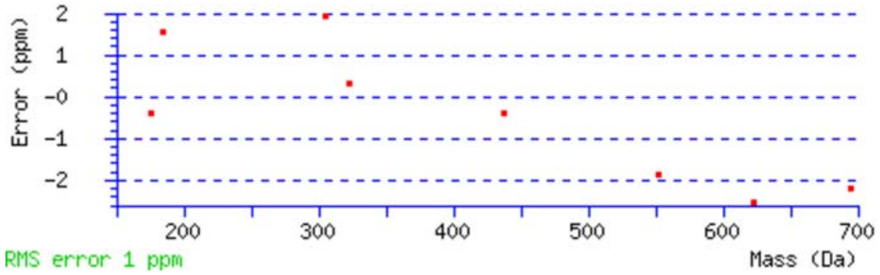

NCBI **BLAST** search of LAADDFR
(Parameters: blastp, nr protein database, expect=20000, no filter, PAM30)
Other BLAST web gateways

All matches to this query

Score	Mr(calc)	Delta	Sequence
43.0	806.3923	-0.0009	LAADDFR
14.6	804.3878	2.0036	RPDNFR
7.4	806.3844	0.0070	LAAQMQK
5.9	806.3922	-0.0009	GELANFR
5.9	804.3878	2.0036	NRPDFR
5.9	806.3923	-0.0009	QLEDFR
5.7	806.3923	-0.0009	LDQQFR
5.7	806.3923	-0.0009	LDQQFR
3.7	806.3923	-0.0009	IAGFDER

Mascot: http://www.matrixscience.com/

Mascot Search Results

Peptide View

MS/MS Fragmentation of **LAADDFR**
Found in **ch17u_O76014|KRT37_HUMAN** in **uni_human**, Keratin, type I cuticular Ha7 OS=Homo sapiens GN=KRT37 PE=3 SV=3

Match to Query 372: 806.391568 from(404.203060,2+) intensity(91693.5781) rtinseconds(1159) scans(6179) index(19040)
Title: 160219_Sunil_KAP_A1_Spectrum071489_scans__6179_RTINSECONDS=1159
Data file L:\\QE_2016\\160219_Sunil_KAP_LKC\\TMgf\\T\\T160219_Sunil_KAP_A1.mgf

Click mouse within plot area to zoom in by factor of two about that point
Or, Plot from 50 to 700 Da Full range
Label all possible matches ○ Label matches used for scoring ◉
Show Y-axis ☐

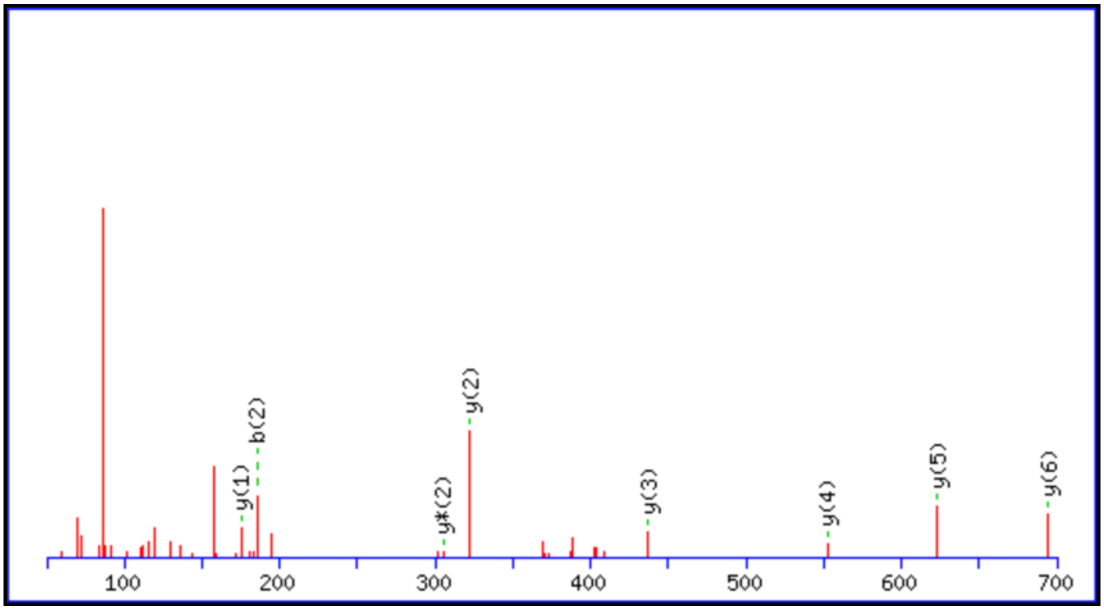

Monoisotopic mass of neutral peptide Mr(calc): 806.3923
Fixed modifications: Carbamidomethyl (C) (apply to specified residues or termini only)
Ions Score: 53 **Expect:** 0.00058
Matches : 8/50 fragment ions using 11 most intense peaks (help)

#	b	b^{++}	b^0	b^{0++}	Seq.	y	y^{++}	y*	y^{*++}	y^0	y^{0++}	#
1	114.0913	57.5493			L							7
2	**185.1285**	93.0679			A	***694.3155***	347.6614	677.2889	339.1481	676.3049	338.6561	6
3	256.1656	128.5864			A	***623.2784***	312.1428	606.2518	303.6295	605.2678	303.1375	5
4	371.1925	186.0999	353.1819	177.0946	D	***552.2413***	276.6243	535.2147	268.1110	534.2307	267.6190	4
5	486.2195	243.6134	468.2089	234.6081	D	***437.2143***	219.1108	420.1878	210.5975	419.2037	210.1055	3
6	633.2879	317.1476	615.2773	308.1423	F	***322.1874***	161.5973	305.1608	153.0840			2

7					R	*175.1190*	88.0631	158.0924	79.5498			1

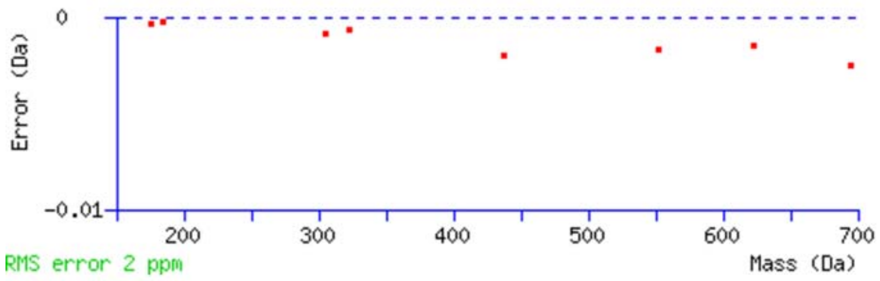

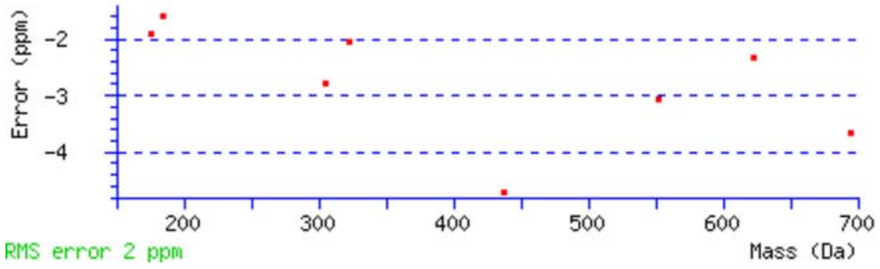

NCBI **BLAST** search of LAADDFR
(Parameters: blastp, nr protein database, expect=20000, no filter, PAM30)
Other BLAST web gateways

All matches to this query

Score	Mr(calc)	Delta	Sequence
52.9	806.3923	-0.0007	LAADDFR
18.7	804.3878	2.0037	RPDNFR
13.4	806.3844	0.0072	LAAQMQK
8.5	806.3923	-0.0007	LDQQFR
8.5	806.3923	-0.0007	LDQQFR
8.0	806.3922	-0.0007	GELANFR
8.0	804.3878	2.0037	NRPDFR
8.0	806.3923	-0.0007	QLEDFR
8.0	806.3923	-0.0007	IAGFDER
2.9	806.3891	0.0025	IACCRK

Mascot: http://www.matrixscience.com/

Mascot Search Results

Peptide View

MS/MS Fragmentation of **LAADDFR**
Found in **ch17u_O76014|KRT37_HUMAN** in **uni_human**, Keratin, type I cuticular Ha7 OS=Homo sapiens GN=KRT37 PE=3 SV=3

Match to Query 374: 806.391768 from(404.203160,2+) intensity(599164.4375) rtinseconds(1105) scans(5728) index(4244)
Title: 160219_Sunil_KAP_A1_Spectrum055207_scans__5728_RTINSECONDS=1105
Data file L:\\QE_2016\\160219_Sunil_KAP_LKC\\TMgf\\T\\T160219_Sunil_KAP_A1.mgf

Click mouse within plot area to zoom in by factor of two about that point
Or, Plot from 50 to 700 Da Full range
Label all possible matches ○ Label matches used for scoring ◉
Show Y-axis ☐

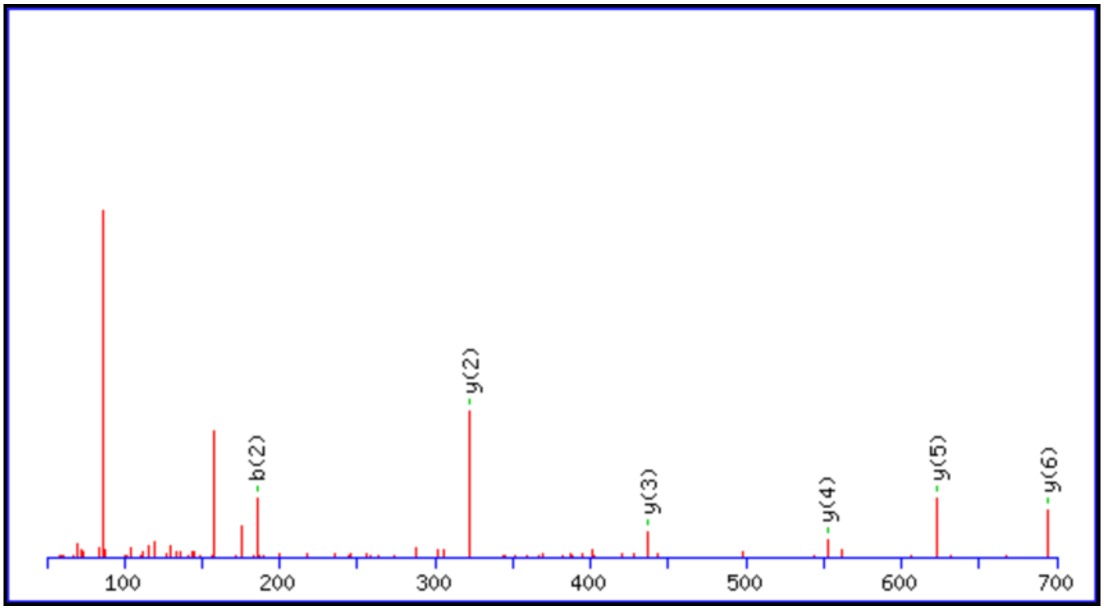

Monoisotopic mass of neutral peptide Mr(calc): 806.3923
Fixed modifications: Carbamidomethyl (C) (apply to specified residues or termini only)
Ions Score: 47 **Expect:** 0.0025
Matches : 6/50 fragment ions using 7 most intense peaks (help)

#	b	b^{++}	b^0	b^{0++}	Seq.	y	y^{++}	y*	y^{*++}	y^0	y^{0++}	#
1	114.0913	57.5493			L							7
2	**185.1285**	93.0679			A	***694.3155***	347.6614	677.2889	339.1481	676.3049	338.6561	6
3	256.1656	128.5864			A	***623.2784***	312.1428	606.2518	303.6295	605.2678	303.1375	5
4	371.1925	186.0999	353.1819	177.0946	D	***552.2413***	276.6243	535.2147	268.1110	534.2307	267.6190	4
5	486.2195	243.6134	468.2089	234.6081	D	***437.2143***	219.1108	420.1878	210.5975	419.2037	210.1055	3
6	633.2879	317.1476	615.2773	308.1423	F	***322.1874***	161.5973	305.1608	153.0840			2

7					R	175.1190	88.0631	158.0924	79.5498			1

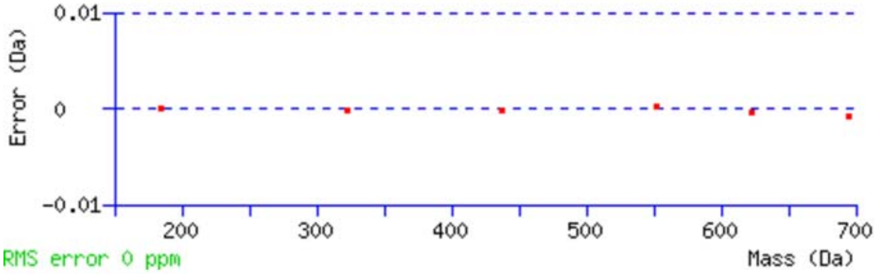

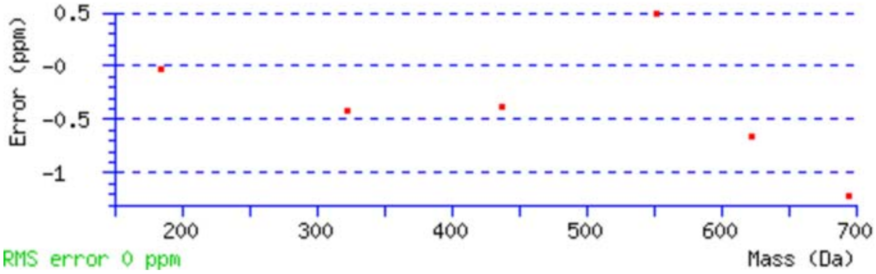

NCBI **BLAST** search of LAADDFR
(Parameters: blastp, nr protein database, expect=20000, no filter, PAM30)
Other BLAST web gateways

All matches to this query

Score	Mr(calc)	Delta	Sequence
46.5	806.3923	-0.0005	LAADDFR
13.9	806.3844	0.0074	LAAQMQK
12.9	804.3878	2.0039	RPDNFR
5.9	806.3923	-0.0005	IAGFDER
5.8	806.3956	-0.0038	MGNSLLR
4.9	806.3923	-0.0005	LDQQFR
4.9	806.3923	-0.0005	LDQQFR
4.4	806.3922	-0.0005	GELANFR
4.4	804.3878	2.0039	NRPDFR
4.4	806.3923	-0.0005	QLEDFR

Mascot: http://www.matrixscience.com/

Mascot Search Results

Peptide View

MS/MS Fragmentation of **LAADDFR**
Found in **ch17u_O76014|KRT37_HUMAN** in **uni_human**, Keratin, type I cuticular Ha7 OS=Homo sapiens GN=KRT37 PE=3 SV=3

Match to Query 375: 806.391768 from(404.203160,2+) intensity(1148643.7500) rtinseconds(1062) scans(5625) index(18570)
Title: 160219_Sunil_KAP_A1_Spectrum071019_scans__5625_RTINSECONDS=1062
Data file L:\\QE_2016\\160219_Sunil_KAP_LKC\\TMgf\\T\\T160219_Sunil_KAP_A1.mgf

Click mouse within plot area to zoom in by factor of two about that point
Or, Plot from 50 to 850 Da Full range
Label all possible matches ○ Label matches used for scoring ◉
Show Y-axis ☐

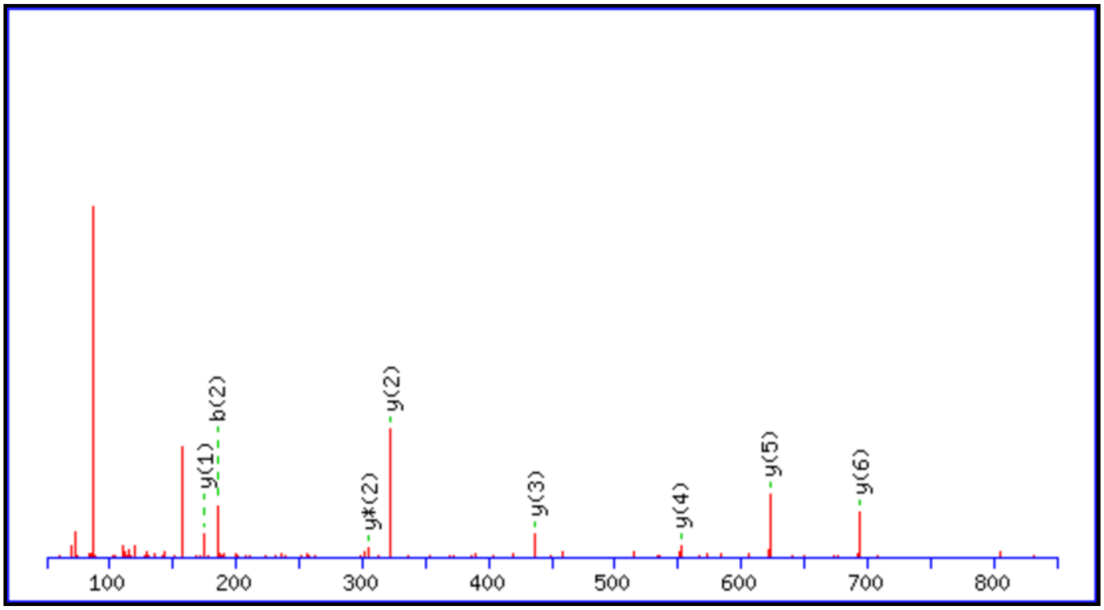

Monoisotopic mass of neutral peptide Mr(calc): 806.3923
Fixed modifications: Carbamidomethyl (C) (apply to specified residues or termini only)
Ions Score: 43 **Expect:** 0.0054
Matches : 8/50 fragment ions using 15 most intense peaks (help)

#	b	b++	b0	b0++	Seq.	y	y++	y*	y*++	y0	y0++	#
1	114.0913	57.5493			L							7
2	**185.1285**	93.0679			A	***694.3155***	347.6614	677.2889	339.1481	676.3049	338.6561	6
3	256.1656	128.5864			A	***623.2784***	312.1428	606.2518	303.6295	605.2678	303.1375	5
4	371.1925	186.0999	353.1819	177.0946	D	***552.2413***	276.6243	535.2147	268.1110	534.2307	267.6190	4
5	486.2195	243.6134	468.2089	234.6081	D	***437.2143***	219.1108	420.1878	210.5975	419.2037	210.1055	3
6	633.2879	317.1476	615.2773	308.1423	F	***322.1874***	161.5973	305.1608	153.0840			2

7					R	175.1190	88.0631	158.0924	79.5498			1

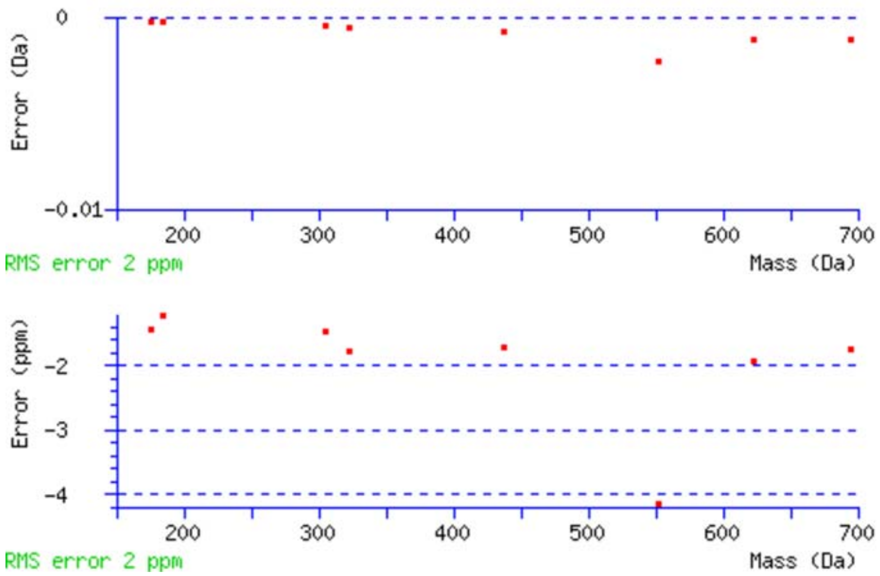

NCBI **BLAST** search of LAADDFR
(Parameters: blastp, nr protein database, expect=20000, no filter, PAM30)
Other BLAST web gateways

All matches to this query

Score	Mr(calc)	Delta	Sequence
43.2	806.3923	-0.0005	LAADDFR
13.7	804.3878	2.0039	RPDNFR
12.0	806.3844	0.0074	LAAQMQK
7.8	806.3923	-0.0005	IAGFDER
5.5	806.3923	-0.0005	LDQQFR
5.5	806.3923	-0.0005	LDQQFR
5.0	806.3922	-0.0005	GELANFR
5.0	804.3878	2.0039	NRPDFR
5.0	806.3923	-0.0005	QLEDFR
1.6	804.3912	2.0006	LASQACR

Mascot: http://www.matrixscience.com/

Mascot Search Results

Peptide View

MS/MS Fragmentation of **LAADDFR**
Found in **ch17u_O76014|KRT37_HUMAN** in **uni_human**, Keratin, type I cuticular Ha7 OS=Homo sapiens GN=KRT37 PE=3 SV=3

Match to Query 376: 806.392008 from(404.203280,2+) intensity(843326.4375) rtinseconds(1027) scans(5355) index(32624)
Title: 160219_Sunil_KAP_A1_Spectrum086881_scans__5355_RTINSECONDS=1027
Data file L:\\QE_2016\\160219_Sunil_KAP_LKC\\TMgf\\T\\T160219_Sunil_KAP_A1.mgf

Click mouse within plot area to zoom in by factor of two about that point
Or, Plot from 50 to 800 Da Full range
Label all possible matches ○ Label matches used for scoring ◉
Show Y-axis ☐

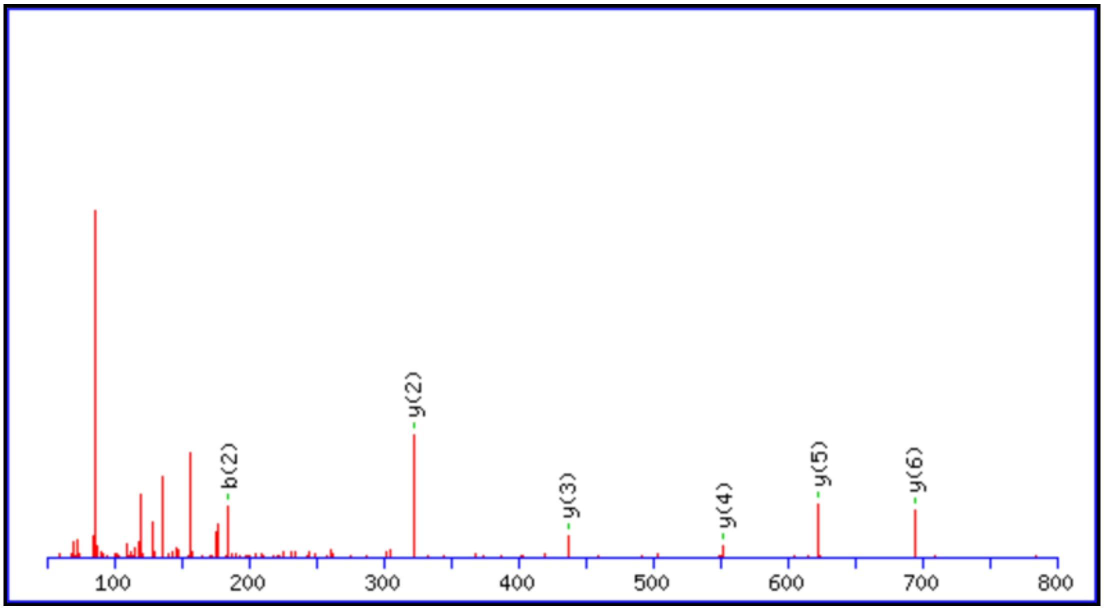

Monoisotopic mass of neutral peptide Mr(calc): 806.3923
Fixed modifications: Carbamidomethyl (C) (apply to specified residues or termini only)
Ions Score: 41 **Expect:** 0.008
Matches : 6/50 fragment ions using 8 most intense peaks (help)

#	b	b^{++}	b^0	b^{0++}	Seq.	y	y^{++}	y*	y^{*++}	y^0	y^{0++}	#
1	114.0913	57.5493			L							7
2	**185.1285**	93.0679			A	***694.3155***	347.6614	677.2889	339.1481	676.3049	338.6561	6
3	256.1656	128.5864			A	***623.2784***	312.1428	606.2518	303.6295	605.2678	303.1375	5
4	371.1925	186.0999	353.1819	177.0946	D	***552.2413***	276.6243	535.2147	268.1110	534.2307	267.6190	4
5	486.2195	243.6134	468.2089	234.6081	D	***437.2143***	219.1108	420.1878	210.5975	419.2037	210.1055	3
6	633.2879	317.1476	615.2773	308.1423	F	***322.1874***	161.5973	305.1608	153.0840			2

7					R	175.1190	88.0631	158.0924	79.5498			1

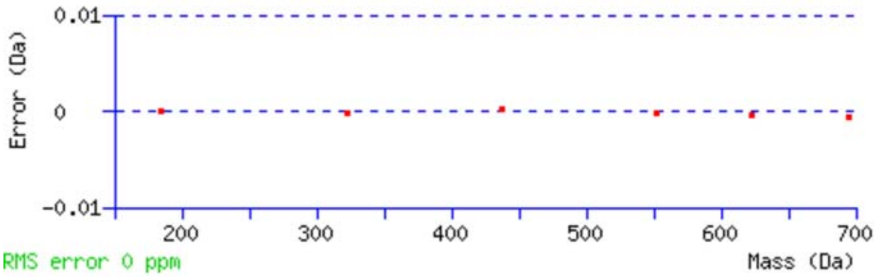

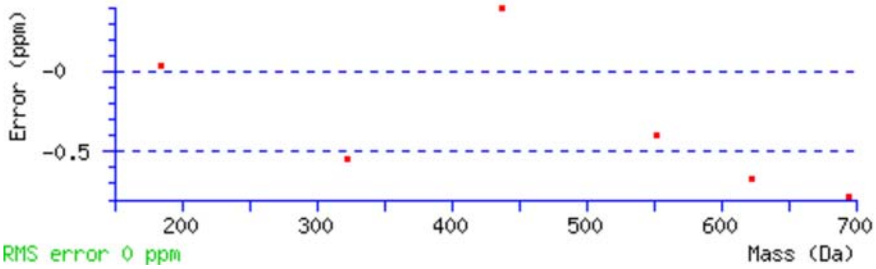

NCBI **BLAST** search of LAADDFR
(Parameters: blastp, nr protein database, expect=20000, no filter, PAM30)
Other BLAST web gateways

All matches to this query

Score	Mr(calc)	Delta	Sequence
41.5	806.3923	-0.0003	LAADDFR
11.1	806.3844	0.0076	LAAQMQK
9.5	804.3878	2.0042	RPDNFR
3.2	806.3923	-0.0003	QLEDFR
3.0	806.3922	-0.0002	GELANFR
2.2	804.3878	2.0042	NRPDFR
2.2	806.3923	-0.0003	IAGFDER

Mascot: http://www.matrixscience.com/

Mascot Search Results

Peptide View

MS/MS Fragmentation of **LAADDFR**
Found in **ch17u_O76014|KRT37_HUMAN** in **uni_human**, Keratin, type I cuticular Ha7 OS=Homo sapiens GN=KRT37 PE=3 SV=3

Match to Query 377: 806.392068 from(404.203310,2+) intensity(379124.4375) rtinseconds(1000) scans(5267) index(18299)
Title: 160219_Sunil_KAP_A1_Spectrum070746_scans__5267_RTINSECONDS=1000
Data file L:\\QE_2016\\160219_Sunil_KAP_LKC\\TMgf\\T\\T160219_Sunil_KAP_A1.mgf

Click mouse within plot area to zoom in by factor of two about that point
Or, Plot from 50 to 700 Da Full range
Label all possible matches ○ Label matches used for scoring ◉
Show Y-axis ☐

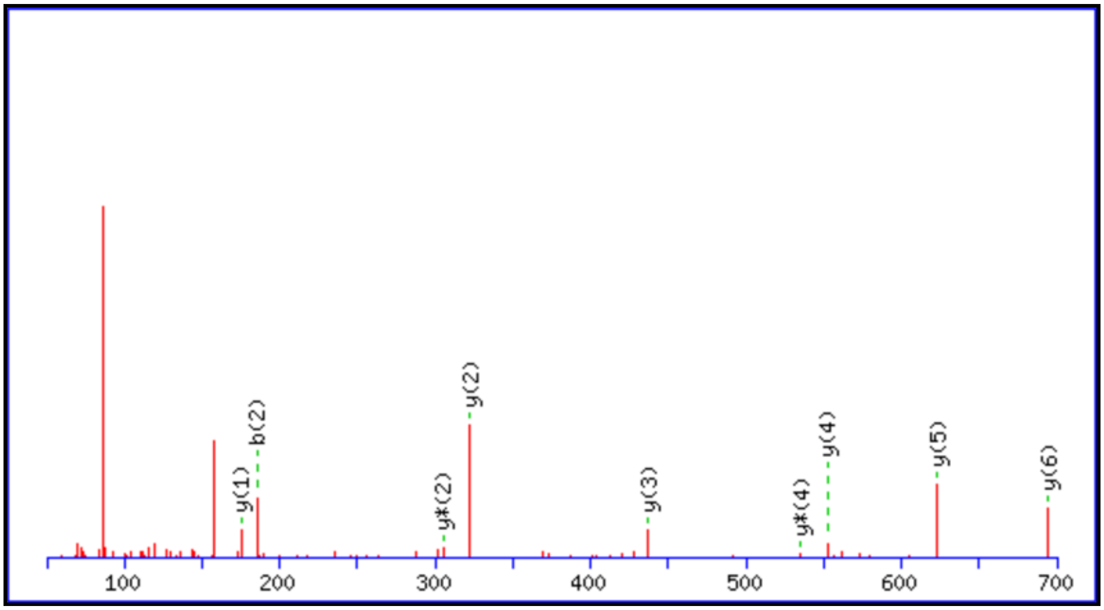

Monoisotopic mass of neutral peptide Mr(calc): 806.3923
Fixed modifications: Carbamidomethyl (C) (apply to specified residues or termini only)
Ions Score: 51 **Expect:** 0.00087
Matches : 9/50 fragment ions using 13 most intense peaks (help)

#	b	b^{++}	b^0	b^{0++}	Seq.	y	y^{++}	y*	y^{*++}	y^0	y^{0++}	#
1	114.0913	57.5493			L							7
2	**185.1285**	93.0679			A	***694.3155***	347.6614	677.2889	339.1481	676.3049	338.6561	6
3	256.1656	128.5864			A	***623.2784***	312.1428	606.2518	303.6295	605.2678	303.1375	5
4	371.1925	186.0999	353.1819	177.0946	D	***552.2413***	276.6243	535.2147	268.1110	534.2307	267.6190	4
5	486.2195	243.6134	468.2089	234.6081	D	***437.2143***	219.1108	420.1878	210.5975	419.2037	210.1055	3
6	633.2879	317.1476	615.2773	308.1423	F	***322.1874***	161.5973	305.1608	153.0840			2

7					R	*175.1190*	88.0631	158.0924	79.5498			1

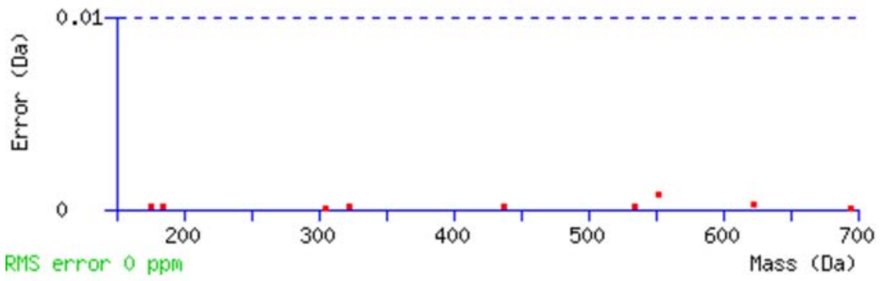

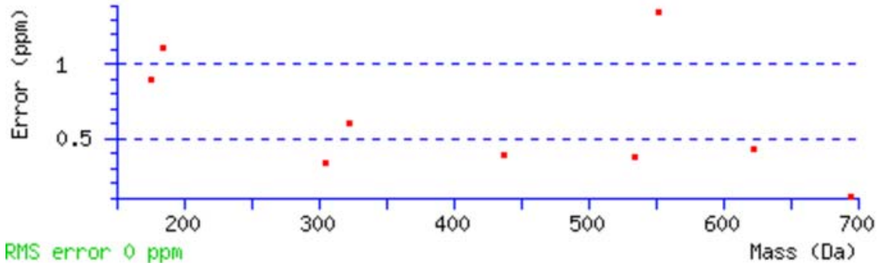

NCBI **BLAST** search of LAADDFR
(Parameters: blastp, nr protein database, expect=20000, no filter, PAM30)
Other BLAST web gateways

All matches to this query

Score	Mr(calc)	Delta	Sequence
51.1	806.3923	-0.0002	LAADDFR
18.5	804.3878	2.0042	RPDNFR
14.3	806.3844	0.0077	LAAQMQK
7.2	806.3923	-0.0002	LDQQFR
7.2	806.3923	-0.0002	LDQQFR
7.0	806.3923	-0.0002	IAGFDER
6.7	806.3922	-0.0002	GELANFR
6.7	804.3878	2.0042	NRPDFR
6.7	806.3923	-0.0002	QLEDFR
3.8	806.3891	0.0030	IACCRK

Mascot: http://www.matrixscience.com/

Mascot Search Results

Peptide View

MS/MS Fragmentation of **LAADDFR**
Found in **ch17u_O76014|KRT37_HUMAN** in **uni_human**, Keratin, type I cuticular Ha7 OS=Homo sapiens GN=KRT37 PE=3 SV=3

Match to Query 378: 806.392248 from(404.203400,2+) intensity(5551629.5000) rtinseconds(1137) scans(5913) index(4387)
Title: 160219_Sunil_KAP_A1_Spectrum055350_scans__5913_RTINSECONDS=1137
Data file L:\\QE_2016\\160219_Sunil_KAP_LKC\\TMgf\\T\\T160219_Sunil_KAP_A1.mgf

Click mouse within plot area to zoom in by factor of two about that point
Or, Plot from 50 to 850 Da Full range
Label all possible matches ○ Label matches used for scoring ◉
Show Y-axis ☐

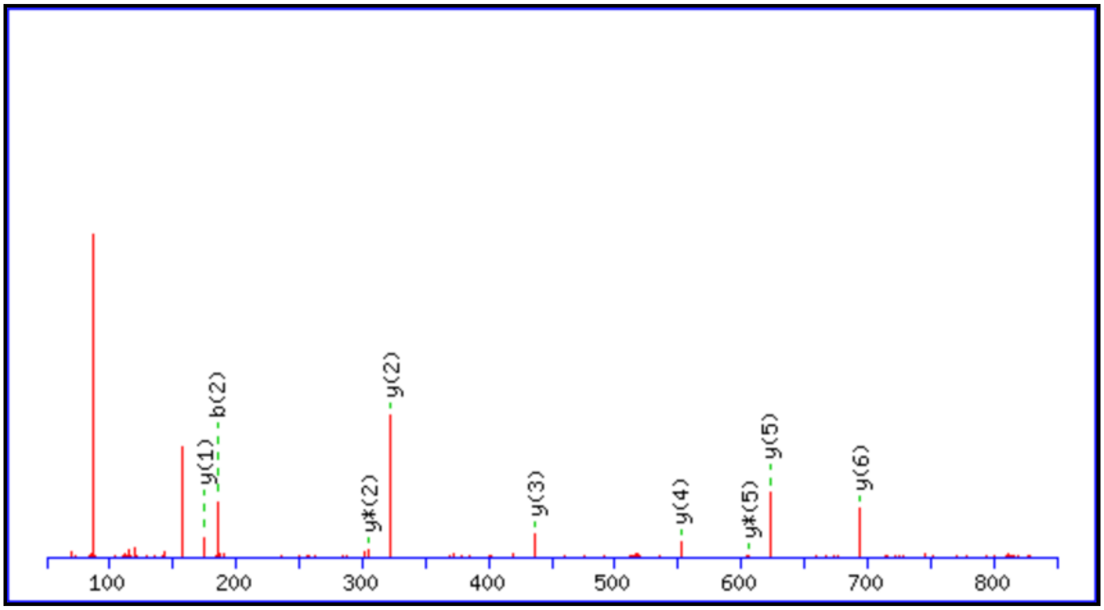

Monoisotopic mass of neutral peptide Mr(calc): 806.3923
Fixed modifications: Carbamidomethyl (C) (apply to specified residues or termini only)
Ions Score: 44 **Expect:** 0.004
Matches : 9/50 fragment ions using 16 most intense peaks (help)

#	b	b++	b0	b0++	Seq.	y	y++	y*	y*++	y0	y0++	#
1	114.0913	57.5493			L							7
2	**185.1285**	93.0679			A	***694.3155***	347.6614	677.2889	339.1481	676.3049	338.6561	6
3	256.1656	128.5864			A	***623.2784***	312.1428	606.2518	303.6295	605.2678	303.1375	5
4	371.1925	186.0999	353.1819	177.0946	D	***552.2413***	276.6243	535.2147	268.1110	534.2307	267.6190	4
5	486.2195	243.6134	468.2089	234.6081	D	***437.2143***	219.1108	420.1878	210.5975	419.2037	210.1055	3
6	633.2879	317.1476	615.2773	308.1423	F	***322.1874***	161.5973	305.1608	153.0840			2

7					R	175.1190	88.0631	158.0924	79.5498			1

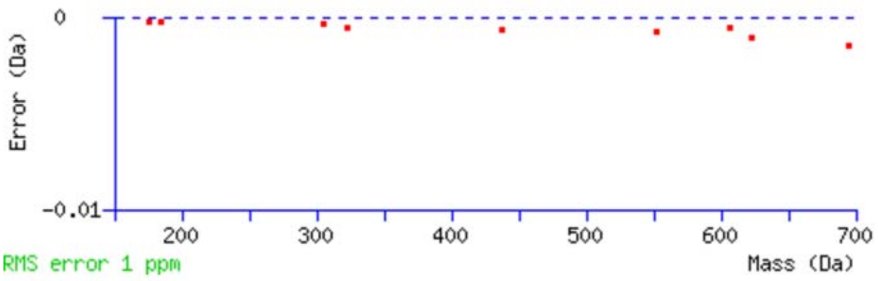

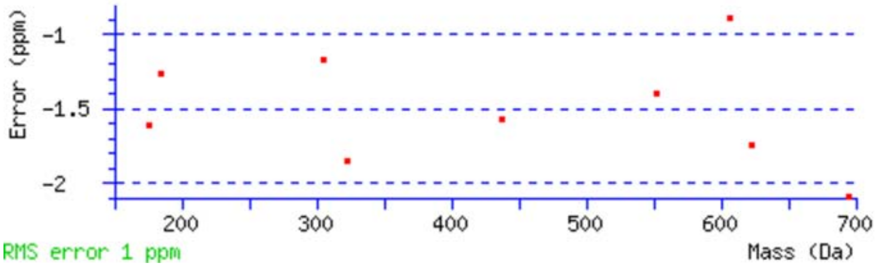

NCBI **BLAST** search of LAADDFR
(Parameters: blastp, nr protein database, expect=20000, no filter, PAM30)
Other BLAST web gateways

All matches to this query

Score	Mr(calc)	Delta	Sequence
44.5	806.3923	-0.0000	LAADDFR
13.3	804.3878	2.0044	RPDNFR
12.7	806.3844	0.0079	LAAQMQK
10.5	806.3923	-0.0000	IAGFDER
6.3	804.3878	2.0044	NRPDFR
5.5	806.3923	-0.0000	LDQQFR
5.5	806.3923	-0.0000	LDQQFR
4.9	806.3922	0.0000	GELANFR
4.9	806.3923	-0.0000	QLEDFR

Mascot: http://www.matrixscience.com/

Mascot Search Results

Peptide View

MS/MS Fragmentation of **LAADDFR**
Found in **ch17u_O76014|KRT37_HUMAN** in **uni_human**, Keratin, type I cuticular Ha7 OS=Homo sapiens GN=KRT37 PE=3 SV=3

Match to Query 379: 806.392308 from(404.203430,2+) intensity(232525.6719) rtinseconds(1242) scans(6518) index(4900)
Title: 160219_Sunil_KAP_A1_Spectrum055864_scans__6518_RTINSECONDS=1242
Data file L:\\QE_2016\\160219_Sunil_KAP_LKC\\TMgf\\T\\T160219_Sunil_KAP_A1.mgf

Click mouse within plot area to zoom in by factor of two about that point
Or, Plot from 50 to 700 Da Full range
Label all possible matches ○ Label matches used for scoring ◉
Show Y-axis ☐

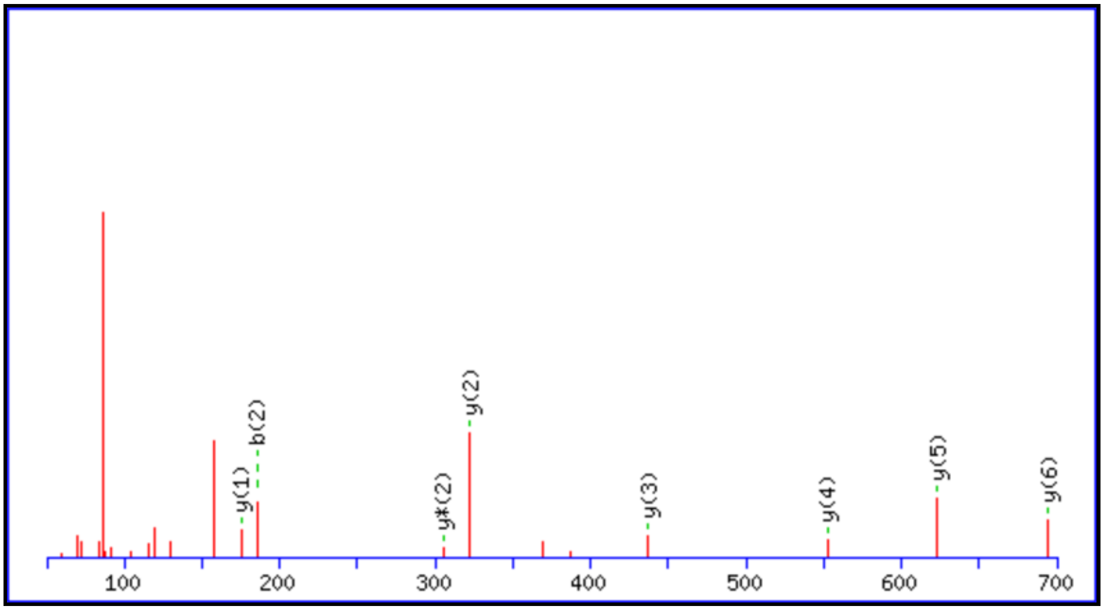

Monoisotopic mass of neutral peptide Mr(calc): 806.3923
Fixed modifications: Carbamidomethyl (C) (apply to specified residues or termini only)
Ions Score: 53 **Expect:** 0.00054
Matches : 8/50 fragment ions using 11 most intense peaks (help)

#	b	b^{++}	b^{0}	b^{0++}	Seq.	y	y^{++}	y*	y^{*++}	y^{0}	y^{0++}	#
1	114.0913	57.5493			L							7
2	**185.1285**	93.0679			A	***694.3155***	347.6614	677.2889	339.1481	676.3049	338.6561	6
3	256.1656	128.5864			A	***623.2784***	312.1428	606.2518	303.6295	605.2678	303.1375	5
4	371.1925	186.0999	353.1819	177.0946	D	***552.2413***	276.6243	535.2147	268.1110	534.2307	267.6190	4
5	486.2195	243.6134	468.2089	234.6081	D	***437.2143***	219.1108	420.1878	210.5975	419.2037	210.1055	3
6	633.2879	317.1476	615.2773	308.1423	F	***322.1874***	161.5973	305.1608	153.0840			2

7					R	*175.1190*	88.0631	158.0924	79.5498			1

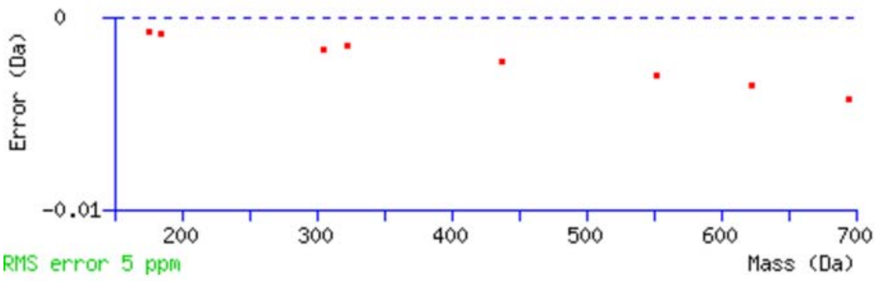

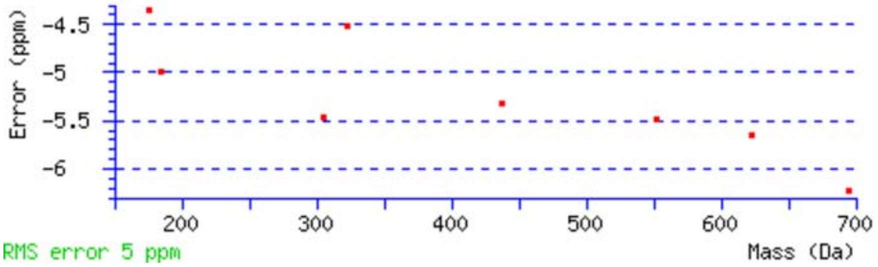

NCBI **BLAST** search of LAADDFR
(Parameters: blastp, nr protein database, expect=20000, no filter, PAM30)
Other BLAST web gateways

All matches to this query

Score	Mr(calc)	Delta	Sequence
53.3	806.3923	0.0001	LAADDFR
19.2	804.3878	2.0045	RPDNFR
13.9	806.3844	0.0079	LAAQMQK
8.8	806.3923	0.0001	LDQQFR
8.8	806.3923	0.0001	LDQQFR
8.4	806.3922	0.0001	GELANFR
8.4	804.3878	2.0045	NRPDFR
8.4	806.3923	0.0001	QLEDFR
8.3	806.3923	0.0001	IAGFDER
3.2	806.3891	0.0032	IACCRK

Mascot: http://www.matrixscience.com/

Mascot Search Results

Peptide View

MS/MS Fragmentation of **LAADDFR**
Found in **ch17u_O76014|KRT37_HUMAN** in **uni_human**, Keratin, type I cuticular Ha7 OS=Homo sapiens GN=KRT37 PE=3 SV=3

Match to Query 380: 806.392308 from(404.203430,2+) intensity(11950775.0000) rtinseconds(1032) scans(5448) index(18435)
Title: 160219_Sunil_KAP_A1_Spectrum070884_scans__5448_RTINSECONDS=1032
Data file L:\\QE_2016\\160219_Sunil_KAP_LKC\\TMgf\\T\\T160219_Sunil_KAP_A1.mgf

Click mouse within plot area to zoom in by factor of two about that point
Or, Plot from 50 to 850 Da Full range
Label all possible matches ○ Label matches used for scoring ◉
Show Y-axis ☐

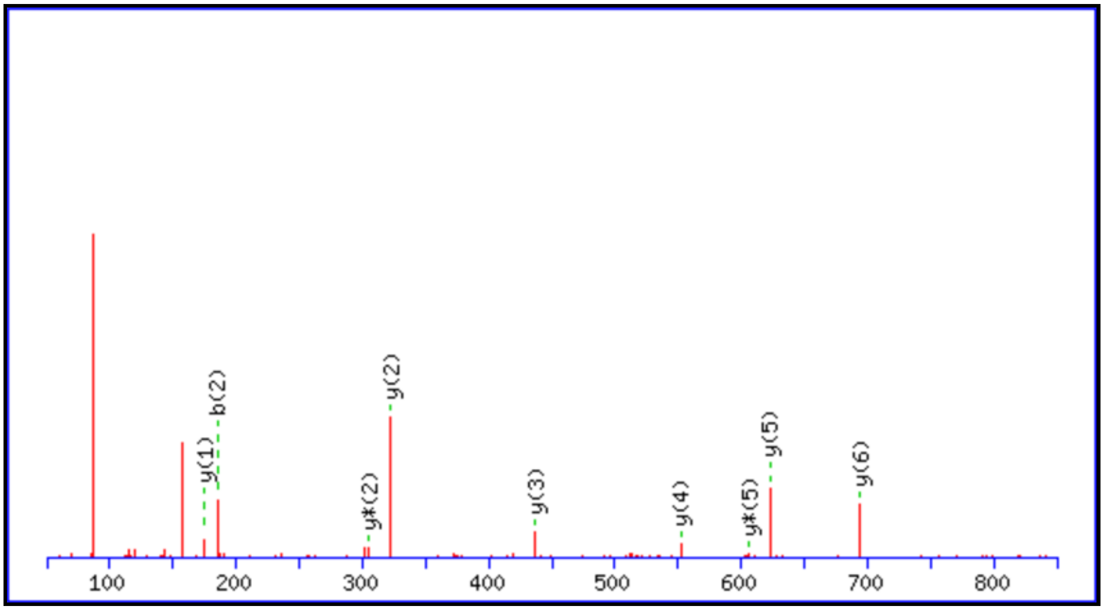

Monoisotopic mass of neutral peptide Mr(calc): 806.3923
Fixed modifications: Carbamidomethyl (C) (apply to specified residues or termini only)
Ions Score: 45 **Expect:** 0.0041
Matches : 9/50 fragment ions using 16 most intense peaks (help)

#	b	b^{++}	b^{0}	b^{0++}	Seq.	y	y^{++}	y*	y^{*++}	y^{0}	y^{0++}	#
1	114.0913	57.5493			L							7
2	**185.1285**	93.0679			A	***694.3155***	347.6614	677.2889	339.1481	676.3049	338.6561	6
3	256.1656	128.5864			A	***623.2784***	312.1428	606.2518	303.6295	605.2678	303.1375	5
4	371.1925	186.0999	353.1819	177.0946	D	***552.2413***	276.6243	535.2147	268.1110	534.2307	267.6190	4
5	486.2195	243.6134	468.2089	234.6081	D	***437.2143***	219.1108	420.1878	210.5975	419.2037	210.1055	3
6	633.2879	317.1476	615.2773	308.1423	F	***322.1874***	161.5973	305.1608	153.0840			2

7					R	*175.1190*	88.0631	158.0924	79.5498			1

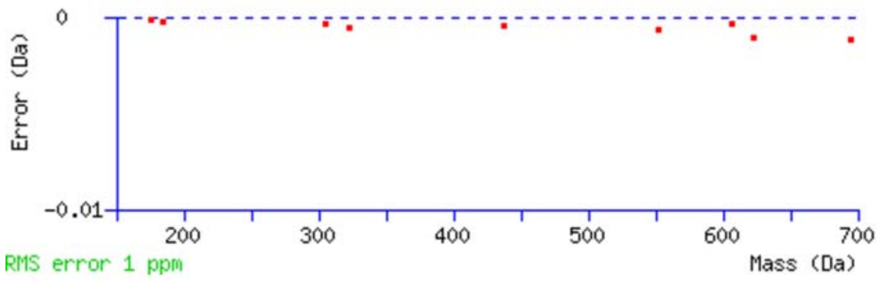

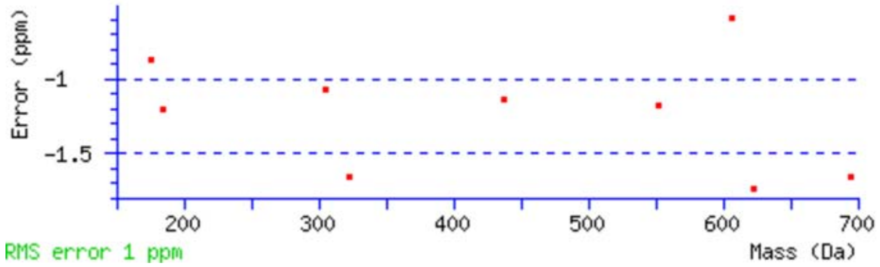

NCBI **BLAST** search of LAADDFR
(Parameters: blastp, nr protein database, expect=20000, no filter, PAM30)
Other BLAST web gateways

All matches to this query

Score	Mr(calc)	Delta	Sequence
44.5	806.3923	0.0001	LAADDFR
13.2	804.3878	2.0045	RPDNFR
12.8	806.3844	0.0079	LAAQMQK
6.3	806.3923	0.0001	IAGFDER
5.5	806.3923	0.0001	LDQQFR
5.5	806.3923	0.0001	LDQQFR
4.9	806.3922	0.0001	GELANFR
4.9	804.3878	2.0045	NRPDFR
4.9	806.3923	0.0001	QLEDFR

Mascot: http://www.matrixscience.com/

Mascot Search Results

Peptide View

MS/MS Fragmentation of **LAADDFR**
Found in **ch17u_O76014|KRT37_HUMAN** in **uni_human**, Keratin, type I cuticular Ha7 OS=Homo sapiens GN=KRT37 PE=3 SV=3

Match to Query 383: 806.393228 from(404.203890,2+) intensity(142941.6406) rtinseconds(1199) scans(6407) index(19241)
Title: 160219_Sunil_KAP_A1_Spectrum071690_scans__6407_RTINSECONDS=1199
Data file L:\\QE_2016\\160219_Sunil_KAP_LKC\\TMgf\\T\\T160219_Sunil_KAP_A1.mgf

Click mouse within plot area to zoom in by factor of two about that point
Or, Plot from 50 to 750 Da Full range
Label all possible matches ○ Label matches used for scoring ◉
Show Y-axis ☐

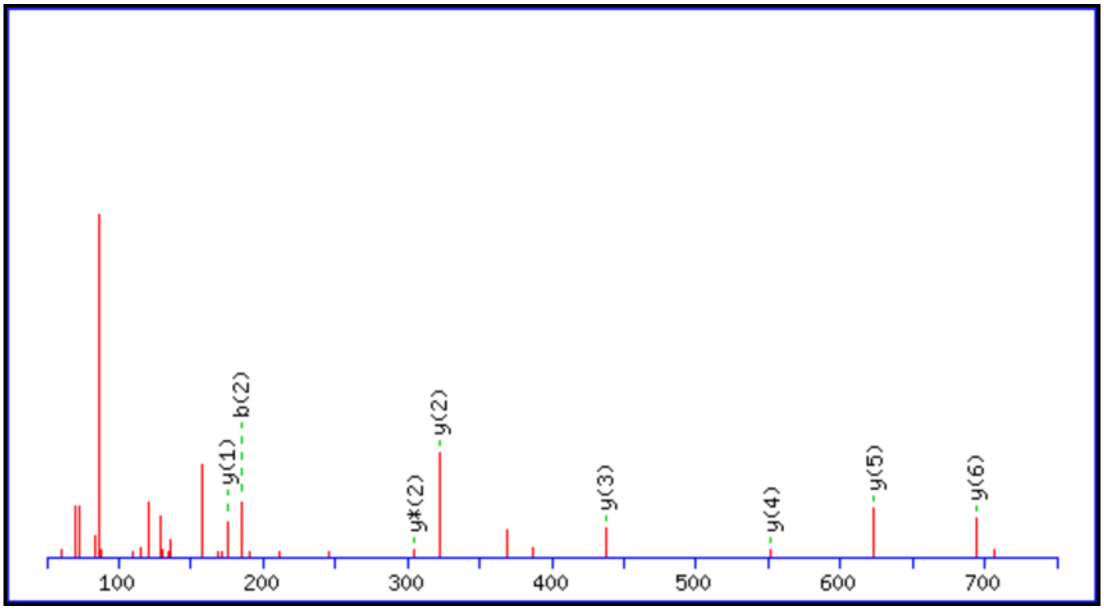

Monoisotopic mass of neutral peptide Mr(calc): 806.3923
Fixed modifications: Carbamidomethyl (C) (apply to specified residues or termini only)
Ions Score: 49 **Expect:** 0.0011
Matches : 8/50 fragment ions using 12 most intense peaks (help)

#	b	b^{++}	b^0	b^{0++}	Seq.	y	y^{++}	y*	y^{*++}	y^0	y^{0++}	#
1	114.0913	57.5493			L							7
2	**185.1285**	93.0679			A	***694.3155***	347.6614	677.2889	339.1481	676.3049	338.6561	6
3	256.1656	128.5864			A	***623.2784***	312.1428	606.2518	303.6295	605.2678	303.1375	5
4	371.1925	186.0999	353.1819	177.0946	D	***552.2413***	276.6243	535.2147	268.1110	534.2307	267.6190	4
5	486.2195	243.6134	468.2089	234.6081	D	***437.2143***	219.1108	420.1878	210.5975	419.2037	210.1055	3
6	633.2879	317.1476	615.2773	308.1423	F	***322.1874***	161.5973	305.1608	153.0840			2

7					R	*175.1190*	88.0631	158.0924	79.5498			1

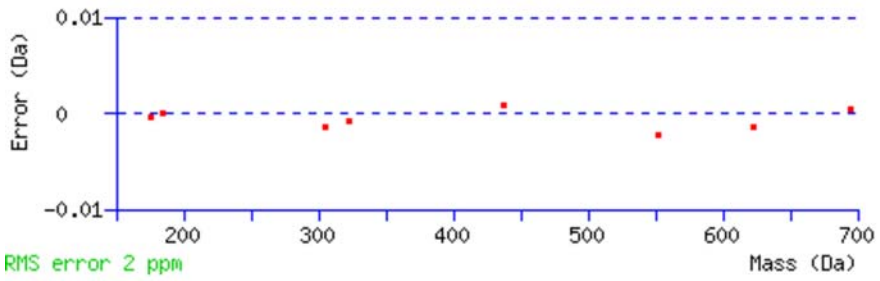

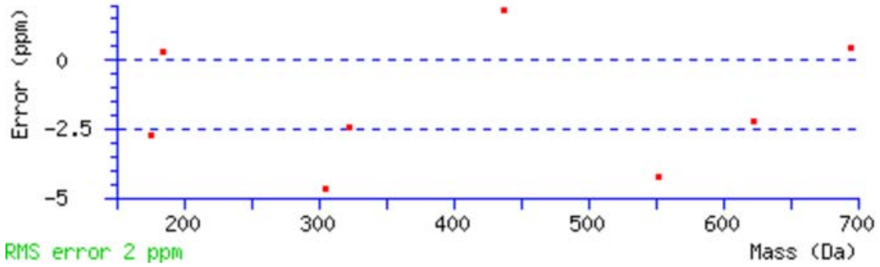

NCBI **BLAST** search of LAADDFR
(Parameters: blastp, nr protein database, expect=20000, no filter, PAM30)
Other BLAST web gateways

All matches to this query

Score	Mr(calc)	Delta	Sequence
49.5	806.3923	0.0010	LAADDFR
16.7	804.3878	2.0054	RPDNFR
7.0	806.3923	0.0010	LDQQFR
7.0	806.3923	0.0010	LDQQFR
6.8	806.3923	0.0010	IAGFDER
6.7	806.3922	0.0010	GELANFR
6.7	804.3878	2.0054	NRPDFR
6.7	806.3923	0.0010	QLEDFR
2.5	806.3891	0.0041	IACCRK
2.5	806.3956	-0.0024	LACTNTK

Mascot: http://www.matrixscience.com/

Mascot Search Results

Peptide View

MS/MS Fragmentation of **LAADDFR**
Found in **ch17u_O76014|KRT37_HUMAN** in **uni_human**, Keratin, type I cuticular Ha7 OS=Homo sapiens GN=KRT37 PE=3 SV=3

Match to Query 383: 806.393228 from(404.203890,2+) intensity(142941.6406) rtinseconds(1199) scans(6407) index(19241)
Title: 160219_Sunil_KAP_A1_Spectrum071690_scans__6407_RTINSECONDS=1199
Data file L:\\QE_2016\\160219_Sunil_KAP_LKC\\TMgf\\T\\T160219_Sunil_KAP_A1.mgf

Click mouse within plot area to zoom in by factor of two about that point
Or, Plot from 50 to 750 Da Full range
Label all possible matches ○ Label matches used for scoring ◉
Show Y-axis ☐

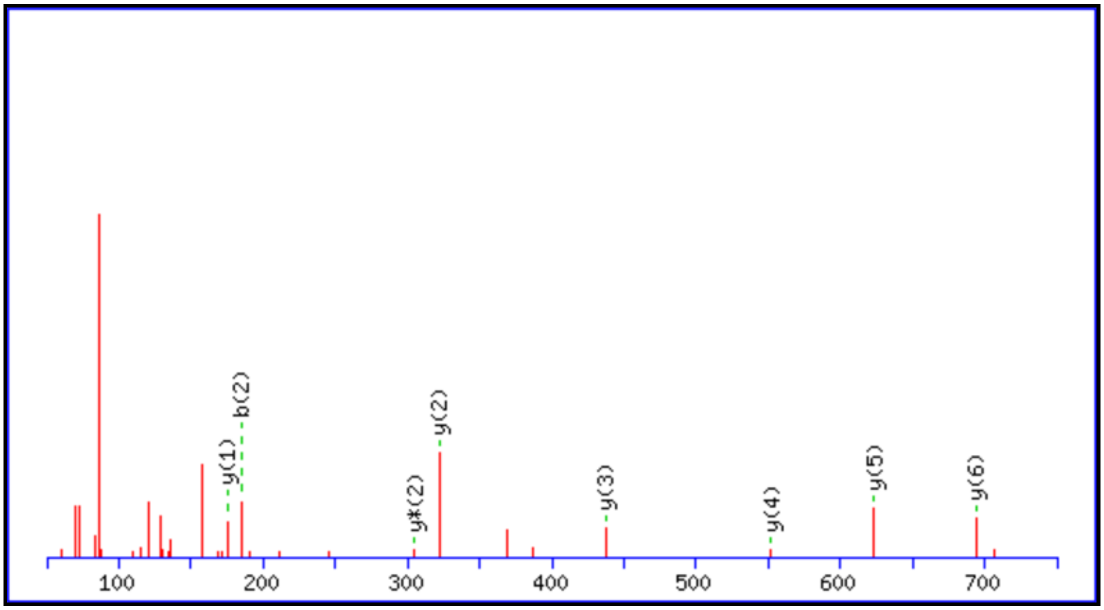

Monoisotopic mass of neutral peptide Mr(calc): 806.3923
Fixed modifications: Carbamidomethyl (C) (apply to specified residues or termini only)
Ions Score: 49 **Expect:** 0.0011
Matches : 8/50 fragment ions using 12 most intense peaks (help)

#	b	b^{++}	b^0	b^{0++}	Seq.	y	y^{++}	y*	y^{*++}	y^0	y^{0++}	#
1	114.0913	57.5493			L							7
2	**185.1285**	93.0679			A	***694.3155***	347.6614	677.2889	339.1481	676.3049	338.6561	6
3	256.1656	128.5864			A	***623.2784***	312.1428	606.2518	303.6295	605.2678	303.1375	5
4	371.1925	186.0999	353.1819	177.0946	D	***552.2413***	276.6243	535.2147	268.1110	534.2307	267.6190	4
5	486.2195	243.6134	468.2089	234.6081	D	***437.2143***	219.1108	420.1878	210.5975	419.2037	210.1055	3
6	633.2879	317.1476	615.2773	308.1423	F	***322.1874***	161.5973	305.1608	153.0840			2

7					R	*175.1190*	88.0631	158.0924	79.5498			1

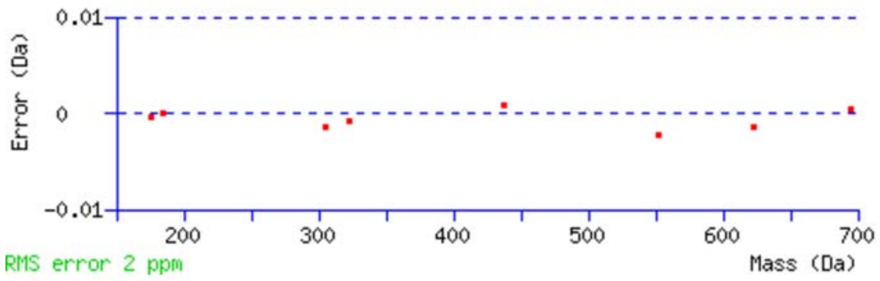

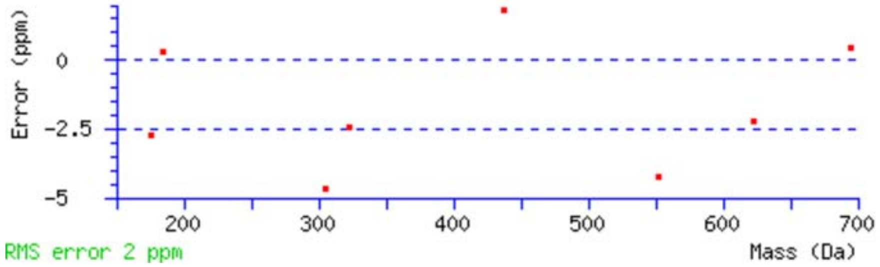

NCBI **BLAST** search of LAADDFR
(Parameters: blastp, nr protein database, expect=20000, no filter, PAM30)
Other BLAST web gateways

All matches to this query

Score	Mr(calc)	Delta	Sequence
49.5	806.3923	0.0010	LAADDFR
16.7	804.3878	2.0054	RPDNFR
7.0	806.3923	0.0010	LDQQFR
7.0	806.3923	0.0010	LDQQFR
6.8	806.3923	0.0010	IAGFDER
6.7	806.3922	0.0010	GELANFR
6.7	804.3878	2.0054	NRPDFR
6.7	806.3923	0.0010	QLEDFR
2.5	806.3891	0.0041	IACCRK
2.5	806.3956	-0.0024	LACTNTK

Mascot: http://www.matrixscience.com/

Mascot Search Results

Peptide View

MS/MS Fragmentation of **LAADDFR**
Found in **ch17u_O76014|KRT37_HUMAN** in **uni_human**, Keratin, type I cuticular Ha7 OS=Homo sapiens GN=KRT37 PE=3 SV=3

Match to Query 384: 806.393288 from(404.203920,2+) intensity(435034.6875) rtinseconds(1095) scans(5812) index(18722)
Title: 160219_Sunil_KAP_A1_Spectrum071171_scans__5812_RTINSECONDS=1095
Data file L:\\QE_2016\\160219_Sunil_KAP_LKC\\TMgf\\T\\T160219_Sunil_KAP_A1.mgf

Click mouse within plot area to zoom in by factor of two about that point
Or, Plot from 50 to 700 Da Full range
Label all possible matches ○ Label matches used for scoring ◉
Show Y-axis ☐

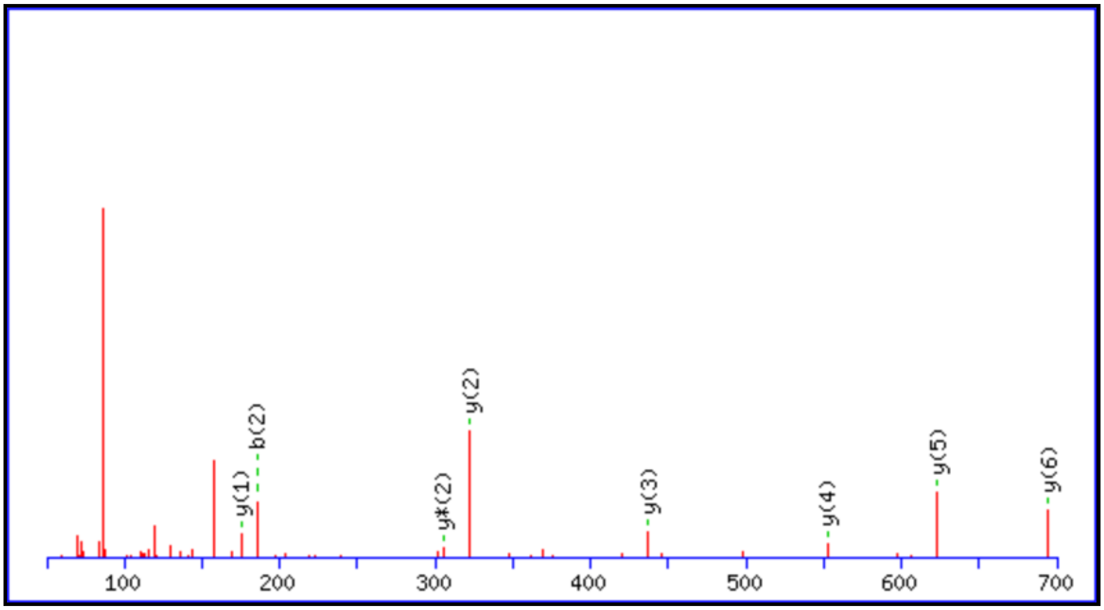

Monoisotopic mass of neutral peptide Mr(calc): 806.3923
Fixed modifications: Carbamidomethyl (C) (apply to specified residues or termini only)
Ions Score: 48 **Expect:** 0.0016
Matches : 8/50 fragment ions using 13 most intense peaks (help)

#	b	b^{++}	b^0	b^{0++}	Seq.	y	y^{++}	y*	y^{*++}	y^0	y^{0++}	#
1	114.0913	57.5493			L							7
2	**185.1285**	93.0679			A	***694.3155***	347.6614	677.2889	339.1481	676.3049	338.6561	6
3	256.1656	128.5864			A	***623.2784***	312.1428	606.2518	303.6295	605.2678	303.1375	5
4	371.1925	186.0999	353.1819	177.0946	D	***552.2413***	276.6243	535.2147	268.1110	534.2307	267.6190	4
5	486.2195	243.6134	468.2089	234.6081	D	***437.2143***	219.1108	420.1878	210.5975	419.2037	210.1055	3
6	633.2879	317.1476	615.2773	308.1423	F	***322.1874***	161.5973	305.1608	153.0840			2

7					R	*175.1190*	88.0631	158.0924	79.5498			1

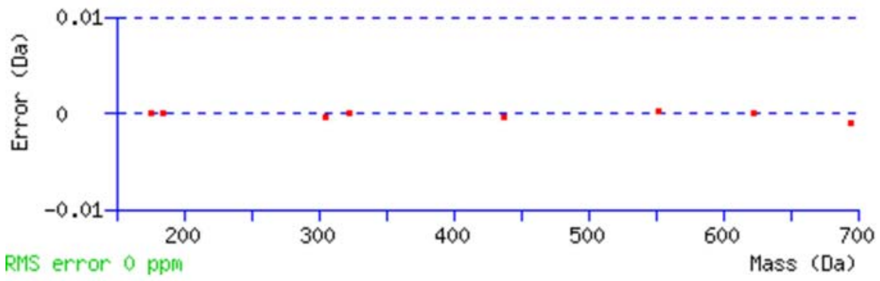

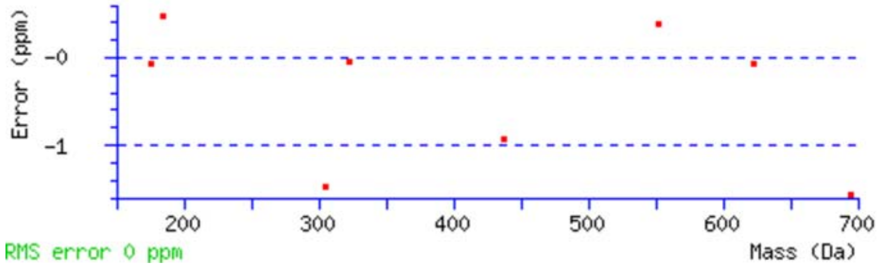

NCBI **BLAST** search of LAADDFR
(Parameters: blastp, nr protein database, expect=20000, no filter, PAM30)
Other BLAST web gateways

All matches to this query

Score	Mr(calc)	Delta	Sequence
47.9	806.3923	0.0010	LAADDFR
16.3	804.3878	2.0055	RPDNFR
7.1	806.3923	0.0010	LDQQFR
7.1	806.3923	0.0010	LDQQFR
6.8	806.3923	0.0010	IAGFDER
6.6	806.3922	0.0010	GELANFR
6.6	804.3878	2.0055	NRPDFR
6.6	806.3923	0.0010	QLEDFR
3.6	806.3891	0.0042	IACCRK
3.6	806.3956	-0.0023	LACTNTK

Mascot: http://www.matrixscience.com/

Mascot Search Results

Peptide View

MS/MS Fragmentation of **LAADDFR**
Found in **ch17u_O76014|KRT37_HUMAN** in **uni_human**, Keratin, type I cuticular Ha7 OS=Homo sapiens GN=KRT37 PE=3 SV=3

Match to Query 384: 806.393288 from(404.203920,2+) intensity(435034.6875) rtinseconds(1095) scans(5812) index(18722)
Title: 160219_Sunil_KAP_A1_Spectrum071171_scans__5812_RTINSECONDS=1095
Data file L:\\QE_2016\\160219_Sunil_KAP_LKC\\TMgf\\T\\T160219_Sunil_KAP_A1.mgf

Click mouse within plot area to zoom in by factor of two about that point
Or, Plot from 50 to 700 Da Full range
Label all possible matches ○ Label matches used for scoring ◉
Show Y-axis ☐

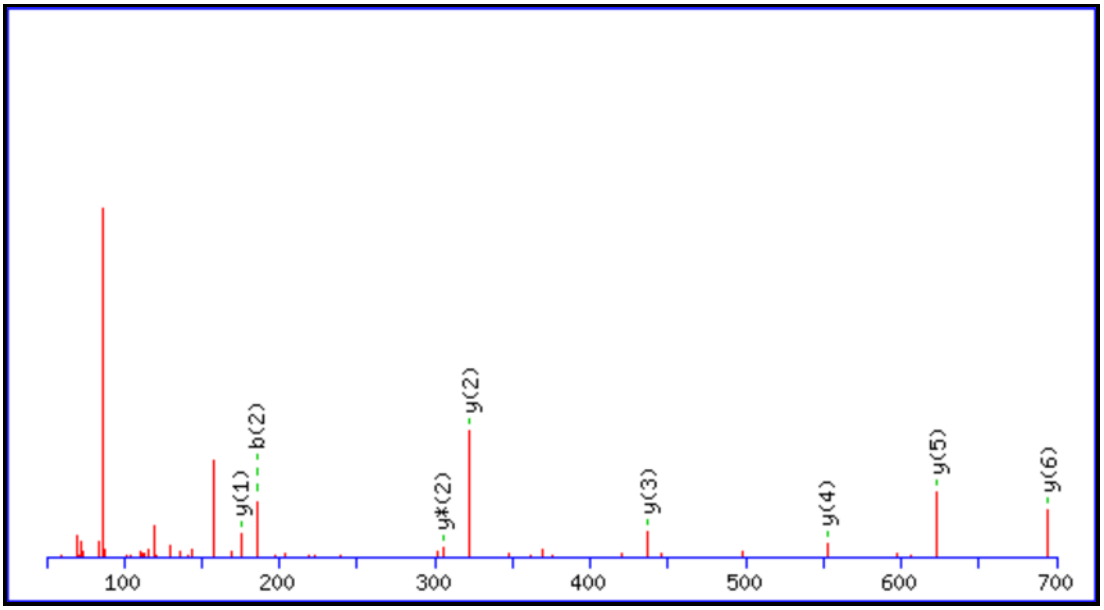

Monoisotopic mass of neutral peptide Mr(calc): 806.3923
Fixed modifications: Carbamidomethyl (C) (apply to specified residues or termini only)
Ions Score: 48 **Expect:** 0.0016
Matches : 8/50 fragment ions using 13 most intense peaks (help)

#	b	b^{++}	b^{0}	b^{0++}	Seq.	y	y^{++}	y*	y^{*++}	y^{0}	y^{0++}	#
1	114.0913	57.5493			L							7
2	**185.1285**	93.0679			A	***694.3155***	347.6614	677.2889	339.1481	676.3049	338.6561	6
3	256.1656	128.5864			A	***623.2784***	312.1428	606.2518	303.6295	605.2678	303.1375	5
4	371.1925	186.0999	353.1819	177.0946	D	***552.2413***	276.6243	535.2147	268.1110	534.2307	267.6190	4
5	486.2195	243.6134	468.2089	234.6081	D	***437.2143***	219.1108	420.1878	210.5975	419.2037	210.1055	3
6	633.2879	317.1476	615.2773	308.1423	F	***322.1874***	161.5973	305.1608	153.0840			2

7					R	*175.1190*	88.0631	158.0924	79.5498			1

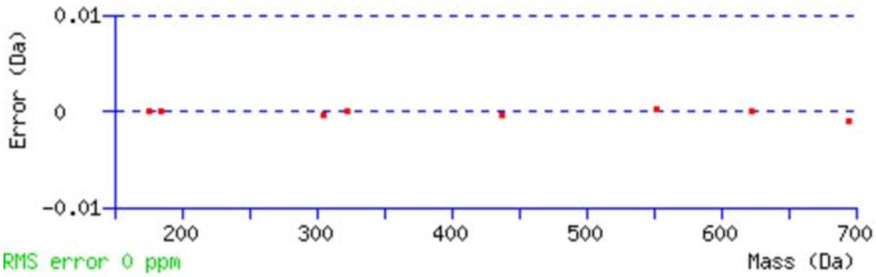

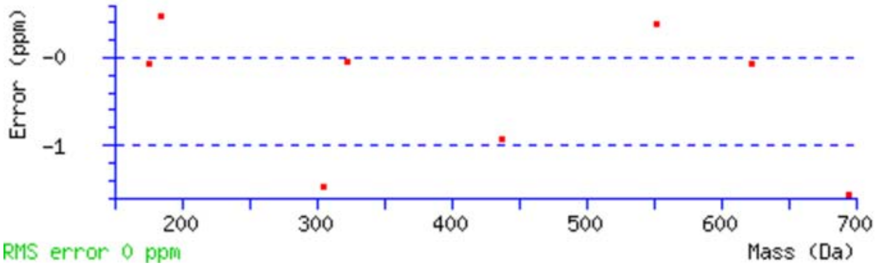

NCBI **BLAST** search of LAADDFR
(Parameters: blastp, nr protein database, expect=20000, no filter, PAM30)
Other BLAST web gateways

All matches to this query

Score	Mr(calc)	Delta	Sequence
47.9	806.3923	0.0010	LAADDFR
16.3	804.3878	2.0055	RPDNFR
7.1	806.3923	0.0010	LDQQFR
7.1	806.3923	0.0010	LDQQFR
6.8	806.3923	0.0010	IAGFDER
6.6	806.3922	0.0010	GELANFR
6.6	804.3878	2.0055	NRPDFR
6.6	806.3923	0.0010	QLEDFR
3.6	806.3891	0.0042	IACCRK
3.6	806.3956	-0.0023	LACTNTK

Mascot: http://www.matrixscience.com/

Mascot Search Results

Peptide View

MS/MS Fragmentation of **TIEELQQKILCSK**
Found in **ch17u_O76014|KRT37_HUMAN** in **uni_human**, Keratin, type I cuticular Ha7 OS=Homo sapiens GN=KRT37 PE=3 SV=3

Match to Query 17617: 1588.849872 from(530.623900,3+) intensity(446856.4688) rtinseconds(1998) scans(10864) index(8423)
Title: 160219_Sunil_KAP_A1_Spectrum059394_scans__10864_RTINSECONDS=1998
Data file L:\\QE_2016\\160219_Sunil_KAP_LKC\\TMgf\\T\\T160219_Sunil_KAP_A1.mgf

Click mouse within plot area to zoom in by factor of two about that point
Or, Plot from 100 to 1600 Da Full range
Label all possible matches ○ Label matches used for scoring ◉
Show Y-axis ☐

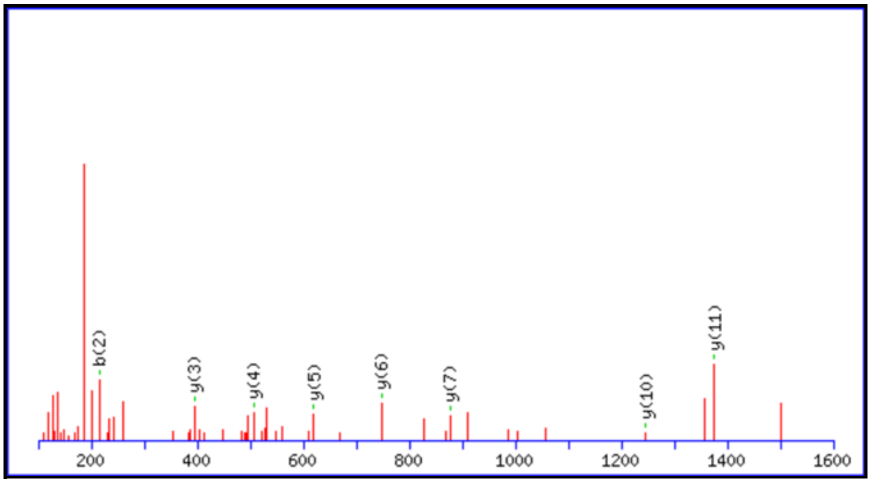

Monoisotopic mass of neutral peptide Mr(calc): 1588.8494
Fixed modifications: Carbamidomethyl (C) (apply to specified residues or termini only)
Ions Score: 46 **Expect:** 0.0039
Matches : 8/132 fragment ions using 13 most intense peaks (help)

#	b	b^{++}	b*	b^{*++}	b^0	b^{0++}	Seq.	y	y^{++}	y*	y^{*++}	y^0	y^{0++}	#
1	102.0550	51.5311			84.0444	42.5258	T							13
2	**215.1390**	108.0731			197.1285	99.0679	I	1488.8090	744.9082	1471.7825	736.3949	1470.7985	735.9029	12
3	344.1816	172.5944			326.1710	163.5892	E	***1375.7250***	688.3661	1358.6984	679.8529	1357.7144	679.3608	11
4	473.2242	237.1157			455.2136	228.1105	E	***1246.6824***	623.8448	1229.6558	615.3316	1228.6718	614.8395	10
5	586.3083	293.6578			568.2977	284.6525	L	1117.6398	559.3235	1100.6132	550.8103	1099.6292	550.3183	9
6	714.3668	357.6871	697.3403	349.1738	696.3563	348.6818	Q	1004.5557	502.7815	987.5292	494.2682	986.5452	493.7762	8
7	842.4254	421.7164	825.3989	413.2031	824.4149	412.7111	Q	***876.4972***	438.7522	859.4706	430.2389	858.4866	429.7469	7
8	970.5204	485.7638	953.4938	477.2506	952.5098	476.7585	K	***748.4386***	374.7229	731.4120	366.2096	730.4280	365.7176	6
9	1083.6045	542.3059	1066.5779	533.7926	1065.5939	533.3006	I	***620.3436***	310.6754	603.3171	302.1622	602.3330	301.6702	5
10	1196.6885	598.8479	1179.6620	590.3346	1178.6780	589.8426	L	***507.2595***	254.1334	490.2330	245.6201	489.2490	245.1281	4
11	1356.7192	678.8632	1339.6926	670.3499	1338.7086	669.8579	C	***394.1755***	197.5914	377.1489	189.0781	376.1649	188.5861	3
12	1443.7512	722.3792	1426.7246	713.8660	1425.7406	713.3740	S	234.1448	117.5761	217.1183	109.0628	216.1343	108.5708	2
13							K	147.1128	74.0600	130.0863	65.5468			1

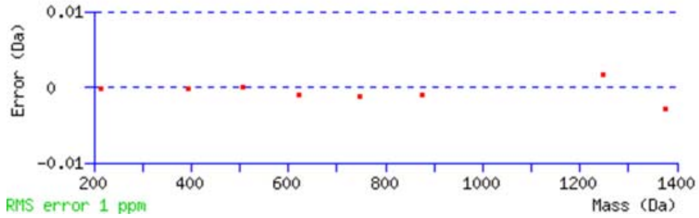

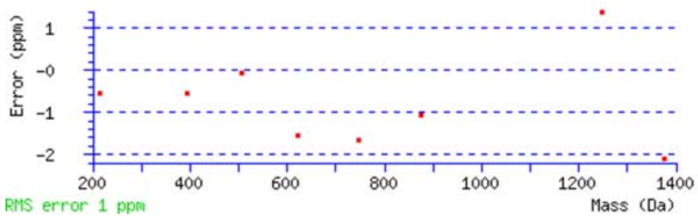

NCBI **BLAST** search of TIEELQQKILCSK
(Parameters: blastp, nr protein database, expect=20000, no filter, PAM30)
Other BLAST web gateways

All matches to this query

Score	Mr(calc)	Delta	Sequence
45.8	1588.8494	0.0005	TIEELQQKILCSK

Mascot: http://www.matrixscience.com/

Mascot Search Results

Peptide View

MS/MS Fragmentation of **LAADDFR**
Found in **ch17u_O76014|KRT37_HUMAN** in **uni_human**, Keratin, type I cuticular Ha7 OS=Homo sapiens GN=KRT37 PE=3 SV=3

Match to Query 407: 806.390728 from(404.202640,2+) intensity(123577.6172) rtinseconds(1148) scans(5721) index(18250)
Title: 160219_Sunil_KAP_B_Spectrum068106_scans__5721_RTINSECONDS=1148
Data file L:\\QE_2016\\160219_Sunil_KAP_LKC\\TMgf\\T\\T160219_Sunil_KAP_B.mgf

Click mouse within plot area to zoom in by factor of two about that point
Or, Plot from 50 to 750 Da Full range
Label all possible matches ○ Label matches used for scoring ◉
Show Y-axis ☐

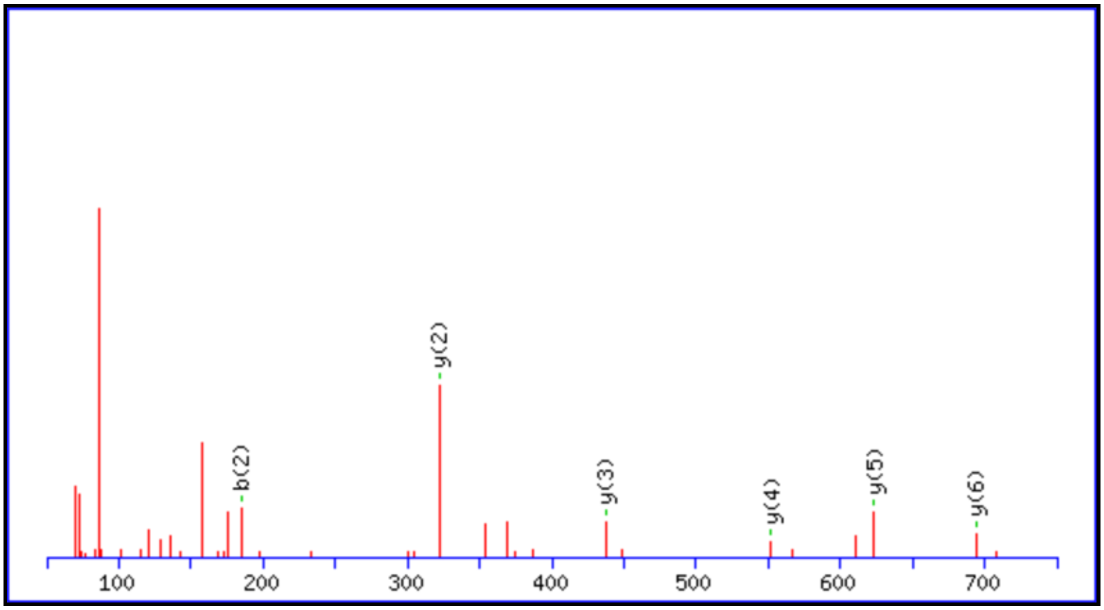

Monoisotopic mass of neutral peptide Mr(calc): 806.3923
Fixed modifications: Carbamidomethyl (C) (apply to specified residues or termini only)
Ions Score: 46 **Expect:** 0.0032
Matches : 6/50 fragment ions using 7 most intense peaks (help)

#	b	b^{++}	b^0	b^{0++}	Seq.	y	y^{++}	y*	y^{*++}	y^0	y^{0++}	#
1	114.0913	57.5493			L							7
2	**185.1285**	93.0679			A	***694.3155***	347.6614	677.2889	339.1481	676.3049	338.6561	6
3	256.1656	128.5864			A	***623.2784***	312.1428	606.2518	303.6295	605.2678	303.1375	5
4	371.1925	186.0999	353.1819	177.0946	D	***552.2413***	276.6243	535.2147	268.1110	534.2307	267.6190	4
5	486.2195	243.6134	468.2089	234.6081	D	***437.2143***	219.1108	420.1878	210.5975	419.2037	210.1055	3
6	633.2879	317.1476	615.2773	308.1423	F	***322.1874***	161.5973	305.1608	153.0840			2

7					R	175.1190	88.0631	158.0924	79.5498			1

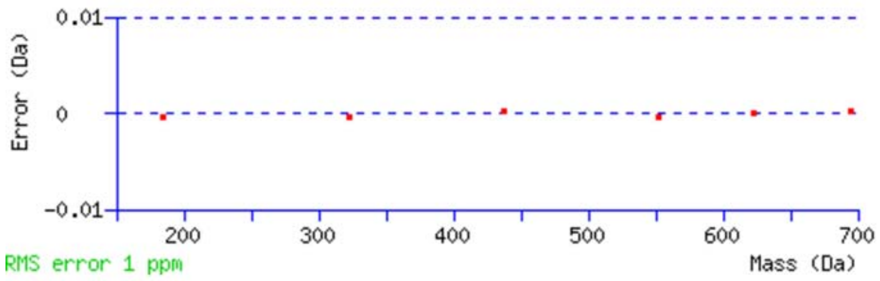

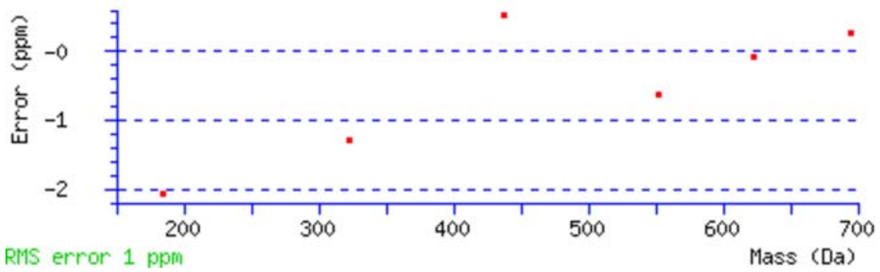

NCBI **BLAST** search of LAADDFR
(Parameters: blastp, nr protein database, expect=20000, no filter, PAM30)
Other BLAST web gateways

All matches to this query

Score	Mr(calc)	Delta	Sequence
45.7	806.3923	-0.0015	LAADDFR
13.1	804.3878	2.0029	RPDNFR
11.8	806.3844	0.0064	LAAQMQK
6.0	804.3919	1.9989	PWQAFR
4.7	806.3922	-0.0015	GELANFR
4.7	804.3878	2.0029	NRPDFR
4.7	806.3923	-0.0015	QLEDFR
4.5	806.3923	-0.0015	LDQQFR
4.5	806.3923	-0.0015	LDQQFR
4.3	806.3923	-0.0015	IAGFDER

Mascot: http://www.matrixscience.com/

Mascot Search Results

Peptide View

MS/MS Fragmentation of **LAADDFR**
Found in **ch17u_O76014|KRT37_HUMAN** in **uni_human**, Keratin, type I cuticular Ha7 OS=Homo sapiens GN=KRT37 PE=3 SV=3

Match to Query 409: 806.391568 from(404.203060,2+) intensity(99124.8906) rtinseconds(1255) scans(6339) index(18722)
Title: 160219_Sunil_KAP_B_Spectrum068582_scans__6339_RTINSECONDS=1255
Data file L:\\QE_2016\\160219_Sunil_KAP_LKC\\TMgf\\T\\T160219_Sunil_KAP_B.mgf

Click mouse within plot area to zoom in by factor of two about that point
Or, Plot from 50 to 700 Da Full range
Label all possible matches ○ Label matches used for scoring ◉
Show Y-axis ☐

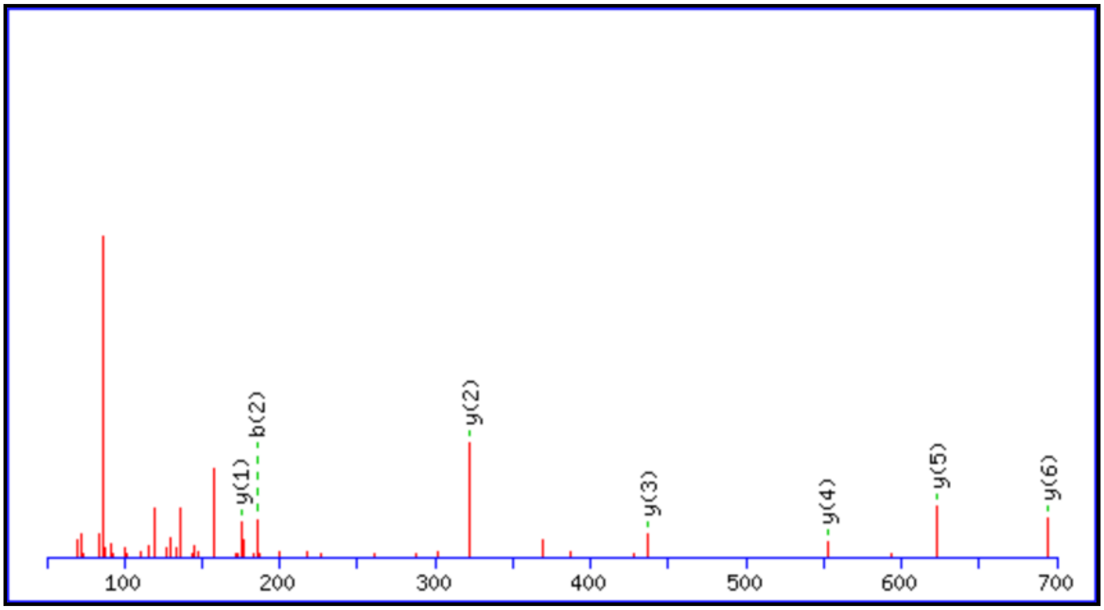

Monoisotopic mass of neutral peptide Mr(calc): 806.3923
Fixed modifications: Carbamidomethyl (C) (apply to specified residues or termini only)
Ions Score: 46 **Expect:** 0.0026
Matches : 7/50 fragment ions using 12 most intense peaks (help)

#	b	b^{++}	b^0	b^{0++}	Seq.	y	y^{++}	y*	y^{*++}	y^0	y^{0++}	#
1	114.0913	57.5493			L							7
2	**185.1285**	93.0679			A	***694.3155***	347.6614	677.2889	339.1481	676.3049	338.6561	6
3	256.1656	128.5864			A	***623.2784***	312.1428	606.2518	303.6295	605.2678	303.1375	5
4	371.1925	186.0999	353.1819	177.0946	D	***552.2413***	276.6243	535.2147	268.1110	534.2307	267.6190	4
5	486.2195	243.6134	468.2089	234.6081	D	***437.2143***	219.1108	420.1878	210.5975	419.2037	210.1055	3
6	633.2879	317.1476	615.2773	308.1423	F	***322.1874***	161.5973	305.1608	153.0840			2

7					R	*175.1190*	88.0631	158.0924	79.5498			1

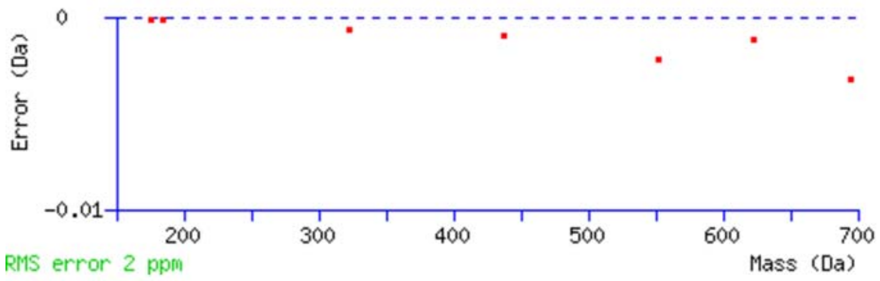

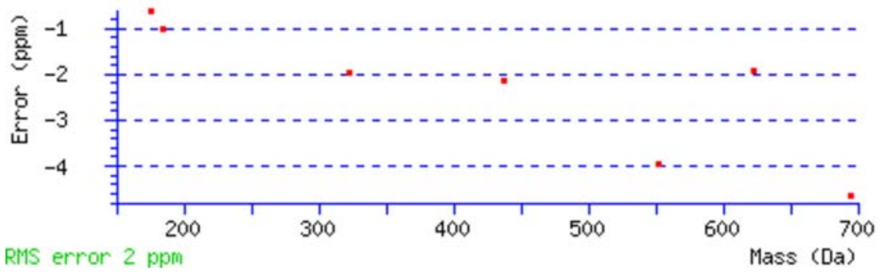

NCBI **BLAST** search of LAADDFR
(Parameters: blastp, nr protein database, expect=20000, no filter, PAM30)
Other BLAST web gateways

All matches to this query

Score	Mr(calc)	Delta	Sequence
46.3	806.3923	-0.0007	LAADDFR
14.8	804.3878	2.0037	RPDNFR
12.9	806.3844	0.0072	LAAQMQK
6.7	806.3923	-0.0007	IAGFDER
5.7	806.3923	-0.0007	LDQQFR
5.7	806.3923	-0.0007	LDQQFR
5.3	806.3922	-0.0007	GELANFR
5.3	804.3878	2.0037	NRPDFR
5.3	806.3923	-0.0007	QLEDFR
2.3	806.3891	0.0025	IACCRK

Mascot: http://www.matrixscience.com/

Mascot Search Results

Peptide View

MS/MS Fragmentation of **LAADDFR**
Found in **ch17u_O76014|KRT37_HUMAN** in **uni_human**, Keratin, type I cuticular Ha7 OS=Homo sapiens GN=KRT37 PE=3 SV=3

Match to Query 410: 806.391768 from(404.203160,2+) intensity(178511.6406) rtinseconds(1529) scans(8046) index(5905)
Title: 160219_Sunil_KAP_B_Spectrum055021_scans__8046_RTINSECONDS=1529
Data file L:\\QE_2016\\160219_Sunil_KAP_LKC\\TMgf\\T\\T160219_Sunil_KAP_B.mgf

Click mouse within plot area to zoom in by factor of two about that point
Or, Plot from 50 to 700 Da Full range
Label all possible matches ○ Label matches used for scoring ◉
Show Y-axis ☐

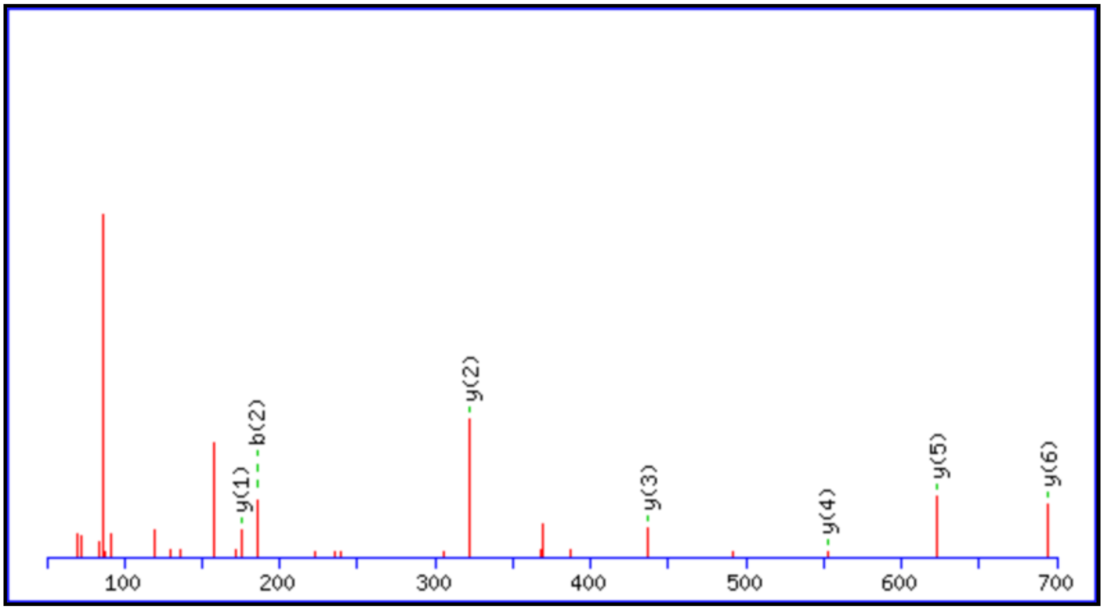

Monoisotopic mass of neutral peptide Mr(calc): 806.3923
Fixed modifications: Carbamidomethyl (C) (apply to specified residues or termini only)
Ions Score: 47 **Expect:** 0.0023
Matches : 7/50 fragment ions using 12 most intense peaks (help)

#	b	b^{++}	b^0	b^{0++}	Seq.	y	y^{++}	y*	y^{*++}	y^0	y^{0++}	#
1	114.0913	57.5493			L							7
2	**185.1285**	93.0679			A	***694.3155***	347.6614	677.2889	339.1481	676.3049	338.6561	6
3	256.1656	128.5864			A	***623.2784***	312.1428	606.2518	303.6295	605.2678	303.1375	5
4	371.1925	186.0999	353.1819	177.0946	D	***552.2413***	276.6243	535.2147	268.1110	534.2307	267.6190	4
5	486.2195	243.6134	468.2089	234.6081	D	***437.2143***	219.1108	420.1878	210.5975	419.2037	210.1055	3
6	633.2879	317.1476	615.2773	308.1423	F	***322.1874***	161.5973	305.1608	153.0840			2

7					R	175.1190	88.0631	158.0924	79.5498			1

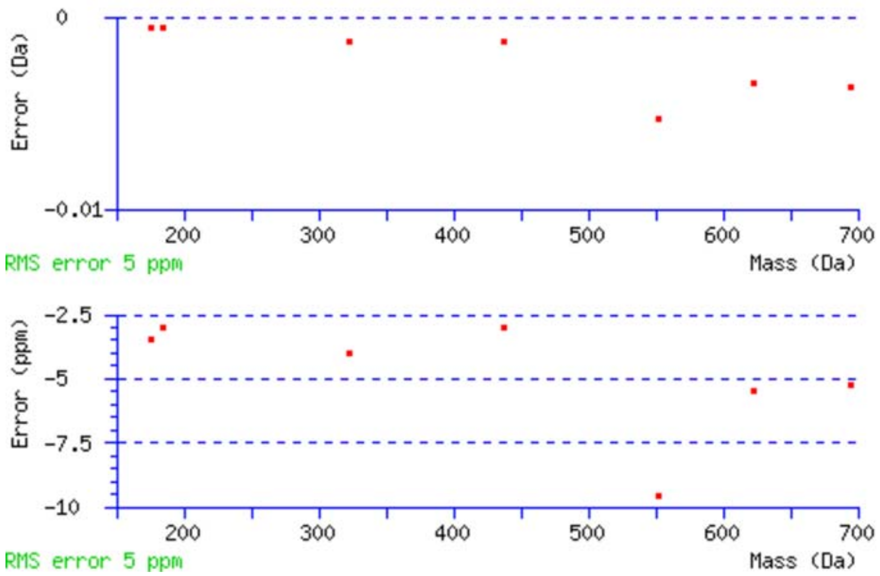

NCBI **BLAST** search of LAADDFR
(Parameters: blastp, nr protein database, expect=20000, no filter, PAM30)
Other BLAST web gateways

All matches to this query

Score	Mr(calc)	Delta	Sequence
47.0	806.3923	-0.0005	LAADDFR
15.0	804.3878	2.0039	RPDNFR
13.8	806.3844	0.0074	LAAQMQK
7.6	806.3923	-0.0005	IAGFDER
6.3	806.3923	-0.0005	LDQQFR
6.3	806.3923	-0.0005	LDQQFR
5.7	806.3922	-0.0005	GELANFR
5.7	804.3878	2.0039	NRPDFR
5.7	806.3923	-0.0005	QLEDFR
3.5	806.3891	0.0027	IACCRK

Mascot: http://www.matrixscience.com/

Mascot Search Results

Peptide View

MS/MS Fragmentation of **LAADDFR**
Found in **ch17u_O76014|KRT37_HUMAN** in **uni_human**, Keratin, type I cuticular Ha7 OS=Homo sapiens GN=KRT37 PE=3 SV=3

Match to Query 411: 806.391828 from(404.203190,2+) intensity(148655.1563) rtinseconds(1411) scans(7235) index(19478)
Title: 160219_Sunil_KAP_B_Spectrum069349_scans__7235_RTINSECONDS=1411
Data file L:\\QE_2016\\160219_Sunil_KAP_LKC\\TMgf\\T\\T160219_Sunil_KAP_B.mgf

Click mouse within plot area to zoom in by factor of two about that point
Or, Plot from 50 to 850 Da Full range
Label all possible matches ○ Label matches used for scoring ◉
Show Y-axis ☐

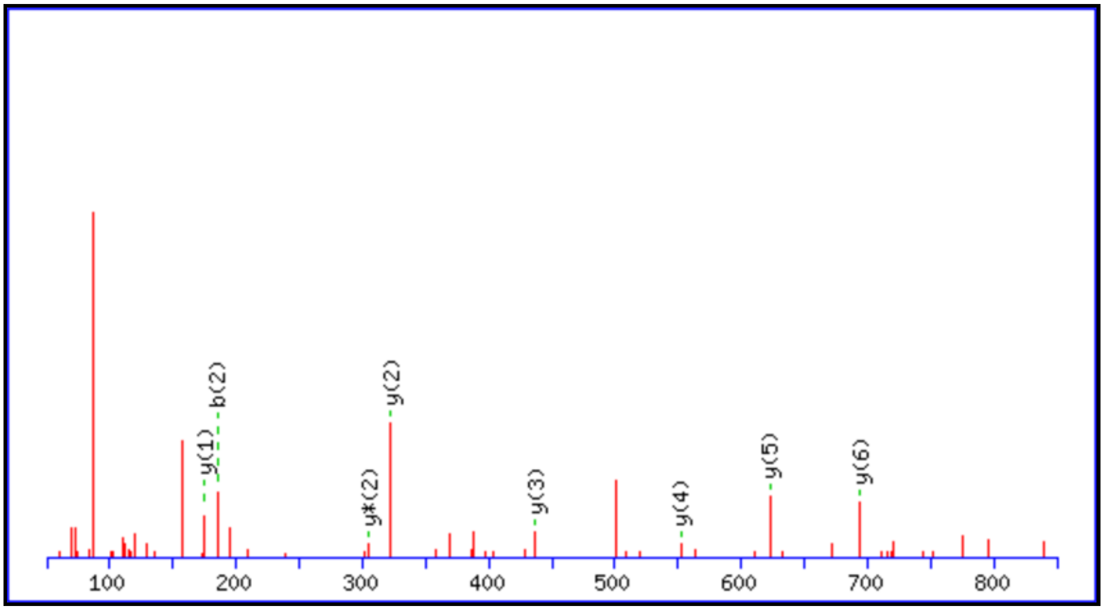

Monoisotopic mass of neutral peptide Mr(calc): 806.3923
Fixed modifications: Carbamidomethyl (C) (apply to specified residues or termini only)
Ions Score: 40 **Expect:** 0.01
Matches : 8/50 fragment ions using 16 most intense peaks (help)

#	b	b^{++}	b^{0}	b^{0++}	Seq.	y	y^{++}	y*	y^{*++}	y^{0}	y^{0++}	#
1	114.0913	57.5493			L							7
2	**185.1285**	93.0679			A	***694.3155***	347.6614	677.2889	339.1481	676.3049	338.6561	6
3	256.1656	128.5864			A	***623.2784***	312.1428	606.2518	303.6295	605.2678	303.1375	5
4	371.1925	186.0999	353.1819	177.0946	D	***552.2413***	276.6243	535.2147	268.1110	534.2307	267.6190	4
5	486.2195	243.6134	468.2089	234.6081	D	***437.2143***	219.1108	420.1878	210.5975	419.2037	210.1055	3
6	633.2879	317.1476	615.2773	308.1423	F	***322.1874***	161.5973	305.1608	153.0840			2

7					R	*175.1190*	88.0631	158.0924	79.5498			1

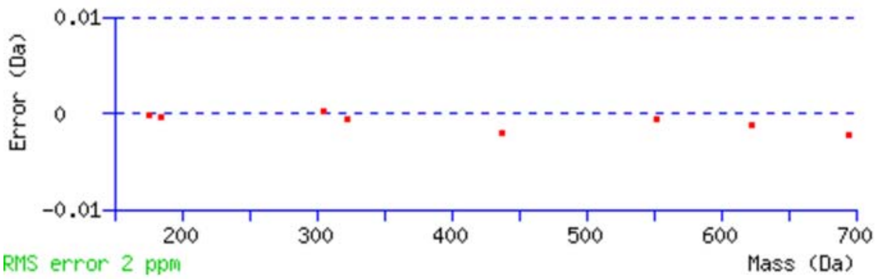

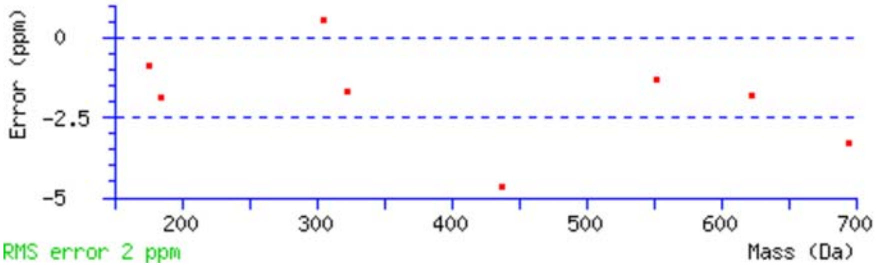

NCBI **BLAST** search of LAADDFR
(Parameters: blastp, nr protein database, expect=20000, no filter, PAM30)
Other BLAST web gateways

All matches to this query

Score	Mr(calc)	Delta	Sequence
40.5	806.3923	-0.0004	LAADDFR
11.9	804.3878	2.0040	RPDNFR
4.2	806.3923	-0.0004	LDQQFR
4.2	806.3923	-0.0004	LDQQFR
3.8	806.3923	-0.0004	IAGFDER
3.6	806.3922	-0.0004	GELANFR
3.6	804.3878	2.0040	NRPDFR
3.6	806.3923	-0.0004	QLEDFR
3.2	806.3844	0.0075	LAAQMQK

Mascot: http://www.matrixscience.com/

Mascot Search Results

Peptide View

MS/MS Fragmentation of **LAADDFR**
Found in **ch17u_O76014|KRT37_HUMAN** in **uni_human**, Keratin, type I cuticular Ha7 OS=Homo sapiens GN=KRT37 PE=3 SV=3

Match to Query 413: 806.391948 from(404.203250,2+) intensity(1540734.3750) rtinseconds(1285) scans(6485) index(32934)
Title: 160219_Sunil_KAP_B_Spectrum084179_scans__6485_RTINSECONDS=1285
Data file L:\\QE_2016\\160219_Sunil_KAP_LKC\\TMgf\\T\\T160219_Sunil_KAP_B.mgf

Click mouse within plot area to zoom in by factor of two about that point
Or, Plot from 50 to 850 Da Full range
Label all possible matches ○ Label matches used for scoring ◉
Show Y-axis ☐

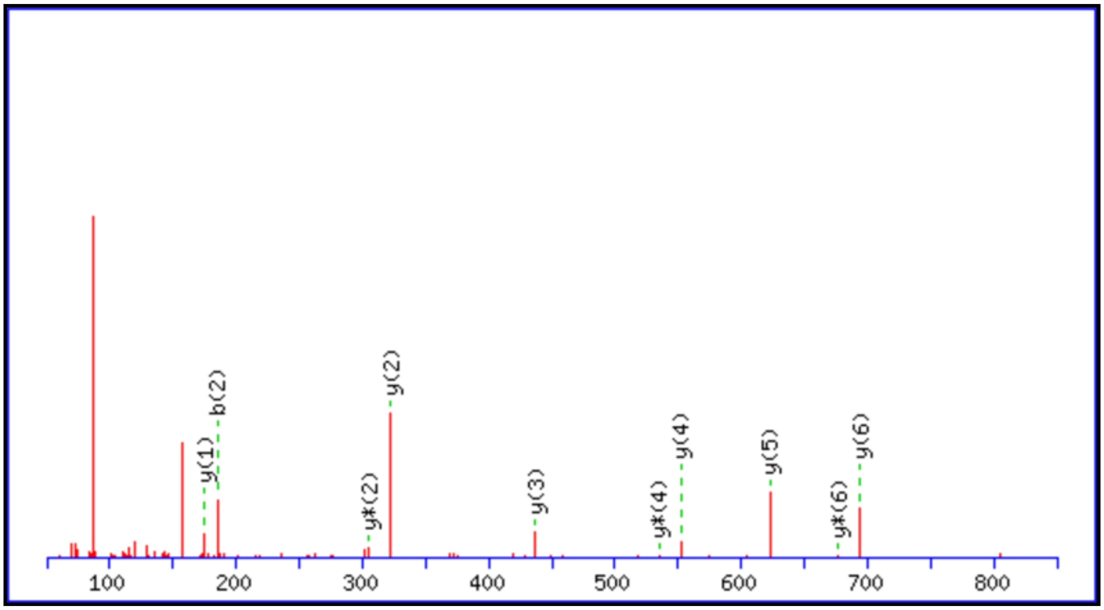

Monoisotopic mass of neutral peptide Mr(calc): 806.3923
Fixed modifications: Carbamidomethyl (C) (apply to specified residues or termini only)
Ions Score: 49 **Expect:** 0.0014
Matches : 10/50 fragment ions using 15 most intense peaks (help)

#	b	b^{++}	b^{0}	b^{0++}	Seq.	y	y^{++}	y*	y^{*++}	y^{0}	y^{0++}	#
1	114.0913	57.5493			L							7
2	**185.1285**	93.0679			A	***694.3155***	347.6614	677.2889	339.1481	676.3049	338.6561	6
3	256.1656	128.5864			A	***623.2784***	312.1428	606.2518	303.6295	605.2678	303.1375	5
4	371.1925	186.0999	353.1819	177.0946	D	***552.2413***	276.6243	535.2147	268.1110	534.2307	267.6190	4
5	486.2195	243.6134	468.2089	234.6081	D	***437.2143***	219.1108	420.1878	210.5975	419.2037	210.1055	3
6	633.2879	317.1476	615.2773	308.1423	F	***322.1874***	161.5973	305.1608	153.0840			2

7					R	*175.1190*	88.0631	158.0924	79.5498			1

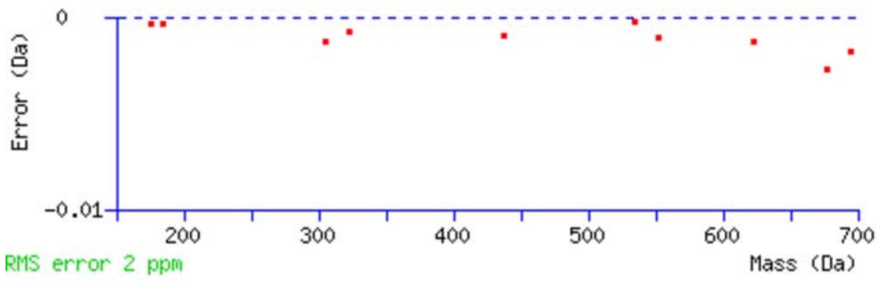

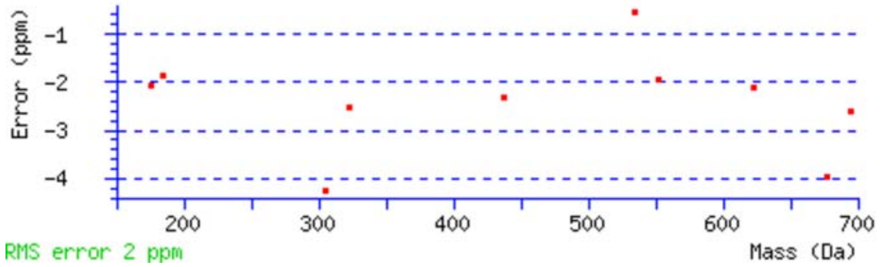

NCBI **BLAST** search of LAADDFR
(Parameters: blastp, nr protein database, expect=20000, no filter, PAM30)
Other BLAST web gateways

All matches to this query

Score	Mr(calc)	Delta	Sequence
49.1	806.3923	-0.0003	LAADDFR
16.2	804.3878	2.0041	RPDNFR
12.4	806.3844	0.0076	LAAQMQK
7.4	806.3923	-0.0003	LDQQFR
7.4	806.3923	-0.0003	LDQQFR
6.8	806.3923	-0.0003	IAGFDER
5.5	806.3922	-0.0003	GELANFR
5.5	804.3878	2.0041	NRPDFR
5.5	806.3923	-0.0003	QLEDFR

Mascot: http://www.matrixscience.com/

Mascot Search Results

Peptide View

MS/MS Fragmentation of **LAADDFR**
Found in **ch17u_O76014|KRT37_HUMAN** in **uni_human**, Keratin, type I cuticular Ha7 OS=Homo sapiens GN=KRT37 PE=3 SV=3

Match to Query 414: 806.392008 from(404.203280,2+) intensity(138793.0938) rtinseconds(1370) scans(7130) index(5156)
Title: 160219_Sunil_KAP_B_Spectrum054265_scans__7130_RTINSECONDS=1370
Data file L:\\QE_2016\\160219_Sunil_KAP_LKC\\TMgf\\T\\T160219_Sunil_KAP_B.mgf

Click mouse within plot area to zoom in by factor of two about that point
Or, Plot from 50 to 700 Da Full range
Label all possible matches ○ Label matches used for scoring ◉
Show Y-axis ☐

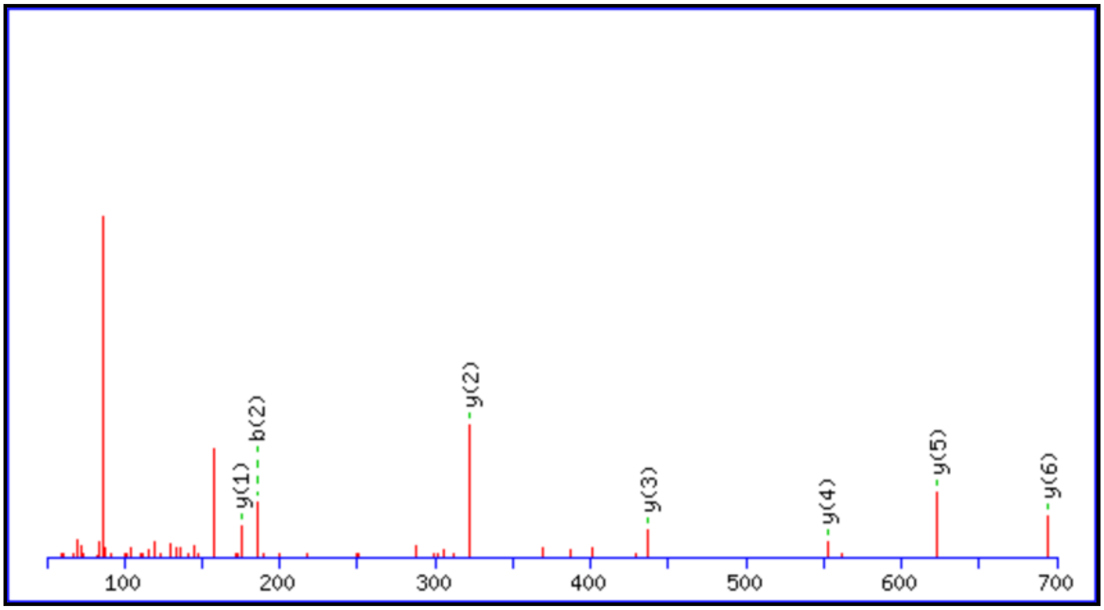

Monoisotopic mass of neutral peptide Mr(calc): 806.3923
Fixed modifications: Carbamidomethyl (C) (apply to specified residues or termini only)
Ions Score: 47 **Expect:** 0.0022
Matches : 7/50 fragment ions using 12 most intense peaks (help)

#	b	b^{++}	b^0	b^{0++}	Seq.	y	y^{++}	y*	y^{*++}	y^0	y^{0++}	#
1	114.0913	57.5493			L							7
2	**185.1285**	93.0679			A	***694.3155***	347.6614	677.2889	339.1481	676.3049	338.6561	6
3	256.1656	128.5864			A	***623.2784***	312.1428	606.2518	303.6295	605.2678	303.1375	5
4	371.1925	186.0999	353.1819	177.0946	D	***552.2413***	276.6243	535.2147	268.1110	534.2307	267.6190	4
5	486.2195	243.6134	468.2089	234.6081	D	***437.2143***	219.1108	420.1878	210.5975	419.2037	210.1055	3
6	633.2879	317.1476	615.2773	308.1423	F	***322.1874***	161.5973	305.1608	153.0840			2

7					R	*175.1190*	88.0631	158.0924	79.5498			1

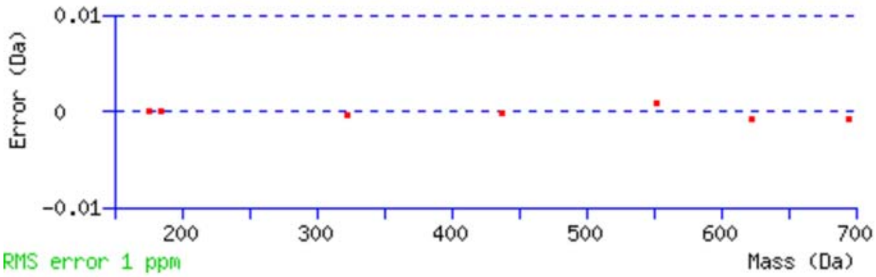

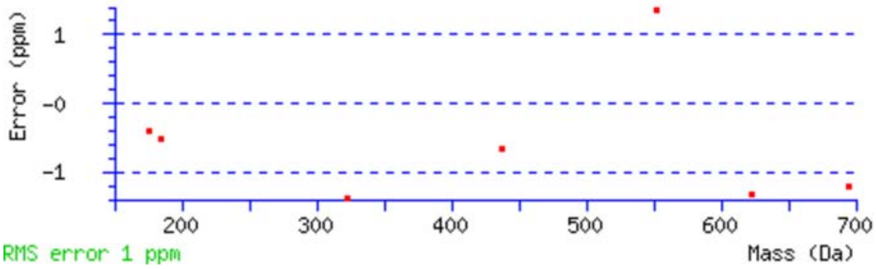

NCBI **BLAST** search of LAADDFR
(Parameters: blastp, nr protein database, expect=20000, no filter, PAM30)
Other BLAST web gateways

All matches to this query

Score	Mr(calc)	Delta	Sequence
47.1	806.3923	-0.0003	LAADDFR
15.2	804.3878	2.0042	RPDNFR
13.9	806.3844	0.0076	LAAQMQK
7.5	806.3923	-0.0003	IAGFDER
7.4	806.3956	-0.0036	MGNSLLR
6.1	806.3923	-0.0003	LDQQFR
6.1	806.3923	-0.0003	LDQQFR
5.8	806.3922	-0.0002	GELANFR
5.8	804.3878	2.0042	NRPDFR
5.8	806.3923	-0.0003	QLEDFR

Mascot: http://www.matrixscience.com/

Mascot Search Results

Peptide View

MS/MS Fragmentation of **LAADDFR**
Found in **ch17u_O76014|KRT37_HUMAN** in **uni_human**, Keratin, type I cuticular Ha7 OS=Homo sapiens GN=KRT37 PE=3 SV=3

Match to Query 415: 806.392128 from(404.203340,2+) intensity(966312.2500) rtinseconds(1432) scans(7484) index(5430)
Title: 160219_Sunil_KAP_B_Spectrum054541_scans__7484_RTINSECONDS=1432
Data file L:\\QE_2016\\160219_Sunil_KAP_LKC\\TMgf\\T\\T160219_Sunil_KAP_B.mgf

Click mouse within plot area to zoom in by factor of two about that point
Or, Plot from 50 to 850 Da Full range
Label all possible matches ○ Label matches used for scoring ◉
Show Y-axis ☐

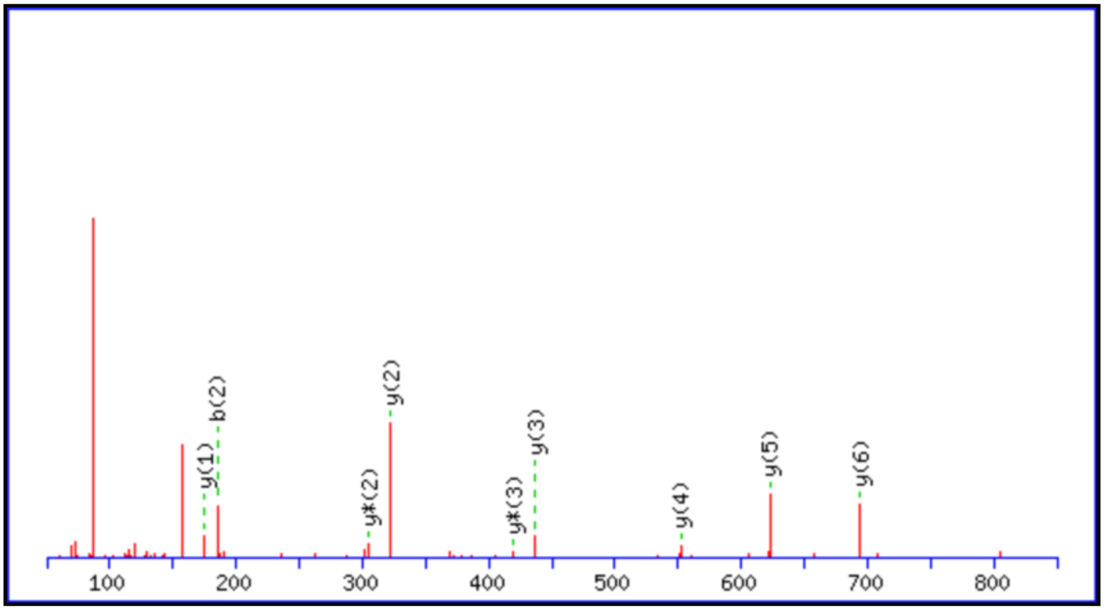

Monoisotopic mass of neutral peptide Mr(calc): 806.3923
Fixed modifications: Carbamidomethyl (C) (apply to specified residues or termini only)
Ions Score: 46 **Expect:** 0.0026
Matches : 9/50 fragment ions using 15 most intense peaks (help)

#	b	b^{++}	b^0	b^{0++}	Seq.	y	y^{++}	y*	y^{*++}	y^0	y^{0++}	#
1	114.0913	57.5493			L							7
2	**185.1285**	93.0679			A	***694.3155***	347.6614	677.2889	339.1481	676.3049	338.6561	6
3	256.1656	128.5864			A	***623.2784***	312.1428	606.2518	303.6295	605.2678	303.1375	5
4	371.1925	186.0999	353.1819	177.0946	D	***552.2413***	276.6243	535.2147	268.1110	534.2307	267.6190	4
5	486.2195	243.6134	468.2089	234.6081	D	***437.2143***	219.1108	420.1878	210.5975	419.2037	210.1055	3
6	633.2879	317.1476	615.2773	308.1423	F	***322.1874***	161.5973	305.1608	153.0840			2

7					R	*175.1190*	88.0631	158.0924	79.5498			1

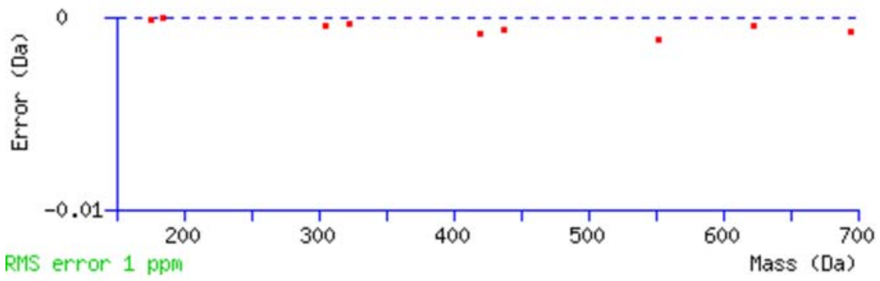

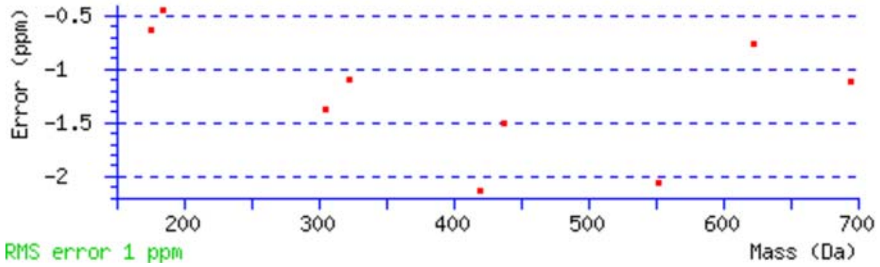

NCBI **BLAST** search of LAADDFR
(Parameters: blastp, nr protein database, expect=20000, no filter, PAM30)
Other BLAST web gateways

All matches to this query

Score	Mr(calc)	Delta	Sequence
46.3	806.3923	-0.0001	LAADDFR
16.1	804.3878	2.0043	RPDNFR
12.4	806.3844	0.0078	LAAQMQK
7.4	806.3923	-0.0001	IAGFDER
6.8	806.3922	-0.0001	GELANFR
6.8	804.3878	2.0043	NRPDFR
6.8	806.3923	-0.0001	QLEDFR
6.0	806.3923	-0.0001	LDQQFR
6.0	806.3923	-0.0001	LDQQFR

Mascot: http://www.matrixscience.com/

Mascot Search Results

Peptide View

MS/MS Fragmentation of **LAADDFR**
Found in **ch17u_O76014|KRT37_HUMAN** in **uni_human**, Keratin, type I cuticular Ha7 OS=Homo sapiens GN=KRT37 PE=3 SV=3

Match to Query 416: 806.392188 from(404.203370,2+) intensity(206619.6250) rtinseconds(1380) scans(7060) index(19328)
Title: 160219_Sunil_KAP_B_Spectrum069197_scans__7060_RTINSECONDS=1380
Data file L:\\QE_2016\\160219_Sunil_KAP_LKC\\TMgf\\T\\T160219_Sunil_KAP_B.mgf

Click mouse within plot area to zoom in by factor of two about that point
Or, Plot from 50 to 700 Da Full range
Label all possible matches ○ Label matches used for scoring ◉
Show Y-axis ☐

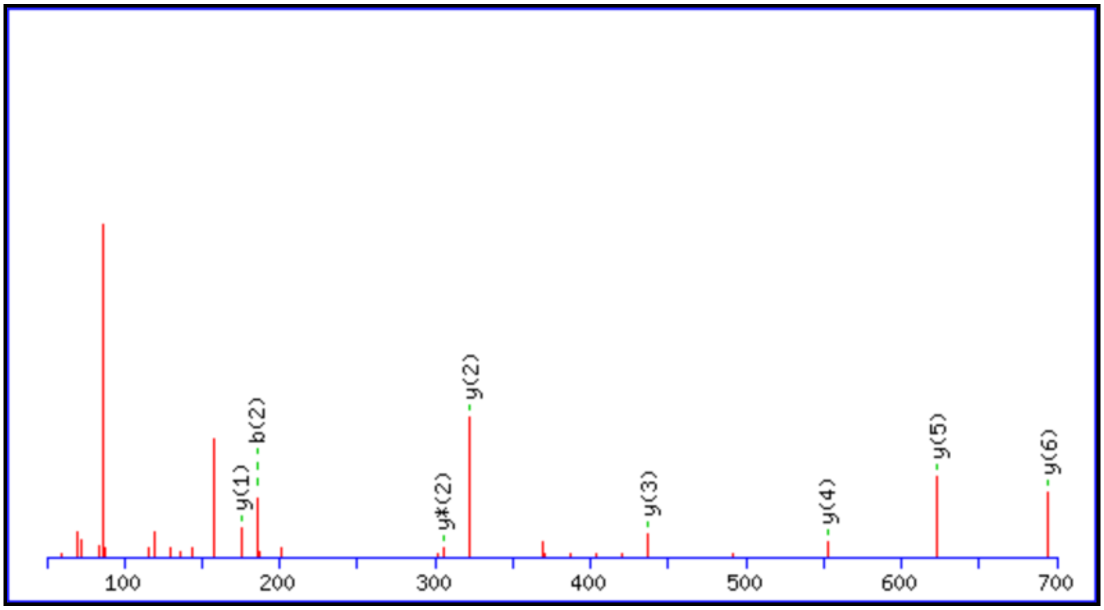

Monoisotopic mass of neutral peptide Mr(calc): 806.3923
Fixed modifications: Carbamidomethyl (C) (apply to specified residues or termini only)
Ions Score: 51 **Expect:** 0.00094
Matches : 8/50 fragment ions using 12 most intense peaks (help)

#	b	b^{++}	b^0	b^{0++}	Seq.	y	y^{++}	y*	y^{*++}	y^0	y^{0++}	#
1	114.0913	57.5493			L							7
2	**185.1285**	93.0679			A	***694.3155***	347.6614	677.2889	339.1481	676.3049	338.6561	6
3	256.1656	128.5864			A	***623.2784***	312.1428	606.2518	303.6295	605.2678	303.1375	5
4	371.1925	186.0999	353.1819	177.0946	D	***552.2413***	276.6243	535.2147	268.1110	534.2307	267.6190	4
5	486.2195	243.6134	468.2089	234.6081	D	***437.2143***	219.1108	420.1878	210.5975	419.2037	210.1055	3
6	633.2879	317.1476	615.2773	308.1423	F	***322.1874***	161.5973	305.1608	153.0840			2

7					R	*175.1190*	88.0631	158.0924	79.5498			1

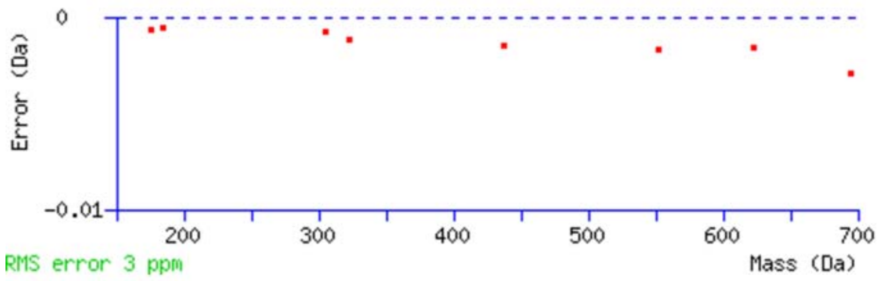

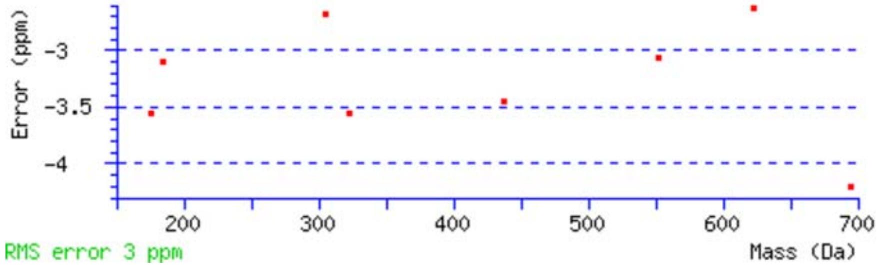

NCBI **BLAST** search of LAADDFR
(Parameters: blastp, nr protein database, expect=20000, no filter, PAM30)
Other BLAST web gateways

All matches to this query

Score	Mr(calc)	Delta	Sequence
50.8	806.3923	-0.0001	LAADDFR
17.7	804.3878	2.0044	RPDNFR
14.7	806.3844	0.0078	LAAQMQK
9.3	806.3922	-0.0001	GELANFR
8.4	806.3923	-0.0001	LDQQFR
8.4	806.3923	-0.0001	LDQQFR
8.2	806.3923	-0.0001	IAGFDER
7.6	804.3878	2.0044	NRPDFR
7.6	806.3923	-0.0001	QLEDFR
4.2	806.3891	0.0031	IACCRK

Mascot: http://www.matrixscience.com/

Mascot Search Results

Peptide View

MS/MS Fragmentation of **LAADDFR**
Found in **ch17u_O76014|KRT37_HUMAN** in **uni_human**, Keratin, type I cuticular Ha7 OS=Homo sapiens GN=KRT37 PE=3 SV=3

Match to Query 417: 806.392308 from(404.203430,2+) intensity(61366.9883) rtinseconds(1442) scans(7416) index(19637)
Title: 160219_Sunil_KAP_B_Spectrum069509_scans__7416_RTINSECONDS=1442
Data file L:\\QE_2016\\160219_Sunil_KAP_LKC\\TMgf\\T\\T160219_Sunil_KAP_B.mgf

Click mouse within plot area to zoom in by factor of two about that point
Or, Plot from 50 to 750 Da Full range
Label all possible matches ○ Label matches used for scoring ◉
Show Y-axis ☐

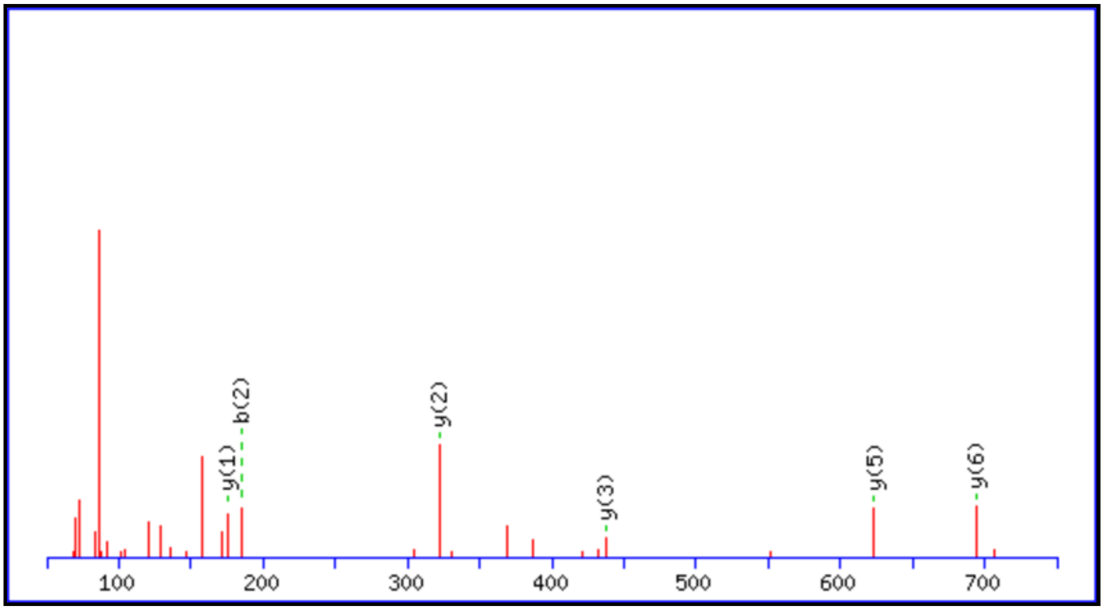

Monoisotopic mass of neutral peptide Mr(calc): 806.3923
Fixed modifications: Carbamidomethyl (C) (apply to specified residues or termini only)
Ions Score: 32 **Expect:** 0.081
Matches : 6/50 fragment ions using 12 most intense peaks (help)

#	b	b^{++}	b^0	b^{0++}	Seq.	y	y^{++}	y*	y^{*++}	y^0	y^{0++}	#
1	114.0913	57.5493			L							7
2	**185.1285**	93.0679			A	***694.3155***	347.6614	677.2889	339.1481	676.3049	338.6561	6
3	256.1656	128.5864			A	***623.2784***	312.1428	606.2518	303.6295	605.2678	303.1375	5
4	371.1925	186.0999	353.1819	177.0946	D	552.2413	276.6243	535.2147	268.1110	534.2307	267.6190	4
5	486.2195	243.6134	468.2089	234.6081	D	***437.2143***	219.1108	420.1878	210.5975	419.2037	210.1055	3
6	633.2879	317.1476	615.2773	308.1423	F	***322.1874***	161.5973	305.1608	153.0840			2

7					R	*175.1190*	88.0631	158.0924	79.5498			1

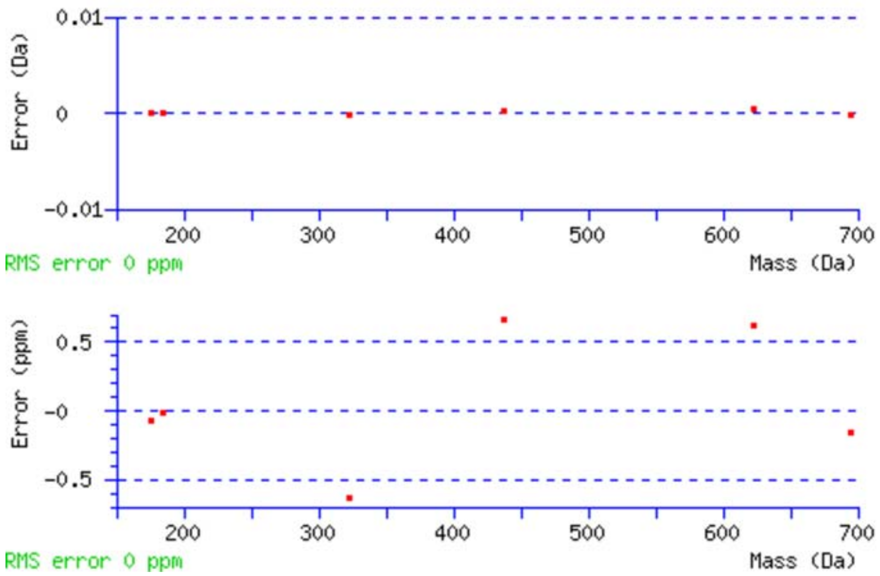

NCBI **BLAST** search of LAADDFR
(Parameters: blastp, nr protein database, expect=20000, no filter, PAM30)
Other BLAST web gateways

All matches to this query

Score	Mr(calc)	Delta	Sequence
31.6	806.3923	0.0001	LAADDFR
7.2	806.3923	0.0001	IAGFDER
5.8	806.3923	0.0001	LDQQFR
5.8	806.3923	0.0001	LDQQFR
5.1	806.3922	0.0001	GELANFR
5.1	804.3878	2.0045	NRPDFR
5.1	806.3923	0.0001	QLEDFR
5.1	804.3878	2.0045	RPDNFR
2.7	806.3891	0.0032	IACCRK
2.7	806.3844	0.0079	LAAQMQK

Mascot: http://www.matrixscience.com/

Mascot Search Results

Peptide View

MS/MS Fragmentation of **LAADDFR**
Found in **ch17u_O76014|KRT37_HUMAN** in **uni_human**, Keratin, type I cuticular Ha7 OS=Homo sapiens GN=KRT37 PE=3 SV=3

Match to Query 418: 806.392308 from(404.203430,2+) intensity(10343354.0000) rtinseconds(1401) scans(7308) index(5291)
Title: 160219_Sunil_KAP_B_Spectrum054402_scans__7308_RTINSECONDS=1401
Data file L:\\QE_2016\\160219_Sunil_KAP_LKC\\TMgf\\T\\T160219_Sunil_KAP_B.mgf

Click mouse within plot area to zoom in by factor of two about that point
Or, Plot from 50 to 850 Da Full range
Label all possible matches ○ Label matches used for scoring ◉
Show Y-axis ☐

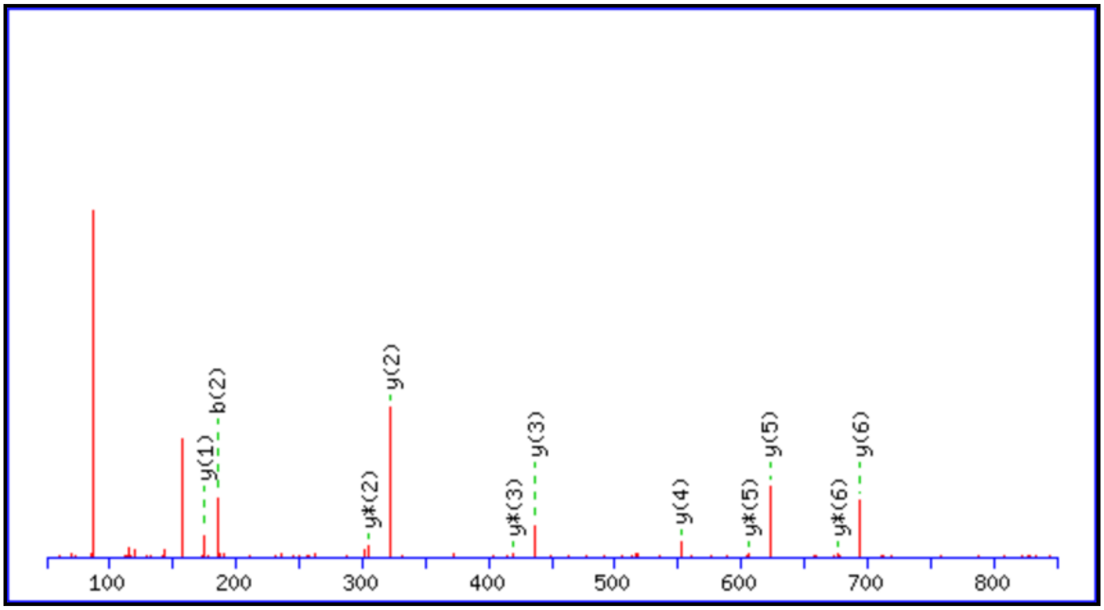

Monoisotopic mass of neutral peptide Mr(calc): 806.3923
Fixed modifications: Carbamidomethyl (C) (apply to specified residues or termini only)
Ions Score: 52 **Expect:** 0.0007
Matches : 11/50 fragment ions using 15 most intense peaks (help)

#	b	b^{++}	b^{0}	b^{0++}	Seq.	y	y^{++}	y*	y^{*++}	y^{0}	y^{0++}	#
1	114.0913	57.5493			L							7
2	**185.1285**	93.0679			A	***694.3155***	347.6614	677.2889	339.1481	676.3049	338.6561	6
3	256.1656	128.5864			A	***623.2784***	312.1428	606.2518	303.6295	605.2678	303.1375	5
4	371.1925	186.0999	353.1819	177.0946	D	***552.2413***	276.6243	535.2147	268.1110	534.2307	267.6190	4
5	486.2195	243.6134	468.2089	234.6081	D	***437.2143***	219.1108	420.1878	210.5975	419.2037	210.1055	3
6	633.2879	317.1476	615.2773	308.1423	F	***322.1874***	161.5973	305.1608	153.0840			2

7					R	*175.1190*	88.0631	158.0924	79.5498			1

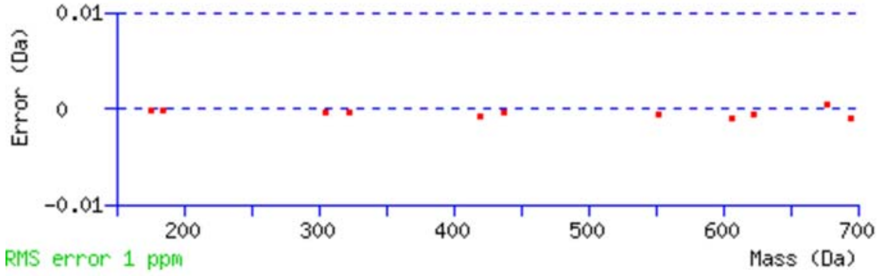

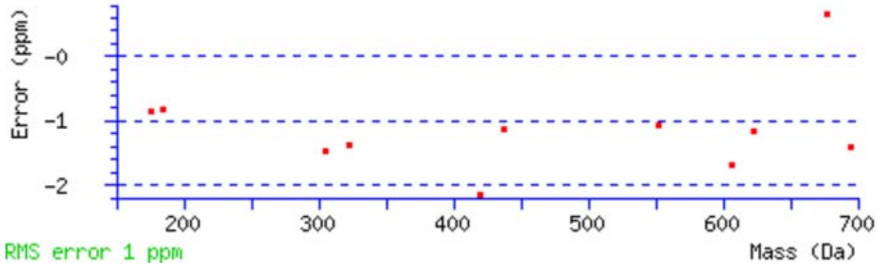

NCBI **BLAST** search of LAADDFR
(Parameters: blastp, nr protein database, expect=20000, no filter, PAM30)
Other BLAST web gateways

All matches to this query

Score	Mr(calc)	Delta	Sequence
52.2	806.3923	0.0001	LAADDFR
16.4	804.3878	2.0045	RPDNFR
12.7	806.3844	0.0079	LAAQMQK
9.2	806.3923	0.0001	IAGFDER
7.6	806.3923	0.0001	LDQQFR
7.6	806.3923	0.0001	LDQQFR
7.1	806.3922	0.0001	GELANFR
7.1	804.3878	2.0045	NRPDFR
7.1	806.3923	0.0001	QLEDFR

Mascot: http://www.matrixscience.com/

Mascot Search Results

Peptide View

MS/MS Fragmentation of **LAADDFR**
Found in **ch17u_O76014|KRT37_HUMAN** in **uni_human**, Keratin, type I cuticular Ha7 OS=Homo sapiens GN=KRT37 PE=3 SV=3

Match to Query 421: 806.392368 from(404.203460,2+) intensity(13669628.0000) rtinseconds(1286) scans(6520) index(18870)
Title: 160219_Sunil_KAP_B_Spectrum068731_scans__6520_RTINSECONDS=1286
Data file L:\\QE_2016\\160219_Sunil_KAP_LKC\\TMgf\\T\\T160219_Sunil_KAP_B.mgf

Click mouse within plot area to zoom in by factor of two about that point
Or, Plot from 50 to 850 Da Full range
Label all possible matches ○ Label matches used for scoring ◉
Show Y-axis ☐

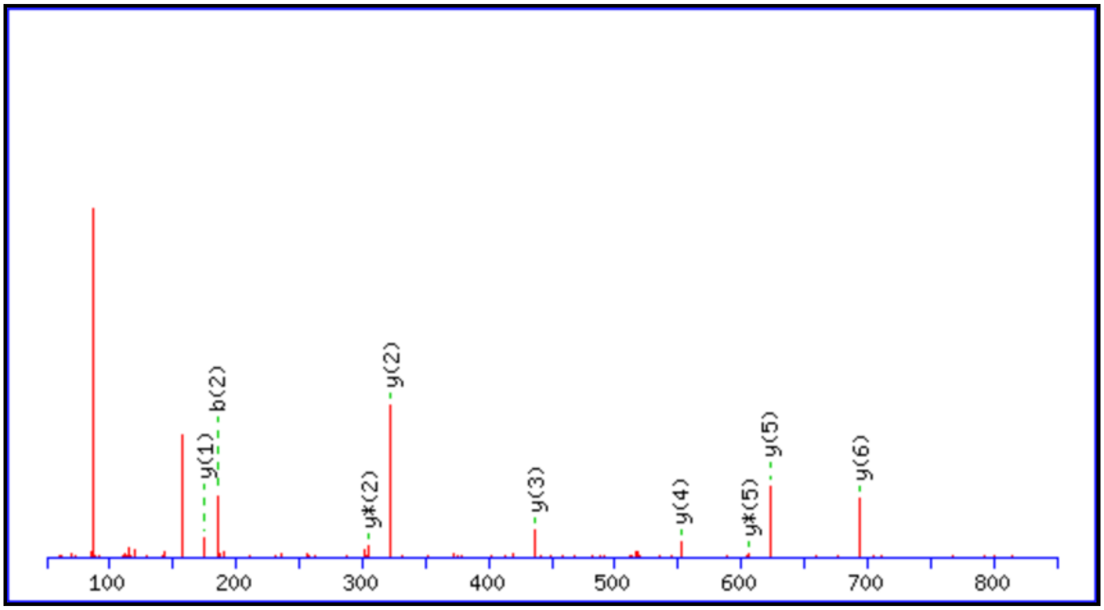

Monoisotopic mass of neutral peptide Mr(calc): 806.3923
Fixed modifications: Carbamidomethyl (C) (apply to specified residues or termini only)
Ions Score: 45 **Expect:** 0.0041
Matches : 9/50 fragment ions using 16 most intense peaks (help)

#	b	b^{++}	b^0	b^{0++}	Seq.	y	y^{++}	y^*	y^{*++}	y^0	y^{0++}	#
1	114.0913	57.5493			L							7
2	**185.1285**	93.0679			A	***694.3155***	347.6614	677.2889	339.1481	676.3049	338.6561	6
3	256.1656	128.5864			A	***623.2784***	312.1428	606.2518	303.6295	605.2678	303.1375	5
4	371.1925	186.0999	353.1819	177.0946	D	***552.2413***	276.6243	535.2147	268.1110	534.2307	267.6190	4
5	486.2195	243.6134	468.2089	234.6081	D	***437.2143***	219.1108	420.1878	210.5975	419.2037	210.1055	3
6	633.2879	317.1476	615.2773	308.1423	F	***322.1874***	161.5973	305.1608	153.0840			2

7					R	*175.1190*	88.0631	158.0924	79.5498			1

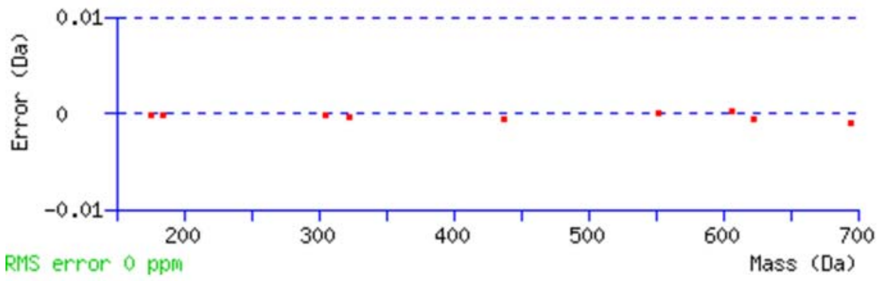

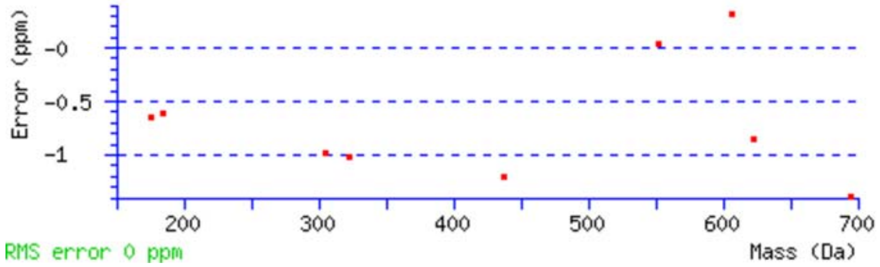

NCBI **BLAST** search of LAADDFR
(Parameters: blastp, nr protein database, expect=20000, no filter, PAM30)
Other BLAST web gateways

All matches to this query

Score	Mr(calc)	Delta	Sequence
44.5	806.3923	0.0001	LAADDFR
13.3	804.3878	2.0045	RPDNFR
12.8	806.3844	0.0080	LAAQMQK
6.3	806.3923	0.0001	IAGFDER
5.6	806.3923	0.0001	LDQQFR
5.6	806.3923	0.0001	LDQQFR
5.0	806.3922	0.0001	GELANFR
5.0	804.3878	2.0045	NRPDFR
5.0	806.3923	0.0001	QLEDFR

Mascot: http://www.matrixscience.com/

Mascot Search Results

Peptide View

MS/MS Fragmentation of **LAADDFR**
Found in **ch17u_O76014|KRT37_HUMAN** in **uni_human**, Keratin, type I cuticular Ha7 OS=Homo sapiens GN=KRT37 PE=3 SV=3

Match to Query 422: 806.392608 from(404.203580,2+) intensity(1355356.1250) rtinseconds(1317) scans(6696) index(19024)
Title: 160219_Sunil_KAP_B_Spectrum068886_scans__6696_RTINSECONDS=1317
Data file L:\\QE_2016\\160219_Sunil_KAP_LKC\\TMgf\\T\\T160219_Sunil_KAP_B.mgf

Click mouse within plot area to zoom in by factor of two about that point
Or, Plot from 50 to 750 Da Full range
Label all possible matches ○ Label matches used for scoring ◉
Show Y-axis ☐

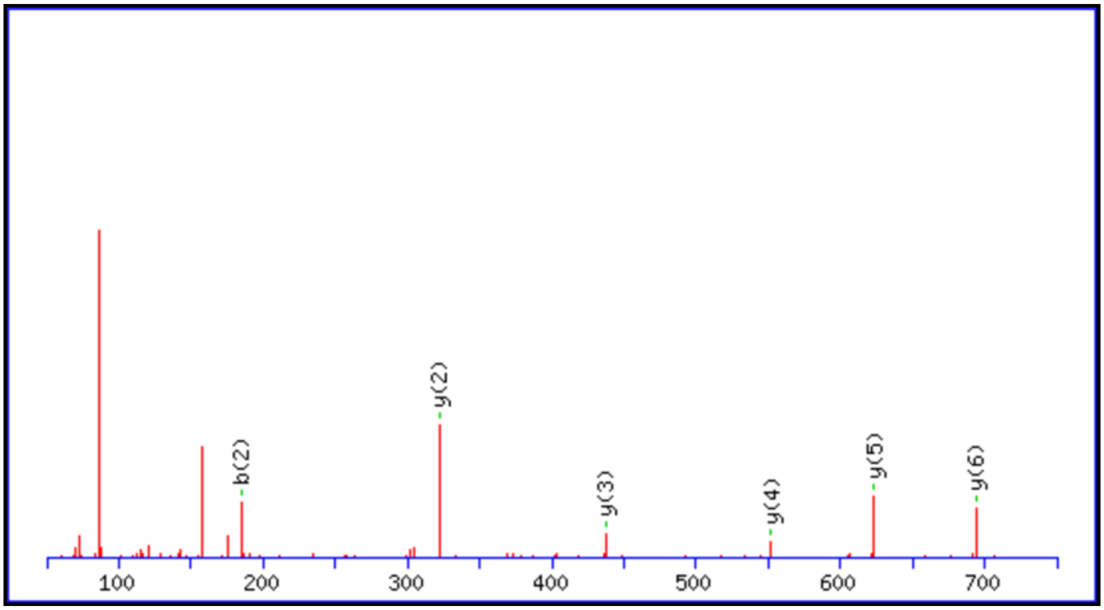

Monoisotopic mass of neutral peptide Mr(calc): 806.3923
Fixed modifications: Carbamidomethyl (C) (apply to specified residues or termini only)
Ions Score: 47 **Expect:** 0.0023
Matches : 6/50 fragment ions using 7 most intense peaks (help)

#	b	b++	b0	b0++	Seq.	y	y++	y*	y*++	y0	y0++	#
1	114.0913	57.5493			L							7
2	**185.1285**	93.0679			A	***694.3155***	347.6614	677.2889	339.1481	676.3049	338.6561	6
3	256.1656	128.5864			A	***623.2784***	312.1428	606.2518	303.6295	605.2678	303.1375	5
4	371.1925	186.0999	353.1819	177.0946	D	***552.2413***	276.6243	535.2147	268.1110	534.2307	267.6190	4
5	486.2195	243.6134	468.2089	234.6081	D	***437.2143***	219.1108	420.1878	210.5975	419.2037	210.1055	3
6	633.2879	317.1476	615.2773	308.1423	F	***322.1874***	161.5973	305.1608	153.0840			2

**7**					**R**	175.1190	88.0631	158.0924	79.5498			**1**

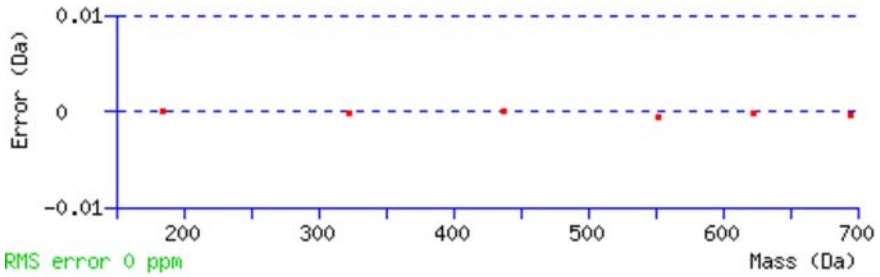

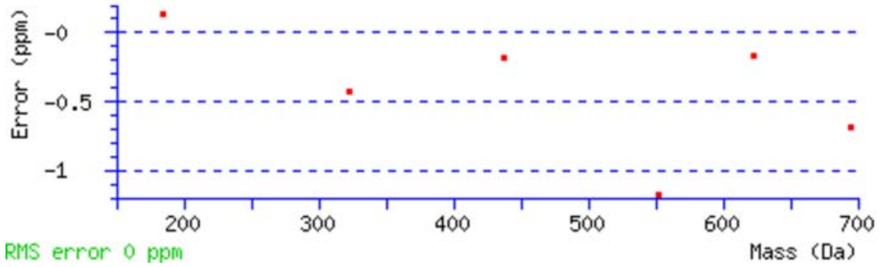

NCBI **BLAST** search of LAADDFR
(Parameters: blastp, nr protein database, expect=20000, no filter, PAM30)
Other BLAST web gateways

All matches to this query

Score	Mr(calc)	Delta	Sequence
46.8	806.3923	0.0004	LAADDFR
15.2	804.3878	2.0048	RPDNFR
10.4	806.3923	0.0004	IAGFDER
6.6	806.3923	0.0004	LDQQFR
6.6	806.3923	0.0004	LDQQFR
6.0	806.3922	0.0004	GELANFR
6.0	804.3878	2.0048	NRPDFR
6.0	806.3923	0.0004	QLEDFR
5.2	804.3912	2.0014	LASQACR
3.8	806.3891	0.0035	IACCRK

Mascot: http://www.matrixscience.com/

Mascot Search Results

Peptide View

MS/MS Fragmentation of **LAADDFR**
Found in **ch17u_O76014|KRT37_HUMAN** in **uni_human**, Keratin, type I cuticular Ha7 OS=Homo sapiens GN=KRT37 PE=3 SV=3

Match to Query 424: 806.392988 from(404.203770,2+) intensity(212462.7188) rtinseconds(1465) scans(7679) index(5597)
Title: 160219_Sunil_KAP_B_Spectrum054710_scans__7679_RTINSECONDS=1465
Data file L:\\QE_2016\\160219_Sunil_KAP_LKC\\TMgf\\T\\T160219_Sunil_KAP_B.mgf

Click mouse within plot area to zoom in by factor of two about that point
Or, Plot from 50 to 700 Da Full range
Label all possible matches ○ Label matches used for scoring ◉
Show Y-axis ☐

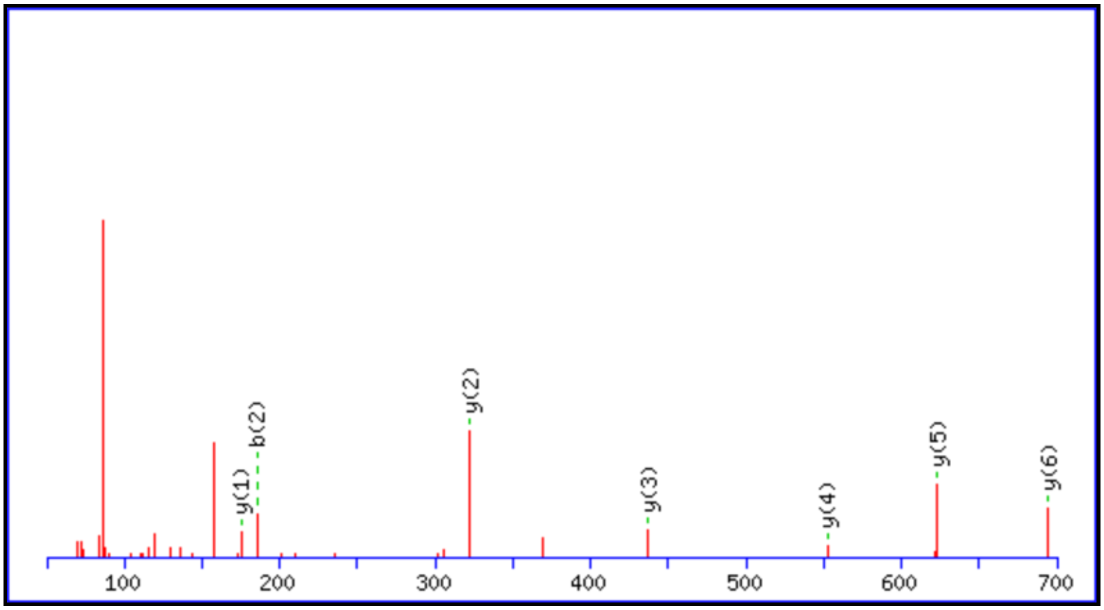

Monoisotopic mass of neutral peptide Mr(calc): 806.3923
Fixed modifications: Carbamidomethyl (C) (apply to specified residues or termini only)
Ions Score: 50 **Expect:** 0.0011
Matches : 7/50 fragment ions using 11 most intense peaks (help)

#	b	b^{++}	b^0	b^{0++}	Seq.	y	y^{++}	y*	y^{*++}	y^0	y^{0++}	#
1	114.0913	57.5493			L							7
2	**185.1285**	93.0679			A	***694.3155***	347.6614	677.2889	339.1481	676.3049	338.6561	6
3	256.1656	128.5864			A	***623.2784***	312.1428	606.2518	303.6295	605.2678	303.1375	5
4	371.1925	186.0999	353.1819	177.0946	D	***552.2413***	276.6243	535.2147	268.1110	534.2307	267.6190	4
5	486.2195	243.6134	468.2089	234.6081	D	***437.2143***	219.1108	420.1878	210.5975	419.2037	210.1055	3
6	633.2879	317.1476	615.2773	308.1423	F	***322.1874***	161.5973	305.1608	153.0840			2

7					R	*175.1190*	88.0631	158.0924	79.5498			1

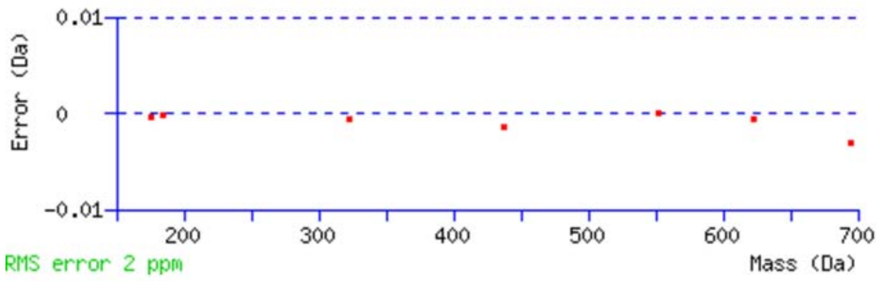

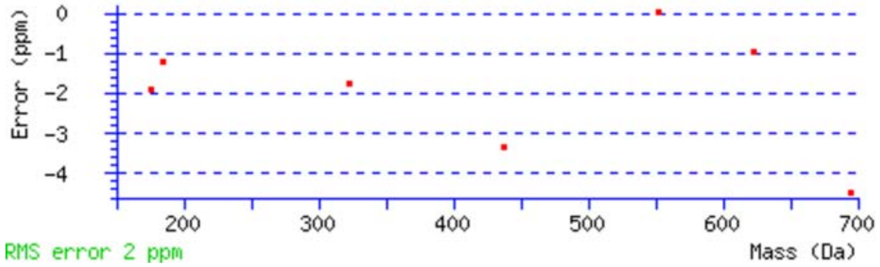

NCBI **BLAST** search of LAADDFR
(Parameters: blastp, nr protein database, expect=20000, no filter, PAM30)
Other BLAST web gateways

All matches to this query

Score	Mr(calc)	Delta	Sequence
49.8	806.3923	0.0007	LAADDFR
16.4	804.3878	2.0052	RPDNFR
8.6	806.3923	0.0007	IAGFDER
7.0	806.3923	0.0007	LDQQFR
7.0	806.3923	0.0007	LDQQFR
6.5	806.3922	0.0007	GELANFR
6.5	804.3878	2.0052	NRPDFR
6.5	806.3923	0.0007	QLEDFR
3.6	806.3891	0.0039	IACCRK
3.6	806.3956	-0.0026	LACTNTK

Mascot: http://www.matrixscience.com/

Mascot Search Results

Peptide View

MS/MS Fragmentation of **TIEELQQKILCSK**
Found in **ch17u_O76014|KRT37_HUMAN** in **uni_human**, Keratin, type I cuticular Ha7 OS=Homo sapiens GN=KRT37 PE=3 SV=3

Match to Query 17886: 1588.848972 from(530.623600,3+) intensity(161562.1250) rtinseconds(2409) scans(13104) index(9953)
Title: 160219_Sunil_KAP_B_Spectrum059103_scans__13104_RTINSECONDS=2409
Data file L:\\QE_2016\\160219_Sunil_KAP_LKC\\TMgf\\T\\T160219_Sunil_KAP_B.mgf

Click mouse within plot area to zoom in by factor of two about that point
Or, Plot from 100 to 1600 Da Full range
Label all possible matches ○ Label matches used for scoring ◉
Show Y-axis □

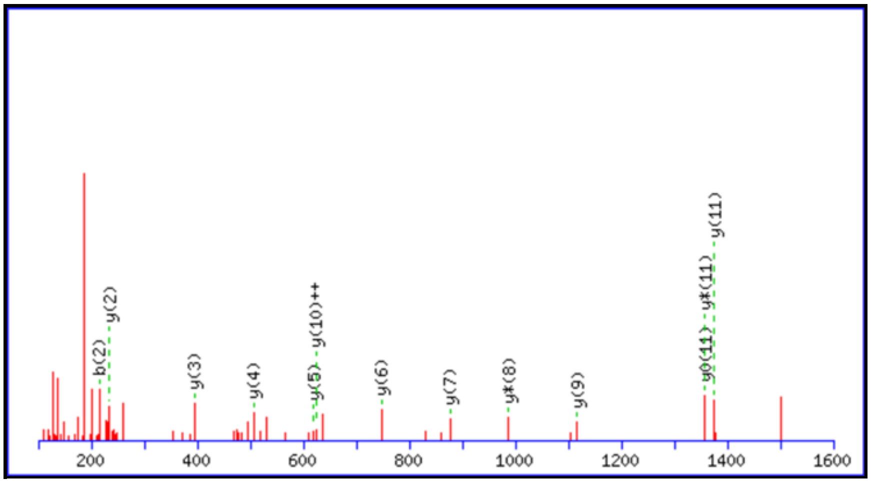

Monoisotopic mass of neutral peptide Mr(calc): 1588.8494
Fixed modifications: Carbamidomethyl (C) (apply to specified residues or termini only)
Ions Score: 40 **Expect:** 0.015
Matches : 13/132 fragment ions using 29 most intense peaks (help)

#	b	b^{++}	b*	b^{*++}	b^0	b^{0++}	Seq.	y	y^{++}	y*	y^{*++}	y^0	y^{0++}	#
1	102.0550	51.5311			84.0444	42.5258	T							13
2	**215.1390**	108.0731			197.1285	99.0679	I	1488.8090	744.9082	1471.7825	736.3949	1470.7985	735.9029	12
3	344.1816	172.5944			326.1710	163.5892	E	***1375.7250***	688.3661	1358.6984	679.8529	1357.7144	679.3608	11
4	473.2242	237.1157			455.2136	228.1105	E	1246.6824	623.8448	1229.6558	615.3316	1228.6718	614.8395	10
5	586.3083	293.6578			568.2977	284.6525	L	***1117.6398***	559.3235	1100.6132	550.8103	1099.6292	550.3183	9
6	714.3668	357.6871	697.3403	349.1738	696.3563	348.6818	Q	1004.5557	502.7815	987.5292	494.2682	986.5452	493.7762	8
7	842.4254	421.7164	825.3989	413.2031	824.4149	412.7111	Q	***876.4972***	438.7522	859.4706	430.2389	858.4866	429.7469	7
8	970.5204	485.7638	953.4938	477.2506	952.5098	476.7585	K	***748.4386***	374.7229	731.4120	366.2096	730.4280	365.7176	6
9	1083.6045	542.3059	1066.5779	533.7926	1065.5939	533.3006	I	***620.3436***	310.6754	603.3171	302.1622	602.3330	301.6702	5
10	1196.6885	598.8479	1179.6620	590.3346	1178.6780	589.8426	L	***507.2595***	254.1334	490.2330	245.6201	489.2490	245.1281	4
11	1356.7192	678.8632	1339.6926	670.3499	1338.7086	669.8579	C	***394.1755***	197.5914	377.1489	189.0781	376.1649	188.5861	3
12	1443.7512	722.3792	1426.7246	713.8660	1425.7406	713.3740	S	***234.1448***	117.5761	217.1183	109.0628	216.1343	108.5708	2
13							K	147.1128	74.0600	130.0863	65.5468			1

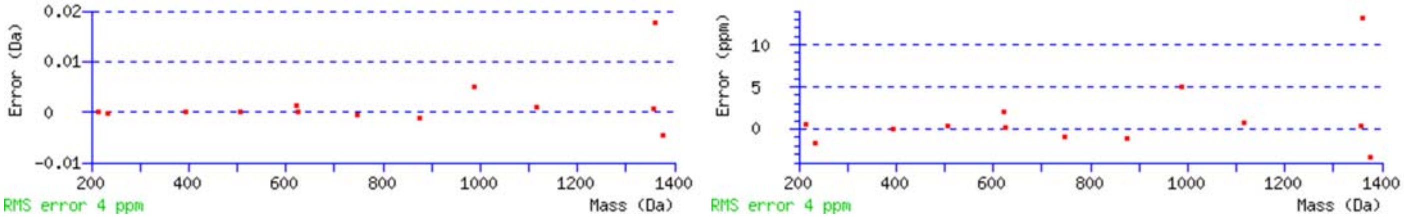

NCBI **BLAST** search of TIEELQQKILCSK
(Parameters: blastp, nr protein database, expect=20000, no filter, PAM30)
Other BLAST web gateways

All matches to this query

Score	Mr(calc)	Delta	Sequence
40.0	1588.8494	-0.0004	TIEELQQKILCSK

Mascot: http://www.matrixscience.com/

Mascot Search Results

Peptide View

MS/MS Fragmentation of **LAADDFR**
Found in **ch17u_O76014|KRT37_HUMAN** in **uni_human**, Keratin, type I cuticular Ha7 OS=Homo sapiens GN=KRT37 PE=3 SV=3

Match to Query 412: 806.391948 from(404.203250,2+) intensity(445841.9063) rtinseconds(1349) scans(6881) index(19179)
Title: 160219_Sunil_KAP_B_Spectrum069044_scans__6881_RTINSECONDS=1349
Data file L:\\QE_2016\\160219_Sunil_KAP_LKC\\TMgf\\T\\T160219_Sunil_KAP_B.mgf

Click mouse within plot area to zoom in by factor of two about that point
Or, Plot from 50 to 700 Da Full range
Label all possible matches ○ Label matches used for scoring ◉
Show Y-axis ☐

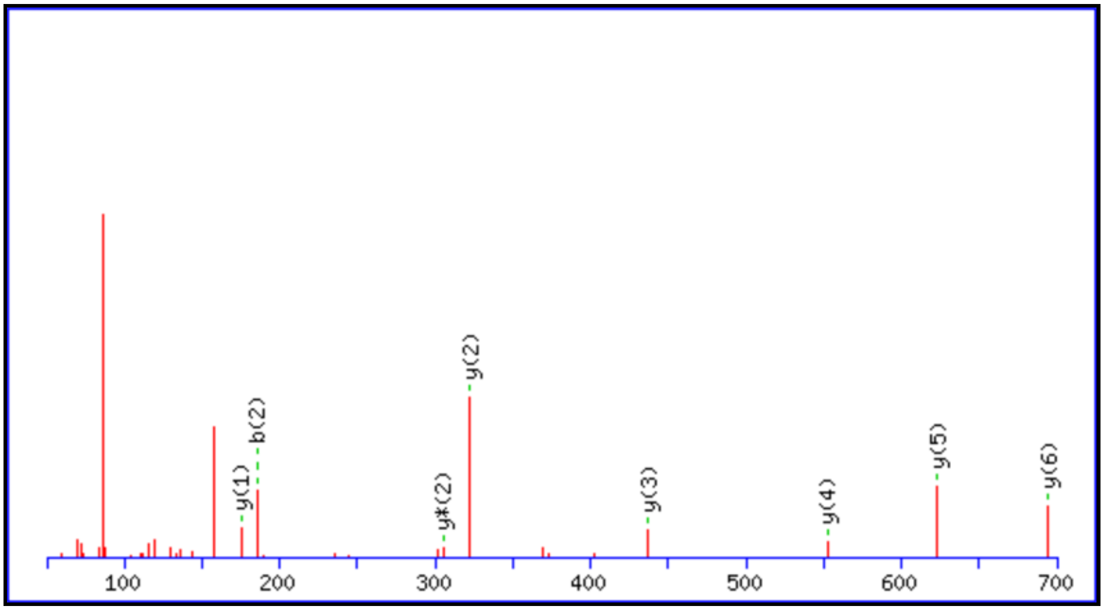

Monoisotopic mass of neutral peptide Mr(calc): 806.3923
Fixed modifications: Carbamidomethyl (C) (apply to specified residues or termini only)
Ions Score: 54 **Expect:** 0.00046
Matches : 8/50 fragment ions using 11 most intense peaks (help)

#	b	b^{++}	b^0	b^{0++}	Seq.	y	y^{++}	y*	y^{*++}	y^0	y^{0++}	#
1	114.0913	57.5493			L							7
2	**185.1285**	93.0679			A	***694.3155***	347.6614	677.2889	339.1481	676.3049	338.6561	6
3	256.1656	128.5864			A	***623.2784***	312.1428	606.2518	303.6295	605.2678	303.1375	5
4	371.1925	186.0999	353.1819	177.0946	D	***552.2413***	276.6243	535.2147	268.1110	534.2307	267.6190	4
5	486.2195	243.6134	468.2089	234.6081	D	***437.2143***	219.1108	420.1878	210.5975	419.2037	210.1055	3
6	633.2879	317.1476	615.2773	308.1423	F	***322.1874***	161.5973	305.1608	153.0840			2

7					R	*175.1190*	88.0631	158.0924	79.5498			1

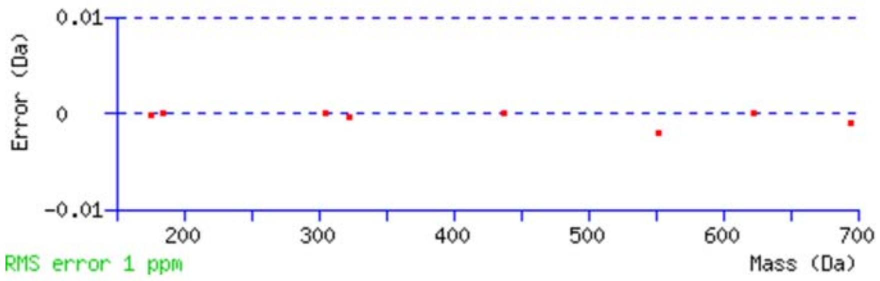

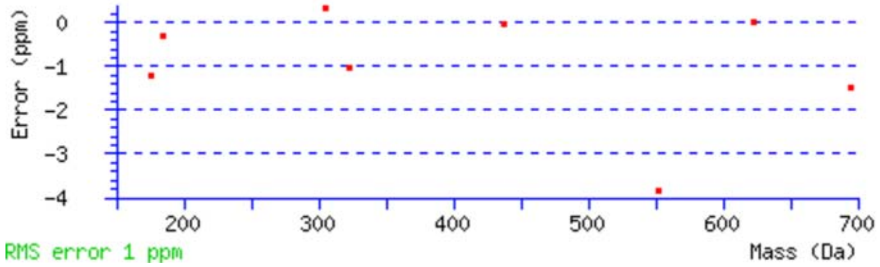

NCBI **BLAST** search of LAADDFR
(Parameters: blastp, nr protein database, expect=20000, no filter, PAM30)
Other BLAST web gateways

All matches to this query

Score	Mr(calc)	Delta	Sequence
53.9	806.3923	-0.0003	LAADDFR
19.8	804.3878	2.0041	RPDNFR
14.4	806.3844	0.0076	LAAQMQK
9.6	806.3923	-0.0003	LDQQFR
9.6	806.3923	-0.0003	LDQQFR
9.1	806.3922	-0.0003	GELANFR
9.1	804.3878	2.0041	NRPDFR
9.1	806.3923	-0.0003	QLEDFR
8.9	806.3923	-0.0003	IAGFDER
3.9	806.3891	0.0028	IACCRK

Mascot: http://www.matrixscience.com/

Mascot Search Results

Peptide View

MS/MS Fragmentation of **TIEELQQKILCSK**
Found in **ch17u_O76014|KRT37_HUMAN** in **uni_human**, Keratin, type I cuticular Ha7 OS=Homo sapiens GN=KRT37 PE=3 SV=3

Match to Query 17887: 1588.849152 from(530.623660,3+) intensity(633011.5625) rtinseconds(2140) scans(11410) index(22980)
Title: 160219_Sunil_KAP_B_Spectrum072891_scans__11410_RTINSECONDS=2140
Data file L:\\QE_2016\\160219_Sunil_KAP_LKC\\TMgf\\T\\T160219_Sunil_KAP_B.mgf

Click mouse within plot area to zoom in by factor of two about that point
Or, Plot from 100 to 1600 Da Full range
Label all possible matches ○ Label matches used for scoring ◉
Show Y-axis ☐

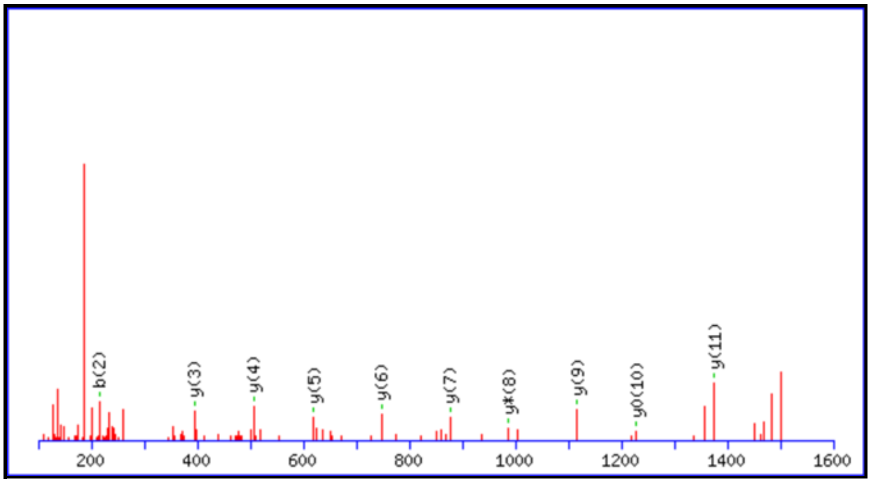

Monoisotopic mass of neutral peptide Mr(calc): 1588.8494
Fixed modifications: Carbamidomethyl (C) (apply to specified residues or termini only)
Ions Score: 53 **Expect:** 0.00079
Matches : 10/132 fragment ions using 13 most intense peaks (help)

#	b	b^{++}	b*	b^{*++}	b^{0}	b^{0++}	Seq.	y	y^{++}	y*	y^{*++}	y^{0}	y^{0++}	#
1	102.0550	51.5311			84.0444	42.5258	T							13
2	**215.1390**	108.0731			197.1285	99.0679	I	1488.8090	744.9082	1471.7825	736.3949	1470.7985	735.9029	12
3	344.1816	172.5944			326.1710	163.5892	E	***1375.7250***	688.3661	1358.6984	679.8529	1357.7144	679.3608	11
4	473.2242	237.1157			455.2136	228.1105	E	1246.6824	623.8448	1229.6558	615.3316	1228.6718	614.8395	10
5	586.3083	293.6578			568.2977	284.6525	L	***1117.6398***	559.3235	1100.6132	550.8103	1099.6292	550.3183	9
6	714.3668	357.6871	697.3403	349.1738	696.3563	348.6818	Q	1004.5557	502.7815	987.5292	494.2682	986.5452	493.7762	8
7	842.4254	421.7164	825.3989	413.2031	824.4149	412.7111	Q	***876.4972***	438.7522	859.4706	430.2389	858.4866	429.7469	7
8	970.5204	485.7638	953.4938	477.2506	952.5098	476.7585	K	***748.4386***	374.7229	731.4120	366.2096	730.4280	365.7176	6
9	1083.6045	542.3059	1066.5779	533.7926	1065.5939	533.3006	I	***620.3436***	310.6754	603.3171	302.1622	602.3330	301.6702	5
10	1196.6885	598.8479	1179.6620	590.3346	1178.6780	589.8426	L	***507.2595***	254.1334	490.2330	245.6201	489.2490	245.1281	4
11	1356.7192	678.8632	1339.6926	670.3499	1338.7086	669.8579	C	***394.1755***	197.5914	377.1489	189.0781	376.1649	188.5861	3
12	1443.7512	722.3792	1426.7246	713.8660	1425.7406	713.3740	S	234.1448	117.5761	217.1183	109.0628	216.1343	108.5708	2
13							K	147.1128	74.0600	130.0863	65.5468			1

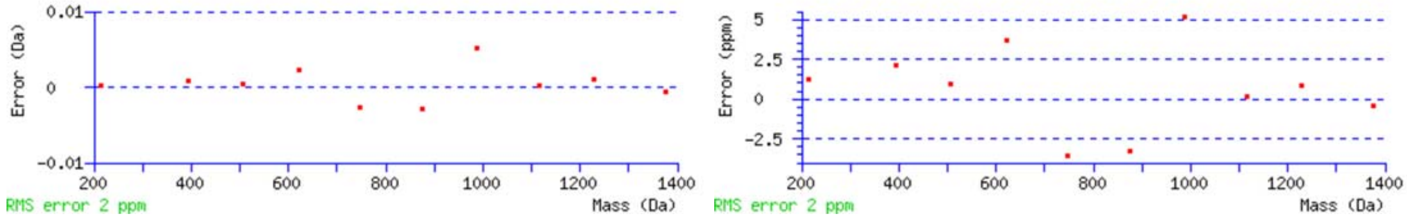

NCBI **BLAST** search of TIEELQQKILCSK
(Parameters: blastp, nr protein database, expect=20000, no filter, PAM30)
Other BLAST web gateways

All matches to this query

Score	Mr(calc)	Delta	Sequence
52.6	1588.8494	-0.0003	TIEELQQKILCSK

Mascot: http://www.matrixscience.com/

Mascot Search Results

Peptide View

MS/MS Fragmentation of **LAADDFR**
Found in **ch17u_O76014|KRT37_HUMAN** in **uni_human**, Keratin, type I cuticular Ha7 OS=Homo sapiens GN=KRT37 PE=3 SV=3

Match to Query 569: 806.392068 from(404.203310,2+) intensity(7757240.0000) rtinseconds(1078) scans(5598) index(34100)
Title: 160219_Sunil_SDSII_A_Spectrum088056_scans__5598_RTINSECONDS=1078
Data file L:\\QE_2016\\160219_Sunil_KAP_LKC\\TMgf\\T\\T160219_Sunil_SDSII_A.mgf

Click mouse within plot area to zoom in by factor of two about that point
Or, Plot from 50 to 700 Da Full range
Label all possible matches ○ Label matches used for scoring ◉
Show Y-axis ☐

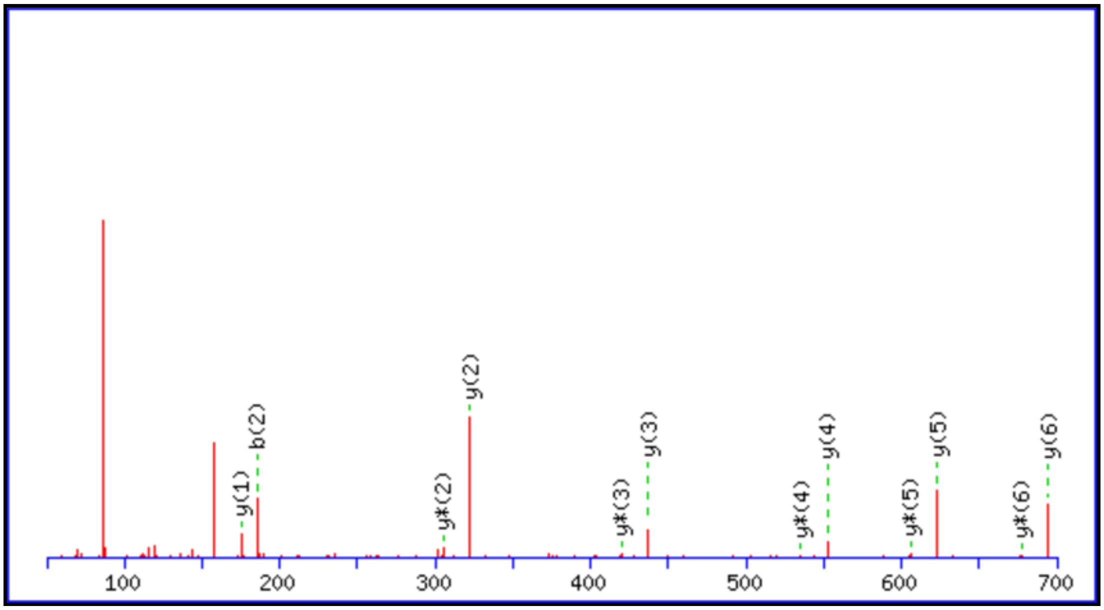

Monoisotopic mass of neutral peptide Mr(calc): 806.3923
Fixed modifications: Carbamidomethyl (C) (apply to specified residues or termini only)
Ions Score: 58 **Expect:** 0.00017
Matches : 12/50 fragment ions using 14 most intense peaks (help)

#	b	b^{++}	b^{0}	b^{0++}	Seq.	y	y^{++}	y*	y^{*++}	y^{0}	y^{0++}	#
1	114.0913	57.5493			L							7
2	**185.1285**	93.0679			A	***694.3155***	347.6614	677.2889	339.1481	676.3049	338.6561	6
3	256.1656	128.5864			A	***623.2784***	312.1428	606.2518	303.6295	605.2678	303.1375	5
4	371.1925	186.0999	353.1819	177.0946	D	***552.2413***	276.6243	535.2147	268.1110	534.2307	267.6190	4
5	486.2195	243.6134	468.2089	234.6081	D	***437.2143***	219.1108	420.1878	210.5975	419.2037	210.1055	3
6	633.2879	317.1476	615.2773	308.1423	F	***322.1874***	161.5973	305.1608	153.0840			2

7					R	*175.1190*	88.0631	158.0924	79.5498			1

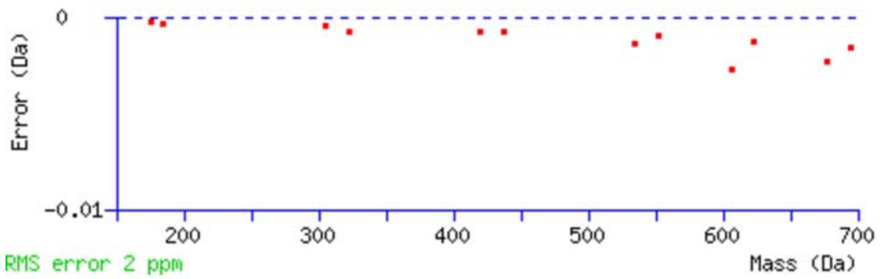

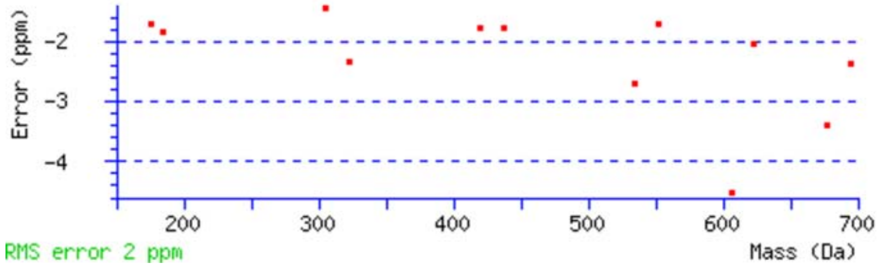

NCBI **BLAST** search of LAADDFR
(Parameters: blastp, nr protein database, expect=20000, no filter, PAM30)
Other BLAST web gateways

All matches to this query

Score	Mr(calc)	Delta	Sequence
58.3	806.3923	-0.0002	LAADDFR
19.6	804.3878	2.0042	RPDNFR
14.5	806.3844	0.0077	LAAQMQK
9.2	806.3923	-0.0002	IAGFDER
8.3	806.3923	-0.0002	LDQQFR
8.3	806.3923	-0.0002	LDQQFR
7.8	806.3922	-0.0002	GELANFR
7.8	804.3878	2.0042	NRPDFR
7.8	806.3923	-0.0002	QLEDFR
4.0	806.3891	0.0030	IACCRK

Mascot: http://www.matrixscience.com/

Mascot Search Results

Peptide View

MS/MS Fragmentation of **TIEELQQKILCSK**
Found in **ch17u_O76014|KRT37_HUMAN** in **uni_human**, Keratin, type I cuticular Ha7 OS=Homo sapiens GN=KRT37 PE=3 SV=3

Match to Query 21082: 1588.849332 from(530.623720,3+) intensity(2398165.5000) rtinseconds(2216) scans(11794) index(9213)
Title: 160219_Sunil_SDSII_A_Spectrum060537_scans__11794_RTINSECONDS=2216
Data file L:\\QE_2016\\160219_Sunil_KAP_LKC\\TMgf\\T\\T160219_Sunil_SDSII_A.mgf

Click mouse within plot area to zoom in by factor of two about that point
Or, Plot from 100 to 1600 Da Full range
Label all possible matches ○ Label matches used for scoring ◉
Show Y-axis ☐

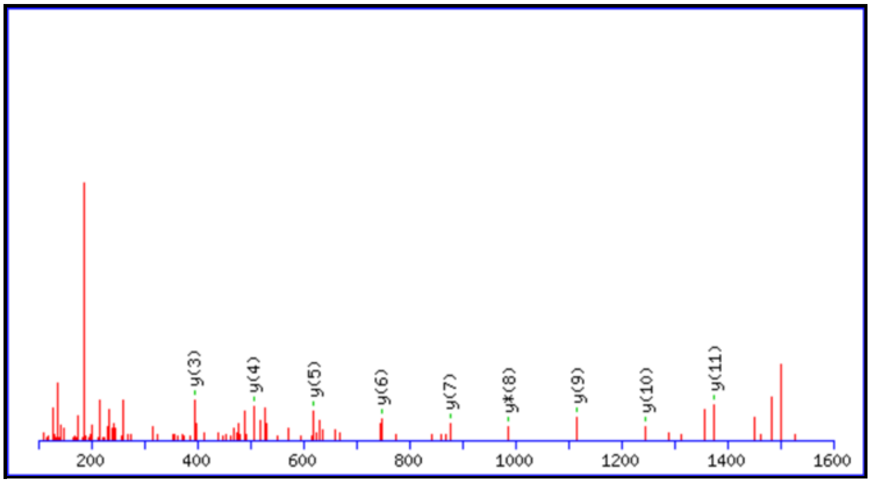

Monoisotopic mass of neutral peptide Mr(calc): 1588.8494
Fixed modifications: Carbamidomethyl (C) (apply to specified residues or termini only)
Ions Score: 56 **Expect:** 0.00039
Matches : 9/132 fragment ions using 14 most intense peaks (help)

#	b	b^{++}	b*	b^{*++}	b^0	b^{0++}	Seq.	y	y^{++}	y*	y^{*++}	y^0	y^{0++}	#
1	102.0550	51.5311			84.0444	42.5258	T							13
2	215.1390	108.0731			197.1285	99.0679	I	1488.8090	744.9082	1471.7825	736.3949	1470.7985	735.9029	12
3	344.1816	172.5944			326.1710	163.5892	E	***1375.7250***	688.3661	1358.6984	679.8529	1357.7144	679.3608	11
4	473.2242	237.1157			455.2136	228.1105	E	***1246.6824***	623.8448	1229.6558	615.3316	1228.6718	614.8395	10
5	586.3083	293.6578			568.2977	284.6525	L	***1117.6398***	559.3235	1100.6132	550.8103	1099.6292	550.3183	9
6	714.3668	357.6871	697.3403	349.1738	696.3563	348.6818	Q	1004.5557	502.7815	987.5292	494.2682	986.5452	493.7762	8
7	842.4254	421.7164	825.3989	413.2031	824.4149	412.7111	Q	***876.4972***	438.7522	859.4706	430.2389	858.4866	429.7469	7
8	970.5204	485.7638	953.4938	477.2506	952.5098	476.7585	K	***748.4386***	374.7229	731.4120	366.2096	730.4280	365.7176	6
9	1083.6045	542.3059	1066.5779	533.7926	1065.5939	533.3006	I	***620.3436***	310.6754	603.3171	302.1622	602.3330	301.6702	5
10	1196.6885	598.8479	1179.6620	590.3346	1178.6780	589.8426	L	***507.2595***	254.1334	490.2330	245.6201	489.2490	245.1281	4
11	1356.7192	678.8632	1339.6926	670.3499	1338.7086	669.8579	C	***394.1755***	197.5914	377.1489	189.0781	376.1649	188.5861	3
12	1443.7512	722.3792	1426.7246	713.8660	1425.7406	713.3740	S	234.1448	117.5761	217.1183	109.0628	216.1343	108.5708	2
13							K	147.1128	74.0600	130.0863	65.5468			1

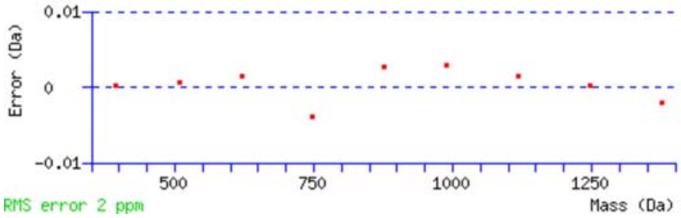

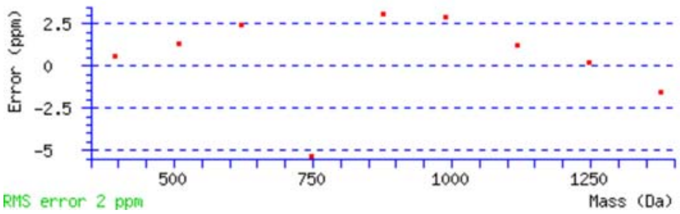

NCBI **BLAST** search of TIEELQQKILCSK
(Parameters: blastp, nr protein database, expect=20000, no filter, PAM30)
Other BLAST web gateways

All matches to this query

Score	Mr(calc)	Delta	Sequence
55.7	1588.8494	-0.0001	TIEELQQKILCSK

Mascot: http://www.matrixscience.com/

Mascot Search Results

Peptide View

MS/MS Fragmentation of **TIEELQQKILCSK**
Found in **ch17u_O76014|KRT37_HUMAN** in **uni_human**, Keratin, type I cuticular Ha7 OS=Homo sapiens GN=KRT37 PE=3 SV=3

Match to Query 21086: 1588.850232 from(530.624020,3+) intensity(378250.7813) rtinseconds(1938) scans(10565) index(38476)
Title: 160219_Sunil_SDSII_A_Spectrum092434_scans__10565_RTINSECONDS=1938
Data file L:\\QE_2016\\160219_Sunil_KAP_LKC\\TMgf\\T\\T160219_Sunil_SDSII_A.mgf

Click mouse within plot area to zoom in by factor of two about that point
Or, Plot from 100 to 1600 Da Full range
Label all possible matches ○ Label matches used for scoring ◉
Show Y-axis ☐

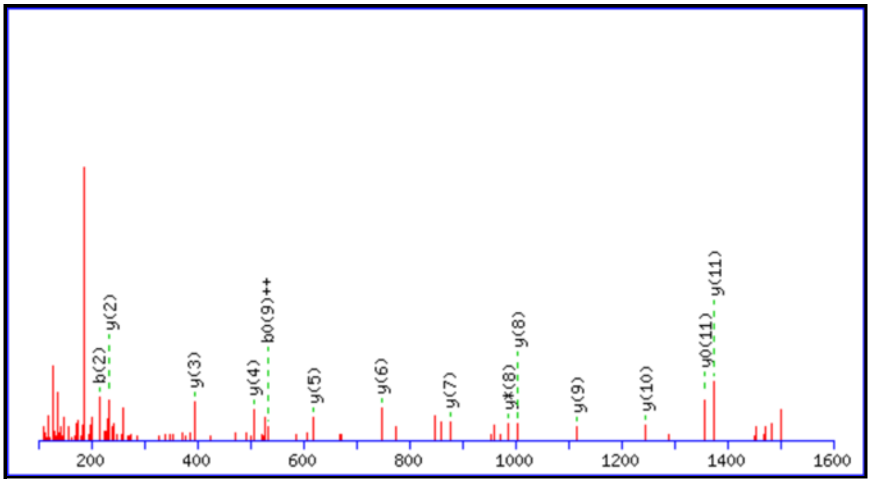

Monoisotopic mass of neutral peptide Mr(calc): 1588.8494
Fixed modifications: Carbamidomethyl (C) (apply to specified residues or termini only)
Ions Score: 69 **Expect:** 1.7e-005
Matches : 14/132 fragment ions using 25 most intense peaks (help)

#	b	b^{++}	b*	b^{*++}	b^0	b^{0++}	Seq.	y	y^{++}	y*	y^{*++}	y^0	y^{0++}	#
1	102.0550	51.5311			84.0444	42.5258	T							13
2	**215.1390**	108.0731			197.1285	99.0679	I	1488.8090	744.9082	1471.7825	736.3949	1470.7985	735.9029	12
3	344.1816	172.5944			326.1710	163.5892	E	***1375.7250***	688.3661	1358.6984	679.8529	1357.7144	679.3608	11
4	473.2242	237.1157			455.2136	228.1105	E	***1246.6824***	623.8448	1229.6558	615.3316	1228.6718	614.8395	10
5	586.3083	293.6578			568.2977	284.6525	L	***1117.6398***	559.3235	1100.6132	550.8103	1099.6292	550.3183	9
6	714.3668	357.6871	697.3403	349.1738	696.3563	348.6818	Q	***1004.5557***	502.7815	987.5292	494.2682	986.5452	493.7762	8
7	842.4254	421.7164	825.3989	413.2031	824.4149	412.7111	Q	***876.4972***	438.7522	859.4706	430.2389	858.4866	429.7469	7
8	970.5204	485.7638	953.4938	477.2506	952.5098	476.7585	K	***748.4386***	374.7229	731.4120	366.2096	730.4280	365.7176	6
9	1083.6045	542.3059	1066.5779	533.7926	1065.5939	533.3006	I	***620.3436***	310.6754	603.3171	302.1622	602.3330	301.6702	5
10	1196.6885	598.8479	1179.6620	590.3346	1178.6780	589.8426	L	***507.2595***	254.1334	490.2330	245.6201	489.2490	245.1281	4
11	1356.7192	678.8632	1339.6926	670.3499	1338.7086	669.8579	C	***394.1755***	197.5914	377.1489	189.0781	376.1649	188.5861	3
12	1443.7512	722.3792	1426.7246	713.8660	1425.7406	713.3740	S	***234.1448***	117.5761	217.1183	109.0628	216.1343	108.5708	2
13							K	147.1128	74.0600	130.0863	65.5468			1

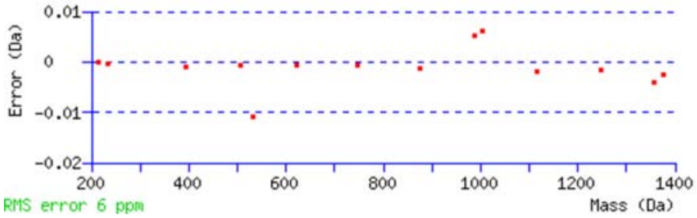

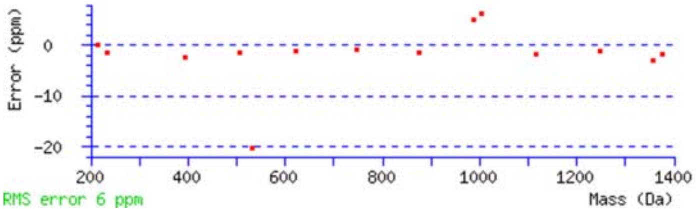

NCBI **BLAST** search of TIEELQQKILCSK
(Parameters: blastp, nr protein database, expect=20000, no filter, PAM30)
Other BLAST web gateways

All matches to this query

Score	Mr(calc)	Delta	Sequence
69.3	1588.8494	0.0008	TIEELQQKILCSK

Mascot: http://www.matrixscience.com/

Mascot Search Results

Peptide View

MS/MS Fragmentation of **LAADDFR**
Found in **ch17u_O76014|KRT37_HUMAN** in **uni_human**, Keratin, type I cuticular Ha7 OS=Homo sapiens GN=KRT37 PE=3 SV=3

Match to Query 548: 806.390608 from(404.202580,2+) intensity(1764171.6250) rtinseconds(1018) scans(5155) index(18593)
Title: 160219_Sunil_SDSII_A_Spectrum071154_scans__5155_RTINSECONDS=1018
Data file L:\\QE_2016\\160219_Sunil_KAP_LKC\\TMgf\\T\\T160219_Sunil_SDSII_A.mgf

Click mouse within plot area to zoom in by factor of two about that point
Or, Plot from 50 to 750 Da Full range
Label all possible matches ○ Label matches used for scoring ◉
Show Y-axis ☐

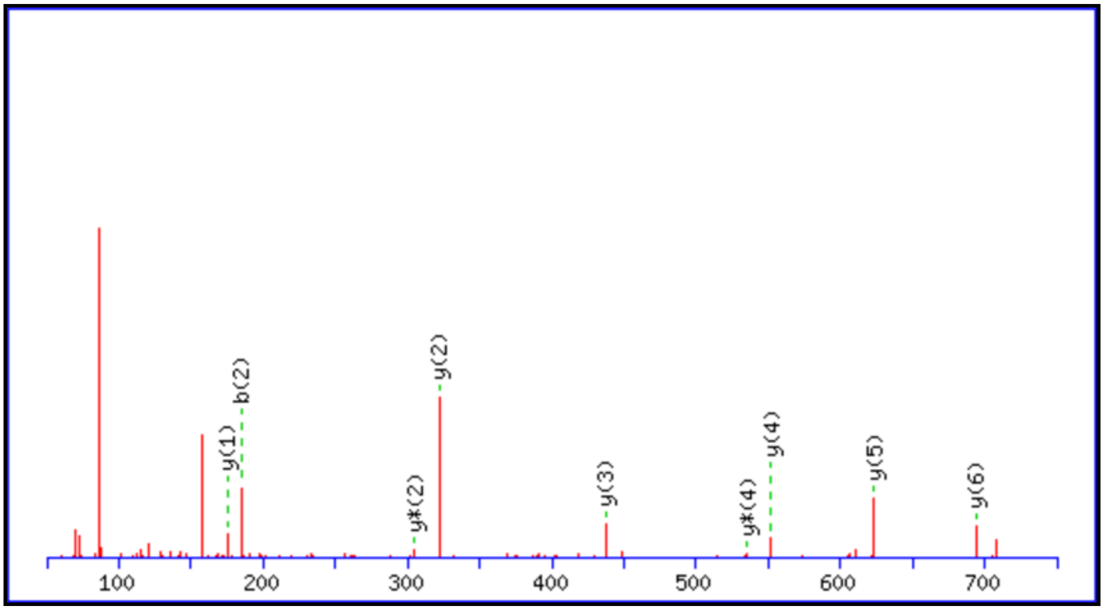

Monoisotopic mass of neutral peptide Mr(calc): 806.3923
Fixed modifications: Carbamidomethyl (C) (apply to specified residues or termini only)
Ions Score: 49 **Expect:** 0.0017
Matches : 9/50 fragment ions using 14 most intense peaks (help)

#	b	b^{++}	b^0	b^{0++}	Seq.	y	y^{++}	y*	y^{*++}	y^0	y^{0++}	#
1	114.0913	57.5493			L							7
2	**185.1285**	93.0679			A	***694.3155***	347.6614	677.2889	339.1481	676.3049	338.6561	6
3	256.1656	128.5864			A	***623.2784***	312.1428	606.2518	303.6295	605.2678	303.1375	5
4	371.1925	186.0999	353.1819	177.0946	D	***552.2413***	276.6243	535.2147	268.1110	534.2307	267.6190	4
5	486.2195	243.6134	468.2089	234.6081	D	***437.2143***	219.1108	420.1878	210.5975	419.2037	210.1055	3
6	633.2879	317.1476	615.2773	308.1423	F	***322.1874***	161.5973	305.1608	153.0840			2

7					R	175.1190	88.0631	158.0924	79.5498			1

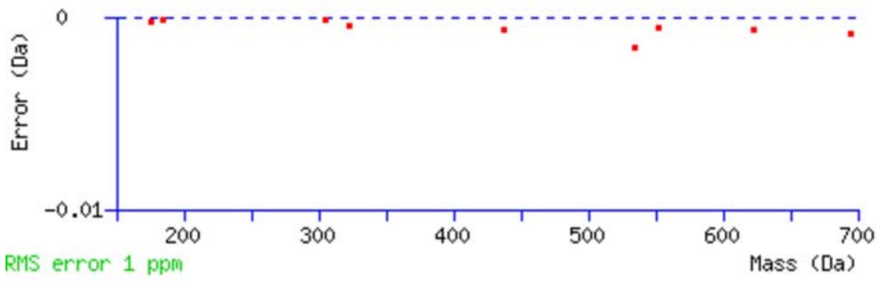

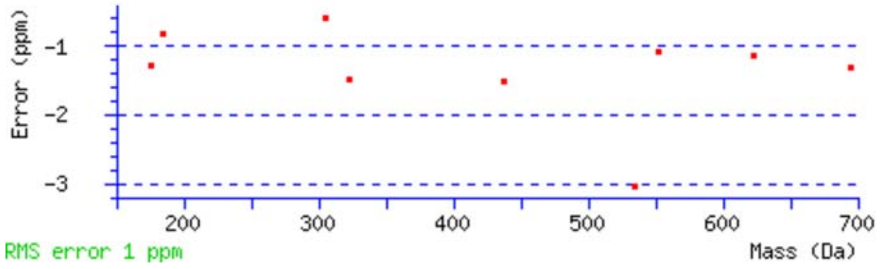

NCBI **BLAST** search of LAADDFR
(Parameters: blastp, nr protein database, expect=20000, no filter, PAM30)
Other BLAST web gateways

All matches to this query

Score	Mr(calc)	Delta	Sequence
48.6	806.3923	-0.0017	LAADDFR
17.7	804.3878	2.0028	RPDNFR
13.8	806.3844	0.0062	LAAQMQK
9.9	806.3923	-0.0017	IAGFDER
6.4	806.3922	-0.0016	GELANFR
6.4	804.3878	2.0028	NRPDFR
6.4	806.3923	-0.0017	QLEDFR
6.4	806.3923	-0.0017	LDQQFR
6.4	806.3923	-0.0017	LDQQFR
6.1	804.3919	1.9987	PWQAFR

Mascot: http://www.matrixscience.com/

Mascot Search Results

Peptide View

MS/MS Fragmentation of **LAADDFR**
Found in **ch17u_O76014|KRT37_HUMAN** in **uni_human**, Keratin, type I cuticular Ha7 OS=Homo sapiens GN=KRT37 PE=3 SV=3

Match to Query 549: 806.390668 from(404.202610,2+) intensity(899590.2500) rtinseconds(1549) scans(7916) index(5819)
Title: 160219_Sunil_SDSII_A_Spectrum057142_scans__7916_RTINSECONDS=1549
Data file L:\\QE_2016\\160219_Sunil_KAP_LKC\\TMgf\\T\\T160219_Sunil_SDSII_A.mgf

Click mouse within plot area to zoom in by factor of two about that point
Or, Plot from 50 to 750 Da Full range
Label all possible matches ○ Label matches used for scoring ◉
Show Y-axis ☐

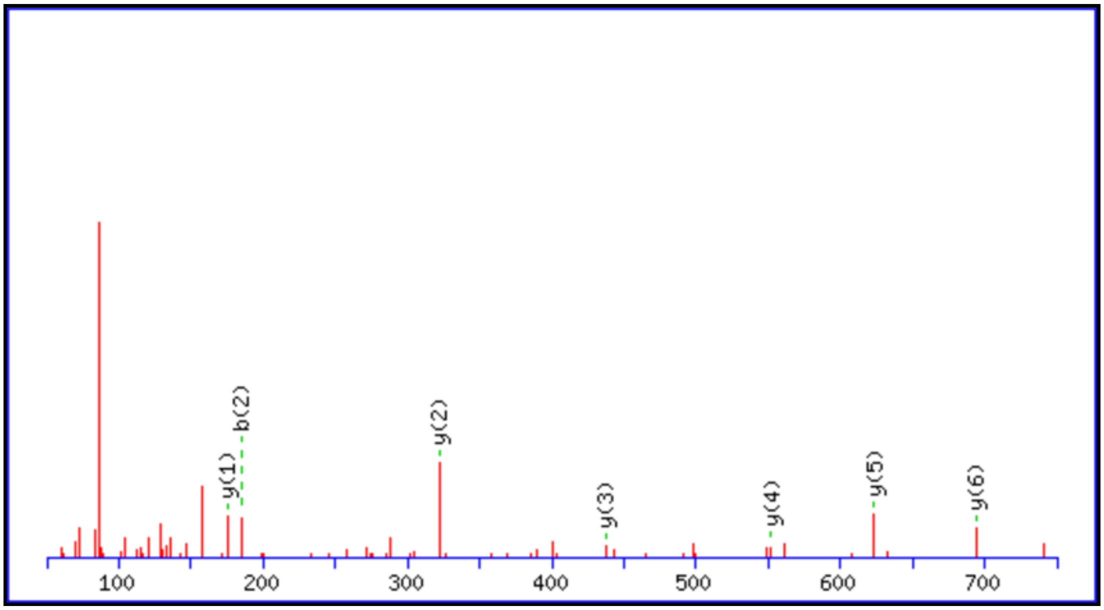

Monoisotopic mass of neutral peptide Mr(calc): 806.3923
Fixed modifications: Carbamidomethyl (C) (apply to specified residues or termini only)
Ions Score: 41 **Expect:** 0.0094
Matches : 7/50 fragment ions using 14 most intense peaks (help)

#	b	b^{++}	b^0	b^{0++}	Seq.	y	y^{++}	y^*	y^{*++}	y^0	y^{0++}	#
1	114.0913	57.5493			L							7
2	**185.1285**	93.0679			A	***694.3155***	347.6614	677.2889	339.1481	676.3049	338.6561	6
3	256.1656	128.5864			A	***623.2784***	312.1428	606.2518	303.6295	605.2678	303.1375	5
4	371.1925	186.0999	353.1819	177.0946	D	***552.2413***	276.6243	535.2147	268.1110	534.2307	267.6190	4
5	486.2195	243.6134	468.2089	234.6081	D	***437.2143***	219.1108	420.1878	210.5975	419.2037	210.1055	3
6	633.2879	317.1476	615.2773	308.1423	F	***322.1874***	161.5973	305.1608	153.0840			2

**7**					**R**	*175.1190*	88.0631	158.0924	79.5498			**1**

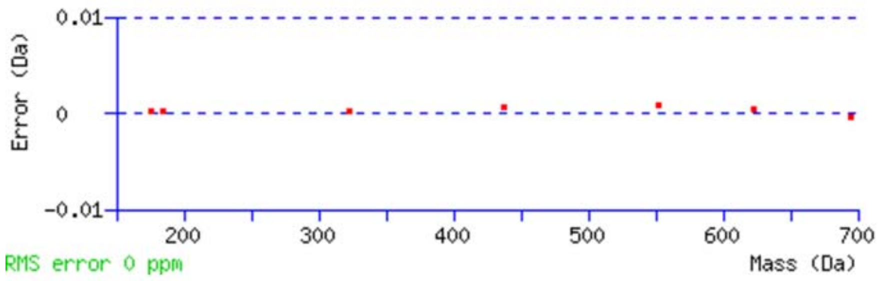

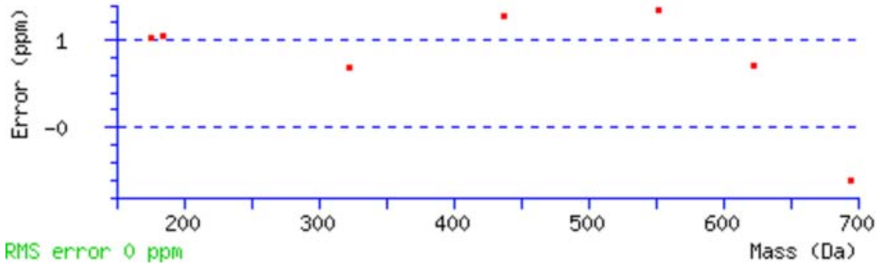

NCBI **BLAST** search of LAADDFR
(Parameters: blastp, nr protein database, expect=20000, no filter, PAM30)
Other BLAST web gateways

All matches to this query

Score	Mr(calc)	Delta	Sequence
41.0	806.3923	-0.0016	LAADDFR
11.5	804.3878	2.0028	RPDNFR
10.9	806.3923	-0.0016	LDQQFR
10.9	806.3923	-0.0016	LDQQFR
9.3	806.3923	-0.0016	IAGFDER
5.3	806.3956	-0.0049	MGNSLLR
3.8	806.3844	0.0063	LAAQMQK
3.1	806.3922	-0.0016	GELANFR
3.1	804.3878	2.0028	NRPDFR
3.1	806.3923	-0.0016	QLEDFR

Mascot: http://www.matrixscience.com/

Mascot Search Results

Peptide View

MS/MS Fragmentation of **LAADDFR**
Found in **ch17u_O76014|KRT37_HUMAN** in **uni_human**, Keratin, type I cuticular Ha7 OS=Homo sapiens GN=KRT37 PE=3 SV=3

Match to Query 550: 806.390968 from(404.202760,2+) intensity(366711.5000) rtinseconds(1710) scans(8858) index(6655)
Title: 160219_Sunil_SDSII_A_Spectrum057978_scans__8858_RTINSECONDS=1710
Data file L:\\QE_2016\\160219_Sunil_KAP_LKC\\TMgf\\T\\T160219_Sunil_SDSII_A.mgf

Click mouse within plot area to zoom in by factor of two about that point
Or, Plot from 50 to 700 Da Full range
Label all possible matches ○ Label matches used for scoring ◉
Show Y-axis ☐

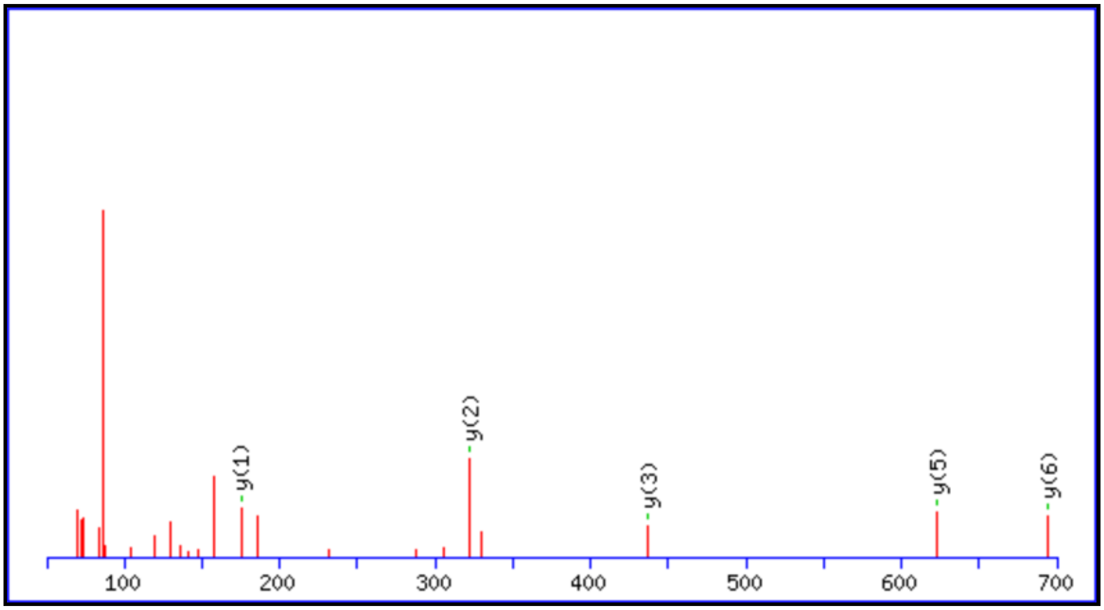

Monoisotopic mass of neutral peptide Mr(calc): 806.3923
Fixed modifications: Carbamidomethyl (C) (apply to specified residues or termini only)
Ions Score: 45 **Expect:** 0.0037
Matches : 5/50 fragment ions using 6 most intense peaks (help)

#	b	b^{++}	b^0	b^{0++}	Seq.	y	y^{++}	y*	y^{*++}	y^0	y^{0++}	#
1	114.0913	57.5493			L							7
2	185.1285	93.0679			A	***694.3155***	347.6614	677.2889	339.1481	676.3049	338.6561	6
3	256.1656	128.5864			A	***623.2784***	312.1428	606.2518	303.6295	605.2678	303.1375	5
4	371.1925	186.0999	353.1819	177.0946	D	552.2413	276.6243	535.2147	268.1110	534.2307	267.6190	4
5	486.2195	243.6134	468.2089	234.6081	D	***437.2143***	219.1108	420.1878	210.5975	419.2037	210.1055	3
6	633.2879	317.1476	615.2773	308.1423	F	***322.1874***	161.5973	305.1608	153.0840			2

7					R	*175.1190*	88.0631	158.0924	79.5498			1

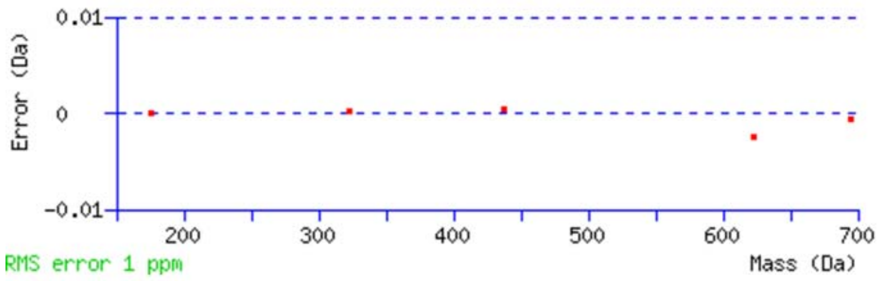

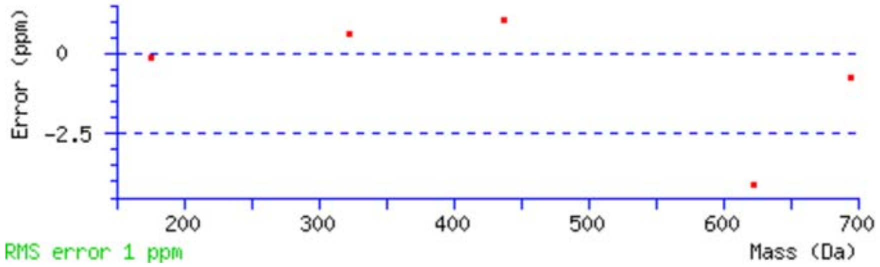

NCBI **BLAST** search of LAADDFR
(Parameters: blastp, nr protein database, expect=20000, no filter, PAM30)
Other BLAST web gateways

All matches to this query

Score	Mr(calc)	Delta	Sequence
45.1	806.3923	-0.0013	LAADDFR
13.4	806.3923	-0.0013	LDQQFR
13.4	806.3923	-0.0013	LDQQFR
13.2	806.3922	-0.0013	GELANFR
13.2	804.3878	2.0031	NRPDFR
13.2	806.3923	-0.0013	QLEDFR
13.2	804.3878	2.0031	RPDNFR
11.9	806.3923	-0.0013	IAGFDER
4.8	806.3891	0.0019	IACCRK
4.8	806.3956	-0.0047	LACTNTK

**Mascot:** http://www.matrixscience.com/

Mascot Search Results

Peptide View

MS/MS Fragmentation of **LAADDFR**
Found in **ch17u_O76014|KRT37_HUMAN** in **uni_human**, Keratin, type I cuticular Ha7 OS=Homo sapiens GN=KRT37 PE=3 SV=3

Match to Query 551: 806.391268 from(404.202910,2+) intensity(924215.0625) rtinseconds(1275) scans(6314) index(4440)
Title: 160219_Sunil_SDSII_A_Spectrum055763_scans__6314_RTINSECONDS=1275
Data file L:\\QE_2016\\160219_Sunil_KAP_LKC\\TMgf\\T\\T160219_Sunil_SDSII_A.mgf

Click mouse within plot area to zoom in by factor of two about that point
Or, Plot from 50 to 750 Da Full range
Label all possible matches ○ Label matches used for scoring ◉
Show Y-axis ☐

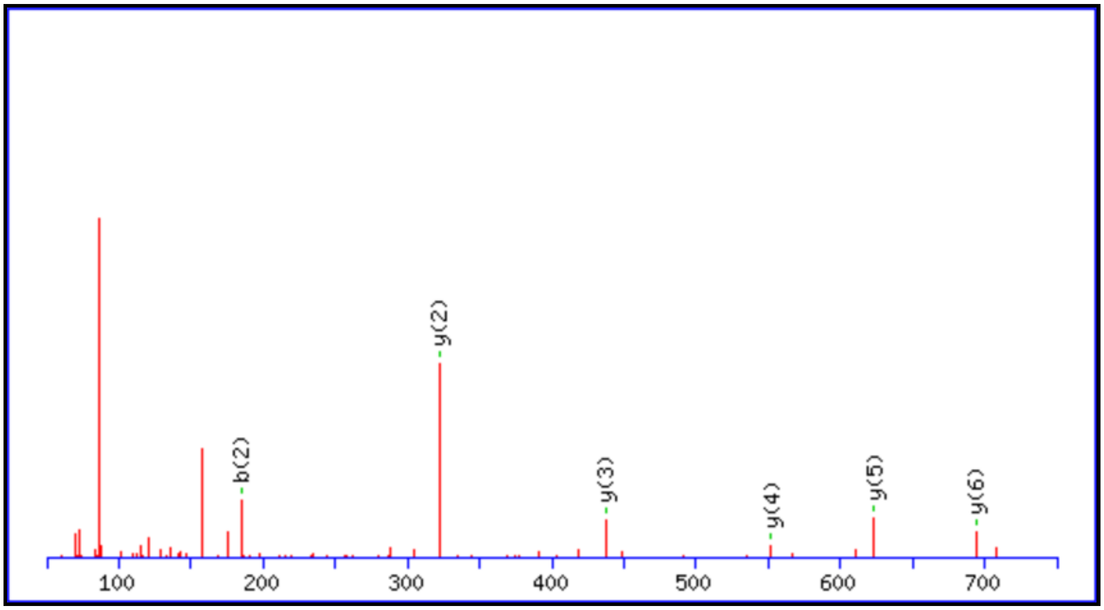

Monoisotopic mass of neutral peptide Mr(calc): 806.3923
Fixed modifications: Carbamidomethyl (C) (apply to specified residues or termini only)
Ions Score: 47 **Expect:** 0.0025
Matches : 6/50 fragment ions using 7 most intense peaks (help)

#	b	b++	b0	b0++	Seq.	y	y++	y*	y*++	y0	y0++	#
1	114.0913	57.5493			L							7
2	**185.1285**	93.0679			A	***694.3155***	347.6614	677.2889	339.1481	676.3049	338.6561	6
3	256.1656	128.5864			A	***623.2784***	312.1428	606.2518	303.6295	605.2678	303.1375	5
4	371.1925	186.0999	353.1819	177.0946	D	***552.2413***	276.6243	535.2147	268.1110	534.2307	267.6190	4
5	486.2195	243.6134	468.2089	234.6081	D	***437.2143***	219.1108	420.1878	210.5975	419.2037	210.1055	3
6	633.2879	317.1476	615.2773	308.1423	F	***322.1874***	161.5973	305.1608	153.0840			2

7					R	175.1190	88.0631	158.0924	79.5498			1

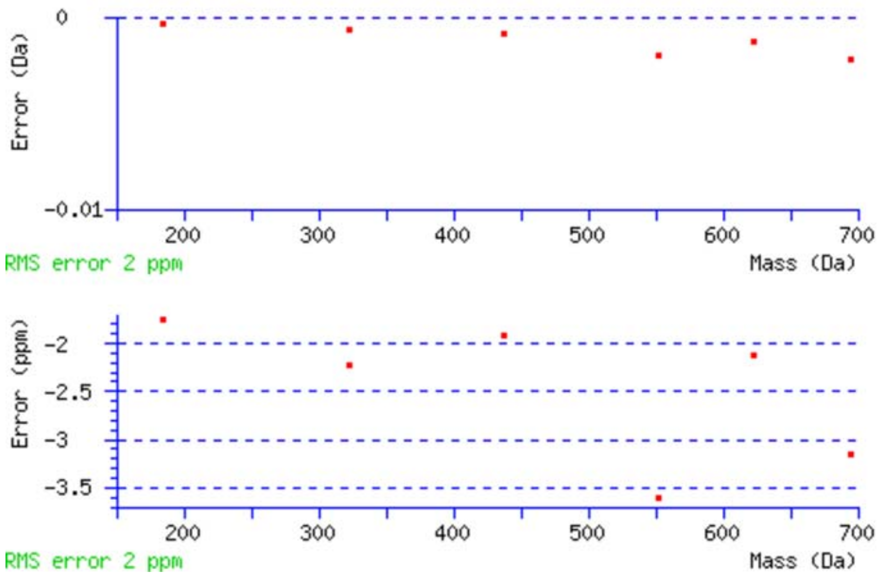

NCBI **BLAST** search of LAADDFR
(Parameters: blastp, nr protein database, expect=20000, no filter, PAM30)
Other BLAST web gateways

All matches to this query

Score	Mr(calc)	Delta	Sequence
46.7	806.3923	-0.0010	LAADDFR
13.8	804.3878	2.0034	RPDNFR
13.6	806.3923	-0.0010	IAGFDER
12.5	806.3844	0.0069	LAAQMQK
7.1	804.3878	2.0034	NRPDFR
7.1	806.3923	-0.0010	QLEDFR
7.0	806.3922	-0.0010	GELANFR
5.3	806.3923	-0.0010	LDQQFR
5.3	806.3923	-0.0010	LDQQFR
5.0	804.3919	1.9994	PWQAFR

Mascot: http://www.matrixscience.com/

Mascot Search Results

Peptide View

MS/MS Fragmentation of **LAADDFR**
Found in **ch17u_O76014|KRT37_HUMAN** in **uni_human**, Keratin, type I cuticular Ha7 OS=Homo sapiens GN=KRT37 PE=3 SV=3

Match to Query 553: 806.391328 from(404.202940,2+) intensity(501542.6250) rtinseconds(1101) scans(5632) index(18982)
Title: 160219_Sunil_SDSII_A_Spectrum071543_scans__5632_RTINSECONDS=1101
Data file L:\\QE_2016\\160219_Sunil_KAP_LKC\\TMgf\\T\\T160219_Sunil_SDSII_A.mgf

Click mouse within plot area to zoom in by factor of two about that point
Or, Plot from 50 to 700 Da Full range
Label all possible matches ○ Label matches used for scoring ◉
Show Y-axis ☐

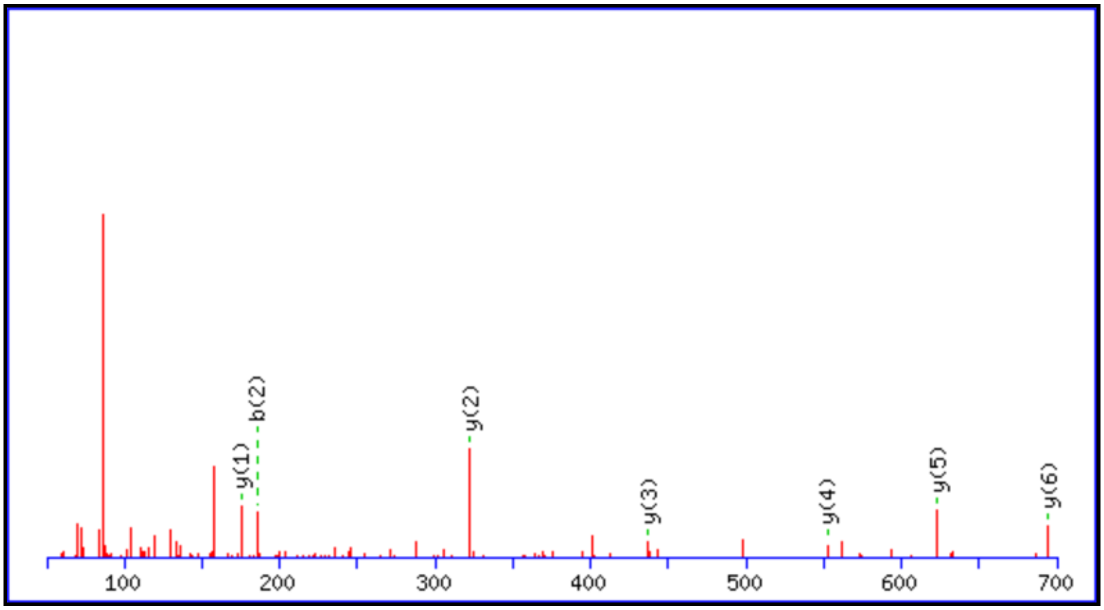

Monoisotopic mass of neutral peptide Mr(calc): 806.3923
Fixed modifications: Carbamidomethyl (C) (apply to specified residues or termini only)
Ions Score: 41 **Expect:** 0.0088
Matches : 7/50 fragment ions using 14 most intense peaks (help)

#	b	b^{++}	b^0	b^{0++}	Seq.	y	y^{++}	y*	y^{*++}	y^0	y^{0++}	#
1	114.0913	57.5493			L							7
2	**185.1285**	93.0679			A	***694.3155***	347.6614	677.2889	339.1481	676.3049	338.6561	6
3	256.1656	128.5864			A	***623.2784***	312.1428	606.2518	303.6295	605.2678	303.1375	5
4	371.1925	186.0999	353.1819	177.0946	D	***552.2413***	276.6243	535.2147	268.1110	534.2307	267.6190	4
5	486.2195	243.6134	468.2089	234.6081	D	***437.2143***	219.1108	420.1878	210.5975	419.2037	210.1055	3
6	633.2879	317.1476	615.2773	308.1423	F	***322.1874***	161.5973	305.1608	153.0840			2

7					R	*175.1190*	88.0631	158.0924	79.5498			1

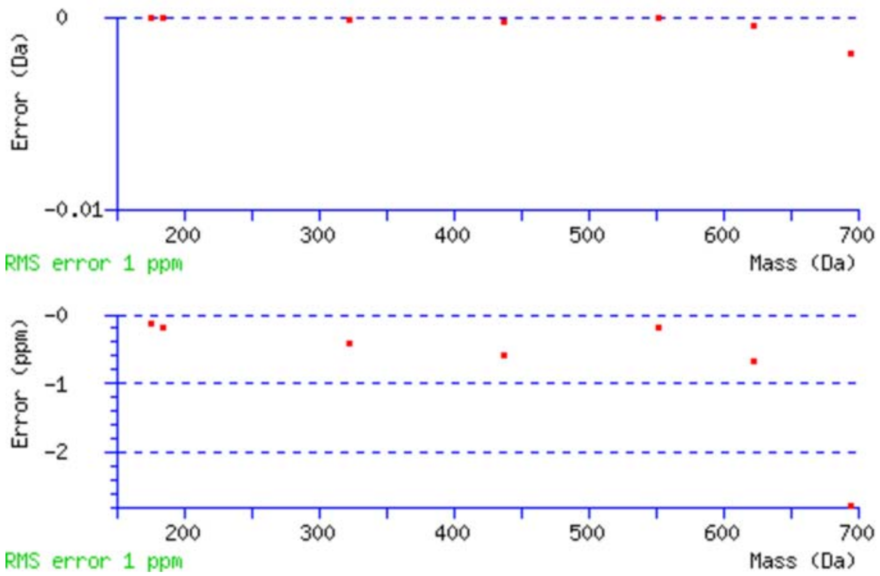

NCBI **BLAST** search of LAADDFR
(Parameters: blastp, nr protein database, expect=20000, no filter, PAM30)
Other BLAST web gateways

All matches to this query

Score	Mr(calc)	Delta	Sequence
41.3	806.3923	-0.0009	LAADDFR
11.9	804.3878	2.0035	RPDNFR
11.1	806.3923	-0.0009	LDQQFR
11.1	806.3923	-0.0009	LDQQFR
9.4	806.3923	-0.0009	IAGFDER
5.4	806.3956	-0.0043	MGNSLLR
3.8	806.3844	0.0070	LAAQMQK
3.6	806.3922	-0.0009	GELANFR
3.5	804.3878	2.0035	NRPDFR
3.5	806.3923	-0.0009	QLEDFR

Mascot: http://www.matrixscience.com/

Mascot Search Results

Peptide View

MS/MS Fragmentation of **LAADDFR**
Found in **ch17u_O76014|KRT37_HUMAN** in **uni_human**, Keratin, type I cuticular Ha7 OS=Homo sapiens GN=KRT37 PE=3 SV=3

Match to Query 554: 806.391388 from(404.202970,2+) intensity(415041.4063) rtinseconds(1519) scans(8052) index(21109)
Title: 160219_Sunil_SDSII_A_Spectrum073670_scans__8052_RTINSECONDS=1519
Data file L:\\QE_2016\\160219_Sunil_KAP_LKC\\TMgf\\T\\T160219_Sunil_SDSII_A.mgf

Click mouse within plot area to zoom in by factor of two about that point
Or, Plot from 50 to 700 Da Full range
Label all possible matches ○ Label matches used for scoring ◉
Show Y-axis ☐

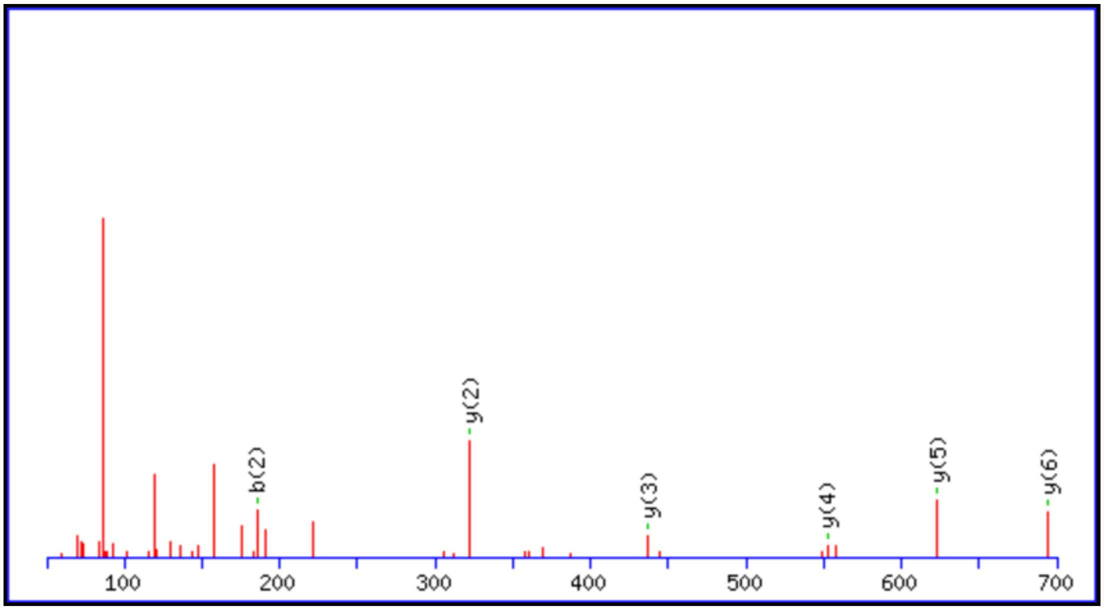

Monoisotopic mass of neutral peptide Mr(calc): 806.3923
Fixed modifications: Carbamidomethyl (C) (apply to specified residues or termini only)
Ions Score: 46 **Expect:** 0.003
Matches : 6/50 fragment ions using 7 most intense peaks (help)

#	b	b++	b0	b0++	Seq.	y	y++	y*	y*++	y0	y0++	#
1	114.0913	57.5493			L							7
2	**185.1285**	93.0679			A	***694.3155***	347.6614	677.2889	339.1481	676.3049	338.6561	6
3	256.1656	128.5864			A	***623.2784***	312.1428	606.2518	303.6295	605.2678	303.1375	5
4	371.1925	186.0999	353.1819	177.0946	D	***552.2413***	276.6243	535.2147	268.1110	534.2307	267.6190	4
5	486.2195	243.6134	468.2089	234.6081	D	***437.2143***	219.1108	420.1878	210.5975	419.2037	210.1055	3
6	633.2879	317.1476	615.2773	308.1423	F	***322.1874***	161.5973	305.1608	153.0840			2

**7**					**R**	175.1190	88.0631	158.0924	79.5498			**1**

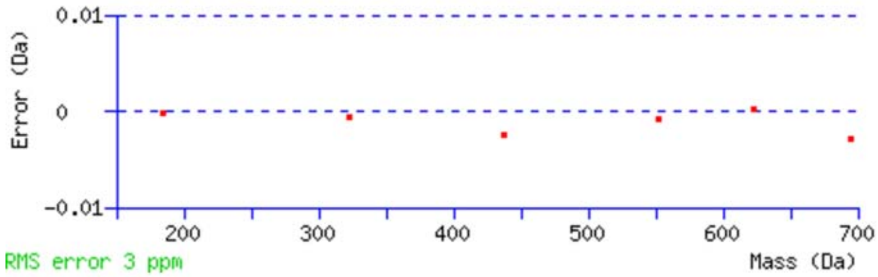

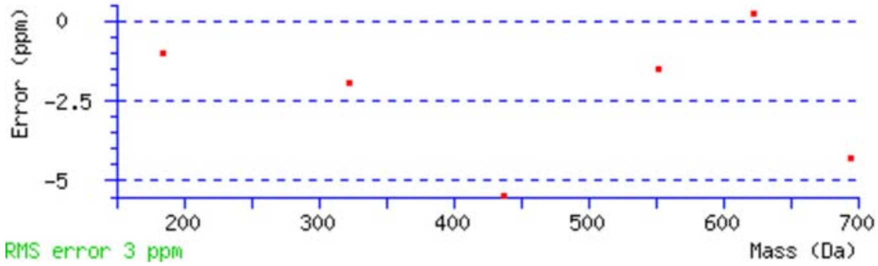

NCBI **BLAST** search of LAADDFR
(Parameters: blastp, nr protein database, expect=20000, no filter, PAM30)
Other BLAST web gateways

All matches to this query

Score	Mr(calc)	Delta	Sequence
45.7	806.3923	-0.0009	LAADDFR
13.2	806.3844	0.0070	LAAQMQK
11.3	804.3878	2.0036	RPDNFR
4.7	806.3923	-0.0009	IAGFDER
4.6	806.3923	-0.0009	QLEDFR
3.9	806.3923	-0.0009	LDQQFR
3.9	806.3923	-0.0009	LDQQFR
3.3	806.3922	-0.0009	GELANFR
3.3	804.3878	2.0036	NRPDFR
2.7	806.3891	0.0023	IACCRK

Mascot: http://www.matrixscience.com/

Mascot Search Results

Peptide View

MS/MS Fragmentation of **LAADDFR**
Found in **ch17u_O76014|KRT37_HUMAN** in **uni_human**, Keratin, type I cuticular Ha7 OS=Homo sapiens GN=KRT37 PE=3 SV=3

Match to Query 555: 806.391508 from(404.203030,2+) intensity(521091.1563) rtinseconds(1486) scans(7865) index(20942)
Title: 160219_Sunil_SDSII_A_Spectrum073503_scans__7865_RTINSECONDS=1486
Data file L:\\QE_2016\\160219_Sunil_KAP_LKC\\TMgf\\T\\T160219_Sunil_SDSII_A.mgf

Click mouse within plot area to zoom in by factor of two about that point
Or, Plot from 50 to 700 Da Full range
Label all possible matches ○ Label matches used for scoring ◉
Show Y-axis ☐

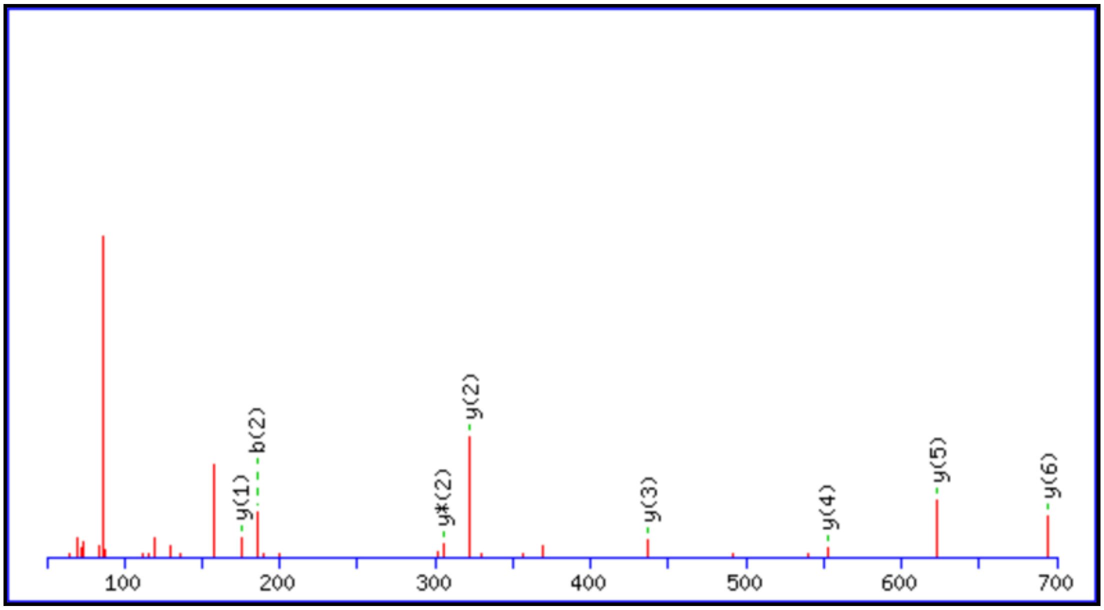

Monoisotopic mass of neutral peptide Mr(calc): 806.3923
Fixed modifications: Carbamidomethyl (C) (apply to specified residues or termini only)
Ions Score: 50 **Expect:** 0.001
Matches : 8/50 fragment ions using 12 most intense peaks (help)

#	b	b^{++}	b^0	b^{0++}	Seq.	y	y^{++}	y*	y^{*++}	y^0	y^{0++}	#
1	114.0913	57.5493			L							7
2	**185.1285**	93.0679			A	***694.3155***	347.6614	677.2889	339.1481	676.3049	338.6561	6
3	256.1656	128.5864			A	***623.2784***	312.1428	606.2518	303.6295	605.2678	303.1375	5
4	371.1925	186.0999	353.1819	177.0946	D	***552.2413***	276.6243	535.2147	268.1110	534.2307	267.6190	4
5	486.2195	243.6134	468.2089	234.6081	D	***437.2143***	219.1108	420.1878	210.5975	419.2037	210.1055	3
6	633.2879	317.1476	615.2773	308.1423	F	***322.1874***	161.5973	305.1608	153.0840			2

7					R	*175.1190*	88.0631	158.0924	79.5498			1

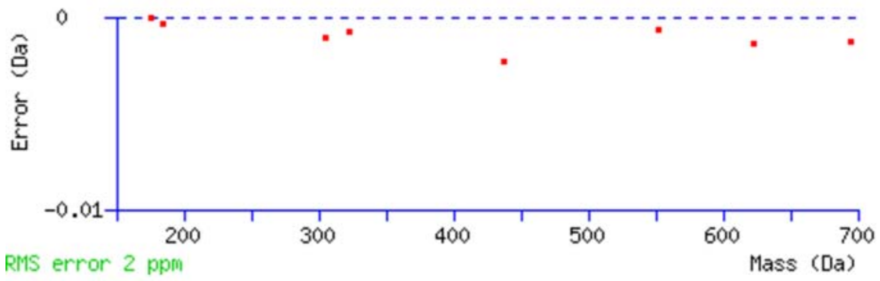

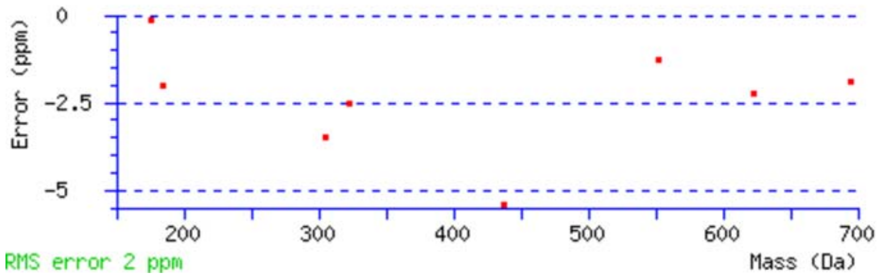

NCBI **BLAST** search of LAADDFR
(Parameters: blastp, nr protein database, expect=20000, no filter, PAM30)
Other BLAST web gateways

All matches to this query

Score	Mr(calc)	Delta	Sequence
50.4	806.3923	-0.0008	LAADDFR
17.6	804.3878	2.0037	RPDNFR
13.9	806.3844	0.0071	LAAQMQK
8.2	806.3923	-0.0008	LDQQFR
8.2	806.3923	-0.0008	LDQQFR
7.6	806.3922	-0.0007	GELANFR
7.6	804.3878	2.0037	NRPDFR
7.6	806.3923	-0.0008	QLEDFR
7.4	806.3923	-0.0008	IAGFDER
3.4	806.3891	0.0024	IACCRK

Mascot: http://www.matrixscience.com/

Mascot Search Results

Peptide View

MS/MS Fragmentation of **LAADDFR**
Found in **ch17u_O76014|KRT37_HUMAN** in **uni_human**, Keratin, type I cuticular Ha7 OS=Homo sapiens GN=KRT37 PE=3 SV=3

Match to Query 557: 806.391628 from(404.203090,2+) intensity(2474562.7500) rtinseconds(1474) scans(7482) index(5443)
Title: 160219_Sunil_SDSII_A_Spectrum056766_scans__7482_RTINSECONDS=1474
Data file L:\\QE_2016\\160219_Sunil_KAP_LKC\\TMgf\\T\\T160219_Sunil_SDSII_A.mgf

Click mouse within plot area to zoom in by factor of two about that point
Or, Plot from 50 to 700 Da Full range
Label all possible matches ○ Label matches used for scoring ◉
Show Y-axis ☐

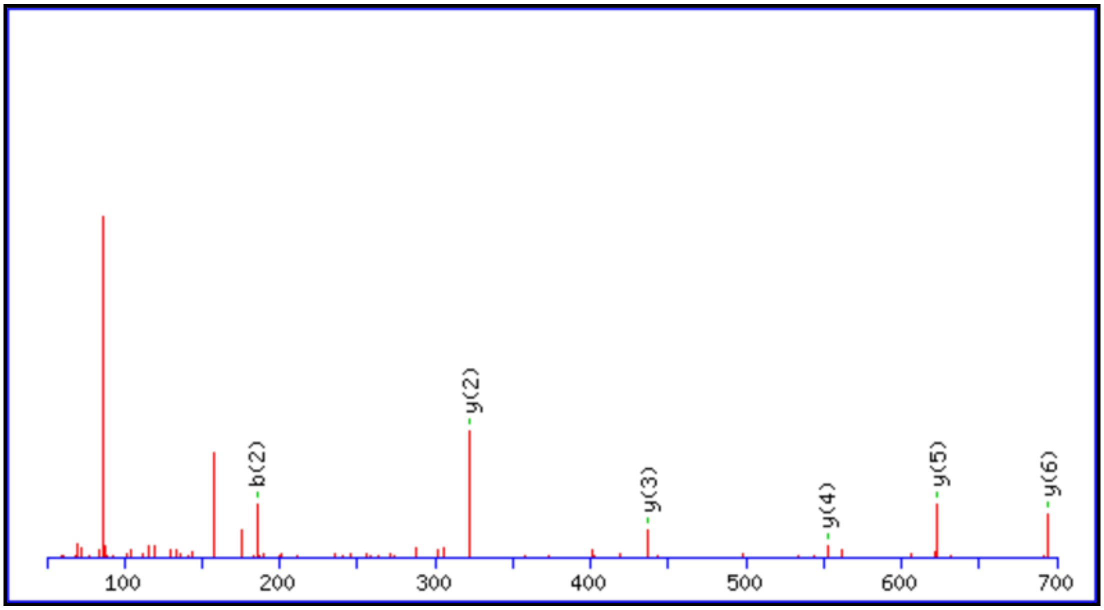

Monoisotopic mass of neutral peptide Mr(calc): 806.3923
Fixed modifications: Carbamidomethyl (C) (apply to specified residues or termini only)
Ions Score: 46 **Expect:** 0.0026
Matches : 6/50 fragment ions using 7 most intense peaks (help)

#	b	b++	b0	b0++	Seq.	y	y++	y*	y*++	y0	y0++	#
1	114.0913	57.5493			L							7
2	**185.1285**	93.0679			A	***694.3155***	347.6614	677.2889	339.1481	676.3049	338.6561	6
3	256.1656	128.5864			A	***623.2784***	312.1428	606.2518	303.6295	605.2678	303.1375	5
4	371.1925	186.0999	353.1819	177.0946	D	***552.2413***	276.6243	535.2147	268.1110	534.2307	267.6190	4
5	486.2195	243.6134	468.2089	234.6081	D	***437.2143***	219.1108	420.1878	210.5975	419.2037	210.1055	3
6	633.2879	317.1476	615.2773	308.1423	F	***322.1874***	161.5973	305.1608	153.0840			2

7					R	175.1190	88.0631	158.0924	79.5498			1

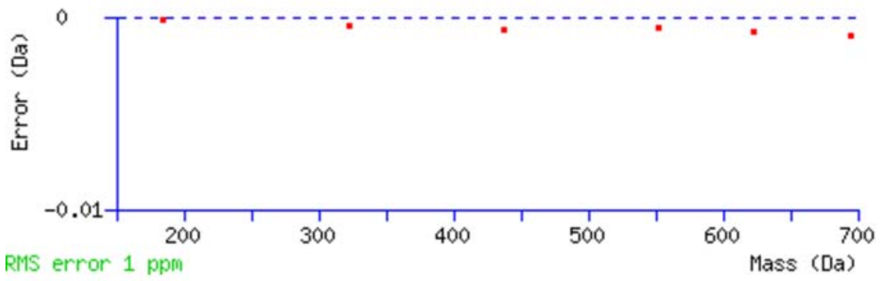

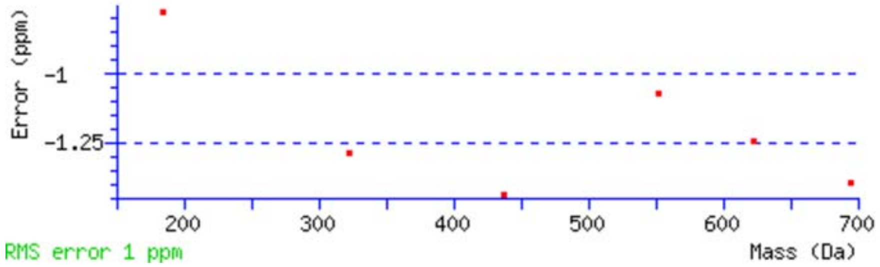

NCBI **BLAST** search of LAADDFR
(Parameters: blastp, nr protein database, expect=20000, no filter, PAM30)
Other BLAST web gateways

All matches to this query

Score	Mr(calc)	Delta	Sequence
46.4	806.3923	-0.0006	LAADDFR
15.2	804.3878	2.0038	RPDNFR
13.7	806.3844	0.0073	LAAQMQK
9.2	806.3923	-0.0006	IAGFDER
6.4	806.3923	-0.0006	LDQQFR
6.4	806.3923	-0.0006	LDQQFR
6.1	806.3922	-0.0006	GELANFR
6.1	804.3878	2.0038	NRPDFR
6.1	806.3923	-0.0006	QLEDFR
3.2	806.3891	0.0025	IACCRK

Mascot: http://www.matrixscience.com/

Mascot Search Results

Peptide View

MS/MS Fragmentation of **LAADDFR**
Found in **ch17u_O76014|KRT37_HUMAN** in **uni_human**, Keratin, type I cuticular Ha7 OS=Homo sapiens GN=KRT37 PE=3 SV=3

Match to Query 558: 806.391708 from(404.203130,2+) intensity(304244.7500) rtinseconds(1626) scans(8667) index(21650)
Title: 160219_Sunil_SDSII_A_Spectrum074211_scans__8667_RTINSECONDS=1626
Data file L:\\QE_2016\\160219_Sunil_KAP_LKC\\TMgf\\T\\T160219_Sunil_SDSII_A.mgf

Click mouse within plot area to zoom in by factor of two about that point
Or, Plot from 50 to 700 Da Full range
Label all possible matches ○ Label matches used for scoring ◉
Show Y-axis ☐

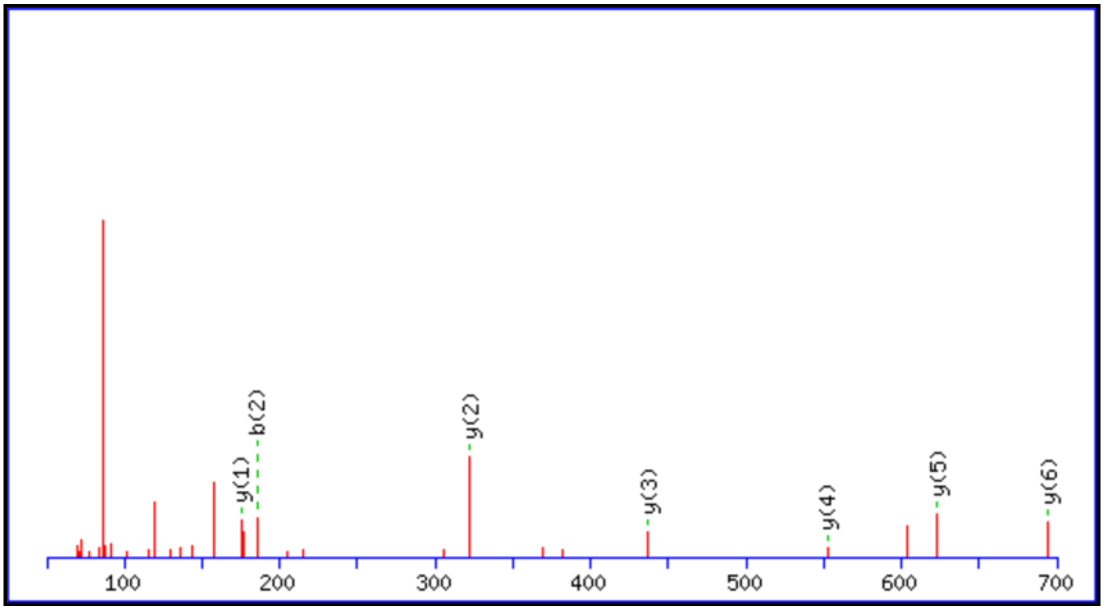

Monoisotopic mass of neutral peptide Mr(calc): 806.3923
Fixed modifications: Carbamidomethyl (C) (apply to specified residues or termini only)
Ions Score: 46 **Expect:** 0.0026
Matches : 7/50 fragment ions using 12 most intense peaks (help)

#	b	b++	b0	b0++	Seq.	y	y++	y*	y*++	y0	y0++	#
1	114.0913	57.5493			L							7
2	**185.1285**	93.0679			A	***694.3155***	347.6614	677.2889	339.1481	676.3049	338.6561	6
3	256.1656	128.5864			A	***623.2784***	312.1428	606.2518	303.6295	605.2678	303.1375	5
4	371.1925	186.0999	353.1819	177.0946	D	***552.2413***	276.6243	535.2147	268.1110	534.2307	267.6190	4
5	486.2195	243.6134	468.2089	234.6081	D	***437.2143***	219.1108	420.1878	210.5975	419.2037	210.1055	3
6	633.2879	317.1476	615.2773	308.1423	F	***322.1874***	161.5973	305.1608	153.0840			2

7					R	*175.1190*	88.0631	158.0924	79.5498			1

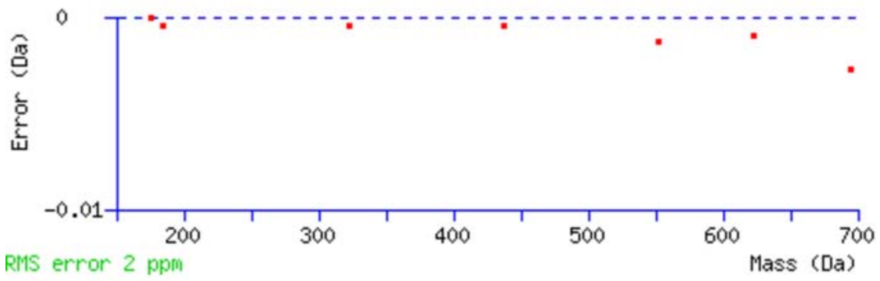

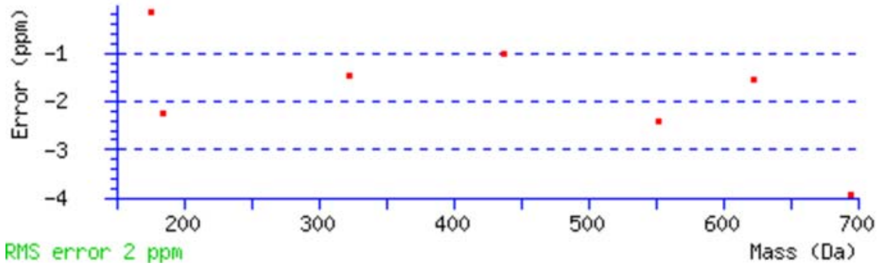

NCBI **BLAST** search of LAADDFR
(Parameters: blastp, nr protein database, expect=20000, no filter, PAM30)
Other BLAST web gateways

All matches to this query

Score	Mr(calc)	Delta	Sequence
46.4	806.3923	-0.0006	LAADDFR
14.8	804.3878	2.0039	RPDNFR
12.9	806.3844	0.0073	LAAQMQK
7.9	806.3922	-0.0005	GELANFR
6.9	806.3923	-0.0006	IAGFDER
5.7	806.3923	-0.0006	LDQQFR
5.7	806.3923	-0.0006	LDQQFR
5.4	804.3878	2.0039	NRPDFR
5.4	806.3923	-0.0006	QLEDFR
2.4	806.3891	0.0026	IACCRK

Mascot: http://www.matrixscience.com/

Mascot Search Results

Peptide View

MS/MS Fragmentation of **LAADDFR**
Found in **ch17u_O76014|KRT37_HUMAN** in **uni_human**, Keratin, type I cuticular Ha7 OS=Homo sapiens GN=KRT37 PE=3 SV=3

Match to Query 560: 806.391708 from(404.203130,2+) intensity(39378792.0000) rtinseconds(1164) scans(6004) index(19300)
Title: 160219_Sunil_SDSII_A_Spectrum071861_scans__6004_RTINSECONDS=1164
Data file L:\\QE_2016\\160219_Sunil_KAP_LKC\\TMgf\\T\\T160219_Sunil_SDSII_A.mgf

Click mouse within plot area to zoom in by factor of two about that point
Or, Plot from 50 to 700 Da Full range
Label all possible matches ○ Label matches used for scoring ◉
Show Y-axis ☐

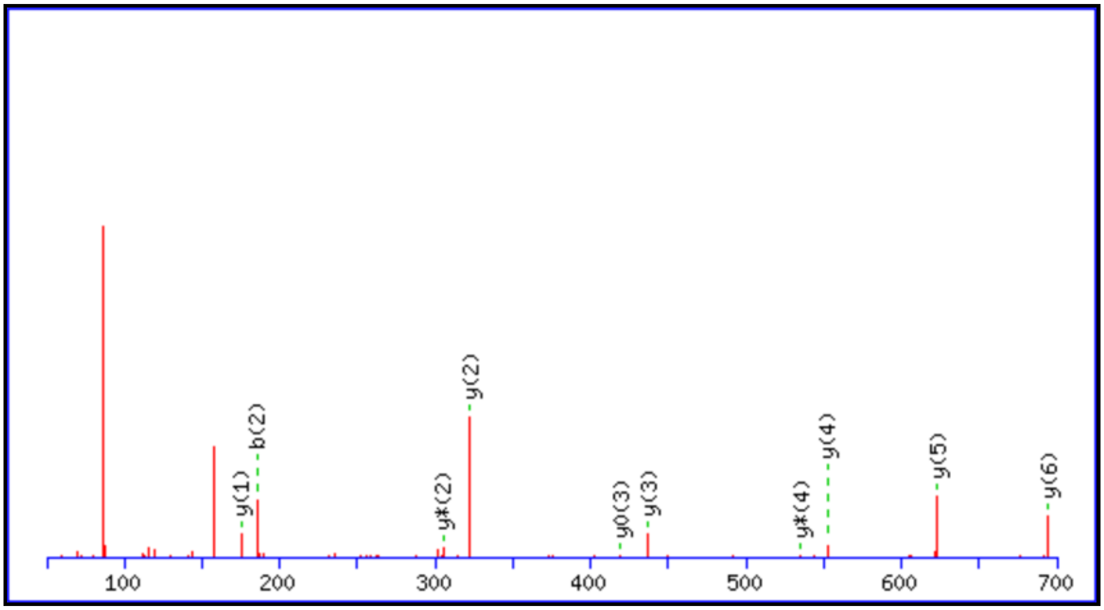

Monoisotopic mass of neutral peptide Mr(calc): 806.3923
Fixed modifications: Carbamidomethyl (C) (apply to specified residues or termini only)
Ions Score: 52 **Expect:** 0.00076
Matches : 10/50 fragment ions using 14 most intense peaks (help)

#	b	b^{++}	b^{0}	b^{0++}	Seq.	y	y^{++}	y*	y^{*++}	y^{0}	y^{0++}	#
1	114.0913	57.5493			L							7
2	**185.1285**	93.0679			A	***694.3155***	347.6614	677.2889	339.1481	676.3049	338.6561	6
3	256.1656	128.5864			A	***623.2784***	312.1428	606.2518	303.6295	605.2678	303.1375	5
4	371.1925	186.0999	353.1819	177.0946	D	***552.2413***	276.6243	535.2147	268.1110	534.2307	267.6190	4
5	486.2195	243.6134	468.2089	234.6081	D	***437.2143***	219.1108	420.1878	210.5975	419.2037	210.1055	3
6	633.2879	317.1476	615.2773	308.1423	F	***322.1874***	161.5973	305.1608	153.0840			2

7					R	*175.1190*	88.0631	158.0924	79.5498			1

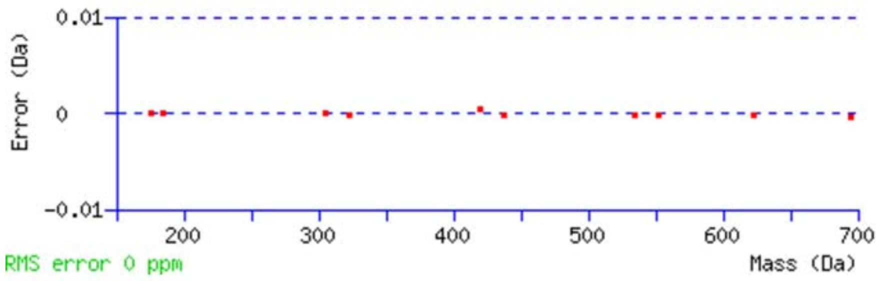

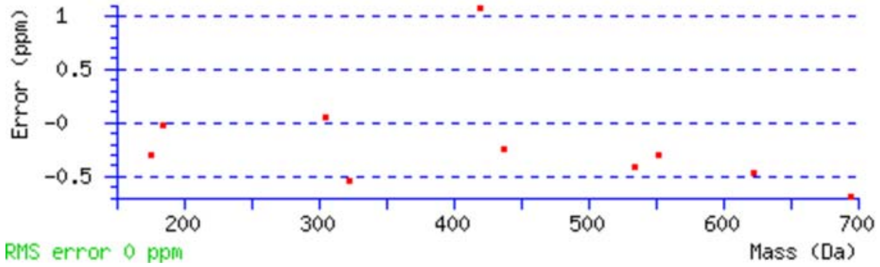

NCBI **BLAST** search of LAADDFR
(Parameters: blastp, nr protein database, expect=20000, no filter, PAM30)
Other BLAST web gateways

All matches to this query

Score	Mr(calc)	Delta	Sequence
51.7	806.3923	-0.0006	LAADDFR
17.6	804.3878	2.0039	RPDNFR
16.6	806.3923	-0.0006	IAGFDER
14.2	806.3844	0.0073	LAAQMQK
7.9	804.3878	2.0039	NRPDFR
7.9	806.3923	-0.0006	QLEDFR
6.8	806.3923	-0.0006	LDQQFR
6.8	806.3923	-0.0006	LDQQFR
6.4	806.3922	-0.0005	GELANFR
3.8	806.3891	0.0026	IACCRK

Mascot: http://www.matrixscience.com/

Mascot Search Results

Peptide View

MS/MS Fragmentation of **LAADDFR**
Found in **ch17u_O76014|KRT37_HUMAN** in **uni_human**, Keratin, type I cuticular Ha7 OS=Homo sapiens GN=KRT37 PE=3 SV=3

Match to Query 564: 806.391888 from(404.203220,2+) intensity(747366.8125) rtinseconds(1423) scans(7499) index(20612)
Title: 160219_Sunil_SDSII_A_Spectrum073173_scans__7499_RTINSECONDS=1423
Data file L:\\QE_2016\\160219_Sunil_KAP_LKC\\TMgf\\T\\T160219_Sunil_SDSII_A.mgf

Click mouse within plot area to zoom in by factor of two about that point
Or, Plot from 50 to 700 Da Full range
Label all possible matches ○ Label matches used for scoring ◉
Show Y-axis ☐

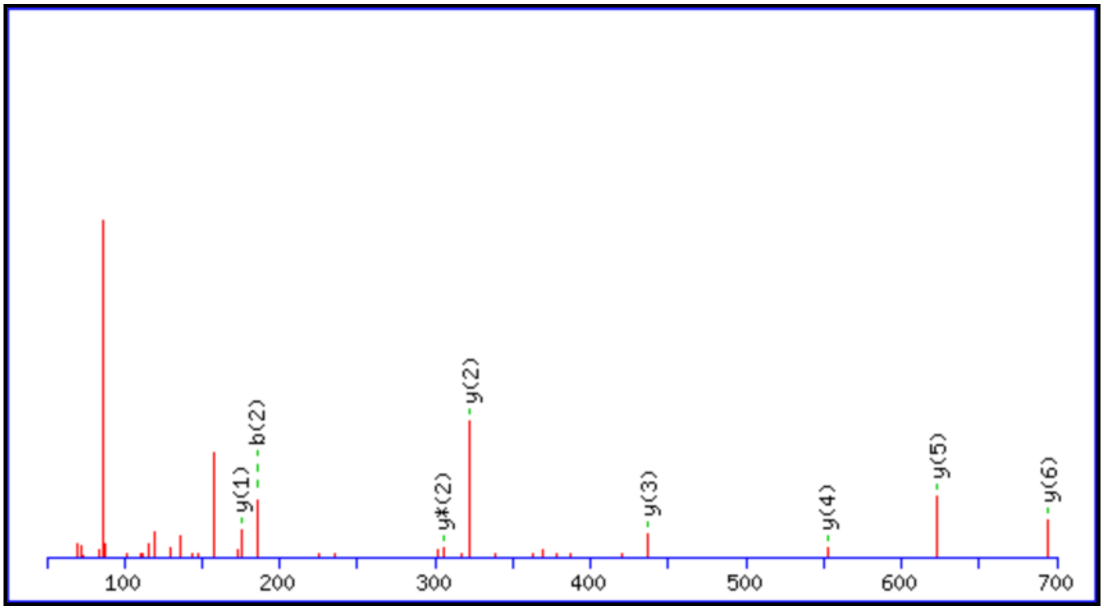

Monoisotopic mass of neutral peptide Mr(calc): 806.3923
Fixed modifications: Carbamidomethyl (C) (apply to specified residues or termini only)
Ions Score: 53 **Expect:** 0.00051
Matches : 8/50 fragment ions using 11 most intense peaks (help)

#	b	b^{++}	b^0	b^{0++}	Seq.	y	y^{++}	y*	y^{*++}	y^0	y^{0++}	#
1	114.0913	57.5493			L							7
2	**185.1285**	93.0679			A	***694.3155***	347.6614	677.2889	339.1481	676.3049	338.6561	6
3	256.1656	128.5864			A	***623.2784***	312.1428	606.2518	303.6295	605.2678	303.1375	5
4	371.1925	186.0999	353.1819	177.0946	D	***552.2413***	276.6243	535.2147	268.1110	534.2307	267.6190	4
5	486.2195	243.6134	468.2089	234.6081	D	***437.2143***	219.1108	420.1878	210.5975	419.2037	210.1055	3
6	633.2879	317.1476	615.2773	308.1423	F	***322.1874***	161.5973	305.1608	153.0840			2

7					R	*175.1190*	88.0631	158.0924	79.5498			1

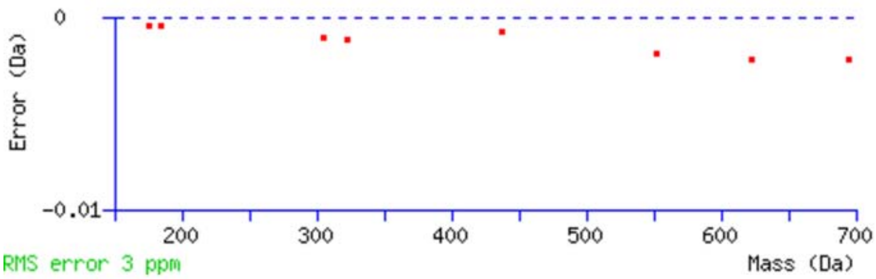

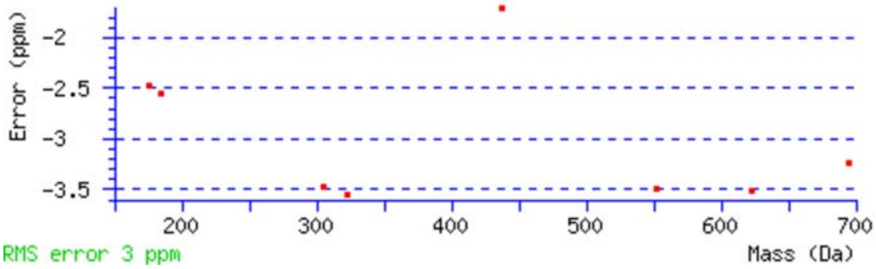

NCBI **BLAST** search of LAADDFR
(Parameters: blastp, nr protein database, expect=20000, no filter, PAM30)
Other BLAST web gateways

All matches to this query

Score	Mr(calc)	Delta	Sequence
53.5	806.3923	-0.0004	LAADDFR
19.3	804.3878	2.0041	RPDNFR
13.8	806.3844	0.0075	LAAQMQK
9.1	806.3923	-0.0004	LDQQFR
9.1	806.3923	-0.0004	LDQQFR
8.8	806.3922	-0.0004	GELANFR
8.8	804.3878	2.0041	NRPDFR
8.8	806.3923	-0.0004	QLEDFR
8.5	806.3923	-0.0004	IAGFDER
3.4	806.3891	0.0028	IACCRK

Mascot: http://www.matrixscience.com/

Mascot Search Results

Peptide View

MS/MS Fragmentation of **LAADDFR**
Found in **ch17u_O76014|KRT37_HUMAN** in **uni_human**, Keratin, type I cuticular Ha7 OS=Homo sapiens GN=KRT37 PE=3 SV=3

Match to Query 565: 806.391948 from(404.203250,2+) intensity(832624.5625) rtinseconds(1388) scans(7294) index(20425)
Title: 160219_Sunil_SDSII_A_Spectrum072986_scans__7294_RTINSECONDS=1388
Data file L:\\QE_2016\\160219_Sunil_KAP_LKC\\TMgf\\T\\T160219_Sunil_SDSII_A.mgf

Click mouse within plot area to zoom in by factor of two about that point
Or, Plot from 50 to 700 Da Full range
Label all possible matches ○ Label matches used for scoring ◉
Show Y-axis ☐

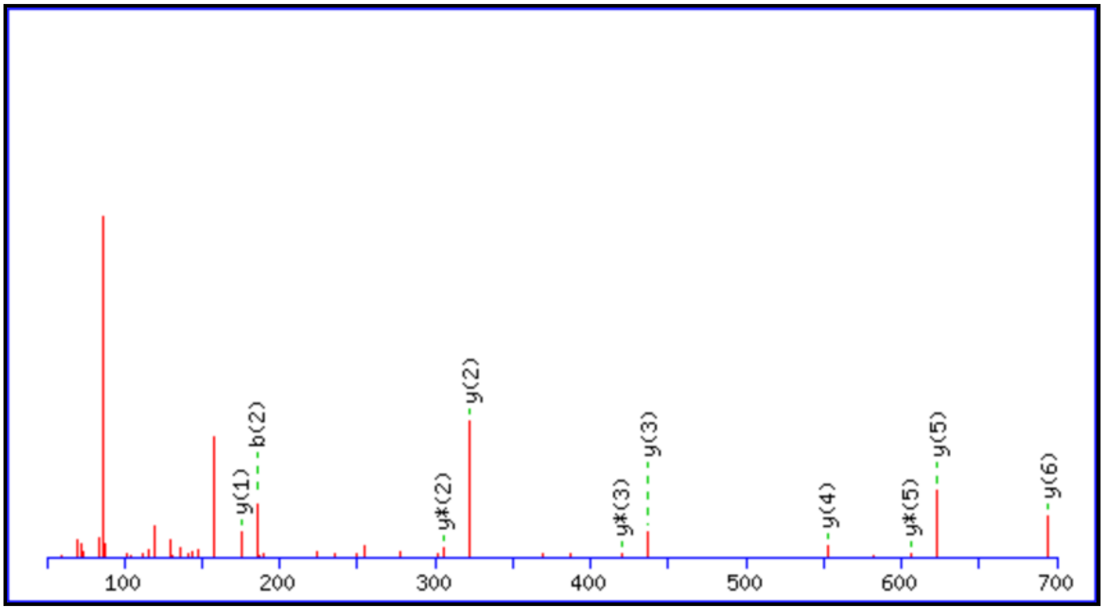

Monoisotopic mass of neutral peptide Mr(calc): 806.3923
Fixed modifications: Carbamidomethyl (C) (apply to specified residues or termini only)
Ions Score: 57 **Expect:** 0.00021
Matches : 10/50 fragment ions using 12 most intense peaks (help)

#	b	b^{++}	b^{0}	b^{0++}	Seq.	y	y^{++}	y*	y^{*++}	y^{0}	y^{0++}	#
1	114.0913	57.5493			L							7
2	**185.1285**	93.0679			A	***694.3155***	347.6614	677.2889	339.1481	676.3049	338.6561	6
3	256.1656	128.5864			A	***623.2784***	312.1428	606.2518	303.6295	605.2678	303.1375	5
4	371.1925	186.0999	353.1819	177.0946	D	***552.2413***	276.6243	535.2147	268.1110	534.2307	267.6190	4
5	486.2195	243.6134	468.2089	234.6081	D	***437.2143***	219.1108	420.1878	210.5975	419.2037	210.1055	3
6	633.2879	317.1476	615.2773	308.1423	F	***322.1874***	161.5973	305.1608	153.0840			2

7					R	*175.1190*	88.0631	158.0924	79.5498			1

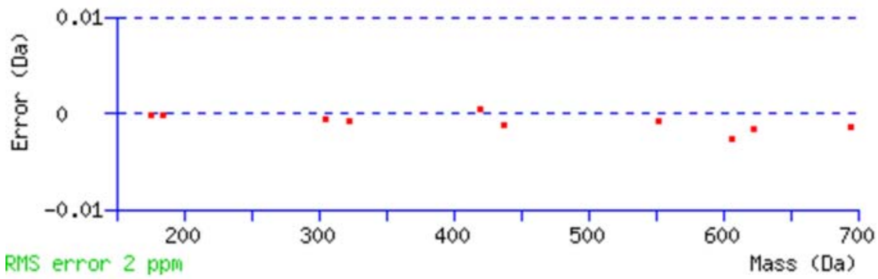

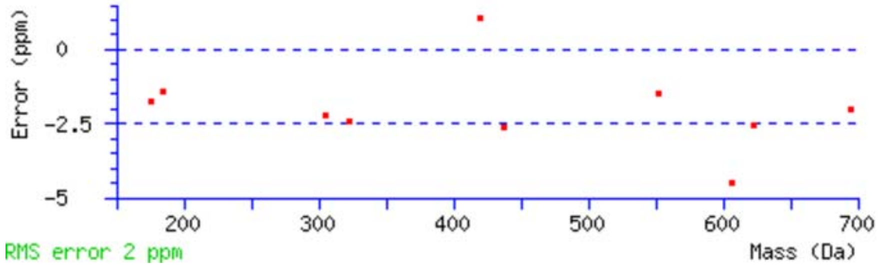

NCBI **BLAST** search of LAADDFR
(Parameters: blastp, nr protein database, expect=20000, no filter, PAM30)
Other BLAST web gateways

All matches to this query

Score	Mr(calc)	Delta	Sequence
57.4	806.3923	-0.0003	LAADDFR
19.9	804.3878	2.0041	RPDNFR
13.8	806.3844	0.0076	LAAQMQK
9.3	806.3922	-0.0003	GELANFR
9.3	804.3878	2.0041	NRPDFR
9.3	806.3923	-0.0003	QLEDFR
9.1	806.3923	-0.0003	IAGFDER
7.9	806.3923	-0.0003	LDQQFR
7.9	806.3923	-0.0003	LDQQFR
3.3	806.3891	0.0028	IACCRK

Mascot: http://www.matrixscience.com/

Mascot Search Results

Peptide View

MS/MS Fragmentation of **LAADDFR**
Found in **ch17u_O76014|KRT37_HUMAN** in **uni_human**, Keratin, type I cuticular Ha7 OS=Homo sapiens GN=KRT37 PE=3 SV=3

Match to Query 566: 806.391948 from(404.203250,2+) intensity(3050788.2500) rtinseconds(1262) scans(6569) index(19791)
Title: 160219_Sunil_SDSII_A_Spectrum072352_scans__6569_RTINSECONDS=1262
Data file L:\\QE_2016\\160219_Sunil_KAP_LKC\\TMgf\\T\\T160219_Sunil_SDSII_A.mgf

Click mouse within plot area to zoom in by factor of two about that point
Or, Plot from 50 to 700 Da Full range
Label all possible matches ○ Label matches used for scoring ◉
Show Y-axis ☐

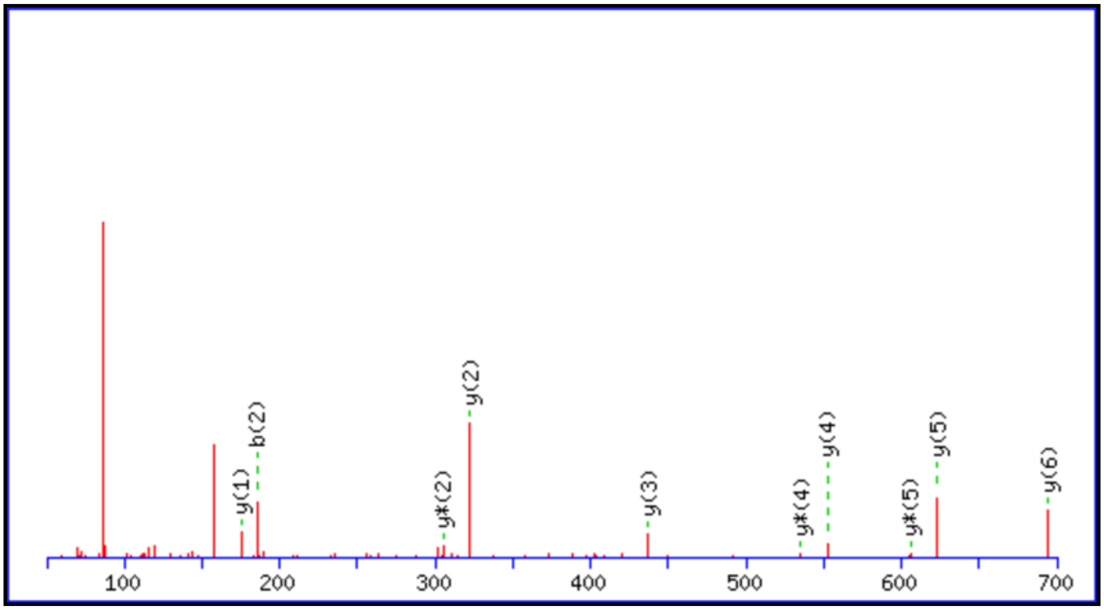

Monoisotopic mass of neutral peptide Mr(calc): 806.3923
Fixed modifications: Carbamidomethyl (C) (apply to specified residues or termini only)
Ions Score: 54 **Expect:** 0.00041
Matches : 10/50 fragment ions using 13 most intense peaks (help)

#	b	b^{++}	b^{0}	b^{0++}	Seq.	y	y^{++}	y*	y^{*++}	y^{0}	y^{0++}	#
1	114.0913	57.5493			L							7
2	**185.1285**	93.0679			A	***694.3155***	347.6614	677.2889	339.1481	676.3049	338.6561	6
3	256.1656	128.5864			A	***623.2784***	312.1428	606.2518	303.6295	605.2678	303.1375	5
4	371.1925	186.0999	353.1819	177.0946	D	***552.2413***	276.6243	535.2147	268.1110	534.2307	267.6190	4
5	486.2195	243.6134	468.2089	234.6081	D	***437.2143***	219.1108	420.1878	210.5975	419.2037	210.1055	3
6	633.2879	317.1476	615.2773	308.1423	F	***322.1874***	161.5973	305.1608	153.0840			2

7					R	*175.1190*	88.0631	158.0924	79.5498			1

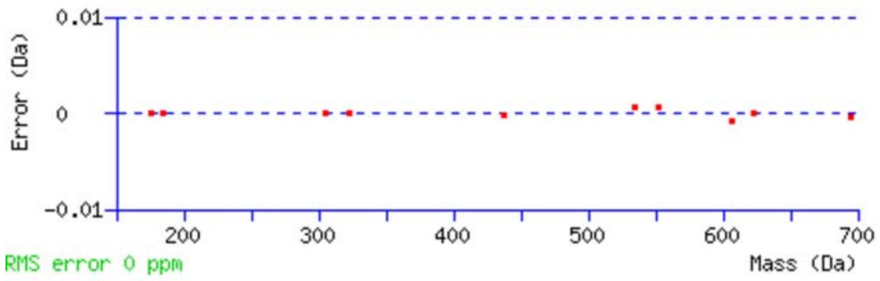

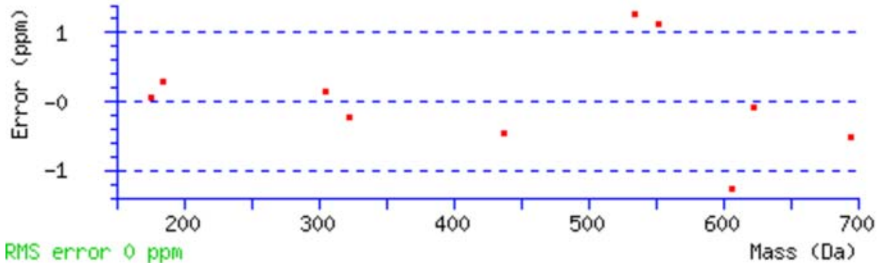

NCBI **BLAST** search of LAADDFR
(Parameters: blastp, nr protein database, expect=20000, no filter, PAM30)
Other BLAST web gateways

All matches to this query

Score	Mr(calc)	Delta	Sequence
54.4	806.3923	-0.0003	LAADDFR
18.8	804.3878	2.0041	RPDNFR
14.2	806.3844	0.0076	LAAQMQK
8.4	806.3923	-0.0003	IAGFDER
7.5	806.3923	-0.0003	LDQQFR
7.5	806.3923	-0.0003	LDQQFR
7.0	806.3922	-0.0003	GELANFR
7.0	804.3878	2.0041	NRPDFR
7.0	806.3923	-0.0003	QLEDFR
3.7	806.3891	0.0028	IACCRK

Mascot: http://www.matrixscience.com/

Mascot Search Results

Peptide View

MS/MS Fragmentation of **LAADDFR**
Found in **ch17u_O76014|KRT37_HUMAN** in **uni_human**, Keratin, type I cuticular Ha7 OS=Homo sapiens GN=KRT37 PE=3 SV=3

Match to Query 566: 806.391948 from(404.203250,2+) intensity(3050788.2500) rtinseconds(1262) scans(6569) index(19791)
Title: 160219_Sunil_SDSII_A_Spectrum072352_scans__6569_RTINSECONDS=1262
Data file L:\\QE_2016\\160219_Sunil_KAP_LKC\\TMgf\\T\\T160219_Sunil_SDSII_A.mgf

Click mouse within plot area to zoom in by factor of two about that point
Or, Plot from 50 to 700 Da Full range
Label all possible matches ○ Label matches used for scoring ◉
Show Y-axis ☐

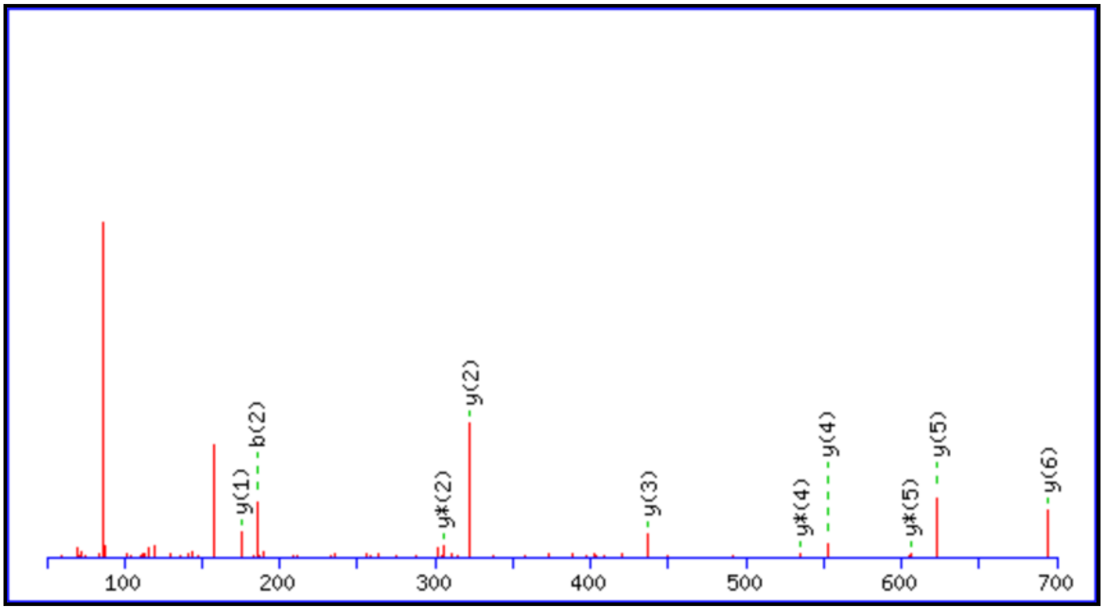

Monoisotopic mass of neutral peptide Mr(calc): 806.3923
Fixed modifications: Carbamidomethyl (C) (apply to specified residues or termini only)
Ions Score: 54 **Expect:** 0.00041
Matches : 10/50 fragment ions using 13 most intense peaks (help)

#	b	b^{++}	b^{0}	b^{0++}	Seq.	y	y^{++}	y*	y^{*++}	y^{0}	y^{0++}	#
1	114.0913	57.5493			L							7
2	**185.1285**	93.0679			A	***694.3155***	347.6614	677.2889	339.1481	676.3049	338.6561	6
3	256.1656	128.5864			A	***623.2784***	312.1428	606.2518	303.6295	605.2678	303.1375	5
4	371.1925	186.0999	353.1819	177.0946	D	***552.2413***	276.6243	535.2147	268.1110	534.2307	267.6190	4
5	486.2195	243.6134	468.2089	234.6081	D	***437.2143***	219.1108	420.1878	210.5975	419.2037	210.1055	3
6	633.2879	317.1476	615.2773	308.1423	F	***322.1874***	161.5973	305.1608	153.0840			2

7					R	175.1190	88.0631	158.0924	79.5498			1

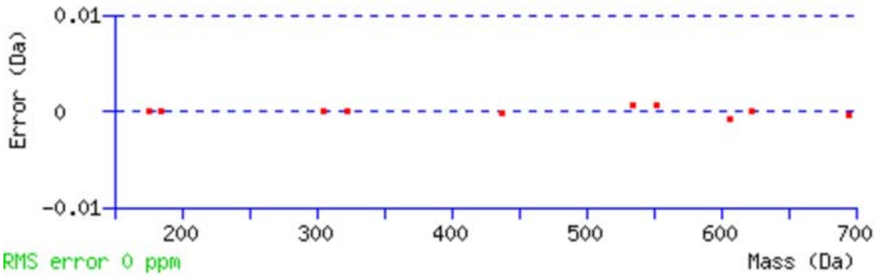

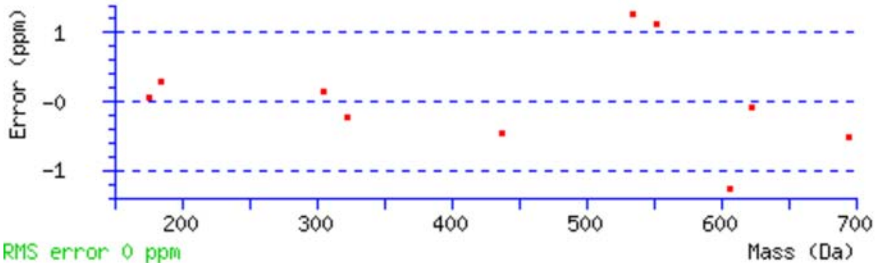

NCBI **BLAST** search of LAADDFR
(Parameters: blastp, nr protein database, expect=20000, no filter, PAM30)
Other BLAST web gateways

All matches to this query

Score	Mr(calc)	Delta	Sequence
54.4	806.3923	-0.0003	LAADDFR
18.8	804.3878	2.0041	RPDNFR
14.2	806.3844	0.0076	LAAQMQK
8.4	806.3923	-0.0003	IAGFDER
7.5	806.3923	-0.0003	LDQQFR
7.5	806.3923	-0.0003	LDQQFR
7.0	806.3922	-0.0003	GELANFR
7.0	804.3878	2.0041	NRPDFR
7.0	806.3923	-0.0003	QLEDFR
3.7	806.3891	0.0028	IACCRK

Mascot: http://www.matrixscience.com/

Mascot Search Results

Peptide View

MS/MS Fragmentation of **LAADDFR**
Found in **ch17u_O76014|KRT37_HUMAN** in **uni_human**, Keratin, type I cuticular Ha7 OS=Homo sapiens GN=KRT37 PE=3 SV=3

Match to Query 568: 806.392068 from(404.203310,2+) intensity(557655.3125) rtinseconds(1612) scans(8288) index(6157)
Title: 160219_Sunil_SDSII_A_Spectrum057480_scans__8288_RTINSECONDS=1612
Data file L:\\QE_2016\\160219_Sunil_KAP_LKC\\TMgf\\T\\T160219_Sunil_SDSII_A.mgf

Click mouse within plot area to zoom in by factor of two about that point
Or, Plot from 50 to 700 Da Full range
Label all possible matches ○ Label matches used for scoring ◉
Show Y-axis ☐

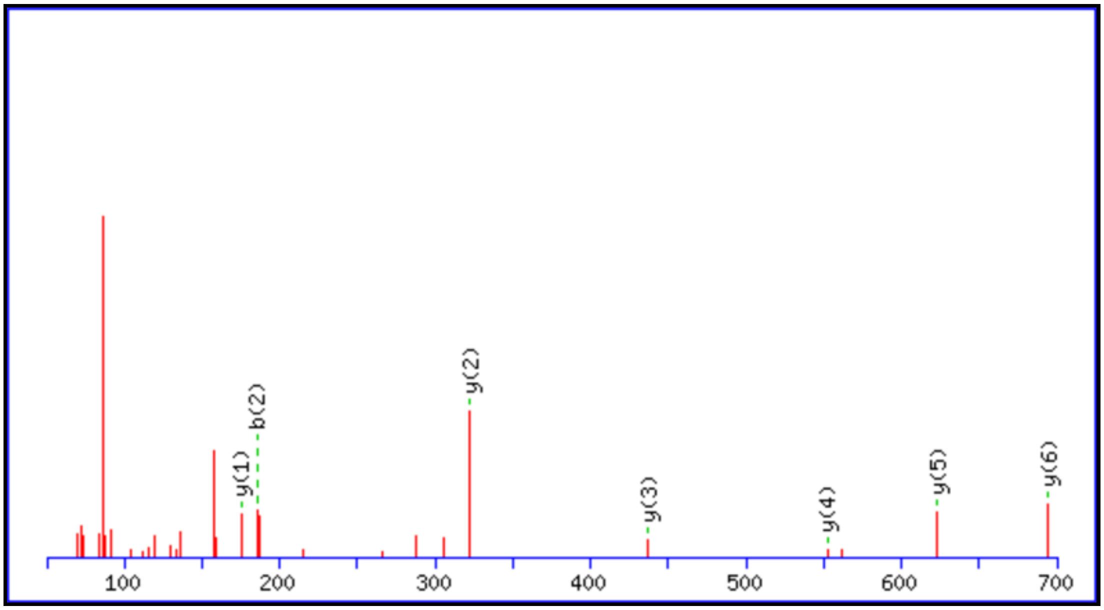

Monoisotopic mass of neutral peptide Mr(calc): 806.3923
Fixed modifications: Carbamidomethyl (C) (apply to specified residues or termini only)
Ions Score: 49 **Expect:** 0.0013
Matches : 7/50 fragment ions using 11 most intense peaks (help)

#	b	b^{++}	b^{0}	b^{0++}	Seq.	y	y^{++}	y*	y^{*++}	y^{0}	y^{0++}	#
1	114.0913	57.5493			L							7
2	**185.1285**	93.0679			A	***694.3155***	347.6614	677.2889	339.1481	676.3049	338.6561	6
3	256.1656	128.5864			A	***623.2784***	312.1428	606.2518	303.6295	605.2678	303.1375	5
4	371.1925	186.0999	353.1819	177.0946	D	***552.2413***	276.6243	535.2147	268.1110	534.2307	267.6190	4
5	486.2195	243.6134	468.2089	234.6081	D	***437.2143***	219.1108	420.1878	210.5975	419.2037	210.1055	3
6	633.2879	317.1476	615.2773	308.1423	F	***322.1874***	161.5973	305.1608	153.0840			2

7					R	175.1190	88.0631	158.0924	79.5498			1

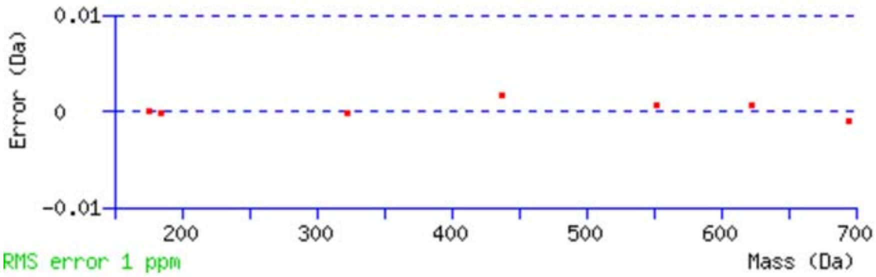

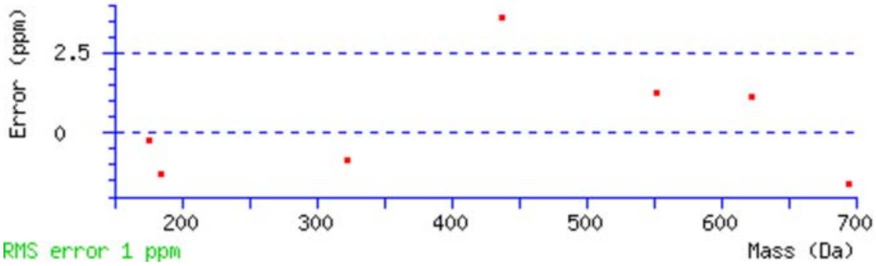

NCBI **BLAST** search of LAADDFR
(Parameters: blastp, nr protein database, expect=20000, no filter, PAM30)
Other BLAST web gateways

All matches to this query

Score	Mr(calc)	Delta	Sequence
49.3	806.3923	-0.0002	LAADDFR
16.4	804.3878	2.0042	RPDNFR
12.9	806.3844	0.0077	LAAQMQK
8.0	806.3923	-0.0002	IAGFDER
7.3	806.3923	-0.0002	LDQQFR
7.3	806.3923	-0.0002	LDQQFR
6.6	806.3922	-0.0002	GELANFR
6.6	804.3878	2.0042	NRPDFR
6.6	806.3923	-0.0002	QLEDFR
4.1	806.3956	-0.0036	LGDMSLR

Mascot: http://www.matrixscience.com/

Mascot Search Results

Peptide View

MS/MS Fragmentation of **LAADDFR**
Found in **ch17u_O76014|KRT37_HUMAN** in **uni_human**, Keratin, type I cuticular Ha7 OS=Homo sapiens GN=KRT37 PE=3 SV=3

Match to Query 572: 806.392188 from(404.203370,2+) intensity(7881964.5000) rtinseconds(1442) scans(7297) index(5282)
Title: 160219_Sunil_SDSII_A_Spectrum056605_scans__7297_RTINSECONDS=1442
Data file L:\\QE_2016\\160219_Sunil_KAP_LKC\\TMgf\\T\\T160219_Sunil_SDSII_A.mgf

Click mouse within plot area to zoom in by factor of two about that point
Or, Plot from 50 to 800 Da Full range
Label all possible matches ○ Label matches used for scoring ◉
Show Y-axis ☐

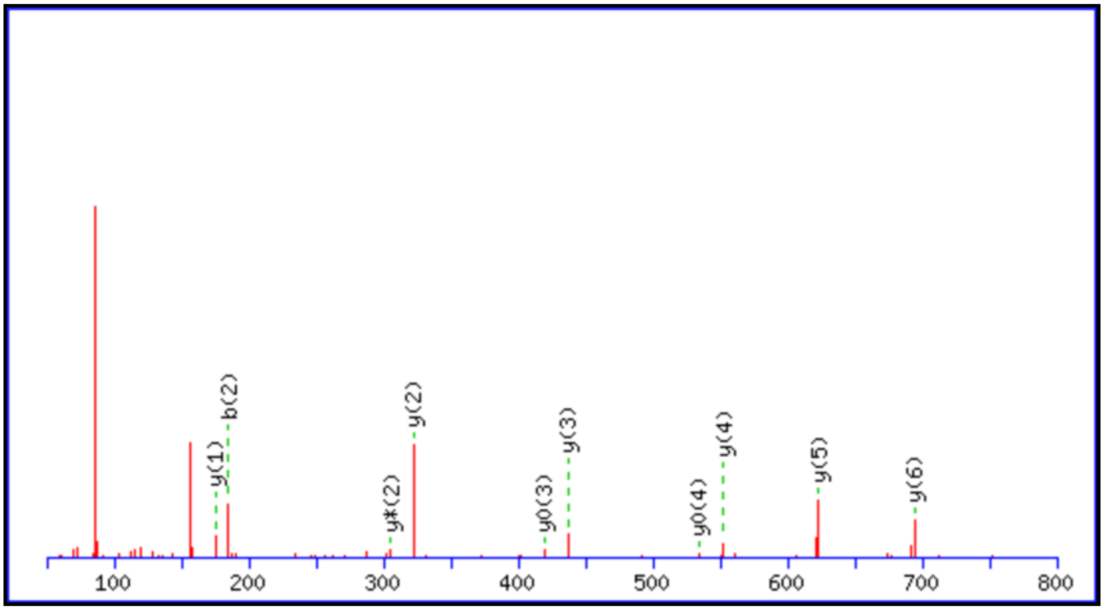

Monoisotopic mass of neutral peptide Mr(calc): 806.3923
Fixed modifications: Carbamidomethyl (C) (apply to specified residues or termini only)
Ions Score: 51 **Expect:** 0.00086
Matches : 10/50 fragment ions using 14 most intense peaks (help)

#	b	b^{++}	b^{0}	b^{0++}	Seq.	y	y^{++}	y*	y^{*++}	y^{0}	y^{0++}	#
1	114.0913	57.5493			L							7
2	**185.1285**	93.0679			A	***694.3155***	347.6614	677.2889	339.1481	676.3049	338.6561	6
3	256.1656	128.5864			A	***623.2784***	312.1428	606.2518	303.6295	605.2678	303.1375	5
4	371.1925	186.0999	353.1819	177.0946	D	***552.2413***	276.6243	535.2147	268.1110	534.2307	267.6190	4
5	486.2195	243.6134	468.2089	234.6081	D	***437.2143***	219.1108	420.1878	210.5975	419.2037	210.1055	3
6	633.2879	317.1476	615.2773	308.1423	F	***322.1874***	161.5973	305.1608	153.0840			2

7					R	*175.1190*	88.0631	158.0924	79.5498			1

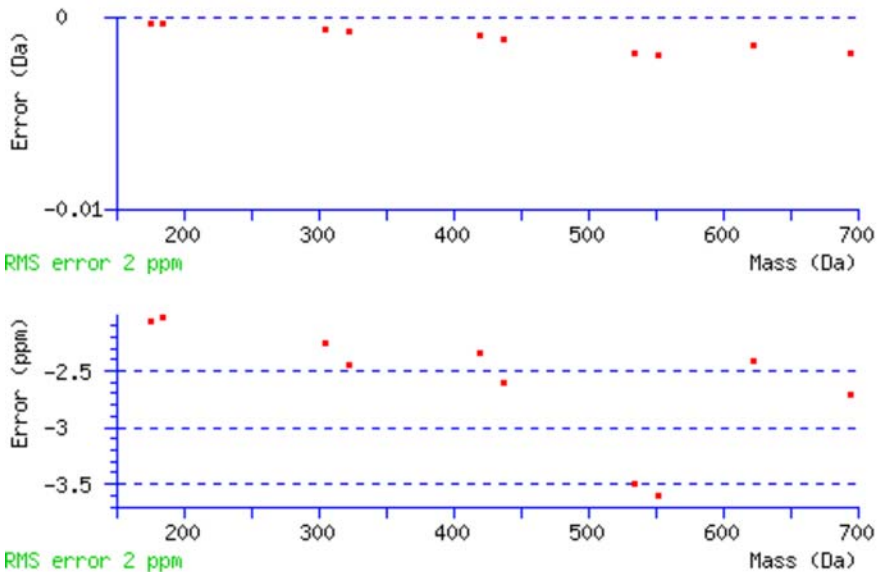

NCBI **BLAST** search of LAADDFR
(Parameters: blastp, nr protein database, expect=20000, no filter, PAM30)
Other BLAST web gateways

All matches to this query

Score	Mr(calc)	Delta	Sequence
51.1	806.3923	-0.0001	LAADDFR
16.8	804.3878	2.0044	RPDNFR
14.7	806.3923	-0.0001	IAGFDER
13.8	806.3844	0.0078	LAAQMQK
12.6	804.3912	2.0010	LASQACR
7.2	804.3878	2.0044	NRPDFR
7.2	806.3923	-0.0001	QLEDFR
5.9	806.3923	-0.0001	LDQQFR
5.9	806.3923	-0.0001	LDQQFR
5.5	806.3922	-0.0001	GELANFR

Mascot: http://www.matrixscience.com/

Mascot Search Results

Peptide View

MS/MS Fragmentation of **LAADDFR**
Found in **ch17u_O76014|KRT37_HUMAN** in **uni_human**, Keratin, type I cuticular Ha7 OS=Homo sapiens GN=KRT37 PE=3 SV=3

Match to Query 572: 806.392188 from(404.203370,2+) intensity(7881964.5000) rtinseconds(1442) scans(7297) index(5282)
Title: 160219_Sunil_SDSII_A_Spectrum056605_scans__7297_RTINSECONDS=1442
Data file L:\\QE_2016\\160219_Sunil_KAP_LKC\\TMgf\\T\\T160219_Sunil_SDSII_A.mgf

Click mouse within plot area to zoom in by factor of two about that point
Or, Plot from 50 to 800 Da Full range
Label all possible matches ○ Label matches used for scoring ◉
Show Y-axis ☐

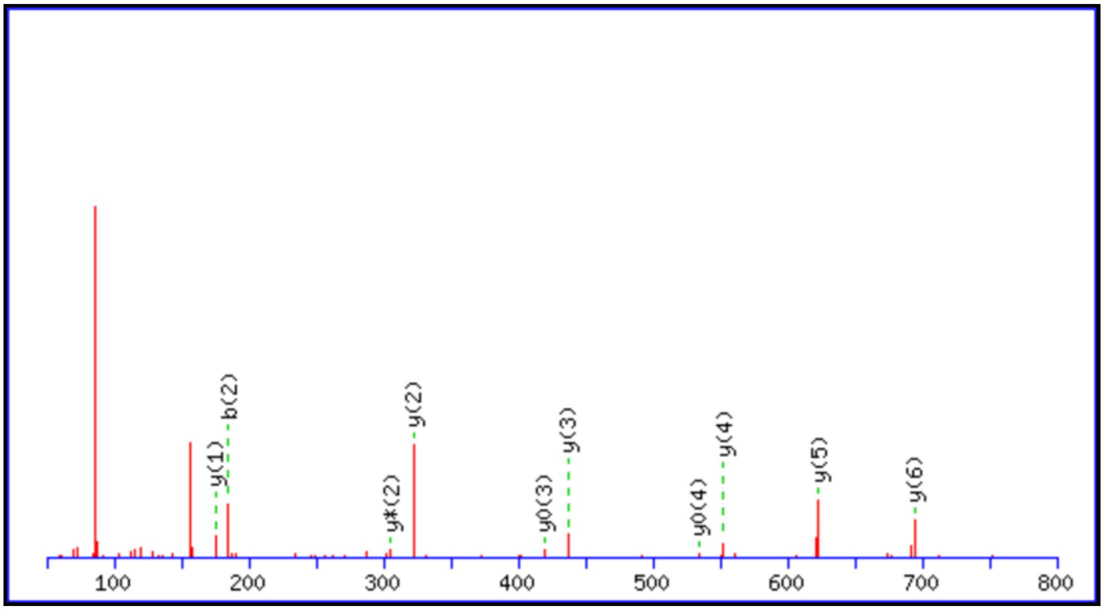

Monoisotopic mass of neutral peptide Mr(calc): 806.3923
Fixed modifications: Carbamidomethyl (C) (apply to specified residues or termini only)
Ions Score: 51 **Expect:** 0.00086
Matches : 10/50 fragment ions using 14 most intense peaks (help)

#	b	b^{++}	b^0	b^{0++}	Seq.	y	y^{++}	y*	y^{*++}	y^0	y^{0++}	#
1	114.0913	57.5493			L							7
2	**185.1285**	93.0679			A	***694.3155***	347.6614	677.2889	339.1481	676.3049	338.6561	6
3	256.1656	128.5864			A	***623.2784***	312.1428	606.2518	303.6295	605.2678	303.1375	5
4	371.1925	186.0999	353.1819	177.0946	D	***552.2413***	276.6243	535.2147	268.1110	534.2307	267.6190	4
5	486.2195	243.6134	468.2089	234.6081	D	***437.2143***	219.1108	420.1878	210.5975	419.2037	210.1055	3
6	633.2879	317.1476	615.2773	308.1423	F	***322.1874***	161.5973	305.1608	153.0840			2

7					R	175.1190	88.0631	158.0924	79.5498			1

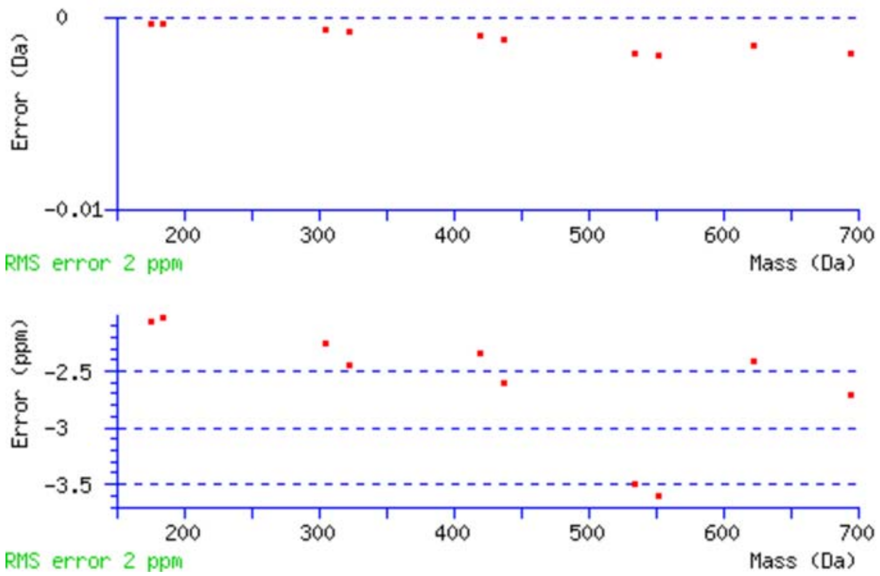

NCBI **BLAST** search of LAADDFR
(Parameters: blastp, nr protein database, expect=20000, no filter, PAM30)
Other BLAST web gateways

All matches to this query

Score	Mr(calc)	Delta	Sequence
51.1	806.3923	-0.0001	LAADDFR
16.8	804.3878	2.0044	RPDNFR
14.7	806.3923	-0.0001	IAGFDER
13.8	806.3844	0.0078	LAAQMQK
12.6	804.3912	2.0010	LASQACR
7.2	804.3878	2.0044	NRPDFR
7.2	806.3923	-0.0001	QLEDFR
5.9	806.3923	-0.0001	LDQQFR
5.9	806.3923	-0.0001	LDQQFR
5.5	806.3922	-0.0001	GELANFR

Mascot: http://www.matrixscience.com/

Mascot Search Results

Peptide View

MS/MS Fragmentation of **LAADDFR**
Found in **ch17u_O76014|KRT37_HUMAN** in **uni_human**, Keratin, type I cuticular Ha7 OS=Homo sapiens GN=KRT37 PE=3 SV=3

Match to Query 573: 806.392188 from(404.203370,2+) intensity(2115241.7500) rtinseconds(1505) scans(7663) index(5597)
Title: 160219_Sunil_SDSII_A_Spectrum056920_scans__7663_RTINSECONDS=1505
Data file L:\\QE_2016\\160219_Sunil_KAP_LKC\\TMgf\\T\\T160219_Sunil_SDSII_A.mgf

Click mouse within plot area to zoom in by factor of two about that point
Or, Plot from 50 to 750 Da Full range
Label all possible matches ○ Label matches used for scoring ◉
Show Y-axis ☐

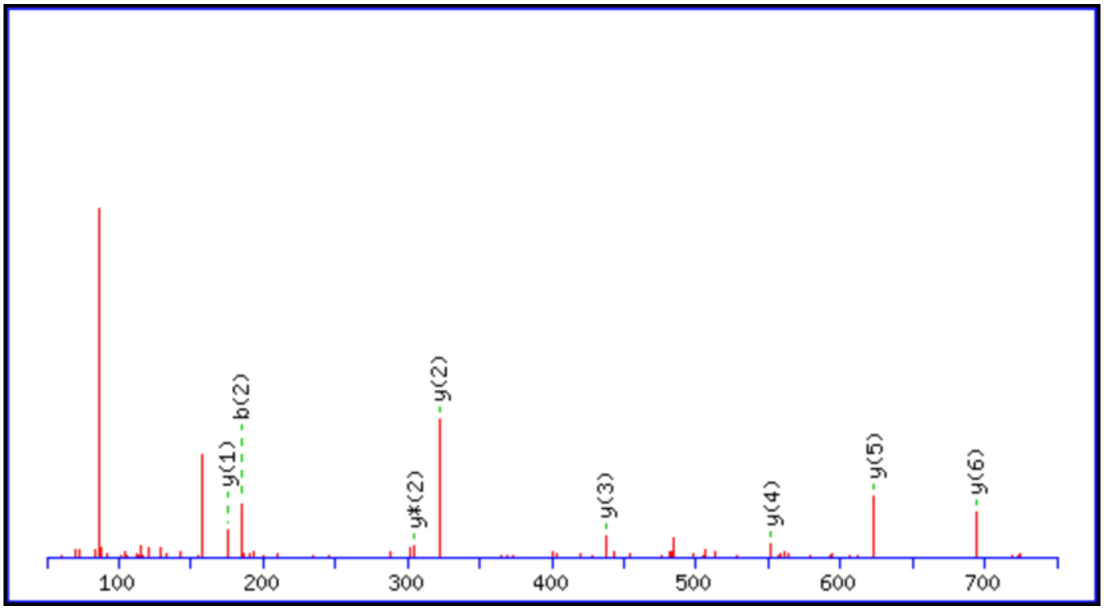

Monoisotopic mass of neutral peptide Mr(calc): 806.3923
Fixed modifications: Carbamidomethyl (C) (apply to specified residues or termini only)
Ions Score: 45 **Expect:** 0.0033
Matches : 8/50 fragment ions using 14 most intense peaks (help)

#	b	b^{++}	b^0	b^{0++}	Seq.	y	y^{++}	y*	y^{*++}	y^0	y^{0++}	#
1	114.0913	57.5493			L							7
2	**185.1285**	93.0679			A	***694.3155***	347.6614	677.2889	339.1481	676.3049	338.6561	6
3	256.1656	128.5864			A	***623.2784***	312.1428	606.2518	303.6295	605.2678	303.1375	5
4	371.1925	186.0999	353.1819	177.0946	D	***552.2413***	276.6243	535.2147	268.1110	534.2307	267.6190	4
5	486.2195	243.6134	468.2089	234.6081	D	***437.2143***	219.1108	420.1878	210.5975	419.2037	210.1055	3
6	633.2879	317.1476	615.2773	308.1423	F	***322.1874***	161.5973	305.1608	153.0840			2

7					R	*175.1190*	88.0631	158.0924	79.5498			1

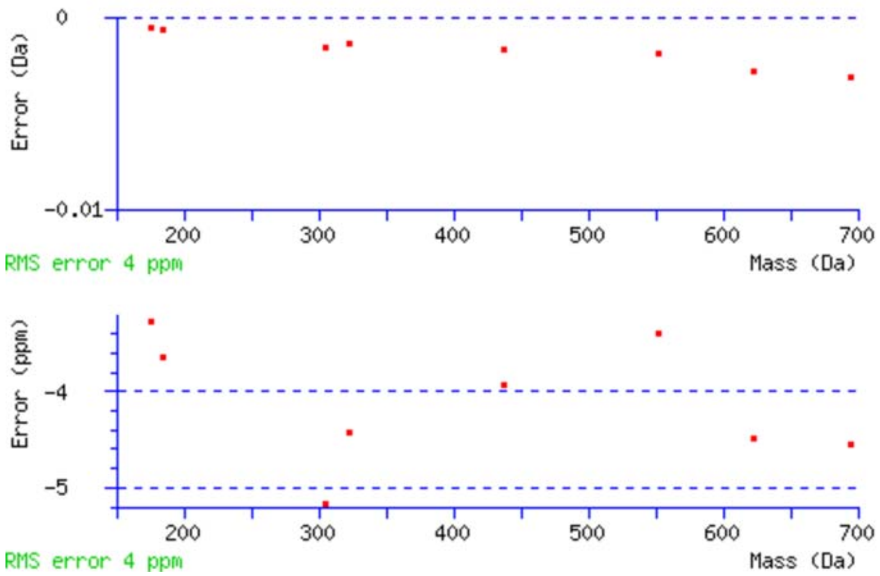

NCBI **BLAST** search of LAADDFR
(Parameters: blastp, nr protein database, expect=20000, no filter, PAM30)
Other BLAST web gateways

All matches to this query

Score	Mr(calc)	Delta	Sequence
45.4	806.3923	-0.0001	LAADDFR
15.1	804.3878	2.0044	RPDNFR
6.5	806.3923	-0.0001	LDQQFR
6.5	806.3923	-0.0001	LDQQFR
6.0	806.3922	-0.0001	GELANFR
6.0	804.3878	2.0044	NRPDFR
6.0	806.3923	-0.0001	QLEDFR
5.7	806.3923	-0.0001	IAGFDER
5.3	806.3844	0.0078	LAAQMQK
3.2	806.3891	0.0031	IACCRK

Mascot: http://www.matrixscience.com/

Mascot Search Results

Peptide View

MS/MS Fragmentation of **LAADDFR**
Found in **ch17u_O76014|KRT37_HUMAN** in **uni_human**, Keratin, type I cuticular Ha7 OS=Homo sapiens GN=KRT37 PE=3 SV=3

Match to Query 572: 806.392188 from(404.203370,2+) intensity(7881964.5000) rtinseconds(1442) scans(7297) index(5282)
Title: 160219_Sunil_SDSII_A_Spectrum056605_scans__7297_RTINSECONDS=1442
Data file L:\\QE_2016\\160219_Sunil_KAP_LKC\\TMgf\\T\\T160219_Sunil_SDSII_A.mgf

Click mouse within plot area to zoom in by factor of two about that point
Or, Plot from 50 to 800 Da Full range
Label all possible matches ○ Label matches used for scoring ◉
Show Y-axis ☐

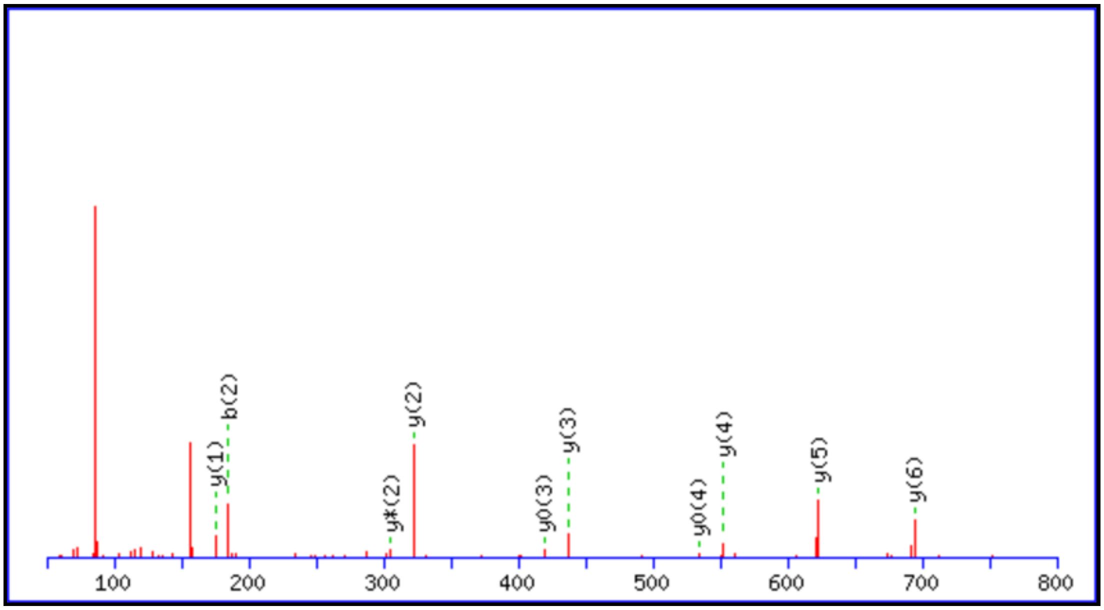

Monoisotopic mass of neutral peptide Mr(calc): 806.3923
Fixed modifications: Carbamidomethyl (C) (apply to specified residues or termini only)
Ions Score: 51 **Expect:** 0.00086
Matches : 10/50 fragment ions using 14 most intense peaks (help)

#	b	b^{++}	b^0	b^{0++}	Seq.	y	y^{++}	y*	y^{*++}	y^0	y^{0++}	#
1	114.0913	57.5493			L							7
2	**185.1285**	93.0679			A	***694.3155***	347.6614	677.2889	339.1481	676.3049	338.6561	6
3	256.1656	128.5864			A	***623.2784***	312.1428	606.2518	303.6295	605.2678	303.1375	5
4	371.1925	186.0999	353.1819	177.0946	D	***552.2413***	276.6243	535.2147	268.1110	534.2307	267.6190	4
5	486.2195	243.6134	468.2089	234.6081	D	***437.2143***	219.1108	420.1878	210.5975	419.2037	210.1055	3
6	633.2879	317.1476	615.2773	308.1423	F	***322.1874***	161.5973	305.1608	153.0840			2

**7**					**R**	***175.1190***	88.0631	158.0924	79.5498			**1**

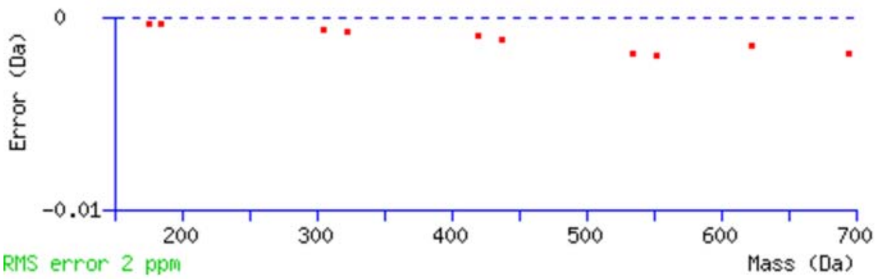

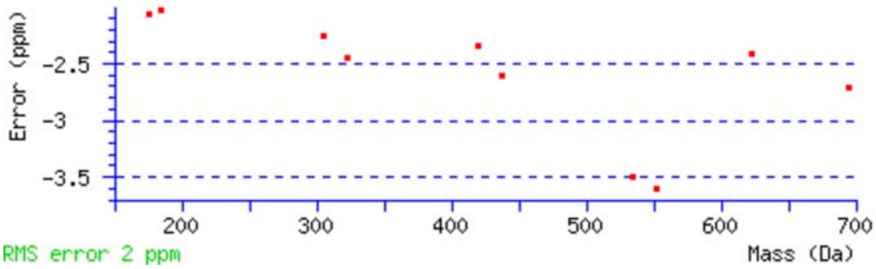

NCBI **BLAST** search of LAADDFR
(Parameters: blastp, nr protein database, expect=20000, no filter, PAM30)
Other BLAST web gateways

All matches to this query

Score	Mr(calc)	Delta	Sequence
51.1	806.3923	-0.0001	LAADDFR
16.8	804.3878	2.0044	RPDNFR
14.7	806.3923	-0.0001	IAGFDER
13.8	806.3844	0.0078	LAAQMQK
12.6	804.3912	2.0010	LASQACR
7.2	804.3878	2.0044	NRPDFR
7.2	806.3923	-0.0001	QLEDFR
5.9	806.3923	-0.0001	LDQQFR
5.9	806.3923	-0.0001	LDQQFR
5.5	806.3922	-0.0001	GELANFR

Mascot: http://www.matrixscience.com/

Mascot Search Results

Peptide View

MS/MS Fragmentation of **LAADDFR**
Found in **ch17u_O76014|KRT37_HUMAN** in **uni_human**, Keratin, type I cuticular Ha7 OS=Homo sapiens GN=KRT37 PE=3 SV=3

Match to Query 576: 806.392248 from(404.203400,2+) intensity(513134.1563) rtinseconds(1172) scans(6141) index(34577)
Title: 160219_Sunil_SDSII_A_Spectrum088534_scans__6141_RTINSECONDS=1172
Data file L:\\QE_2016\\160219_Sunil_KAP_LKC\\TMgf\\T\\T160219_Sunil_SDSII_A.mgf

Click mouse within plot area to zoom in by factor of two about that point
Or, Plot from 50 to 700 Da Full range
Label all possible matches ○ Label matches used for scoring ◉
Show Y-axis ☐

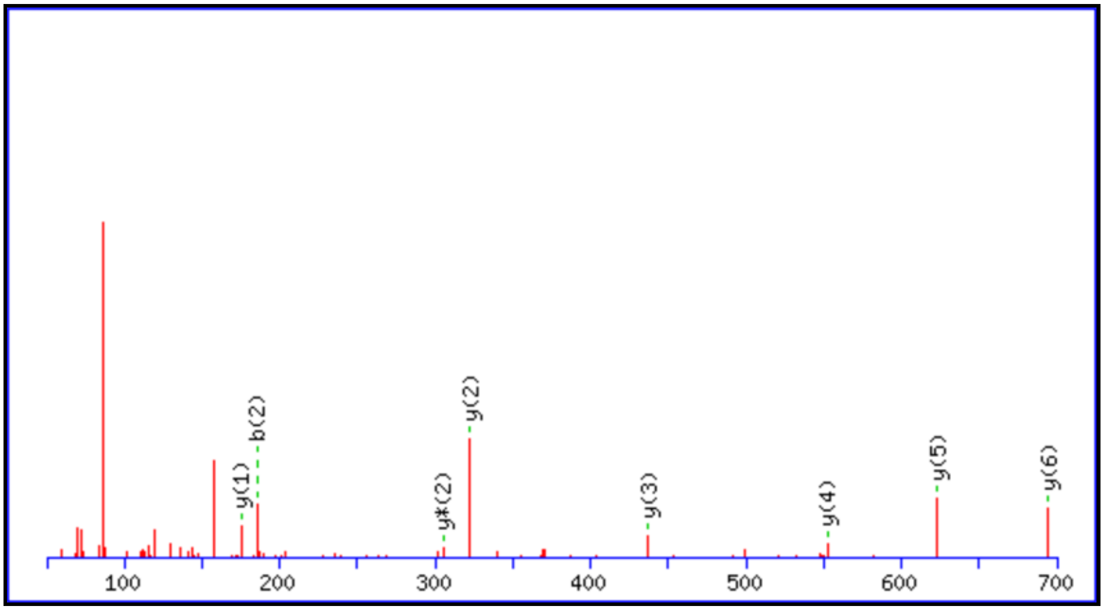

Monoisotopic mass of neutral peptide Mr(calc): 806.3923
Fixed modifications: Carbamidomethyl (C) (apply to specified residues or termini only)
Ions Score: 48 **Expect:** 0.002
Matches : 8/50 fragment ions using 13 most intense peaks (help)

#	b	b^{++}	b^{0}	b^{0++}	Seq.	y	y^{++}	y*	y^{*++}	y^{0}	y^{0++}	#
1	114.0913	57.5493			L							7
2	**185.1285**	93.0679			A	***694.3155***	347.6614	677.2889	339.1481	676.3049	338.6561	6
3	256.1656	128.5864			A	***623.2784***	312.1428	606.2518	303.6295	605.2678	303.1375	5
4	371.1925	186.0999	353.1819	177.0946	D	***552.2413***	276.6243	535.2147	268.1110	534.2307	267.6190	4
5	486.2195	243.6134	468.2089	234.6081	D	***437.2143***	219.1108	420.1878	210.5975	419.2037	210.1055	3
6	633.2879	317.1476	615.2773	308.1423	F	***322.1874***	161.5973	305.1608	153.0840			2

7					R	*175.1190*	88.0631	158.0924	79.5498			1

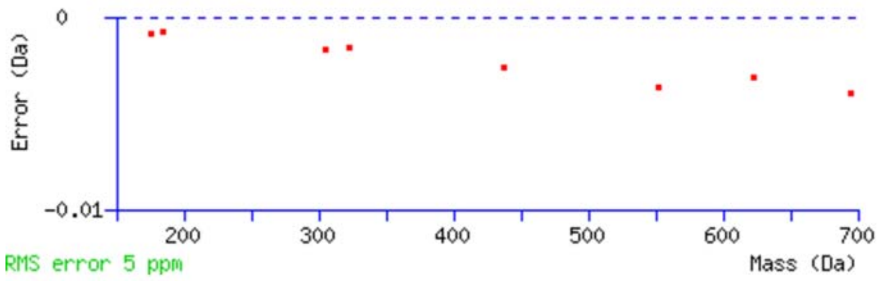

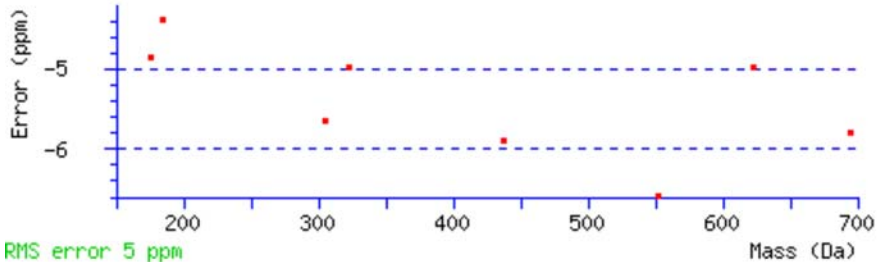

NCBI **BLAST** search of LAADDFR
(Parameters: blastp, nr protein database, expect=20000, no filter, PAM30)
Other BLAST web gateways

All matches to this query

Score	Mr(calc)	Delta	Sequence
47.6	806.3923	-0.0000	LAADDFR
16.0	804.3878	2.0044	RPDNFR
13.8	806.3844	0.0079	LAAQMQK
7.0	806.3923	-0.0000	LDQQFR
7.0	806.3923	-0.0000	LDQQFR
6.6	806.3923	-0.0000	IAGFDER
6.4	806.3922	0.0000	GELANFR
6.4	804.3878	2.0044	NRPDFR
6.4	806.3923	-0.0000	QLEDFR
3.3	806.3891	0.0031	IACCRK

Mascot: http://www.matrixscience.com/

Mascot Search Results

Peptide View

MS/MS Fragmentation of **LAADDFR**
Found in **ch17u_O76014|KRT37_HUMAN** in **uni_human**, Keratin, type I cuticular Ha7 OS=Homo sapiens GN=KRT37 PE=3 SV=3

Match to Query 578: 806.392248 from(404.203400,2+) intensity(2844824.7500) rtinseconds(1047) scans(5420) index(33948)
Title: 160219_Sunil_SDSII_A_Spectrum087904_scans__5420_RTINSECONDS=1047
Data file L:\\QE_2016\\160219_Sunil_KAP_LKC\\TMgf\\T\\T160219_Sunil_SDSII_A.mgf

Click mouse within plot area to zoom in by factor of two about that point
Or, Plot from 50 to 750 Da Full range
Label all possible matches ○ Label matches used for scoring ◉
Show Y-axis ☐

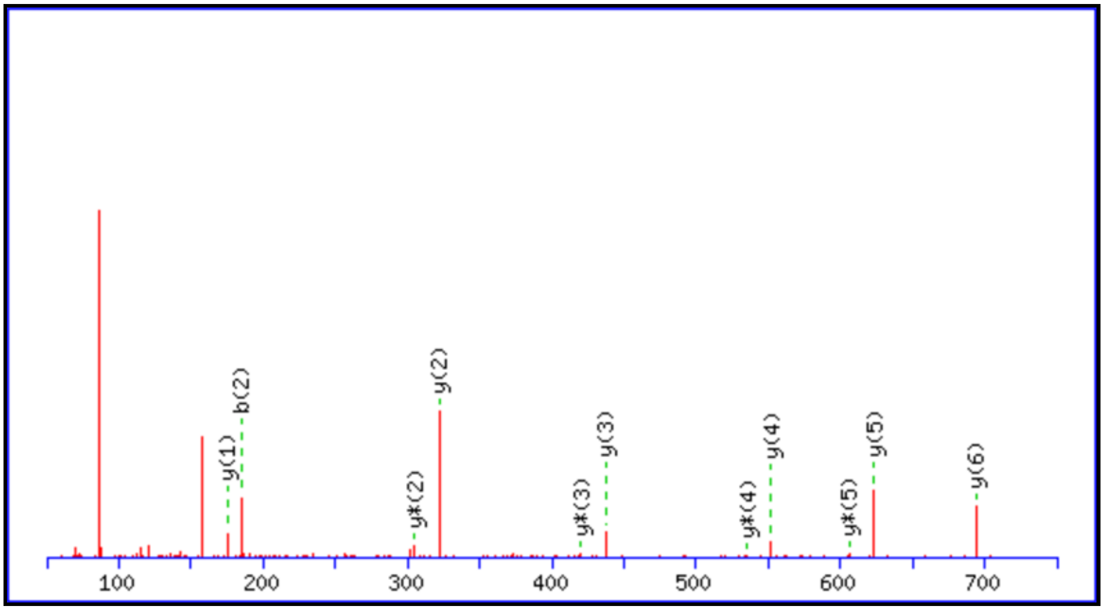

Monoisotopic mass of neutral peptide Mr(calc): 806.3923
Fixed modifications: Carbamidomethyl (C) (apply to specified residues or termini only)
Ions Score: 55 **Expect:** 0.00037
Matches : 11/50 fragment ions using 14 most intense peaks (help)

#	b	b^{++}	b^0	b^{0++}	Seq.	y	y^{++}	y*	y^{*++}	y^0	y^{0++}	#
1	114.0913	57.5493			L							7
2	**185.1285**	93.0679			A	***694.3155***	347.6614	677.2889	339.1481	676.3049	338.6561	6
3	256.1656	128.5864			A	***623.2784***	312.1428	606.2518	303.6295	605.2678	303.1375	5
4	371.1925	186.0999	353.1819	177.0946	D	***552.2413***	276.6243	535.2147	268.1110	534.2307	267.6190	4
5	486.2195	243.6134	468.2089	234.6081	D	***437.2143***	219.1108	420.1878	210.5975	419.2037	210.1055	3
6	633.2879	317.1476	615.2773	308.1423	F	***322.1874***	161.5973	305.1608	153.0840			2

7					R	*175.1190*	88.0631	158.0924	79.5498			1

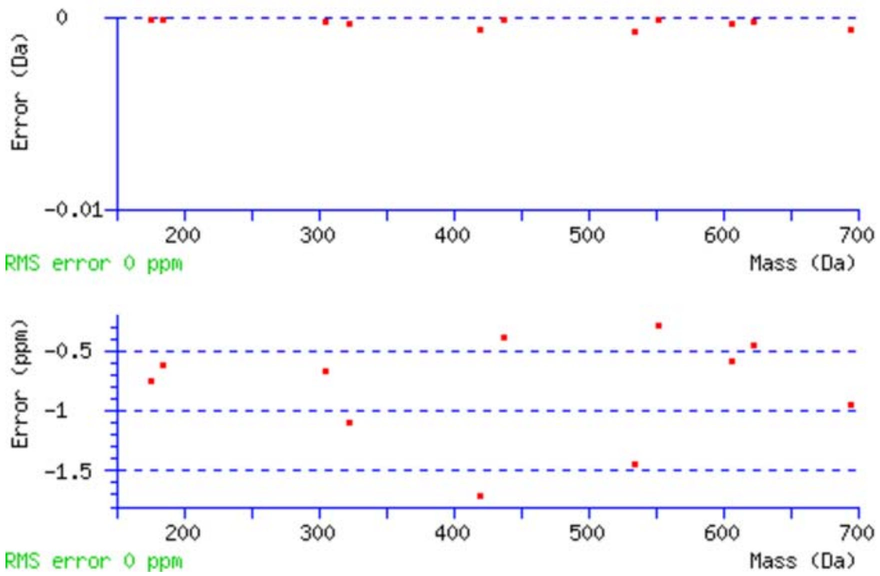

NCBI **BLAST** search of LAADDFR
(Parameters: blastp, nr protein database, expect=20000, no filter, PAM30)
Other BLAST web gateways

All matches to this query

Score	Mr(calc)	Delta	Sequence
54.8	806.3923	-0.0000	LAADDFR
19.5	804.3878	2.0044	RPDNFR
14.4	806.3844	0.0079	LAAQMQK
7.8	806.3922	0.0000	GELANFR
7.8	804.3878	2.0044	NRPDFR
7.8	806.3923	-0.0000	QLEDFR
7.7	806.3923	-0.0000	IAGFDER
6.8	806.3923	-0.0000	LDQQFR
6.8	806.3923	-0.0000	LDQQFR
3.8	806.3891	0.0031	IACCRK

Mascot: http://www.matrixscience.com/

Mascot Search Results

Peptide View

MS/MS Fragmentation of **LAADDFR**
Found in **ch17u_O76014|KRT37_HUMAN** in **uni_human**, Keratin, type I cuticular Ha7 OS=Homo sapiens GN=KRT37 PE=3 SV=3

Match to Query 579: 806.392308 from(404.203430,2+) intensity(4806223.0000) rtinseconds(1228) scans(6372) index(19623)
Title: 160219_Sunil_SDSII_A_Spectrum072184_scans__6372_RTINSECONDS=1228
Data file L:\\QE_2016\\160219_Sunil_KAP_LKC\\TMgf\\T\\T160219_Sunil_SDSII_A.mgf

Click mouse within plot area to zoom in by factor of two about that point
Or, Plot from 50 to 700 Da Full range
Label all possible matches ○ Label matches used for scoring ◉
Show Y-axis ☐

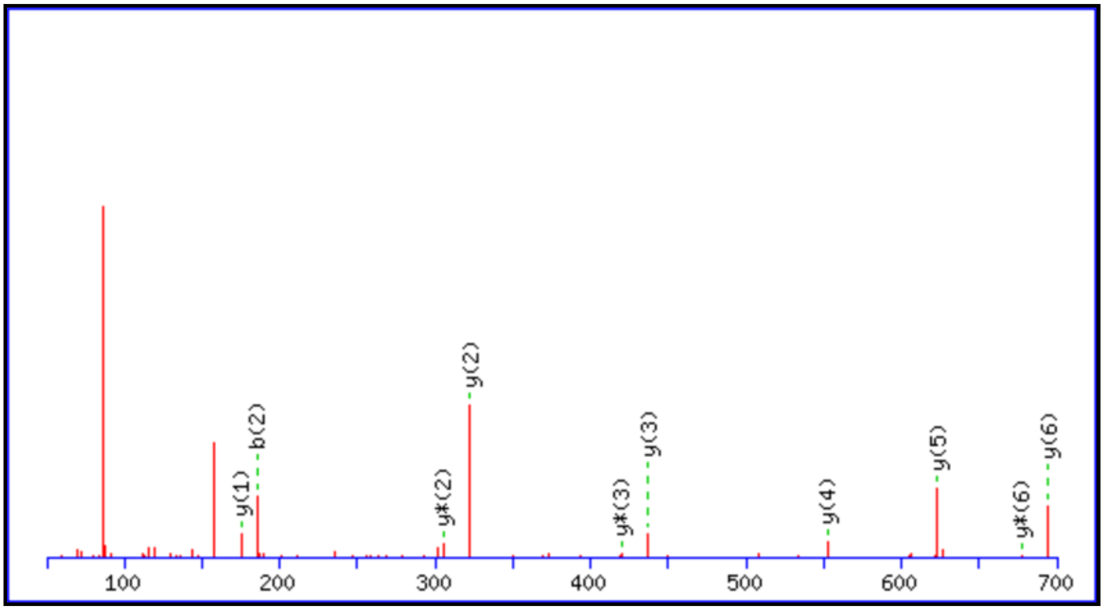

Monoisotopic mass of neutral peptide Mr(calc): 806.3923
Fixed modifications: Carbamidomethyl (C) (apply to specified residues or termini only)
Ions Score: 52 **Expect:** 0.00077
Matches : 10/50 fragment ions using 14 most intense peaks (help)

#	b	b^{++}	b^0	b^{0++}	Seq.	y	y^{++}	y*	y^{*++}	y^0	y^{0++}	#
1	114.0913	57.5493			L							7
2	**185.1285**	93.0679			A	***694.3155***	347.6614	677.2889	339.1481	676.3049	338.6561	6
3	256.1656	128.5864			A	***623.2784***	312.1428	606.2518	303.6295	605.2678	303.1375	5
4	371.1925	186.0999	353.1819	177.0946	D	***552.2413***	276.6243	535.2147	268.1110	534.2307	267.6190	4
5	486.2195	243.6134	468.2089	234.6081	D	***437.2143***	219.1108	420.1878	210.5975	419.2037	210.1055	3
6	633.2879	317.1476	615.2773	308.1423	F	***322.1874***	161.5973	305.1608	153.0840			2

7					R	*175.1190*	88.0631	158.0924	79.5498			1

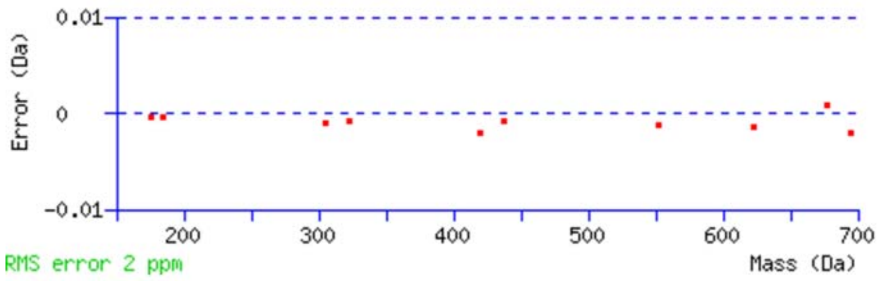

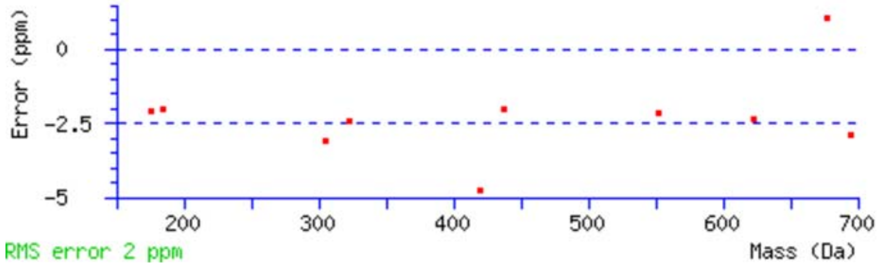

NCBI **BLAST** search of LAADDFR
(Parameters: blastp, nr protein database, expect=20000, no filter, PAM30)
Other BLAST web gateways

All matches to this query

Score	Mr(calc)	Delta	Sequence
51.8	806.3923	0.0001	LAADDFR
17.6	804.3878	2.0045	RPDNFR
14.4	806.3844	0.0079	LAAQMQK
8.4	806.3923	0.0001	LDQQFR
8.4	806.3923	0.0001	LDQQFR
7.9	806.3922	0.0001	GELANFR
7.9	804.3878	2.0045	NRPDFR
7.9	806.3923	0.0001	QLEDFR
7.7	806.3923	0.0001	IAGFDER
3.9	806.3891	0.0032	IACCRK

Mascot: http://www.matrixscience.com/

Mascot Search Results

Peptide View

MS/MS Fragmentation of **LAADDFR**
Found in **ch17u_O76014|KRT37_HUMAN** in **uni_human**, Keratin, type I cuticular Ha7 OS=Homo sapiens GN=KRT37 PE=3 SV=3

Match to Query 583: 806.392368 from(404.203460,2+) intensity(2102181.7500) rtinseconds(1294) scans(6750) index(19942)
Title: 160219_Sunil_SDSII_A_Spectrum072503_scans__6750_RTINSECONDS=1294
Data file L:\\QE_2016\\160219_Sunil_KAP_LKC\\TMgf\\T\\T160219_Sunil_SDSII_A.mgf

Click mouse within plot area to zoom in by factor of two about that point
Or, Plot from 50 to 750 Da Full range
Label all possible matches ○ Label matches used for scoring ◉
Show Y-axis ☐

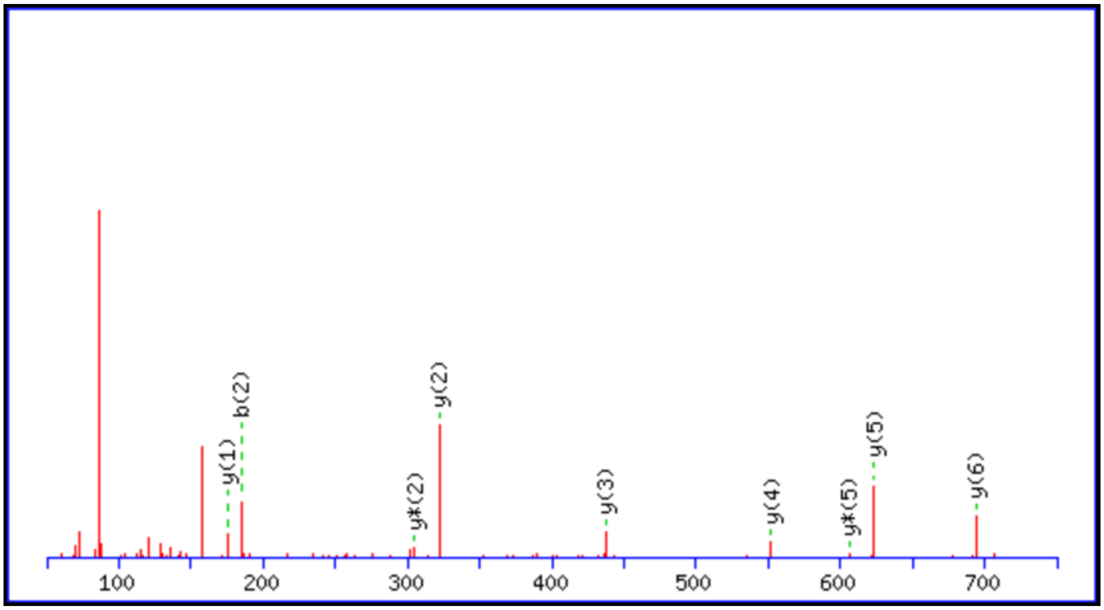

Monoisotopic mass of neutral peptide Mr(calc): 806.3923
Fixed modifications: Carbamidomethyl (C) (apply to specified residues or termini only)
Ions Score: 48 **Expect:** 0.0017
Matches : 9/50 fragment ions using 14 most intense peaks (help)

#	b	b^{++}	b^0	b^{0++}	Seq.	y	y^{++}	y*	y^{*++}	y^0	y^{0++}	#
1	114.0913	57.5493			L							7
2	**185.1285**	93.0679			A	***694.3155***	347.6614	677.2889	339.1481	676.3049	338.6561	6
3	256.1656	128.5864			A	***623.2784***	312.1428	606.2518	303.6295	605.2678	303.1375	5
4	371.1925	186.0999	353.1819	177.0946	D	***552.2413***	276.6243	535.2147	268.1110	534.2307	267.6190	4
5	486.2195	243.6134	468.2089	234.6081	D	***437.2143***	219.1108	420.1878	210.5975	419.2037	210.1055	3
6	633.2879	317.1476	615.2773	308.1423	F	***322.1874***	161.5973	305.1608	153.0840			2

7					R	175.1190	88.0631	158.0924	79.5498			1

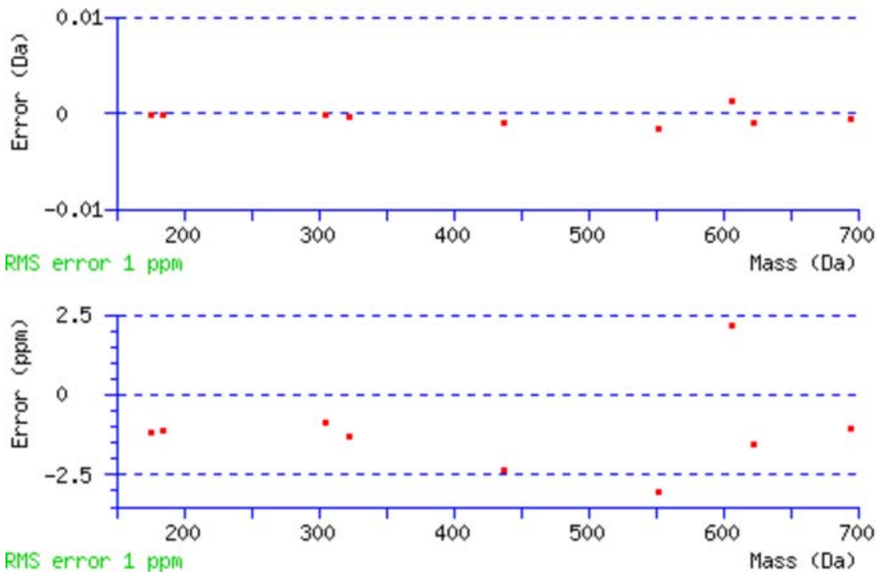

NCBI **BLAST** search of LAADDFR
(Parameters: blastp, nr protein database, expect=20000, no filter, PAM30)
Other BLAST web gateways

All matches to this query

Score	Mr(calc)	Delta	Sequence
48.4	806.3923	0.0001	LAADDFR
15.1	804.3878	2.0045	RPDNFR
14.1	806.3844	0.0080	LAAQMQK
7.4	806.3923	0.0001	IAGFDER
6.2	806.3923	0.0001	LDQQFR
6.2	806.3923	0.0001	LDQQFR
5.8	806.3922	0.0001	GELANFR
5.8	804.3878	2.0045	NRPDFR
5.8	806.3923	0.0001	QLEDFR
5.3	806.3922	0.0001	ELENFR

Mascot: http://www.matrixscience.com/

Mascot Search Results

Peptide View

MS/MS Fragmentation of **LAADDFR**
Found in **ch17u_O76014|KRT37_HUMAN** in **uni_human**, Keratin, type I cuticular Ha7 OS=Homo sapiens GN=KRT37 PE=3 SV=3

Match to Query 588: 806.392608 from(404.203580,2+) intensity(245699.3281) rtinseconds(1594) scans(8488) index(21491)
Title: 160219_Sunil_SDSII_A_Spectrum074052_scans__8488_RTINSECONDS=1594
Data file L:\\QE_2016\\160219_Sunil_KAP_LKC\\TMgf\\T\\T160219_Sunil_SDSII_A.mgf

Click mouse within plot area to zoom in by factor of two about that point
Or, Plot from 50 to 700 Da Full range
Label all possible matches ○ Label matches used for scoring ◉
Show Y-axis ☐

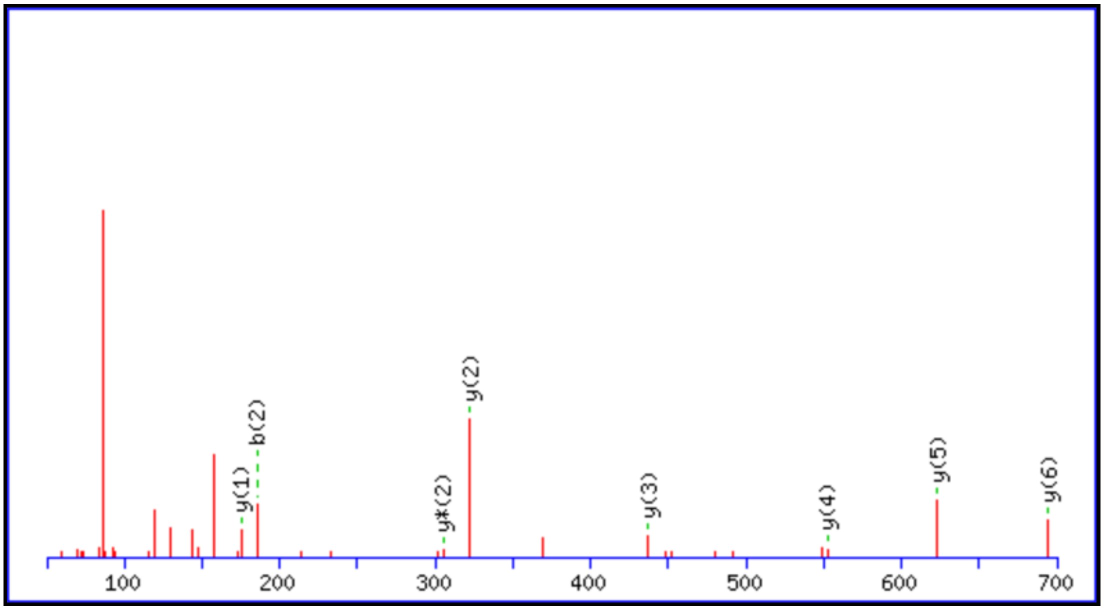

Monoisotopic mass of neutral peptide Mr(calc): 806.3923
Fixed modifications: Carbamidomethyl (C) (apply to specified residues or termini only)
Ions Score: 50 **Expect:** 0.0011
Matches : 8/50 fragment ions using 12 most intense peaks (help)

#	b	b^{++}	b^{0}	b^{0++}	Seq.	y	y^{++}	y*	y^{*++}	y^{0}	y^{0++}	#
1	114.0913	57.5493			L							7
2	**185.1285**	93.0679			A	***694.3155***	347.6614	677.2889	339.1481	676.3049	338.6561	6
3	256.1656	128.5864			A	***623.2784***	312.1428	606.2518	303.6295	605.2678	303.1375	5
4	371.1925	186.0999	353.1819	177.0946	D	***552.2413***	276.6243	535.2147	268.1110	534.2307	267.6190	4
5	486.2195	243.6134	468.2089	234.6081	D	***437.2143***	219.1108	420.1878	210.5975	419.2037	210.1055	3
6	633.2879	317.1476	615.2773	308.1423	F	***322.1874***	161.5973	305.1608	153.0840			2

7					R	*175.1190*	88.0631	158.0924	79.5498			1

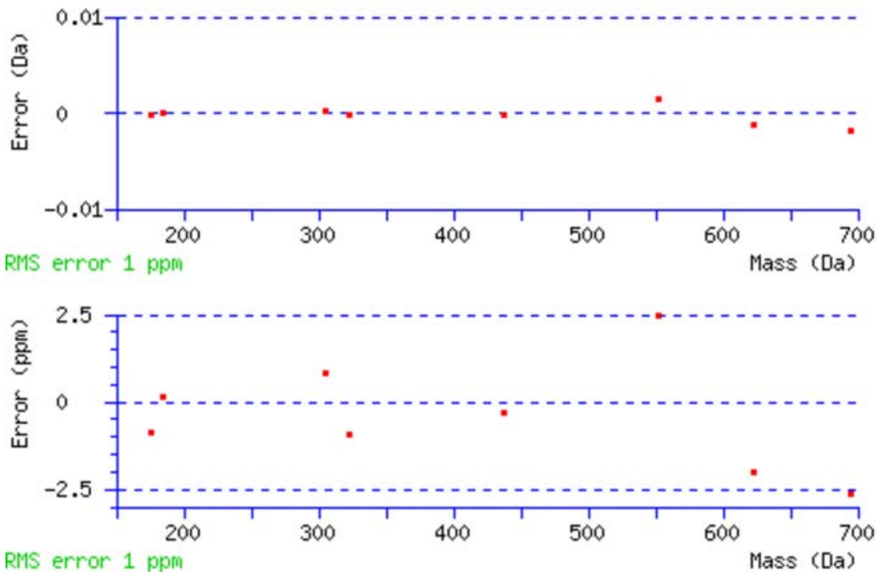

NCBI **BLAST** search of LAADDFR
(Parameters: blastp, nr protein database, expect=20000, no filter, PAM30)
Other BLAST web gateways

All matches to this query

Score	Mr(calc)	Delta	Sequence
50.1	806.3923	0.0004	LAADDFR
17.5	804.3878	2.0048	RPDNFR
7.9	806.3923	0.0004	LDQQFR
7.9	806.3923	0.0004	LDQQFR
7.5	806.3922	0.0004	GELANFR
7.5	804.3878	2.0048	NRPDFR
7.5	806.3923	0.0004	QLEDFR
7.3	806.3923	0.0004	QQQVFR
7.0	806.3923	0.0004	IAGFDER
2.9	806.3891	0.0035	IACCRK

Mascot: http://www.matrixscience.com/

Mascot Search Results

Peptide View

MS/MS Fragmentation of **LAADDFR**
Found in **ch17u_O76014|KRT37_HUMAN** in **uni_human**, Keratin, type I cuticular Ha7 OS=Homo sapiens GN=KRT37 PE=3 SV=3

Match to Query 591: 806.392668 from(404.203610,2+) intensity(713885.1250) rtinseconds(1141) scans(5962) index(34422)
Title: 160219_Sunil_SDSII_A_Spectrum088378_scans__5962_RTINSECONDS=1141
Data file L:\\QE_2016\\160219_Sunil_KAP_LKC\\TMgf\\T\\T160219_Sunil_SDSII_A.mgf

Click mouse within plot area to zoom in by factor of two about that point
Or, Plot from 50 to 700 Da Full range
Label all possible matches ○ Label matches used for scoring ◉
Show Y-axis ☐

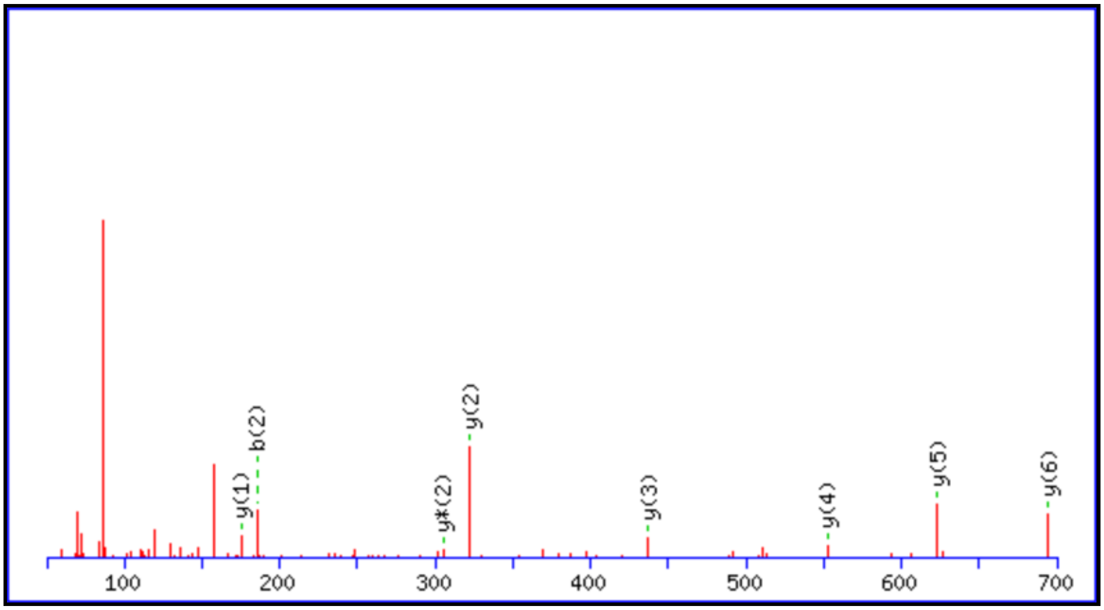

Monoisotopic mass of neutral peptide Mr(calc): 806.3923
Fixed modifications: Carbamidomethyl (C) (apply to specified residues or termini only)
Ions Score: 47 **Expect:** 0.0021
Matches : 8/50 fragment ions using 13 most intense peaks (help)

#	b	b^{++}	b^{0}	b^{0++}	Seq.	y	y^{++}	y*	y^{*++}	y^{0}	y^{0++}	#
1	114.0913	57.5493			L							7
2	**185.1285**	93.0679			A	***694.3155***	347.6614	677.2889	339.1481	676.3049	338.6561	6
3	256.1656	128.5864			A	***623.2784***	312.1428	606.2518	303.6295	605.2678	303.1375	5
4	371.1925	186.0999	353.1819	177.0946	D	***552.2413***	276.6243	535.2147	268.1110	534.2307	267.6190	4
5	486.2195	243.6134	468.2089	234.6081	D	***437.2143***	219.1108	420.1878	210.5975	419.2037	210.1055	3
6	633.2879	317.1476	615.2773	308.1423	F	***322.1874***	161.5973	305.1608	153.0840			2

7					R	*175.1190*	88.0631	158.0924	79.5498			1

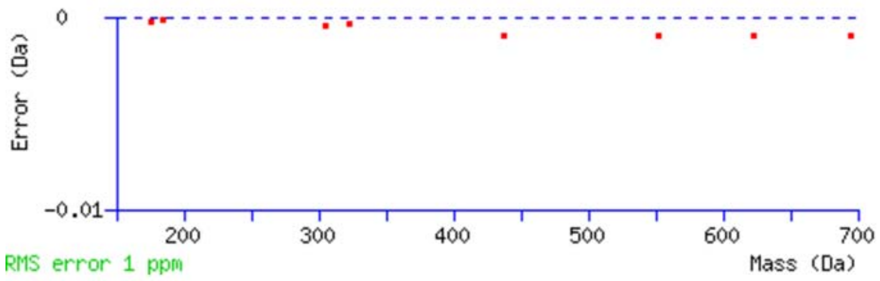

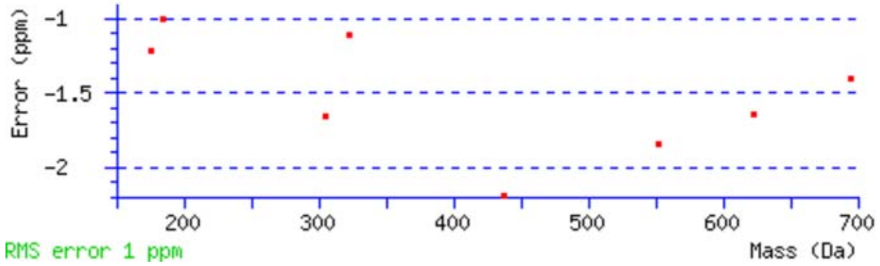

NCBI **BLAST** search of LAADDFR
(Parameters: blastp, nr protein database, expect=20000, no filter, PAM30)
Other BLAST web gateways

All matches to this query

Score	Mr(calc)	Delta	Sequence
47.1	806.3923	0.0004	LAADDFR
15.5	804.3878	2.0048	RPDNFR
6.5	806.3923	0.0004	LDQQFR
6.5	806.3923	0.0004	LDQQFR
6.0	806.3923	0.0004	IAGFDER
5.9	806.3922	0.0004	GELANFR
5.9	804.3878	2.0048	NRPDFR
5.9	806.3923	0.0004	QLEDFR
2.9	806.3891	0.0036	IACCRK
2.9	806.3956	-0.0030	LACTNTK

Mascot: http://www.matrixscience.com/

Mascot Search Results

Peptide View

MS/MS Fragmentation of **LAADDFR**
Found in **ch17u_O76014|KRT37_HUMAN** in **uni_human**, Keratin, type I cuticular Ha7 OS=Homo sapiens GN=KRT37 PE=3 SV=3

Match to Query 594: 806.393168 from(404.203860,2+) intensity(927897344.0000) rtinseconds(1133) scans(5817) index(19136)
Title: 160219_Sunil_SDSII_A_Spectrum071697_scans__5817_RTINSECONDS=1133
Data file L:\\QE_2016\\160219_Sunil_KAP_LKC\\TMgf\\T\\T160219_Sunil_SDSII_A.mgf

Click mouse within plot area to zoom in by factor of two about that point
Or, Plot from 50 to 700 Da Full range
Label all possible matches ○ Label matches used for scoring ◉
Show Y-axis ☐

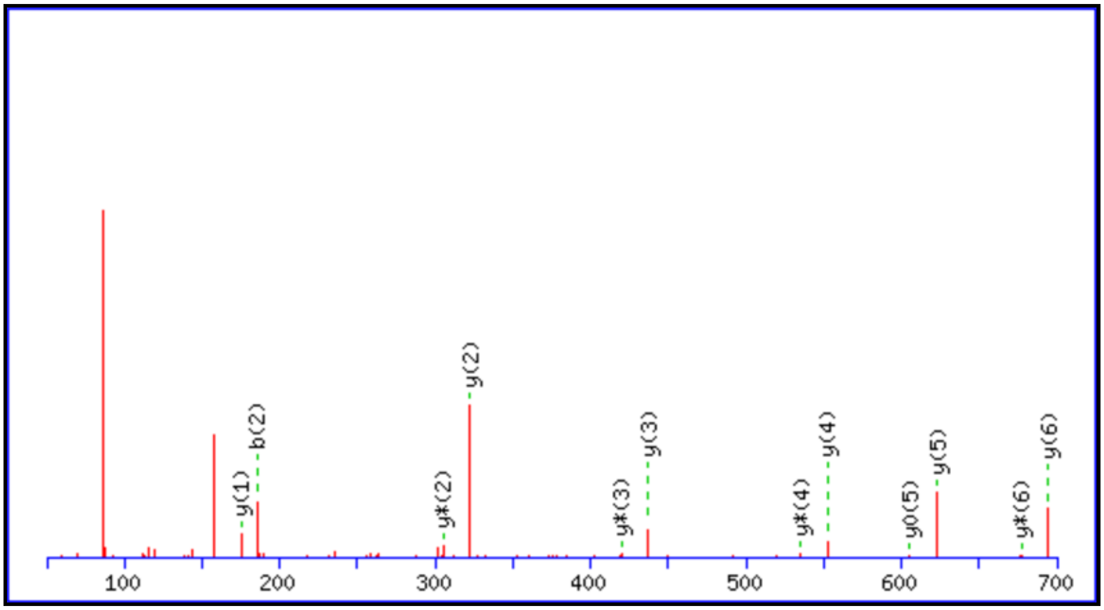

Monoisotopic mass of neutral peptide Mr(calc): 806.3923
Fixed modifications: Carbamidomethyl (C) (apply to specified residues or termini only)
Ions Score: 58 **Expect:** 0.00015
Matches : 12/50 fragment ions using 14 most intense peaks (help)

#	b	b++	b0	b0++	Seq.	y	y++	y*	y*++	y0	y0++	#
1	114.0913	57.5493			L							7
2	**185.1285**	93.0679			A	***694.3155***	347.6614	677.2889	339.1481	676.3049	338.6561	6
3	256.1656	128.5864			A	***623.2784***	312.1428	606.2518	303.6295	605.2678	303.1375	5
4	371.1925	186.0999	353.1819	177.0946	D	***552.2413***	276.6243	535.2147	268.1110	534.2307	267.6190	4
5	486.2195	243.6134	468.2089	234.6081	D	***437.2143***	219.1108	420.1878	210.5975	419.2037	210.1055	3
6	633.2879	317.1476	615.2773	308.1423	F	***322.1874***	161.5973	305.1608	153.0840			2

7					R	*175.1190*	88.0631	158.0924	79.5498		1

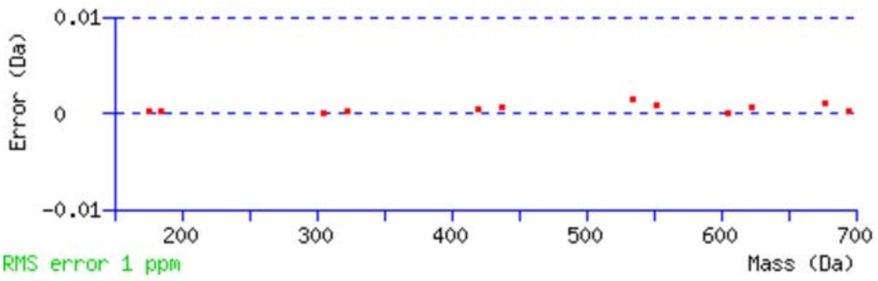

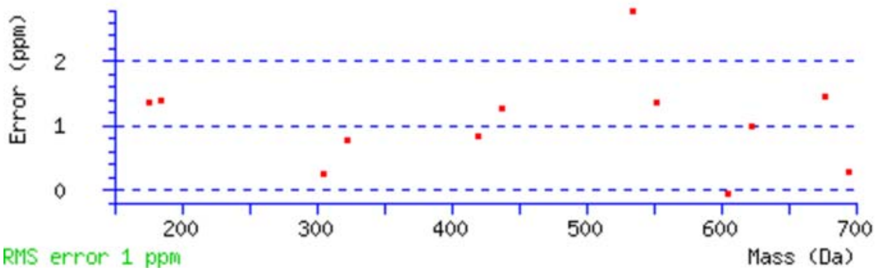

NCBI **BLAST** search of LAADDFR
(Parameters: blastp, nr protein database, expect=20000, no filter, PAM30)
Other BLAST web gateways

All matches to this query

Score	Mr(calc)	Delta	Sequence
58.3	806.3923	0.0009	LAADDFR
19.7	804.3878	2.0053	RPDNFR
16.6	806.3923	0.0009	IAGFDER
8.4	806.3923	0.0009	LDQQFR
8.4	806.3923	0.0009	LDQQFR
8.0	806.3922	0.0009	GELANFR
8.0	804.3878	2.0053	NRPDFR
8.0	806.3923	0.0009	QLEDFR
3.7	806.3891	0.0041	IACCRK
3.7	806.3956	-0.0025	LACTNTK

Mascot: http://www.matrixscience.com/

Mascot Search Results

Peptide View

MS/MS Fragmentation of **LENEIATYR**
Found in **ch17u_O76014|KRT37_HUMAN** in **uni_human**, Keratin, type I cuticular Ha7 OS=Homo sapiens GN=KRT37 PE=3 SV=3

Match to Query 5665: 1107.555888 from(554.785220,2+) intensity(1281169.6250) rtinseconds(1514) scans(7712) index(5640)
Title: 160219_Sunil_SDSII_A_Spectrum056963_scans__7712_RTINSECONDS=1514
Data file L:\\QE_2016\\160219_Sunil_KAP_LKC\\TMgf\\T\\T160219_Sunil_SDSII_A.mgf

Click mouse within plot area to zoom in by factor of two about that point
Or, Plot from 0 to 1200 Da Full range
Label all possible matches ○ Label matches used for scoring ◉
Show Y-axis □

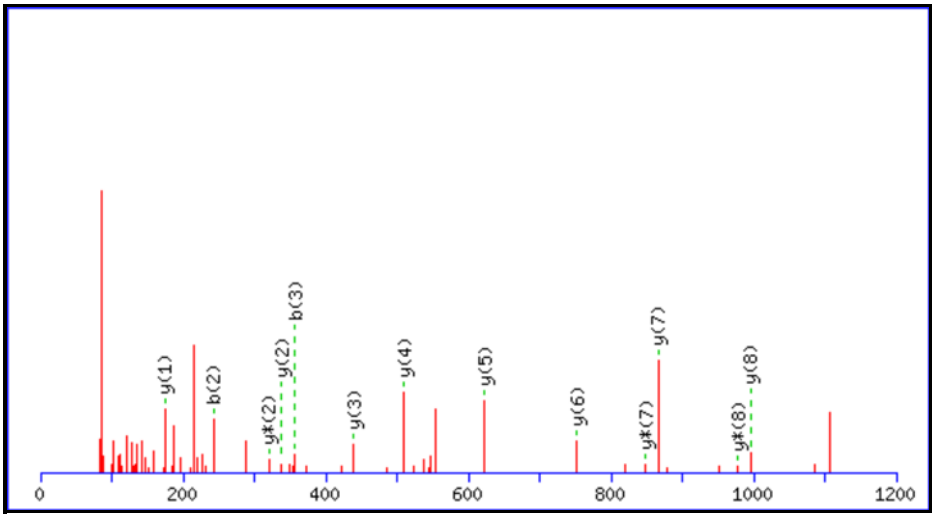

Monoisotopic mass of neutral peptide Mr(calc): 1107.5560
Fixed modifications: Carbamidomethyl (C) (apply to specified residues or termini only)
Ions Score: 54 **Expect:** 0.00048
Matches : 13/86 fragment ions using 29 most intense peaks (help)

#	b	b++	b*	b*++	b0	b0++	Seq.	y	y++	y*	y*++	y0	y0++	#
1	114.0913	57.5493					L							9
2	**243.1339**	122.0706			225.1234	113.0653	E	***995.4793***	498.2433	978.4527	489.7300	977.4687	489.2380	8
3	**357.1769**	179.0921	340.1503	170.5788	339.1663	170.0868	N	***866.4367***	433.7220	849.4101	425.2087	848.4261	424.7167	7
4	486.2195	243.6134	469.1929	235.1001	468.2089	234.6081	E	***752.3937***	376.7005	735.3672	368.1872	734.3832	367.6952	6
5	599.3035	300.1554	582.2770	291.6421	581.2930	291.1501	I	***623.3511***	312.1792	606.3246	303.6659	605.3406	303.1739	5
6	670.3406	335.6740	653.3141	327.1607	652.3301	326.6687	A	***510.2671***	255.6372	493.2405	247.1239	492.2565	246.6319	4
7	771.3883	386.1978	754.3618	377.6845	753.3777	377.1925	T	***439.2300***	220.1186	422.2034	211.6053	421.2194	211.1133	3
8	934.4516	467.7295	917.4251	459.2162	916.4411	458.7242	Y	***338.1823***	169.5948	321.1557	161.0815			2
9							R	***175.1190***	88.0631	158.0924	79.5498			1

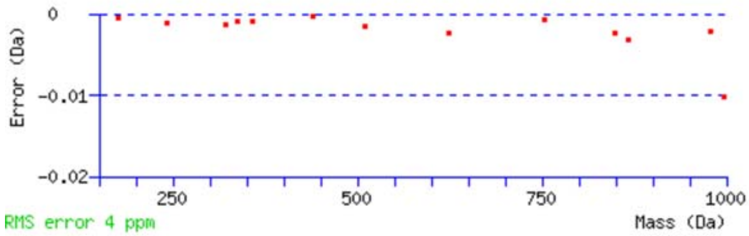

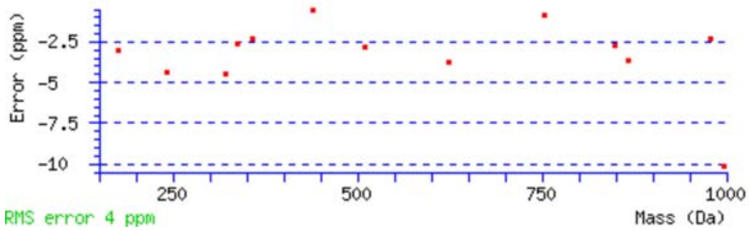

NCBI **BLAST** search of LENEIATYR
(Parameters: blastp, nr protein database, expect=20000, no filter, PAM30)
Other BLAST web gateways

All matches to this query

Score	Mr(calc)	Delta	Sequence
54.2	1107.5560	-0.0001	LENEIATYR
11.7	1107.5560	-0.0001	ELGGYVSNLR
3.6	1107.5594	-0.0035	IEEMLSKSR
3.6	1107.5594	-0.0035	LQSMLKSER
3.0	1107.5560	-0.0001	VPSDSAITYR
1.4	1107.5560	-0.0001	LYSSSGPELR

Mascot: http://www.matrixscience.com/

Mascot Search Results

Peptide View

MS/MS Fragmentation of **TIEELQQKILCSK**
Found in **ch17u_O76014|KRT37_HUMAN** in **uni_human**, Keratin, type I cuticular Ha7 OS=Homo sapiens GN=KRT37 PE=3 SV=3

Match to Query 21083: 1588.849332 from(530.623720,3+) intensity(3064983.7500) rtinseconds(1999) scans(10829) index(23561)
Title: 160219_Sunil_SDSII_A_Spectrum076123_scans__10829_RTINSECONDS=1999
Data file L:\\QE_2016\\160219_Sunil_KAP_LKC\\TMgf\\T\\T160219_Sunil_SDSII_A.mgf

Click mouse within plot area to zoom in by factor of two about that point
Or, Plot from 100 to 1600 Da Full range
Label all possible matches ○ Label matches used for scoring ◉
Show Y-axis ☐

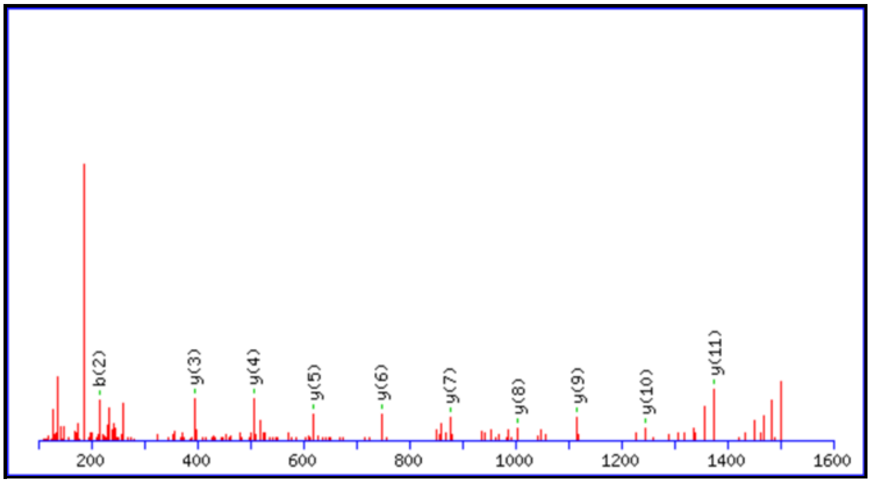

Monoisotopic mass of neutral peptide Mr(calc): 1588.8494
Fixed modifications: Carbamidomethyl (C) (apply to specified residues or termini only)
Ions Score: 72 **Expect:** 8.5e-006
Matches : 10/132 fragment ions using 14 most intense peaks (help)

#	b	b++	b*	b*++	b0	b0++	Seq.	y	y++	y*	y*++	y0	y0++	#
1	102.0550	51.5311			84.0444	42.5258	T							13
2	**215.1390**	108.0731			197.1285	99.0679	I	1488.8090	744.9082	1471.7825	736.3949	1470.7985	735.9029	12
3	344.1816	172.5944			326.1710	163.5892	E	***1375.7250***	688.3661	1358.6984	679.8529	1357.7144	679.3608	11
4	473.2242	237.1157			455.2136	228.1105	E	***1246.6824***	623.8448	1229.6558	615.3316	1228.6718	614.8395	10
5	586.3083	293.6578			568.2977	284.6525	L	***1117.6398***	559.3235	1100.6132	550.8103	1099.6292	550.3183	9
6	714.3668	357.6871	697.3403	349.1738	696.3563	348.6818	Q	***1004.5557***	502.7815	987.5292	494.2682	986.5452	493.7762	8
7	842.4254	421.7164	825.3989	413.2031	824.4149	412.7111	Q	***876.4972***	438.7522	859.4706	430.2389	858.4866	429.7469	7
8	970.5204	485.7638	953.4938	477.2506	952.5098	476.7585	K	***748.4386***	374.7229	731.4120	366.2096	730.4280	365.7176	6
9	1083.6045	542.3059	1066.5779	533.7926	1065.5939	533.3006	I	***620.3436***	310.6754	603.3171	302.1622	602.3330	301.6702	5
10	1196.6885	598.8479	1179.6620	590.3346	1178.6780	589.8426	L	***507.2595***	254.1334	490.2330	245.6201	489.2490	245.1281	4
11	1356.7192	678.8632	1339.6926	670.3499	1338.7086	669.8579	C	***394.1755***	197.5914	377.1489	189.0781	376.1649	188.5861	3
12	1443.7512	722.3792	1426.7246	713.8660	1425.7406	713.3740	S	234.1448	117.5761	217.1183	109.0628	216.1343	108.5708	2
13							K	147.1128	74.0600	130.0863	65.5468			1

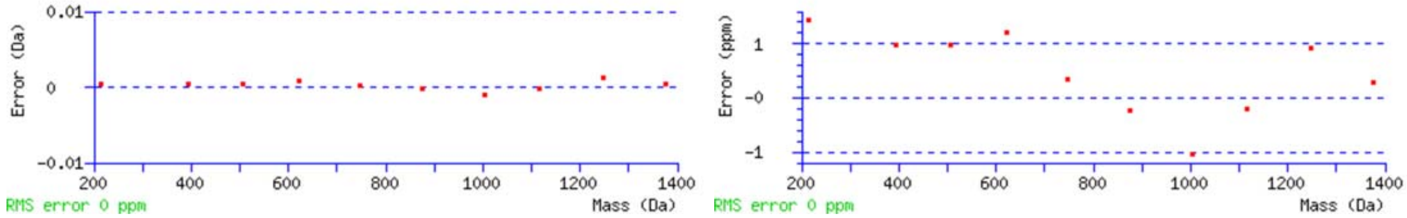

NCBI **BLAST** search of TIEELQQKILCSK
(Parameters: blastp, nr protein database, expect=20000, no filter, PAM30)
Other BLAST web gateways

All matches to this query

Score	Mr(calc)	Delta	Sequence
72.3	1588.8494	-0.0001	TIEELQQKILCSK

Mascot: http://www.matrixscience.com/

Mascot Search Results

Peptide View

MS/MS Fragmentation of **TIEELQQKILCSK**
Found in **ch17u_O76014|KRT37_HUMAN** in **uni_human**, Keratin, type I cuticular Ha7 OS=Homo sapiens GN=KRT37 PE=3 SV=3

Match to Query 21085: 1588.849828 from(795.432190,2+) intensity(958991.0000) rtinseconds(2002) scans(10846) index(23575)
Title: 160219_Sunil_SDSII_A_Spectrum076137_scans__10846_RTINSECONDS=2002
Data file L:\\QE_2016\\160219_Sunil_KAP_LKC\\TMgf\\T\\T160219_Sunil_SDSII_A.mgf

Click mouse within plot area to zoom in by factor of two about that point
Or, Plot from 100 to 1600 Da Full range
Label all possible matches ○ Label matches used for scoring ◉
Show Y-axis ☐

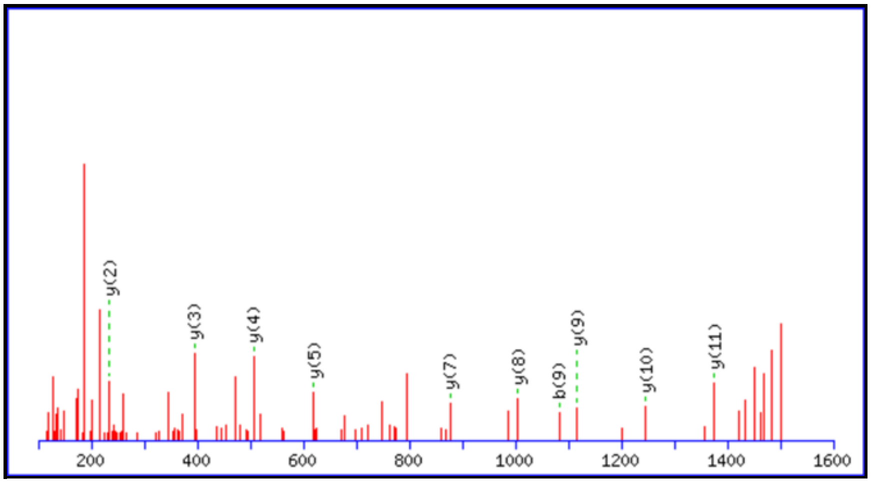

Monoisotopic mass of neutral peptide Mr(calc): 1588.8494
Fixed modifications: Carbamidomethyl (C) (apply to specified residues or termini only)
Ions Score: 72 **Expect:** 9.2e-006
Matches : 10/132 fragment ions using 14 most intense peaks (help)

#	b	b++	b*	b*++	b0	b0++	Seq.	y	y++	y*	y*++	y0	y0++	#
1	102.0550	51.5311			84.0444	42.5258	T							13
2	215.1390	108.0731			197.1285	99.0679	I	1488.8090	744.9082	1471.7825	736.3949	1470.7985	735.9029	12
3	344.1816	172.5944			326.1710	163.5892	E	**1375.7250**	688.3661	1358.6984	679.8529	1357.7144	679.3608	11
4	473.2242	237.1157			455.2136	228.1105	E	**1246.6824**	623.8448	1229.6558	615.3316	1228.6718	614.8395	10
5	586.3083	293.6578			568.2977	284.6525	L	**1117.6398**	559.3235	1100.6132	550.8103	1099.6292	550.3183	9
6	714.3668	357.6871	697.3403	349.1738	696.3563	348.6818	Q	**1004.5557**	502.7815	987.5292	494.2682	986.5452	493.7762	8
7	842.4254	421.7164	825.3989	413.2031	824.4149	412.7111	Q	**876.4972**	438.7522	859.4706	430.2389	858.4866	429.7469	7
8	970.5204	485.7638	953.4938	477.2506	952.5098	476.7585	K	748.4386	374.7229	731.4120	366.2096	730.4280	365.7176	6
9	**1083.6045**	542.3059	1066.5779	533.7926	1065.5939	533.3006	I	**620.3436**	310.6754	603.3171	302.1622	602.3330	301.6702	5
10	1196.6885	598.8479	1179.6620	590.3346	1178.6780	589.8426	L	**507.2595**	254.1334	490.2330	245.6201	489.2490	245.1281	4
11	1356.7192	678.8632	1339.6926	670.3499	1338.7086	669.8579	C	**394.1755**	197.5914	377.1489	189.0781	376.1649	188.5861	3
12	1443.7512	722.3792	1426.7246	713.8660	1425.7406	713.3740	S	**234.1448**	117.5761	217.1183	109.0628	216.1343	108.5708	2
13							K	147.1128	74.0600	130.0863	65.5468			1

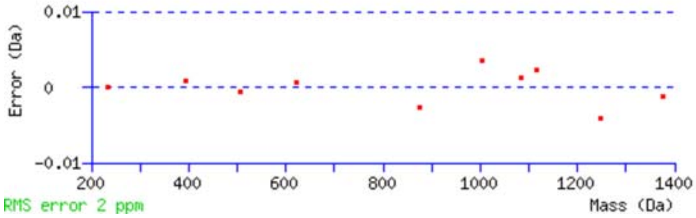

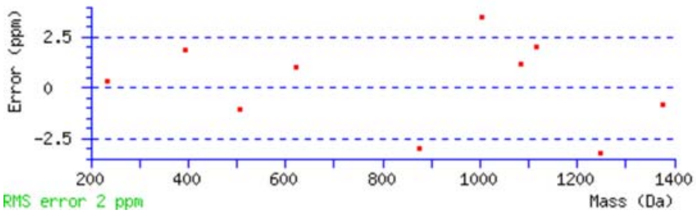

NCBI **BLAST** search of TIEELQQKILCSK
(Parameters: blastp, nr protein database, expect=20000, no filter, PAM30)
Other BLAST web gateways

All matches to this query

Score	Mr(calc)	Delta	Sequence
72.1	1588.8494	0.0004	TIEELQQKILCSK

Mascot: http://www.matrixscience.com/

MS/MS spectrums and fragmentation profile of K84 in Urea, SDSI and SDSII extracted hair shaft

Mascot Search Results

Peptide View

MS/MS Fragmentation of **LGLDIEIATYR**
Found in **ch12u_Q9NSB2|KRT84_HUMAN** in **uni_human**, Keratin, type II cuticular Hb4 OS=Homo sapiens GN=KRT84 PE=2 SV=2

Match to Query 10372: 1262.686988 from(632.350770,2+) intensity(2488661.0000) rtinseconds(2670) scans(14614) index(42248)
Title: 160219_Sunil_SDSI_A_Spectrum096833_scans__14614_RTINSECONDS=2670
Data file L:\\QE_2016\\160219_Sunil_KAP_LKC\\TMgf\\T\\T160219_Sunil_SDSI_A.mgf

Click mouse within plot area to zoom in by factor of two about that point
Or, Plot from 0 to 1200 Da Full range
Label all possible matches ○ Label matches used for scoring ◉
Show Y-axis ☐

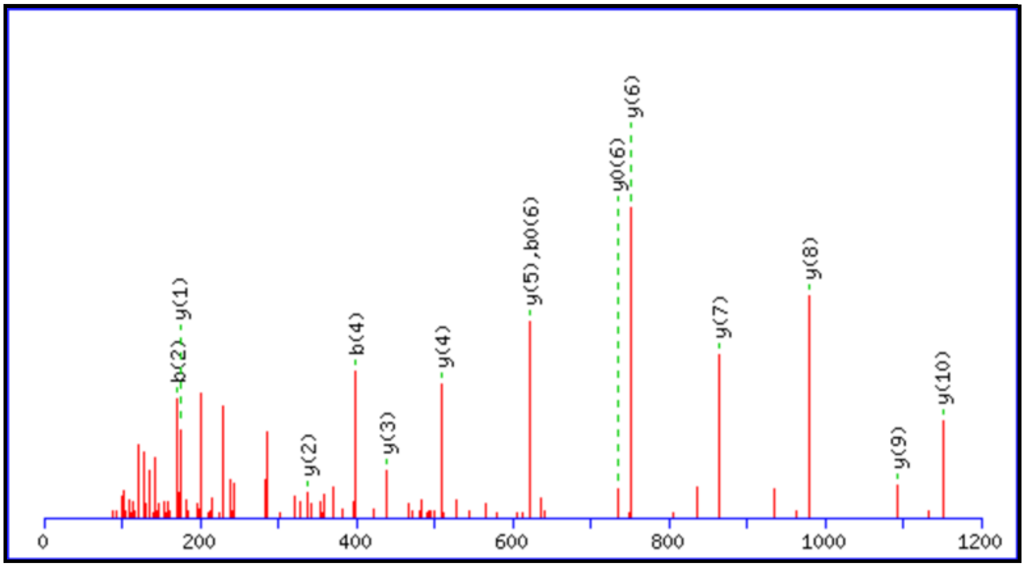

Monoisotopic mass of neutral peptide Mr(calc): 1262.6870
Fixed modifications: Carbamidomethyl (C) (apply to specified residues or termini only)
Ions Score: 89 **Expect:** 1.3e-007
Matches : 14/90 fragment ions using 20 most intense peaks (help)

#	b	b^{++}	b^0	b^{0++}	Seq.	y	y^{++}	y^*	y^{*++}	y^0	y^{0++}	#
1	114.0913	57.5493			L							11
2	**171.1128**	86.0600			G	***1150.6103***	575.8088	1133.5837	567.2955	1132.5997	566.8035	10
3	284.1969	142.6021			L	***1093.5888***	547.2980	1076.5623	538.7848	1075.5782	538.2928	9
4	**399.2238**	200.1155	381.2132	191.1103	D	***980.5047***	490.7560	963.4782	482.2427	962.4942	481.7507	8
5	512.3079	256.6576	494.2973	247.6523	I	***865.4778***	433.2425	848.4512	424.7293	847.4672	424.2373	7
6	641.3505	321.1789	623.3399	312.1736	E	***752.3937***	376.7005	735.3672	368.1872	734.3832	367.6952	6
7	754.4345	377.7209	736.4240	368.7156	I	***623.3511***	312.1792	606.3246	303.6659	605.3406	303.1739	5
8	825.4716	413.2395	807.4611	404.2342	A	***510.2671***	255.6372	493.2405	247.1239	492.2565	246.6319	4
9	926.5193	463.7633	908.5088	454.7580	T	***439.2300***	220.1186	422.2034	211.6053	421.2194	211.1133	3

10	1089.5827	545.2950	1071.5721	536.2897	Y	*338.1823*	169.5948	321.1557	161.0815			2
11					R	*175.1190*	88.0631	158.0924	79.5498			1

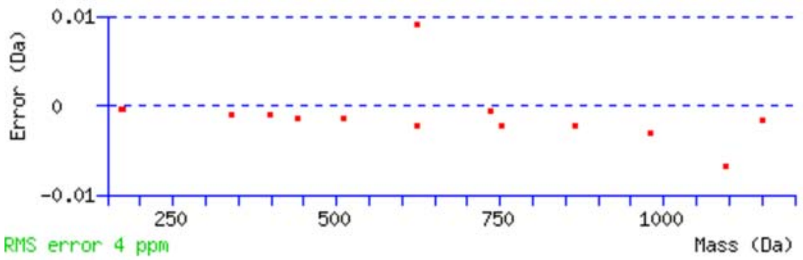

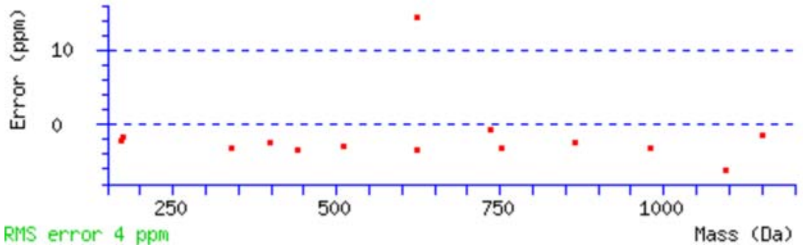

NCBI **BLAST** search of LGLDIEIATYR
(Parameters: blastp, nr protein database, expect=20000, no filter, PAM30)
Other BLAST web gateways

All matches to this query

Score	Mr(calc)	Delta	Sequence
88.9	1262.6870	-0.0000	LGLDIEIATYR
37.1	1262.6870	-0.0000	LALDVEIATYR
22.2	1262.6870	-0.0001	IAVDELFTSLR
8.0	1262.6805	0.0065	VALLIGNMNYR
5.7	1262.6758	0.0112	EVELIGASYIGL
5.3	1262.6983	-0.0113	IGAQPVEIPPSR
1.0	1262.6870	-0.0001	ISLGFELTVER
1.0	1262.6870	-0.0001	LSLGFELTVER
0.7	1262.6983	-0.0113	LVAAKAFTESAR
0.4	1262.6818	0.0051	LARMRGFWAR

Mascot: http://www.matrixscience.com/

Mascot Search Results

Peptide View

MS/MS Fragmentation of **LGLDIEIATYR**
Found in **ch12u_Q9NSB2|KRT84_HUMAN** in **uni_human**, Keratin, type II cuticular Hb4 OS=Homo sapiens GN=KRT84 PE=2 SV=2

Match to Query 10372: 1262.686988 from(632.350770,2+) intensity(2488661.0000) rtinseconds(2670) scans(14614) index(42248)
Title: 160219_Sunil_SDSI_A_Spectrum096833_scans__14614_RTINSECONDS=2670
Data file L:\\QE_2016\\160219_Sunil_KAP_LKC\\TMgf\\T\\T160219_Sunil_SDSI_A.mgf

Click mouse within plot area to zoom in by factor of two about that point
Or, Plot from 0 to 1200 Da Full range
Label all possible matches ○ Label matches used for scoring ◉
Show Y-axis ☐

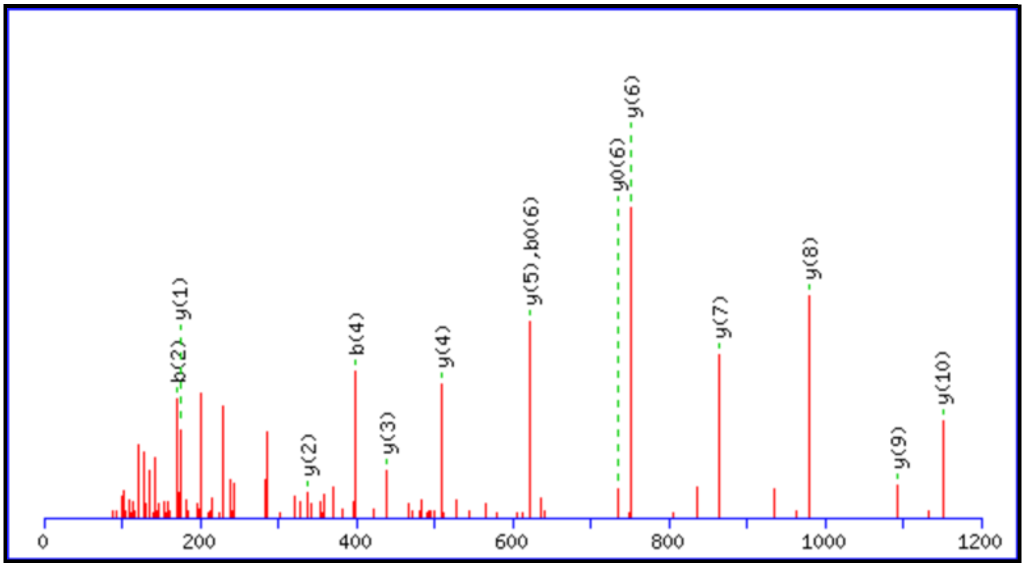

Monoisotopic mass of neutral peptide Mr(calc): 1262.6870
Fixed modifications: Carbamidomethyl (C) (apply to specified residues or termini only)
Ions Score: 89 **Expect:** 1.3e-007
Matches : 14/90 fragment ions using 20 most intense peaks (help)

#	b	b++	b0	b0++	Seq.	y	y++	y*	y*++	y0	y0++	#
1	114.0913	57.5493			L							11
2	**171.1128**	86.0600			G	***1150.6103***	575.8088	1133.5837	567.2955	1132.5997	566.8035	10
3	284.1969	142.6021			L	***1093.5888***	547.2980	1076.5623	538.7848	1075.5782	538.2928	9
4	**399.2238**	200.1155	381.2132	191.1103	D	***980.5047***	490.7560	963.4782	482.2427	962.4942	481.7507	8
5	512.3079	256.6576	494.2973	247.6523	I	***865.4778***	433.2425	848.4512	424.7293	847.4672	424.2373	7
6	641.3505	321.1789	623.3399	312.1736	E	***752.3937***	376.7005	735.3672	368.1872	734.3832	367.6952	6
7	754.4345	377.7209	736.4240	368.7156	I	***623.3511***	312.1792	606.3246	303.6659	605.3406	303.1739	5
8	825.4716	413.2395	807.4611	404.2342	A	***510.2671***	255.6372	493.2405	247.1239	492.2565	246.6319	4
9	926.5193	463.7633	908.5088	454.7580	T	***439.2300***	220.1186	422.2034	211.6053	421.2194	211.1133	3

10	1089.5827	545.2950	1071.5721	536.2897	Y	338.1823	169.5948	321.1557	161.0815			2
11					R	175.1190	88.0631	158.0924	79.5498			1

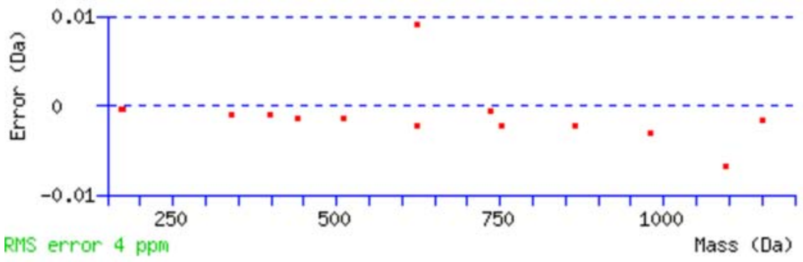

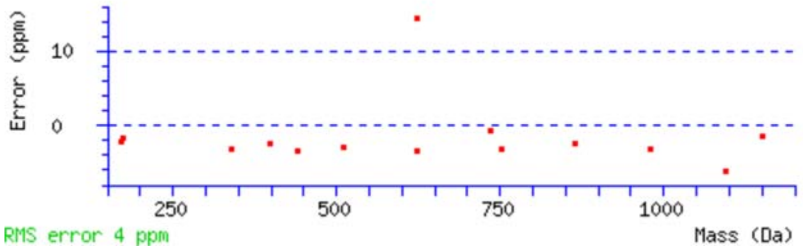

NCBI **BLAST** search of LGLDIEIATYR
(Parameters: blastp, nr protein database, expect=20000, no filter, PAM30)
Other BLAST web gateways

All matches to this query

Score	Mr(calc)	Delta	Sequence
88.9	1262.6870	-0.0000	LGLDIEIATYR
37.1	1262.6870	-0.0000	LALDVEIATYR
22.2	1262.6870	-0.0001	IAVDELFTSLR
8.0	1262.6805	0.0065	VALLIGNMNYR
5.7	1262.6758	0.0112	EVELIGASYIGL
5.3	1262.6983	-0.0113	IGAQPVEIPPSR
1.0	1262.6870	-0.0001	ISLGFELTVER
1.0	1262.6870	-0.0001	LSLGFELTVER
0.7	1262.6983	-0.0113	LVAAKAFTESAR
0.4	1262.6818	0.0051	LARMRGFWAR

Mascot: http://www.matrixscience.com/

Mascot Search Results

Peptide View

MS/MS Fragmentation of **LGLDIEIATYRR**
Found in **ch12u_Q9NSB2|KRT84_HUMAN** in **uni_human**, Keratin, type II cuticular Hb4 OS=Homo sapiens GN=KRT84 PE=2 SV=2

Match to Query 14971: 1418.786848 from(710.400700,2+) intensity(6355424.5000) rtinseconds(2300) scans(12635) index(25348)
Title: 160219_Sunil_SDSI_A_Spectrum078654_scans__12635_RTINSECONDS=2300
Data file L:\\QE_2016\\160219_Sunil_KAP_LKC\\TMgf\\T\\T160219_Sunil_SDSI_A.mgf

Click mouse within plot area to zoom in by factor of two about that point
Or, Plot from 100 to 1500 Da Full range
Label all possible matches ○ Label matches used for scoring ◉
Show Y-axis ☐

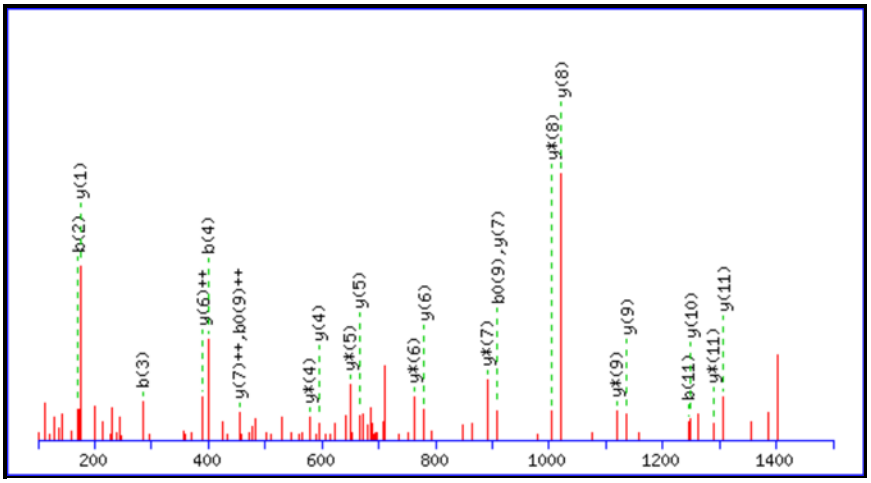

Monoisotopic mass of neutral peptide Mr(calc): 1418.7881
Fixed modifications: Carbamidomethyl (C) (apply to specified residues or termini only)
Ions Score: 50 **Expect:** 0.00083
Matches : 24/100 fragment ions using 50 most intense peaks (help)

#	b	b^{++}	b*	b^{*++}	b^0	b^{0++}	Seq.	y	y^{++}	y*	y^{*++}	y^0	y^{0++}	#
1	114.0913	57.5493					L							12
2	**171.1128**	86.0600					G	***1306.7114***	653.8593	1289.6848	645.3461	1288.7008	644.8540	11
3	**284.1969**	142.6021					L	***1249.6899***	625.3486	1232.6634	616.8353	1231.6793	616.3433	10
4	**399.2238**	200.1155			381.2132	191.1103	D	***1136.6058***	568.8066	1119.5793	560.2933	1118.5953	559.8013	9
5	512.3079	256.6576			494.2973	247.6523	I	***1021.5789***	511.2931	1004.5524	502.7798	1003.5683	502.2878	8
6	641.3505	321.1789			623.3399	312.1736	E	***908.4948***	454.7511	891.4683	446.2378	890.4843	445.7458	7
7	754.4345	377.7209			736.4240	368.7156	I	***779.4522***	390.2298	762.4257	381.7165	761.4417	381.2245	6
8	825.4716	413.2395			807.4611	404.2342	A	***666.3682***	333.6877	649.3416	325.1745	648.3576	324.6824	5
9	926.5193	463.7633			908.5088	454.7580	T	***595.3311***	298.1692	578.3045	289.6559	577.3205	289.1639	4
10	1089.5827	545.2950			1071.5721	536.2897	Y	494.2834	247.6453	477.2568	239.1321			3
11	**1245.6838**	623.3455	1228.6572	614.8322	1227.6732	614.3402	R	331.2201	166.1137	314.1935	157.6004			2
12							R	***175.1190***	88.0631	158.0924	79.5498			1

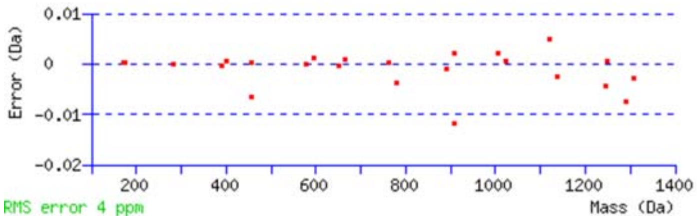

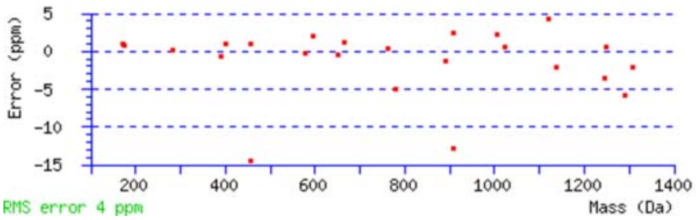

NCBI **BLAST** search of LGLDIEIATYRR
(Parameters: blastp, nr protein database, expect=20000, no filter, PAM30)
Other BLAST web gateways

All matches to this query

Score	Mr(calc)	Delta	Sequence
50.5	1418.7881	-0.0013	LGLDIEIATYRR
12.9	1418.7881	-0.0013	LGLLDRIVNYSR
8.4	1418.7881	-0.0013	LQLRSLQYLER
8.4	1418.7769	0.0099	LLGNTFVALSDLR
0.3	1418.7881	-0.0013	LLQRRQIFSQK

Mascot: http://www.matrixscience.com/

Mascot Search Results

Peptide View

MS/MS Fragmentation of **FLEQQNKLLETK**
Found in **ch12u_Q9NSB2|KRT84_HUMAN** in **uni_human**, Keratin, type II cuticular Hb4 OS=Homo sapiens GN=KRT84 PE=2 SV=2

Match to Query 17388: 1489.814788 from(745.914670,2+) intensity(3362591.2500) rtinseconds(1617) scans(8502) index(6357)
Title: 160219_Sunil_SDSI_A_Spectrum058465_scans__8502_RTINSECONDS=1617
Data file L:\\QE_2016\\160219_Sunil_KAP_LKC\\TMgf\\T\\T160219_Sunil_SDSI_A.mgf

Click mouse within plot area to zoom in by factor of two about that point
Or, Plot from 100 to 1500 Da Full range
Label all possible matches ○ Label matches used for scoring ◉
Show Y-axis □

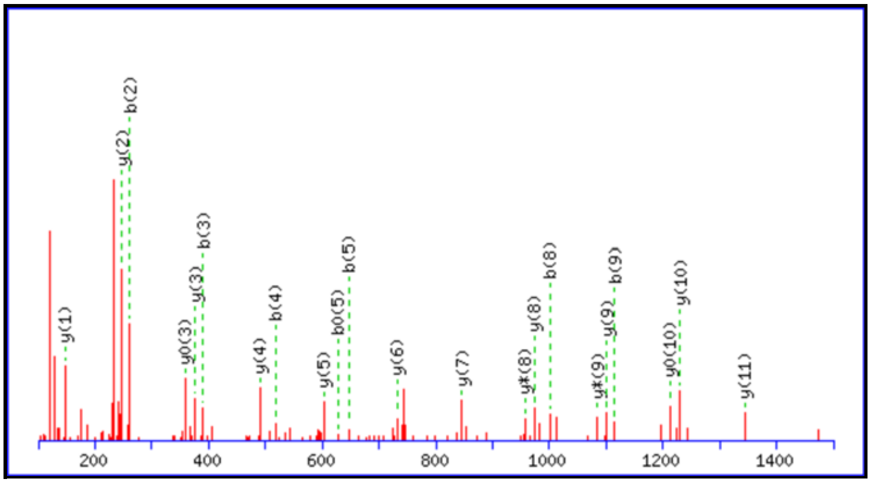

Monoisotopic mass of neutral peptide Mr(calc): 1489.8140
Fixed modifications: Carbamidomethyl (C) (apply to specified residues or termini only)
Ions Score: 85 **Expect:** 3.7e-007
Matches : 22/120 fragment ions using 39 most intense peaks (help)

#	b	b^{++}	b^{*}	b^{*++}	b^{0}	b^{0++}	Seq.	y	y^{++}	y^{*}	y^{*++}	y^{0}	y^{0++}	#
1	148.0757	74.5415					F							12
2	**261.1598**	131.0835					L	***1343.7529***	672.3801	1326.7264	663.8668	1325.7423	663.3748	11
3	**390.2023**	195.6048			372.1918	186.5995	E	***1230.6688***	615.8381	1213.6423	607.3248	1212.6583	606.8328	10
4	**518.2609**	259.6341	501.2344	251.1208	500.2504	250.6288	Q	***1101.6263***	551.3168	1084.5997	542.8035	1083.6157	542.3115	9
5	**646.3195**	323.6634	629.2930	315.1501	628.3089	314.6581	Q	***973.5677***	487.2875	956.5411	478.7742	955.5571	478.2822	8
6	760.3624	380.6849	743.3359	372.1716	742.3519	371.6796	N	***845.5091***	423.2582	828.4825	414.7449	827.4985	414.2529	7
7	888.4574	444.7323	871.4308	436.2191	870.4468	435.7271	K	***731.4662***	366.2367	714.4396	357.7234	713.4556	357.2314	6
8	**1001.5415**	501.2744	984.5149	492.7611	983.5309	492.2691	L	***603.3712***	302.1892	586.3447	293.6760	585.3606	293.1840	5
9	**1114.6255**	557.8164	1097.5990	549.3031	1096.6150	548.8111	L	***490.2871***	245.6472	473.2606	237.1339	472.2766	236.6419	4
10	1243.6681	622.3377	1226.6416	613.8244	1225.6575	613.3324	E	***377.2031***	189.1052	360.1765	180.5919	359.1925	180.0999	3
11	1344.7158	672.8615	1327.6892	664.3483	1326.7052	663.8563	T	***248.1605***	124.5839	231.1339	116.0706	230.1499	115.5786	2
12							K	***147.1128***	74.0600	130.0863	65.5468			1

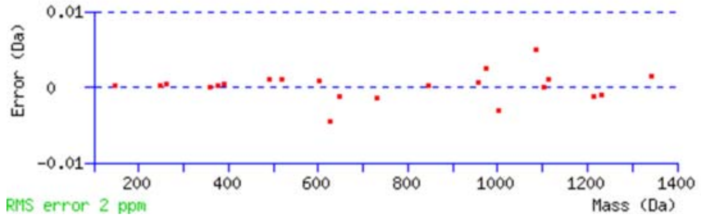

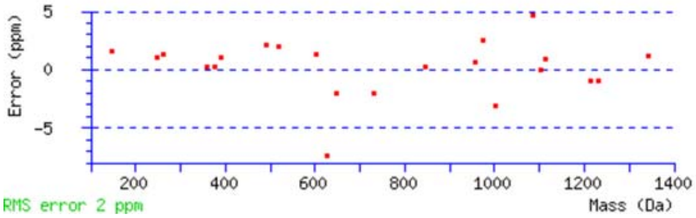

NCBI **BLAST** search of FLEQQNKLLETK
(Parameters: blastp, nr protein database, expect=20000, no filter, PAM30)
Other BLAST web gateways

All matches to this query

Score	Mr(calc)	Delta	Sequence
84.9	1489.8140	0.0008	FLEQQNKLLETK
0.1	1489.8001	0.0147	YEKPQELSGRKR

Mascot: http://www.matrixscience.com/

Mascot Search Results

Peptide View

MS/MS Fragmentation of **FLEQQNKLLETK**
Found in **ch12u_Q9NSB2|KRT84_HUMAN** in **uni_human**, Keratin, type II cuticular Hb4 OS=Homo sapiens GN=KRT84 PE=2 SV=2

Match to Query 17363: 1489.810768 from(745.912660,2+) intensity(1739470.6250) rtinseconds(1482) scans(7866) index(21167)
Title: 160219_Sunil_SDSI_A_Spectrum074471_scans__7866_RTINSECONDS=1482
Data file L:\\QE_2016\\160219_Sunil_KAP_LKC\\TMgf\\T\\T160219_Sunil_SDSI_A.mgf

Click mouse within plot area to zoom in by factor of two about that point
Or, Plot from 100 to 1400 Da Full range
Label all possible matches ○ Label matches used for scoring ◉
Show Y-axis ☐

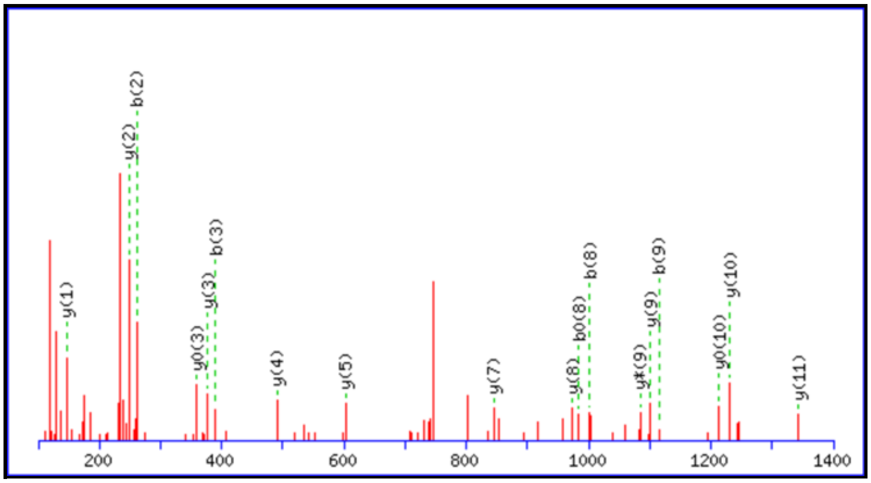

Monoisotopic mass of neutral peptide Mr(calc): 1489.8140
Fixed modifications: Carbamidomethyl (C) (apply to specified residues or termini only)
Ions Score: 72 **Expect:** 7.7e-006
Matches : 18/120 fragment ions using 33 most intense peaks (help)

#	b	b^{++}	b*	b^{*++}	b^0	b^{0++}	Seq.	y	y^{++}	y*	y^{*++}	y^0	y^{0++}	#
1	148.0757	74.5415					F							12
2	**261.1598**	131.0835					L	***1343.7529***	672.3801	1326.7264	663.8668	1325.7423	663.3748	11
3	**390.2023**	195.6048			372.1918	186.5995	E	***1230.6688***	615.8381	1213.6423	607.3248	1212.6583	606.8328	10
4	518.2609	259.6341	501.2344	251.1208	500.2504	250.6288	Q	***1101.6263***	551.3168	1084.5997	542.8035	1083.6157	542.3115	9
5	646.3195	323.6634	629.2930	315.1501	628.3089	314.6581	Q	***973.5677***	487.2875	956.5411	478.7742	955.5571	478.2822	8
6	760.3624	380.6849	743.3359	372.1716	742.3519	371.6796	N	***845.5091***	423.2582	828.4825	414.7449	827.4985	414.2529	7
7	888.4574	444.7323	871.4308	436.2191	870.4468	435.7271	K	731.4662	366.2367	714.4396	357.7234	713.4556	357.2314	6
8	**1001.5415**	501.2744	984.5149	492.7611	983.5309	492.2691	L	***603.3712***	302.1892	586.3447	293.6760	585.3606	293.1840	5
9	**1114.6255**	557.8164	1097.5990	549.3031	1096.6150	548.8111	L	***490.2871***	245.6472	473.2606	237.1339	472.2766	236.6419	4
10	1243.6681	622.3377	1226.6416	613.8244	1225.6575	613.3324	E	***377.2031***	189.1052	360.1765	180.5919	359.1925	180.0999	3
11	1344.7158	672.8615	1327.6892	664.3483	1326.7052	663.8563	T	***248.1605***	124.5839	231.1339	116.0706	230.1499	115.5786	2
12							K	***147.1128***	74.0600	130.0863	65.5468			1

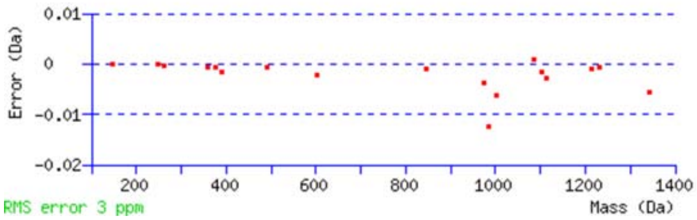

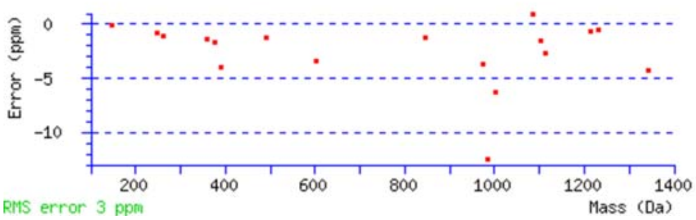

NCBI **BLAST** search of FLEQQNKLLETK
(Parameters: blastp, nr protein database, expect=20000, no filter, PAM30)
Other BLAST web gateways

All matches to this query

Score	Mr(calc)	Delta	Sequence
71.8	1489.8140	-0.0032	FLEQQNKLLETK

Mascot: http://www.matrixscience.com/

Mascot Search Results

Peptide View

MS/MS Fragmentation of **LGLDIEIATYR**
Found in **ch12u_Q9NSB2|KRT84_HUMAN** in **uni_human**, Keratin, type II cuticular Hb4 OS=Homo sapiens GN=KRT84 PE=2 SV=2

Match to Query 10882: 1262.687228 from(632.350890,2+) intensity(2016767.1250) rtinseconds(2661) scans(14659) index(26782)
Title: 160219_Sunil_SDSII_A_Spectrum079345_scans__14659_RTINSECONDS=2661
Data file L:\\QE_2016\\160219_Sunil_KAP_LKC\\TMgf\\T\\T160219_Sunil_SDSII_A.mgf

Click mouse within plot area to zoom in by factor of two about that point
Or, Plot from 100 to 1200 Da Full range
Label all possible matches ○ Label matches used for scoring ◉
Show Y-axis ☐

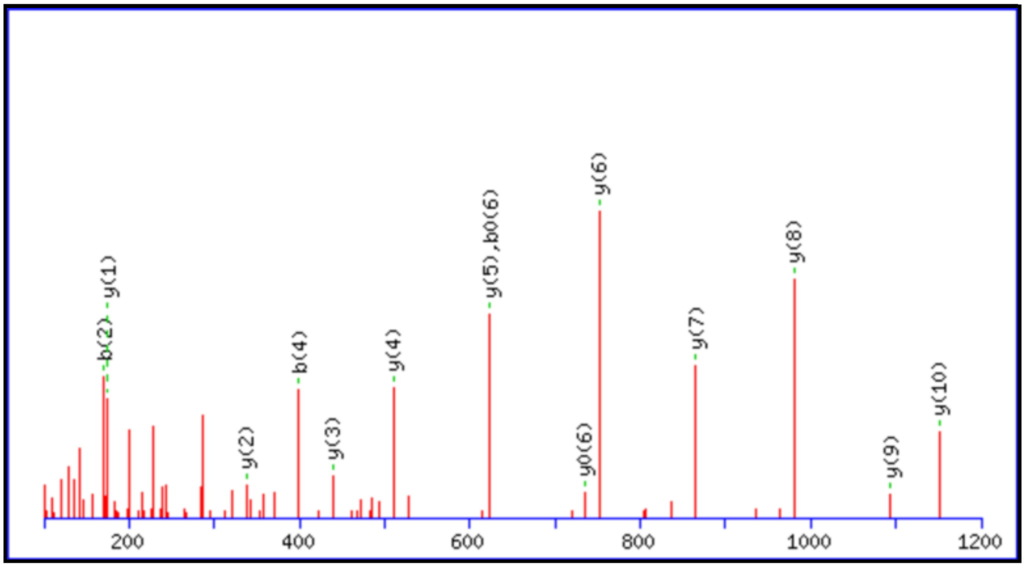

Monoisotopic mass of neutral peptide Mr(calc): 1262.6870
Fixed modifications: Carbamidomethyl (C) (apply to specified residues or termini only)
Ions Score: 89 **Expect:** 1.3e-007
Matches : 14/90 fragment ions using 20 most intense peaks (help)

#	b	b^{++}	b^{0}	b^{0++}	Seq.	y	y^{++}	y^{*}	y^{*++}	y^{0}	y^{0++}	#
1	114.0913	57.5493			L							11
2	**171.1128**	86.0600			G	***1150.6103***	575.8088	1133.5837	567.2955	1132.5997	566.8035	10
3	284.1969	142.6021			L	***1093.5888***	547.2980	1076.5623	538.7848	1075.5782	538.2928	9
4	**399.2238**	200.1155	381.2132	191.1103	D	***980.5047***	490.7560	963.4782	482.2427	962.4942	481.7507	8
5	512.3079	256.6576	494.2973	247.6523	I	***865.4778***	433.2425	848.4512	424.7293	847.4672	424.2373	7
6	641.3505	321.1789	623.3399	312.1736	E	***752.3937***	376.7005	735.3672	368.1872	734.3832	367.6952	6
7	754.4345	377.7209	736.4240	368.7156	I	***623.3511***	312.1792	606.3246	303.6659	605.3406	303.1739	5
8	825.4716	413.2395	807.4611	404.2342	A	***510.2671***	255.6372	493.2405	247.1239	492.2565	246.6319	4
9	926.5193	463.7633	908.5088	454.7580	T	***439.2300***	220.1186	422.2034	211.6053	421.2194	211.1133	3

**10**	1089.5827	545.2950	1071.5721	536.2897	**Y**	*338.1823*	169.5948	321.1557	161.0815			**2**
**11**					**R**	*175.1190*	88.0631	158.0924	79.5498			**1**

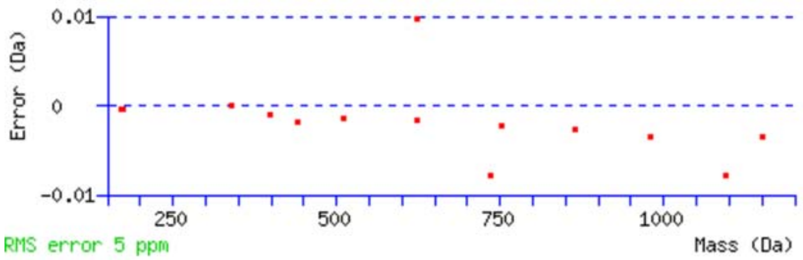

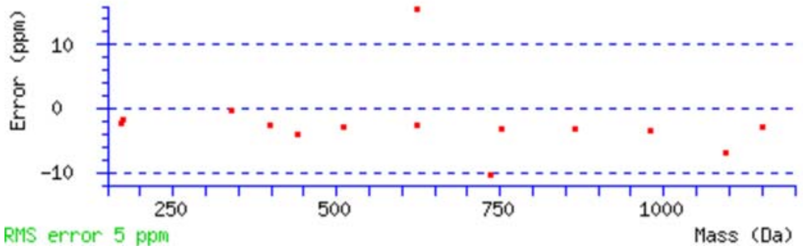

NCBI **BLAST** search of LGLDIEIATYR
(Parameters: blastp, nr protein database, expect=20000, no filter, PAM30)
Other BLAST web gateways

All matches to this query

Score	Mr(calc)	Delta	Sequence
89.3	1262.6870	0.0002	LGLDIEIATYR
37.5	1262.6870	0.0002	LALDVEIATYR
21.1	1262.6870	0.0002	IAVDELFTSLR
8.7	1262.6805	0.0067	VALLIGNMNYR
5.7	1262.6983	-0.0110	IGAQPVEIPPSR
5.2	1262.6983	-0.0111	IGDFGLATVKSR
1.9	1262.6884	-0.0011	GLVQRWRYGK
1.1	1262.6983	-0.0110	LVAAKAFTESAR
0.5	1261.6819	1.0053	DIGIWYNILR

Mascot: http://www.matrixscience.com/

Mascot Search Results

Peptide View

MS/MS Fragmentation of **FLEQQNKLLETK**
Found in **ch12u_Q9NSB2|KRT84_HUMAN** in **uni_human**, Keratin, type II cuticular Hb4 OS=Homo sapiens GN=KRT84 PE=2 SV=2

Match to Query 18174: 1489.811248 from(745.912900,2+) intensity(1430451.5000) rtinseconds(1473) scans(7784) index(20869)
Title: 160219_Sunil_SDSII_A_Spectrum073430_scans__7784_RTINSECONDS=1473
Data file L:\\QE_2016\\160219_Sunil_KAP_LKC\\TMgf\\T\\T160219_Sunil_SDSII_A.mgf

Click mouse within plot area to zoom in by factor of two about that point
Or, Plot from 100 to 1500 Da Full range
Label all possible matches ○ Label matches used for scoring ◉
Show Y-axis ☐

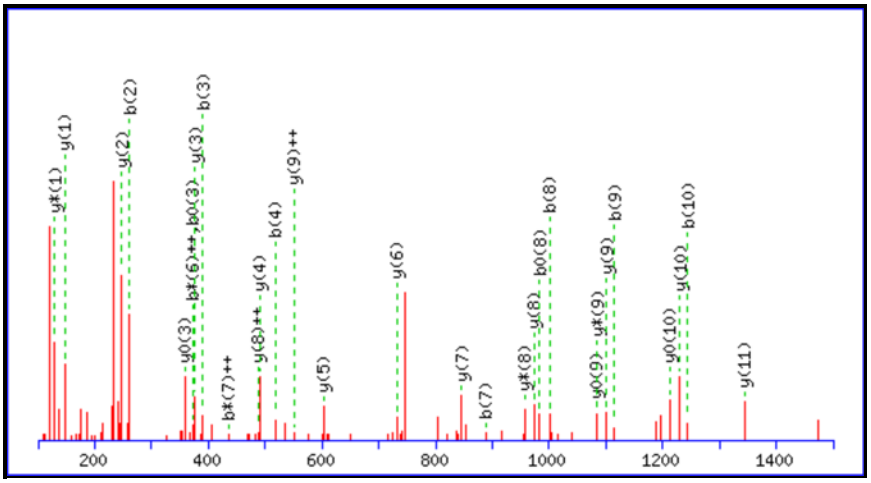

Monoisotopic mass of neutral peptide Mr(calc): 1489.8140
Fixed modifications: Carbamidomethyl (C) (apply to specified residues or termini only)
Ions Score: 84 **Expect:** 4.5e-007
Matches : 30/120 fragment ions using 48 most intense peaks (help)

#	b	b^{++}	b*	b*$^{++}$	b^{0}	b^{0++}	Seq.	y	y^{++}	y*	y*$^{++}$	y^{0}	y^{0++}	#
1	148.0757	74.5415					F							12
2	**261.1598**	131.0835					L	***1343.7529***	672.3801	1326.7264	663.8668	1325.7423	663.3748	11
3	**390.2023**	195.6048			372.1918	186.5995	E	***1230.6688***	615.8381	1213.6423	607.3248	1212.6583	606.8328	10
4	**518.2609**	259.6341	501.2344	251.1208	500.2504	250.6288	Q	***1101.6263***	551.3168	1084.5997	542.8035	1083.6157	542.3115	9
5	646.3195	323.6634	629.2930	315.1501	628.3089	314.6581	Q	***973.5677***	487.2875	956.5411	478.7742	955.5571	478.2822	8
6	760.3624	380.6849	743.3359	372.1716	742.3519	371.6796	N	***845.5091***	423.2582	828.4825	414.7449	827.4985	414.2529	7
7	**888.4574**	444.7323	871.4308	436.2191	870.4468	435.7271	K	***731.4662***	366.2367	714.4396	357.7234	713.4556	357.2314	6
8	**1001.5415**	501.2744	984.5149	492.7611	983.5309	492.2691	L	***603.3712***	302.1892	586.3447	293.6760	585.3606	293.1840	5
9	**1114.6255**	557.8164	1097.5990	549.3031	1096.6150	548.8111	L	***490.2871***	245.6472	473.2606	237.1339	472.2766	236.6419	4
10	**1243.6681**	622.3377	1226.6416	613.8244	1225.6575	613.3324	E	***377.2031***	189.1052	360.1765	180.5919	359.1925	180.0999	3
11	1344.7158	672.8615	1327.6892	664.3483	1326.7052	663.8563	T	***248.1605***	124.5839	231.1339	116.0706	230.1499	115.5786	2
12							K	***147.1128***	74.0600	130.0863	65.5468			1

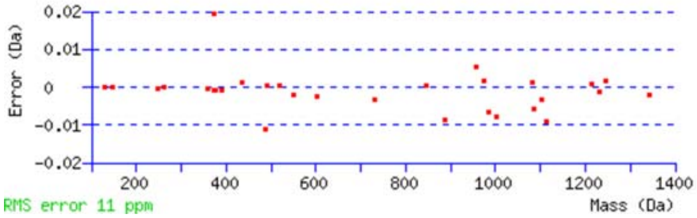

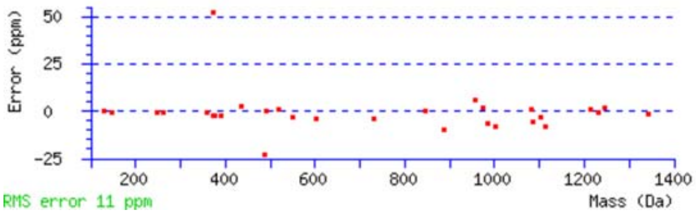

NCBI **BLAST** search of FLEQQNKLLETK
(Parameters: blastp, nr protein database, expect=20000, no filter, PAM30)
Other BLAST web gateways

All matches to this query

Score	Mr(calc)	Delta	Sequence
84.1	1489.8140	-0.0028	FLEQQNKLLETK

Mascot: http://www.matrixscience.com/

Mascot Search Results

Peptide View

MS/MS Fragmentation of **LGLDIEIATYRR**
Found in **ch12u_Q9NSB2|KRT84_HUMAN** in **uni_human**, Keratin, type II cuticular Hb4 OS=Homo sapiens GN=KRT84 PE=2 SV=2

Match to Query 15708: 1418.788572 from(473.936800,3+) intensity(9031432.0000) rtinseconds(2527) scans(13593) index(10742)
Title: 160219_Sunil_SDSII_A_Spectrum062071_scans__13593_RTINSECONDS=2527
Data file L:\\QE_2016\\160219_Sunil_KAP_LKC\\TMgf\\T\\T160219_Sunil_SDSII_A.mgf

Click mouse within plot area to zoom in by factor of two about that point
Or, Plot from 100 to 1400 Da Full range
Label all possible matches ○ Label matches used for scoring ◉
Show Y-axis ☐

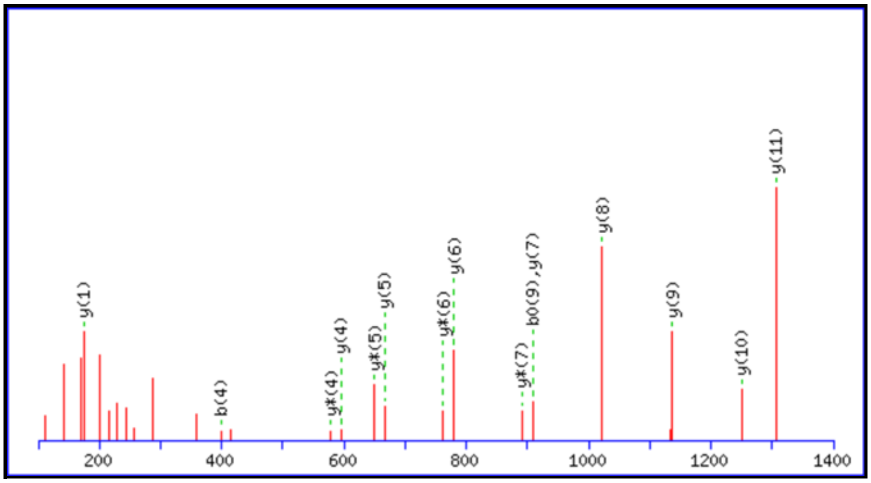

Monoisotopic mass of neutral peptide Mr(calc): 1418.7881
Fixed modifications: Carbamidomethyl (C) (apply to specified residues or termini only)
Ions Score: 77 **Expect:** 1.9e-006
Matches : 15/100 fragment ions using 20 most intense peaks (help)

#	b	b++	b*	b*++	b0	b0++	Seq.	y	y++	y*	y*++	y0	y0++	#
1	114.0913	57.5493					L							12
2	171.1128	86.0600					G	***1306.7114***	653.8593	1289.6848	645.3461	1288.7008	644.8540	11
3	284.1969	142.6021					L	***1249.6899***	625.3486	1232.6634	616.8353	1231.6793	616.3433	10
4	**399.2238**	200.1155			381.2132	191.1103	D	***1136.6058***	568.8066	1119.5793	560.2933	1118.5953	559.8013	9
5	512.3079	256.6576			494.2973	247.6523	I	***1021.5789***	511.2931	1004.5524	502.7798	1003.5683	502.2878	8
6	641.3505	321.1789			623.3399	312.1736	E	***908.4948***	454.7511	891.4683	446.2378	890.4843	445.7458	7
7	754.4345	377.7209			736.4240	368.7156	I	***779.4522***	390.2298	762.4257	381.7165	761.4417	381.2245	6
8	825.4716	413.2395			807.4611	404.2342	A	***666.3682***	333.6877	649.3416	325.1745	648.3576	324.6824	5
9	926.5193	463.7633			908.5088	454.7580	T	***595.3311***	298.1692	578.3045	289.6559	577.3205	289.1639	4
10	1089.5827	545.2950			1071.5721	536.2897	Y	494.2834	247.6453	477.2568	239.1321			3
11	1245.6838	623.3455	1228.6572	614.8322	1227.6732	614.3402	R	331.2201	166.1137	314.1935	157.6004			2
12							R	***175.1190***	88.0631	158.0924	79.5498			1

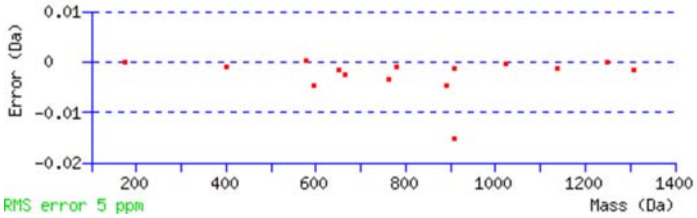

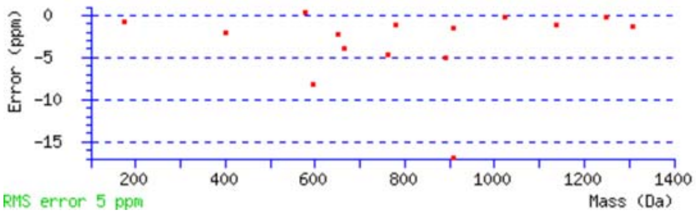

NCBI **BLAST** search of LGLDIEIATYRR
(Parameters: blastp, nr protein database, expect=20000, no filter, PAM30)
Other BLAST web gateways

All matches to this query

Score	Mr(calc)	Delta	Sequence
76.9	1418.7881	0.0004	LGLDIEIATYRR
17.2	1418.7881	0.0004	LGLLDRIVNYSR
16.4	1418.7769	0.0117	LLGNTFVALSDLR
8.6	1418.7881	0.0005	LQLRSLQYLER
4.1	1418.7769	0.0117	GLVLQLIQSYQR
1.5	1418.7994	-0.0108	LGPESVPPPKRSR
1.2	1418.7769	0.0117	LYVALKELGEER
0.6	1418.7769	0.0117	INELLKYIEER
0.1	1418.7881	0.0004	IKQFTLEEKQR
0.1	1418.7769	0.0117	LIQFLQASITER

Mascot: http://www.matrixscience.com/

Mascot Search Results

Peptide View

MS/MS Fragmentation of **FLEQQNKLLETK**
Found in **ch12u_Q9NSB2|KRT84_HUMAN** in **uni_human**, Keratin, type II cuticular Hb4 OS=Homo sapiens GN=KRT84 PE=2 SV=2

Match to Query 18174: 1489.811248 from(745.912900,2+) intensity(1430451.5000) rtinseconds(1473) scans(7784) index(20869)
Title: 160219_Sunil_SDSII_A_Spectrum073430_scans__7784_RTINSECONDS=1473
Data file L:\\QE_2016\\160219_Sunil_KAP_LKC\\TMgf\\T\\T160219_Sunil_SDSII_A.mgf

Click mouse within plot area to zoom in by factor of two about that point
Or, Plot from 100 to 1500 Da Full range
Label all possible matches ○ Label matches used for scoring ◉
Show Y-axis ☐

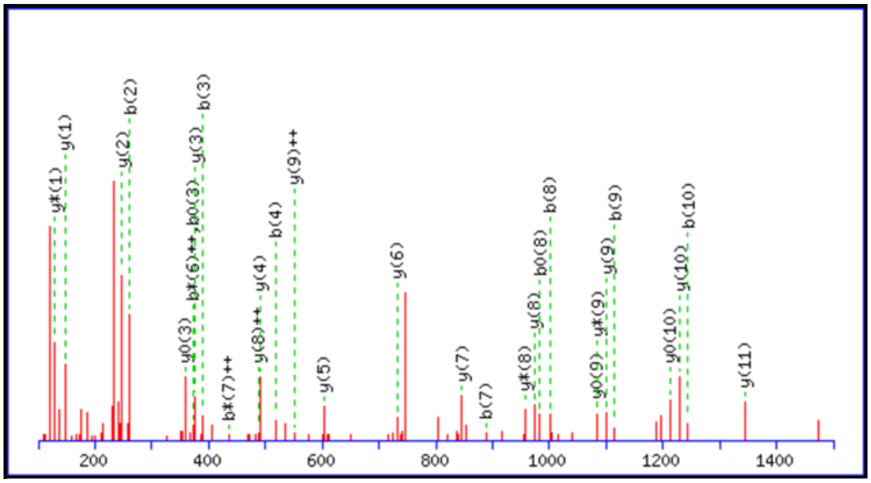

Monoisotopic mass of neutral peptide Mr(calc): 1489.8140
Fixed modifications: Carbamidomethyl (C) (apply to specified residues or termini only)
Ions Score: 84 **Expect:** 4.5e-007
Matches : 30/120 fragment ions using 48 most intense peaks (help)

#	b	b^{++}	b*	b^{*++}	b^0	b^{0++}	Seq.	y	y^{++}	y*	y^{*++}	y^0	y^{0++}	#
1	148.0757	74.5415					F							12
2	**261.1598**	131.0835					L	***1343.7529***	672.3801	1326.7264	663.8668	1325.7423	663.3748	11
3	**390.2023**	195.6048			372.1918	186.5995	E	***1230.6688***	615.8381	1213.6423	607.3248	1212.6583	606.8328	10
4	**518.2609**	259.6341	501.2344	251.1208	500.2504	250.6288	Q	***1101.6263***	551.3168	1084.5997	542.8035	1083.6157	542.3115	9
5	646.3195	323.6634	629.2930	315.1501	628.3089	314.6581	Q	***973.5677***	487.2875	956.5411	478.7742	955.5571	478.2822	8
6	760.3624	380.6849	743.3359	372.1716	742.3519	371.6796	N	***845.5091***	423.2582	828.4825	414.7449	827.4985	414.2529	7
7	**888.4574**	444.7323	871.4308	436.2191	870.4468	435.7271	K	***731.4662***	366.2367	714.4396	357.7234	713.4556	357.2314	6
8	**1001.5415**	501.2744	984.5149	492.7611	983.5309	492.2691	L	***603.3712***	302.1892	586.3447	293.6760	585.3606	293.1840	5
9	**1114.6255**	557.8164	1097.5990	549.3031	1096.6150	548.8111	L	***490.2871***	245.6472	473.2606	237.1339	472.2766	236.6419	4
10	**1243.6681**	622.3377	1226.6416	613.8244	1225.6575	613.3324	E	***377.2031***	189.1052	360.1765	180.5919	359.1925	180.0999	3
11	1344.7158	672.8615	1327.6892	664.3483	1326.7052	663.8563	T	***248.1605***	124.5839	231.1339	116.0706	230.1499	115.5786	2
12							K	***147.1128***	74.0600	130.0863	65.5468			1

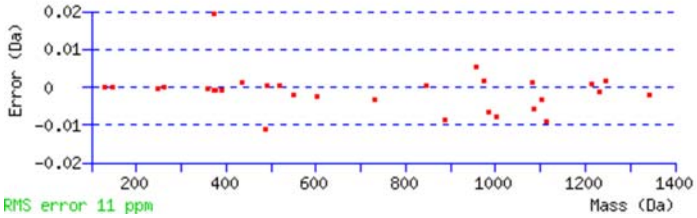

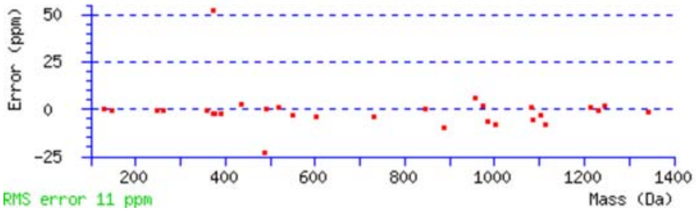

NCBI **BLAST** search of FLEQQNKLLETK
(Parameters: blastp, nr protein database, expect=20000, no filter, PAM30)
Other BLAST web gateways

All matches to this query

Score	Mr(calc)	Delta	Sequence
84.1	1489.8140	-0.0028	FLEQQNKLLETK

Mascot: http://www.matrixscience.com/

Mascot Search Results

Peptide View

MS/MS Fragmentation of **FLEQQNKLLETK**
Found in **ch12u_Q9NSB2|KRT84_HUMAN** in **uni_human**, Keratin, type II cuticular Hb4 OS=Homo sapiens GN=KRT84 PE=2 SV=2

Match to Query 18189: 1489.813902 from(497.611910,3+) intensity(56885288.0000) rtinseconds(1729) scans(8963) index(6747)
Title: 160219_Sunil_SDSII_A_Spectrum058070_scans__8963_RTINSECONDS=1729
Data file L:\\QE_2016\\160219_Sunil_KAP_LKC\\TMgf\\T\\T160219_Sunil_SDSII_A.mgf

Click mouse within plot area to zoom in by factor of two about that point
Or, Plot from 100 to 1400 Da Full range
Label all possible matches ○ Label matches used for scoring ◉
Show Y-axis ☐

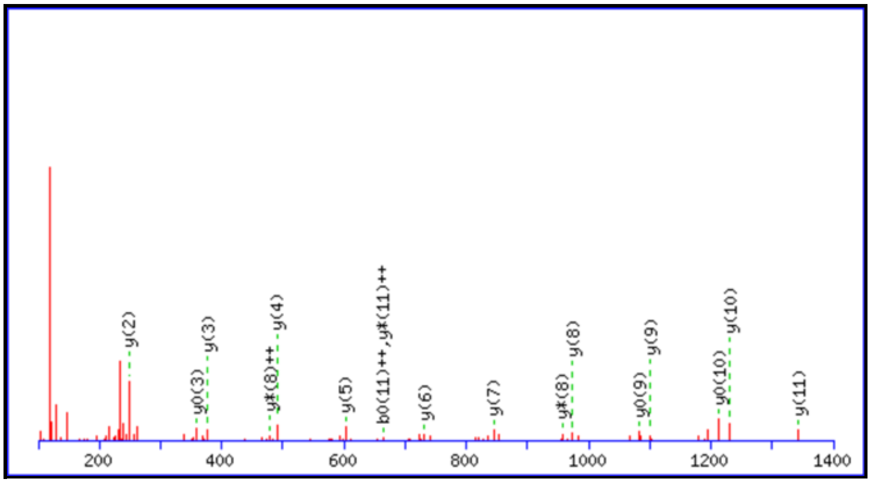

Monoisotopic mass of neutral peptide Mr(calc): 1489.8140
Fixed modifications: Carbamidomethyl (C) (apply to specified residues or termini only)
Ions Score: 76 **Expect:** 2.8e-006
Matches : 17/120 fragment ions using 25 most intense peaks (help)

#	b	b^{++}	b*	b^{*++}	b^0	b^{0++}	Seq.	y	y^{++}	y*	y^{*++}	y^0	y^{0++}	#
1	148.0757	74.5415					F							12
2	261.1598	131.0835					L	***1343.7529***	672.3801	1326.7264	663.8668	1325.7423	663.3748	11
3	390.2023	195.6048			372.1918	186.5995	E	***1230.6688***	615.8381	1213.6423	607.3248	1212.6583	606.8328	10
4	518.2609	259.6341	501.2344	251.1208	500.2504	250.6288	Q	***1101.6263***	551.3168	1084.5997	542.8035	1083.6157	542.3115	9
5	646.3195	323.6634	629.2930	315.1501	628.3089	314.6581	Q	***973.5677***	487.2875	956.5411	478.7742	955.5571	478.2822	8
6	760.3624	380.6849	743.3359	372.1716	742.3519	371.6796	N	***845.5091***	423.2582	828.4825	414.7449	827.4985	414.2529	7
7	888.4574	444.7323	871.4308	436.2191	870.4468	435.7271	K	***731.4662***	366.2367	714.4396	357.7234	713.4556	357.2314	6
8	1001.5415	501.2744	984.5149	492.7611	983.5309	492.2691	L	***603.3712***	302.1892	586.3447	293.6760	585.3606	293.1840	5
9	1114.6255	557.8164	1097.5990	549.3031	1096.6150	548.8111	L	***490.2871***	245.6472	473.2606	237.1339	472.2766	236.6419	4
10	1243.6681	622.3377	1226.6416	613.8244	1225.6575	613.3324	E	***377.2031***	189.1052	360.1765	180.5919	359.1925	180.0999	3
11	1344.7158	672.8615	1327.6892	664.3483	1326.7052	663.8563	T	***248.1605***	124.5839	231.1339	116.0706	230.1499	115.5786	2
12							K	147.1128	74.0600	130.0863	65.5468			1

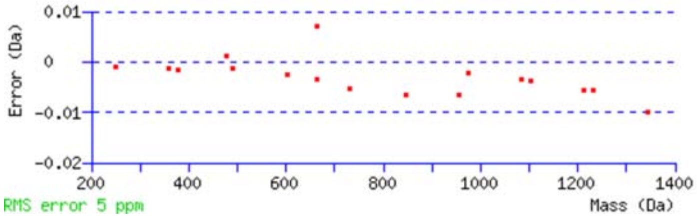

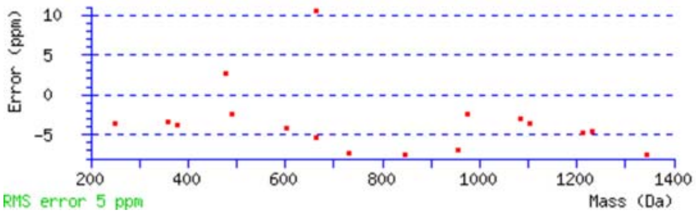

NCBI **BLAST** search of FLEQQNKLLETK
(Parameters: blastp, nr protein database, expect=20000, no filter, PAM30)
Other BLAST web gateways

All matches to this query

Score	Mr(calc)	Delta	Sequence
76.0	1489.8140	-0.0001	FLEQQNKLLETK
0.9	1487.8069	2.0070	NVHRPPRQRDIT

Mascot: http://www.matrixscience.com/

Mascot Search Results

Peptide View

MS/MS Fragmentation of **FLEQQNKLLETK**
Found in **ch12u_Q9NSB2|KRT84_HUMAN** in **uni_human**, Keratin, type II cuticular Hb4 OS=Homo sapiens GN=KRT84 PE=2 SV=2

Match to Query 18255: 1490.799788 from(746.407170,2+) intensity(3397827.2500) rtinseconds(1868) scans(9779) index(7472)
Title: 160219_Sunil_SDSII_A_Spectrum058796_scans__9779_RTINSECONDS=1868
Data file L:\\QE_2016\\160219_Sunil_KAP_LKC\\TMgf\\T\\T160219_Sunil_SDSII_A.mgf

Click mouse within plot area to zoom in by factor of two about that point
Or, Plot from 100 to 1500 Da Full range
Label all possible matches ○ Label matches used for scoring ◉
Show Y-axis ☐

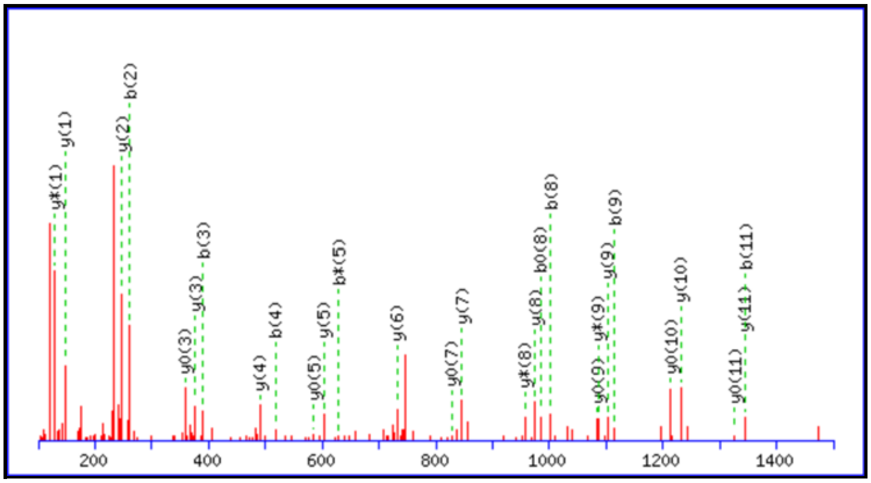

Monoisotopic mass of neutral peptide Mr(calc): 1490.7980
Fixed modifications: Carbamidomethyl (C) (apply to specified residues or termini only)
Variable modifications:
N6 : Deamidated (NQ)
Ions Score: 76 **Expect:** 2.8e-006
Matches : 28/120 fragment ions using 52 most intense peaks (help)

#	b	b++	b*	b*++	b0	b0++	Seq.	y	y++	y*	y*++	y0	y0++	#
1	148.0757	74.5415					F							12
2	**261.1598**	131.0835					L	***1344.7369***	672.8721	1327.7104	664.3588	1326.7264	663.8668	11
3	**390.2023**	195.6048			372.1918	186.5995	E	***1231.6529***	616.3301	1214.6263	607.8168	1213.6423	607.3248	10
4	**518.2609**	259.6341	501.2344	251.1208	500.2504	250.6288	Q	***1102.6103***	551.8088	1085.5837	543.2955	1084.5997	542.8035	9
5	646.3195	323.6634	629.2930	315.1501	628.3089	314.6581	Q	***974.5517***	487.7795	957.5251	479.2662	956.5411	478.7742	8
6	761.3464	381.1769	744.3199	372.6636	743.3359	372.1716	N	***846.4931***	423.7502	829.4666	415.2369	828.4825	414.7449	7
7	889.4414	445.2243	872.4149	436.7111	871.4308	436.2191	K	***731.4662***	366.2367	714.4396	357.7234	713.4556	357.2314	6
8	**1002.5255**	501.7664	985.4989	493.2531	984.5149	492.7611	L	***603.3712***	302.1892	586.3447	293.6760	585.3606	293.1840	5
9	**1115.6095**	558.3084	1098.5830	549.7951	1097.5990	549.3031	L	***490.2871***	245.6472	473.2606	237.1339	472.2766	236.6419	4
10	1244.6521	622.8297	1227.6256	614.3164	1226.6416	613.8244	E	***377.2031***	189.1052	360.1765	180.5919	359.1925	180.0999	3
11	**1345.6998**	673.3535	1328.6733	664.8403	1327.6892	664.3483	T	***248.1605***	124.5839	231.1339	116.0706	230.1499	115.5786	2
12							K	***147.1128***	74.0600	130.0863	65.5468			1

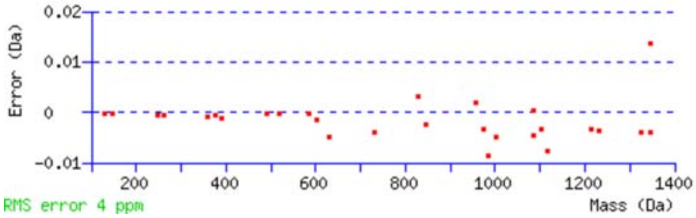

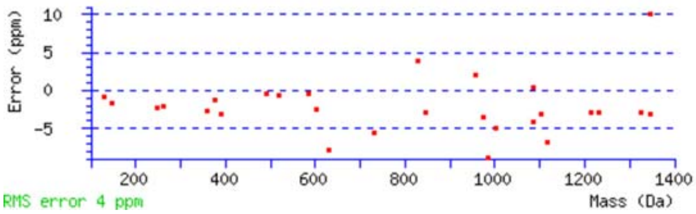

NCBI **BLAST** search of FLEQQNKLLETK
(Parameters: blastp, nr protein database, expect=20000, no filter, PAM30)
Other BLAST web gateways

All matches to this query

Score	Mr(calc)	Delta	Sequence	Site Analysis
76.4	1490.7980	0.0018	FLEQQNKLLETK	Deamidated N6 95.21%
63.3	1490.7980	0.0018	FLEQQNKLLETK	Deamidated Q5 4.63%
48.7	1490.7980	0.0018	FLEQQNKLLETK	Deamidated Q4 0.16%
16.7	1490.7981	0.0017	FQALQSVDILETK	

Mascot: http://www.matrixscience.com/

Mascot Search Results

Peptide View

MS/MS Fragmentation of **VRFLEQQNKLLETK**
Found in **ch12u_Q9NSB2|KRT84_HUMAN** in **uni_human**, Keratin, type II cuticular Hb4 OS=Homo sapiens GN=KRT84 PE=2 SV=2

Match to Query 24723: 1744.982982 from(582.668270,3+) intensity(1018731.4375) rtinseconds(1496) scans(7919) index(20989)
Title: 160219_Sunil_SDSII_A_Spectrum073550_scans__7919_RTINSECONDS=1496
Data file L:\\QE_2016\\160219_Sunil_KAP_LKC\\TMgf\\T\\T160219_Sunil_SDSII_A.mgf

Click mouse within plot area to zoom in by factor of two about that point
Or, Plot from 100 to 1800 Da Full range
Label all possible matches ○ Label matches used for scoring ◉
Show Y-axis ☐

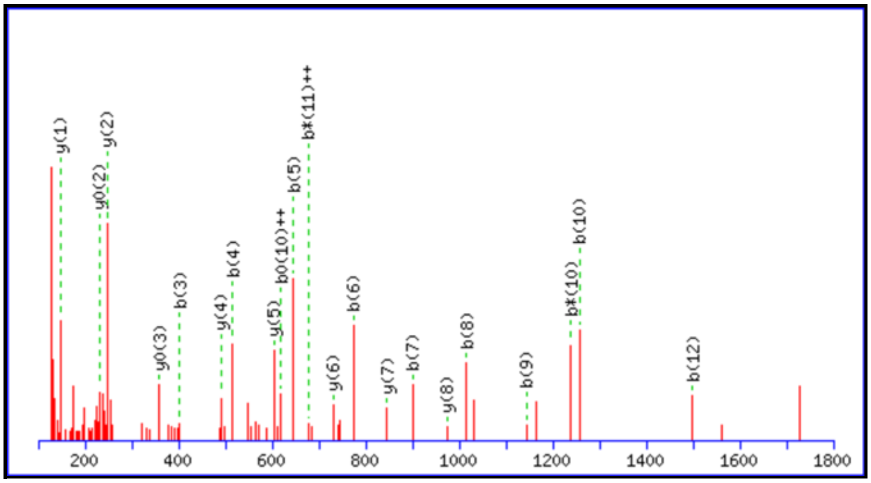

Monoisotopic mass of neutral peptide Mr(calc): 1744.9835
Fixed modifications: Carbamidomethyl (C) (apply to specified residues or termini only)
Ions Score: 78 **Expect:** 9.4e-007
Matches : 21/144 fragment ions using 26 most intense peaks (help)

#	b	b^{++}	b*	b^{*++}	b^0	b^{0++}	Seq.	y	y^{++}	y*	y^{*++}	y^0	y^{0++}	#
1	100.0757	50.5415					V							14
2	256.1768	128.5920	239.1503	120.0788			R	1646.9224	823.9649	1629.8959	815.4516	1628.9119	814.9596	13
3	403.2452	202.1262	386.2187	193.6130			F	1490.8213	745.9143	1473.7948	737.4010	1472.8108	736.9090	12
4	516.3293	258.6683	499.3027	250.1550			L	1343.7529	672.3801	1326.7264	663.8668	1325.7423	663.3748	11
5	645.3719	323.1896	628.3453	314.6763	627.3613	314.1843	E	1230.6688	615.8381	1213.6423	607.3248	1212.6583	606.8328	10
6	773.4305	387.2189	756.4039	378.7056	755.4199	378.2136	Q	1101.6263	551.3168	1084.5997	542.8035	1083.6157	542.3115	9
7	901.4890	451.2482	884.4625	442.7349	883.4785	442.2429	Q	973.5677	487.2875	956.5411	478.7742	955.5571	478.2822	8
8	1015.5320	508.2696	998.5054	499.7563	997.5214	499.2643	N	845.5091	423.2582	828.4825	414.7449	827.4985	414.2529	7
9	1143.6269	572.3171	1126.6004	563.8038	1125.6164	563.3118	K	731.4662	366.2367	714.4396	357.7234	713.4556	357.2314	6
10	1256.7110	628.8591	1239.6844	620.3459	1238.7004	619.8538	L	603.3712	302.1892	586.3447	293.6760	585.3606	293.1840	5
11	1369.7950	685.4012	1352.7685	676.8879	1351.7845	676.3959	L	490.2871	245.6472	473.2606	237.1339	472.2766	236.6419	4
12	1498.8376	749.9225	1481.8111	741.4092	1480.8271	740.9172	E	377.2031	189.1052	360.1765	180.5919	359.1925	180.0999	3
13	1599.8853	800.4463	1582.8588	791.9330	1581.8748	791.4410	T	248.1605	124.5839	231.1339	116.0706	230.1499	115.5786	2
14							K	147.1128	74.0600	130.0863	65.5468			1

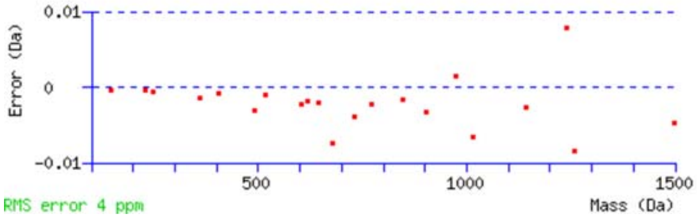

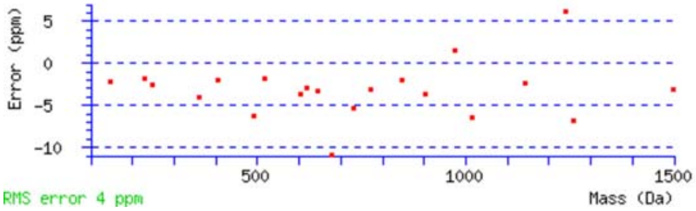

NCBI **BLAST** search of VRFLEQQNKLLETK
(Parameters: blastp, nr protein database, expect=20000, no filter, PAM30)
Other BLAST web gateways

All matches to this query

Score	Mr(calc)	Delta	Sequence
78.1	1744.9835	-0.0006	VRFLEQQNKLLETK
6.9	1743.9784	1.0046	KGFRLLNPHPKPNPK
0.0	1743.9659	1.0171	GSLTFEPLTLVPIQTK

Mascot: http://www.matrixscience.com/

Mascot Search Results

Peptide View

MS/MS Fragmentation of **LGLDIEIATYRR**
Found in **ch12u_Q9NSB2|KRT84_HUMAN** in **uni_human**, Keratin, type II cuticular Hb4 OS=Homo sapiens GN=KRT84 PE=2 SV=2

Match to Query 13040: 1418.788392 from(473.936740,3+) intensity(561901.3125) rtinseconds(2205) scans(12190) index(24079)
Title: 160219_Sunil_KAP_A1_Spectrum076549_scans__12190_RTINSECONDS=2205
Data file L:\\QE_2016\\160219_Sunil_KAP_LKC\\TMgf\\T\\T160219_Sunil_KAP_A1.mgf

Click mouse within plot area to zoom in by factor of two about that point
Or, Plot from 100 to 1500 Da Full range
Label all possible matches ○ Label matches used for scoring ◉
Show Y-axis ☐

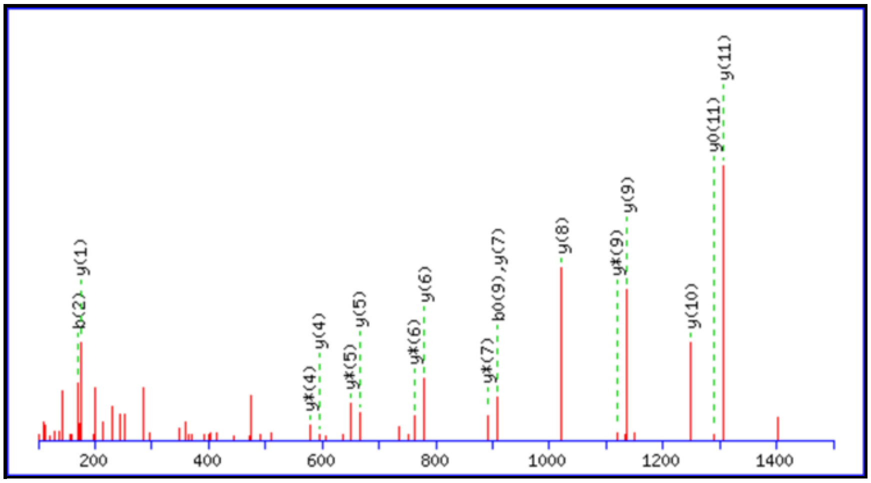

Monoisotopic mass of neutral peptide Mr(calc): 1418.7881
Fixed modifications: Carbamidomethyl (C) (apply to specified residues or termini only)
Ions Score: 63 **Expect:** 5.1e-005
Matches : 17/100 fragment ions using 32 most intense peaks (help)

#	b	b++	b*	b*++	b0	b0++	Seq.	y	y++	y*	y*++	y0	y0++	#
1	114.0913	57.5493					L							12
2	171.1128	86.0600					G	1306.7114	653.8593	1289.6848	645.3461	1288.7008	644.8540	11
3	284.1969	142.6021					L	1249.6899	625.3486	1232.6634	616.8353	1231.6793	616.3433	10
4	399.2238	200.1155			381.2132	191.1103	D	1136.6058	568.8066	1119.5793	560.2933	1118.5953	559.8013	9
5	512.3079	256.6576			494.2973	247.6523	I	1021.5789	511.2931	1004.5524	502.7798	1003.5683	502.2878	8
6	641.3505	321.1789			623.3399	312.1736	E	908.4948	454.7511	891.4683	446.2378	890.4843	445.7458	7
7	754.4345	377.7209			736.4240	368.7156	I	779.4522	390.2298	762.4257	381.7165	761.4417	381.2245	6
8	825.4716	413.2395			807.4611	404.2342	A	666.3682	333.6877	649.3416	325.1745	648.3576	324.6824	5
9	926.5193	463.7633			908.5088	454.7580	T	595.3311	298.1692	578.3045	289.6559	577.3205	289.1639	4
10	1089.5827	545.2950			1071.5721	536.2897	Y	494.2834	247.6453	477.2568	239.1321			3
11	1245.6838	623.3455	1228.6572	614.8322	1227.6732	614.3402	R	331.2201	166.1137	314.1935	157.6004			2
12							R	175.1190	88.0631	158.0924	79.5498			1

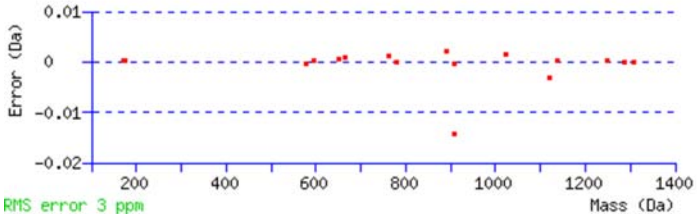

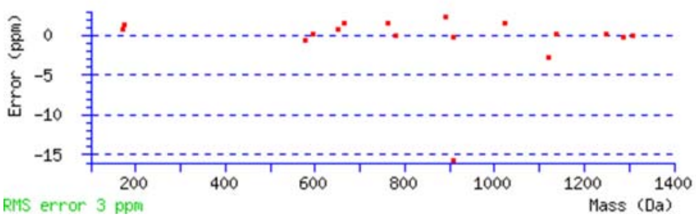

NCBI **BLAST** search of LGLDIEIATYRR
(Parameters: blastp, nr protein database, expect=20000, no filter, PAM30)
Other BLAST web gateways

All matches to this query

Score	Mr(calc)	Delta	Sequence
62.7	1418.7881	0.0003	LGLDIEIATYRR
23.9	1418.7881	0.0003	LGLLDRIVNYSR
12.9	1418.7769	0.0115	LLGNTFVALSDLR
7.3	1418.7881	0.0003	LQLRSLQYLER
5.4	1418.7769	0.0115	GLVLQLIQSYQR
3.6	1418.7769	0.0115	GLVLQLIQSYQR
2.4	1418.7742	0.0142	LGEARHPQVSLGR
2.4	1418.7994	-0.0110	LGPESVPPPKRSR

Mascot: http://www.matrixscience.com/

Mascot Search Results

Peptide View

MS/MS Fragmentation of **FLEQQNKLLETK**
Found in **ch12u_Q9NSB2|KRT84_HUMAN** in **uni_human**, Keratin, type II cuticular Hb4 OS=Homo sapiens GN=KRT84 PE=2 SV=2

Match to Query 15105: 1489.813208 from(745.913880,2+) intensity(344580.4063) rtinseconds(1370) scans(7390) index(20115)
Title: 160219_Sunil_KAP_A1_Spectrum072565_scans__7390_RTINSECONDS=1370
Data file L:\\QE_2016\\160219_Sunil_KAP_LKC\\TMgf\\T\\T160219_Sunil_KAP_A1.mgf

Click mouse within plot area to zoom in by factor of two about that point
Or, Plot from 100 to 1300 Da Full range
Label all possible matches ○ Label matches used for scoring ◉
Show Y-axis ☐

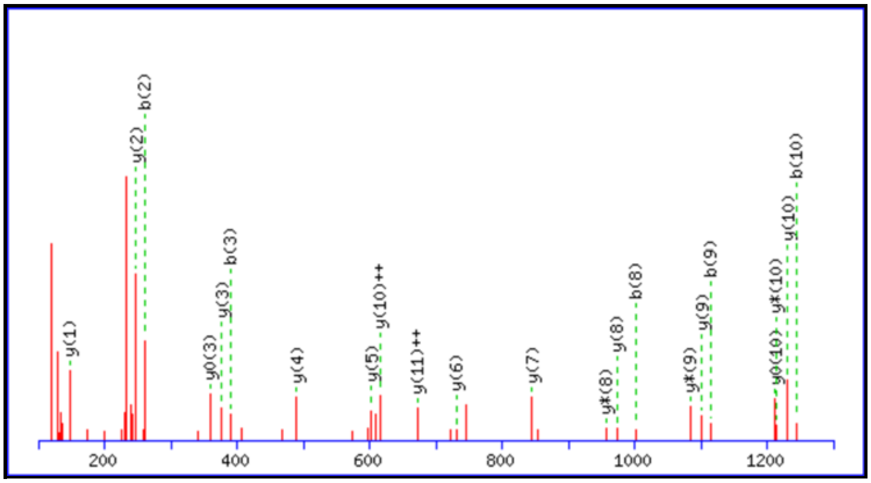

Monoisotopic mass of neutral peptide Mr(calc): 1489.8140
Fixed modifications: Carbamidomethyl (C) (apply to specified residues or termini only)
Ions Score: 83 **Expect:** 5.3e-007
Matches : 22/120 fragment ions using 30 most intense peaks (help)

#	b	b^{++}	b*	b^{*++}	b^0	b^{0++}	Seq.	y	y^{++}	y*	y^{*++}	y^0	y^{0++}	#
1	148.0757	74.5415					F							12
2	**261.1598**	131.0835					L	1343.7529	672.3801	1326.7264	663.8668	1325.7423	663.3748	11
3	**390.2023**	195.6048			372.1918	186.5995	E	***1230.6688***	615.8381	1213.6423	607.3248	1212.6583	606.8328	10
4	518.2609	259.6341	501.2344	251.1208	500.2504	250.6288	Q	***1101.6263***	551.3168	1084.5997	542.8035	1083.6157	542.3115	9
5	646.3195	323.6634	629.2930	315.1501	628.3089	314.6581	Q	***973.5677***	487.2875	956.5411	478.7742	955.5571	478.2822	8
6	760.3624	380.6849	743.3359	372.1716	742.3519	371.6796	N	***845.5091***	423.2582	828.4825	414.7449	827.4985	414.2529	7
7	888.4574	444.7323	871.4308	436.2191	870.4468	435.7271	K	***731.4662***	366.2367	714.4396	357.7234	713.4556	357.2314	6
8	**1001.5415**	501.2744	984.5149	492.7611	983.5309	492.2691	L	***603.3712***	302.1892	586.3447	293.6760	585.3606	293.1840	5
9	**1114.6255**	557.8164	1097.5990	549.3031	1096.6150	548.8111	L	***490.2871***	245.6472	473.2606	237.1339	472.2766	236.6419	4
10	**1243.6681**	622.3377	1226.6416	613.8244	1225.6575	613.3324	E	***377.2031***	189.1052	360.1765	180.5919	359.1925	180.0999	3
11	1344.7158	672.8615	1327.6892	664.3483	1326.7052	663.8563	T	***248.1605***	124.5839	231.1339	116.0706	230.1499	115.5786	2
12							K	***147.1128***	74.0600	130.0863	65.5468			1

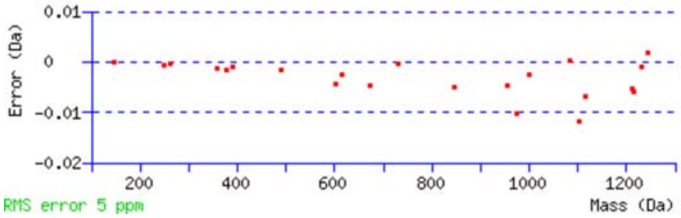

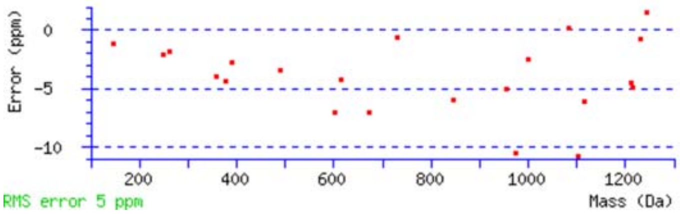

NCBI **BLAST** search of FLEQQNKLLETK
(Parameters: blastp, nr protein database, expect=20000, no filter, PAM30)
Other BLAST web gateways

All matches to this query

Score	Mr(calc)	Delta	Sequence
83.4	1489.8140	-0.0008	FLEQQNKLLETK

Mascot: http://www.matrixscience.com/

Mascot Search Results

Peptide View

MS/MS Fragmentation of **FLEQQNKLLETK**
Found in **ch12u_Q9NSB2|KRT84_HUMAN** in **uni_human**, Keratin, type II cuticular Hb4 OS=Homo sapiens GN=KRT84 PE=2 SV=2

Match to Query 15117: 1489.814668 from(745.914610,2+) intensity(567330.1875) rtinseconds(1348) scans(7210) index(34213)
Title: 160219_Sunil_KAP_A1_Spectrum088470_scans__7210_RTINSECONDS=1348
Data file L:\\QE_2016\\160219_Sunil_KAP_LKC\\TMgf\\T\\T160219_Sunil_KAP_A1.mgf

Click mouse within plot area to zoom in by factor of two about that point
Or, Plot from 100 to 1300 Da Full range
Label all possible matches ○ Label matches used for scoring ◉
Show Y-axis ☐

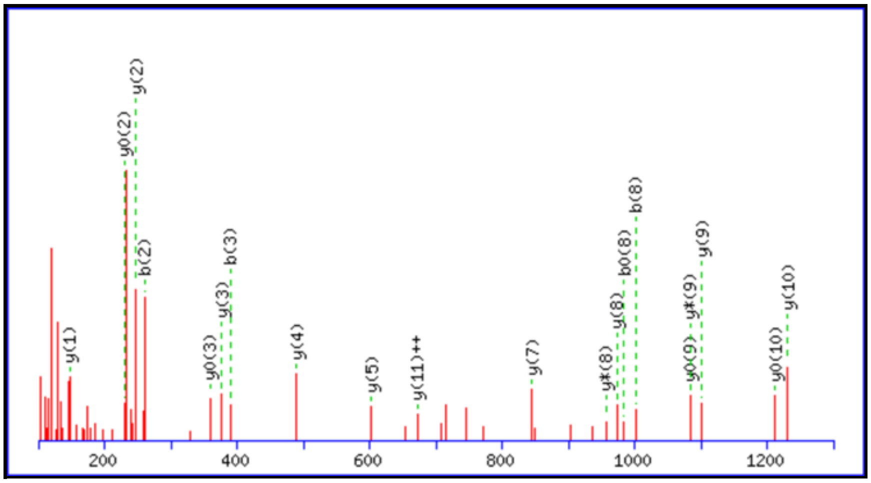

Monoisotopic mass of neutral peptide Mr(calc): 1489.8140
Fixed modifications: Carbamidomethyl (C) (apply to specified residues or termini only)
Ions Score: 63 **Expect:** 5.2e-005
Matches : 20/120 fragment ions using 33 most intense peaks (help)

#	b	b^{++}	b*	b^{*++}	b^0	b^{0++}	Seq.	y	y^{++}	y*	y^{*++}	y^0	y^{0++}	#
1	148.0757	74.5415					F							12
2	**261.1598**	131.0835					L	1343.7529	672.3801	1326.7264	663.8668	1325.7423	663.3748	11
3	**390.2023**	195.6048			372.1918	186.5995	E	***1230.6688***	615.8381	1213.6423	607.3248	1212.6583	606.8328	10
4	518.2609	259.6341	501.2344	251.1208	500.2504	250.6288	Q	***1101.6263***	551.3168	1084.5997	542.8035	1083.6157	542.3115	9
5	646.3195	323.6634	629.2930	315.1501	628.3089	314.6581	Q	***973.5677***	487.2875	956.5411	478.7742	955.5571	478.2822	8
6	760.3624	380.6849	743.3359	372.1716	742.3519	371.6796	N	***845.5091***	423.2582	828.4825	414.7449	827.4985	414.2529	7
7	888.4574	444.7323	871.4308	436.2191	870.4468	435.7271	K	731.4662	366.2367	714.4396	357.7234	713.4556	357.2314	6
8	**1001.5415**	501.2744	984.5149	492.7611	983.5309	492.2691	L	***603.3712***	302.1892	586.3447	293.6760	585.3606	293.1840	5
9	1114.6255	557.8164	1097.5990	549.3031	1096.6150	548.8111	L	***490.2871***	245.6472	473.2606	237.1339	472.2766	236.6419	4
10	1243.6681	622.3377	1226.6416	613.8244	1225.6575	613.3324	E	***377.2031***	189.1052	360.1765	180.5919	359.1925	180.0999	3
11	1344.7158	672.8615	1327.6892	664.3483	1326.7052	663.8563	T	***248.1605***	124.5839	231.1339	116.0706	230.1499	115.5786	2
12							K	***147.1128***	74.0600	130.0863	65.5468			1

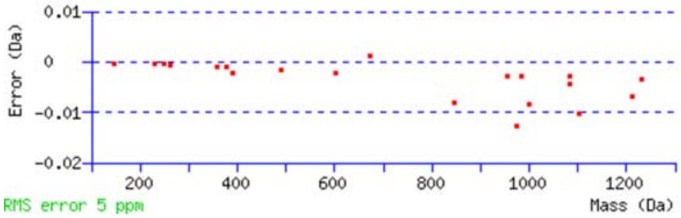

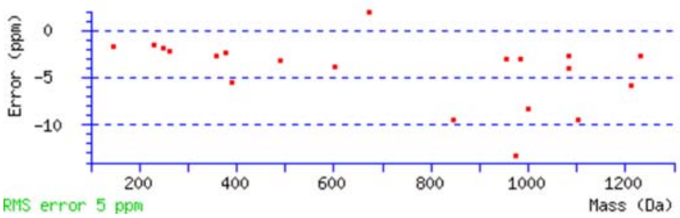

NCBI **BLAST** search of FLEQQNKLLETK
(Parameters: blastp, nr protein database, expect=20000, no filter, PAM30)
Other BLAST web gateways

All matches to this query

Score	Mr(calc)	Delta	Sequence
63.4	1489.8140	0.0007	FLEQQNKLLETK

Mascot: http://www.matrixscience.com/

Mascot Search Results

Peptide View

MS/MS Fragmentation of **FASFIDKVR**
Found in **ch12u_Q9NSB2|KRT84_HUMAN** in **uni_human**, Keratin, type II cuticular Hb4 OS=Homo sapiens GN=KRT84 PE=2 SV=2

Match to Query 4576: 1081.591332 from(361.537720,3+) intensity(231063.7969) rtinseconds(1617) scans(8528) index(37014)
Title: 160219_Sunil_SDSI_A_Spectrum091599_scans__8528_RTINSECONDS=1617
Data file L:\\QE_2016\\160219_Sunil_KAP_LKC\\TMgf\\T\\T160219_Sunil_SDSI_A.mgf

Click mouse within plot area to zoom in by factor of two about that point
Or, Plot from 50 to 900 Da Full range
Label all possible matches ○ Label matches used for scoring ◉
Show Y-axis ☐

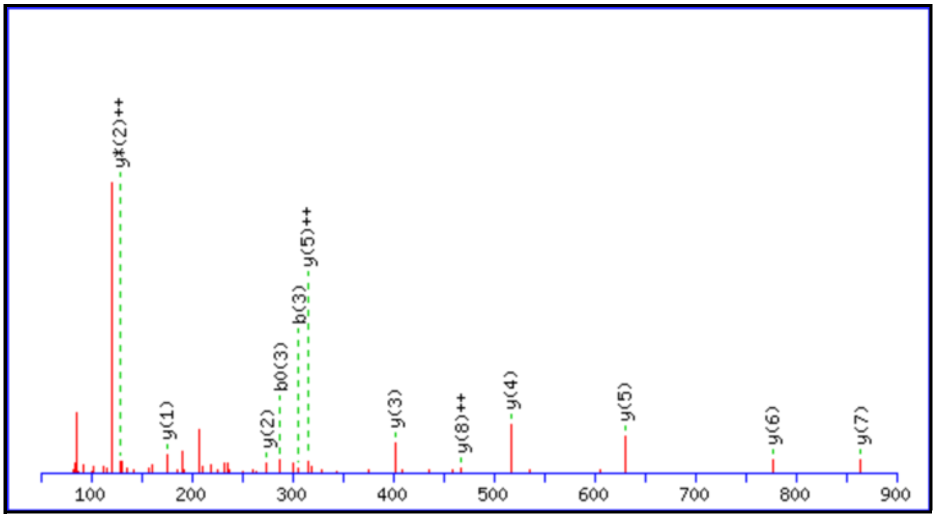

Monoisotopic mass of neutral peptide Mr(calc): 1081.5920
Fixed modifications: Carbamidomethyl (C) (apply to specified residues or termini only)
Ions Score: 42 **Expect:** 0.0046
Matches : 12/74 fragment ions using 28 most intense peaks (help)

#	b	b^{++}	b*	b^{*++}	b^0	b^{0++}	Seq.	y	y^{++}	y*	y^{*++}	y^0	y^{0++}	#
1	148.0757	74.5415					F							9
2	219.1128	110.0600					A	935.5309	468.2691	918.5043	459.7558	917.5203	459.2638	8
3	**306.1448**	153.5761			288.1343	144.5708	S	***864.4938***	432.7505	847.4672	424.2373	846.4832	423.7452	7
4	453.2132	227.1103			435.2027	218.1050	F	***777.4618***	389.2345	760.4352	380.7212	759.4512	380.2292	6
5	566.2973	283.6523			548.2867	274.6470	I	***630.3933***	315.7003	613.3668	307.1870	612.3828	306.6950	5
6	681.3243	341.1658			663.3137	332.1605	D	***517.3093***	259.1583	500.2827	250.6450	499.2987	250.1530	4
7	809.4192	405.2132	792.3927	396.7000	791.4087	396.2080	K	***402.2823***	201.6448	385.2558	193.1315			3
8	908.4876	454.7475	891.4611	446.2342	890.4771	445.7422	V	***274.1874***	137.5973	257.1608	129.0840			2
9							R	***175.1190***	88.0631	158.0924	79.5498			1

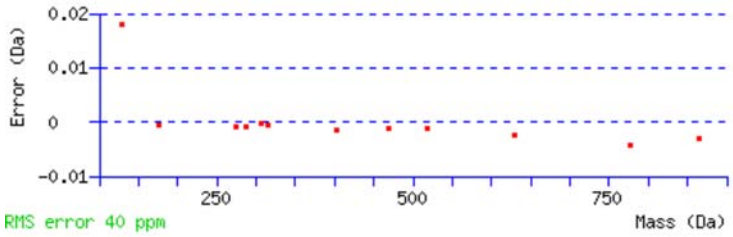

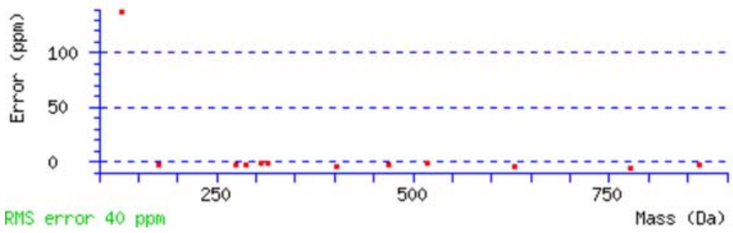

NCBI **BLAST** search of FASFIDKVR
(Parameters: blastp, nr protein database, expect=20000, no filter, PAM30)
Other BLAST web gateways

All matches to this query

Score	Mr(calc)	Delta	Sequence
41.7	1081.5920	-0.0007	FASFIDKVR
41.7	1081.5920	-0.0007	FASFINKVR
9.7	1081.5920	-0.0007	AFSFVKDLR
4.5	1079.5909	2.0004	RYMLKNVR
3.3	1081.5992	-0.0079	LSDHLSVRR
3.3	1080.5927	0.9986	KVTYRNLST

Mascot: http://www.matrixscience.com/

Mascot Search Results

Peptide View

MS/MS Fragmentation of **FLEQQNKLLETK**
Found in **ch12u_Q9NSB2|KRT84_HUMAN** in **uni_human**, Keratin, type II cuticular Hb4 OS=Homo sapiens GN=KRT84 PE=2 SV=2

Match to Query 17452: 1490.801008 from(746.407780,2+) intensity(6235103.5000) rtinseconds(1792) scans(9531) index(7263)
Title: 160219_Sunil_SDSI_A_Spectrum059371_scans__9531_RTINSECONDS=1792
Data file L:\\QE_2016\\160219_Sunil_KAP_LKC\\TMgf\\T\\T160219_Sunil_SDSI_A.mgf

Click mouse within plot area to zoom in by factor of two about that point
Or, Plot from 100 to 1500 Da Full range
Label all possible matches ○ Label matches used for scoring ◉
Show Y-axis ☐

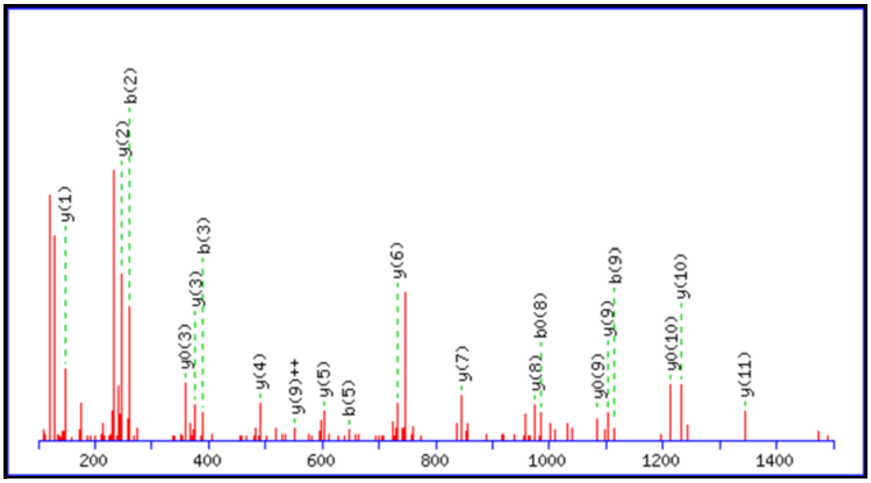

Monoisotopic mass of neutral peptide Mr(calc): 1490.7980
Fixed modifications: Carbamidomethyl (C) (apply to specified residues or termini only)
Variable modifications:
N6 : Deamidated (NQ)
Ions Score: 79 **Expect:** 1.4e-006
Matches : 20/120 fragment ions using 38 most intense peaks (help)

#	b	b^{++}	b*	b^{*++}	b^0	b^{0++}	Seq.	y	y^{++}	y*	y^{*++}	y^0	y^{0++}	#
1	148.0757	74.5415					F							12
2	**261.1598**	131.0835					L	***1344.7369***	672.8721	1327.7104	664.3588	1326.7264	663.8668	11
3	**390.2023**	195.6048			372.1918	186.5995	E	***1231.6529***	616.3301	1214.6263	607.8168	1213.6423	607.3248	10
4	518.2609	259.6341	501.2344	251.1208	500.2504	250.6288	Q	***1102.6103***	551.8088	1085.5837	543.2955	1084.5997	542.8035	9
5	**646.3195**	323.6634	629.2930	315.1501	628.3089	314.6581	Q	***974.5517***	487.7795	957.5251	479.2662	956.5411	478.7742	8
6	761.3464	381.1769	744.3199	372.6636	743.3359	372.1716	N	***846.4931***	423.7502	829.4666	415.2369	828.4825	414.7449	7
7	889.4414	445.2243	872.4149	436.7111	871.4308	436.2191	K	***731.4662***	366.2367	714.4396	357.7234	713.4556	357.2314	6
8	1002.5255	501.7664	985.4989	493.2531	984.5149	492.7611	L	***603.3712***	302.1892	586.3447	293.6760	585.3606	293.1840	5
9	**1115.6095**	558.3084	1098.5830	549.7951	1097.5990	549.3031	L	***490.2871***	245.6472	473.2606	237.1339	472.2766	236.6419	4
10	1244.6521	622.8297	1227.6256	614.3164	1226.6416	613.8244	E	***377.2031***	189.1052	360.1765	180.5919	359.1925	180.0999	3
11	1345.6998	673.3535	1328.6733	664.8403	1327.6892	664.3483	T	***248.1605***	124.5839	231.1339	116.0706	230.1499	115.5786	2
12							K	***147.1128***	74.0600	130.0863	65.5468			1

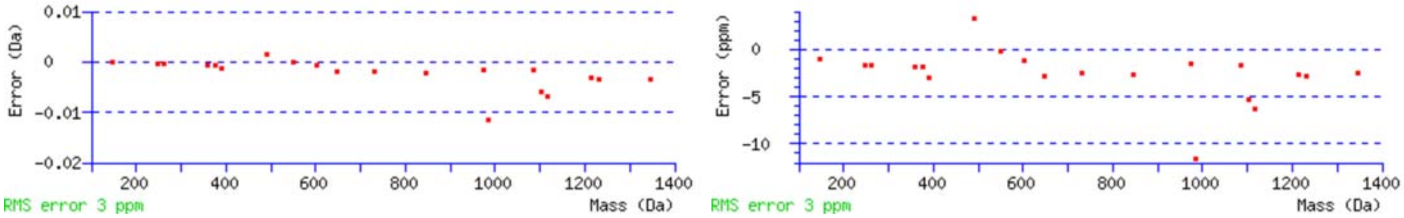

NCBI **BLAST** search of FLEQQNKLLETK
(Parameters: blastp, nr protein database, expect=20000, no filter, PAM30)
Other BLAST web gateways

All matches to this query

Score	Mr(calc)	Delta	Sequence	Site Analysis
79.4	1490.7980	0.0030	FLEQQNKLLETK	Deamidated N6 97.33%
63.5	1490.7980	0.0030	FLEQQNKLLETK	Deamidated Q5 2.53%
51.0	1490.7980	0.0030	FLEQQNKLLETK	Deamidated Q4 0.14%
12.9	1490.7981	0.0030	FQALQSVDILETK	

Mascot: http://www.matrixscience.com/

Mascot Search Results

Peptide View

MS/MS Fragmentation of **FLEQQNKLLETK**
Found in **ch12u_Q9NSB2|KRT84_HUMAN** in **uni_human**, Keratin, type II cuticular Hb4 OS=Homo sapiens GN=KRT84 PE=2 SV=2

Match to Query 17452: 1490.801008 from(746.407780,2+) intensity(6235103.5000) rtinseconds(1792) scans(9531) index(7263)
Title: 160219_Sunil_SDSI_A_Spectrum059371_scans__9531_RTINSECONDS=1792
Data file L:\\QE_2016\\160219_Sunil_KAP_LKC\\TMgf\\T\\T160219_Sunil_SDSI_A.mgf

Click mouse within plot area to zoom in by factor of two about that point
Or, Plot from 100 to 1500 Da Full range
Label all possible matches ○ Label matches used for scoring ◉
Show Y-axis □

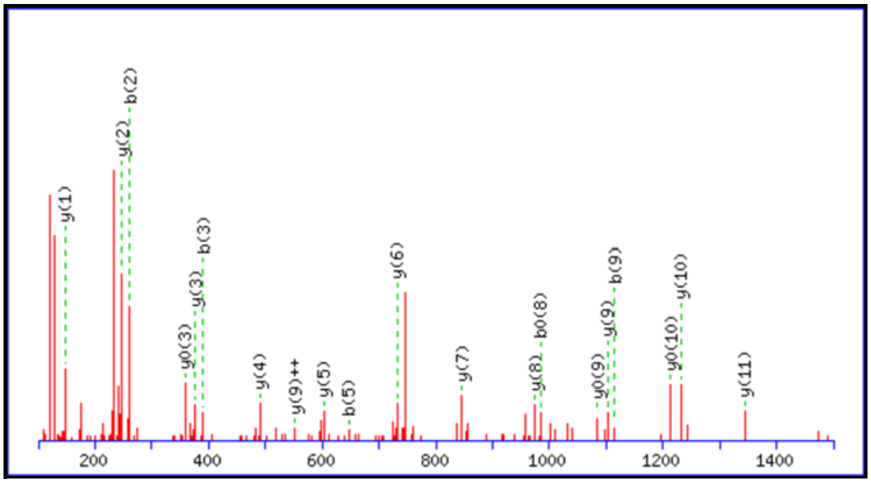

Monoisotopic mass of neutral peptide Mr(calc): 1490.7980
Fixed modifications: Carbamidomethyl (C) (apply to specified residues or termini only)
Variable modifications:
N6 : Deamidated (NQ)
Ions Score: 79 **Expect:** 1.4e-006
Matches : 20/120 fragment ions using 38 most intense peaks (help)

#	b	b^{++}	b*	b^{*++}	b^0	b^{0++}	Seq.	y	y^{++}	y*	y^{*++}	y^0	y^{0++}	#
1	148.0757	74.5415					F							12
2	**261.1598**	131.0835					L	***1344.7369***	672.8721	1327.7104	664.3588	1326.7264	663.8668	11
3	**390.2023**	195.6048			372.1918	186.5995	E	***1231.6529***	616.3301	1214.6263	607.8168	1213.6423	607.3248	10
4	518.2609	259.6341	501.2344	251.1208	500.2504	250.6288	Q	***1102.6103***	551.8088	1085.5837	543.2955	1084.5997	542.8035	9
5	**646.3195**	323.6634	629.2930	315.1501	628.3089	314.6581	Q	***974.5517***	487.7795	957.5251	479.2662	956.5411	478.7742	8
6	761.3464	381.1769	744.3199	372.6636	743.3359	372.1716	N	***846.4931***	423.7502	829.4666	415.2369	828.4825	414.7449	7
7	889.4414	445.2243	872.4149	436.7111	871.4308	436.2191	K	***731.4662***	366.2367	714.4396	357.7234	713.4556	357.2314	6
8	1002.5255	501.7664	985.4989	493.2531	984.5149	492.7611	L	***603.3712***	302.1892	586.3447	293.6760	585.3606	293.1840	5
9	**1115.6095**	558.3084	1098.5830	549.7951	1097.5990	549.3031	L	***490.2871***	245.6472	473.2606	237.1339	472.2766	236.6419	4
10	1244.6521	622.8297	1227.6256	614.3164	1226.6416	613.8244	E	***377.2031***	189.1052	360.1765	180.5919	359.1925	180.0999	3
11	1345.6998	673.3535	1328.6733	664.8403	1327.6892	664.3483	T	***248.1605***	124.5839	231.1339	116.0706	230.1499	115.5786	2
12							K	***147.1128***	74.0600	130.0863	65.5468			1

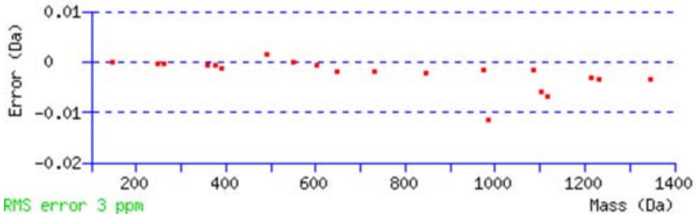

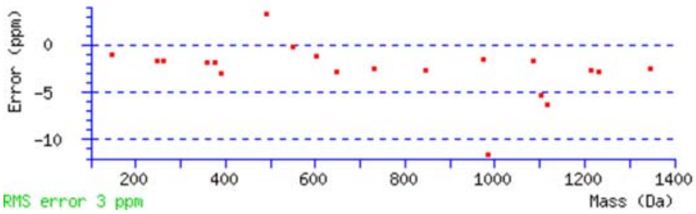

NCBI **BLAST** search of FLEQQNKLLETK
(Parameters: blastp, nr protein database, expect=20000, no filter, PAM30)
Other BLAST web gateways

All matches to this query

Score	Mr(calc)	Delta	Sequence	Site Analysis
79.4	1490.7980	0.0030	FLEQQNKLLETK	Deamidated N6 97.33%
63.5	1490.7980	0.0030	FLEQQNKLLETK	Deamidated Q5 2.53%
51.0	1490.7980	0.0030	FLEQQNKLLETK	Deamidated Q4 0.14%
12.9	1490.7981	0.0030	FQALQSVDILETK	

Mascot: http://www.matrixscience.com/

Mascot Search Results

Peptide View

MS/MS Fragmentation of **FLEQQNKLLETK**
Found in **ch12u_Q9NSB2|KRT84_HUMAN** in **uni_human**, Keratin, type II cuticular Hb4 OS=Homo sapiens GN=KRT84 PE=2 SV=2

Match to Query 17504: 1491.802468 from(746.908510,2+) intensity(2214994.5000) rtinseconds(1725) scans(9134) index(6903)
Title: 160219_Sunil_SDSI_A_Spectrum059011_scans__9134_RTINSECONDS=1725
Data file L:\\QE_2016\\160219_Sunil_KAP_LKC\\TMgf\\T\\T160219_Sunil_SDSI_A.mgf

Click mouse within plot area to zoom in by factor of two about that point
Or, Plot from 100 to 1400 Da Full range
Label all possible matches ○ Label matches used for scoring ◉
Show Y-axis □

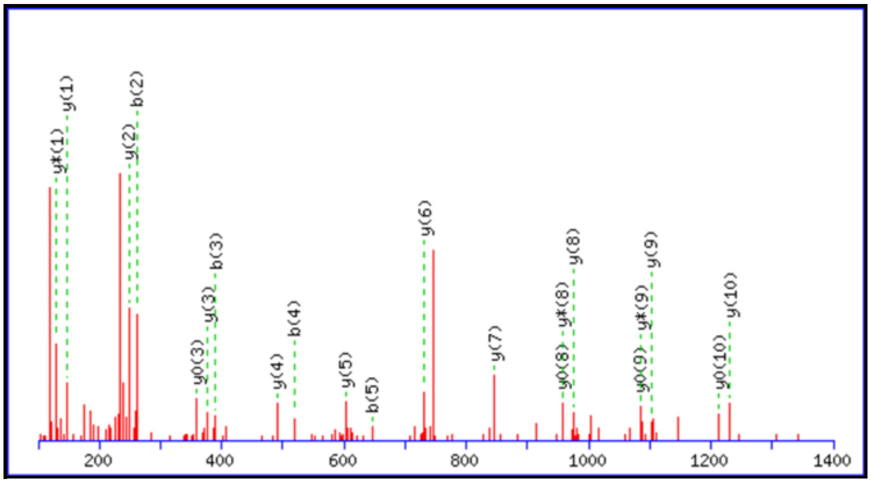

Monoisotopic mass of neutral peptide Mr(calc): 1490.7980
Fixed modifications: Carbamidomethyl (C) (apply to specified residues or termini only)
Variable modifications:
N6 : Deamidated (NQ)
Ions Score: 53 **Expect:** 0.0006
Matches : 21/120 fragment ions using 50 most intense peaks (help)

#	b	b^{++}	b*	b^{*++}	b^{0}	b^{0++}	Seq.	y	y^{++}	y*	y^{*++}	y^{0}	y^{0++}	#
1	148.0757	74.5415					F							12
2	**261.1598**	131.0835					L	1344.7369	672.8721	1327.7104	664.3588	1326.7264	663.8668	11
3	**390.2023**	195.6048			372.1918	186.5995	E	***1231.6529***	616.3301	1214.6263	607.8168	1213.6423	607.3248	10
4	**518.2609**	259.6341	501.2344	251.1208	500.2504	250.6288	Q	***1102.6103***	551.8088	1085.5837	543.2955	1084.5997	542.8035	9
5	**646.3195**	323.6634	629.2930	315.1501	628.3089	314.6581	Q	***974.5517***	487.7795	957.5251	479.2662	956.5411	478.7742	8
6	761.3464	381.1769	744.3199	372.6636	743.3359	372.1716	N	***846.4931***	423.7502	829.4666	415.2369	828.4825	414.7449	7
7	889.4414	445.2243	872.4149	436.7111	871.4308	436.2191	K	***731.4662***	366.2367	714.4396	357.7234	713.4556	357.2314	6
8	1002.5255	501.7664	985.4989	493.2531	984.5149	492.7611	L	***603.3712***	302.1892	586.3447	293.6760	585.3606	293.1840	5
9	1115.6095	558.3084	1098.5830	549.7951	1097.5990	549.3031	L	***490.2871***	245.6472	473.2606	237.1339	472.2766	236.6419	4
10	1244.6521	622.8297	1227.6256	614.3164	1226.6416	613.8244	E	***377.2031***	189.1052	360.1765	180.5919	359.1925	180.0999	3
11	1345.6998	673.3535	1328.6733	664.8403	1327.6892	664.3483	T	***248.1605***	124.5839	231.1339	116.0706	230.1499	115.5786	2
12							K	***147.1128***	74.0600	130.0863	65.5468			1

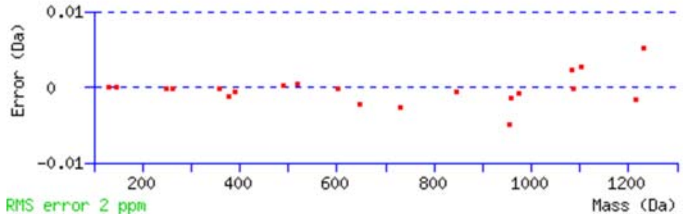

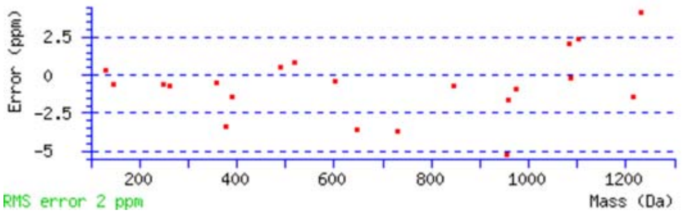

NCBI **BLAST** search of FLEQQNKLLETK
(Parameters: blastp, nr protein database, expect=20000, no filter, PAM30)
Other BLAST web gateways

All matches to this query

Score	Mr(calc)	Delta	Sequence	Site Analysis
53.0	1490.7980	1.0044	FLEQQNKLLETK	Deamidated N6 59.38%
51.1	1490.7980	1.0044	FLEQQNKLLETK	Deamidated Q5 39.05%
37.2	1490.7980	1.0044	FLEQQNKLLETK	Deamidated Q4 1.57%

Mascot: http://www.matrixscience.com/

Mascot Search Results

Peptide View

MS/MS Fragmentation of **FLEQQNKLLETK**
Found in **ch12u_Q9NSB2|KRT84_HUMAN** in **uni_human**, Keratin, type II cuticular Hb4 OS=Homo sapiens GN=KRT84 PE=2 SV=2

Match to Query 17454: 1490.802108 from(746.408330,2+) intensity(2734239.7500) rtinseconds(1540) scans(8205) index(21467)
Title: 160219_Sunil_SDSI_A_Spectrum074771_scans__8205_RTINSECONDS=1540
Data file L:\\QE_2016\\160219_Sunil_KAP_LKC\\TMgf\\T\\T160219_Sunil_SDSI_A.mgf

Click mouse within plot area to zoom in by factor of two about that point
Or, Plot from 100 to 1500 Da Full range
Label all possible matches ○ Label matches used for scoring ◉
Show Y-axis ☐

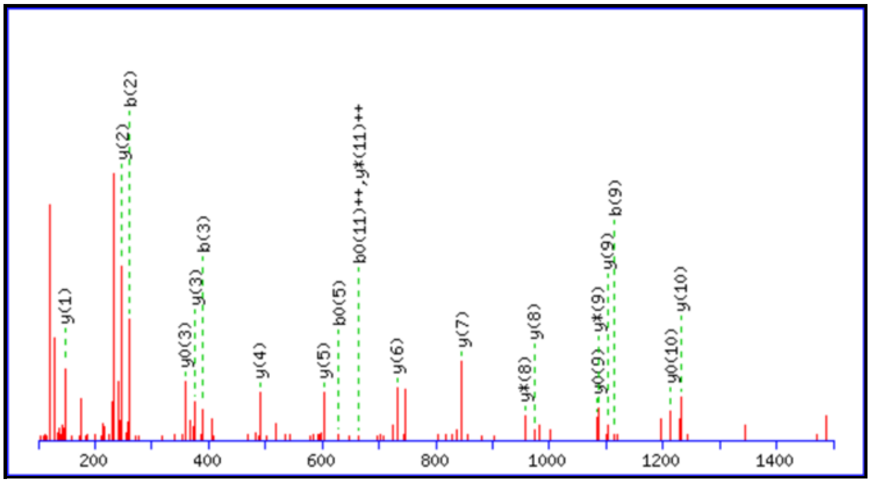

Monoisotopic mass of neutral peptide Mr(calc): 1490.7980
Fixed modifications: Carbamidomethyl (C) (apply to specified residues or termini only)
Variable modifications:
Q4 : Deamidated (NQ)
Ions Score: 65 **Expect:** 4.1e-005
Matches : 21/120 fragment ions using 39 most intense peaks (help)

#	b	b^{++}	b*	b^{*++}	b^0	b^{0++}	Seq.	y	y^{++}	y*	y^{*++}	y^0	y^{0++}	#
1	148.0757	74.5415					F							12
2	**261.1598**	131.0835					L	1344.7369	672.8721	1327.7104	664.3588	1326.7264	663.8668	11
3	**390.2023**	195.6048			372.1918	186.5995	E	***1231.6529***	616.3301	1214.6263	607.8168	1213.6423	607.3248	10
4	519.2449	260.1261	502.2184	251.6128	501.2344	251.1208	Q	***1102.6103***	551.8088	1085.5837	543.2955	1084.5997	542.8035	9
5	647.3035	324.1554	630.2770	315.6421	629.2930	315.1501	Q	***973.5677***	487.2875	956.5411	478.7742	955.5571	478.2822	8
6	761.3464	381.1769	744.3199	372.6636	743.3359	372.1716	N	***845.5091***	423.2582	828.4825	414.7449	827.4985	414.2529	7
7	889.4414	445.2243	872.4149	436.7111	871.4308	436.2191	K	***731.4662***	366.2367	714.4396	357.7234	713.4556	357.2314	6
8	1002.5255	501.7664	985.4989	493.2531	984.5149	492.7611	L	***603.3712***	302.1892	586.3447	293.6760	585.3606	293.1840	5
9	**1115.6095**	558.3084	1098.5830	549.7951	1097.5990	549.3031	L	***490.2871***	245.6472	473.2606	237.1339	472.2766	236.6419	4
10	1244.6521	622.8297	1227.6256	614.3164	1226.6416	613.8244	E	***377.2031***	189.1052	360.1765	180.5919	359.1925	180.0999	3
11	1345.6998	673.3535	1328.6733	664.8403	1327.6892	664.3483	T	***248.1605***	124.5839	231.1339	116.0706	230.1499	115.5786	2
12							K	***147.1128***	74.0600	130.0863	65.5468			1

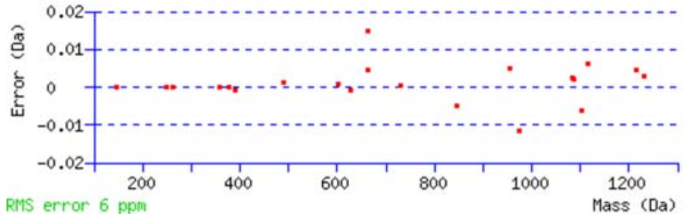

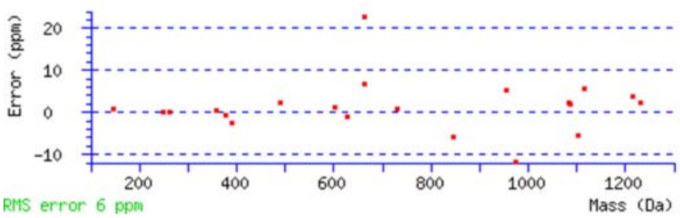

NCBI **BLAST** search of FLEQQNKLLETK
(Parameters: blastp, nr protein database, expect=20000, no filter, PAM30)
Other BLAST web gateways

All matches to this query

Score	Mr(calc)	Delta	Sequence	Site Analysis
64.6	1490.7980	0.0041	FLEQQNKLLETK	Deamidated Q4 85.03%
54.2	1490.7980	0.0041	FLEQQNKLLETK	Deamidated N6 7.74%
53.9	1490.7980	0.0041	FLEQQNKLLETK	Deamidated Q5 7.24%
5.9	1490.7981	0.0041	FQALQSVDILETK	

Mascot: http://www.matrixscience.com/

Mascot Search Results

Peptide View

MS/MS Fragmentation of **VRFLEQQNKLLETK**
Found in **ch12u_Q9NSB2|KRT84_HUMAN** in **uni_human**, Keratin, type II cuticular Hb4 OS=Homo sapiens GN=KRT84 PE=2 SV=2

Match to Query 23721: 1744.983912 from(582.668580,3+) intensity(2841219.7500) rtinseconds(1627) scans(8560) index(6406)
Title: 160219_Sunil_SDSI_A_Spectrum058514_scans__8560_RTINSECONDS=1627
Data file L:\\QE_2016\\160219_Sunil_KAP_LKC\\TMgf\\T\\T160219_Sunil_SDSI_A.mgf

Click mouse within plot area to zoom in by factor of two about that point
Or, Plot from 100 to 1800 Da Full range
Label all possible matches ○ Label matches used for scoring ◉
Show Y-axis ☐

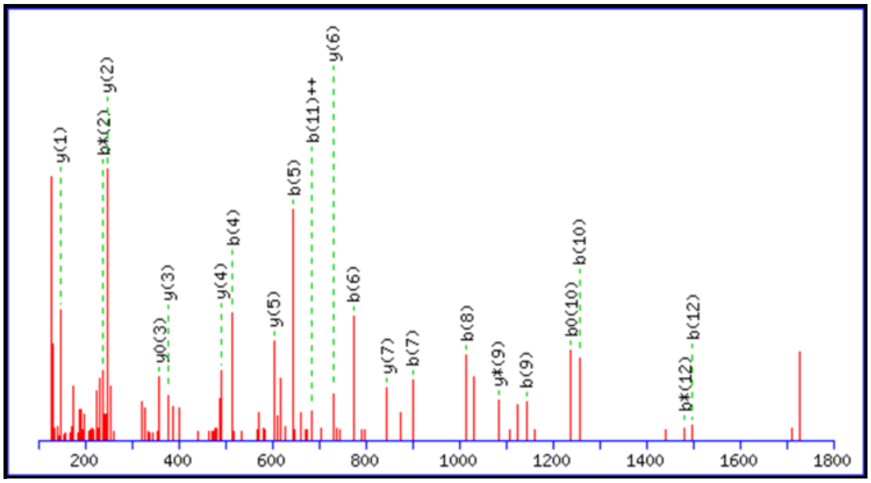

Monoisotopic mass of neutral peptide Mr(calc): 1744.9835
Fixed modifications: Carbamidomethyl (C) (apply to specified residues or termini only)
Ions Score: 74 **Expect:** 2.3e-006
Matches : 21/144 fragment ions using 27 most intense peaks (help)

#	b	b^{++}	b*	b^{*++}	b^{0}	b^{0++}	Seq.	y	y^{++}	y*	y^{*++}	y^{0}	y^{0++}	#
1	100.0757	50.5415					V							14
2	256.1768	128.5920	239.1503	120.0788			R	1646.9224	823.9649	1629.8959	815.4516	1628.9119	814.9596	13
3	403.2452	202.1262	386.2187	193.6130			F	1490.8213	745.9143	1473.7948	737.4010	1472.8108	736.9090	12
4	***516.3293***	258.6683	499.3027	250.1550			L	1343.7529	672.3801	1326.7264	663.8668	1325.7423	663.3748	11
5	***645.3719***	323.1896	628.3453	314.6763	627.3613	314.1843	E	1230.6688	615.8381	1213.6423	607.3248	1212.6583	606.8328	10
6	***773.4305***	387.2189	756.4039	378.7056	755.4199	378.2136	Q	1101.6263	551.3168	1084.5997	542.8035	1083.6157	542.3115	9
7	***901.4890***	451.2482	884.4625	442.7349	883.4785	442.2429	Q	973.5677	487.2875	956.5411	478.7742	955.5571	478.2822	8
8	***1015.5320***	508.2696	998.5054	499.7563	997.5214	499.2643	N	***845.5091***	423.2582	828.4825	414.7449	827.4985	414.2529	7
9	***1143.6269***	572.3171	1126.6004	563.8038	1125.6164	563.3118	K	***731.4662***	366.2367	714.4396	357.7234	713.4556	357.2314	6
10	***1256.7110***	628.8591	1239.6844	620.3459	1238.7004	619.8538	L	***603.3712***	302.1892	586.3447	293.6760	585.3606	293.1840	5
11	1369.7950	**685.4012**	1352.7685	676.8879	1351.7845	676.3959	L	***490.2871***	245.6472	473.2606	237.1339	472.2766	236.6419	4
12	***1498.8376***	749.9225	1481.8111	741.4092	1480.8271	740.9172	E	***377.2031***	189.1052	360.1765	180.5919	359.1925	180.0999	3
13	1599.8853	800.4463	1582.8588	791.9330	1581.8748	791.4410	T	***248.1605***	124.5839	231.1339	116.0706	230.1499	115.5786	2
14							K	***147.1128***	74.0600	130.0863	65.5468			1

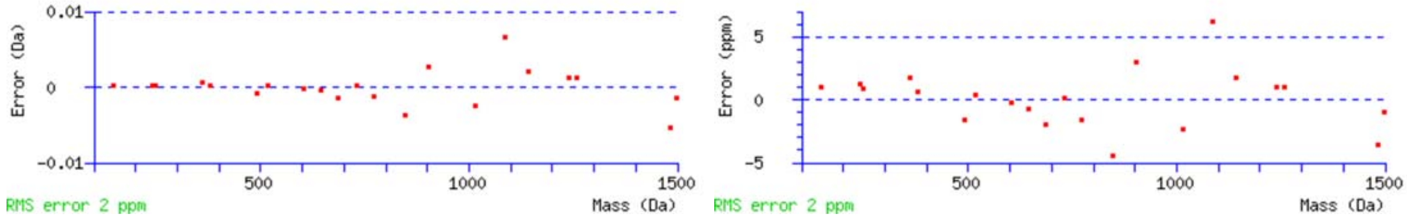

NCBI **BLAST** search of VRFLEQQNKLLETK
(Parameters: blastp, nr protein database, expect=20000, no filter, PAM30)
Other BLAST web gateways

All matches to this query

Score	Mr(calc)	Delta	Sequence
74.2	1744.9835	0.0004	VRFLEQQNKLLETK
7.9	1744.9869	-0.0030	KSLESINSRLQLVMK
4.9	1743.9784	1.0055	KGFRLLNPHPKPNPK
0.3	1743.9659	1.0181	GSLTFEPLTLVPIQTK

Mascot: http://www.matrixscience.com/

Mascot Search Results

Peptide View

MS/MS Fragmentation of **VRFLEQQNK**
Found in **ch12u_Q9NSB2|KRT84_HUMAN** in **uni_human**, Keratin, type II cuticular Hb4 OS=Homo sapiens GN=KRT84 PE=2 SV=2

Match to Query 7141: 1160.629988 from(581.322270,2+) intensity(6149513.5000) rtinseconds(771) scans(3600) index(2200)
Title: 160219_Sunil_SDSI_A_Spectrum054308_scans__3600_RTINSECONDS=771
Data file L:\\QE_2016\\160219_Sunil_KAP_LKC\\TMgf\\T\\T160219_Sunil_SDSI_A.mgf

Click mouse within plot area to zoom in by factor of two about that point
Or, Plot from 0 to 1200 Da Full range
Label all possible matches ○ Label matches used for scoring ◉
Show Y-axis ☐

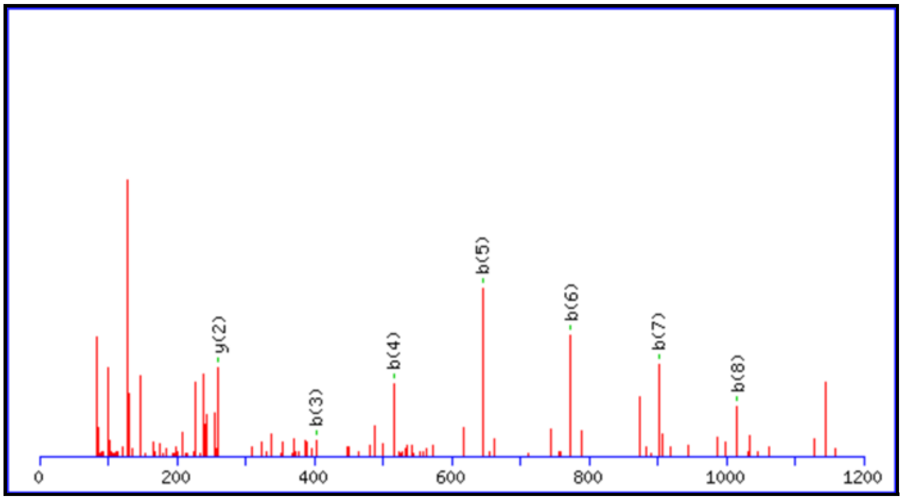

Monoisotopic mass of neutral peptide Mr(calc): 1160.6302
Fixed modifications: Carbamidomethyl (C) (apply to specified residues or termini only)
Ions Score: 47 **Expect:** 0.0032
Matches : 7/78 fragment ions using 11 most intense peaks (help)

#	b	b^{++}	b*	b^{*++}	b^0	b^{0++}	Seq.	y	y^{++}	y*	y^{*++}	y^0	y^{0++}	#
1	100.0757	50.5415					V							9
2	256.1768	128.5920	239.1503	120.0788			R	1062.5691	531.7882	1045.5425	523.2749	1044.5585	522.7829	8
3	***403.2452***	202.1262	386.2187	193.6130			F	906.4680	453.7376	889.4414	445.2243	888.4574	444.7323	7
4	***516.3293***	258.6683	499.3027	250.1550			L	759.3995	380.2034	742.3730	371.6901	741.3890	371.1981	6
5	***645.3719***	323.1896	628.3453	314.6763	627.3613	314.1843	E	646.3155	323.6614	629.2889	315.1481	628.3049	314.6561	5
6	***773.4305***	387.2189	756.4039	378.7056	755.4199	378.2136	Q	517.2729	259.1401	500.2463	250.6268			4
7	***901.4890***	451.2482	884.4625	442.7349	883.4785	442.2429	Q	389.2143	195.1108	372.1878	186.5975			3
8	***1015.5320***	508.2696	998.5054	499.7563	997.5214	499.2643	N	**261.1557**	131.0815	244.1292	122.5682			2
9							K	147.1128	74.0600	130.0863	65.5468			1

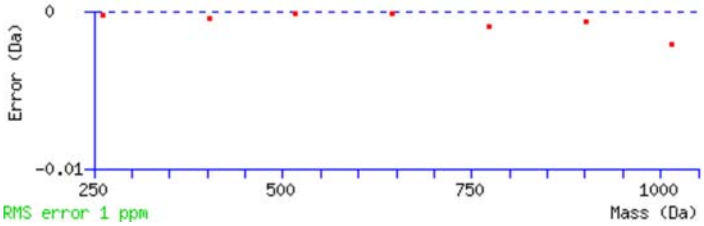

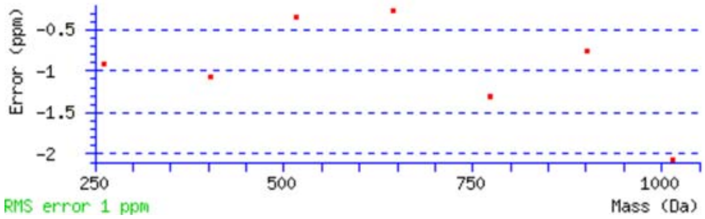

NCBI **BLAST** search of VRFLEQQNK
(Parameters: blastp, nr protein database, expect=20000, no filter, PAM30)
Other BLAST web gateways

All matches to this query

Score	Mr(calc)	Delta	Sequence
46.6	1160.6302	-0.0002	VRFLEQQNK
19.6	1160.6302	-0.0002	RINLFQQNK
12.9	1160.6223	0.0077	KCLADSLLNK
8.4	1160.6302	-0.0002	RINLFQQNK

Mascot: http://www.matrixscience.com/

Mascot Search Results

Peptide View

MS/MS Fragmentation of **LGLDIEIATYR**
Found in **ch12u_Q9NSB2|KRT84_HUMAN** in **uni_human**, Keratin, type II cuticular Hb4 OS=Homo sapiens GN=KRT84 PE=2 SV=2

Match to Query 10373: 1262.687108 from(632.350830,2+) intensity(49064860.0000) rtinseconds(2755) scans(15140) index(11942)
Title: 160219_Sunil_SDSI_A_Spectrum064071_scans__15140_RTINSECONDS=2755
Data file L:\\QE_2016\\160219_Sunil_KAP_LKC\\TMgf\\T\\T160219_Sunil_SDSI_A.mgf

Click mouse within plot area to zoom in by factor of two about that point
Or, Plot from 100 to 1200 Da Full range
Label all possible matches ○ Label matches used for scoring ◉
Show Y-axis ☐

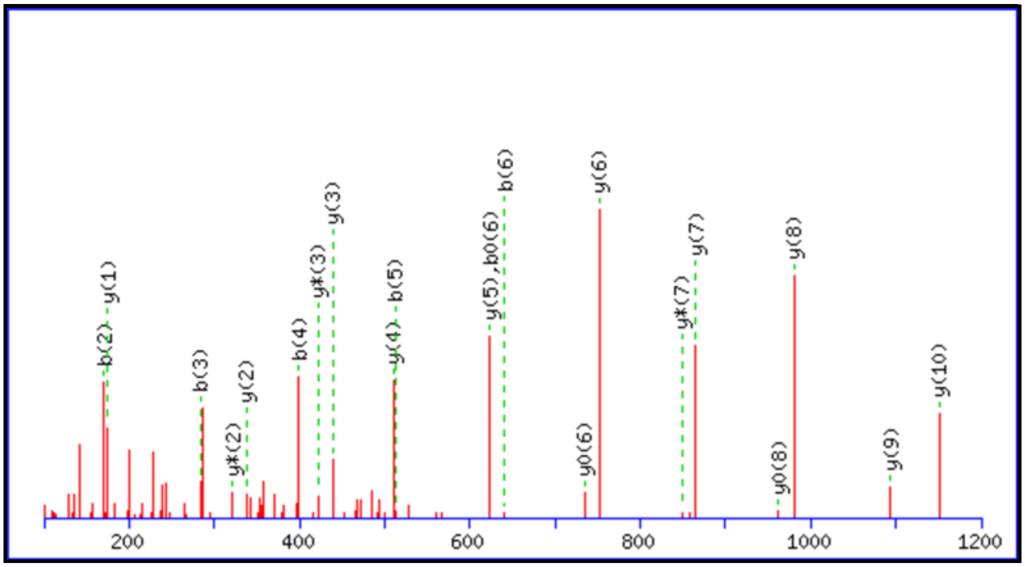

Monoisotopic mass of neutral peptide Mr(calc): 1262.6870
Fixed modifications: Carbamidomethyl (C) (apply to specified residues or termini only)
Ions Score: 84 **Expect:** 4.4e-007
Matches : 21/90 fragment ions using 31 most intense peaks (help)

#	b	b^{++}	b^0	b^{0++}	Seq.	y	y^{++}	y*	y^{*++}	y^0	y^{0++}	#
1	114.0913	57.5493			L							11
2	**171.1128**	86.0600			G	***1150.6103***	575.8088	1133.5837	567.2955	1132.5997	566.8035	10
3	**284.1969**	142.6021			L	***1093.5888***	547.2980	1076.5623	538.7848	1075.5782	538.2928	9
4	**399.2238**	200.1155	381.2132	191.1103	D	***980.5047***	490.7560	963.4782	482.2427	962.4942	481.7507	8
5	**512.3079**	256.6576	494.2973	247.6523	I	***865.4778***	433.2425	848.4512	424.7293	847.4672	424.2373	7
6	**641.3505**	321.1789	623.3399	312.1736	E	***752.3937***	376.7005	735.3672	368.1872	734.3832	367.6952	6
7	754.4345	377.7209	736.4240	368.7156	I	***623.3511***	312.1792	606.3246	303.6659	605.3406	303.1739	5
8	825.4716	413.2395	807.4611	404.2342	A	***510.2671***	255.6372	493.2405	247.1239	492.2565	246.6319	4
9	926.5193	463.7633	908.5088	454.7580	T	***439.2300***	220.1186	422.2034	211.6053	421.2194	211.1133	3

10	1089.5827	545.2950	1071.5721	536.2897	**Y**	*338.1823*	169.5948	321.1557	161.0815			2
11					**R**	*175.1190*	88.0631	158.0924	79.5498			1

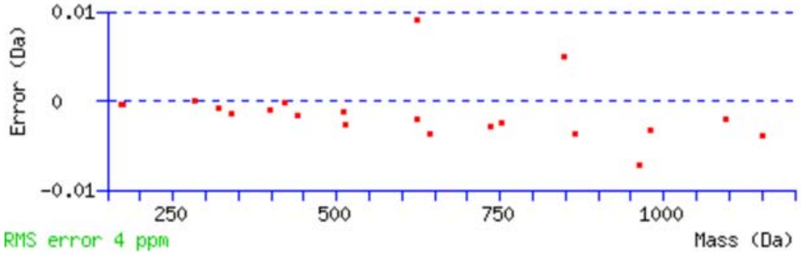

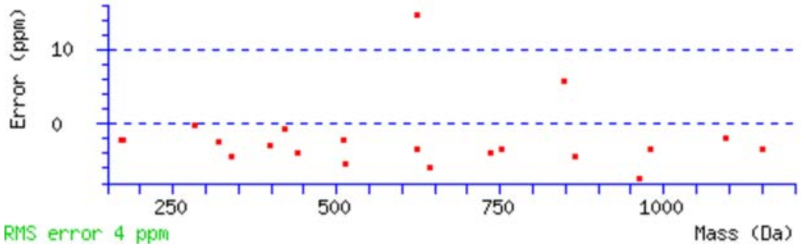

NCBI **BLAST** search of LGLDIEIATYR
(Parameters: blastp, nr protein database, expect=20000, no filter, PAM30)
Other BLAST web gateways

All matches to this query

Score	Mr(calc)	Delta	Sequence
83.7	1262.6870	0.0001	LGLDIEIATYR
32.6	1262.6870	0.0001	LALDVEIATYR
24.7	1262.6870	0.0001	IAVDELFTSLR
6.7	1262.6805	0.0066	VALLIGNMNYR
5.9	1262.6983	-0.0112	IGAQPVEIPPSR
5.3	1262.6983	-0.0112	IGDFGLATVKSR
5.2	1262.6758	0.0113	EVELIGASYIGL
2.2	1262.6884	-0.0013	GLVQRWRYGK
1.3	1262.6983	-0.0111	LVAAKAFTESAR
0.3	1261.6819	1.0052	DIGIWYNILR

Mascot: http://www.matrixscience.com/

Mascot Search Results

Peptide View

MS/MS Fragmentation of **LGLDIEIATYR**
Found in **ch12u_Q9NSB2|KRT84_HUMAN** in **uni_human**, Keratin, type II cuticular Hb4 OS=Homo sapiens GN=KRT84 PE=2 SV=2

Match to Query 10373: 1262.687108 from(632.350830,2+) intensity(49064860.0000) rtinseconds(2755) scans(15140) index(11942)
Title: 160219_Sunil_SDSI_A_Spectrum064071_scans__15140_RTINSECONDS=2755
Data file L:\\QE_2016\\160219_Sunil_KAP_LKC\\TMgf\\T\\T160219_Sunil_SDSI_A.mgf

Click mouse within plot area to zoom in by factor of two about that point
Or, Plot from 100 to 1200 Da Full range
Label all possible matches ○ Label matches used for scoring ◉
Show Y-axis ☐

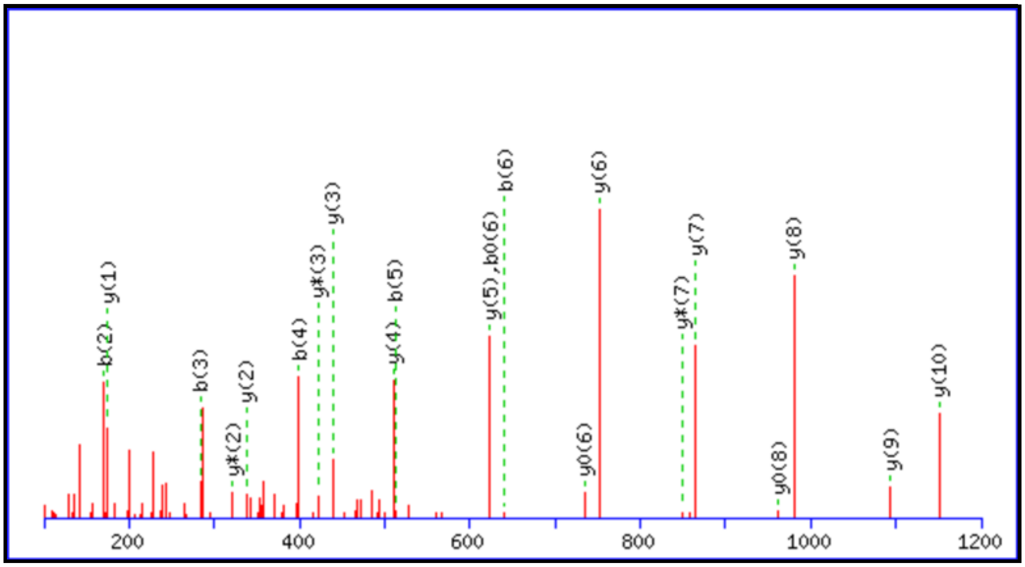

Monoisotopic mass of neutral peptide Mr(calc): 1262.6870
Fixed modifications: Carbamidomethyl (C) (apply to specified residues or termini only)
Ions Score: 84 **Expect:** 4.4e-007
Matches : 21/90 fragment ions using 31 most intense peaks (help)

#	b	b^{++}	b^0	b^{0++}	Seq.	y	y^{++}	y^*	y^{*++}	y^0	y^{0++}	#
1	114.0913	57.5493			L							11
2	**171.1128**	86.0600			G	***1150.6103***	575.8088	1133.5837	567.2955	1132.5997	566.8035	10
3	**284.1969**	142.6021			L	***1093.5888***	547.2980	1076.5623	538.7848	1075.5782	538.2928	9
4	**399.2238**	200.1155	381.2132	191.1103	D	***980.5047***	490.7560	963.4782	482.2427	962.4942	481.7507	8
5	**512.3079**	256.6576	494.2973	247.6523	I	***865.4778***	433.2425	848.4512	424.7293	847.4672	424.2373	7
6	**641.3505**	321.1789	623.3399	312.1736	E	***752.3937***	376.7005	735.3672	368.1872	734.3832	367.6952	6
7	754.4345	377.7209	736.4240	368.7156	I	***623.3511***	312.1792	606.3246	303.6659	605.3406	303.1739	5
8	825.4716	413.2395	807.4611	404.2342	A	***510.2671***	255.6372	493.2405	247.1239	492.2565	246.6319	4
9	926.5193	463.7633	908.5088	454.7580	T	***439.2300***	220.1186	422.2034	211.6053	421.2194	211.1133	3

**10**	1089.5827	545.2950	1071.5721	536.2897	**Y**	***338.1823***	169.5948	321.1557	161.0815			**2**
**11**					**R**	***175.1190***	88.0631	158.0924	79.5498			**1**

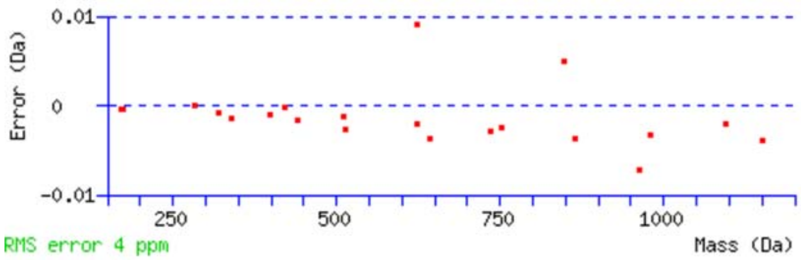

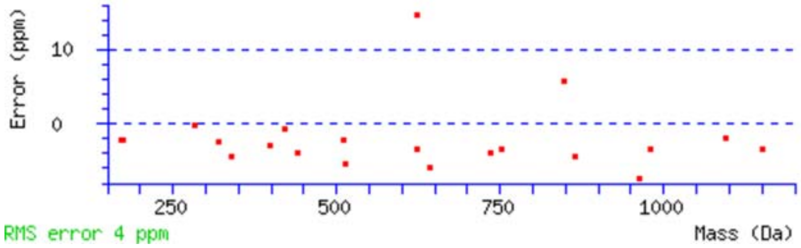

NCBI **BLAST** search of LGLDIEIATYR
(Parameters: blastp, nr protein database, expect=20000, no filter, PAM30)
Other BLAST web gateways

All matches to this query

Score	Mr(calc)	Delta	Sequence
83.7	1262.6870	0.0001	LGLDIEIATYR
32.6	1262.6870	0.0001	LALDVEIATYR
24.7	1262.6870	0.0001	IAVDELFTSLR
6.7	1262.6805	0.0066	VALLIGNMNYR
5.9	1262.6983	-0.0112	IGAQPVEIPPSR
5.3	1262.6983	-0.0112	IGDFGLATVKSR
5.2	1262.6758	0.0113	EVELIGASYIGL
2.2	1262.6884	-0.0013	GLVQRWRYGK
1.3	1262.6983	-0.0111	LVAAKAFTESAR
0.3	1261.6819	1.0052	DIGIWYNILR

Mascot: http://www.matrixscience.com/

Mascot Search Results

Peptide View

MS/MS Fragmentation of **LGLDIEIATYR**
Found in **ch12u_Q9NSB2|KRT84_HUMAN** in **uni_human**, Keratin, type II cuticular Hb4 OS=Homo sapiens GN=KRT84 PE=2 SV=2

Match to Query 10374: 1262.687728 from(632.351140,2+) intensity(1547500.2500) rtinseconds(2657) scans(14718) index(26918)
Title: 160219_Sunil_SDSI_A_Spectrum080226_scans__14718_RTINSECONDS=2657
Data file L:\\QE_2016\\160219_Sunil_KAP_LKC\\TMgf\\T\\T160219_Sunil_SDSI_A.mgf

Click mouse within plot area to zoom in by factor of two about that point
Or, Plot from 100 to 1200 Da Full range
Label all possible matches ○ Label matches used for scoring ◉
Show Y-axis ☐

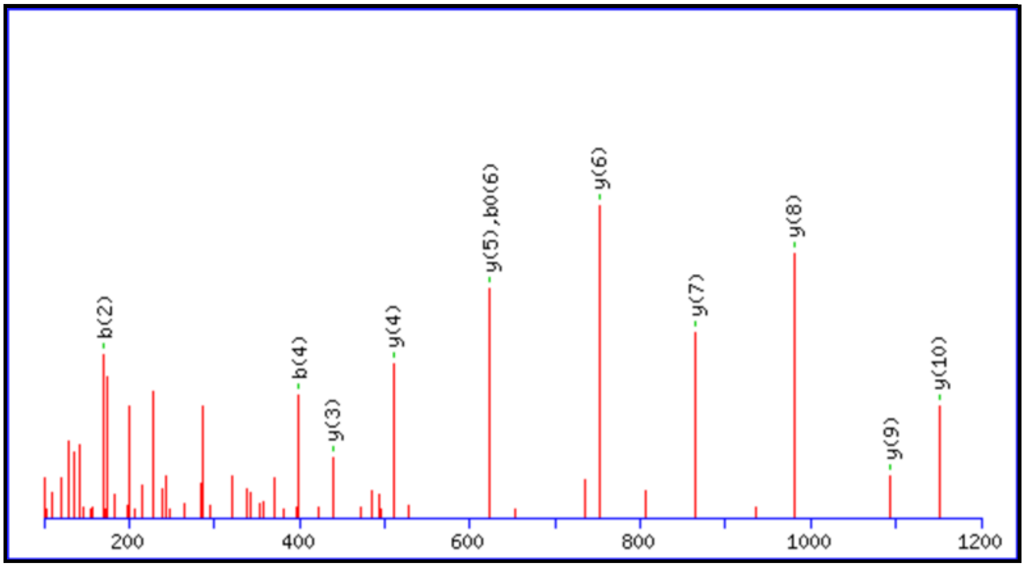

Monoisotopic mass of neutral peptide Mr(calc): 1262.6870
Fixed modifications: Carbamidomethyl (C) (apply to specified residues or termini only)
Ions Score: 82 **Expect:** 6.4e-007
Matches : 11/90 fragment ions using 11 most intense peaks (help)

#	b	b^{++}	b^0	b^{0++}	Seq.	y	y^{++}	y*	y^{*++}	y^0	y^{0++}	#
1	114.0913	57.5493			L							11
2	**171.1128**	86.0600			G	***1150.6103***	575.8088	1133.5837	567.2955	1132.5997	566.8035	10
3	284.1969	142.6021			L	***1093.5888***	547.2980	1076.5623	538.7848	1075.5782	538.2928	9
4	**399.2238**	200.1155	381.2132	191.1103	D	***980.5047***	490.7560	963.4782	482.2427	962.4942	481.7507	8
5	512.3079	256.6576	494.2973	247.6523	I	***865.4778***	433.2425	848.4512	424.7293	847.4672	424.2373	7
6	641.3505	321.1789	623.3399	312.1736	E	***752.3937***	376.7005	735.3672	368.1872	734.3832	367.6952	6
7	754.4345	377.7209	736.4240	368.7156	I	***623.3511***	312.1792	606.3246	303.6659	605.3406	303.1739	5
8	825.4716	413.2395	807.4611	404.2342	A	***510.2671***	255.6372	493.2405	247.1239	492.2565	246.6319	4
9	926.5193	463.7633	908.5088	454.7580	T	***439.2300***	220.1186	422.2034	211.6053	421.2194	211.1133	3

**10**	1089.5827	545.2950	1071.5721	536.2897	**Y**	338.1823	169.5948	321.1557	161.0815			**2**
**11**					**R**	175.1190	88.0631	158.0924	79.5498			**1**

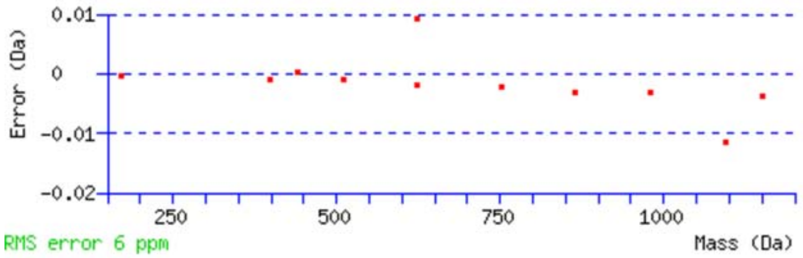

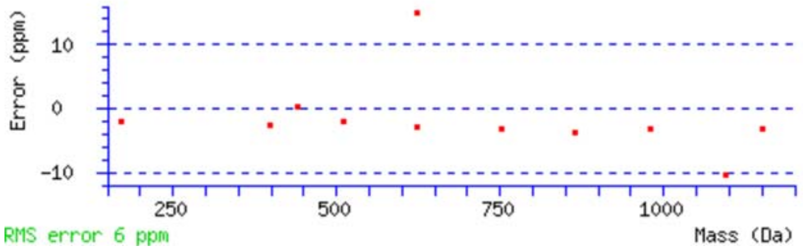

NCBI **BLAST** search of LGLDIEIATYR
(Parameters: blastp, nr protein database, expect=20000, no filter, PAM30)
Other BLAST web gateways

All matches to this query

Score	Mr(calc)	Delta	Sequence
82.2	1262.6870	0.0007	LGLDIEIATYR
31.9	1262.6870	0.0007	LALDVEIATYR
21.1	1262.6870	0.0007	IAVDELFTSLR
6.5	1262.6805	0.0072	VALLIGNMNYR
2.3	1262.6983	-0.0106	IGDFGLATVKSR
1.9	1262.6884	-0.0006	GLVQRWRYGK
1.3	1262.6983	-0.0105	LVAAKAFTESAR
0.5	1261.6819	1.0058	DIGIWYNILR

Mascot: http://www.matrixscience.com/

Mascot Search Results

Peptide View

MS/MS Fragmentation of **ANAENEFVALKK**
Found in **ch12u_Q9NSB2|KRT84_HUMAN** in **uni_human**, Keratin, type II cuticular Hb4 OS=Homo sapiens GN=KRT84 PE=2 SV=2

Match to Query 12416: 1333.688328 from(667.851440,2+) intensity(1229037.2500) rtinseconds(2364) scans(12856) index(10071)
Title: 160219_Sunil_SDSI_A_Spectrum062191_scans__12856_RTINSECONDS=2364
Data file L:\\QE_2016\\160219_Sunil_KAP_LKC\\TMgf\\T\\T160219_Sunil_SDSI_A.mgf

Click mouse within plot area to zoom in by factor of two about that point
Or, Plot from 50 to 1100 Da Full range
Label all possible matches ○ Label matches used for scoring ◉
Show Y-axis □

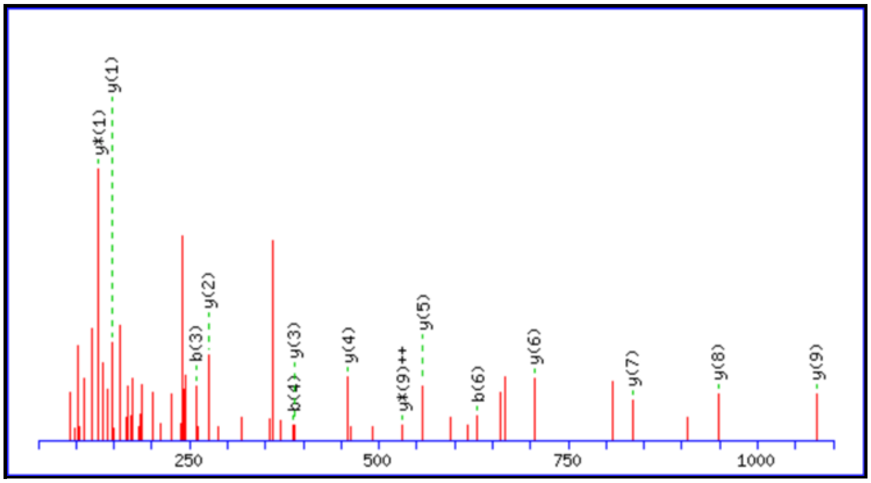

Monoisotopic mass of neutral peptide Mr(calc): 1333.6877
Fixed modifications: Carbamidomethyl (C) (apply to specified residues or termini only)
Variable modifications:
N2 : Deamidated (NQ)
Ions Score: 47 **Expect:** 0.0032
Matches : 14/112 fragment ions using 35 most intense peaks (help)

#	b	b++	b*	b*++	b0	b0++	Seq.	y	y++	y*	y*++	y0	y0++	#
1	72.0444	36.5258					A							12
2	187.0713	94.0393	170.0448	85.5260			N	1263.6579	632.3326	1246.6314	623.8193	1245.6474	623.3273	11
3	**258.1084**	129.5579	241.0819	121.0446			A	1148.6310	574.8191	1131.6045	566.3059	1130.6204	565.8139	10
4	**387.1510**	194.0792	370.1245	185.5659	369.1405	185.0739	E	***1077.5939***	539.3006	1060.5673	530.7873	1059.5833	530.2953	9
5	501.1940	251.1006	484.1674	242.5873	483.1834	242.0953	N	***948.5513***	474.7793	931.5247	466.2660	930.5407	465.7740	8
6	**630.2366**	315.6219	613.2100	307.1086	612.2260	306.6166	E	***834.5084***	417.7578	817.4818	409.2445	816.4978	408.7525	7
7	777.3050	389.1561	760.2784	380.6429	759.2944	380.1508	F	***705.4658***	353.2365	688.4392	344.7232			6
8	876.3734	438.6903	859.3468	430.1771	858.3628	429.6850	V	***558.3974***	279.7023	541.3708	271.1890			5
9	947.4105	474.2089	930.3840	465.6956	929.3999	465.2036	A	***459.3289***	230.1681	442.3024	221.6548			4
10	1060.4946	530.7509	1043.4680	522.2376	1042.4840	521.7456	L	***388.2918***	194.6496	371.2653	186.1363			3
11	1188.5895	594.7984	1171.5630	586.2851	1170.5790	585.7931	K	***275.2078***	138.1075	258.1812	129.5942			2
12							K	***147.1128***	74.0600	130.0863	65.5468			1

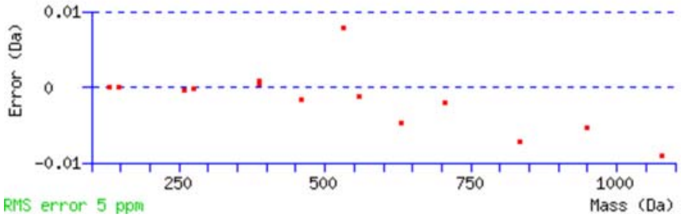

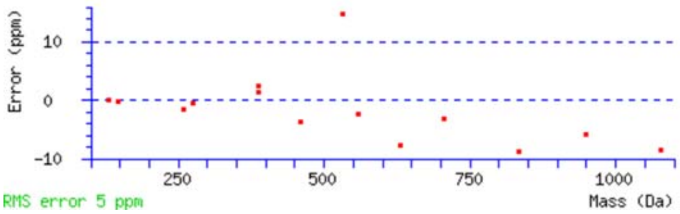

NCBI **BLAST** search of ANAENEFVALKK
(Parameters: blastp, nr protein database, expect=20000, no filter, PAM30)
Other BLAST web gateways

All matches to this query

Score	Mr(calc)	Delta	Sequence	Site Analysis
47.0	1333.6877	0.0006	ANAENEFVALKK	Deamidated N2 99.41%
24.8	1333.6877	0.0006	ANAENEFVALKK	Deamidated N5 0.59%
4.3	1333.6911	-0.0028	DKDTGEMVALKK	

Mascot: http://www.matrixscience.com/

Mascot Search Results

Peptide View

MS/MS Fragmentation of **LGLDIEIATYRR**
Found in **ch12u_Q9NSB2|KRT84_HUMAN** in **uni_human**, Keratin, type II cuticular Hb4 OS=Homo sapiens GN=KRT84 PE=2 SV=2

Match to Query 15008: 1418.788572 from(473.936800,3+) intensity(5597850.5000) rtinseconds(2514) scans(13732) index(10758)
Title: 160219_Sunil_SDSI_A_Spectrum062881_scans__13732_RTINSECONDS=2514
Data file L:\\QE_2016\\160219_Sunil_KAP_LKC\\TMgf\\T\\T160219_Sunil_SDSI_A.mgf

Click mouse within plot area to zoom in by factor of two about that point
Or, Plot from 100 to 1400 Da Full range
Label all possible matches ○ Label matches used for scoring ◉
Show Y-axis ☐

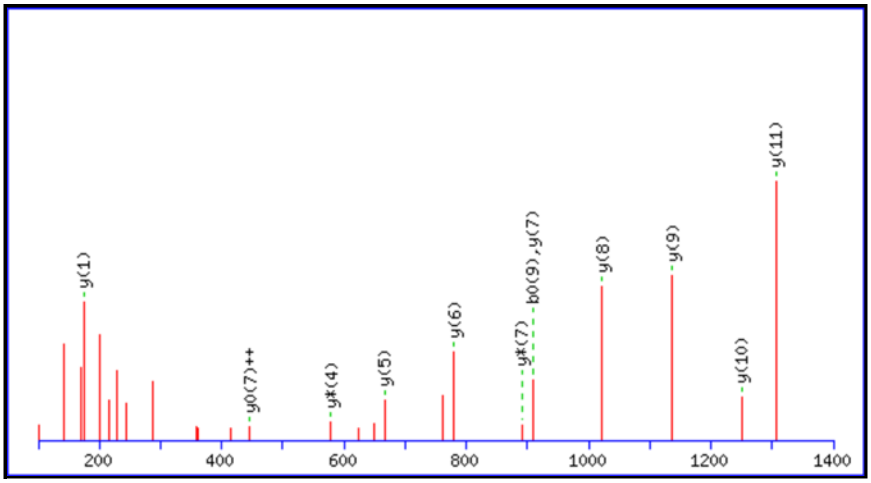

Monoisotopic mass of neutral peptide Mr(calc): 1418.7881
Fixed modifications: Carbamidomethyl (C) (apply to specified residues or termini only)
Ions Score: 76 **Expect:** 2.3e-006
Matches : 12/100 fragment ions using 13 most intense peaks (help)

#	b	b^{++}	b*	b^{*++}	b^0	b^{0++}	Seq.	y	y^{++}	y*	y^{*++}	y^0	y^{0++}	#
1	114.0913	57.5493					L							12
2	171.1128	86.0600					G	***1306.7114***	653.8593	1289.6848	645.3461	1288.7008	644.8540	11
3	284.1969	142.6021					L	***1249.6899***	625.3486	1232.6634	616.8353	1231.6793	616.3433	10
4	399.2238	200.1155			381.2132	191.1103	D	***1136.6058***	568.8066	1119.5793	560.2933	1118.5953	559.8013	9
5	512.3079	256.6576			494.2973	247.6523	I	***1021.5789***	511.2931	1004.5524	502.7798	1003.5683	502.2878	8
6	641.3505	321.1789			623.3399	312.1736	E	***908.4948***	454.7511	891.4683	446.2378	890.4843	445.7458	7
7	754.4345	377.7209			736.4240	368.7156	I	***779.4522***	390.2298	762.4257	381.7165	761.4417	381.2245	6
8	825.4716	413.2395			807.4611	404.2342	A	***666.3682***	333.6877	649.3416	325.1745	648.3576	324.6824	5
9	926.5193	463.7633			908.5088	454.7580	T	595.3311	298.1692	578.3045	289.6559	577.3205	289.1639	4
10	1089.5827	545.2950			1071.5721	536.2897	Y	494.2834	247.6453	477.2568	239.1321			3
11	1245.6838	623.3455	1228.6572	614.8322	1227.6732	614.3402	R	331.2201	166.1137	314.1935	157.6004			2
12							R	***175.1190***	88.0631	158.0924	79.5498			1

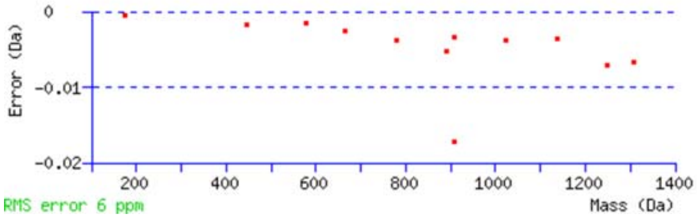

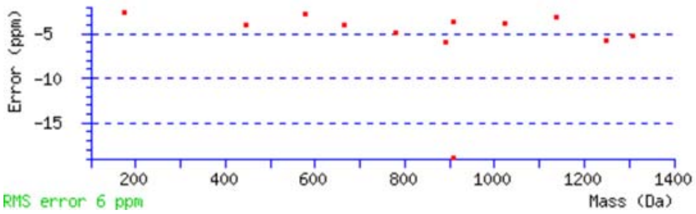

NCBI **BLAST** search of LGLDIEIATYRR
(Parameters: blastp, nr protein database, expect=20000, no filter, PAM30)
Other BLAST web gateways

All matches to this query

Score	Mr(calc)	Delta	Sequence
76.1	1418.7881	0.0004	LGLDIEIATYRR
28.1	1418.7881	0.0004	LGLLDRIVNYSR
14.1	1418.7769	0.0117	LLGNTFVALSDLR
8.1	1418.7881	0.0005	LQLRSLQYLER
5.5	1418.7769	0.0117	GLVLQLIQSYQR
5.5	1418.7769	0.0117	GLVLQLIQSYQR
2.6	1418.7994	-0.0108	LGPESVPPPKRSR

Mascot: http://www.matrixscience.com/

Mascot Search Results

Peptide View

MS/MS Fragmentation of **LGLDIEIATYRR**
Found in **ch12u_Q9NSB2|KRT84_HUMAN** in **uni_human**, Keratin, type II cuticular Hb4 OS=Homo sapiens GN=KRT84 PE=2 SV=2

Match to Query 14977: 1418.787372 from(473.936400,3+) intensity(1745967.7500) rtinseconds(2361) scans(12843) index(10060)
Title: 160219_Sunil_SDSI_A_Spectrum062180_scans__12843_RTINSECONDS=2361
Data file L:\\QE_2016\\160219_Sunil_KAP_LKC\\TMgf\\T\\T160219_Sunil_SDSI_A.mgf

Click mouse within plot area to zoom in by factor of two about that point
Or, Plot from 100 to 1400 Da Full range
Label all possible matches ○ Label matches used for scoring ◉
Show Y-axis ☐

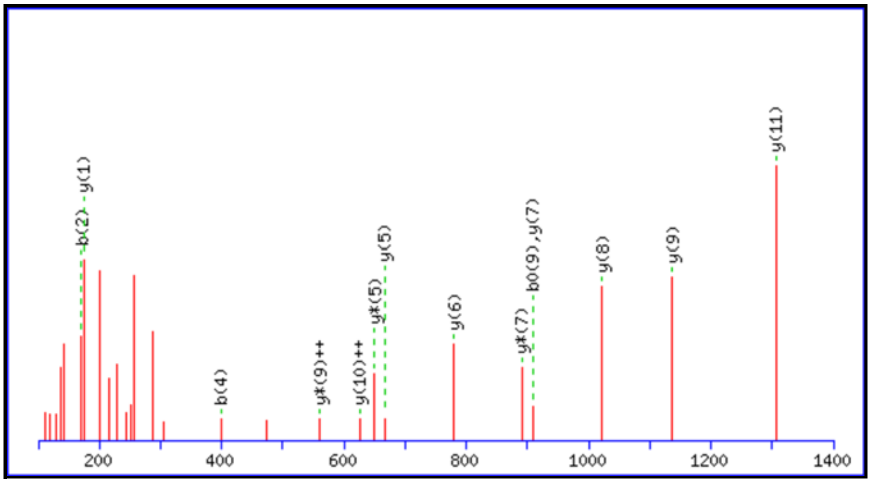

Monoisotopic mass of neutral peptide Mr(calc): 1418.7881
Fixed modifications: Carbamidomethyl (C) (apply to specified residues or termini only)
Ions Score: 55 **Expect:** 0.00031
Matches : 14/100 fragment ions using 19 most intense peaks (help)

#	b	b^{++}	b*	b^{*++}	b^0	b^{0++}	Seq.	y	y^{++}	y*	y^{*++}	y^0	y^{0++}	#
1	114.0913	57.5493					L							12
2	**171.1128**	86.0600					G	***1306.7114***	653.8593	1289.6848	645.3461	1288.7008	644.8540	11
3	284.1969	142.6021					L	1249.6899	625.3486	1232.6634	616.8353	1231.6793	616.3433	10
4	**399.2238**	200.1155			381.2132	191.1103	D	***1136.6058***	568.8066	1119.5793	560.2933	1118.5953	559.8013	9
5	512.3079	256.6576			494.2973	247.6523	I	***1021.5789***	511.2931	1004.5524	502.7798	1003.5683	502.2878	8
6	641.3505	321.1789			623.3399	312.1736	E	***908.4948***	454.7511	891.4683	446.2378	890.4843	445.7458	7
7	754.4345	377.7209			736.4240	368.7156	I	***779.4522***	390.2298	762.4257	381.7165	761.4417	381.2245	6
8	825.4716	413.2395			807.4611	404.2342	A	***666.3682***	333.6877	649.3416	325.1745	648.3576	324.6824	5
9	926.5193	463.7633			908.5088	454.7580	T	595.3311	298.1692	578.3045	289.6559	577.3205	289.1639	4
10	1089.5827	545.2950			1071.5721	536.2897	Y	494.2834	247.6453	477.2568	239.1321			3
11	1245.6838	623.3455	1228.6572	614.8322	1227.6732	614.3402	R	331.2201	166.1137	314.1935	157.6004			2
12							R	***175.1190***	88.0631	158.0924	79.5498			1

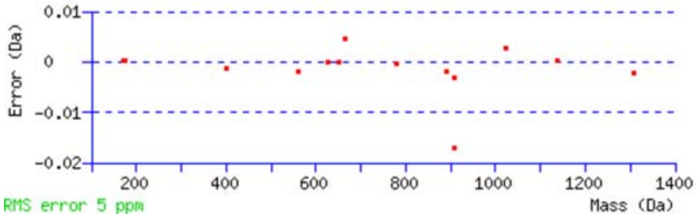

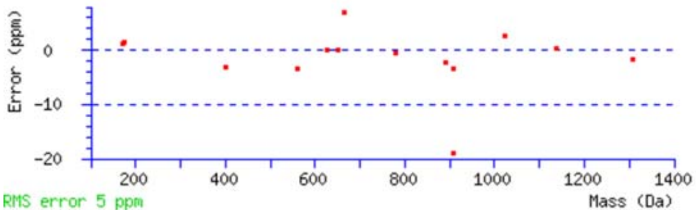

NCBI **BLAST** search of LGLDIEIATYRR
(Parameters: blastp, nr protein database, expect=20000, no filter, PAM30)
Other BLAST web gateways

All matches to this query

Score	Mr(calc)	Delta	Sequence
54.8	1418.7881	-0.0008	LGLDIEIATYRR
20.1	1418.7769	0.0105	LLGNTFVALSDLR
16.2	1418.7881	-0.0008	LGLLDRIVNYSR
7.9	1418.7881	-0.0007	LQLRSLQYLER

Mascot: http://www.matrixscience.com/

Mascot Search Results

Peptide View

MS/MS Fragmentation of **LGLDIEIATYRR**
Found in **ch12u_Q9NSB2|KRT84_HUMAN** in **uni_human**, Keratin, type II cuticular Hb4 OS=Homo sapiens GN=KRT84 PE=2 SV=2

Match to Query 14981: 1418.787732 from(473.936520,3+) intensity(2230058.2500) rtinseconds(2646) scans(14499) index(11390)
Title: 160219_Sunil_SDSI_A_Spectrum063517_scans__14499_RTINSECONDS=2646
Data file L:\\QE_2016\\160219_Sunil_KAP_LKC\\TMgf\\T\\T160219_Sunil_SDSI_A.mgf

Click mouse within plot area to zoom in by factor of two about that point
Or, Plot from 100 to 1400 Da Full range
Label all possible matches ○ Label matches used for scoring ◉
Show Y-axis ☐

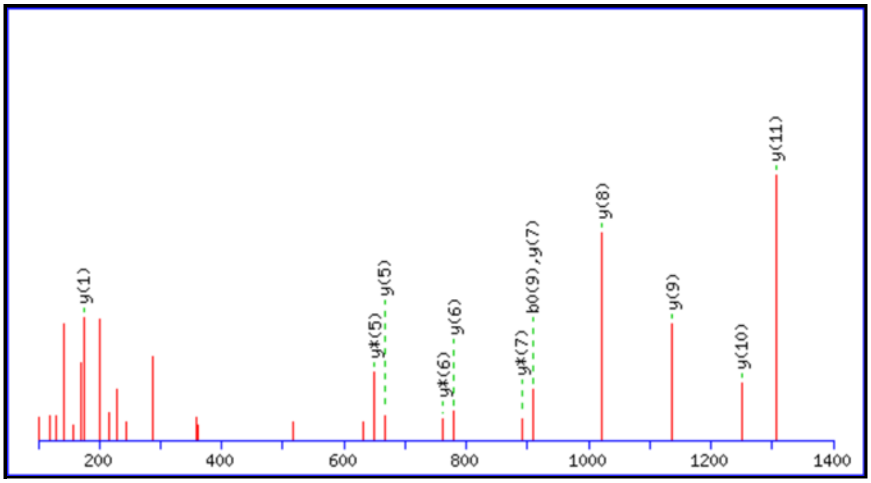

Monoisotopic mass of neutral peptide Mr(calc): 1418.7881
Fixed modifications: Carbamidomethyl (C) (apply to specified residues or termini only)
Ions Score: 63 **Expect:** 4.8e-005
Matches : 12/100 fragment ions using 17 most intense peaks (help)

#	b	b^{++}	b*	b^{*++}	b^0	b^{0++}	Seq.	y	y^{++}	y*	y^{*++}	y^0	y^{0++}	#
1	114.0913	57.5493					L							12
2	171.1128	86.0600					G	***1306.7114***	653.8593	1289.6848	645.3461	1288.7008	644.8540	11
3	284.1969	142.6021					L	***1249.6899***	625.3486	1232.6634	616.8353	1231.6793	616.3433	10
4	399.2238	200.1155			381.2132	191.1103	D	***1136.6058***	568.8066	1119.5793	560.2933	1118.5953	559.8013	9
5	512.3079	256.6576			494.2973	247.6523	I	***1021.5789***	511.2931	1004.5524	502.7798	1003.5683	502.2878	8
6	641.3505	321.1789			623.3399	312.1736	E	***908.4948***	454.7511	891.4683	446.2378	890.4843	445.7458	7
7	754.4345	377.7209			736.4240	368.7156	I	***779.4522***	390.2298	762.4257	381.7165	761.4417	381.2245	6
8	825.4716	413.2395			807.4611	404.2342	A	***666.3682***	333.6877	649.3416	325.1745	648.3576	324.6824	5
9	926.5193	463.7633			908.5088	454.7580	T	595.3311	298.1692	578.3045	289.6559	577.3205	289.1639	4
10	1089.5827	545.2950			1071.5721	536.2897	Y	494.2834	247.6453	477.2568	239.1321			3
11	1245.6838	623.3455	1228.6572	614.8322	1227.6732	614.3402	R	331.2201	166.1137	314.1935	157.6004			2
12							R	***175.1190***	88.0631	158.0924	79.5498			1

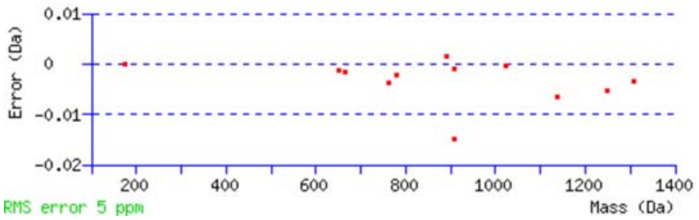

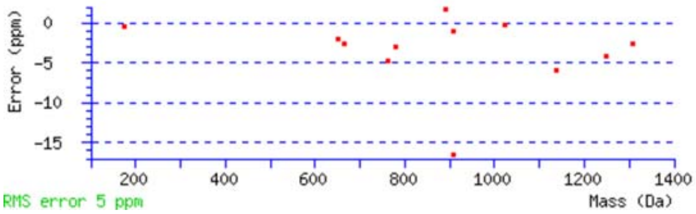

NCBI **BLAST** search of LGLDIEIATYRR
(Parameters: blastp, nr protein database, expect=20000, no filter, PAM30)
Other BLAST web gateways

All matches to this query

Score	Mr(calc)	Delta	Sequence
62.9	1418.7881	-0.0004	LGLDIEIATYRR
27.0	1418.7881	-0.0004	LGLLDRIVNYSR
15.2	1418.7769	0.0108	LLGNTFVALSDLR
9.4	1418.7881	-0.0004	LQLRSLQYLER
7.4	1418.7742	0.0135	LGEARHPQVSLGR
7.3	1418.7769	0.0108	GLVLQLIQSYQR
4.8	1418.7769	0.0108	GLVLQLIQSYQR
3.1	1418.7994	-0.0116	LGPESVPPPKRSR
0.4	1418.7769	0.0108	LYVALKELGEER
0.4	1418.7881	-0.0004	LQLRSLQYLER

Mascot: http://www.matrixscience.com/

Mascot Search Results

Peptide View

MS/MS Fragmentation of **LGLDIEIATYRR**
Found in **ch12u_Q9NSB2|KRT84_HUMAN** in **uni_human**, Keratin, type II cuticular Hb4 OS=Homo sapiens GN=KRT84 PE=2 SV=2

Match to Query 14989: 1418.788212 from(473.936680,3+) intensity(3337706.7500) rtinseconds(2419) scans(13176) index(10326)
Title: 160219_Sunil_SDSI_A_Spectrum062447_scans__13176_RTINSECONDS=2419
Data file L:\\QE_2016\\160219_Sunil_KAP_LKC\\TMgf\\T\\T160219_Sunil_SDSI_A.mgf

Click mouse within plot area to zoom in by factor of two about that point
Or, Plot from 100 to 1400 Da Full range
Label all possible matches ○ Label matches used for scoring ◉
Show Y-axis ☐

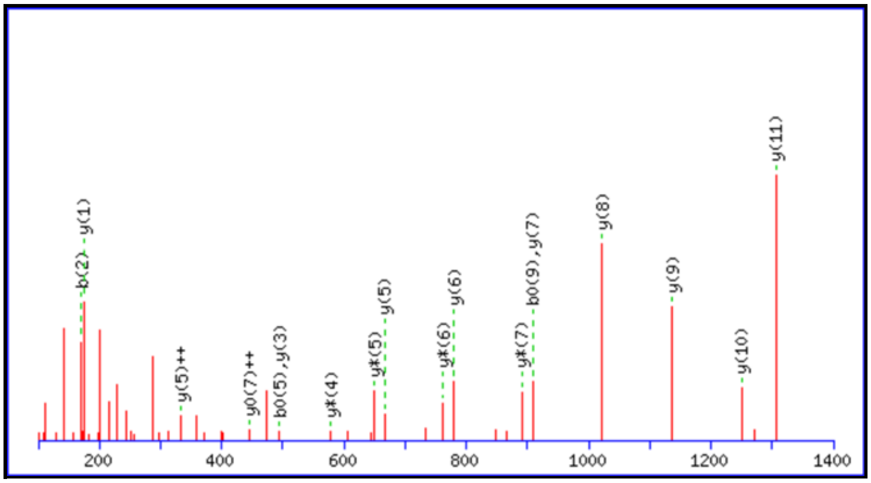

Monoisotopic mass of neutral peptide Mr(calc): 1418.7881
Fixed modifications: Carbamidomethyl (C) (apply to specified residues or termini only)
Ions Score: 67 **Expect:** 1.7e-005
Matches : 18/100 fragment ions using 29 most intense peaks (help)

#	b	b^{++}	b*	b^{*++}	b^0	b^{0++}	Seq.	y	y^{++}	y*	y^{*++}	y^0	y^{0++}	#
1	114.0913	57.5493					L							12
2	**171.1128**	86.0600					G	***1306.7114***	653.8593	1289.6848	645.3461	1288.7008	644.8540	11
3	284.1969	142.6021					L	***1249.6899***	625.3486	1232.6634	616.8353	1231.6793	616.3433	10
4	399.2238	200.1155			381.2132	191.1103	D	***1136.6058***	568.8066	1119.5793	560.2933	1118.5953	559.8013	9
5	512.3079	256.6576			494.2973	247.6523	I	***1021.5789***	511.2931	1004.5524	502.7798	1003.5683	502.2878	8
6	641.3505	321.1789			623.3399	312.1736	E	***908.4948***	454.7511	891.4683	446.2378	890.4843	445.7458	7
7	754.4345	377.7209			736.4240	368.7156	I	***779.4522***	390.2298	762.4257	381.7165	761.4417	381.2245	6
8	825.4716	413.2395			807.4611	404.2342	A	***666.3682***	333.6877	649.3416	325.1745	648.3576	324.6824	5
9	926.5193	463.7633			908.5088	454.7580	T	595.3311	298.1692	578.3045	289.6559	577.3205	289.1639	4
10	1089.5827	545.2950			1071.5721	536.2897	Y	***494.2834***	247.6453	477.2568	239.1321			3
11	1245.6838	623.3455	1228.6572	614.8322	1227.6732	614.3402	R	331.2201	166.1137	314.1935	157.6004			2
12							R	***175.1190***	88.0631	158.0924	79.5498			1

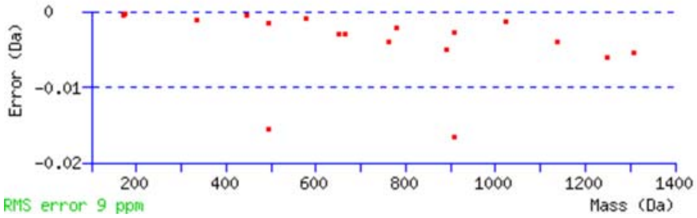

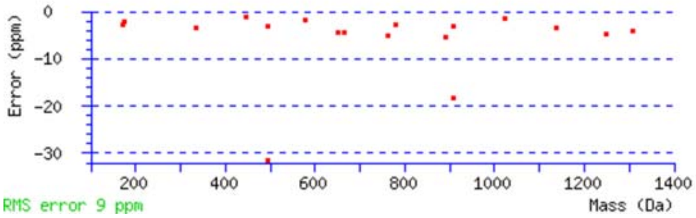

NCBI **BLAST** search of LGLDIEIATYRR
(Parameters: blastp, nr protein database, expect=20000, no filter, PAM30)
Other BLAST web gateways

All matches to this query

Score	Mr(calc)	Delta	Sequence
67.4	1418.7881	0.0001	LGLDIEIATYRR
24.9	1418.7881	0.0001	LGLLDRIVNYSR
13.5	1418.7769	0.0113	LLGNTFVALSDLR
3.5	1418.7994	-0.0112	LGPESVPPPKRSR
3.0	1418.7881	0.0001	LQLRSLQYLER
2.0	1418.7742	0.0140	LGEARHPQVSLGR
0.7	1418.7769	0.0113	GLVLQLIQSYQR
0.7	1418.7769	0.0113	GLVLQLIQSYQR

Mascot: http://www.matrixscience.com/

Mascot Search Results

Peptide View

MS/MS Fragmentation of **LGLDIEIATYRR**
Found in **ch12u_Q9NSB2|KRT84_HUMAN** in **uni_human**, Keratin, type II cuticular Hb4 OS=Homo sapiens GN=KRT84 PE=2 SV=2

Match to Query 14994: 1418.788302 from(473.936710,3+) intensity(1524156.3750) rtinseconds(2678) scans(14688) index(11559)
Title: 160219_Sunil_SDSI_A_Spectrum063687_scans__14688_RTINSECONDS=2678
Data file L:\\QE_2016\\160219_Sunil_KAP_LKC\\TMgf\\T\\T160219_Sunil_SDSI_A.mgf

Click mouse within plot area to zoom in by factor of two about that point
Or, Plot from 100 to 1400 Da Full range
Label all possible matches ○ Label matches used for scoring ◉
Show Y-axis ☐

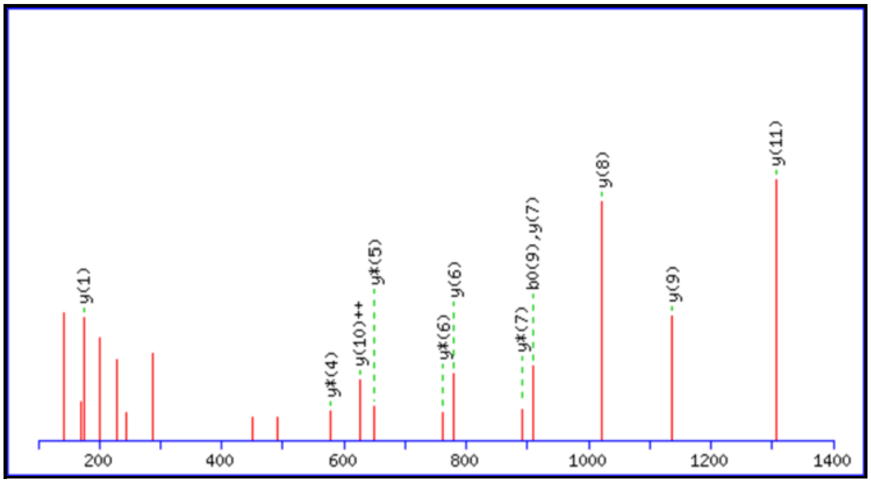

Monoisotopic mass of neutral peptide Mr(calc): 1418.7881
Fixed modifications: Carbamidomethyl (C) (apply to specified residues or termini only)
Ions Score: 44 **Expect:** 0.004
Matches : 12/100 fragment ions using 16 most intense peaks (help)

#	b	b^{++}	b*	b^{*++}	b^0	b^{0++}	Seq.	y	y^{++}	y*	y^{*++}	y^0	y^{0++}	#
1	114.0913	57.5493					L							12
2	171.1128	86.0600					G	***1306.7114***	653.8593	1289.6848	645.3461	1288.7008	644.8540	11
3	284.1969	142.6021					L	1249.6899	625.3486	1232.6634	616.8353	1231.6793	616.3433	10
4	399.2238	200.1155			381.2132	191.1103	D	***1136.6058***	568.8066	1119.5793	560.2933	1118.5953	559.8013	9
5	512.3079	256.6576			494.2973	247.6523	I	***1021.5789***	511.2931	1004.5524	502.7798	1003.5683	502.2878	8
6	641.3505	321.1789			623.3399	312.1736	E	***908.4948***	454.7511	891.4683	446.2378	890.4843	445.7458	7
7	754.4345	377.7209			736.4240	368.7156	I	***779.4522***	390.2298	762.4257	381.7165	761.4417	381.2245	6
8	825.4716	413.2395			807.4611	404.2342	A	666.3682	333.6877	649.3416	325.1745	648.3576	324.6824	5
9	926.5193	463.7633			908.5088	454.7580	T	595.3311	298.1692	578.3045	289.6559	577.3205	289.1639	4
10	1089.5827	545.2950			1071.5721	536.2897	Y	494.2834	247.6453	477.2568	239.1321			3
11	1245.6838	623.3455	1228.6572	614.8322	1227.6732	614.3402	R	331.2201	166.1137	314.1935	157.6004			2
12							R	***175.1190***	88.0631	158.0924	79.5498			1

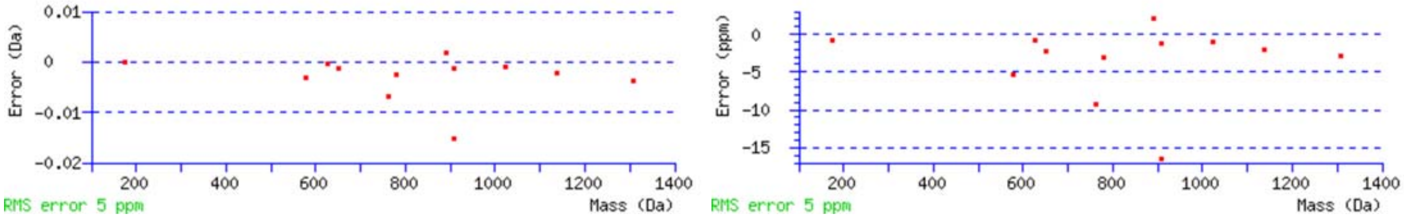

NCBI **BLAST** search of LGLDIEIATYRR
(Parameters: blastp, nr protein database, expect=20000, no filter, PAM30)
Other BLAST web gateways

All matches to this query

Score	Mr(calc)	Delta	Sequence
43.7	1418.7881	0.0002	LGLDIEIATYRR
14.5	1418.7881	0.0002	LGLLDRIVNYSR
11.9	1418.7769	0.0114	LLGNTFVALSDLR
8.1	1418.7881	0.0002	LQLRSLQYLER

Mascot: http://www.matrixscience.com/

Mascot Search Results

Peptide View

MS/MS Fragmentation of **LGLDIEIATYRR**
Found in **ch12u_Q9NSB2|KRT84_HUMAN** in **uni_human**, Keratin, type II cuticular Hb4 OS=Homo sapiens GN=KRT84 PE=2 SV=2

Match to Query 15002: 1418.788482 from(473.936770,3+) intensity(20338996.0000) rtinseconds(2297) scans(12614) index(25331)
Title: 160219_Sunil_SDSI_A_Spectrum078637_scans__12614_RTINSECONDS=2297
Data file L:\\QE_2016\\160219_Sunil_KAP_LKC\\TMgf\\T\\T160219_Sunil_SDSI_A.mgf

Click mouse within plot area to zoom in by factor of two about that point
Or, Plot from 100 to 1500 Da Full range
Label all possible matches ○ Label matches used for scoring ◉
Show Y-axis ☐

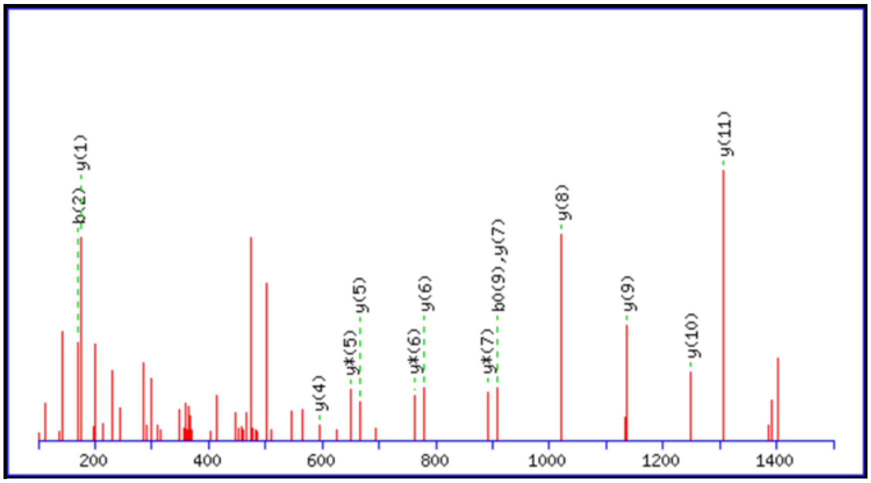

Monoisotopic mass of neutral peptide Mr(calc): 1418.7881
Fixed modifications: Carbamidomethyl (C) (apply to specified residues or termini only)
Ions Score: 52 **Expect:** 0.00056
Matches : 14/100 fragment ions using 31 most intense peaks (help)

#	b	b^{++}	b*	b^{*++}	b^0	b^{0++}	Seq.	y	y^{++}	y*	y^{*++}	y^0	y^{0++}	#
1	114.0913	57.5493					L							12
2	171.1128	86.0600					G	1306.7114	653.8593	1289.6848	645.3461	1288.7008	644.8540	11
3	284.1969	142.6021					L	1249.6899	625.3486	1232.6634	616.8353	1231.6793	616.3433	10
4	399.2238	200.1155			381.2132	191.1103	D	1136.6058	568.8066	1119.5793	560.2933	1118.5953	559.8013	9
5	512.3079	256.6576			494.2973	247.6523	I	1021.5789	511.2931	1004.5524	502.7798	1003.5683	502.2878	8
6	641.3505	321.1789			623.3399	312.1736	E	908.4948	454.7511	891.4683	446.2378	890.4843	445.7458	7
7	754.4345	377.7209			736.4240	368.7156	I	779.4522	390.2298	762.4257	381.7165	761.4417	381.2245	6
8	825.4716	413.2395			807.4611	404.2342	A	666.3682	333.6877	649.3416	325.1745	648.3576	324.6824	5
9	926.5193	463.7633			908.5088	454.7580	T	595.3311	298.1692	578.3045	289.6559	577.3205	289.1639	4
10	1089.5827	545.2950			1071.5721	536.2897	Y	494.2834	247.6453	477.2568	239.1321			3
11	1245.6838	623.3455	1228.6572	614.8322	1227.6732	614.3402	R	331.2201	166.1137	314.1935	157.6004			2
12							R	175.1190	88.0631	158.0924	79.5498			1

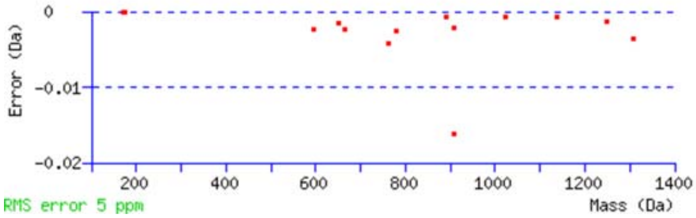

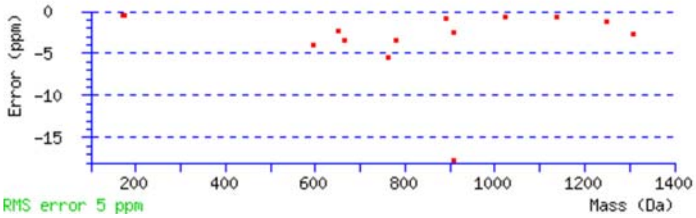

NCBI **BLAST** search of LGLDIEIATYRR
(Parameters: blastp, nr protein database, expect=20000, no filter, PAM30)
Other BLAST web gateways

All matches to this query

Score	Mr(calc)	Delta	Sequence
52.2	1418.7881	0.0004	LGLDIEIATYRR
22.2	1418.7881	0.0004	LGLLDRIVNYSR
11.3	1418.7769	0.0116	LLGNTFVALSDLR
6.3	1418.7881	0.0004	LQLRSLQYLER
4.7	1418.7769	0.0116	GLVLQLIQSYQR
0.8	1418.7994	-0.0109	LGPESVPPPKRSR

Mascot: http://www.matrixscience.com/

Mascot Search Results

Peptide View

MS/MS Fragmentation of **LGLDIEIATYRR**
Found in **ch12u_Q9NSB2|KRT84_HUMAN** in **uni_human**, Keratin, type II cuticular Hb4 OS=Homo sapiens GN=KRT84 PE=2 SV=2

Match to Query 15009: 1418.788572 from(473.936800,3+) intensity(5009538.5000) rtinseconds(2579) scans(14106) index(11048)
Title: 160219_Sunil_SDSI_A_Spectrum063174_scans__14106_RTINSECONDS=2579
Data file L:\\QE_2016\\160219_Sunil_KAP_LKC\\TMgf\\T\\T160219_Sunil_SDSI_A.mgf

Click mouse within plot area to zoom in by factor of two about that point
Or, Plot from 100 to 1400 Da Full range
Label all possible matches ○ Label matches used for scoring ◉
Show Y-axis ☐

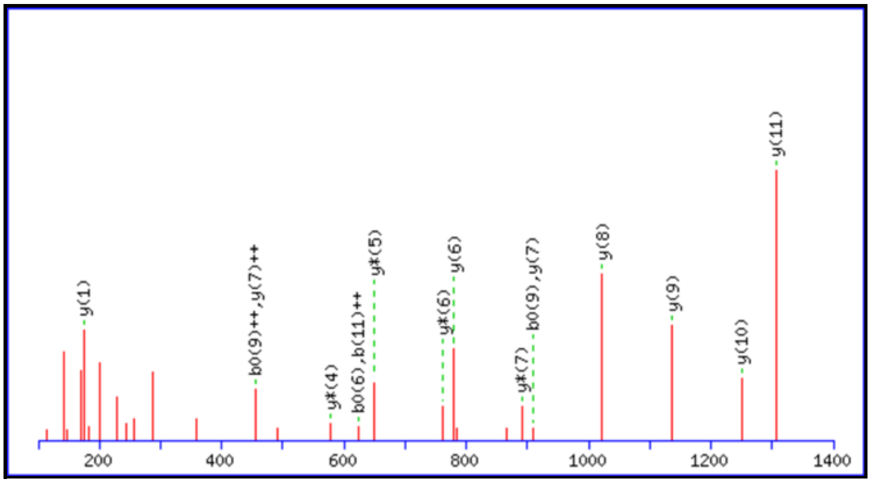

Monoisotopic mass of neutral peptide Mr(calc): 1418.7881
Fixed modifications: Carbamidomethyl (C) (apply to specified residues or termini only)
Ions Score: 55 **Expect:** 0.00027
Matches : 16/100 fragment ions using 18 most intense peaks (help)

#	b	b^{++}	b*	b*$^{++}$	b^0	b^{0++}	Seq.	y	y^{++}	y*	y*$^{++}$	y^0	y^{0++}	#
1	114.0913	57.5493					L							12
2	171.1128	86.0600					G	***1306.7114***	653.8593	1289.6848	645.3461	1288.7008	644.8540	11
3	284.1969	142.6021					L	***1249.6899***	625.3486	1232.6634	616.8353	1231.6793	616.3433	10
4	399.2238	200.1155			381.2132	191.1103	D	***1136.6058***	568.8066	1119.5793	560.2933	1118.5953	559.8013	9
5	512.3079	256.6576			494.2973	247.6523	I	***1021.5789***	511.2931	1004.5524	502.7798	1003.5683	502.2878	8
6	641.3505	321.1789			623.3399	312.1736	E	***908.4948***	454.7511	891.4683	446.2378	890.4843	445.7458	7
7	754.4345	377.7209			736.4240	368.7156	I	***779.4522***	390.2298	762.4257	381.7165	761.4417	381.2245	6
8	825.4716	413.2395			807.4611	404.2342	A	666.3682	333.6877	649.3416	325.1745	648.3576	324.6824	5
9	926.5193	463.7633			908.5088	454.7580	T	595.3311	298.1692	578.3045	289.6559	577.3205	289.1639	4
10	1089.5827	545.2950			1071.5721	536.2897	Y	494.2834	247.6453	477.2568	239.1321			3
11	1245.6838	**623.3455**	1228.6572	614.8322	1227.6732	614.3402	R	331.2201	166.1137	314.1935	157.6004			2
12							R	***175.1190***	88.0631	158.0924	79.5498			1

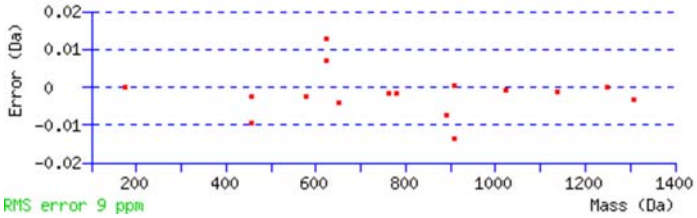

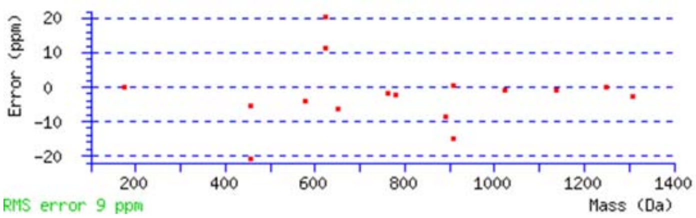

NCBI **BLAST** search of LGLDIEIATYRR
(Parameters: blastp, nr protein database, expect=20000, no filter, PAM30)
Other BLAST web gateways

All matches to this query

Score	Mr(calc)	Delta	Sequence
55.3	1418.7881	0.0004	LGLDIEIATYRR
23.0	1418.7881	0.0004	LGLLDRIVNYSR
16.5	1418.7769	0.0117	LLGNTFVALSDLR
7.7	1418.7881	0.0005	LQLRSLQYLER
5.7	1418.7769	0.0117	GLVLQLIQSYQR
3.9	1418.7769	0.0117	GLVLQLIQSYQR
1.9	1418.7994	-0.0108	LGPESVPPPKRSR
1.3	1418.7881	0.0005	LLQRRQIFSQK
1.1	1418.7881	0.0005	LQLRSLQYLER
0.9	1418.7769	0.0117	LYVALKELGEER

Mascot: http://www.matrixscience.com/

Mascot Search Results

Peptide View

MS/MS Fragmentation of **LGLDIEIATYRR**
Found in **ch12u_Q9NSB2|KRT84_HUMAN** in **uni_human**, Keratin, type II cuticular Hb4 OS=Homo sapiens GN=KRT84 PE=2 SV=2

Match to Query 15015: 1418.788662 from(473.936830,3+) intensity(2527766.2500) rtinseconds(2306) scans(12520) index(40409)
Title: 160219_Sunil_SDSI_A_Spectrum094994_scans__12520_RTINSECONDS=2306
Data file L:\\QE_2016\\160219_Sunil_KAP_LKC\\TMgf\\T\\T160219_Sunil_SDSI_A.mgf

Click mouse within plot area to zoom in by factor of two about that point
Or, Plot from 100 to 1500 Da Full range
Label all possible matches ○ Label matches used for scoring ◉
Show Y-axis ☐

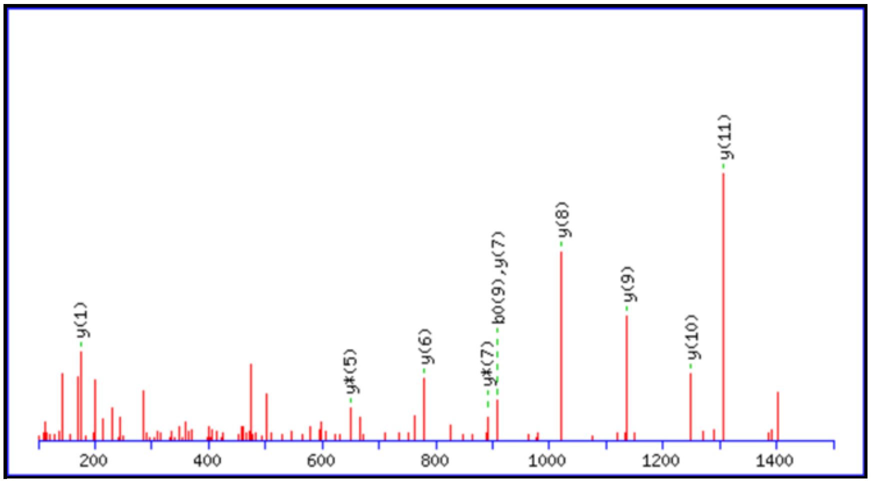

Monoisotopic mass of neutral peptide Mr(calc): 1418.7881
Fixed modifications: Carbamidomethyl (C) (apply to specified residues or termini only)
Ions Score: 52 **Expect:** 0.00058
Matches : 10/100 fragment ions using 14 most intense peaks (help)

#	b	b++	b*	b*++	b0	b0++	Seq.	y	y++	y*	y*++	y0	y0++	#
1	114.0913	57.5493					L							12
2	171.1128	86.0600					G	***1306.7114***	653.8593	1289.6848	645.3461	1288.7008	644.8540	11
3	284.1969	142.6021					L	***1249.6899***	625.3486	1232.6634	616.8353	1231.6793	616.3433	10
4	399.2238	200.1155			381.2132	191.1103	D	***1136.6058***	568.8066	1119.5793	560.2933	1118.5953	559.8013	9
5	512.3079	256.6576			494.2973	247.6523	I	***1021.5789***	511.2931	1004.5524	502.7798	1003.5683	502.2878	8
6	641.3505	321.1789			623.3399	312.1736	E	***908.4948***	454.7511	891.4683	446.2378	890.4843	445.7458	7
7	754.4345	377.7209			736.4240	368.7156	I	***779.4522***	390.2298	762.4257	381.7165	761.4417	381.2245	6
8	825.4716	413.2395			807.4611	404.2342	A	666.3682	333.6877	649.3416	325.1745	648.3576	324.6824	5
9	926.5193	463.7633			908.5088	454.7580	T	595.3311	298.1692	578.3045	289.6559	577.3205	289.1639	4
10	1089.5827	545.2950			1071.5721	536.2897	Y	494.2834	247.6453	477.2568	239.1321			3
11	1245.6838	623.3455	1228.6572	614.8322	1227.6732	614.3402	R	331.2201	166.1137	314.1935	157.6004			2
12							R	***175.1190***	88.0631	158.0924	79.5498			1

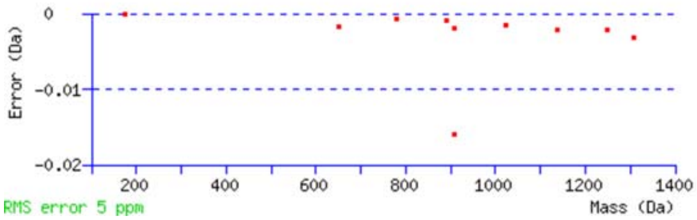

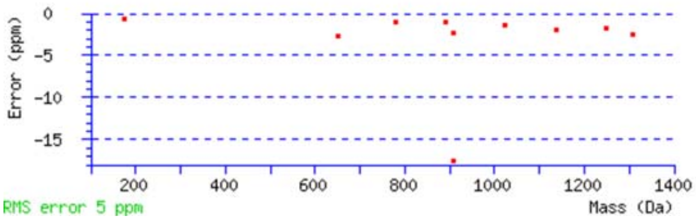

NCBI **BLAST** search of LGLDIEIATYRR
(Parameters: blastp, nr protein database, expect=20000, no filter, PAM30)
Other BLAST web gateways

All matches to this query

Score	Mr(calc)	Delta	Sequence
51.9	1418.7881	0.0005	LGLDIEIATYRR
22.8	1418.7881	0.0005	LGLLDRIVNYSR
12.1	1418.7769	0.0117	LLGNTFVALSDLR
3.2	1418.7769	0.0118	GLVLQLIQSYQR
2.3	1418.7994	-0.0107	LGPESVPPPKRSR
2.2	1418.7881	0.0005	LLQRRQIFSQK
2.0	1418.7769	0.0118	GLVLQLIQSYQR
1.7	1418.7881	0.0005	LQLRSLQYLER
1.1	1418.7882	0.0005	LVSSTLFGNTKPR

Mascot: http://www.matrixscience.com/

Mascot Search Results

Peptide View

MS/MS Fragmentation of **LGLDIEIATYRR**
Found in **ch12u_Q9NSB2|KRT84_HUMAN** in **uni_human**, Keratin, type II cuticular Hb4 OS=Homo sapiens GN=KRT84 PE=2 SV=2

Match to Query 15015: 1418.788662 from(473.936830,3+) intensity(2527766.2500) rtinseconds(2306) scans(12520) index(40409)
Title: 160219_Sunil_SDSI_A_Spectrum094994_scans__12520_RTINSECONDS=2306
Data file L:\\QE_2016\\160219_Sunil_KAP_LKC\\TMgf\\T\\T160219_Sunil_SDSI_A.mgf

Click mouse within plot area to zoom in by factor of two about that point
Or, Plot from 100 to 1500 Da Full range
Label all possible matches ○ Label matches used for scoring ◉
Show Y-axis ☐

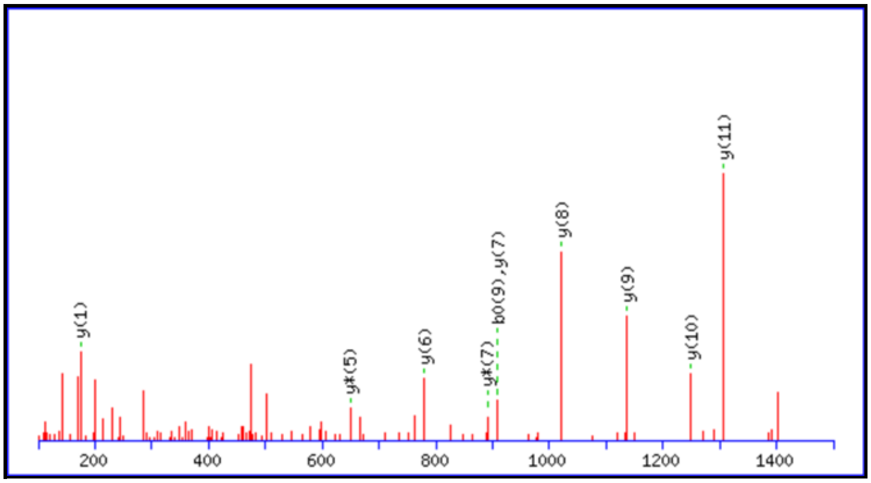

Monoisotopic mass of neutral peptide Mr(calc): 1418.7881
Fixed modifications: Carbamidomethyl (C) (apply to specified residues or termini only)
Ions Score: 52 **Expect:** 0.00058
Matches : 10/100 fragment ions using 14 most intense peaks (help)

#	b	b^{++}	b*	b^{*++}	b^0	b^{0++}	Seq.	y	y^{++}	y*	y^{*++}	y^0	y^{0++}	#
1	114.0913	57.5493					L							12
2	171.1128	86.0600					G	***1306.7114***	653.8593	1289.6848	645.3461	1288.7008	644.8540	11
3	284.1969	142.6021					L	***1249.6899***	625.3486	1232.6634	616.8353	1231.6793	616.3433	10
4	399.2238	200.1155			381.2132	191.1103	D	***1136.6058***	568.8066	1119.5793	560.2933	1118.5953	559.8013	9
5	512.3079	256.6576			494.2973	247.6523	I	***1021.5789***	511.2931	1004.5524	502.7798	1003.5683	502.2878	8
6	641.3505	321.1789			623.3399	312.1736	E	***908.4948***	454.7511	891.4683	446.2378	890.4843	445.7458	7
7	754.4345	377.7209			736.4240	368.7156	I	***779.4522***	390.2298	762.4257	381.7165	761.4417	381.2245	6
8	825.4716	413.2395			807.4611	404.2342	A	666.3682	333.6877	649.3416	325.1745	648.3576	324.6824	5
9	926.5193	463.7633			908.5088	454.7580	T	595.3311	298.1692	578.3045	289.6559	577.3205	289.1639	4
10	1089.5827	545.2950			1071.5721	536.2897	Y	494.2834	247.6453	477.2568	239.1321			3
11	1245.6838	623.3455	1228.6572	614.8322	1227.6732	614.3402	R	331.2201	166.1137	314.1935	157.6004			2
12							R	***175.1190***	88.0631	158.0924	79.5498			1

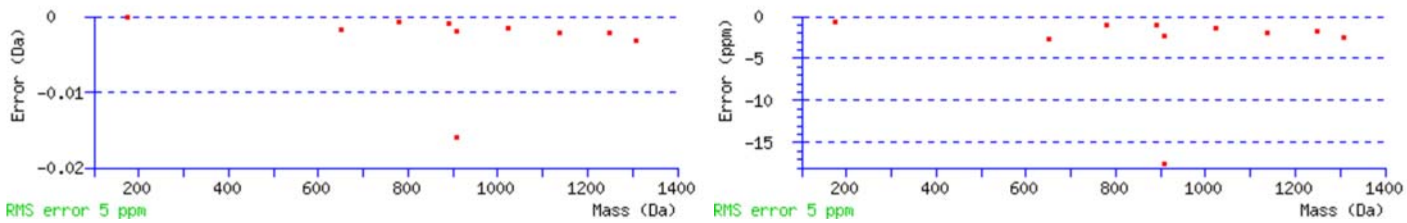

NCBI **BLAST** search of LGLDIEIATYRR
(Parameters: blastp, nr protein database, expect=20000, no filter, PAM30)
Other BLAST web gateways

All matches to this query

Score	Mr(calc)	Delta	Sequence
51.9	1418.7881	0.0005	LGLDIEIATYRR
22.8	1418.7881	0.0005	LGLLDRIVNYSR
12.1	1418.7769	0.0117	LLGNTFVALSDLR
3.2	1418.7769	0.0118	GLVLQLIQSYQR
2.3	1418.7994	-0.0107	LGPESVPPPKRSR
2.2	1418.7881	0.0005	LLQRRQIFSQK
2.0	1418.7769	0.0118	GLVLQLIQSYQR
1.7	1418.7881	0.0005	LQLRSLQYLER
1.1	1418.7882	0.0005	LVSSTLFGNTKPR

Mascot: http://www.matrixscience.com/

Mascot Search Results

Peptide View

MS/MS Fragmentation of **LGLDIEIATYRR**
Found in **ch12u_Q9NSB2|KRT84_HUMAN** in **uni_human**, Keratin, type II cuticular Hb4 OS=Homo sapiens GN=KRT84 PE=2 SV=2

Match to Query 15018: 1418.788752 from(473.936860,3+) intensity(15089622.0000) rtinseconds(2482) scans(13545) index(10621)
Title: 160219_Sunil_SDSI_A_Spectrum062744_scans__13545_RTINSECONDS=2482
Data file L:\\QE_2016\\160219_Sunil_KAP_LKC\\TMgf\\T\\T160219_Sunil_SDSI_A.mgf

Click mouse within plot area to zoom in by factor of two about that point
Or, Plot from 100 to 1400 Da Full range
Label all possible matches ○ Label matches used for scoring ◉
Show Y-axis ☐

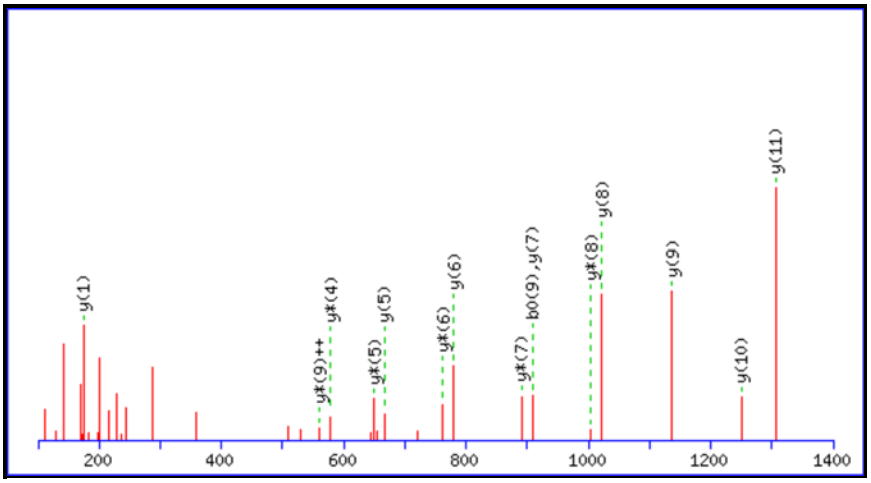

Monoisotopic mass of neutral peptide Mr(calc): 1418.7881
Fixed modifications: Carbamidomethyl (C) (apply to specified residues or termini only)
Ions Score: 67 **Expect:** 1.7e-005
Matches : 15/100 fragment ions using 19 most intense peaks (help)

#	b	b^{++}	b*	b^{*++}	b^0	b^{0++}	Seq.	y	y^{++}	y*	y^{*++}	y^0	y^{0++}	#
1	114.0913	57.5493					L							12
2	171.1128	86.0600					G	***1306.7114***	653.8593	1289.6848	645.3461	1288.7008	644.8540	11
3	284.1969	142.6021					L	***1249.6899***	625.3486	1232.6634	616.8353	1231.6793	616.3433	10
4	399.2238	200.1155			381.2132	191.1103	D	***1136.6058***	568.8066	1119.5793	560.2933	1118.5953	559.8013	9
5	512.3079	256.6576			494.2973	247.6523	I	***1021.5789***	511.2931	1004.5524	502.7798	1003.5683	502.2878	8
6	641.3505	321.1789			623.3399	312.1736	E	***908.4948***	454.7511	891.4683	446.2378	890.4843	445.7458	7
7	754.4345	377.7209			736.4240	368.7156	I	***779.4522***	390.2298	762.4257	381.7165	761.4417	381.2245	6
8	825.4716	413.2395			807.4611	404.2342	A	***666.3682***	333.6877	649.3416	325.1745	648.3576	324.6824	5
9	926.5193	463.7633			908.5088	454.7580	T	595.3311	298.1692	578.3045	289.6559	577.3205	289.1639	4
10	1089.5827	545.2950			1071.5721	536.2897	Y	494.2834	247.6453	477.2568	239.1321			3
11	1245.6838	623.3455	1228.6572	614.8322	1227.6732	614.3402	R	331.2201	166.1137	314.1935	157.6004			2
12							R	***175.1190***	88.0631	158.0924	79.5498			1

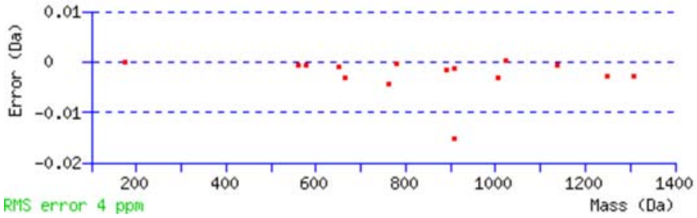

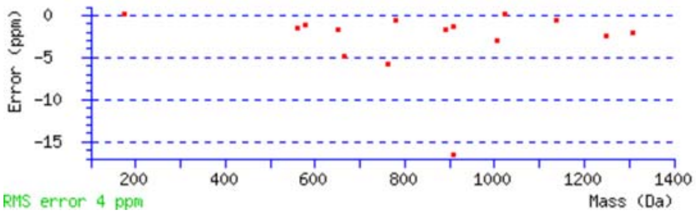

NCBI **BLAST** search of LGLDIEIATYRR
(Parameters: blastp, nr protein database, expect=20000, no filter, PAM30)
Other BLAST web gateways

All matches to this query

Score	Mr(calc)	Delta	Sequence
67.0	1418.7881	0.0006	LGLDIEIATYRR
19.8	1418.7881	0.0006	LGLLDRIVNYSR
17.1	1418.7769	0.0118	LLGNTFVALSDLR
7.8	1418.7881	0.0006	LQLRSLQYLER
1.7	1418.7881	0.0006	LLQRRQIFSQK
0.6	1418.7994	-0.0106	LGPESVPPPKRSR
0.4	1418.7769	0.0119	GLVLQLIQSYQR
0.3	1418.7769	0.0119	LYVALKELGEER
0.1	1418.7881	0.0006	LQYASLLSQLQR

Mascot: http://www.matrixscience.com/

Mascot Search Results

Peptide View

MS/MS Fragmentation of **LGLDIEIATYRR**
Found in **ch12u_Q9NSB2|KRT84_HUMAN** in **uni_human**, Keratin, type II cuticular Hb4 OS=Homo sapiens GN=KRT84 PE=2 SV=2

Match to Query 15020: 1418.788842 from(473.936890,3+) intensity(3289272.2500) rtinseconds(2612) scans(14301) index(11214)
Title: 160219_Sunil_SDSI_A_Spectrum063340_scans__14301_RTINSECONDS=2612
Data file L:\\QE_2016\\160219_Sunil_KAP_LKC\\TMgf\\T\\T160219_Sunil_SDSI_A.mgf

Click mouse within plot area to zoom in by factor of two about that point
Or, Plot from 100 to 1400 Da Full range
Label all possible matches ○ Label matches used for scoring ◉
Show Y-axis ☐

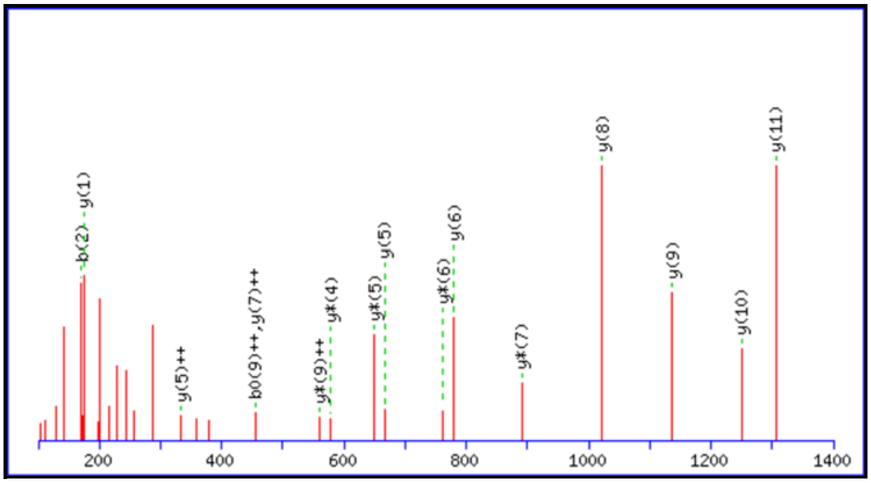

Monoisotopic mass of neutral peptide Mr(calc): 1418.7881
Fixed modifications: Carbamidomethyl (C) (apply to specified residues or termini only)
Ions Score: 61 **Expect:** 6.8e-005
Matches : 16/100 fragment ions using 18 most intense peaks (help)

#	b	b^{++}	b*	b^{*++}	b^0	b^{0++}	Seq.	y	y^{++}	y*	y^{*++}	y^0	y^{0++}	#
1	114.0913	57.5493					L							12
2	**171.1128**	86.0600					G	***1306.7114***	653.8593	1289.6848	645.3461	1288.7008	644.8540	11
3	284.1969	142.6021					L	***1249.6899***	625.3486	1232.6634	616.8353	1231.6793	616.3433	10
4	399.2238	200.1155			381.2132	191.1103	D	***1136.6058***	568.8066	1119.5793	560.2933	1118.5953	559.8013	9
5	512.3079	256.6576			494.2973	247.6523	I	***1021.5789***	511.2931	1004.5524	502.7798	1003.5683	502.2878	8
6	641.3505	321.1789			623.3399	312.1736	E	908.4948	454.7511	891.4683	446.2378	890.4843	445.7458	7
7	754.4345	377.7209			736.4240	368.7156	I	***779.4522***	390.2298	762.4257	381.7165	761.4417	381.2245	6
8	825.4716	413.2395			807.4611	404.2342	A	***666.3682***	333.6877	649.3416	325.1745	648.3576	324.6824	5
9	926.5193	463.7633			908.5088	454.7580	T	595.3311	298.1692	578.3045	289.6559	577.3205	289.1639	4
10	1089.5827	545.2950			1071.5721	536.2897	Y	494.2834	247.6453	477.2568	239.1321			3
11	1245.6838	623.3455	1228.6572	614.8322	1227.6732	614.3402	R	331.2201	166.1137	314.1935	157.6004			2
12							R	***175.1190***	88.0631	158.0924	79.5498			1

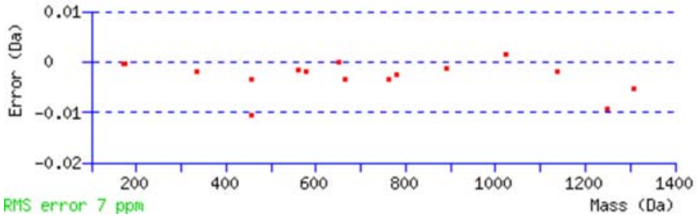

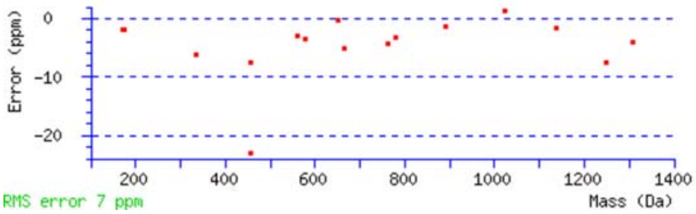

NCBI **BLAST** search of LGLDIEIATYRR
(Parameters: blastp, nr protein database, expect=20000, no filter, PAM30)
Other BLAST web gateways

All matches to this query

Score	Mr(calc)	Delta	Sequence
61.2	1418.7881	0.0007	LGLDIEIATYRR
20.2	1418.7881	0.0007	LGLLDRIVNYSR
16.7	1418.7769	0.0119	LLGNTFVALSDLR
0.4	1418.7881	0.0007	LQLRSLQYLER

Mascot: http://www.matrixscience.com/

Mascot Search Results

Peptide View

MS/MS Fragmentation of **FLEQQNKLLETK**
Found in **ch12u_Q9NSB2|KRT84_HUMAN** in **uni_human**, Keratin, type II cuticular Hb4 OS=Homo sapiens GN=KRT84 PE=2 SV=2

Match to Query 17367: 1489.812848 from(745.913700,2+) intensity(9408938.0000) rtinseconds(1649) scans(8687) index(6515)
Title: 160219_Sunil_SDSI_A_Spectrum058623_scans__8687_RTINSECONDS=1649
Data file L:\\QE_2016\\160219_Sunil_KAP_LKC\\TMgf\\T\\T160219_Sunil_SDSI_A.mgf

Click mouse within plot area to zoom in by factor of two about that point
Or, Plot from 100 to 1500 Da Full range
Label all possible matches ○ Label matches used for scoring ◉
Show Y-axis ☐

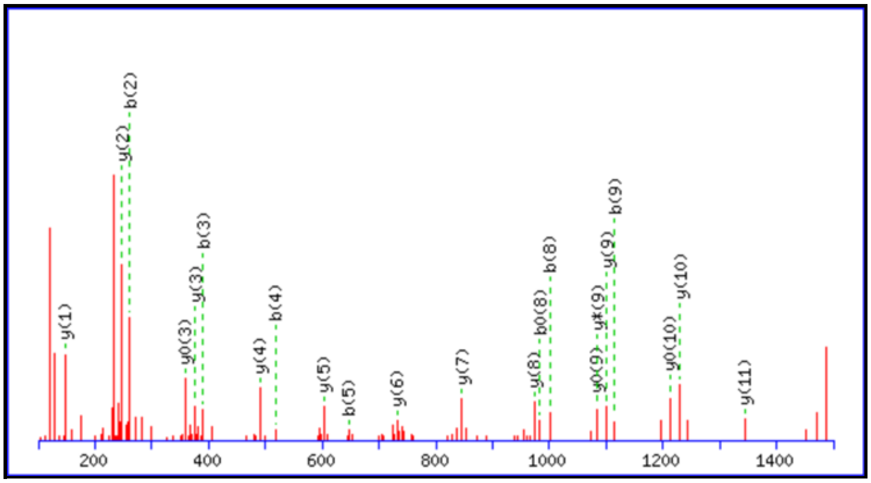

Monoisotopic mass of neutral peptide Mr(calc): 1489.8140
Fixed modifications: Carbamidomethyl (C) (apply to specified residues or termini only)
Ions Score: 79 **Expect:** 1.4e-006
Matches : 22/120 fragment ions using 40 most intense peaks (help)

#	b	b++	b*	b*++	b0	b0++	Seq.	y	y++	y*	y*++	y0	y0++	#
1	148.0757	74.5415					F							12
2	**261.1598**	131.0835					L	***1343.7529***	672.3801	1326.7264	663.8668	1325.7423	663.3748	11
3	**390.2023**	195.6048			372.1918	186.5995	E	***1230.6688***	615.8381	1213.6423	607.3248	1212.6583	606.8328	10
4	**518.2609**	259.6341	501.2344	251.1208	500.2504	250.6288	Q	***1101.6263***	551.3168	1084.5997	542.8035	1083.6157	542.3115	9
5	**646.3195**	323.6634	629.2930	315.1501	628.3089	314.6581	Q	***973.5677***	487.2875	956.5411	478.7742	955.5571	478.2822	8
6	760.3624	380.6849	743.3359	372.1716	742.3519	371.6796	N	***845.5091***	423.2582	828.4825	414.7449	827.4985	414.2529	7
7	888.4574	444.7323	871.4308	436.2191	870.4468	435.7271	K	***731.4662***	366.2367	714.4396	357.7234	713.4556	357.2314	6
8	**1001.5415**	501.2744	984.5149	492.7611	983.5309	492.2691	L	***603.3712***	302.1892	586.3447	293.6760	585.3606	293.1840	5
9	**1114.6255**	557.8164	1097.5990	549.3031	1096.6150	548.8111	L	***490.2871***	245.6472	473.2606	237.1339	472.2766	236.6419	4
10	1243.6681	622.3377	1226.6416	613.8244	1225.6575	613.3324	E	***377.2031***	189.1052	360.1765	180.5919	359.1925	180.0999	3
11	1344.7158	672.8615	1327.6892	664.3483	1326.7052	663.8563	T	***248.1605***	124.5839	231.1339	116.0706	230.1499	115.5786	2
12							K	***147.1128***	74.0600	130.0863	65.5468			1

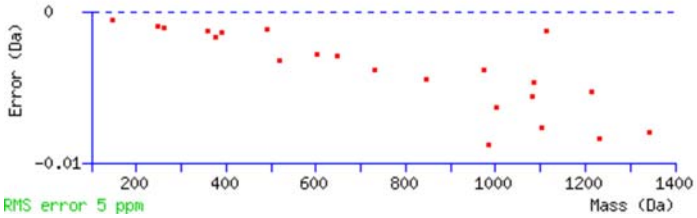

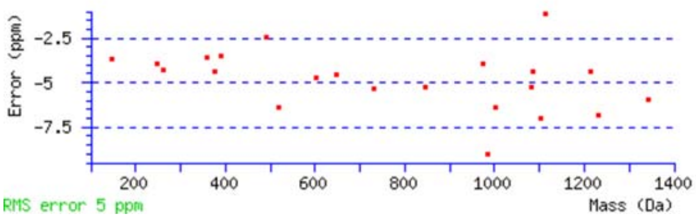

NCBI **BLAST** search of FLEQQNKLLETK
(Parameters: blastp, nr protein database, expect=20000, no filter, PAM30)
Other BLAST web gateways

All matches to this query

Score	Mr(calc)	Delta	Sequence
79.3	1489.8140	-0.0012	FLEQQNKLLETK

Mascot: http://www.matrixscience.com/

Mascot Search Results

Peptide View

MS/MS Fragmentation of **FLEQQNKLLETK**
Found in **ch12u_Q9NSB2|KRT84_HUMAN** in **uni_human**, Keratin, type II cuticular Hb4 OS=Homo sapiens GN=KRT84 PE=2 SV=2

Match to Query 17397: 1489.816182 from(497.612670,3+) intensity(41201200.0000) rtinseconds(1619) scans(8513) index(6367)
Title: 160219_Sunil_SDSI_A_Spectrum058475_scans__8513_RTINSECONDS=1619
Data file L:\\QE_2016\\160219_Sunil_KAP_LKC\\TMgf\\T\\T160219_Sunil_SDSI_A.mgf

Click mouse within plot area to zoom in by factor of two about that point
Or, Plot from 100 to 1400 Da Full range
Label all possible matches ○ Label matches used for scoring ◉
Show Y-axis ☐

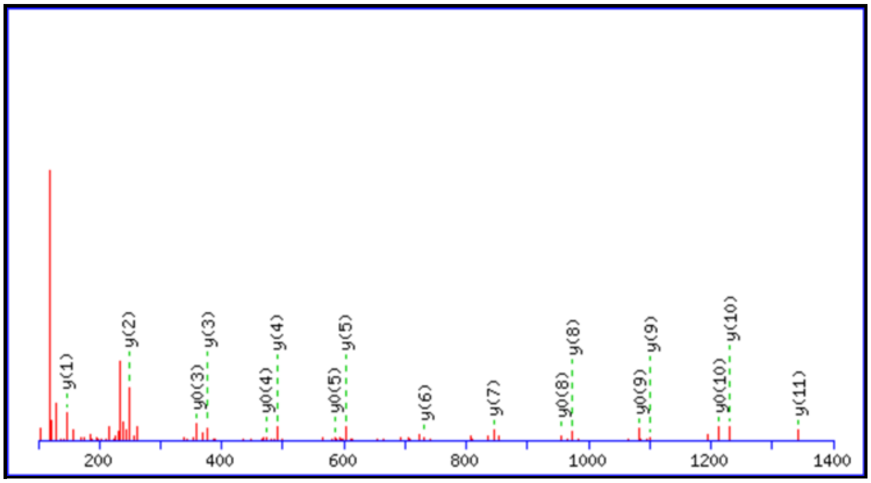

Monoisotopic mass of neutral peptide Mr(calc): 1489.8140
Fixed modifications: Carbamidomethyl (C) (apply to specified residues or termini only)
Ions Score: 76 **Expect:** 2.5e-006
Matches : 17/120 fragment ions using 34 most intense peaks (help)

#	b	b^{++}	b*	b^{*++}	b^0	b^{0++}	Seq.	y	y^{++}	y*	y^{*++}	y^0	y^{0++}	#
1	148.0757	74.5415					F							12
2	261.1598	131.0835					L	**1343.7529**	672.3801	1326.7264	663.8668	1325.7423	663.3748	11
3	390.2023	195.6048			372.1918	186.5995	E	**1230.6688**	615.8381	1213.6423	607.3248	1212.6583	606.8328	10
4	518.2609	259.6341	501.2344	251.1208	500.2504	250.6288	Q	**1101.6263**	551.3168	1084.5997	542.8035	1083.6157	542.3115	9
5	646.3195	323.6634	629.2930	315.1501	628.3089	314.6581	Q	**973.5677**	487.2875	956.5411	478.7742	955.5571	478.2822	8
6	760.3624	380.6849	743.3359	372.1716	742.3519	371.6796	N	**845.5091**	423.2582	828.4825	414.7449	827.4985	414.2529	7
7	888.4574	444.7323	871.4308	436.2191	870.4468	435.7271	K	**731.4662**	366.2367	714.4396	357.7234	713.4556	357.2314	6
8	1001.5415	501.2744	984.5149	492.7611	983.5309	492.2691	L	**603.3712**	302.1892	586.3447	293.6760	585.3606	293.1840	5
9	1114.6255	557.8164	1097.5990	549.3031	1096.6150	548.8111	L	**490.2871**	245.6472	473.2606	237.1339	472.2766	236.6419	4
10	1243.6681	622.3377	1226.6416	613.8244	1225.6575	613.3324	E	**377.2031**	189.1052	360.1765	180.5919	359.1925	180.0999	3
11	1344.7158	672.8615	1327.6892	664.3483	1326.7052	663.8563	T	**248.1605**	124.5839	231.1339	116.0706	230.1499	115.5786	2
12							K	**147.1128**	74.0600	130.0863	65.5468			1

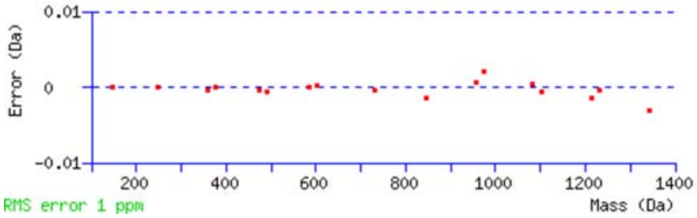

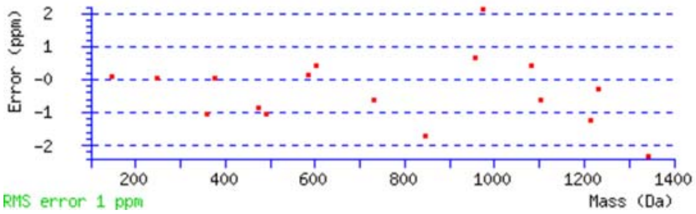

NCBI **BLAST** search of FLEQQNKLLETK
(Parameters: blastp, nr protein database, expect=20000, no filter, PAM30)
Other BLAST web gateways

All matches to this query

Score	Mr(calc)	Delta	Sequence
76.1	1489.8140	0.0022	FLEQQNKLLETK
1.0	1487.8069	2.0093	NVHRPPRQRDIT

Mascot: http://www.matrixscience.com/

Mascot Search Results

Peptide View

MS/MS Fragmentation of **FLEQQNKLLETK**
Found in **ch12u_Q9NSB2|KRT84_HUMAN** in **uni_human**, Keratin, type II cuticular Hb4 OS=Homo sapiens GN=KRT84 PE=2 SV=2

Match to Query 17370: 1489.813332 from(497.611720,3+) intensity(2131135.0000) rtinseconds(1745) scans(9254) index(7011)
Title: 160219_Sunil_SDSI_A_Spectrum059119_scans__9254_RTINSECONDS=1745
Data file L:\\QE_2016\\160219_Sunil_KAP_LKC\\TMgf\\T\\T160219_Sunil_SDSI_A.mgf

Click mouse within plot area to zoom in by factor of two about that point
Or, Plot from 100 to 1500 Da Full range
Label all possible matches ○ Label matches used for scoring ◉
Show Y-axis ☐

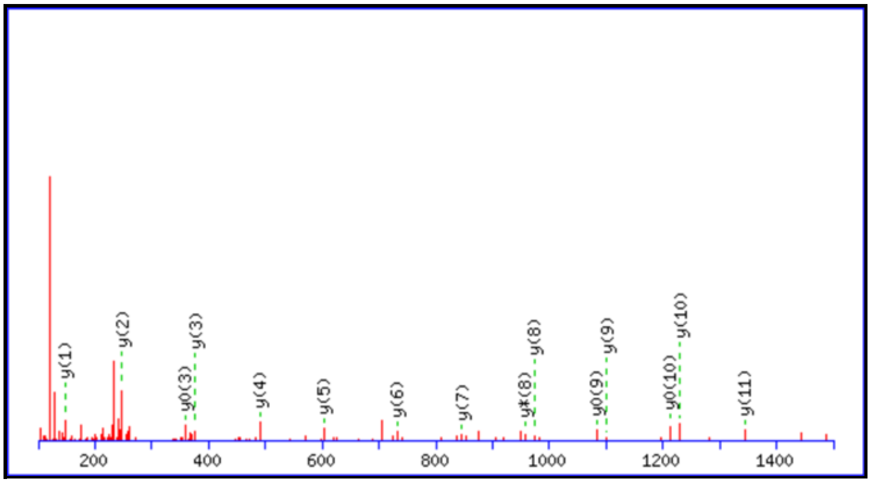

Monoisotopic mass of neutral peptide Mr(calc): 1489.8140
Fixed modifications: Carbamidomethyl (C) (apply to specified residues or termini only)
Ions Score: 64 **Expect:** 4.5e-005
Matches : 15/120 fragment ions using 37 most intense peaks (help)

#	b	b^{++}	b*	b*$^{++}$	b^{0}	b^{0++}	Seq.	y	y^{++}	y*	y*$^{++}$	y^{0}	y^{0++}	#
1	148.0757	74.5415					F							12
2	261.1598	131.0835					L	***1343.7529***	672.3801	1326.7264	663.8668	1325.7423	663.3748	11
3	390.2023	195.6048			372.1918	186.5995	E	***1230.6688***	615.8381	1213.6423	607.3248	1212.6583	606.8328	10
4	518.2609	259.6341	501.2344	251.1208	500.2504	250.6288	Q	***1101.6263***	551.3168	1084.5997	542.8035	1083.6157	542.3115	9
5	646.3195	323.6634	629.2930	315.1501	628.3089	314.6581	Q	***973.5677***	487.2875	956.5411	478.7742	955.5571	478.2822	8
6	760.3624	380.6849	743.3359	372.1716	742.3519	371.6796	N	***845.5091***	423.2582	828.4825	414.7449	827.4985	414.2529	7
7	888.4574	444.7323	871.4308	436.2191	870.4468	435.7271	K	***731.4662***	366.2367	714.4396	357.7234	713.4556	357.2314	6
8	1001.5415	501.2744	984.5149	492.7611	983.5309	492.2691	L	***603.3712***	302.1892	586.3447	293.6760	585.3606	293.1840	5
9	1114.6255	557.8164	1097.5990	549.3031	1096.6150	548.8111	L	***490.2871***	245.6472	473.2606	237.1339	472.2766	236.6419	4
10	1243.6681	622.3377	1226.6416	613.8244	1225.6575	613.3324	E	***377.2031***	189.1052	360.1765	180.5919	359.1925	180.0999	3
11	1344.7158	672.8615	1327.6892	664.3483	1326.7052	663.8563	T	***248.1605***	124.5839	231.1339	116.0706	230.1499	115.5786	2
12							K	***147.1128***	74.0600	130.0863	65.5468			1

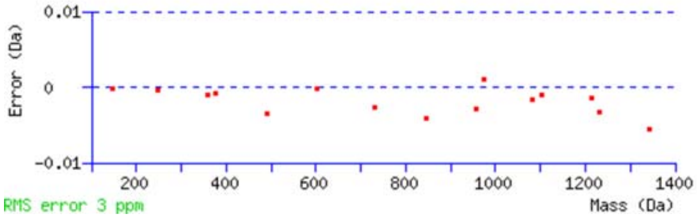

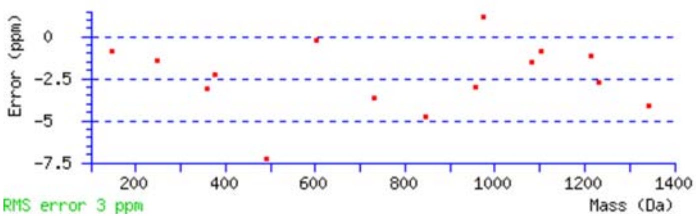

NCBI **BLAST** search of FLEQQNKLLETK
(Parameters: blastp, nr protein database, expect=20000, no filter, PAM30)
Other BLAST web gateways

All matches to this query

Score	Mr(calc)	Delta	Sequence
64.0	1489.8140	-0.0007	FLEQQNKLLETK
0.4	1487.8069	2.0064	NVHRPPRQRDIT

Mascot: http://www.matrixscience.com/

Mascot Search Results

Peptide View

MS/MS Fragmentation of **FLEQQNKLLETK**
Found in **ch12u_Q9NSB2|KRT84_HUMAN** in **uni_human**, Keratin, type II cuticular Hb4 OS=Homo sapiens GN=KRT84 PE=2 SV=2

Match to Query 17372: 1489.813542 from(497.611790,3+) intensity(4951518.0000) rtinseconds(1547) scans(8099) index(6001)
Title: 160219_Sunil_SDSI_A_Spectrum058109_scans__8099_RTINSECONDS=1547
Data file L:\\QE_2016\\160219_Sunil_KAP_LKC\\TMgf\\T\\T160219_Sunil_SDSI_A.mgf

Click mouse within plot area to zoom in by factor of two about that point
Or, Plot from 100 to 1500 Da Full range
Label all possible matches ○ Label matches used for scoring ◉
Show Y-axis ☐

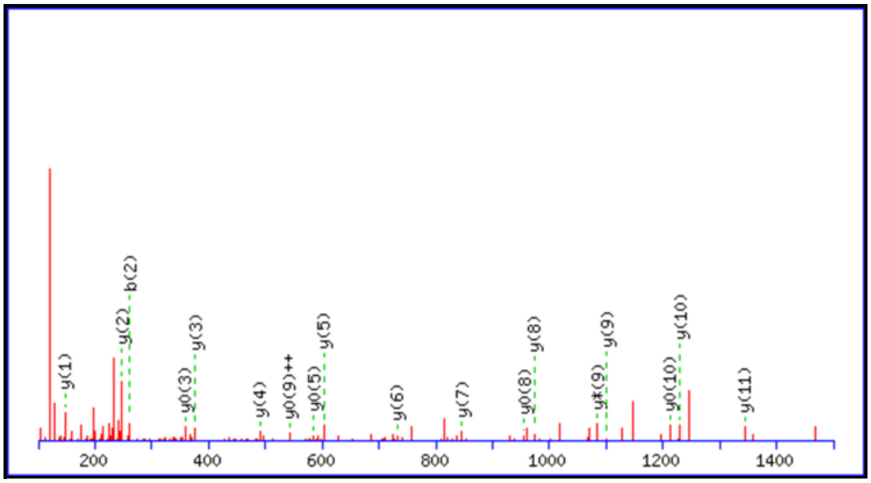

Monoisotopic mass of neutral peptide Mr(calc): 1489.8140
Fixed modifications: Carbamidomethyl (C) (apply to specified residues or termini only)
Ions Score: 49 **Expect:** 0.0015
Matches : 18/120 fragment ions using 59 most intense peaks (help)

#	b	b^{++}	b^{*}	b^{*++}	b^{0}	b^{0++}	Seq.	y	y^{++}	y^{*}	y^{*++}	y^{0}	y^{0++}	#
1	148.0757	74.5415					F							12
2	**261.1598**	131.0835					L	***1343.7529***	672.3801	1326.7264	663.8668	1325.7423	663.3748	11
3	390.2023	195.6048			372.1918	186.5995	E	***1230.6688***	615.8381	1213.6423	607.3248	1212.6583	606.8328	10
4	518.2609	259.6341	501.2344	251.1208	500.2504	250.6288	Q	***1101.6263***	551.3168	1084.5997	542.8035	1083.6157	542.3115	9
5	646.3195	323.6634	629.2930	315.1501	628.3089	314.6581	Q	***973.5677***	487.2875	956.5411	478.7742	955.5571	478.2822	8
6	760.3624	380.6849	743.3359	372.1716	742.3519	371.6796	N	***845.5091***	423.2582	828.4825	414.7449	827.4985	414.2529	7
7	888.4574	444.7323	871.4308	436.2191	870.4468	435.7271	K	***731.4662***	366.2367	714.4396	357.7234	713.4556	357.2314	6
8	1001.5415	501.2744	984.5149	492.7611	983.5309	492.2691	L	***603.3712***	302.1892	586.3447	293.6760	585.3606	293.1840	5
9	1114.6255	557.8164	1097.5990	549.3031	1096.6150	548.8111	L	***490.2871***	245.6472	473.2606	237.1339	472.2766	236.6419	4
10	1243.6681	622.3377	1226.6416	613.8244	1225.6575	613.3324	E	***377.2031***	189.1052	360.1765	180.5919	359.1925	180.0999	3
11	1344.7158	672.8615	1327.6892	664.3483	1326.7052	663.8563	T	***248.1605***	124.5839	231.1339	116.0706	230.1499	115.5786	2
12							K	***147.1128***	74.0600	130.0863	65.5468			1

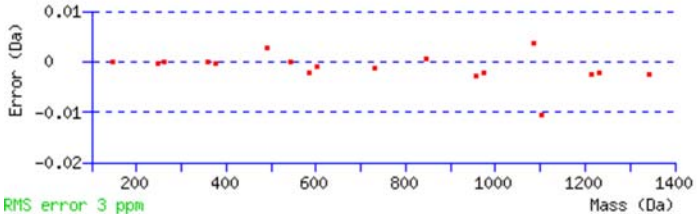

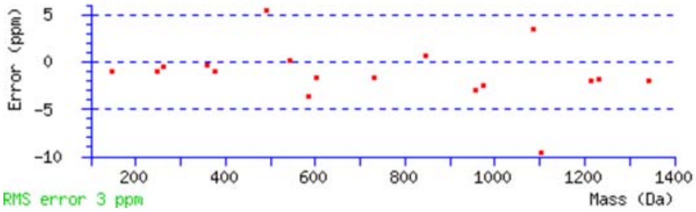

NCBI **BLAST** search of FLEQQNKLLETK
(Parameters: blastp, nr protein database, expect=20000, no filter, PAM30)
Other BLAST web gateways

All matches to this query

Score	Mr(calc)	Delta	Sequence
48.8	1489.8140	-0.0005	FLEQQNKLLETK
3.0	1489.8041	0.0094	FRLWNVSNEVVK

Mascot: http://www.matrixscience.com/

Mascot Search Results

Peptide View

MS/MS Fragmentation of **FLEQQNKLLETK**
Found in **ch12u_Q9NSB2|KRT84_HUMAN** in **uni_human**, Keratin, type II cuticular Hb4 OS=Homo sapiens GN=KRT84 PE=2 SV=2

Match to Query 17376: 1489.814082 from(497.611970,3+) intensity(1265225.2500) rtinseconds(1471) scans(7681) index(36255)
Title: 160219_Sunil_SDSI_A_Spectrum090840_scans__7681_RTINSECONDS=1471
Data file L:\\QE_2016\\160219_Sunil_KAP_LKC\\TMgf\\T\\T160219_Sunil_SDSI_A.mgf

Click mouse within plot area to zoom in by factor of two about that point
Or, Plot from 100 to 1500 Da Full range
Label all possible matches ○ Label matches used for scoring ◉
Show Y-axis ☐

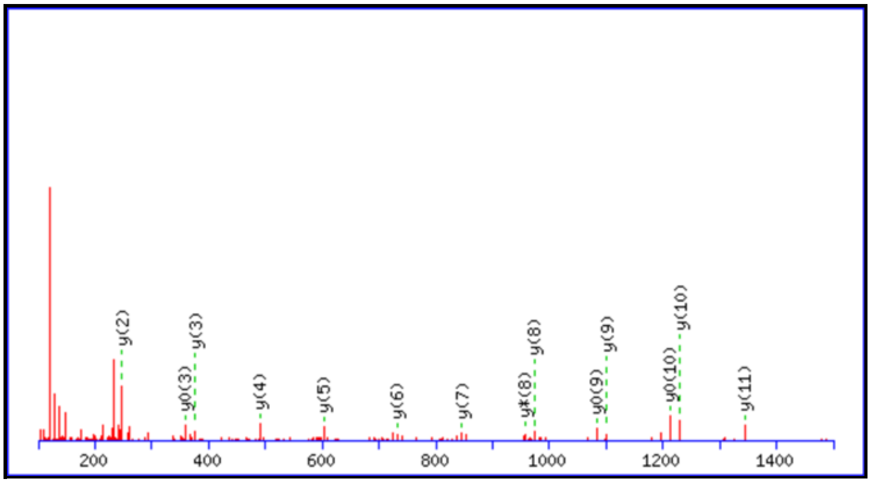

Monoisotopic mass of neutral peptide Mr(calc): 1489.8140
Fixed modifications: Carbamidomethyl (C) (apply to specified residues or termini only)
Ions Score: 64 **Expect:** 4e-005
Matches : 14/120 fragment ions using 28 most intense peaks (help)

#	b	b^{++}	b*	b^{*++}	b^0	b^{0++}	Seq.	y	y^{++}	y*	y^{*++}	y^0	y^{0++}	#
1	148.0757	74.5415					F							12
2	261.1598	131.0835					L	1343.7529	672.3801	1326.7264	663.8668	1325.7423	663.3748	11
3	390.2023	195.6048			372.1918	186.5995	E	1230.6688	615.8381	1213.6423	607.3248	1212.6583	606.8328	10
4	518.2609	259.6341	501.2344	251.1208	500.2504	250.6288	Q	1101.6263	551.3168	1084.5997	542.8035	1083.6157	542.3115	9
5	646.3195	323.6634	629.2930	315.1501	628.3089	314.6581	Q	973.5677	487.2875	956.5411	478.7742	955.5571	478.2822	8
6	760.3624	380.6849	743.3359	372.1716	742.3519	371.6796	N	845.5091	423.2582	828.4825	414.7449	827.4985	414.2529	7
7	888.4574	444.7323	871.4308	436.2191	870.4468	435.7271	K	731.4662	366.2367	714.4396	357.7234	713.4556	357.2314	6
8	1001.5415	501.2744	984.5149	492.7611	983.5309	492.2691	L	603.3712	302.1892	586.3447	293.6760	585.3606	293.1840	5
9	1114.6255	557.8164	1097.5990	549.3031	1096.6150	548.8111	L	490.2871	245.6472	473.2606	237.1339	472.2766	236.6419	4
10	1243.6681	622.3377	1226.6416	613.8244	1225.6575	613.3324	E	377.2031	189.1052	360.1765	180.5919	359.1925	180.0999	3
11	1344.7158	672.8615	1327.6892	664.3483	1326.7052	663.8563	T	248.1605	124.5839	231.1339	116.0706	230.1499	115.5786	2
12							K	147.1128	74.0600	130.0863	65.5468			1

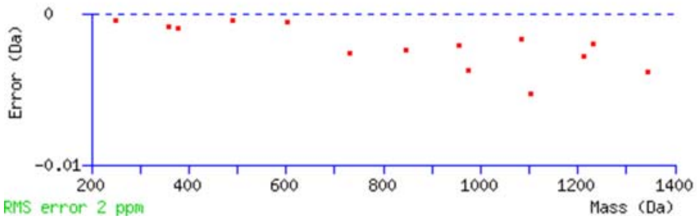

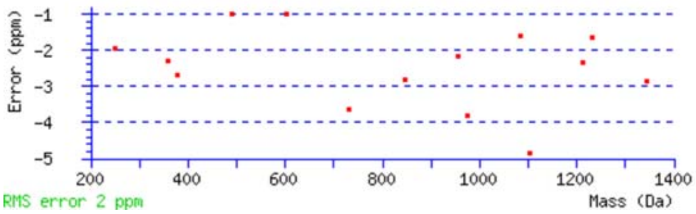

NCBI **BLAST** search of FLEQQNKLLETK
(Parameters: blastp, nr protein database, expect=20000, no filter, PAM30)
Other BLAST web gateways

All matches to this query

Score	Mr(calc)	Delta	Sequence
64.5	1489.8140	0.0001	FLEQQNKLLETK
1.3	1487.8130	2.0011	VLEERGREILMK

Mascot: http://www.matrixscience.com/

Mascot Search Results

Peptide View

MS/MS Fragmentation of **FLEQQNKLLETK**
Found in **ch12u_Q9NSB2|KRT84_HUMAN** in **uni_human**, Keratin, type II cuticular Hb4 OS=Homo sapiens GN=KRT84 PE=2 SV=2

Match to Query 17386: 1489.814532 from(497.612120,3+) intensity(1645731.6250) rtinseconds(2043) scans(10992) index(39103)
Title: 160219_Sunil_SDSI_A_Spectrum093688_scans__10992_RTINSECONDS=2043
Data file L:\\QE_2016\\160219_Sunil_KAP_LKC\\TMgf\\T\\T160219_Sunil_SDSI_A.mgf

Click mouse within plot area to zoom in by factor of two about that point
Or, Plot from 100 to 1500 Da Full range
Label all possible matches ○ Label matches used for scoring ◉
Show Y-axis ☐

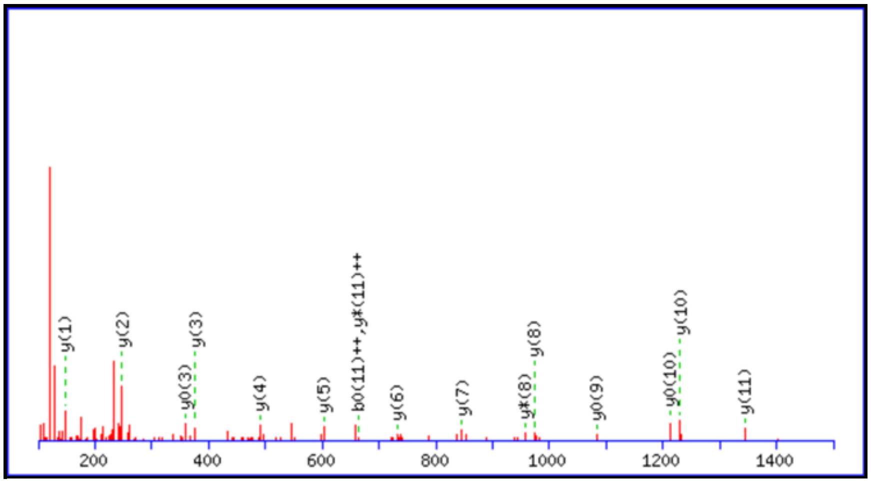

Monoisotopic mass of neutral peptide Mr(calc): 1489.8140
Fixed modifications: Carbamidomethyl (C) (apply to specified residues or termini only)
Ions Score: 61 **Expect:** 8.7e-005
Matches : 16/120 fragment ions using 33 most intense peaks (help)

#	b	b++	b*	b*++	b0	b0++	Seq.	y	y++	y*	y*++	y0	y0++	#
1	148.0757	74.5415					F							12
2	261.1598	131.0835					L	***1343.7529***	672.3801	1326.7264	663.8668	1325.7423	663.3748	11
3	390.2023	195.6048			372.1918	186.5995	E	***1230.6688***	615.8381	1213.6423	607.3248	1212.6583	606.8328	10
4	518.2609	259.6341	501.2344	251.1208	500.2504	250.6288	Q	1101.6263	551.3168	1084.5997	542.8035	1083.6157	542.3115	9
5	646.3195	323.6634	629.2930	315.1501	628.3089	314.6581	Q	***973.5677***	487.2875	956.5411	478.7742	955.5571	478.2822	8
6	760.3624	380.6849	743.3359	372.1716	742.3519	371.6796	N	***845.5091***	423.2582	828.4825	414.7449	827.4985	414.2529	7
7	888.4574	444.7323	871.4308	436.2191	870.4468	435.7271	K	***731.4662***	366.2367	714.4396	357.7234	713.4556	357.2314	6
8	1001.5415	501.2744	984.5149	492.7611	983.5309	492.2691	L	***603.3712***	302.1892	586.3447	293.6760	585.3606	293.1840	5
9	1114.6255	557.8164	1097.5990	549.3031	1096.6150	548.8111	L	***490.2871***	245.6472	473.2606	237.1339	472.2766	236.6419	4
10	1243.6681	622.3377	1226.6416	613.8244	1225.6575	613.3324	E	***377.2031***	189.1052	360.1765	180.5919	359.1925	180.0999	3
11	1344.7158	672.8615	1327.6892	664.3483	1326.7052	663.8563	T	***248.1605***	124.5839	231.1339	116.0706	230.1499	115.5786	2
12							K	***147.1128***	74.0600	130.0863	65.5468			1

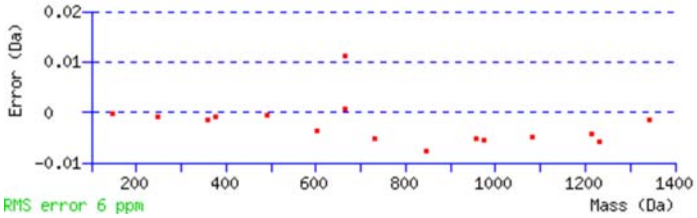

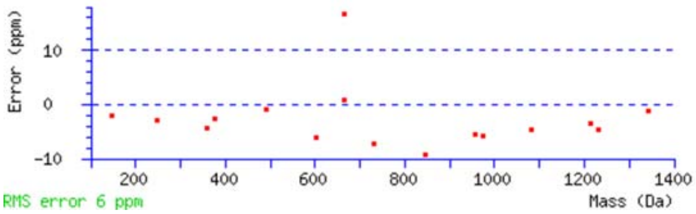

NCBI **BLAST** search of FLEQQNKLLETK
(Parameters: blastp, nr protein database, expect=20000, no filter, PAM30)
Other BLAST web gateways

All matches to this query

Score	Mr(calc)	Delta	Sequence
61.2	1489.8140	0.0005	FLEQQNKLLETK
0.2	1487.8069	2.0076	NVHRPPRQRDIT

Mascot: http://www.matrixscience.com/

Mascot Search Results

Peptide View

MS/MS Fragmentation of **FLEQQNKLLETK**
Found in **ch12u_Q9NSB2|KRT84_HUMAN** in **uni_human**, Keratin, type II cuticular Hb4 OS=Homo sapiens GN=KRT84 PE=2 SV=2

Match to Query 17392: 1489.815372 from(497.612400,3+) intensity(2807921.7500) rtinseconds(2180) scans(11790) index(9170)
Title: 160219_Sunil_SDSI_A_Spectrum061284_scans__11790_RTINSECONDS=2180
Data file L:\\QE_2016\\160219_Sunil_KAP_LKC\\TMgf\\T\\T160219_Sunil_SDSI_A.mgf

Click mouse within plot area to zoom in by factor of two about that point
Or, Plot from 100 to 1300 Da Full range
Label all possible matches ○ Label matches used for scoring ◉
Show Y-axis □

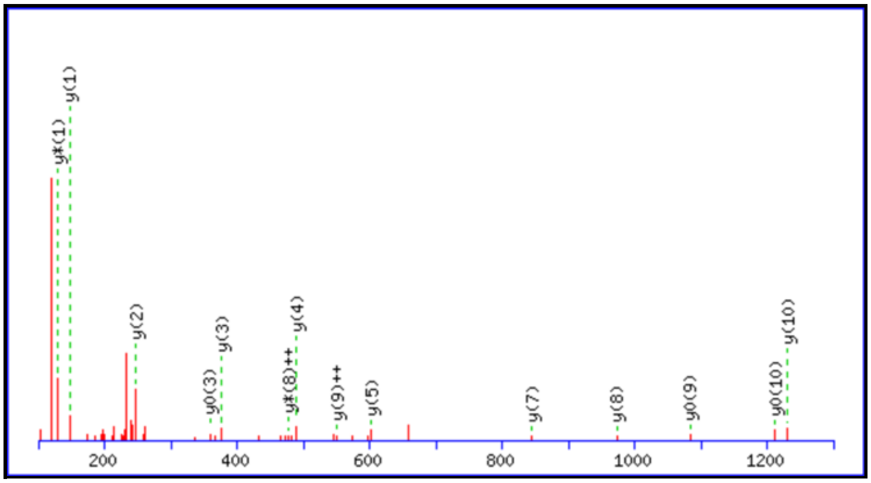

Monoisotopic mass of neutral peptide Mr(calc): 1489.8140
Fixed modifications: Carbamidomethyl (C) (apply to specified residues or termini only)
Ions Score: 45 **Expect:** 0.0032
Matches : 14/120 fragment ions using 27 most intense peaks (help)

#	b	b^{++}	b*	b^{*++}	b^0	b^{0++}	Seq.	y	y^{++}	y*	y^{*++}	y^0	y^{0++}	#
1	148.0757	74.5415					F							12
2	261.1598	131.0835					L	1343.7529	672.3801	1326.7264	663.8668	1325.7423	663.3748	11
3	390.2023	195.6048			372.1918	186.5995	E	***1230.6688***	615.8381	1213.6423	607.3248	1212.6583	606.8328	10
4	518.2609	259.6341	501.2344	251.1208	500.2504	250.6288	Q	1101.6263	551.3168	1084.5997	542.8035	1083.6157	542.3115	9
5	646.3195	323.6634	629.2930	315.1501	628.3089	314.6581	Q	***973.5677***	487.2875	956.5411	478.7742	955.5571	478.2822	8
6	760.3624	380.6849	743.3359	372.1716	742.3519	371.6796	N	***845.5091***	423.2582	828.4825	414.7449	827.4985	414.2529	7
7	888.4574	444.7323	871.4308	436.2191	870.4468	435.7271	K	731.4662	366.2367	714.4396	357.7234	713.4556	357.2314	6
8	1001.5415	501.2744	984.5149	492.7611	983.5309	492.2691	L	***603.3712***	302.1892	586.3447	293.6760	585.3606	293.1840	5
9	1114.6255	557.8164	1097.5990	549.3031	1096.6150	548.8111	L	***490.2871***	245.6472	473.2606	237.1339	472.2766	236.6419	4
10	1243.6681	622.3377	1226.6416	613.8244	1225.6575	613.3324	E	***377.2031***	189.1052	360.1765	180.5919	359.1925	180.0999	3
11	1344.7158	672.8615	1327.6892	664.3483	1326.7052	663.8563	T	***248.1605***	124.5839	231.1339	116.0706	230.1499	115.5786	2
12							K	***147.1128***	74.0600	130.0863	65.5468			1

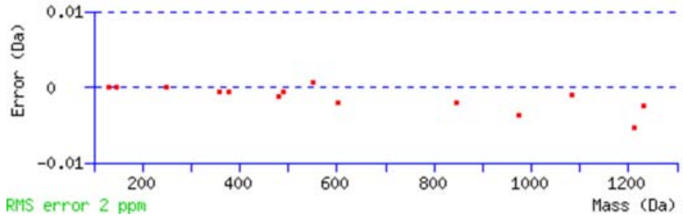

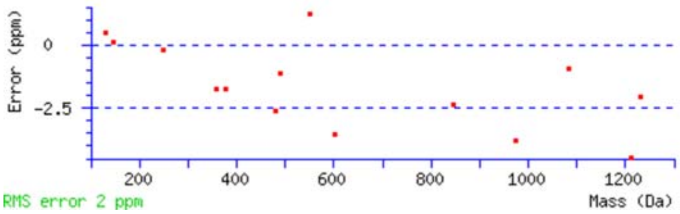

NCBI **BLAST** search of FLEQQNKLLETK
(Parameters: blastp, nr protein database, expect=20000, no filter, PAM30)
Other BLAST web gateways

All matches to this query

Score	Mr(calc)	Delta	Sequence
45.2	1489.8140	0.0014	FLEQQNKLLETK
2.1	1487.8069	2.0084	NVHRPPRQRDIT

Mascot: http://www.matrixscience.com/

Mascot Search Results

Peptide View

MS/MS Fragmentation of **FLEQQNKLLETK**
Found in **ch12u_Q9NSB2|KRT84_HUMAN** in **uni_human**, Keratin, type II cuticular Hb4 OS=Homo sapiens GN=KRT84 PE=2 SV=2

Match to Query 17394: 1489.815912 from(497.612580,3+) intensity(3816337.0000) rtinseconds(1480) scans(7859) index(21162)
Title: 160219_Sunil_SDSI_A_Spectrum074466_scans__7859_RTINSECONDS=1480
Data file L:\\QE_2016\\160219_Sunil_KAP_LKC\\TMgf\\T\\T160219_Sunil_SDSI_A.mgf

Click mouse within plot area to zoom in by factor of two about that point
Or, Plot from 100 to 1500 Da Full range
Label all possible matches ○ Label matches used for scoring ◉
Show Y-axis □

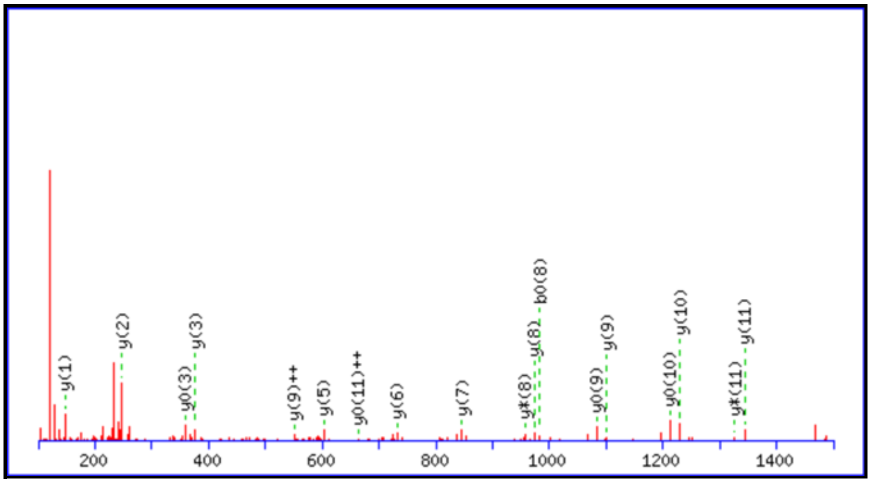

Monoisotopic mass of neutral peptide Mr(calc): 1489.8140
Fixed modifications: Carbamidomethyl (C) (apply to specified residues or termini only)
Ions Score: 54 **Expect:** 0.0004
Matches : 18/120 fragment ions using 40 most intense peaks (help)

#	b	b^{++}	b*	b^{*++}	b^{0}	b^{0++}	Seq.	y	y^{++}	y*	y^{*++}	y^{0}	y^{0++}	#
1	148.0757	74.5415					F							12
2	261.1598	131.0835					L	***1343.7529***	672.3801	1326.7264	663.8668	1325.7423	663.3748	11
3	390.2023	195.6048			372.1918	186.5995	E	***1230.6688***	615.8381	1213.6423	607.3248	1212.6583	606.8328	10
4	518.2609	259.6341	501.2344	251.1208	500.2504	250.6288	Q	***1101.6263***	551.3168	1084.5997	542.8035	1083.6157	542.3115	9
5	646.3195	323.6634	629.2930	315.1501	628.3089	314.6581	Q	***973.5677***	487.2875	956.5411	478.7742	955.5571	478.2822	8
6	760.3624	380.6849	743.3359	372.1716	742.3519	371.6796	N	***845.5091***	423.2582	828.4825	414.7449	827.4985	414.2529	7
7	888.4574	444.7323	871.4308	436.2191	870.4468	435.7271	K	***731.4662***	366.2367	714.4396	357.7234	713.4556	357.2314	6
8	1001.5415	501.2744	984.5149	492.7611	983.5309	492.2691	L	***603.3712***	302.1892	586.3447	293.6760	585.3606	293.1840	5
9	1114.6255	557.8164	1097.5990	549.3031	1096.6150	548.8111	L	490.2871	245.6472	473.2606	237.1339	472.2766	236.6419	4
10	1243.6681	622.3377	1226.6416	613.8244	1225.6575	613.3324	E	***377.2031***	189.1052	360.1765	180.5919	359.1925	180.0999	3
11	1344.7158	672.8615	1327.6892	664.3483	1326.7052	663.8563	T	***248.1605***	124.5839	231.1339	116.0706	230.1499	115.5786	2
12							K	***147.1128***	74.0600	130.0863	65.5468			1

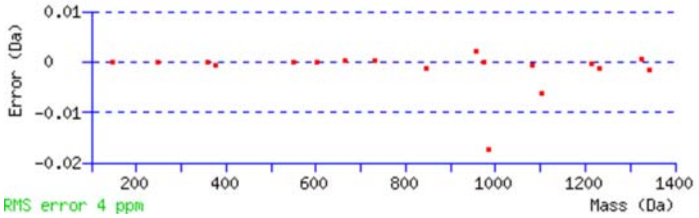

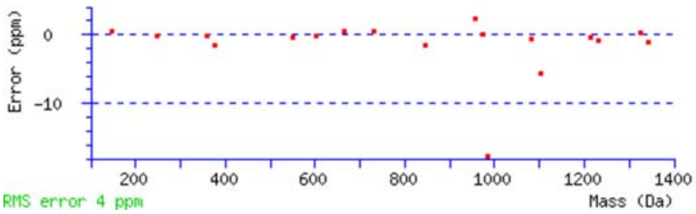

NCBI **BLAST** search of FLEQQNKLLETK
(Parameters: blastp, nr protein database, expect=20000, no filter, PAM30)
Other BLAST web gateways

All matches to this query

Score	Mr(calc)	Delta	Sequence
54.2	1489.8140	0.0019	FLEQQNKLLETK
0.4	1487.8069	2.0090	NVHRPPRQRDIT

Mascot: http://www.matrixscience.com/

Mascot Search Results

Peptide View

MS/MS Fragmentation of **FLEQQNKLLETK**
Found in **ch12u_Q9NSB2|KRT84_HUMAN** in **uni_human**, Keratin, type II cuticular Hb4 OS=Homo sapiens GN=KRT84 PE=2 SV=2

Match to Query 17396: 1489.816092 from(497.612640,3+) intensity(369647.0313) rtinseconds(1566) scans(8234) index(36752)
Title: 160219_Sunil_SDSI_A_Spectrum091337_scans__8234_RTINSECONDS=1566
Data file L:\\QE_2016\\160219_Sunil_KAP_LKC\\TMgf\\T\\T160219_Sunil_SDSI_A.mgf

Click mouse within plot area to zoom in by factor of two about that point
Or, Plot from 100 to 1400 Da Full range
Label all possible matches ○ Label matches used for scoring ◉
Show Y-axis □

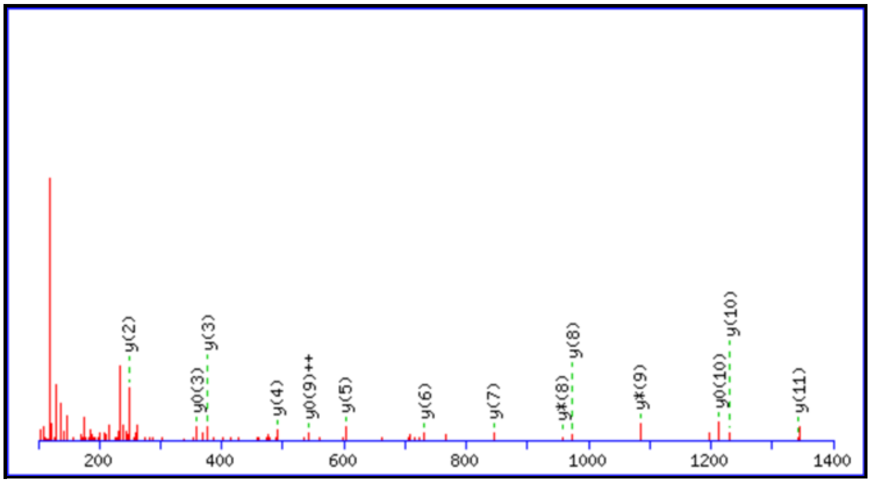

Monoisotopic mass of neutral peptide Mr(calc): 1489.8140
Fixed modifications: Carbamidomethyl (C) (apply to specified residues or termini only)
Ions Score: 62 **Expect:** 6.1e-005
Matches : 14/120 fragment ions using 23 most intense peaks (help)

#	b	b^{++}	b^{*}	b^{*++}	b^{0}	b^{0++}	Seq.	y	y^{++}	y^{*}	y^{*++}	y^{0}	y^{0++}	#
1	148.0757	74.5415					F							12
2	261.1598	131.0835					L	***1343.7529***	672.3801	1326.7264	663.8668	1325.7423	663.3748	11
3	390.2023	195.6048			372.1918	186.5995	E	***1230.6688***	615.8381	1213.6423	607.3248	1212.6583	606.8328	10
4	518.2609	259.6341	501.2344	251.1208	500.2504	250.6288	Q	1101.6263	551.3168	1084.5997	542.8035	1083.6157	542.3115	9
5	646.3195	323.6634	629.2930	315.1501	628.3089	314.6581	Q	***973.5677***	487.2875	956.5411	478.7742	955.5571	478.2822	8
6	760.3624	380.6849	743.3359	372.1716	742.3519	371.6796	N	***845.5091***	423.2582	828.4825	414.7449	827.4985	414.2529	7
7	888.4574	444.7323	871.4308	436.2191	870.4468	435.7271	K	***731.4662***	366.2367	714.4396	357.7234	713.4556	357.2314	6
8	1001.5415	501.2744	984.5149	492.7611	983.5309	492.2691	L	***603.3712***	302.1892	586.3447	293.6760	585.3606	293.1840	5
9	1114.6255	557.8164	1097.5990	549.3031	1096.6150	548.8111	L	***490.2871***	245.6472	473.2606	237.1339	472.2766	236.6419	4
10	1243.6681	622.3377	1226.6416	613.8244	1225.6575	613.3324	E	***377.2031***	189.1052	360.1765	180.5919	359.1925	180.0999	3
11	1344.7158	672.8615	1327.6892	664.3483	1326.7052	663.8563	T	***248.1605***	124.5839	231.1339	116.0706	230.1499	115.5786	2
12							K	147.1128	74.0600	130.0863	65.5468			1

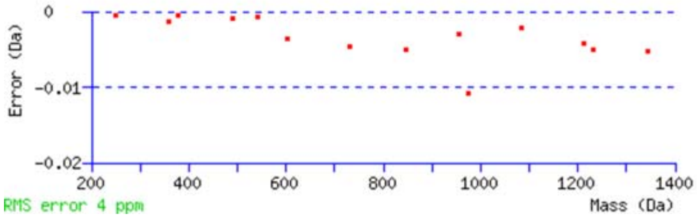

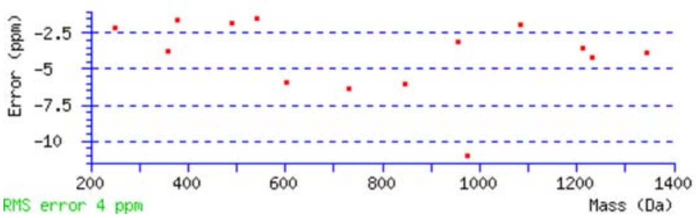

NCBI **BLAST** search of FLEQQNKLLETK
(Parameters: blastp, nr protein database, expect=20000, no filter, PAM30)
Other BLAST web gateways

All matches to this query

Score	Mr(calc)	Delta	Sequence
62.3	1489.8140	0.0021	FLEQQNKLLETK
0.5	1487.8069	2.0092	NVHRPPRQRDIT

Mascot: http://www.matrixscience.com/

Mascot Search Results

Peptide View

MS/MS Fragmentation of **FLEQQNKLLETK**
Found in **ch12u_Q9NSB2|KRT84_HUMAN** in **uni_human**, Keratin, type II cuticular Hb4 OS=Homo sapiens GN=KRT84 PE=2 SV=2

Match to Query 17455: 1490.804052 from(497.941960,3+) intensity(8270727.5000) rtinseconds(1543) scans(8227) index(21485)
Title: 160219_Sunil_SDSI_A_Spectrum074789_scans__8227_RTINSECONDS=1543
Data file L:\\QE_2016\\160219_Sunil_KAP_LKC\\TMgf\\T\\T160219_Sunil_SDSI_A.mgf

Click mouse within plot area to zoom in by factor of two about that point
Or, Plot from 100 to 1600 Da Full range
Label all possible matches ○ Label matches used for scoring ◉
Show Y-axis □

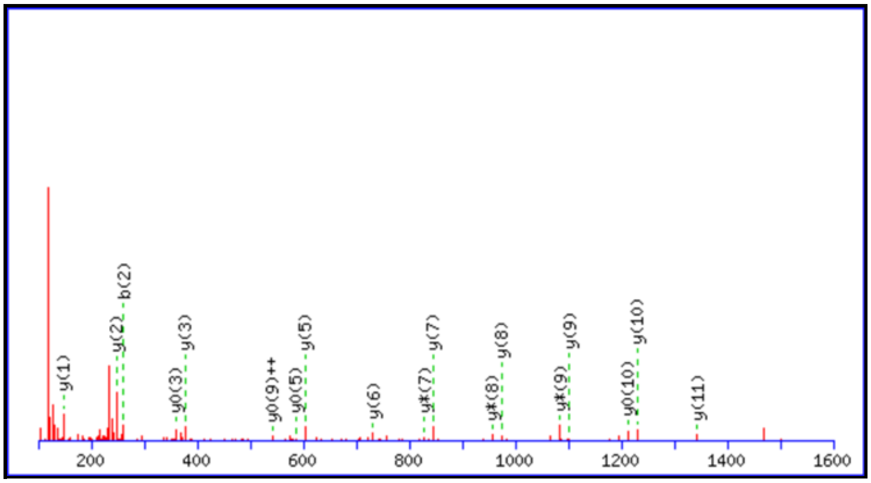

Monoisotopic mass of neutral peptide Mr(calc): 1489.8140
Fixed modifications: Carbamidomethyl (C) (apply to specified residues or termini only)
Ions Score: 51 **Expect:** 0.00093
Matches : 18/120 fragment ions using 46 most intense peaks (help)

#	b	b^{++}	b*	b^{*++}	b^{0}	b^{0++}	Seq.	y	y^{++}	y*	y^{*++}	y^{0}	y^{0++}	#
1	148.0757	74.5415					F							12
2	**261.1598**	131.0835					L	***1343.7529***	672.3801	1326.7264	663.8668	1325.7423	663.3748	11
3	390.2023	195.6048			372.1918	186.5995	E	***1230.6688***	615.8381	1213.6423	607.3248	1212.6583	606.8328	10
4	518.2609	259.6341	501.2344	251.1208	500.2504	250.6288	Q	***1101.6263***	551.3168	1084.5997	542.8035	1083.6157	542.3115	9
5	646.3195	323.6634	629.2930	315.1501	628.3089	314.6581	Q	***973.5677***	487.2875	956.5411	478.7742	955.5571	478.2822	8
6	760.3624	380.6849	743.3359	372.1716	742.3519	371.6796	N	***845.5091***	423.2582	828.4825	414.7449	827.4985	414.2529	7
7	888.4574	444.7323	871.4308	436.2191	870.4468	435.7271	K	***731.4662***	366.2367	714.4396	357.7234	713.4556	357.2314	6
8	1001.5415	501.2744	984.5149	492.7611	983.5309	492.2691	L	***603.3712***	302.1892	586.3447	293.6760	585.3606	293.1840	5
9	1114.6255	557.8164	1097.5990	549.3031	1096.6150	548.8111	L	490.2871	245.6472	473.2606	237.1339	472.2766	236.6419	4
10	1243.6681	622.3377	1226.6416	613.8244	1225.6575	613.3324	E	***377.2031***	189.1052	360.1765	180.5919	359.1925	180.0999	3
11	1344.7158	672.8615	1327.6892	664.3483	1326.7052	663.8563	T	***248.1605***	124.5839	231.1339	116.0706	230.1499	115.5786	2
12							K	***147.1128***	74.0600	130.0863	65.5468			1

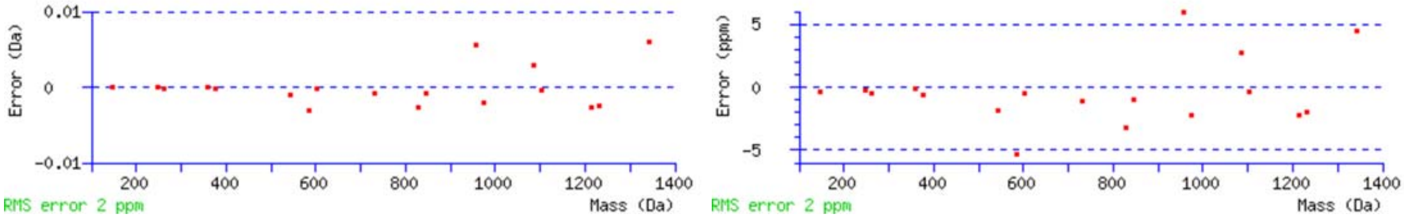

NCBI **BLAST** search of FLEQQNKLLETK
(Parameters: blastp, nr protein database, expect=20000, no filter, PAM30)
Other BLAST web gateways

All matches to this query

Score	Mr(calc)	Delta	Sequence
51.2	1489.8140	0.9900	FLEQQNKLLETK
23.9	1490.7980	0.0060	FLEQQNKLLETK
18.3	1490.7980	0.0060	FLEQQNKLLETK
18.1	1490.7980	0.0060	FLEQQNKLLETK
0.6	1488.7909	2.0131	NVHRPPRQRDIT
0.6	1488.7909	2.0131	NVHRPPRQRDIT

Mascot: http://www.matrixscience.com/

Mascot Search Results

Peptide View

MS/MS Fragmentation of **FLEQQNKLLETK**
Found in **ch12u_Q9NSB2|KRT84_HUMAN** in **uni_human**, Keratin, type II cuticular Hb4 OS=Homo sapiens GN=KRT84 PE=2 SV=2

Match to Query 17445: 1490.797328 from(746.405940,2+) intensity(1330040.1250) rtinseconds(1879) scans(10041) index(7723)
Title: 160219_Sunil_SDSI_A_Spectrum059831_scans__10041_RTINSECONDS=1879
Data file L:\\QE_2016\\160219_Sunil_KAP_LKC\\TMgf\\T\\T160219_Sunil_SDSI_A.mgf

Click mouse within plot area to zoom in by factor of two about that point
Or, Plot from 100 to 1300 Da Full range
Label all possible matches ○ Label matches used for scoring ◉
Show Y-axis ☐

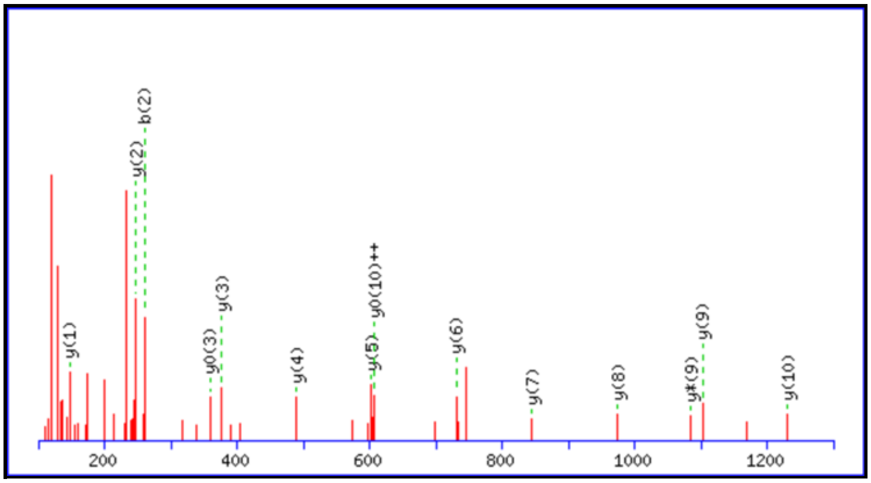

Monoisotopic mass of neutral peptide Mr(calc): 1490.7980
Fixed modifications: Carbamidomethyl (C) (apply to specified residues or termini only)
Variable modifications:
Q5 : Deamidated (NQ)
Ions Score: 74 **Expect:** 5e-006
Matches : 14/120 fragment ions using 24 most intense peaks (help)

#	b	b^{++}	b*	b*$^{++}$	b^{0}	b^{0++}	Seq.	y	y^{++}	y*	y*$^{++}$	y^{0}	y^{0++}	#
1	148.0757	74.5415					F							12
2	**261.1598**	131.0835					L	1344.7369	672.8721	1327.7104	664.3588	1326.7264	663.8668	11
3	390.2023	195.6048			372.1918	186.5995	E	***1231.6529***	616.3301	1214.6263	607.8168	1213.6423	607.3248	10
4	518.2609	259.6341	501.2344	251.1208	500.2504	250.6288	Q	***1102.6103***	551.8088	1085.5837	543.2955	1084.5997	542.8035	9
5	647.3035	324.1554	630.2770	315.6421	629.2930	315.1501	Q	***974.5517***	487.7795	957.5251	479.2662	956.5411	478.7742	8
6	761.3464	381.1769	744.3199	372.6636	743.3359	372.1716	N	***845.5091***	423.2582	828.4825	414.7449	827.4985	414.2529	7
7	889.4414	445.2243	872.4149	436.7111	871.4308	436.2191	K	***731.4662***	366.2367	714.4396	357.7234	713.4556	357.2314	6
8	1002.5255	501.7664	985.4989	493.2531	984.5149	492.7611	L	***603.3712***	302.1892	586.3447	293.6760	585.3606	293.1840	5
9	1115.6095	558.3084	1098.5830	549.7951	1097.5990	549.3031	L	***490.2871***	245.6472	473.2606	237.1339	472.2766	236.6419	4
10	1244.6521	622.8297	1227.6256	614.3164	1226.6416	613.8244	E	***377.2031***	189.1052	360.1765	180.5919	359.1925	180.0999	3
11	1345.6998	673.3535	1328.6733	664.8403	1327.6892	664.3483	T	***248.1605***	124.5839	231.1339	116.0706	230.1499	115.5786	2
12							K	***147.1128***	74.0600	130.0863	65.5468			1

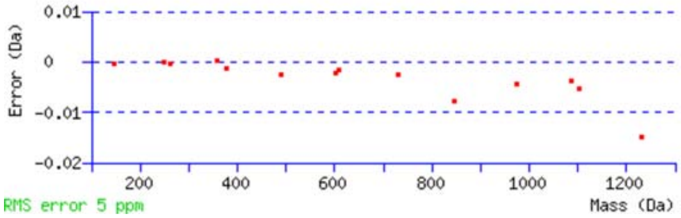

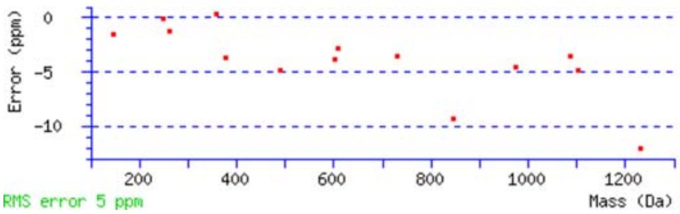

NCBI **BLAST** search of FLEQQNKLLETK
(Parameters: blastp, nr protein database, expect=20000, no filter, PAM30)
Other BLAST web gateways

All matches to this query

Score	Mr(calc)	Delta	Sequence	Site Analysis
74.2	1490.7980	-0.0007	FLEQQNKLLETK	Deamidated Q5 93.13%
59.9	1490.7980	-0.0007	FLEQQNKLLETK	Deamidated N6 3.44%
59.8	1490.7980	-0.0007	FLEQQNKLLETK	Deamidated Q4 3.42%
9.8	1490.7981	-0.0007	FQALQSVDILETK	

Mascot: http://www.matrixscience.com/

Mascot Search Results

Peptide View

MS/MS Fragmentation of **FLEQQNKLLETK**
Found in **ch12u_Q9NSB2|KRT84_HUMAN** in **uni_human**, Keratin, type II cuticular Hb4 OS=Homo sapiens GN=KRT84 PE=2 SV=2

Match to Query 17449: 1490.798352 from(497.940060,3+) intensity(5874160.0000) rtinseconds(1809) scans(9629) index(7352)
Title: 160219_Sunil_SDSI_A_Spectrum059460_scans__9629_RTINSECONDS=1809
Data file L:\\QE_2016\\160219_Sunil_KAP_LKC\\TMgf\\T\\T160219_Sunil_SDSI_A.mgf

Click mouse within plot area to zoom in by factor of two about that point
Or, Plot from 100 to 1400 Da Full range
Label all possible matches ○ Label matches used for scoring ◉
Show Y-axis □

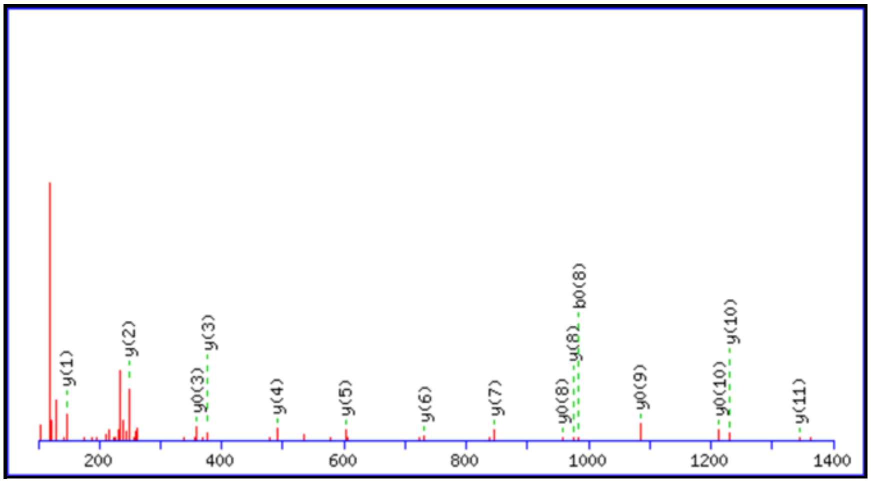

Monoisotopic mass of neutral peptide Mr(calc): 1490.7980
Fixed modifications: Carbamidomethyl (C) (apply to specified residues or termini only)
Variable modifications:
N6 : Deamidated (NQ)
Ions Score: 67 **Expect:** 2.7e-005
Matches : 15/120 fragment ions using 27 most intense peaks (help)

#	b	b^{++}	b*	b^{*++}	b^0	b^{0++}	Seq.	y	y^{++}	y*	y^{*++}	y^0	y^{0++}	#
1	148.0757	74.5415					F							12
2	261.1598	131.0835					L	***1344.7369***	672.8721	1327.7104	664.3588	1326.7264	663.8668	11
3	390.2023	195.6048			372.1918	186.5995	E	***1231.6529***	616.3301	1214.6263	607.8168	1213.6423	607.3248	10
4	518.2609	259.6341	501.2344	251.1208	500.2504	250.6288	Q	1102.6103	551.8088	1085.5837	543.2955	1084.5997	542.8035	9
5	646.3195	323.6634	629.2930	315.1501	628.3089	314.6581	Q	***974.5517***	487.7795	957.5251	479.2662	956.5411	478.7742	8
6	761.3464	381.1769	744.3199	372.6636	743.3359	372.1716	N	***846.4931***	423.7502	829.4666	415.2369	828.4825	414.7449	7
7	889.4414	445.2243	872.4149	436.7111	871.4308	436.2191	K	***731.4662***	366.2367	714.4396	357.7234	713.4556	357.2314	6
8	1002.5255	501.7664	985.4989	493.2531	984.5149	492.7611	L	***603.3712***	302.1892	586.3447	293.6760	585.3606	293.1840	5
9	1115.6095	558.3084	1098.5830	549.7951	1097.5990	549.3031	L	***490.2871***	245.6472	473.2606	237.1339	472.2766	236.6419	4
10	1244.6521	622.8297	1227.6256	614.3164	1226.6416	613.8244	E	***377.2031***	189.1052	360.1765	180.5919	359.1925	180.0999	3
11	1345.6998	673.3535	1328.6733	664.8403	1327.6892	664.3483	T	***248.1605***	124.5839	231.1339	116.0706	230.1499	115.5786	2
12							K	***147.1128***	74.0600	130.0863	65.5468			1

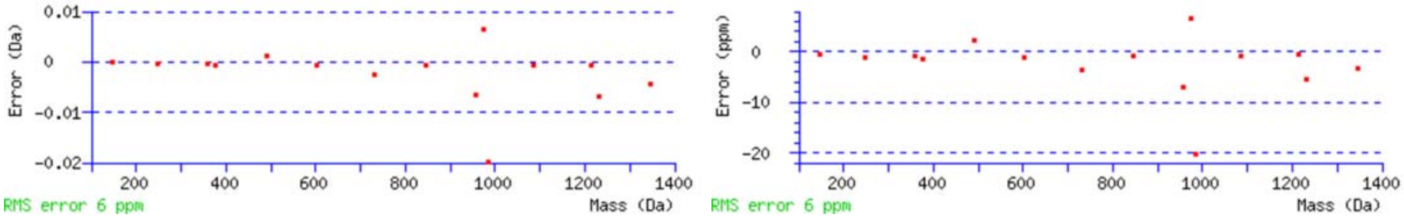

NCBI **BLAST** search of FLEQQNKLLETK
(Parameters: blastp, nr protein database, expect=20000, no filter, PAM30)
Other BLAST web gateways

All matches to this query

Score	Mr(calc)	Delta	Sequence	Site Analysis
66.8	1490.7980	0.0003	FLEQQNKLLETK	Deamidated N6 95.13%
53.6	1490.7980	0.0003	FLEQQNKLLETK	Deamidated Q5 4.56%
41.8	1490.7980	0.0003	FLEQQNKLLETK	Deamidated Q4 0.30%
14.5	1490.7981	0.0003	FQALQSVDILETK	
1.6	1488.7909	2.0074	NVHRPPRQRDIT	
1.6	1488.7909	2.0074	NVHRPPRQRDIT	

Mascot: http://www.matrixscience.com/

Mascot Search Results

Peptide View

MS/MS Fragmentation of **FLEQQNKLLETK**
Found in **ch12u_Q9NSB2|KRT84_HUMAN** in **uni_human**, Keratin, type II cuticular Hb4 OS=Homo sapiens GN=KRT84 PE=2 SV=2

Match to Query 17448: 1490.798262 from(497.940030,3+) intensity(3326579.7500) rtinseconds(1644) scans(8686) index(37157)
Title: 160219_Sunil_SDSI_A_Spectrum091742_scans__8686_RTINSECONDS=1644
Data file L:\\QE_2016\\160219_Sunil_KAP_LKC\\TMgf\\T\\T160219_Sunil_SDSI_A.mgf

Click mouse within plot area to zoom in by factor of two about that point
Or, Plot from 100 to 1400 Da Full range
Label all possible matches ○ Label matches used for scoring ◉
Show Y-axis ☐

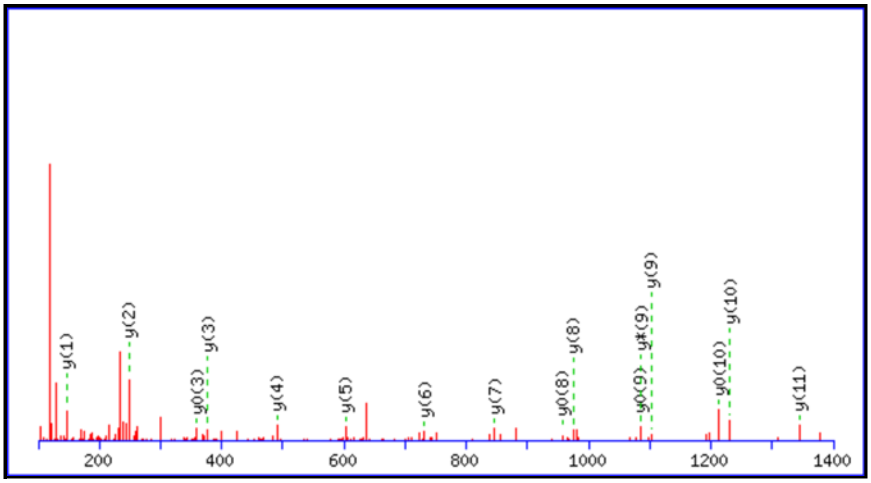


```
Monoisotopic mass of neutral peptide Mr(calc): 1490.7980
Fixed modifications: Carbamidomethyl (C) (apply to specified residues or termini only)
Variable modifications:
N6     : Deamidated (NQ)
Ions Score: 67  Expect: 2.9e-005
Matches : 16/120 fragment ions using 37 most intense peaks   (help)
```

#	b	b^{++}	b*	b^{*++}	b^{0}	b^{0++}	Seq.	y	y^{++}	y*	y^{*++}	y^{0}	y^{0++}	#
1	148.0757	74.5415					F							12
2	261.1598	131.0835					L	***1344.7369***	672.8721	1327.7104	664.3588	1326.7264	663.8668	11
3	390.2023	195.6048			372.1918	186.5995	E	***1231.6529***	616.3301	1214.6263	607.8168	1213.6423	607.3248	10
4	518.2609	259.6341	501.2344	251.1208	500.2504	250.6288	Q	***1102.6103***	551.8088	1085.5837	543.2955	1084.5997	542.8035	9
5	646.3195	323.6634	629.2930	315.1501	628.3089	314.6581	Q	***974.5517***	487.7795	957.5251	479.2662	956.5411	478.7742	8
6	761.3464	381.1769	744.3199	372.6636	743.3359	372.1716	N	***846.4931***	423.7502	829.4666	415.2369	828.4825	414.7449	7
7	889.4414	445.2243	872.4149	436.7111	871.4308	436.2191	K	***731.4662***	366.2367	714.4396	357.7234	713.4556	357.2314	6
8	1002.5255	501.7664	985.4989	493.2531	984.5149	492.7611	L	***603.3712***	302.1892	586.3447	293.6760	585.3606	293.1840	5
9	1115.6095	558.3084	1098.5830	549.7951	1097.5990	549.3031	L	***490.2871***	245.6472	473.2606	237.1339	472.2766	236.6419	4
10	1244.6521	622.8297	1227.6256	614.3164	1226.6416	613.8244	E	***377.2031***	189.1052	360.1765	180.5919	359.1925	180.0999	3
11	1345.6998	673.3535	1328.6733	664.8403	1327.6892	664.3483	T	***248.1605***	124.5839	231.1339	116.0706	230.1499	115.5786	2
12							K	***147.1128***	74.0600	130.0863	65.5468			1

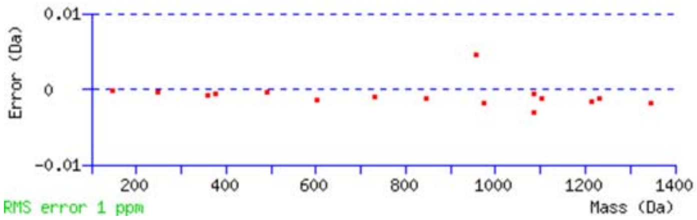

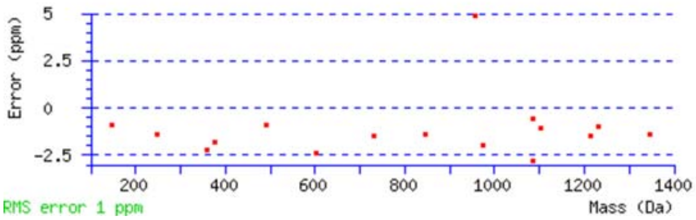

NCBI **BLAST** search of FLEQQNKLLETK
(Parameters: blastp, nr protein database, expect=20000, no filter, PAM30)
Other BLAST web gateways

All matches to this query

Score	Mr(calc)	Delta	Sequence	Site Analysis
66.6	1490.7980	0.0002	FLEQQNKLLETK	Deamidated N6 94.97%
53.5	1490.7980	0.0002	FLEQQNKLLETK	Deamidated Q5 4.72%
41.8	1490.7980	0.0002	FLEQQNKLLETK	Deamidated Q4 0.31%
7.6	1490.7981	0.0002	FQALQSVDILETK	

Mascot: http://www.matrixscience.com/

Mascot Search Results

Peptide View

MS/MS Fragmentation of **VRFLEQQNKLLETK**
Found in **ch12u_Q9NSB2|KRT84_HUMAN** in **uni_human**, Keratin, type II cuticular Hb4 OS=Homo sapiens GN=KRT84 PE=2 SV=2

Match to Query 23719: 1744.980792 from(582.667540,3+) intensity(430751.9688) rtinseconds(1495) scans(7948) index(21240)
Title: 160219_Sunil_SDSI_A_Spectrum074544_scans__7948_RTINSECONDS=1495
Data file L:\\QE_2016\\160219_Sunil_KAP_LKC\\TMgf\\T\\T160219_Sunil_SDSI_A.mgf

Click mouse within plot area to zoom in by factor of two about that point
Or, Plot from 100 to 1800 Da Full range
Label all possible matches ○ Label matches used for scoring ◉
Show Y-axis □

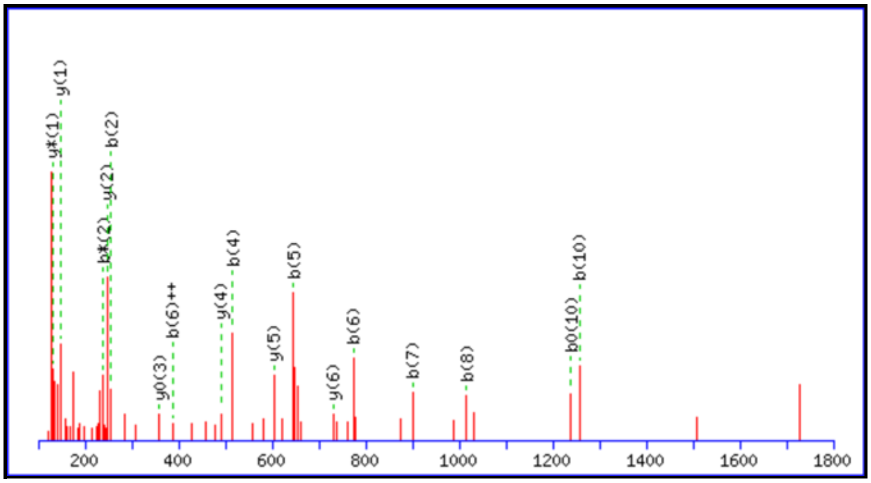

Monoisotopic mass of neutral peptide Mr(calc): 1744.9835
Fixed modifications: Carbamidomethyl (C) (apply to specified residues or termini only)
Ions Score: 44 **Expect:** 0.0023
Matches : 17/144 fragment ions using 30 most intense peaks (help)

#	b	b^{++}	b*	b^{*++}	b^0	b^{0++}	Seq.	y	y^{++}	y*	y^{*++}	y^0	y^{0++}	#
1	100.0757	50.5415					V							14
2	**256.1768**	128.5920	239.1503	120.0788			R	1646.9224	823.9649	1629.8959	815.4516	1628.9119	814.9596	13
3	403.2452	202.1262	386.2187	193.6130			F	1490.8213	745.9143	1473.7948	737.4010	1472.8108	736.9090	12
4	**516.3293**	258.6683	499.3027	250.1550			L	1343.7529	672.3801	1326.7264	663.8668	1325.7423	663.3748	11
5	**645.3719**	323.1896	628.3453	314.6763	627.3613	314.1843	E	1230.6688	615.8381	1213.6423	607.3248	1212.6583	606.8328	10
6	**773.4305**	**387.2189**	756.4039	378.7056	755.4199	378.2136	Q	1101.6263	551.3168	1084.5997	542.8035	1083.6157	542.3115	9
7	**901.4890**	451.2482	884.4625	442.7349	883.4785	442.2429	Q	973.5677	487.2875	956.5411	478.7742	955.5571	478.2822	8
8	**1015.5320**	508.2696	998.5054	499.7563	997.5214	499.2643	N	845.5091	423.2582	828.4825	414.7449	827.4985	414.2529	7
9	1143.6269	572.3171	1126.6004	563.8038	1125.6164	563.3118	K	**731.4662**	366.2367	714.4396	357.7234	713.4556	357.2314	6
10	**1256.7110**	628.8591	1239.6844	620.3459	1238.7004	619.8538	L	**603.3712**	302.1892	586.3447	293.6760	585.3606	293.1840	5
11	1369.7950	685.4012	1352.7685	676.8879	1351.7845	676.3959	L	**490.2871**	245.6472	473.2606	237.1339	472.2766	236.6419	4
12	1498.8376	749.9225	1481.8111	741.4092	1480.8271	740.9172	E	377.2031	189.1052	360.1765	180.5919	359.1925	180.0999	3
13	1599.8853	800.4463	1582.8588	791.9330	1581.8748	791.4410	T	**248.1605**	124.5839	231.1339	116.0706	230.1499	115.5786	2
14							K	**147.1128**	74.0600	130.0863	65.5468			1

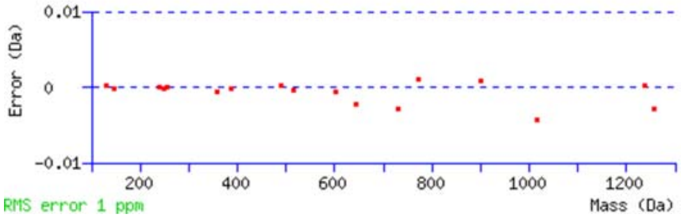

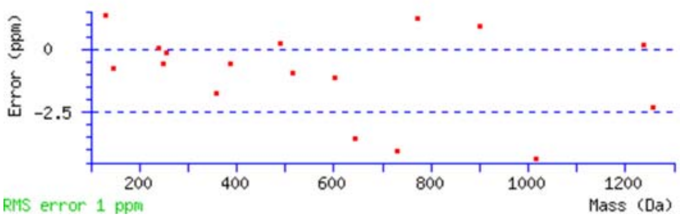

NCBI **BLAST** search of VRFLEQQNKLLETK
(Parameters: blastp, nr protein database, expect=20000, no filter, PAM30)
Other BLAST web gateways

All matches to this query

Score	Mr(calc)	Delta	Sequence
44.4	1744.9835	-0.0027	VRFLEQQNKLLETK
0.2	1744.9869	-0.0061	KSLESINSRLQLVMK
0.1	1743.9784	1.0024	KGFRLLNPHPKPNPK

Mascot: http://www.matrixscience.com/

Mascot Search Results

Peptide View

MS/MS Fragmentation of **LGLDIEIATYR**
Found in **ch12u_Q9NSB2|KRT84_HUMAN** in **uni_human**, Keratin, type II cuticular Hb4 OS=Homo sapiens GN=KRT84 PE=2 SV=2

Match to Query 10372: 1262.686988 from(632.350770,2+) intensity(2488661.0000) rtinseconds(2670) scans(14614) index(42248)
Title: 160219_Sunil_SDSI_A_Spectrum096833_scans__14614_RTINSECONDS=2670
Data file L:\\QE_2016\\160219_Sunil_KAP_LKC\\TMgf\\T\\T160219_Sunil_SDSI_A.mgf

Click mouse within plot area to zoom in by factor of two about that point
Or, Plot from 0 to 1200 Da Full range
Label all possible matches ○ Label matches used for scoring ◉
Show Y-axis ☐

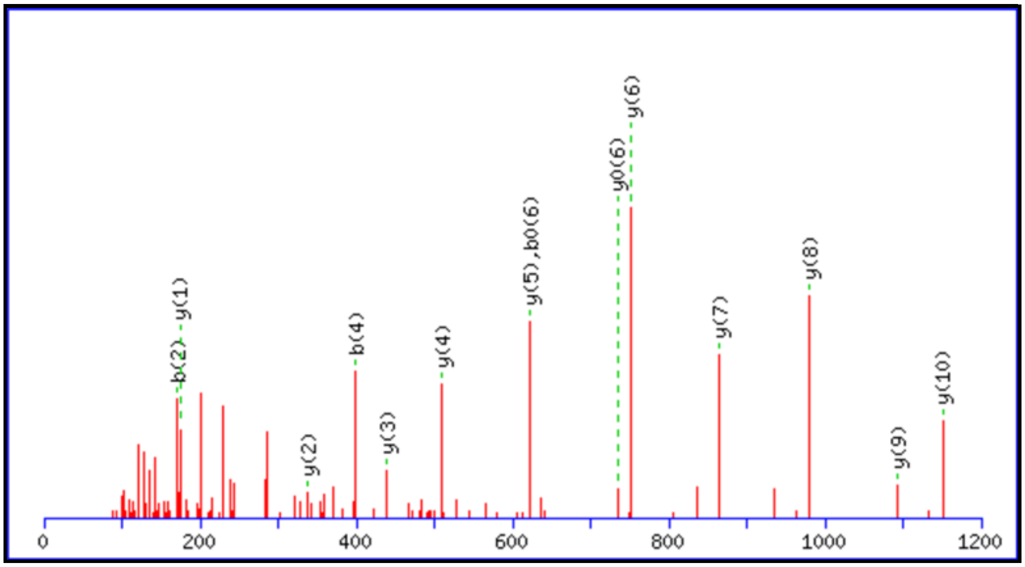

Monoisotopic mass of neutral peptide Mr(calc): 1262.6870
Fixed modifications: Carbamidomethyl (C) (apply to specified residues or termini only)
Ions Score: 89 **Expect:** 1.3e-007
Matches : 14/90 fragment ions using 20 most intense peaks (help)

#	b	b^{++}	b^{0}	b^{0++}	Seq.	y	y^{++}	y^{*}	y^{*++}	y^{0}	y^{0++}	#
1	114.0913	57.5493			L							11
2	171.1128	86.0600			G	1150.6103	575.8088	1133.5837	567.2955	1132.5997	566.8035	10
3	284.1969	142.6021			L	1093.5888	547.2980	1076.5623	538.7848	1075.5782	538.2928	9
4	399.2238	200.1155	381.2132	191.1103	D	980.5047	490.7560	963.4782	482.2427	962.4942	481.7507	8
5	512.3079	256.6576	494.2973	247.6523	I	865.4778	433.2425	848.4512	424.7293	847.4672	424.2373	7
6	641.3505	321.1789	623.3399	312.1736	E	752.3937	376.7005	735.3672	368.1872	734.3832	367.6952	6
7	754.4345	377.7209	736.4240	368.7156	I	623.3511	312.1792	606.3246	303.6659	605.3406	303.1739	5
8	825.4716	413.2395	807.4611	404.2342	A	510.2671	255.6372	493.2405	247.1239	492.2565	246.6319	4
9	926.5193	463.7633	908.5088	454.7580	T	439.2300	220.1186	422.2034	211.6053	421.2194	211.1133	3

10	1089.5827	545.2950	1071.5721	536.2897	Y	338.1823	169.5948	321.1557	161.0815			2
11					R	175.1190	88.0631	158.0924	79.5498			1

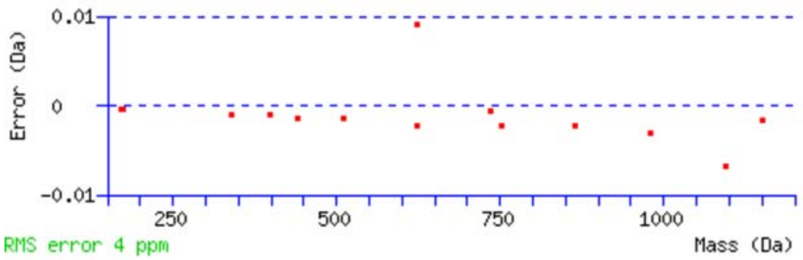

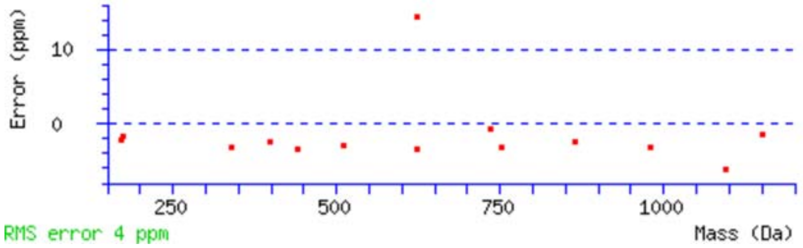

NCBI **BLAST** search of LGLDIEIATYR
(Parameters: blastp, nr protein database, expect=20000, no filter, PAM30)
Other BLAST web gateways

All matches to this query

Score	Mr(calc)	Delta	Sequence
88.9	1262.6870	-0.0000	LGLDIEIATYR
37.1	1262.6870	-0.0000	LALDVEIATYR
22.2	1262.6870	-0.0001	IAVDELFTSLR
8.0	1262.6805	0.0065	VALLIGNMNYR
5.7	1262.6758	0.0112	EVELIGASYIGL
5.3	1262.6983	-0.0113	IGAQPVEIPPSR
1.0	1262.6870	-0.0001	ISLGFELTVER
1.0	1262.6870	-0.0001	LSLGFELTVER
0.7	1262.6983	-0.0113	LVAAKAFTESAR
0.4	1262.6818	0.0051	LARMRGFWAR

Mascot: http://www.matrixscience.com/

Mascot Search Results

Peptide View

MS/MS Fragmentation of **LGLDIEIATYR**
Found in **ch12u_Q9NSB2|KRT84_HUMAN** in **uni_human**, Keratin, type II cuticular Hb4 OS=Homo sapiens GN=KRT84 PE=2 SV=2

Match to Query 10372: 1262.686988 from(632.350770,2+) intensity(2488661.0000) rtinseconds(2670) scans(14614) index(42248)
Title: 160219_Sunil_SDSI_A_Spectrum096833_scans__14614_RTINSECONDS=2670
Data file L:\\QE_2016\\160219_Sunil_KAP_LKC\\TMgf\\T\\T160219_Sunil_SDSI_A.mgf

Click mouse within plot area to zoom in by factor of two about that point
Or, Plot from 0 to 1200 Da Full range
Label all possible matches ○ Label matches used for scoring ◉
Show Y-axis ☐

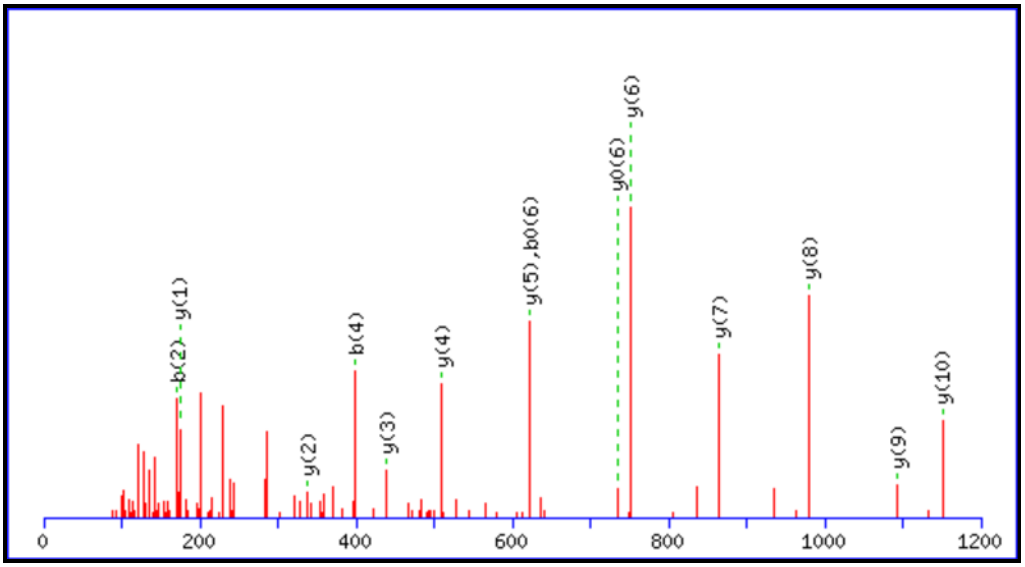

Monoisotopic mass of neutral peptide Mr(calc): 1262.6870
Fixed modifications: Carbamidomethyl (C) (apply to specified residues or termini only)
Ions Score: 89 **Expect:** 1.3e-007
Matches : 14/90 fragment ions using 20 most intense peaks (help)

#	b	b^{++}	b^{0}	b^{0++}	Seq.	y	y^{++}	y^{*}	y^{*++}	y^{0}	y^{0++}	#
1	114.0913	57.5493			L							11
2	**171.1128**	86.0600			G	***1150.6103***	575.8088	1133.5837	567.2955	1132.5997	566.8035	10
3	284.1969	142.6021			L	***1093.5888***	547.2980	1076.5623	538.7848	1075.5782	538.2928	9
4	**399.2238**	200.1155	381.2132	191.1103	D	***980.5047***	490.7560	963.4782	482.2427	962.4942	481.7507	8
5	512.3079	256.6576	494.2973	247.6523	I	***865.4778***	433.2425	848.4512	424.7293	847.4672	424.2373	7
6	641.3505	321.1789	623.3399	312.1736	E	***752.3937***	376.7005	735.3672	368.1872	734.3832	367.6952	6
7	754.4345	377.7209	736.4240	368.7156	I	***623.3511***	312.1792	606.3246	303.6659	605.3406	303.1739	5
8	825.4716	413.2395	807.4611	404.2342	A	***510.2671***	255.6372	493.2405	247.1239	492.2565	246.6319	4
9	926.5193	463.7633	908.5088	454.7580	T	***439.2300***	220.1186	422.2034	211.6053	421.2194	211.1133	3

**10**	1089.5827	545.2950	1071.5721	536.2897	**Y**	*338.1823*	169.5948	321.1557	161.0815			**2**
**11**					**R**	*175.1190*	88.0631	158.0924	79.5498			**1**

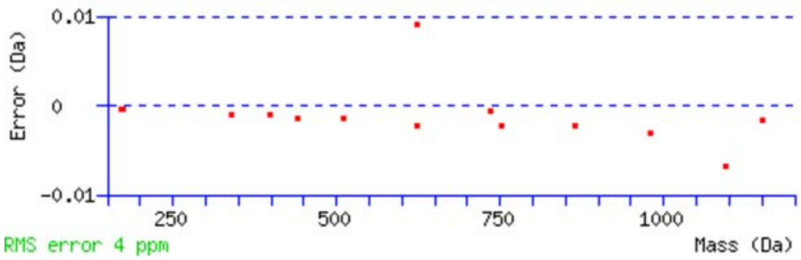

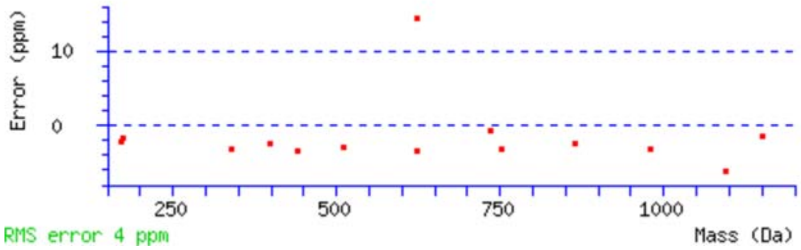

NCBI **BLAST** search of LGLDIEIATYR
(Parameters: blastp, nr protein database, expect=20000, no filter, PAM30)
Other BLAST web gateways

All matches to this query

Score	Mr(calc)	Delta	Sequence
88.9	1262.6870	-0.0000	LGLDIEIATYR
37.1	1262.6870	-0.0000	LALDVEIATYR
22.2	1262.6870	-0.0001	IAVDELFTSLR
8.0	1262.6805	0.0065	VALLIGNMNYR
5.7	1262.6758	0.0112	EVELIGASYIGL
5.3	1262.6983	-0.0113	IGAQPVEIPPSR
1.0	1262.6870	-0.0001	ISLGFELTVER
1.0	1262.6870	-0.0001	LSLGFELTVER
0.7	1262.6983	-0.0113	LVAAKAFTESAR
0.4	1262.6818	0.0051	LARMRGFWAR

Mascot: http://www.matrixscience.com/

Mascot Search Results

Peptide View

MS/MS Fragmentation of **LGLDIEIATYRR**
Found in **ch12u_Q9NSB2|KRT84_HUMAN** in **uni_human**, Keratin, type II cuticular Hb4 OS=Homo sapiens GN=KRT84 PE=2 SV=2

Match to Query 14971: 1418.786848 from(710.400700,2+) intensity(6355424.5000) rtinseconds(2300) scans(12635) index(25348)
Title: 160219_Sunil_SDSI_A_Spectrum078654_scans__12635_RTINSECONDS=2300
Data file L:\\QE_2016\\160219_Sunil_KAP_LKC\\TMgf\\T\\T160219_Sunil_SDSI_A.mgf

Click mouse within plot area to zoom in by factor of two about that point
Or, Plot from 100 to 1500 Da Full range
Label all possible matches ○ Label matches used for scoring ◉
Show Y-axis ☐

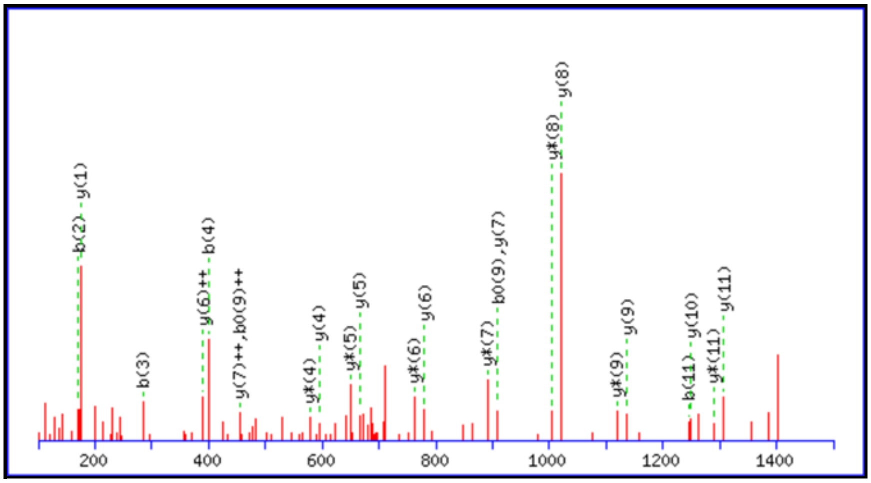

Monoisotopic mass of neutral peptide Mr(calc): 1418.7881
Fixed modifications: Carbamidomethyl (C) (apply to specified residues or termini only)
Ions Score: 50 **Expect:** 0.00083
Matches : 24/100 fragment ions using 50 most intense peaks (help)

#	b	b++	b*	b*++	b0	b0++	Seq.	y	y++	y*	y*++	y0	y0++	#
1	114.0913	57.5493					L							12
2	**171.1128**	86.0600					G	***1306.7114***	653.8593	1289.6848	645.3461	1288.7008	644.8540	11
3	**284.1969**	142.6021					L	***1249.6899***	625.3486	1232.6634	616.8353	1231.6793	616.3433	10
4	**399.2238**	200.1155			381.2132	191.1103	D	***1136.6058***	568.8066	1119.5793	560.2933	1118.5953	559.8013	9
5	512.3079	256.6576			494.2973	247.6523	I	***1021.5789***	511.2931	1004.5524	502.7798	1003.5683	502.2878	8
6	641.3505	321.1789			623.3399	312.1736	E	***908.4948***	454.7511	891.4683	446.2378	890.4843	445.7458	7
7	754.4345	377.7209			736.4240	368.7156	I	***779.4522***	390.2298	762.4257	381.7165	761.4417	381.2245	6
8	825.4716	413.2395			807.4611	404.2342	A	***666.3682***	333.6877	649.3416	325.1745	648.3576	324.6824	5
9	926.5193	463.7633			908.5088	454.7580	T	***595.3311***	298.1692	578.3045	289.6559	577.3205	289.1639	4
10	1089.5827	545.2950			1071.5721	536.2897	Y	494.2834	247.6453	477.2568	239.1321			3
11	**1245.6838**	623.3455	1228.6572	614.8322	1227.6732	614.3402	R	331.2201	166.1137	314.1935	157.6004			2
12							R	***175.1190***	88.0631	158.0924	79.5498			1

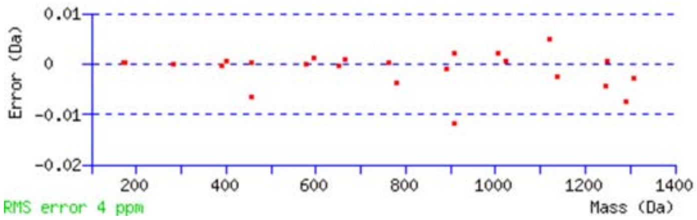

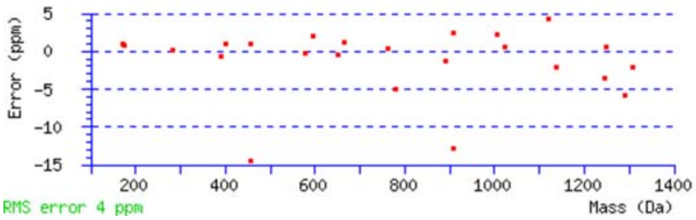

NCBI **BLAST** search of LGLDIEIATYRR
(Parameters: blastp, nr protein database, expect=20000, no filter, PAM30)
Other BLAST web gateways

All matches to this query

Score	Mr(calc)	Delta	Sequence
50.5	1418.7881	-0.0013	LGLDIEIATYRR
12.9	1418.7881	-0.0013	LGLLDRIVNYSR
8.4	1418.7881	-0.0013	LQLRSLQYLER
8.4	1418.7769	0.0099	LLGNTFVALSDLR
0.3	1418.7881	-0.0013	LLQRRQIFSQK

Mascot: http://www.matrixscience.com/

Mascot Search Results

Peptide View

MS/MS Fragmentation of **FLEQQNKLLETK**
Found in **ch12u_Q9NSB2|KRT84_HUMAN** in **uni_human**, Keratin, type II cuticular Hb4 OS=Homo sapiens GN=KRT84 PE=2 SV=2

Match to Query 17388: 1489.814788 from(745.914670,2+) intensity(3362591.2500) rtinseconds(1617) scans(8502) index(6357)
Title: 160219_Sunil_SDSI_A_Spectrum058465_scans__8502_RTINSECONDS=1617
Data file L:\\QE_2016\\160219_Sunil_KAP_LKC\\TMgf\\T\\T160219_Sunil_SDSI_A.mgf

Click mouse within plot area to zoom in by factor of two about that point
Or, Plot from 100 to 1500 Da Full range
Label all possible matches ○ Label matches used for scoring ◉
Show Y-axis ☐

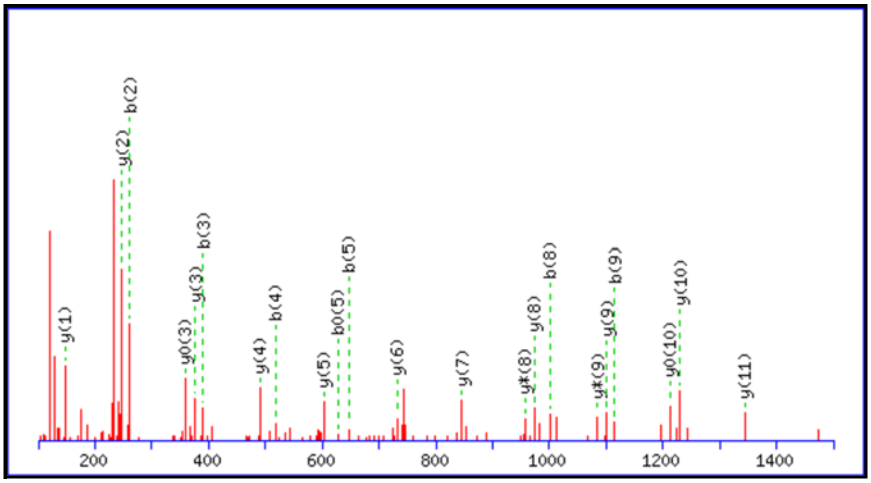

Monoisotopic mass of neutral peptide Mr(calc): 1489.8140
Fixed modifications: Carbamidomethyl (C) (apply to specified residues or termini only)
Ions Score: 85 **Expect:** 3.7e-007
Matches : 22/120 fragment ions using 39 most intense peaks (help)

#	b	b^{++}	b*	b^{*++}	b^0	b^{0++}	Seq.	y	y^{++}	y*	y^{*++}	y^0	y^{0++}	#
1	148.0757	74.5415					F							12
2	**261.1598**	131.0835					L	***1343.7529***	672.3801	1326.7264	663.8668	1325.7423	663.3748	11
3	**390.2023**	195.6048			372.1918	186.5995	E	***1230.6688***	615.8381	1213.6423	607.3248	1212.6583	606.8328	10
4	**518.2609**	259.6341	501.2344	251.1208	500.2504	250.6288	Q	***1101.6263***	551.3168	1084.5997	542.8035	1083.6157	542.3115	9
5	**646.3195**	323.6634	629.2930	315.1501	628.3089	314.6581	Q	***973.5677***	487.2875	956.5411	478.7742	955.5571	478.2822	8
6	760.3624	380.6849	743.3359	372.1716	742.3519	371.6796	N	***845.5091***	423.2582	828.4825	414.7449	827.4985	414.2529	7
7	888.4574	444.7323	871.4308	436.2191	870.4468	435.7271	K	***731.4662***	366.2367	714.4396	357.7234	713.4556	357.2314	6
8	**1001.5415**	501.2744	984.5149	492.7611	983.5309	492.2691	L	***603.3712***	302.1892	586.3447	293.6760	585.3606	293.1840	5
9	**1114.6255**	557.8164	1097.5990	549.3031	1096.6150	548.8111	L	***490.2871***	245.6472	473.2606	237.1339	472.2766	236.6419	4
10	1243.6681	622.3377	1226.6416	613.8244	1225.6575	613.3324	E	***377.2031***	189.1052	360.1765	180.5919	359.1925	180.0999	3
11	1344.7158	672.8615	1327.6892	664.3483	1326.7052	663.8563	T	***248.1605***	124.5839	231.1339	116.0706	230.1499	115.5786	2
12							K	***147.1128***	74.0600	130.0863	65.5468			1

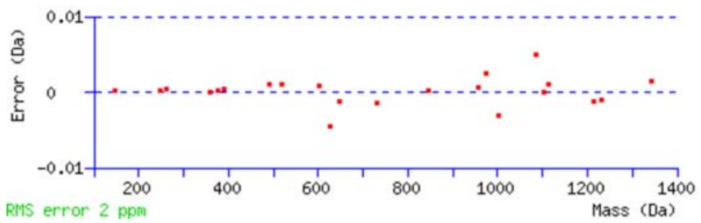

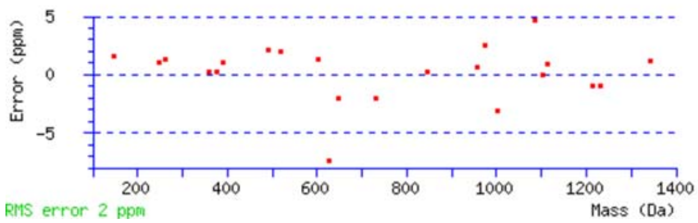

NCBI **BLAST** search of FLEQQNKLLETK
(Parameters: blastp, nr protein database, expect=20000, no filter, PAM30)
Other BLAST web gateways

All matches to this query

Score	Mr(calc)	Delta	Sequence
84.9	1489.8140	0.0008	FLEQQNKLLETK
0.1	1489.8001	0.0147	YEKPQELSGRKR

Mascot: http://www.matrixscience.com/

Mascot Search Results

Peptide View

MS/MS Fragmentation of **FLEQQNKLLETK**
Found in **ch12u_Q9NSB2|KRT84_HUMAN** in **uni_human**, Keratin, type II cuticular Hb4 OS=Homo sapiens GN=KRT84 PE=2 SV=2

Match to Query 17363: 1489.810768 from(745.912660,2+) intensity(1739470.6250) rtinseconds(1482) scans(7866) index(21167)
Title: 160219_Sunil_SDSI_A_Spectrum074471_scans__7866_RTINSECONDS=1482
Data file L:\\QE_2016\\160219_Sunil_KAP_LKC\\TMgf\\T\\T160219_Sunil_SDSI_A.mgf

Click mouse within plot area to zoom in by factor of two about that point
Or, Plot from 100 to 1400 Da Full range
Label all possible matches ○ Label matches used for scoring ◉
Show Y-axis ☐

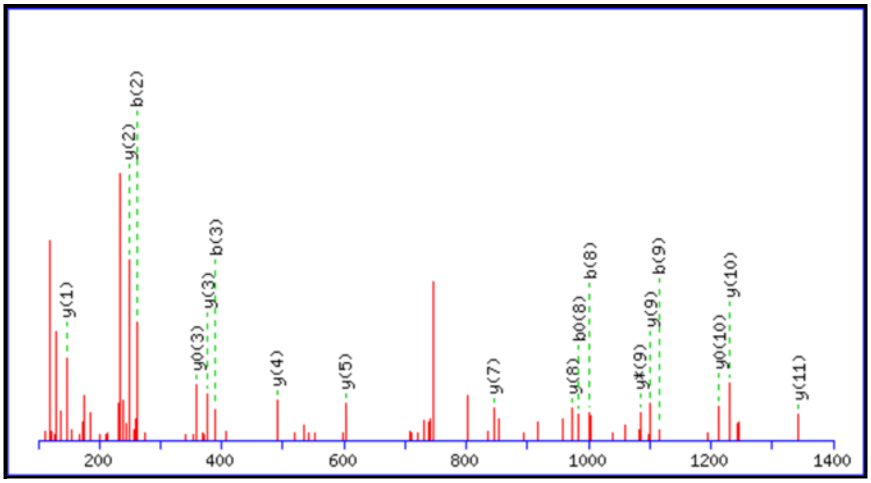

Monoisotopic mass of neutral peptide Mr(calc): 1489.8140
Fixed modifications: Carbamidomethyl (C) (apply to specified residues or termini only)
Ions Score: 72 **Expect:** 7.7e-006
Matches : 18/120 fragment ions using 33 most intense peaks (help)

#	b	b^{++}	b*	b^{*++}	b^0	b^{0++}	Seq.	y	y^{++}	y*	y^{*++}	y^0	y^{0++}	#
1	148.0757	74.5415					F							12
2	**261.1598**	131.0835					L	***1343.7529***	672.3801	1326.7264	663.8668	1325.7423	663.3748	11
3	**390.2023**	195.6048			372.1918	186.5995	E	***1230.6688***	615.8381	1213.6423	607.3248	1212.6583	606.8328	10
4	518.2609	259.6341	501.2344	251.1208	500.2504	250.6288	Q	***1101.6263***	551.3168	1084.5997	542.8035	1083.6157	542.3115	9
5	646.3195	323.6634	629.2930	315.1501	628.3089	314.6581	Q	***973.5677***	487.2875	956.5411	478.7742	955.5571	478.2822	8
6	760.3624	380.6849	743.3359	372.1716	742.3519	371.6796	N	***845.5091***	423.2582	828.4825	414.7449	827.4985	414.2529	7
7	888.4574	444.7323	871.4308	436.2191	870.4468	435.7271	K	731.4662	366.2367	714.4396	357.7234	713.4556	357.2314	6
8	**1001.5415**	501.2744	984.5149	492.7611	983.5309	492.2691	L	***603.3712***	302.1892	586.3447	293.6760	585.3606	293.1840	5
9	**1114.6255**	557.8164	1097.5990	549.3031	1096.6150	548.8111	L	***490.2871***	245.6472	473.2606	237.1339	472.2766	236.6419	4
10	1243.6681	622.3377	1226.6416	613.8244	1225.6575	613.3324	E	***377.2031***	189.1052	360.1765	180.5919	359.1925	180.0999	3
11	1344.7158	672.8615	1327.6892	664.3483	1326.7052	663.8563	T	***248.1605***	124.5839	231.1339	116.0706	230.1499	115.5786	2
12							K	***147.1128***	74.0600	130.0863	65.5468			1

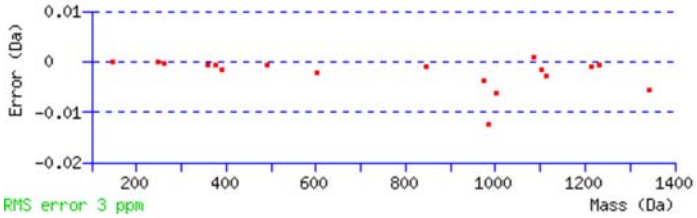

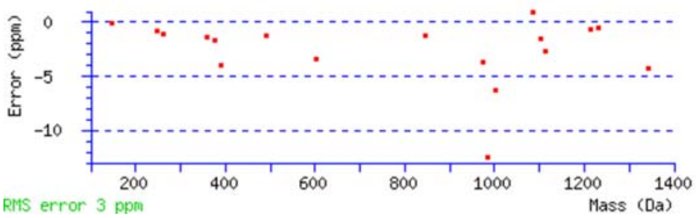

NCBI **BLAST** search of FLEQQNKLLETK
(Parameters: blastp, nr protein database, expect=20000, no filter, PAM30)
Other BLAST web gateways

All matches to this query

Score	Mr(calc)	Delta	Sequence
71.8	1489.8140	-0.0032	FLEQQNKLLETK

Mascot: http://www.matrixscience.com/

Mascot Search Results

Peptide View

MS/MS Fragmentation of **LGLDIEIATYR**
Found in **ch12u_Q9NSB2|KRT84_HUMAN** in **uni_human**, Keratin, type II cuticular Hb4 OS=Homo sapiens GN=KRT84 PE=2 SV=2

Match to Query 10884: 1262.688568 from(632.351560,2+) intensity(136234.8125) rtinseconds(2605) scans(14407) index(41903)
Title: 160219_Sunil_SDSII_A_Spectrum095867_scans__14407_RTINSECONDS=2605
Data file L:\\QE_2016\\160219_Sunil_KAP_LKC\\TMgf\\T\\T160219_Sunil_SDSII_A.mgf

Click mouse within plot area to zoom in by factor of two about that point
Or, Plot from 0 to 1300 Da Full range
Label all possible matches ○ Label matches used for scoring ◉
Show Y-axis ☐

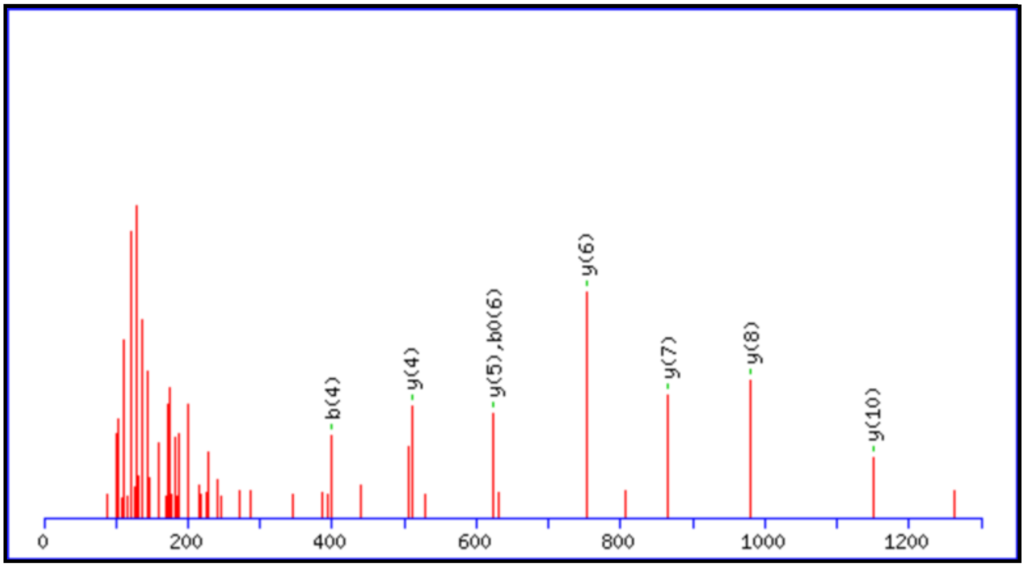

Monoisotopic mass of neutral peptide Mr(calc): 1262.6870
Fixed modifications: Carbamidomethyl (C) (apply to specified residues or termini only)
Ions Score: 42 **Expect:** 0.0075
Matches : 8/90 fragment ions using 11 most intense peaks (help)

#	b	b^{++}	b^0	b^{0++}	Seq.	y	y^{++}	y*	y^{*++}	y^0	y^{0++}	#
1	114.0913	57.5493			L							11
2	171.1128	86.0600			G	*1150.6103*	575.8088	1133.5837	567.2955	1132.5997	566.8035	10
3	284.1969	142.6021			L	1093.5888	547.2980	1076.5623	538.7848	1075.5782	538.2928	9
4	**399.2238**	200.1155	381.2132	191.1103	D	*980.5047*	490.7560	963.4782	482.2427	962.4942	481.7507	8
5	512.3079	256.6576	494.2973	247.6523	I	*865.4778*	433.2425	848.4512	424.7293	847.4672	424.2373	7
6	641.3505	321.1789	623.3399	312.1736	E	*752.3937*	376.7005	735.3672	368.1872	734.3832	367.6952	6
7	754.4345	377.7209	736.4240	368.7156	I	*623.3511*	312.1792	606.3246	303.6659	605.3406	303.1739	5
8	825.4716	413.2395	807.4611	404.2342	A	*510.2671*	255.6372	493.2405	247.1239	492.2565	246.6319	4
9	926.5193	463.7633	908.5088	454.7580	T	439.2300	220.1186	422.2034	211.6053	421.2194	211.1133	3

**10**	1089.5827	545.2950	1071.5721	536.2897	**Y**	338.1823	169.5948	321.1557	161.0815			**2**
**11**					**R**	175.1190	88.0631	158.0924	79.5498			**1**

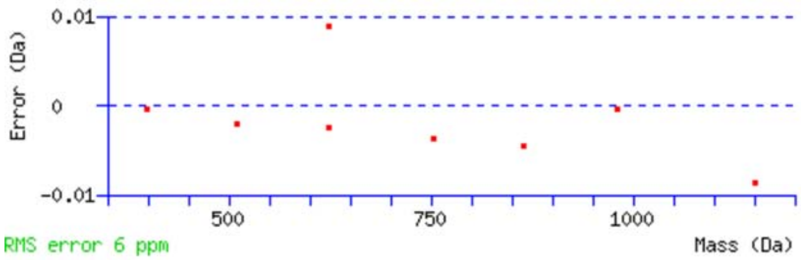

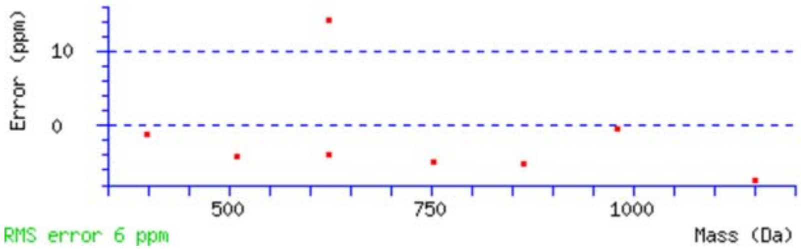

NCBI **BLAST** search of LGLDIEIATYR
(Parameters: blastp, nr protein database, expect=20000, no filter, PAM30)
Other BLAST web gateways

All matches to this query

Score	Mr(calc)	Delta	Sequence
41.5	1262.6870	0.0015	LGLDIEIATYR
17.9	1262.6870	0.0015	IAVDELFTSLR
13.7	1262.6870	0.0015	LALDVEIATYR

Mascot: http://www.matrixscience.com/

Mascot Search Results

Peptide View

MS/MS Fragmentation of **LGLDIEIATYRR**
Found in **ch12u_Q9NSB2|KRT84_HUMAN** in **uni_human**, Keratin, type II cuticular Hb4 OS=Homo sapiens GN=KRT84 PE=2 SV=2

Match to Query 15698: 1418.788002 from(473.936610,3+) intensity(938331.3125) rtinseconds(2496) scans(13414) index(10593)
Title: 160219_Sunil_SDSII_A_Spectrum061922_scans__13414_RTINSECONDS=2496
Data file L:\\QE_2016\\160219_Sunil_KAP_LKC\\TMgf\\T\\T160219_Sunil_SDSII_A.mgf

Click mouse within plot area to zoom in by factor of two about that point
Or, Plot from 100 to 1500 Da Full range
Label all possible matches ○ Label matches used for scoring ◉
Show Y-axis ☐

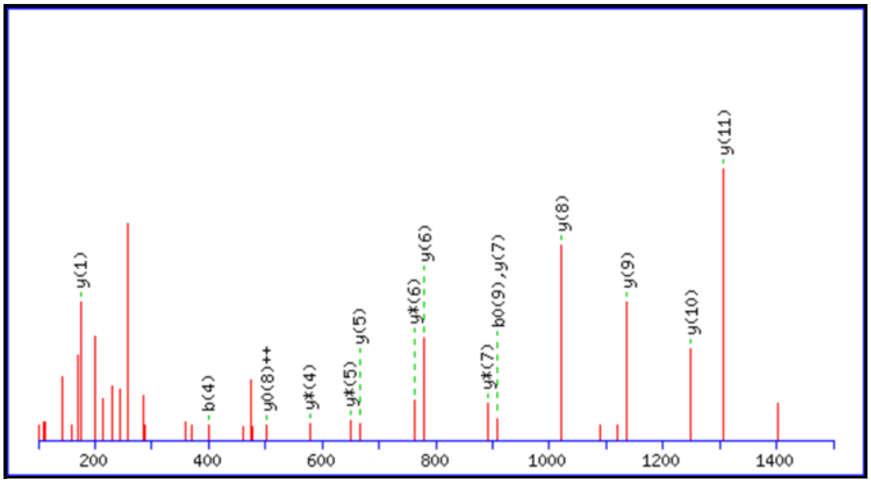

Monoisotopic mass of neutral peptide Mr(calc): 1418.7881
Fixed modifications: Carbamidomethyl (C) (apply to specified residues or termini only)
Ions Score: 58 **Expect:** 0.00016
Matches : 15/100 fragment ions using 24 most intense peaks (help)

#	b	b^{++}	b^{*}	b^{*++}	b^{0}	b^{0++}	Seq.	y	y^{++}	y^{*}	y^{*++}	y^{0}	y^{0++}	#
1	114.0913	57.5493					L							12
2	171.1128	86.0600					G	***1306.7114***	653.8593	1289.6848	645.3461	1288.7008	644.8540	11
3	284.1969	142.6021					L	***1249.6899***	625.3486	1232.6634	616.8353	1231.6793	616.3433	10
4	**399.2238**	200.1155			381.2132	191.1103	D	***1136.6058***	568.8066	1119.5793	560.2933	1118.5953	559.8013	9
5	512.3079	256.6576			494.2973	247.6523	I	***1021.5789***	511.2931	1004.5524	502.7798	1003.5683	502.2878	8
6	641.3505	321.1789			623.3399	312.1736	E	***908.4948***	454.7511	891.4683	446.2378	890.4843	445.7458	7
7	754.4345	377.7209			736.4240	368.7156	I	***779.4522***	390.2298	762.4257	381.7165	761.4417	381.2245	6
8	825.4716	413.2395			807.4611	404.2342	A	***666.3682***	333.6877	649.3416	325.1745	648.3576	324.6824	5
9	926.5193	463.7633			908.5088	454.7580	T	595.3311	298.1692	578.3045	289.6559	577.3205	289.1639	4
10	1089.5827	545.2950			1071.5721	536.2897	Y	494.2834	247.6453	477.2568	239.1321			3
11	1245.6838	623.3455	1228.6572	614.8322	1227.6732	614.3402	R	331.2201	166.1137	314.1935	157.6004			2
12							R	***175.1190***	88.0631	158.0924	79.5498			1

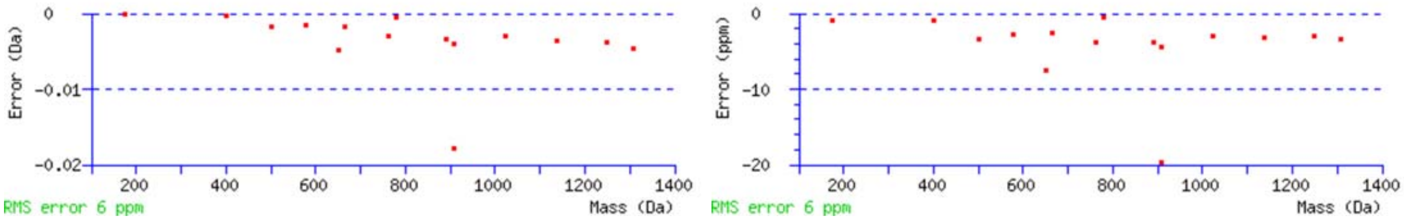

NCBI **BLAST** search of LGLDIEIATYRR
(Parameters: blastp, nr protein database, expect=20000, no filter, PAM30)
Other BLAST web gateways

All matches to this query

Score	Mr(calc)	Delta	Sequence
57.8	1418.7881	-0.0001	LGLDIEIATYRR
23.1	1418.7881	-0.0001	LGLLDRIVNYSR
12.3	1418.7769	0.0111	LLGNTFVALSDLR
1.9	1418.7881	-0.0001	LQLRSLQYLER
1.8	1418.7742	0.0138	LGEARHPQVSLGR
1.8	1418.7994	-0.0114	LGPESVPPPKRSR
0.4	1418.7881	-0.0001	IYDKERLNVLR

Mascot: http://www.matrixscience.com/

Mascot Search Results

Peptide View

MS/MS Fragmentation of **LGLDIEIATYRR**
Found in **ch12u_Q9NSB2|KRT84_HUMAN** in **uni_human**, Keratin, type II cuticular Hb4 OS=Homo sapiens GN=KRT84 PE=2 SV=2

Match to Query 15702: 1418.788122 from(473.936650,3+) intensity(1095911.5000) rtinseconds(2237) scans(12291) index(40015)
Title: 160219_Sunil_SDSII_A_Spectrum093976_scans__12291_RTINSECONDS=2237
Data file L:\\QE_2016\\160219_Sunil_KAP_LKC\\TMgf\\T\\T160219_Sunil_SDSII_A.mgf

Click mouse within plot area to zoom in by factor of two about that point
Or, Plot from 100 to 1500 Da Full range
Label all possible matches ○ Label matches used for scoring ◉
Show Y-axis ☐

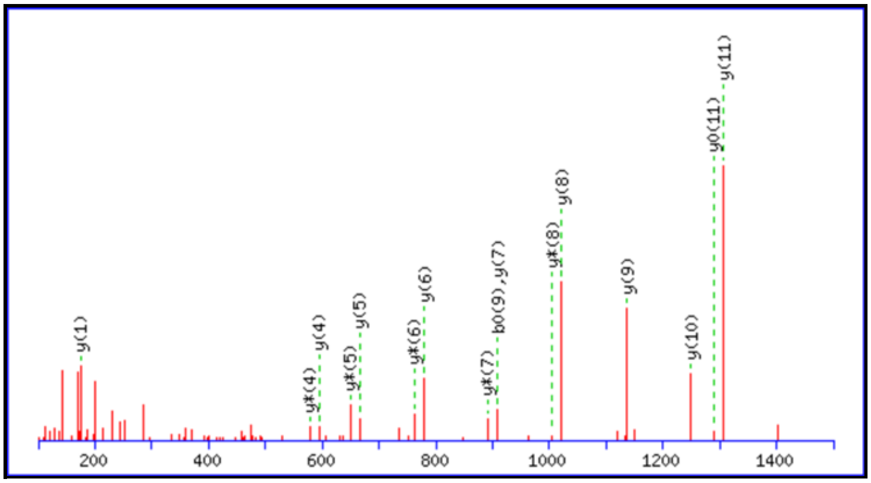

Monoisotopic mass of neutral peptide Mr(calc): 1418.7881
Fixed modifications: Carbamidomethyl (C) (apply to specified residues or termini only)
Ions Score: 68 **Expect:** 1.4e-005
Matches : 16/100 fragment ions using 26 most intense peaks (help)

#	b	b^{++}	b*	b^{*++}	b^0	b^{0++}	Seq.	y	y^{++}	y*	y^{*++}	y^0	y^{0++}	#
1	114.0913	57.5493					L							12
2	171.1128	86.0600					G	***1306.7114***	653.8593	1289.6848	645.3461	1288.7008	644.8540	11
3	284.1969	142.6021					L	***1249.6899***	625.3486	1232.6634	616.8353	1231.6793	616.3433	10
4	399.2238	200.1155			381.2132	191.1103	D	***1136.6058***	568.8066	1119.5793	560.2933	1118.5953	559.8013	9
5	512.3079	256.6576			494.2973	247.6523	I	***1021.5789***	511.2931	1004.5524	502.7798	1003.5683	502.2878	8
6	641.3505	321.1789			623.3399	312.1736	E	***908.4948***	454.7511	891.4683	446.2378	890.4843	445.7458	7
7	754.4345	377.7209			736.4240	368.7156	I	***779.4522***	390.2298	762.4257	381.7165	761.4417	381.2245	6
8	825.4716	413.2395			807.4611	404.2342	A	***666.3682***	333.6877	649.3416	325.1745	648.3576	324.6824	5
9	926.5193	463.7633			908.5088	454.7580	T	***595.3311***	298.1692	578.3045	289.6559	577.3205	289.1639	4
10	1089.5827	545.2950			1071.5721	536.2897	Y	494.2834	247.6453	477.2568	239.1321			3
11	1245.6838	623.3455	1228.6572	614.8322	1227.6732	614.3402	R	331.2201	166.1137	314.1935	157.6004			2
12							R	***175.1190***	88.0631	158.0924	79.5498			1

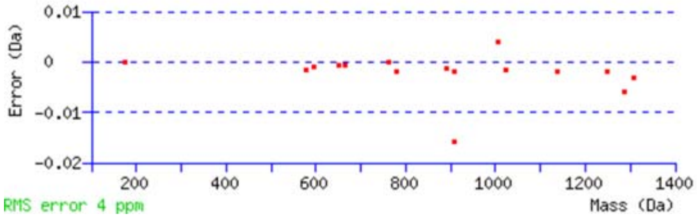

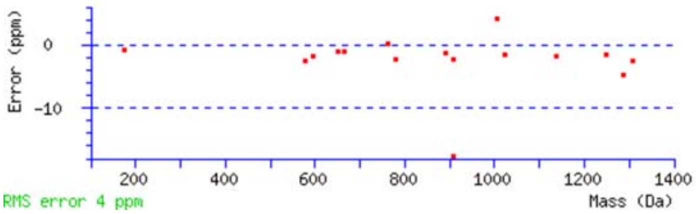

NCBI **BLAST** search of LGLDIEIATYRR
(Parameters: blastp, nr protein database, expect=20000, no filter, PAM30)
Other BLAST web gateways

All matches to this query

Score	Mr(calc)	Delta	Sequence
68.3	1418.7881	-0.0000	LGLDIEIATYRR
23.8	1418.7881	-0.0000	LGLLDRIVNYSR
13.0	1418.7769	0.0112	LLGNTFVALSDLR
7.2	1418.7881	0.0000	LQLRSLQYLER
5.0	1418.7769	0.0112	GLVLQLIQSYQR
3.4	1418.7769	0.0112	GLVLQLIQSYQR
2.3	1418.7742	0.0139	LGEARHPQVSLGR
2.3	1418.7994	-0.0113	LGPESVPPPKRSR

Mascot: http://www.matrixscience.com/

Mascot Search Results

Peptide View

MS/MS Fragmentation of **LGLDIEIATYRR**
Found in **ch12u_Q9NSB2|KRT84_HUMAN** in **uni_human**, Keratin, type II cuticular Hb4 OS=Homo sapiens GN=KRT84 PE=2 SV=2

Match to Query 15705: 1418.788302 from(473.936710,3+) intensity(752125.1875) rtinseconds(2338) scans(12787) index(25287)
Title: 160219_Sunil_SDSII_A_Spectrum077849_scans__12787_RTINSECONDS=2338
Data file L:\\QE_2016\\160219_Sunil_KAP_LKC\\TMgf\\T\\T160219_Sunil_SDSII_A.mgf

Click mouse within plot area to zoom in by factor of two about that point
Or, Plot from 100 to 1500 Da Full range
Label all possible matches ○ Label matches used for scoring ◉
Show Y-axis ☐

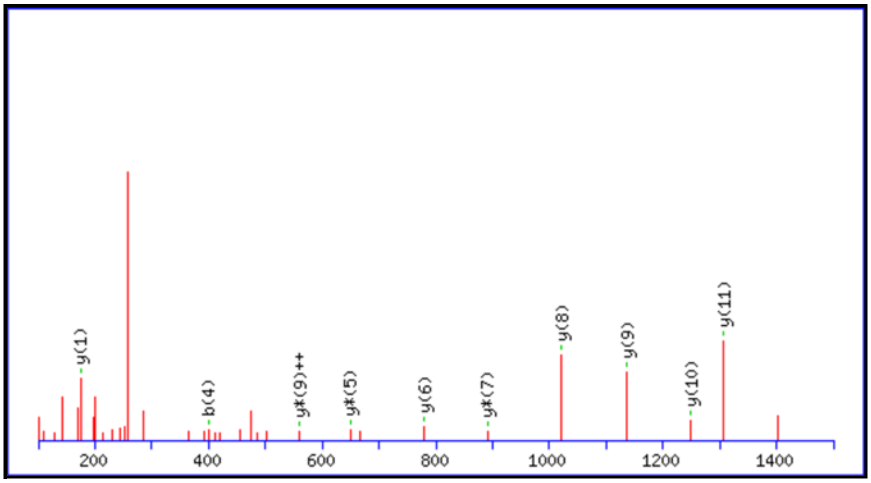

Monoisotopic mass of neutral peptide Mr(calc): 1418.7881
Fixed modifications: Carbamidomethyl (C) (apply to specified residues or termini only)
Ions Score: 46 **Expect:** 0.0024
Matches : 10/100 fragment ions using 13 most intense peaks (help)

#	b	b^{++}	b*	b^{*++}	b^0	b^{0++}	Seq.	y	y^{++}	y*	y^{*++}	y^0	y^{0++}	#
1	114.0913	57.5493					L							12
2	171.1128	86.0600					G	***1306.7114***	653.8593	1289.6848	645.3461	1288.7008	644.8540	11
3	284.1969	142.6021					L	***1249.6899***	625.3486	1232.6634	616.8353	1231.6793	616.3433	10
4	**399.2238**	200.1155			381.2132	191.1103	D	***1136.6058***	568.8066	1119.5793	560.2933	1118.5953	559.8013	9
5	512.3079	256.6576			494.2973	247.6523	I	***1021.5789***	511.2931	1004.5524	502.7798	1003.5683	502.2878	8
6	641.3505	321.1789			623.3399	312.1736	E	908.4948	454.7511	891.4683	446.2378	890.4843	445.7458	7
7	754.4345	377.7209			736.4240	368.7156	I	***779.4522***	390.2298	762.4257	381.7165	761.4417	381.2245	6
8	825.4716	413.2395			807.4611	404.2342	A	666.3682	333.6877	649.3416	325.1745	648.3576	324.6824	5
9	926.5193	463.7633			908.5088	454.7580	T	595.3311	298.1692	578.3045	289.6559	577.3205	289.1639	4
10	1089.5827	545.2950			1071.5721	536.2897	Y	494.2834	247.6453	477.2568	239.1321			3
11	1245.6838	623.3455	1228.6572	614.8322	1227.6732	614.3402	R	331.2201	166.1137	314.1935	157.6004			2
12							R	***175.1190***	88.0631	158.0924	79.5498			1

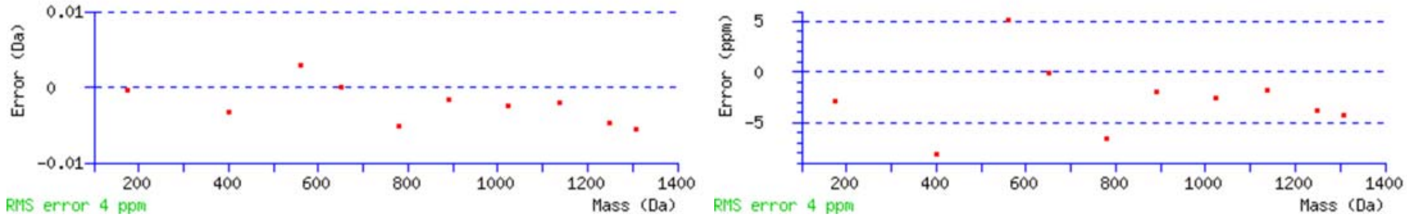

NCBI **BLAST** search of LGLDIEIATYRR
(Parameters: blastp, nr protein database, expect=20000, no filter, PAM30)
Other BLAST web gateways

All matches to this query

Score	Mr(calc)	Delta	Sequence
45.9	1418.7881	0.0002	LGLDIEIATYRR
16.5	1418.7769	0.0114	LLGNTFVALSDLR
15.6	1418.7881	0.0002	LGLLDRIVNYSR
0.8	1418.7742	0.0141	LGEARHPQVSLGR
0.8	1418.7994	-0.0111	LGPESVPPPKRSR
0.5	1418.7881	0.0002	IKQFTLEEKQR

Mascot: http://www.matrixscience.com/

Mascot Search Results

Peptide View

MS/MS Fragmentation of **LGLDIEIATYRR**
Found in **ch12u_Q9NSB2|KRT84_HUMAN** in **uni_human**, Keratin, type II cuticular Hb4 OS=Homo sapiens GN=KRT84 PE=2 SV=2

Match to Query 15707: 1418.788392 from(473.936740,3+) intensity(777548.2500) rtinseconds(2591) scans(13963) index(11041)
Title: 160219_Sunil_SDSII_A_Spectrum062370_scans__13963_RTINSECONDS=2591
Data file L:\\QE_2016\\160219_Sunil_KAP_LKC\\TMgf\\T\\T160219_Sunil_SDSII_A.mgf

Click mouse within plot area to zoom in by factor of two about that point
Or, Plot from 100 to 1400 Da Full range
Label all possible matches ○ Label matches used for scoring ◉
Show Y-axis ☐

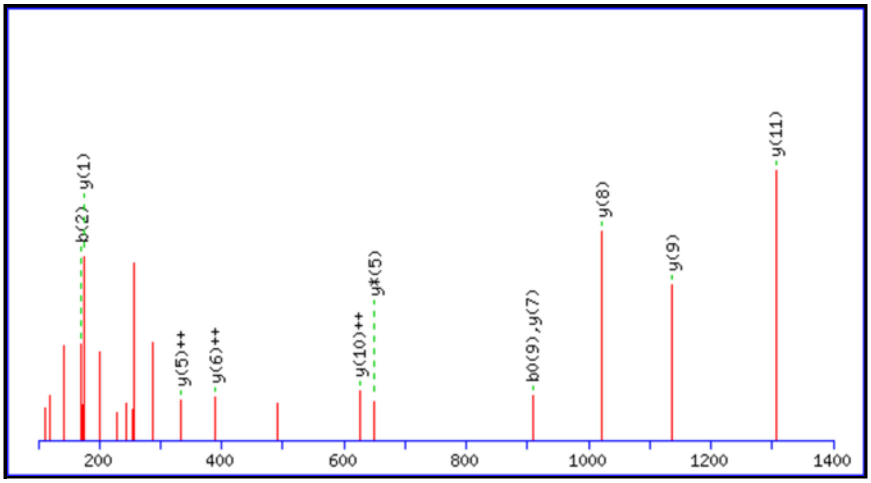

Monoisotopic mass of neutral peptide Mr(calc): 1418.7881
Fixed modifications: Carbamidomethyl (C) (apply to specified residues or termini only)
Ions Score: 45 **Expect:** 0.0032
Matches : 11/100 fragment ions using 13 most intense peaks (help)

#	b	b^{++}	b*	b^{*++}	b^0	b^{0++}	Seq.	y	y^{++}	y*	y^{*++}	y^0	y^{0++}	#
1	114.0913	57.5493					L							12
2	**171.1128**	86.0600					G	***1306.7114***	653.8593	1289.6848	645.3461	1288.7008	644.8540	11
3	284.1969	142.6021					L	1249.6899	625.3486	1232.6634	616.8353	1231.6793	616.3433	10
4	399.2238	200.1155			381.2132	191.1103	D	***1136.6058***	568.8066	1119.5793	560.2933	1118.5953	559.8013	9
5	512.3079	256.6576			494.2973	247.6523	I	***1021.5789***	511.2931	1004.5524	502.7798	1003.5683	502.2878	8
6	641.3505	321.1789			623.3399	312.1736	E	***908.4948***	454.7511	891.4683	446.2378	890.4843	445.7458	7
7	754.4345	377.7209			736.4240	368.7156	I	779.4522	390.2298	762.4257	381.7165	761.4417	381.2245	6
8	825.4716	413.2395			807.4611	404.2342	A	666.3682	333.6877	649.3416	325.1745	648.3576	324.6824	5
9	926.5193	463.7633			908.5088	454.7580	T	595.3311	298.1692	578.3045	289.6559	577.3205	289.1639	4
10	1089.5827	545.2950			1071.5721	536.2897	Y	494.2834	247.6453	477.2568	239.1321			3
11	1245.6838	623.3455	1228.6572	614.8322	1227.6732	614.3402	R	331.2201	166.1137	314.1935	157.6004			2
12							R	***175.1190***	88.0631	158.0924	79.5498			1

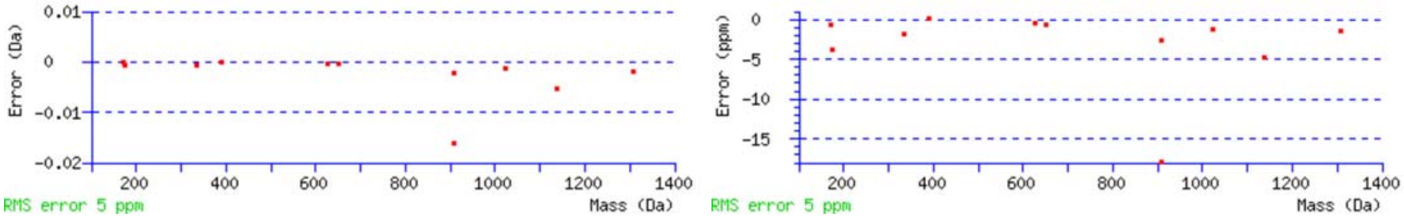

NCBI **BLAST** search of LGLDIEIATYRR
(Parameters: blastp, nr protein database, expect=20000, no filter, PAM30)
Other BLAST web gateways

All matches to this query

Score	Mr(calc)	Delta	Sequence
44.6	1418.7881	0.0003	LGLDIEIATYRR
21.6	1418.7881	0.0003	LGLLDRIVNYSR
19.6	1418.7769	0.0115	LLGNTFVALSDLR
8.5	1418.7881	0.0003	LQLRSLQYLER
3.5	1418.7742	0.0142	LGEARHPQVSLGR
0.8	1418.7994	-0.0110	LGPESVPPPKRSR

Mascot: http://www.matrixscience.com/

Mascot Search Results

Peptide View

MS/MS Fragmentation of **LGLDIEIATYRR**
Found in **ch12u_Q9NSB2|KRT84_HUMAN** in **uni_human**, Keratin, type II cuticular Hb4 OS=Homo sapiens GN=KRT84 PE=2 SV=2

Match to Query 15712: 1418.788842 from(473.936890,3+) intensity(2373411.5000) rtinseconds(2559) scans(13779) index(10891)
Title: 160219_Sunil_SDSII_A_Spectrum062220_scans__13779_RTINSECONDS=2559
Data file L:\\QE_2016\\160219_Sunil_KAP_LKC\\TMgf\\T\\T160219_Sunil_SDSII_A.mgf

Click mouse within plot area to zoom in by factor of two about that point
Or, Plot from 100 to 1400 Da Full range
Label all possible matches ○ Label matches used for scoring ◉
Show Y-axis ☐

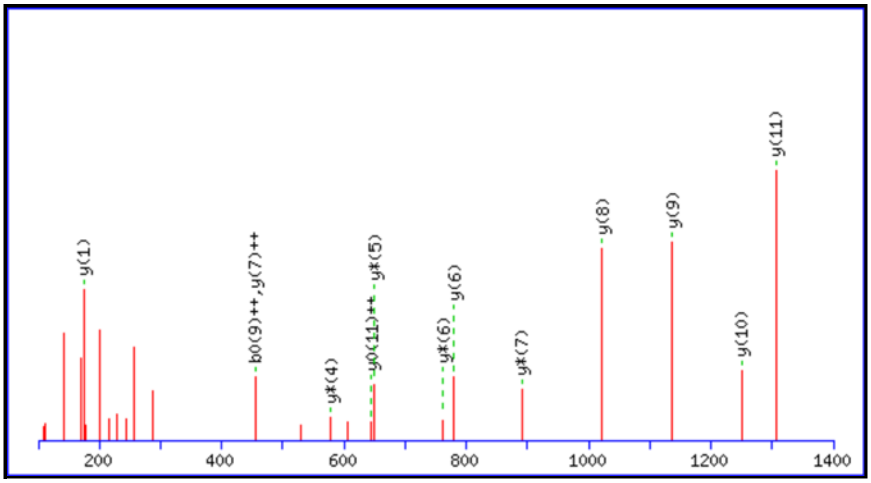

Monoisotopic mass of neutral peptide Mr(calc): 1418.7881
Fixed modifications: Carbamidomethyl (C) (apply to specified residues or termini only)
Ions Score: 46 **Expect:** 0.0021
Matches : 13/100 fragment ions using 16 most intense peaks (help)

#	b	b^{++}	b*	b^{*++}	b^0	b^{0++}	Seq.	y	y^{++}	y*	y^{*++}	y^0	y^{0++}	#
1	114.0913	57.5493					L							12
2	171.1128	86.0600					G	***1306.7114***	653.8593	1289.6848	645.3461	1288.7008	644.8540	11
3	284.1969	142.6021					L	***1249.6899***	625.3486	1232.6634	616.8353	1231.6793	616.3433	10
4	399.2238	200.1155			381.2132	191.1103	D	***1136.6058***	568.8066	1119.5793	560.2933	1118.5953	559.8013	9
5	512.3079	256.6576			494.2973	247.6523	I	***1021.5789***	511.2931	1004.5524	502.7798	1003.5683	502.2878	8
6	641.3505	321.1789			623.3399	312.1736	E	908.4948	454.7511	891.4683	446.2378	890.4843	445.7458	7
7	754.4345	377.7209			736.4240	368.7156	I	***779.4522***	390.2298	762.4257	381.7165	761.4417	381.2245	6
8	825.4716	413.2395			807.4611	404.2342	A	666.3682	333.6877	649.3416	325.1745	648.3576	324.6824	5
9	926.5193	463.7633			908.5088	454.7580	T	595.3311	298.1692	578.3045	289.6559	577.3205	289.1639	4
10	1089.5827	545.2950			1071.5721	536.2897	Y	494.2834	247.6453	477.2568	239.1321			3
11	1245.6838	623.3455	1228.6572	614.8322	1227.6732	614.3402	R	331.2201	166.1137	314.1935	157.6004			2
12							R	***175.1190***	88.0631	158.0924	79.5498			1

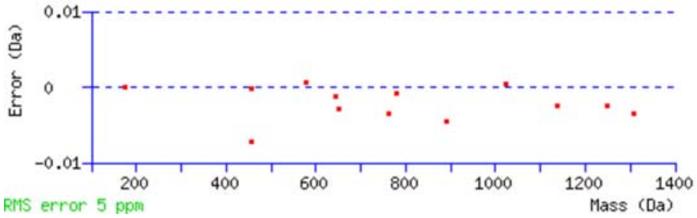

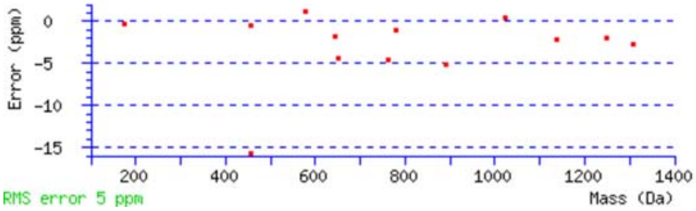

NCBI **BLAST** search of LGLDIEIATYRR
(Parameters: blastp, nr protein database, expect=20000, no filter, PAM30)
Other BLAST web gateways

All matches to this query

Score	Mr(calc)	Delta	Sequence
46.3	1418.7881	0.0007	LGLDIEIATYRR
18.1	1418.7769	0.0119	LLGNTFVALSDLR
16.4	1418.7881	0.0007	LGLLDRIVNYSR
3.4	1418.7994	-0.0105	LGPESVPPPKRSR
2.5	1418.7769	0.0119	LIQFLQASITER
2.5	1418.7769	0.0119	LIQFLQASITER
1.3	1418.7881	0.0007	LQLRSLQYLER
0.3	1418.7881	0.0007	IYDKERLNVLR
0.3	1418.7881	0.0007	LQLRSLQYLER

Mascot: http://www.matrixscience.com/

Mascot Search Results

Peptide View

MS/MS Fragmentation of **FLEQQNKLLETK**
Found in **ch12u_Q9NSB2|KRT84_HUMAN** in **uni_human**, Keratin, type II cuticular Hb4 OS=Homo sapiens GN=KRT84 PE=2 SV=2

Match to Query 18173: 1489.810408 from(745.912480,2+) intensity(233483.4063) rtinseconds(1395) scans(7426) index(35719)
Title: 160219_Sunil_SDSII_A_Spectrum089676_scans__7426_RTINSECONDS=1395
Data file L:\\QE_2016\\160219_Sunil_KAP_LKC\\TMgf\\T\\T160219_Sunil_SDSII_A.mgf

Click mouse within plot area to zoom in by factor of two about that point
Or, Plot from 100 to 1300 Da Full range
Label all possible matches ○ Label matches used for scoring ◉
Show Y-axis ☐

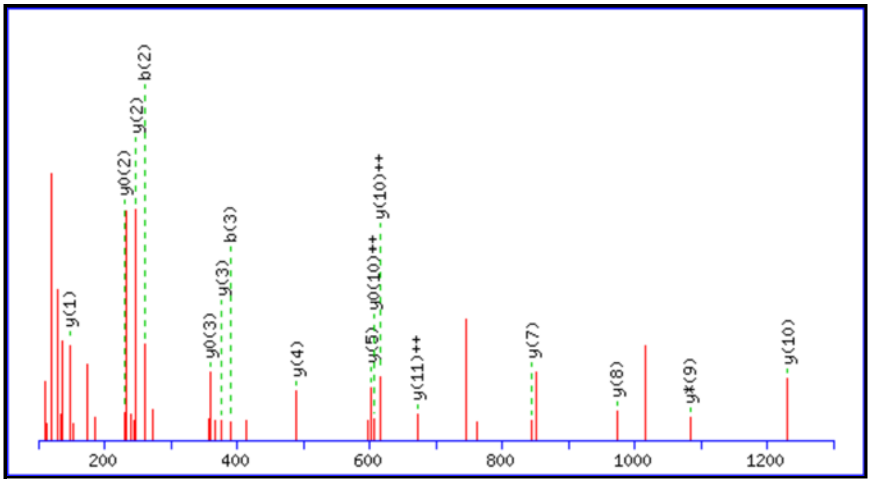

Monoisotopic mass of neutral peptide Mr(calc): 1489.8140
Fixed modifications: Carbamidomethyl (C) (apply to specified residues or termini only)
Ions Score: 44 **Expect:** 0.0046
Matches : 16/120 fragment ions using 32 most intense peaks (help)

#	b	b^{++}	b^{*}	b^{*++}	b^{0}	b^{0++}	Seq.	y	y^{++}	y^{*}	y^{*++}	y^{0}	y^{0++}	#
1	148.0757	74.5415					F							12
2	**261.1598**	131.0835					L	1343.7529	672.3801	1326.7264	663.8668	1325.7423	663.3748	11
3	**390.2023**	195.6048			372.1918	186.5995	E	***1230.6688***	615.8381	1213.6423	607.3248	1212.6583	606.8328	10
4	518.2609	259.6341	501.2344	251.1208	500.2504	250.6288	Q	1101.6263	551.3168	1084.5997	542.8035	1083.6157	542.3115	9
5	646.3195	323.6634	629.2930	315.1501	628.3089	314.6581	Q	***973.5677***	487.2875	956.5411	478.7742	955.5571	478.2822	8
6	760.3624	380.6849	743.3359	372.1716	742.3519	371.6796	N	***845.5091***	423.2582	828.4825	414.7449	827.4985	414.2529	7
7	888.4574	444.7323	871.4308	436.2191	870.4468	435.7271	K	731.4662	366.2367	714.4396	357.7234	713.4556	357.2314	6
8	1001.5415	501.2744	984.5149	492.7611	983.5309	492.2691	L	***603.3712***	302.1892	586.3447	293.6760	585.3606	293.1840	5
9	1114.6255	557.8164	1097.5990	549.3031	1096.6150	548.8111	L	***490.2871***	245.6472	473.2606	237.1339	472.2766	236.6419	4
10	1243.6681	622.3377	1226.6416	613.8244	1225.6575	613.3324	E	***377.2031***	189.1052	360.1765	180.5919	359.1925	180.0999	3
11	1344.7158	672.8615	1327.6892	664.3483	1326.7052	663.8563	T	***248.1605***	124.5839	231.1339	116.0706	230.1499	115.5786	2
12							K	***147.1128***	74.0600	130.0863	65.5468			1

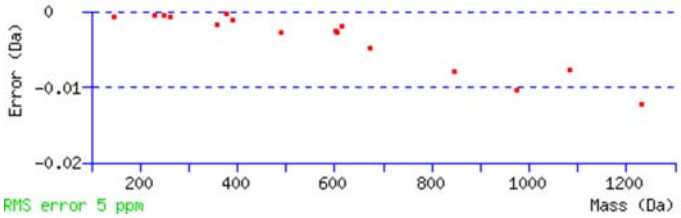

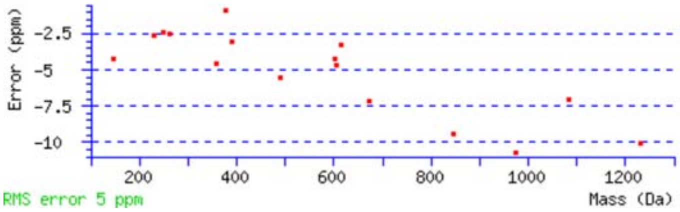

NCBI **BLAST** search of FLEQQNKLLETK
(Parameters: blastp, nr protein database, expect=20000, no filter, PAM30)
Other BLAST web gateways

All matches to this query

Score	Mr(calc)	Delta	Sequence
44.0	1489.8140	-0.0036	FLEQQNKLLETK

Mascot: http://www.matrixscience.com/

Mascot Search Results

Peptide View

MS/MS Fragmentation of **FLEQQNKLLETK**
Found in **ch12u_Q9NSB2|KRT84_HUMAN** in **uni_human**, Keratin, type II cuticular Hb4 OS=Homo sapiens GN=KRT84 PE=2 SV=2

Match to Query 18180: 1489.812968 from(745.913760,2+) intensity(908432.9375) rtinseconds(2254) scans(12014) index(9407)
Title: 160219_Sunil_SDSII_A_Spectrum060731_scans__12014_RTINSECONDS=2254
Data file L:\\QE_2016\\160219_Sunil_KAP_LKC\\TMgf\\T\\T160219_Sunil_SDSII_A.mgf

Click mouse within plot area to zoom in by factor of two about that point
Or, Plot from 100 to 1500 Da Full range
Label all possible matches ○ Label matches used for scoring ◉
Show Y-axis □

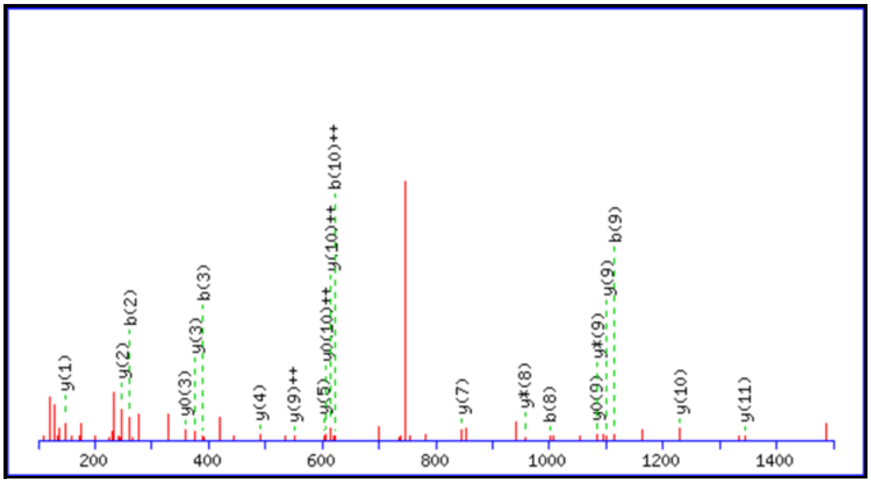

Monoisotopic mass of neutral peptide Mr(calc): 1489.8140
Fixed modifications: Carbamidomethyl (C) (apply to specified residues or termini only)
Ions Score: 48 **Expect:** 0.0017
Matches : 21/120 fragment ions using 43 most intense peaks (help)

#	b	b^{++}	b*	b^{*++}	b^{0}	b^{0++}	Seq.	y	y^{++}	y*	y^{*++}	y^{0}	y^{0++}	#
1	148.0757	74.5415					F							12
2	**261.1598**	131.0835					L	***1343.7529***	672.3801	1326.7264	663.8668	1325.7423	663.3748	11
3	**390.2023**	195.6048			372.1918	186.5995	E	***1230.6688***	615.8381	1213.6423	607.3248	1212.6583	606.8328	10
4	518.2609	259.6341	501.2344	251.1208	500.2504	250.6288	Q	***1101.6263***	551.3168	1084.5997	542.8035	1083.6157	542.3115	9
5	646.3195	323.6634	629.2930	315.1501	628.3089	314.6581	Q	973.5677	487.2875	956.5411	478.7742	955.5571	478.2822	8
6	760.3624	380.6849	743.3359	372.1716	742.3519	371.6796	N	***845.5091***	423.2582	828.4825	414.7449	827.4985	414.2529	7
7	888.4574	444.7323	871.4308	436.2191	870.4468	435.7271	K	731.4662	366.2367	714.4396	357.7234	713.4556	357.2314	6
8	**1001.5415**	501.2744	984.5149	492.7611	983.5309	492.2691	L	***603.3712***	302.1892	586.3447	293.6760	585.3606	293.1840	5
9	**1114.6255**	557.8164	1097.5990	549.3031	1096.6150	548.8111	L	***490.2871***	245.6472	473.2606	237.1339	472.2766	236.6419	4
10	1243.6681	**622.3377**	1226.6416	613.8244	1225.6575	613.3324	E	***377.2031***	189.1052	360.1765	180.5919	359.1925	180.0999	3
11	1344.7158	672.8615	1327.6892	664.3483	1326.7052	663.8563	T	***248.1605***	124.5839	231.1339	116.0706	230.1499	115.5786	2
12							K	***147.1128***	74.0600	130.0863	65.5468			1

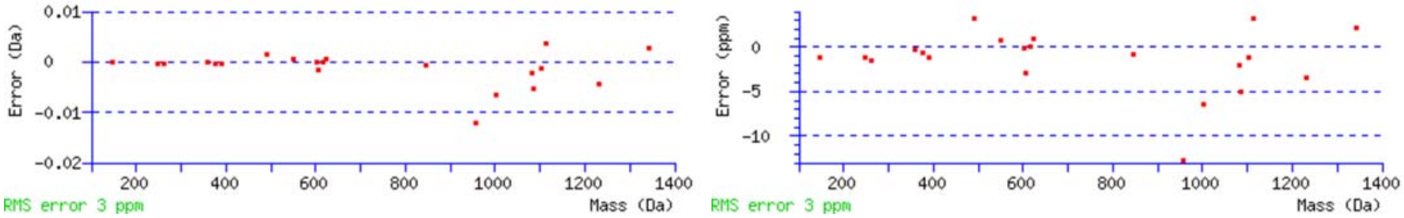

NCBI **BLAST** search of FLEQQNKLLETK
(Parameters: blastp, nr protein database, expect=20000, no filter, PAM30)
Other BLAST web gateways

All matches to this query

Score	Mr(calc)	Delta	Sequence
48.5	1489.8140	-0.0010	FLEQQNKLLETK

Mascot: http://www.matrixscience.com/

Mascot Search Results

Peptide View

MS/MS Fragmentation of **FLEQQNKLLETK**
Found in **ch12u_Q9NSB2|KRT84_HUMAN** in **uni_human**, Keratin, type II cuticular Hb4 OS=Homo sapiens GN=KRT84 PE=2 SV=2

Match to Query 18176: 1489.812612 from(497.611480,3+) intensity(632382.0000) rtinseconds(1595) scans(8490) index(21492)
Title: 160219_Sunil_SDSII_A_Spectrum074053_scans__8490_RTINSECONDS=1595
Data file L:\\QE_2016\\160219_Sunil_KAP_LKC\\TMgf\\T\\T160219_Sunil_SDSII_A.mgf

Click mouse within plot area to zoom in by factor of two about that point
Or, Plot from 100 to 1500 Da Full range
Label all possible matches ○ Label matches used for scoring ◉
Show Y-axis ☐

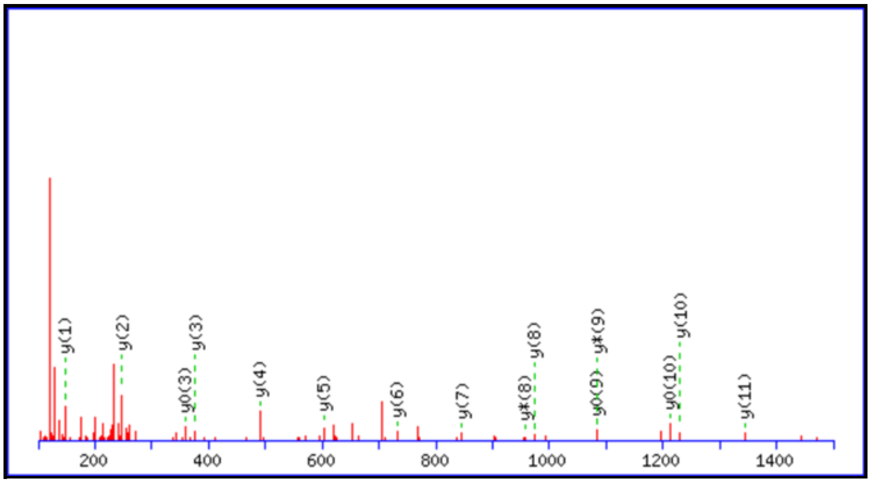

Monoisotopic mass of neutral peptide Mr(calc): 1489.8140
Fixed modifications: Carbamidomethyl (C) (apply to specified residues or termini only)
Ions Score: 56 **Expect:** 0.0003
Matches : 15/120 fragment ions using 35 most intense peaks (help)

#	b	b++	b*	b*++	b0	b0++	Seq.	y	y++	y*	y*++	y0	y0++	#
1	148.0757	74.5415					F							12
2	261.1598	131.0835					L	***1343.7529***	672.3801	1326.7264	663.8668	1325.7423	663.3748	11
3	390.2023	195.6048			372.1918	186.5995	E	***1230.6688***	615.8381	1213.6423	607.3248	1212.6583	606.8328	10
4	518.2609	259.6341	501.2344	251.1208	500.2504	250.6288	Q	1101.6263	551.3168	1084.5997	542.8035	1083.6157	542.3115	9
5	646.3195	323.6634	629.2930	315.1501	628.3089	314.6581	Q	***973.5677***	487.2875	956.5411	478.7742	955.5571	478.2822	8
6	760.3624	380.6849	743.3359	372.1716	742.3519	371.6796	N	***845.5091***	423.2582	828.4825	414.7449	827.4985	414.2529	7
7	888.4574	444.7323	871.4308	436.2191	870.4468	435.7271	K	***731.4662***	366.2367	714.4396	357.7234	713.4556	357.2314	6
8	1001.5415	501.2744	984.5149	492.7611	983.5309	492.2691	L	***603.3712***	302.1892	586.3447	293.6760	585.3606	293.1840	5
9	1114.6255	557.8164	1097.5990	549.3031	1096.6150	548.8111	L	***490.2871***	245.6472	473.2606	237.1339	472.2766	236.6419	4
10	1243.6681	622.3377	1226.6416	613.8244	1225.6575	613.3324	E	***377.2031***	189.1052	360.1765	180.5919	359.1925	180.0999	3
11	1344.7158	672.8615	1327.6892	664.3483	1326.7052	663.8563	T	***248.1605***	124.5839	231.1339	116.0706	230.1499	115.5786	2
12							K	***147.1128***	74.0600	130.0863	65.5468			1

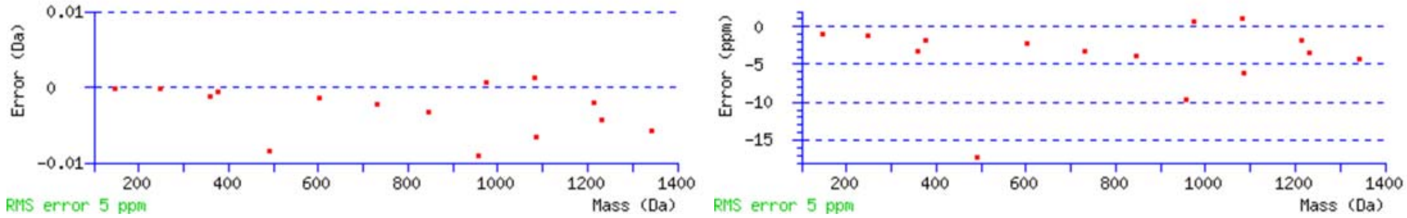

NCBI **BLAST** search of FLEQQNKLLETK
(Parameters: blastp, nr protein database, expect=20000, no filter, PAM30)
Other BLAST web gateways

All matches to this query

Score	Mr(calc)	Delta	Sequence
55.7	1489.8140	-0.0014	FLEQQNKLLETK

Mascot: http://www.matrixscience.com/

Mascot Search Results

Peptide View

MS/MS Fragmentation of **FLEQQNKLLETK**
Found in **ch12u_Q9NSB2|KRT84_HUMAN** in **uni_human**, Keratin, type II cuticular Hb4 OS=Homo sapiens GN=KRT84 PE=2 SV=2

Match to Query 18185: 1489.813632 from(497.611820,3+) intensity(445336.0938) rtinseconds(1392) scans(7410) index(35705)
Title: 160219_Sunil_SDSII_A_Spectrum089662_scans__7410_RTINSECONDS=1392
Data file L:\\QE_2016\\160219_Sunil_KAP_LKC\\TMgf\\T\\T160219_Sunil_SDSII_A.mgf

Click mouse within plot area to zoom in by factor of two about that point
Or, Plot from 100 to 1400 Da Full range
Label all possible matches ○ Label matches used for scoring ◉
Show Y-axis ☐

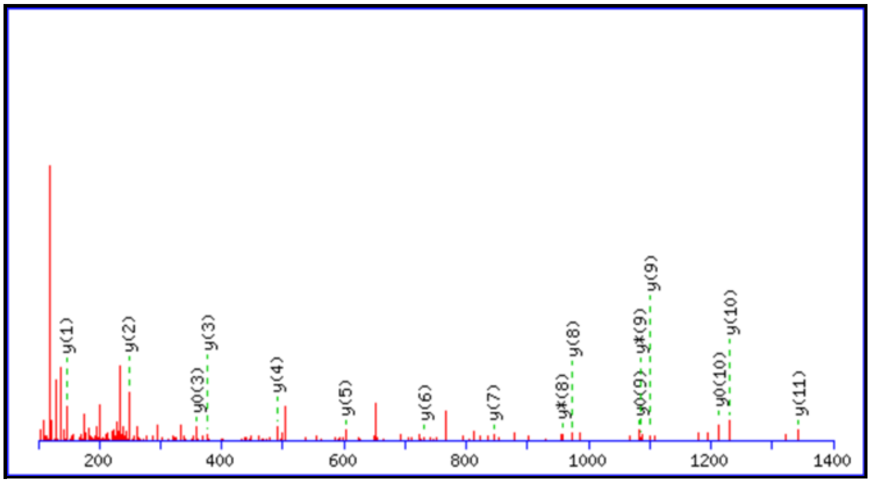

Monoisotopic mass of neutral peptide Mr(calc): 1489.8140
Fixed modifications: Carbamidomethyl (C) (apply to specified residues or termini only)
Ions Score: 43 **Expect:** 0.0053
Matches : 16/120 fragment ions using 58 most intense peaks (help)

#	b	b^{++}	b*	b^{*++}	b^0	b^{0++}	Seq.	y	y^{++}	y*	y^{*++}	y^0	y^{0++}	#
1	148.0757	74.5415					F							12
2	261.1598	131.0835					L	***1343.7529***	672.3801	1326.7264	663.8668	1325.7423	663.3748	11
3	390.2023	195.6048			372.1918	186.5995	E	***1230.6688***	615.8381	1213.6423	607.3248	1212.6583	606.8328	10
4	518.2609	259.6341	501.2344	251.1208	500.2504	250.6288	Q	***1101.6263***	551.3168	1084.5997	542.8035	1083.6157	542.3115	9
5	646.3195	323.6634	629.2930	315.1501	628.3089	314.6581	Q	***973.5677***	487.2875	956.5411	478.7742	955.5571	478.2822	8
6	760.3624	380.6849	743.3359	372.1716	742.3519	371.6796	N	***845.5091***	423.2582	828.4825	414.7449	827.4985	414.2529	7
7	888.4574	444.7323	871.4308	436.2191	870.4468	435.7271	K	***731.4662***	366.2367	714.4396	357.7234	713.4556	357.2314	6
8	1001.5415	501.2744	984.5149	492.7611	983.5309	492.2691	L	***603.3712***	302.1892	586.3447	293.6760	585.3606	293.1840	5
9	1114.6255	557.8164	1097.5990	549.3031	1096.6150	548.8111	L	***490.2871***	245.6472	473.2606	237.1339	472.2766	236.6419	4
10	1243.6681	622.3377	1226.6416	613.8244	1225.6575	613.3324	E	***377.2031***	189.1052	360.1765	180.5919	359.1925	180.0999	3
11	1344.7158	672.8615	1327.6892	664.3483	1326.7052	663.8563	T	***248.1605***	124.5839	231.1339	116.0706	230.1499	115.5786	2
12							K	***147.1128***	74.0600	130.0863	65.5468			1

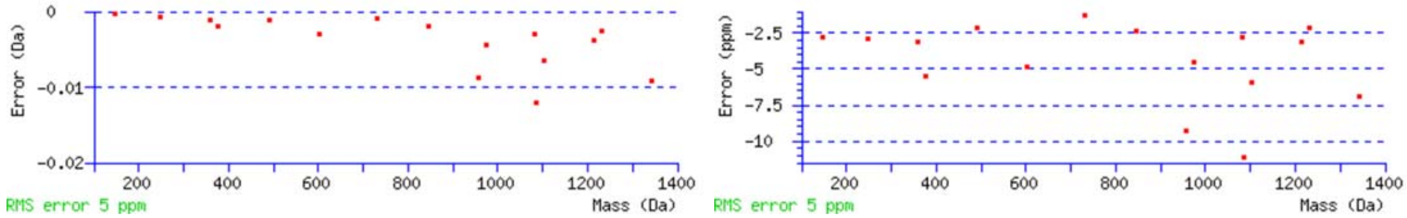

NCBI **BLAST** search of FLEQQNKLLETK
(Parameters: blastp, nr protein database, expect=20000, no filter, PAM30)
Other BLAST web gateways

All matches to this query

Score	Mr(calc)	Delta	Sequence
43.3	1489.8140	-0.0004	FLEQQNKLLETK

Mascot: http://www.matrixscience.com/

Mascot Search Results

Peptide View

MS/MS Fragmentation of **FLEQQNKLLETK**
Found in **ch12u_Q9NSB2|KRT84_HUMAN** in **uni_human**, Keratin, type II cuticular Hb4 OS=Homo sapiens GN=KRT84 PE=2 SV=2

Match to Query 18197: 1489.814892 from(497.612240,3+) intensity(929317.0625) rtinseconds(1456) scans(7783) index(36044)
Title: 160219_Sunil_SDSII_A_Spectrum090001_scans__7783_RTINSECONDS=1456
Data file L:\\QE_2016\\160219_Sunil_KAP_LKC\\TMgf\\T\\T160219_Sunil_SDSII_A.mgf

Click mouse within plot area to zoom in by factor of two about that point
Or, Plot from 100 to 1400 Da Full range
Label all possible matches ○ Label matches used for scoring ◉
Show Y-axis ☐

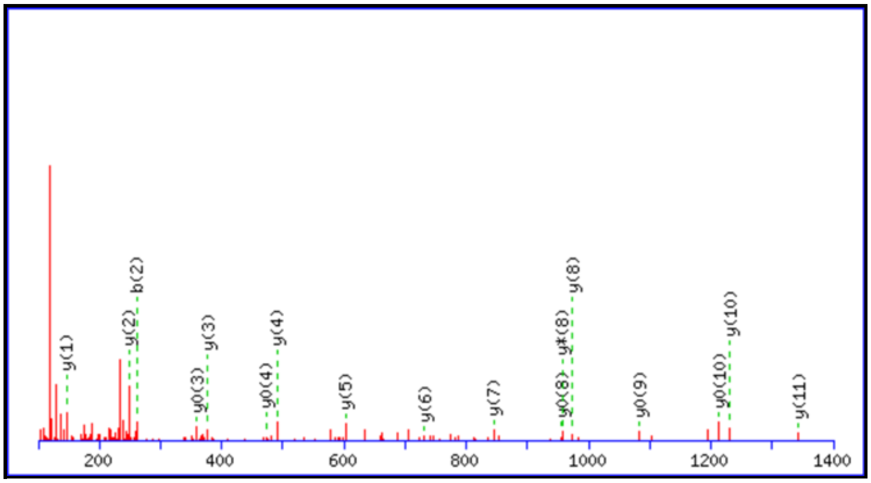

Monoisotopic mass of neutral peptide Mr(calc): 1489.8140
Fixed modifications: Carbamidomethyl (C) (apply to specified residues or termini only)
Ions Score: 53 **Expect:** 0.00064
Matches : 17/120 fragment ions using 43 most intense peaks (help)

#	b	b^{++}	b*	b^{*++}	b^0	b^{0++}	Seq.	y	y^{++}	y*	y^{*++}	y^0	y^{0++}	#
1	148.0757	74.5415					F							12
2	**261.1598**	131.0835					L	***1343.7529***	672.3801	1326.7264	663.8668	1325.7423	663.3748	11
3	390.2023	195.6048			372.1918	186.5995	E	***1230.6688***	615.8381	1213.6423	607.3248	1212.6583	606.8328	10
4	518.2609	259.6341	501.2344	251.1208	500.2504	250.6288	Q	1101.6263	551.3168	1084.5997	542.8035	1083.6157	542.3115	9
5	646.3195	323.6634	629.2930	315.1501	628.3089	314.6581	Q	***973.5677***	487.2875	956.5411	478.7742	955.5571	478.2822	8
6	760.3624	380.6849	743.3359	372.1716	742.3519	371.6796	N	***845.5091***	423.2582	828.4825	414.7449	827.4985	414.2529	7
7	888.4574	444.7323	871.4308	436.2191	870.4468	435.7271	K	***731.4662***	366.2367	714.4396	357.7234	713.4556	357.2314	6
8	1001.5415	501.2744	984.5149	492.7611	983.5309	492.2691	L	***603.3712***	302.1892	586.3447	293.6760	585.3606	293.1840	5
9	1114.6255	557.8164	1097.5990	549.3031	1096.6150	548.8111	L	***490.2871***	245.6472	473.2606	237.1339	472.2766	236.6419	4
10	1243.6681	622.3377	1226.6416	613.8244	1225.6575	613.3324	E	***377.2031***	189.1052	360.1765	180.5919	359.1925	180.0999	3
11	1344.7158	672.8615	1327.6892	664.3483	1326.7052	663.8563	T	***248.1605***	124.5839	231.1339	116.0706	230.1499	115.5786	2
12							K	***147.1128***	74.0600	130.0863	65.5468			1

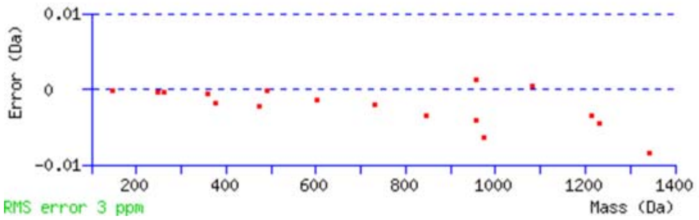

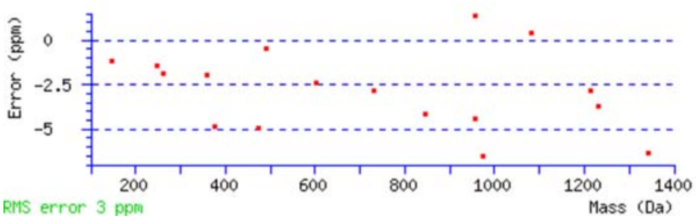

NCBI **BLAST** search of FLEQQNKLLETK
(Parameters: blastp, nr protein database, expect=20000, no filter, PAM30)
Other BLAST web gateways

All matches to this query

Score	Mr(calc)	Delta	Sequence
52.5	1489.8140	0.0009	FLEQQNKLLETK
0.3	1487.8069	2.0080	NVHRPPRQRDIT

Mascot: http://www.matrixscience.com/

Mascot Search Results

Peptide View

MS/MS Fragmentation of **FLEQQNKLLETK**
Found in **ch12u_Q9NSB2|KRT84_HUMAN** in **uni_human**, Keratin, type II cuticular Hb4 OS=Homo sapiens GN=KRT84 PE=2 SV=2

Match to Query 18199: 1489.815372 from(497.612400,3+) intensity(1418781.8750) rtinseconds(1564) scans(8310) index(21333)
Title: 160219_Sunil_SDSII_A_Spectrum073894_scans__8310_RTINSECONDS=1564
Data file L:\\QE_2016\\160219_Sunil_KAP_LKC\\TMgf\\T\\T160219_Sunil_SDSII_A.mgf

Click mouse within plot area to zoom in by factor of two about that point
Or, Plot from 100 to 1500 Da Full range
Label all possible matches ○ Label matches used for scoring ◉
Show Y-axis ☐

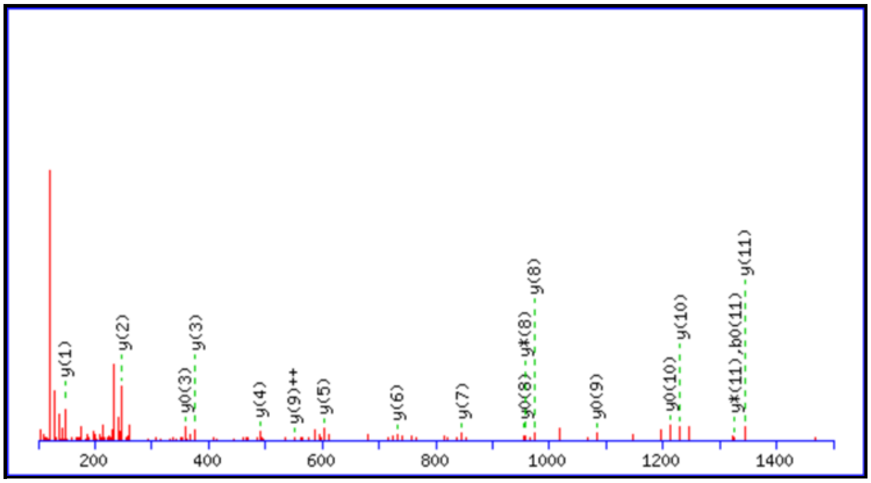

Monoisotopic mass of neutral peptide Mr(calc): 1489.8140
Fixed modifications: Carbamidomethyl (C) (apply to specified residues or termini only)
Ions Score: 55 **Expect:** 0.00034
Matches : 18/120 fragment ions using 39 most intense peaks (help)

#	b	b++	b*	b*++	b0	b0++	Seq.	y	y++	y*	y*++	y0	y0++	#
1	148.0757	74.5415					F							12
2	261.1598	131.0835					L	**1343.7529**	672.3801	1326.7264	663.8668	1325.7423	663.3748	11
3	390.2023	195.6048			372.1918	186.5995	E	**1230.6688**	615.8381	1213.6423	607.3248	1212.6583	606.8328	10
4	518.2609	259.6341	501.2344	251.1208	500.2504	250.6288	Q	1101.6263	551.3168	1084.5997	542.8035	1083.6157	542.3115	9
5	646.3195	323.6634	629.2930	315.1501	628.3089	314.6581	Q	**973.5677**	487.2875	956.5411	478.7742	955.5571	478.2822	8
6	760.3624	380.6849	743.3359	372.1716	742.3519	371.6796	N	**845.5091**	423.2582	828.4825	414.7449	827.4985	414.2529	7
7	888.4574	444.7323	871.4308	436.2191	870.4468	435.7271	K	**731.4662**	366.2367	714.4396	357.7234	713.4556	357.2314	6
8	1001.5415	501.2744	984.5149	492.7611	983.5309	492.2691	L	**603.3712**	302.1892	586.3447	293.6760	585.3606	293.1840	5
9	1114.6255	557.8164	1097.5990	549.3031	1096.6150	548.8111	L	**490.2871**	245.6472	473.2606	237.1339	472.2766	236.6419	4
10	1243.6681	622.3377	1226.6416	613.8244	1225.6575	613.3324	E	**377.2031**	189.1052	360.1765	180.5919	359.1925	180.0999	3
11	1344.7158	672.8615	1327.6892	664.3483	1326.7052	663.8563	T	**248.1605**	124.5839	231.1339	116.0706	230.1499	115.5786	2
12							K	**147.1128**	74.0600	130.0863	65.5468			1

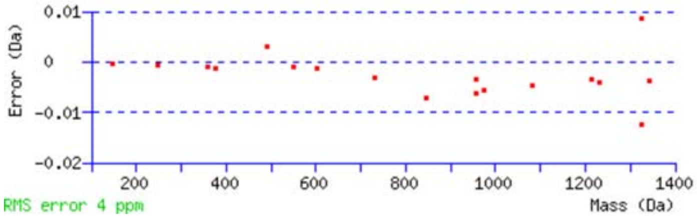

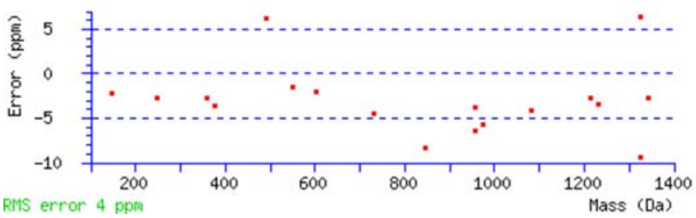

NCBI **BLAST** search of FLEQQNKLLETK
(Parameters: blastp, nr protein database, expect=20000, no filter, PAM30)
Other BLAST web gateways

All matches to this query

Score	Mr(calc)	Delta	Sequence
55.0	1489.8140	0.0014	FLEQQNKLLETK
0.2	1487.8069	2.0084	NVHRPPRQRDIT

Mascot: http://www.matrixscience.com/

Mascot Search Results

Peptide View

MS/MS Fragmentation of **FLEQQNKLLETK**
Found in **ch12u_Q9NSB2|KRT84_HUMAN** in **uni_human**, Keratin, type II cuticular Hb4 OS=Homo sapiens GN=KRT84 PE=2 SV=2

Match to Query 18202: 1489.815552 from(497.612460,3+) intensity(2472952.5000) rtinseconds(2039) scans(11060) index(23770)
Title: 160219_Sunil_SDSII_A_Spectrum076332_scans__11060_RTINSECONDS=2039
Data file L:\\QE_2016\\160219_Sunil_KAP_LKC\\TMgf\\T\\T160219_Sunil_SDSII_A.mgf

Click mouse within plot area to zoom in by factor of two about that point
Or, Plot from 100 to 1500 Da Full range
Label all possible matches ○ Label matches used for scoring ◉
Show Y-axis □

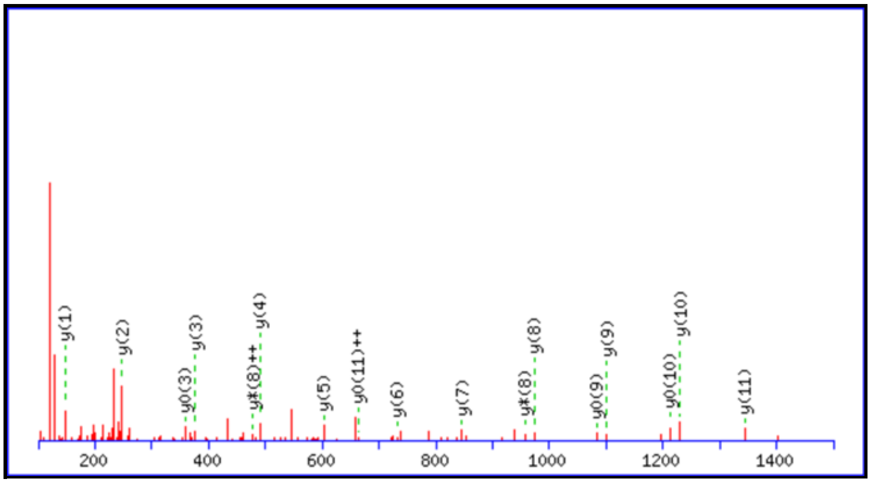

Monoisotopic mass of neutral peptide Mr(calc): 1489.8140
Fixed modifications: Carbamidomethyl (C) (apply to specified residues or termini only)
Ions Score: 62 **Expect:** 7.5e-005
Matches : 17/120 fragment ions using 44 most intense peaks (help)

#	b	b^{++}	b*	b^{*++}	b^0	b^{0++}	Seq.	y	y^{++}	y*	y^{*++}	y^0	y^{0++}	#
1	148.0757	74.5415					F							12
2	261.1598	131.0835					L	**1343.7529**	672.3801	1326.7264	663.8668	1325.7423	663.3748	11
3	390.2023	195.6048			372.1918	186.5995	E	**1230.6688**	615.8381	1213.6423	607.3248	1212.6583	606.8328	10
4	518.2609	259.6341	501.2344	251.1208	500.2504	250.6288	Q	**1101.6263**	551.3168	1084.5997	542.8035	1083.6157	542.3115	9
5	646.3195	323.6634	629.2930	315.1501	628.3089	314.6581	Q	**973.5677**	487.2875	956.5411	478.7742	955.5571	478.2822	8
6	760.3624	380.6849	743.3359	372.1716	742.3519	371.6796	N	**845.5091**	423.2582	828.4825	414.7449	827.4985	414.2529	7
7	888.4574	444.7323	871.4308	436.2191	870.4468	435.7271	K	**731.4662**	366.2367	714.4396	357.7234	713.4556	357.2314	6
8	1001.5415	501.2744	984.5149	492.7611	983.5309	492.2691	L	**603.3712**	302.1892	586.3447	293.6760	585.3606	293.1840	5
9	1114.6255	557.8164	1097.5990	549.3031	1096.6150	548.8111	L	**490.2871**	245.6472	473.2606	237.1339	472.2766	236.6419	4
10	1243.6681	622.3377	1226.6416	613.8244	1225.6575	613.3324	E	**377.2031**	189.1052	360.1765	180.5919	359.1925	180.0999	3
11	1344.7158	672.8615	1327.6892	664.3483	1326.7052	663.8563	T	**248.1605**	124.5839	231.1339	116.0706	230.1499	115.5786	2
12							K	**147.1128**	74.0600	130.0863	65.5468			1

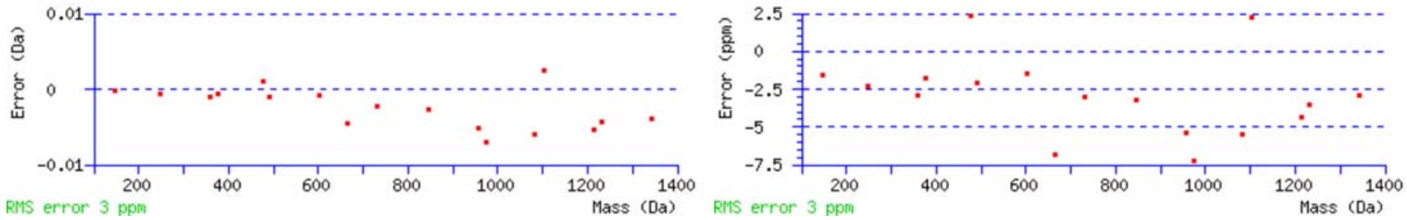

NCBI **BLAST** search of FLEQQNKLLETK
(Parameters: blastp, nr protein database, expect=20000, no filter, PAM30)
Other BLAST web gateways

All matches to this query

Score	Mr(calc)	Delta	Sequence
61.5	1489.8140	0.0015	FLEQQNKLLETK

Mascot: http://www.matrixscience.com/

Mascot Search Results

Peptide View

MS/MS Fragmentation of **FLEQQNKLLETK**
Found in **ch12u_Q9NSB2|KRT84_HUMAN** in **uni_human**, Keratin, type II cuticular Hb4 OS=Homo sapiens GN=KRT84 PE=2 SV=2

Match to Query 18204: 1489.816092 from(497.612640,3+) intensity(4722457.5000) rtinseconds(1792) scans(9332) index(7069)
Title: 160219_Sunil_SDSII_A_Spectrum058392_scans__9332_RTINSECONDS=1792
Data file L:\\QE_2016\\160219_Sunil_KAP_LKC\\TMgf\\T\\T160219_Sunil_SDSII_A.mgf

Click mouse within plot area to zoom in by factor of two about that point
Or, Plot from 100 to 1400 Da Full range
Label all possible matches ○ Label matches used for scoring ◉
Show Y-axis □

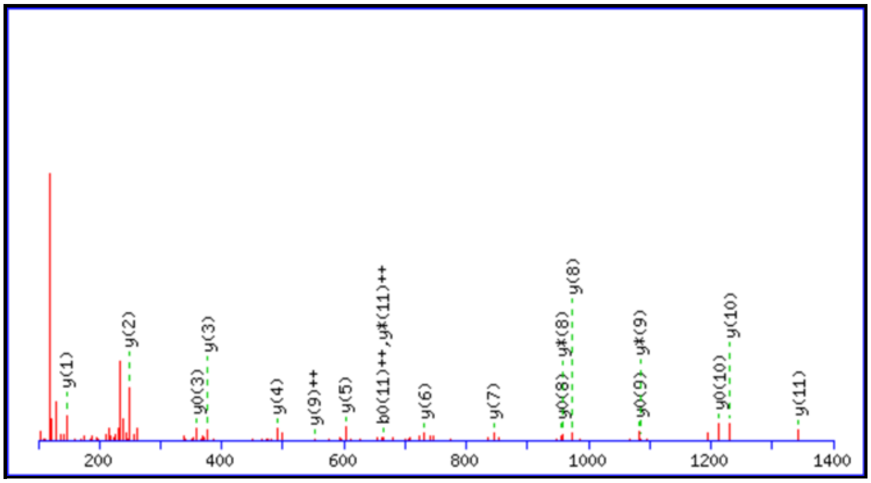

Monoisotopic mass of neutral peptide Mr(calc): 1489.8140
Fixed modifications: Carbamidomethyl (C) (apply to specified residues or termini only)
Ions Score: 64 **Expect:** 3.8e-005
Matches : 19/120 fragment ions using 34 most intense peaks (help)

#	b	b^{++}	b*	b^{*++}	b^0	b^{0++}	Seq.	y	y^{++}	y*	y^{*++}	y^0	y^{0++}	#
1	148.0757	74.5415					F							12
2	261.1598	131.0835					L	1343.7529	672.3801	1326.7264	663.8668	1325.7423	663.3748	11
3	390.2023	195.6048			372.1918	186.5995	E	1230.6688	615.8381	1213.6423	607.3248	1212.6583	606.8328	10
4	518.2609	259.6341	501.2344	251.1208	500.2504	250.6288	Q	1101.6263	551.3168	1084.5997	542.8035	1083.6157	542.3115	9
5	646.3195	323.6634	629.2930	315.1501	628.3089	314.6581	Q	973.5677	487.2875	956.5411	478.7742	955.5571	478.2822	8
6	760.3624	380.6849	743.3359	372.1716	742.3519	371.6796	N	845.5091	423.2582	828.4825	414.7449	827.4985	414.2529	7
7	888.4574	444.7323	871.4308	436.2191	870.4468	435.7271	K	731.4662	366.2367	714.4396	357.7234	713.4556	357.2314	6
8	1001.5415	501.2744	984.5149	492.7611	983.5309	492.2691	L	603.3712	302.1892	586.3447	293.6760	585.3606	293.1840	5
9	1114.6255	557.8164	1097.5990	549.3031	1096.6150	548.8111	L	490.2871	245.6472	473.2606	237.1339	472.2766	236.6419	4
10	1243.6681	622.3377	1226.6416	613.8244	1225.6575	613.3324	E	377.2031	189.1052	360.1765	180.5919	359.1925	180.0999	3
11	1344.7158	672.8615	1327.6892	664.3483	1326.7052	663.8563	T	248.1605	124.5839	231.1339	116.0706	230.1499	115.5786	2
12							K	147.1128	74.0600	130.0863	65.5468			1

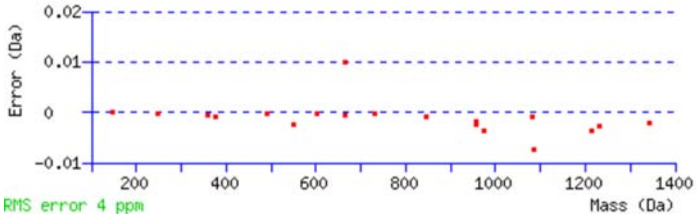

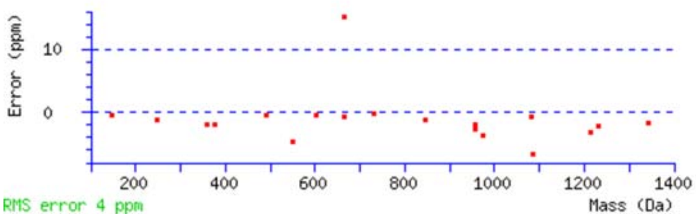

NCBI **BLAST** search of FLEQQNKLLETK
(Parameters: blastp, nr protein database, expect=20000, no filter, PAM30)
Other BLAST web gateways

All matches to this query

Score	Mr(calc)	Delta	Sequence
64.3	1489.8140	0.0021	FLEQQNKLLETK
0.9	1487.8069	2.0092	NVHRPPRQRDIT

Mascot: http://www.matrixscience.com/

Mascot Search Results

Peptide View

MS/MS Fragmentation of **FLEQQNKLLETK**
Found in **ch12u_Q9NSB2|KRT84_HUMAN** in **uni_human**, Keratin, type II cuticular Hb4 OS=Homo sapiens GN=KRT84 PE=2 SV=2

Match to Query 18206: 1489.817202 from(497.613010,3+) intensity(27789082.0000) rtinseconds(1695) scans(8767) index(6583)
Title: 160219_Sunil_SDSII_A_Spectrum057906_scans__8767_RTINSECONDS=1695
Data file L:\\QE_2016\\160219_Sunil_KAP_LKC\\TMgf\\T\\T160219_Sunil_SDSII_A.mgf

Click mouse within plot area to zoom in by factor of two about that point
Or, Plot from 100 to 1400 Da Full range
Label all possible matches ○ Label matches used for scoring ◉
Show Y-axis ☐

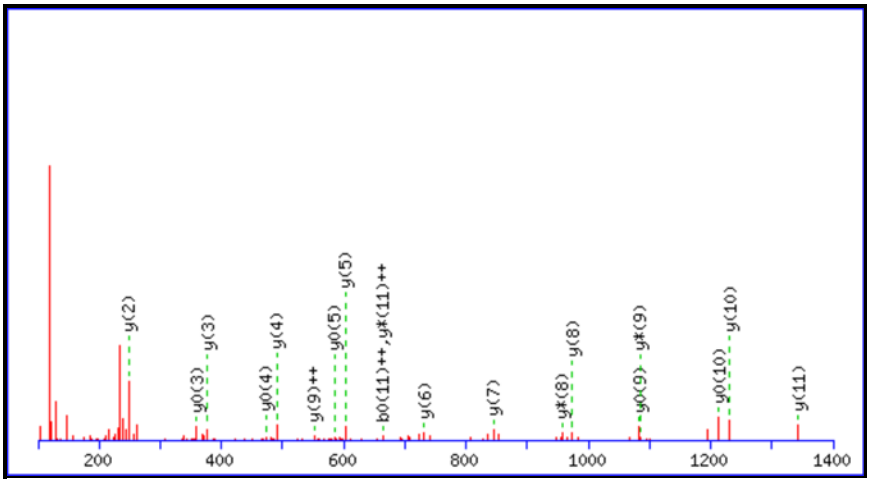

Monoisotopic mass of neutral peptide Mr(calc): 1489.8140
Fixed modifications: Carbamidomethyl (C) (apply to specified residues or termini only)
Ions Score: 72 **Expect:** 6.8e-006
Matches : 19/120 fragment ions using 24 most intense peaks (help)

#	b	b++	b*	b*++	b0	b0++	Seq.	y	y++	y*	y*++	y0	y0++	#
1	148.0757	74.5415					F							12
2	261.1598	131.0835					L	**1343.7529**	672.3801	1326.7264	663.8668	1325.7423	663.3748	11
3	390.2023	195.6048			372.1918	186.5995	E	**1230.6688**	615.8381	1213.6423	607.3248	1212.6583	606.8328	10
4	518.2609	259.6341	501.2344	251.1208	500.2504	250.6288	Q	1101.6263	551.3168	1084.5997	542.8035	1083.6157	542.3115	9
5	646.3195	323.6634	629.2930	315.1501	628.3089	314.6581	Q	**973.5677**	487.2875	956.5411	478.7742	955.5571	478.2822	8
6	760.3624	380.6849	743.3359	372.1716	742.3519	371.6796	N	**845.5091**	423.2582	828.4825	414.7449	827.4985	414.2529	7
7	888.4574	444.7323	871.4308	436.2191	870.4468	435.7271	K	**731.4662**	366.2367	714.4396	357.7234	713.4556	357.2314	6
8	1001.5415	501.2744	984.5149	492.7611	983.5309	492.2691	L	**603.3712**	302.1892	586.3447	293.6760	585.3606	293.1840	5
9	1114.6255	557.8164	1097.5990	549.3031	1096.6150	548.8111	L	**490.2871**	245.6472	473.2606	237.1339	472.2766	236.6419	4
10	1243.6681	622.3377	1226.6416	613.8244	1225.6575	613.3324	E	**377.2031**	189.1052	360.1765	180.5919	359.1925	180.0999	3
11	1344.7158	672.8615	1327.6892	664.3483	1326.7052	663.8563	T	**248.1605**	124.5839	231.1339	116.0706	230.1499	115.5786	2
12							K	147.1128	74.0600	130.0863	65.5468			1

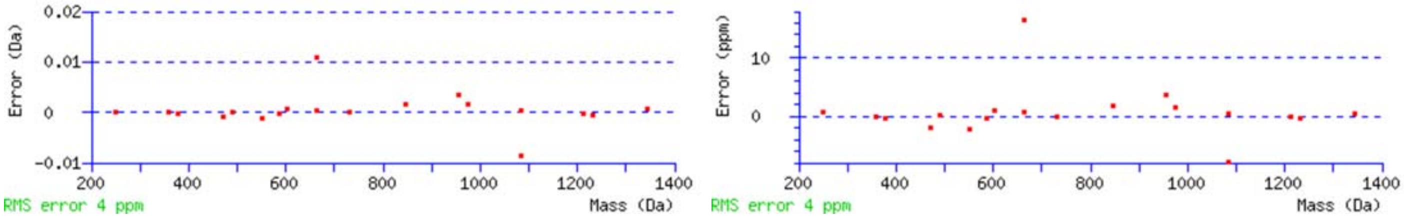

NCBI **BLAST** search of FLEQQNKLLETK
(Parameters: blastp, nr protein database, expect=20000, no filter, PAM30)
Other BLAST web gateways

All matches to this query

Score	Mr(calc)	Delta	Sequence
71.8	1489.8140	0.0032	FLEQQNKLLETK
0.8	1487.8069	2.0103	NVHRPPRQRDIT

Mascot: http://www.matrixscience.com/

Mascot Search Results

Peptide View

MS/MS Fragmentation of **FLEQQNKLLETK**
Found in **ch12u_Q9NSB2|KRT84_HUMAN** in **uni_human**, Keratin, type II cuticular Hb4 OS=Homo sapiens GN=KRT84 PE=2 SV=2

Match to Query 18206: 1489.817202 from(497.613010,3+) intensity(27789082.0000) rtinseconds(1695) scans(8767) index(6583)
Title: 160219_Sunil_SDSII_A_Spectrum057906_scans__8767_RTINSECONDS=1695
Data file L:\\QE_2016\\160219_Sunil_KAP_LKC\\TMgf\\T\\T160219_Sunil_SDSII_A.mgf

Click mouse within plot area to zoom in by factor of two about that point
Or, Plot from 100 to 1400 Da Full range
Label all possible matches ○ Label matches used for scoring ◉
Show Y-axis ☐

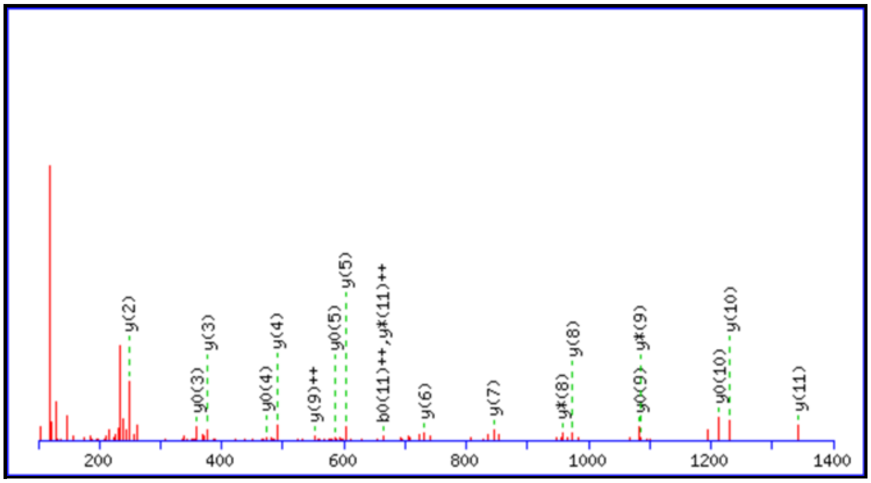


```
Monoisotopic mass of neutral peptide Mr(calc): 1489.8140
Fixed modifications: Carbamidomethyl (C) (apply to specified residues or termini only)
Ions Score: 72  Expect: 6.8e-006
Matches : 19/120 fragment ions using 24 most intense peaks   (help)
```

#	b	b^{++}	b*	b^{*++}	b^0	b^{0++}	Seq.	y	y^{++}	y*	y^{*++}	y^0	y^{0++}	#
1	148.0757	74.5415					F							12
2	261.1598	131.0835					L	1343.7529	672.3801	1326.7264	663.8668	1325.7423	663.3748	11
3	390.2023	195.6048			372.1918	186.5995	E	1230.6688	615.8381	1213.6423	607.3248	1212.6583	606.8328	10
4	518.2609	259.6341	501.2344	251.1208	500.2504	250.6288	Q	1101.6263	551.3168	1084.5997	542.8035	1083.6157	542.3115	9
5	646.3195	323.6634	629.2930	315.1501	628.3089	314.6581	Q	973.5677	487.2875	956.5411	478.7742	955.5571	478.2822	8
6	760.3624	380.6849	743.3359	372.1716	742.3519	371.6796	N	845.5091	423.2582	828.4825	414.7449	827.4985	414.2529	7
7	888.4574	444.7323	871.4308	436.2191	870.4468	435.7271	K	731.4662	366.2367	714.4396	357.7234	713.4556	357.2314	6
8	1001.5415	501.2744	984.5149	492.7611	983.5309	492.2691	L	603.3712	302.1892	586.3447	293.6760	585.3606	293.1840	5
9	1114.6255	557.8164	1097.5990	549.3031	1096.6150	548.8111	L	490.2871	245.6472	473.2606	237.1339	472.2766	236.6419	4
10	1243.6681	622.3377	1226.6416	613.8244	1225.6575	613.3324	E	377.2031	189.1052	360.1765	180.5919	359.1925	180.0999	3
11	1344.7158	672.8615	1327.6892	664.3483	1326.7052	663.8563	T	248.1605	124.5839	231.1339	116.0706	230.1499	115.5786	2
12							K	147.1128	74.0600	130.0863	65.5468			1

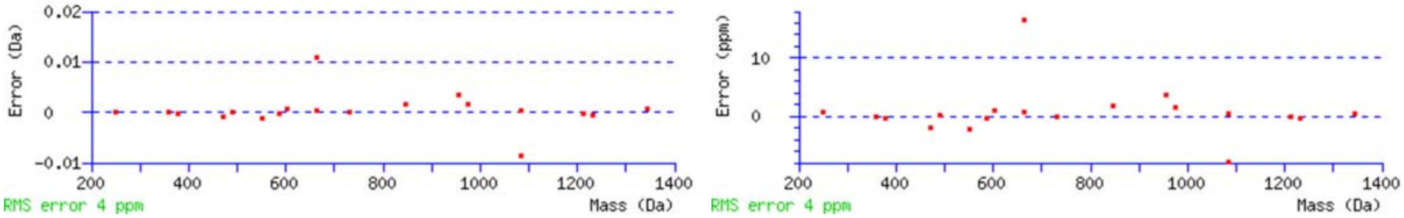

NCBI **BLAST** search of FLEQQNKLLETK
(Parameters: blastp, nr protein database, expect=20000, no filter, PAM30)
Other BLAST web gateways

All matches to this query

Score	Mr(calc)	Delta	Sequence
71.8	1489.8140	0.0032	FLEQQNKLLETK
0.8	1487.8069	2.0103	NVHRPPRQRDIT

Mascot: http://www.matrixscience.com/

Mascot Search Results

Peptide View

MS/MS Fragmentation of **FLEQQNKLLETK**
Found in **ch12u_Q9NSB2|KRT84_HUMAN** in **uni_human**, Keratin, type II cuticular Hb4 OS=Homo sapiens GN=KRT84 PE=2 SV=2

Match to Query 18259: 1490.803302 from(497.941710,3+) intensity(18317834.0000) rtinseconds(1759) scans(9140) index(6899)
Title: 160219_Sunil_SDSII_A_Spectrum058222_scans__9140_RTINSECONDS=1759
Data file L:\\QE_2016\\160219_Sunil_KAP_LKC\\TMgf\\T\\T160219_Sunil_SDSII_A.mgf

Click mouse within plot area to zoom in by factor of two about that point
Or, Plot from 100 to 1400 Da Full range
Label all possible matches ○ Label matches used for scoring ◉
Show Y-axis ☐

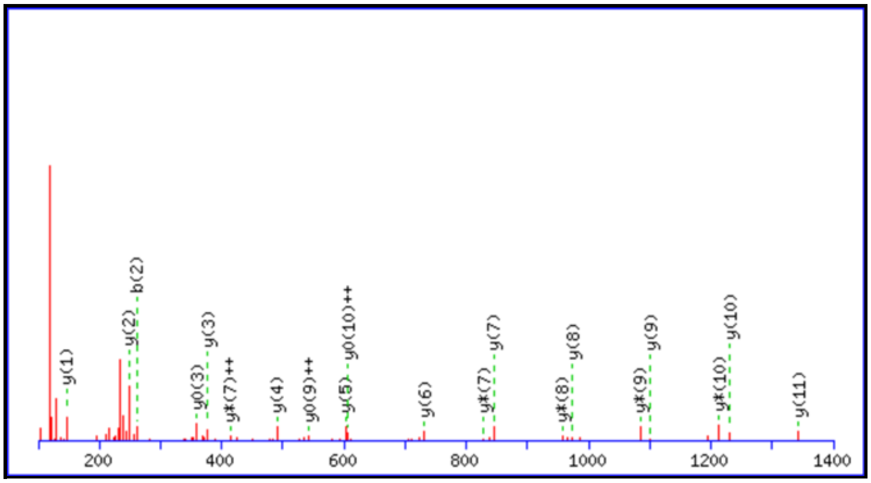

Monoisotopic mass of neutral peptide Mr(calc): 1489.8140
Fixed modifications: Carbamidomethyl (C) (apply to specified residues or termini only)
Ions Score: 72 **Expect:** 7e-006
Matches : 20/120 fragment ions using 42 most intense peaks (help)

#	b	b^{++}	b*	b^{*++}	b^0	b^{0++}	Seq.	y	y^{++}	y*	y^{*++}	y^0	y^{0++}	#
1	148.0757	74.5415					F							12
2	**261.1598**	131.0835					L	***1343.7529***	672.3801	1326.7264	663.8668	1325.7423	663.3748	11
3	390.2023	195.6048			372.1918	186.5995	E	***1230.6688***	615.8381	1213.6423	607.3248	1212.6583	606.8328	10
4	518.2609	259.6341	501.2344	251.1208	500.2504	250.6288	Q	***1101.6263***	551.3168	1084.5997	542.8035	1083.6157	542.3115	9
5	646.3195	323.6634	629.2930	315.1501	628.3089	314.6581	Q	***973.5677***	487.2875	956.5411	478.7742	955.5571	478.2822	8
6	760.3624	380.6849	743.3359	372.1716	742.3519	371.6796	N	***845.5091***	423.2582	828.4825	414.7449	827.4985	414.2529	7
7	888.4574	444.7323	871.4308	436.2191	870.4468	435.7271	K	***731.4662***	366.2367	714.4396	357.7234	713.4556	357.2314	6
8	1001.5415	501.2744	984.5149	492.7611	983.5309	492.2691	L	***603.3712***	302.1892	586.3447	293.6760	585.3606	293.1840	5
9	1114.6255	557.8164	1097.5990	549.3031	1096.6150	548.8111	L	***490.2871***	245.6472	473.2606	237.1339	472.2766	236.6419	4
10	1243.6681	622.3377	1226.6416	613.8244	1225.6575	613.3324	E	***377.2031***	189.1052	360.1765	180.5919	359.1925	180.0999	3
11	1344.7158	672.8615	1327.6892	664.3483	1326.7052	663.8563	T	***248.1605***	124.5839	231.1339	116.0706	230.1499	115.5786	2
12							K	***147.1128***	74.0600	130.0863	65.5468			1

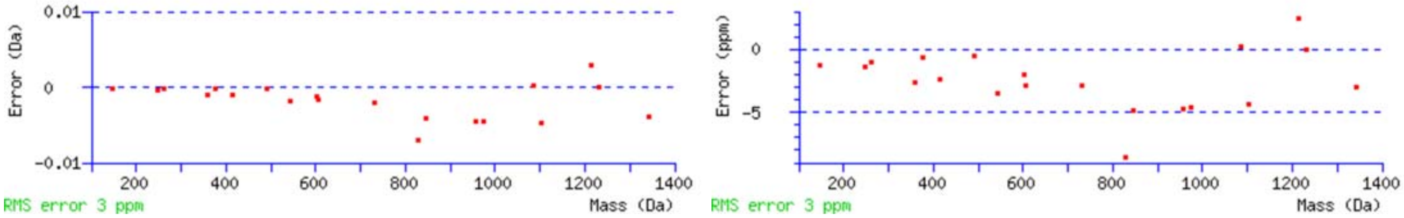

NCBI **BLAST** search of FLEQQNKLLETK
(Parameters: blastp, nr protein database, expect=20000, no filter, PAM30)
Other BLAST web gateways

All matches to this query

Score	Mr(calc)	Delta	Sequence
72.5	1489.8140	0.9893	FLEQQNKLLETK
33.6	1490.7980	0.0053	FLEQQNKLLETK
28.2	1490.7980	0.0053	FLEQQNKLLETK
28.0	1490.7980	0.0053	FLEQQNKLLETK
1.1	1490.8093	-0.0059	GRIYLKAEVADEK
1.0	1488.7909	2.0124	NVHRPPRQRDIT
1.0	1488.7909	2.0124	NVHRPPRQRDIT

Mascot: http://www.matrixscience.com/

Mascot Search Results

Peptide View

MS/MS Fragmentation of **VRFLEQQNKLLETK**
Found in **ch12u_Q9NSB2|KRT84_HUMAN** in **uni_human**, Keratin, type II cuticular Hb4 OS=Homo sapiens GN=KRT84 PE=2 SV=2

Match to Query 24746: 1745.987142 from(583.002990,3+) intensity(1040928.2500) rtinseconds(1699) scans(8793) index(6605)
Title: 160219_Sunil_SDSII_A_Spectrum057928_scans__8793_RTINSECONDS=1699
Data file L:\\QE_2016\\160219_Sunil_KAP_LKC\\TMgf\\T\\T160219_Sunil_SDSII_A.mgf

Click mouse within plot area to zoom in by factor of two about that point
Or, Plot from 100 to 1800 Da Full range
Label all possible matches ○ Label matches used for scoring ◉
Show Y-axis ☐

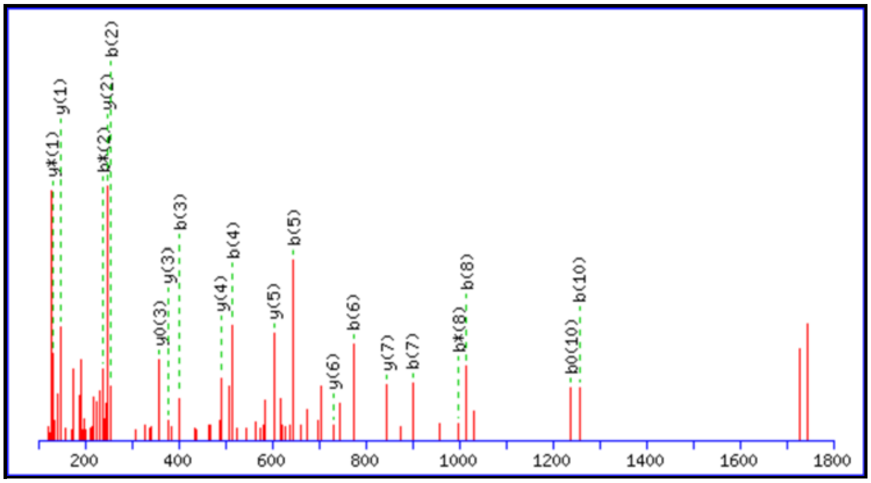

Monoisotopic mass of neutral peptide Mr(calc): 1744.9835
Fixed modifications: Carbamidomethyl (C) (apply to specified residues or termini only)
Ions Score: 69 **Expect:** 6.3e-006
Matches : 20/144 fragment ions using 31 most intense peaks (help)

#	b	b++	b*	b*++	b^0	b^{0++}	Seq.	y	y++	y*	y*++	y^0	y^{0++}	#
1	100.0757	50.5415					V							14
2	***256.1768***	128.5920	239.1503	120.0788			R	1646.9224	823.9649	1629.8959	815.4516	1628.9119	814.9596	13
3	***403.2452***	202.1262	386.2187	193.6130			F	1490.8213	745.9143	1473.7948	737.4010	1472.8108	736.9090	12
4	***516.3293***	258.6683	499.3027	250.1550			L	1343.7529	672.3801	1326.7264	663.8668	1325.7423	663.3748	11
5	***645.3719***	323.1896	628.3453	314.6763	627.3613	314.1843	E	1230.6688	615.8381	1213.6423	607.3248	1212.6583	606.8328	10
6	***773.4305***	387.2189	756.4039	378.7056	755.4199	378.2136	Q	1101.6263	551.3168	1084.5997	542.8035	1083.6157	542.3115	9
7	***901.4890***	451.2482	884.4625	442.7349	883.4785	442.2429	Q	973.5677	487.2875	956.5411	478.7742	955.5571	478.2822	8
8	***1015.5320***	508.2696	998.5054	499.7563	997.5214	499.2643	N	***845.5091***	423.2582	828.4825	414.7449	827.4985	414.2529	7
9	1143.6269	572.3171	1126.6004	563.8038	1125.6164	563.3118	K	***731.4662***	366.2367	714.4396	357.7234	713.4556	357.2314	6
10	***1256.7110***	628.8591	1239.6844	620.3459	1238.7004	619.8538	L	***603.3712***	302.1892	586.3447	293.6760	585.3606	293.1840	5
11	1369.7950	685.4012	1352.7685	676.8879	1351.7845	676.3959	L	***490.2871***	245.6472	473.2606	237.1339	472.2766	236.6419	4
12	1498.8376	749.9225	1481.8111	741.4092	1480.8271	740.9172	E	***377.2031***	189.1052	360.1765	180.5919	359.1925	180.0999	3
13	1599.8853	800.4463	1582.8588	791.9330	1581.8748	791.4410	T	***248.1605***	124.5839	231.1339	116.0706	230.1499	115.5786	2
14							K	***147.1128***	74.0600	130.0863	65.5468			1

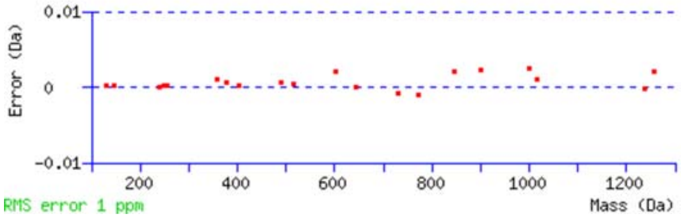

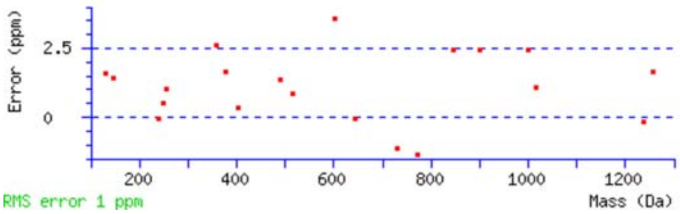

NCBI **BLAST** search of VRFLEQQNKLLETK
(Parameters: blastp, nr protein database, expect=20000, no filter, PAM30)
Other BLAST web gateways

All matches to this query

Score	Mr(calc)	Delta	Sequence
69.0	1744.9835	1.0036	VRFLEQQNKLLETK
7.9	1744.9869	1.0002	KSLESINSRLQLVMK
5.8	1745.9709	0.0162	KSLESINSRLQLVMK
1.5	1745.9709	0.0162	KSLESINSRLQLVMK

Mascot: http://www.matrixscience.com/

Mascot Search Results

Peptide View

MS/MS Fragmentation of **VRFLEQQNKLLETK**
Found in **ch12u_Q9NSB2|KRT84_HUMAN** in **uni_human**, Keratin, type II cuticular Hb4 OS=Homo sapiens GN=KRT84 PE=2 SV=2

Match to Query 24746: 1745.987142 from(583.002990,3+) intensity(1040928.2500) rtinseconds(1699) scans(8793) index(6605)
Title: 160219_Sunil_SDSII_A_Spectrum057928_scans__8793_RTINSECONDS=1699
Data file L:\\QE_2016\\160219_Sunil_KAP_LKC\\TMgf\\T\\T160219_Sunil_SDSII_A.mgf

Click mouse within plot area to zoom in by factor of two about that point
Or, Plot from 100 to 1800 Da Full range
Label all possible matches ○ Label matches used for scoring ◉
Show Y-axis ☐

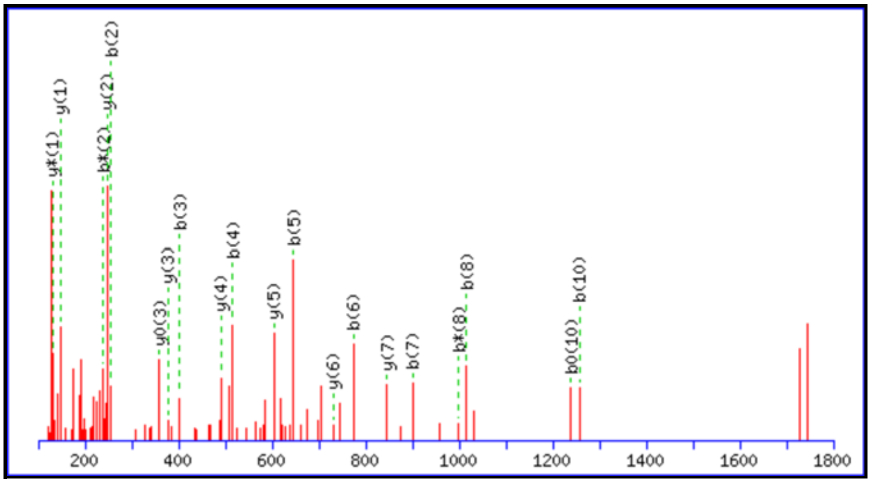

Monoisotopic mass of neutral peptide Mr(calc): 1744.9835
Fixed modifications: Carbamidomethyl (C) (apply to specified residues or termini only)
Ions Score: 69 **Expect:** 6.3e-006
Matches : 20/144 fragment ions using 31 most intense peaks (help)

#	b	b^{++}	b*	b^{*++}	b^{0}	b^{0++}	Seq.	y	y^{++}	y*	y^{*++}	y^{0}	y^{0++}	#
1	100.0757	50.5415					V							14
2	***256.1768***	128.5920	239.1503	120.0788			R	1646.9224	823.9649	1629.8959	815.4516	1628.9119	814.9596	13
3	***403.2452***	202.1262	386.2187	193.6130			F	1490.8213	745.9143	1473.7948	737.4010	1472.8108	736.9090	12
4	***516.3293***	258.6683	499.3027	250.1550			L	1343.7529	672.3801	1326.7264	663.8668	1325.7423	663.3748	11
5	***645.3719***	323.1896	628.3453	314.6763	627.3613	314.1843	E	1230.6688	615.8381	1213.6423	607.3248	1212.6583	606.8328	10
6	***773.4305***	387.2189	756.4039	378.7056	755.4199	378.2136	Q	1101.6263	551.3168	1084.5997	542.8035	1083.6157	542.3115	9
7	***901.4890***	451.2482	884.4625	442.7349	883.4785	442.2429	Q	973.5677	487.2875	956.5411	478.7742	955.5571	478.2822	8
8	***1015.5320***	508.2696	998.5054	499.7563	997.5214	499.2643	N	***845.5091***	423.2582	828.4825	414.7449	827.4985	414.2529	7
9	1143.6269	572.3171	1126.6004	563.8038	1125.6164	563.3118	K	***731.4662***	366.2367	714.4396	357.7234	713.4556	357.2314	6
10	***1256.7110***	628.8591	1239.6844	620.3459	1238.7004	619.8538	L	***603.3712***	302.1892	586.3447	293.6760	585.3606	293.1840	5
11	1369.7950	685.4012	1352.7685	676.8879	1351.7845	676.3959	L	***490.2871***	245.6472	473.2606	237.1339	472.2766	236.6419	4
12	1498.8376	749.9225	1481.8111	741.4092	1480.8271	740.9172	E	***377.2031***	189.1052	360.1765	180.5919	359.1925	180.0999	3
13	1599.8853	800.4463	1582.8588	791.9330	1581.8748	791.4410	T	***248.1605***	124.5839	231.1339	116.0706	230.1499	115.5786	2
14							K	***147.1128***	74.0600	130.0863	65.5468			1

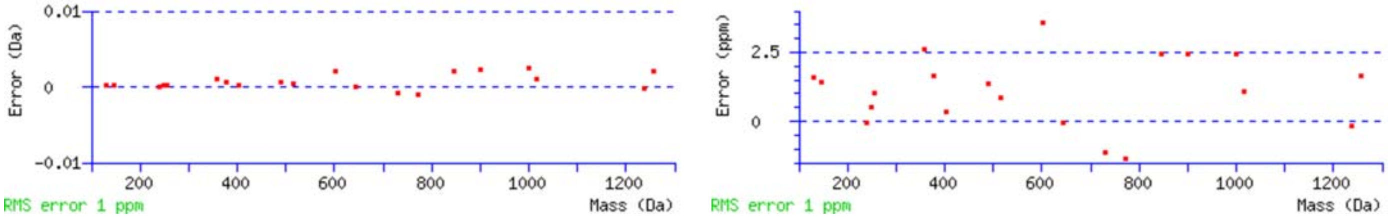

NCBI **BLAST** search of VRFLEQQNKLLETK
(Parameters: blastp, nr protein database, expect=20000, no filter, PAM30)
Other BLAST web gateways

All matches to this query

Score	Mr(calc)	Delta	Sequence
69.0	1744.9835	1.0036	VRFLEQQNKLLETK
7.9	1744.9869	1.0002	KSLESINSRLQLVMK
5.8	1745.9709	0.0162	KSLESINSRLQLVMK
1.5	1745.9709	0.0162	KSLESINSRLQLVMK

Mascot: http://www.matrixscience.com/

Mascot Search Results

Peptide View

MS/MS Fragmentation of **LGLDIEIATYR**
Found in **ch12u_Q9NSB2|KRT84_HUMAN** in **uni_human**, Keratin, type II cuticular Hb4 OS=Homo sapiens GN=KRT84 PE=2 SV=2

Match to Query 10882: 1262.687228 from(632.350890,2+) intensity(2016767.1250) rtinseconds(2661) scans(14659) index(26782)
Title: 160219_Sunil_SDSII_A_Spectrum079345_scans__14659_RTINSECONDS=2661
Data file L:\\QE_2016\\160219_Sunil_KAP_LKC\\TMgf\\T\\T160219_Sunil_SDSII_A.mgf

Click mouse within plot area to zoom in by factor of two about that point
Or, Plot from 100 to 1200 Da Full range
Label all possible matches ○ Label matches used for scoring ◉
Show Y-axis ☐

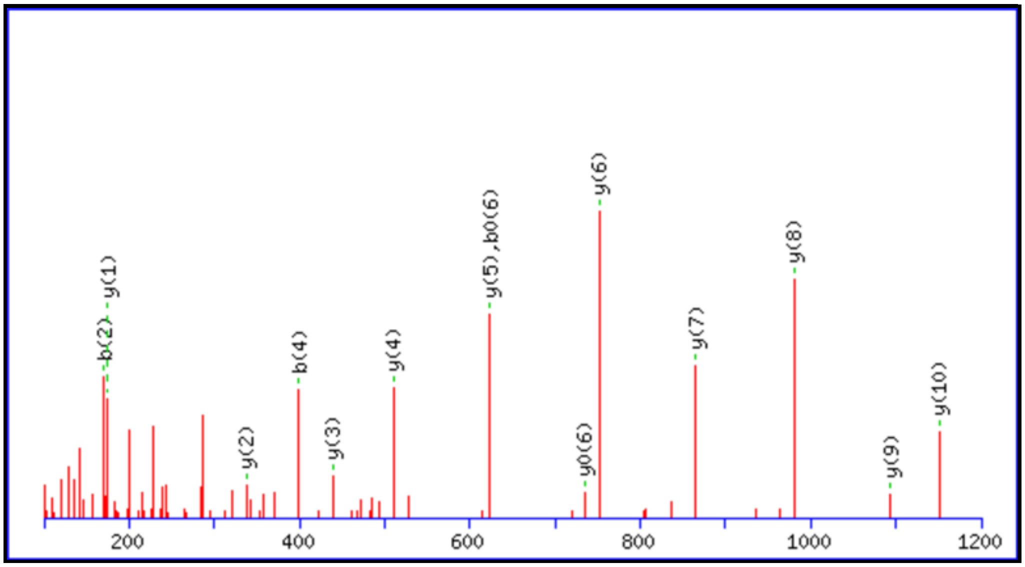

Monoisotopic mass of neutral peptide Mr(calc): 1262.6870
Fixed modifications: Carbamidomethyl (C) (apply to specified residues or termini only)
Ions Score: 89 **Expect:** 1.3e-007
Matches : 14/90 fragment ions using 20 most intense peaks (help)

#	b	b^{++}	b^0	b^{0++}	Seq.	y	y^{++}	y*	y^{*++}	y^0	y^{0++}	#
1	114.0913	57.5493			L							11
2	**171.1128**	86.0600			G	***1150.6103***	575.8088	1133.5837	567.2955	1132.5997	566.8035	10
3	284.1969	142.6021			L	***1093.5888***	547.2980	1076.5623	538.7848	1075.5782	538.2928	9
4	**399.2238**	200.1155	381.2132	191.1103	D	***980.5047***	490.7560	963.4782	482.2427	962.4942	481.7507	8
5	512.3079	256.6576	494.2973	247.6523	I	***865.4778***	433.2425	848.4512	424.7293	847.4672	424.2373	7
6	641.3505	321.1789	623.3399	312.1736	E	***752.3937***	376.7005	735.3672	368.1872	734.3832	367.6952	6
7	754.4345	377.7209	736.4240	368.7156	I	***623.3511***	312.1792	606.3246	303.6659	605.3406	303.1739	5
8	825.4716	413.2395	807.4611	404.2342	A	***510.2671***	255.6372	493.2405	247.1239	492.2565	246.6319	4
9	926.5193	463.7633	908.5088	454.7580	T	***439.2300***	220.1186	422.2034	211.6053	421.2194	211.1133	3

10	1089.5827	545.2950	1071.5721	536.2897	Y	338.1823	169.5948	321.1557	161.0815			2
11					R	175.1190	88.0631	158.0924	79.5498			1

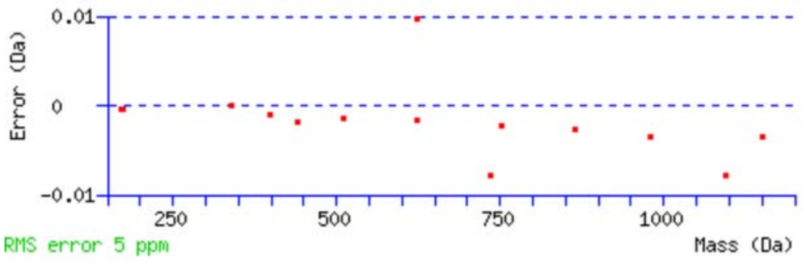

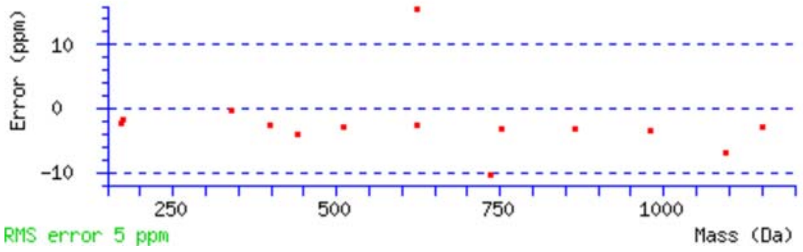

NCBI **BLAST** search of LGLDIEIATYR
(Parameters: blastp, nr protein database, expect=20000, no filter, PAM30)
Other BLAST web gateways

All matches to this query

Score	Mr(calc)	Delta	Sequence
89.3	1262.6870	0.0002	LGLDIEIATYR
37.5	1262.6870	0.0002	LALDVEIATYR
21.1	1262.6870	0.0002	IAVDELFTSLR
8.7	1262.6805	0.0067	VALLIGNMNYR
5.7	1262.6983	-0.0110	IGAQPVEIPPSR
5.2	1262.6983	-0.0111	IGDFGLATVKSR
1.9	1262.6884	-0.0011	GLVQRWRYGK
1.1	1262.6983	-0.0110	LVAAKAFTESAR
0.5	1261.6819	1.0053	DIGIWYNILR

Mascot: http://www.matrixscience.com/

Mascot Search Results

Peptide View

MS/MS Fragmentation of **FLEQQNKLLETK**
Found in **ch12u_Q9NSB2|KRT84_HUMAN** in **uni_human**, Keratin, type II cuticular Hb4 OS=Homo sapiens GN=KRT84 PE=2 SV=2

Match to Query 18174: 1489.811248 from(745.912900,2+) intensity(1430451.5000) rtinseconds(1473) scans(7784) index(20869)
Title: 160219_Sunil_SDSII_A_Spectrum073430_scans__7784_RTINSECONDS=1473
Data file L:\\QE_2016\\160219_Sunil_KAP_LKC\\TMgf\\T\\T160219_Sunil_SDSII_A.mgf

Click mouse within plot area to zoom in by factor of two about that point
Or, Plot from 100 to 1500 Da Full range
Label all possible matches ○ Label matches used for scoring ◉
Show Y-axis ☐

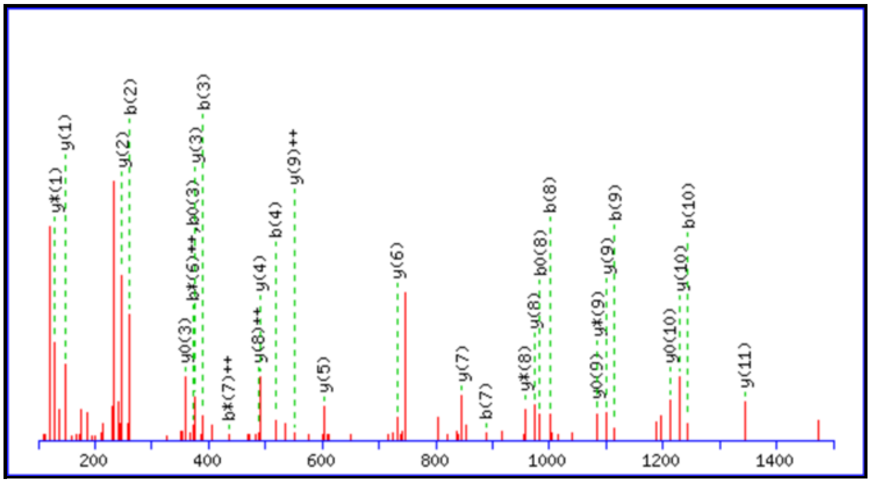

Monoisotopic mass of neutral peptide Mr(calc): 1489.8140
Fixed modifications: Carbamidomethyl (C) (apply to specified residues or termini only)
Ions Score: 84 **Expect:** 4.5e-007
Matches : 30/120 fragment ions using 48 most intense peaks (help)

#	b	b^{++}	b*	b^{*++}	b^0	b^{0++}	Seq.	y	y^{++}	y*	y^{*++}	y^0	y^{0++}	#
1	148.0757	74.5415					F							12
2	**261.1598**	131.0835					L	***1343.7529***	672.3801	1326.7264	663.8668	1325.7423	663.3748	11
3	**390.2023**	195.6048			372.1918	186.5995	E	***1230.6688***	615.8381	1213.6423	607.3248	1212.6583	606.8328	10
4	**518.2609**	259.6341	501.2344	251.1208	500.2504	250.6288	Q	***1101.6263***	551.3168	1084.5997	542.8035	1083.6157	542.3115	9
5	646.3195	323.6634	629.2930	315.1501	628.3089	314.6581	Q	***973.5677***	487.2875	956.5411	478.7742	955.5571	478.2822	8
6	760.3624	380.6849	743.3359	372.1716	742.3519	371.6796	N	***845.5091***	423.2582	828.4825	414.7449	827.4985	414.2529	7
7	**888.4574**	444.7323	871.4308	436.2191	870.4468	435.7271	K	***731.4662***	366.2367	714.4396	357.7234	713.4556	357.2314	6
8	**1001.5415**	501.2744	984.5149	492.7611	983.5309	492.2691	L	***603.3712***	302.1892	586.3447	293.6760	585.3606	293.1840	5
9	**1114.6255**	557.8164	1097.5990	549.3031	1096.6150	548.8111	L	***490.2871***	245.6472	473.2606	237.1339	472.2766	236.6419	4
10	**1243.6681**	622.3377	1226.6416	613.8244	1225.6575	613.3324	E	***377.2031***	189.1052	360.1765	180.5919	359.1925	180.0999	3
11	1344.7158	672.8615	1327.6892	664.3483	1326.7052	663.8563	T	***248.1605***	124.5839	231.1339	116.0706	230.1499	115.5786	2
12							K	***147.1128***	74.0600	130.0863	65.5468			1

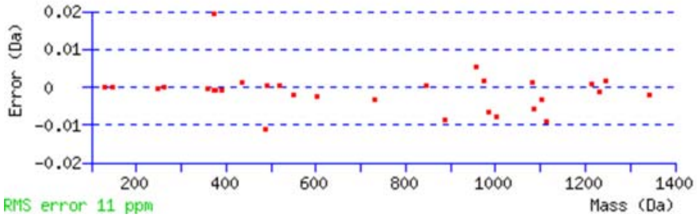

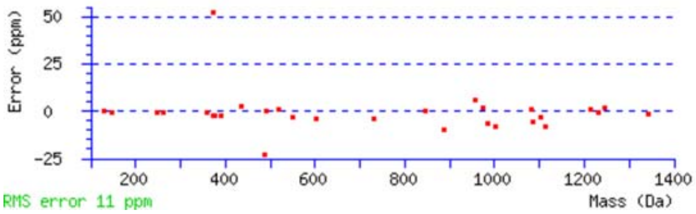

NCBI **BLAST** search of FLEQQNKLLETK
(Parameters: blastp, nr protein database, expect=20000, no filter, PAM30)
Other BLAST web gateways

All matches to this query

Score	Mr(calc)	Delta	Sequence
84.1	1489.8140	-0.0028	FLEQQNKLLETK

Mascot: http://www.matrixscience.com/

Mascot Search Results

Peptide View

MS/MS Fragmentation of **LGLDIEIATYRR**
Found in **ch12u_Q9NSB2|KRT84_HUMAN** in **uni_human**, Keratin, type II cuticular Hb4 OS=Homo sapiens GN=KRT84 PE=2 SV=2

Match to Query 15708: 1418.788572 from(473.936800,3+) intensity(9031432.0000) rtinseconds(2527) scans(13593) index(10742)
Title: 160219_Sunil_SDSII_A_Spectrum062071_scans__13593_RTINSECONDS=2527
Data file L:\\QE_2016\\160219_Sunil_KAP_LKC\\TMgf\\T\\T160219_Sunil_SDSII_A.mgf

Click mouse within plot area to zoom in by factor of two about that point
Or, Plot from 100 to 1400 Da Full range
Label all possible matches ○ Label matches used for scoring ◉
Show Y-axis ☐

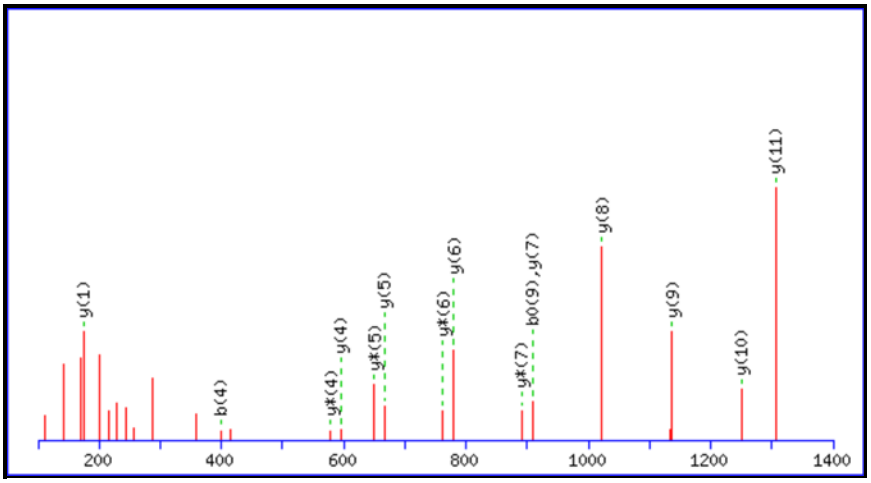

Monoisotopic mass of neutral peptide Mr(calc): 1418.7881
Fixed modifications: Carbamidomethyl (C) (apply to specified residues or termini only)
Ions Score: 77 **Expect:** 1.9e-006
Matches : 15/100 fragment ions using 20 most intense peaks (help)

#	b	b^{++}	b^*	b^{*++}	b^0	b^{0++}	Seq.	y	y^{++}	y^*	y^{*++}	y^0	y^{0++}	#
1	114.0913	57.5493					L							12
2	171.1128	86.0600					G	***1306.7114***	653.8593	1289.6848	645.3461	1288.7008	644.8540	11
3	284.1969	142.6021					L	***1249.6899***	625.3486	1232.6634	616.8353	1231.6793	616.3433	10
4	**399.2238**	200.1155			381.2132	191.1103	D	***1136.6058***	568.8066	1119.5793	560.2933	1118.5953	559.8013	9
5	512.3079	256.6576			494.2973	247.6523	I	***1021.5789***	511.2931	1004.5524	502.7798	1003.5683	502.2878	8
6	641.3505	321.1789			623.3399	312.1736	E	***908.4948***	454.7511	891.4683	446.2378	890.4843	445.7458	7
7	754.4345	377.7209			736.4240	368.7156	I	***779.4522***	390.2298	762.4257	381.7165	761.4417	381.2245	6
8	825.4716	413.2395			807.4611	404.2342	A	***666.3682***	333.6877	649.3416	325.1745	648.3576	324.6824	5
9	926.5193	463.7633			908.5088	454.7580	T	***595.3311***	298.1692	578.3045	289.6559	577.3205	289.1639	4
10	1089.5827	545.2950			1071.5721	536.2897	Y	494.2834	247.6453	477.2568	239.1321			3
11	1245.6838	623.3455	1228.6572	614.8322	1227.6732	614.3402	R	331.2201	166.1137	314.1935	157.6004			2
12							R	***175.1190***	88.0631	158.0924	79.5498			1

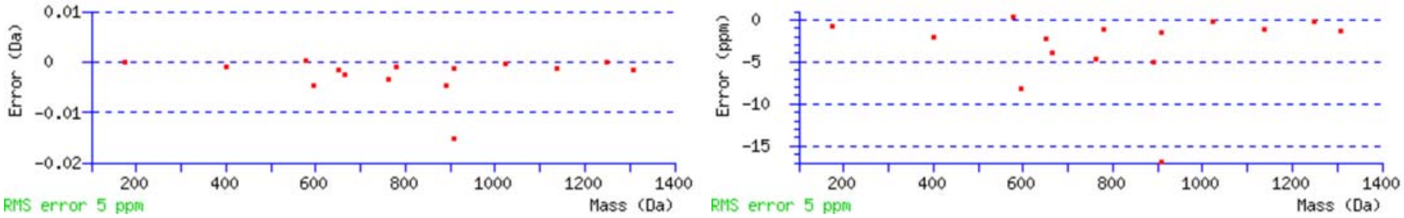

NCBI **BLAST** search of LGLDIEIATYRR
(Parameters: blastp, nr protein database, expect=20000, no filter, PAM30)
Other BLAST web gateways

All matches to this query

Score	Mr(calc)	Delta	Sequence
76.9	1418.7881	0.0004	LGLDIEIATYRR
17.2	1418.7881	0.0004	LGLLDRIVNYSR
16.4	1418.7769	0.0117	LLGNTFVALSDLR
8.6	1418.7881	0.0005	LQLRSLQYLER
4.1	1418.7769	0.0117	GLVLQLIQSYQR
1.5	1418.7994	-0.0108	LGPESVPPPKRSR
1.2	1418.7769	0.0117	LYVALKELGEER
0.6	1418.7769	0.0117	INELLKYIEER
0.1	1418.7881	0.0004	IKQFTLEEKQR
0.1	1418.7769	0.0117	LIQFLQASITER

Mascot: http://www.matrixscience.com/

Mascot Search Results

Peptide View

MS/MS Fragmentation of **FLEQQNKLLETK**
Found in **ch12u_Q9NSB2|KRT84_HUMAN** in **uni_human**, Keratin, type II cuticular Hb4 OS=Homo sapiens GN=KRT84 PE=2 SV=2

Match to Query 18174: 1489.811248 from(745.912900,2+) intensity(1430451.5000) rtinseconds(1473) scans(7784) index(20869)
Title: 160219_Sunil_SDSII_A_Spectrum073430_scans__7784_RTINSECONDS=1473
Data file L:\\QE_2016\\160219_Sunil_KAP_LKC\\TMgf\\T\\T160219_Sunil_SDSII_A.mgf

Click mouse within plot area to zoom in by factor of two about that point
Or, Plot from 100 to 1500 Da Full range
Label all possible matches ○ Label matches used for scoring ◉
Show Y-axis ☐

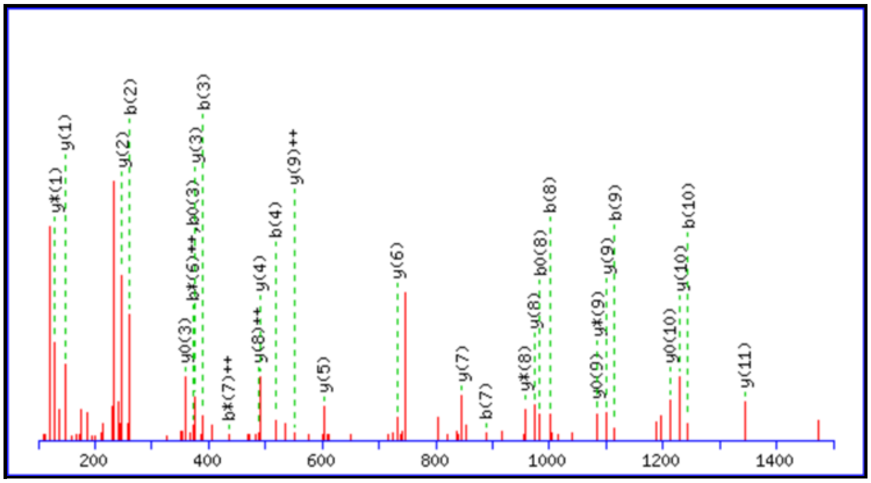

Monoisotopic mass of neutral peptide Mr(calc): 1489.8140
Fixed modifications: Carbamidomethyl (C) (apply to specified residues or termini only)
Ions Score: 84 **Expect:** 4.5e-007
Matches : 30/120 fragment ions using 48 most intense peaks (help)

#	b	b^{++}	b*	b^{*++}	b^0	b^{0++}	Seq.	y	y^{++}	y*	y^{*++}	y^0	y^{0++}	#
1	148.0757	74.5415					F							12
2	**261.1598**	131.0835					L	***1343.7529***	672.3801	1326.7264	663.8668	1325.7423	663.3748	11
3	**390.2023**	195.6048			372.1918	186.5995	E	***1230.6688***	615.8381	1213.6423	607.3248	1212.6583	606.8328	10
4	**518.2609**	259.6341	501.2344	251.1208	500.2504	250.6288	Q	***1101.6263***	551.3168	1084.5997	542.8035	1083.6157	542.3115	9
5	646.3195	323.6634	629.2930	315.1501	628.3089	314.6581	Q	***973.5677***	487.2875	956.5411	478.7742	955.5571	478.2822	8
6	760.3624	380.6849	743.3359	372.1716	742.3519	371.6796	N	***845.5091***	423.2582	828.4825	414.7449	827.4985	414.2529	7
7	**888.4574**	444.7323	871.4308	436.2191	870.4468	435.7271	K	***731.4662***	366.2367	714.4396	357.7234	713.4556	357.2314	6
8	**1001.5415**	501.2744	984.5149	492.7611	983.5309	492.2691	L	***603.3712***	302.1892	586.3447	293.6760	585.3606	293.1840	5
9	**1114.6255**	557.8164	1097.5990	549.3031	1096.6150	548.8111	L	***490.2871***	245.6472	473.2606	237.1339	472.2766	236.6419	4
10	**1243.6681**	622.3377	1226.6416	613.8244	1225.6575	613.3324	E	***377.2031***	189.1052	360.1765	180.5919	359.1925	180.0999	3
11	1344.7158	672.8615	1327.6892	664.3483	1326.7052	663.8563	T	***248.1605***	124.5839	231.1339	116.0706	230.1499	115.5786	2
12							K	***147.1128***	74.0600	130.0863	65.5468			1

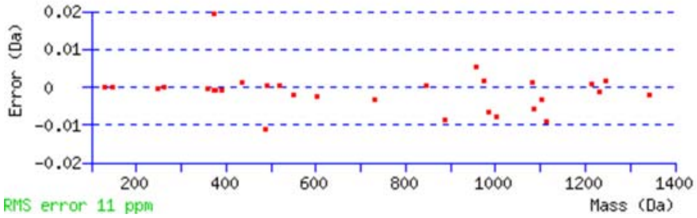

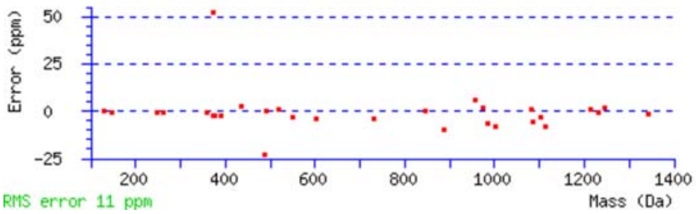

NCBI **BLAST** search of FLEQQNKLLETK
(Parameters: blastp, nr protein database, expect=20000, no filter, PAM30)
Other BLAST web gateways

All matches to this query

Score	Mr(calc)	Delta	Sequence
84.1	1489.8140	-0.0028	FLEQQNKLLETK

Mascot: http://www.matrixscience.com/

Mascot Search Results

Peptide View

MS/MS Fragmentation of **FLEQQNKLLETK**
Found in **ch12u_Q9NSB2|KRT84_HUMAN** in **uni_human**, Keratin, type II cuticular Hb4 OS=Homo sapiens GN=KRT84 PE=2 SV=2

Match to Query 18189: 1489.813902 from(497.611910,3+) intensity(56885288.0000) rtinseconds(1729) scans(8963) index(6747)
Title: 160219_Sunil_SDSII_A_Spectrum058070_scans__8963_RTINSECONDS=1729
Data file L:\\QE_2016\\160219_Sunil_KAP_LKC\\TMgf\\T\\T160219_Sunil_SDSII_A.mgf

Click mouse within plot area to zoom in by factor of two about that point
Or, Plot from 100 to 1400 Da Full range
Label all possible matches ○ Label matches used for scoring ◉
Show Y-axis ☐

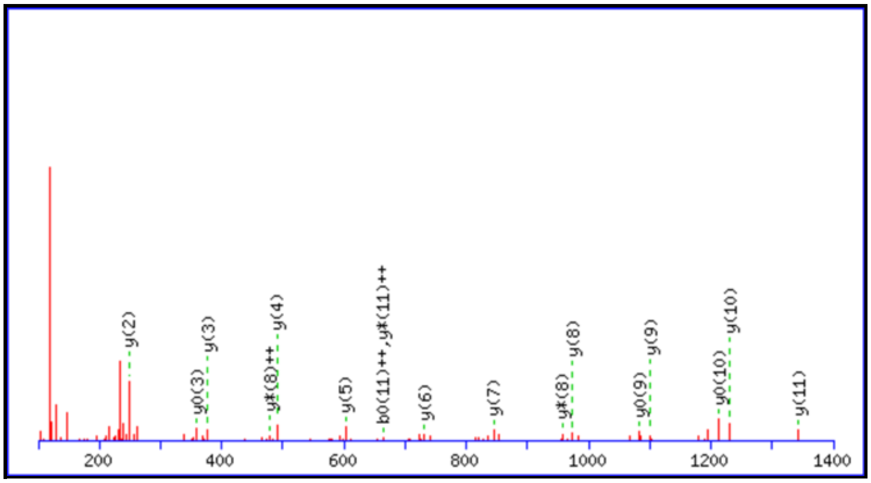

Monoisotopic mass of neutral peptide Mr(calc): 1489.8140
Fixed modifications: Carbamidomethyl (C) (apply to specified residues or termini only)
Ions Score: 76 **Expect:** 2.8e-006
Matches : 17/120 fragment ions using 25 most intense peaks (help)

#	b	b++	b*	b*++	b0	b0++	Seq.	y	y++	y*	y*++	y0	y0++	#
1	148.0757	74.5415					F							12
2	261.1598	131.0835					L	1343.7529	672.3801	1326.7264	663.8668	1325.7423	663.3748	11
3	390.2023	195.6048			372.1918	186.5995	E	1230.6688	615.8381	1213.6423	607.3248	1212.6583	606.8328	10
4	518.2609	259.6341	501.2344	251.1208	500.2504	250.6288	Q	1101.6263	551.3168	1084.5997	542.8035	1083.6157	542.3115	9
5	646.3195	323.6634	629.2930	315.1501	628.3089	314.6581	Q	973.5677	487.2875	956.5411	478.7742	955.5571	478.2822	8
6	760.3624	380.6849	743.3359	372.1716	742.3519	371.6796	N	845.5091	423.2582	828.4825	414.7449	827.4985	414.2529	7
7	888.4574	444.7323	871.4308	436.2191	870.4468	435.7271	K	731.4662	366.2367	714.4396	357.7234	713.4556	357.2314	6
8	1001.5415	501.2744	984.5149	492.7611	983.5309	492.2691	L	603.3712	302.1892	586.3447	293.6760	585.3606	293.1840	5
9	1114.6255	557.8164	1097.5990	549.3031	1096.6150	548.8111	L	490.2871	245.6472	473.2606	237.1339	472.2766	236.6419	4
10	1243.6681	622.3377	1226.6416	613.8244	1225.6575	613.3324	E	377.2031	189.1052	360.1765	180.5919	359.1925	180.0999	3
11	1344.7158	672.8615	1327.6892	664.3483	1326.7052	663.8563	T	248.1605	124.5839	231.1339	116.0706	230.1499	115.5786	2
12							K	147.1128	74.0600	130.0863	65.5468			1

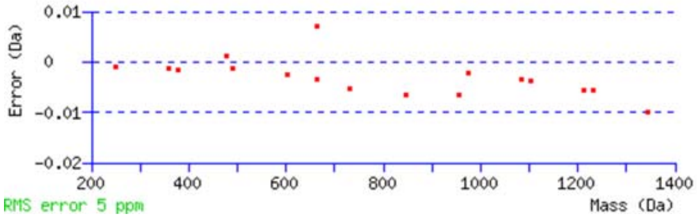

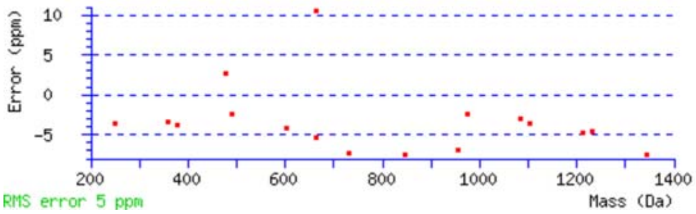

NCBI **BLAST** search of FLEQQNKLLETK
(Parameters: blastp, nr protein database, expect=20000, no filter, PAM30)
Other BLAST web gateways

All matches to this query

Score	Mr(calc)	Delta	Sequence
76.0	1489.8140	-0.0001	FLEQQNKLLETK
0.9	1487.8069	2.0070	NVHRPPRQRDIT

Mascot: http://www.matrixscience.com/

Mascot Search Results

Peptide View

MS/MS Fragmentation of **FLEQQNKLLETK**
Found in **ch12u_Q9NSB2|KRT84_HUMAN** in **uni_human**, Keratin, type II cuticular Hb4 OS=Homo sapiens GN=KRT84 PE=2 SV=2

Match to Query 18255: 1490.799788 from(746.407170,2+) intensity(3397827.2500) rtinseconds(1868) scans(9779) index(7472)
Title: 160219_Sunil_SDSII_A_Spectrum058796_scans__9779_RTINSECONDS=1868
Data file L:\\QE_2016\\160219_Sunil_KAP_LKC\\TMgf\\T\\T160219_Sunil_SDSII_A.mgf

Click mouse within plot area to zoom in by factor of two about that point
Or, Plot from 100 to 1500 Da Full range
Label all possible matches ○ Label matches used for scoring ◉
Show Y-axis □

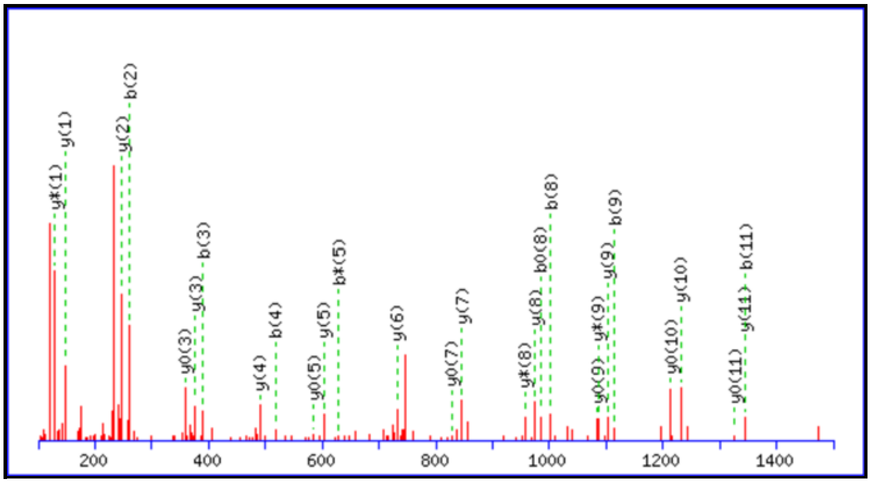

Monoisotopic mass of neutral peptide Mr(calc): 1490.7980
Fixed modifications: Carbamidomethyl (C) (apply to specified residues or termini only)
Variable modifications:
N6 : Deamidated (NQ)
Ions Score: 76 **Expect:** 2.8e-006
Matches : 28/120 fragment ions using 52 most intense peaks (help)

#	b	b^{++}	b*	b^{*++}	b^0	b^{0++}	Seq.	y	y^{++}	y*	y^{*++}	y^0	y^{0++}	#
1	148.0757	74.5415					F							12
2	261.1598	131.0835					L	1344.7369	672.8721	1327.7104	664.3588	1326.7264	663.8668	11
3	390.2023	195.6048			372.1918	186.5995	E	1231.6529	616.3301	1214.6263	607.8168	1213.6423	607.3248	10
4	518.2609	259.6341	501.2344	251.1208	500.2504	250.6288	Q	1102.6103	551.8088	1085.5837	543.2955	1084.5997	542.8035	9
5	646.3195	323.6634	629.2930	315.1501	628.3089	314.6581	Q	974.5517	487.7795	957.5251	479.2662	956.5411	478.7742	8
6	761.3464	381.1769	744.3199	372.6636	743.3359	372.1716	N	846.4931	423.7502	829.4666	415.2369	828.4825	414.7449	7
7	889.4414	445.2243	872.4149	436.7111	871.4308	436.2191	K	731.4662	366.2367	714.4396	357.7234	713.4556	357.2314	6
8	1002.5255	501.7664	985.4989	493.2531	984.5149	492.7611	L	603.3712	302.1892	586.3447	293.6760	585.3606	293.1840	5
9	1115.6095	558.3084	1098.5830	549.7951	1097.5990	549.3031	L	490.2871	245.6472	473.2606	237.1339	472.2766	236.6419	4
10	1244.6521	622.8297	1227.6256	614.3164	1226.6416	613.8244	E	377.2031	189.1052	360.1765	180.5919	359.1925	180.0999	3
11	1345.6998	673.3535	1328.6733	664.8403	1327.6892	664.3483	T	248.1605	124.5839	231.1339	116.0706	230.1499	115.5786	2
12							K	147.1128	74.0600	130.0863	65.5468			1

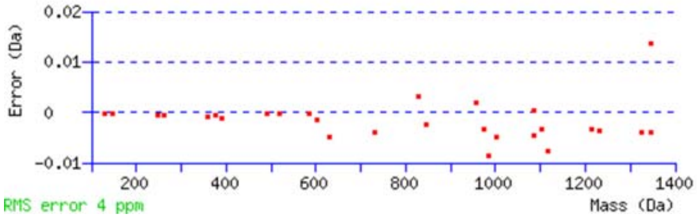

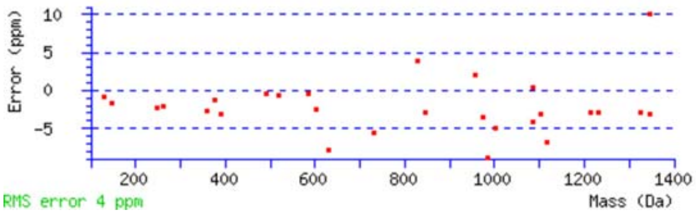

NCBI **BLAST** search of FLEQQNKLLETK
(Parameters: blastp, nr protein database, expect=20000, no filter, PAM30)
Other BLAST web gateways

All matches to this query

Score	Mr(calc)	Delta	Sequence	Site Analysis
76.4	1490.7980	0.0018	FLEQQNKLLETK	Deamidated N6 95.21%
63.3	1490.7980	0.0018	FLEQQNKLLETK	Deamidated Q5 4.63%
48.7	1490.7980	0.0018	FLEQQNKLLETK	Deamidated Q4 0.16%
16.7	1490.7981	0.0017	FQALQSVDILETK	

Mascot: http://www.matrixscience.com/

Mascot Search Results

Peptide View

MS/MS Fragmentation of **VRFLEQQNKLLETK**
Found in **ch12u_Q9NSB2|KRT84_HUMAN** in **uni_human**, Keratin, type II cuticular Hb4 OS=Homo sapiens GN=KRT84 PE=2 SV=2

Match to Query 24723: 1744.982982 from(582.668270,3+) intensity(1018731.4375) rtinseconds(1496) scans(7919) index(20989)
Title: 160219_Sunil_SDSII_A_Spectrum073550_scans__7919_RTINSECONDS=1496
Data file L:\\QE_2016\\160219_Sunil_KAP_LKC\\TMgf\\T\\T160219_Sunil_SDSII_A.mgf

Click mouse within plot area to zoom in by factor of two about that point
Or, Plot from 100 to 1800 Da Full range
Label all possible matches ○ Label matches used for scoring ◉
Show Y-axis ☐

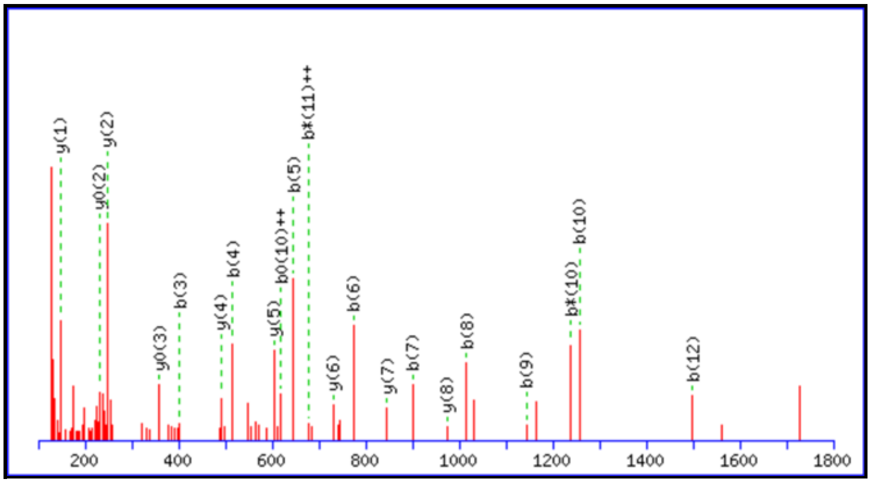

Monoisotopic mass of neutral peptide Mr(calc): 1744.9835
Fixed modifications: Carbamidomethyl (C) (apply to specified residues or termini only)
Ions Score: 78 **Expect:** 9.4e-007
Matches : 21/144 fragment ions using 26 most intense peaks (help)

#	b	b^{++}	b*	b^{*++}	b^0	b^{0++}	Seq.	y	y^{++}	y*	y^{*++}	y^0	y^{0++}	#
1	100.0757	50.5415					V							14
2	256.1768	128.5920	239.1503	120.0788			R	1646.9224	823.9649	1629.8959	815.4516	1628.9119	814.9596	13
3	***403.2452***	202.1262	386.2187	193.6130			F	1490.8213	745.9143	1473.7948	737.4010	1472.8108	736.9090	12
4	***516.3293***	258.6683	499.3027	250.1550			L	1343.7529	672.3801	1326.7264	663.8668	1325.7423	663.3748	11
5	***645.3719***	323.1896	628.3453	314.6763	627.3613	314.1843	E	1230.6688	615.8381	1213.6423	607.3248	1212.6583	606.8328	10
6	***773.4305***	387.2189	756.4039	378.7056	755.4199	378.2136	Q	1101.6263	551.3168	1084.5997	542.8035	1083.6157	542.3115	9
7	***901.4890***	451.2482	884.4625	442.7349	883.4785	442.2429	Q	**973.5677**	487.2875	956.5411	478.7742	955.5571	478.2822	8
8	***1015.5320***	508.2696	998.5054	499.7563	997.5214	499.2643	N	**845.5091**	423.2582	828.4825	414.7449	827.4985	414.2529	7
9	***1143.6269***	572.3171	1126.6004	563.8038	1125.6164	563.3118	K	**731.4662**	366.2367	714.4396	357.7234	713.4556	357.2314	6
10	***1256.7110***	628.8591	1239.6844	620.3459	1238.7004	619.8538	L	**603.3712**	302.1892	586.3447	293.6760	585.3606	293.1840	5
11	1369.7950	685.4012	1352.7685	676.8879	1351.7845	676.3959	L	**490.2871**	245.6472	473.2606	237.1339	472.2766	236.6419	4
12	***1498.8376***	749.9225	1481.8111	741.4092	1480.8271	740.9172	E	377.2031	189.1052	360.1765	180.5919	359.1925	180.0999	3
13	1599.8853	800.4463	1582.8588	791.9330	1581.8748	791.4410	T	**248.1605**	124.5839	231.1339	116.0706	230.1499	115.5786	2
14							K	**147.1128**	74.0600	130.0863	65.5468			1

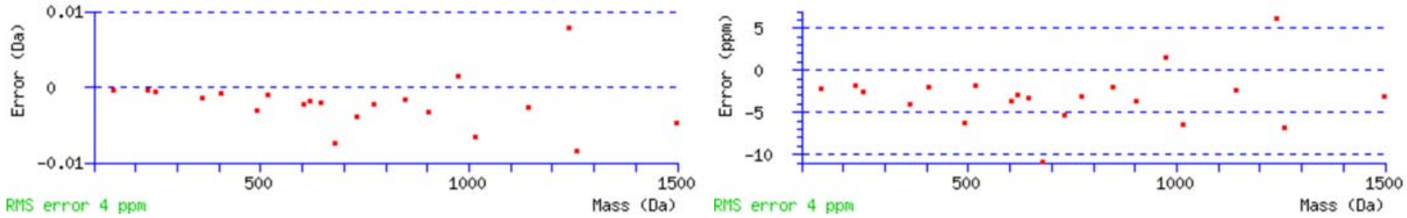

NCBI **BLAST** search of VRFLEQQNKLLETK
(Parameters: blastp, nr protein database, expect=20000, no filter, PAM30)
Other BLAST web gateways

All matches to this query

Score	Mr(calc)	Delta	Sequence
78.1	1744.9835	-0.0006	VRFLEQQNKLLETK
6.9	1743.9784	1.0046	KGFRLLNPHPKPNPK
0.0	1743.9659	1.0171	GSLTFEPLTLVPIQTK

Mascot: http://www.matrixscience.com/

Mascot Search Results

Peptide View

MS/MS Fragmentation of **FASFIDKVR**
Found in **ch12u_Q9NSB2|KRT84_HUMAN** in **uni_human**, Keratin, type II cuticular Hb4 OS=Homo sapiens GN=KRT84 PE=2 SV=2

Match to Query 4005: 1081.592142 from(361.537990,3+) intensity(224838.6250) rtinseconds(1614) scans(8660) index(6768)
Title: 160219_Sunil_KAP_A1_Spectrum057735_scans__8660_RTINSECONDS=1614
Data file L:\\QE_2016\\160219_Sunil_KAP_LKC\\TMgf\\T\\T160219_Sunil_KAP_A1.mgf

Click mouse within plot area to zoom in by factor of two about that point
Or, Plot from 50 to 950 Da Full range
Label all possible matches ○ Label matches used for scoring ◉
Show Y-axis ☐

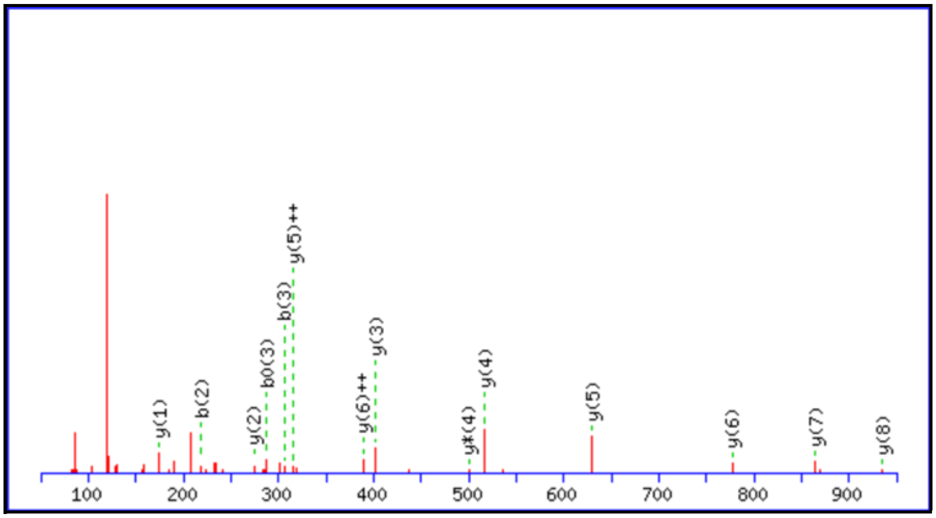

Monoisotopic mass of neutral peptide Mr(calc): 1081.5920
Fixed modifications: Carbamidomethyl (C) (apply to specified residues or termini only)
Ions Score: 52 **Expect:** 0.00045
Matches : 14/74 fragment ions using 29 most intense peaks (help)

#	b	b^{++}	b*	b^{*++}	b^0	b^{0++}	Seq.	y	y^{++}	y*	y^{*++}	y^0	y^{0++}	#
1	148.0757	74.5415					F							9
2	**219.1128**	110.0600					A	***935.5309***	468.2691	918.5043	459.7558	917.5203	459.2638	8
3	**306.1448**	153.5761			288.1343	144.5708	S	***864.4938***	432.7505	847.4672	424.2373	846.4832	423.7452	7
4	453.2132	227.1103			435.2027	218.1050	F	***777.4618***	389.2345	760.4352	380.7212	759.4512	380.2292	6
5	566.2973	283.6523			548.2867	274.6470	I	***630.3933***	315.7003	613.3668	307.1870	612.3828	306.6950	5
6	681.3243	341.1658			663.3137	332.1605	D	***517.3093***	259.1583	500.2827	250.6450	499.2987	250.1530	4
7	809.4192	405.2132	792.3927	396.7000	791.4087	396.2080	K	***402.2823***	201.6448	385.2558	193.1315			3
8	908.4876	454.7475	891.4611	446.2342	890.4771	445.7422	V	***274.1874***	137.5973	257.1608	129.0840			2
9							R	***175.1190***	88.0631	158.0924	79.5498			1

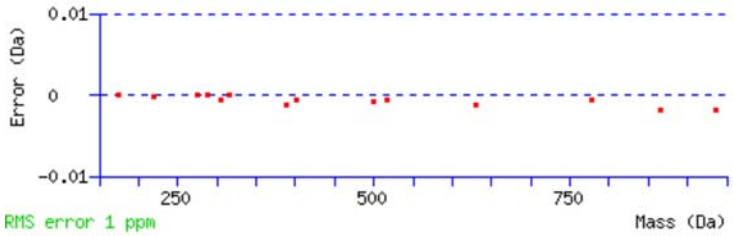

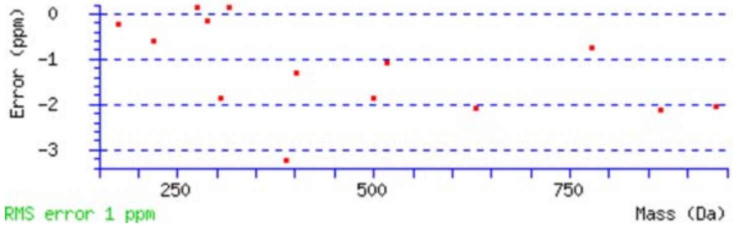

NCBI **BLAST** search of FASFIDKVR
(Parameters: blastp, nr protein database, expect=20000, no filter, PAM30)
Other BLAST web gateways

All matches to this query

Score	Mr(calc)	Delta	Sequence
51.6	1081.5920	0.0001	FASFIDKVR
51.6	1081.5920	0.0001	FASFINKVR
10.8	1079.5909	2.0012	RYMLKNVR
9.3	1081.5920	0.0001	AFSFVKDLR
3.5	1081.5992	-0.0071	LSDHLSVRR
3.3	1080.5927	0.9994	KVTYRNLST
3.1	1080.5815	1.0106	PEPQLQQLK

Mascot: http://www.matrixscience.com/

Mascot Search Results

Peptide View

MS/MS Fragmentation of **LGLDIEIATYRR**
Found in **ch12u_Q9NSB2|KRT84_HUMAN** in **uni_human**, Keratin, type II cuticular Hb4 OS=Homo sapiens GN=KRT84 PE=2 SV=2

Match to Query 13040: 1418.788392 from(473.936740,3+) intensity(561901.3125) rtinseconds(2205) scans(12190) index(24079)
Title: 160219_Sunil_KAP_A1_Spectrum076549_scans__12190_RTINSECONDS=2205
Data file L:\\QE_2016\\160219_Sunil_KAP_LKC\\TMgf\\T\\T160219_Sunil_KAP_A1.mgf

Click mouse within plot area to zoom in by factor of two about that point
Or, Plot from 100 to 1500 Da Full range
Label all possible matches ○ Label matches used for scoring ◉
Show Y-axis ☐

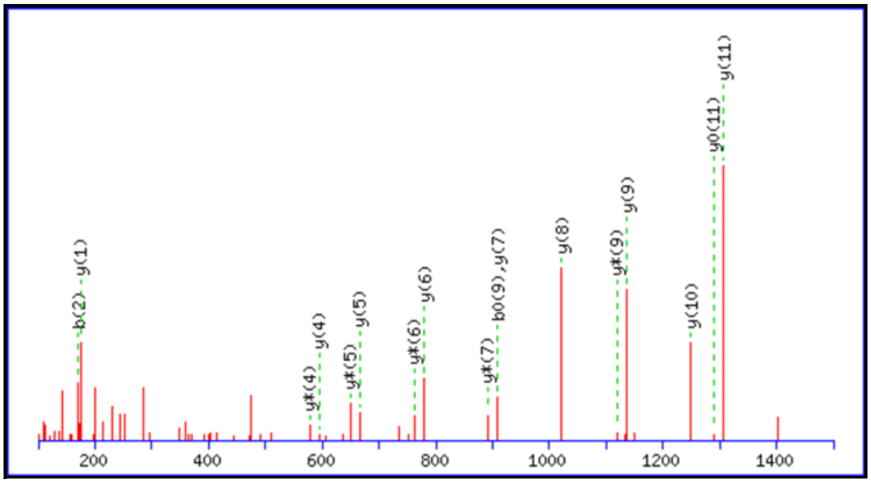

Monoisotopic mass of neutral peptide Mr(calc): 1418.7881
Fixed modifications: Carbamidomethyl (C) (apply to specified residues or termini only)
Ions Score: 63 **Expect:** 5.1e-005
Matches : 17/100 fragment ions using 32 most intense peaks (help)

#	b	b++	b*	b*++	b0	b0++	Seq.	y	y++	y*	y*++	y0	y0++	#
1	114.0913	57.5493					L							12
2	171.1128	86.0600					G	1306.7114	653.8593	1289.6848	645.3461	1288.7008	644.8540	11
3	284.1969	142.6021					L	1249.6899	625.3486	1232.6634	616.8353	1231.6793	616.3433	10
4	399.2238	200.1155			381.2132	191.1103	D	1136.6058	568.8066	1119.5793	560.2933	1118.5953	559.8013	9
5	512.3079	256.6576			494.2973	247.6523	I	1021.5789	511.2931	1004.5524	502.7798	1003.5683	502.2878	8
6	641.3505	321.1789			623.3399	312.1736	E	908.4948	454.7511	891.4683	446.2378	890.4843	445.7458	7
7	754.4345	377.7209			736.4240	368.7156	I	779.4522	390.2298	762.4257	381.7165	761.4417	381.2245	6
8	825.4716	413.2395			807.4611	404.2342	A	666.3682	333.6877	649.3416	325.1745	648.3576	324.6824	5
9	926.5193	463.7633			908.5088	454.7580	T	595.3311	298.1692	578.3045	289.6559	577.3205	289.1639	4
10	1089.5827	545.2950			1071.5721	536.2897	Y	494.2834	247.6453	477.2568	239.1321			3
11	1245.6838	623.3455	1228.6572	614.8322	1227.6732	614.3402	R	331.2201	166.1137	314.1935	157.6004			2
12							R	175.1190	88.0631	158.0924	79.5498			1

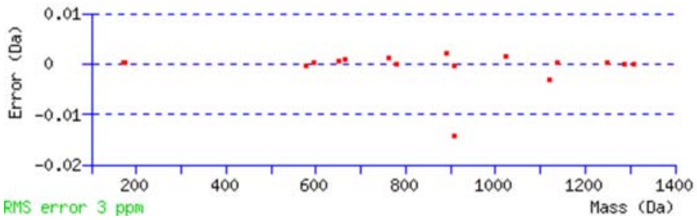

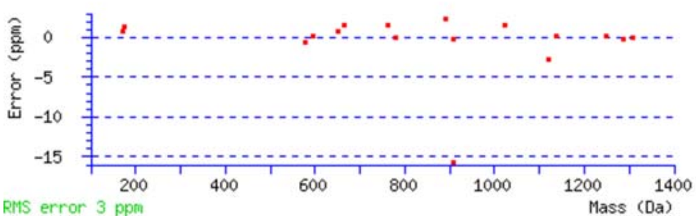

NCBI **BLAST** search of LGLDIEIATYRR
(Parameters: blastp, nr protein database, expect=20000, no filter, PAM30)
Other BLAST web gateways

All matches to this query

Score	Mr(calc)	Delta	Sequence
62.7	1418.7881	0.0003	LGLDIEIATYRR
23.9	1418.7881	0.0003	LGLLDRIVNYSR
12.9	1418.7769	0.0115	LLGNTFVALSDLR
7.3	1418.7881	0.0003	LQLRSLQYLER
5.4	1418.7769	0.0115	GLVLQLIQSYQR
3.6	1418.7769	0.0115	GLVLQLIQSYQR
2.4	1418.7742	0.0142	LGEARHPQVSLGR
2.4	1418.7994	-0.0110	LGPESVPPPKRSR

Mascot: http://www.matrixscience.com/

Mascot Search Results

Peptide View

MS/MS Fragmentation of **FLEQQNKLLETK**
Found in **ch12u_Q9NSB2|KRT84_HUMAN** in **uni_human**, Keratin, type II cuticular Hb4 OS=Homo sapiens GN=KRT84 PE=2 SV=2

Match to Query 15105: 1489.813208 from(745.913880,2+) intensity(344580.4063) rtinseconds(1370) scans(7390) index(20115)
Title: 160219_Sunil_KAP_A1_Spectrum072565_scans__7390_RTINSECONDS=1370
Data file L:\\QE_2016\\160219_Sunil_KAP_LKC\\TMgf\\T\\T160219_Sunil_KAP_A1.mgf

Click mouse within plot area to zoom in by factor of two about that point
Or, Plot from 100 to 1300 Da Full range
Label all possible matches ○ Label matches used for scoring ◉
Show Y-axis ☐

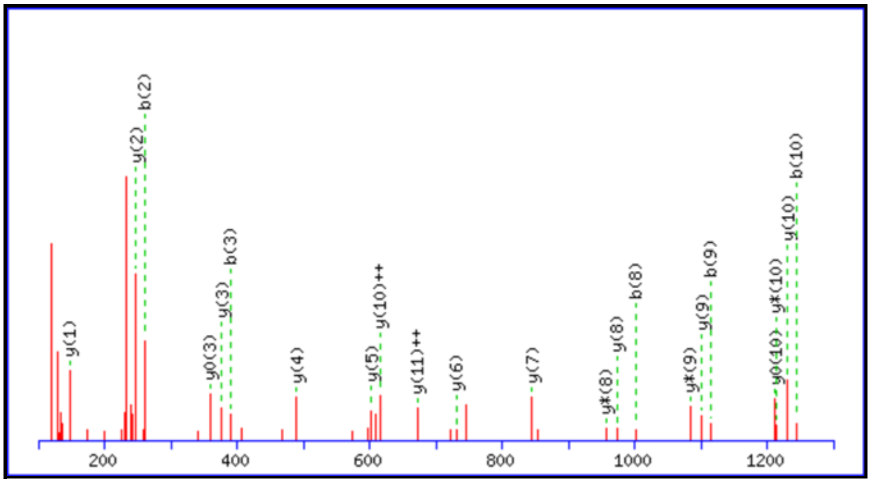

Monoisotopic mass of neutral peptide Mr(calc): 1489.8140
Fixed modifications: Carbamidomethyl (C) (apply to specified residues or termini only)
Ions Score: 83 **Expect:** 5.3e-007
Matches : 22/120 fragment ions using 30 most intense peaks (help)

#	b	b^{++}	b*	b^{*++}	b^0	b^{0++}	Seq.	y	y^{++}	y*	y^{*++}	y^0	y^{0++}	#
1	148.0757	74.5415					F							12
2	**261.1598**	131.0835					L	1343.7529	672.3801	1326.7264	663.8668	1325.7423	663.3748	11
3	**390.2023**	195.6048			372.1918	186.5995	E	***1230.6688***	615.8381	1213.6423	607.3248	1212.6583	606.8328	10
4	518.2609	259.6341	501.2344	251.1208	500.2504	250.6288	Q	***1101.6263***	551.3168	1084.5997	542.8035	1083.6157	542.3115	9
5	646.3195	323.6634	629.2930	315.1501	628.3089	314.6581	Q	***973.5677***	487.2875	956.5411	478.7742	955.5571	478.2822	8
6	760.3624	380.6849	743.3359	372.1716	742.3519	371.6796	N	***845.5091***	423.2582	828.4825	414.7449	827.4985	414.2529	7
7	888.4574	444.7323	871.4308	436.2191	870.4468	435.7271	K	***731.4662***	366.2367	714.4396	357.7234	713.4556	357.2314	6
8	**1001.5415**	501.2744	984.5149	492.7611	983.5309	492.2691	L	***603.3712***	302.1892	586.3447	293.6760	585.3606	293.1840	5
9	**1114.6255**	557.8164	1097.5990	549.3031	1096.6150	548.8111	L	***490.2871***	245.6472	473.2606	237.1339	472.2766	236.6419	4
10	**1243.6681**	622.3377	1226.6416	613.8244	1225.6575	613.3324	E	***377.2031***	189.1052	360.1765	180.5919	359.1925	180.0999	3
11	1344.7158	672.8615	1327.6892	664.3483	1326.7052	663.8563	T	***248.1605***	124.5839	231.1339	116.0706	230.1499	115.5786	2
12							K	***147.1128***	74.0600	130.0863	65.5468			1

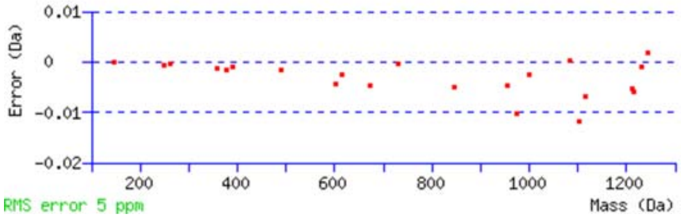

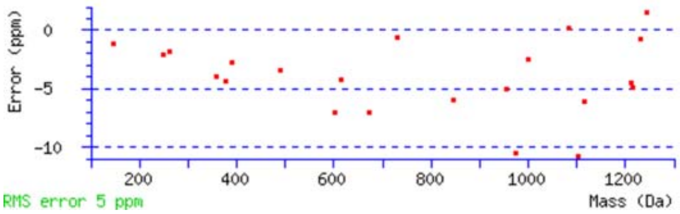

NCBI **BLAST** search of FLEQQNKLLETK
(Parameters: blastp, nr protein database, expect=20000, no filter, PAM30)
Other BLAST web gateways

All matches to this query

Score	Mr(calc)	Delta	Sequence
83.4	1489.8140	-0.0008	FLEQQNKLLETK

Mascot: http://www.matrixscience.com/

Mascot Search Results

Peptide View

MS/MS Fragmentation of **FLEQQNKLLETK**
Found in **ch12u_Q9NSB2|KRT84_HUMAN** in **uni_human**, Keratin, type II cuticular Hb4 OS=Homo sapiens GN=KRT84 PE=2 SV=2

Match to Query 15117: 1489.814668 from(745.914610,2+) intensity(567330.1875) rtinseconds(1348) scans(7210) index(34213)
Title: 160219_Sunil_KAP_A1_Spectrum088470_scans__7210_RTINSECONDS=1348
Data file L:\\QE_2016\\160219_Sunil_KAP_LKC\\TMgf\\T\\T160219_Sunil_KAP_A1.mgf

Click mouse within plot area to zoom in by factor of two about that point
Or, Plot from 100 to 1300 Da Full range
Label all possible matches ○ Label matches used for scoring ◉
Show Y-axis □

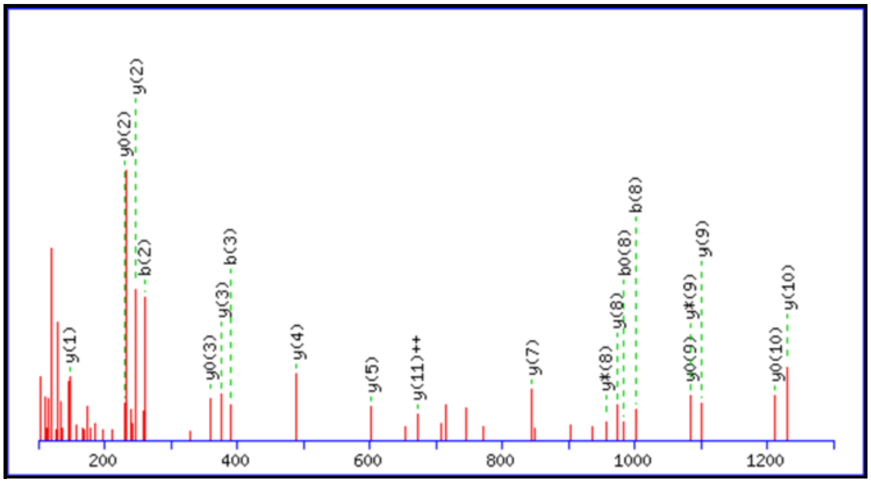

Monoisotopic mass of neutral peptide Mr(calc): 1489.8140
Fixed modifications: Carbamidomethyl (C) (apply to specified residues or termini only)
Ions Score: 63 **Expect:** 5.2e-005
Matches : 20/120 fragment ions using 33 most intense peaks (help)

#	b	b^{++}	b*	b^{*++}	b^0	b^{0++}	Seq.	y	y^{++}	y*	y^{*++}	y^0	y^{0++}	#
1	148.0757	74.5415					F							12
2	**261.1598**	131.0835					L	1343.7529	672.3801	1326.7264	663.8668	1325.7423	663.3748	11
3	**390.2023**	195.6048			372.1918	186.5995	E	***1230.6688***	615.8381	1213.6423	607.3248	1212.6583	606.8328	10
4	518.2609	259.6341	501.2344	251.1208	500.2504	250.6288	Q	***1101.6263***	551.3168	1084.5997	542.8035	1083.6157	542.3115	9
5	646.3195	323.6634	629.2930	315.1501	628.3089	314.6581	Q	***973.5677***	487.2875	956.5411	478.7742	955.5571	478.2822	8
6	760.3624	380.6849	743.3359	372.1716	742.3519	371.6796	N	***845.5091***	423.2582	828.4825	414.7449	827.4985	414.2529	7
7	888.4574	444.7323	871.4308	436.2191	870.4468	435.7271	K	731.4662	366.2367	714.4396	357.7234	713.4556	357.2314	6
8	**1001.5415**	501.2744	984.5149	492.7611	983.5309	492.2691	L	***603.3712***	302.1892	586.3447	293.6760	585.3606	293.1840	5
9	1114.6255	557.8164	1097.5990	549.3031	1096.6150	548.8111	L	***490.2871***	245.6472	473.2606	237.1339	472.2766	236.6419	4
10	1243.6681	622.3377	1226.6416	613.8244	1225.6575	613.3324	E	***377.2031***	189.1052	360.1765	180.5919	359.1925	180.0999	3
11	1344.7158	672.8615	1327.6892	664.3483	1326.7052	663.8563	T	***248.1605***	124.5839	231.1339	116.0706	230.1499	115.5786	2
12							K	***147.1128***	74.0600	130.0863	65.5468			1

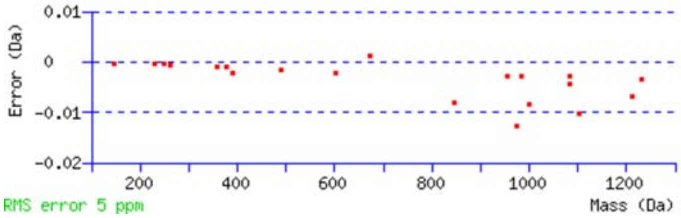

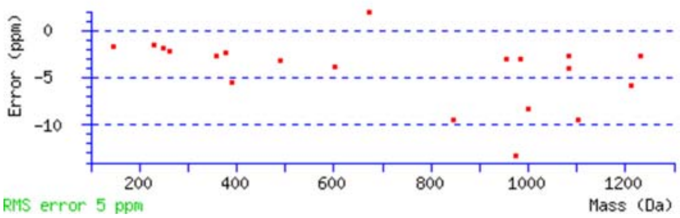

NCBI **BLAST** search of FLEQQNKLLETK
(Parameters: blastp, nr protein database, expect=20000, no filter, PAM30)
Other BLAST web gateways

All matches to this query

Score	Mr(calc)	Delta	Sequence
63.4	1489.8140	0.0007	FLEQQNKLLETK

Mascot: http://www.matrixscience.com/

Mascot Search Results

Peptide View

MS/MS Fragmentation of **FASFIDKVR**
Found in **ch12u_Q9NSB2|KRT84_HUMAN** in **uni_human**, Keratin, type II cuticular Hb4 OS=Homo sapiens GN=KRT84 PE=2 SV=2

Match to Query 4004: 1081.592142 from(361.537990,3+) intensity(141545.2500) rtinseconds(1510) scans(8197) index(20835)
Title: 160219_Sunil_KAP_A1_Spectrum073288_scans__8197_RTINSECONDS=1510
Data file L:\\QE_2016\\160219_Sunil_KAP_LKC\\TMgf\\T\\T160219_Sunil_KAP_A1.mgf

Click mouse within plot area to zoom in by factor of two about that point
Or, Plot from 50 to 900 Da Full range
Label all possible matches ○ Label matches used for scoring ◉
Show Y-axis ☐

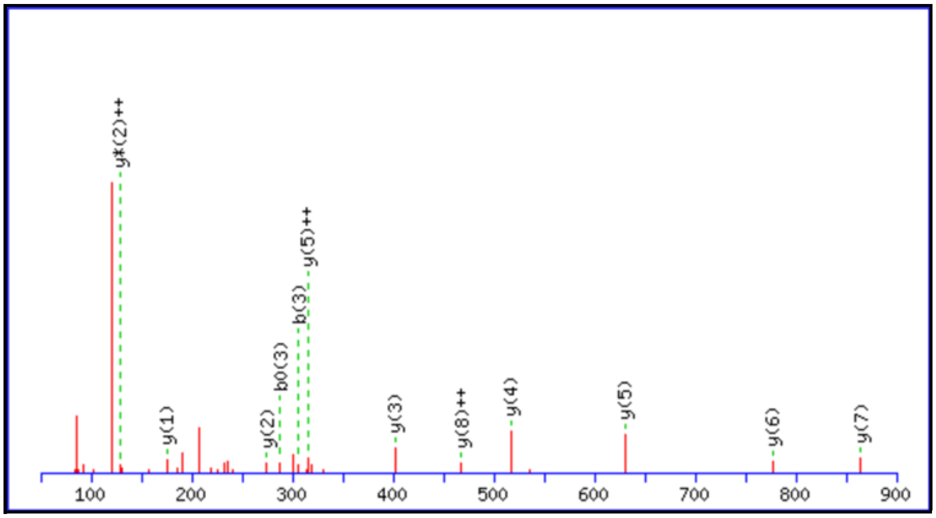

Monoisotopic mass of neutral peptide Mr(calc): 1081.5920
Fixed modifications: Carbamidomethyl (C) (apply to specified residues or termini only)
Ions Score: 47 **Expect:** 0.0014
Matches : 12/74 fragment ions using 23 most intense peaks (help)

#	b	b^{++}	b^{*}	b^{*++}	b^{0}	b^{0++}	Seq.	y	y^{++}	y^{*}	y^{*++}	y^{0}	y^{0++}	#
1	148.0757	74.5415					F							9
2	219.1128	110.0600					A	935.5309	468.2691	918.5043	459.7558	917.5203	459.2638	8
3	**306.1448**	153.5761			288.1343	144.5708	S	***864.4938***	432.7505	847.4672	424.2373	846.4832	423.7452	7
4	453.2132	227.1103			435.2027	218.1050	F	***777.4618***	389.2345	760.4352	380.7212	759.4512	380.2292	6
5	566.2973	283.6523			548.2867	274.6470	I	***630.3933***	315.7003	613.3668	307.1870	612.3828	306.6950	5
6	681.3243	341.1658			663.3137	332.1605	D	***517.3093***	259.1583	500.2827	250.6450	499.2987	250.1530	4
7	809.4192	405.2132	792.3927	396.7000	791.4087	396.2080	K	***402.2823***	201.6448	385.2558	193.1315			3
8	908.4876	454.7475	891.4611	446.2342	890.4771	445.7422	V	***274.1874***	137.5973	257.1608	129.0840			2
9							R	***175.1190***	88.0631	158.0924	79.5498			1

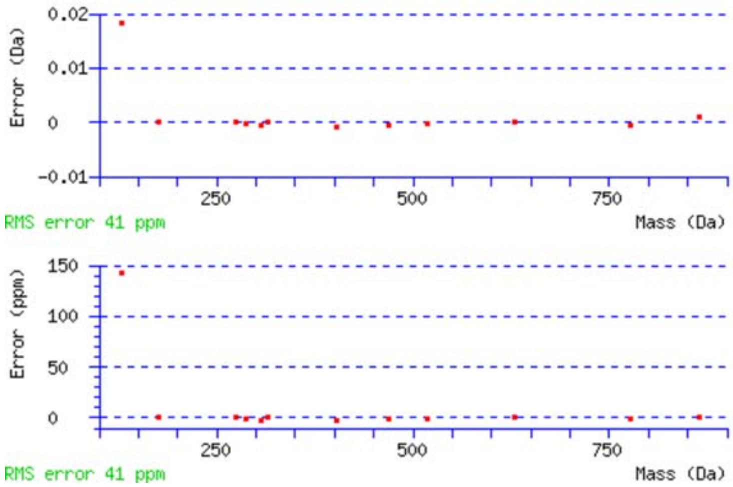

NCBI **BLAST** search of FASFIDKVR
(Parameters: blastp, nr protein database, expect=20000, no filter, PAM30)
Other BLAST web gateways

All matches to this query

Score	Mr(calc)	Delta	Sequence
46.9	1081.5920	0.0001	FASFIDKVR
46.9	1081.5920	0.0001	FASFINKVR
11.3	1081.5920	0.0001	AFSFVKDLR
6.9	1079.5909	2.0012	RYMLKNVR
3.1	1080.5927	0.9994	KVTYRNLST

Mascot: http://www.matrixscience.com/

Mascot Search Results

Peptide View

MS/MS Fragmentation of **FASFIDKVR**
Found in **ch12u_Q9NSB2|KRT84_HUMAN** in **uni_human**, Keratin, type II cuticular Hb4 OS=Homo sapiens GN=KRT84 PE=2 SV=2

Match to Query 4259: 1081.591512 from(361.537780,3+) intensity(190555.5625) rtinseconds(1967) scans(10565) index(8087)
Title: 160219_Sunil_KAP_B_Spectrum057218_scans__10565_RTINSECONDS=1967
Data file L:\\QE_2016\\160219_Sunil_KAP_LKC\\TMgf\\T\\T160219_Sunil_KAP_B.mgf

Click mouse within plot area to zoom in by factor of two about that point
Or, Plot from 50 to 950 Da Full range
Label all possible matches ○ Label matches used for scoring ◉
Show Y-axis ☐

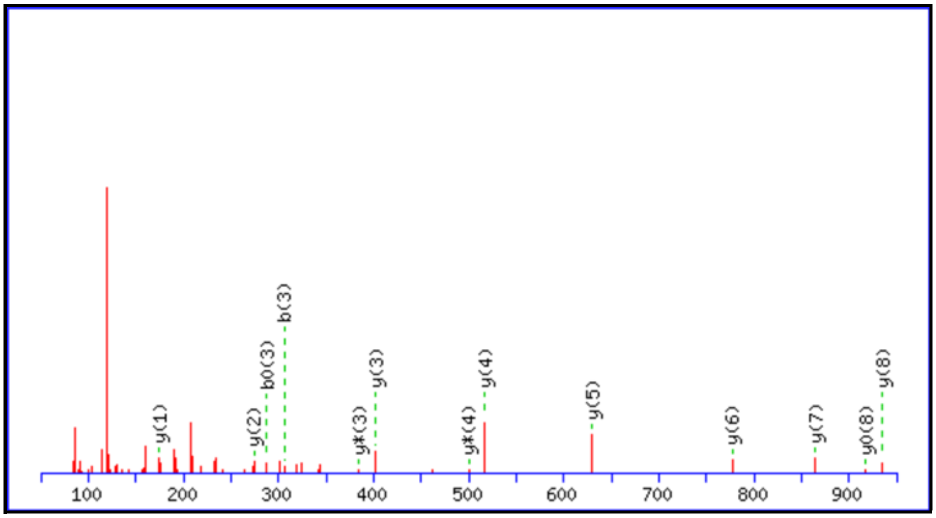

Monoisotopic mass of neutral peptide Mr(calc): 1081.5920
Fixed modifications: Carbamidomethyl (C) (apply to specified residues or termini only)
Ions Score: 52 **Expect:** 0.00042
Matches : 13/74 fragment ions using 31 most intense peaks (help)

#	b	b^{++}	b*	b^{*++}	b^0	b^{0++}	Seq.	y	y^{++}	y*	y^{*++}	y^0	y^{0++}	#
1	148.0757	74.5415					F							9
2	219.1128	110.0600					A	***935.5309***	468.2691	918.5043	459.7558	917.5203	459.2638	8
3	**306.1448**	153.5761			288.1343	144.5708	S	***864.4938***	432.7505	847.4672	424.2373	846.4832	423.7452	7
4	453.2132	227.1103			435.2027	218.1050	F	***777.4618***	389.2345	760.4352	380.7212	759.4512	380.2292	6
5	566.2973	283.6523			548.2867	274.6470	I	***630.3933***	315.7003	613.3668	307.1870	612.3828	306.6950	5
6	681.3243	341.1658			663.3137	332.1605	D	***517.3093***	259.1583	500.2827	250.6450	499.2987	250.1530	4
7	809.4192	405.2132	792.3927	396.7000	791.4087	396.2080	K	***402.2823***	201.6448	385.2558	193.1315			3
8	908.4876	454.7475	891.4611	446.2342	890.4771	445.7422	V	***274.1874***	137.5973	257.1608	129.0840			2
9							R	***175.1190***	88.0631	158.0924	79.5498			1

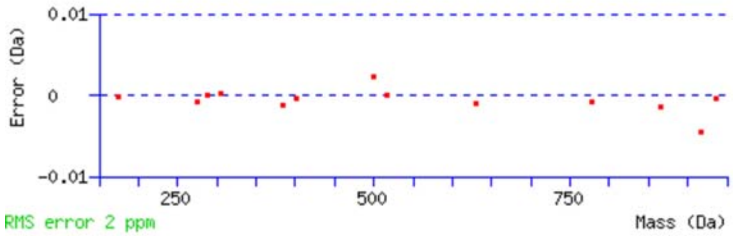

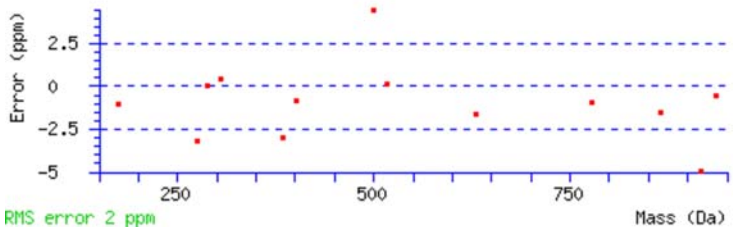

NCBI **BLAST** search of FASFIDKVR
(Parameters: blastp, nr protein database, expect=20000, no filter, PAM30)
Other BLAST web gateways

All matches to this query

Score	Mr(calc)	Delta	Sequence
52.1	1081.5920	-0.0005	FASFIDKVR
52.1	1081.5920	-0.0005	FASFINKVR
5.1	1081.5920	-0.0005	AFSFVKDLR
2.7	1079.5909	2.0006	RYMLKNVR
2.1	1080.5927	0.9988	KVTYRNLST

Mascot: http://www.matrixscience.com/

Mascot Search Results

Peptide View

MS/MS Fragmentation of **LGLDIEIATYR**
Found in **ch12u_Q9NSB2|KRT84_HUMAN** in **uni_human**, Keratin, type II cuticular Hb4 OS=Homo sapiens GN=KRT84 PE=2 SV=2

Match to Query 9395: 1262.686488 from(632.350520,2+) intensity(101573.7656) rtinseconds(2769) scans(15005) index(26079)
Title: 160219_Sunil_KAP_B_Spectrum076015_scans__15005_RTINSECONDS=2769
Data file L:\\QE_2016\\160219_Sunil_KAP_LKC\\TMgf\\T\\T160219_Sunil_KAP_B.mgf

Click mouse within plot area to zoom in by factor of two about that point
Or, Plot from 100 to 1200 Da Full range
Label all possible matches ○ Label matches used for scoring ◉
Show Y-axis ☐

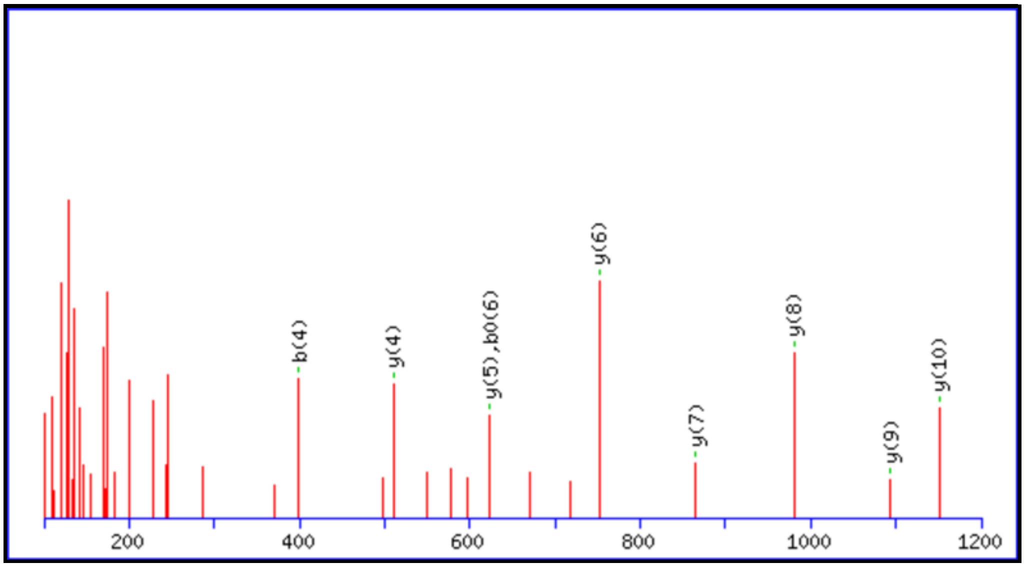

Monoisotopic mass of neutral peptide Mr(calc): 1262.6870
Fixed modifications: Carbamidomethyl (C) (apply to specified residues or termini only)
Ions Score: 57 **Expect:** 0.00022
Matches : 9/90 fragment ions using 11 most intense peaks (help)

#	b	b^{++}	b^{0}	b^{0++}	Seq.	y	y^{++}	y*	y^{*++}	y^{0}	y^{0++}	#
1	114.0913	57.5493			L							11
2	171.1128	86.0600			G	***1150.6103***	575.8088	1133.5837	567.2955	1132.5997	566.8035	10
3	284.1969	142.6021			L	***1093.5888***	547.2980	1076.5623	538.7848	1075.5782	538.2928	9
4	**399.2238**	200.1155	381.2132	191.1103	D	***980.5047***	490.7560	963.4782	482.2427	962.4942	481.7507	8
5	512.3079	256.6576	494.2973	247.6523	I	***865.4778***	433.2425	848.4512	424.7293	847.4672	424.2373	7
6	641.3505	321.1789	623.3399	312.1736	E	***752.3937***	376.7005	735.3672	368.1872	734.3832	367.6952	6
7	754.4345	377.7209	736.4240	368.7156	I	***623.3511***	312.1792	606.3246	303.6659	605.3406	303.1739	5
8	825.4716	413.2395	807.4611	404.2342	A	***510.2671***	255.6372	493.2405	247.1239	492.2565	246.6319	4
9	926.5193	463.7633	908.5088	454.7580	T	439.2300	220.1186	422.2034	211.6053	421.2194	211.1133	3

10	1089.5827	545.2950	1071.5721	536.2897	Y	338.1823	169.5948	321.1557	161.0815			2
11					R	175.1190	88.0631	158.0924	79.5498			1

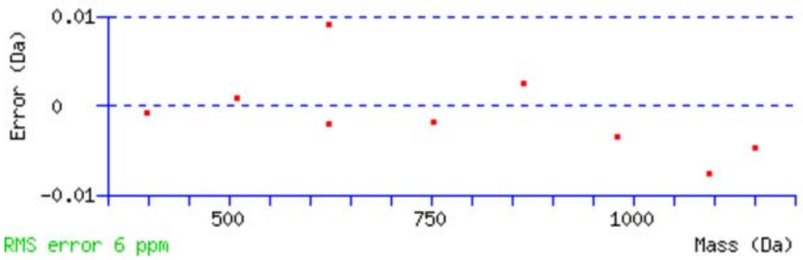

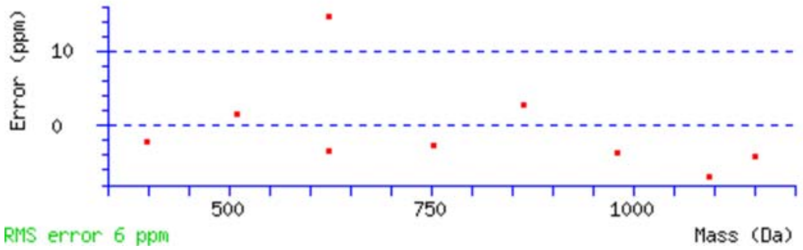

NCBI **BLAST** search of LGLDIEIATYR
(Parameters: blastp, nr protein database, expect=20000, no filter, PAM30)
Other BLAST web gateways

All matches to this query

Score	Mr(calc)	Delta	Sequence
56.9	1262.6870	-0.0005	LGLDIEIATYR
19.4	1262.6870	-0.0006	IAVDELFTSLR
13.2	1262.6870	-0.0005	LALDVEIATYR
9.3	1262.6983	-0.0118	LVAAKAFTESAR
7.1	1260.6687	2.0178	APLPHADASRAR

Mascot: http://www.matrixscience.com/

Mascot Search Results

Peptide View

MS/MS Fragmentation of **FLEQQNKLLETK**
Found in **ch12u_Q9NSB2|KRT84_HUMAN** in **uni_human**, Keratin, type II cuticular Hb4 OS=Homo sapiens GN=KRT84 PE=2 SV=2

Match to Query 15377: 1489.814442 from(497.612090,3+) intensity(571680.9375) rtinseconds(1610) scans(8374) index(20484)
Title: 160219_Sunil_KAP_B_Spectrum070366_scans__8374_RTINSECONDS=1610
Data file L:\\QE_2016\\160219_Sunil_KAP_LKC\\TMgf\\T\\T160219_Sunil_KAP_B.mgf

Click mouse within plot area to zoom in by factor of two about that point
Or, Plot from 100 to 1400 Da Full range
Label all possible matches ○ Label matches used for scoring ◉
Show Y-axis ☐

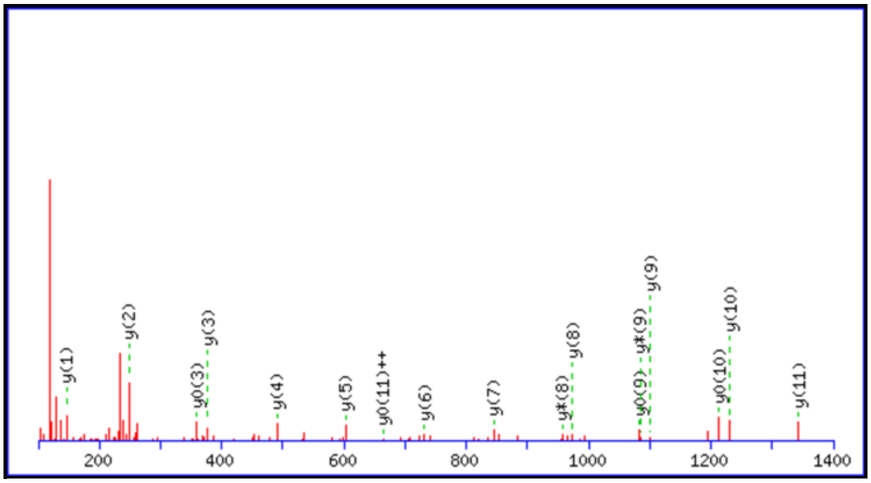

Monoisotopic mass of neutral peptide Mr(calc): 1489.8140
Fixed modifications: Carbamidomethyl (C) (apply to specified residues or termini only)
Ions Score: 76 **Expect:** 2.6e-006
Matches : 17/120 fragment ions using 34 most intense peaks (help)

#	b	b++	b*	b*++	b0	b0++	Seq.	y	y++	y*	y*++	y0	y0++	#
1	148.0757	74.5415					F							12
2	261.1598	131.0835					L	1343.7529	672.3801	1326.7264	663.8668	1325.7423	663.3748	11
3	390.2023	195.6048			372.1918	186.5995	E	1230.6688	615.8381	1213.6423	607.3248	1212.6583	606.8328	10
4	518.2609	259.6341	501.2344	251.1208	500.2504	250.6288	Q	1101.6263	551.3168	1084.5997	542.8035	1083.6157	542.3115	9
5	646.3195	323.6634	629.2930	315.1501	628.3089	314.6581	Q	973.5677	487.2875	956.5411	478.7742	955.5571	478.2822	8
6	760.3624	380.6849	743.3359	372.1716	742.3519	371.6796	N	845.5091	423.2582	828.4825	414.7449	827.4985	414.2529	7
7	888.4574	444.7323	871.4308	436.2191	870.4468	435.7271	K	731.4662	366.2367	714.4396	357.7234	713.4556	357.2314	6
8	1001.5415	501.2744	984.5149	492.7611	983.5309	492.2691	L	603.3712	302.1892	586.3447	293.6760	585.3606	293.1840	5
9	1114.6255	557.8164	1097.5990	549.3031	1096.6150	548.8111	L	490.2871	245.6472	473.2606	237.1339	472.2766	236.6419	4
10	1243.6681	622.3377	1226.6416	613.8244	1225.6575	613.3324	E	377.2031	189.1052	360.1765	180.5919	359.1925	180.0999	3
11	1344.7158	672.8615	1327.6892	664.3483	1326.7052	663.8563	T	248.1605	124.5839	231.1339	116.0706	230.1499	115.5786	2
12							K	147.1128	74.0600	130.0863	65.5468			1

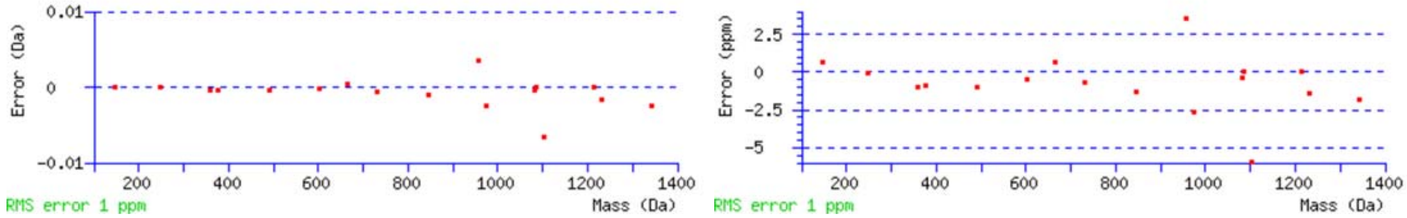

NCBI **BLAST** search of FLEQQNKLLETK
(Parameters: blastp, nr protein database, expect=20000, no filter, PAM30)
Other BLAST web gateways

All matches to this query

Score	Mr(calc)	Delta	Sequence
76.4	1489.8140	0.0004	FLEQQNKLLETK
0.7	1487.8069	2.0075	NVHRPPRQRDIT

Mascot: http://www.matrixscience.com/

Mascot Search Results

Peptide View

MS/MS Fragmentation of **FLEQQNKLLETK**
Found in **ch12u_Q9NSB2|KRT84_HUMAN** in **uni_human**, Keratin, type II cuticular Hb4 OS=Homo sapiens GN=KRT84 PE=2 SV=2

Match to Query 15374: 1489.813812 from(497.611880,3+) intensity(388909.9688) rtinseconds(1596) scans(8282) index(34503)
Title: 160219_Sunil_KAP_B_Spectrum085748_scans__8282_RTINSECONDS=1596
Data file L:\\QE_2016\\160219_Sunil_KAP_LKC\\TMgf\\T\\T160219_Sunil_KAP_B.mgf

Click mouse within plot area to zoom in by factor of two about that point
Or, Plot from 100 to 1400 Da Full range
Label all possible matches ○ Label matches used for scoring ◉
Show Y-axis ☐

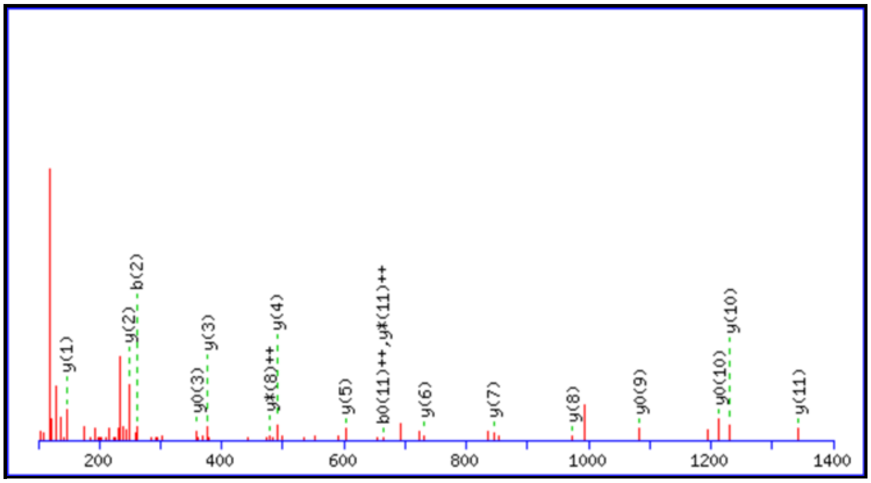

Monoisotopic mass of neutral peptide Mr(calc): 1489.8140
Fixed modifications: Carbamidomethyl (C) (apply to specified residues or termini only)
Ions Score: 66 **Expect:** 2.7e-005
Matches : 17/120 fragment ions using 30 most intense peaks (help)

#	b	b^{++}	b*	b^{*++}	b^0	b^{0++}	Seq.	y	y^{++}	y*	y^{*++}	y^0	y^{0++}	#
1	148.0757	74.5415					F							12
2	**261.1598**	131.0835					L	***1343.7529***	672.3801	1326.7264	663.8668	1325.7423	663.3748	11
3	390.2023	195.6048			372.1918	186.5995	E	***1230.6688***	615.8381	1213.6423	607.3248	1212.6583	606.8328	10
4	518.2609	259.6341	501.2344	251.1208	500.2504	250.6288	Q	1101.6263	551.3168	1084.5997	542.8035	1083.6157	542.3115	9
5	646.3195	323.6634	629.2930	315.1501	628.3089	314.6581	Q	***973.5677***	487.2875	956.5411	478.7742	955.5571	478.2822	8
6	760.3624	380.6849	743.3359	372.1716	742.3519	371.6796	N	***845.5091***	423.2582	828.4825	414.7449	827.4985	414.2529	7
7	888.4574	444.7323	871.4308	436.2191	870.4468	435.7271	K	***731.4662***	366.2367	714.4396	357.7234	713.4556	357.2314	6
8	1001.5415	501.2744	984.5149	492.7611	983.5309	492.2691	L	***603.3712***	302.1892	586.3447	293.6760	585.3606	293.1840	5
9	1114.6255	557.8164	1097.5990	549.3031	1096.6150	548.8111	L	***490.2871***	245.6472	473.2606	237.1339	472.2766	236.6419	4
10	1243.6681	622.3377	1226.6416	613.8244	1225.6575	613.3324	E	***377.2031***	189.1052	360.1765	180.5919	359.1925	180.0999	3
11	1344.7158	672.8615	1327.6892	664.3483	1326.7052	663.8563	T	***248.1605***	124.5839	231.1339	116.0706	230.1499	115.5786	2
12							K	***147.1128***	74.0600	130.0863	65.5468			1

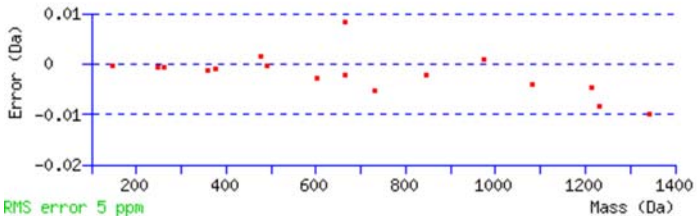

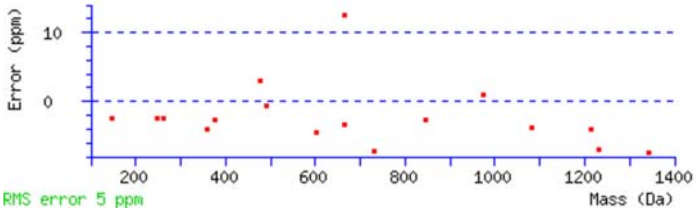

NCBI **BLAST** search of FLEQQNKLLETK
(Parameters: blastp, nr protein database, expect=20000, no filter, PAM30)
Other BLAST web gateways

All matches to this query

Score	Mr(calc)	Delta	Sequence
66.2	1489.8140	-0.0002	FLEQQNKLLETK
0.6	1487.8069	2.0069	NVHRPPRQRDIT

Mascot: http://www.matrixscience.com/

Mascot Search Results

Peptide View

MS/MS Fragmentation of **FLEQQNKLLETK**
Found in **ch12u_Q9NSB2|KRT84_HUMAN** in **uni_human**, Keratin, type II cuticular Hb4 OS=Homo sapiens GN=KRT84 PE=2 SV=2

Match to Query 15380: 1489.814622 from(497.612150,3+) intensity(15984167.0000) rtinseconds(1813) scans(9674) index(7312)
Title: 160219_Sunil_KAP_B_Spectrum056440_scans__9674_RTINSECONDS=1813
Data file L:\\QE_2016\\160219_Sunil_KAP_LKC\\TMgf\\T\\T160219_Sunil_KAP_B.mgf

Click mouse within plot area to zoom in by factor of two about that point
Or, Plot from 100 to 1400 Da Full range
Label all possible matches ○ Label matches used for scoring ◉
Show Y-axis ☐

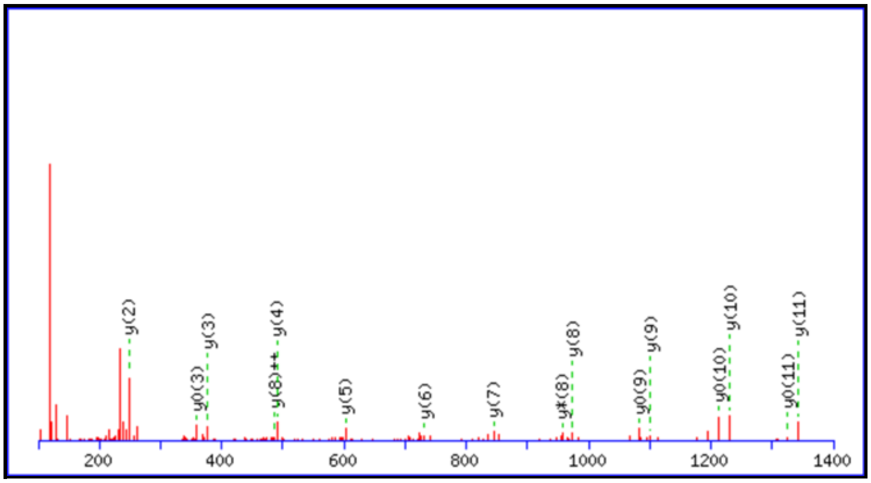

Monoisotopic mass of neutral peptide Mr(calc): 1489.8140
Fixed modifications: Carbamidomethyl (C) (apply to specified residues or termini only)
Ions Score: 74 **Expect:** 4.6e-006
Matches : 16/120 fragment ions using 26 most intense peaks (help)

#	b	b^{++}	b*	b^{*++}	b^{0}	b^{0++}	Seq.	y	y^{++}	y*	y^{*++}	y^{0}	y^{0++}	#
1	148.0757	74.5415					F							12
2	261.1598	131.0835					L	**1343.7529**	672.3801	1326.7264	663.8668	1325.7423	663.3748	11
3	390.2023	195.6048			372.1918	186.5995	E	**1230.6688**	615.8381	1213.6423	607.3248	1212.6583	606.8328	10
4	518.2609	259.6341	501.2344	251.1208	500.2504	250.6288	Q	**1101.6263**	551.3168	1084.5997	542.8035	1083.6157	542.3115	9
5	646.3195	323.6634	629.2930	315.1501	628.3089	314.6581	Q	**973.5677**	487.2875	956.5411	478.7742	955.5571	478.2822	8
6	760.3624	380.6849	743.3359	372.1716	742.3519	371.6796	N	**845.5091**	423.2582	828.4825	414.7449	827.4985	414.2529	7
7	888.4574	444.7323	871.4308	436.2191	870.4468	435.7271	K	**731.4662**	366.2367	714.4396	357.7234	713.4556	357.2314	6
8	1001.5415	501.2744	984.5149	492.7611	983.5309	492.2691	L	**603.3712**	302.1892	586.3447	293.6760	585.3606	293.1840	5
9	1114.6255	557.8164	1097.5990	549.3031	1096.6150	548.8111	L	**490.2871**	245.6472	473.2606	237.1339	472.2766	236.6419	4
10	1243.6681	622.3377	1226.6416	613.8244	1225.6575	613.3324	E	**377.2031**	189.1052	360.1765	180.5919	359.1925	180.0999	3
11	1344.7158	672.8615	1327.6892	664.3483	1326.7052	663.8563	T	**248.1605**	124.5839	231.1339	116.0706	230.1499	115.5786	2
12							K	147.1128	74.0600	130.0863	65.5468			1

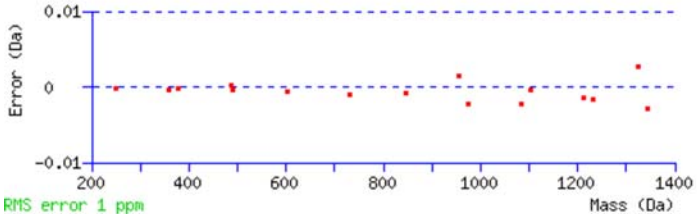

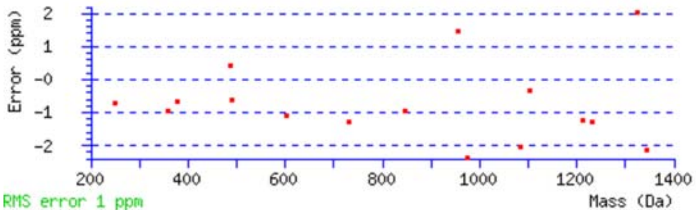

NCBI **BLAST** search of FLEQQNKLLETK
(Parameters: blastp, nr protein database, expect=20000, no filter, PAM30)
Other BLAST web gateways

All matches to this query

Score	Mr(calc)	Delta	Sequence
74.0	1489.8140	0.0006	FLEQQNKLLETK
3.6	1487.8130	2.0017	VLEERGREILMK
0.7	1487.8069	2.0077	NVHRPPRQRDIT

Mascot: http://www.matrixscience.com/

Mascot Search Results

Peptide View

MS/MS Fragmentation of **FLEQQNKLLETK**
Found in **ch12u_Q9NSB2|KRT84_HUMAN** in **uni_human**, Keratin, type II cuticular Hb4 OS=Homo sapiens GN=KRT84 PE=2 SV=2

Match to Query 15429: 1490.798262 from(497.940030,3+) intensity(435085.0938) rtinseconds(1786) scans(9384) index(21380)
Title: 160219_Sunil_KAP_B_Spectrum071273_scans__9384_RTINSECONDS=1786
Data file L:\\QE_2016\\160219_Sunil_KAP_LKC\\TMgf\\T\\T160219_Sunil_KAP_B.mgf

Click mouse within plot area to zoom in by factor of two about that point
Or, Plot from 100 to 1400 Da Full range
Label all possible matches ○ Label matches used for scoring ◉
Show Y-axis ☐

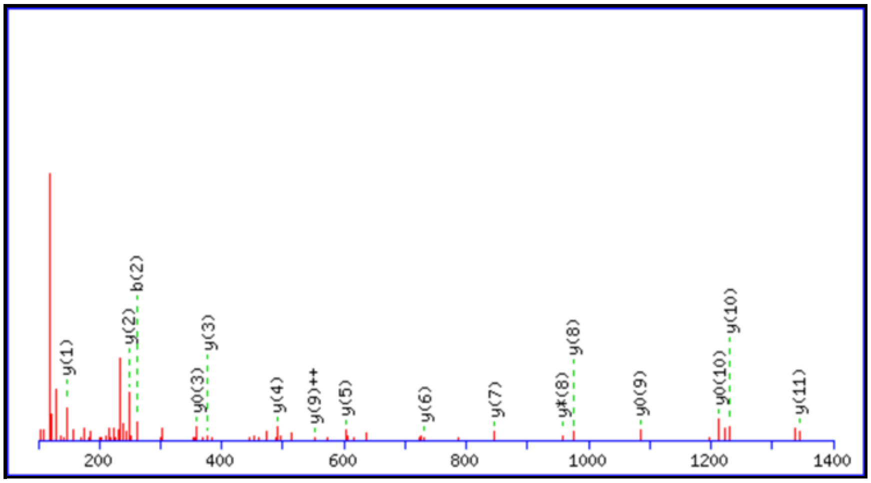

Monoisotopic mass of neutral peptide Mr(calc): 1490.7980
Fixed modifications: Carbamidomethyl (C) (apply to specified residues or termini only)
Variable modifications:
N6 : Deamidated (NQ)
Ions Score: 65 **Expect:** 4.5e-005
Matches : 16/120 fragment ions using 31 most intense peaks (help)

#	b	b^{++}	b*	b^{*++}	b^{0}	b^{0++}	Seq.	y	y^{++}	y*	y^{*++}	y^{0}	y^{0++}	#
1	148.0757	74.5415					F							12
2	**261.1598**	131.0835					L	***1344.7369***	672.8721	1327.7104	664.3588	1326.7264	663.8668	11
3	390.2023	195.6048			372.1918	186.5995	E	***1231.6529***	616.3301	1214.6263	607.8168	1213.6423	607.3248	10
4	518.2609	259.6341	501.2344	251.1208	500.2504	250.6288	Q	1102.6103	551.8088	1085.5837	543.2955	1084.5997	542.8035	9
5	646.3195	323.6634	629.2930	315.1501	628.3089	314.6581	Q	***974.5517***	487.7795	957.5251	479.2662	956.5411	478.7742	8
6	761.3464	381.1769	744.3199	372.6636	743.3359	372.1716	N	***846.4931***	423.7502	829.4666	415.2369	828.4825	414.7449	7
7	889.4414	445.2243	872.4149	436.7111	871.4308	436.2191	K	***731.4662***	366.2367	714.4396	357.7234	713.4556	357.2314	6
8	1002.5255	501.7664	985.4989	493.2531	984.5149	492.7611	L	***603.3712***	302.1892	586.3447	293.6760	585.3606	293.1840	5
9	1115.6095	558.3084	1098.5830	549.7951	1097.5990	549.3031	L	***490.2871***	245.6472	473.2606	237.1339	472.2766	236.6419	4
10	1244.6521	622.8297	1227.6256	614.3164	1226.6416	613.8244	E	***377.2031***	189.1052	360.1765	180.5919	359.1925	180.0999	3
11	1345.6998	673.3535	1328.6733	664.8403	1327.6892	664.3483	T	***248.1605***	124.5839	231.1339	116.0706	230.1499	115.5786	2
12							K	***147.1128***	74.0600	130.0863	65.5468			1

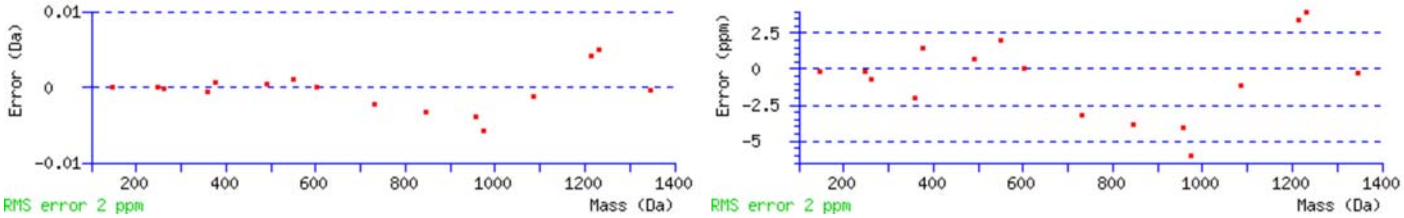

NCBI **BLAST** search of FLEQQNKLLETK
(Parameters: blastp, nr protein database, expect=20000, no filter, PAM30)
Other BLAST web gateways

All matches to this query

Score	Mr(calc)	Delta	Sequence	Site Analysis
64.6	1490.7980	0.0002	FLEQQNKLLETK	Deamidated N6 95.12%
51.5	1490.7980	0.0002	FLEQQNKLLETK	Deamidated Q5 4.68%
37.7	1490.7980	0.0002	FLEQQNKLLETK	Deamidated Q4 0.20%
11.0	1490.7981	0.0002	FQALQSVDILETK	
0.7	1488.7909	2.0073	NVHRPPRQRDIT	
0.7	1488.7909	2.0073	NVHRPPRQRDIT	

Mascot: http://www.matrixscience.com/

Mascot Search Results

Peptide View

MS/MS Fragmentation of **VRFLEQQNKLLETK**
Found in **ch12u_Q9NSB2|KRT84_HUMAN** in **uni_human**, Keratin, type II cuticular Hb4 OS=Homo sapiens GN=KRT84 PE=2 SV=2

Match to Query 21563: 1744.981902 from(582.667910,3+) intensity(160654.1563) rtinseconds(1629) scans(8486) index(20580)
Title: 160219_Sunil_KAP_B_Spectrum070464_scans__8486_RTINSECONDS=1629
Data file L:\\QE_2016\\160219_Sunil_KAP_LKC\\TMgf\\T\\T160219_Sunil_KAP_B.mgf

Click mouse within plot area to zoom in by factor of two about that point
Or, Plot from 100 to 1400 Da Full range
Label all possible matches ○ Label matches used for scoring ◉
Show Y-axis □

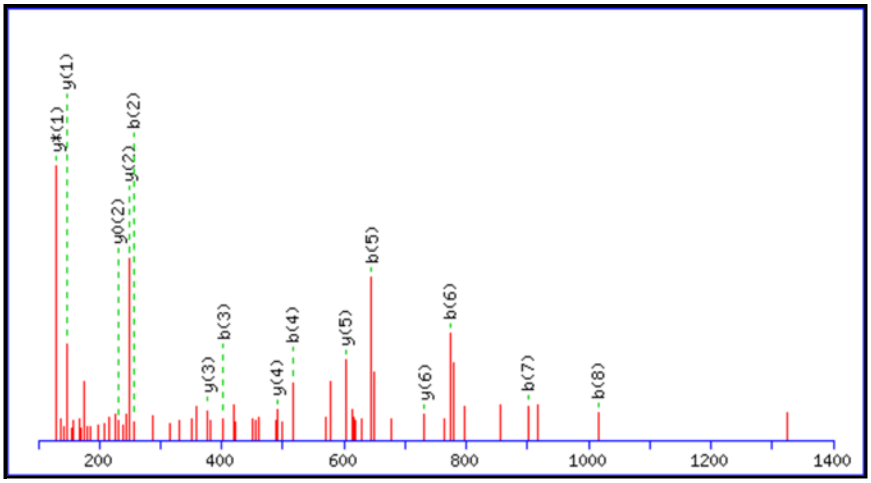

Monoisotopic mass of neutral peptide Mr(calc): 1744.9835
Fixed modifications: Carbamidomethyl (C) (apply to specified residues or termini only)
Ions Score: 41 **Expect:** 0.005
Matches : 15/144 fragment ions using 39 most intense peaks (help)

#	b	b++	b*	b*++	b0	b0++	Seq.	y	y++	y*	y*++	y0	y0++	#
1	100.0757	50.5415					V							14
2	***256.1768***	128.5920	239.1503	120.0788			R	1646.9224	823.9649	1629.8959	815.4516	1628.9119	814.9596	13
3	***403.2452***	202.1262	386.2187	193.6130			F	1490.8213	745.9143	1473.7948	737.4010	1472.8108	736.9090	12
4	***516.3293***	258.6683	499.3027	250.1550			L	1343.7529	672.3801	1326.7264	663.8668	1325.7423	663.3748	11
5	***645.3719***	323.1896	628.3453	314.6763	627.3613	314.1843	E	1230.6688	615.8381	1213.6423	607.3248	1212.6583	606.8328	10
6	***773.4305***	387.2189	756.4039	378.7056	755.4199	378.2136	Q	1101.6263	551.3168	1084.5997	542.8035	1083.6157	542.3115	9
7	***901.4890***	451.2482	884.4625	442.7349	883.4785	442.2429	Q	973.5677	487.2875	956.5411	478.7742	955.5571	478.2822	8
8	***1015.5320***	508.2696	998.5054	499.7563	997.5214	499.2643	N	845.5091	423.2582	828.4825	414.7449	827.4985	414.2529	7
9	1143.6269	572.3171	1126.6004	563.8038	1125.6164	563.3118	K	***731.4662***	366.2367	714.4396	357.7234	713.4556	357.2314	6
10	1256.7110	628.8591	1239.6844	620.3459	1238.7004	619.8538	L	***603.3712***	302.1892	586.3447	293.6760	585.3606	293.1840	5
11	1369.7950	685.4012	1352.7685	676.8879	1351.7845	676.3959	L	***490.2871***	245.6472	473.2606	237.1339	472.2766	236.6419	4
12	1498.8376	749.9225	1481.8111	741.4092	1480.8271	740.9172	E	***377.2031***	189.1052	360.1765	180.5919	359.1925	180.0999	3
13	1599.8853	800.4463	1582.8588	791.9330	1581.8748	791.4410	T	***248.1605***	124.5839	231.1339	116.0706	230.1499	115.5786	2
14							K	***147.1128***	74.0600	130.0863	65.5468			1

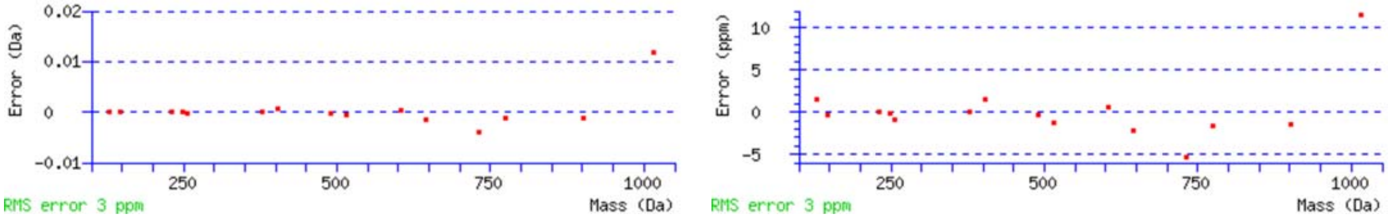

NCBI **BLAST** search of VRFLEQQNKLLETK
(Parameters: blastp, nr protein database, expect=20000, no filter, PAM30)
Other BLAST web gateways

All matches to this query

Score	Mr(calc)	Delta	Sequence
40.9	1744.9835	-0.0016	VRFLEQQNKLLETK
0.6	1744.9869	-0.0050	KSLESINSRLQLVMK
0.4	1743.9659	1.0161	GSLTFEPLTLVPIQTK

Mascot: http://www.matrixscience.com/

Mascot Search Results

Peptide View

MS/MS Fragmentation of **FLEQQNKLLETK**
Found in **ch12u_Q9NSB2|KRT84_HUMAN** in **uni_human**, Keratin, type II cuticular Hb4 OS=Homo sapiens GN=KRT84 PE=2 SV=2

Match to Query 15368: 1489.812848 from(745.913700,2+) intensity(456717.9688) rtinseconds(1614) scans(8398) index(20504)
Title: 160219_Sunil_KAP_B_Spectrum070387_scans__8398_RTINSECONDS=1614
Data file L:\\QE_2016\\160219_Sunil_KAP_LKC\\TMgf\\T\\T160219_Sunil_KAP_B.mgf

Click mouse within plot area to zoom in by factor of two about that point
Or, Plot from 100 to 1500 Da Full range
Label all possible matches ○ Label matches used for scoring ◉
Show Y-axis ☐

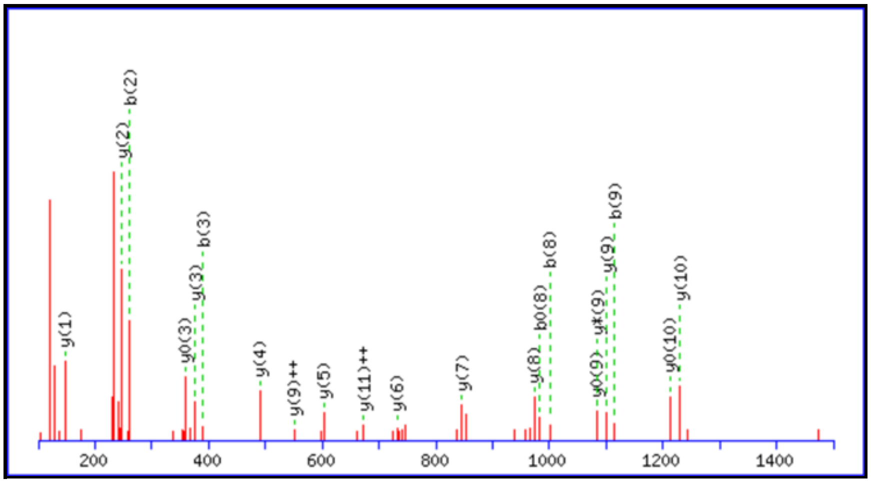

Monoisotopic mass of neutral peptide Mr(calc): 1489.8140
Fixed modifications: Carbamidomethyl (C) (apply to specified residues or termini only)
Ions Score: 82 **Expect:** 8e-007
Matches : 21/120 fragment ions using 32 most intense peaks (help)

#	b	b++	b*	b*++	b0	b0++	Seq.	y	y++	y*	y*++	y0	y0++	#
1	148.0757	74.5415					F							12
2	**261.1598**	131.0835					L	1343.7529	672.3801	1326.7264	663.8668	1325.7423	663.3748	11
3	**390.2023**	195.6048			372.1918	186.5995	E	***1230.6688***	615.8381	1213.6423	607.3248	1212.6583	606.8328	10
4	518.2609	259.6341	501.2344	251.1208	500.2504	250.6288	Q	***1101.6263***	551.3168	1084.5997	542.8035	1083.6157	542.3115	9
5	646.3195	323.6634	629.2930	315.1501	628.3089	314.6581	Q	***973.5677***	487.2875	956.5411	478.7742	955.5571	478.2822	8
6	760.3624	380.6849	743.3359	372.1716	742.3519	371.6796	N	***845.5091***	423.2582	828.4825	414.7449	827.4985	414.2529	7
7	888.4574	444.7323	871.4308	436.2191	870.4468	435.7271	K	***731.4662***	366.2367	714.4396	357.7234	713.4556	357.2314	6
8	**1001.5415**	501.2744	984.5149	492.7611	983.5309	492.2691	L	***603.3712***	302.1892	586.3447	293.6760	585.3606	293.1840	5
9	**1114.6255**	557.8164	1097.5990	549.3031	1096.6150	548.8111	L	***490.2871***	245.6472	473.2606	237.1339	472.2766	236.6419	4
10	1243.6681	622.3377	1226.6416	613.8244	1225.6575	613.3324	E	***377.2031***	189.1052	360.1765	180.5919	359.1925	180.0999	3
11	1344.7158	672.8615	1327.6892	664.3483	1326.7052	663.8563	T	***248.1605***	124.5839	231.1339	116.0706	230.1499	115.5786	2
12							K	***147.1128***	74.0600	130.0863	65.5468			1

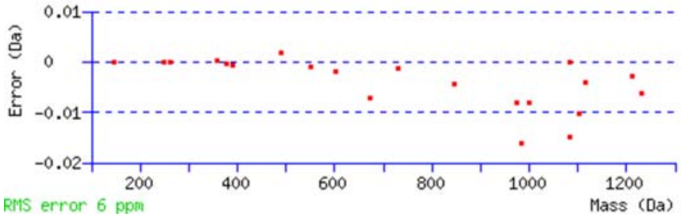

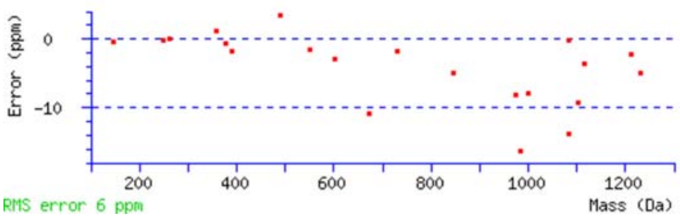

NCBI **BLAST** search of FLEQQNKLLETK
(Parameters: blastp, nr protein database, expect=20000, no filter, PAM30)
Other BLAST web gateways

All matches to this query

Score	Mr(calc)	Delta	Sequence
81.7	1489.8140	-0.0012	FLEQQNKLLETK

Mascot: http://www.matrixscience.com/

Mascot Search Results

Peptide View

MS/MS Fragmentation of **FLEQQNKLLETK**
Found in **ch12u_Q9NSB2|KRT84_HUMAN** in **uni_human**, Keratin, type II cuticular Hb4 OS=Homo sapiens GN=KRT84 PE=2 SV=2

Match to Query 15369: 1489.813448 from(745.914000,2+) intensity(453900.9688) rtinseconds(1818) scans(9706) index(7339)
Title: 160219_Sunil_KAP_B_Spectrum056468_scans__9706_RTINSECONDS=1818
Data file L:\\QE_2016\\160219_Sunil_KAP_LKC\\TMgf\\T\\T160219_Sunil_KAP_B.mgf

Click mouse within plot area to zoom in by factor of two about that point
Or, Plot from 100 to 1300 Da Full range
Label all possible matches ○ Label matches used for scoring ◉
Show Y-axis ☐

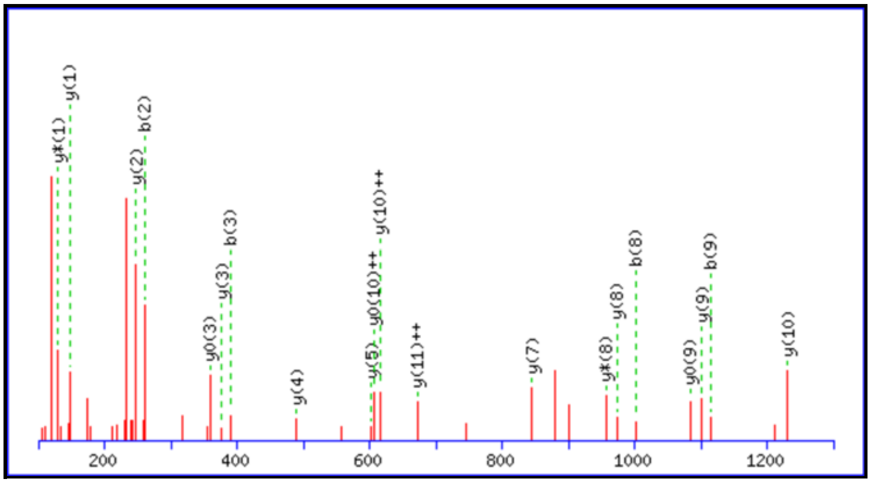

Monoisotopic mass of neutral peptide Mr(calc): 1489.8140
Fixed modifications: Carbamidomethyl (C) (apply to specified residues or termini only)
Ions Score: 62 **Expect:** 7.7e-005
Matches : 20/120 fragment ions using 33 most intense peaks (help)

#	b	b^{++}	b*	b^{*++}	b^{0}	b^{0++}	Seq.	y	y^{++}	y*	y^{*++}	y^{0}	y^{0++}	#
1	148.0757	74.5415					F							12
2	**261.1598**	131.0835					L	1343.7529	672.3801	1326.7264	663.8668	1325.7423	663.3748	11
3	**390.2023**	195.6048			372.1918	186.5995	E	***1230.6688***	615.8381	1213.6423	607.3248	1212.6583	606.8328	10
4	518.2609	259.6341	501.2344	251.1208	500.2504	250.6288	Q	***1101.6263***	551.3168	1084.5997	542.8035	1083.6157	542.3115	9
5	646.3195	323.6634	629.2930	315.1501	628.3089	314.6581	Q	***973.5677***	487.2875	956.5411	478.7742	955.5571	478.2822	8
6	760.3624	380.6849	743.3359	372.1716	742.3519	371.6796	N	***845.5091***	423.2582	828.4825	414.7449	827.4985	414.2529	7
7	888.4574	444.7323	871.4308	436.2191	870.4468	435.7271	K	731.4662	366.2367	714.4396	357.7234	713.4556	357.2314	6
8	**1001.5415**	501.2744	984.5149	492.7611	983.5309	492.2691	L	***603.3712***	302.1892	586.3447	293.6760	585.3606	293.1840	5
9	**1114.6255**	557.8164	1097.5990	549.3031	1096.6150	548.8111	L	***490.2871***	245.6472	473.2606	237.1339	472.2766	236.6419	4
10	1243.6681	622.3377	1226.6416	613.8244	1225.6575	613.3324	E	***377.2031***	189.1052	360.1765	180.5919	359.1925	180.0999	3
11	1344.7158	672.8615	1327.6892	664.3483	1326.7052	663.8563	T	***248.1605***	124.5839	231.1339	116.0706	230.1499	115.5786	2
12							K	***147.1128***	74.0600	130.0863	65.5468			1

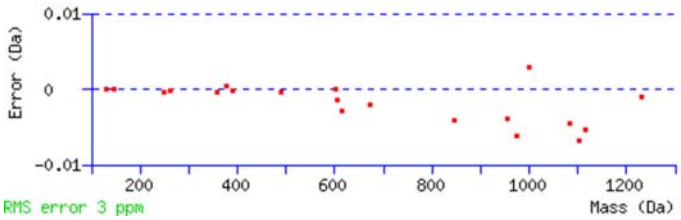

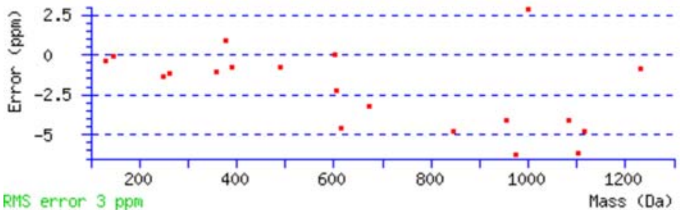

NCBI **BLAST** search of FLEQQNKLLETK
(Parameters: blastp, nr protein database, expect=20000, no filter, PAM30)
Other BLAST web gateways

All matches to this query

Score	Mr(calc)	Delta	Sequence
61.7	1489.8140	-0.0006	FLEQQNKLLETK

Mascot: http://www.matrixscience.com/

Mascot Search Results

Peptide View

MS/MS Fragmentation of **FLEQQNKLLETK**
Found in **ch12u_Q9NSB2|KRT84_HUMAN** in **uni_human**, Keratin, type II cuticular Hb4 OS=Homo sapiens GN=KRT84 PE=2 SV=2

Match to Query 15115: 1489.814532 from(497.612120,3+) intensity(1257133.5000) rtinseconds(1928) scans(10597) index(22763)
Title: 160219_Sunil_KAP_A1_Spectrum075224_scans__10597_RTINSECONDS=1928
Data file L:\\QE_2016\\160219_Sunil_KAP_LKC\\TMgf\\T\\T160219_Sunil_KAP_A1.mgf

Click mouse within plot area to zoom in by factor of two about that point
Or, Plot from 100 to 1400 Da Full range
Label all possible matches ○ Label matches used for scoring ◉
Show Y-axis ☐

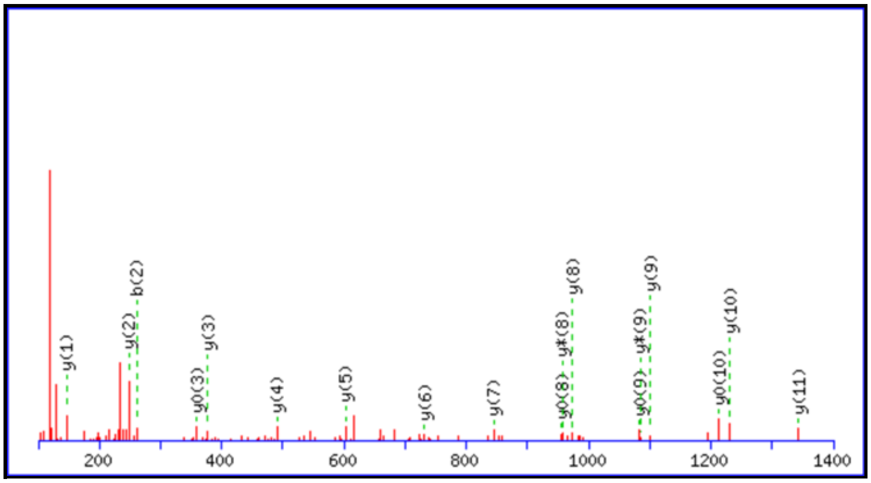

Monoisotopic mass of neutral peptide Mr(calc): 1489.8140
Fixed modifications: Carbamidomethyl (C) (apply to specified residues or termini only)
Ions Score: 76 **Expect:** 2.8e-006
Matches : 18/120 fragment ions using 34 most intense peaks (help)

#	b	b^{++}	b^{*}	b^{*++}	b^{0}	b^{0++}	Seq.	y	y^{++}	y^{*}	y^{*++}	y^{0}	y^{0++}	#
1	148.0757	74.5415					F							12
2	**261.1598**	131.0835					L	***1343.7529***	672.3801	1326.7264	663.8668	1325.7423	663.3748	11
3	390.2023	195.6048			372.1918	186.5995	E	***1230.6688***	615.8381	1213.6423	607.3248	1212.6583	606.8328	10
4	518.2609	259.6341	501.2344	251.1208	500.2504	250.6288	Q	***1101.6263***	551.3168	1084.5997	542.8035	1083.6157	542.3115	9
5	646.3195	323.6634	629.2930	315.1501	628.3089	314.6581	Q	***973.5677***	487.2875	956.5411	478.7742	955.5571	478.2822	8
6	760.3624	380.6849	743.3359	372.1716	742.3519	371.6796	N	***845.5091***	423.2582	828.4825	414.7449	827.4985	414.2529	7
7	888.4574	444.7323	871.4308	436.2191	870.4468	435.7271	K	***731.4662***	366.2367	714.4396	357.7234	713.4556	357.2314	6
8	1001.5415	501.2744	984.5149	492.7611	983.5309	492.2691	L	***603.3712***	302.1892	586.3447	293.6760	585.3606	293.1840	5
9	1114.6255	557.8164	1097.5990	549.3031	1096.6150	548.8111	L	***490.2871***	245.6472	473.2606	237.1339	472.2766	236.6419	4
10	1243.6681	622.3377	1226.6416	613.8244	1225.6575	613.3324	E	***377.2031***	189.1052	360.1765	180.5919	359.1925	180.0999	3
11	1344.7158	672.8615	1327.6892	664.3483	1326.7052	663.8563	T	***248.1605***	124.5839	231.1339	116.0706	230.1499	115.5786	2
12							K	***147.1128***	74.0600	130.0863	65.5468			1

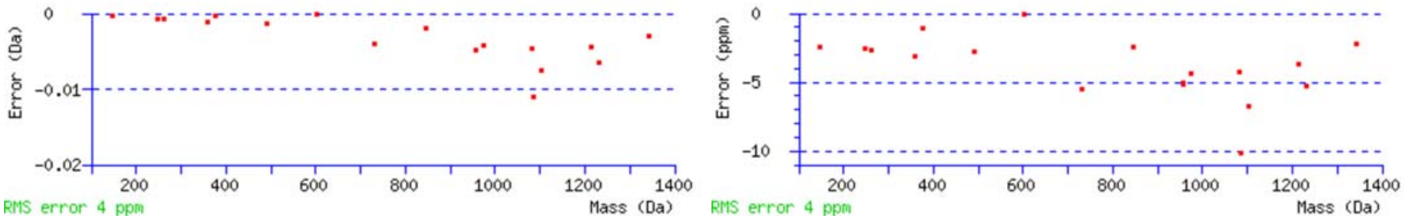

NCBI **BLAST** search of FLEQQNKLLETK
(Parameters: blastp, nr protein database, expect=20000, no filter, PAM30)
Other BLAST web gateways

All matches to this query

Score	Mr(calc)	Delta	Sequence
76.1	1489.8140	0.0005	FLEQQNKLLETK
0.5	1487.8069	2.0076	NVHRPPRQRDIT

Mascot: http://www.matrixscience.com/

Mascot Search Results

Peptide View

MS/MS Fragmentation of **FLEQQNKLLETK**
Found in **ch12u_Q9NSB2|KRT84_HUMAN** in **uni_human**, Keratin, type II cuticular Hb4 OS=Homo sapiens GN=KRT84 PE=2 SV=2

Match to Query 15106: 1489.813452 from(497.611760,3+) intensity(162960.5625) rtinseconds(1461) scans(7777) index(5999)
Title: 160219_Sunil_KAP_A1_Spectrum056965_scans__7777_RTINSECONDS=1461
Data file L:\\QE_2016\\160219_Sunil_KAP_LKC\\TMgf\\T\\T160219_Sunil_KAP_A1.mgf

Click mouse within plot area to zoom in by factor of two about that point
Or, Plot from 100 to 1400 Da Full range
Label all possible matches ○ Label matches used for scoring ◉
Show Y-axis ☐

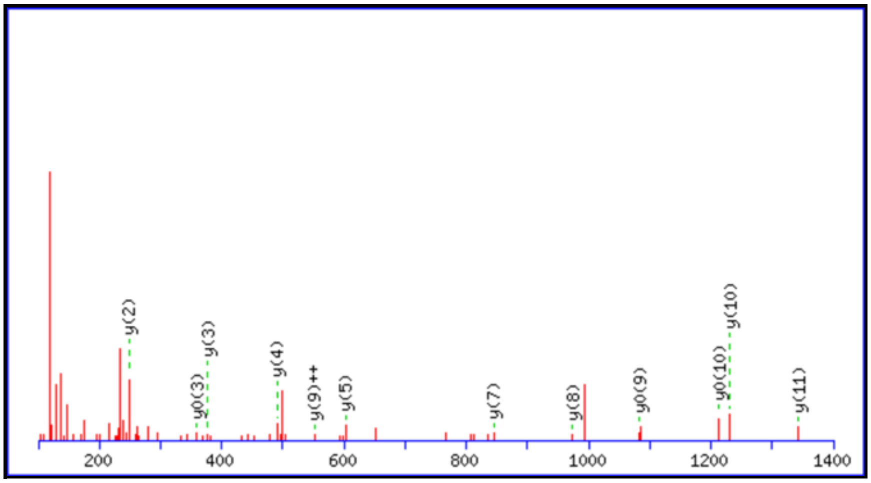

Monoisotopic mass of neutral peptide Mr(calc): 1489.8140
Fixed modifications: Carbamidomethyl (C) (apply to specified residues or termini only)
Ions Score: 48 **Expect:** 0.002
Matches : 12/120 fragment ions using 22 most intense peaks (help)

#	b	b^{++}	b*	b*$^{++}$	b^{0}	b^{0++}	Seq.	y	y^{++}	y*	y*$^{++}$	y^{0}	y^{0++}	#
1	148.0757	74.5415					F							12
2	261.1598	131.0835					L	***1343.7529***	672.3801	1326.7264	663.8668	1325.7423	663.3748	11
3	390.2023	195.6048			372.1918	186.5995	E	***1230.6688***	615.8381	1213.6423	607.3248	1212.6583	606.8328	10
4	518.2609	259.6341	501.2344	251.1208	500.2504	250.6288	Q	1101.6263	551.3168	1084.5997	542.8035	1083.6157	542.3115	9
5	646.3195	323.6634	629.2930	315.1501	628.3089	314.6581	Q	***973.5677***	487.2875	956.5411	478.7742	955.5571	478.2822	8
6	760.3624	380.6849	743.3359	372.1716	742.3519	371.6796	N	***845.5091***	423.2582	828.4825	414.7449	827.4985	414.2529	7
7	888.4574	444.7323	871.4308	436.2191	870.4468	435.7271	K	731.4662	366.2367	714.4396	357.7234	713.4556	357.2314	6
8	1001.5415	501.2744	984.5149	492.7611	983.5309	492.2691	L	***603.3712***	302.1892	586.3447	293.6760	585.3606	293.1840	5
9	1114.6255	557.8164	1097.5990	549.3031	1096.6150	548.8111	L	***490.2871***	245.6472	473.2606	237.1339	472.2766	236.6419	4
10	1243.6681	622.3377	1226.6416	613.8244	1225.6575	613.3324	E	***377.2031***	189.1052	360.1765	180.5919	359.1925	180.0999	3
11	1344.7158	672.8615	1327.6892	664.3483	1326.7052	663.8563	T	***248.1605***	124.5839	231.1339	116.0706	230.1499	115.5786	2
12							K	147.1128	74.0600	130.0863	65.5468			1

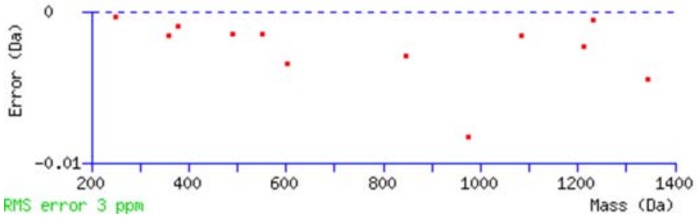

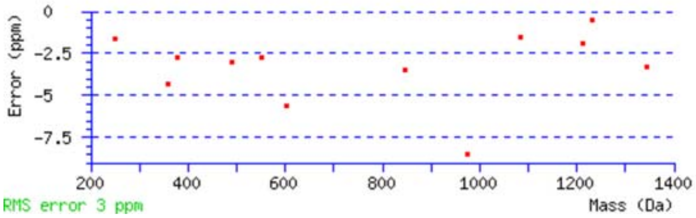

NCBI **BLAST** search of FLEQQNKLLETK
(Parameters: blastp, nr protein database, expect=20000, no filter, PAM30)
Other BLAST web gateways

All matches to this query

Score	Mr(calc)	Delta	Sequence
47.7	1489.8140	-0.0006	FLEQQNKLLETK
0.2	1487.8069	2.0065	NVHRPPRQRDIT

Mascot: http://www.matrixscience.com/

Mascot Search Results

Peptide View

MS/MS Fragmentation of **FLEQQNKLLETK**
Found in **ch12u_Q9NSB2|KRT84_HUMAN** in **uni_human**, Keratin, type II cuticular Hb4 OS=Homo sapiens GN=KRT84 PE=2 SV=2

Match to Query 15109: 1489.813992 from(497.611940,3+) intensity(1373482.0000) rtinseconds(1427) scans(7718) index(20404)
Title: 160219_Sunil_KAP_A1_Spectrum072856_scans__7718_RTINSECONDS=1427
Data file L:\\QE_2016\\160219_Sunil_KAP_LKC\\TMgf\\T\\T160219_Sunil_KAP_A1.mgf

Click mouse within plot area to zoom in by factor of two about that point
Or, Plot from 100 to 1400 Da Full range
Label all possible matches ○ Label matches used for scoring ◉
Show Y-axis □

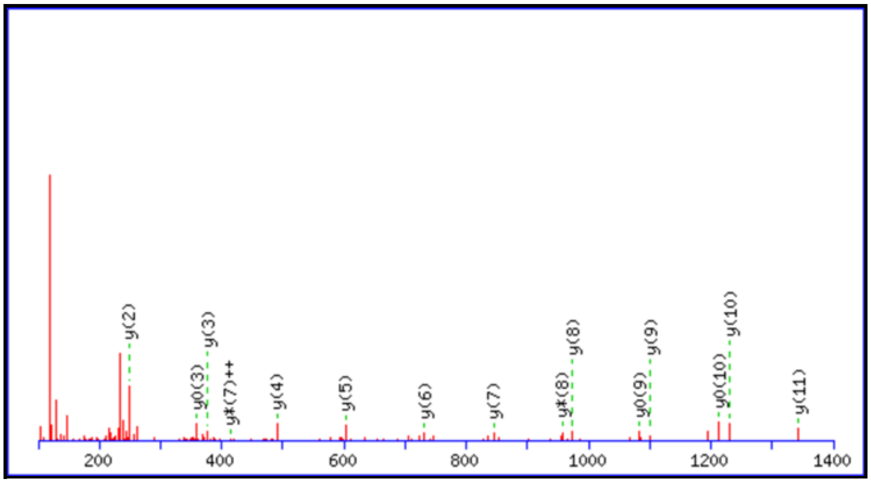

Monoisotopic mass of neutral peptide Mr(calc): 1489.8140
Fixed modifications: Carbamidomethyl (C) (apply to specified residues or termini only)
Ions Score: 75 **Expect:** 3.2e-006
Matches : 15/120 fragment ions using 24 most intense peaks (help)

#	b	b^{++}	b^{*}	b^{*++}	b^{0}	b^{0++}	Seq.	y	y^{++}	y^{*}	y^{*++}	y^{0}	y^{0++}	#
1	148.0757	74.5415					F							12
2	261.1598	131.0835					L	**1343.7529**	672.3801	1326.7264	663.8668	1325.7423	663.3748	11
3	390.2023	195.6048			372.1918	186.5995	E	**1230.6688**	615.8381	1213.6423	607.3248	1212.6583	606.8328	10
4	518.2609	259.6341	501.2344	251.1208	500.2504	250.6288	Q	**1101.6263**	551.3168	1084.5997	542.8035	1083.6157	542.3115	9
5	646.3195	323.6634	629.2930	315.1501	628.3089	314.6581	Q	**973.5677**	487.2875	956.5411	478.7742	955.5571	478.2822	8
6	760.3624	380.6849	743.3359	372.1716	742.3519	371.6796	N	**845.5091**	423.2582	828.4825	414.7449	827.4985	414.2529	7
7	888.4574	444.7323	871.4308	436.2191	870.4468	435.7271	K	**731.4662**	366.2367	714.4396	357.7234	713.4556	357.2314	6
8	1001.5415	501.2744	984.5149	492.7611	983.5309	492.2691	L	**603.3712**	302.1892	586.3447	293.6760	585.3606	293.1840	5
9	1114.6255	557.8164	1097.5990	549.3031	1096.6150	548.8111	L	**490.2871**	245.6472	473.2606	237.1339	472.2766	236.6419	4
10	1243.6681	622.3377	1226.6416	613.8244	1225.6575	613.3324	E	**377.2031**	189.1052	360.1765	180.5919	359.1925	180.0999	3
11	1344.7158	672.8615	1327.6892	664.3483	1326.7052	663.8563	T	**248.1605**	124.5839	231.1339	116.0706	230.1499	115.5786	2
12							K	147.1128	74.0600	130.0863	65.5468			1

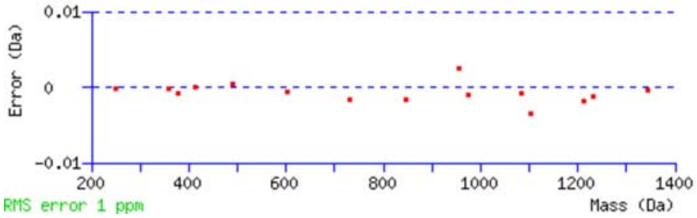

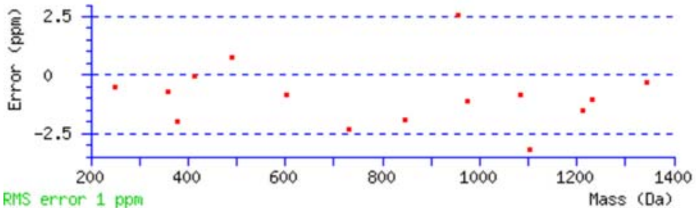

NCBI **BLAST** search of FLEQQNKLLETK
(Parameters: blastp, nr protein database, expect=20000, no filter, PAM30)
Other BLAST web gateways

All matches to this query

Score	Mr(calc)	Delta	Sequence
75.4	1489.8140	-0.0000	FLEQQNKLLETK
0.9	1487.8069	2.0071	NVHRPPRQRDIT

Mascot: http://www.matrixscience.com/

Mascot Search Results

Peptide View

MS/MS Fragmentation of **FLEQQNKLLETK**
Found in **ch12u_Q9NSB2|KRT84_HUMAN** in **uni_human**, Keratin, type II cuticular Hb4 OS=Homo sapiens GN=KRT84 PE=2 SV=2

Match to Query 15112: 1489.814262 from(497.612030,3+) intensity(507888.0000) rtinseconds(1341) scans(7171) index(34178)
Title: 160219_Sunil_KAP_A1_Spectrum088435_scans__7171_RTINSECONDS=1341
Data file L:\\QE_2016\\160219_Sunil_KAP_LKC\\TMgf\\T\\T160219_Sunil_KAP_A1.mgf

Click mouse within plot area to zoom in by factor of two about that point
Or, Plot from 100 to 1400 Da Full range
Label all possible matches ○ Label matches used for scoring ◉
Show Y-axis ☐

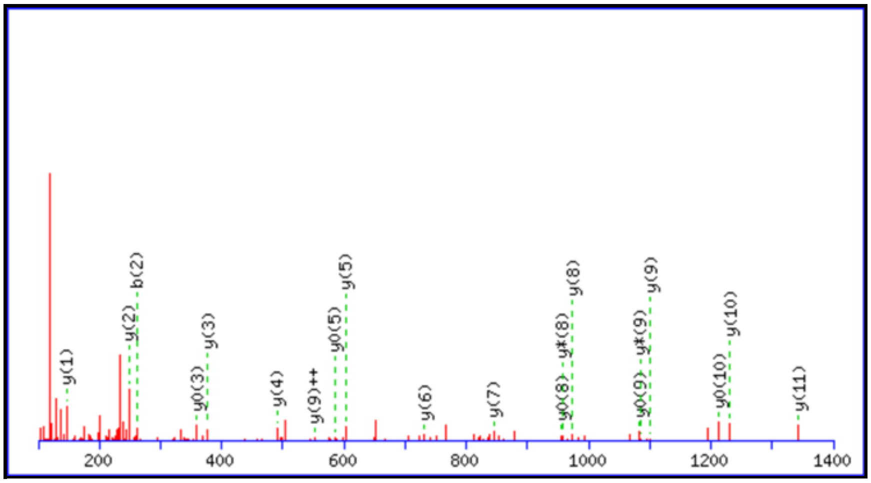

Monoisotopic mass of neutral peptide Mr(calc): 1489.8140
Fixed modifications: Carbamidomethyl (C) (apply to specified residues or termini only)
Ions Score: 66 **Expect:** 3.1e-005
Matches : 20/120 fragment ions using 44 most intense peaks (help)

#	b	b^{++}	b*	b^{*++}	b^0	b^{0++}	Seq.	y	y^{++}	y*	y^{*++}	y^0	y^{0++}	#
1	148.0757	74.5415					F							12
2	**261.1598**	131.0835					L	***1343.7529***	672.3801	1326.7264	663.8668	1325.7423	663.3748	11
3	390.2023	195.6048			372.1918	186.5995	E	***1230.6688***	615.8381	1213.6423	607.3248	1212.6583	606.8328	10
4	518.2609	259.6341	501.2344	251.1208	500.2504	250.6288	Q	***1101.6263***	551.3168	1084.5997	542.8035	1083.6157	542.3115	9
5	646.3195	323.6634	629.2930	315.1501	628.3089	314.6581	Q	***973.5677***	487.2875	956.5411	478.7742	955.5571	478.2822	8
6	760.3624	380.6849	743.3359	372.1716	742.3519	371.6796	N	***845.5091***	423.2582	828.4825	414.7449	827.4985	414.2529	7
7	888.4574	444.7323	871.4308	436.2191	870.4468	435.7271	K	***731.4662***	366.2367	714.4396	357.7234	713.4556	357.2314	6
8	1001.5415	501.2744	984.5149	492.7611	983.5309	492.2691	L	***603.3712***	302.1892	586.3447	293.6760	585.3606	293.1840	5
9	1114.6255	557.8164	1097.5990	549.3031	1096.6150	548.8111	L	***490.2871***	245.6472	473.2606	237.1339	472.2766	236.6419	4
10	1243.6681	622.3377	1226.6416	613.8244	1225.6575	613.3324	E	***377.2031***	189.1052	360.1765	180.5919	359.1925	180.0999	3
11	1344.7158	672.8615	1327.6892	664.3483	1326.7052	663.8563	T	***248.1605***	124.5839	231.1339	116.0706	230.1499	115.5786	2
12							K	***147.1128***	74.0600	130.0863	65.5468			1

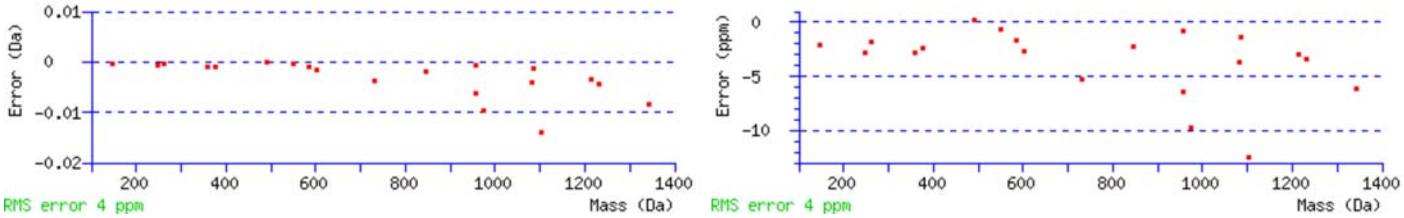

NCBI **BLAST** search of FLEQQNKLLETK
(Parameters: blastp, nr protein database, expect=20000, no filter, PAM30)
Other BLAST web gateways

All matches to this query

Score	Mr(calc)	Delta	Sequence
65.6	1489.8140	0.0003	FLEQQNKLLETK
0.3	1487.8069	2.0073	NVHRPPRQRDIT

Mascot: http://www.matrixscience.com/

Mascot Search Results

Peptide View

MS/MS Fragmentation of **FLEQQNKLLETK**
Found in **ch12u_Q9NSB2|KRT84_HUMAN** in **uni_human**, Keratin, type II cuticular Hb4 OS=Homo sapiens GN=KRT84 PE=2 SV=2

Match to Query 15116: 1489.814622 from(497.612150,3+) intensity(8553404.0000) rtinseconds(1493) scans(7964) index(6161)
Title: 160219_Sunil_KAP_A1_Spectrum057128_scans__7964_RTINSECONDS=1493
Data file L:\\QE_2016\\160219_Sunil_KAP_LKC\\TMgf\\T\\T160219_Sunil_KAP_A1.mgf

Click mouse within plot area to zoom in by factor of two about that point
Or, Plot from 100 to 1400 Da Full range
Label all possible matches ○ Label matches used for scoring ◉
Show Y-axis ☐

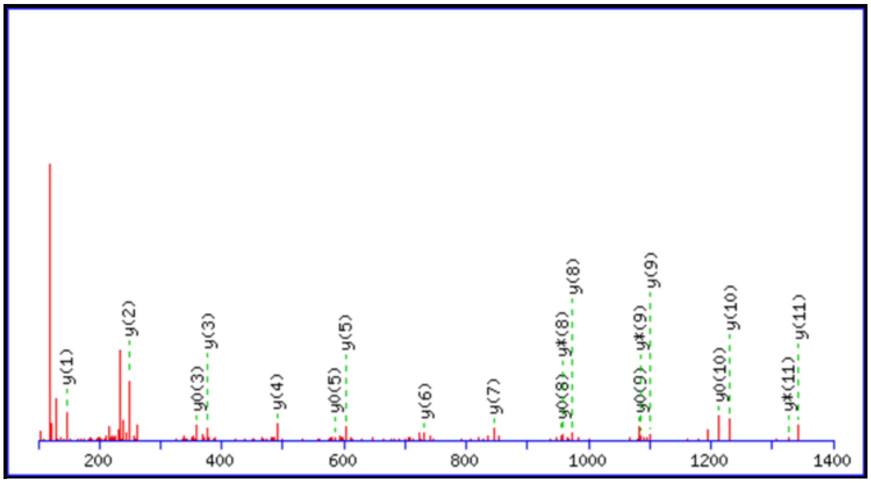

Monoisotopic mass of neutral peptide Mr(calc): 1489.8140
Fixed modifications: Carbamidomethyl (C) (apply to specified residues or termini only)
Ions Score: 72 **Expect:** 6.7e-006
Matches : 19/120 fragment ions using 38 most intense peaks (help)

#	b	b^{++}	b*	b^{*++}	b^0	b^{0++}	Seq.	y	y^{++}	y*	y^{*++}	y^0	y^{0++}	#
1	148.0757	74.5415					F							12
2	261.1598	131.0835					L	***1343.7529***	672.3801	1326.7264	663.8668	1325.7423	663.3748	11
3	390.2023	195.6048			372.1918	186.5995	E	***1230.6688***	615.8381	1213.6423	607.3248	1212.6583	606.8328	10
4	518.2609	259.6341	501.2344	251.1208	500.2504	250.6288	Q	***1101.6263***	551.3168	1084.5997	542.8035	1083.6157	542.3115	9
5	646.3195	323.6634	629.2930	315.1501	628.3089	314.6581	Q	***973.5677***	487.2875	956.5411	478.7742	955.5571	478.2822	8
6	760.3624	380.6849	743.3359	372.1716	742.3519	371.6796	N	***845.5091***	423.2582	828.4825	414.7449	827.4985	414.2529	7
7	888.4574	444.7323	871.4308	436.2191	870.4468	435.7271	K	***731.4662***	366.2367	714.4396	357.7234	713.4556	357.2314	6
8	1001.5415	501.2744	984.5149	492.7611	983.5309	492.2691	L	***603.3712***	302.1892	586.3447	293.6760	585.3606	293.1840	5
9	1114.6255	557.8164	1097.5990	549.3031	1096.6150	548.8111	L	***490.2871***	245.6472	473.2606	237.1339	472.2766	236.6419	4
10	1243.6681	622.3377	1226.6416	613.8244	1225.6575	613.3324	E	***377.2031***	189.1052	360.1765	180.5919	359.1925	180.0999	3
11	1344.7158	672.8615	1327.6892	664.3483	1326.7052	663.8563	T	***248.1605***	124.5839	231.1339	116.0706	230.1499	115.5786	2
12							K	***147.1128***	74.0600	130.0863	65.5468			1

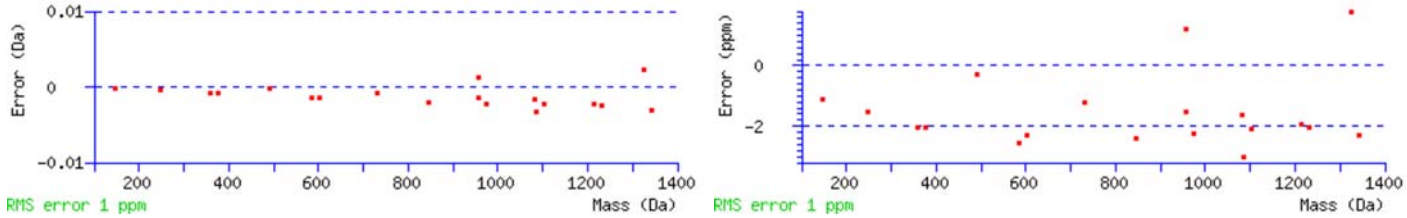

NCBI **BLAST** search of FLEQQNKLLETK
(Parameters: blastp, nr protein database, expect=20000, no filter, PAM30)
Other BLAST web gateways

All matches to this query

Score	Mr(calc)	Delta	Sequence
72.3	1489.8140	0.0006	FLEQQNKLLETK
0.6	1487.8069	2.0077	NVHRPPRQRDIT

Mascot: http://www.matrixscience.com/

Mascot Search Results

Peptide View

MS/MS Fragmentation of **FLEQQNKLLETK**
Found in **ch12u_Q9NSB2|KRT84_HUMAN** in **uni_human**, Keratin, type II cuticular Hb4 OS=Homo sapiens GN=KRT84 PE=2 SV=2

Match to Query 15120: 1489.814802 from(497.612210,3+) intensity(1461620.6250) rtinseconds(1525) scans(8146) index(6312)
Title: 160219_Sunil_KAP_A1_Spectrum057279_scans__8146_RTINSECONDS=1525
Data file L:\\QE_2016\\160219_Sunil_KAP_LKC\\TMgf\\T\\T160219_Sunil_KAP_A1.mgf

Click mouse within plot area to zoom in by factor of two about that point
Or, Plot from 100 to 1400 Da Full range
Label all possible matches ○ Label matches used for scoring ◉
Show Y-axis ☐

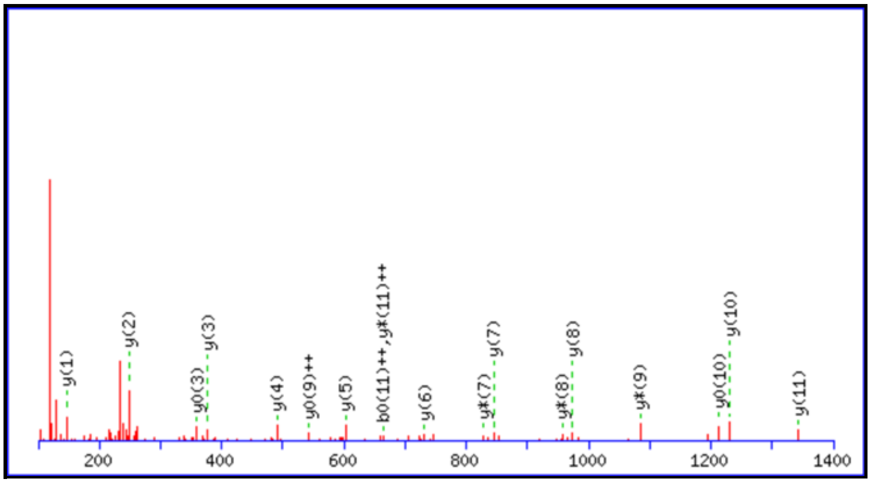

Monoisotopic mass of neutral peptide Mr(calc): 1489.8140
Fixed modifications: Carbamidomethyl (C) (apply to specified residues or termini only)
Ions Score: 67 **Expect:** 2.4e-005
Matches : 18/120 fragment ions using 34 most intense peaks (help)

#	b	b^{++}	b*	b^{*++}	b^{0}	b^{0++}	Seq.	y	y^{++}	y*	y^{*++}	y^{0}	y^{0++}	#
1	148.0757	74.5415					F							12
2	261.1598	131.0835					L	***1343.7529***	672.3801	1326.7264	663.8668	1325.7423	663.3748	11
3	390.2023	195.6048			372.1918	186.5995	E	***1230.6688***	615.8381	1213.6423	607.3248	1212.6583	606.8328	10
4	518.2609	259.6341	501.2344	251.1208	500.2504	250.6288	Q	1101.6263	551.3168	1084.5997	542.8035	1083.6157	542.3115	9
5	646.3195	323.6634	629.2930	315.1501	628.3089	314.6581	Q	***973.5677***	487.2875	956.5411	478.7742	955.5571	478.2822	8
6	760.3624	380.6849	743.3359	372.1716	742.3519	371.6796	N	***845.5091***	423.2582	828.4825	414.7449	827.4985	414.2529	7
7	888.4574	444.7323	871.4308	436.2191	870.4468	435.7271	K	***731.4662***	366.2367	714.4396	357.7234	713.4556	357.2314	6
8	1001.5415	501.2744	984.5149	492.7611	983.5309	492.2691	L	***603.3712***	302.1892	586.3447	293.6760	585.3606	293.1840	5
9	1114.6255	557.8164	1097.5990	549.3031	1096.6150	548.8111	L	***490.2871***	245.6472	473.2606	237.1339	472.2766	236.6419	4
10	1243.6681	622.3377	1226.6416	613.8244	1225.6575	613.3324	E	***377.2031***	189.1052	360.1765	180.5919	359.1925	180.0999	3
11	1344.7158	672.8615	1327.6892	664.3483	1326.7052	663.8563	T	***248.1605***	124.5839	231.1339	116.0706	230.1499	115.5786	2
12							K	***147.1128***	74.0600	130.0863	65.5468			1

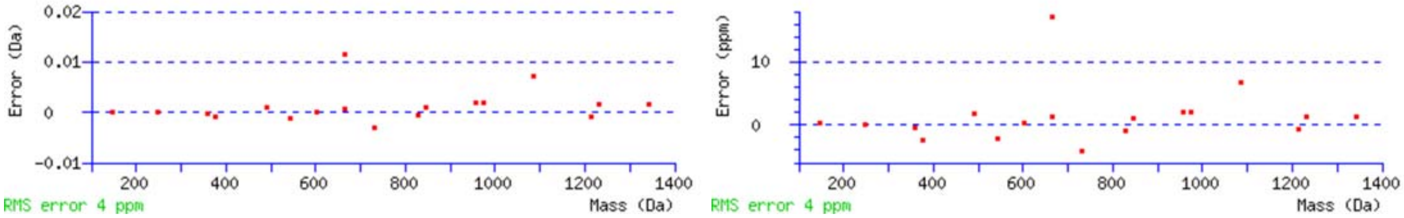

NCBI **BLAST** search of FLEQQNKLLETK
(Parameters: blastp, nr protein database, expect=20000, no filter, PAM30)
Other BLAST web gateways

All matches to this query

Score	Mr(calc)	Delta	Sequence
66.8	1489.8140	0.0008	FLEQQNKLLETK
0.8	1487.8069	2.0079	NVHRPPRQRDIT

Mascot: http://www.matrixscience.com/

Mascot Search Results

Peptide View

MS/MS Fragmentation of **FLEQQNKLLETK**
Found in **ch12u_Q9NSB2|KRT84_HUMAN** in **uni_human**, Keratin, type II cuticular Hb4 OS=Homo sapiens GN=KRT84 PE=2 SV=2

Match to Query 15123: 1489.815462 from(497.612430,3+) intensity(248258.7813) rtinseconds(1519) scans(8246) index(20879)
Title: 160219_Sunil_KAP_A1_Spectrum073332_scans__8246_RTINSECONDS=1519
Data file L:\\QE_2016\\160219_Sunil_KAP_LKC\\TMgf\\T\\T160219_Sunil_KAP_A1.mgf

Click mouse within plot area to zoom in by factor of two about that point
Or, Plot from 100 to 1400 Da Full range
Label all possible matches ○ Label matches used for scoring ◉
Show Y-axis ☐

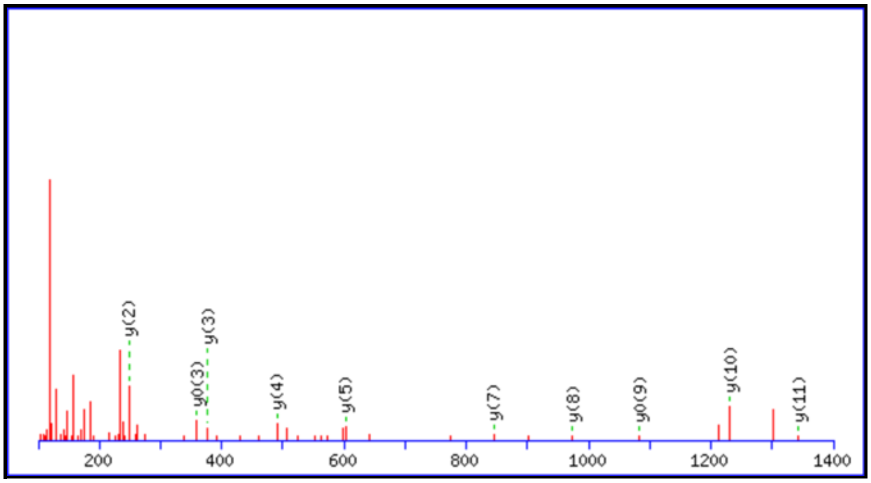

Monoisotopic mass of neutral peptide Mr(calc): 1489.8140
Fixed modifications: Carbamidomethyl (C) (apply to specified residues or termini only)
Ions Score: 46 **Expect:** 0.0025
Matches : 10/120 fragment ions using 20 most intense peaks (help)

#	b	b^{++}	b*	b^{*++}	b^0	b^{0++}	Seq.	y	y^{++}	y*	y^{*++}	y^0	y^{0++}	#
1	148.0757	74.5415					F							12
2	261.1598	131.0835					L	***1343.7529***	672.3801	1326.7264	663.8668	1325.7423	663.3748	11
3	390.2023	195.6048			372.1918	186.5995	E	***1230.6688***	615.8381	1213.6423	607.3248	1212.6583	606.8328	10
4	518.2609	259.6341	501.2344	251.1208	500.2504	250.6288	Q	1101.6263	551.3168	1084.5997	542.8035	1083.6157	542.3115	9
5	646.3195	323.6634	629.2930	315.1501	628.3089	314.6581	Q	***973.5677***	487.2875	956.5411	478.7742	955.5571	478.2822	8
6	760.3624	380.6849	743.3359	372.1716	742.3519	371.6796	N	***845.5091***	423.2582	828.4825	414.7449	827.4985	414.2529	7
7	888.4574	444.7323	871.4308	436.2191	870.4468	435.7271	K	731.4662	366.2367	714.4396	357.7234	713.4556	357.2314	6
8	1001.5415	501.2744	984.5149	492.7611	983.5309	492.2691	L	***603.3712***	302.1892	586.3447	293.6760	585.3606	293.1840	5
9	1114.6255	557.8164	1097.5990	549.3031	1096.6150	548.8111	L	***490.2871***	245.6472	473.2606	237.1339	472.2766	236.6419	4
10	1243.6681	622.3377	1226.6416	613.8244	1225.6575	613.3324	E	***377.2031***	189.1052	360.1765	180.5919	359.1925	180.0999	3
11	1344.7158	672.8615	1327.6892	664.3483	1326.7052	663.8563	T	***248.1605***	124.5839	231.1339	116.0706	230.1499	115.5786	2
12							K	147.1128	74.0600	130.0863	65.5468			1

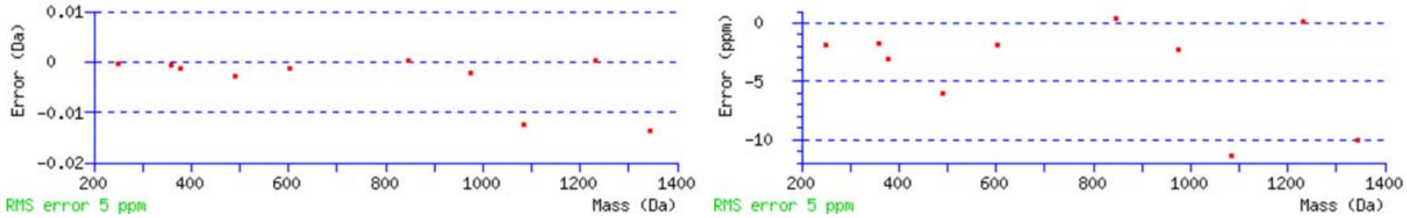

NCBI **BLAST** search of FLEQQNKLLETK
(Parameters: blastp, nr protein database, expect=20000, no filter, PAM30)
Other BLAST web gateways

All matches to this query

Score	Mr(calc)	Delta	Sequence
46.3	1489.8140	0.0015	FLEQQNKLLETK
0.6	1487.8069	2.0085	NVHRPPRQRDIT

Mascot: http://www.matrixscience.com/

Mascot Search Results

Peptide View

MS/MS Fragmentation of **FLEQQNKLLETK**
Found in **ch12u_Q9NSB2|KRT84_HUMAN** in **uni_human**, Keratin, type II cuticular Hb4 OS=Homo sapiens GN=KRT84 PE=2 SV=2

Match to Query 15166: 1490.805582 from(497.942470,3+) intensity(303248.1250) rtinseconds(1621) scans(8698) index(6802)
Title: 160219_Sunil_KAP_A1_Spectrum057769_scans__8698_RTINSECONDS=1621
Data file L:\\QE_2016\\160219_Sunil_KAP_LKC\\TMgf\\T\\T160219_Sunil_KAP_A1.mgf

Click mouse within plot area to zoom in by factor of two about that point
Or, Plot from 100 to 1400 Da Full range
Label all possible matches ○ Label matches used for scoring ◉
Show Y-axis ☐

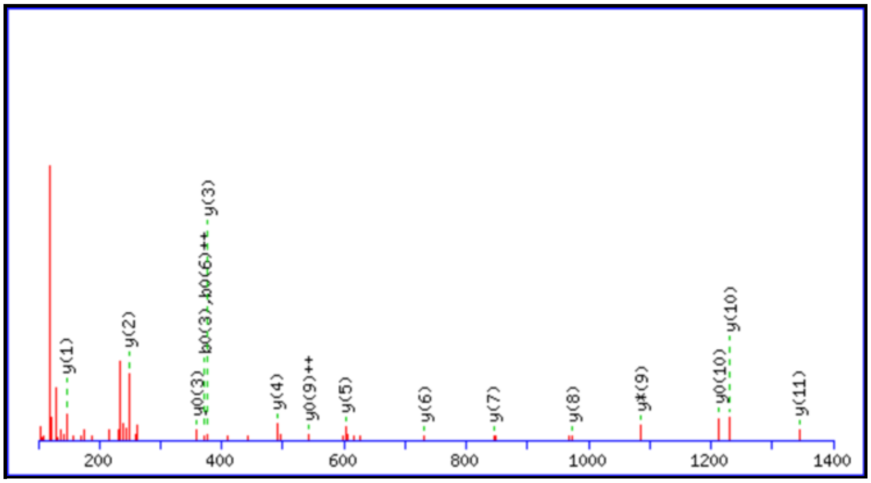


```
Monoisotopic mass of neutral peptide Mr(calc): 1490.7980
Fixed modifications: Carbamidomethyl (C) (apply to specified residues or termini only)
Variable modifications:
Q4     : Deamidated (NQ)
Ions Score: 69  Expect: 1.5e-005
Matches : 16/120 fragment ions using 26 most intense peaks   (help)
```

#	b	b^{++}	b*	b^{*++}	b^{0}	b^{0++}	Seq.	y	y^{++}	y*	y^{*++}	y^{0}	y^{0++}	#
1	148.0757	74.5415					F							12
2	261.1598	131.0835					L	1344.7369	672.8721	1327.7104	664.3588	1326.7264	663.8668	11
3	390.2023	195.6048			372.1918	186.5995	E	1231.6529	616.3301	1214.6263	607.8168	1213.6423	607.3248	10
4	519.2449	260.1261	502.2184	251.6128	501.2344	251.1208	Q	1102.6103	551.8088	1085.5837	543.2955	1084.5997	542.8035	9
5	647.3035	324.1554	630.2770	315.6421	629.2930	315.1501	Q	973.5677	487.2875	956.5411	478.7742	955.5571	478.2822	8
6	761.3464	381.1769	744.3199	372.6636	743.3359	372.1716	N	845.5091	423.2582	828.4825	414.7449	827.4985	414.2529	7
7	889.4414	445.2243	872.4149	436.7111	871.4308	436.2191	K	731.4662	366.2367	714.4396	357.7234	713.4556	357.2314	6
8	1002.5255	501.7664	985.4989	493.2531	984.5149	492.7611	L	603.3712	302.1892	586.3447	293.6760	585.3606	293.1840	5
9	1115.6095	558.3084	1098.5830	549.7951	1097.5990	549.3031	L	490.2871	245.6472	473.2606	237.1339	472.2766	236.6419	4
10	1244.6521	622.8297	1227.6256	614.3164	1226.6416	613.8244	E	377.2031	189.1052	360.1765	180.5919	359.1925	180.0999	3
11	1345.6998	673.3535	1328.6733	664.8403	1327.6892	664.3483	T	248.1605	124.5839	231.1339	116.0706	230.1499	115.5786	2
12							K	147.1128	74.0600	130.0863	65.5468			1

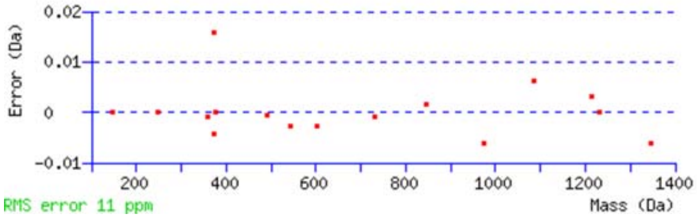

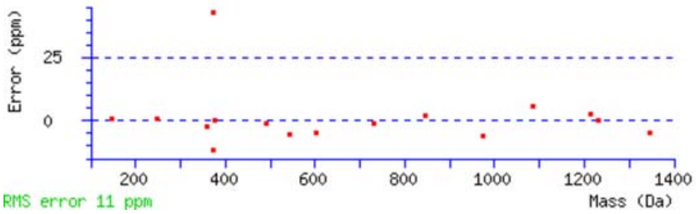

NCBI **BLAST** search of FLEQQNKLLETK
(Parameters: blastp, nr protein database, expect=20000, no filter, PAM30)
Other BLAST web gateways

All matches to this query

Score	Mr(calc)	Delta	Sequence	Site Analysis
69.0	1490.7980	0.0076	FLEQQNKLLETK	Deamidated Q4 95.70%
55.3	1490.7980	0.0076	FLEQQNKLLETK	Deamidated Q5 4.07%
48.9	1489.8140	0.9916	FLEQQNKLLETK	
42.9	1490.7980	0.0076	FLEQQNKLLETK	Deamidated N6 0.23%
15.4	1490.7981	0.0075	FQALQSVDILETK	
1.3	1488.7909	2.0146	NVHRPPRQRDIT	
1.3	1488.7909	2.0146	NVHRPPRQRDIT	
0.8	1489.8140	0.9916	RVYLATDDPSLLK	

Mascot: http://www.matrixscience.com/

Mascot Search Results

Peptide View

MS/MS Fragmentation of **VRFLEQQNKLLETK**
Found in **ch12u_Q9NSB2|KRT84_HUMAN** in **uni_human**, Keratin, type II cuticular Hb4 OS=Homo sapiens GN=KRT84 PE=2 SV=2

Match to Query 21478: 1744.981542 from(582.667790,3+) intensity(194135.5938) rtinseconds(1393) scans(7527) index(20234)
Title: 160219_Sunil_KAP_A1_Spectrum072686_scans__7527_RTINSECONDS=1393
Data file L:\\QE_2016\\160219_Sunil_KAP_LKC\\TMgf\\T\\T160219_Sunil_KAP_A1.mgf

Click mouse within plot area to zoom in by factor of two about that point
Or, Plot from 100 to 1800 Da Full range
Label all possible matches ○ Label matches used for scoring ◉
Show Y-axis ☐

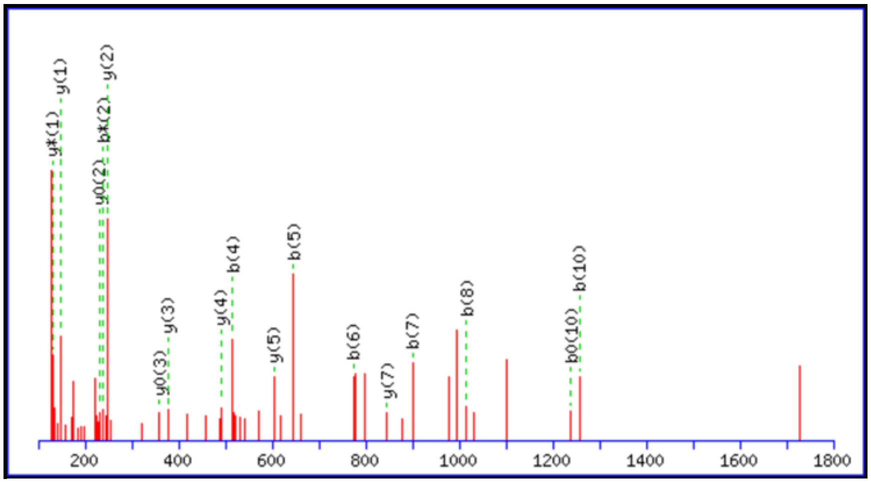

Monoisotopic mass of neutral peptide Mr(calc): 1744.9835
Fixed modifications: Carbamidomethyl (C) (apply to specified residues or termini only)
Ions Score: 43 **Expect:** 0.003
Matches : 17/144 fragment ions using 32 most intense peaks (help)

#	b	b^{++}	b*	b^{*++}	b^0	b^{0++}	Seq.	y	y^{++}	y*	y^{*++}	y^0	y^{0++}	#
1	100.0757	50.5415					V							14
2	256.1768	128.5920	239.1503	120.0788			R	1646.9224	823.9649	1629.8959	815.4516	1628.9119	814.9596	13
3	403.2452	202.1262	386.2187	193.6130			F	1490.8213	745.9143	1473.7948	737.4010	1472.8108	736.9090	12
4	***516.3293***	258.6683	499.3027	250.1550			L	1343.7529	672.3801	1326.7264	663.8668	1325.7423	663.3748	11
5	***645.3719***	323.1896	628.3453	314.6763	627.3613	314.1843	E	1230.6688	615.8381	1213.6423	607.3248	1212.6583	606.8328	10
6	***773.4305***	387.2189	756.4039	378.7056	755.4199	378.2136	Q	1101.6263	551.3168	1084.5997	542.8035	1083.6157	542.3115	9
7	***901.4890***	451.2482	884.4625	442.7349	883.4785	442.2429	Q	973.5677	487.2875	956.5411	478.7742	955.5571	478.2822	8
8	***1015.5320***	508.2696	998.5054	499.7563	997.5214	499.2643	N	***845.5091***	423.2582	828.4825	414.7449	827.4985	414.2529	7
9	1143.6269	572.3171	1126.6004	563.8038	1125.6164	563.3118	K	731.4662	366.2367	714.4396	357.7234	713.4556	357.2314	6
10	***1256.7110***	628.8591	1239.6844	620.3459	1238.7004	619.8538	L	***603.3712***	302.1892	586.3447	293.6760	585.3606	293.1840	5
11	1369.7950	685.4012	1352.7685	676.8879	1351.7845	676.3959	L	***490.2871***	245.6472	473.2606	237.1339	472.2766	236.6419	4
12	1498.8376	749.9225	1481.8111	741.4092	1480.8271	740.9172	E	***377.2031***	189.1052	360.1765	180.5919	359.1925	180.0999	3
13	1599.8853	800.4463	1582.8588	791.9330	1581.8748	791.4410	T	***248.1605***	124.5839	231.1339	116.0706	230.1499	115.5786	2
14							K	***147.1128***	74.0600	130.0863	65.5468			1

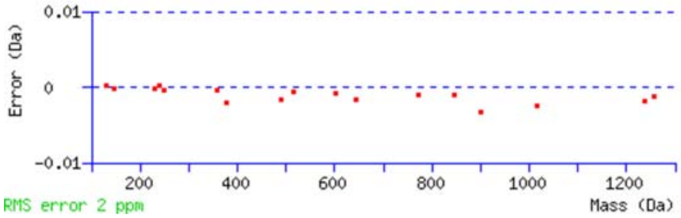

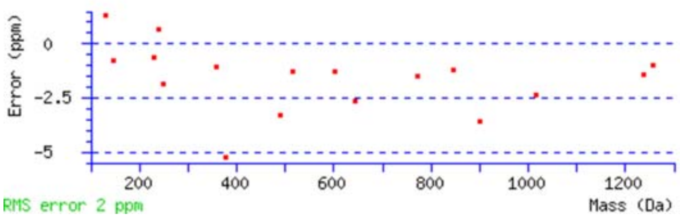

NCBI **BLAST** search of VRFLEQQNKLLETK
(Parameters: blastp, nr protein database, expect=20000, no filter, PAM30)
Other BLAST web gateways

All matches to this query

Score	Mr(calc)	Delta	Sequence
43.1	1744.9835	-0.0020	VRFLEQQNKLLETK
0.5	1743.9659	1.0157	GSLTFEPLTLVPIQTK

Mascot: http://www.matrixscience.com/

Mascot Search Results

Peptide View

MS/MS Fragmentation of **VRFLEQQNKLLETK**
Found in **ch12u_Q9NSB2|KRT84_HUMAN** in **uni_human**, Keratin, type II cuticular Hb4 OS=Homo sapiens GN=KRT84 PE=2 SV=2

Match to Query 21478: 1744.981542 from(582.667790,3+) intensity(194135.5938) rtinseconds(1393) scans(7527) index(20234)
Title: 160219_Sunil_KAP_A1_Spectrum072686_scans__7527_RTINSECONDS=1393
Data file L:\\QE_2016\\160219_Sunil_KAP_LKC\\TMgf\\T\\T160219_Sunil_KAP_A1.mgf

Click mouse within plot area to zoom in by factor of two about that point
Or, Plot from 100 to 1800 Da Full range
Label all possible matches ○ Label matches used for scoring ◉
Show Y-axis ☐

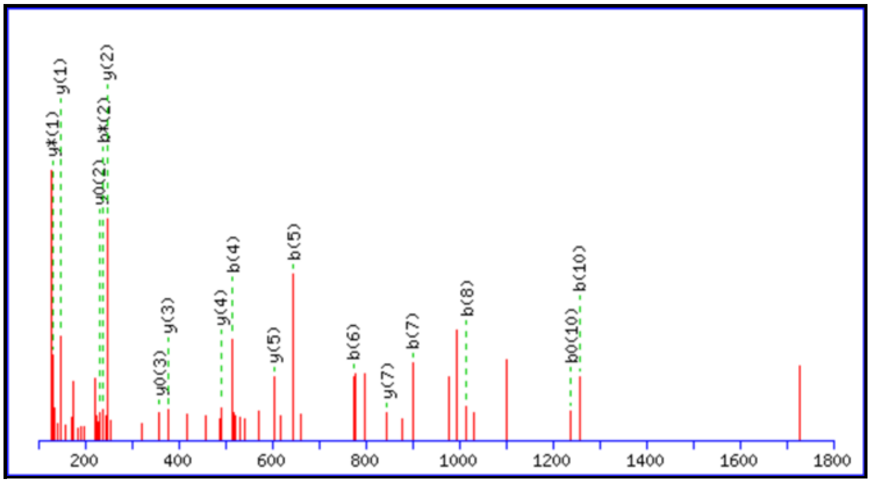

Monoisotopic mass of neutral peptide Mr(calc): 1744.9835
Fixed modifications: Carbamidomethyl (C) (apply to specified residues or termini only)
Ions Score: 43 **Expect:** 0.003
Matches : 17/144 fragment ions using 32 most intense peaks (help)

#	b	b^{++}	b*	b^{*++}	b^0	b^{0++}	Seq.	y	y^{++}	y*	y^{*++}	y^0	y^{0++}	#
1	100.0757	50.5415					V							14
2	256.1768	128.5920	239.1503	120.0788			R	1646.9224	823.9649	1629.8959	815.4516	1628.9119	814.9596	13
3	403.2452	202.1262	386.2187	193.6130			F	1490.8213	745.9143	1473.7948	737.4010	1472.8108	736.9090	12
4	***516.3293***	258.6683	499.3027	250.1550			L	1343.7529	672.3801	1326.7264	663.8668	1325.7423	663.3748	11
5	***645.3719***	323.1896	628.3453	314.6763	627.3613	314.1843	E	1230.6688	615.8381	1213.6423	607.3248	1212.6583	606.8328	10
6	***773.4305***	387.2189	756.4039	378.7056	755.4199	378.2136	Q	1101.6263	551.3168	1084.5997	542.8035	1083.6157	542.3115	9
7	***901.4890***	451.2482	884.4625	442.7349	883.4785	442.2429	Q	973.5677	487.2875	956.5411	478.7742	955.5571	478.2822	8
8	***1015.5320***	508.2696	998.5054	499.7563	997.5214	499.2643	N	***845.5091***	423.2582	828.4825	414.7449	827.4985	414.2529	7
9	1143.6269	572.3171	1126.6004	563.8038	1125.6164	563.3118	K	731.4662	366.2367	714.4396	357.7234	713.4556	357.2314	6
10	***1256.7110***	628.8591	1239.6844	620.3459	1238.7004	619.8538	L	***603.3712***	302.1892	586.3447	293.6760	585.3606	293.1840	5
11	1369.7950	685.4012	1352.7685	676.8879	1351.7845	676.3959	L	***490.2871***	245.6472	473.2606	237.1339	472.2766	236.6419	4
12	1498.8376	749.9225	1481.8111	741.4092	1480.8271	740.9172	E	***377.2031***	189.1052	360.1765	180.5919	359.1925	180.0999	3
13	1599.8853	800.4463	1582.8588	791.9330	1581.8748	791.4410	T	***248.1605***	124.5839	231.1339	116.0706	230.1499	115.5786	2
14							K	***147.1128***	74.0600	130.0863	65.5468			1

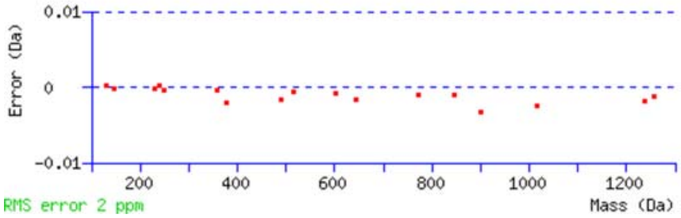

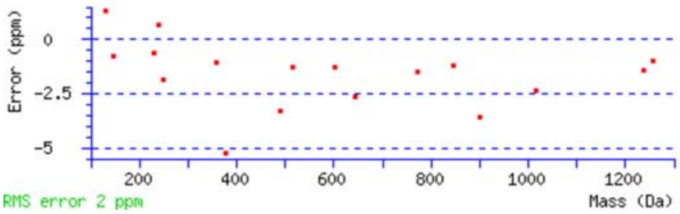

NCBI **BLAST** search of VRFLEQQNKLLETK
(Parameters: blastp, nr protein database, expect=20000, no filter, PAM30)
Other BLAST web gateways

All matches to this query

Score	Mr(calc)	Delta	Sequence
43.1	1744.9835	-0.0020	VRFLEQQNKLLETK
0.5	1743.9659	1.0157	GSLTFEPLTLVPIQTK

Mascot: http://www.matrixscience.com/

Mascot Search Results

Peptide View

MS/MS Fragmentation of **VRFLEQQNKLLETK**
Found in **ch12u_Q9NSB2|KRT84_HUMAN** in **uni_human**, Keratin, type II cuticular Hb4 OS=Homo sapiens GN=KRT84 PE=2 SV=2

Match to Query 21479: 1744.983552 from(582.668460,3+) intensity(234656.3750) rtinseconds(1489) scans(7941) index(6141)
Title: 160219_Sunil_KAP_A1_Spectrum057108_scans__7941_RTINSECONDS=1489
Data file L:\\QE_2016\\160219_Sunil_KAP_LKC\\TMgf\\T\\T160219_Sunil_KAP_A1.mgf

Click mouse within plot area to zoom in by factor of two about that point
Or, Plot from 100 to 1300 Da Full range
Label all possible matches ○ Label matches used for scoring ◉
Show Y-axis □

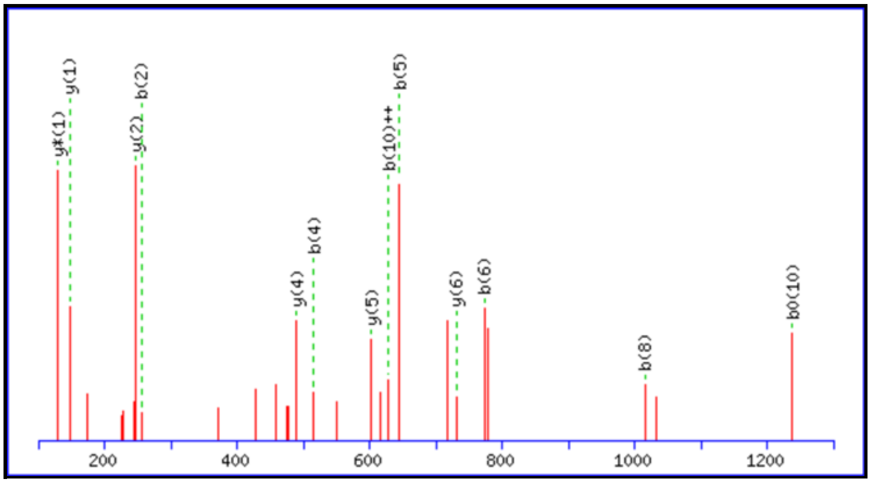

Monoisotopic mass of neutral peptide Mr(calc): 1744.9835
Fixed modifications: Carbamidomethyl (C) (apply to specified residues or termini only)
Ions Score: 40 **Expect:** 0.0062
Matches : 13/144 fragment ions using 22 most intense peaks (help)

#	b	b^{++}	b*	b^{*++}	b^0	b^{0++}	Seq.	y	y^{++}	y*	y^{*++}	y^0	y^{0++}	#
1	100.0757	50.5415					V							14
2	***256.1768***	128.5920	239.1503	120.0788			R	1646.9224	823.9649	1629.8959	815.4516	1628.9119	814.9596	13
3	403.2452	202.1262	386.2187	193.6130			F	1490.8213	745.9143	1473.7948	737.4010	1472.8108	736.9090	12
4	***516.3293***	258.6683	499.3027	250.1550			L	1343.7529	672.3801	1326.7264	663.8668	1325.7423	663.3748	11
5	***645.3719***	323.1896	628.3453	314.6763	627.3613	314.1843	E	1230.6688	615.8381	1213.6423	607.3248	1212.6583	606.8328	10
6	***773.4305***	387.2189	756.4039	378.7056	755.4199	378.2136	Q	1101.6263	551.3168	1084.5997	542.8035	1083.6157	542.3115	9
7	901.4890	451.2482	884.4625	442.7349	883.4785	442.2429	Q	973.5677	487.2875	956.5411	478.7742	955.5571	478.2822	8
8	***1015.5320***	508.2696	998.5054	499.7563	997.5214	499.2643	N	845.5091	423.2582	828.4825	414.7449	827.4985	414.2529	7
9	1143.6269	572.3171	1126.6004	563.8038	1125.6164	563.3118	K	***731.4662***	366.2367	714.4396	357.7234	713.4556	357.2314	6
10	1256.7110	**628.8591**	1239.6844	620.3459	1238.7004	619.8538	L	***603.3712***	302.1892	586.3447	293.6760	585.3606	293.1840	5
11	1369.7950	685.4012	1352.7685	676.8879	1351.7845	676.3959	L	***490.2871***	245.6472	473.2606	237.1339	472.2766	236.6419	4
12	1498.8376	749.9225	1481.8111	741.4092	1480.8271	740.9172	E	377.2031	189.1052	360.1765	180.5919	359.1925	180.0999	3
13	1599.8853	800.4463	1582.8588	791.9330	1581.8748	791.4410	T	***248.1605***	124.5839	231.1339	116.0706	230.1499	115.5786	2
14							K	***147.1128***	74.0600	130.0863	65.5468			1

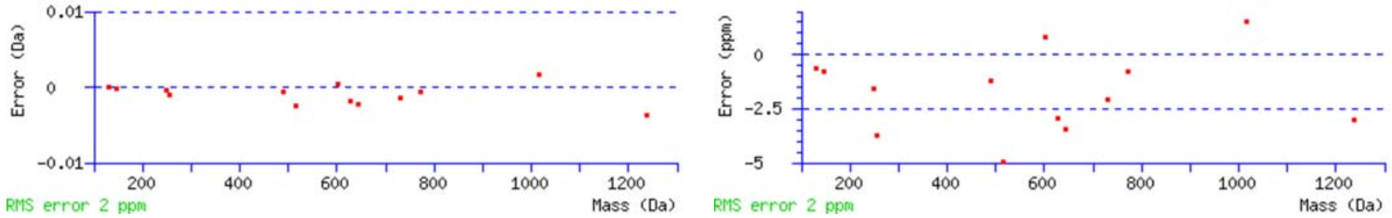

NCBI **BLAST** search of VRFLEQQNKLLETK
(Parameters: blastp, nr protein database, expect=20000, no filter, PAM30)
Other BLAST web gateways

All matches to this query

Score	Mr(calc)	Delta	Sequence
39.8	1744.9835	0.0000	VRFLEQQNKLLETK
5.9	1744.9869	-0.0033	KSLESINSRLQLVMK
0.3	1743.9659	1.0177	GSLTFEPLTLVPIQTK

Mascot: http://www.matrixscience.com/

Mascot Search Results

Peptide View

MS/MS Fragmentation of **LGLDIEIATYRR**
Found in **ch12u_Q9NSB2|KRT84_HUMAN** in **uni_human**, Keratin, type II cuticular Hb4 OS=Homo sapiens GN=KRT84 PE=2 SV=2

Match to Query 13040: 1418.788392 from(473.936740,3+) intensity(561901.3125) rtinseconds(2205) scans(12190) index(24079)
Title: 160219_Sunil_KAP_A1_Spectrum076549_scans__12190_RTINSECONDS=2205
Data file L:\\QE_2016\\160219_Sunil_KAP_LKC\\TMgf\\T\\T160219_Sunil_KAP_A1.mgf

Click mouse within plot area to zoom in by factor of two about that point
Or, Plot from 100 to 1500 Da Full range
Label all possible matches ○ Label matches used for scoring ◉
Show Y-axis ☐

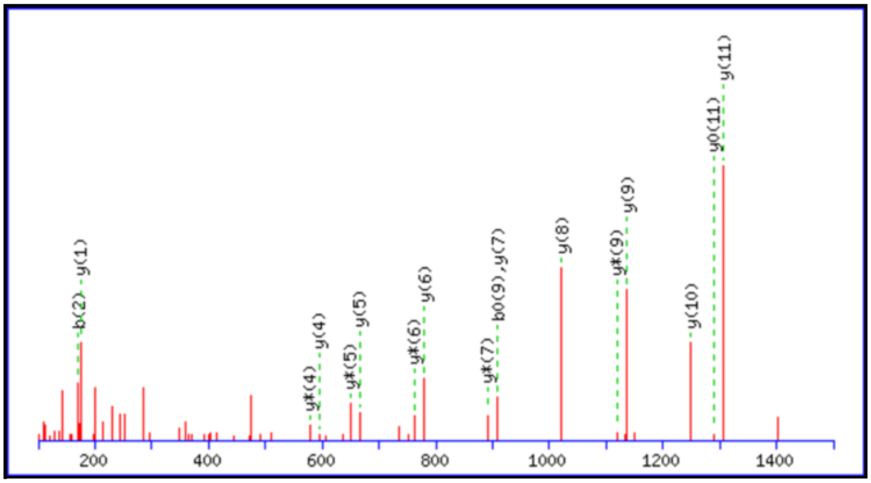

Monoisotopic mass of neutral peptide Mr(calc): 1418.7881
Fixed modifications: Carbamidomethyl (C) (apply to specified residues or termini only)
Ions Score: 63 **Expect:** 5.1e-005
Matches : 17/100 fragment ions using 32 most intense peaks (help)

#	b	b^{++}	b^{*}	b^{*++}	b^{0}	b^{0++}	Seq.	y	y^{++}	y^{*}	y^{*++}	y^{0}	y^{0++}	#
1	114.0913	57.5493					L							12
2	171.1128	86.0600					G	1306.7114	653.8593	1289.6848	645.3461	1288.7008	644.8540	11
3	284.1969	142.6021					L	1249.6899	625.3486	1232.6634	616.8353	1231.6793	616.3433	10
4	399.2238	200.1155			381.2132	191.1103	D	1136.6058	568.8066	1119.5793	560.2933	1118.5953	559.8013	9
5	512.3079	256.6576			494.2973	247.6523	I	1021.5789	511.2931	1004.5524	502.7798	1003.5683	502.2878	8
6	641.3505	321.1789			623.3399	312.1736	E	908.4948	454.7511	891.4683	446.2378	890.4843	445.7458	7
7	754.4345	377.7209			736.4240	368.7156	I	779.4522	390.2298	762.4257	381.7165	761.4417	381.2245	6
8	825.4716	413.2395			807.4611	404.2342	A	666.3682	333.6877	649.3416	325.1745	648.3576	324.6824	5
9	926.5193	463.7633			908.5088	454.7580	T	595.3311	298.1692	578.3045	289.6559	577.3205	289.1639	4
10	1089.5827	545.2950			1071.5721	536.2897	Y	494.2834	247.6453	477.2568	239.1321			3
11	1245.6838	623.3455	1228.6572	614.8322	1227.6732	614.3402	R	331.2201	166.1137	314.1935	157.6004			2
12							R	175.1190	88.0631	158.0924	79.5498			1

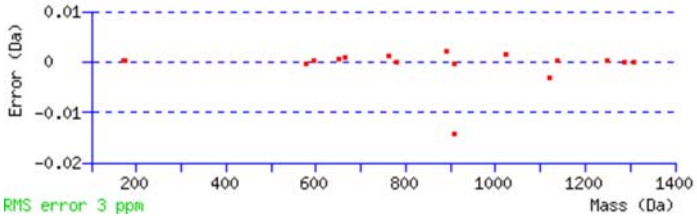

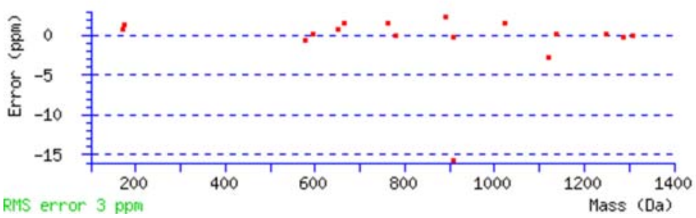

NCBI **BLAST** search of LGLDIEIATYRR
(Parameters: blastp, nr protein database, expect=20000, no filter, PAM30)
Other BLAST web gateways

All matches to this query

Score	Mr(calc)	Delta	Sequence
62.7	1418.7881	0.0003	LGLDIEIATYRR
23.9	1418.7881	0.0003	LGLLDRIVNYSR
12.9	1418.7769	0.0115	LLGNTFVALSDLR
7.3	1418.7881	0.0003	LQLRSLQYLER
5.4	1418.7769	0.0115	GLVLQLIQSYQR
3.6	1418.7769	0.0115	GLVLQLIQSYQR
2.4	1418.7742	0.0142	LGEARHPQVSLGR
2.4	1418.7994	-0.0110	LGPESVPPPKRSR

Mascot: http://www.matrixscience.com/
